# Stress-induced changes in gene interactions in human cells

**DOI:** 10.1093/nar/gkt999

**Published:** 2013-10-28

**Authors:** Renuka R. Nayak, William E. Bernal, Jessica W. Lee, Michael J. Kearns, Vivian G. Cheung

**Affiliations:** ^1^Medical Scientist Training Program, University of Pennsylvania Perelman School of Medicine, Philadelphia, PA 19104, USA, ^2^Division of Rheumatology, The Children’s Hospital of Philadelphia, Philadelphia, PA 19104, USA, ^3^HHMI Medical Research Fellows Program, University of Pennsylvania, Philadelphia, PA 19104, USA, ^4^Department of Computer and Information Science, University of Pennsylvania, Philadelphia, PA 19104, USA, ^5^Department of Pediatrics, University of Pennsylvania, Philadelphia, PA 19104, USA, ^6^Department of Genetics, University of Pennsylvania, Philadelphia, PA 19104, USA and ^7^Howard Hughes Medical Institute, Chevy Chase, MD 20815, USA

## Abstract

Cells respond to variable environments by changing gene expression and gene interactions. To study how human cells response to stress, we analyzed the expression of >5000 genes in cultured B cells from nearly 100 normal individuals following endoplasmic reticulum stress and exposure to ionizing radiation. We identified thousands of genes that are induced or repressed. Then, we constructed coexpression networks and inferred interactions among genes. We used coexpression and machine learning analyses to study how genes interact with each other in response to stress. The results showed that for most genes, their interactions with each other are the same at baseline and in response to different stresses; however, a small set of genes acquired new interacting partners to engage in stress-specific responses. These genes with altered interacting partners are associated with diseases in which endoplasmic reticulum stress response or sensitivity to radiation has been implicated. Thus, our findings showed that to understand disease-specific pathways, it is important to identify not only genes that change expression levels but also those that alter interactions with other genes.

## INTRODUCTION

Human cells exist in dynamic environments where proper function depends on coordinated responses to stresses. These responses include changes in gene expression and gene interactions. Ineffective responses can lead to the development of diseases. Knowing how genes are induced and how they interact is important for modulating stress response for disease prevention and treatment. In addition, effective therapies rely on knowing which part of the response network to target and how that intervention will be propagated through gene networks.

In different cellular states, genes change how they interact with each other. Although studies have identified genes that constitute stress responses, changes in gene interaction in response to stress are less well known. In particular, there is a paucity of information on the extent to which gene interactions are specific to various stimuli. In this study, we focused on the response of human cells to two different stresses: endoplasmic reticulum (ER) stress and exposure to ionizing radiation (IR). We examined changes in gene expression and gene interactions in human cultured B cells from ∼100 normal individuals. Previous studies examined changes in the expression level in response to stressful stimuli (1–3), but not in gene interactions. Here, we took advantage of individual differences in human gene expression to infer interactions among thousands of genes at baseline and in response to these two stresses (4). We compared the results to determine changes in gene interactions and to identify the changes that are generalized and those that are stress-specific. To our knowledge, these analyses are the first genome-wide examination of gene interactions in human cells before and after stress.

We examined cellular responses to two different stresses: ER stress and IR. The ER is the organelle in which secreted or transmembrane proteins are folded. When the demand for protein-folding machinery exceeds capacity, cells activate a complex set of gene pathways in a process known as ER stress response. These processes include attenuation of global translation and increased expression of protein-folding machinery. Second, we examined cellular response to IR, which is present in the environment and commonly used in medicine for diagnostic and therapeutic purposes. Radiation induces DNA double-strand breaks and damages cellular structures (5,6). In response, cells repair the damages or undergo cell death. Failure to respond properly to radiation exposure or protein load has been implicated in many human diseases from cancers to neurodegenerative diseases (7) and immunodeficiencies (8); thus, a better understanding of stress response should allow for more precise approaches to disease prevention and treatment.

## MATERIALS AND METHODS

### Cells and gene expression experiments

Cultured B cells were obtained from Coriell Cell Repositories. Cell lines were treated with dimethyl sulfoxide (DMSO)/tunicamycin (N = 131) or IR (N = 95). There were 56 individuals who were studied in both experiments. Gene expression was measured as described previously. Briefly, cells were grown in RPMI 1640 with 15% fetal bovine serum, 2 mM l-glutamine and 100 U/ml penicillin–streptomycin. RNA was extracted using the RNeasy Mini-Kit (Qiagen), amplified, labeled and hybridized as per the manufacturer’s instructions. Expression levels of genes were measured using Human Genome U133 Plus 2.0 or Affymetrix Human U133A 2.0 Arrays (Affymetrix, CA, USA). Gene expression signals were normalized using the MAS 5.0 algorithm (Affymetrix, CA, USA). Expression intensity was scaled to 500 and log_2_-transformed. Some genes are represented multiple times on the Human Genome U133 Plus 2.0 Arrays (ER dataset) and Affymetrix Human U133A 2.0 Arrays (IR dataset). If a gene was represented more than once, only one of the probesets was selected (randomly) to include in our analysis. Probesets annotated as ‘x_at’ were excluded from this analysis.

Statement of ethical approval: all the cells used in this study are from anonymized donors and have been approved for exception by the Human Subject Review Board of the University of Pennsylvania.

### Tunicamycin treatment

Cells were treated with 4 μg/ml of tunicamycin (T7765 Sigma, MO, USA) in DMSO or DMSO alone (vehicle control) for 8 h. The dose and time points were optimized in our previous study (9).

### IR treatment

Cells were harvested before radiation and at 2 and 6 h following exposure to IR (10 Gy in a ^137^Cs irradiator).

### Gene correlation and construction of coexpression networks

There were 12 660 unique genes that were represented on both the Human Genome U133 Plus 2.0 and Affymetrix Human U133A 2.0 Arrays. Of these, 6775 genes were called ‘present’ by the MAS 5.0 algorithm in at least 80% of samples in either the DMSO- or tunicamycin-treated samples. There were 5975 genes called ‘present’ in at least 80% of samples at 0, 2 or 6 h after radiation treatment. We considered these genes as ‘expressed’ and focused on these for all analyses. For all possible pairs of genes, we calculated the Pearson correlation of expression levels across individuals. This calculation was done separately for each treatment or time point. The distributions of correlations were normal. Fisher’s test of homogeneity was used to identify correlations that significantly differed (Bonferroni corrected *P* < 0.05) between treatments or among time points. Correlated gene pairs were connected to construct a coexpression network. We constructed multiple networks using different thresholds and measured topological properties of the resulting networks. Correlations and topological properties of the network were analyzed using MATLAB (The MathWorks, Inc, Natick, MA, USA). Networks were represented as adjacency matrices in MATLAB, and standard MATLAB functions were used to calculate the number of genes, the number of connections and the distribution of connections in each network. MATLAB functions for determining the clustering coefficient (10), gamma (11) and scale-free topology criteria (12) were implemented as previously described. Code will be provided on request.

A similar series of analyses was done examining fold changes across individuals, so that changes in expression levels are normalized against each person’s baseline. This type of analysis reduces the number of genes included in the network: only those genes showing changes in expression levels are included when examining correlation in fold changes. This method also eliminates the baseline or 0-h time point, which is used for normalization, as a basis for comparison. Nonetheless, we found similar findings using these smaller networks and present the results of the more inclusive analysis here.

### Random gene pairs and networks

Random gene pairs were genes that were paired randomly as opposed to being paired based on correlation patterns. These random gene pairs were generated from random networks described later. Random gene pairs were used to assess frequency of shared GO biological terms by chance alone.

Random networks were constructed as described previously (13). MATLAB code provided by S. Maslov (http://www.cmth.bnl.gov/∼maslov/matlab.htm) was used to generate random networks. Briefly, random networks consisted of the same number of genes as in the observed networks and were constructed to have the same topology as observed networks. To do this, a gene in the random network had the same number of connections as in the observed network, but its connections to other genes were random instead of being based on correlation patterns.

### Significance testing

For genes examined in tunicamycin-treated samples, changes in expression levels were assessed by Student’s paired *t*-test with Bonferroni correction for 6775 tests (denoted *P_c_* < 0.05). For genes examined in samples treated with IR, changes in gene expression levels were assessed by Student’s paired *t*-test with Bonferroni correction for 5975 tests (denoted *P_c_* < 0.05). Significant results after correction are denoted as ‘P_c_’ throughout the text.

### Caspase assays

Cells from 95 unrelated individuals were irradiated at 10 Gy in a ^137^Cs irradiator. Cellular response to radiation exposure was measured 24 h after irradiation using the Caspase-Glo 3/7 assay (Promega). Caspase activity levels were log_2_-normalized for comparison with log_2_-transformed gene expression levels.

### *THAP1* small interfering RNA knockdown

Primary fibroblasts were transfected with Silencer Select siRNAs (Applied Biosystems) directed against *THAP1* or a non-target control using the RNAiMAX reagent according to the manufacturer’s instructions (Invitrogen). Primary skin fibroblasts were selected to provide an additional cell type for replication. Cells were treated for 8 h with DMSO or tunicamycin 24 h after transfection. RNA was harvested to assess knockdown efficiency. Effect of small interfering RNA (siRNA) on gene expression was analyzed by quantitative polymerase chain reaction (PCR; 7900HT Analyzer, Applied Biosystems). Expression of *NDUFA4* was used as a control for normalization, and expression levels were calculated relative to *NDUFA4*. Sequences of PCR primers were as follows: *ATF4* (forward–CCAACAACAGCAAGGAGGAT, reverse–GTGTCATCCAACGTGGTCAG), *DDIT3* (forward–TCACCTCCTGGAAATGAAGA, reverse–CTCCTCCTCAGTCAGCCAAG), *HSPA5* (forward–GGAAAGAAGGTTACCCATGC, reverse–CCGTAGGCTCGTTGATGAT), *THAP1* (forward–TGCTGTGCCCACAATATTTC, reverse–AGGAGGCGGTAAAGGAGGT) and *NDUFA4* (forward–GTCAGGCCAAGAAGCATCC, reverse–GCTCCAGTAGCTCCAGTTCC). We pooled three siRNAs against *THAP1*: sense 5'-AGGACAAGCCCGUUUCUUUtt-3', antisense 5'-AAAGAAACGGGCUUGUCCUtg-3'; sense 5'-ACUUAAAAUUAGUACUGUUtt-3', antisense 5'-AACAGUACUAAUUUUAAGUtt-3'; and sense: 5'-UGAUUAUCAUCACAGCAGAtt-3', antisense: 5'-UCUGCUGUGAUGAUAAUCAaa-3'. Protein levels of *THAP1* were assessed using rabbit antibody directed against *Thap1* (ProteinTech, IL, USA) and normalized to glyceraldehyde 3-phosphate dehydrogenase (sc-137179, Santa Cruz Biotechnology, CA, USA).

### *ARHGAP1* and *RAB35* siRNA knockdown

Cultured B cells from two individuals were transfected with Accell SMARTpool siRNAs (Thermo Scientific) directed against *ARHGAP1*, *RAB35* or a non-target control according to the manufacturer’s instructions. One hundred twenty hours after transfection, cells were treated for 8 h with DMSO or tunicamycin and then harvested for RNA. Effect of siRNA on gene expression was analyzed by quantitative PCR (7900HT Analyzer, Applied Biosystems).

Expression levels were normalized to *NDUFA4*. Sequences of PCR primers for *ATF4*, *DDIT3*, *HSPA5* and *NDUFA4* are the same as those used for *THAP1* siRNA knockdown. Sequences of PCR primers were as follows: *ARHGAP1* (forward–GGTGGGCTTCCTCAACATTG, reverse–CAGTCAGGAAACGAAGCACC) and *RAB35* (forward–GATCGCGAGGCCAGTACC, reverse–CTCTTGCCCACACCGCTG). The pooled siRNA against *ARHGAP1* targeted the following sequences: 5'-CUGGUAGCUUGGAUGACAU-3', 5'-UCAUCAGCCUUCAUGUUCU-3', 5'-CCUUUGUGUAUCAAGUGUC-3' and 5'-GCCAAGUGCUCAAAUAUGA-3'. The pooled siRNA against *RAB35* targeted the following sequences: 5'-GGAGUAUUUCUGUAUUGAA-3', 5'-GGAUUAUUUUAACAGAUCA-3', 5'-GUUUCGUGCCGUUAUUUAA-3' and 5'-CCCUGAGGUUUGAUUGGCA-3'.

### Enrichment analysis

Enrichment analysis of Gene Ontology Biological Processes was done using DAVID NIH (14,15). Default parameters were used except the background was modified to be the genes examined here (either 6775 genes for the ER dataset or 5975 for the IR dataset). Enrichment results that were significant after Benjamini–Hochberg (16) are noted. We performed functional annotation clustering using ‘GOTERM-BP’ categories.

### Literature mining

To determine whether a gene has been previously implicated in ER or IR stress, PubMed was searched with the following queries:

- *Gene symbol* and (‘unfolded protein response’ or ‘endoplasmic reticulum stress’).
- *Gene symbol* and (‘ionizing radiation’ or ‘DNA damage’).

The number of PubMed abstracts returned indicated the number of times the gene has been implicated in the specific stress response. We note there are limitations to searching the literature this way, especially when the official gene symbol is not the common symbol used by investigators.

### ConsensusPathDB mining

ConsensusPathDB is an online database that integrates interaction networks in humans and includes protein–protein, genetic, metabolic, signaling, gene regulatory and drug–target interactions as well as biochemical pathways. Using their ‘shortest interaction paths’ tool, we queried the shortest interaction paths between gene pairs of interest. We focused on 284 and 50 gene pairs that change interactions following ER and IR stress, respectively. Gene symbols were used in the analysis, and each gene in a pair was used as the path start or path end. The top interaction generated by the database was used in our analysis.

### Machine learning using support vector machines

Support vector machine (SVM) linear regression models were trained to capture quantitative relationships between a given target gene and all other genes. For each gene, an expression prediction model was developed using baseline samples only. If gene interactions change on stress, then a model developed from baseline data would not be expected to produce accurate predictions in stressed samples. In such cases, a decline in model performance is a proxy for a target gene that alters interactions on stress. Model prediction performance was assessed using *R*^2^, the square of the correlation between the predicted and actual expression level of the target gene. Details of SVM training, testing and parameter selection are given later.

We developed models to predict the expression of a ‘target’ gene using the expression levels of all other genes in each individual:

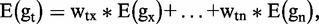

where ‘E(g_x_)’ represents the ‘expression level of gene x’ and where the learned weight, ‘w_tx_’, captures the relationship between the target gene *t* and gene *x*. A model was developed for a gene using baseline samples only (not stressed samples).

Each gene was treated as a target gene whose expression was predicted using the other genes as ‘predictor variables.’ Expression was modeled using SVM regression with a linear kernel. Standard least-squares fit linear regressions were also used, but SVM linear regression outperformed standard linear regression. The amount of tolerated error was set to 0.1 (epsilon = 0.1), with remaining parameters set to their default. SVM performance was assessed using alternative parameters (varying epsilon or using non-linear kernel), and those that performed best (epsilon = 0.1 and linear kernel) were used in the present analyses. For each of the 6775 genes in the ER dataset, the remaining 6774 genes were used to train an SVM using unstressed (DMSO) samples. For each of the 5975 genes in the IR dataset, the remaining 5974 genes were used to train an SVM using unstressed (0 h) samples. To train and test the SVM models, individuals were randomly divided into training and testing datasets. In the ER dataset, 66 individuals were used to train each SVM and the remaining 65 were used to test it. In the IR dataset, 48 individuals were used for training and 47 individuals for testing. Models were then tested on stressed samples.

For modeling analyses, expression of each gene was normalized to have a mean of 0 and standard deviation of 1 in each dataset (training and testing sets were normalized separately). SVM performance was assessed by calculating *R*^2^ (square of the correlation coefficient) between expected and observed expression levels in individuals. In the ER dataset, *R*^2^ was calculated across 65 individuals in unstressed samples and across 131 samples in stressed samples. In the IR dataset, *R*^2^ was calculated in 47 individuals at 0 h and 95 individuals at 2 and 6 h.

### Permutation and randomization procedures

We assessed the significance of the difference in *R*^2^ between baseline and stressed samples for each SVM gene model empirically by permutation test. Using baseline (testing samples only) and stressed samples, we randomly assigned each sample to one of the two groups; we then calculated the *R*^2^ of model performance on each group and computed the difference between the two groups (ΔR^2^). After repeating the procedure 1000 times, we had a distribution of ΔR^2^. For each gene model, we determined whether the real ΔR^2^ fell among the largest 1% of values resulting from permutation. *P-*value was assigned by counting how frequently a difference in *R*^2^ greater than or equal to that seen in the real data was seen in the permuted data. False discovery rates (FDR) were also calculated from the permuted data.

## RESULTS

### Stress induces extensive changes in gene expression levels

First, we studied human cultured B cells following ER stress and exposure to radiation. We induced ER stress in cultured human cultured B cells from 131 unrelated individuals with tunicamycin, a drug that prevents *N*-glycosylation and leads to accumulation of proteins in the ER (17). We then measured gene expression at baseline and 8 h following treatment (9). Second, we exposed human cultured B cells from 95 unrelated individuals to IR. Gene expression was measured before irradiation, and 2 and 6 h after radiation as described previously and extended here (18). We focused our analysis on 6775 and 5975 genes that were expressed at baseline and in response to ER and IR stress, respectively (see Methods). ER and IR stress both led to extensive changes in gene expression levels. ER stress induced changes in 71% of the genes (4801/6775) (Supplementary Table S1, *P_c_* < 0.05, *t*-test with Bonferroni correction), and IR caused changes in 23% (1381/5975) and 32% (1902/5975) of genes at 2 and 6 h, respectively (Supplementary Table S2, *P_c_* < 0.05, *t*-test with Bonferroni correction).

**Figure 1.**
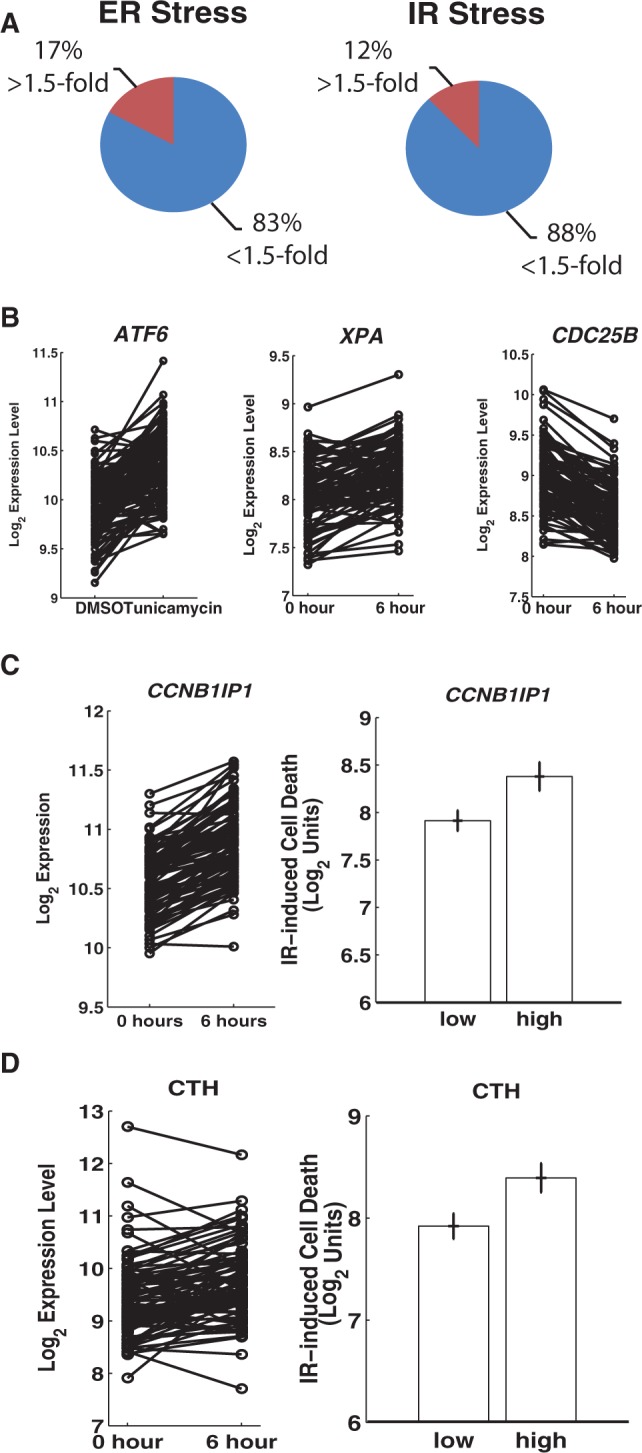
Extensive changes in gene expression following ER stress and exposure to radiation. (**A**) Many of the genes that showed significant changes in expression are only modestly up- or downregulated (<1.5-fold). (**B**) *ATF6*, *XPA* and *CDC25B* show modest but consistent expression changes on stress. (**C**) Panel 1: The expression of *CCNB1IP1* is induced among 95 individuals on IR stress. Panel 2: Individuals with less upregulation of *CCNB1IP1* (N = 10) show less cell death than high-inducers (N = 10). (**D**) Panel 1: Expression of *CTH* in 95 individuals before and 6 h after exposure to IR. Panel 2: Changes in *CTH* are associated with IR-induced cell death (N = 10 in each sample). Error bars represents S.E.M.

We identified a large number of ER and IR responsive genes. Some of them showed >1.5-fold change in expression levels, but the majority (>80%) of genes showed changes that were <1.5-fold, including those known to play critical roles in the stress response (Figure 1A). For example, well-characterized genes in ER stress such as *ATF6* and *ERN1* (also called IRE1) showed 1.4- and 1.3-fold increase, respectively (Figure 1B; *P_c_* < 0.05) (19), and in response to IR stress, genes such as *XPA* and *CDC25B* showed 1.2- and 1.3-fold change, respectively (Figure 1B; *P_c_* < 0.05) (20,21). This suggests that modest changes (<1.5-fold) may be biologically important but may require larger sample sizes, such as those used here (131 individuals in ER stress and 95 individuals in IR stress), to be identified. However, the modest changes could be a result of large individual variation in expression response where some individuals showed positive fold changes and others showed negative response, thus giving a misleadingly small average fold change. We examined these genes closely and did not find this artifact. This is illustrated in Figure 1B, where all individuals showed modest yet consistent changes in expression.

This led us to ask whether modest changes in gene expression affect cellular processes. Previously, using the same individuals studied here, we measured IR-induced cell death as an indicator of IR stress response (18). We used these data to determine whether the 183 genes that showed modest and variable changes in expression (fold change 1.2–1.3 at 6 h after irradiation and variance across individuals >0.02) are associated with cell death. We found 14 genes that showed significant correlation with cell death (*P* < 0.05; Pearson’s correlation >0.17). An example is the expression response of the cell cycle gene *CCNB1IP1*, which increased by an average of 1.2-fold on IR treatment (*P*_c_ < 10^−^^23^ at 6 h, *t*-test) (Figure 1C). Individuals with the most induction of *CCNB1IP1* showed more cell death than those with less induction (*P* = 0.02, *t*-test, Figure 1C). Similarly, cystathionine gamma-lyase (*CTH*) increased by an average of 1.2-fold following radiation (Figure 1D); in individuals who repressed *CTH* following IR, cell death was less extensive (Figure 1D) (*P* = 0.02, *t*-test). These findings suggest that the expression responses of *CCNB1IP1* and *CTH* correlate with cellular changes following radiation exposure. Although these two genes were not known to be involved in IR response, they are associated with clinical outcomes in cancer: low levels of *CCNB1IP1* are associated with poor prognosis in breast and lung cancer (22), and high levels of *CTH* are correlated with resistance to anti-tumor drugs, such as methotrexate and cisplatin, which affect DNA synthesis or repair (23). These findings illustrate that our sample sizes are sufficiently large to allow us to identify genes with subtle yet biologically relevant roles in stress responses.

### Stress induces limited changes in gene interaction networks

**Figure 2.**
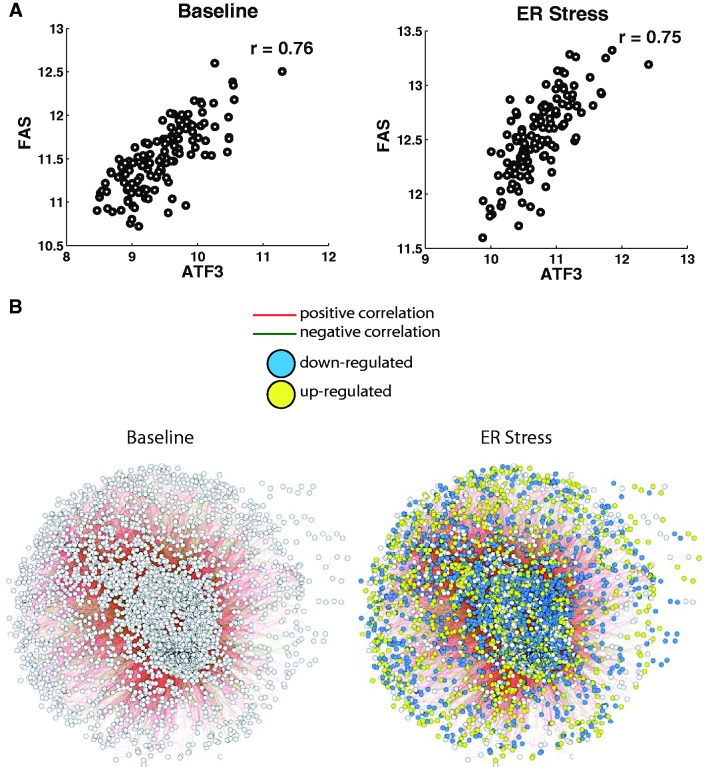
Coexpression analysis of ER stress-induced changes in gene expression. (**A**) *FAS* and *ATF3* are positively correlated among 131 individuals at baseline and on ER stress. (**B**) Coexpression network at baseline and on ER stress. Nodes represent genes and edges represent correlations between genes.

Next, we assessed whether correlations among genes change in response to stress. We built correlation networks, measured global properties of these networks and then studied the connections among genes. First, we calculated gene correlation using their expression levels across unrelated individuals (9,24,25). We analyzed each treatment separately; for each perturbation we examined all possible gene pairs (22 946 925 and 17 847 325 pairs for ER and IR stress, respectively). An example of a gene pair is shown in Figure 2A, which shows the coexpression of *ATF3* and *FAS* across 131 individuals before and after ER stress. Previously, we demonstrated that coexpression interactions capture known associations between genes and provide information on gene functions (4). Similarly, in this study, we found >20 000 genes that are significantly correlated (|r| > 0.60) and are involved in similar biological processes (significantly more than random pairs of genes, *P* < 10^−^^16^) (see Methods).

Pairwise coexpression interactions were assembled to construct gene networks, and network properties were measured. We built separate networks for different conditions and then compared the networks. We measured global network properties of coexpression networks constructed using different correlation thresholds (Supplementary Table S3). Figure 2B shows two coexpression networks: before and following ER stress. The clustering coefficient (10) of the ER stress response network is only slightly higher than that of the baseline network (0.56 versus 0.55, *P* = 0.03, Supplementary Table S3). The clustering coefficient measures the amount of ‘cliquishness’ among genes and represents the probability that two genes that are connected to a common gene are also connected to each other. The higher clustering coefficient in the gene network of ER stress suggests that genes may function in tighter gene clusters or ‘cliques’ on stress. Similarly, the gene network for cells after radiation exposure also had a slightly higher clustering coefficient than the network at baseline (0.51 versus 0.49, *P* = 0.001). Next, we examined ‘hub’ genes and found that many of the most highly connected genes at baseline remained as hubs after stress, consistent with the idea that there is limited remodeling of gene networks in response to stress. Of the 1000 most-connected genes at baseline, 79% of them are still among the 1000 most-connected genes after ER stress. Similarly, in the IR study, at least 75% of the hubs in the post-radiation exposure network are the same as those in baseline cells. Thus, gene networks representing stressed states had similar properties as those of cells at baseline.

**Figure 3.**
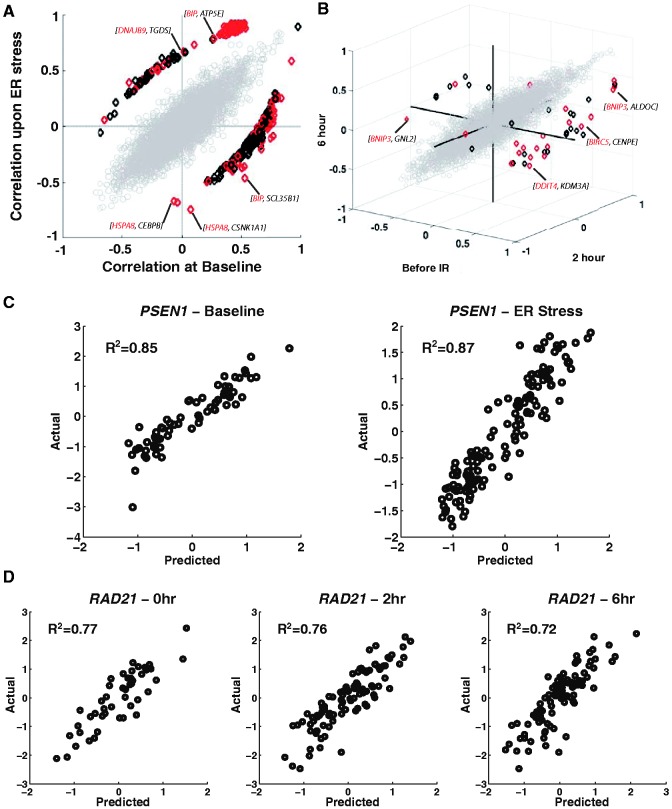
Most gene interactions do not change following stress. Gene pairs that do not alter coexpression patterns on stress are colored grey and a representative sampling of 5000 pairs are plotted. The few genes that alter interactions on ER or IR stress are represented by diamonds. Pairs with altered coexpression patterns that include at least one gene that has been implicated in previous studies in (**A**) ER stress or (**B**) IR stress are highlighted in red. A few examples are shown: the expression of (**C**) *PSEN1* and (**D**) *RAD21* are well predicted at baseline, and prediction by the model does not decline on stressed samples.

We then focused on pairwise coexpression interactions and found most gene interactions are not altered following stress. Of 22 946 925 (6775 choose 2) gene pairs examined, we found the correlation coefficient for >99% did not change significantly before and after ER stress (*P_c_* > 0.05; Fisher’s test of homogeneity with Bonferroni correction) (Figure 3A) (26). In the example mentioned earlier, *ATF3* and *FAS* show positive correlations both before and after ER stress, and these correlations were not significantly different (*r*_baseline_ = 0.76; *r*_ER stress_ = 0.75; *P* > 0.01; Figure 2A). Similarly on IR stress, >99% of the 17 847 325 gene pairs showed no evidence of altered coexpression patterns (*P_c_* > 0.05) (Figure 3B, Supplementary Figure S1).

After examining pairwise interactions, we extended our analysis to study the interactions of each gene with all other genes by a machine learning method involving SVMs. Briefly, a support vector regression was used to learn the relationships between each gene and all other genes and to predict expression level based on these relationships. A model that learns relationships from unstressed samples is expected to produce accurate predictions of the expression level of a gene following stress unless gene relationships change on stress. In such cases, a decline in model performance, measured using *R*^2^, is a proxy for identifying a gene that alters interactions on stress. Using non-parametric permutation testing to determine whether predictions were significantly worse in stressed samples, we found that the expression levels for >90% of genes in stressed samples were predicted with gene relationships at baseline (Supplementary Tables S4 and S5, *P* > 0.01), suggesting that most interactions are maintained on both ER and IR stress. For example, Figure 3C shows that the SVM model predicts *PSEN1* expression levels equally well for samples at baseline and following ER stress (*R*^2 ^= 0.85 versus *R*^2 ^= 0.87; *P* > 0.01) (ER stress); similarly, the prediction of *RAD21* expression (Figure 3D) is nearly the same for samples examined at 0, 2 and 6 h after IR treatment (*R*^2^ of 0.77, 0.76 and 0.72 at 0, 2 and 6 h, respectively; *P* > 0.01) (IR stress), implying that following stress, these genes maintain their relationships with other genes. We found 434 (6%, Supplementary Table S4) and 165 (3%, Supplementary Table S5) gene models showed significantly decreased performance following ER stress and IR stress, respectively (*P* < 0.01, permutation test; FDR = 14% for ER and FDR = 33% for IR as determined by permutation testing). Together, the findings suggest that although there are extensive changes in gene expression following ER stress and IR exposure, there are only limited changes in gene interactions.

### Genes that alter interactions are critical to stress response

**Figure 4.**
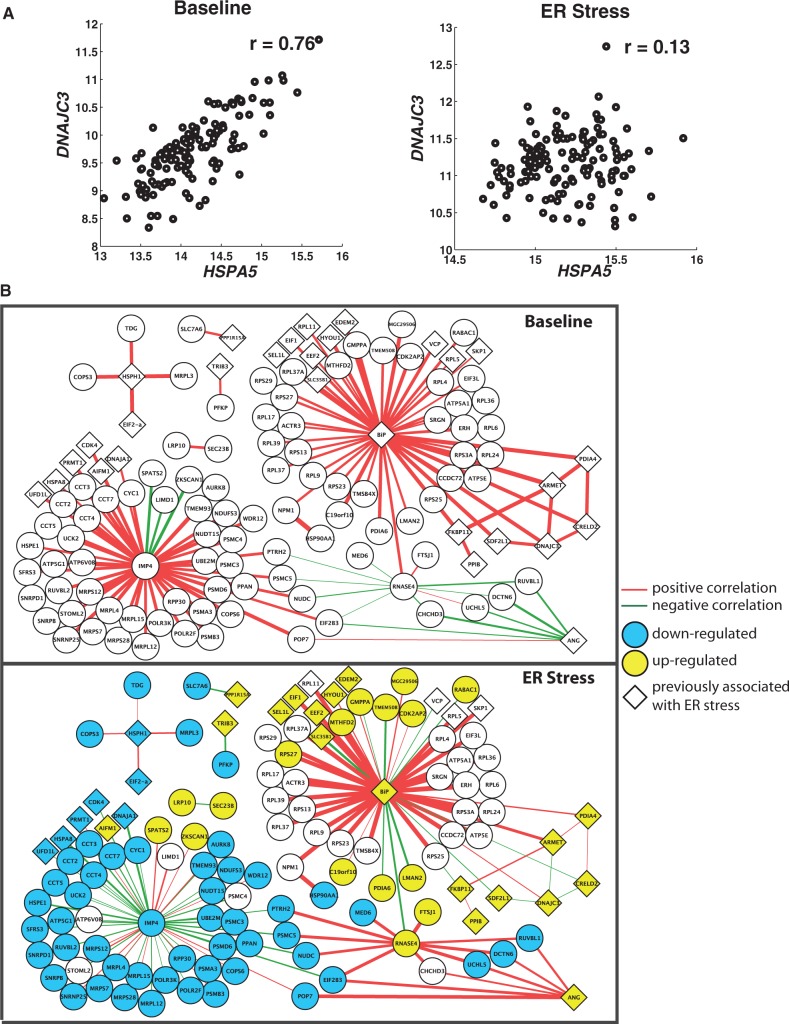
Gene pairs that altered coexpression relationships on ER stress. (**A**) Expression level of *HSPA5* (BIP) is less correlated with that of *DNAJC3* following ER stress. (**B**) Subnetworks of genes interact with different partners before and after ER stress. The thickness of the lines is proportional to the strength of the correlations.

Next, we examined those gene interactions that change in response to cellular stress. Coexpression analysis identified 284 gene pairs (comprising 257 unique genes) that altered interactions following ER stress (*P_c_* < 0.05, Fisher’s test of homogeneity with Bonferroni correction; Supplementary Table S6, Figure 4 and Supplementary Figure S2). Given that differences in the extent of individual variation in expression levels can influence gene correlations, we compared the variances of expression levels for the 257 genes that altered interactions in response to ER stress. We found that the variances of expression levels before and after stress were highly similar (average differences in their variance are ∼0.01), suggesting that changes in interactions are not due to differences in individual variability. Among the genes that changed interactions, there are 43 genes previously implicated in ER stress, including *HSPA5* (BIP) (Figure 4A), *PDIA4 (ERp72)*, *GADD34*, *VCP (p97)*, *EDEM2* and *TRIB3* (Figure 4B) (19,27–31). Gene Ontology (GO) analysis of these 257 genes showed enrichment for ‘protein folding’ (*P* < 9.3 × 10^−^^6^, Benjamini–Hochberg correction). Machine learning analysis identified 434 genes with altered interactions (*P* < 0.01, permutation test; FDR = 14%), with 47 genes implicated in ER stress. These genes showed GO functional enrichment for ‘response to unfolded protein’ (*P* = 0.005, Benjamini–Hochberg correction). Examining the overlap between results from coexpression and machine learning, we found a significant number of genes altered interactions using both methods (50 genes total; hypergeometric test, *P* = 5 × 10^−^^13^). These 50 genes showed specific enrichment for ‘response to unfolded protein’ (*P* = 8.4 × 10^−^^4^). ER stress has been implicated in neurodegenerative diseases such as Alzheimer’s and Parkinson’s, and genes associated with these diseases are among those with altered interactions. For example, *DHCR24* and *PIN1* are associated with Alzheimer’s disease. *DHCR24* encodes seladin−1, which protects against amyloid-beta peptide-induced toxicity, and is downregulated in affected neurons of patients with Alzheimer’s disease (32). *PIN1* is involved in processing of tau protein (33) and amyloid precursor protein (34) that accumulate in AD lesions. *TRAF6* is an ubiquitin ligase that has been shown to bind misfolded proteins and promote accumulation of protein aggregates in patients with Parkinson’s disease (35). Thus, some of the genes that acquired new correlation partners following ER stress have been associated with ER stress-related diseases.

**Figure 5.**
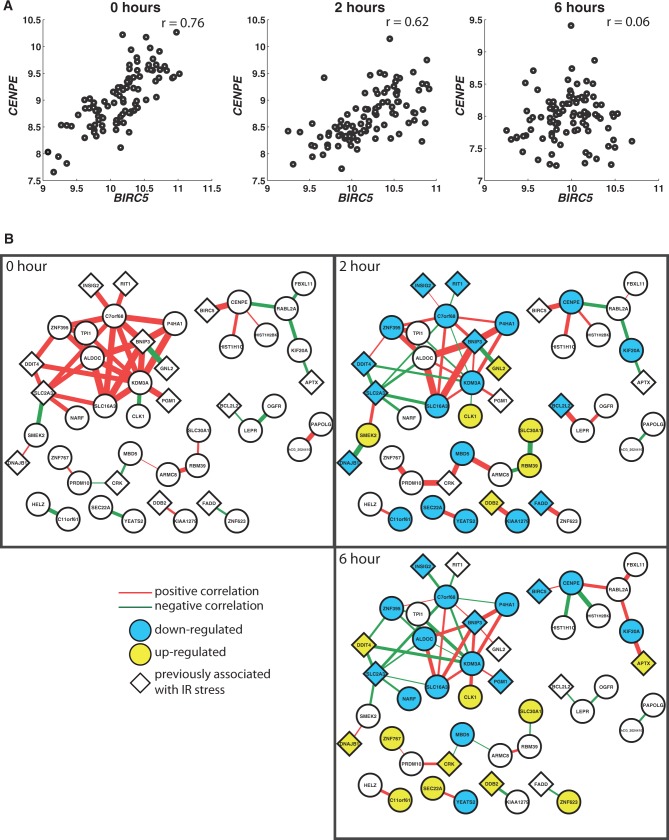
Gene pairs that altered coexpression relationships on IR stress. (**A**) Expression level of *BIRC5* (Survivin) is less correlated with that of *CENPE* in irradiated cells. (**B**) Subnetworks of genes that interact with different partners before and after IR stress. The thickness of the lines is proportional to the strength of the correlations.

Similarly, on IR stress, 50 gene pairs (comprising 46 unique genes) alter coexpression interactions (*P_c_* < 0.05, Fisher’s test of homogeneity with Bonferroni correction; Supplementary Table S7; as above the extent of individual differences in gene expression before and after IR exposure is similar and therefore cannot explain the changes in interactions). These included 13 genes previously implicated in IR stress, such as *BNIP3*, *FADD*, *DDB2* and *BIRC5* (Figure 5A and B) (36–40). Increased expression of *BIRC5*, which encodes antiapoptotic protein survivin, has been associated with increased resistance to IR therapy in rectal cancer (41) and is a potential drug target of lung cancer (42). Machine learning analysis identified 165 genes with altered interactions (*P* < 0.01, permutation test; FDR = 33%), with 47 previously implicated in IR stress, including cyclin B1 (*CCNB1*), *BNIP3*, *GADD45A*, *FAS*, *PLK1, ATF3, CDC20, DDB2* and *CDKN1A* (also called p21) (36,40,43–49). Genes that changed the most were enriched for ‘mitotic cell cycle’ (*P* = 0.004, Benjamini–Hochberg correction) and ‘microtubule-based process’ (*P* = 0.04, Benjamini–Hochberg correction); both processes are critical in the response to IR. These processes have also been targeted therapeutically to induce cell death in cancer cells (50).

To investigate the nature of interactions between 284 gene pairs that change following ER stress, we mined a database of human interactions using ConsensusPathDB, which compiles known protein–protein, genetic, signaling, drug–target, gene regulatory and metabolic pathway interactions (51). We found some gene pairs (∼9%, Supplementary Table S8) produce proteins that directly interact, such as *PDIA4* and *HSP90B1*, which are both known to function in the unfolded protein response. The remaining gene pairs interact through a third gene, via co-regulation, signaling or protein–protein interaction. For example, many gene pairs (25%, Supplementary Table S9) interact through *UBC3* that encodes an E2 ubiquitin-conjugating enzyme. It is known that the yeast homolog of XBP1 (Hac1p) is regulated by UBC3 (Pal *et al.* 2007); we postulate that altered interactions between genes in response to ER stress may be occurring either directly through UBC3 or through UBC3's effect on XBP1.

Of the 50 gene pairs that alter interactions in response to IR stress, we found that CENPE and BIRC5 directly interact at the protein level via formation of a kinetochore complex, whereas the remaining gene pairs interact through a third gene. For example, the interaction between *KDM3A* and *BNIP3* was found to be altered in our study. *HIF1A* interacts with both of these genes and is known to be activated on IR stress. HIF1A physically interacts with KDM3A, a histone demethylase, to regulate expression of various genes (52) including *BNIP3* (53,54). Thus, the interaction between KDM3A and BNIP3 may be altered via the activation of *HIF1A* in response to IR stress. Interestingly, although *KDM3A* is not known to play a role in IR stress, we found that in irradiated cells, it alters interactions with many genes (e.g. *DDIT4*, *P4HA1*, *PGM1*, *ALDOC, SLC16A3, TPI1 and CLK1*); thus, it may be important in IR stress through its physical interaction with *HIF1A*. These analyses do not identify the exact nature of interactions; however, they enable us to select candidates for functional follow-ups.

**Figure 6.**
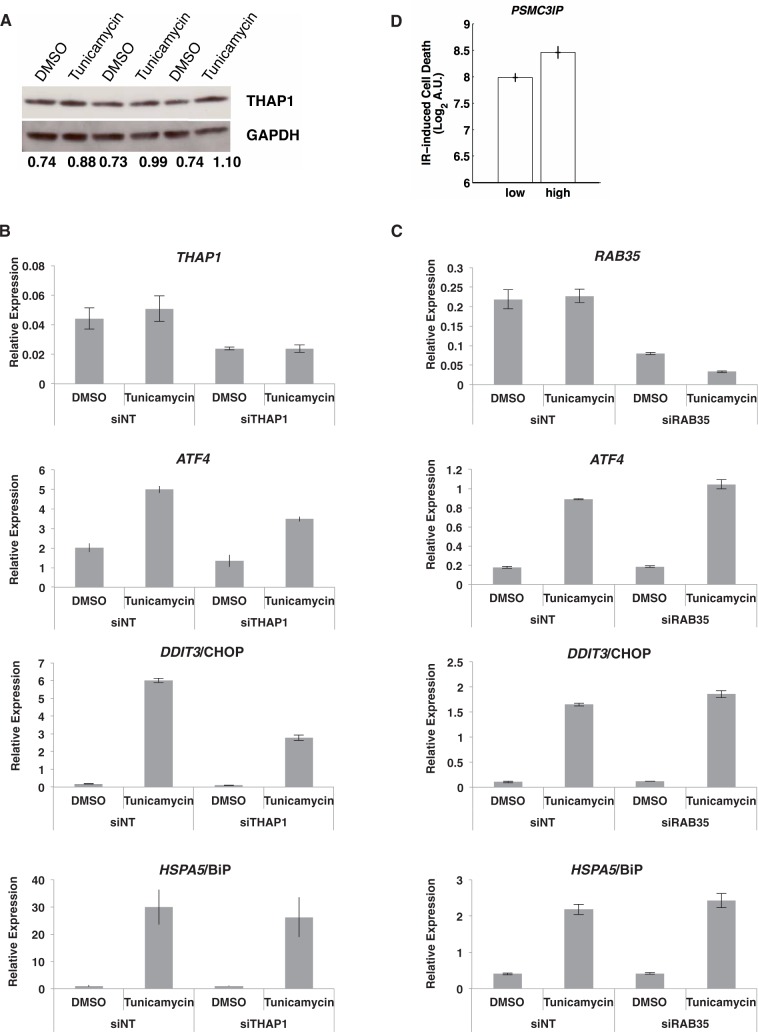
*THAP1* and *RAB35,* two ER stress-responsive genes and *PSMC3IP,* an IR stress-responsive gene identified in this study. (**A**) THAP1 protein levels are increased in response to ER stress induced by tunicamycin in primary human fibroblasts; results of biological triplicates. The ratio of THAP1 to glyceraldehyde 3-phosphate dehydrogenase is reported below each lane. (**B**) Knockdown of *THAP1* (average knockdown of 49%) (*P* = 0.03) resulted in attenuated induction of *ATF4* and *DDIT3*/CHOP (*P* < 0.005) but not *HSPA5*/BIP (*P* = 0.75). (**C**) Knockdown of *RAB35* (average knockdown 63%) (*P* = 0.005) resulted in increased induction of *ATF4* (*P* = 0.04) and *DDIT3*/CHOP (*P* = 0.05). (**D**) Induction of *PSMC3IP* is associated with IR-induced cell death in B-cells (*n* = 10). Error bars represent S.E.M.

After finding that many genes with altered interactions have known roles in stress response, we asked whether the remaining genes might also play key roles. We focused on 35 genes that showed altered interactions following ER stress (Supplementary Table S4), but without changes in messenger RNA levels (Supplementary Table S1). *THAP1* was the top candidate showing the greatest changes in gene interactions, so we studied it experimentally. First, we determined whether *THAP1* induction is also found in other human cells besides cultured B cells by measuring its protein levels in primary skin cells. Results from immunoblotting confirmed that *THAP1* protein expression increased following ER stress in human skin cells (Figure 6A) (*P* = 0.008, one-tailed *t*-test). Then, to examine the role of *THAP1* in ER stress, we knocked down *THAP1* by RNA interference and measured the expression of three well-known mediators of the ER stress response: *HSPA5*, *ATF4* and *DDIT3*. We found that knockdown of *THAP1* resulted in attenuated ER stress response where induction of *ATF4* and *DDIT3* was reduced compared with non-target control (Figure 6B) (*P* < 0.005). Thus, data on gene interactions rather than expression levels allowed us to identify a role for *THAP1* in ER stress response. In addition to *THAP1*, we examined two additional genes that are part of the Ras/Raf/Rho pathway: *ARHGAP1* and *RAB35*. These were also among the genes that change interactions without changing expression. We examined B cells from two different individuals and knocked down *ARHGAP1* and *RAB35* using siRNA; then we assessed response to ER stress by measuring *HSPA5*, *ATF4* and *DDIT3.* Knockdown of *RAB35* led to an increase in the induction of *ATF4* and *DDIT3* in response to ER stress (Figure 6C) (*P* ≤ 0.05), suggesting that *RAB35* negatively regulates ER stress response. Knockdown of *ARHGAP1* showed similar trends, but the effects on *ATF4* and *DDIT3* were not significant with just two samples. Together, these experimental results show that genes can be involved in the ER stress response by altering their interacting gene partners but without changing their expression levels. Thus, studying gene expression levels alone may not provide a comprehensive view of the ER stress response.

Similarly, we examined genes that alter their interactions with other genes following IR stress (Supplementary Table S5). We compared radiation-induced changes in expression of these genes with caspase measurements of radiation-induced cell death from the same subjects. We found that among 165 genes showing altered interactions, the induction or repression of 19 (12%) of these genes is significantly correlated with the extent of cell death following radiation exposure (*P* < 0.05). One of these genes, *PSMC3IP*, is a factor that ensures proper homologous pairing during meiosis (55). It has not been linked to DNA damage or response to IR. In our data, it interacts with different sets of genes at baseline and post-radiation (Supplementary Table S5). *PSMC3IP* was correlated (|r| > 0.6) with 27 genes at baseline, and these genes showed no significant enrichment for any biological process (*P_c_* = 0.56). However, 6 h after IR, *PSMC3IP* was correlated to 25 genes that are enriched for roles in DNA replication (*P*_c_ = 0.08), including *RFC4*, *RPA3* and *PRIM1*. We find that cells from individuals who downregulated the expression of *PSMC3IP* show less cell death compared with those who upregulated its expression (Figure 6D) (*P* = 0.002, *t*-test). Taken together, these findings suggest that genes that alter interactions have important roles in the human stress response.

### Stress-specific alterations in gene interactions

We next examined whether changes in gene expression and interactions are stress-specific. We compared genes that change expression following IR or ER stress and found that the expression levels of ∼1500 genes (25% of the genes studied) changed in response to both stresses. The shared genes were not enriched for any particular functions. Previous studies have also demonstrated that ∼25–30% of changed genes are shared across multiple stressors (56). Next, we compared the genes that changed interactions following IR and ER stress. In contrast to genes that change expression, none of the genes that changed coexpression interactions were shared between the two stresses, suggesting that changes in gene interactions are highly stress-specific. As described earlier, there were 257 genes that were correlated with a different set of genes (Supplementary Table S6) specifically following ER stress; these genes showed enrichment for ‘response to unfolded protein’. And there were 46 genes that changed interactions only following IR exposure (Supplementary Table S7); these include known IR-stress response genes *DDB2*, *FADD* and *BIRC5*. When we looked at genes identified using machine learning analysis (SVM), we saw the same pattern; only 23 genes (<1% of the genes studied) changed interactions following both ER and IR stress (Supplementary Table S10). Compared with the 25% of genes that showed changes in expression levels following ER or IR stress, far fewer genes changed interactions (<1%) following both stresses; however, the genes that changed interactions were enriched for those that are critical to the stress response and unique to each stress. Together, these data suggest that genes that alter interactions are predominantly stress-specific.

## DISCUSSION

Cells respond to perturbations by changing gene expression levels and altering interactions among genes. Studying changes in expression levels is straightforward, but examining the extent to which cells rewire gene network connections is more difficult. However, knowledge of these gene interactions provides a more comprehensive view of cellular response and is important for the development of interventions that improve response to perturbations. Here, we identified genes that changed in expression levels and in their interactions with other genes following ER stress or exposure to IR. These cellular stresses have clinical relevance with respect to diseases in which ER stress has been implicated and to the use of IR as a treatment for cancer.

Our analyses revealed that many genes change expression levels, and fewer genes change interactions, but genes that change interactions tend to be specific to the particular stress response. First, our results show extensive changes in gene expression following perturbation; many (>80%) of the stress-responsive genes showed consistent but modest levels of induction or repression across our ∼100 individuals. Second, coexpression network analysis revealed that hubs (genes with numerous links) maintained many of their connections, and there was an increase in clustering among genes following stress. Third, although many genes did not change interactions, those that did played critical and specific roles in ER and IR stress response. Moreover, genes with key roles in ER stress-related diseases such as Alzheimer’s disease and Parkinson’s disease showed altered gene interactions in response to ER stress.

Finally, we identified genes that alter interactions without altering expression. These stress-responsive genes would have been missed if we had focused only on gene expression changes. One of these genes, *THAP1*, which we showed, promotes ER stress response through *ATF4* and *DDIT3*. This gene is mutated in patients with dystonia, and initial studies suggest the pathogenesis may involve ER stress (57). Taken together, for the most part, cells use preexisting network connections to respond to stress. However, some new connections are made. Identifying genes with altered connections may provide specific therapeutic targets for diseases that result from dysfunction in cellular responses to stress.

Our results are in agreement with findings in yeast where genes that altered epistasis interactions were found to be critical (58). Bandyopadhyay *et al.* examined changes in epistasis interactions in 418 yeast genes in response to MMS-induced DNA damage. These 418 genes were selected to give coverage of kinases, phosphatases, transcription factors and known DNA repair factors. They found 873 differential interactions of ∼80 000 examined (1% of interactions change), with many interactions showing enrichment for genes functioning in the DNA damage response. Although they identified a slightly higher percentage of altered interactions in yeast compared with our study in humans, this difference may be due to the genes included in their analysis: they selected genes with known roles in DNA damage response, whereas our study analyzed all expressed genes in the B cell transcriptome. Nevertheless, the main finding in yeast is recapitulated in humans: genes showing altered interactions tend to be enriched for critical players in the stress response.

Our findings in two distinct stresses suggest that cells respond to stress by inducing extensive changes in gene expression and selective changes in gene interactions. While many genes that change expression are shared between the two stresses, the subset of genes engaging in dynamic interactions tends to be stress-specific. Diseases can result from inefficient responses to stresses. Therapies that augment appropriate stress response could be effective long-term treatments. However, developing such therapies has been difficult, as many of the response pathways are shared across stresses. Our findings that identified stress-specific responsive genes provide potential candidates for therapeutic interventions.

Our results illustrate that individual variation in gene expression response uncovers the complexity of cellular responses. Future work that elucidates these complex interactions will be important for development of interventions based on understanding disease-specific pathways rather than genes in isolation.

## SUPPLEMENTARY DATA

Supplementary Data are available at NAR Online.

## FUNDING

The National Institutes of Health (NIH) and the Howard Hughes Medical Institute (HHMI). Funding for open access charge: NIH and HHMI.

*Conflict of interest statement*. None declared.

## Supplementary Material

Supplementary Data
