# Supplementary material for: Stress-induced changes in gene interactions in human cells
Source: Nucleic Acids Res. 2013 Oct 28;42(3):1757–71. doi: 10.1093/nar/gkt999 (PMC3919594; doi:10.1093/nar/gkt999)
Supplement: Supplementary Data [file supp_42_3_1757__index.html]

Stress-induced changes in gene interactions in human cells — Stress-induced changes in gene interactions in human cells — Supplementary Data 

# Stress-induced changes in gene interactions in human cells

## Supplementary Data

files

**Files in this Data Supplement:**

- Supplementary Data - pdf file
