# Supplementary material for: Stress-induced changes in gene interactions in human cells
Source: Nucleic Acids Res. 2013 Oct 28;42(3):1757–71. doi: 10.1093/nar/gkt999 (PMC3919594; doi:10.1093/nar/gkt999)
Supplement: Supplementary Data [file supp_gkt999_nar-01501-n-2013-File003.pdf]

Supplemental Material For

Stress-induced changes in gene interactions in human cells

Renuka R. Nayak, William E. Bernal, Jessica Lee, Michael J. Kearns, Vivian G. Cheung

Figures

**Page 2**	**Supplemental Figure 1**: A gene pair that does not alter interactions upon IR stress.
**Page 3**	**Supplemental Figure 2**: All genes with altered connections before and after ER stress.
**Page 4**	**Supplemental Figure 3:** Coexpression network at baseline and upon IR stress.

Tables

**Page 5 – 130**	**Supplemental Table 1**: Changes in gene expression levels induced by tunicamycin (ER stress).
**Page 131 – 230**	**Supplemental Table 2**: Changes in gene expression levels induced by ionizing radiation 2 and 6 hours after treatment.
**Page 231 – 232**	**Supplemental Table 3**: Properties of coexpression networks using different correlation thresholds.
**Page 233 – 358**	**Supplemental Table 4**: SVM modeling results upon endoplasmic reticulum stress.
**Page 359 – 471**	**Supplemental Table 5**: SVM modeling results upon ionizing radiation stress.
**Page 472 – 476**	**Supplemental Table 6**: Gene pairs that are differentially coexpressed following ER stress.
**Page 477**	**Supplemental Table 7**: Gene pairs that are differentially coexpressed following IR stress.
**Page 478**	**Supplemental Table 8.** Genes that are differentially coexpressed following ER stress that are known to have direct protein-protein interactions
**Page 479-480**	**Supplemental Table 9.** Gene pairs that are differentially coexpressed after ER stress that interact via UBC3
**Page 481**	**Supplemental Table 10.** Gene pairs that alter interactions after both ER and IR stress

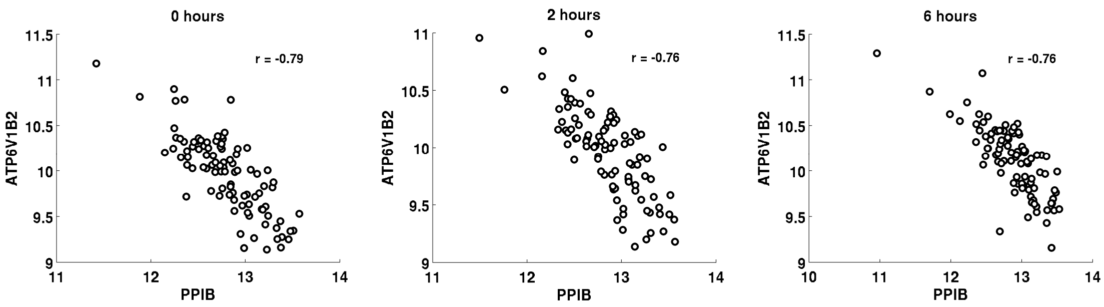

Supplemental Figure 1. A gene pair that does not alter interactions upon IR stress.

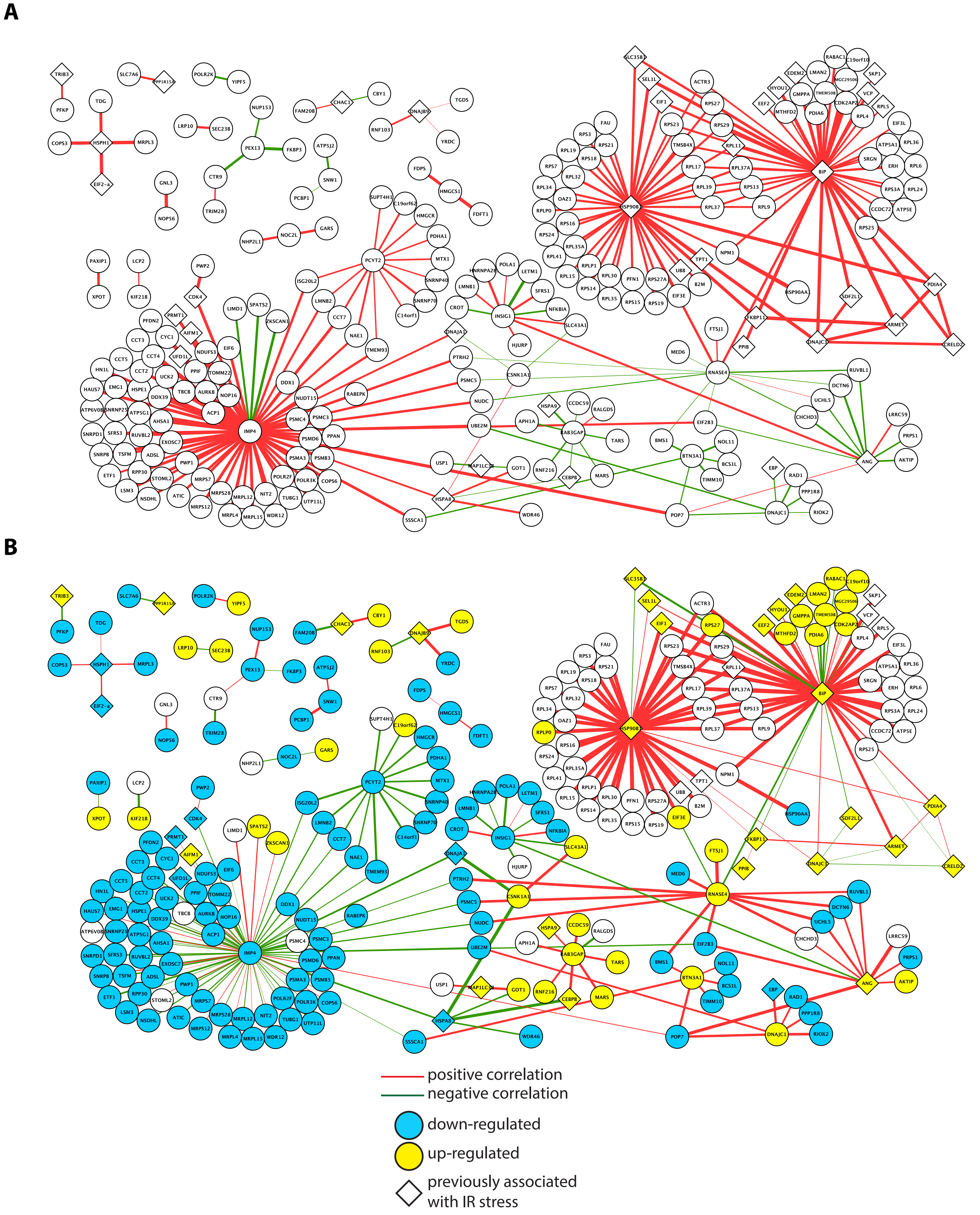

Supplemental Figure 2. All genes with altered connections (A) before and (B) after ER stress.

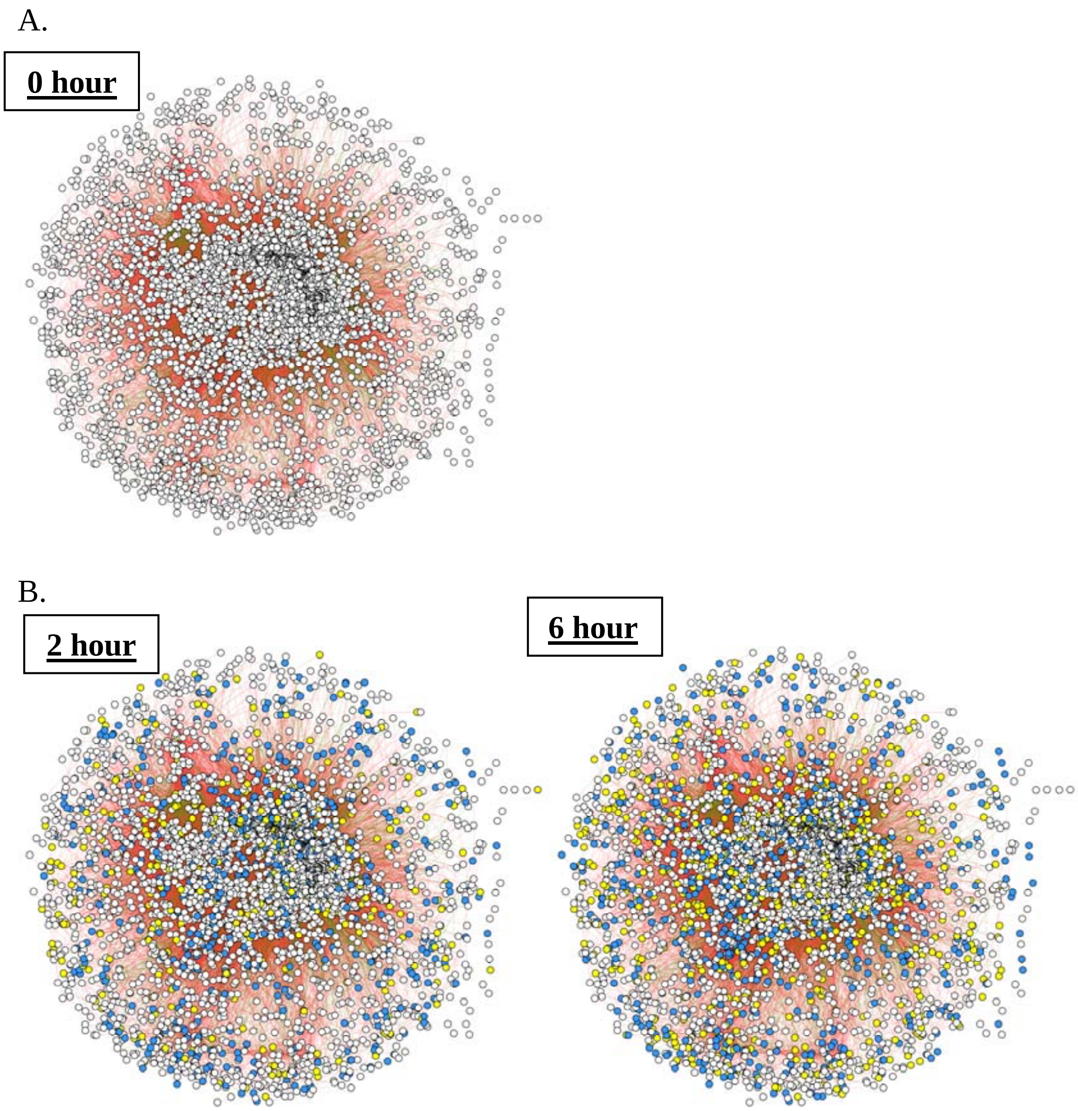

Supplemental Figure 3. Coexpression network at baseline (A) and upon IR stress (B). Nodes represent genes and edges represent positive (red) and negative (green) correlations between genes. Only showing connections $|r|>0.60$. Genes that increase or decrease upon IR stress are colored yellow and blue, respectively.

Supplemental Table 1

Supplemental Table 1. Changes in gene expression levels induced by tunicamycin (ER stress)				
**Affymetrix ID**	**Gene Symbol**	**# of articles gene associated with ER stress in Pubmed**	**Fold Change**	**$P_{corrected}$ value**
1007_s_at	DDR1	0	-1.16	4.51E-12
1053_at	RFC2	0	-1.35	3.54E-39
117_at	HSPA6	1		N.S.
1405_i_at	CCL5	0	1.08	4.27E-06
1487_at	ESRRA	0	-1.14	<1.00E-120
1729_at	TRADD	2	1.44	3.37E-39
1861_at	BAD	15	1.23	2.19E-32
200000_s_at	PRPF8	0		N.S.
200001_at	CAPNS1	0	-1.04	1.39E-02
200002_at	RPL35	0		N.S.
200003_s_at	RPL28	0	1.05	4.16E-03
200004_at	EIF4G2	0	1.04	8.98E-03
200005_at	EIF3D	0	-1.09	<1.00E-120
200006_at	PARK7	1	-1.08	<1.00E-120
200007_at	SRP14	0		N.S.
200009_at	GDI2	0	-1.05	1.09E-07
200010_at	RPL11	1		N.S.
200013_at	RPL24	0		N.S.
200015_s_at	SEPT2	0	-1.06	1.16E-04
200017_at	RPS27A	0		N.S.
200018_at	RPS13	0		N.S.
200019_s_at	FAU	0		N.S.
200020_at	TARDBP	0	-1.35	1.67E-63
200021_at	CFL1	0		N.S.
200024_at	RPS5	0		N.S.
200026_at	RPL34	0		N.S.
200027_at	NARS	0	1.38	2.48E-74
200029_at	RPL19	0		N.S.
200030_s_at	SLC25A3	0		N.S.
200032_s_at	RPL9	0		N.S.
200033_at	DDX5	0	-1.09	3.76E-12
200034_s_at	RPL6	0		N.S.
200035_at	DULLARD	0		N.S.
200036_s_at	RPL10A	0	1.06	3.67E-05
200038_s_at	RPL17	0		N.S.
200039_s_at	PSMB2	0	-1.10	<1.00E-120
200040_at	KHDRBS1	0	-1.17	1.26E-43
200041_s_at	BAT1	0	-1.05	2.37E-05
200042_at	C22orf28	0	-1.17	8.92E-23
200043_at	ERH	0		N.S.
200044_at	SFRS9	0	1.04	1.18E-02
200045_at	ABCF1	0	-1.37	1.00E-76
200046_at	DAD1	0	1.13	2.36E-20
200048_s_at	JTB	0	1.11	1.91E-28
200049_at	MYST2	0	1.09	3.01E-12
200050_at	ZNF146	0		N.S.
200051_at	SART1	0		N.S.
200052_s_at	ILF2	1		N.S.
200053_at	SPAG7	0	1.08	2.09E-03

Supplemental Table 1

200054_at	ZNF259	0	-1.30	4.66E-41
200055_at	TAF10	0	-1.06	4.80E-08
200056_s_at	C1D	0	-1.20	9.17E-23
200059_s_at	RHOA	3		N.S.
200060_s_at	RNPS1	0	-1.27	7.92E-49
200061_s_at	RPS24	0		N.S.
200062_s_at	RPL30	0		N.S.
200063_s_at	NPM1	0		N.S.
200064_at	HSP90AB1	1	-1.34	1.71E-43
200065_s_at	ARF1	0	1.20	5.62E-34
200066_at	IK	0	-1.13	7.85E-22
200071_at	SMNDC1	0	-1.06	1.00E-08
200073_s_at	HNRNPD	0	-1.39	1.18E-44
200074_s_at	RPL14	0		N.S.
200077_s_at	OAZ1	0		N.S.
200078_s_at	ATP6V0B	0		N.S.
200080_s_at	H3F3A	0	-1.10	<1.00E-120
200082_s_at	RPS7	0		N.S.
200084_at	C11orf58	0	1.03	6.15E-03
200085_s_at	TCEB2	0		N.S.
200089_s_at	RPL4	1		N.S.
200090_at	FNTA	0		N.S.
200091_s_at	RPS25	0		N.S.
200092_s_at	RPL37	0		N.S.
200093_s_at	HINT1	0		N.S.
200096_s_at	ATP6V0E1	0		N.S.
200099_s_at	RPS3A	0		N.S.
200593_s_at	HNRNPU	0	-1.64	5.77E-86
200596_s_at	EIF3A	0		N.S.
200599_s_at	HSP90B1	2	1.48	1.11E-43
200600_at	MSN	0	-1.07	6.16E-07
200601_at	ACTN4	0	-1.75	3.44E-52
200604_s_at	PRKAR1A	0		N.S.
200607_s_at	RAD21	0	1.10	1.34E-02
200609_s_at	WDR1	0	-1.11	4.66E-11
200610_s_at	NCL	2	-1.26	2.06E-36
200613_at	AP2M1	0		N.S.
200615_s_at	AP2B1	0	1.30	1.24E-45
200616_s_at	MLEC	0	1.28	6.71E-36
200618_at	LASP1	0		N.S.
200619_at	SF3B2	0	-1.12	<1.00E-120
200620_at	TMEM59	0	1.38	4.82E-52
200621_at	CSRP1	0	-1.19	1.73E-28
200623_s_at	CALM3	0	-1.12	<1.00E-120
200626_s_at	MATR3	0	-1.15	7.62E-38
200627_at	PTGES3	0	-1.26	1.86E-53
200629_at	WARS	0	2.93	7.92E-84
200632_s_at	NDRG1	0	1.23	1.87E-03
200633_at	UBB	1		N.S.
200634_at	PFN1	0		N.S.
200642_at	SOD1	11	-1.10	<1.00E-120
200644_at	MARCKSL1	0	-1.54	1.61E-56
200645_at	GABARAP	0	1.05	2.77E-06
200649_at	NUCB1	2	1.66	1.20E-59

Supplemental Table 1

200650_s_at	LDHA	0	-1.26	2.00E-35
200652_at	SSR2	0	1.64	4.82E-72
200654_at	P4HB	1	1.69	7.24E-64
200657_at	SLC25A5	0	-1.10	5.23E-18
200658_s_at	PHB	1	-1.32	1.62E-63
200660_at	S100A11	0	1.23	2.50E-21
200661_at	CTSA	2	-1.15	<1.00E-120
200663_at	CD63	1	-1.26	4.43E-57
200665_s_at	SPARC	1	-1.63	1.09E-42
200666_s_at	DNAJB1	4	-2.16	5.87E-84
200668_s_at	UBE2D3	0		N.S.
200670_at	XBP1	224	1.73	2.56E-41
200673_at	LAPTM4A	0	1.29	3.95E-48
200674_s_at	RPL32	0		N.S.
200675_at	CD81	1	-1.15	2.21E-29
200677_at	PTTG1IP	0	1.48	2.70E-70
200681_at	GLO1	0	-1.36	1.91E-76
200682_s_at	UBE2L3	3	-1.34	2.22E-61
200687_s_at	SF3B3	0	-1.65	1.35E-84
200692_s_at	HSPA9	1	1.25	1.95E-48
200695_at	PPP2R1A	0	-1.25	<1.00E-120
200696_s_at	GSN	0		N.S.
200697_at	HK1	0	-1.39	2.60E-26
200699_at	KDELR2	0	1.74	7.23E-59
200701_at	NPC2	1		N.S.
200702_s_at	DDX24	0	-1.56	1.22E-41
200703_at	DYNLL1	0	-1.28	2.01E-47
200705_s_at	EEF1B2	0	1.05	3.11E-04
200706_s_at	LITAF	0		N.S.
200707_at	PRKCSH	0	1.30	1.95E-38
200708_at	GOT2	0	-1.16	2.64E-25
200709_at	FKBP1A	0	1.07	1.29E-06
200710_at	ACADVL	0	1.22	2.30E-29
200713_s_at	MAPRE1	0	-1.14	3.37E-33
200718_s_at	SKP1	1		N.S.
200720_s_at	ACTR1A	0	-1.23	1.27E-30
200722_s_at	CAPRIN1	0	-1.54	1.33E-34
200726_at	PPP1CC	0	1.06	1.50E-12
200729_s_at	ACTR2	0		N.S.
200733_s_at	PTP4A1	0		N.S.
200734_s_at	ARF3	0	-1.07	1.25E-07
200736_s_at	GPX1	0	-1.06	1.24E-07
200740_s_at	SUMO3	0	-1.41	1.03E-68
200741_s_at	RPS27	0	1.06	1.37E-05
200743_s_at	TPP1	0		N.S.
200746_s_at	GNB1	0	1.10	2.95E-18
200748_s_at	FTH1	1	1.20	2.61E-33
200749_at	RAN	5	-1.36	8.00E-55
200751_s_at	HNRNPC	0	-1.13	<1.00E-120
200752_s_at	CAPN1	2		N.S.
200755_s_at	CALU	1	1.30	3.34E-21
200761_s_at	ARL6IP5	0	1.04	3.81E-03
200762_at	DPYSL2	2	1.21	8.96E-23
200763_s_at	RPLP1	0		N.S.

Supplemental Table 1

200766_at	CTSD	0	1.26	<1.00E-120
200767_s_at	FAM120A	0	-1.08	4.75E-02
200770_s_at	LAMC1	0		N.S.
200775_s_at	HNRNPK	0	-1.11	1.81E-25
200776_s_at	BZW1	0	-1.39	7.54E-46
200779_at	ATF4	145	2.52	3.30E-105
200782_at	ANXA5	4	1.09	<1.00E-120
200783_s_at	STMN1	0	-1.18	9.86E-30
200786_at	PSMB7	0	-1.08	5.84E-20
200788_s_at	PEA15	0	-1.10	2.82E-10
200789_at	ECH1	0	1.07	2.56E-04
200790_at	ODC1	0	-1.28	4.34E-49
200792_at	XRCC6	0	-1.14	4.95E-22
200793_s_at	ACO2	0		N.S.
200802_at	SARS	5	2.53	8.30E-116
200804_at	TMBIM6	0	1.71	1.53E-85
200805_at	LMAN2	1	1.49	1.00E-72
200806_s_at	HSPD1	1	-1.23	4.40E-29
200808_s_at	ZYX	0	-1.20	2.70E-22
200811_at	CIRBP	0	1.31	7.41E-38
200812_at	CCT7	0	-1.36	1.22E-66
200814_at	PSME1	1		N.S.
200815_s_at	PAFAH1B1	0	1.06	9.26E-03
200818_at	ATP5O	0	-1.04	1.94E-02
200819_s_at	RPS15	0		N.S.
200820_at	PSMD8	0		N.S.
200824_at	GSTP1	0		N.S.
200825_s_at	HYOU1	2	3.11	3.42E-85
200826_at	SNRPD2	0	-1.12	4.84E-18
200827_at	PLOD1	0	1.07	2.41E-02
200828_s_at	ZNF207	0	-1.21	2.84E-44
200830_at	PSMD2	0	-1.21	1.55E-40
200833_s_at	hCG_1757335	0	1.07	1.56E-09
200834_s_at	RPS21	0		N.S.
200837_at	BCAP31	4	-1.07	9.54E-06
200840_at	KARS	0	-1.09	2.90E-19
200842_s_at	EPRS	0	1.32	4.29E-29
200845_s_at	PRDX6	1	-1.26	1.57E-53
200846_s_at	PPP1CA	0		N.S.
200847_s_at	TMEM66	0	1.55	2.96E-66
200851_s_at	KIAA0174	0		N.S.
200853_at	H2AFZ	0		N.S.
200854_at	NCOR1	2	1.21	5.44E-32
200855_at	C20orf191	0		N.S.
200860_s_at	CNOT1	0		N.S.
200862_at	DHCR24	1	-2.70	1.17E-90
200863_s_at	RAB11A	0	-1.14	3.88E-25
200870_at	STRAP	0	-1.05	1.49E-06
200871_s_at	PSAP	0	1.12	<1.00E-120
200873_s_at	CCT8	0	-1.08	<1.00E-120
200875_s_at	NOP56	0	-1.74	1.18E-84
200877_at	CCT4	0	-1.26	4.25E-43
200881_s_at	DNAJA1	1	-2.14	5.39E-76
200882_s_at	PSMD4	0		N.S.

Supplemental Table 1

200885_at	RHOC	0	-1.08	2.80E-04
200886_s_at	PGAM1	0	-1.21	1.74E-43
200891_s_at	SSR1	0	1.57	1.67E-54
200892_s_at	TRA2B	0	-1.22	4.44E-37
200894_s_at	FKBP4	0	-3.29	9.91E-100
200900_s_at	M6PR	0	-1.14	<1.00E-120
200902_at	SEP15	1	1.38	1.16E-51
200903_s_at	AHCY	0	-1.12	2.22E-22
200904_at	HLA-E	0	1.55	1.21E-61
200908_s_at	RPLP2	0		N.S.
200910_at	CCT3	0	-1.51	7.02E-75
200911_s_at	TACC1	0		N.S.
200912_s_at	EIF4A2	0	-1.24	5.58E-43
200913_at	PPM1G	0	-1.31	9.77E-36
200918_s_at	SRPR	0	1.27	6.73E-30
200919_at	PHC2	0	-1.20	1.04E-18
200920_s_at	BTG1	0	1.28	2.07E-30
200922_at	KDELR1	1	1.26	5.71E-27
200924_s_at	SLC3A2	0	4.85	2.56E-112
200925_at	COX6A1	0		N.S.
200926_at	RPS23	0		N.S.
200928_s_at	RAB14	0		N.S.
200932_s_at	DCTN2	0	1.08	4.03E-08
200934_at	DEK	0		N.S.
200935_at	CALR	3	2.23	7.37E-70
200936_at	RPL8	0		N.S.
200937_s_at	RPL5	1		N.S.
200941_at	HSBP1	0	-1.08	3.22E-04
200944_s_at	HMGN1	0	-1.03	2.47E-03
200945_s_at	SEC31A	0	1.37	4.97E-57
200947_s_at	GLUD1	0	1.17	<1.00E-120
200948_at	MLF2	0	-1.19	7.48E-25
200951_s_at	CCND2	0	-1.24	1.44E-22
200955_at	IMMT	0	1.06	1.73E-04
200957_s_at	SSRP1	0		N.S.
200958_s_at	SDCBP	0		N.S.
200959_at	FUS	0	-1.18	1.18E-25
200961_at	SEPHS2	0	1.24	3.64E-33
200964_at	UBA1	0	-1.15	9.56E-28
200965_s_at	ABLIM1	0	1.26	5.71E-37
200967_at	PPIB	1	1.36	5.11E-47
200971_s_at	SERP1	1	1.24	2.84E-40
200973_s_at	TSPAN3	0		N.S.
200975_at	PPT1	4		N.S.
200977_s_at	TAX1BP1	0	1.30	2.05E-66
200978_at	MDH1	0	-1.05	1.86E-03
200980_s_at	PDHA1	0	-1.11	<1.00E-120
200982_s_at	ANXA6	0		N.S.
200984_s_at	CD59	0	-1.49	7.12E-59
200989_at	HIF1A	14	1.07	5.41E-18
200990_at	TRIM28	0	-1.38	1.58E-53
200991_s_at	SNX17	0	-1.07	2.30E-02
200994_at	IPO7	0	-1.42	8.34E-48
200997_at	RBM4	0	1.05	1.40E-07

Supplemental Table 1

200998_s_at	CKAP4	0	1.26	7.51E-22
201000_at	AARS	0	2.55	1.89E-95
201002_s_at	TMEM189-UBE2V1	0	-1.12	8.93E-35
201004_at	SSR4	0	1.31	2.87E-44
201005_at	CD9	0	-1.53	2.53E-26
201007_at	HADHB	0	1.12	4.30E-26
201008_s_at	TXNIP	1	1.85	3.60E-33
201011_at	RPN1	0	1.81	3.99E-83
201012_at	ANXA1	0	1.80	4.17E-40
201017_at	EIF1AX	0	-1.29	1.30E-41
201019_s_at	EIF1AP1	0	-1.17	6.64E-19
201020_at	YWHAH	1	-1.70	2.19E-85
201021_s_at	DSTN	0	1.65	1.81E-60
201023_at	TAF7	0	-1.05	1.50E-06
201028_s_at	CD99	0	1.08	3.76E-06
201031_s_at	HNRNPH1	0		N.S.
201032_at	BLCAP	0	1.10	7.52E-13
201037_at	PFKP	0	-1.90	6.14E-60
201039_s_at	RAD23A	1	-1.45	3.62E-46
201041_s_at	DUSP1	1	-1.19	<1.00E-120
201049_s_at	RPS18	0		N.S.
201050_at	PLD3	0	1.40	1.93E-28
201051_at	ANP32A	0		N.S.
201053_s_at	PSMF1	0		N.S.
201055_s_at	HNRNPA0	0	-1.16	1.59E-25
201056_at	GOLGB1	0	1.63	1.39E-51
201063_at	RCN1	0	1.44	7.42E-64
201064_s_at	PABPC4	0	1.11	<1.00E-120
201066_at	CYC1	2	-1.19	1.20E-36
201068_s_at	PSMC2	0	-1.11	7.27E-07
201074_at	SMARCC1	0	-1.12	4.18E-23
201077_s_at	NHP2L1	0		N.S.
201078_at	TM9SF2	0	1.20	1.60E-26
201079_at	SYNGR2	0		N.S.
201081_s_at	PIP4K2B	0	-1.27	9.78E-12
201085_s_at	SON	4	-1.33	8.40E-24
201087_at	PXN	0	1.30	7.43E-09
201088_at	KPNA2	0	-1.20	2.44E-23
201089_at	ATP6V1B2	1	-1.08	1.70E-06
201091_s_at	CBX3	0	-1.26	2.61E-23
201092_at	RBBP7	0	-1.17	4.95E-42
201094_at	RPS29	0		N.S.
201095_at	DAP	1	1.27	7.30E-31
201097_s_at	ARF4	0	1.49	2.17E-51
201098_at	COPB2	0	1.18	7.95E-28
201099_at	USP9X	0	1.07	<1.00E-120
201101_s_at	BCLAF1	0		N.S.
201102_s_at	PFKL	0	-1.12	<1.00E-120
201106_at	GPX4	0	1.06	6.08E-03
201112_s_at	CSE1L	0	-1.66	6.96E-90
201113_at	TUFM	0	-1.08	8.14E-18
201115_at	POLD2	0	-1.33	4.35E-30
201118_at	PGD	0	-1.36	3.39E-39
201119_s_at	COX8A	0		N.S.

Supplemental Table 1

201121_s_at	PGRMC1	0	-1.28	7.02E-46
201126_s_at	MGAT1	0		N.S.
201128_s_at	ACLY	0	-1.43	4.22E-47
201129_at	SFRS7	0	-1.91	1.01E-101
201132_at	HNRNPH2	0	-1.42	2.46E-51
201133_s_at	PJA2	0	1.33	1.38E-45
201135_at	ECHS1	0	-1.05	1.77E-06
201136_at	PLP2	0	1.12	<1.00E-120
201137_s_at	HLA-DPB1	0		N.S.
201139_s_at	SSB	0	-1.58	1.30E-65
201141_at	GPNMB	0	-1.19	1.81E-11
201144_s_at	EIF2S1	0	-1.45	5.69E-66
201145_at	HAX1	1	2.09	2.58E-109
201146_at	NFE2L2	9	1.32	8.58E-50
201155_s_at	MFN2	0	-1.25	2.77E-31
201156_s_at	RAB5C	0	-1.24	2.30E-43
201158_at	NMT1	0		N.S.
201165_s_at	PUM1	0		N.S.
201170_s_at	BHLHE40	0	-1.14	4.73E-09
201174_s_at	TERF2IP	0	1.25	1.45E-46
201175_at	TMX2	0	1.20	2.89E-37
201176_s_at	ARCN1	0	1.25	2.05E-37
201177_s_at	UBA2	0	-1.16	1.36E-19
201178_at	FBXO7	0	1.09	4.84E-25
201180_s_at	GNAI3	0		N.S.
201182_s_at	CHD4	0	-1.32	1.59E-21
201186_at	LRPAP1	0	-1.06	2.30E-02
201189_s_at	ITPR3	0	-1.21	4.71E-18
201191_at	PITPNA	0		N.S.
201193_at	IDH1	0		N.S.
201194_at	SEPW1	0	-1.42	3.80E-50
201195_s_at	SLC7A5	0	3.80	5.51E-97
201197_at	AMD1	0	-1.72	9.03E-58
201198_s_at	PSMD1	0	-1.64	7.10E-79
201200_at	CREG1	0	1.23	2.22E-23
201201_at	CSTB	0		N.S.
201204_s_at	RRBP1	0	1.40	1.39E-30
201209_at	HDAC1	1	-1.23	5.69E-56
201212_at	LGMN	0		N.S.
201214_s_at	PPP1R7	0		N.S.
201215_at	PLS3	0		N.S.
201216_at	ERP29	7	1.37	1.18E-60
201221_s_at	SNRNP70	0	-1.84	1.18E-68
201223_s_at	RAD23B	0		N.S.
201224_s_at	SRRM1	0	-1.72	1.53E-63
201226_at	NDUFB8	0		N.S.
201228_s_at	ARIH2	0		N.S.
201231_s_at	ENO1	0	-1.32	1.67E-53
201232_s_at	PSMD13	0	-1.14	7.78E-27
201234_at	ILK	1	-1.10	8.63E-10
201235_s_at	BTG2	0	-1.36	1.30E-21
201238_s_at	CAPZA2	0	-1.08	1.08E-04
201240_s_at	LOC653566	0	1.34	5.93E-57
201241_at	DDX1	0	-1.30	2.55E-61

Supplemental Table 1

201243_s_at	ATP1B1	0	-1.33	6.51E-47
201244_s_at	RAF1	0		N.S.
201246_s_at	OTUB1	0	-1.12	7.86E-08
201248_s_at	SREBF2	4	-1.16	3.44E-09
201250_s_at	SLC2A1	1		N.S.
201252_at	PSMC4	0		N.S.
201253_s_at	CDIPT	0	1.14	4.14E-06
201256_at	COX7A2L	0	1.30	8.20E-73
201258_at	RPS16	0		N.S.
201260_s_at	SYPL1	0	1.15	4.03E-08
201263_at	TARS	0	1.34	2.25E-49
201266_at	TXNRD1	0	-1.12	7.52E-13
201267_s_at	PSMC3	0	-1.18	6.20E-25
201268_at	NME1	0		N.S.
201271_s_at	RALY	0	-1.10	5.27E-12
201272_at	AKR1B1	1		N.S.
201273_s_at	SRP9	0	-1.08	7.52E-13
201274_at	PSMA5	0		N.S.
201275_at	FDPS	0	-1.55	1.95E-71
201276_at	RAB5B	0	1.63	3.41E-65
201277_s_at	HNRNPAB	0	-1.58	2.86E-77
201281_at	ADRM1	0	-1.17	8.03E-30
201282_at	OGDH	0	-1.60	2.86E-60
201284_s_at	APEH	0	-1.22	2.97E-26
201288_at	ARHGDIB	0	-1.09	1.10E-10
201290_at	SEC11A	0	1.18	1.43E-41
201291_s_at	TOP2A	0		N.S.
201299_s_at	MOBKL1B	0	-1.35	5.86E-23
201302_at	ANXA4	0		N.S.
201303_at	EIF4A3	0	-1.51	2.33E-79
201304_at	NDUFA5	0		N.S.
201306_s_at	ANP32B	0	1.04	1.88E-02
201312_s_at	SH3BGRL	0	1.06	3.93E-06
201313_at	ENO2	0	-1.99	4.03E-68
201314_at	STK25	0	-1.09	9.16E-06
201316_at	PSMA2	0	-1.31	5.65E-53
201319_at	MYL12A	0		N.S.
201321_s_at	SMARCC2	0	-1.22	1.58E-26
201322_at	ATP5B	0	-1.04	7.75E-03
201323_at	EBNA1BP2	0	-1.25	1.14E-44
201327_s_at	CCT6A	0	-1.70	1.02E-83
201330_at	RARS	1	-1.99	2.20E-60
201332_s_at	STAT6	1	-1.25	<1.00E-120
201339_s_at	SCP2	0	-1.06	1.49E-10
201341_at	ENC1	0		N.S.
201342_at	SNRPC	0	-1.12	<1.00E-120
201346_at	ADIPOR2	0	-1.28	7.16E-31
201349_at	SLC9A3R1	0	-1.25	9.19E-28
201350_at	FLOT2	0		N.S.
201351_s_at	YME1L1	1	1.17	3.00E-42
201354_s_at	BAZ2A	0		N.S.
201356_at	SF3A1	0	-1.33	2.53E-48
201358_s_at	COPB1	0	1.23	4.11E-57
201360_at	CST3	0		N.S.

Supplemental Table 1

201361_at	TMEM109	0	1.20	1.23E-28
201363_s_at	IVNS1ABP	0	-1.40	2.17E-39
201364_s_at	OAZ2	0	-1.39	9.00E-40
201366_at	ANXA7	0	1.10	2.20E-02
201368_at	ZFP36L2	0	1.10	4.40E-09
201371_s_at	CUL3	0		N.S.
201375_s_at	PPP2CB	4	-1.08	2.25E-10
201376_s_at	HNRNPF	0	-1.60	6.99E-68
201379_s_at	TPD52L2	0	1.17	<1.00E-120
201380_at	CRTAP	0	1.15	1.42E-06
201382_at	CACYBP	0		N.S.
201383_s_at	LOC100133166	0	1.28	3.41E-32
201385_at	DHX15	0	-1.48	3.15E-64
201387_s_at	UCHL1	1	1.06	2.39E-04
201388_at	PSMD3	0	-1.49	2.44E-65
201390_s_at	CSNK2B	0		N.S.
201391_at	TRAP1	0	-1.14	<1.00E-120
201392_s_at	IGF2R	0	1.13	1.94E-06
201395_at	RBM5	0	1.13	<1.00E-120
201396_s_at	SGTA	0	-1.17	2.21E-09
201397_at	PHGDH	0	4.38	2.82E-90
201400_at	PSMB3	0	-1.10	<1.00E-120
201403_s_at	MGST3	0		N.S.
201405_s_at	COPS6	0	-1.11	1.66E-19
201407_s_at	PPP1CB	0		N.S.
201411_s_at	PLEKHB2	0		N.S.
201412_at	LRP10	0	1.10	8.09E-10
201413_at	HSD17B4	0	1.22	1.62E-35
201415_at	GSS	0		N.S.
201417_at	SOX4	0	-1.58	2.19E-50
201419_at	BAP1	0		N.S.
201420_s_at	WDR77	0	-1.48	1.21E-64
201422_at	IFI30	0	-1.28	1.43E-24
201423_s_at	CUL4A	0	-1.17	1.85E-24
201425_at	ALDH2	1	1.67	6.05E-56
201426_s_at	VIM	0	1.16	1.29E-21
201427_s_at	SEPP1	0	1.31	5.79E-06
201429_s_at	RPL37A	0		N.S.
201433_s_at	PTDSS1	0	1.14	5.17E-28
201434_at	TTC1	0	-1.12	1.50E-12
201437_s_at	EIF4E	4	-1.35	3.63E-31
201439_at	GBF1	1	1.32	9.41E-44
201441_at	COX6B1	0	-1.17	5.91E-27
201444_s_at	ATP6AP2	0		N.S.
201445_at	CNN3	0	-1.47	3.27E-35
201447_at	TIA1	1		N.S.
201459_at	RUVBL2	0	-1.32	1.48E-41
201460_at	MAPKAPK2	2		N.S.
201462_at	SCRN1	0		N.S.
201463_s_at	LOC100133665	0	-1.07	3.01E-12
201466_s_at	JUN	137	-1.38	6.42E-18
201468_s_at	NQO1	2		N.S.
201469_s_at	SHC1	0	1.73	5.33E-22
201470_at	GSTO1	0	-1.27	2.53E-45

Supplemental Table 1

201472_at	VBP1	0	-1.05	<1.00E-120
201473_at	JUNB	1	-1.40	6.20E-33
201477_s_at	RRM1	0	-1.38	2.64E-65
201478_s_at	DKC1	0	-1.48	1.29E-46
201480_s_at	SUPT5H	0	-1.10	4.77E-09
201484_at	SUPT4H1	0		N.S.
201486_at	RCN2	0		N.S.
201487_at	CTSC	1	-1.18	1.28E-31
201489_at	PPIF	1	-1.74	1.91E-66
201491_at	AHSA1	0	-2.02	9.52E-103
201492_s_at	RPL41	0		N.S.
201493_s_at	PUM2	0	-1.10	<1.00E-120
201494_at	PRCP	0		N.S.
201499_s_at	USP7	0		N.S.
201500_s_at	PPP1R11	0		N.S.
201502_s_at	NFKBIA	0	-1.28	2.81E-33
201507_at	PFDN1	0	-1.32	3.52E-38
201511_at	AAMP	0	-1.17	9.03E-12
201512_s_at	TOMM70A	0	-1.08	4.51E-12
201513_at	TSN	0		N.S.
201514_s_at	G3BP1	0	-1.95	5.35E-51
201516_at	SRM	0	-1.14	3.03E-26
201521_s_at	NCBP2	0	-1.61	2.72E-67
201526_at	ARF5	0		N.S.
201527_at	ATP6V1F	0	-1.14	2.84E-27
201528_at	RPA1	0	-1.11	<1.00E-120
201531_at	ZFP36	0	-1.27	2.27E-20
201532_at	PSMA3	0	-1.09	<1.00E-120
201533_at	CTNNB1	6	-1.08	2.00E-02
201534_s_at	UBL3	0	1.40	1.48E-20
201536_at	DUSP3	0	-1.45	2.26E-12
201540_at	FHL1	0	-1.38	5.75E-07
201541_s_at	ZNHIT1	0		N.S.
201543_s_at	SAR1A	1	-1.46	1.45E-61
201546_at	TRIP12	0	-1.09	1.29E-19
201553_s_at	LAMP1	2	1.17	3.33E-32
201555_at	MCM3	0	-1.42	7.24E-61
201557_at	VAMP2	1	1.38	1.30E-28
201561_s_at	CLSTN1	0		N.S.
201563_at	SORD	0	-1.44	3.40E-53
201564_s_at	FSCN1	0	-2.14	3.93E-87
201565_s_at	ID2	0	-1.10	3.81E-06
201567_s_at	GOLGA4	0	1.27	3.42E-31
201568_at	UQCRQ	0	-1.05	1.59E-06
201570_at	SAMM50	0	-1.16	6.87E-29
201574_at	ETF1	0	-1.11	8.67E-22
201576_s_at	GLB1	0	1.32	1.42E-40
201580_s_at	TMX4	0	1.40	3.99E-42
201582_at	SEC23B	0	1.84	2.55E-64
201584_s_at	DDX39	0	-1.23	1.19E-46
201585_s_at	SFPQ	0	-1.47	4.90E-46
201587_s_at	IRAK1	0	-1.13	<1.00E-120
201588_at	TXNL1	0	1.14	1.58E-43
201591_s_at	NISCH	0	-1.07	1.12E-03

Supplemental Table 1

201592_at	EIF3H	0	1.07	7.52E-13
201594_s_at	PPP4R1	0	-1.16	6.38E-27
201597_at	COX7A2	0		N.S.
201598_s_at	INPPL1	0		N.S.
201599_at	OAT	0	-1.23	3.31E-11
201600_at	PHB2	0	-1.06	4.61E-07
201604_s_at	PPP1R12A	0		N.S.
201608_s_at	PWP1	0	-1.20	2.96E-39
201612_at	ALDH9A1	0	1.10	<1.00E-120
201613_s_at	AP1G2	0		N.S.
201614_s_at	RUVBL1	0	-1.44	2.30E-54
201619_at	PRDX3	0	-1.13	4.44E-27
201620_at	MBTPS1	1		N.S.
201622_at	SND1	0	1.27	1.11E-42
201624_at	DARS	0		N.S.
201626_at	INSIG1	1	-3.10	1.65E-63
201628_s_at	RRAGA	0	-1.13	2.10E-28
201629_s_at	ACP1	0	-1.08	1.09E-08
201631_s_at	IER3	0	-1.25	4.53E-21
201632_at	EIF2B1	9	-1.20	3.34E-35
201633_s_at	CYB5B	0	-1.29	1.27E-24
201637_s_at	FXR1	0		N.S.
201639_s_at	CPSF1	0		N.S.
201641_at	BST2	0	-1.26	2.82E-36
201642_at	IFNGR2	0	-1.17	9.71E-23
201644_at	TSTA3	0	-1.06	1.10E-04
201645_at	TNC	0	4.57	8.93E-32
201647_s_at	SCARB2	0		N.S.
201648_at	JAK1	0	1.17	3.85E-25
201649_at	UBE2L6	0	1.05	3.02E-02
201651_s_at	PACSIN2	0	1.14	<1.00E-120
201652_at	COPS5	2		N.S.
201653_at	CNIH	0		N.S.
201657_at	ARL1	0	1.42	2.42E-38
201662_s_at	ACSL3	0	-1.11	3.23E-11
201663_s_at	SMC4	0	-1.25	5.20E-47
201666_at	TIMP1	0	-1.09	3.59E-10
201672_s_at	USP14	1		N.S.
201673_s_at	GYS1	0	-1.33	5.36E-45
201677_at	C3orf37	0	1.13	3.60E-02
201682_at	PMPCB	0	1.04	3.10E-08
201684_s_at	TOX4	0	1.13	5.38E-24
201687_s_at	API5	0	-1.17	2.12E-22
201689_s_at	TPD52	0	1.23	<1.00E-120
201694_s_at	EGR1	1	1.17	3.74E-02
201695_s_at	NP	6	-1.49	1.05E-49
201696_at	SFRS4	0	-1.14	5.81E-25
201697_s_at	DNMT1	1	-1.28	9.83E-46
201699_at	PSMC6	0	-1.18	8.38E-30
201700_at	CCND3	2	-1.43	8.72E-66
201702_s_at	PPP1R10	0		N.S.
201704_at	ENTPD6	0	-1.11	7.45E-07
201705_at	PSMD7	0		N.S.
201707_at	PEX19	0		N.S.

Supplemental Table 1

201709_s_at	NIPSNAP1	1	-1.07	1.82E-04
201710_at	MYBL2	0	-1.31	1.77E-34
201713_s_at	RANBP2	0	-1.12	<1.00E-120
201714_at	TUBG1	0	-1.58	1.01E-81
201715_s_at	ACIN1	0	-1.19	1.71E-30
201716_at	SNX1	0	1.30	1.35E-45
201717_at	MRPL49	1	-1.17	1.49E-19
201719_s_at	EPB41L2	0	-1.22	2.64E-24
201721_s_at	LAPTM5	0	1.14	9.12E-23
201724_s_at	GALNT1	0	1.15	1.96E-25
201725_at	CDC123	0	-1.24	1.22E-62
201726_at	ELAVL1	0	-1.22	3.19E-59
201728_s_at	KIAA0100	0	-1.34	7.28E-20
201731_s_at	TPR	2		N.S.
201732_s_at	CLCN3	0	1.36	1.50E-33
201738_at	EIF1B	0	1.18	1.07E-25
201739_at	SGK1	0		N.S.
201740_at	NDUFS3	0	-1.13	9.10E-27
201746_at	TP53	5	1.07	5.95E-07
201747_s_at	SAFB	0	-1.21	<1.00E-120
201751_at	JOSD1	0		N.S.
201752_s_at	ADD3	0	1.18	2.19E-23
201754_at	COX6C	0		N.S.
201755_at	MCM5	0	-1.41	9.42E-49
201756_at	RPA2	0	-1.32	1.61E-57
201757_at	NDUFS5	0	-1.16	3.24E-27
201758_at	TSG101	0	1.12	8.15E-36
201760_s_at	WSB2	0	-1.36	3.25E-45
201761_at	MTHFD2	0	2.01	6.41E-100
201762_s_at	PSME2	1		N.S.
201763_s_at	DAXX	0	-1.20	1.20E-11
201764_at	TMEM106C	0	-1.58	1.89E-69
201768_s_at	CLINT1	0		N.S.
201770_at	SNRPA	0	1.10	<1.00E-120
201771_at	SCAMP3	0	1.13	6.19E-19
201772_at	AZIN1	0	-1.14	1.13E-25
201774_s_at	NCAPD2	0	1.06	1.04E-02
201777_s_at	KIAA0494	0	-1.06	1.08E-02
201780_s_at	RNF13	0	1.80	6.87E-73
201781_s_at	AIP	1	-1.08	2.98E-07
201783_s_at	RELA	7		N.S.
201786_s_at	ADAR	0		N.S.
201788_at	DDX42	0	-1.24	7.95E-30
201791_s_at	DHCR7	0	-2.77	5.32E-76
201795_at	LBR	0	-1.06	7.81E-04
201797_s_at	VARS	0	-1.52	1.17E-53
201800_s_at	OSBP	0	1.78	3.09E-68
201802_at	SLC29A1	0	-1.62	2.74E-51
201803_at	POLR2B	0	-1.20	1.51E-52
201805_at	PRKAG1	0	1.36	1.38E-59
201806_s_at	ATXN2L	0	1.46	1.86E-35
201807_at	VPS26A	0		N.S.
201810_s_at	SH3BP5	0	-1.35	2.95E-28
201812_s_at	C4orf46	0		N.S.

Supplemental Table 1

201815_s_at	TBC1D5	0		N.S.
201816_s_at	GBAS	0	-1.17	9.85E-26
201817_at	UBE3C	0	-1.18	9.24E-27
201818_at	LPCAT1	0	-1.37	9.84E-43
201819_at	SCARB1	0		N.S.
201821_s_at	TIMM17A	0		N.S.
201823_s_at	RNF14	0	1.08	1.07E-07
201826_s_at	SCCPDH	0		N.S.
201827_at	SMARCD2	0		N.S.
201830_s_at	NET1	0	-1.20	1.18E-26
201833_at	HDAC2	0	-1.29	1.94E-52
201834_at	PRKAB1	0	1.19	1.79E-18
201837_s_at	SUPT7L	0	1.38	2.15E-58
201840_at	NEDD8	0	-1.26	3.19E-43
201841_s_at	HSPB1	4	-1.55	4.33E-60
201845_s_at	RYBP	0	1.23	3.09E-43
201847_at	LIPA	0	1.36	2.47E-49
201849_at	BNIP3	3	-1.37	1.05E-22
201850_at	CAPG	0		N.S.
201851_at	SH3GL1	0		N.S.
201853_s_at	CDC25B	0		N.S.
201854_s_at	ATMIN	0	-1.06	1.40E-02
201856_s_at	ZFR	0		N.S.
201859_at	SRGN	0		N.S.
201861_s_at	LRRFIP1	0	1.09	4.47E-04
201863_at	FAM32A	0	-1.16	8.28E-24
201864_at	GDI1	0	-1.21	2.11E-11
201868_s_at	TBL1X	0	1.48	3.01E-12
201870_at	TOMM34	0	-1.22	1.02E-18
201872_s_at	ABCE1	0	-1.53	2.34E-84
201874_at	MPZL1	0	-1.19	7.32E-22
201881_s_at	ARIH1	0		N.S.
201885_s_at	CYB5R3	0	-1.08	2.27E-05
201889_at	FAM3C	0	1.34	2.23E-38
201890_at	RRM2	0	-1.62	6.24E-73
201892_s_at	IMPDH2	0	-1.08	2.36E-10
201895_at	ARAF	0	1.17	3.00E-25
201896_s_at	PSRC1	0	1.22	5.95E-22
201897_s_at	CKS1B	0	1.37	3.36E-63
201899_s_at	UBE2A	0		N.S.
201900_s_at	AKR1A1	0	1.12	8.18E-21
201903_at	UQCRC1	0	-1.05	3.50E-05
201908_at	DVL3	0		N.S.
201912_s_at	GSPT1	0	-1.30	9.57E-64
201913_s_at	COASY	0	-1.12	4.06E-10
201914_s_at	SEC63	1	2.08	2.87E-58
201917_s_at	SLC25A36	0		N.S.
201920_at	SLC20A1	0	-1.67	8.29E-72
201921_at	GNG10	0	-1.24	3.71E-23
201922_at	TINP1	0		N.S.
201923_at	PRDX4	0	1.10	6.48E-07
201924_at	AFF1	0	1.12	<1.00E-120
201925_s_at	CD55	0	1.50	7.13E-45
201928_at	PKP4	0		N.S.

Supplemental Table 1

201930_at	MCM6	0	-1.48	5.75E-65
201931_at	ETFA	1	1.04	5.08E-06
201932_at	LRRC41	0	-1.27	9.18E-40
201933_at	CHMP1A	0	-1.26	7.23E-20
201934_at	WDR82	0	-1.16	2.71E-11
201936_s_at	EIF4G3	0		N.S.
201937_s_at	DNPEP	0	-1.30	2.49E-29
201938_at	CDK2AP1	0	-1.23	1.92E-44
201944_at	HEXB	0		N.S.
201945_at	FURIN	1		N.S.
201947_s_at	CCT2	0	-1.59	3.35E-81
201948_at	GNL2	0	-1.48	6.51E-58
201952_at	ALCAM	0	1.16	9.61E-20
201953_at	CIB1	0	-1.15	5.89E-25
201954_at	ARPC1B	0		N.S.
201955_at	CCNC	0	1.12	2.05E-24
201956_s_at	GNPAT	0		N.S.
201957_at	PPP1R12B	0	1.14	1.54E-04
201959_s_at	MYCBP2	0		N.S.
201961_s_at	RNF41	0	1.81	2.31E-66
201963_at	ACSL1	0		N.S.
201964_at	SETX	0	1.91	2.04E-94
201966_at	NDUFS2	0		N.S.
201967_at	RBM6	0		N.S.
201968_s_at	PGM1	0	-1.12	<1.00E-120
201970_s_at	NASP	0	-1.55	2.55E-80
201971_s_at	ATP6V1A	0	-1.32	7.82E-19
201973_s_at	C7orf28A	0	1.22	8.35E-55
201975_at	CLIP1	0	1.22	<1.00E-120
201977_s_at	KIAA0141	0		N.S.
201985_at	KIAA0196	0	-1.28	1.64E-20
201986_at	MED13	0	1.16	<1.00E-120
201990_s_at	CREBL2	0	1.28	3.73E-36
201991_s_at	KIF5B	0	1.07	3.12E-02
201994_at	MORF4L2	0		N.S.
201997_s_at	SPEN	0	-1.04	2.09E-02
201999_s_at	DYNLT1	0	-1.08	4.58E-05
202001_s_at	NDUFA6	0	-1.10	<1.00E-120
202007_at	NID1	0	-1.45	<1.00E-120
202009_at	TWF2	0	1.21	8.05E-25
202010_s_at	ZNF410	0	1.19	2.09E-38
202012_s_at	EXT2	0		N.S.
202016_at	MEST	0	-1.57	3.64E-20
202019_s_at	LANCL1	0		N.S.
202022_at	ALDOC	0	-1.68	1.88E-47
202024_at	ASNA1	0		N.S.
202026_at	SDHD	0	-1.16	1.08E-23
202027_at	TMEM184B	0		N.S.
202028_s_at	RPL38	0		N.S.
202030_at	BCKDK	0	-1.27	2.77E-28
202033_s_at	RB1CC1	0	1.26	4.92E-46
202038_at	UBE4A	0	1.06	4.43E-05
202039_at	MYO18A	0		N.S.
202040_s_at	KDM5A	0	1.16	4.19E-27

Supplemental Table 1

202041_s_at	FIBP	0		N.S.
202042_at	HARS	0		N.S.
202043_s_at	SMS	0	-1.13	3.60E-29
202045_s_at	GRLF1	0		N.S.
202048_s_at	CBX6	0	-1.42	3.88E-44
202050_s_at	ZMYM4	0		N.S.
202053_s_at	ALDH3A2	1	-1.27	1.18E-42
202055_at	KPNA1	0	-1.09	2.64E-03
202060_at	CTR9	0		N.S.
202064_s_at	SEL1L	4	3.22	1.28E-82
202069_s_at	IDH3A	0	-1.63	2.18E-80
202071_at	SDC4	0	-1.11	2.19E-03
202072_at	HNRNPL	0		N.S.
202074_s_at	OPTN	0	1.31	6.20E-43
202075_s_at	PLTP	0	1.19	2.13E-09
202076_at	BIRC2	1	1.32	1.69E-58
202077_at	NDUFAB1	0	-1.07	8.27E-11
202078_at	COPS3	0	-1.07	2.44E-08
202080_s_at	TRAK1	0	1.10	1.04E-04
202081_at	IER2	0		N.S.
202083_s_at	SEC14L1	0		N.S.
202085_at	TJP2	0	1.91	1.30E-77
202086_at	MX1	0		N.S.
202089_s_at	SLC39A6	0		N.S.
202090_s_at	UQCR	0	-1.08	2.57E-06
202092_s_at	ARL2BP	0		N.S.
202093_s_at	PAF1	0	-1.10	3.26E-07
202095_s_at	BIRC5	0	-1.20	1.21E-30
202096_s_at	TSPO	0		N.S.
202097_at	NUP153	0	-1.53	2.67E-60
202100_at	RALB	0		N.S.
202103_at	BRD4	0	1.14	3.00E-08
202104_s_at	SPG7	0	-1.27	4.13E-24
202105_at	IGBP1	0	1.17	5.06E-28
202106_at	GOLGA3	0	1.32	2.24E-31
202107_s_at	MCM2	0	-1.65	1.36E-73
202108_at	PEPD	0		N.S.
202109_at	ARFIP2	0		N.S.
202110_at	COX7B	0	-1.12	1.85E-21
202111_at	SLC4A2	0		N.S.
202113_s_at	SNX2	0	1.06	2.81E-02
202115_s_at	NOC2L	0	-1.53	5.78E-38
202116_at	DPF2	0	1.17	5.87E-11
202117_at	ARHGAP1	0		N.S.
202119_s_at	CPNE3	0	-1.39	3.30E-57
202121_s_at	CHMP2A	0		N.S.
202122_s_at	M6PRBP1	0		N.S.
202123_s_at	ABL1	0		N.S.
202124_s_at	TRAK2	0	-1.11	1.13E-03
202126_at	PRPF4B	0	-1.42	1.87E-61
202128_at	KIAA0317	0		N.S.
202129_s_at	RIOK3	0		N.S.
202135_s_at	ACTR1B	0		N.S.
202136_at	ZMYND11	0		N.S.

Supplemental Table 1

202139_at	AKR7A2	0		N.S.
202140_s_at	CLK3	0	-1.33	5.10E-37
202143_s_at	COPS8	0	-1.15	6.02E-12
202144_s_at	ADSL	0	-1.08	2.26E-12
202145_at	LY6E	0		N.S.
202146_at	IFRD1	0	2.94	2.15E-88
202148_s_at	PYCR1	0	2.34	5.23E-86
202149_at	NEDD9	0		N.S.
202151_s_at	UBAC1	0	-1.32	4.56E-41
202161_at	PKN1	0		N.S.
202162_s_at	CNOT8	0	-1.16	3.70E-10
202166_s_at	PPP1R2	0		N.S.
202167_s_at	MMS19	0		N.S.
202168_at	TAF9	0	1.04	1.12E-02
202170_s_at	AASDHPPT	0	-1.25	2.52E-38
202173_s_at	VEZF1	0	1.11	4.00E-03
202174_s_at	PCM1	0	1.19	1.77E-30
202175_at	CHPF	0	1.70	5.31E-36
202179_at	BLMH	0	-1.30	1.84E-41
202180_s_at	MVP	0	-1.43	1.29E-40
202181_at	KIAA0247	0	1.22	1.47E-37
202182_at	KAT2A	0	-1.46	1.01E-31
202184_s_at	NUP133	0	-1.16	1.39E-31
202185_at	PLOD3	0		N.S.
202187_s_at	PPP2R5A	0	1.10	2.76E-05
202188_at	NUP93	0	-1.30	7.30E-40
202190_at	CSTF1	0	-1.47	4.11E-54
202191_s_at	GAS7	0	1.56	3.18E-36
202193_at	LIMK2	1	-1.21	1.75E-20
202195_s_at	TMED5	0	1.84	1.45E-66
202200_s_at	SRPK1	0	-1.26	7.32E-57
202201_at	BLVRB	0	1.14	1.02E-08
202204_s_at	AMFR	0	1.09	2.88E-04
202205_at	VASP	0	-1.13	9.01E-06
202209_at	LSM3	0	-1.25	1.19E-40
202211_at	ARFGAP3	0	1.78	1.53E-58
202212_at	PES1	0	-1.57	5.06E-56
202213_s_at	CUL4B	0		N.S.
202215_s_at	NFYC	0	1.24	6.79E-31
202217_at	C21orf33	0	-1.16	2.36E-18
202218_s_at	FADS2	0	-1.89	6.93E-49
202220_at	KIAA0907	0		N.S.
202221_s_at	EP300	0		N.S.
202223_at	STT3A	2	1.90	1.47E-77
202225_at	CRK	0		N.S.
202227_s_at	BRD8	0	1.16	8.11E-32
202228_s_at	NPTN	0		N.S.
202230_s_at	CHERP	0	-1.44	3.79E-71
202233_s_at	UQCRH	1	-1.05	7.23E-04
202234_s_at	SLC16A1	0	1.18	<1.00E-120
202239_at	PARP4	0	1.18	<1.00E-120
202240_at	PLK1	0		N.S.
202241_at	TRIB1	0		N.S.
202243_s_at	PSMB4	0	-1.09	9.66E-19

Supplemental Table 1

202246_s_at	CDK4	3	-1.24	7.42E-46
202249_s_at	WDR42A	0		N.S.
202251_at	PRPF3	0	-1.10	1.47E-08
202252_at	RAB13	0		N.S.
202253_s_at	DNM2	0	1.16	8.58E-11
202254_at	SIPA1L1	0	1.11	3.91E-05
202257_s_at	CD2BP2	0		N.S.
202261_at	VPS72	0	-1.12	<1.00E-120
202263_at	CYB5R1	0		N.S.
202264_s_at	TOMM40	0	-1.66	2.71E-76
202265_at	BMI1	0	1.11	2.09E-09
202266_at	TTRAP	0	-1.10	1.52E-24
202268_s_at	NAE1	0	-1.35	4.29E-58
202270_at	GBP1	0	-1.38	1.89E-19
202272_s_at	FBXO28	0	-1.06	7.02E-06
202275_at	G6PD	1	-1.06	1.27E-03
202276_at	SHFM1	0	-1.10	2.79E-08
202277_at	SPTLC1	0		N.S.
202279_at	C14orf2	0	-1.21	3.50E-45
202282_at	HSD17B10	0		N.S.
202284_s_at	CDKN1A	9	1.35	4.57E-56
202288_at	FRAP1	0		N.S.
202294_at	STAG1	0	1.18	7.58E-09
202295_s_at	CTSH	0	-1.21	6.21E-36
202297_s_at	RER1	0	1.10	9.83E-18
202298_at	NDUFA1	0		N.S.
202300_at	HBXIP	0	-1.28	1.27E-33
202301_s_at	RSRC2	0	-1.23	2.03E-30
202306_at	POLR2G	0		N.S.
202307_s_at	TAP1	1	1.30	2.39E-37
202308_at	SREBF1	9	-1.40	9.22E-20
202309_at	MTHFD1	0	-1.29	3.27E-20
202313_at	PPP2R2A	0	-1.06	5.80E-07
202321_at	GGPS1	0	1.23	6.50E-19
202323_s_at	ACBD3	0		N.S.
202325_s_at	ATP5J	0	-1.16	8.95E-32
202328_s_at	PKD1	0		N.S.
202329_at	CSK	0	-1.31	1.02E-38
202330_s_at	UNG	0	-2.13	6.14E-73
202331_at	BCKDHA	0	1.42	1.85E-56
202336_s_at	PAM	0	1.18	1.98E-27
202337_at	PMF1	0		N.S.
202338_at	TK1	0	-1.17	1.52E-17
202339_at	SYMPK	0		N.S.
202344_at	HSF1	7		N.S.
202345_s_at	FABP5	0	-1.57	3.02E-67
202347_s_at	UBE2K	0		N.S.
202349_at	TOR1A	0		N.S.
202351_at	ITGAV	0	1.08	3.07E-03
202352_s_at	PSMD12	0	-1.39	1.66E-71
202355_s_at	GTF2F1	0		N.S.
202359_s_at	SNX19	0	1.16	3.17E-10
202360_at	MAML1	0	-1.15	1.47E-24
202361_at	SEC24C	0	1.22	5.05E-36

Supplemental Table 1

202362_at	RAP1A	0		N.S.
202364_at	MXI1	0	1.21	3.95E-07
202365_at	UNC119B	0		N.S.
202366_at	ACADS	0		N.S.
202367_at	CUX1	0	1.12	1.03E-02
202369_s_at	TRAM2	0	1.14	<1.00E-120
202371_at	TCEAL4	0	-1.10	2.93E-11
202378_s_at	LEPROT	0	1.11	1.16E-07
202381_at	ADAM9	0	1.54	1.09E-60
202382_s_at	GNPDA1	0	-1.32	6.04E-32
202383_at	KDM5C	0		N.S.
202384_s_at	TCOF1	0	-1.67	3.59E-63
202386_s_at	KIAA0430	0	1.32	7.50E-46
202388_at	RGS2	0	-1.75	4.87E-36
202390_s_at	HTT	2	-1.13	5.56E-04
202391_at	BASP1	1		N.S.
202392_s_at	PISD	0	1.47	2.91E-42
202393_s_at	KLF10	0		N.S.
202394_s_at	ABCF3	0		N.S.
202395_at	NSF	1	1.06	1.18E-02
202396_at	TCERG1	0	-1.51	1.94E-74
202397_at	NUTF2	0	-1.16	5.93E-37
202399_s_at	AP3S2	0	1.23	1.81E-44
202402_s_at	CARS	0	2.58	6.43E-91
202406_s_at	TIAL1	0	1.06	2.71E-06
202413_s_at	USP1	0		N.S.
202414_at	ERCC5	0	1.28	2.35E-40
202415_s_at	HSPBP1	0	-1.10	1.86E-03
202417_at	KEAP1	4	-1.21	1.25E-32
202418_at	YIF1A	0	1.08	1.92E-04
202419_at	KDSR	0		N.S.
202420_s_at	DHX9	0	-1.86	7.53E-82
202421_at	IGSF3	0	-1.31	<1.00E-120
202422_s_at	ACSL4	0	-1.38	<1.00E-120
202424_at	MAP2K2	0	1.21	2.82E-38
202427_s_at	BRP44	0	-1.10	1.57E-17
202431_s_at	MYC	14	-1.26	1.29E-21
202432_at	PPP3CB	0	1.18	1.19E-36
202433_at	SLC35B1	1	2.65	7.62E-88
202435_s_at	CYP1B1	1	1.72	4.76E-38
202439_s_at	IDS	0	-1.07	4.16E-04
202444_s_at	ERLIN1	0	-1.28	<1.00E-120
202446_s_at	PLSCR1	0	-1.12	3.69E-11
202447_at	DECR1	0	1.06	5.03E-07
202449_s_at	RXRA	0	1.13	8.69E-05
202450_s_at	CTSK	0	1.22	7.07E-11
202451_at	GTF2H1	0		N.S.
202457_s_at	PPP3CA	0	1.31	6.67E-31
202459_s_at	LPIN2	0	-1.18	<1.00E-120
202461_at	EIF2B2	0	-1.16	2.52E-24
202462_s_at	DDX46	0	-1.19	<1.00E-120
202466_at	POLS	0	1.33	5.98E-30
202468_s_at	CTNNAL1	0	-1.10	4.10E-07
202470_s_at	CPSF6	0	-1.19	<1.00E-120

202471_s_at	IDH3G	0		N.S.
202472_at	MPI	1	-1.27	1.47E-24
202474_s_at	HCFC1	0	-1.23	<1.00E-120
202475_at	TMEM147	0	-1.04	4.52E-02
202480_s_at	DEDD	0		N.S.
202486_at	AFG3L2	1	-1.24	3.08E-31
202487_s_at	H2AFV	0		N.S.
202488_s_at	FXYD3	0		N.S.
202490_at	IKBKAP	0		N.S.
202492_at	ATG9A	0	1.26	1.47E-22
202495_at	TBCC	0	-1.10	1.52E-05
202496_at	EDC4	0	-1.32	1.33E-27
202499_s_at	SLC2A3	0	1.33	3.98E-24
202500_at	DNAJB2	0	1.15	1.13E-07
202502_at	ACADM	0		N.S.
202503_s_at	KIAA0101	0	-1.07	1.13E-11
202505_at	SNRPB2	1	-1.15	<1.00E-120
202506_at	SSFA2	0	1.07	4.07E-04
202510_s_at	TNFAIP2	0	1.11	3.83E-04
202513_s_at	PPP2R5D	0	-1.19	6.69E-11
202514_at	DLG1	0	1.09	6.29E-04
202518_at	BCL7B	0		N.S.
202520_s_at	MLH1	0	1.08	<1.00E-120
202521_at	CTCF	0	-1.07	3.09E-08
202522_at	PITPNB	0	-1.07	7.44E-10
202527_s_at	SMAD4	0		N.S.
202528_at	GALE	1	-1.29	3.46E-07
202529_at	PRPSAP1	0		N.S.
202530_at	MAPK14	0		N.S.
202531_at	IRF1	2	1.14	1.35E-09
202532_s_at	DHFR	0	-1.52	2.89E-56
202535_at	FADD	4	1.31	5.86E-33
202538_s_at	CHMP2B	0		N.S.
202540_s_at	HMGCR	0	-2.31	4.37E-96
202541_at	SCYE1	0	-1.13	1.12E-08
202543_s_at	GMFB	0		N.S.
202545_at	PRKCD	0	-1.23	1.14E-27
202546_at	VAMP8	0	-1.14	6.75E-22
202548_s_at	ARHGEF7	0		N.S.
202550_s_at	VAPB	6	-1.06	1.33E-05
202552_s_at	CRIM1	0		N.S.
202553_s_at	SYF2	0	1.40	5.29E-58
202556_s_at	MCRS1	0	1.09	6.89E-09
202557_at	HSPA13	0	3.01	1.84E-90
202567_at	SNRPD3	0	-1.26	6.41E-58
202569_s_at	MARK3	0		N.S.
202573_at	CSNK1G2	0	-1.19	<1.00E-120
202578_s_at	DDX19A	0	-1.69	4.43E-65
202581_at	HSPA1A	2	-2.69	1.63E-68
202583_s_at	RANBP9	0	1.25	1.41E-35
202584_at	NFX1	1		N.S.
202586_at	POLR2L	0	-1.23	1.50E-12
202587_s_at	AK1	0		N.S.
202592_at	BLOC1S1	0	-1.11	3.84E-09

Supplemental Table 1

202593_s_at	GDE1	0		N.S.
202594_at	LEPROTL1	0		N.S.
202600_s_at	NRIP1	0		N.S.
202602_s_at	HTATSF1	0	-1.15	<1.00E-120
202605_at	GUSB	0		N.S.
202609_at	EPS8	0		N.S.
202613_at	CTPS	0	-1.91	1.61E-86
202614_at	SLC30A9	0	1.07	5.55E-07
202617_s_at	MECP2	0		N.S.
202620_s_at	PLOD2	0		N.S.
202621_at	IRF3	0	-1.15	6.36E-27
202622_s_at	ATXN2	0		N.S.
202623_at	EAPP	0	1.32	4.89E-53
202624_s_at	CABIN1	0		N.S.
202625_at	LYN	0		N.S.
202631_s_at	APPBP2	0	1.21	4.84E-34
202632_at	DPH1	0		N.S.
202633_at	TOPBP1	0	-1.28	5.71E-60
202635_s_at	POLR2K	0	-1.22	4.02E-40
202636_at	RNF103	0	1.18	<1.00E-120
202640_s_at	RANBP3	0	-1.21	1.70E-21
202643_s_at	TNFAIP3	0		N.S.
202645_s_at	MEN1	0		N.S.
202647_s_at	NRAS	1	-1.07	3.42E-06
202650_s_at	KIAA0195	0	1.11	3.78E-05
202651_at	LPGAT1	0	1.17	6.71E-29
202653_s_at	MARCH7	0		N.S.
202655_at	ARMET	3	3.12	3.34E-70
202657_s_at	SERTAD2	0	1.04	2.03E-02
202658_at	PEX11B	0		N.S.
202659_at	PSMB10	0	-1.12	2.27E-23
202662_s_at	ITPR2	0	1.21	2.13E-23
202665_s_at	WIPF1	0	1.13	9.03E-12
202666_s_at	ACTL6A	0	-1.34	1.15E-19
202667_s_at	SLC39A7	0	1.55	4.07E-41
202670_at	MAP2K1	0	1.17	1.29E-40
202672_s_at	ATF3	27	2.43	2.39E-81
202673_at	DPM1	0		N.S.
202677_at	RASA1	0		N.S.
202678_at	GTF2A2	0	-1.21	5.00E-45
202680_at	GTF2E2	0	-1.07	8.60E-05
202681_at	USP4	0		N.S.
202683_s_at	RNMT	0		N.S.
202688_at	TNFSF10	18	1.14	3.59E-03
202689_at	RBM15B	0	-1.20	9.99E-18
202691_at	SNRPD1	0	-1.62	3.34E-62
202693_s_at	STK17A	0		N.S.
202696_at	OXSR1	0	-1.18	5.87E-40
202702_at	TRIM26	0	1.13	<1.00E-120
202703_at	DUSP11	0	1.15	3.52E-33
202704_at	TOB1	0		N.S.
202705_at	CCNB2	0	1.05	3.30E-02
202708_s_at	HIST2H2BE	0	1.16	2.84E-05
202709_at	FMOD	0	1.21	8.22E-03

Supplemental Table 1

202710_at	BET1	0	2.10	2.47E-78
202713_s_at	KIAA0391	0	-1.44	2.52E-58
202715_at	CAD	1	-1.34	4.62E-38
202716_at	PTPN1	2	-1.07	3.69E-02
202720_at	TES	0	2.17	2.70E-70
202721_s_at	GFPT1	0	1.67	4.47E-57
202724_s_at	FOXO1	7		N.S.
202725_at	POLR2A	0	-1.27	1.58E-18
202726_at	LIG1	0	-1.37	7.19E-42
202730_s_at	PDCD4	0	1.88	8.78E-59
202732_at	PKIG	0	1.20	3.21E-25
202733_at	P4HA2	1	-1.37	1.06E-18
202734_at	TRIP10	0	-1.19	6.35E-21
202736_s_at	LSM4	0	-1.06	6.90E-05
202738_s_at	PHKB	0	1.14	1.07E-22
202740_at	ACY1	0	1.09	1.87E-07
202742_s_at	PRKACB	0		N.S.
202743_at	PIK3R3	0	-1.35	1.64E-36
202745_at	USP8	0		N.S.
202746_at	ITM2A	0	-1.08	1.26E-02
202748_at	GBP2	0	-1.10	4.90E-02
202749_at	WRB	1		N.S.
202750_s_at	TFIP11	0		N.S.
202753_at	PSMD6	0	-1.15	4.35E-39
202754_at	R3HDM1	0	1.07	1.30E-04
202757_at	COBRA1	0	-1.09	1.60E-09
202758_s_at	RFXANK	0		N.S.
202759_s_at	AKAP2	0		N.S.
202760_s_at	PALM2-AKAP2	0	-1.06	2.65E-03
202761_s_at	SYNE2	0		N.S.
202762_at	ROCK2	0	-1.05	3.69E-02
202763_at	CASP3	223	-1.07	1.78E-02
202764_at	STIM1	1	1.26	<1.00E-120
202765_s_at	FBN1	0		N.S.
202767_at	ACP2	0		N.S.
202769_at	CCNG2	1	2.00	3.11E-43
202771_at	FAM38A	0	1.25	2.05E-18
202772_at	HMGCL	0	1.16	1.02E-17
202775_s_at	SFRS8	0	-1.08	7.58E-07
202776_at	DNTTIP2	0	-1.56	7.64E-82
202777_at	SHOC2	0	1.36	2.12E-49
202779_s_at	LOC731049	0	-1.11	<1.00E-120
202780_at	OXCT1	0	-1.04	1.16E-02
202781_s_at	INPP5K	0		N.S.
202783_at	NNT	1	-1.09	4.01E-07
202785_at	NDUFA7	0		N.S.
202786_at	STK39	0		N.S.
202787_s_at	MAPKAPK3	0	-1.29	1.61E-25
202789_at	PLCG1	1	-1.28	3.69E-28
202793_at	LPCAT3	1	-1.43	7.86E-26
202794_at	INPP1	0	1.19	6.36E-24
202796_at	SYNPO	0		N.S.
202797_at	SACM1L	0		N.S.
202798_at	SEC24B	0	-1.09	7.52E-13

Supplemental Table 1

202799_at	CLPP	3	-1.12	1.04E-22
202803_s_at	ITGB2	0		N.S.
202804_at	ABCC1	0		N.S.
202808_at	C10orf26	0	1.08	2.21E-06
202809_s_at	INTS3	0	1.29	3.24E-48
202810_at	DRG1	2	-1.21	3.82E-46
202811_at	STAMBP	0	1.19	7.81E-35
202812_at	GAA	0		N.S.
202813_at	TARBP1	0	-1.17	1.59E-19
202815_s_at	HEXIM1	0	-1.16	3.06E-07
202820_at	AHR	3	-1.33	4.92E-22
202824_s_at	TCEB1	0	-1.14	8.85E-35
202825_at	SLC25A4	0	-1.41	9.05E-55
202829_s_at	VAMP7	1	1.24	2.56E-69
202832_at	GCC2	0	1.33	4.59E-55
202837_at	TRAFD1	0	-1.14	8.51E-05
202838_at	FUCA1	0	1.31	9.37E-50
202839_s_at	NDUFB7	0	-1.17	<1.00E-120
202840_at	TAF15	0		N.S.
202843_at	DNAJB9	3	6.32	1.56E-80
202844_s_at	RALBP1	0		N.S.
202847_at	PCK2	0	3.47	5.88E-117
202850_at	ABCD3	0	-1.27	2.14E-43
202853_s_at	RYK	0	1.10	<1.00E-120
202854_at	HPRT1	0	-1.29	1.62E-60
202856_s_at	SLC16A3	0	-1.25	4.59E-09
202858_at	U2AF1	0	-1.13	<1.00E-120
202860_at	DENND4B	0		N.S.
202862_at	FAH	1	1.05	1.38E-02
202868_s_at	POP4	0	-1.28	3.10E-49
202870_s_at	CDC20	0		N.S.
202871_at	TRAF4	0	-1.21	<1.00E-120
202874_s_at	ATP6V1C1	0	-1.32	5.31E-47
202876_s_at	PBX2	0		N.S.
202887_s_at	DDIT4	2	1.82	5.76E-54
202891_at	NIT1	0	1.06	3.10E-05
202892_at	CDC23	0	1.04	3.35E-02
202897_at	SIRPA	0		N.S.
202899_s_at	SFRS3	0	-1.26	1.47E-46
2028_s_at	E2F1	4	-1.42	1.00E-48
202900_s_at	NUP88	0		N.S.
202902_s_at	CTSS	0	1.17	8.82E-18
202904_s_at	LSM5	0	-1.13	1.39E-09
202908_at	WFS1	11	2.74	2.46E-70
202909_at	EPM2AIP1	0	-1.16	7.52E-13
202910_s_at	CD97	0	1.39	8.31E-45
202912_at	ADM	0	-1.78	<1.00E-120
202915_s_at	FAM20B	0	-1.10	2.94E-06
202919_at	MOBKL3	0	-1.13	2.29E-04
202922_at	GCLC	1	1.12	1.20E-08
202925_s_at	PLAGL2	1	1.93	1.81E-77
202926_at	NBAS	0	1.26	<1.00E-120
202927_at	PIN1	0	-1.34	7.81E-42
202929_s_at	DDT	1	-1.13	9.16E-20

Supplemental Table 1

202930_s_at	SUCLA2	0	-1.09	7.52E-13
202935_s_at	SOX9	0	-1.33	1.37E-31
202939_at	ZMPSTE24	0	1.09	<1.00E-120
202941_at	NDUFV2	1	-1.15	1.33E-37
202942_at	ETFB	1	-1.05	2.79E-02
202943_s_at	NAGA	0		N.S.
202945_at	FPGS	0	-1.21	<1.00E-120
202946_s_at	BTBD3	0		N.S.
202947_s_at	GYPC	0	1.33	6.04E-56
202949_s_at	FHL2	0	1.28	4.51E-12
202950_at	CRYZ	0	1.56	7.01E-55
202954_at	UBE2C	0		N.S.
202957_at	HCLS1	0		N.S.
202958_at	PTPN9	0	1.12	5.79E-10
202960_s_at	MUT	2	1.09	2.24E-02
202961_s_at	ATP5J2	0	-1.19	1.58E-33
202962_at	KIF13B	0	-1.20	6.02E-12
202963_at	RFX5	0		N.S.
202967_at	GSTA4	0	1.47	1.27E-46
202968_s_at	DYRK2	0	-1.24	2.98E-33
202974_at	MPP1	0	-1.09	7.38E-05
202976_s_at	RHOBTB3	0	1.12	6.79E-03
202980_s_at	SIAH1	0	1.44	8.53E-28
202982_s_at	ACOT1	0		N.S.
202983_at	HLTF	0	1.16	1.06E-29
202984_s_at	BAG5	0	-1.22	1.17E-21
202987_at	TRAF3IP2	0		N.S.
202991_at	STARD3	0	1.12	2.29E-02
202996_at	POLD4	0		N.S.
203004_s_at	MEF2D	0		N.S.
203006_at	INPP5A	0	1.29	1.49E-29
203010_at	STAT5A	0	-1.29	6.92E-30
203011_at	IMPA1	0	-1.27	2.89E-55
203013_at	ECD	0	-1.10	1.87E-07
203017_s_at	SSX2IP	0	-1.26	7.52E-13
203022_at	RNASEH2A	0	-1.30	8.82E-49
203024_s_at	C5orf15	0		N.S.
203025_at	ARD1A	0	-1.14	2.65E-22
203026_at	ZBTB5	0	1.39	1.12E-55
203027_s_at	MVD	0	-1.47	7.52E-13
203028_s_at	CYBA	1		N.S.
203031_s_at	UROS	0	-1.10	1.15E-19
203035_s_at	PIAS3	0	1.21	1.69E-19
203038_at	PTPRK	0	1.31	1.34E-04
203039_s_at	NDUFS1	0	-1.10	1.28E-24
203040_s_at	HMBS	0		N.S.
203041_s_at	LAMP2	0	1.26	3.82E-27
203043_at	ZBED1	0	-1.34	4.12E-21
203044_at	CHSY1	0	1.11	2.56E-11
203046_s_at	TIMELESS	1	-1.32	5.30E-48
203049_s_at	TTC37	0	1.13	3.02E-03
203050_at	TP53BP1	0	1.11	4.86E-07
203051_at	BAHD1	0	-1.18	2.26E-11
203053_at	BCAS2	0	1.06	2.93E-07

Supplemental Table 1

203054_s_at	TCTA	0	1.08	1.40E-03
203055_s_at	ARHGEF1	0	-1.11	1.79E-04
203058_s_at	PAPSS2	0		N.S.
203062_s_at	MDC1	0		N.S.
203064_s_at	FOXK2	0	-1.26	4.44E-50
203065_s_at	CAV1	0		N.S.
203067_at	PDHX	0		N.S.
203068_at	KLHL21	0	-1.38	1.21E-08
203069_at	SV2A	0		N.S.
203072_at	MYO1E	0	-1.28	1.20E-33
203073_at	COG2	0	-1.22	4.26E-34
203075_at	SMAD2	0	1.16	2.36E-33
203079_s_at	CUL2	0	-1.21	5.74E-25
203080_s_at	BAZ2B	0	1.29	2.16E-25
203081_at	CTNNBIP1	0		N.S.
203082_at	BMS1	0	-1.24	3.84E-41
203085_s_at	TGFB1	3	-1.40	1.52E-59
203089_s_at	HTRA2	1	-1.06	2.15E-05
203090_at	SDF2	3		N.S.
203093_s_at	TIMM44	0	1.27	8.99E-25
203094_at	MAD2L1BP	0	-1.21	2.87E-22
203095_at	MTIF2	0	1.16	5.08E-30
203099_s_at	CDYL	0		N.S.
203102_s_at	MGAT2	0	1.71	3.00E-69
203103_s_at	PRPF19	0	1.13	1.13E-11
203105_s_at	DNM1L	0	-1.33	5.19E-57
203106_s_at	VPS41	0		N.S.
203109_at	UBE2M	0	-1.22	2.59E-31
203112_s_at	WHSC2	0	-1.22	7.71E-19
203113_s_at	EEF1D	0	1.11	3.02E-19
203114_at	SSSCA1	0	-1.38	3.91E-44
203115_at	FECH	0	1.40	6.80E-34
203117_s_at	PAN2	0	1.34	8.61E-42
203118_at	PCSK7	0	1.23	<1.00E-120
203119_at	CCDC86	0	-2.58	1.61E-81
203120_at	TP53BP2	0		N.S.
203122_at	TTC15	0		N.S.
203124_s_at	SLC11A2	0		N.S.
203126_at	IMPA2	3	-1.85	7.18E-36
203127_s_at	SPTLC2	0	1.11	<1.00E-120
203133_at	SEC61B	0	1.13	<1.00E-120
203135_at	TBP	2	-1.12	3.01E-12
203136_at	RABAC1	0	1.50	1.27E-38
203138_at	HAT1	0	-1.34	3.64E-62
203139_at	DAPK1	0	1.23	7.06E-39
203141_s_at	AP3B1	1		N.S.
203143_s_at	KIAA0040	0	1.13	2.32E-09
203145_at	SPAG5	0	-1.48	1.53E-74
203147_s_at	TRIM14	0	-1.37	8.88E-55
203150_at	RABEPK	0	-1.17	2.99E-19
203152_at	MRPL40	0		N.S.
203153_at	IFIT1	0	-1.27	2.51E-05
203155_at	SETDB1	0		N.S.
203156_at	AKAP11	0	-1.14	1.78E-29

Supplemental Table 1

203158_s_at	GLS	0	1.30	6.85E-32
203163_at	KATNB1	0		N.S.
203165_s_at	SLC33A1	0	2.68	3.51E-79
203169_at	RGP1	0	1.21	9.28E-25
203171_s_at	RRP8	0	-1.30	9.18E-33
203173_s_at	C16orf62	0	1.19	7.46E-24
203175_at	RHOG	0	-1.22	3.76E-42
203176_s_at	TFAM	0	-1.37	1.80E-39
203178_at	GATM	0	-2.25	4.69E-53
203179_at	GALT	1	-1.12	7.52E-13
203183_s_at	SMARCD1	0		N.S.
203185_at	RASSF2	0	-1.81	1.36E-76
203186_s_at	S100A4	0	-1.41	4.28E-19
203188_at	B3GNT1	0	-1.16	5.19E-11
203189_s_at	NDUFS8	0		N.S.
203192_at	ABCB6	0	1.29	2.11E-27
203196_at	ABCC4	0		N.S.
203197_s_at	C1orf123	0		N.S.
203198_at	CDK9	0	-1.20	2.57E-22
203200_s_at	MTRR	0	1.35	6.54E-43
203201_at	PMM2	0	-1.19	<1.00E-120
203202_at	KRR1	0		N.S.
203205_at	KDM4A	0	1.64	2.01E-57
203206_at	FAM53B	0		N.S.
203208_s_at	MTFR1	0	1.41	4.59E-35
203209_at	RFC5	0	-1.61	3.88E-90
203212_s_at	MTMR2	0		N.S.
203218_at	MAPK9	0		N.S.
203219_s_at	APRT	0	-1.10	<1.00E-120
203223_at	RABEP1	0	-1.18	<1.00E-120
203225_s_at	RFK	0	-1.55	1.57E-39
203226_s_at	TSPAN31	0	1.26	1.33E-27
203228_at	PAFAH1B3	0	-1.20	1.76E-22
203229_s_at	CLK2	0	-1.13	3.01E-12
203230_at	DVL1	0	-1.30	1.89E-21
203231_s_at	ATXN1	0		N.S.
203233_at	IL4R	0		N.S.
203234_at	UPP1	0	2.25	7.87E-58
203235_at	THOP1	0	-1.28	1.37E-21
203236_s_at	LGALS9	0	-1.24	1.30E-28
203241_at	UVRAG	0	-1.08	2.71E-11
203244_at	PEX5	0	-1.32	1.10E-25
203245_s_at	NCRNA00094	0		N.S.
203246_s_at	TUSC4	0	-1.07	1.33E-04
203247_s_at	ZNF24	0		N.S.
203250_at	RBM16	0	-1.07	5.85E-10
203252_at	CDK2AP2	0	2.66	1.13E-84
203253_s_at	HISPPD1	0	1.28	8.41E-24
203254_s_at	TLN1	0	1.05	3.45E-02
203258_at	DRAP1	0	-1.06	3.19E-02
203260_at	HDDC2	0		N.S.
203261_at	DCTN6	0	-1.07	2.08E-07
203262_s_at	FAM50A	0	-1.09	7.52E-13
203263_s_at	ARHGEF9	0	1.38	1.57E-50

203266_s_at	MAP2K4	0		N.S.
203268_s_at	DRG2	0		N.S.
203269_at	NSMAF	0		N.S.
203270_at	DTYMK	0	-1.22	7.00E-18
203271_s_at	UNC119	0		N.S.
203272_s_at	TUSC2	0	-1.26	2.09E-41
203274_at	F8A1	0	-1.32	5.33E-44
203275_at	IRF2	0		N.S.
203276_at	LMNB1	0	-1.24	5.27E-40
203277_at	DFFA	0	-1.49	3.74E-54
203278_s_at	PHF21A	0	1.37	5.70E-41
203279_at	EDEM1	9	2.63	3.27E-95
203280_at	SAFB2	0		N.S.
203281_s_at	UBA7	0	1.10	1.22E-03
203282_at	GBE1	0	-1.23	1.29E-37
203286_at	RNF44	0		N.S.
203288_at	KIAA0355	0	1.32	3.95E-42
203289_s_at	C16orf35	0		N.S.
203292_s_at	VPS11	0	1.16	2.26E-12
203298_s_at	JARID2	0	1.23	1.31E-30
203299_s_at	AP1S2	0	1.20	1.19E-05
203301_s_at	DMTF1	0	1.10	7.54E-06
203302_at	DCK	0	-1.33	6.46E-23
203303_at	DYNLT3	0		N.S.
203304_at	BAMBI	0	2.34	2.53E-19
203306_s_at	SLC35A1	0	1.17	8.32E-29
203307_at	GNL1	0		N.S.
203309_s_at	HPS1	1		N.S.
203310_at	STXBP3	0		N.S.
203311_s_at	ARF6	0		N.S.
203313_s_at	TGIF1	0	1.87	4.30E-29
203314_at	GTPBP6	0	-1.29	3.02E-47
203315_at	NCK2	2	1.13	1.38E-18
203316_s_at	SNRPE	0	-1.18	3.06E-30
203318_s_at	ZNF148	0		N.S.
203320_at	SH2B3	0	1.31	4.41E-46
203321_s_at	ADNP2	0	-1.08	6.31E-04
203330_s_at	STX5	0	2.32	2.80E-73
203331_s_at	INPP5D	0		N.S.
203333_at	KIFAP3	0		N.S.
203334_at	DHX8	0		N.S.
203335_at	PHYH	0		N.S.
203336_s_at	ITGB1BP1	0	-1.19	3.04E-37
203338_at	PPP2R5E	0	1.12	4.86E-21
203339_at	SLC25A12	0		N.S.
203341_at	CEBPZ	0	-1.51	7.09E-69
203342_at	TIMM17B	0	-1.08	5.42E-06
203343_at	UGDH	0	1.80	2.67E-65
203344_s_at	RBBP8	0	-1.06	2.73E-05
203350_at	AP1G1	0	1.12	4.87E-20
203351_s_at	ORC4L	0	-1.08	7.52E-13
203356_at	CAPN7	0	1.14	9.85E-11
203361_s_at	MYCBP	0	-1.11	6.28E-06
203362_s_at	MAD2L1	0	-1.39	1.81E-34

Supplemental Table 1

203363_s_at	KIAA0652	0	1.72	6.17E-68
203366_at	POLG	1	-1.28	3.58E-31
203367_at	DUSP14	0	-1.14	<1.00E-120
203368_at	CRELD1	0	3.84	1.23E-82
203371_s_at	NDUFB3	0	-1.22	2.22E-32
203373_at	SOCS2	0	-1.27	2.73E-25
203375_s_at	TPP2	0	1.18	4.65E-46
203377_s_at	CDC40	0	-1.12	7.31E-07
203378_at	PCF11	0	1.18	7.46E-24
203379_at	RPS6KA1	0		N.S.
203384_s_at	GOLGA1	0	1.32	8.68E-29
203385_at	DGKA	0	1.14	<1.00E-120
203387_s_at	TBC1D4	0		N.S.
203388_at	ARRB2	0	-1.33	4.02E-20
203391_at	FKBP2	1	2.82	2.65E-67
203396_at	PSMA4	0	1.06	4.53E-07
203397_s_at	GALNT3	0	2.38	1.65E-36
203401_at	PRPS2	0	-1.53	1.59E-64
203404_at	ARMCX2	0		N.S.
203405_at	PSMG1	0		N.S.
203406_at	MFAP1	0	-1.35	4.99E-70
203409_at	DDB2	0	1.26	1.38E-34
203410_at	AP3M2	0	1.53	1.90E-41
203411_s_at	LMNA	0	-1.11	5.16E-06
203412_at	LZTR1	0		N.S.
203414_at	MMD	0	-1.11	1.05E-11
203415_at	PDCD6	0	-1.23	8.53E-36
203416_at	CD53	0	-1.19	2.33E-30
203420_at	FAM8A1	0	1.21	1.02E-33
203422_at	POLD1	0	-1.52	1.56E-64
203427_at	ASF1A	0	1.25	2.89E-29
203429_s_at	C1orf9	0	1.74	5.53E-51
203430_at	HEBP2	0	1.08	1.85E-08
203433_at	MTHFS	0		N.S.
203436_at	RPP30	0	-1.25	3.84E-42
203437_at	TMEM11	0	-1.32	2.15E-46
203438_at	STC2	3	6.06	2.39E-77
203444_s_at	MTA2	0	-1.20	4.72E-10
203445_s_at	CTDSP2	0	1.29	2.79E-37
203449_s_at	TERF1	0		N.S.
203450_at	CBY1	0	1.34	1.67E-35
203452_at	B3GAT3	0		N.S.
203454_s_at	ATOX1	1	-1.14	<1.00E-120
203455_s_at	SAT1	0		N.S.
203456_at	PRAF2	0	1.18	3.76E-11
203457_at	STX7	0		N.S.
203458_at	SPR	0	-1.95	6.36E-59
203459_s_at	VPS16A	0		N.S.
203460_s_at	PSEN1	13	1.49	1.06E-70
203464_s_at	EPN2	0		N.S.
203465_at	MRPL19	0	-1.22	<1.00E-120
203466_at	MPV17	0	-1.13	<1.00E-120
203467_at	PMM1	0	1.66	4.31E-75
203468_at	CDK10	0		N.S.

Supplemental Table 1

203471_s_at	PLEK	0	1.14	3.26E-19
203474_at	IQGAP2	0		N.S.
203476_at	TPBG	0	1.23	4.56E-08
203478_at	NDUFC1	0	-1.13	1.37E-24
203480_s_at	OTUD4	0		N.S.
203482_at	FAM178A	0	1.18	<1.00E-120
203484_at	SEC61G	1	1.26	5.23E-44
203487_s_at	ARMC8	0		N.S.
203493_s_at	CEP57	0	1.49	1.08E-40
203495_at	LRRC14	0		N.S.
203496_s_at	MED1	1		N.S.
203500_at	GCDH	0	-1.14	1.25E-06
203502_at	BPGM	0		N.S.
203508_at	TNFRSF1B	0	-1.63	6.28E-62
203511_s_at	TRAPPC3	0	-1.16	4.38E-31
203513_at	SPG11	0	1.28	1.10E-42
203514_at	MAP3K3	0		N.S.
203515_s_at	PMVK	0		N.S.
203517_at	MTX2	0	-1.07	1.04E-08
203519_s_at	UPF2	0	-1.15	9.77E-27
203521_s_at	ZNF318	0	-1.09	2.26E-04
203522_at	CCS	2	1.17	<1.00E-120
203523_at	LSP1	0		N.S.
203524_s_at	MPST	0	-1.17	<1.00E-120
203526_s_at	APC	0	-1.10	4.10E-02
203528_at	SEMA4D	0		N.S.
203529_at	PPP6C	0		N.S.
203530_s_at	STX4	0		N.S.
203531_at	CUL5	0	1.04	4.59E-03
203534_at	LSM1	0	-1.12	<1.00E-120
203537_at	PRPSAP2	0	1.13	2.70E-26
203538_at	CAMLG	0	1.24	3.20E-45
203542_s_at	KLF9	0	1.72	1.04E-39
203544_s_at	STAM	1		N.S.
203545_at	ALG8	1	1.07	9.69E-08
203546_at	IPO13	0	-1.06	1.23E-04
203550_s_at	C1orf2	0	-1.27	2.80E-24
203552_at	MAP4K5	0	1.24	<1.00E-120
203556_at	ZHX2	0	1.17	2.72E-20
203557_s_at	PCBD1	0	-1.32	8.69E-37
203560_at	GGH	1		N.S.
203561_at	FCGR2A	0		N.S.
203562_at	FEZ1	1		N.S.
203564_at	FANCG	0	-1.13	1.81E-11
203565_s_at	MNAT1	0		N.S.
203566_s_at	AGL	0	-1.14	<1.00E-120
203567_s_at	TRIM38	0	1.07	1.48E-03
203569_s_at	OFD1	0	1.08	6.36E-06
203572_s_at	TAF6	0		N.S.
203573_s_at	RABGGTA	0		N.S.
203574_at	NFIL3	0	1.55	7.64E-30
203575_at	CSNK2A2	0	1.51	4.58E-72
203577_at	GTF2H4	0	-1.10	1.04E-10
203579_s_at	SLC7A6	0	-1.18	<1.00E-120

Supplemental Table 1

203581_at	RAB4A	0		N.S.
203583_at	UNC50	0	1.40	1.65E-58
203584_at	TTC35	0	1.18	1.33E-26
203588_s_at	TFDP2	0	1.06	1.02E-03
203590_at	DYNC1LI2	0	-1.14	2.98E-10
203592_s_at	FSTL3	0	-1.28	8.61E-08
203593_at	CD2AP	0		N.S.
203596_s_at	IFIT5	0	1.35	4.17E-23
203599_s_at	WBP4	0	1.08	1.14E-10
203600_s_at	C4orf8	0	1.06	5.79E-06
203604_at	ZNF516	0	1.26	6.09E-11
203605_at	SRP54	0	1.10	7.15E-11
203606_at	NDUFS6	0	-1.15	2.11E-31
203607_at	INPP5F	0		N.S.
203608_at	ALDH5A1	0		N.S.
203611_at	TERF2	0	-1.08	1.68E-09
203612_at	BYSL	0	-2.04	1.31E-72
203613_s_at	NDUFB6	0	-1.10	2.04E-10
203614_at	UTP14C	0	1.37	2.50E-60
203616_at	POLB	0		N.S.
203620_s_at	FCHSD2	0		N.S.
203621_at	NDUFB5	0	1.04	6.84E-05
203622_s_at	PNO1	0	-1.74	1.38E-81
203630_s_at	COG5	0	1.25	9.15E-21
203634_s_at	CPT1A	0		N.S.
203635_at	DSCR3	0	-1.09	2.67E-06
203643_at	ERF	0	-1.48	1.71E-39
203644_s_at	MON1B	0	1.36	1.27E-33
203647_s_at	FDX1	0	-1.09	<1.00E-120
203648_at	TATDN2	0	-1.18	7.79E-27
203651_at	ZFYVE16	0	1.21	3.69E-21
203652_at	MAP3K11	0	-1.08	7.76E-06
203653_s_at	COIL	3	-1.36	2.20E-29
203655_at	XRCC1	0		N.S.
203656_at	FIG4	0	1.08	1.74E-07
203658_at	SLC25A20	0		N.S.
203659_s_at	TRIM13	0	-1.33	1.28E-42
203660_s_at	PCNT	0	1.22	2.11E-31
203662_s_at	TMOD1	0	1.10	3.12E-08
203665_at	HMOX1	21	1.87	2.52E-48
203668_at	MAN2C1	0		N.S.
203669_s_at	DGAT1	1		N.S.
203671_at	TPMT	0	1.17	7.80E-04
203674_at	HELZ	0		N.S.
203675_at	NUCB2	0	2.67	1.98E-63
203677_s_at	TARBP2	0		N.S.
203678_at	MTMR15	0		N.S.
203679_at	TMED1	0		N.S.
203683_s_at	VEGFB	0		N.S.
203684_s_at	BCL2	8	1.20	6.37E-09
203686_at	MPG	8	1.12	7.52E-13
203688_at	PKD2	0	1.98	2.96E-95
203689_s_at	FMR1	0		N.S.
203693_s_at	E2F3	0	-1.27	5.84E-33

Supplemental Table 1

203694_s_at	DHX16	0		N.S.
203695_s_at	DFNA5	0	1.13	3.67E-35
203701_s_at	TRMT1	0	-1.18	5.21E-21
203702_s_at	TTLL4	0	-1.31	1.19E-35
203705_s_at	FZD7	0	1.38	1.64E-05
203707_at	ZNF263	0	1.07	1.22E-07
203709_at	PHKG2	0		N.S.
203711_s_at	HIBCH	0	-1.35	6.12E-38
203712_at	KIAA0020	0	-1.68	1.81E-83
203713_s_at	LLGL2	0	1.31	7.02E-04
203715_at	TBCE	0	-1.09	4.26E-02
203718_at	PNPLA6	0	-1.07	1.55E-02
203720_s_at	ERCC1	0	1.09	9.43E-07
203722_at	ALDH4A1	0	1.17	4.46E-08
203723_at	ITPKB	0	1.27	<1.00E-120
203725_at	GADD45A	4	1.82	5.86E-62
203727_at	SKIV2L	0	-1.34	4.34E-28
203728_at	BAK1	7	-1.13	5.27E-12
203729_at	EMP3	0	-1.20	3.95E-21
203731_s_at	ZKSCAN5	0		N.S.
203732_at	TRIP4	0	1.14	2.15E-24
203733_at	DEXI	0		N.S.
203734_at	FOXJ2	0	1.10	1.07E-06
203737_s_at	PPRC1	0	-1.78	1.09E-75
203738_at	C5orf22	0		N.S.
203739_at	ZNF217	0		N.S.
203740_at	MPHOSPH6	0	-1.23	1.84E-35
203741_s_at	ADCY7	0		N.S.
203743_s_at	TDG	0	-1.50	7.84E-72
203744_at	HMGB3	0	-1.31	8.60E-37
203745_at	HCCS	5	-1.06	9.56E-03
203752_s_at	JUND	0	-1.50	1.50E-68
203755_at	BUB1B	0	-1.26	3.39E-46
203758_at	CTSO	0	1.58	5.47E-64
203762_s_at	DYNC2LI1	0	1.37	5.15E-36
203764_at	DLGAP5	0	-1.27	5.63E-43
203765_at	GCA	1	-1.11	3.13E-09
203774_at	MTR	0	-1.13	1.65E-10
203775_at	SLC25A13	0	-1.26	3.03E-33
203776_at	GPKOW	0	1.05	1.33E-02
203777_s_at	RPS6KB2	0	-1.09	1.83E-03
203778_at	MANBA	0	1.16	4.27E-06
203781_at	MRPL33	0	-1.16	1.25E-26
203782_s_at	POLRMT	0	-1.55	1.82E-57
203787_at	SSBP2	0	-1.11	8.05E-11
203790_s_at	HRSP12	0	-1.12	1.16E-17
203791_at	DMXL1	0	1.24	1.38E-18
203795_s_at	BCL7A	0	-1.22	<1.00E-120
203799_at	CD302	0	-1.13	1.66E-08
203800_s_at	MRPS14	0		N.S.
203803_at	PCYOX1	0		N.S.
203804_s_at	CROP	4		N.S.
203805_s_at	FANCA	0	-1.43	1.02E-52
203810_at	DNAJB4	0	1.10	5.93E-03

Supplemental Table 1

203814_s_at	NQO2	0	1.25	6.49E-23
203815_at	GSTT1	0		N.S.
203816_at	DGUOK	0	1.22	5.85E-43
203817_at	GUCY1B3	0	-1.09	1.40E-02
203818_s_at	SF3A3	0	-1.19	5.49E-44
203820_s_at	IGF2BP3	0	1.22	6.24E-27
203822_s_at	ELF2	9		N.S.
203825_at	BRD3	0	1.28	4.18E-24
203826_s_at	PITPNM1	0	1.15	1.51E-05
203827_at	WIPI1	0	3.91	6.01E-96
203829_at	ELP4	0	1.07	6.22E-07
203830_at	C17orf75	0		N.S.
203831_at	R3HDM2	0	1.44	1.38E-45
203832_at	SNRPF	0	-1.30	2.93E-51
203836_s_at	MAP3K5	8	1.14	2.26E-12
203840_at	BLZF1	0		N.S.
203843_at	RPS6KA3	0	-1.15	<1.00E-120
203845_at	KAT2B	0	1.19	2.26E-12
203846_at	TRIM32	0		N.S.
203852_s_at	SMN1	0		N.S.
203855_at	WDR47	0	-1.09	8.85E-04
203856_at	VRK1	0	-1.56	8.42E-92
203857_s_at	PDIA5	0	1.75	1.66E-62
203858_s_at	COX10	0		N.S.
203860_at	PCCA	0		N.S.
203866_at	NLE1	0	-1.21	1.80E-09
203868_s_at	VCAM1	0		N.S.
203869_at	USP46	0	-1.40	3.98E-52
203871_at	SENP3	0	-1.29	5.17E-39
203879_at	PIK3CD	0	-1.22	4.63E-20
203880_at	COX17	0	-1.18	6.33E-38
203881_s_at	DMD	0		N.S.
203883_s_at	RAB11FIP2	0		N.S.
203885_at	RAB21	0	1.04	4.75E-03
203890_s_at	DAPK3	0		N.S.
203892_at	WFDC2	0		N.S.
203894_at	TUBG2	0	-1.10	7.61E-03
203897_at	LYRM1	0	1.20	5.27E-45
203899_s_at	CRCP	0	-1.18	3.46E-05
203900_at	KIAA0467	0	1.11	4.01E-05
203901_at	MAP3K7IP1	0		N.S.
203903_s_at	HEPH	0		N.S.
203905_at	PARN	0	1.06	4.99E-04
203907_s_at	IQSEC1	0	-1.08	5.82E-03
203909_at	SLC9A6	0	1.08	1.84E-08
203912_s_at	DNASE1L1	0		N.S.
203916_at	NDST2	0	1.09	1.39E-02
203919_at	TCEA2	0	1.23	1.17E-17
203920_at	NR1H3	0	-1.14	1.07E-05
203921_at	CHST2	0	-1.47	6.52E-42
203923_s_at	CYBB	0	1.62	1.57E-45
203925_at	GCLM	0	1.14	1.50E-12
203927_at	NFKBIE	0	-1.20	6.53E-24
203931_s_at	MRPL12	0	-1.56	3.77E-57

Supplemental Table 1

203932_at	HLA-DMB	0		N.S.
203933_at	RAB11FIP3	0	-1.23	9.03E-12
203935_at	ACVR1	0	1.60	9.59E-24
203936_s_at	MMP9	0		N.S.
203938_s_at	TAF1C	0		N.S.
203939_at	NT5E	0		N.S.
203941_at	INTS9	0	-1.29	7.10E-44
203942_s_at	MARK2	0		N.S.
203943_at	KIF3B	0	-1.15	1.02E-20
203945_at	ARG2	1	1.20	3.31E-07
203947_at	CSTF3	0		N.S.
203952_at	ATF6	247	1.31	9.02E-21
203955_at	KIAA0649	0		N.S.
203957_at	E2F6	0	-1.15	3.56E-20
203958_s_at	ZBTB40	0		N.S.
203960_s_at	HSPB11	0	-1.20	1.39E-21
203964_at	NMI	0		N.S.
203965_at	USP20	0	1.10	4.42E-02
203966_s_at	PPM1A	0		N.S.
203967_at	CDC6	0	1.41	6.66E-36
203970_s_at	PEX3	1		N.S.
203971_at	SLC31A1	0	1.28	9.70E-18
203973_s_at	CEBPD	1	-1.33	1.28E-11
203974_at	HDHD1A	0	-1.31	2.66E-49
203975_s_at	CHAF1A	0	-1.88	9.47E-57
203978_at	NUBP1	0	-1.18	2.93E-32
203983_at	TSNAX	0		N.S.
203984_s_at	CASP9	82		N.S.
203985_at	ZNF212	0		N.S.
203987_at	FZD6	0	1.14	2.78E-07
203988_s_at	FUT8	0		N.S.
203990_s_at	KDM6A	0		N.S.
204001_at	SNAPC3	0		N.S.
204003_s_at	NUPL2	0		N.S.
204004_at	PAWR	0	-1.20	1.83E-21
204008_at	DNAL4	0	1.44	<1.00E-120
204015_s_at	DUSP4	0	-1.33	5.16E-29
204016_at	LARS2	0	-1.32	1.19E-30
204019_s_at	SH3YL1	0	-1.12	2.49E-08
204021_s_at	PURA	2	-1.15	<1.00E-120
204023_at	RFC4	0	-1.17	3.44E-32
204025_s_at	PDCD2	0		N.S.
204026_s_at	ZWINT	0	-1.49	8.25E-79
204027_s_at	METTL1	0	-1.38	6.27E-39
204028_s_at	RABGAP1	0	1.30	1.01E-25
204030_s_at	SCHIP1	0		N.S.
204032_at	BCAR3	0	-1.55	2.78E-30
204033_at	TRIP13	0	-1.79	2.77E-74
204034_at	ETHE1	0		N.S.
204044_at	QPRT	0	-1.40	5.82E-48
204045_at	TCEAL1	0	-1.16	8.05E-08
204049_s_at	PHACTR2	0	-1.65	1.52E-21
204054_at	PTEN	5	1.36	3.64E-46
204057_at	IRF8	0	-1.09	6.53E-08

Supplemental Table 1

204059_s_at	ME1	1	1.35	1.60E-45
204061_at	PRKX	0	-1.10	1.90E-07
204064_at	THOC1	0	-1.12	3.08E-09
204065_at	CHST10	0	-1.09	5.70E-03
204067_at	SUOX	0	-1.18	5.31E-18
204068_at	STK3	0		N.S.
204070_at	RARRES3	0	1.29	2.20E-22
204071_s_at	TOPORS	0	-1.18	3.29E-06
204074_s_at	KIAA0562	0		N.S.
204076_at	ENTPD4	0	1.18	<1.00E-120
204079_at	TPST2	0		N.S.
204080_at	TOE1	0	-1.58	1.90E-63
204081_at	NRGN	0	-1.52	9.84E-24
204082_at	PBX3	0		N.S.
204085_s_at	CLN5	0	1.21	<1.00E-120
204087_s_at	SLC5A6	0	-1.30	1.48E-34
204088_at	P2RX4	0	1.81	9.10E-59
204091_at	PDE6D	0		N.S.
204093_at	CCNH	0	-1.09	<1.00E-120
204094_s_at	TSC22D2	0	1.09	4.95E-07
204096_s_at	ELL	1		N.S.
204098_at	RBMX2	0		N.S.
204102_s_at	EEF2	3	1.09	6.09E-11
204103_at	CCL4	2	-1.60	1.70E-31
204104_at	SNAPC2	0		N.S.
204106_at	TESK1	0		N.S.
204108_at	NFYA	0	-1.25	7.05E-34
204113_at	CUGBP1	0	-1.20	3.69E-11
204115_at	GNG11	0		N.S.
204116_at	IL2RG	0	1.22	6.32E-39
204117_at	PREP	0	-1.29	1.19E-30
204118_at	CD48	0	1.13	1.94E-20
204120_s_at	ADK	0		N.S.
204123_at	LIG3	0	-1.20	4.23E-04
204125_at	NDUFAF1	0	-1.39	8.84E-39
204126_s_at	CDC45L	0	-1.11	1.92E-07
204128_s_at	RFC3	0	-1.89	1.19E-57
204131_s_at	FOXO3	2	1.16	1.86E-04
204133_at	RRP9	0	-1.95	3.33E-57
204135_at	FILIP1L	0		N.S.
204137_at	GPR137B	0	-1.10	1.66E-06
204140_at	TPST1	0		N.S.
204141_at	TUBB2A	0	-1.65	2.98E-61
204142_at	ENOSF1	0		N.S.
204144_s_at	PIGQ	0		N.S.
204145_at	FRG1	0	1.07	3.92E-04
204146_at	RAD51AP1	0		N.S.
204149_s_at	GSTM4	0		N.S.
204153_s_at	MFNG	0	-1.35	8.56E-56
204156_at	KIAA0999	0		N.S.
204158_s_at	TCIRG1	0		N.S.
204160_s_at	ENPP4	0		N.S.
204162_at	NDC80	0		N.S.
204164_at	SIPA1	0	-1.56	9.81E-48

Supplemental Table 1

204165_at	WASF1	0	-1.38	1.73E-22
204168_at	MGST2	0		N.S.
204169_at	IMPDH1	0	-1.33	1.13E-23
204170_s_at	CKS2	0	-1.35	3.67E-60
204171_at	RPS6KB1	0	-1.30	<1.00E-120
204172_at	CPOX	0		N.S.
204173_at	MYL6B	0		N.S.
204174_at	ALOX5AP	0	3.57	1.03E-71
204175_at	ZNF593	0	-1.43	5.31E-49
204178_s_at	RBM14	0	-1.57	3.35E-66
204181_s_at	ZBTB43	0	1.18	<1.00E-120
204183_s_at	ADRBK2	0		N.S.
204186_s_at	PPID	0	-1.16	9.50E-29
204190_at	USPL1	0	-1.07	2.55E-03
204191_at	IFNAR1	4	1.14	5.94E-06
204192_at	CD37	0	1.10	1.50E-12
204198_s_at	RUNX3	0		N.S.
204201_s_at	PTPN13	0		N.S.
204203_at	CEBPG	0	2.31	4.55E-96
204205_at	APOBEC3G	0		N.S.
204206_at	MNT	0	1.22	4.78E-22
204208_at	RNGTT	0	1.09	1.19E-05
204209_at	PCYT1A	1	1.08	1.88E-05
204212_at	ACOT8	0	1.17	<1.00E-120
204215_at	C7orf23	0	1.28	2.34E-57
204216_s_at	ZC3H14	0	-1.17	1.13E-17
204218_at	C11orf51	0		N.S.
204219_s_at	PSMC1	0	-1.24	2.02E-36
204220_at	GMFG	0		N.S.
204222_s_at	GLIPR1	0	1.36	1.26E-35
204224_s_at	GCH1	1	1.44	1.87E-73
204225_at	HDAC4	0		N.S.
204226_at	STAU2	0	-1.24	3.38E-40
204228_at	PPIH	0	-1.16	1.14E-26
204232_at	FCER1G	0		N.S.
204233_s_at	CHKA	0		N.S.
204234_s_at	ZNF195	0		N.S.
204241_at	ACOX3	0		N.S.
204243_at	RLF	0	-1.10	2.26E-09
204244_s_at	DBF4	0	-1.19	5.38E-22
204245_s_at	RPP14	0	-1.32	8.24E-43
204246_s_at	DCTN3	0	1.14	7.52E-13
204247_s_at	CDK5	1	-1.15	8.00E-22
204249_s_at	LMO2	0	1.67	5.75E-29
204250_s_at	CEP164	0		N.S.
204258_at	CHD1	0	-1.16	5.01E-24
204263_s_at	CPT2	0		N.S.
204265_s_at	GPSM3	0		N.S.
204269_at	PIM2	0	1.42	1.14E-23
204275_at	SOLH	0	-1.25	3.02E-24
204276_at	TK2	0	1.50	<1.00E-120
204278_s_at	EBAG9	0	1.30	3.12E-63
204279_at	PSMB9	0	1.12	5.57E-18
204286_s_at	PMAIP1	7	1.53	1.27E-46

Supplemental Table 1

204291_at	ZNF518A	0	1.20	<1.00E-120
204294_at	AMT	0	1.42	9.21E-31
204295_at	SURF1	0	1.25	7.98E-27
204296_at	DCTN1	0	1.90	1.09E-31
204297_at	PIK3C3	2	1.08	1.36E-09
204299_at	FUSIP1	0	-1.22	1.56E-21
204300_at	PET112L	0		N.S.
204305_at	MIPEP	0	-1.13	3.76E-12
204306_s_at	CD151	0	-1.59	<1.00E-120
204308_s_at	TECPR2	0		N.S.
204319_s_at	RGS10	0	1.08	<1.00E-120
204327_s_at	ZNF202	0	-1.14	2.81E-08
204331_s_at	MRPS12	0	-1.40	5.41E-61
204332_s_at	AGA	0	1.97	1.71E-79
204334_at	KLF7	0	1.27	3.39E-19
204335_at	CCDC94	0		N.S.
204336_s_at	RGS19	0	-1.27	5.49E-37
204340_at	TMEM187	0	1.22	1.25E-09
204341_at	TRIM16	0	1.18	1.12E-06
204342_at	SLC25A24	0		N.S.
204346_s_at	RASSF1	0		N.S.
204347_at	AK3L1	0	-1.72	3.10E-37
204350_s_at	MED7	0	-1.17	1.32E-22
204352_at	TRAF5	0	-1.10	4.10E-10
204354_at	POT1	0	1.19	1.01E-33
204369_at	PIK3CA	0	1.19	2.50E-30
204370_at	CLP1	0	-1.30	4.31E-56
204372_s_at	KHSRP	0	-1.14	2.05E-33
204373_s_at	CEP350	0	1.09	1.37E-05
204375_at	CLSTN3	0		N.S.
204377_s_at	VPRBP	0	-1.21	5.04E-03
204378_at	BCAS1	0		N.S.
204382_at	NAT9	0		N.S.
204394_at	SLC43A1	0	2.97	1.15E-82
204401_at	KCNN4	0		N.S.
204404_at	SLC12A2	0	-1.22	9.82E-35
204407_at	TTF2	0	-1.60	2.23E-25
204408_at	APEX2	0	-1.42	6.62E-19
204411_at	KIF21B	0	1.59	7.69E-26
204413_at	TRAF2	12	-1.18	7.28E-03
204415_at	IFI6	0		N.S.
204421_s_at	FGF2	0	1.77	1.62E-35
204423_at	MKLN1	0	-1.23	2.65E-35
204425_at	ARHGAP4	0		N.S.
204426_at	TMED2	0	1.63	2.61E-18
204430_s_at	SLC2A5	0		N.S.
204432_at	SOX12	0	-1.53	9.26E-34
204433_s_at	SPATA2	0		N.S.
204435_at	NUPL1	0	-1.25	8.65E-26
204436_at	PLEKHO2	0		N.S.
204439_at	IFI44L	0	1.17	<1.00E-120
204440_at	CD83	0	-1.69	3.40E-60
204441_s_at	POLA2	0	-1.39	1.54E-43
204444_at	KIF11	0	-1.15	5.54E-18

Supplemental Table 1

204448_s_at	PDCL	0	-1.16	4.51E-12
204453_at	ZNF84	0	-1.15	1.66E-22
204458_at	PLA2G15	0		N.S.
204459_at	CSTF2	0	-1.33	8.50E-46
204460_s_at	RAD1	0	-1.39	6.38E-43
204472_at	GEM	0		N.S.
204473_s_at	ZNF592	0		N.S.
204474_at	ZNF142	0	-1.08	2.32E-04
204477_at	RABIF	0		N.S.
204479_at	OSTF1	0	-1.09	9.50E-08
204481_at	BRPF1	0	-1.15	3.15E-07
204483_at	ENO3	0	1.14	1.07E-04
204484_at	PIK3C2B	0	1.20	1.20E-11
204485_s_at	TOM1L1	0		N.S.
204488_at	DOLK	0	-1.08	1.23E-04
204492_at	ARHGAP11A	0	-1.16	6.58E-07
204502_at	SAMHD1	0	-1.63	2.03E-69
204504_s_at	HIRIP3	0	1.08	2.69E-08
204506_at	PPP3R1	0	1.18	1.85E-22
204510_at	CDC7	0	-1.51	1.34E-66
204511_at	FARP2	0	1.30	3.67E-10
204512_at	HIVEP1	0		N.S.
204513_s_at	ELMO1	0	1.11	3.01E-12
204514_at	DPH2	0	-1.60	2.39E-47
204516_at	ATXN7	0	1.27	1.66E-05
204521_at	C12orf24	0	-1.65	3.43E-67
204522_at	DOM3Z	0	1.25	<1.00E-120
204523_at	ZNF140	0	1.36	1.14E-56
204531_s_at	BRCA1	2	-1.10	9.55E-11
204533_at	CXCL10	1	-1.91	8.85E-25
204544_at	HPS5	0	-1.10	8.74E-20
204545_at	PEX6	0	-1.14	1.63E-03
204546_at	KIAA0513	0	1.67	1.52E-17
204547_at	RAB40B	0		N.S.
204552_at	INPP4A	0	1.39	6.82E-51
204554_at	PPP1R3D	0	1.10	3.32E-06
204558_at	RAD54L	0	-1.73	9.59E-45
204559_s_at	LSM7	0	-1.10	8.63E-08
204562_at	IRF4	2	1.41	1.24E-48
204563_at	SELL	1	-1.16	<1.00E-120
204565_at	ACOT13	0	-1.18	1.60E-29
204566_at	PPM1D	0		N.S.
204568_at	KIAA0831	0	1.15	2.23E-18
204569_at	ICK	0	1.37	<1.00E-120
204573_at	CROT	0	-1.29	1.42E-20
204576_s_at	CLUAP1	0	1.19	2.26E-12
204578_at	HISPPD2A	0	1.46	<1.00E-120
204588_s_at	SLC7A7	0	1.63	7.67E-45
204593_s_at	SMCR7L	0	-1.06	5.32E-07
204599_s_at	MRPL28	0	-1.27	2.30E-23
204603_at	EXO1	0	-1.70	1.84E-79
204605_at	CGRRF1	0	1.64	5.32E-75
204608_at	ASL	1	1.14	1.69E-08
204610_s_at	CCDC85B	0	-2.07	3.11E-75

Supplemental Table 1

204612_at	PKIA	0	-1.42	1.59E-40
204613_at	PLCG2	0		N.S.
204616_at	UCHL3	0	-1.06	2.36E-02
204617_s_at	ACD	0	-1.24	1.40E-30
204618_s_at	GABPB1	0	1.18	<1.00E-120
204630_s_at	GOSR1	0		N.S.
204632_at	RPS6KA4	0	-1.35	1.50E-12
204634_at	NEK4	0	1.08	3.55E-05
204635_at	RPS6KA5	0	1.33	<1.00E-120
204638_at	ACP5	0	-1.18	9.40E-09
204641_at	NEK2	0		N.S.
204642_at	S1PR1	0		N.S.
204646_at	DPYD	0	1.22	1.44E-03
204649_at	TROAP	0		N.S.
204650_s_at	APBB3	0	1.20	5.74E-07
204651_at	NRF1	1		N.S.
204659_s_at	GFER	0		N.S.
204662_at	CP110	0		N.S.
204666_s_at	RP5-1000E10.4	0		N.S.
204668_at	RNF24	0	1.17	7.48E-04
204672_s_at	ANKRD6	0		N.S.
204674_at	LRMP	0	1.29	3.46E-25
204676_at	TMEM186	0	-1.13	1.98E-09
204678_s_at	KCNK1	0	1.21	1.50E-12
204683_at	ICAM2	0		N.S.
204687_at	DKFZP564O0823	0	1.23	<1.00E-120
204688_at	SGCE	0	1.25	4.71E-34
204690_at	STX8	0		N.S.
204695_at	CDC25A	1	-1.97	7.43E-63
204698_at	ISG20	0	1.27	1.16E-23
204702_s_at	NFE2L3	0	-1.15	1.48E-10
204703_at	IFT88	0	1.11	<1.00E-120
204706_at	INPP5E	0		N.S.
204709_s_at	KIF23	0	-1.15	3.60E-09
204710_s_at	WIPI2	0	1.23	2.73E-38
204711_at	KIAA0753	0		N.S.
204715_at	PANX1	0	-1.08	1.03E-05
204716_at	CCDC6	0		N.S.
204717_s_at	SLC29A2	0	-2.52	4.07E-38
204720_s_at	DNAJC6	0	1.71	7.11E-30
204725_s_at	NCK1	2		N.S.
204727_at	WDHD1	0	-1.62	1.84E-29
204730_at	RIMS3	0	1.68	<1.00E-120
204731_at	TGFBR3	0		N.S.
204735_at	PDE4A	0	-1.24	8.28E-23
204739_at	CENPC1	0	-1.13	<1.00E-120
204740_at	CNKSR1	0		N.S.
204742_s_at	PDS5B	0	-1.22	3.89E-20
204744_s_at	IARS	0	1.67	1.93E-90
204747_at	IFIT3	0		N.S.
204757_s_at	C2CD2L	0	1.48	5.73E-23
204759_at	RCBTB2	0		N.S.
204761_at	USP6NL	0		N.S.
204765_at	ARHGEF5	0	-1.20	4.67E-07

Supplemental Table 1

204766_s_at	NUDT1	0	-1.20	<1.00E-120
204767_s_at	FEN1	0	-1.81	6.86E-74
204770_at	TAP2	0		N.S.
204771_s_at	TTF1	0	-1.37	1.94E-39
204773_at	IL11RA	0		N.S.
204774_at	EVI2A	0	1.57	1.51E-48
204777_s_at	MAL	0	1.27	<1.00E-120
204779_s_at	HOXB7	0	1.13	2.41E-11
204781_s_at	FAS	28	2.00	2.10E-88
204786_s_at	IFNAR2	0		N.S.
204788_s_at	PPOX	0	1.20	1.59E-29
204789_at	FMNL1	0		N.S.
204790_at	SMAD7	0	1.36	1.48E-31
204793_at	GPRASP1	0	1.25	2.28E-02
204794_at	DUSP2	0	-1.15	7.52E-13
204795_at	PRR3	0	-1.73	6.46E-72
204798_at	MYB	1	-1.31	4.76E-24
204804_at	TRIM21	0	-1.27	1.43E-22
204805_s_at	H1FX	0	1.50	4.70E-47
204807_at	TMEM5	0		N.S.
204809_at	CLPX	0		N.S.
204812_at	ZW10	0		N.S.
204813_at	MAPK10	0		N.S.
204817_at	ESPL1	0	-1.11	3.01E-07
204821_at	BTN3A3	0	1.50	6.48E-53
204822_at	TTK	0	-1.08	1.93E-08
204824_at	ENDOG	0	-1.43	5.91E-33
204825_at	MELK	0	-1.20	8.91E-34
204827_s_at	CCNF	0	-1.35	3.10E-18
204828_at	RAD9A	0	1.61	1.27E-62
204831_at	CDK8	0		N.S.
204832_s_at	BMPR1A	0		N.S.
204834_at	FGL2	0	-1.23	1.29E-08
204835_at	POLA1	0	-1.08	4.58E-05
204836_at	GLDC	0	-1.38	4.49E-38
204838_s_at	MLH3	0	-1.50	6.14E-44
204839_at	POP5	0	-1.12	2.54E-18
204840_s_at	EEA1	1	-1.39	1.35E-11
204847_at	ZBTB11	0	-1.15	8.15E-30
204849_at	TCFL5	0	-1.25	1.73E-26
204852_s_at	PTPN7	0	-1.06	1.11E-03
204853_at	ORC2L	0	-1.33	6.82E-40
204857_at	MAD1L1	0	1.10	6.36E-08
204858_s_at	TYMP	0		N.S.
204861_s_at	NAIP	0		N.S.
204862_s_at	NME3	0	-1.18	<1.00E-120
204863_s_at	IL6ST	0	1.38	<1.00E-120
204866_at	PHF16	0	-1.28	7.37E-03
204867_at	GCHFR	0		N.S.
204868_at	ICT1	0	1.06	1.02E-02
204871_at	MTERF	0	-1.22	<1.00E-120
204880_at	MGMT	0		N.S.
204881_s_at	UGCG	0	-1.21	<1.00E-120
204883_s_at	HUS1	0	-1.20	1.68E-18

Supplemental Table 1

204887_s_at	PLK4	0	-1.41	1.58E-45
204890_s_at	LCK	0	-1.26	3.09E-25
204902_s_at	ATG4B	0	1.23	4.59E-11
204905_s_at	EEF1E1	0	-1.43	1.83E-57
204909_at	DDX6	0	-1.27	<1.00E-120
204912_at	IL10RA	0	1.39	1.75E-36
204917_s_at	MLLT3	0	1.10	1.12E-05
204919_at	PRR4	0	1.35	<1.00E-120
204923_at	SASH3	0		N.S.
204928_s_at	SLC10A3	0	1.24	7.52E-13
204932_at	TNFRSF11B	2	-1.38	9.89E-04
204936_at	MAP4K2	0		N.S.
204937_s_at	ZNF274	0	1.10	<1.00E-120
204946_s_at	TOP3A	0	-1.26	7.72E-22
204949_at	ICAM3	0	1.08	3.76E-12
204950_at	CARD8	0	1.16	9.98E-26
204959_at	MNDA	0	-1.56	3.88E-70
204960_at	PTPRCAP	0		N.S.
204961_s_at	LOC648998	0		N.S.
204962_s_at	CENPA	0	1.16	1.02E-29
204968_at	C6orf47	0		N.S.
204970_s_at	MAFG	0	1.53	2.76E-48
204971_at	CSTA	0	2.98	2.01E-41
204976_s_at	AMMECR1	0	1.27	9.70E-37
204977_at	DDX10	0	-1.66	2.04E-76
204978_at	SFRS16	0		N.S.
204979_s_at	SH3BGR	0	1.29	8.99E-05
204981_at	SLC22A18	0	1.32	<1.00E-120
204982_at	GIT2	0		N.S.
204985_s_at	TRAPPC6A	0	1.29	8.54E-44
204992_s_at	PFN2	0	-1.09	2.22E-05
204994_at	MX2	0		N.S.
204995_at	CDK5R1	0	1.41	7.89E-31
205002_at	AHDC1	0	-1.14	3.52E-07
205003_at	DOCK4	0		N.S.
205004_at	NKRF	0	-1.66	2.62E-68
205010_at	GNL3L	0	-1.25	<1.00E-120
205012_s_at	HAGH	0	1.30	9.61E-26
205013_s_at	ADORA2A	0		N.S.
205020_s_at	ARL4A	0		N.S.
205025_at	ZBTB48	0	1.14	1.87E-05
205027_s_at	MAP3K8	1	1.41	4.10E-24
205034_at	CCNE2	0	-2.75	9.13E-78
205035_at	CTDP1	0		N.S.
205036_at	LSM6	0	-1.31	3.30E-45
205042_at	GNE	0	-1.09	5.73E-05
205046_at	CENPE	0		N.S.
205047_s_at	ASNS	3	3.85	7.52E-109
205048_s_at	PSPH	0	2.65	2.46E-49
205049_s_at	CD79A	0	1.19	6.06E-29
205052_at	AUH	0	1.14	4.44E-11
205053_at	PRIM1	0	1.08	4.45E-08
205055_at	ITGAE	0	1.22	3.02E-25
205060_at	PARG	0		N.S.

Supplemental Table 1

205061_s_at	EXOSC9	0	-1.62	7.04E-75
205063_at	SIP1	0		N.S.
205069_s_at	ARHGAP26	0	1.31	1.80E-03
205070_at	ING3	0	1.17	3.34E-20
205074_at	SLC22A5	0	1.13	3.58E-04
205078_at	PIGF	0		N.S.
205081_at	CRIP1	0	-1.27	1.34E-32
205085_at	ORC1L	0	-1.42	6.89E-46
205087_at	RWDD3	0		N.S.
205089_at	ZNF7	0		N.S.
205090_s_at	NAGPA	0		N.S.
205094_at	PEX12	0	1.19	1.05E-29
205097_at	SLC26A2	0	1.46	1.53E-40
205098_at	CCR1	0	-1.44	5.59E-22
205101_at	CIITA	0		N.S.
205105_at	MAN2A1	0	-1.09	1.09E-03
205107_s_at	EFNA4	0		N.S.
205114_s_at	CCL3	1	-1.31	7.08E-26
205124_at	LOC729991-MEF2B	0	4.15	1.03E-77
205126_at	VRK2	0	1.12	<1.00E-120
205129_at	NPM3	0	-1.13	1.75E-19
205132_at	ACTC1	0		N.S.
205133_s_at	HSPE1	0	-2.20	1.62E-88
205134_s_at	NUFIP1	0	-1.63	6.56E-56
205140_at	FPGT	0	-1.18	1.11E-09
205141_at	ANG	2	3.83	7.92E-57
205145_s_at	LOC649851	0	1.13	8.11E-03
205158_at	RNASE4	0	7.00	1.33E-64
205159_at	CSF2RB	0	-1.48	5.29E-46
205162_at	ERCC8	0	1.44	4.80E-40
205168_at	DDR2	0	2.25	1.08E-26
205169_at	RBBP5	0	-1.09	1.39E-04
205170_at	STAT2	0		N.S.
205171_at	PTPN4	0	1.16	1.50E-12
205174_s_at	QPCT	0		N.S.
205176_s_at	ITGB3BP	0	-1.04	1.73E-02
205178_s_at	RBBP6	1	1.26	5.29E-36
205181_at	ZNF193	0	1.13	5.28E-07
205188_s_at	SMAD5	0		N.S.
205189_s_at	FANCC	0		N.S.
205190_at	PLS1	0	-1.42	6.24E-11
205191_at	RP2	0		N.S.
205192_at	MAP3K14	1		N.S.
205196_s_at	AP1S1	0	-1.43	2.03E-36
205198_s_at	ATP7A	1		N.S.
205205_at	RELB	0	1.11	2.13E-04
205210_at	TGFBRAP1	0	-1.44	<1.00E-120
205211_s_at	RIN1	0		N.S.
205212_s_at	ACAP1	0		N.S.
205215_at	RNF2	0		N.S.
205217_at	TIMM8A	0	-2.26	9.78E-66
205218_at	POLR3F	0		N.S.
205220_at	NIACR2	0	3.91	1.86E-66
205222_at	EHHADH	0		N.S.

Supplemental Table 1

205223_at	DEPDC5	0		N.S.
205224_at	SURF2	0	-1.29	7.90E-19
205229_s_at	COCH	0	-1.65	3.21E-57
205231_s_at	EPM2A	2		N.S.
205233_s_at	PAFAH2	0	1.16	1.48E-04
205235_s_at	KIF20B	0		N.S.
205238_at	TRMT2B	0	1.13	6.30E-06
205241_at	SCO2	0	-1.30	4.55E-46
205245_at	PARD6A	0	-1.20	6.77E-12
205246_at	PEX13	0	-1.15	1.13E-11
205247_at	NOTCH4	0		N.S.
205248_at	DOPEY2	0		N.S.
205249_at	EGR2	0		N.S.
205252_at	ZNF174	0		N.S.
205256_at	ZBTB39	0		N.S.
205260_s_at	ACYP1	0	-1.35	7.37E-34
205263_at	BCL10	0		N.S.
205264_at	CD3EAP	0	-2.61	4.33E-77
205267_at	POU2AF1	1	-1.05	2.63E-03
205269_at	LCP2	0		N.S.
205273_s_at	PITRM1	0		N.S.
205283_at	FKTN	0		N.S.
205291_at	IL2RB	0		N.S.
205292_s_at	HNRNPA2B1	0	-1.17	1.89E-28
205296_at	RBL1	0	-1.22	1.50E-12
205297_s_at	CD79B	0	1.20	3.55E-31
205298_s_at	BTN2A2	0	1.17	1.20E-22
205300_s_at	SNRNP35	0	-1.30	3.18E-29
205301_s_at	OGG1	0	-1.38	1.01E-23
205307_s_at	KMO	0		N.S.
205308_at	FAM164A	0	1.15	4.48E-07
205310_at	FBXO46	0		N.S.
205312_at	SPI1	0		N.S.
205313_at	HNF1B	1	-1.23	4.24E-05
205315_s_at	SNTB2	0	-1.36	5.45E-30
205317_s_at	SLC15A2	0	1.25	2.24E-05
205321_at	EIF2S3	0		N.S.
205322_s_at	MTF1	0		N.S.
205327_s_at	ACVR2A	0	1.26	3.21E-07
205329_s_at	SNX4	0	-1.07	5.66E-10
205333_s_at	RCE1	0		N.S.
205335_s_at	SRP19	0	1.32	1.11E-47
205339_at	STIL	0		N.S.
205340_at	ZBTB24	1	-1.20	7.14E-21
205345_at	BARD1	0	-1.41	1.13E-46
205346_at	ST3GAL2	0	-1.50	<1.00E-120
205347_s_at	TMSB15A	0	-1.25	6.30E-03
205349_at	GNA15	0	-1.13	6.48E-03
205352_at	SERPINI1	0	1.63	2.64E-61
205354_at	GAMT	0	-1.17	4.03E-07
205355_at	ACADSB	0		N.S.
205356_at	USP13	0	-1.48	2.00E-49
205361_s_at	PFDN4	0		N.S.
205367_at	SH2B2	0	1.08	3.04E-04

Supplemental Table 1

205372_at	PLAG1	0	1.34	1.81E-25
205373_at	CTNNA2	0	1.74	<1.00E-120
205376_at	INPP4B	0	1.25	2.71E-11
205393_s_at	CHEK1	0	-1.34	8.91E-39
205400_at	WAS	2961	-1.15	5.08E-04
205401_at	AGPS	0	-1.37	6.87E-45
205406_s_at	SPA17	0	-1.07	1.18E-03
205407_at	RECK	0	1.38	4.86E-04
205408_at	MLLT10	0		N.S.
205411_at	STK4	0		N.S.
205412_at	ACAT1	0		N.S.
205414_s_at	RICH2	0		N.S.
205416_s_at	ATXN3	1	1.23	2.27E-25
205417_s_at	DAG1	1	-1.08	4.30E-03
205419_at	GPR183	0	-1.64	5.21E-33
205423_at	AP1B1	0	-1.19	1.08E-21
205425_at	HIP1	0		N.S.
205427_at	ZNF354A	0		N.S.
205429_s_at	MPP6	0	-1.31	3.37E-32
205433_at	BCHE	0	2.34	2.72E-21
205436_s_at	H2AFX	0	-1.35	4.31E-62
205437_at	ZNF211	0	1.17	<1.00E-120
205441_at	OCEL1	0		N.S.
205443_at	SNAPC1	0		N.S.
205449_at	SAC3D1	0	-1.36	7.68E-36
205452_at	PIGB	0	1.13	3.49E-20
205453_at	HOXB2	0	1.23	1.07E-07
205461_at	RAB35	0		N.S.
205462_s_at	HPCAL1	0		N.S.
205463_s_at	PDGFA	0		N.S.
205467_at	CASP10	1	1.19	1.06E-04
205469_s_at	IRF5	0		N.S.
205474_at	CRLF3	0	-1.04	8.16E-06
205480_s_at	UGP2	0	1.33	7.01E-67
205483_s_at	ISG15	0		N.S.
205484_at	SIT1	0	-1.23	2.03E-06
205486_at	TESK2	0	1.34	1.37E-36
205497_at	ZNF175	0		N.S.
205500_at	C5	0		N.S.
205504_at	BTK	0		N.S.
205505_at	GCNT1	0	-1.36	<1.00E-120
205511_at	FLJ10038	0		N.S.
205512_s_at	AIFM1	4	1.07	2.37E-05
205518_s_at	CMAH	0	1.45	1.08E-39
205519_at	WDR76	0	-1.35	1.18E-24
205521_at	EXOG	0	-1.26	2.67E-29
205526_s_at	KATNA1	0		N.S.
205527_s_at	GEMIN4	0	-2.22	2.22E-86
205536_at	VAV2	0		N.S.
205539_at	AVIL	1	1.73	8.19E-31
205540_s_at	RRAGB	0		N.S.
205541_s_at	GSPT2	0	-1.21	3.10E-38
205542_at	STEAP1	0	2.09	7.16E-50
205543_at	HSPA4L	0	-1.46	8.80E-67

Supplemental Table 1

205544_s_at	CR2	0	-1.08	4.25E-05
205546_s_at	TYK2	0		N.S.
205547_s_at	TAGLN	1	1.25	6.32E-11
205550_s_at	BRE	0	1.09	<1.00E-120
205551_at	SV2B	0	2.89	2.61E-52
205552_s_at	OAS1	0	1.16	2.39E-08
205554_s_at	DNASE1L3	0		N.S.
205558_at	TRAF6	0		N.S.
205562_at	RPP38	0	-1.21	6.15E-28
205565_s_at	FXN	0		N.S.
205569_at	LAMP3	1	1.62	4.19E-58
205570_at	PIP4K2A	0	-1.15	7.52E-13
205571_at	LIPT1	0	-1.09	1.43E-07
205584_at	ALG13	0	1.53	1.18E-31
205585_at	ETV6	0	1.08	1.37E-02
205588_s_at	FGFR1OP	0	-1.11	<1.00E-120
205590_at	RASGRP1	0		N.S.
205594_at	ZNF652	0	1.16	7.76E-06
205596_s_at	SMURF2	0	1.09	2.33E-10
205599_at	TRAF1	1	-1.71	1.64E-67
205603_s_at	DIAPH2	0		N.S.
205607_s_at	SCYL3	0	1.09	7.52E-12
205621_at	ALKBH1	0	-1.11	2.63E-09
205628_at	PRIM2	0	-1.12	1.69E-02
205633_s_at	ALAS1	1	-1.13	3.01E-12
205642_at	CEP110	0	1.13	<1.00E-120
205644_s_at	SNRPG	0	-1.22	1.69E-44
205647_at	RAD52	0		N.S.
205652_s_at	TTLL1	0	1.53	2.43E-24
205657_at	HAAO	0		N.S.
205658_s_at	SNAPC4	0	-1.46	1.53E-41
205659_at	HDAC9	0	-1.12	4.05E-02
205664_at	KIN	0	-1.05	2.29E-02
205667_at	WRN	0		N.S.
205668_at	LY75	0	1.35	8.92E-43
205671_s_at	HLA-DOB	0	1.14	1.82E-09
205672_at	XPA	1		N.S.
205677_s_at	DLEU1	0	-1.27	1.12E-43
205681_at	BCL2A1	0	-1.17	7.91E-18
205684_s_at	DENND4C	0	1.12	2.70E-09
205687_at	UBFD1	0		N.S.
205691_at	SYNGR3	0	1.48	8.09E-28
205692_s_at	CD38	0	1.21	<1.00E-120
205704_s_at	ATP6V0A2	0	1.77	1.91E-50
205705_at	ANKRD26	0		N.S.
205707_at	IL17RA	0	1.11	4.35E-04
205708_s_at	TRPM2	2		N.S.
205716_at	SLC25A40	0		N.S.
205718_at	ITGB7	0	1.25	4.27E-27
205733_at	BLM	0	-1.31	1.50E-12
205740_s_at	RBM42	0		N.S.
205746_s_at	ADAM17	0	1.54	3.36E-32
205748_s_at	RNF126	0	-1.37	2.85E-51
205750_at	BPHL	0	-1.19	4.11E-19

Supplemental Table 1

205756_s_at	F8	0	1.35	7.27E-38
205761_s_at	DUS4L	0	1.25	1.03E-31
205770_at	GSR	0	-1.24	1.63E-21
205773_at	CPEB3	0	1.49	7.52E-12
205774_at	F12	0	-1.20	5.27E-12
205775_at	FAM50B	0		N.S.
205780_at	BIK	3	-1.40	9.13E-20
205781_at	C16orf7	0	1.36	7.52E-13
205788_s_at	ZC3H11A	0	1.23	1.79E-57
205790_at	SKAP1	0	1.11	5.37E-10
205796_at	TCP11L1	0	-1.14	1.76E-17
205801_s_at	RASGRP3	0	1.68	4.36E-76
205804_s_at	TRAF3IP3	0	1.75	9.45E-56
205807_s_at	TUFT1	0	1.42	6.02E-25
205809_s_at	WASL	0		N.S.
205811_at	POLG2	0	-1.14	1.64E-18
205830_at	CLGN	0	2.04	<1.00E-120
205832_at	CPA4	0		N.S.
205839_s_at	BZRAP1	0	-1.17	6.06E-07
205841_at	JAK2	3		N.S.
205848_at	GAS2	0	1.47	3.65E-27
205849_s_at	UQCRB	0		N.S.
205851_at	NME6	0		N.S.
205855_at	ZNF197	0		N.S.
205859_at	LY86	0		N.S.
205861_at	SPIB	0	-2.45	4.13E-80
205865_at	ARID3A	0		N.S.
205873_at	PIGL	0		N.S.
205877_s_at	ZC3H7B	0	-1.22	<1.00E-120
205881_at	ZNF74	0		N.S.
205884_at	ITGA4	0	-1.46	8.02E-30
205890_s_at	GABBR1	0	-1.66	2.96E-36
205895_s_at	NOLC1	0	-2.05	4.48E-83
205901_at	PNOC	0	1.13	1.24E-08
205902_at	KCNN3	0	1.15	1.92E-10
205909_at	POLE2	0	-1.85	5.70E-85
205917_at	ZNF264	0	1.15	8.12E-11
205922_at	VNN2	0	1.81	2.33E-66
205928_at	ZNF443	0	-1.29	3.64E-31
205930_at	GTF2E1	0	-1.22	2.14E-28
205932_s_at	MSX1	0		N.S.
205933_at	SETBP1	0	-1.23	2.54E-02
205945_at	IL6R	0	-1.38	1.35E-24
205953_at	LRIG2	0	1.06	3.89E-02
205955_at	TAF6L	0		N.S.
205963_s_at	DNAJA3	0		N.S.
205964_at	ZNF426	0	1.17	4.82E-05
205965_at	BATF	0	-1.30	6.87E-32
205966_at	TAF13	0	-1.38	1.96E-11
205967_at	HIST1H4C	0		N.S.
205978_at	KL	0		N.S.
205981_s_at	ING2	0	-1.36	4.21E-43
205990_s_at	WNT5A	0	2.28	2.34E-25
205992_s_at	IL15	1	2.11	4.05E-75

Supplemental Table 1

205994_at	ELK4	0		N.S.
205996_s_at	AK2	0	-1.51	8.77E-54
206006_s_at	KIAA1009	0		N.S.
206016_at	CCDC22	0	-1.06	5.30E-03
206020_at	SOCS6	0	-1.20	1.92E-02
206026_s_at	TNFAIP6	0	1.26	1.73E-11
206031_s_at	USP5	0	-1.16	1.72E-02
206034_at	SERPINB8	0	1.36	4.69E-32
206035_at	REL	1	-1.37	2.05E-06
206037_at	CCBL1	0	-1.45	1.21E-33
206038_s_at	NR2C2	0		N.S.
206039_at	RAB33A	0	1.79	7.03E-68
206044_s_at	BRAF	1	1.35	2.48E-33
206045_s_at	NOL4	0	-1.36	6.29E-07
206050_s_at	RNH1	0		N.S.
206052_s_at	SLBP	0	-1.90	4.66E-92
206053_at	ZNF510	0		N.S.
206055_s_at	SNRPA1	0	-1.43	1.49E-46
206059_at	ZNF91	0		N.S.
206061_s_at	DICER1	0	-1.21	<1.00E-120
206074_s_at	HMGA1	2	-1.30	3.42E-55
206082_at	HCP5	0	-1.23	4.71E-38
206096_at	ZNF35	0		N.S.
206098_at	ZBTB6	0		N.S.
206102_at	GINS1	0	-1.73	1.65E-78
206106_at	MAPK12	0	-1.50	2.25E-36
206108_s_at	SFRS6	0	-1.27	<1.00E-120
206110_at	HIST1H3J	0	-1.25	<1.00E-120
206115_at	EGR3	0	-1.41	<1.00E-120
206129_s_at	ARSB	0	-1.13	1.33E-02
206133_at	XAF1	0	1.12	8.79E-03
206141_at	MOCS3	0	-1.22	7.52E-13
206150_at	CD27	0	1.33	1.34E-33
206158_s_at	CNBP	0	-1.20	1.02E-34
206181_at	SLAMF1	0	1.30	2.31E-51
206182_at	ZNF134	0		N.S.
206183_s_at	HERC3	0		N.S.
206184_at	CRKL	0		N.S.
206188_at	ZNF623	0	1.21	4.03E-06
206194_at	HOXC4	0	-1.21	5.55E-18
206200_s_at	ANXA11	0	-1.07	1.06E-06
206219_s_at	VAV1	0	-1.12	2.32E-10
206233_at	B4GALT6	0		N.S.
206235_at	LIG4	0		N.S.
206238_s_at	YAF2	0	-1.15	4.33E-25
206240_s_at	ZNF136	0		N.S.
206241_at	KPNA5	0	-1.31	7.52E-13
206247_at	MICB	0	-1.31	4.75E-31
206255_at	BLK	0	1.46	2.13E-41
206257_at	CCDC9	0		N.S.
206261_at	ZNF239	0	-1.45	1.98E-09
206263_at	FMO4	1		N.S.
206278_at	PTAFR	0	1.46	7.52E-13
206279_at	PRKY	0		N.S.

Supplemental Table 1

206302_s_at	NUDT4	0	-1.18	5.43E-31
206308_at	TRDMT1	0		N.S.
206313_at	HLA-DOA	0		N.S.
206314_at	ZNF167	0	1.43	1.02E-41
206316_s_at	KNTC1	0	-1.09	5.37E-05
206335_at	GALNS	0		N.S.
206337_at	CCR7	0		N.S.
206352_s_at	PEX10	0	-1.10	3.42E-03
206357_at	OPA3	0	-1.12	4.25E-04
206364_at	KIF14	0	-1.11	9.39E-10
206398_s_at	CD19	3	1.09	1.50E-12
206412_at	FER	0		N.S.
206437_at	S1PR4	0		N.S.
206440_at	LIN7A	0		N.S.
206445_s_at	PRMT1	1	-1.16	3.67E-30
206451_at	TBCCD1	0		N.S.
206478_at	KIAA0125	0	-1.40	4.23E-07
206491_s_at	NAPA	0	1.05	1.45E-02
206492_at	FHIT	0	1.24	9.84E-20
206495_s_at	HINFP	0		N.S.
206497_at	C7orf44	0	1.34	<1.00E-120
206500_s_at	C14orf106	0		N.S.
206507_at	ZSCAN12	0		N.S.
206508_at	CD70	0	1.08	2.37E-02
206512_at	ZRSR1	0	2.06	7.18E-24
206513_at	AIM2	0		N.S.
206515_at	CYP4F3	0	1.82	<1.00E-120
206521_s_at	GTF2A1	0		N.S.
206530_at	RAB30	0	-1.22	7.52E-13
206533_at	CHRNA5	0		N.S.
206536_s_at	XIAP	11	1.26	5.27E-12
206540_at	GLB1L	0		N.S.
206542_s_at	SMARCA2	0		N.S.
206550_s_at	NUP155	0	-1.60	3.55E-73
206553_at	OAS2	1		N.S.
206566_at	SLC7A1	0	2.07	4.20E-37
206583_at	ZNF673	0	1.50	4.14E-58
206584_at	LY96	0	1.68	8.50E-58
206586_at	CNR2	0	1.44	<1.00E-120
206587_at	CCT6B	0	1.37	9.07E-33
206589_at	GFI1	1		N.S.
206592_s_at	AP3D1	0	1.42	2.97E-70
206593_s_at	MED22	0		N.S.
206613_s_at	TAF1A	0	-1.10	2.70E-05
206618_at	IL18R1	0	1.19	2.77E-09
206620_at	GRAP	0		N.S.
206621_s_at	EIF4H	0	1.05	8.79E-03
206632_s_at	APOBEC3B	0	1.13	2.12E-10
206636_at	RASA2	0	1.18	4.62E-06
206641_at	TNFRSF17	0	1.14	4.63E-18
206648_at	ZNF571	0	1.46	2.54E-18
206649_s_at	TFE3	0	1.31	2.30E-42
206650_at	IQCC	0		N.S.
206652_at	ZMYM5	0	1.30	<1.00E-120

206654_s_at	POLR3G	0	-1.71	1.42E-24
206656_s_at	C20orf3	0	1.13	2.35E-19
206659_at	FLJ14082	0		N.S.
206661_at	DBF4B	0		N.S.
206667_s_at	SCAMP1	0	-1.22	2.23E-06
206683_at	ZNF165	0	2.38	9.18E-61
206686_at	PDK1	1		N.S.
206687_s_at	PTPN6	0	-1.19	2.47E-31
206688_s_at	CPSF4	0		N.S.
206693_at	IL7	0	1.72	3.16E-61
206703_at	CHRNB1	0	1.84	3.92E-48
206704_at	CLCN5	0		N.S.
206708_at	FOXN2	0	-1.19	2.94E-02
206724_at	CBX4	0	2.43	5.07E-70
206729_at	TNFRSF8	0	1.12	7.52E-13
206734_at	JRKL	0	-1.15	9.14E-08
206752_s_at	DFFB	0	-1.17	6.02E-12
206756_at	CHST7	0	1.35	1.05E-52
206759_at	FCER2	0	1.12	1.29E-18
206766_at	ITGA10	0	1.13	2.01E-02
206788_s_at	CBFB	0	-1.20	<1.00E-120
206789_s_at	POU2F1	0	-1.17	1.62E-24
206790_s_at	NDUFB1	0	-1.12	2.57E-21
206809_s_at	HNRNPA3	0	-1.23	<1.00E-120
206818_s_at	CNNM2	0	1.20	1.55E-06
206825_at	OXTR	0	-1.49	4.93E-39
206828_at	TXK	0	1.84	8.58E-32
206833_s_at	ACYP2	0	1.16	<1.00E-120
206838_at	TBX19	0	1.32	<1.00E-120
206845_s_at	RNF40	0		N.S.
206848_at	FAM36A	0	-1.13	1.14E-02
206853_s_at	MAP3K7	0	-1.22	<1.00E-120
206855_s_at	HYAL2	0	-1.54	2.74E-40
206858_s_at	HOXC6	0		N.S.
206860_s_at	MIOS	0		N.S.
206861_s_at	CGGBP1	0		N.S.
206875_s_at	SLK	1	-1.28	3.82E-31
206907_at	TNFSF9	0		N.S.
206918_s_at	CPNE1	0	1.18	4.01E-26
206925_at	ST8SIA4	0	1.36	8.60E-18
206928_at	ZNF124	0	-1.40	2.06E-25
206931_at	ZNF141	0		N.S.
206949_s_at	RUSC1	0	-1.33	3.82E-44
206956_at	BGLAP	0	-1.22	1.87E-09
206958_s_at	UPF3A	0	1.10	<1.00E-120
206967_at	CCNT1	0		N.S.
206975_at	LTA	0	-1.11	6.96E-05
206976_s_at	HSPH1	1	-2.57	3.30E-97
206983_at	CCR6	0	-1.14	5.34E-04
206992_s_at	ATP5S	0	-1.13	7.52E-13
207000_s_at	PPP3CC	0	1.09	3.22E-09
207002_s_at	PLAGL1	0	1.31	<1.00E-120
207006_s_at	CCDC106	0		N.S.
207030_s_at	CSRP2	0	-1.66	<1.00E-120

Supplemental Table 1

207038_at	SLC16A6	0	1.32	3.15E-26
207040_s_at	ST13	0	-1.10	3.83E-25
207046_at	HIST2H4A	0	1.28	5.27E-12
207061_at	ERN1	32	1.25	7.99E-06
207064_s_at	AOC2	0		N.S.
207071_s_at	ACO1	0	-1.25	3.10E-30
207076_s_at	ASS1	0	6.23	2.61E-71
207079_s_at	MED6	0	-1.10	3.32E-10
207088_s_at	SLC25A11	0	-1.48	8.73E-55
207103_at	KCND2	0	1.18	9.11E-09
207113_s_at	TNF	66	-1.16	1.06E-07
207121_s_at	MAPK6	0	1.22	1.40E-46
207124_s_at	GNB5	0	1.09	2.54E-03
207125_at	ZNF225	0		N.S.
207127_s_at	HNRNPH3	0	-1.19	6.00E-23
207128_s_at	ZNF223	0		N.S.
207143_at	CDK6	1		N.S.
207153_s_at	GLMN	0	-1.44	1.67E-58
207156_at	HIST1H2AG	0		N.S.
207157_s_at	GNG5	0	-1.07	1.34E-08
207160_at	IL12A	0	-1.15	5.13E-06
207163_s_at	AKT1	10	-1.51	7.15E-54
207170_s_at	LETMD1	0	1.45	2.70E-67
207176_s_at	CD80	1		N.S.
207178_s_at	FRK	0		N.S.
207181_s_at	CASP7	31	1.08	8.02E-04
207183_at	GPR19	0	1.61	1.59E-44
207186_s_at	BPTF	0	1.10	1.08E-09
207196_s_at	TNIP1	0		N.S.
207198_s_at	LIMS1	0	-1.09	3.26E-04
207219_at	ZNF643	0	2.46	7.32E-60
207232_s_at	DZIP3	0	1.20	9.09E-10
207234_at	RFX3	0	1.26	2.45E-05
207243_s_at	CALM2	0	-1.08	1.81E-11
207245_at	UGT2B17	0		N.S.
207283_at	RPL23AP32	0		N.S.
207286_at	CEP135	0		N.S.
207291_at	PRRG4	0	-1.62	3.18E-37
207305_s_at	KIAA1012	0	1.07	3.83E-08
207315_at	CD226	0	1.09	6.23E-07
207332_s_at	TFRC	0	-1.66	8.98E-65
207338_s_at	ZNF200	0	-1.16	9.61E-21
207339_s_at	LTB	0	-1.27	1.26E-25
207347_at	ERCC6	0	1.18	2.69E-19
207350_s_at	VAMP4	0	1.20	<1.00E-120
207375_s_at	IL15RA	0		N.S.
207386_at	CYP7B1	0		N.S.
207394_at	ZNF137	0	1.23	<1.00E-120
207396_s_at	ALG3	1	1.18	2.06E-25
207399_at	BFSP2	0	-1.21	3.06E-10
207405_s_at	RAD17	0	1.26	2.67E-29
207417_s_at	ZNF177	0	1.38	1.95E-18
207426_s_at	TNFSF4	0		N.S.
207431_s_at	DEGS1	0	1.21	<1.00E-120

Supplemental Table 1

207433_at	IL10	7	-1.29	4.82E-07
207435_s_at	SRRM2	0	-1.18	1.20E-27
207438_s_at	SNUPN	0	-1.17	2.82E-34
207440_at	SLC35A2	0		N.S.
207446_at	TLR6	0	1.36	7.52E-13
207469_s_at	PIR	0	-1.18	2.10E-23
207480_s_at	MEIS2	0		N.S.
207492_at	NGLY1	0		N.S.
207508_at	ATP5G3	0	-1.23	1.84E-33
207513_s_at	ZNF189	0	1.21	3.75E-41
207515_s_at	POLR1C	0	-1.50	6.44E-50
207518_at	DGKE	0		N.S.
207520_at	TROVE2	0	-1.17	1.93E-02
207525_s_at	GIPC1	0	-1.13	4.94E-04
207528_s_at	SLC7A11	2	5.98	1.64E-76
207536_s_at	TNFRSF9	0		N.S.
207540_s_at	SYK	0	-1.34	6.55E-37
207541_s_at	EXOSC10	0	-1.18	3.99E-38
207543_s_at	P4HA1	1		N.S.
207545_s_at	NUMB	0	1.28	<1.00E-120
207551_s_at	MSL3	0	1.32	3.18E-38
207556_s_at	DGKZ	0		N.S.
207559_s_at	ZMYM3	0	-1.19	5.18E-19
207563_s_at	OGT	0	1.72	4.89E-61
207583_at	ABCD2	0		N.S.
207585_s_at	RPL36AL	0	1.07	8.92E-09
207604_s_at	SLC4A7	0	-1.71	3.23E-18
207606_s_at	ARHGAP12	0	1.30	2.31E-22
207610_s_at	EMR2	0	-1.34	1.78E-07
207614_s_at	CUL1	0	-1.32	6.72E-45
207618_s_at	BCS1L	0	-1.28	3.69E-51
207621_s_at	PEMT	0	1.28	2.41E-25
207622_s_at	ABCF2	0	-1.41	1.02E-42
207624_s_at	RPGR	0	1.15	1.21E-07
207627_s_at	TFCP2	0		N.S.
207628_s_at	WBSCR22	0	1.08	3.76E-12
207641_at	TNFRSF13B	0		N.S.
207643_s_at	TNFRSF1A	0	1.86	8.60E-53
207655_s_at	BLNK	0	-1.05	1.01E-08
207677_s_at	NCF4	0	1.09	2.34E-07
207687_at	INHBC	0		N.S.
207707_s_at	SEC13	0	1.20	8.18E-18
207711_at	C20orf117	0	-1.23	9.43E-03
207713_s_at	RBCK1	0	1.12	1.08E-10
207714_s_at	SERPINH1	0	1.46	2.32E-18
207722_s_at	BTBD2	0		N.S.
207727_s_at	MUTYH	0	1.19	6.06E-21
207734_at	LAX1	0		N.S.
207735_at	RNF125	0		N.S.
207740_s_at	NUP62	0	-2.30	1.51E-67
207746_at	POLQ	0	-1.47	2.57E-22
207753_at	ZNF304	0		N.S.
207761_s_at	METTL7A	0		N.S.
207765_s_at	KIAA1539	0	1.42	1.54E-22

Supplemental Table 1

207777_s_at	SP140	0	1.25	1.62E-31
207785_s_at	RBPJ	0		N.S.
207786_at	CYP2R1	0	2.12	5.95E-36
207805_s_at	PSMD9	0		N.S.
207809_s_at	ATP6AP1	0	1.21	7.06E-24
207812_s_at	GORASP2	0	1.43	7.84E-56
207813_s_at	FDXR	0	1.08	7.20E-05
207819_s_at	ABCB4	0	2.99	1.49E-73
207824_s_at	MAZ	1	-1.34	1.72E-17
207826_s_at	ID3	0	-1.70	1.05E-44
207830_s_at	PPP1R8	0	-1.22	1.62E-41
207839_s_at	C9orf127	0		N.S.
207842_s_at	CASC3	0		N.S.
207845_s_at	ANAPC10	0		N.S.
207855_s_at	CLCC1	0	1.20	1.13E-11
207856_s_at	LOC150776	0	-1.24	2.61E-24
207861_at	CCL22	0	-1.35	2.21E-47
207871_s_at	ST7	0	1.14	5.02E-22
207877_s_at	NVL	0	1.13	3.15E-07
207891_s_at	HAUS7	0	-1.62	4.07E-56
207900_at	CCL17	0		N.S.
207901_at	IL12B	0		N.S.
207904_s_at	LNPEP	0	1.09	2.58E-02
207907_at	TNFSF14	0	-1.23	1.65E-05
207917_at	NUDT13	0	-1.12	1.96E-02
207922_s_at	MAEA	0		N.S.
207945_s_at	CSNK1D	0	-1.20	3.63E-41
207957_s_at	PRKCB	0		N.S.
207966_s_at	GLG1	0	1.17	<1.00E-120
207980_s_at	CITED2	0	1.18	1.67E-08
207996_s_at	C18orf1	0	2.02	3.51E-51
208003_s_at	NFAT5	0	1.31	1.41E-19
208018_s_at	HCK	0	1.27	1.47E-07
208021_s_at	RFC1	0	1.56	2.43E-58
208024_s_at	DGCR6	0		N.S.
208047_s_at	NAB1	0	-1.31	6.14E-07
208050_s_at	CASP2	0	-1.27	7.67E-19
208051_s_at	PAIP1	0		N.S.
208055_s_at	HERC4	0		N.S.
208056_s_at	CBFA2T3	0	-1.21	1.07E-18
208066_s_at	GTF2B	0		N.S.
208070_s_at	REV3L	0	1.24	5.40E-23
208072_s_at	DGKD	0	1.62	2.29E-58
208074_s_at	AP2S1	0		N.S.
208076_at	HIST1H4D	0		N.S.
208081_s_at	ZNF442	0		N.S.
208089_s_at	TDRD3	0		N.S.
208091_s_at	ECOP	0	-1.08	9.95E-18
208093_s_at	NDEL1	0	1.17	<1.00E-120
208094_s_at	CCDC130	0	1.20	<1.00E-120
208095_s_at	SRP72	0	1.42	2.19E-47
208101_s_at	URM1	0		N.S.
208104_s_at	TSC22D4	0		N.S.
208107_s_at	LOC81691	0	-1.33	8.62E-37

Supplemental Table 1

208117_s_at	LAS1L	0	-1.51	3.20E-56
208119_s_at	ZNF93	0	1.07	4.19E-02
208152_s_at	DDX21	0	-1.36	9.79E-65
208154_at	LOC51336	0		N.S.
208165_s_at	PRSS16	0	3.32	5.48E-50
208184_s_at	TRAPPC10	0		N.S.
208190_s_at	LSR	0	-1.18	2.98E-09
208194_s_at	STAM2	0		N.S.
208195_at	TTN	0	1.23	<1.00E-120
208249_s_at	TGDS	0	1.27	9.64E-31
208270_s_at	RNPEP	0		N.S.
208290_s_at	EIF5	0	1.22	1.09E-21
208302_at	HMHB1	0	-1.15	3.20E-06
208309_s_at	MALT1	0	-1.32	9.00E-55
208319_s_at	RBM3	0		N.S.
208328_s_at	MEF2A	1	1.15	1.28E-11
208336_s_at	GPSN2	0	-1.11	2.06E-19
208361_s_at	POLR3D	0		N.S.
208368_s_at	BRCA2	1	-1.19	5.19E-06
208382_s_at	DMC1	0	-1.35	3.44E-25
208398_s_at	TBPL1	0		N.S.
208405_s_at	CD164	0	1.16	5.64E-29
208424_s_at	CIAPIN1	0	-1.24	1.23E-39
208433_s_at	LRP8	0	-1.45	1.11E-43
208436_s_at	IRF7	0	1.34	1.22E-29
208438_s_at	FGR	0	1.44	3.25E-41
208442_s_at	ATM	5	1.39	6.21E-28
208447_s_at	PRPS1	0	-1.32	6.30E-38
208453_s_at	XPNPEP1	0	-1.16	<1.00E-120
208460_at	GJC1	0	-1.76	4.29E-11
208498_s_at	AMY1A	0	1.76	9.02E-18
208499_s_at	DNAJC3	11	2.71	1.27E-63
208503_s_at	GATAD1	0	1.15	1.70E-06
208506_at	HIST1H3F	0	-1.51	2.90E-27
208511_at	PTTG3	0		N.S.
208515_at	HIST1H2BM	0		N.S.
208524_at	GPR15	0	-1.25	6.70E-09
208534_s_at	RASA4	0	-1.28	8.86E-18
208553_at	HIST1H1E	0	-1.44	6.92E-08
208576_s_at	HIST1H3B	0		N.S.
208588_at	FKSG2	0		N.S.
208612_at	PDIA3	6	1.84	3.80E-73
208613_s_at	FLNB	0		N.S.
208616_s_at	PTP4A2	0	1.04	4.45E-03
208619_at	DDB1	0	-1.07	9.65E-06
208620_at	PCBP1	0	-1.34	1.13E-51
208623_s_at	EZR	0	-1.17	6.32E-44
208624_s_at	EIF4G1	0	-1.37	2.41E-31
208626_s_at	VAT1	0	-1.24	6.77E-12
208627_s_at	YBX1	0		N.S.
208629_s_at	HADHA	0		N.S.
208632_at	RNF10	0	1.14	5.85E-20
208633_s_at	MACF1	0		N.S.
208636_at	ACTN1	0	-1.37	1.84E-22

Supplemental Table 1

208641_s_at	RAC1	2		N.S.
208642_s_at	XRCC5	0	-1.41	1.35E-75
208644_at	PARP1	1	-1.08	5.04E-07
208645_s_at	RPS14	0		N.S.
208647_at	FDFT1	0	-2.02	1.16E-88
208649_s_at	VCP	12		N.S.
208652_at	PPP2CA	4	-1.11	1.00E-33
208655_at	CCNI	0	1.32	2.93E-43
208659_at	CLIC1	0	1.06	4.66E-07
208660_at	CS	22	-1.14	1.76E-36
208664_s_at	TTC3	0		N.S.
208670_s_at	EID1	0	-1.40	3.08E-35
208671_at	SERINC1	0	1.82	5.42E-70
208675_s_at	DDOST	0	1.45	2.19E-59
208676_s_at	PA2G4	0	-1.52	3.34E-55
208677_s_at	BSG	0	-1.12	6.77E-08
208678_at	ATP6V1E1	0	-1.13	<1.00E-120
208679_s_at	ARPC2	0		N.S.
208680_at	PRDX1	1	-1.29	3.79E-42
208684_at	COPA	0	1.34	7.19E-45
208686_s_at	BRD2	0		N.S.
208689_s_at	RPN2	0	1.47	3.56E-68
208690_s_at	PDLIM1	0		N.S.
208692_at	RPS3	0		N.S.
208693_s_at	GARS	0	2.32	1.63E-101
208695_s_at	RPL39	0		N.S.
208696_at	CCT5	0	-1.50	2.87E-68
208697_s_at	EIF3E	2	1.04	8.85E-03
208698_s_at	NONO	0	-1.13	1.88E-11
208700_s_at	TKT	0	-1.17	1.25E-26
208709_s_at	NRD1	0	1.07	3.44E-07
208713_at	HNRNPUL1	0	1.09	4.67E-02
208714_at	NDUFV1	0	1.06	1.10E-09
208715_at	TMCO1	0	1.35	1.25E-47
208717_at	OXA1L	0		N.S.
208719_s_at	DDX17	0		N.S.
208720_s_at	RBM39	0	1.08	<1.00E-120
208721_s_at	ANAPC5	0	-1.21	3.36E-10
208723_at	USP11	0	1.20	1.42E-29
208724_s_at	RAB1A	0	1.18	2.37E-32
208726_s_at	EIF2S2	0	1.40	8.24E-69
208736_at	ARPC3	0	-1.11	2.03E-20
208737_at	ATP6V1G1	0		N.S.
208741_at	SAP18	0		N.S.
208745_at	ATP5L	0		N.S.
208753_s_at	NAP1L1	0		N.S.
208756_at	EIF3I	0	-1.10	1.45E-20
208757_at	TMED9	0	1.67	1.22E-43
208758_at	ATIC	0	-1.44	1.13E-62
208759_at	NCSTN	0	1.31	<1.00E-120
208760_at	UBE2I	0	1.13	6.40E-05
208762_at	SUMO1	0		N.S.
208763_s_at	TSC22D3	0	2.24	9.74E-85
208765_s_at	HNRNPR	0	-1.41	2.15E-42

Supplemental Table 1

208771_s_at	LTA4H	0	-1.05	4.77E-06
208777_s_at	PSMD11	0	-1.34	1.19E-48
208784_s_at	KLHDC3	0	-1.08	2.63E-06
208785_s_at	MAP1LC3B	2	1.86	2.29E-73
208787_at	MRPL3	0	-1.07	8.27E-12
208796_s_at	CCNG1	1	1.17	2.17E-46
208799_at	PSMB5	1	-1.34	1.37E-57
208807_s_at	CHD3	0	-1.07	1.05E-04
208808_s_at	HMGB2	3	1.08	3.16E-07
208813_at	GOT1	0	2.22	6.46E-91
208819_at	RAB8A	0	-1.33	3.28E-69
208820_at	PTK2	1		N.S.
208821_at	SNRPB	0	-1.18	3.92E-34
208822_s_at	DAP3	0	-1.09	<1.00E-120
208827_at	PSMB6	1	-1.07	1.71E-09
208828_at	POLE3	0	-1.75	3.10E-81
208829_at	TAPBP	0		N.S.
208830_s_at	SUPT6H	0		N.S.
208833_s_at	ATXN10	0	-1.10	3.12E-07
208836_at	ATP1B3	0	-1.10	2.23E-23
208837_at	TMED3	0	-1.19	5.43E-18
208839_s_at	CAND1	0	-1.31	1.21E-50
208841_s_at	G3BP2	0	-1.20	2.42E-22
208845_at	VDAC3	0	-1.22	5.90E-50
208847_s_at	ADH5	0		N.S.
208853_s_at	CANX	1	1.70	2.37E-67
208854_s_at	STK24	0	1.26	5.97E-47
208857_s_at	PCMT1	0		N.S.
208858_s_at	FAM62A	0	1.22	3.26E-21
208861_s_at	ATRX	0	1.09	4.59E-11
208862_s_at	CTNND1	0		N.S.
208872_s_at	REEP5	0	1.20	1.07E-39
208876_s_at	PAK2	0	-1.20	2.54E-18
208880_s_at	PRPF6	0	-1.10	1.84E-10
208883_at	UBR5	0		N.S.
208885_at	LCP1	0	-1.16	7.07E-28
208886_at	H1F0	0	3.09	2.27E-55
208887_at	EIF3G	0		N.S.
208894_at	HLA-DRA	0	-1.06	3.72E-04
208897_s_at	DDX18	0	-1.31	4.37E-59
208900_s_at	TOP1	0	-1.47	2.93E-32
208905_at	CYCS	0	-1.10	<1.00E-120
208906_at	BSCL2	3	1.31	2.58E-41
208909_at	UQCRFS1	0	-1.19	1.19E-43
208910_s_at	C1QBP	0	-1.21	8.03E-51
208911_s_at	PDHB	0	-1.09	1.17E-10
208912_s_at	CNP	1	-1.27	1.11E-31
208916_at	SLC1A5	0	3.78	1.51E-104
208920_at	SRI	0	-1.19	<1.00E-120
208922_s_at	NXF1	0		N.S.
208923_at	CYFIP1	0	-1.43	2.01E-66
208924_at	RNF11	0	-1.10	1.61E-05
208925_at	CLDND1	0	1.17	1.68E-17
208926_at	NEU1	0	1.11	5.49E-09

Supplemental Table 1

208927_at	SPOP	0	1.19	7.05E-34
208928_at	POR	1	-1.25	3.90E-26
208932_at	PPP4C	0	-1.08	1.10E-03
208938_at	PRCC	0	-1.16	5.68E-21
208941_s_at	SEPHS1	0	-1.16	3.38E-28
208942_s_at	SEC62	0		N.S.
208944_at	TGFBR2	0		N.S.
208946_s_at	BECN1	4	1.12	6.71E-23
208949_s_at	LGALS3	1	-1.08	9.36E-10
208954_s_at	LARP5	0		N.S.
208959_s_at	ERP44	2	1.42	8.16E-55
208964_s_at	FADS1	0	-2.22	1.87E-82
208965_s_at	IFI16	0	-1.11	5.62E-05
208969_at	NDUFA9	1		N.S.
208971_at	UROD	0	-1.10	6.90E-09
208972_s_at	ATP5G1	0	-1.62	1.32E-70
208973_at	ERI3	0	-1.20	6.40E-28
208979_at	NCOA6	0		N.S.
208980_s_at	UBC	0	1.06	4.21E-04
208985_s_at	EIF3J	0	-1.10	1.50E-12
208986_at	TCF12	0	1.33	7.95E-49
208987_s_at	KDM2A	0		N.S.
208991_at	STAT3	11	1.07	2.28E-06
208995_s_at	PPIG	0	-1.42	7.93E-48
208996_s_at	POLR2C	0	-1.16	1.44E-17
208998_at	UCP2	1	-1.30	7.80E-53
208999_at	SEPT8	0	-1.29	4.27E-40
209001_s_at	ANAPC13	0	1.13	4.37E-27
209002_s_at	CALCOCO1	0	1.39	2.12E-35
209004_s_at	FBXL5	0		N.S.
209007_s_at	C1orf63	0	1.25	3.51E-23
209014_at	MAGED1	0	-1.44	1.06E-49
209015_s_at	DNAJB6	0	-1.08	6.58E-06
209017_s_at	LONP1	0	1.76	5.12E-72
209018_s_at	PINK1	2	1.35	1.23E-18
209020_at	C20orf111	0	1.09	1.48E-08
209023_s_at	STAG2	0		N.S.
209028_s_at	ABI1	1	1.10	2.59E-10
209029_at	COPS7A	0	-1.16	3.69E-27
209030_s_at	CADM1	1		N.S.
209034_at	PNRC1	0	1.51	1.18E-29
209037_s_at	EHD1	0		N.S.
209040_s_at	PSMB8	0	-1.11	5.27E-12
209042_s_at	UBE2G2	0	-1.50	8.15E-57
209043_at	PAPSS1	0	1.09	2.26E-12
209046_s_at	GABARAPL2	0		N.S.
209049_s_at	ZMYND8	0	-1.05	2.47E-03
209050_s_at	RALGDS	0		N.S.
209052_s_at	WHSC1	0	-1.14	3.49E-07
209055_s_at	CDC5L	0	-1.41	1.86E-31
209058_at	EDF1	0	1.16	2.48E-29
209068_at	HNRPDL	0	-1.22	1.72E-23
209075_s_at	ISCU	0	1.15	8.12E-26
209076_s_at	WDR45L	0		N.S.

Supplemental Table 1

209077_at	TXN2	1		N.S.
209083_at	CORO1A	0		N.S.
209084_s_at	RAB28	0	1.28	2.23E-23
209088_s_at	UBN1	0	-1.20	<1.00E-120
209090_s_at	SH3GLB1	0	1.25	1.96E-36
209092_s_at	GLOD4	0	-1.15	9.95E-23
209095_at	DLD	1	1.12	5.31E-30
209096_at	UBE2V2	0	-1.39	6.11E-39
209100_at	IFRD2	0	-1.60	8.83E-55
209102_s_at	HBP1	0	2.17	7.95E-67
209103_s_at	UFD1L	2	-1.15	4.51E-29
209104_s_at	NHP2	0	-1.15	3.91E-34
209106_at	NCOA1	0	1.19	1.53E-19
209110_s_at	RGL2	0		N.S.
209111_at	RNF5	1	1.07	7.57E-06
209112_at	CDKN1B	4	1.58	3.95E-59
209113_s_at	HMG20B	0		N.S.
209115_at	UBA3	0	-1.12	1.98E-20
209117_at	WBP2	0		N.S.
209118_s_at	TUBA1A	0	-1.22	4.06E-18
209122_at	ADFP	1	1.20	2.31E-29
209123_at	QDPR	0	-1.33	5.56E-52
209124_at	MYD88	1	-1.36	1.74E-52
209127_s_at	SART3	0	-1.09	6.85E-05
209130_at	SNAP23	0	1.05	1.87E-05
209132_s_at	COMMD4	0	-1.19	1.37E-21
209135_at	ASPH	0		N.S.
209136_s_at	USP10	0	-1.24	1.67E-21
209139_s_at	PRKRA	0	1.06	1.66E-03
209142_s_at	UBE2G1	0	-1.15	1.62E-21
209143_s_at	CLNS1A	0	-1.09	2.34E-26
209146_at	SC4MOL	0	-1.98	7.57E-72
209148_at	RXRB	0	1.21	<1.00E-120
209150_s_at	TM9SF1	0	1.57	1.41E-59
209154_at	TAX1BP3	0	-1.08	4.20E-10
209155_s_at	NT5C2	0	1.55	1.75E-55
209157_at	DNAJA2	0	-1.36	5.67E-36
209158_s_at	CYTH2	0	1.39	3.10E-45
209162_s_at	PRPF4	0	-1.53	1.86E-55
209163_at	CYB561	0	2.72	1.26E-60
209165_at	AATF	1	-1.24	4.96E-32
209166_s_at	MAN2B1	0		N.S.
209171_at	ITPA	0	-1.17	2.57E-31
209174_s_at	QRICH1	0		N.S.
209175_at	SEC23IP	0	-1.16	1.53E-24
209177_at	NDUFAF3	0		N.S.
209178_at	DHX38	0	-1.09	5.20E-05
209179_s_at	MBOAT7	0	-1.19	9.03E-12
209187_at	DR1	0		N.S.
209190_s_at	DIAPH1	0	-1.32	1.04E-57
209191_at	TUBB6	0	-1.29	<1.00E-120
209193_at	PIM1	0		N.S.
209194_at	CETN2	0	1.13	<1.00E-120
209196_at	WDR46	0	-1.52	1.40E-28

Supplemental Table 1

209198_s_at	SYT11	0	1.11	<1.00E-120
209199_s_at	MEF2C	1	-1.14	1.27E-29
209205_s_at	LMO4	0	1.84	5.10E-64
209206_at	SEC22B	2	1.41	8.19E-51
209208_at	MPDU1	0		N.S.
209210_s_at	FERMT2	0	2.77	2.86E-79
209213_at	CBR1	0	-1.70	1.39E-59
209215_at	MFSD10	0		N.S.
209217_s_at	WDR45	0	2.03	5.27E-68
209219_at	RDBP	1	-1.07	5.53E-08
209221_s_at	OSBPL2	0	1.28	1.03E-22
209224_s_at	NDUFA2	0	-1.20	4.10E-33
209229_s_at	SAPS1	0	-1.10	<1.00E-120
209231_s_at	DCTN5	0	-1.31	3.71E-44
209233_at	EMG1	0	-1.54	2.21E-63
209234_at	KIF1B	0	1.08	2.53E-02
209239_at	NFKB1	2	-1.10	6.11E-09
209249_s_at	GHITM	0	1.32	1.40E-63
209252_at	HARS2	0	1.07	1.34E-07
209254_at	KLHDC10	0		N.S.
209258_s_at	SMC3	0	-1.17	2.93E-09
209265_s_at	METTL3	0	-1.21	2.90E-43
209267_s_at	SLC39A8	0		N.S.
209268_at	VPS45	0	1.20	1.68E-25
209275_s_at	CLN3	0	1.22	1.31E-10
209276_s_at	GLRX	0	1.12	8.27E-12
209279_s_at	NSDHL	0	-1.53	3.53E-46
209282_at	PRKD2	0		N.S.
209285_s_at	C3orf63	0	-1.11	4.69E-07
209287_s_at	CDC42EP3	0	-1.12	2.28E-06
209295_at	TNFRSF10B	15	1.71	3.73E-78
209300_s_at	NECAP1	0	1.13	4.51E-12
209301_at	CA2	85	-2.07	4.88E-28
209302_at	POLR2H	0	-1.60	6.43E-82
209303_at	NDUFS4	0	1.12	4.60E-30
209306_s_at	SWAP70	0	-1.17	1.35E-18
209308_s_at	BNIP2	0	1.37	6.22E-43
209310_s_at	CASP4	19	1.09	1.18E-02
209311_at	BCL2L2	0	1.18	2.14E-22
209313_at	GPN1	0	-1.12	4.01E-22
209316_s_at	HBS1L	0		N.S.
209321_s_at	ADCY3	0	-1.23	3.43E-34
209323_at	PRKRIR	0	-1.06	1.11E-03
209324_s_at	RGS16	2	2.02	1.39E-71
209331_s_at	MAX	22	1.06	2.44E-02
209333_at	ULK1	0	1.34	2.27E-19
209336_at	PWP2	0	-1.69	5.08E-55
209339_at	SIAH2	0	1.50	1.67E-40
209340_at	UAP1	0	1.15	4.29E-11
209342_s_at	IKBKB	4		N.S.
209344_at	TPM4	0		N.S.
209349_at	RAD50	0	1.11	2.26E-12
209352_s_at	SIN3B	0		N.S.
209354_at	TNFRSF14	0	1.20	<1.00E-120

Supplemental Table 1

209358_at	TAF11	0	-1.26	2.82E-33
209361_s_at	PCBP4	0	-1.11	3.73E-02
209363_s_at	MED21	0	-1.23	3.34E-28
209367_at	STXBP2	0		N.S.
209374_s_at	IGHM	0	-1.17	3.36E-02
209375_at	XPC	0		N.S.
209377_s_at	HMGN3	0	-1.23	1.02E-37
209378_s_at	KIAA1128	0	1.59	1.69E-47
209383_at	DDIT3	190	9.77	1.13E-100
209385_s_at	PROSC	0	-1.10	5.55E-08
209390_at	TSC1	3	1.38	5.45E-49
209391_at	DPM2	0		N.S.
209392_at	ENPP2	0	1.87	3.64E-58
209393_s_at	EIF4E2	0	-1.62	7.41E-68
209394_at	ASMTL	0	-1.31	2.15E-30
209398_at	HIST1H1C	0	1.11	3.88E-10
209403_at	LOC653498	0	1.28	<1.00E-120
209405_s_at	FAM3A	0		N.S.
209406_at	BAG2	0	-1.67	1.46E-76
209407_s_at	DEAF1	0	-1.49	4.27E-33
209408_at	KIF2C	0		N.S.
209411_s_at	GGA3	0	-1.07	7.09E-03
209413_at	B4GALT2	0		N.S.
209417_s_at	IFI35	0	1.35	5.47E-36
209421_at	MSH2	0	-1.20	8.33E-30
209427_at	SMTN	0	-1.19	9.42E-08
209428_s_at	ZFPL1	0	1.27	2.71E-26
209430_at	BTAF1	0		N.S.
209432_s_at	CREB3	8	1.55	3.45E-48
209433_s_at	PPAT	0	-1.49	1.81E-54
209435_s_at	ARHGEF2	0	-1.09	3.58E-03
209438_at	PHKA2	0		N.S.
209449_at	LSM2	0	-1.19	2.27E-29
209450_at	OSGEP	1		N.S.
209451_at	TANK	0		N.S.
209452_s_at	VTI1B	0	-1.05	2.20E-02
209453_at	SLC9A1	0	1.31	9.03E-12
209456_s_at	FBXW11	0	1.18	5.24E-04
209457_at	DUSP5	1	1.20	4.03E-20
209459_s_at	ABAT	0	1.49	2.19E-27
209463_s_at	TAF12	0	-1.11	<1.00E-120
209464_at	AURKB	0	-1.14	<1.00E-120
209467_s_at	MKNK1	0	-1.12	7.52E-13
209468_at	LRP5	0	-1.44	5.16E-25
209472_at	CCBL2	0		N.S.
209474_s_at	ENTPD1	0		N.S.
209475_at	USP15	0		N.S.
209476_at	TMX1	0		N.S.
209477_at	EMD	0	1.06	1.38E-02
209478_at	STRA13	0	-1.65	1.31E-64
209479_at	CCDC28A	0	1.72	1.54E-63
209481_at	SNRK	0		N.S.
209482_at	POP7	0	-1.17	1.21E-29
209484_s_at	NSL1	0		N.S.

Supplemental Table 1

209486_at	UTP3	0	-1.43	7.30E-59
209497_s_at	RBM4B	0	1.14	<1.00E-120
209501_at	CDR2	0	1.16	8.41E-09
209503_s_at	PSMC5	0	-1.11	2.28E-29
209507_at	RPA3	0		N.S.
209509_s_at	DPAGT1	0	1.64	2.56E-68
209510_at	RNF139	0	1.17	1.91E-36
209511_at	POLR2F	0	-1.53	2.77E-45
209512_at	HSDL2	0	1.22	1.59E-22
209514_s_at	RAB27A	0	1.20	1.57E-23
209516_at	SMYD5	0	-1.22	6.47E-11
209517_s_at	ASH2L	0		N.S.
209520_s_at	NCBP1	0	-1.52	1.98E-60
209523_at	TAF2	0		N.S.
209524_at	HDGFRP3	0		N.S.
209531_at	GSTZ1	0	1.25	3.66E-32
209532_at	PLAA	0	-1.21	4.92E-07
209536_s_at	EHD4	0	1.13	6.92E-11
209537_at	EXTL2	0	1.14	4.04E-22
209538_at	ZNF32	0		N.S.
209539_at	ARHGEF6	0	1.51	2.42E-48
209545_s_at	RIPK2	0	1.24	1.00E-31
209546_s_at	APOL1	0	-1.40	2.42E-18
209553_at	VPS8	0	1.18	1.87E-25
209556_at	NCDN	0	-1.36	2.14E-28
209565_at	RNF113A	0	1.28	4.43E-45
209566_at	INSIG2	1	1.28	8.59E-27
209567_at	RRS1	1	-1.62	9.99E-44
209568_s_at	RGL1	0		N.S.
209571_at	CIR	0	1.13	4.23E-09
209575_at	IL10RB	0	1.24	3.05E-22
209577_at	PCYT2	0	-1.48	4.25E-24
209581_at	PLA2G16	0	-1.09	6.92E-05
209583_s_at	CD200	0		N.S.
209585_s_at	MINPP1	0	-1.13	<1.00E-120
209593_s_at	TOR1B	1		N.S.
209595_at	GTF2F2	0		N.S.
209604_s_at	GATA3	0	1.16	8.50E-08
209605_at	TST	0	-1.35	4.38E-41
209606_at	CYTIP	0	1.16	2.73E-19
209608_s_at	ACAT2	0	-1.99	7.02E-82
209609_s_at	MRPL9	0	-1.08	<1.00E-120
209615_s_at	PAK1	0	-1.23	3.73E-24
209619_at	CD74	1		N.S.
209620_s_at	ABCB7	0		N.S.
209622_at	STK16	0	1.14	5.27E-12
209624_s_at	MCCC2	0	-1.18	9.03E-12
209625_at	PIGH	0	1.22	2.65E-20
209627_s_at	OSBPL3	0		N.S.
209628_at	NXT2	0		N.S.
209630_s_at	FBXW2	0		N.S.
209636_at	NFKB2	0		N.S.
209645_s_at	ALDH1B1	0	-1.98	2.92E-32
209647_s_at	SOCS5	0		N.S.

Supplemental Table 1

209653_at	KPNA4	0	-1.17	2.88E-18
209654_at	KIAA0947	0		N.S.
209657_s_at	HSF2	0	-1.33	1.26E-46
209659_s_at	CDC16	0	1.04	3.08E-02
209662_at	CETN3	0		N.S.
209665_at	CYB561D2	0	1.08	1.32E-03
209666_s_at	CHUK	0	-1.17	<1.00E-120
209667_at	CES2	0	1.09	1.08E-05
209669_s_at	SERBP1	0	-1.30	8.23E-58
209670_at	TRAC	1	-1.09	7.29E-05
209674_at	CRY1	0		N.S.
209678_s_at	PRKCI	0	-1.21	<1.00E-120
209680_s_at	KIFC1	0	-1.43	6.47E-33
209681_at	SLC19A2	0	-1.13	1.75E-06
209682_at	CBLB	0	1.60	8.48E-65
209694_at	PTS	0	-1.19	6.94E-29
209695_at	PTP4A3	0	-1.56	4.88E-38
209704_at	MTF2	0	-1.41	5.57E-58
209707_at	PIGK	0	1.17	7.60E-21
209709_s_at	HMMR	0	-1.08	1.00E-08
209711_at	SLC35D1	0	1.20	1.26E-19
209714_s_at	CDKN3	0		N.S.
209724_s_at	ZFP161	0		N.S.
209725_at	UTP20	0	-1.74	1.17E-70
209726_at	CA11	0		N.S.
209727_at	GM2A	0	-1.36	<1.00E-120
209731_at	NTHL1	0	-1.46	1.68E-41
209732_at	CLEC2B	0		N.S.
209739_s_at	PNPLA4	0	1.26	4.07E-26
209748_at	SPAST	0	1.08	7.52E-03
209753_s_at	TMPO	0	-1.46	4.29E-54
209759_s_at	DCI	0	1.42	9.31E-49
209760_at	KIAA0922	0	1.26	4.25E-58
209761_s_at	SP110	0	-1.09	1.06E-04
209764_at	MGAT3	0		N.S.
209770_at	BTN3A1	0	1.64	3.74E-21
209778_at	TRIP11	0	1.12	<1.00E-120
209780_at	PHTF2	0	-1.20	8.45E-28
209786_at	HMGN4	0		N.S.
209788_s_at	ERAP1	0	1.45	5.28E-63
209790_s_at	CASP6	2	-1.22	7.32E-09
209795_at	CD69	0		N.S.
209796_s_at	CNPY2	0	1.18	3.21E-42
209799_at	PRKAA1	0		N.S.
209805_at	PMS2	0	1.09	1.17E-02
209806_at	HIST1H2BK	0	1.25	2.60E-42
209814_at	ZNF330	0	-1.17	1.06E-39
209820_s_at	TBL3	0	-1.99	7.50E-68
209822_s_at	VLDLR	1	6.00	8.54E-74
209825_s_at	UCK2	0	-1.63	1.92E-89
209827_s_at	IL16	0	1.50	1.02E-48
209829_at	FAM65B	0		N.S.
209832_s_at	CDT1	1	-1.31	2.77E-26
209833_at	CRADD	1		N.S.

Supplemental Table 1

209837_at	AP4M1	0	-1.32	2.02E-18
209838_at	COPS2	0	-1.29	1.01E-28
209845_at	MKRN1	0		N.S.
209849_s_at	RAD51C	0		N.S.
209853_s_at	PSME3	0	-1.64	2.24E-67
209861_s_at	METAP2	0	-1.17	4.17E-36
209863_s_at	TP63	0	1.40	3.54E-22
209864_at	FRAT2	0	1.37	1.42E-31
209865_at	SLC35A3	0		N.S.
209879_at	SELPLG	0		N.S.
209882_at	RIT1	0	-1.12	1.31E-08
209883_at	GLT25D2	0	1.98	3.04E-31
209891_at	SPC25	0	-1.39	4.41E-51
209893_s_at	FUT4	0	-1.23	2.10E-04
209894_at	LEPR	2	1.33	5.88E-20
209899_s_at	PUF60	0	-1.33	1.10E-52
209903_s_at	ATR	0	-1.40	8.88E-68
209910_at	SLC25A16	0		N.S.
209912_s_at	KIAA0415	0	1.50	1.07E-46
209916_at	DHTKD1	0	1.17	1.24E-23
209925_at	OCLN	0		N.S.
209926_at	LOC729991	0	-1.52	7.06E-27
209927_s_at	C1orf77	0	-1.26	2.53E-24
209928_s_at	MSC	1	1.62	1.45E-52
209929_s_at	IKBKG	0		N.S.
209932_s_at	DUT	0	-1.27	5.19E-53
209933_s_at	CD300A	0	-1.22	6.07E-18
209940_at	PARP3	0		N.S.
209941_at	RIPK1	1		N.S.
209943_at	FBXL4	0		N.S.
209945_s_at	GSK3B	1	-1.20	1.01E-25
209949_at	NCF2	0	1.24	3.23E-29
209953_s_at	CDC37	0	-1.25	2.08E-25
209962_at	EPOR	0		N.S.
209965_s_at	RAD51L3	0	-1.16	<1.00E-120
209967_s_at	CREM	2		N.S.
209969_s_at	STAT1	5		N.S.
209972_s_at	JTV1	0		N.S.
209973_at	NFKBIL1	0	1.82	<1.00E-120
209974_s_at	BUB3	0		N.S.
209989_at	ZNF268	0		N.S.
209994_s_at	ABCB1	2	2.81	1.17E-69
210006_at	ABHD14A	0		N.S.
210010_s_at	SLC25A1	0	-1.69	5.22E-71
210022_at	PCGF1	0	1.41	7.52E-12
210024_s_at	UBE2E3	0	-1.19	1.94E-32
210027_s_at	APEX1	0	-1.14	3.08E-31
210028_s_at	ORC3L	0	1.10	5.05E-10
210041_s_at	PGM3	0	1.49	1.69E-33
210045_at	IDH2	0		N.S.
210048_at	NAPG	0	-1.09	1.58E-08
210052_s_at	TPX2	0	-1.11	1.09E-21
210053_at	TAF5	0	-1.30	1.33E-60
210054_at	HAUS3	0	-1.15	6.38E-20

Supplemental Table 1

210057_at	SMG1	0		N.S.
210058_at	MAPK13	0		N.S.
210070_s_at	CHKB	0	1.24	1.11E-19
210075_at	MARCH2	0		N.S.
210092_at	MAGOH	0		N.S.
210097_s_at	NOL7	0	1.08	6.93E-06
210105_s_at	FYN	0	1.72	1.29E-58
210109_at	C7orf54	0	1.28	1.26E-02
210114_at	INVS	0		N.S.
210115_at	RPL39L	0		N.S.
210117_at	SPAG1	0	1.06	4.30E-03
210125_s_at	BANF1	0	-1.33	5.95E-45
210128_s_at	LTB4R	0	1.15	3.46E-02
210130_s_at	TM7SF2	0	-1.40	1.21E-26
210137_s_at	DCTD	0	-1.08	8.73E-09
210138_at	RGS20	0	1.47	4.07E-28
210144_at	TBC1D22A	0	1.18	2.33E-05
210145_at	PLA2G4A	2	-1.50	2.82E-51
210149_s_at	ATP5H	0	-1.07	4.91E-09
210151_s_at	DYRK3	0	1.21	6.77E-12
210152_at	LILRB4	0	1.18	3.10E-03
210154_at	ME2	0	-1.35	5.67E-31
210160_at	PAFAH1B2	0	-1.21	2.26E-12
210169_at	SEC14L5	0		N.S.
210172_at	SF1	0		N.S.
210176_at	TLR1	0	1.18	9.17E-18
210188_at	GABPA	0		N.S.
210200_at	WWP2	0		N.S.
210202_s_at	BIN1	0	1.26	8.77E-07
210205_at	B3GALT4	0	1.19	1.43E-06
210206_s_at	DDX11	0	-1.47	1.17E-20
210213_s_at	EIF6	0	-1.09	1.79E-10
210214_s_at	BMPR2	0		N.S.
210219_at	SP100	0		N.S.
210220_at	FZD2	0	2.16	2.58E-30
210235_s_at	PPFIA1	0		N.S.
210241_s_at	TP53TG1	0	1.15	4.38E-10
210243_s_at	B4GALT3	0	1.67	1.19E-56
210247_at	SYN2	0		N.S.
210253_at	HTATIP2	0	-1.11	2.76E-03
210258_at	RGS13	0		N.S.
210260_s_at	TNFAIP8	0	1.15	7.41E-24
210269_s_at	SFRS17A	0	1.93	9.18E-65
210275_s_at	ZFAND5	0	1.09	7.52E-13
210276_s_at	TRIOBP	0	1.08	8.06E-03
210278_s_at	AP4S1	0	1.19	<1.00E-120
210279_at	GPR18	0	1.65	1.17E-31
210280_at	MPZ	1	1.17	8.83E-05
210281_s_at	ZMYM2	0	-1.14	2.49E-02
210284_s_at	MAP3K7IP2	0		N.S.
210296_s_at	PXMP3	0		N.S.
210312_s_at	IFT20	0	1.38	2.62E-55
210338_s_at	HSPA8	2	-2.42	1.10E-63
210346_s_at	CLK4	0	1.14	8.52E-28

Supplemental Table 1

210349_at	CAMK4	0		N.S.
210371_s_at	RBBP4	0		N.S.
210377_at	ACSM3	0	1.34	1.25E-22
210378_s_at	SSNA1	0		N.S.
210379_s_at	TLK1	0		N.S.
210386_s_at	MTX1	0	-1.15	1.59E-26
210396_s_at	BOLA2	0	-1.07	5.30E-03
210406_s_at	RAB6A	0	1.37	1.98E-58
210415_s_at	ODF2	0		N.S.
210416_s_at	CHEK2	0	-1.19	3.27E-21
210417_s_at	PI4KB	0		N.S.
210418_s_at	IDH3B	0	-1.10	<1.00E-120
210428_s_at	HGS	0	-1.12	8.68E-20
210448_s_at	P2RX5	0		N.S.
210450_at	LOC90925	0	1.78	1.23E-19
210474_s_at	CDC2L1	0		N.S.
210479_s_at	RORA	0	1.22	1.99E-07
210480_s_at	MYO6	0		N.S.
210502_s_at	PPIE	0	1.17	3.05E-28
210512_s_at	VEGFA	7	2.83	1.24E-70
210528_at	MR1	1	1.23	6.88E-09
210531_at	NR2C1	0	-1.29	7.66E-10
210538_s_at	BIRC3	1	-1.38	3.54E-38
210543_s_at	PRKDC	0	-1.24	7.77E-35
210556_at	NFATC3	0		N.S.
210559_s_at	CDC2	3	-1.62	4.20E-79
210561_s_at	WSB1	0		N.S.
210573_s_at	POLR3C	0	1.49	9.46E-38
210574_s_at	NUDC	0	-1.56	5.03E-79
210587_at	INHBE	1	19.91	2.47E-101
210589_s_at	GBAP	0		N.S.
210596_at	LOC100129513	0	2.15	1.04E-45
210609_s_at	TP53I3	0	1.08	1.11E-05
210620_s_at	GTF3C2	0	-1.22	3.83E-09
210625_s_at	AKAP1	0	-1.79	1.59E-54
210627_s_at	MOGS	0	1.28	2.44E-20
210631_at	NF1	0		N.S.
210635_s_at	KLHL20	0		N.S.
210638_s_at	FBXO9	0		N.S.
210639_s_at	ATG5	7		N.S.
210643_at	TNFSF11	0	-1.24	7.73E-20
210649_s_at	ARID1A	0	-1.09	1.23E-04
210656_at	EED	0	-1.34	1.50E-11
210685_s_at	UBE4B	1	1.08	1.22E-02
210701_at	CFDP1	0	-1.27	8.68E-04
210705_s_at	TRIM5	0	1.38	3.42E-28
210715_s_at	SPINT2	0	1.14	1.24E-03
210718_s_at	ARL17P1	0		N.S.
210720_s_at	NECAB3	0	-1.12	6.12E-04
210732_s_at	LGALS8	0	-1.09	3.00E-04
210733_at	TRAM1	1	2.13	3.43E-38
210740_s_at	ITPK1	0	-1.20	5.58E-24
210752_s_at	MLX	0	-1.12	2.52E-03
210758_at	PSIP1	0	1.26	4.09E-19

210759_s_at	PSMA1	0	-1.10	1.18E-18
210771_at	PPARA	0		N.S.
210774_s_at	NCOA4	0		N.S.
210785_s_at	C1orf38	0		N.S.
210786_s_at	FLI1	0	1.19	1.50E-12
210793_s_at	NUP98	0	-1.25	7.16E-32
210797_s_at	OASL	0	1.19	7.08E-05
210807_s_at	SLC16A7	0	1.17	2.15E-03
210811_s_at	DDX49	0	1.10	4.20E-06
210813_s_at	XRCC4	0		N.S.
210817_s_at	CALCOCO2	0	1.62	2.57E-63
210822_at	RPL13P5	0		N.S.
210830_s_at	PON2	3	-1.32	7.04E-24
210868_s_at	ELOVL6	0	-1.15	3.24E-05
210878_s_at	KDM3B	0	-1.07	1.84E-06
210889_s_at	FCGR2B	0	-1.08	1.94E-06
210892_s_at	GTF2I	2	-1.35	2.23E-19
210895_s_at	CD86	1	1.14	<1.00E-120
210907_s_at	PDCD10	0		N.S.
210908_s_at	PFDN5	0		N.S.
210910_s_at	POMZP3	0		N.S.
210926_at	ACTBL3	0	-1.15	1.15E-03
210942_s_at	ST3GAL6	0		N.S.
210943_s_at	LYST	0		N.S.
210944_s_at	CAPN3	0	1.28	<1.00E-120
210946_at	PPAP2A	0	1.59	3.47E-56
210947_s_at	MSH3	0		N.S.
210949_s_at	EIF3C	0	1.04	5.35E-03
210959_s_at	SRD5A1	0	1.51	8.66E-52
210968_s_at	RTN4	0	-1.09	7.37E-11
210971_s_at	ARNTL	1		N.S.
210976_s_at	PFKM	0	-1.55	4.42E-71
210978_s_at	TAGLN2	0	-1.20	2.56E-21
210983_s_at	MCM7	0	-1.54	2.69E-79
210996_s_at	YWHAE	0	-1.19	1.73E-17
211009_s_at	ZNF271	0	1.17	2.14E-22
211010_s_at	NCR3	0		N.S.
211012_s_at	LOC161527	0	-1.23	2.76E-19
211015_s_at	HSPA4	1	-1.70	8.10E-81
211026_s_at	MGLL	0	-1.37	1.63E-46
211028_s_at	KHK	0	-1.26	2.67E-07
211031_s_at	CLIP2	0	1.12	1.22E-05
211033_s_at	PEX7	0		N.S.
211034_s_at	C12orf51	0	1.13	7.52E-13
211038_s_at	CROCCL1	0		N.S.
211043_s_at	CLTB	0	-1.15	7.57E-18
211048_s_at	PDIA4	3	2.74	7.65E-74
211052_s_at	TBCD	0	-1.19	2.02E-23
211059_s_at	GOLGA2	0		N.S.
211064_at	ZNF493	0	-1.19	4.04E-03
211071_s_at	MLLT11	0	-1.56	3.02E-69
211074_at	FOLR1	0		N.S.
211075_s_at	CD47	0	1.13	8.83E-22
211089_s_at	NEK3	1	1.21	2.68E-06

Supplemental Table 1

211113_s_at	ABCG1	1		N.S.
211136_s_at	CLPTM1	0	1.10	5.77E-09
211141_s_at	CNOT3	0	1.60	1.94E-39
211168_s_at	UPF1	0	-1.22	1.19E-32
211178_s_at	PSTPIP1	0	1.25	2.66E-10
211212_s_at	ORC5L	0	-1.25	<1.00E-120
211250_s_at	SH3BP2	0	1.09	3.79E-03
211275_s_at	GYG1	0	-1.09	1.65E-11
211284_s_at	GRN	0		N.S.
211285_s_at	UBE3A	1	-1.07	1.51E-08
211297_s_at	CDK7	0	1.17	1.73E-31
211310_at	EZH1	0		N.S.
211317_s_at	CFLAR	0	1.25	4.51E-18
211330_s_at	HFE	5		N.S.
211339_s_at	ITK	0		N.S.
211352_s_at	NCOA3	0		N.S.
211358_s_at	CIZ1	0	1.22	9.05E-32
211364_at	MTAP	0		N.S.
211368_s_at	CASP1	5	1.09	4.61E-05
211373_s_at	PSEN2	5		N.S.
211391_s_at	PATZ1	0	-1.25	5.00E-06
211404_s_at	APLP2	0		N.S.
211406_at	IER3IP1	0	1.31	2.16E-20
211450_s_at	MSH6	0	-1.94	5.39E-60
211475_s_at	BAG1	0	-1.47	9.01E-67
211501_s_at	EIF3B	0	-1.58	7.87E-52
211502_s_at	PFTK1	0		N.S.
211505_s_at	STAU1	0		N.S.
211512_s_at	OGFR	0	-1.19	5.37E-07
211538_s_at	HSPA2	1	-2.34	5.44E-39
211543_s_at	GRK6	0	1.12	5.27E-10
211558_s_at	DHPS	0		N.S.
211563_s_at	C19orf2	0	-1.11	4.42E-07
211569_s_at	HADH	0	-1.19	5.41E-33
211572_s_at	SLC23A2	0		N.S.
211574_s_at	CD46	0		N.S.
211593_s_at	MAST2	0	-1.20	3.63E-23
211596_s_at	LRIG1	0		N.S.
211600_at	PTPRO	0		N.S.
211612_s_at	IL13RA1	0		N.S.
211623_s_at	FBL	0	-1.10	4.34E-22
211662_s_at	VDAC2	0	-1.09	9.42E-10
211665_s_at	SOS2	0	1.45	2.70E-19
211671_s_at	NR3C1	0	1.39	1.36E-50
211672_s_at	ARPC4	0	-1.36	1.23E-25
211676_s_at	IFNGR1	0		N.S.
211678_s_at	RNF114	0	1.23	1.16E-54
211684_s_at	DYNC1I2	0	-1.06	<1.00E-120
211685_s_at	NCALD	0	-1.14	5.48E-03
211686_s_at	MAK16	0	-1.44	9.14E-68
211692_s_at	BBC3	7	1.35	3.13E-21
211702_s_at	USP32	0		N.S.
211704_s_at	SPIN2A	0	-1.13	6.02E-03
211707_s_at	IQCB1	0	1.13	<1.00E-120

Supplemental Table 1

211708_s_at	SCD	0	-2.07	9.89E-51
211715_s_at	BDH1	0	-1.67	5.39E-61
211717_at	ANKRD40	0		N.S.
211721_s_at	ZNF551	0	-1.35	1.84E-23
211725_s_at	BID	18	-1.58	1.72E-81
211727_s_at	COX11	0	-1.09	<1.00E-120
211742_s_at	EVI2B	0	1.35	2.78E-46
211749_s_at	VAMP3	0		N.S.
211752_s_at	NDUFS7	0	1.07	1.78E-05
211753_s_at	RLN1	0	-1.43	1.10E-25
211754_s_at	SLC25A17	0	-1.38	1.06E-49
211755_s_at	ATP5F1	0	-1.06	9.88E-06
211763_s_at	UBE2B	0	1.23	2.74E-30
211764_s_at	UBE2D1	0	-1.20	4.21E-20
211767_at	GINS4	0	-1.44	2.39E-32
211773_s_at	ZKSCAN3	0		N.S.
211774_s_at	MMACHC	0	-1.24	<1.00E-120
211783_s_at	MTA1	0		N.S.
211784_s_at	SFRS1	0	-1.20	1.16E-46
211787_s_at	EIF4A1	0	-1.12	2.12E-20
211792_s_at	CDKN2C	0		N.S.
211796_s_at	TRBC1	0		N.S.
211810_s_at	GALC	0		N.S.
211812_s_at	B3GALNT1	0		N.S.
211825_s_at	EWSR1	0	1.16	8.62E-04
211828_s_at	TNIK	0	-1.12	2.24E-07
211833_s_at	BAX	94		N.S.
211855_s_at	SLC25A14	0		N.S.
211913_s_at	MERTK	0		N.S.
211926_s_at	MYH9	0	-1.06	6.39E-04
211928_at	DYNC1H1	0		N.S.
211935_at	ARL6IP1	0	1.24	1.13E-48
211936_at	HSPA5	75	2.07	2.89E-49
211938_at	EIF4B	0		N.S.
211946_s_at	BAT2D1	0	-1.11	<1.00E-120
211950_at	UBR4	0	1.29	3.05E-44
211955_at	IPO5	0	-1.07	2.08E-06
211956_s_at	EIF1	1	1.25	9.86E-46
211960_s_at	RAB7A	0	-1.20	9.32E-19
211962_s_at	ZFP36L1	0	1.07	3.40E-02
211963_s_at	ARPC5	0	-1.24	4.04E-38
211967_at	TMEM123	0	1.18	<1.00E-120
211971_s_at	LRPPRC	0		N.S.
211975_at	ARFGAP2	0		N.S.
211985_s_at	CALM1	0		N.S.
211987_at	TOP2B	0	1.07	4.65E-05
211989_at	SMARCE1	0	-1.17	1.50E-30
211990_at	HLA-DPA1	0		N.S.
211994_at	WNK1	0	1.09	<1.00E-120
211998_at	H3F3B	0	1.18	7.52E-20
212005_at	C1orf144	0	-1.27	4.26E-34
212007_at	UBXN4	2	1.33	7.76E-32
212009_s_at	STIP1	0	-2.04	7.56E-49
212016_s_at	PTBP1	0	-1.43	1.01E-31

Supplemental Table 1

212017_at	FAM168B	0		N.S.
212019_at	RSL1D1	0		N.S.
212021_s_at	MKI67	1	-1.20	8.62E-27
212025_s_at	FLII	0		N.S.
212027_at	RBM25	0	-1.22	3.54E-11
212032_s_at	PTOV1	0	-1.37	7.61E-46
212036_s_at	PNN	0	-1.33	2.14E-47
212038_s_at	VDAC1	1	-1.16	1.87E-31
212040_at	TGOLN2	0	1.18	4.21E-22
212041_at	ATP6V0D1	0	1.11	2.24E-06
212044_s_at	RPL27A	0		N.S.
212047_s_at	RNF167	0	1.22	<1.00E-120
212048_s_at	YARS	0	1.62	4.28E-70
212050_at	WIPF2	0		N.S.
212052_s_at	TBC1D9B	0	-1.22	2.65E-30
212053_at	PDXDC1	0	-1.23	6.46E-22
212055_at	C18orf10	0	-1.14	2.19E-20
212057_at	KIAA0182	0	-1.24	9.36E-27
212058_at	SR140	0		N.S.
212059_s_at	TRPC4AP	0	1.35	3.84E-37
212066_s_at	USP34	0	1.06	1.16E-17
212069_s_at	BAT2L	0		N.S.
212072_s_at	CSNK2A1	0		N.S.
212078_s_at	MLL	0		N.S.
212082_s_at	MYL6	0		N.S.
212083_at	TEX261	0	1.11	3.01E-12
212087_s_at	ERAL1	0	-1.15	8.14E-28
212088_at	PMPCA	0	-1.58	1.32E-60
212090_at	GRINA	0	-1.16	<1.00E-120
212092_at	PEG10	0	-1.70	2.07E-40
212098_at	LOC151162	0	-1.48	2.19E-52
212099_at	RHOB	0	-1.30	1.11E-25
212100_s_at	POLDIP3	1	-1.08	3.60E-10
212101_at	KPNA6	0	-1.13	9.93E-27
212106_at	FAF2	0		N.S.
212110_at	SLC39A14	0	2.53	3.65E-88
212112_s_at	STX12	0		N.S.
212115_at	HN1L	0	-1.59	3.32E-54
212118_at	TRIM27	0	1.22	5.94E-26
212119_at	RHOQ	0	2.06	1.16E-74
212121_at	TCTN3	0		N.S.
212124_at	ZMIZ1	0	-1.27	5.21E-37
212126_at	CBX5	0	-1.20	1.62E-25
212127_at	RANGAP1	0	-1.20	9.25E-06
212129_at	NIPA2	0	1.14	2.22E-27
212131_at	LSM14A	0		N.S.
212140_at	PDS5A	0	1.05	1.93E-03
212141_at	MCM4	0	-2.60	3.65E-77
212144_at	UNC84B	0	1.22	9.46E-21
212145_at	MRPS27	0		N.S.
212146_at	PLEKHM2	0		N.S.
212150_at	EFR3A	0	1.08	3.63E-05
212153_at	POGZ	0	1.24	3.73E-26
212155_at	RNF187	0	2.15	7.29E-93

Supplemental Table 1

212156_at	VPS39	0	1.23	<1.00E-120
212160_at	XPOT	0	2.30	3.64E-104
212161_at	AP2A2	0	-1.18	8.98E-03
212162_at	KIDINS220	0	1.30	2.04E-24
212165_at	TMEM183A	0	-1.18	4.96E-42
212166_at	XPO7	0	-1.13	<1.00E-120
212169_at	FKBP9	0	1.33	<1.00E-120
212170_at	RBM12	0	-2.05	2.65E-73
212177_at	SFRS18	0	1.15	1.46E-02
212186_at	ACACA	0	-1.70	1.69E-70
212188_at	KCTD12	0	-2.45	4.53E-56
212189_s_at	COG4	0	1.15	2.57E-08
212190_at	SERPINE2	0	1.86	1.61E-19
212193_s_at	LARP1	0		N.S.
212194_s_at	TM9SF4	0	-1.12	4.59E-11
212199_at	MRFAP1L1	0	-1.06	1.64E-08
212200_at	ANKLE2	0	-1.13	3.76E-12
212202_s_at	TMEM87A	0	1.30	7.81E-54
212208_at	MED13L	0		N.S.
212211_at	ANKRD17	0	-1.27	7.86E-41
212217_at	PREPL	0	1.24	3.67E-34
212218_s_at	FASN	1	-3.16	1.78E-88
212219_at	PSME4	0		N.S.
212228_s_at	COQ9	0		N.S.
212231_at	FBXO21	0	-1.21	1.24E-32
212232_at	FNBP4	0		N.S.
212233_at	MAP1B	0	3.01	2.11E-46
212238_at	ASXL1	0	-1.11	2.26E-12
212242_at	TUBA4A	0	-2.10	4.79E-75
212244_at	GCOM1	0	1.43	1.85E-47
212245_at	MCFD2	2	1.96	2.27E-70
212247_at	NUP205	0	-1.25	3.06E-59
212249_at	PIK3R1	2	-1.16	4.05E-03
212251_at	MTDH	0	1.22	1.13E-36
212255_s_at	ATP2C1	0	1.22	5.92E-22
212260_at	GIGYF2	0	-1.11	7.00E-10
212263_at	QKI	0	-1.07	7.07E-03
212266_s_at	SFRS5	0	-1.14	4.89E-25
212268_at	SERPINB1	0		N.S.
212271_at	MAPK1	9	1.17	1.95E-31
212274_at	LPIN1	1		N.S.
212277_at	MTMR4	0	1.40	4.01E-39
212281_s_at	TMEM97	0	-1.84	2.30E-84
212287_at	SUZ12	0	-1.07	3.04E-10
212293_at	HIPK1	0		N.S.
212296_at	PSMD14	0	-1.06	2.51E-07
212297_at	ATP13A3	0	-1.31	1.47E-23
212300_at	TXLNA	0	1.25	8.06E-35
212302_at	RTF1	0	-1.14	4.74E-06
212308_at	CLASP2	0		N.S.
212310_at	MIA3	0	1.58	8.04E-46
212311_at	KIAA0746	0		N.S.
212312_at	BCL2L1	18	-1.16	<1.00E-120
212313_at	CHMP7	0		N.S.

Supplemental Table 1

212317_at	TNPO3	0		N.S.
212320_at	TUBB	0	-1.34	1.98E-65
212323_s_at	VPS13D	0		N.S.
212329_at	SCAP	0	1.07	4.99E-04
212330_at	TFDP1	0	-1.30	1.44E-53
212332_at	RBL2	0		N.S.
212333_at	FAM98A	0	-1.50	4.54E-78
212338_at	MYO1D	0	-1.17	4.24E-03
212340_at	YIPF6	0	1.21	1.31E-19
212345_s_at	CREB3L2	2	1.89	1.28E-81
212348_s_at	KDM1	0	-1.34	1.75E-50
212350_at	TBC1D1	1	-1.07	2.19E-06
212351_at	EIF2B5	0	-1.24	7.86E-38
212352_s_at	TMED10	0	1.57	2.78E-74
212355_at	KIAA0323	0	1.10	9.03E-11
212357_at	FAM168A	0		N.S.
212360_at	AMPD2	0	-1.10	<1.00E-120
212366_at	ZNF292	0		N.S.
212371_at	PPPDE1	0	1.23	1.10E-42
212372_at	MYH10	0	-1.35	6.21E-50
212374_at	FEM1B	0		N.S.
212376_s_at	EP400	0	-1.23	1.44E-20
212380_at	FTSJD2	0		N.S.
212381_at	USP24	0	-1.20	9.85E-09
212383_at	ATP6V0A1	0	1.42	8.63E-47
212398_at	RDX	0	-1.26	1.53E-18
212400_at	FAM102A	0	1.46	4.82E-43
212401_s_at	CDC2L2	0	-1.14	1.37E-21
212402_at	ZC3H13	0	-1.15	3.79E-20
212403_at	UBE3B	0	1.13	<1.00E-120
212405_s_at	METTL13	0	-1.36	1.19E-54
212406_s_at	PCMTD2	0	1.15	7.54E-21
212408_at	TOR1AIP1	0		N.S.
212410_at	EFHA1	0		N.S.
212411_at	IMP4	0	-1.37	<1.00E-120
212415_at	SEPT6	0		N.S.
212420_at	ELF1	0	1.29	5.27E-20
212422_at	PDCD11	0	-1.43	1.03E-70
212430_at	RBM38	0	-1.21	8.05E-27
212434_at	GRPEL1	0	-1.36	9.57E-46
212436_at	TRIM33	0		N.S.
212437_at	CENPB	0	-1.64	5.87E-60
212439_at	IP6K1	0		N.S.
212440_at	SNRNP27	0		N.S.
212441_at	KIAA0232	0		N.S.
212443_at	NBEAL2	0	-1.17	2.26E-11
212445_s_at	NEDD4L	0	-1.24	9.40E-11
212446_s_at	LASS6	1	1.75	1.11E-73
212447_at	KBTBD2	0	1.07	2.26E-02
212449_s_at	LYPLA1	0		N.S.
212451_at	SECISBP2L	0	1.16	7.68E-05
212453_at	KIAA1279	0	-1.04	8.43E-03
212456_at	KIAA0664	0	-1.79	4.60E-67
212458_at	SPRED2	0	1.33	7.44E-38

Supplemental Table 1

212462_at	MYST4	0	1.32	1.01E-58
212465_at	SETD3	0	1.16	3.54E-11
212467_at	DNAJC13	0		N.S.
212470_at	SPAG9	0		N.S.
212471_at	AVL9	0	-1.12	8.41E-03
212473_s_at	MICAL2	0	1.91	2.54E-25
212476_at	ACAP2	0	1.16	8.25E-19
212479_s_at	RMND5A	0	1.38	1.09E-50
212480_at	CYTSA	0		N.S.
212483_at	NIPBL	0	1.13	3.13E-18
212484_at	FAM89B	0	-1.24	5.96E-09
212487_at	GPATCH8	0	-1.13	4.47E-04
212491_s_at	DNAJC8	0	-1.13	4.45E-22
212499_s_at	FCF1	0		N.S.
212500_at	ADO	0		N.S.
212501_at	CEBPB	1	7.68	2.20E-122
212505_s_at	KIAA0892	0	1.17	<1.00E-120
212507_at	TMEM131	0	1.22	3.81E-41
212508_at	MOAP1	0		N.S.
212509_s_at	MXRA7	0	-1.17	3.31E-05
212510_at	GPD1L	0	-1.35	4.25E-21
212512_s_at	CARM1	0	-1.15	4.46E-23
212515_s_at	DDX3X	0	-1.28	1.94E-28
212516_at	ARAP1	0		N.S.
212517_at	ATRN	0	1.19	4.46E-09
212518_at	PIP5K1C	0	-1.07	3.40E-02
212520_s_at	SMARCA4	0	-1.50	7.65E-53
212523_s_at	KIAA0146	0		N.S.
212526_at	SPG20	0	-1.27	6.67E-04
212527_at	PPPDE2	0	1.46	4.22E-71
212528_at	D15Wsu75e	0	1.30	4.23E-32
212529_at	LSM12	0	-1.26	3.00E-29
212530_at	NEK7	0	1.65	2.01E-89
212536_at	ATP11B	0	1.31	1.52E-27
212538_at	DOCK9	0		N.S.
212539_at	CHD1L	0	-1.08	1.25E-06
212540_at	CDC34	0		N.S.
212541_at	FLAD1	0	-1.08	1.07E-02
212542_s_at	PHIP	0		N.S.
212543_at	AIM1	0	1.39	3.52E-51
212544_at	ZNHIT3	0	1.11	4.04E-19
212548_s_at	FRYL	0	-1.22	2.46E-27
212550_at	STAT5B	0	-1.13	1.76E-06
212553_at	RPRD2	0	1.10	1.25E-05
212556_at	SCRIB	0	-1.39	5.69E-56
212557_at	ZNF451	0	1.11	1.47E-08
212558_at	SPRY1	0	-1.23	4.01E-04
212560_at	SORL1	0		N.S.
212561_at	DENND5A	0	1.46	2.70E-39
212568_s_at	DLAT	0	-1.35	2.28E-54
212571_at	CHD8	0	-1.16	3.12E-25
212572_at	STK38L	0	-1.29	1.20E-39
212573_at	ENDOD1	0	1.11	7.66E-07
212576_at	MGRN1	0		N.S.

Supplemental Table 1

212579_at	SMCHD1	0	-1.20	<1.00E-120
212584_at	AQR	0	-1.18	3.79E-39
212585_at	OSBPL8	0		N.S.
212586_at	CAST	0	1.08	3.99E-19
212587_s_at	PTPRC	0		N.S.
212589_at	RRAS2	0	-1.09	1.67E-19
212592_at	IGJ	0	-1.53	<1.00E-120
212595_s_at	DAZAP2	0	-1.26	8.63E-30
212597_s_at	HMGXB4	0	-1.33	2.67E-45
212600_s_at	UQCRC2	0	-1.11	2.46E-31
212601_at	ZZEF1	0	1.06	1.02E-03
212603_at	MRPS31	0	-1.24	2.79E-29
212608_s_at	NUDT3	0	-1.15	1.09E-06
212610_at	PTPN11	0	-1.08	2.25E-21
212611_at	DTX4	0		N.S.
212612_at	RCOR1	0		N.S.
212613_at	BTN3A2	0	1.33	2.55E-26
212614_at	ARID5B	0	1.35	8.55E-33
212621_at	TMEM194A	0	1.19	2.36E-22
212623_at	TMEM41B	0	2.01	6.99E-87
212625_at	STX10	0	-1.06	3.45E-04
212627_s_at	EXOSC7	0	-1.41	1.19E-40
212630_at	EXOC3	0		N.S.
212634_at	KIAA0776	0	1.40	1.06E-39
212637_s_at	WWP1	0	-1.43	3.78E-27
212640_at	PTPLB	0	1.12	6.21E-22
212642_s_at	HIVEP2	0	1.16	6.09E-11
212643_at	MAPK1IP1L	0	-1.41	8.30E-69
212646_at	RFTN1	0	1.12	<1.00E-120
212647_at	RRAS	0	1.53	1.33E-21
212649_at	DHX29	0	-1.13	2.14E-04
212653_s_at	EHBP1	0		N.S.
212655_at	ZCCHC14	0	1.33	2.13E-06
212656_at	TSFM	0	-1.20	9.87E-27
212658_at	LHFPL2	0	-1.11	1.26E-07
212663_at	FKBP15	0	-1.18	7.46E-26
212664_at	TUBB4	0	-1.25	2.11E-05
212665_at	TIPARP	0	1.21	1.07E-26
212666_at	SMURF1	0	1.10	3.07E-03
212673_at	METAP1	0	-1.35	2.35E-45
212674_s_at	DHX30	0	-1.58	7.68E-58
212677_s_at	CEP68	0	-1.39	2.93E-20
212685_s_at	TBL2	0	1.30	2.14E-43
212688_at	PIK3CB	0	1.08	4.48E-07
212689_s_at	KDM3A	0	1.15	2.26E-12
212690_at	DDHD2	0		N.S.
212692_s_at	LRBA	0		N.S.
212693_at	MDN1	0	-1.41	6.05E-71
212694_s_at	PCCB	0	1.10	<1.00E-120
212696_s_at	RNF4	0	1.06	2.88E-07
212697_at	FAM134C	0	1.32	2.58E-43
212698_s_at	SEPT10	0		N.S.
212699_at	SCAMP5	1		N.S.
212704_at	ZCCHC11	0	1.41	2.60E-60

Supplemental Table 1

212707_s_at	LOC100133005	0	-1.20	<1.00E-120
212708_at	MSL1	0		N.S.
212712_at	CAMSAP1	0	-1.14	1.50E-12
212716_s_at	EIF3K	0		N.S.
212718_at	PAPOLA	0		N.S.
212721_at	SFRS12	0	-1.10	3.10E-22
212723_at	JMJD6	0	-1.26	3.50E-42
212726_at	PHF2	0	-1.11	2.16E-08
212729_at	DLG3	0	1.39	2.60E-22
212731_at	ANKRD46	0	-1.20	4.89E-32
212735_at	KIAA0226	0		N.S.
212738_at	ARHGAP19	0	1.08	3.56E-04
212739_s_at	NME4	0	-1.47	8.45E-61
212742_at	RNF115	0	1.26	5.39E-41
212745_s_at	BBS4	0	1.14	1.24E-06
212746_s_at	CEP170	0	-1.09	1.86E-03
212747_at	ANKS1A	0	1.15	<1.00E-120
212751_at	UBE2N	0	-1.49	1.95E-62
212752_at	CLASP1	0	1.16	2.04E-24
212753_at	PCGF3	0	-2.23	5.72E-71
212754_s_at	MON2	0	1.15	2.86E-36
212756_s_at	UBR2	0	1.11	9.03E-12
212758_s_at	ZEB1	0		N.S.
212763_at	CAMSAP1L1	0	-1.24	1.41E-17
212766_s_at	ISG20L2	0	-1.79	9.69E-80
212767_at	MTG1	0	1.21	2.26E-12
212770_at	TLE3	0	-1.11	1.37E-06
212773_s_at	TOMM20	0	1.10	3.23E-18
212774_at	ZNF238	0	1.05	8.44E-03
212779_at	KIAA1109	0	1.25	2.57E-26
212780_at	SOS1	1	1.24	2.46E-35
212784_at	CIC	0		N.S.
212785_s_at	LARP7	0		N.S.
212786_at	CLEC16A	0		N.S.
212787_at	YLPM1	0	-1.39	3.28E-58
212789_at	NCAPD3	0	-1.31	1.87E-45
212791_at	C1orf216	0	-1.52	1.52E-58
212794_s_at	KIAA1033	0	1.14	5.23E-07
212801_at	CIT	0	-1.12	8.33E-08
212802_s_at	GAPVD1	0	-1.19	6.36E-37
212810_s_at	SLC1A4	1	2.64	1.37E-106
212813_at	JAM3	0		N.S.
212815_at	ASCC3	0		N.S.
212816_s_at	CBS	4	6.31	2.19E-85
212817_at	DNAJB5	0	1.91	7.29E-52
212819_at	ASB1	0		N.S.
212820_at	DMXL2	0		N.S.
212822_at	HEG1	0	1.13	2.05E-07
212824_at	FUBP3	0		N.S.
212825_at	PAXIP1	0	-1.68	7.37E-73
212826_s_at	SLC25A6	0	1.05	5.75E-03
212828_at	SYNJ2	0	-1.45	1.25E-19
212830_at	MEGF9	0		N.S.
212832_s_at	CKAP5	0		N.S.

Supplemental Table 1

212833_at	SLC25A46	0		N.S.
212834_at	DDX52	0	1.14	<1.00E-120
212836_at	POLD3	0	-1.51	3.63E-72
212837_at	FAM175B	0	1.08	1.35E-03
212838_at	DNMBP	0		N.S.
212841_s_at	PPFIBP2	0	-1.12	1.79E-04
212846_at	RRP1B	0	-1.57	9.25E-55
212847_at	FUBP1	0	-1.15	4.56E-08
212851_at	DCUN1D4	0		N.S.
212856_at	GRAMD4	0	-1.16	5.81E-10
212858_at	PAQR4	0	-1.38	3.56E-30
212860_at	ZDHHC18	0		N.S.
212861_at	MFSD5	0	-1.10	1.43E-05
212862_at	CDS2	0	1.19	1.53E-29
212866_at	R3HCC1	0	1.18	2.23E-19
212867_at	NCOA2	0	1.29	8.64E-34
212871_at	MAPKAPK5	0	-1.10	2.59E-08
212873_at	HMHA1	0		N.S.
212875_s_at	C2CD2	0	-1.07	1.26E-02
212876_at	B4GALT4	0	1.26	<1.00E-120
212877_at	KLC1	0	-1.13	<1.00E-120
212880_at	WDR7	0	-1.09	4.66E-11
212881_at	PIAS4	0		N.S.
212885_at	MPHOSPH10	0	-1.40	5.02E-58
212886_at	CCDC69	0	1.18	4.31E-20
212887_at	SEC23A	1	1.12	4.34E-07
212890_at	SLC38A10	0	1.80	1.95E-58
212891_s_at	GADD45GIP1	0	-1.16	<1.00E-120
212892_at	ZNF282	0	-1.15	2.26E-09
212893_at	ZZZ3	0		N.S.
212894_at	SUPV3L1	0	1.18	<1.00E-120
212896_at	SKIV2L2	0	-1.20	5.17E-52
212898_at	KIAA0406	0	-1.45	5.74E-47
212901_s_at	CSTF2T	0	-1.28	9.59E-28
212902_at	SEC24A	0		N.S.
212904_at	LRRC47	0	-1.09	4.51E-12
212906_at	GRAMD1B	0	-1.10	1.25E-03
212907_at	SLC30A1	0	1.46	7.42E-38
212908_at	DNAJC16	0	1.27	4.69E-46
212910_at	THAP11	0	-1.21	7.54E-33
212912_at	RPS6KA2	0	4.86	2.55E-62
212913_at	C6orf26	0		N.S.
212914_at	CBX7	0	2.13	3.84E-66
212916_at	PHF8	0		N.S.
212918_at	RECQL	0		N.S.
212919_at	DCP2	0	-1.20	1.92E-31
212926_at	SMC5	0	-1.13	1.99E-06
212928_at	TSPYL4	0	-1.38	7.23E-50
212929_s_at	FAM21A	0		N.S.
212930_at	ATP2B1	0	1.26	<1.00E-120
212931_at	TCF20	0	-1.10	1.50E-12
212934_at	UBXN2B	0	1.09	3.75E-03
212936_at	FAM172A	0	1.24	9.25E-23
212943_at	KIAA0528	0	1.19	2.79E-37

Supplemental Table 1

212944_at	SLC5A3	0	-1.47	2.60E-51
212945_s_at	MGA	0	-1.14	1.50E-11
212947_at	SLC9A8	0	1.24	7.52E-13
212948_at	CAMTA2	0		N.S.
212949_at	NCAPH	0	-1.64	8.92E-66
212954_at	DYRK4	0	1.98	3.03E-67
212955_s_at	POLR2I	0	-1.09	6.05E-10
212956_at	TBC1D9	0	-1.65	7.12E-28
212959_s_at	GNPTAB	0	-1.18	3.75E-18
212963_at	TM2D1	0		N.S.
212964_at	HIC2	0	-1.31	8.76E-36
212968_at	RFNG	0	1.18	<1.00E-120
212973_at	RPIA	0	-1.54	1.14E-72
212974_at	DENND3	0	-1.20	2.93E-11
212977_at	CXCR7	0	1.96	1.34E-65
212978_at	LRRC8B	0	-1.31	2.84E-24
212979_s_at	FAM115A	0	-1.22	<1.00E-120
212983_at	HRAS	1	-1.47	1.53E-51
212984_at	ATF2	2	1.53	5.03E-45
212986_s_at	TLK2	0	1.09	5.18E-06
212989_at	SGMS1	0		N.S.
212990_at	SYNJ1	1	1.14	<1.00E-120
212994_at	THOC2	0	-1.15	<1.00E-120
213000_at	MORC3	0	1.21	2.80E-09
213002_at	MARCKS	0	1.18	6.77E-04
213008_at	FANCI	0	-1.14	1.33E-29
213009_s_at	TRIM37	0	-1.21	7.56E-38
213010_at	PRKCDBP	0	1.36	2.26E-12
213011_s_at	TPI1	0	-1.22	4.72E-42
213012_at	NEDD4	0		N.S.
213016_at	BBX	0	1.27	4.08E-38
213017_at	ABHD3	0		N.S.
213019_at	RANBP6	0	-1.28	1.24E-44
213025_at	THUMPD1	0	-1.15	6.69E-24
213026_at	ATG12	0	1.22	1.73E-43
213028_at	NFRKB	0	-1.21	2.15E-09
213031_s_at	WDR73	0	1.08	5.76E-05
213035_at	ANKRD28	0	1.30	1.66E-31
213038_at	RNF19B	0	1.44	1.78E-51
213039_at	ARHGEF18	0	-1.14	<1.00E-120
213041_s_at	ATP5D	0	-1.09	2.33E-11
213043_s_at	MED24	0	-1.17	4.21E-26
213044_at	ROCK1	0	1.15	9.35E-44
213045_at	MAST3	0	-1.38	3.45E-36
213046_at	PABPN1	0		N.S.
213048_s_at	SET	71		N.S.
213052_at	PRKAR2A	0	-1.10	<1.00E-120
213054_at	HAUS5	0		N.S.
213058_at	TTC28	0		N.S.
213060_s_at	CHI3L2	0		N.S.
213061_s_at	NTAN1	0		N.S.
213065_at	ZFC3H1	0		N.S.
213070_at	PIK3C2A	0	1.06	2.00E-03
213073_at	ZFYVE26	0	-1.07	3.85E-05

Supplemental Table 1

213076_at	ITPKC	0	1.39	6.31E-24
213077_at	YTHDC2	0	-1.12	1.50E-12
213079_at	TSR2	0	-1.11	1.13E-07
213081_at	ZBTB22	0		N.S.
213082_s_at	SLC35D2	0	1.35	1.18E-07
213086_s_at	CSNK1A1	0	1.54	2.90E-76
213088_s_at	DNAJC9	0	-1.37	5.77E-62
213089_at	LOC100272216	0		N.S.
213090_s_at	TAF4	0	-1.26	3.22E-30
213097_s_at	DNAJC2	0	-1.24	3.25E-30
213098_at	RQCD1	0	-1.17	<1.00E-120
213101_s_at	ACTR3	0		N.S.
213103_at	STARD13	0	1.67	2.16E-49
213105_s_at	C16orf42	0	-1.14	2.26E-11
213106_at	ATP8A1	0	1.21	5.15E-20
213111_at	PIKFYVE	0	1.12	4.90E-18
213113_s_at	SLC43A3	0	-1.16	5.05E-18
213115_at	ATG4A	0	1.11	<1.00E-120
213117_at	KLHL9	0	-1.22	1.89E-27
213119_at	SLC36A1	0		N.S.
213122_at	TSPYL5	0	-1.22	3.71E-03
213126_at	MED8	0	1.66	1.19E-51
213130_at	ZNF473	0	-1.21	2.90E-02
213132_s_at	MCAT	0	-1.53	4.07E-42
213133_s_at	GCSH	0	-1.19	1.07E-27
213137_s_at	PTPN2	0	1.13	<1.00E-120
213138_at	ARID5A	0		N.S.
213140_s_at	SS18L1	0	-1.15	<1.00E-120
213141_at	PSKH1	0		N.S.
213145_at	FBXL14	0	1.21	2.21E-41
213151_s_at	SEPT7	0		N.S.
213152_s_at	SFRS2B	0	1.13	4.44E-10
213153_at	SETD1B	0		N.S.
213154_s_at	BICD2	0	-1.28	9.97E-32
213160_at	DOCK2	0	1.14	2.09E-21
213161_at	C9orf97	0	1.21	7.88E-41
213168_at	SP3	1	1.05	4.68E-04
213170_at	GPX7	0		N.S.
213172_at	TTC9	0		N.S.
213173_at	PCNX	0		N.S.
213185_at	KIAA0556	0	1.07	2.87E-02
213188_s_at	MINA	2		N.S.
213190_at	COG7	0	-1.15	4.66E-11
213191_at	TICAM1	0	-1.24	2.33E-11
213192_at	THAP3	0	1.20	4.25E-06
213194_at	ROBO1	0	1.24	6.73E-24
213203_at	SNAPC5	0	-1.21	9.69E-36
213204_at	CUL9	0	1.16	<1.00E-120
213205_s_at	RAD54L2	0	-1.18	5.14E-04
213206_at	GOSR2	1	1.41	1.68E-27
213213_at	DIDO1	0		N.S.
213216_at	OTUD3	0		N.S.
213218_at	ZNF187	0	1.27	7.42E-26
213221_s_at	SIK2	0		N.S.

Supplemental Table 1

213224_s_at	NCRNA00081	0	1.47	6.02E-51
213225_at	PPM1B	0	1.19	3.44E-04
213226_at	CCNA2	0	-1.37	2.31E-58
213227_at	PGRMC2	0	1.18	2.77E-18
213237_at	C16orf88	0	-1.94	1.72E-72
213238_at	ATP10D	0	1.06	4.49E-02
213239_at	PIBF1	0		N.S.
213244_at	SCAMP4	0		N.S.
213246_at	C14orf109	0	-1.17	2.19E-26
213251_at	SMARCA5	0		N.S.
213252_at	SH3PXD2A	0		N.S.
213253_at	SMC2	0	-1.22	1.35E-11
213254_at	TNRC6B	0	1.16	1.28E-09
213256_at	MARCH3	0	1.32	3.84E-26
213259_s_at	SARM1	0		N.S.
213261_at	LBA1	0	1.45	2.30E-27
213262_at	SACS	1	-1.44	7.18E-55
213266_at	TUBGCP4	0		N.S.
213267_at	DOPEY1	0		N.S.
213269_at	ZNF248	0	1.21	<1.00E-120
213272_s_at	TMEM159	0	-1.14	1.20E-07
213274_s_at	CTSB	1		N.S.
213278_at	MTMR9	0		N.S.
213279_at	DHRS1	0		N.S.
213282_at	APOOL	0		N.S.
213283_s_at	SALL2	0	-1.34	5.27E-12
213287_s_at	KRT10	0	1.09	9.07E-03
213293_s_at	TRIM22	0	1.26	1.48E-41
213294_at	EIF2AK2	0	-1.23	1.17E-41
213297_at	RMND5B	0		N.S.
213298_at	NFIC	0	-1.35	1.50E-12
213300_at	ATG2A	0	1.20	<1.00E-120
213302_at	PFAS	0	-1.71	9.31E-78
213304_at	FAM179B	0	1.21	1.35E-32
213305_s_at	PPP2R5C	1		N.S.
213310_at	EIF2C2	0	1.13	2.52E-08
213312_at	C6orf162	0	-1.32	5.51E-29
213318_s_at	BAT3	1		N.S.
213320_at	PRMT3	0	-1.78	1.10E-69
213322_at	C6orf130	0	1.28	5.14E-51
213324_at	SRC	12	-1.16	7.52E-13
213326_at	VAMP1	0		N.S.
213327_s_at	USP12	0		N.S.
213328_at	NEK1	0	1.26	<1.00E-120
213333_at	MDH2	0	-1.26	3.45E-23
213340_s_at	KIAA0495	0	-1.14	3.84E-10
213341_at	FEM1C	0	1.08	5.61E-05
213346_at	C13orf27	0	-1.36	1.10E-58
213350_at	RPS11	0		N.S.
213351_s_at	TMCC1	0	1.14	1.24E-09
213353_at	ABCA5	0	1.46	2.26E-46
213357_at	GTF2H5	0	-1.44	8.83E-67
213361_at	TDRD7	0	-1.10	<1.00E-120
213365_at	ERI2	0	-1.15	1.76E-22

Supplemental Table 1

213370_s_at	SFMBT1	0	-1.52	1.18E-51
213372_at	PAQR3	0		N.S.
213373_s_at	CASP8	47	-1.21	7.09E-18
213376_at	ZBTB1	0	-1.21	4.50E-34
213379_at	COQ2	0	-1.08	1.84E-05
213383_at	SAPS2	0		N.S.
213387_at	ATAD2B	0		N.S.
213390_at	ZC3H4	0	-1.15	1.34E-21
213391_at	DPY19L4	0	1.27	4.36E-26
213392_at	IQCK	0	1.14	3.66E-04
213394_at	MAPKBP1	0	-1.25	3.94E-20
213398_s_at	SDR39U1	0		N.S.
213402_at	ZNF787	0		N.S.
213403_at	MFSD9	0	1.62	1.07E-21
213405_at	RAB22A	0	1.10	<1.00E-120
213408_s_at	PI4KA	0		N.S.
213409_s_at	RHEB	1		N.S.
213410_at	C10orf137	0		N.S.
213414_s_at	RPS19	0		N.S.
213415_at	CLIC2	0	1.24	2.28E-20
213420_at	DHX57	0		N.S.
213427_at	RPP40	0	-1.19	<1.00E-120
213430_at	RUFY3	1		N.S.
213433_at	ARL3	0		N.S.
213434_at	STX2	0	-1.09	5.92E-03
213436_at	CNR1	0	1.18	6.51E-09
213445_at	ZC3H3	0		N.S.
213446_s_at	IQGAP1	0		N.S.
213447_at	IPW	0	1.42	3.00E-33
213449_at	POP1	0	-1.68	9.29E-50
213452_at	ZNF184	0	1.23	5.80E-18
213454_at	APITD1	0		N.S.
213455_at	FAM114A1	0		N.S.
213457_at	MFHAS1	0	-1.10	1.93E-04
213461_at	NUDT21	0		N.S.
213469_at	PGAP1	0		N.S.
213471_at	NPHP4	0		N.S.
213474_at	KCTD7	0	1.13	1.73E-03
213475_s_at	ITGAL	0	1.43	5.52E-41
213478_at	RP1-21O18.1	0		N.S.
213483_at	PPWD1	0	1.10	1.00E-09
213485_s_at	ABCC10	0		N.S.
213489_at	MAPRE2	0	-1.30	<1.00E-120
213494_s_at	YY1	8		N.S.
213508_at	C14orf147	0		N.S.
213517_at	PCBP2	0		N.S.
213520_at	RECQL4	0	-1.53	2.36E-24
213521_at	PTPN18	0		N.S.
213523_at	CCNE1	0	-2.25	3.51E-72
213524_s_at	G0S2	0	3.24	7.29E-41
213526_s_at	LIN37	0	1.46	4.75E-55
213527_s_at	ZNF688	0	1.56	2.21E-67
213528_at	C1orf156	0	-1.07	7.73E-03
213531_s_at	RAB3GAP1	0	1.63	1.45E-63

Supplemental Table 1

213534_s_at	PASK	1		N.S.
213540_at	HSD17B8	0	1.33	1.82E-27
213546_at	DKFZP586I1420	0	1.11	3.69E-06
213548_s_at	CDV3	0		N.S.
213549_at	SLC18A2	0	-1.06	9.73E-03
213552_at	GLCE	0		N.S.
213555_at	RWDD2A	0	1.77	4.76E-54
213556_at	LOC390940	0		N.S.
213557_at	CRKRS	0	1.20	1.50E-12
213560_at	GADD45B	3		N.S.
213566_at	RNASE6	0	-1.57	2.64E-24
213568_at	OSR2	0	-1.15	1.52E-02
213577_at	SQLE	0	-2.11	5.63E-60
213587_s_at	ATP6V0E2	0	-1.15	1.17E-25
213590_at	LOC100133772	0		N.S.
213593_s_at	TRA2A	0	1.17	2.67E-02
213599_at	OIP5	0	-1.20	2.62E-38
213603_s_at	RAC2	0		N.S.
213604_at	TCEB3	0	-1.13	1.85E-08
213605_s_at	LOC100134401	0		N.S.
213606_s_at	ARHGDIA	0	-1.96	5.36E-55
213608_s_at	SRRD	0	-1.22	3.61E-28
213618_at	ARAP2	0	1.10	1.58E-11
213622_at	COL9A2	0	1.22	1.24E-26
213623_at	KIF3A	0	1.09	2.35E-05
213625_at	ZKSCAN4	0		N.S.
213626_at	CBR4	0	1.18	1.90E-19
213627_at	MAGED2	0	-1.29	4.70E-26
213634_s_at	TRMU	0	-1.47	1.51E-38
213638_at	PHACTR1	0		N.S.
213642_at	RPL27	0		N.S.
213647_at	DNA2	0	-1.70	1.44E-58
213650_at	GOLGA8A	0		N.S.
213654_at	TAF5L	0	-1.05	4.57E-03
213660_s_at	TOP3B	0		N.S.
213664_at	SLC1A1	0	1.27	3.03E-50
213671_s_at	MARS	0	2.21	2.10E-99
213677_s_at	PMS1	0	-1.22	3.20E-43
213679_at	TTC30A	0	-1.20	2.60E-05
213681_at	CYHR1	0		N.S.
213687_s_at	RPL35A	0		N.S.
213694_at	RSBN1	0	-1.08	2.61E-07
213699_s_at	YWHAQ	0	-1.08	3.01E-12
213701_at	C12orf29	0	-1.13	<1.00E-120
213703_at	LOC150759	0		N.S.
213704_at	RABGGTB	0	-1.33	2.02E-21
213705_at	MAT2A	0	-1.51	5.00E-22
213736_at	COX5B	0		N.S.
213738_s_at	ATP5A1	0		N.S.
213742_at	SFRS11	0		N.S.
213743_at	CCNT2	0		N.S.
213746_s_at	FLNA	0		N.S.
213748_at	TRIM66	0		N.S.
213757_at	EIF5A	1	-1.14	1.36E-06

Supplemental Table 1

213761_at	MDM1	0	1.24	<1.00E-120
213763_at	HIPK2	0		N.S.
213779_at	EMID1	0		N.S.
213787_s_at	EBP	174	-1.45	6.96E-54
213792_s_at	INSR	0	1.54	4.70E-41
213793_s_at	HOMER1	0	-1.45	1.47E-39
213794_s_at	NGDN	0	-1.17	3.34E-34
213798_s_at	CAP1	0	-1.17	3.29E-48
213799_s_at	PTPRA	0		N.S.
213803_at	KPNB1	0	-1.11	1.27E-06
213804_at	INPP5B	0	1.29	7.17E-32
213810_s_at	AKIRIN2	0	1.21	1.28E-11
213812_s_at	CAMKK2	0		N.S.
213820_s_at	STARD5	2	1.63	7.56E-43
213826_s_at	LOC100133109	0	-1.19	3.70E-05
213846_at	COX7C	0	-1.08	<1.00E-120
213850_s_at	SFRS2IP	0	-1.20	1.02E-23
213851_at	TMEM110	0	1.37	8.95E-34
213853_at	DNAJC24	0	1.22	1.65E-11
213861_s_at	FAM119B	0		N.S.
213872_at	C6orf62	0		N.S.
213878_at	PYROXD1	0	1.64	5.79E-71
213879_at	SUMO2	0		N.S.
213887_s_at	POLR2E	0	-1.20	1.11E-30
213888_s_at	LOC100133233	0	1.48	9.09E-62
213891_s_at	TCF4	1	1.10	9.45E-10
213897_s_at	MRPL23	0	-1.14	6.25E-22
213906_at	MYBL1	0	-1.25	3.01E-12
213908_at	WHAMML1	0	1.64	<1.00E-120
213916_at	ZNF20	0		N.S.
213919_at	DNAJC4	0		N.S.
213922_at	TTBK2	0	1.32	6.84E-18
213923_at	RAP2B	0	-1.18	1.13E-21
213927_at	MAP3K9	1	1.12	1.15E-04
213934_s_at	ZNF23	0		N.S.
213937_s_at	FTSJ1	0	1.48	1.75E-46
213938_at	ERC2	0	5.82	1.05E-40
213940_s_at	FNBP1	0		N.S.
213951_s_at	PSMC3IP	0	-1.33	1.31E-40
213959_s_at	RPGRIP1L	0	-1.74	2.19E-47
213963_s_at	SAP30	0		N.S.
213970_at	RABL3	0	-1.11	1.32E-06
213979_s_at	CTBP1	1		N.S.
213982_s_at	RABGAP1L	0		N.S.
214004_s_at	VGLL4	0		N.S.
214006_s_at	GGCX	0		N.S.
214007_s_at	TWF1	0	-1.43	4.81E-28
214011_s_at	NOP16	0	-1.80	6.00E-63
214022_s_at	IFITM1	0	1.16	4.94E-26
214030_at	CRYBG3	0	-1.21	1.13E-11
214036_at	EFNA5	0		N.S.
214039_s_at	LAPTM4B	0	-1.54	1.34E-28
214042_s_at	RPL22	0	1.05	2.64E-06
214045_at	LIAS	0	1.18	4.52E-19

Supplemental Table 1

214048_at	MBD4	0	1.35	2.65E-26
214051_at	TMSB15B	0	-1.18	1.59E-22
214054_at	DOK2	0	-1.15	6.46E-04
214057_at	MCL1	1		N.S.
214060_at	SSBP1	0		N.S.
214061_at	WDR67	0		N.S.
214075_at	NENF	0	-1.20	5.63E-23
214079_at	DHRS2	0	1.57	1.07E-24
214083_at	LOC100132532	0		N.S.
214086_s_at	PARP2	0	-1.06	4.63E-02
214096_s_at	SHMT2	0	2.25	2.91E-101
214101_s_at	NPEPPS	0	-1.15	4.43E-05
214106_s_at	GMDS	0	1.07	4.17E-05
214112_s_at	CXorf40A	0	1.32	1.23E-46
214113_s_at	RBM8A	0	-1.08	5.66E-10
214116_at	BTD	0		N.S.
214126_at	MCART1	0	1.38	1.23E-27
214132_at	ATP5C1	0		N.S.
214144_at	POLR2D	0	-1.42	2.11E-35
214148_at	FOXM1	0	1.18	4.76E-05
214152_at	CCPG1	0	3.54	2.43E-75
214153_at	ELOVL5	0	-1.51	1.40E-09
214155_s_at	LARP4	0	-1.35	6.95E-21
214167_s_at	RPLP0	0	1.07	3.20E-06
214179_s_at	NFE2L1	0	3.62	3.88E-114
214182_at	LOC100132430	0		N.S.
214186_s_at	HCG26	0		N.S.
214202_at	PGGT1B	0		N.S.
214221_at	ALMS1	0	-1.12	1.28E-05
214224_s_at	PIN4	0	-1.08	1.55E-08
214231_s_at	KIAA0564	0	-1.12	4.80E-03
214264_s_at	C14orf143	0		N.S.
214274_s_at	ACAA1	0		N.S.
214281_s_at	RCHY1	0	1.14	1.28E-31
214287_s_at	CDC2L5	0		N.S.
214290_s_at	HIST2H2AA3	0	1.11	<1.00E-120
214291_at	LOC729046	0	-1.51	3.27E-45
214305_s_at	SF3B1	0	-1.40	1.74E-19
214313_s_at	EIF5B	0	-1.31	3.01E-12
214328_s_at	HSP90AA1	0	-1.29	2.81E-45
214339_s_at	MAP4K1	0	-1.11	<1.00E-120
214352_s_at	KRAS	0	1.14	2.16E-04
214356_s_at	KIAA0368	0	1.24	7.37E-41
214364_at	MTERFD2	0	1.13	<1.00E-120
214366_s_at	ALOX5	0		N.S.
214374_s_at	PPFIBP1	0		N.S.
214377_s_at	CTRL	0		N.S.
214422_at	LOC131185	0		N.S.
214427_at	NOP2	0	-1.85	4.04E-68
214429_at	MTMR6	0	1.39	7.89E-23
214430_at	GLA	1	-1.46	1.56E-58
214431_at	GMPS	0	-1.40	3.04E-61
214440_at	NAT1	1	-1.15	8.11E-34
214441_at	STX6	0		N.S.

Supplemental Table 1

214446_at	ELL2	0		N.S.
214447_at	ETS1	0	1.24	<1.00E-120
214452_at	BCAT1	0	2.49	2.04E-58
214453_s_at	IFI44	0	-1.10	2.11E-06
214455_at	HIST1H2BC	0		N.S.
214467_at	GPR65	0	1.81	1.33E-64
214469_at	HIST1H2AE	0	-1.26	1.54E-22
214472_at	HIST1H2AD	0	1.15	4.80E-07
214474_at	PRKAB2	0		N.S.
214481_at	HIST1H2AM	0	-1.35	1.86E-23
214482_at	ZBTB25	0	-1.16	1.02E-18
214484_s_at	SIGMAR1	0	-1.54	6.81E-25
214502_at	HIST1H2BJ	0	1.64	7.66E-19
214507_s_at	EXOSC2	0	-2.00	6.63E-67
214513_s_at	CREB1	4	-1.23	<1.00E-120
214519_s_at	RLN2	0	-1.30	1.71E-21
214527_s_at	PQBP1	0	1.09	1.12E-18
214539_at	SERPINB10	0	1.49	1.77E-34
214554_at	HIST1H2AL	0	-1.21	2.10E-09
214572_s_at	INSL3	0		N.S.
214583_at	RSC1A1	0	-1.57	1.02E-52
214585_s_at	VPS52	0	-1.10	5.20E-06
214597_at	SSTR2	0		N.S.
214614_at	MNX1	0	1.40	7.47E-29
214615_at	P2RY10	0		N.S.
214617_at	PRF1	0		N.S.
214626_s_at	GANAB	0	1.11	9.69E-06
214657_s_at	NCRNA00084	0		N.S.
214658_at	TMED7	0	1.94	6.37E-58
214661_s_at	NOP14	0	-1.21	3.18E-21
214662_at	WDR43	0	-1.52	6.37E-63
214670_at	ZKSCAN1	0	1.17	3.20E-10
214672_at	TTLL5	0	-1.17	2.31E-24
214674_at	USP19	1		N.S.
214681_at	GK	0	1.19	7.13E-05
214683_s_at	CLK1	0	1.30	3.18E-43
214686_at	ZNF266	0	1.21	5.53E-42
214688_at	TLE4	0		N.S.
214690_at	TAF1B	0		N.S.
214694_at	LOC729143	0		N.S.
214696_at	C17orf91	0	2.85	1.53E-69
214697_s_at	ROD1	0		N.S.
214703_s_at	MAN2B2	0	1.18	2.22E-18
214709_s_at	KTN1	0		N.S.
214710_s_at	CCNB1	0	-1.29	1.54E-59
214711_at	GATC	0	-1.43	<1.00E-120
214714_at	ZNF394	0		N.S.
214717_at	DKFZp434H1419	0		N.S.
214719_at	SLC46A3	0	1.95	2.57E-71
214722_at	NOTCH2NL	0	-1.15	5.21E-04
214729_at	TWISTNB	0	-1.70	6.55E-31
214731_at	CTTNBP2NL	0		N.S.
214733_s_at	YIPF1	0	1.30	7.49E-30
214735_at	IPCEF1	0		N.S.

Supplemental Table 1

214736_s_at	ADD1	0		N.S.
214739_at	LRCH3	0	1.11	3.87E-10
214741_at	ZNF131	0		N.S.
214742_at	AZI1	0	-1.13	1.87E-03
214744_s_at	RPL23	1	-1.14	2.06E-09
214747_at	ZBED4	0		N.S.
214748_at	N4BP2L2	0	3.09	3.20E-66
214749_s_at	ARMCX6	0	1.16	4.93E-31
214751_at	ZNF468	0	-1.19	6.30E-08
214755_at	UAP1L1	0	-1.13	3.64E-07
214757_at	PMS2L2	0	1.14	3.56E-06
214759_at	WTAP	0		N.S.
214762_at	ATP6V1G2	0	1.44	7.52E-13
214764_at	RRP15	0	-1.36	<1.00E-120
214765_s_at	NAAA	0	-1.32	4.71E-22
214766_s_at	AHCTF1	0	-1.15	5.25E-07
214772_at	C11orf41	0		N.S.
214778_at	MEGF8	0		N.S.
214779_s_at	SGSM3	0	1.22	<1.00E-120
214780_s_at	MYO9B	0		N.S.
214785_at	VPS13A	1	-1.31	<1.00E-120
214787_at	DENND4A	0		N.S.
214790_at	SENP6	0		N.S.
214791_at	SP140L	0	1.23	<1.00E-120
214801_at	IFRG15	0	-1.30	2.09E-29
214804_at	CENPI	0	-1.40	9.45E-38
214806_at	BICD1	0		N.S.
214813_at	ZNF75D	0	1.73	3.56E-48
214814_at	YTHDC1	0		N.S.
214828_s_at	RRP7A	0	-1.65	1.79E-42
214830_at	SLC38A6	0		N.S.
214838_at	SFT2D2	0		N.S.
214843_s_at	USP33	0	-1.18	2.62E-20
214848_at	YWHAZ	0		N.S.
214849_at	KCTD20	0	-1.16	2.41E-11
214850_at	LOC100170939	0	1.14	5.46E-04
214857_at	C10orf95	0	-1.31	1.61E-26
214861_at	KDM4C	0		N.S.
214864_s_at	GRHPR	0	-1.04	5.91E-04
214876_s_at	TUBGCP5	0	-1.38	6.52E-25
214878_at	ZNF37A	0		N.S.
214881_s_at	UBTF	0	-1.71	6.34E-51
214882_s_at	SFRS2	0	-2.01	1.39E-80
214895_s_at	ADAM10	0		N.S.
214910_s_at	APOM	0	1.36	1.49E-33
214918_at	HNRNPM	0		N.S.
214919_s_at	ANKHD1-EIF4EBP3	0	1.29	5.22E-45
214931_s_at	SRPK2	0	-1.13	1.60E-02
214941_s_at	PRPF40A	0	-1.14	9.10E-11
214943_s_at	RBM34	0		N.S.
214948_s_at	TMF1	0	-1.43	1.24E-52
214949_at	hCG_1795560	0	-1.37	2.47E-70
214953_s_at	APP	15		N.S.
214958_s_at	TMC6	0	1.15	2.38E-08

Supplemental Table 1

214962_s_at	NUP160	0	-1.47	5.08E-30
214965_at	SPATA2L	0	-1.14	5.96E-07
214972_at	MGEA5	0		N.S.
214975_s_at	MTMR1	0	-1.26	4.98E-09
214976_at	RPL13	0	-1.85	9.09E-29
214982_at	SNRNP200	0		N.S.
214991_s_at	PIGO	0	1.17	6.60E-04
214992_s_at	DNASE2	0	2.81	2.39E-85
214994_at	APOBEC3F	0	-1.20	9.85E-11
214997_at	C9orf126	0		N.S.
215001_s_at	GLUL	0	-1.74	2.39E-71
215006_at	EZH2	0		N.S.
215011_at	SNHG3	0	-1.82	1.96E-56
215023_s_at	PEX1	0		N.S.
215024_at	C7orf28B	0	1.19	1.04E-08
215029_at	C1orf108	0		N.S.
215030_at	GRSF1	0	-1.24	2.09E-29
215043_s_at	LOC653188	0		N.S.
215046_at	C2orf67	0	1.21	5.35E-06
215058_at	DENND5B	0		N.S.
215068_s_at	FBXL18	0	-1.41	1.60E-34
215071_s_at	HIST1H2AC	0	1.61	8.63E-58
215075_s_at	GRB2	0	-1.06	1.26E-06
215084_s_at	LRRC42	0	-1.09	1.41E-04
215087_at	C15orf39	0	-1.31	1.09E-25
215088_s_at	SDHC	0	-1.14	2.69E-08
215089_s_at	RBM10	0	1.11	2.98E-10
215091_s_at	GTF3A	0	-1.09	3.01E-12
215096_s_at	ESD	0	-1.12	8.22E-19
215109_at	KIAA0492	0		N.S.
215111_s_at	TSC22D1	0		N.S.
215123_at	LOC642778	0		N.S.
215127_s_at	RBMS1	0	-1.35	5.27E-11
215134_at	PI4K2A	0	1.15	7.61E-09
215136_s_at	EXOSC8	0	1.35	1.83E-66
215143_at	DPY19L2P2	0	-1.62	1.47E-30
215148_s_at	APBA3	0	1.18	1.13E-32
215159_s_at	NADK	0	-1.23	2.55E-08
215170_s_at	CEP152	0	-1.11	3.90E-03
215190_at	EIF3M	0	-1.24	1.49E-10
215191_at	FBXL11	0		N.S.
215201_at	REPS1	0		N.S.
215210_s_at	DLST	0	-1.10	3.28E-23
215218_s_at	WDR62	0		N.S.
215223_s_at	SOD2	0	-1.24	1.09E-28
215224_at	SNORA21	0	-1.87	2.05E-23
215271_at	TNN	0	1.19	4.52E-04
215285_s_at	PHTF1	0	-1.11	2.99E-10
215307_at	ZNF529	0		N.S.
215318_at	CG012	0	1.95	4.38E-23
215338_s_at	NKTR	0	-1.19	8.09E-10
215343_at	CCDC88C	0		N.S.
215351_at	RTCD1	0	1.30	1.17E-05
215354_s_at	PELP1	0	-1.30	1.20E-28

Supplemental Table 1

215378_at	ANKHD1	0		N.S.
215380_s_at	GGCT	0		N.S.
215390_at	C9orf5	0		N.S.
215399_s_at	OS9	0	1.76	2.23E-57
215416_s_at	STOML2	0		N.S.
215424_s_at	SNW1	0	-1.06	4.42E-04
215429_s_at	ZNF428	0		N.S.
215440_s_at	BEX4	0	1.46	1.44E-10
215465_at	ABCA12	1	1.76	3.01E-12
215470_at	GTF2H2B	0		N.S.
215482_s_at	EIF2B4	0	-1.14	<1.00E-120
215485_s_at	ICAM1	0	-1.14	2.07E-04
215499_at	MAP2K3	1	-1.08	3.58E-04
215501_s_at	DUSP10	0	-1.23	2.02E-23
215505_s_at	STRN3	0		N.S.
215509_s_at	BUB1	0		N.S.
215543_s_at	LARGE	111		N.S.
215545_at	ERCC3	0		N.S.
215548_s_at	SCFD1	0	1.33	1.67E-51
215577_at	UBE2E1	0		N.S.
215596_s_at	RNF160	0	1.10	2.14E-08
215602_at	FGD2	0		N.S.
215606_s_at	ERC1	0		N.S.
215631_s_at	BRMS1	0	-1.10	<1.00E-120
215639_at	SH2D3C	0	2.47	2.89E-32
215641_at	SEC24D	0	2.75	3.27E-41
215648_at	NUDCD3	0		N.S.
215684_s_at	ASCC2	0		N.S.
215694_at	SPATA5L1	0	-1.41	2.63E-11
215696_s_at	SEC16A	1	1.08	8.82E-08
215707_s_at	PRNP	0	1.27	3.23E-11
215711_s_at	WEE1	1	-1.26	1.38E-20
215718_s_at	PHF3	0		N.S.
215728_s_at	ACOT7	0	-1.39	1.37E-34
215732_s_at	DTX2	0	1.54	2.51E-29
215734_at	C19orf36	0		N.S.
215739_s_at	TUBGCP3	0		N.S.
215743_at	NMT2	0		N.S.
215747_s_at	RCC1	0	-1.58	2.48E-35
215749_s_at	GORASP1	0		N.S.
215750_at	KIAA1659	0		N.S.
215767_at	ZNF804A	0	1.58	9.47E-35
215779_s_at	HIST1H2BG	0		N.S.
215780_s_at	hCG_1644608	0	-1.20	2.81E-09
215785_s_at	CYFIP2	0	1.12	7.37E-18
215792_s_at	DNAJC11	0	-1.33	1.45E-45
215836_s_at	PCDHGA1	0		N.S.
215854_at	FBXO22	0		N.S.
215884_s_at	UBQLN2	0	-1.10	<1.00E-120
215905_s_at	SNRNP40	0	-1.26	3.57E-54
215910_s_at	FNDC3A	0	1.52	5.86E-31
215919_s_at	MRPS11	0	-1.21	9.31E-04
215925_s_at	CD72	0		N.S.
215930_s_at	CTAGE5	0	2.40	5.20E-47

215931_s_at	ARFGEF2	0		N.S.
215938_s_at	PLA2G6	2	1.69	1.32E-24
215942_s_at	GTSE1	0	-1.18	1.15E-23
215947_s_at	FAM136A	0	-1.54	2.09E-52
215954_s_at	C19orf29	0	-1.14	7.25E-05
215967_s_at	LY9	0	2.94	1.59E-58
215983_s_at	UBXN8	0		N.S.
215984_s_at	ARFRP1	0		N.S.
215985_at	NCRNA00171	0	1.31	<1.00E-120
216006_at	RAPGEFL1	0		N.S.
216020_at	IFIH1	0	-1.38	4.21E-25
216026_s_at	POLE	3	-1.52	3.72E-53
216028_at	DKFZP564C152	0	1.09	3.17E-02
216032_s_at	ERGIC3	0	1.09	<1.00E-120
216060_s_at	DAAM1	0	1.21	1.24E-18
216088_s_at	PSMA7	0	-1.16	6.78E-21
216114_at	NCKIPSD	0	-1.35	1.27E-06
216177_at	LOC391132	0		N.S.
216194_s_at	TBCB	0		N.S.
216199_s_at	MAP3K4	0		N.S.
216218_s_at	PLCL2	0		N.S.
216226_at	TAF4B	0	-1.19	8.09E-10
216231_s_at	B2M	1		N.S.
216232_s_at	GCN1L1	0	-1.34	4.72E-55
216241_s_at	TCEA1	0	1.88	6.21E-113
216247_at	RPS20	0	-1.25	3.89E-03
216248_s_at	NR4A2	1	1.30	1.62E-05
216250_s_at	LPXN	0	-1.06	1.02E-05
216251_s_at	TTLL12	0	-2.43	3.27E-88
216262_s_at	TGIF2	0	-1.13	7.52E-13
216266_s_at	ARFGEF1	0		N.S.
216267_s_at	TMEM115	0	1.25	8.92E-21
216278_at	KIAA0256	0	1.34	5.34E-04
216288_at	CYSLTR1	0	-1.40	5.02E-06
216305_s_at	C2orf3	0	-1.21	6.68E-18
216326_s_at	HDAC3	0	-1.13	2.44E-20
216338_s_at	YIPF3	0	1.41	2.00E-50
216347_s_at	PPP1R13B	0		N.S.
216348_at	RPS17P5	0		N.S.
216361_s_at	MYST3	0		N.S.
216383_at	RPL18A	0		N.S.
216389_s_at	WDR23	0	1.15	5.31E-09
216396_s_at	EI24	0	-1.36	1.87E-44
216397_s_at	BOP1	0	-1.56	2.89E-37
216411_s_at	GALK2	0	1.62	2.16E-34
216421_at	GTSF1L	0		N.S.
216438_s_at	TMSB4X	0		N.S.
216520_s_at	TPT1	1		N.S.
216521_s_at	BRCC3	0	-1.39	4.65E-24
216531_at	YY2	0		N.S.
216547_at	LOC127406	0		N.S.
216555_at	C22orf30	0	-1.46	2.03E-10
216563_at	ANKRD12	0		N.S.
216574_s_at	hCG_2024410	0	-1.51	7.74E-49

216591_s_at	hCG_1776980	0	-1.12	1.28E-09
216602_s_at	FARSA	0	-1.28	3.89E-29
216607_s_at	CYP51A1	0	-1.68	5.10E-64
216609_at	TXN	0		N.S.
216627_s_at	B4GALT1	0		N.S.
216640_s_at	PDIA6	0	1.75	1.86E-66
216650_at	LOC283412	0		N.S.
216678_at	IFT122	0		N.S.
216699_s_at	KLK1	0		N.S.
216705_s_at	ADA	3		N.S.
216783_at	LOC283677	0		N.S.
216806_at	RPSA	0		N.S.
216834_at	RGS1	0		N.S.
216835_s_at	DOK1	0	-1.22	5.94E-26
216836_s_at	ERBB2	1	1.27	<1.00E-120
216860_s_at	GDF11	0	-1.43	<1.00E-120
216862_s_at	MTCP1NB	0		N.S.
216863_s_at	MORC2	0	-1.27	1.67E-29
216873_s_at	ATP8B2	0		N.S.
216899_s_at	SKAP2	0	-1.11	7.70E-05
216902_s_at	LOC653390	0		N.S.
216903_s_at	CBARA1	0	1.07	6.07E-08
216942_s_at	CD58	0	-1.21	3.53E-29
216944_s_at	ITPR1	0	2.26	7.71E-70
216952_s_at	LMNB2	0	-1.34	2.99E-36
216961_s_at	RPAIN	0		N.S.
216969_s_at	KIF22	0		N.S.
216993_s_at	COL11A2	4		N.S.
216996_s_at	FASTKD2	0	-1.64	1.43E-74
217019_at	RPS4X	0		N.S.
217025_s_at	DBN1	0	1.28	6.73E-07
217028_at	CXCR4	0		N.S.
217042_at	RDH11	0	-1.35	1.85E-05
217043_s_at	MFN1	0	1.07	3.84E-03
217047_s_at	FAM13A	0	1.30	1.51E-09
217094_s_at	ITCH	1	1.19	<1.00E-120
217100_s_at	UBXN7	0		N.S.
217104_at	ST20	0	1.49	2.80E-28
217118_s_at	C22orf9	0	-1.42	5.22E-56
217122_s_at	RP11-345P4.4	0	1.38	1.65E-51
217124_at	IQCE	0		N.S.
217125_at	UBBP2	0		N.S.
217127_at	CTH	0	8.42	1.59E-109
217144_at	LOC648390	0		N.S.
217168_s_at	HERPUD1	18	2.90	2.59E-75
217176_s_at	ZFX	0		N.S.
217188_s_at	C14orf1	0	-1.35	7.05E-36
217211_at	ACTBP9	0		N.S.
217266_at	RPL15P22	0		N.S.
217286_s_at	NDRG3	0	-1.07	1.69E-02
217289_s_at	SLC37A4	0		N.S.
217299_s_at	NBN	0	1.14	6.71E-10
217310_s_at	FOXJ3	0		N.S.
217317_s_at	HERC2P2	0	1.11	3.76E-12

Supplemental Table 1

217336_at	RPS10	0		N.S.
217340_at	RPL21P68	0		N.S.
217346_at	LOC128192	0		N.S.
217365_at	PRAMEF11	0		N.S.
217368_at	ATP5G2	0		N.S.
217379_at	RPL10	0	1.17	4.88E-08
217383_at	PGK1	0	-1.26	3.52E-04
217388_s_at	KYNU	0	-1.23	6.46E-49
217403_s_at	ZNF227	0	1.26	3.07E-26
217408_at	MRPS18B	0	1.23	1.08E-43
217427_s_at	HIRA	1	-1.14	<1.00E-120
217445_s_at	GART	0	-1.38	8.02E-10
217448_s_at	LOC285412	0		N.S.
217457_s_at	RAP1GDS1	0	-1.13	6.81E-08
217478_s_at	HLA-DMA	0	-1.09	7.52E-13
217494_s_at	PTENP1	0		N.S.
217496_s_at	IDE	2		N.S.
217501_at	CIAO1	0		N.S.
217503_at	STK17B	0	-1.23	4.43E-18
217504_at	ABCA6	0	1.41	1.38E-29
217506_at	LOC339290	0		N.S.
217523_at	CD44	2		N.S.
217527_s_at	NFATC2IP	0	-1.26	3.43E-27
217538_at	SGSM2	0	-1.23	7.95E-03
217539_at	C18orf25	0		N.S.
217540_at	FAM55C	0		N.S.
217544_at	LOC729806	0	1.20	4.70E-04
217549_at	NCKAP1L	0	1.38	4.12E-38
217555_at	SMC1A	0	-1.21	2.89E-02
217559_at	RPL10L	0	1.33	1.54E-04
217588_at	CATSPER2	0	1.30	3.08E-10
217591_at	SKIL	0		N.S.
217599_s_at	MDFIC	0		N.S.
217608_at	SFRS12IP1	0	-1.19	3.99E-11
217609_at	LRRC23	0	1.25	4.51E-12
217612_at	TIMM50	0	-1.18	2.78E-06
217627_at	ZNF573	0		N.S.
217645_at	COX16	0	-1.16	<1.00E-120
217663_at	ZNF234	0		N.S.
217667_at	LOC729799	0		N.S.
217677_at	PLEKHA2	0	-1.38	7.52E-13
217682_at	C16orf72	0	1.33	<1.00E-120
217716_s_at	SEC61A1	1	2.22	2.37E-83
217718_s_at	YWHAB	0	1.05	5.09E-07
217719_at	EIF3L	0		N.S.
217720_at	CHCHD2	0	1.05	1.77E-04
217722_s_at	NGRN	0	1.17	9.10E-30
217726_at	COPZ1	0	1.35	9.66E-31
217728_at	S100A6	0	-1.14	7.78E-03
217729_s_at	AES	0	-1.16	<1.00E-120
217730_at	TMBIM1	0	1.25	2.30E-23
217731_s_at	ITM2B	0	1.29	6.75E-07
217733_s_at	TMSB10	0	-1.06	1.23E-02
217734_s_at	WDR6	0		N.S.

217736_s_at	EIF2AK1	196	1.08	<1.00E-120
217739_s_at	NAMPT	0	1.11	1.28E-09
217742_s_at	WAC	0	1.14	4.46E-36
217743_s_at	TMEM30A	0	1.15	<1.00E-120
217744_s_at	PERP	0		N.S.
217745_s_at	NAT13	0	-1.51	5.39E-50
217746_s_at	PDCD6IP	0		N.S.
217747_s_at	RPS9	0	1.07	6.31E-06
217748_at	ADIPOR1	0	-1.07	4.76E-04
217749_at	COPG	0	1.55	1.14E-55
217750_s_at	UBE2Z	0	1.12	3.18E-26
217751_at	GSTK1	0		N.S.
217752_s_at	CNDP2	0		N.S.
217753_s_at	RPS26	0	-1.11	3.76E-12
217754_at	DDX56	0	-1.36	6.79E-47
217755_at	HN1	0	-1.43	1.20E-76
217758_s_at	TM9SF3	0	1.51	1.13E-71
217759_at	TRIM44	0		N.S.
217761_at	ADI1	0	-1.06	1.89E-02
217763_s_at	RAB31	0	-1.20	1.40E-09
217765_at	NRBP1	0	1.11	2.80E-09
217766_s_at	TMEM50A	0	1.11	9.18E-21
217768_at	C14orf166	0		N.S.
217769_s_at	POMP	0		N.S.
217770_at	PIGT	0	1.08	3.87E-03
217771_at	GOLM1	0		N.S.
217772_s_at	MTCH2	0	-1.24	7.16E-48
217773_s_at	NDUFA4	0	-1.05	2.47E-04
217774_s_at	HSPC152	0	-1.11	2.78E-18
217777_s_at	PTPLAD1	1	-1.61	4.57E-61
217778_at	SLC39A1	0		N.S.
217779_s_at	LOC100132235	0	1.23	8.57E-49
217780_at	C19orf56	0	1.09	2.26E-12
217781_s_at	ZFP106	0		N.S.
217782_s_at	GPS1	0	-1.16	3.01E-12
217783_s_at	YPEL5	0	1.91	2.67E-70
217784_at	YKT6	0	1.12	2.74E-07
217786_at	PRMT5	0	-1.32	4.97E-36
217788_s_at	GALNT2	0		N.S.
217789_at	SNX6	0	1.30	2.44E-32
217790_s_at	SSR3	0	2.17	5.32E-64
217791_s_at	ALDH18A1	0	-1.45	8.73E-59
217792_at	SNX5	0	1.09	1.35E-10
217794_at	PRR13	0	-1.24	1.62E-51
217795_s_at	TMEM43	0	1.29	2.50E-52
217796_s_at	NPLOC4	1	-1.16	7.52E-13
217797_at	UFC1	0	1.09	<1.00E-120
217800_s_at	NDFIP1	0	1.56	7.57E-49
217801_at	ATP5E	0		N.S.
217803_at	GOLPH3	0		N.S.
217805_at	ILF3	0	-1.43	8.83E-56
217806_s_at	POLDIP2	0	-1.09	3.84E-11
217807_s_at	GLTSCR2	0	1.14	6.20E-27
217808_s_at	MAPKAP1	0		N.S.

Supplemental Table 1

217809_at	BZW2	0	-1.51	2.08E-49
217811_at	SELT	0		N.S.
217812_at	YTHDF2	0	-1.24	6.21E-38
217813_s_at	SPIN1	0	-1.20	9.26E-07
217814_at	CCDC47	0	1.06	1.98E-10
217815_at	SUPT16H	0	-1.36	4.05E-57
217816_s_at	PCNP	0	1.07	1.07E-08
217819_at	GOLGA7	0		N.S.
217822_at	WBP11	0	-1.30	2.38E-47
217826_s_at	UBE2J1	1	1.39	4.94E-51
217827_s_at	SPG21	0	-1.10	<1.00E-120
217828_at	SLTM	0	1.22	1.25E-43
217829_s_at	USP39	0	-1.28	2.18E-30
217830_s_at	NSFL1C	0		N.S.
217833_at	SYNCRIP	0	-1.56	1.10E-74
217836_s_at	YY1AP1	0	1.18	5.70E-30
217837_s_at	VPS24	0	1.19	1.35E-40
217838_s_at	EVL	0	1.06	4.99E-04
217840_at	DDX41	0	-1.16	3.90E-34
217841_s_at	PPME1	0	-1.12	4.14E-11
217843_s_at	MED4	0	-1.24	4.09E-18
217844_at	CTDSP1	0	1.08	9.57E-05
217846_at	QARS	0		N.S.
217847_s_at	THRAP3	0	-1.47	1.99E-26
217848_s_at	PPA1	0	-1.13	2.80E-37
217849_s_at	CDC42BPB	0	1.16	4.28E-03
217850_at	GNL3	0		N.S.
217851_s_at	SLMO2	0		N.S.
217852_s_at	ARL8B	0	1.20	3.59E-44
217853_at	TNS3	0		N.S.
217858_s_at	ARMCX3	0	1.92	5.25E-70
217860_at	LOC732160	0		N.S.
217861_s_at	PREB	0	1.50	1.22E-49
217862_at	PIAS1	0		N.S.
217866_at	CPSF7	0		N.S.
217868_s_at	METTL9	0		N.S.
217869_at	HSD17B12	0	1.12	2.43E-30
217870_s_at	CMPK1	0	1.08	<1.00E-120
217871_s_at	MIF	1	-1.08	1.50E-12
217872_at	PIH1D1	0	1.08	4.65E-09
217873_at	CAB39	0	-1.10	<1.00E-120
217874_at	SUCLG1	0	1.07	<1.00E-120
217876_at	GTF3C5	0		N.S.
217877_s_at	GPBP1L1	0	1.14	2.34E-20
217878_s_at	CDC27	0		N.S.
217882_at	TMEM111	0	1.10	1.58E-17
217883_at	MMADHC	0	1.07	9.19E-09
217884_at	NAT10	0	-1.64	1.76E-84
217885_at	IPO9	0	-1.32	2.82E-46
217886_at	EPS15	0	1.23	9.97E-28
217888_s_at	ARFGAP1	0		N.S.
217889_s_at	CYBRD1	0	1.21	1.66E-17
217892_s_at	LIMA1	0	-1.06	4.88E-05
217893_s_at	AKIRIN1	0	-1.13	7.00E-11

Supplemental Table 1

217894_at	KCTD3	0	-1.39	2.68E-19
217895_at	PTCD3	0	-1.07	1.27E-07
217896_s_at	NIP30	0		N.S.
217898_at	C15orf24	0	1.14	6.71E-21
217899_at	TMEM214	0	1.38	6.50E-30
217900_at	IARS2	0	-1.14	3.11E-30
217901_at	DSG2	0	-1.09	7.08E-04
217902_s_at	HERC2	0		N.S.
217903_at	STRN4	0		N.S.
217905_at	C10orf119	0	-1.35	2.75E-23
217906_at	KLHDC2	0	1.85	9.52E-92
217907_at	MRPL18	0	-1.36	1.94E-57
217908_s_at	IQWD1	0		N.S.
217911_s_at	BAG3	0		N.S.
217912_at	DUS1L	0	-1.09	1.84E-04
217913_at	VPS4A	0	-1.12	2.92E-10
217914_at	TPCN1	0	1.51	9.74E-49
217915_s_at	RSL24D1	0	1.45	3.53E-69
217918_at	DYNLRB1	0	1.05	3.47E-06
217919_s_at	MRPL42	0	-1.16	8.83E-30
217923_at	PEF1	0	-1.09	<1.00E-120
217924_at	C6orf106	0	-1.20	6.68E-21
217926_at	C19orf53	0	-1.19	2.57E-35
217927_at	SPCS1	0	1.09	2.62E-20
217928_s_at	SAPS3	0	-1.08	9.09E-04
217929_s_at	KIAA0319L	0	1.51	6.37E-39
217930_s_at	TOLLIP	0	-1.08	7.36E-04
217931_at	CNPY3	0	1.25	1.35E-25
217932_at	MRPS7	0	-1.31	1.16E-39
217933_s_at	LAP3	0		N.S.
217935_s_at	UQCC	0		N.S.
217936_at	ARHGAP5	0	1.27	1.71E-23
217938_s_at	KCMF1	0	1.18	1.21E-45
217939_s_at	AFTPH	0	1.21	1.47E-48
217940_s_at	CARKD	0	1.18	2.54E-21
217941_s_at	ERBB2IP	0	1.46	5.02E-52
217942_at	MRPS35	0		N.S.
217943_s_at	MAP7D1	0		N.S.
217944_at	POMGNT1	0	-1.23	<1.00E-120
217945_at	BTBD1	0		N.S.
217946_s_at	SAE1	0	-1.20	3.16E-23
217947_at	CMTM6	0	1.09	2.06E-19
217949_s_at	VKORC1	0	-1.28	2.38E-49
217950_at	NOSIP	0		N.S.
217955_at	BCL2L13	0	1.10	3.24E-06
217956_s_at	ENOPH1	0	-1.19	8.02E-38
217957_at	C16orf80	0	-1.12	<1.00E-120
217959_s_at	TRAPPC4	0	-1.17	2.80E-30
217960_s_at	TOMM22	0	-1.28	1.61E-39
217961_at	SLC25A38	0		N.S.
217962_at	NOP10	0	-1.22	9.30E-41
217963_s_at	NGFRAP1	0		N.S.
217964_at	TTC19	0	1.13	5.82E-19
217965_s_at	SAP30BP	0		N.S.

Supplemental Table 1

217966_s_at	FAM129A	1	2.47	1.60E-74
217968_at	TSSC1	0	1.17	3.93E-28
217969_at	C11orf2	0	1.08	1.13E-11
217970_s_at	CNOT6	0	-1.09	5.72E-09
217971_at	MAPKSP1	0	1.10	3.01E-12
217972_at	CHCHD3	0		N.S.
217973_at	DCXR	0	-1.06	1.56E-05
217974_at	TM7SF3	0		N.S.
217975_at	WBP5	0	-1.09	3.55E-02
217976_s_at	DYNC1LI1	0		N.S.
217977_at	SEPX1	0	-1.51	5.79E-46
217978_s_at	UBE2Q1	0		N.S.
217980_s_at	MRPL16	0	-1.39	5.28E-62
217981_s_at	FXC1	0	-1.15	1.20E-11
217982_s_at	MORF4L1	0		N.S.
217984_at	RNASET2	0	-1.09	2.76E-07
217986_s_at	BAZ1A	0	-1.60	3.95E-65
217987_at	ASNSD1	0		N.S.
217988_at	CCNB1IP1	0	1.38	3.81E-54
217989_at	HSD17B11	0	1.86	2.64E-77
217990_at	GMPR2	0	1.08	5.75E-08
217992_s_at	EFHD2	0	-1.85	1.11E-74
217993_s_at	MAT2B	0	1.19	5.42E-51
217995_at	SQRDL	0	1.06	2.65E-03
217997_at	PHLDA1	2		N.S.
218001_at	MRPS2	0	-1.37	7.06E-50
218003_s_at	FKBP3	0	-1.14	5.27E-12
218005_at	ZNF22	0	1.07	8.73E-11
218007_s_at	RPS27L	0	1.11	1.16E-30
218008_at	C7orf42	0	1.09	7.52E-13
218009_s_at	PRC1	0	-1.24	1.68E-45
218011_at	UBL5	0	-1.22	8.06E-35
218012_at	TSPYL2	0	1.66	1.46E-33
218014_at	NUP85	0	-1.65	1.25E-82
218016_s_at	POLR3E	0	-1.37	8.20E-65
218017_s_at	HGSNAT	0		N.S.
218018_at	PDXK	0	1.10	3.64E-02
218020_s_at	ZFAND3	0	1.71	6.71E-57
218021_at	DHRS4	0	-1.11	4.36E-05
218022_at	VRK3	0	1.10	1.49E-08
218023_s_at	FAM53C	0		N.S.
218024_at	BRP44L	0	1.22	1.94E-31
218025_s_at	PECI	0	1.08	6.84E-11
218026_at	CCDC56	0	-1.43	1.64E-73
218027_at	MRPL15	0	-1.16	7.24E-30
218030_at	GIT1	1		N.S.
218032_at	SNN	0	-1.63	1.44E-67
218034_at	FIS1	0		N.S.
218035_s_at	RBM47	0	1.18	1.44E-22
218039_at	NUSAP1	0		N.S.
218040_at	PRPF38B	0	-1.09	3.14E-05
218042_at	COPS4	0	-1.07	2.77E-19
218043_s_at	AZI2	0	1.68	3.87E-35
218046_s_at	MRPS16	0	-1.15	3.08E-31

Supplemental Table 1

218047_at	OSBPL9	0	1.20	1.12E-51
218048_at	COMMD3	0	1.12	5.07E-23
218049_s_at	MRPL13	0	-1.17	7.25E-44
218050_at	UFM1	2	1.76	3.09E-65
218051_s_at	NT5DC2	0	1.27	9.01E-19
218052_s_at	ATP13A1	0	1.16	<1.00E-120
218055_s_at	WDR41	0	1.94	1.74E-87
218056_at	BFAR	0	1.09	7.52E-13
218058_at	CXXC1	0	-1.39	1.03E-55
218059_at	ZNF706	0	-1.05	1.11E-04
218060_s_at	C16orf57	0	-1.29	<1.00E-120
218061_at	MEA1	0	-1.18	1.32E-25
218065_s_at	TMEM9B	0		N.S.
218066_at	SLC12A7	0	-1.17	6.51E-03
218067_s_at	ARGLU1	0	-1.06	2.45E-05
218068_s_at	ZNF672	0	-1.49	1.95E-67
218069_at	DCTPP1	0	-1.87	1.10E-73
218070_s_at	GMPPA	0	2.26	6.79E-73
218071_s_at	MKRN2	0	1.22	6.06E-49
218072_at	COMMD9	0		N.S.
218073_s_at	TMEM48	0		N.S.
218074_at	FAM96B	0		N.S.
218075_at	AAAS	0	-1.15	4.21E-11
218076_s_at	ARHGAP17	0	-1.07	5.17E-05
218077_s_at	ZDHHC3	0	-1.08	1.31E-05
218079_s_at	GGNBP2	0	1.10	<1.00E-120
218081_at	C20orf27	0	-1.49	2.83E-36
218082_s_at	UBP1	0	-1.11	1.94E-26
218083_at	PTGES2	0	-1.40	2.48E-35
218085_at	CHMP5	0	1.23	3.43E-24
218088_s_at	RRAGC	0	1.07	9.70E-11
218089_at	C20orf4	0	-1.11	7.52E-13
218090_s_at	BRWD2	0	1.33	1.78E-35
218093_s_at	ANKRD10	0	1.07	1.56E-04
218095_s_at	TMEM165	0	1.32	1.62E-33
218096_at	AGPAT5	0	1.06	6.67E-06
218097_s_at	CUEDC2	0	-1.09	6.67E-08
218099_at	TEX2	0	-1.32	4.36E-35
218100_s_at	IFT57	0		N.S.
218101_s_at	NDUFC2	0		N.S.
218102_at	DERA	0	-1.09	3.69E-20
218103_at	FTSJ3	0	-1.59	2.91E-76
218104_at	TEX10	0	-1.37	5.55E-59
218105_s_at	MRPL4	0	-1.27	6.90E-46
218106_s_at	MRPS10	0	1.19	7.20E-39
218107_at	WDR26	0	1.14	1.21E-32
218108_at	UBR7	0	-1.23	1.02E-47
218109_s_at	MFSD1	0	1.18	9.63E-33
218110_at	XAB2	0	-1.11	2.19E-05
218111_s_at	CMAS	0	-1.12	3.01E-12
218112_at	MRPS34	0	-1.54	2.05E-55
218115_at	ASF1B	0	-1.61	1.40E-79
218116_at	C9orf78	0	-1.23	3.84E-20
218117_at	RBX1	1	-1.17	3.00E-28

218118_s_at	TIMM23	0	-1.52	6.12E-81
218121_at	HMOX2	0	-1.29	2.14E-19
218122_s_at	SENP2	0	1.42	2.19E-37
218123_at	C21orf59	0	-1.30	1.15E-46
218124_at	RETSAT	0	1.29	1.26E-46
218125_s_at	CCDC25	0	1.15	2.41E-10
218126_at	FAM82A2	0	1.16	6.73E-35
218129_s_at	NFYB	0	-1.20	6.90E-21
218130_at	C17orf62	0		N.S.
218131_s_at	GATAD2A	0	-1.14	1.53E-24
218132_s_at	TSEN34	0	-1.19	7.02E-38
218133_s_at	NIF3L1	0	-1.09	<1.00E-120
218134_s_at	RBM22	0		N.S.
218135_at	ERGIC2	0	1.14	3.71E-19
218137_s_at	SMAP1	0	1.15	5.41E-35
218138_at	MKKS	0	1.38	1.85E-42
218139_s_at	MUDENG	0	1.10	3.32E-28
218141_at	UBE2O	0	-1.13	2.29E-10
218142_s_at	CRBN	0	1.33	5.34E-48
218143_s_at	SCAMP2	0	1.18	2.74E-31
218144_s_at	INF2	0	-1.54	6.29E-32
218145_at	TRIB3	10	9.16	6.29E-132
218147_s_at	GLT8D1	0	1.35	2.28E-30
218148_at	CENPT	0	-1.11	6.54E-06
218149_s_at	ZNF395	0	-1.59	1.10E-34
218150_at	ARL5A	0	-1.30	2.46E-41
218152_at	HMG20A	0		N.S.
218153_at	CARS2	0	-1.23	4.44E-31
218154_at	GSDMD	0		N.S.
218158_s_at	APPL1	0		N.S.
218159_at	DDRGK1	0	1.11	6.77E-12
218160_at	NDUFA8	0	-1.39	3.64E-73
218161_s_at	CLN6	1		N.S.
218163_at	MCTS1	0		N.S.
218164_at	SPATA20	0		N.S.
218165_at	C1orf149	0	1.19	3.27E-19
218166_s_at	RSF1	0	-1.13	1.57E-05
218167_at	AMZ2	0	1.13	4.03E-29
218168_s_at	CABC1	0		N.S.
218170_at	ISOC1	0	-1.36	5.75E-20
218171_at	VPS4B	0	1.10	<1.00E-120
218172_s_at	DERL1	6	1.54	5.16E-47
218174_s_at	C10orf57	0		N.S.
218175_at	CCDC92	0	1.35	5.61E-32
218176_at	MAGEF1	0	1.14	4.33E-10
218178_s_at	CHMP1B	0		N.S.
218179_s_at	C4orf41	0		N.S.
218184_at	TULP4	0	1.11	5.04E-06
218185_s_at	ARMC1	0		N.S.
218187_s_at	C8orf33	0	-1.39	4.97E-44
218188_s_at	TIMM13	0	-1.30	1.26E-42
218189_s_at	NANS	0	1.75	7.83E-64
218190_s_at	UCRC	0	-1.20	5.43E-32
218191_s_at	LMBRD1	0	1.89	4.04E-81

Supplemental Table 1

218192_at	IP6K2	0	-1.08	4.74E-02
218193_s_at	GOLT1B	0	1.52	1.50E-55
218194_at	REXO2	0	-1.12	1.95E-18
218195_at	C6orf211	0	-1.13	9.78E-12
218196_at	OSTM1	0	1.14	1.98E-07
218197_s_at	OXR1	0		N.S.
218198_at	DHX32	0		N.S.
218199_s_at	NOL6	0	-1.95	3.76E-53
218201_at	NDUFB2	1	-1.19	2.71E-30
218203_at	ALG5	1	1.93	2.65E-87
218204_s_at	FYCO1	0		N.S.
218205_s_at	MKNK2	0	-1.24	1.70E-30
218208_at	LOC100131178	0	-1.11	<1.00E-120
218209_s_at	RPRD1A	0	1.09	1.93E-05
218210_at	FN3KRP	0	-1.23	5.06E-24
218211_s_at	MLPH	0		N.S.
218212_s_at	MOCS2	0		N.S.
218213_s_at	C11orf10	0	1.15	4.55E-35
218214_at	C12orf44	0	1.08	7.17E-06
218215_s_at	NR1H2	0	1.09	2.37E-04
218217_at	SCPEP1	0	1.23	3.06E-41
218218_at	APPL2	0	1.08	4.76E-02
218219_s_at	LANCL2	0		N.S.
218220_at	C12orf10	0	1.11	8.78E-19
218221_at	ARNT	2		N.S.
218223_s_at	PLEKHO1	0	1.07	9.72E-05
218224_at	PNMA1	0	-1.06	1.25E-03
218225_at	ECSIT	0	1.07	1.36E-03
218226_s_at	NDUFB4	0	-1.18	3.25E-34
218227_at	NUBP2	0		N.S.
218228_s_at	TNKS2	0	1.37	3.68E-44
218229_s_at	POGK	0	-1.18	4.16E-19
218230_at	ARFIP1	0		N.S.
218231_at	NAGK	0	1.08	1.80E-03
218233_s_at	PRICKLE4	0	-1.53	1.06E-65
218235_s_at	UTP11L	0	-1.34	1.49E-61
218236_s_at	PRKD3	0		N.S.
218237_s_at	SLC38A1	0	1.68	1.68E-99
218239_s_at	GTPBP4	0	-1.69	2.13E-68
218241_at	GOLGA5	0	1.44	9.16E-60
218242_s_at	SUV420H1	0	1.35	2.35E-45
218243_at	RUFY1	0	-1.19	5.02E-23
218244_at	NOL8	0	-1.20	1.32E-37
218247_s_at	MEX3C	0	1.34	2.71E-27
218248_at	FAM111A	0	-1.08	3.04E-07
218249_at	ZDHHC6	0	-1.14	4.77E-26
218250_s_at	CNOT7	0		N.S.
218251_at	MID1IP1	1	1.39	6.50E-27
218252_at	CKAP2	0	-1.10	4.95E-08
218253_s_at	LGTN	0	1.26	6.55E-49
218254_s_at	SAR1B	1	1.10	4.22E-08
218255_s_at	FBRS	0		N.S.
218257_s_at	UGCGL1	0	1.60	4.55E-67
218258_at	POLR1D	0	-1.05	4.87E-02

Supplemental Table 1

218259_at	MKL2	0	-1.07	2.16E-04
218260_at	DDA1	0	-1.08	1.39E-04
218263_s_at	ZBED5	0	1.10	4.15E-23
218264_at	BCCIP	0	-1.16	<1.00E-120
218265_at	SECISBP2	0	1.15	5.19E-10
218267_at	CINP	0	-1.21	3.24E-28
218268_at	TBC1D15	0	1.09	7.52E-13
218269_at	RNASEN	0	-1.14	2.10E-32
218270_at	MRPL24	0		N.S.
218271_s_at	PARL	0	1.14	3.97E-27
218272_at	TTC38	0		N.S.
218273_s_at	PPM2C	0		N.S.
218274_s_at	ANKZF1	0		N.S.
218275_at	SLC25A10	0	-1.63	1.31E-54
218276_s_at	SAV1	0	1.16	8.64E-28
218277_s_at	DHX40	0	1.46	8.31E-66
218278_at	LOC649169	0	-1.33	4.96E-11
218281_at	MRPL48	0	1.07	<1.00E-120
218282_at	EDEM2	2	2.43	8.24E-78
218283_at	SS18L2	0	-1.08	2.47E-10
218284_at	SMAD3	0	1.49	2.92E-49
218285_s_at	BDH2	0		N.S.
218286_s_at	RNF7	0		N.S.
218287_s_at	EIF2C1	0	-1.14	8.07E-18
218288_s_at	CCDC90B	0	1.36	3.33E-58
218289_s_at	UBA5	0	1.37	3.61E-52
218290_at	PLEKHJ1	0	-1.06	1.46E-02
218291_at	ROBLD3	0		N.S.
218294_s_at	NUP50	0	-1.10	6.42E-04
218297_at	C10orf97	0		N.S.
218298_s_at	C14orf159	0	1.13	<1.00E-120
218300_at	C16orf53	0	-1.15	2.65E-20
218301_at	RNPEPL1	0	1.19	5.34E-11
218302_at	PSENEN	0	1.27	1.41E-36
218304_s_at	OSBPL11	0	-1.34	5.84E-47
218305_at	IPO4	0	-1.56	6.00E-66
218306_s_at	HERC1	0	-1.07	6.63E-05
218307_at	RSAD1	0	-1.33	6.74E-41
218308_at	TACC3	0	-1.36	2.02E-34
218310_at	RABGEF1	0	1.10	<1.00E-120
218311_at	MAP4K3	0		N.S.
218314_s_at	C11orf57	0	1.09	<1.00E-120
218315_s_at	CDK5RAP1	0	-1.06	6.60E-05
218316_at	TIMM9	0	1.24	6.50E-45
218318_s_at	NLK	0	1.14	1.11E-07
218319_at	PELI1	0	1.10	2.26E-12
218320_s_at	NDUFB11	0	1.04	2.82E-03
218322_s_at	ACSL5	0	1.07	1.61E-03
218324_s_at	SPATS2	0	1.17	1.74E-20
218326_s_at	LGR4	0	-1.44	4.81E-11
218327_s_at	SNAP29	0	-1.09	5.01E-04
218328_at	COQ4	0	1.19	4.03E-22
218330_s_at	NAV2	0	1.65	1.54E-32
218331_s_at	C10orf18	0		N.S.

218333_at	DERL2	1	2.39	7.47E-83
218334_at	THOC7	0	-1.09	1.42E-06
218336_at	PFDN2	0	-1.14	1.19E-26
218337_at	FAM160B2	0	-1.09	2.53E-03
218339_at	MRPL22	0	-1.10	<1.00E-120
218340_s_at	UBA6	0		N.S.
218341_at	PPCS	0	1.12	2.23E-06
218342_s_at	ERMP1	0	-1.28	7.88E-27
218343_s_at	GTF3C3	0	-1.14	2.01E-22
218344_s_at	RCOR3	0	1.18	4.90E-23
218346_s_at	SESN1	0		N.S.
218347_at	TYW1	0	1.12	4.64E-18
218348_s_at	ZC3H7A	0	1.28	1.21E-49
218349_s_at	ZWILCH	0	-1.34	1.33E-39
218350_s_at	GMNN	0	-1.74	6.54E-96
218351_at	COMMD8	0		N.S.
218352_at	RCBTB1	0	1.13	7.15E-08
218354_at	TRAPPC2L	0	-1.26	1.72E-26
218355_at	KIF4A	0		N.S.
218357_s_at	TIMM8B	0	-1.40	2.67E-59
218358_at	CRELD2	2	3.02	1.20E-69
218361_at	GOLPH3L	0	1.74	8.81E-77
218362_s_at	DIS3	0		N.S.
218363_at	EXD2	0	1.10	4.71E-06
218364_at	LRRFIP2	0	1.51	3.33E-61
218365_s_at	DARS2	0	-1.37	1.42E-34
218370_s_at	S100PBP	0	1.08	1.88E-07
218372_at	MED9	0	-1.24	<1.00E-120
218373_at	AKTIP	0	1.39	5.64E-44
218374_s_at	C12orf4	0		N.S.
218375_at	NUDT9	0	-1.28	2.26E-36
218376_s_at	MICAL1	0	1.18	1.35E-21
218377_s_at	RWDD2B	0	1.18	1.28E-19
218378_s_at	PRKRIP1	0	1.18	1.67E-09
218379_at	RBM7	0		N.S.
218381_s_at	U2AF2	0	-1.46	4.16E-41
218383_at	HAUS4	0		N.S.
218384_at	CARHSP1	0	-1.16	<1.00E-120
218385_at	MRPS18A	0	-1.09	2.60E-07
218388_at	PGLS	0	1.05	2.83E-03
218389_s_at	APH1A	0		N.S.
218390_s_at	C10orf84	0		N.S.
218391_at	SNF8	0		N.S.
218393_s_at	SMU1	0		N.S.
218394_at	ROGDI	0		N.S.
218395_at	ACTR6	0	1.07	1.54E-05
218396_at	VPS13C	0	1.22	1.23E-21
218397_at	FANCL	0	-1.24	6.50E-28
218399_s_at	CDCA4	0	-1.32	6.85E-50
218400_at	OAS3	0		N.S.
218401_s_at	ZNF281	0	-1.17	<1.00E-120
218403_at	TRIAP1	0	-1.21	6.40E-30
218404_at	SNX10	0	-1.18	8.41E-27
218405_at	ABT1	0	-1.19	3.14E-19

Supplemental Table 1

218408_at	TIMM10	0	-1.06	4.29E-02
218409_s_at	DNAJC1	0	2.00	4.49E-54
218411_s_at	MBIP	0		N.S.
218412_s_at	GTF2IRD1	0	-1.23	<1.00E-120
218414_s_at	NDE1	0	1.10	4.33E-06
218415_at	VPS33B	0	-1.09	1.85E-04
218419_s_at	TMUB2	0	1.50	1.37E-43
218420_s_at	C13orf23	0	1.39	9.85E-58
218421_at	CERK	0		N.S.
218422_s_at	RBM26	0	-1.08	6.38E-06
218424_s_at	STEAP3	0		N.S.
218426_s_at	RNF216	0	1.08	7.01E-06
218427_at	SDCCAG3	0	-1.24	1.49E-08
218428_s_at	REV1	0		N.S.
218429_s_at	C19orf66	0		N.S.
218430_s_at	RFX7	0		N.S.
218431_at	C14orf133	0	1.26	1.11E-35
218432_at	FBXO3	0	1.33	3.65E-29
218433_at	PANK3	0	-1.73	1.55E-35
218434_s_at	AACS	0	1.11	<1.00E-120
218435_at	DNAJC15	0	1.17	1.55E-30
218436_at	SIL1	4	1.88	8.89E-71
218437_s_at	LZTFL1	0	2.51	6.19E-51
218439_s_at	COMMD10	0		N.S.
218440_at	MCCC1	0	1.16	3.30E-28
218441_s_at	RPAP1	0	-1.24	1.91E-34
218443_s_at	DAZAP1	0	-1.14	1.89E-20
218444_at	ALG12	0	1.81	1.35E-45
218446_s_at	FAM18B	0	1.51	3.99E-32
218447_at	C16orf61	0	-1.24	1.06E-36
218448_at	C20orf11	0	-1.12	<1.00E-120
218449_at	UFSP2	0	1.35	6.51E-51
218452_at	SMARCAL1	0		N.S.
218455_at	NFS1	0	-1.27	2.41E-22
218456_at	CAPRIN2	0	1.09	1.11E-06
218457_s_at	DNMT3A	0	-1.19	1.50E-12
218458_at	GMCL1	0	-1.10	1.71E-03
218459_at	TOR3A	0		N.S.
218460_at	HEATR2	0	-1.56	6.20E-86
218461_at	GPN3	0	-1.29	1.25E-52
218462_at	BXDC5	0		N.S.
218463_s_at	MUS81	0	1.09	<1.00E-120
218464_s_at	C17orf63	0		N.S.
218465_at	TMEM33	0		N.S.
218466_at	TBC1D17	0	1.29	1.86E-18
218467_at	PSMG2	0	1.04	6.80E-03
218470_at	YARS2	0	-1.21	2.98E-19
218471_s_at	BBS1	0	1.32	6.35E-40
218473_s_at	GLT25D1	0	-1.63	2.29E-58
218474_s_at	KCTD5	0		N.S.
218476_at	POMT1	1	1.16	7.24E-06
218477_at	TMEM14A	0	-1.13	7.52E-13
218478_s_at	ZCCHC8	0	1.49	6.39E-53
218479_s_at	XPO4	0	-1.17	9.21E-25

Supplemental Table 1

218480_at	AGBL5	0		N.S.
218481_at	EXOSC5	0	-1.09	8.52E-08
218482_at	ENY2	0	-1.24	5.39E-48
218483_s_at	C11orf60	0	1.18	<1.00E-120
218487_at	ALAD	0		N.S.
218488_at	EIF2B3	0	-1.11	7.45E-11
218490_s_at	ZNF302	0	1.22	7.00E-38
218491_s_at	THYN1	0		N.S.
218492_s_at	THAP7	0	-1.18	1.19E-18
218493_at	SNRNP25	0	-1.61	6.01E-72
218494_s_at	SLC2A4RG	0	-1.18	<1.00E-120
218495_at	UXT	0	1.12	1.30E-27
218496_at	RNASEH1	0	1.09	5.18E-04
218498_s_at	ERO1L	6	1.09	3.49E-02
218499_at	RP6-213H19.1	0	-1.11	<1.00E-120
218500_at	C8orf55	0	-1.69	6.42E-33
218501_at	ARHGEF3	0		N.S.
218503_at	KIAA1797	0	1.20	6.55E-35
218507_at	C7orf68	0	-1.44	1.05E-28
218508_at	DCP1A	0	1.08	1.13E-06
218511_s_at	PNPO	0	1.07	9.60E-04
218512_at	WDR12	0	-1.61	2.04E-90
218513_at	C4orf43	0	-1.34	1.58E-07
218514_at	C17orf71	0	-1.29	9.63E-63
218515_at	C21orf66	0	-1.25	2.25E-26
218516_s_at	IMPAD1	0		N.S.
218517_at	PHF17	0	1.06	5.51E-03
218518_at	FAM13B	0	1.37	4.93E-53
218519_at	SLC35A5	0	1.55	1.24E-87
218520_at	TBK1	0	-1.07	4.37E-06
218521_s_at	UBE2W	0		N.S.
218522_s_at	MAP1S	0	-1.10	6.67E-07
218524_at	E4F1	0	-1.13	<1.00E-120
218525_s_at	HIF1AN	0		N.S.
218526_s_at	RANGRF	0	-1.08	7.52E-13
218527_at	APTX	0	-1.09	1.11E-10
218528_s_at	RNF38	0	1.13	2.60E-08
218529_at	CD320	0	-1.60	2.54E-49
218530_at	FHOD1	0	-1.47	1.71E-55
218531_at	TMEM134	0		N.S.
218532_s_at	FAM134B	0	1.44	1.50E-12
218533_s_at	UCKL1	0		N.S.
218534_s_at	AGGF1	0		N.S.
218535_s_at	RIOK2	0	-1.06	4.11E-03
218536_at	MRS2	0	-1.24	3.09E-34
218537_at	HCFC1R1	0		N.S.
218539_at	FBXO34	0	1.06	1.21E-02
218542_at	CEP55	0	-1.15	1.48E-28
218543_s_at	PARP12	0	1.19	<1.00E-120
218544_s_at	RCL1	0	-1.36	1.16E-45
218545_at	CCDC91	0	1.39	3.28E-50
218547_at	DHDDS	0	1.13	5.27E-12
218549_s_at	FAM82B	0	1.07	1.05E-11
218550_s_at	LRRC20	0	-1.94	1.26E-62

Supplemental Table 1

218552_at	ECHDC2	0	1.09	3.64E-03
218554_s_at	ASH1L	0		N.S.
218555_at	ANAPC2	0	-1.24	6.40E-29
218556_at	ORMDL2	0	-1.14	<1.00E-120
218557_at	NIT2	0	-1.13	1.23E-29
218558_s_at	MRPL39	0	-1.13	1.61E-21
218561_s_at	LYRM4	0	-1.16	1.33E-34
218562_s_at	TMEM57	0	1.64	3.61E-57
218563_at	NDUFA3	0	-1.22	6.72E-30
218564_at	RFWD3	0	-1.46	4.55E-49
218565_at	C9orf114	0	-1.49	3.01E-49
218566_s_at	CHORDC1	0	-1.69	1.77E-81
218568_at	AGK	0	-1.05	3.08E-02
218570_at	KBTBD4	0	-1.43	1.05E-47
218571_s_at	CHMP4A	0	1.13	9.04E-21
218573_at	MAGEH1	0	-1.12	3.10E-04
218574_s_at	LMCD1	0		N.S.
218575_at	ANAPC1	0	-1.20	5.36E-33
218576_s_at	DUSP12	0	1.31	1.42E-39
218577_at	LRRC40	0	-1.16	<1.00E-120
218578_at	CDC73	0		N.S.
218579_s_at	DHX35	0		N.S.
218581_at	ABHD4	0	1.42	3.55E-35
218582_at	MARCH5	0		N.S.
218583_s_at	DCUN1D1	0	-1.13	3.86E-24
218584_at	TCTN1	0	1.31	4.17E-18
218585_s_at	DTL	0	-1.78	3.25E-74
218586_at	C20orf20	0	-1.28	4.74E-35
218587_s_at	KTELC1	0	1.34	2.53E-40
218588_s_at	FAM114A2	0	1.12	<1.00E-120
218590_at	C10orf2	1	-1.61	3.13E-58
218592_s_at	CECR5	0	-1.33	1.91E-52
218593_at	RBM28	0	-1.36	2.98E-47
218594_at	HEATR1	0	-1.47	1.85E-56
218596_at	TBC1D13	0		N.S.
218597_s_at	CISD1	0	-1.22	4.01E-48
218598_at	RINT1	0		N.S.
218599_at	REC8	0	-1.36	6.71E-33
218600_at	LIMD2	0	-1.17	3.69E-11
218602_s_at	HAUS6	0	-1.30	<1.00E-120
218603_at	HECA	0	1.29	4.74E-38
218604_at	LEMD3	0		N.S.
218605_at	TFB2M	0	-1.23	1.21E-23
218606_at	ZDHHC7	0		N.S.
218607_s_at	SDAD1	0		N.S.
218608_at	ATP13A2	0	-1.13	8.97E-04
218609_s_at	NUDT2	0	1.21	1.17E-18
218610_s_at	CPPED1	0		N.S.
218611_at	IER5	0	-1.06	1.78E-05
218612_s_at	TSSC4	0		N.S.
218614_at	C12orf35	0	1.21	4.56E-29
218615_s_at	TMEM39A	0	2.63	1.34E-82
218616_at	INTS12	0		N.S.
218617_at	TRIT1	0	-1.14	7.64E-27

Supplemental Table 1

218618_s_at	FNDC3B	0	1.36	8.64E-25
218619_s_at	SUV39H1	0	-1.27	1.02E-40
218620_s_at	HEMK1	0		N.S.
218622_at	NUP37	0	-1.14	6.81E-41
218624_s_at	MGC2752	0		N.S.
218626_at	EIF4ENIF1	0	-1.07	4.83E-08
218627_at	DRAM	0	1.18	1.53E-23
218628_at	CCDC53	0	1.21	7.16E-36
218630_at	MKS1	0	1.08	5.10E-03
218631_at	AVPI1	0		N.S.
218632_at	HECTD3	0	-1.07	7.12E-05
218634_at	PHLDA3	0	1.23	6.47E-22
218636_s_at	MAN1B1	0		N.S.
218637_at	IMPACT	78	1.43	1.86E-18
218639_s_at	ZXDC	0	1.29	<1.00E-120
218640_s_at	PLEKHF2	0		N.S.
218641_at	LOC65998	0		N.S.
218642_s_at	CHCHD7	0	-1.15	8.31E-22
218643_s_at	CRIPT	0	-1.11	3.69E-10
218645_at	ZNF277	0	1.25	8.42E-23
218646_at	C4orf27	0	-1.14	1.92E-28
218647_s_at	YRDC	0	-1.25	2.91E-31
218648_at	CRTC3	0	1.08	2.94E-08
218650_at	DGCR8	0	-1.18	1.15E-22
218652_s_at	PIGG	0	1.20	3.22E-34
218653_at	SLC25A15	0	-1.75	1.11E-73
218654_s_at	MRPS33	0	-1.07	3.63E-06
218655_s_at	CCDC49	0		N.S.
218656_s_at	LHFP	0		N.S.
218658_s_at	ACTR8	0	1.14	5.29E-07
218659_at	ASXL2	0	1.15	8.35E-27
218661_at	NAT15	0	1.11	3.19E-04
218663_at	NCAPG	0	-1.23	7.18E-41
218664_at	MECR	0	-1.09	3.27E-05
218666_s_at	STX17	0		N.S.
218667_at	PJA1	0	1.31	1.96E-25
218669_at	RAP2C	0	1.14	1.63E-29
218670_at	PUS1	0	-1.30	1.44E-35
218671_s_at	ATPIF1	0	1.06	9.85E-11
218672_at	SCNM1	0	1.15	8.51E-24
218673_s_at	ATG7	4	-1.21	2.60E-18
218674_at	C5orf44	0		N.S.
218676_s_at	PCTP	0	1.10	9.48E-11
218679_s_at	VPS28	0	1.22	1.17E-39
218681_s_at	SDF2L1	1	3.91	4.37E-79
218682_s_at	SLC4A1AP	0		N.S.
218683_at	PTBP2	0	-1.21	1.50E-11
218684_at	LRRC8D	0	1.34	1.31E-30
218685_s_at	SMUG1	0	-1.11	3.64E-04
218688_at	DAK	0		N.S.
218689_at	FANCF	0	1.11	6.42E-09
218692_at	GOLSYN	0	-1.27	<1.00E-120
218694_at	ARMCX1	0	1.41	1.62E-08
218696_at	EIF2AK3	6	1.63	8.27E-56

Supplemental Table 1

218698_at	APIP	0		N.S.
218699_at	RAB7L1	0	-1.05	1.02E-02
218701_at	LACTB2	0	1.26	1.93E-23
218703_at	SEC22A	1	1.28	2.93E-19
218705_s_at	SNX24	0	1.21	2.39E-08
218706_s_at	GRAMD3	0		N.S.
218708_at	NXT1	0	-1.38	5.37E-49
218709_s_at	IFT52	0	1.14	1.06E-22
218710_at	TTC27	0	-1.18	<1.00E-120
218712_at	C1orf109	0		N.S.
218713_at	NARG2	0	1.05	1.74E-03
218715_at	UTP6	0	-1.28	2.48E-22
218719_s_at	GINS3	0	-1.82	2.95E-64
218721_s_at	C1orf27	0		N.S.
218722_s_at	CCDC51	0	-1.28	2.70E-36
218723_s_at	C13orf15	0	-2.06	8.98E-56
218725_at	SLC25A22	0	-1.28	<1.00E-120
218726_at	HJURP	0		N.S.
218728_s_at	CNIH4	0	1.28	2.68E-48
218729_at	LXN	0		N.S.
218732_at	PTRH2	0	-1.34	1.66E-53
218733_at	MSL2	0	1.17	<1.00E-120
218735_s_at	ZNF544	0	-1.07	2.91E-04
218738_s_at	RNF138	0	-1.23	6.16E-34
218739_at	ABHD5	0	-1.09	1.76E-02
218740_s_at	CDK5RAP3	0	1.18	5.26E-33
218741_at	CENPM	0	-1.30	3.33E-26
218742_at	NARFL	0		N.S.
218743_at	CHMP6	0	-1.37	7.72E-40
218746_at	TAPBPL	0		N.S.
218748_s_at	EXOC5	0	-1.24	3.47E-10
218751_s_at	FBXW7	0	1.17	<1.00E-120
218752_at	ZMAT5	0	1.18	5.57E-11
218753_at	XKR8	0		N.S.
218754_at	NOL9	0	-1.19	7.24E-25
218755_at	KIF20A	0		N.S.
218756_s_at	DHRS11	0		N.S.
218757_s_at	UPF3B	0		N.S.
218758_s_at	RRP1	0	-1.55	5.01E-46
218759_at	DVL2	0	-1.44	1.73E-20
218760_at	COQ6	0	1.17	8.48E-09
218761_at	RNF111	0	1.09	3.28E-09
218762_at	ZNF574	0	-1.25	<1.00E-120
218763_at	STX18	0		N.S.
218764_at	PRKCH	0	1.23	2.63E-08
218766_s_at	WARS2	0		N.S.
218767_at	REXO4	0	-1.12	<1.00E-120
218768_at	NUP107	0	-1.43	5.34E-96
218769_s_at	ANKRA2	0	1.51	6.35E-48
218770_s_at	TMEM39B	0	1.35	1.36E-58
218771_at	PANK4	0	-1.32	8.90E-35
218774_at	DCPS	0	-1.07	7.94E-08
218776_s_at	TMEM62	0	1.29	1.06E-22
218777_at	REEP4	0	1.27	<1.00E-120

Supplemental Table 1

218781_at	SMC6	0	1.25	2.31E-31
218782_s_at	ATAD2	0	-1.74	1.21E-61
218785_s_at	RABL5	0	1.19	4.98E-06
218786_at	NT5DC3	0		N.S.
218788_s_at	SMYD3	0	1.12	<1.00E-120
218789_s_at	C11orf71	0	-1.29	8.18E-35
218791_s_at	C15orf29	0	1.10	7.52E-13
218794_s_at	TXNL4B	0	1.29	1.06E-39
218795_at	ACP6	0	1.26	2.02E-25
218797_s_at	SIRT7	0		N.S.
218798_at	KRI1	0	-1.15	3.08E-05
218799_at	GPN2	0		N.S.
218800_at	SRD5A3	1	1.42	8.39E-09
218801_at	UGCGL2	0	1.22	2.72E-24
218802_at	CCDC109B	0	1.08	1.71E-03
218803_at	CHFR	0		N.S.
218809_at	PANK2	0	-1.11	1.09E-22
218810_at	ZC3H12A	0		N.S.
218812_s_at	ORAI2	0	-1.08	1.27E-02
218813_s_at	SH3GLB2	0	-1.12	1.86E-05
218817_at	SPCS3	0	1.49	1.63E-64
218823_s_at	KCTD9	0		N.S.
218826_at	SLC35F2	0	-1.25	6.04E-43
218827_s_at	CEP192	0		N.S.
218830_at	RPL26L1	0	-1.19	6.39E-45
218833_at	ZAK	0		N.S.
218836_at	RPP21	0	-1.15	5.64E-11
218837_s_at	UBE2D4	0		N.S.
218838_s_at	TTC31	0	1.14	2.84E-20
218840_s_at	NADSYN1	0		N.S.
218841_at	ASB8	0	1.26	4.75E-24
218842_at	RPAP3	0		N.S.
218844_at	ACSF2	0		N.S.
218845_at	DUSP22	0	1.31	3.49E-49
218846_at	MED23	0	1.17	1.31E-09
218848_at	THOC6	0		N.S.
218850_s_at	LIMD1	0		N.S.
218851_s_at	WDR33	0		N.S.
218852_at	PPP2R3C	0	1.25	7.41E-47
218853_s_at	MOSPD1	0	1.52	8.31E-70
218854_at	DSE	0	-1.15	8.78E-37
218855_at	GPR175	0	1.45	1.19E-51
218858_at	DEPDC6	0	4.19	1.22E-49
218859_s_at	ESF1	0	-1.53	2.02E-49
218860_at	NOC4L	0	-1.52	8.02E-47
218861_at	RNF25	0		N.S.
218866_s_at	POLR3K	0	-1.81	2.44E-74
218867_s_at	C12orf49	0		N.S.
218868_at	ACTR3B	0	-1.43	1.23E-37
218869_at	MLYCD	0		N.S.
218870_at	ARHGAP15	0	1.33	1.03E-51
218872_at	TESC	0	-1.12	5.26E-06
218873_at	GON4L	0		N.S.
218874_s_at	C6orf134	0		N.S.

Supplemental Table 1

218875_s_at	FBXO5	0	-1.42	3.83E-25
218877_s_at	TRMT11	0	-1.20	5.63E-40
218878_s_at	SIRT1	2	1.09	5.98E-03
218879_s_at	MTHFSD	0		N.S.
218882_s_at	WDR3	0	-1.75	3.04E-73
218883_s_at	MLF1IP	0		N.S.
218884_s_at	GUF1	0	-1.38	3.74E-37
218886_at	PAK1IP1	0	-1.79	4.57E-43
218887_at	MRPL2	0		N.S.
218888_s_at	NETO2	0	1.20	3.99E-05
218889_at	NOC3L	0	-1.27	1.92E-41
218893_at	ISOC2	0	1.39	2.61E-54
218894_s_at	MAGOHB	0		N.S.
218895_at	GPATCH3	0		N.S.
218896_s_at	C17orf85	0		N.S.
218897_at	TMEM177	0	-1.63	1.05E-64
218898_at	FAM57A	0	-1.52	2.12E-47
218900_at	CNNM4	0		N.S.
218902_at	NOTCH1	0		N.S.
218903_s_at	OBFC2B	0	-1.14	7.52E-13
218904_s_at	C9orf40	0	-1.49	3.18E-37
218905_at	INTS8	0		N.S.
218907_s_at	LRRC61	0		N.S.
218908_at	ASPSCR1	0		N.S.
218909_at	RPS6KC1	0	1.33	6.38E-41
218911_at	YEATS4	0		N.S.
218912_at	GCC1	0	1.57	2.33E-51
218913_s_at	GMIP	0	-1.32	6.25E-36
218914_at	C1orf66	0	1.28	7.52E-13
218916_at	ZNF768	0	1.26	6.98E-18
218919_at	ZFAND1	0	1.70	6.49E-69
218920_at	FLJ10404	0		N.S.
218922_s_at	LASS4	0		N.S.
218924_s_at	CTBS	0	1.52	8.94E-48
218926_at	MYNN	0	1.08	9.93E-11
218927_s_at	CHST12	0	1.31	7.32E-27
218928_s_at	SLC37A1	0	1.28	1.27E-22
218929_at	CDKN2AIP	0	-1.23	8.10E-39
218930_s_at	TMEM106B	0	1.13	7.64E-19
218932_at	ZNHIT6	0	-1.12	1.82E-18
218935_at	EHD3	0		N.S.
218936_s_at	CCDC59	0	1.11	7.52E-13
218937_at	ZNF434	0		N.S.
218938_at	FBXL15	0		N.S.
218940_at	C14orf138	0	-1.08	7.64E-03
218942_at	PIP4K2C	0		N.S.
218943_s_at	DDX58	0		N.S.
218945_at	C16orf68	0	1.29	4.30E-36
218946_at	NFU1	0	1.11	2.81E-31
218947_s_at	MTPAP	0	-1.54	6.72E-67
218949_s_at	QRSL1	0		N.S.
218951_s_at	PLCXD1	0	1.26	2.26E-12
218953_s_at	PCYOX1L	0	-1.40	2.91E-49
218954_s_at	BRF2	0	-1.16	2.04E-07

218956_s_at	PTCD1	0	-1.32	1.04E-20
218957_s_at	PAAF1	0	-1.08	2.29E-09
218958_at	C19orf60	0		N.S.
218961_s_at	PNKP	0	-1.09	1.83E-06
218962_s_at	TMEM168	0	1.14	<1.00E-120
218964_at	ARID3B	0	1.16	7.52E-13
218965_s_at	TUT1	0		N.S.
218966_at	MYO5C	0	1.11	1.11E-03
218967_s_at	PTER	0		N.S.
218968_s_at	ZFP64	0	1.08	4.82E-05
218969_at	Magmas	0	-1.18	2.04E-21
218970_s_at	CUTC	0	-1.32	2.88E-44
218971_s_at	WDR91	0	-1.16	3.76E-12
218972_at	TTC17	0	2.20	3.31E-79
218973_at	EFTUD1	0	-1.22	5.71E-35
218974_at	SOBP	0		N.S.
218976_at	DNAJC12	0	3.36	4.10E-45
218977_s_at	TRNAU1AP	0	-1.14	<1.00E-120
218979_at	RMI1	0	-1.46	1.30E-64
218981_at	ACN9	0	-1.16	5.65E-05
218982_s_at	MRPS17	0	-1.99	6.95E-100
218983_at	C1RL	0	1.20	<1.00E-120
218984_at	PUS7	0	-1.72	2.48E-77
218985_at	SLC2A8	0	1.32	<1.00E-120
218986_s_at	DDX60	0	1.31	1.27E-17
218987_at	ATF7IP	0		N.S.
218988_at	SLC35E3	0	1.32	3.85E-26
218991_at	HEATR6	0	-1.18	1.18E-04
218992_at	C9orf46	0	1.34	4.00E-60
218993_at	RNMTL1	0	-1.44	7.54E-54
218994_s_at	STAG3L4	0	1.34	1.35E-11
218996_at	TFPT	0		N.S.
218997_at	POLR1E	0	-1.26	7.36E-37
218998_at	C9orf6	0	1.32	1.32E-30
218999_at	TMEM140	0	2.70	5.42E-71
219001_s_at	WDR32	0	1.12	2.26E-12
219002_at	FASTKD1	0	-1.49	6.72E-78
219003_s_at	MANEA	0	-1.27	1.50E-12
219004_s_at	C21orf45	0	-1.30	1.25E-49
219006_at	NDUFAF4	0	-1.46	1.78E-60
219007_at	NUP43	0	-1.06	6.95E-03
219009_at	C14orf93	0		N.S.
219010_at	C1orf106	0	-1.25	1.17E-17
219012_s_at	C11orf30	0		N.S.
219013_at	GALNT11	0	-1.44	2.47E-19
219014_at	PLAC8	0		N.S.
219016_at	FASTKD5	0	-1.25	1.63E-33
219020_at	HS1BP3	0	1.21	<1.00E-120
219021_at	RNF121	0		N.S.
219022_at	C12orf43	0	-1.25	8.27E-29
219023_at	C4orf16	0	1.32	9.60E-27
219027_s_at	MYO9A	0	1.13	5.49E-07
219029_at	C5orf28	0	2.25	9.83E-92
219030_at	TPRKB	0	-1.04	3.58E-04

Supplemental Table 1

219031_s_at	NIP7	0	-1.40	3.93E-27
219033_at	PARP8	0	1.53	1.02E-54
219034_at	PARP16	0		N.S.
219035_s_at	RNF34	0	1.05	1.28E-03
219036_at	CEP70	0	1.65	2.71E-31
219040_at	CORO7	0		N.S.
219041_s_at	REPIN1	0	-1.32	2.15E-53
219043_s_at	LOC285359	0	-1.37	2.28E-67
219045_at	RHOF	0		N.S.
219047_s_at	ZNF668	0	-1.51	5.45E-23
219048_at	PIGN	0	1.12	3.54E-05
219052_at	HPS6	1	-1.28	<1.00E-120
219053_s_at	VPS37C	0	1.22	3.15E-30
219055_at	SRBD1	0	1.12	2.65E-09
219060_at	WDYHV1	0	-1.08	8.20E-11
219061_s_at	LAGE3	0		N.S.
219062_s_at	ZCCHC2	0	-1.25	4.81E-29
219063_at	C1orf35	0	-1.38	1.26E-42
219065_s_at	MEMO1	0	-1.05	4.10E-05
219066_at	PPCDC	0	1.39	3.87E-46
219067_s_at	NSMCE4A	0	-1.16	1.73E-37
219069_at	ANKRD49	0	1.05	5.57E-05
219070_s_at	MOSPD3	0		N.S.
219072_at	BCL7C	0		N.S.
219074_at	TMEM184C	0	1.17	4.74E-05
219076_s_at	PXMP2	0	-1.07	4.99E-02
219077_s_at	WWOX	0	1.25	4.40E-20
219078_at	GPATCH2	0		N.S.
219079_at	CYB5R4	0	1.21	5.31E-32
219080_s_at	CTPS2	0	-1.26	3.05E-39
219084_at	NSD1	0		N.S.
219086_at	ZNF839	0		N.S.
219089_s_at	ZNF576	0	-1.20	<1.00E-120
219092_s_at	IPPK	0	-1.14	9.13E-07
219096_at	ARMC7	0		N.S.
219098_at	MYBBP1A	0	-1.61	9.65E-55
219099_at	C12orf5	0	-1.09	6.43E-10
219100_at	OBFC1	0	1.08	2.15E-05
219104_at	RNF141	0		N.S.
219109_at	SPAG16	0		N.S.
219110_at	GAR1	0	-1.49	3.18E-68
219111_s_at	DDX54	0		N.S.
219112_at	RAPGEF6	0	-1.21	2.48E-25
219116_s_at	DCUN1D2	0		N.S.
219117_s_at	FKBP11	1	2.03	2.15E-49
219119_at	LSM8	0		N.S.
219120_at	C2orf44	0	-1.62	2.14E-48
219122_s_at	THG1L	0	1.08	6.75E-09
219123_at	ZNF232	0	-1.70	7.21E-66
219124_at	C8orf41	0	-1.25	1.72E-35
219125_s_at	RAG1AP1	0	1.40	9.41E-34
219126_at	PHF10	0	-1.23	3.23E-32
219128_at	C2orf42	0	1.26	3.31E-33
219129_s_at	SAP30L	0		N.S.

Supplemental Table 1

219130_at	CCDC76	0		N.S.
219131_at	UBIAD1	0	-1.30	2.35E-39
219133_at	OXSM	0	1.13	3.64E-23
219137_s_at	MFF	0	1.12	4.76E-20
219143_s_at	RPP25	0	-2.18	7.70E-67
219146_at	C17orf42	0	-1.14	2.13E-09
219147_s_at	C9orf95	0	1.64	1.88E-62
219148_at	PBK	0	-1.33	1.45E-67
219150_s_at	ADAP1	0	-1.24	1.56E-06
219155_at	PITPNC1	0	-1.17	5.27E-12
219156_at	SYNJ2BP	0	-1.22	1.39E-21
219157_at	KLHL2	0	1.10	2.75E-06
219158_s_at	NARG1	0	-1.65	5.91E-60
219159_s_at	SLAMF7	0	1.30	6.12E-25
219162_s_at	MRPL11	0	-1.14	1.02E-23
219163_at	ZNF562	0	-1.15	1.39E-08
219164_s_at	ATG2B	0	1.26	6.63E-21
219165_at	PDLIM2	0		N.S.
219166_at	C14orf104	0	-1.27	2.42E-09
219169_s_at	TFB1M	0	-1.05	1.01E-03
219174_at	IFT74	0	1.26	2.93E-28
219175_s_at	SLC41A3	0	1.21	<1.00E-120
219176_at	C2orf47	0		N.S.
219177_at	BXDC2	0	-2.05	5.34E-55
219178_at	QTRTD1	0	-1.40	9.42E-53
219180_s_at	PEX26	0	-1.10	4.16E-09
219187_at	FKBPL	0		N.S.
219189_at	FBXL6	0		N.S.
219190_s_at	EIF2C4	0	1.39	1.16E-07
219191_s_at	BIN2	0	1.51	1.66E-35
219192_at	UBAP2	0	-1.42	3.24E-70
219193_at	WDR70	0	1.09	1.27E-08
219198_at	GTF3C4	0	-1.46	1.38E-41
219199_at	AFF4	0		N.S.
219200_at	FASTKD3	0	-1.11	<1.00E-120
219201_s_at	TWSG1	0	-1.13	3.32E-06
219202_at	RHBDF2	0		N.S.
219203_at	FAM158A	0	1.29	2.20E-44
219205_at	SRR	0		N.S.
219207_at	EDC3	0	-1.21	<1.00E-120
219210_s_at	RAB8B	0	1.19	<1.00E-120
219211_at	USP18	0		N.S.
219212_at	HSPA14	0	-1.32	4.16E-48
219213_at	JAM2	0	1.24	6.80E-06
219214_s_at	NT5C	0	-1.13	4.13E-09
219215_s_at	SLC39A4	0	-1.54	6.19E-36
219216_at	ETAA1	0		N.S.
219217_at	NARS2	0	-1.17	5.39E-26
219219_at	TMEM160	0		N.S.
219221_at	ZBTB38	0	1.15	3.86E-10
219228_at	ZNF331	0	1.15	4.04E-06
219231_at	TGS1	0	-1.32	5.53E-31
219232_s_at	EGLN3	1	-1.82	4.64E-18
219235_s_at	PHACTR4	0	1.12	5.72E-11

219237_s_at	DNAJB14	0	1.07	6.61E-03
219238_at	PIGV	0	1.43	4.79E-34
219239_s_at	ZNF654	0		N.S.
219240_s_at	C10orf88	0		N.S.
219242_at	CEP63	0	1.24	4.00E-24
219244_s_at	MRPL46	0	-1.20	6.51E-33
219246_s_at	OGFOD2	0	1.27	1.20E-18
219248_at	THUMPD2	0	-1.44	4.18E-55
219252_s_at	GEMIN8	0		N.S.
219253_at	TMEM185B	0	-1.20	7.18E-26
219254_at	C17orf101	0		N.S.
219256_s_at	SH3TC1	0	1.71	1.34E-60
219258_at	TIPIN	0	-1.53	1.61E-66
219259_at	SEMA4A	0	1.76	2.25E-48
219260_s_at	C17orf81	0	-1.20	1.02E-20
219262_at	SUV39H2	0	-1.97	2.89E-47
219264_s_at	LOC100134089	0		N.S.
219266_at	ZNF350	0	1.20	<1.00E-120
219267_at	GLTP	0		N.S.
219269_at	HMBOX1	0	1.28	1.57E-24
219270_at	CHAC1	2	15.92	5.14E-91
219274_at	TSPAN12	0	1.25	7.52E-13
219275_at	PDCD5	0	-1.17	2.28E-30
219279_at	DOCK10	0	-1.14	2.08E-23
219280_at	BRWD1	0		N.S.
219281_at	MSRA	0	1.14	3.64E-20
219282_s_at	TRPV2	0		N.S.
219283_at	C1GALT1C1	0	-1.43	1.57E-60
219284_at	HSPBAP1	0		N.S.
219286_s_at	RBM15	0	-1.09	3.38E-19
219287_at	KCNMB4	0		N.S.
219288_at	C3orf14	0		N.S.
219289_at	HEATR3	0	-1.40	3.27E-56
219291_at	DTWD1	0		N.S.
219292_at	THAP1	0		N.S.
219293_s_at	OLA1	0		N.S.
219294_at	CENPQ	0	-1.23	<1.00E-120
219296_at	ZDHHC13	0	-1.11	<1.00E-120
219297_at	WDR44	0	1.15	7.52E-13
219298_at	ECHDC3	0		N.S.
219299_at	TRMT12	0	-1.20	<1.00E-120
219303_at	RNF219	0	-1.37	6.79E-32
219304_s_at	PDGFD	0		N.S.
219306_at	KIF15	0	-1.23	8.38E-37
219311_at	CEP76	0	-1.24	4.33E-26
219312_s_at	ZBTB10	0	1.41	4.21E-20
219317_at	POLI	0	1.31	8.39E-34
219320_at	MYO19	0	-1.20	1.02E-10
219321_at	MPP5	0	-1.19	<1.00E-120
219322_s_at	WDR8	0	-1.11	1.80E-03
219324_at	NOL12	0	-1.29	2.61E-41
219325_s_at	ELAC1	0		N.S.
219326_s_at	B3GNT2	0	1.29	<1.00E-120
219329_s_at	C2orf28	0	1.10	2.37E-24

Supplemental Table 1

219330_at	VANGL1	0	1.11	1.95E-06
219334_s_at	OBFC2A	0		N.S.
219335_at	ARMCX5	0	1.48	7.05E-67
219336_s_at	ASCC1	0	1.13	<1.00E-120
219337_at	C1orf159	0		N.S.
219338_s_at	LRRC49	0	1.36	1.40E-28
219342_at	CASD1	0		N.S.
219343_at	CDC37L1	0	1.16	1.14E-27
219345_at	BOLA1	0		N.S.
219347_at	NUDT15	0	-1.43	1.77E-38
219348_at	USE1	0	1.17	<1.00E-120
219349_s_at	EXOC2	0		N.S.
219350_s_at	DIABLO	8	-1.03	4.91E-02
219351_at	TRAPPC2	0	1.20	1.14E-34
219352_at	HERC6	0	1.53	4.89E-42
219353_at	NHLRC2	0		N.S.
219354_at	KLHL26	0	-1.29	2.26E-12
219357_at	GTPBP1	0	1.10	1.02E-03
219358_s_at	ADAP2	0		N.S.
219359_at	ATHL1	0	1.44	<1.00E-120
219361_s_at	AEN	0		N.S.
219363_s_at	MTERFD1	0	-1.36	1.59E-47
219366_at	AVEN	0	-1.25	7.28E-37
219368_at	NAP1L2	0		N.S.
219371_s_at	KLF2	0		N.S.
219372_at	IFT81	0		N.S.
219373_at	DPM3	0		N.S.
219374_s_at	ALG9	1	1.23	4.23E-41
219375_at	CEPT1	0	1.14	1.69E-28
219376_at	ZNF322B	0	1.10	5.53E-08
219378_at	NARG1L	0	-1.47	2.46E-44
219381_at	C5orf42	0		N.S.
219382_at	SERTAD3	0	1.23	6.02E-12
219384_s_at	ADAT1	0		N.S.
219387_at	CCDC88A	0	-1.31	<1.00E-120
219390_at	FKBP14	0	3.55	3.51E-73
219394_at	PGS1	0		N.S.
219397_at	COQ10B	0	1.17	5.23E-30
219398_at	CIDEC	0	1.27	5.04E-11
219400_at	CNTNAP1	0		N.S.
219401_at	XYLT2	0	-1.30	6.09E-07
219405_at	TRIM68	0	1.15	2.91E-20
219406_at	C1orf50	0	1.12	9.78E-12
219408_at	PRMT7	0	-1.65	3.96E-47
219409_at	SNIP1	0		N.S.
219410_at	TMEM45A	0	1.30	<1.00E-120
219411_at	ELMO3	0	-1.21	1.84E-10
219412_at	RAB38	0	1.58	1.59E-30
219413_at	ACBD4	0		N.S.
219417_s_at	C17orf59	0	1.57	5.83E-39
219420_s_at	C1orf163	0	-2.15	2.23E-61
219421_at	TTC33	0	1.20	1.31E-18
219424_at	EBI3	0	1.09	5.08E-07
219426_at	EIF2C3	0	1.12	5.75E-19

Supplemental Table 1

219428_s_at	PXMP4	0		N.S.
219429_at	FA2H	0	-1.47	1.91E-09
219431_at	ARHGAP10	0	1.11	4.78E-07
219433_at	BCOR	0		N.S.
219435_at	C17orf68	0	-1.22	<1.00E-120
219437_s_at	ANKRD11	0	1.62	2.61E-46
219439_at	C1GALT1	0	-1.19	1.32E-18
219441_s_at	LRRK1	0	-1.26	3.75E-22
219442_at	C16orf67	0	1.12	4.50E-05
219443_at	TASP1	0		N.S.
219444_at	BCORL1	0	-1.26	2.26E-12
219445_at	GLTSCR1	0		N.S.
219446_at	RIC8B	0		N.S.
219447_s_at	SLC35C2	0		N.S.
219449_s_at	TMEM70	0	-1.26	2.85E-49
219451_at	MSRB2	0		N.S.
219453_at	KLHL36	0		N.S.
219458_s_at	NSUN3	0	1.10	5.05E-03
219459_at	POLR3B	0	-1.11	2.26E-12
219460_s_at	TMEM127	0	1.20	<1.00E-120
219462_at	TMEM53	0	-1.24	1.65E-11
219467_at	GIN1	0	-1.11	2.96E-10
219471_at	C13orf18	0	-1.24	6.12E-21
219472_at	CENPO	0		N.S.
219473_at	GDAP2	0	1.18	1.28E-09
219477_s_at	THSD1	0	1.44	1.58E-08
219479_at	KDELC1	0		N.S.
219481_at	TTC13	0		N.S.
219483_s_at	PORCN	0	1.24	<1.00E-120
219484_at	HCFC2	0	1.13	1.05E-11
219485_s_at	PSMD10	1	1.15	1.65E-19
219486_at	DUS2L	0	-1.14	1.91E-04
219487_at	BBS10	0		N.S.
219489_s_at	NXN	0		N.S.
219490_s_at	DCLRE1B	0	-1.49	3.93E-40
219492_at	CHIC2	0	1.60	1.44E-72
219493_at	SHCBP1	0	1.14	1.90E-10
219494_at	RAD54B	1	-1.23	1.30E-20
219495_s_at	ZNF180	0	-1.23	2.76E-21
219496_at	ANKRD57	0		N.S.
219497_s_at	BCL11A	0	-1.64	3.08E-18
219499_at	SEC61A2	0		N.S.
219501_at	ENOX1	0	1.41	2.93E-11
219502_at	NEIL3	0	-1.14	9.03E-12
219504_s_at	RPAP2	0		N.S.
219505_at	CECR1	0	1.41	4.08E-21
219506_at	C1orf54	0	1.11	9.50E-06
219507_at	RSRC1	0	-1.24	2.13E-20
219512_at	DSN1	0		N.S.
219513_s_at	SH2D3A	0	1.20	7.30E-07
219515_at	PRDM10	0	-1.51	8.39E-50
219517_at	ELL3	0	-1.28	4.28E-19
219520_s_at	WWC3	0	1.59	1.03E-39
219522_at	FJX1	0	-2.42	7.77E-49

Supplemental Table 1

219526_at	C14orf169	0	-1.24	5.00E-42
219530_at	PALB2	0	-1.34	1.30E-41
219531_at	CEP72	0	-1.56	2.14E-43
219538_at	WDR5B	0		N.S.
219539_at	GEMIN6	0	-1.97	6.31E-64
219540_at	ZNF267	0	1.22	<1.00E-120
219541_at	LIME1	0	-1.22	6.75E-07
219543_at	PBLD	0	1.38	4.30E-33
219544_at	C13orf34	0	1.19	7.08E-31
219548_at	ZNF16	0	1.16	5.29E-07
219549_s_at	RTN3	2	1.62	4.98E-72
219551_at	EAF2	0		N.S.
219559_at	SLC17A9	0	1.32	1.29E-20
219560_at	C22orf29	0	-1.31	1.20E-11
219563_at	C14orf139	0		N.S.
219565_at	CYP20A1	0	1.49	8.49E-44
219567_s_at	DEM1	0		N.S.
219570_at	KIF16B	0		N.S.
219571_s_at	ZNF12	0	1.20	9.86E-23
219575_s_at	COG8	0	-1.14	1.41E-17
219576_at	MAP7D3	0	-1.20	9.78E-12
219577_s_at	ABCA7	0	1.14	5.17E-07
219581_at	TSEN2	0	-1.64	1.45E-35
219582_at	OGFRL1	0		N.S.
219583_s_at	SPATA7	0	1.36	<1.00E-120
219584_at	PLA1A	0	1.24	1.21E-22
219588_s_at	NCAPG2	0	-1.26	8.36E-29
219593_at	SLC15A3	0	1.08	7.13E-03
219594_at	NINJ2	0		N.S.
219595_at	ZNF26	0		N.S.
219596_at	THAP10	0	2.00	1.34E-55
219598_s_at	RWDD1	0	-1.06	2.76E-10
219600_s_at	TMEM50B	0	2.37	5.52E-76
219602_s_at	FAM38B	0	1.38	2.27E-28
219603_s_at	ZNF226	0		N.S.
219609_at	WDR25	0	1.89	4.03E-27
219613_s_at	SIRT6	0		N.S.
219617_at	C2orf34	0		N.S.
219618_at	IRAK4	0	1.33	2.11E-28
219622_at	RAB20	0	-1.15	6.89E-03
219624_at	BAG4	0	-1.31	1.26E-26
219625_s_at	COL4A3BP	1		N.S.
219627_at	ZNF767	0	1.36	3.25E-33
219628_at	ZMAT3	0	1.42	7.08E-45
219629_at	FAM118A	0		N.S.
219632_s_at	TRPV1	1	1.20	1.23E-08
219633_at	TTPAL	0	-1.10	4.86E-04
219634_at	CHST11	0	1.14	2.48E-20
219635_at	ZNF606	0	1.20	<1.00E-120
219636_s_at	ARMC9	0		N.S.
219640_at	CLDN15	0	1.25	2.56E-19
219641_at	DET1	0	1.18	1.79E-09
219644_at	CCDC41	0	-1.18	<1.00E-120
219646_at	DEF8	0	-1.39	7.28E-36

219648_at	MREG	0	-1.21	8.69E-43
219649_at	ALG6	2		N.S.
219650_at	ERCC6L	0	-2.03	1.42E-67
219653_at	LSM14B	0	-1.15	8.12E-11
219657_s_at	KLF3	0		N.S.
219662_at	C2orf49	0		N.S.
219665_at	NUDT18	0		N.S.
219667_s_at	BANK1	0	1.32	4.82E-05
219673_at	MCM9	0	-1.24	2.10E-24
219675_s_at	UXS1	0	-1.17	1.71E-21
219676_at	ZSCAN16	0	1.24	9.40E-23
219680_at	NLRX1	0		N.S.
219681_s_at	RAB11FIP1	0	-1.08	3.19E-03
219683_at	FZD3	0	-1.20	1.24E-10
219684_at	RTP4	0		N.S.
219688_at	BBS7	0	1.08	2.77E-02
219690_at	TMEM149	0	1.20	<1.00E-120
219691_at	SAMD9	0		N.S.
219696_at	DENND1B	0		N.S.
219698_s_at	METTL4	0	1.55	1.23E-60
219702_at	PLAC1	0	5.30	1.26E-50
219703_at	MNS1	0	-1.36	1.67E-21
219705_at	QSER1	0	-1.52	1.16E-21
219706_at	C20orf29	0		N.S.
219711_at	ZNF586	0		N.S.
219713_at	SHPK	0		N.S.
219715_s_at	TDP1	0	-1.37	5.66E-64
219716_at	APOL6	0	1.88	4.41E-56
219717_at	C4orf30	0	-1.25	1.06E-20
219718_at	FGGY	0	1.11	1.03E-02
219720_s_at	C14orf118	0		N.S.
219724_s_at	KIAA0748	0	1.36	2.23E-24
219731_at	FLJ34077	0	-1.12	2.41E-08
219733_s_at	SLC27A5	0	-1.32	<1.00E-120
219740_at	VASH2	0	-1.28	1.10E-25
219742_at	PRR7	0	-1.27	6.27E-08
219751_at	SETD6	0	-1.35	3.44E-23
219753_at	STAG3	0		N.S.
219754_at	RBM41	0	1.29	1.69E-22
219757_s_at	C14orf101	0	1.28	4.29E-30
219758_at	TTC26	0	-1.14	4.99E-05
219759_at	ERAP2	0	1.26	<1.00E-120
219762_s_at	RPL36	0		N.S.
219763_at	DENND1A	0	1.14	7.24E-06
219765_at	ZNF329	0	1.42	3.80E-25
219767_s_at	CRYZL1	0		N.S.
219770_at	GTDC1	0		N.S.
219774_at	CCDC93	0	-1.14	<1.00E-120
219777_at	GIMAP6	0		N.S.
219783_at	C2orf18	0		N.S.
219785_s_at	FBXO31	0	1.19	3.56E-27
219787_s_at	ECT2	0	-1.13	2.48E-11
219788_at	PILRA	0	1.35	1.36E-04
219793_at	SNX16	0		N.S.

Supplemental Table 1

219797_at	MGAT4A	0	-1.42	4.93E-07
219798_s_at	MEPCE	0	-1.08	8.33E-04
219800_s_at	THNSL1	0	-1.71	<1.00E-120
219801_at	ZNF34	0		N.S.
219805_at	CXorf56	0		N.S.
219806_s_at	C11orf75	0	1.39	2.70E-50
219809_at	WDR55	0	-1.17	<1.00E-120
219812_at	PVRIG	0	1.18	7.27E-25
219814_at	MBNL3	0		N.S.
219816_s_at	RBM23	0	-1.08	<1.00E-120
219817_at	C12orf47	0	1.19	3.54E-10
219818_s_at	GPATCH1	0		N.S.
219819_s_at	MRPS28	0	-1.47	1.48E-81
219821_s_at	GFOD1	0	1.11	7.65E-05
219822_at	MTRF1	0		N.S.
219828_at	C9orf86	0	-1.19	1.05E-11
219831_at	CDKL3	0	1.36	2.72E-19
219833_s_at	EFHC1	0	1.30	4.51E-27
219834_at	ALS2CR8	0	1.26	<1.00E-120
219838_at	TTC23	0		N.S.
219841_at	AICDA	0	-1.19	3.01E-12
219842_at	ARL15	0		N.S.
219843_at	IPP	0	-1.15	7.73E-07
219848_s_at	ZNF432	0	1.19	<1.00E-120
219849_at	ZNF671	0	1.13	2.63E-04
219854_at	ZNF14	0		N.S.
219858_s_at	MFSD6	0		N.S.
219860_at	LY6G5C	0	1.13	3.69E-02
219861_at	DNAJC17	0	-1.33	3.09E-43
219862_s_at	NARF	0		N.S.
219863_at	HERC5	0	1.18	9.73E-19
219865_at	HSPC157	0	1.27	3.53E-20
219868_s_at	ANKFY1	0		N.S.
219870_at	ATF7IP2	0	1.62	1.60E-57
219874_at	SLC12A8	0	1.26	1.07E-31
219876_s_at	GOLGA2L1	0		N.S.
219878_s_at	KLF13	0		N.S.
219885_at	SLFN12	0	-1.10	3.33E-04
219888_at	SPAG4	0		N.S.
219889_at	FRAT1	0	1.62	1.22E-35
219891_at	PGPEP1	0	1.26	7.91E-26
219892_at	TM6SF1	0	7.11	5.40E-97
219901_at	FGD6	0	1.30	1.41E-18
219904_at	ZSCAN5A	0		N.S.
219905_at	ERMAP	0	1.55	8.73E-35
219906_at	FLJ10213	0		N.S.
219910_at	FICD	0	2.80	7.16E-74
219911_s_at	LOC100134295	0	-1.35	2.61E-26
219913_s_at	CRNKL1	0	1.05	1.13E-05
219915_s_at	SLC16A10	0	-1.38	8.95E-11
219917_at	ZCCHC4	0		N.S.
219918_s_at	ASPM	0	-1.08	2.41E-11
219920_s_at	GMPPB	0	1.74	3.06E-46
219922_s_at	LTBP3	0	1.20	1.29E-04

Supplemental Table 1

219923_at	TRIM45	0	-1.20	3.31E-03
219924_s_at	ZMYM6	0	1.20	<1.00E-120
219929_s_at	ZFYVE21	0		N.S.
219931_s_at	KLHL12	0		N.S.
219933_at	GLRX2	0	-1.17	4.62E-25
219938_s_at	PSTPIP2	0		N.S.
219939_s_at	CSDE1	0	1.12	3.09E-28
219940_s_at	PCID2	0	-1.15	<1.00E-120
219941_at	TMEM19	0		N.S.
219944_at	CLIP4	0		N.S.
219947_at	CLEC4A	0	1.30	1.32E-08
219951_s_at	C20orf12	0		N.S.
219952_s_at	MCOLN1	0	1.38	<1.00E-120
219956_at	GALNT6	0	-1.27	1.90E-09
219957_at	RUFY2	0		N.S.
219959_at	MOCOS	0		N.S.
219960_s_at	UCHL5	0	-1.17	2.07E-31
219961_s_at	NCRNA00153	0	2.64	5.47E-77
219967_at	MRM1	0	-1.54	6.34E-30
219968_at	ZNF589	0	-1.29	6.63E-18
219969_at	CXorf15	0	-1.34	<1.00E-120
219971_at	IL21R	0	1.55	4.80E-50
219972_s_at	C14orf135	0	1.30	1.43E-24
219976_at	HOOK1	0	1.40	3.15E-21
219979_s_at	C11orf73	0	1.12	5.52E-08
219980_at	C4orf29	0	-1.13	4.86E-02
219982_s_at	SERF1A	0	-1.09	2.70E-04
219986_s_at	ACAD10	0	1.11	3.16E-03
219988_s_at	RNF220	0		N.S.
219990_at	E2F8	0	-2.07	1.26E-73
219994_at	APBB1IP	0		N.S.
219996_at	ASB7	0		N.S.
219997_s_at	COPS7B	0	-1.16	6.38E-08
219998_at	HSPC159	0	-1.74	6.55E-26
219999_at	MAN2A2	0		N.S.
220002_at	KIF26B	0	-1.36	1.50E-12
220007_at	METTL8	0	-1.14	<1.00E-120
220011_at	C1orf135	0	-1.25	4.09E-31
220012_at	ERO1LB	2	2.25	6.39E-75
220015_at	CASZ1	0		N.S.
220018_at	CBLL1	0		N.S.
220019_s_at	ZNF224	0	1.11	2.60E-02
220020_at	XPNPEP3	0	-1.14	9.04E-04
220028_at	ACVR2B	0		N.S.
220034_at	IRAK3	0		N.S.
220035_at	NUP210	0	-1.32	1.48E-20
220036_s_at	LMBR1L	0	1.22	<1.00E-120
220038_at	C8orf44	0	2.41	1.08E-42
220041_at	PIGZ	0		N.S.
220046_s_at	CCNL1	0		N.S.
220050_at	C9orf9	0	1.26	6.86E-20
220052_s_at	TINF2	0		N.S.
220054_at	IL23A	0	1.57	4.40E-31
220058_at	C17orf39	0		N.S.

Supplemental Table 1

220059_at	STAP1	0	-1.05	2.82E-02
220060_s_at	C12orf48	0	-1.16	<1.00E-120
220063_at	GSTCD	0		N.S.
220066_at	NOD2	0	-1.14	2.85E-06
220068_at	VPREB3	0	-1.28	1.94E-10
220079_s_at	USP48	0	1.14	1.15E-36
220085_at	HELLS	0	-1.43	7.24E-35
220086_at	IKZF5	0		N.S.
220089_at	L2HGDH	0	-1.35	<1.00E-120
220091_at	SLC2A6	0	-1.26	1.89E-18
220094_s_at	CCDC90A	0	1.45	2.77E-65
220099_s_at	LUC7L2	0	-1.06	5.67E-05
220103_s_at	MRPS18C	0		N.S.
220104_at	ZC3HAV1	0	-1.58	9.90E-34
220118_at	ZBTB32	0	-2.26	8.07E-66
220121_at	LINS1	0		N.S.
220122_at	MCTP1	0	1.99	1.76E-47
220123_at	SLC35F5	0		N.S.
220127_s_at	FBXL12	0		N.S.
220132_s_at	CLEC2D	0		N.S.
220145_at	MAP9	0		N.S.
220146_at	TLR7	0	1.48	6.63E-37
220147_s_at	FAM60A	0	-1.07	6.69E-05
220148_at	ALDH8A1	0	1.44	5.23E-06
220153_at	ENTPD7	0	1.40	8.14E-19
220155_s_at	BRD9	0	-1.29	3.25E-43
220158_at	LGALS14	0	1.78	1.44E-62
220159_at	ABCA11P	0	1.23	<1.00E-120
220161_s_at	EPB41L4B	0	-1.49	1.08E-22
220169_at	TMEM156	0	1.75	1.12E-47
220172_at	C2orf37	0	1.14	<1.00E-120
220175_s_at	CBWD1	0	-1.16	4.36E-09
220176_at	NUBPL	0	1.09	2.28E-02
220178_at	C19orf28	0	-1.36	7.75E-22
220182_at	SLC25A23	0	-1.28	<1.00E-120
220183_s_at	NUDT6	0	1.21	<1.00E-120
220189_s_at	MGAT4B	0	-1.24	2.62E-19
220195_at	MBD5	0	-1.15	<1.00E-120
220199_s_at	AIDA	0	1.12	<1.00E-120
220200_s_at	SETD8	0	1.11	1.38E-02
220201_at	RC3H2	0	-1.21	<1.00E-120
220212_s_at	THADA	0		N.S.
220214_at	ZNF215	0		N.S.
220215_at	ZNF669	0		N.S.
220219_s_at	LOC100133503	0	1.18	4.82E-06
220223_at	ATAD5	0	-1.53	2.94E-33
220230_s_at	CYB5R2	0	-1.13	1.57E-06
220235_s_at	C1orf103	0	1.42	4.76E-47
220236_at	PDPR	0		N.S.
220238_s_at	KLHL7	0	-1.16	6.19E-06
220241_at	TMCO3	0	1.66	5.13E-24
220244_at	LOH3CR2A	0		N.S.
220246_at	CAMK1D	0		N.S.
220250_at	ZNF286A	0		N.S.

Supplemental Table 1

220251_at	C1orf107	0		N.S.
220255_at	FANCE	0		N.S.
220260_at	TBC1D19	0	1.50	7.77E-26
220261_s_at	ZDHHC4	0	1.25	5.71E-21
220278_at	KDM4D	0		N.S.
220285_at	FAM108B1	0	-1.34	<1.00E-120
220287_at	ADAMTS9	0		N.S.
220288_at	MYO15A	0		N.S.
220305_at	MAVS	0		N.S.
220311_at	N6AMT1	0	-2.05	9.22E-60
220315_at	PARP11	0	1.41	1.45E-18
220319_s_at	MYLIP	0	-1.40	1.50E-23
220329_s_at	RMND1	0	-1.08	5.23E-06
220330_s_at	SAMSN1	0	1.07	1.51E-04
220346_at	MTHFD2L	0	-1.24	1.48E-08
220349_s_at	ENGASE	0		N.S.
220353_at	FAM86C	0	-1.38	2.54E-26
220355_s_at	PBRM1	0	1.06	6.87E-06
220358_at	BATF3	0	1.54	1.13E-33
220367_s_at	SAP130	0	-1.10	<1.00E-120
220368_s_at	SMEK1	0		N.S.
220370_s_at	USP36	0	-1.16	3.49E-09
220371_s_at	SLC12A9	0		N.S.
220372_at	DNAJC28	0		N.S.
220375_s_at	H2AFY	0		N.S.
220386_s_at	EML4	0	-1.40	8.89E-26
220387_s_at	HHLA3	0	1.36	1.06E-37
220390_at	AGBL2	0		N.S.
220391_at	ZBTB3	0	1.23	2.70E-08
220399_at	NCRNA00115	0	-1.20	5.12E-09
220417_s_at	THAP4	0	-1.26	2.92E-27
220419_s_at	USP25	1	1.15	4.47E-28
220444_at	ZNF557	0	-1.49	4.41E-29
220446_s_at	CHST4	0	-1.23	9.17E-03
220450_at	SMAD1	0		N.S.
220458_at	FLJ10246	0		N.S.
220459_at	MCM3APAS	0	-1.91	5.76E-33
220465_at	LOC80054	0		N.S.
220466_at	CCDC15	0	-1.18	7.52E-13
220467_at	FLJ21272	0	1.85	1.13E-31
220470_at	BET1L	0		N.S.
220477_s_at	C20orf30	0	1.09	1.84E-02
220482_s_at	SERGEF	0	-1.19	1.06E-04
220484_at	MCOLN3	0		N.S.
220488_s_at	BCAS3	0	1.35	2.03E-20
220491_at	HAMP	0		N.S.
220494_s_at	C14orf43	0	1.15	4.08E-08
220495_s_at	TXNDC15	0	1.77	1.17E-69
220500_s_at	RABL2A	0	-1.11	6.62E-03
220525_s_at	AUP1	0	1.07	3.55E-09
220534_at	TRIM48	0		N.S.
220547_s_at	FAM35A	0	-1.22	9.30E-29
220550_at	FBXO4	0	-1.45	5.70E-07
220553_s_at	PRPF39	0	-1.22	1.36E-22

Supplemental Table 1

220565_at	CCR10	0	-1.38	1.80E-31
220566_at	PIK3R5	0		N.S.
220572_at	DKFZp547G183	0	-1.42	4.34E-05
220577_at	GVIN1	0	1.90	9.01E-71
220586_at	CHD9	0		N.S.
220587_s_at	GBL	0	1.14	4.65E-09
220588_at	BCAS4	0		N.S.
220590_at	ITFG2	0		N.S.
220597_s_at	ARL6IP4	0		N.S.
220600_at	C3orf75	0		N.S.
220602_s_at	LOC388152	0		N.S.
220603_s_at	MCTP2	0		N.S.
220605_s_at	SIRT2	1	1.10	1.74E-06
220606_s_at	C17orf48	0	1.23	1.32E-26
220608_s_at	ZNF770	0		N.S.
220609_at	LOC202181	0		N.S.
220615_s_at	FAR2	0	-1.58	9.78E-25
220631_at	OSGEPL1	0	-1.26	2.08E-20
220633_s_at	HP1BP3	0		N.S.
220643_s_at	FAIM	0	1.09	1.13E-06
220647_s_at	CHCHD8	0	-1.59	5.69E-76
220651_s_at	MCM10	0	-2.83	4.36E-97
220658_s_at	ARNTL2	0	-1.12	<1.00E-120
220661_s_at	ZNF692	0	-1.31	1.27E-29
220668_s_at	DNMT3B	0		N.S.
220671_at	CCRN4L	0	-1.23	3.18E-05
220682_s_at	KLHL5	0		N.S.
220685_at	FAM120C	0		N.S.
220688_s_at	MRTO4	0	-2.00	3.14E-69
220690_s_at	DHRS7B	0	1.23	7.44E-28
220703_at	C10orf110	0	-1.66	2.53E-20
220712_at	C8orf60	0		N.S.
220721_at	ZNF614	0	1.30	9.88E-20
220731_s_at	NECAP2	0		N.S.
220734_s_at	GLTPD1	0		N.S.
220735_s_at	SENP7	0	1.30	2.89E-03
220739_s_at	CNNM3	0	-1.17	8.36E-20
220740_s_at	SLC12A6	0	1.09	1.49E-02
220741_s_at	PPA2	0		N.S.
220746_s_at	UIMC1	0		N.S.
220748_s_at	ZNF580	0		N.S.
220750_s_at	LEPRE1	0		N.S.
220753_s_at	CRYL1	0	1.16	5.27E-12
220755_s_at	C6orf48	0	1.74	2.25E-82
220757_s_at	UBXN6	0	1.35	8.39E-19
220761_s_at	TAOK3	0	1.28	7.50E-40
220762_s_at	GNB1L	0	-1.38	1.33E-19
220768_s_at	CSNK1G3	0	1.18	8.61E-21
220770_s_at	C5orf54	0	-1.08	1.45E-02
220773_s_at	GPHN	0	-1.24	5.78E-18
220774_at	DYM	0		N.S.
220775_s_at	UEVLD	0		N.S.
220776_at	KCNJ14	0		N.S.
220788_s_at	IRF9	0		N.S.

Supplemental Table 1

220789_s_at	TBRG4	0	-1.56	5.77E-44
220797_at	METT10D	0	-1.35	2.86E-11
220800_s_at	TMOD3	0	-1.26	<1.00E-120
220840_s_at	C1orf112	0	-1.38	2.55E-38
220864_s_at	NDUFA13	0		N.S.
220865_s_at	PDSS1	0	-1.73	2.60E-78
220885_s_at	CENPJ	0	-1.35	7.39E-26
220890_s_at	DDX47	0	-1.32	1.95E-54
220892_s_at	PSAT1	0	5.06	1.78E-87
220917_s_at	WDR19	0	2.12	5.65E-66
220924_s_at	SLC38A2	1	2.26	3.41E-101
220925_at	MAK10	0	1.13	<1.00E-120
220926_s_at	EDEM3	0	1.66	2.55E-46
220933_s_at	ZCCHC6	0	1.13	6.11E-18
220934_s_at	TMEM223	0	-1.29	6.48E-45
220935_s_at	CDK5RAP2	0		N.S.
220936_s_at	H2AFJ	0		N.S.
220937_s_at	ST6GALNAC4	0	1.35	7.24E-26
220939_s_at	DPP8	0	1.12	8.66E-25
220940_at	ANKRD36B	0		N.S.
220941_s_at	C21orf91	0	-1.12	3.05E-09
220943_s_at	C2orf56	0		N.S.
220944_at	PGLYRP4	0		N.S.
220946_s_at	SETD2	0		N.S.
220947_s_at	TBC1D10B	0		N.S.
220948_s_at	ATP1A1	0		N.S.
220949_s_at	C7orf49	0		N.S.
220953_s_at	MTMR12	0	-1.13	1.19E-03
220954_s_at	PILRB	0	1.28	6.52E-28
220956_s_at	EGLN2	0		N.S.
220964_s_at	RAB1B	0	-1.30	1.16E-37
220973_s_at	SHARPIN	0	1.17	1.45E-09
220980_s_at	ADPGK	0	-1.07	3.79E-04
220984_s_at	SLCO5A1	0		N.S.
220985_s_at	RNF170	0	1.42	5.37E-35
220987_s_at	C11orf17	0		N.S.
220988_s_at	C1QTNF3	0	1.19	1.73E-02
220990_s_at	MIR21	0	1.15	<1.00E-120
220991_s_at	RNF32	0	1.95	2.55E-37
220992_s_at	C1orf25	0	1.07	7.55E-04
220993_s_at	GPR63	0		N.S.
221002_s_at	TSPAN14	0		N.S.
221004_s_at	ITM2C	0	-1.14	<1.00E-120
221006_s_at	SNX27	0		N.S.
221007_s_at	FIP1L1	0	-1.11	<1.00E-120
221011_s_at	LBH	0	-1.27	7.47E-19
221012_s_at	TRIM8	0		N.S.
221014_s_at	RAB33B	0	1.23	1.66E-09
221015_s_at	CDADC1	0		N.S.
221020_s_at	SLC25A32	0	-1.40	1.67E-65
221021_s_at	CTNNBL1	0	-1.17	2.16E-09
221027_s_at	PLA2G12A	0		N.S.
221031_s_at	APOLD1	0	1.28	7.52E-13
221036_s_at	APH1B	0	1.14	<1.00E-120

Supplemental Table 1

221039_s_at	ASAP1	0	1.20	1.02E-20
221041_s_at	SLC17A5	0	1.37	2.72E-26
221042_s_at	CLMN	0	-1.25	<1.00E-120
221044_s_at	TRIM34	0	-1.22	1.01E-21
221046_s_at	GTPBP8	0		N.S.
221050_s_at	GTPBP2	0	1.54	1.89E-45
221053_s_at	TDRKH	0		N.S.
221058_s_at	CKLF	0		N.S.
221059_s_at	COTL1	0	-1.08	<1.00E-120
221069_s_at	CCDC44	0	-1.07	1.51E-05
221073_s_at	NOD1	0		N.S.
221079_s_at	METTL2A	0	-1.32	<1.00E-120
221080_s_at	DENND1C	0	1.06	1.74E-04
221081_s_at	DENND2D	0	1.19	3.73E-22
221087_s_at	APOL3	0	-1.37	1.86E-25
221090_s_at	OGFOD1	0	-1.09	<1.00E-120
221092_at	IKZF3	0	-1.44	8.74E-26
221094_s_at	ELP3	0		N.S.
221096_s_at	TMCO6	0	-1.19	1.68E-20
221103_s_at	WDR52	0	1.18	4.14E-06
221104_s_at	NIPSNAP3B	0		N.S.
221135_s_at	ASTE1	0	-1.11	1.41E-03
221139_s_at	CSAD	0	1.27	<1.00E-120
221142_s_at	PECR	0	-1.07	2.74E-02
221187_s_at	FUZ	0		N.S.
221188_s_at	CIDEB	0	-1.31	<1.00E-120
221189_s_at	TARS2	0	-1.14	8.97E-08
221190_s_at	C18orf8	0	1.21	4.88E-31
221193_s_at	ZCCHC10	0	-1.05	1.90E-02
221194_s_at	RNFT1	0		N.S.
221203_s_at	YEATS2	0	-1.13	3.84E-09
221207_s_at	NBEA	0	1.18	1.63E-05
221208_s_at	C11orf61	0		N.S.
221210_s_at	NPL	0	1.29	<1.00E-120
221211_s_at	C21orf7	0	1.39	2.76E-09
221213_s_at	ZNF280D	0		N.S.
221214_s_at	NELF	0	-1.57	1.56E-43
221216_s_at	SCMH1	0	-1.15	<1.00E-120
221218_s_at	TPK1	0	1.09	1.26E-08
221219_s_at	KLHDC4	0	-1.30	1.15E-25
221220_s_at	SCYL2	0		N.S.
221221_s_at	KLHL3	0	1.19	2.94E-08
221222_s_at	C1orf56	0		N.S.
221229_s_at	TRMT61B	0	-1.15	1.07E-26
221230_s_at	ARID4B	0	1.12	1.59E-07
221235_s_at	LOC644617	0	-1.45	1.74E-48
221238_at	NSBP1	0		N.S.
221244_s_at	PDPK1	0		N.S.
221245_s_at	FZD5	0	-1.50	4.84E-29
221247_s_at	WBSCR16	0	-1.29	6.49E-38
221248_s_at	WHSC1L1	0		N.S.
221249_s_at	FAM117A	0	1.33	2.33E-48
221253_s_at	TXNDC5	0		N.S.
221255_s_at	TMEM93	0	-1.18	1.77E-41

Supplemental Table 1

221256_s_at	HDHD3	0	-1.23	<1.00E-120
221258_s_at	KIF18A	0	-1.19	1.07E-19
221260_s_at	CSRNP2	0	1.28	3.41E-33
221262_s_at	SLC2A11	0		N.S.
221263_s_at	SF3B5	0		N.S.
221264_s_at	LOC100128223	0		N.S.
221265_s_at	C15orf44	0		N.S.
221267_s_at	FAM108A1	0	-1.32	7.39E-51
221268_s_at	SGPP1	0		N.S.
221269_s_at	SH3BGRL3	0	1.06	1.60E-03
221270_s_at	QTRT1	0		N.S.
221274_s_at	LMAN2L	0	1.08	1.23E-03
221277_s_at	PUS3	0	-1.17	8.83E-27
221286_s_at	MGC29506	0	2.51	1.08E-47
221293_s_at	DEF6	0		N.S.
221306_at	GPR27	0		N.S.
221326_s_at	TUBD1	0	-1.13	1.99E-04
221381_s_at	MORF4	0		N.S.
221423_s_at	YIPF5	0	1.41	6.22E-27
221425_s_at	ISCA1	0	-1.13	6.77E-12
221427_s_at	CCNL2	0	1.09	2.91E-02
221428_s_at	TBL1XR1	0	-1.12	7.60E-03
221430_s_at	RNF146	0	1.24	1.61E-20
221432_s_at	SLC25A28	0	1.23	<1.00E-120
221434_s_at	C14orf156	0	-1.36	4.18E-63
221436_s_at	CDCA3	0	-1.28	1.37E-38
221437_s_at	MRPS15	0	-1.12	2.79E-26
221449_s_at	ITFG1	0	1.29	2.32E-42
221452_s_at	TMEM14B	0		N.S.
221471_at	SERINC3	0	1.69	1.24E-83
221474_at	MYL12B	0		N.S.
221475_s_at	RPL15	0		N.S.
221479_s_at	BNIP3L	2		N.S.
221483_s_at	ARPP19	0	-1.19	1.50E-38
221484_at	B4GALT5	0	-1.63	6.63E-56
221486_at	ENSA	0	-1.19	9.83E-10
221488_s_at	CUTA	0	1.04	3.28E-02
221489_s_at	SPRY4	0	1.43	<1.00E-120
221492_s_at	ATG3	0		N.S.
221493_at	TSPYL1	0		N.S.
221495_s_at	TCF25	0	1.14	8.41E-22
221502_at	KPNA3	0	-1.33	1.36E-60
221504_s_at	ATP6V1H	1	1.06	6.80E-05
221505_at	ANP32E	0		N.S.
221506_s_at	TNPO2	0	-1.46	4.70E-53
221509_at	DENR	0	1.13	2.57E-22
221513_s_at	UTP14A	0	-1.26	1.25E-26
221515_s_at	LCMT1	0	1.15	2.78E-21
221517_s_at	MED17	0	-1.06	7.37E-04
221518_s_at	USP47	0	1.17	3.83E-40
221520_s_at	CDCA8	0	-1.08	3.31E-08
221521_s_at	GINS2	0	-1.64	2.05E-60
221522_at	ANKRD27	0	-1.14	3.83E-28
221524_s_at	RRAGD	0	-1.22	6.49E-28

Supplemental Table 1

221532_s_at	WDR61	0	-1.11	6.28E-23
221534_at	C11orf68	0		N.S.
221535_at	LSG1	0	-1.26	6.78E-46
221539_at	EIF4EBP1	3	3.72	1.81E-109
221542_s_at	ERLIN2	0	1.11	4.51E-11
221548_s_at	ILKAP	0	-1.28	2.16E-46
221549_at	GRWD1	0	-1.85	5.13E-52
221550_at	COX15	0	-1.07	1.26E-03
221553_at	MAGT1	0	1.99	5.50E-70
221556_at	CDC14B	0	13.79	2.65E-80
221558_s_at	LEF1	0		N.S.
221559_s_at	MIS12	0	1.80	8.91E-75
221561_at	SOAT1	0		N.S.
221565_s_at	CALHM2	0	1.16	2.70E-09
221568_s_at	LIN7C	0		N.S.
221569_at	AHI1	0		N.S.
221570_s_at	METTL5	0		N.S.
221571_at	TRAF3	0	1.24	5.26E-28
221573_at	C7orf25	0	-1.16	4.81E-07
221575_at	SCLY	0	-1.28	1.65E-34
221580_s_at	TAF1D	0	-1.09	<1.00E-120
221582_at	HIST3H2A	0	1.65	3.75E-48
221586_s_at	E2F5	0		N.S.
221587_s_at	C19orf24	0		N.S.
221591_s_at	FAM64A	0	-1.21	<1.00E-120
221593_s_at	RPL31	0	-1.06	4.39E-03
221595_at	DKFZP564O0523	0	1.43	9.30E-30
221597_s_at	TMEM208	0	1.15	<1.00E-120
221598_s_at	LOC100131612	0		N.S.
221600_s_at	C11orf67	0	1.26	1.13E-20
221602_s_at	FAIM3	0		N.S.
221610_s_at	STAP2	0	-1.16	6.52E-08
221616_s_at	TAF9B	0		N.S.
221619_s_at	MTCH1	0	-1.34	3.80E-55
221620_s_at	APOO	0	-1.17	8.84E-18
221621_at	C17orf86	0		N.S.
221622_s_at	TMEM126B	0	-1.14	2.12E-32
221626_at	ZNF506	0	1.25	3.31E-11
221632_s_at	WDR4	0	-1.32	1.08E-23
221634_at	RPL23AP7	0		N.S.
221636_s_at	MOSC2	0		N.S.
221637_s_at	C11orf48	0	-1.48	1.60E-79
221638_s_at	STX16	0		N.S.
221640_s_at	LRDD	1	-1.30	<1.00E-120
221641_s_at	ACOT9	0	1.15	<1.00E-120
221643_s_at	RERE	0		N.S.
221645_s_at	ZNF83	0	1.14	2.15E-03
221646_s_at	ZDHHC11	0	1.28	1.07E-05
221647_s_at	RIC8A	0	-1.08	1.31E-05
221649_s_at	PPAN	0	-1.86	8.64E-68
221650_s_at	MED18	0	-1.26	3.21E-02
221652_s_at	C12orf11	0	-1.16	2.88E-35
221657_s_at	ASB6	0	-1.27	1.58E-11
221666_s_at	PYCARD	0	-1.21	1.03E-19

Supplemental Table 1

221669_s_at	ACAD8	0	-1.06	2.05E-02
221673_s_at	CSNK1G1	0		N.S.
221675_s_at	CHPT1	0		N.S.
221676_s_at	CORO1C	0	-1.39	1.65E-58
221677_s_at	DONSON	0	-1.15	2.39E-29
221681_s_at	DSPP	1		N.S.
221683_s_at	CEP290	0		N.S.
221685_s_at	CCDC99	0	-1.12	<1.00E-120
221688_s_at	IMP3	0	-1.54	8.33E-67
221689_s_at	PIGP	0	1.41	3.83E-74
221692_s_at	MRPL34	0	-1.60	3.07E-65
221699_s_at	DDX50	0	1.14	8.61E-20
221700_s_at	UBA52	0		N.S.
221701_s_at	STRA6	0		N.S.
221702_s_at	TM2D3	0	1.17	2.00E-45
221703_at	BRIP1	0	-1.51	1.49E-23
221704_s_at	VPS37B	0		N.S.
221708_s_at	UNC45A	0		N.S.
221711_s_at	C19orf62	0	1.05	1.77E-05
221712_s_at	WDR74	0	-1.35	1.40E-36
221725_at	WASF2	0	1.20	2.12E-35
221727_at	SUB1	0	-1.25	4.98E-26
221732_at	CANT1	0		N.S.
221734_at	PRRC1	0	1.24	<1.00E-120
221737_at	GNA12	0		N.S.
221738_at	KIAA1219	0	1.20	1.62E-24
221739_at	C19orf10	0	2.06	3.24E-71
221741_s_at	YTHDF1	0	-1.12	6.61E-19
221744_at	WDR68	0	-1.09	1.62E-05
221746_at	UBL4A	0	-1.41	4.83E-41
221749_at	YTHDF3	0		N.S.
221750_at	HMGCS1	0	-2.51	2.66E-71
221751_at	SLC2A3P1	0	-1.33	6.15E-53
221753_at	SSH1	0		N.S.
221758_at	ARMC6	0	-1.35	1.44E-26
221759_at	G6PC3	1		N.S.
221760_at	MAN1A1	0	1.43	2.86E-58
221761_at	ADSS	0	-1.22	5.76E-22
221763_at	JMJD1C	0	1.67	4.60E-73
221766_s_at	FAM46A	0	-1.25	5.42E-11
221770_at	RPE	4	-1.37	1.49E-26
221771_s_at	MPHOSPH8	0		N.S.
221772_s_at	PPP2R2D	0		N.S.
221776_s_at	BRD7	0	-1.05	2.98E-05
221778_at	JHDM1D	0	1.19	8.56E-03
221780_s_at	DDX27	0	-1.11	8.18E-10
221782_at	DNAJC10	2	2.17	7.81E-71
221786_at	C6orf120	0		N.S.
221791_s_at	CCDC72	0		N.S.
221799_at	CSGLCA-T	0		N.S.
221800_s_at	C17orf70	0	-1.38	6.63E-34
221803_s_at	NRBF2	0	1.24	1.95E-26
221804_s_at	FAM45A	0	1.19	5.71E-40
221806_s_at	SETD5	0	1.15	5.78E-18

Supplemental Table 1

221807_s_at	TRABD	0	1.12	3.54E-10
221808_at	RAB9A	0	1.28	3.46E-45
221813_at	FBXO42	0		N.S.
221816_s_at	PHF11	0	1.11	3.70E-24
221817_at	DOLPP1	0	1.37	4.35E-47
221818_at	INTS5	0	-1.24	3.59E-21
221821_s_at	C12orf41	0		N.S.
221823_at	C5orf30	0	-1.38	4.42E-33
221824_s_at	MARCH8	0	1.39	8.03E-50
221825_at	ANGEL2	0	1.17	2.56E-39
221829_s_at	TNPO1	0	-1.06	1.07E-02
221831_at	LUZP1	0	-1.22	<1.00E-120
221833_at	LONP2	0		N.S.
221840_at	PTPRE	0	1.23	<1.00E-120
221843_s_at	KIAA1609	0	1.34	2.24E-24
221845_s_at	CLPB	0	-1.32	9.35E-31
221847_at	LOC100129361	0	1.04	1.77E-04
221851_at	C19orf72	0		N.S.
221853_s_at	NOMO1	0	1.08	3.10E-08
221858_at	TBC1D12	0	1.12	3.90E-08
221864_at	ORAI3	0	1.15	5.19E-11
221865_at	C9orf91	0	1.66	5.34E-70
221867_at	N4BP1	0	1.33	3.90E-18
221873_at	ZNF143	0	-1.41	1.14E-47
221876_at	ZNF783	0	-1.23	1.64E-08
221879_at	CALML4	0	-1.13	3.75E-03
221881_s_at	CLIC4	0	1.58	2.33E-45
221882_s_at	TMEM8	0	-1.18	7.81E-20
221888_at	CC2D1A	0		N.S.
221896_s_at	HIGD1A	0	-1.13	8.39E-27
221897_at	TRIM52	0	1.28	5.23E-28
221904_at	FAM131A	0	-1.19	7.52E-13
221905_at	CYLD	0	1.21	1.50E-12
221909_at	RNFT2	0	1.11	4.86E-06
221912_s_at	CCDC28B	0	-1.28	1.45E-35
221915_s_at	RANBP1	0	-1.29	3.08E-07
221918_at	PCTK2	0	1.50	1.95E-39
221920_s_at	SLC25A37	0		N.S.
221922_at	GPSM2	0	-1.11	4.73E-03
221925_s_at	CSPP1	0	-1.14	3.62E-02
221927_s_at	ABHD11	0	-1.62	5.81E-49
221931_s_at	SEH1L	0	-1.47	4.61E-67
221932_s_at	GLRX5	0	-1.34	1.59E-50
221934_s_at	DALRD3	0	1.19	3.76E-12
221935_s_at	C3orf64	0	-1.18	<1.00E-120
221937_at	AP1GBP1	0	1.13	1.58E-11
221940_at	RPUSD2	0	-1.19	7.25E-22
221957_at	PDK3	0	-1.10	2.10E-07
221960_s_at	RAB2A	0		N.S.
221962_s_at	UBE2H	0	1.10	2.42E-03
221965_at	MPHOSPH9	0		N.S.
221969_at	PAX5	0		N.S.
221970_s_at	NOL11	0	-1.64	1.85E-66
221972_s_at	SDF4	0	1.45	5.97E-51

221978_at	HLA-F	0		N.S.
221983_at	FAM134A	0		N.S.
221985_at	KLHL24	0	1.69	2.41E-40
221987_s_at	TSR1	0	-2.15	3.11E-79
221988_at	C19orf42	0	1.86	4.88E-68
221995_s_at	MRP63	0	-1.24	5.60E-09
221997_s_at	MRPL52	0		N.S.
222000_at	C1orf174	0	-1.11	<1.00E-120
222006_at	LETM1	0	-1.43	2.05E-44
222010_at	TCP1	0	-1.22	1.75E-36
222016_s_at	ZNF323	0	1.27	7.52E-13
222018_at	NACA	0	-1.28	3.92E-26
222019_at	PFDN6	0	-1.33	2.95E-20
222027_at	NUCKS1	0	1.18	1.60E-07
222028_at	ZNF45	0		N.S.
222030_at	SIVA1	0		N.S.
222031_at	LOC286434	0		N.S.
222034_at	GNB2L1	2	-1.19	<1.00E-120
222039_at	KIF18B	0	-1.22	1.60E-32
222040_at	HNRNPA1	0	-1.34	5.35E-41
222046_at	SRRT	0		N.S.
222048_at	CRYBB2P1	0	1.20	5.03E-09
222052_at	C19orf54	0	1.46	5.24E-60
222064_s_at	AARSD1	0		N.S.
222071_s_at	SLCO4C1	0	1.33	7.56E-08
222077_s_at	RACGAP1	0	-1.09	5.93E-08
222088_s_at	SLC2A14	0	1.32	9.02E-25
222103_at	ATF1	2	1.15	<1.00E-120
222105_s_at	NKIRAS2	0		N.S.
222118_at	CENPN	0	-1.31	4.63E-40
222119_s_at	FBXO11	0	1.32	4.37E-51
222120_at	ZNF764	0		N.S.
222125_s_at	P4HTM	0	1.16	9.66E-24
222127_s_at	EXOC1	0	1.08	9.22E-21
222128_at	NSUN6	0	1.18	2.92E-08
222130_s_at	FTSJ2	0	-1.36	1.40E-39
222138_s_at	WDR13	0	1.34	3.94E-35
222139_at	KIAA1466	0	1.26	1.20E-11
222140_s_at	GPR89A	0	1.35	3.64E-57
222143_s_at	MTMR14	0		N.S.
222147_s_at	ACTR5	0	-1.33	7.03E-26
222148_s_at	RHOT1	0		N.S.
222150_s_at	PION	0	1.08	2.58E-04
222154_s_at	LOC26010	0		N.S.
222155_s_at	GPR172A	0	1.59	4.14E-47
222175_s_at	MED15	0		N.S.
222186_at	ZFAND6	0		N.S.
222190_s_at	C16orf58	0	1.50	9.31E-25
222193_at	C2orf43	0	-1.27	2.08E-23
222199_s_at	BIN3	0	-1.20	2.87E-28
222200_s_at	BSDC1	0	1.25	9.59E-19
222201_s_at	CASP8AP2	0	-1.38	1.16E-48
222203_s_at	RDH14	0		N.S.
222204_s_at	RRN3	1	-1.16	3.59E-10

Supplemental Table 1

222208_s_at	POLR2J4	0		N.S.
222209_s_at	TMEM135	0	-1.19	1.69E-31
222212_s_at	LASS2	1	1.09	2.87E-05
222214_at	SUZ12P	0	-1.23	1.62E-03
222216_s_at	MRPL17	0	-1.81	3.25E-71
222217_s_at	SLC27A3	0	1.23	<1.00E-120
222228_s_at	ALKBH4	0		N.S.
222230_s_at	ACTR10	0	1.30	9.09E-53
222231_s_at	LRRC59	0		N.S.
222233_s_at	DCLRE1C	0	1.17	1.78E-20
222235_s_at	CSGALNACT2	0	1.75	7.79E-46
222238_s_at	POLM	0	-1.15	1.65E-07
222239_s_at	INTS6	0	1.08	5.48E-05
222243_s_at	TOB2	0		N.S.
222244_s_at	TUG1	0		N.S.
222250_s_at	INTS7	0		N.S.
222251_s_at	GMEB2	0		N.S.
222262_s_at	ETNK1	0	-1.34	1.44E-39
222263_at	SLC35E1	0		N.S.
222264_at	HNRNPUL2	0		N.S.
222270_at	SMEK2	0	-1.40	5.00E-08
222273_at	PAPOLG	0		N.S.
222275_at	MRPS30	0	-1.22	5.04E-26
222276_at	METTL2B	0		N.S.
222279_at	RP3-377H14.5	0		N.S.
222283_at	ZNF480	0	-1.41	2.63E-11
222305_at	HK2	0	-1.72	2.84E-25
222307_at	LOC282997	0	1.25	1.50E-12
222310_at	SFRS15	0		N.S.
222313_at	CNOT2	0		N.S.
222316_at	USO1	0	1.92	1.57E-40
222318_at	ZNF324B	0	-1.21	9.26E-07
222326_at	PDE4B	1	-1.54	5.26E-07
222336_at	C4orf34	0	1.38	4.56E-04
222344_at	C5orf13	0		N.S.
222350_at	BCL9	0	-1.32	2.55E-02
222351_at	PPP2R1B	0	-1.19	6.33E-07
222354_at	F11R	0	-1.24	3.42E-02
222360_at	DPH5	0		N.S.
222366_at	ADNP	0		N.S.
222369_at	NAT11	0	-1.44	7.39E-42
222376_at	HACE1	0	-1.17	3.89E-08
266_s_at	CD24	1	-1.70	2.50E-19
31845_at	ELF4	0	-1.17	2.75E-21
32032_at	DGCR14	0		N.S.
32091_at	SLC25A44	0	-1.13	<1.00E-120
32811_at	MYO1C	0	-1.10	7.54E-10
32836_at	AGPAT1	0		N.S.
33322_i_at	SFN	1	-1.17	5.72E-09
33494_at	ETFDH	0	1.20	1.50E-36
33760_at	PEX14	0	-1.09	1.55E-10
33814_at	PAK4	0	-1.17	<1.00E-120
33850_at	MAP4	0	1.08	4.45E-02
34210_at	CD52	0	-1.29	2.14E-34

Supplemental Table 1

34221_at	HMGXB3	0	-1.07	4.34E-02
34260_at	TELO2	0	-1.58	1.68E-53
34406_at	PACS2	0	-1.16	2.48E-06
34408_at	RTN2	0	1.22	1.07E-10
34478_at	RAB11B	0		N.S.
34697_at	LRP6	0		N.S.
34858_at	KCTD2	0	-1.11	7.67E-08
34868_at	SMG5	0	1.17	6.36E-19
35160_at	LDB1	0	-1.20	<1.00E-120
35265_at	FXR2	0		N.S.
35626_at	SGSH	0	1.19	8.27E-11
35671_at	GTF3C1	0	-1.18	1.49E-27
36019_at	STK19	0	1.40	1.33E-61
36030_at	IFFO1	0		N.S.
36084_at	CUL7	0		N.S.
36475_at	GCAT	0		N.S.
36545_s_at	SFI1	0		N.S.
36552_at	C2CD3	0	-1.16	7.94E-25
36711_at	MAFF	0	1.48	8.78E-32
36829_at	PER1	0	1.24	3.41E-20
36865_at	ANGEL1	0	-1.15	1.73E-17
36907_at	MVK	0	-1.27	1.63E-28
36920_at	MTM1	0	1.62	8.69E-39
36994_at	ATP6V0C	0		N.S.
37012_at	CAPZB	0		N.S.
37028_at	PPP1R15A	20	2.50	6.12E-76
37079_at	YDD19	0	1.41	1.00E-19
37152_at	PPARD	0	1.16	<1.00E-120
37170_at	BMP2K	0		N.S.
37226_at	BNIP1	0	1.12	<1.00E-120
37232_at	KIAA0586	0		N.S.
37254_at	ZNF133	0	1.10	7.10E-06
37278_at	TAZ	0	1.20	1.50E-12
37462_i_at	SF3A2	0	-1.19	1.76E-29
37512_at	HSD17B6	0		N.S.
37802_r_at	FAM63B	0		N.S.
37860_at	ZNF337	0		N.S.
37872_at	JRK	0	-1.15	1.50E-12
37966_at	PARVB	0	-1.18	3.97E-28
38290_at	RGS14	0	1.24	2.81E-37
38398_at	MADD	0	1.08	4.89E-05
38447_at	ADRBK1	0		N.S.
38892_at	KIAA0240	0	1.27	2.32E-40
39248_at	AQP3	0	-1.15	6.07E-03
39318_at	TCL1A	0	-1.64	4.82E-69
39729_at	PRDX2	0	-1.06	3.60E-03
39817_s_at	C6orf108	0	-1.08	2.28E-10
40020_at	CELSR3	0		N.S.
40149_at	SH2B1	1		N.S.
40255_at	DDX28	0	-1.38	1.28E-55
40273_at	SPHK2	0	-1.23	1.11E-20
40359_at	RASSF7	0	-1.27	1.08E-27
40420_at	STK10	0	1.39	3.77E-62
40446_at	PHF1	0	1.19	<1.00E-120

Supplemental Table 1

40465_at	DDX23	0	-1.42	4.25E-52
40829_at	WDTC1	0	1.12	<1.00E-120
41160_at	MBD3	0	-1.32	5.20E-53
41220_at	SEPT9	0	-1.05	2.47E-02
41387_r_at	KDM6B	0	1.25	<1.00E-120
41397_at	ZNF821	0	1.63	2.22E-42
41512_at	BRAP	0	1.12	<1.00E-120
41577_at	PPP1R16B	0		N.S.
41657_at	STK11	0		N.S.
41858_at	FRAG1	0	1.12	4.35E-18
43544_at	MED16	0	-1.12	<1.00E-120
43934_at	GPR137	0	1.21	2.12E-07
43977_at	TMEM161A	0	1.19	3.93E-33
44065_at	C12orf52	0	-1.20	5.78E-33
44669_at	LOC644096	0	-1.09	9.78E-12
44702_at	SYDE1	0		N.S.
44783_s_at	HEY1	0	-1.19	7.19E-20
45687_at	PRR14	0		N.S.
46167_at	TTC4	0	-1.33	3.45E-53
46270_at	UBAP1	0		N.S.
46665_at	SEMA4C	0		N.S.
47083_at	C7orf26	0	-1.07	2.52E-03
47571_at	ZNF236	0		N.S.
47608_at	TJAP1	0	1.11	4.12E-10
48117_at	CCDC101	0		N.S.
48531_at	TNIP2	0	-1.24	7.55E-50
48659_at	MIIP	0	1.20	1.35E-33
49306_at	RASSF4	0	-1.12	1.85E-02
49327_at	SIRT3	0	1.28	5.75E-28
49485_at	PRDM4	0		N.S.
50221_at	TFEB	0	1.15	<1.00E-120
50277_at	GGA1	0		N.S.
50374_at	C17orf90	0	-1.17	7.18E-25
51158_at	FAM174B	0	1.62	9.05E-47
51774_s_at	LOC222070	0		N.S.
52078_at	TMEM222	0		N.S.
52164_at	C11orf24	0	-1.25	4.73E-33
52169_at	STRADA	0	-1.11	<1.00E-120
52741_at	TRMT61A	0	-1.28	<1.00E-120
52940_at	SIGIRR	0		N.S.
53076_at	B4GALT7	0	1.13	1.50E-12
53912_at	SNX11	0		N.S.
53987_at	RANBP10	0		N.S.
54037_at	HPS4	0		N.S.
54051_at	PKNOX1	0		N.S.
54970_at	ZMIZ2	0	-1.18	8.35E-19
55065_at	MARK4	0	1.07	2.27E-06
55081_at	MICALL1	0	-1.13	3.01E-11
55616_at	PERLD1	0	1.13	2.04E-05
55662_at	C10orf76	0		N.S.
55692_at	ELMO2	0	1.09	3.17E-09
55705_at	C19orf22	0		N.S.
55872_at	ZNF512B	0	-1.17	<1.00E-120
56256_at	SIDT2	0	1.13	<1.00E-120

Supplemental Table 1

56919_at	WDR48	0	1.36	2.71E-27
57082_at	LDLRAP1	0	-1.12	1.20E-06
57163_at	ELOVL1	0	-1.09	1.46E-03
57539_at	ZGPAT	0	-1.21	3.44E-23
58308_at	TRIM62	0		N.S.
58696_at	EXOSC4	0	-1.82	7.77E-74
59625_at	NOL3	0	1.32	1.73E-20
60528_at	JMJD7-PLA2G4B	2		N.S.
63009_at	SHQ1	0	-1.33	1.07E-46
632_at	GSK3A	0		N.S.
635_s_at	PPP2R5B	0	1.35	1.32E-22
64371_at	SFRS14	0		N.S.
64486_at	CORO1B	0	-1.13	6.71E-18
64488_at	IRGQ	0	-1.15	8.76E-08
64883_at	MOSPD2	0	1.37	3.02E-30
64900_at	FLJ22167	0	1.23	<1.00E-120
65086_at	YIPF2	0	1.22	<1.00E-120
65133_i_at	INO80B	0	-1.35	3.88E-25
65585_at	FAM86B1	0	-1.43	1.69E-46
65588_at	LOC388796	0	-1.14	<1.00E-120
65630_at	TMEM80	0	1.22	1.24E-17
65770_at	RHOT2	0	-1.35	7.88E-47
77508_r_at	LOC100133585	0	-1.11	1.88E-04
78047_s_at	LOC729580	0		N.S.
78383_at	LOC100129250	0		N.S.
87100_at	ABHD2	0		N.S.
89948_at	PCIF1	0	-1.18	7.52E-13
90610_at	LRCH4	0	1.07	4.17E-03
91703_at	EHBP1L1	0	-1.41	5.12E-35
91816_f_at	MEX3D	0	-1.99	9.42E-54

Supplemental Table 2. Changes in gene expression levels induced by ionizing radiation 2 and 6 hours after treatment						
**Affymetrix ID**	**Gene Symbol**	**# of articles gene associated with IR stress in Pubmed**	**Fold Change 2 hours**	**$P_{corrected}$ value (2 hours)**	**Fold Change 6 hours**	**$P_{corrected}$ value (6 hours)**
1007_s_at	DDR1	2		N.S	1.59	2.31E-28
1053_at	RFC2	21		N.S		N.S
1405_i_at	CCL5	4		N.S		N.S
1487_at	ESRRA	0		N.S		N.S
1729_at	TRADD	7		N.S	-1.19	4.92E-03
1861_at	BAD	104		N.S		N.S
200000_s_at	PRPF8	0		N.S		N.S
200001_at	CAPNS1	1		N.S		N.S
200002_at	RPL35	0		N.S		N.S
200003_s_at	RPL28	0		N.S		N.S
200004_at	EIF4G2	0		N.S		N.S
200005_at	EIF3D	0		N.S		N.S
200006_at	PARK7	1		N.S		N.S
200007_at	SRP14	0		N.S		N.S
200009_at	GDI2	0		N.S	-1.08	3.65E-05
200010_at	RPL11	1		N.S		N.S
200013_at	RPL24	0		N.S		N.S
200015_s_at	SEPT2	1		N.S		N.S
200017_at	RPS27A	0		N.S		N.S
200018_at	RPS13	0		N.S		N.S
200019_s_at	FAU	1		N.S		N.S
200020_at	TARDBP	0	-1.2	4.71E-13		N.S
200021_at	CFL1	1		N.S		N.S
200024_at	RPS5	0		N.S		N.S
200026_at	RPL34	0		N.S		N.S
200027_at	NARS	0	-1.1	2.06E-09	1.09	9.99E-04
200029_at	RPL19	0		N.S		N.S
200030_s_at	SLC25A3	0		N.S		N.S
200032_s_at	RPL9	0		N.S		N.S
200033_at	DDX5	7	1.1	1.50E-13		N.S
200034_s_at	RPL6	0		N.S		N.S
200035_at	DULLARD	0		N.S		N.S
200036_s_at	RPL10A	1		N.S		N.S
200038_s_at	RPL17	0		N.S		N.S
200039_s_at	PSMB2	0		N.S		N.S
200040_at	KHDRBS1	2		N.S	1.06	3.31E-03
200041_s_at	BAT1	0		N.S		N.S
200042_at	C22orf28	0		N.S	1.09	6.53E-04
200043_at	ERH	0		N.S		N.S
200044_at	SFRS9	2		N.S		N.S
200045_at	ABCF1	0		N.S		N.S
200046_at	DAD1	1		N.S		N.S
200048_s_at	JTB	0		N.S		N.S
200049_at	MYST2	1		N.S		N.S
200050_at	ZNF146	0	-1.2	2.35E-04		N.S
200051_at	SART1	0		N.S		N.S
200052_s_at	ILF2	0		N.S		N.S
200053_at	SPAG7	0		N.S		N.S
200054_at	ZNF259	0		N.S		N.S
200055_at	TAF10	0		N.S		N.S

Supplemental Table 2

200056_s_at	C1D	3		N.S		N.S
200059_s_at	RHOA	16		N.S	1.07	1.59E-04
200060_s_at	RNPS1	1	1.1	1.94E-07		N.S
200061_s_at	RPS24	0		N.S		N.S
200062_s_at	RPL30	0		N.S		N.S
200063_s_at	NPM1	4		N.S		N.S
200064_at	HSP90AB1	1		N.S		N.S
200065_s_at	ARF1	0		N.S		N.S
200066_at	IK	1		N.S		N.S
200071_at	SMNDC1	0		N.S		N.S
200073_s_at	HNRNPD	0		N.S		N.S
200074_s_at	RPL14	0		N.S		N.S
200077_s_at	OAZ1	0		N.S		N.S
200078_s_at	ATP6V0B	0		N.S		N.S
200080_s_at	H3F3A	0		N.S		N.S
200082_s_at	RPS7	0		N.S		N.S
200084_at	C11orf58	0		N.S		N.S
200085_s_at	TCEB2	1		N.S		N.S
200089_s_at	RPL4	1		N.S		N.S
200090_at	FNTA	0		N.S	1.06	3.48E-02
200091_s_at	RPS25	0		N.S		N.S
200092_s_at	RPL37	1		N.S		N.S
200093_s_at	HINT1	4		N.S		N.S
200096_s_at	ATP6V0E1	0		N.S		N.S
200099_s_at	RPS3A	0		N.S		N.S
200593_s_at	HNRNPU	0		N.S		N.S
200596_s_at	EIF3A	0		N.S		N.S
200599_s_at	HSP90B1	0		N.S		N.S
200600_at	MSN	5		N.S		N.S
200601_at	ACTN4	0		N.S		N.S
200604_s_at	PRKAR1A	1		N.S	-1.21	3.65E-04
200607_s_at	RAD21	21		N.S		N.S
200609_s_at	WDR1	2		N.S	1.11	1.09E-02
200610_s_at	NCL	43		N.S		N.S
200613_at	AP2M1	0		N.S		N.S
200615_s_at	AP2B1	0		N.S		N.S
200616_s_at	MLEC	4		N.S		N.S
200618_at	LASP1	0		N.S		N.S
200619_at	SF3B2	0		N.S		N.S
200620_at	TMEM59	0		N.S		N.S
200621_at	CSRP1	0		N.S	1.20	1.25E-08
200623_s_at	CALM3	0		N.S		N.S
200626_s_at	MATR3	2		N.S		N.S
200627_at	PTGES3	0		N.S	-1.12	4.64E-10
200629_at	WARS	284		N.S		N.S
200632_s_at	NDRG1	3		N.S		N.S
200633_at	UBB	0		N.S		N.S
200634_at	PFN1	0		N.S		N.S
200642_at	SOD1	42		N.S		N.S
200644_at	MARCKSL1	0		N.S		N.S
200645_at	GABARAP	0		N.S		N.S
200649_at	NUCB1	0		N.S		N.S
200650_s_at	LDHA	1		N.S		N.S
200652_at	SSR2	0		N.S		N.S
200654_at	P4HB	1		N.S		N.S
200657_at	SLC25A5	0		N.S		N.S
200658_s_at	PHB	2		N.S		N.S
200660_at	S100A11	1		N.S		N.S
200661_at	CTSA	0		N.S		N.S
200663_at	CD63	1		N.S		N.S

Supplemental Table 2

200665_s_at	SPARC	3		N.S	1.61	5.04E-21
200666_s_at	DNAJB1	1	-1.1	1.71E-02	1.20	5.95E-07
200668_s_at	UBE2D3	2		N.S		N.S
200670_at	XBP1	15		N.S	-1.26	4.60E-15
200673_at	LAPTM4A	0		N.S		N.S
200674_s_at	RPL32	0		N.S		N.S
200675_at	CD81	0		N.S		N.S
200677_at	PTTG1IP	0		N.S		N.S
200681_at	GLO1	0		N.S		N.S
200682_s_at	UBE2L3	0		N.S		N.S
200687_s_at	SF3B3	2		N.S	-1.18	6.72E-06
200692_s_at	HSPA9	2		N.S		N.S
200695_at	PPP2R1A	0		N.S		N.S
200696_s_at	GSN	0		N.S		N.S
200697_at	HK1	1		N.S	-1.23	3.37E-06
200699_at	KDELR2	0	-1.1	2.03E-06	-1.15	1.10E-06
200701_at	NPC2	0		N.S		N.S
200702_s_at	DDX24	0		N.S	-1.13	3.96E-02
200703_at	DYNLL1	1		N.S		N.S
200705_s_at	EEF1B2	1		N.S		N.S
200706_s_at	LITAF	0		N.S		N.S
200707_at	PRKCSH	0		N.S		N.S
200708_at	GOT2	0		N.S		N.S
200709_at	FKBP1A	0		N.S		N.S
200710_at	ACADVL	0		N.S		N.S
200713_s_at	MAPRE1	1		N.S		N.S
200718_s_at	SKP1	20		N.S		N.S
200720_s_at	ACTR1A	0		N.S	1.14	3.64E-08
200722_s_at	CAPRIN1	0		N.S		N.S
200726_at	PPP1CC	1		N.S		N.S
200729_s_at	ACTR2	0		N.S		N.S
200733_s_at	PTP4A1	0	1.2	3.24E-03	1.31	6.19E-08
200734_s_at	ARF3	1		N.S		N.S
200736_s_at	GPX1	24		N.S	1.25	9.85E-09
200740_s_at	SUMO3	1	1.1	7.03E-11	1.07	5.02E-03
200741_s_at	RPS27	3		N.S		N.S
200743_s_at	TPP1	16		N.S		N.S
200746_s_at	GNB1	0		N.S		N.S
200748_s_at	FTH1	0		N.S		N.S
200749_at	RAN	24	1.1	9.29E-07		N.S
200751_s_at	HNRNPC	2		N.S		N.S
200755_s_at	CALU	9		N.S		N.S
200761_s_at	ARL6IP5	9	-1.1	9.95E-12	1.11	1.52E-06
200762_at	DPYSL2	0		N.S		N.S
200763_s_at	RPLP1	0		N.S		N.S
200766_at	CTSD	1		N.S		N.S
200767_s_at	FAM120A	0		N.S		N.S
200770_s_at	LAMC1	0		N.S		N.S
200775_s_at	HNRNPK	0		N.S		N.S
200776_s_at	BZW1	0	1.1	1.96E-02		N.S
200779_at	ATF4	17	1.2	2.90E-09	-1.14	5.13E-06
200782_at	ANXA5	109		N.S		N.S
200783_s_at	STMN1	8	1.2	4.34E-15	1.27	7.11E-16
200786_at	PSMB7	0		N.S		N.S
200788_s_at	PEA15	2	1.2	2.79E-06	1.39	4.51E-22
200789_at	ECH1	1		N.S		N.S
200790_at	ODC1	2		N.S	-1.21	1.66E-17
200792_at	XRCC6	4		N.S		N.S
200793_s_at	ACO2	0		N.S		N.S
200802_at	SARS	21		N.S	1.35	1.09E-25

200804_at	TMBIM6	0		N.S		N.S
200805_at	LMAN2	0		N.S		N.S
200806_s_at	HSPD1	2		N.S		N.S
200808_s_at	ZYX	0		N.S	-1.22	1.69E-03
200811_at	CIRBP	2	1.3	1.67E-19	1.34	2.96E-17
200812_at	CCT7	2		N.S	-1.07	1.72E-03
200814_at	PSME1	0		N.S		N.S
200815_s_at	PAFAH1B1	1		N.S		N.S
200818_at	ATP5O	0		N.S		N.S
200819_s_at	RPS15	0		N.S		N.S
200820_at	PSMD8	0		N.S		N.S
200824_at	GSTP1	130		N.S		N.S
200825_s_at	HYOU1	0		N.S	1.23	1.14E-05
200826_at	SNRPD2	0		N.S		N.S
200827_at	PLOD1	0	-1.2	7.54E-03	-1.40	9.42E-15
200828_s_at	ZNF207	0	1.1	5.55E-06		N.S
200830_at	PSMD2	0		N.S		N.S
200833_s_at	hCG_1757335	0		N.S	1.09	7.12E-03
200834_s_at	RPS21	0		N.S		N.S
200837_at	BCAP31	1		N.S		N.S
200840_at	KARS	2		N.S		N.S
200842_s_at	EPRS	0		N.S		N.S
200845_s_at	PRDX6	4		N.S		N.S
200846_s_at	PPP1CA	1		N.S		N.S
200847_s_at	TMEM66	0		N.S		N.S
200851_s_at	KIAA0174	0		N.S	1.10	8.55E-03
200853_at	H2AFZ	0		N.S		N.S
200854_at	NCOR1	2		N.S		N.S
200855_at	C20orf191	0	-1.2	7.29E-03		N.S
200860_s_at	CNOT1	0		N.S		N.S
200862_at	DHCR24	0		N.S	-1.25	2.15E-08
200863_s_at	RAB11A	0		N.S		N.S
200870_at	STRAP	8		N.S		N.S
200871_s_at	PSAP	0		N.S		N.S
200873_s_at	CCT8	0		N.S		N.S
200875_s_at	NOP56	0	1.1	9.66E-08		N.S
200877_at	CCT4	0		N.S	-1.10	3.93E-10
200881_s_at	DNAJA1	1	-1.1	1.29E-05		N.S
200882_s_at	PSMD4	1		N.S		N.S
200885_at	RHOC	1		N.S	1.18	6.95E-03
200886_s_at	PGAM1	0		N.S		N.S
200891_s_at	SSR1	0		N.S		N.S
200892_s_at	TRA2B	0		N.S		N.S
200894_s_at	FKBP4	0	-1.2	3.55E-02	-1.48	3.15E-18
200900_s_at	M6PR	0		N.S		N.S
200902_at	SEP15	0		N.S		N.S
200903_s_at	AHCY	1		N.S	1.08	2.00E-02
200904_at	HLA-E	1	1.1	3.42E-04	-1.14	5.01E-06
200908_s_at	RPLP2	0		N.S		N.S
200910_at	CCT3	1		N.S	-1.10	1.50E-09
200911_s_at	TACC1	0		N.S		N.S
200912_s_at	EIF4A2	0	-1.1	5.08E-09		N.S
200913_at	PPM1G	1		N.S		N.S
200918_s_at	SRPR	0		N.S		N.S
200919_at	PHC2	0		N.S		N.S
200920_s_at	BTG1	4	1.3	1.72E-18	1.30	3.78E-19
200922_at	KDELR1	0		N.S		N.S
200924_s_at	SLC3A2	0		N.S		N.S
200925_at	COX6A1	0		N.S		N.S
200926_at	RPS23	0		N.S		N.S

Supplemental Table 2

200928_s_at	RAB14	0		N.S		N.S
200932_s_at	DCTN2	0		N.S	1.09	2.15E-03
200934_at	DEK	5		N.S	-1.11	3.71E-06
200935_at	CALR	0		N.S		N.S
200936_at	RPL8	0		N.S		N.S
200937_s_at	RPL5	1		N.S		N.S
200941_at	HSBP1	1	1.1	7.24E-07		N.S
200944_s_at	HMGN1	8		N.S		N.S
200945_s_at	SEC31A	0		N.S		N.S
200947_s_at	GLUD1	0		N.S		N.S
200948_at	MLF2	0		N.S	1.50	5.08E-17
200951_s_at	CCND2	2		N.S		N.S
200955_at	IMMT	0		N.S		N.S
200957_s_at	SSRP1	10		N.S		N.S
200958_s_at	SDCBP	0	-1.1	2.07E-05		N.S
200959_at	FUS	14	1.1	3.55E-05		N.S
200961_at	SEPHS2	0	-1.2	5.04E-11	-1.16	2.00E-10
200964_at	UBA1	1		N.S		N.S
200965_s_at	ABLIM1	0		N.S		N.S
200967_at	PPIB	1		N.S		N.S
200971_s_at	SERP1	1		N.S		N.S
200973_s_at	TSPAN3	0		N.S		N.S
200975_at	PPT1	0		N.S		N.S
200977_s_at	TAX1BP1	0		N.S		N.S
200978_at	MDH1	0		N.S		N.S
200980_s_at	PDHA1	0		N.S		N.S
200982_s_at	ANXA6	1		N.S	-1.13	1.12E-03
200984_s_at	CD59	15		N.S	1.09	5.81E-06
200989_at	HIF1A	68	1.1	6.16E-03		N.S
200990_at	TRIM28	13		N.S		N.S
200991_s_at	SNX17	0		N.S	-1.14	1.08E-02
200994_at	IPO7	0		N.S	1.07	2.00E-02
200997_at	RBM4	0		N.S		N.S
200998_s_at	CKAP4	27		N.S	-1.17	3.11E-05
201000_at	AARS	1		N.S		N.S
201002_s_at	TMEM189-UBE2V1	0		N.S		N.S
201004_at	SSR4	0		N.S		N.S
201007_at	HADHB	0		N.S		N.S
201008_s_at	TXNIP	2		N.S		N.S
201011_at	RPN1	1		N.S		N.S
201012_at	ANXA1	3		N.S		N.S
201017_at	EIF1AX	0		N.S		N.S
201019_s_at	EIF1AP1	0		N.S		N.S
201020_at	YWHAH	2		N.S	-1.10	1.76E-04
201021_s_at	DSTN	0		N.S		N.S
201023_at	TAF7	0	-1.1	1.21E-03	-1.09	5.96E-03
201028_s_at	CD99	0		N.S		N.S
201031_s_at	HNRNPH1	0		N.S		N.S
201032_at	BLCAP	0		N.S	1.33	2.51E-22
201037_at	PFKP	0		N.S	-1.44	2.52E-15
201039_s_at	RAD23A	24		N.S	-1.20	1.04E-04
201041_s_at	DUSP1	6		N.S	-1.28	3.28E-06
201049_s_at	RPS18	0		N.S		N.S
201051_at	ANP32A	0		N.S	-1.08	6.59E-05
201053_s_at	PSMF1	0		N.S	1.11	4.32E-07
201055_s_at	HNRNPA0	0	1.1	7.88E-07		N.S
201056_at	GOLGB1	0		N.S	1.15	4.42E-02
201063_at	RCN1	0		N.S		N.S
201064_s_at	PABPC4	0		N.S		N.S
201066_at	CYC1	12		N.S		N.S

201068_s_at	PSMC2	0		N.S		N.S
201074_at	SMARCC1	0		N.S		N.S
201077_s_at	NHP2L1	1		N.S		N.S
201078_at	TM9SF2	0		N.S		N.S
201079_at	SYNGR2	0		N.S		N.S
201085_s_at	SON	30		N.S		N.S
201088_at	KPNA2	3		N.S	-1.30	5.29E-18
201089_at	ATP6V1B2	0		N.S	1.11	4.22E-02
201091_s_at	CBX3	1		N.S		N.S
201092_at	RBBP7	2		N.S		N.S
201094_at	RPS29	0		N.S		N.S
201095_at	DAP	22		N.S	1.13	5.09E-04
201097_s_at	ARF4	1		N.S		N.S
201098_at	COPB2	0		N.S		N.S
201099_at	USP9X	1		N.S		N.S
201101_s_at	BCLAF1	3		N.S		N.S
201102_s_at	PFKL	0		N.S		N.S
201106_at	GPX4	4		N.S		N.S
201112_s_at	CSE1L	1		N.S	1.10	1.09E-02
201113_at	TUFM	0		N.S		N.S
201115_at	POLD2	1		N.S		N.S
201118_at	PGD	4		N.S		N.S
201119_s_at	COX8A	0		N.S		N.S
201121_s_at	PGRMC1	3		N.S		N.S
201126_s_at	MGAT1	0		N.S		N.S
201128_s_at	ACLY	0		N.S		N.S
201129_at	SFRS7	0	-1.1	6.63E-12	-1.31	2.06E-19
201132_at	HNRNPH2	1		N.S	-1.12	1.84E-04
201133_s_at	PJA2	0		N.S		N.S
201135_at	ECHS1	0		N.S		N.S
201136_at	PLP2	0		N.S		N.S
201137_s_at	HLA-DPB1	0		N.S		N.S
201139_s_at	SSB	605		N.S		N.S
201141_at	GPNMB	0		N.S		N.S
201144_s_at	EIF2S1	0		N.S		N.S
201145_at	HAX1	0		N.S		N.S
201146_at	NFE2L2	26	1.3	1.56E-17	1.21	9.57E-16
201155_s_at	MFN2	2		N.S	1.18	2.11E-02
201156_s_at	RAB5C	0		N.S		N.S
201158_at	NMT1	2		N.S		N.S
201165_s_at	PUM1	0	-1.1	1.24E-02		N.S
201170_s_at	BHLHE40	0	1.2	6.16E-07		N.S
201174_s_at	TERF2IP	5		N.S	1.10	7.75E-03
201175_at	TMX2	0		N.S	1.09	1.56E-02
201176_s_at	ARCN1	0		N.S		N.S
201177_s_at	UBA2	3		N.S		N.S
201178_at	FBXO7	0		N.S	-1.09	1.20E-05
201180_s_at	GNAI3	0		N.S		N.S
201182_s_at	CHD4	2		N.S		N.S
201186_at	LRPAP1	0		N.S	1.26	1.71E-13
201191_at	PITPNA	0		N.S		N.S
201193_at	IDH1	0	-1.3	9.24E-24	-1.42	1.86E-25
201194_at	SEPW1	0		N.S		N.S
201195_s_at	SLC7A5	1		N.S		N.S
201197_at	AMD1	0	1.1	1.18E-04	-1.15	6.17E-04
201198_s_at	PSMD1	0		N.S		N.S
201200_at	CREG1	0	1.1	3.61E-02	1.11	6.62E-03
201201_at	CSTB	1		N.S		N.S
201204_s_at	RRBP1	0		N.S	-1.20	3.91E-04
201209_at	HDAC1	40		N.S		N.S

Supplemental Table 2

201212_at	LGMN	0		N.S	-1.18	1.75E-04
201214_s_at	PPP1R7	0		N.S	-1.10	1.05E-03
201215_at	PLS3	0		N.S		N.S
201216_at	ERP29	2		N.S	-1.10	8.84E-03
201221_s_at	SNRNP70	0		N.S		N.S
201223_s_at	RAD23B	28		N.S	-1.14	3.53E-03
201224_s_at	SRRM1	0		N.S		N.S
201226_at	NDUFB8	0		N.S		N.S
201228_s_at	ARIH2	0		N.S		N.S
201231_s_at	ENO1	1		N.S		N.S
201232_s_at	PSMD13	0		N.S		N.S
201234_at	ILK	9		N.S		N.S
201235_s_at	BTG2	16	1.5	4.47E-08	2.02	3.57E-20
201238_s_at	CAPZA2	0		N.S		N.S
201240_s_at	LOC653566	0		N.S		N.S
201241_at	DDX1	1		N.S		N.S
201243_s_at	ATP1B1	0		N.S		N.S
201244_s_at	RAF1	5		N.S		N.S
201246_s_at	OTUB1	1		N.S		N.S
201248_s_at	SREBF2	1		N.S		N.S
201250_s_at	SLC2A1	4		N.S	-1.34	1.32E-12
201252_at	PSMC4	0		N.S		N.S
201253_s_at	CDIPT	0		N.S		N.S
201256_at	COX7A2L	1		N.S	1.10	1.63E-03
201258_at	RPS16	0		N.S		N.S
201260_s_at	SYPL1	0		N.S		N.S
201263_at	TARS	44		N.S		N.S
201266_at	TXNRD1	9	1.2	3.74E-07		N.S
201267_s_at	PSMC3	0		N.S	1.14	1.67E-10
201268_at	NME1	11		N.S		N.S
201271_s_at	RALY	1		N.S		N.S
201272_at	AKR1B1	0		N.S	1.11	1.44E-03
201273_s_at	SRP9	0		N.S		N.S
201274_at	PSMA5	0		N.S		N.S
201275_at	FDPS	0		N.S	-1.10	7.13E-03
201276_at	RAB5B	0	-1.1	2.89E-04		N.S
201277_s_at	HNRNPAB	0	1.1	4.54E-09		N.S
201281_at	ADRM1	0	1.1	2.87E-03	1.12	4.10E-03
201282_at	OGDH	0		N.S		N.S
201284_s_at	APEH	0		N.S	-1.12	4.72E-03
201288_at	ARHGDIB	1		N.S		N.S
201290_at	SEC11A	0		N.S		N.S
201291_s_at	TOP2A	1		N.S	-1.24	1.19E-04
201299_s_at	MOBKL1B	0		N.S		N.S
201302_at	ANXA4	0		N.S	1.22	4.00E-20
201303_at	EIF4A3	0		N.S		N.S
201304_at	NDUFA5	0		N.S		N.S
201306_s_at	ANP32B	0		N.S		N.S
201312_s_at	SH3BGRL	0		N.S		N.S
201313_at	ENO2	0	-1.2	3.13E-07	-1.33	6.04E-11
201314_at	STK25	0		N.S		N.S
201316_at	PSMA2	0		N.S		N.S
201319_at	MYL12A	0		N.S		N.S
201321_s_at	SMARCC2	1		N.S		N.S
201322_at	ATP5B	1		N.S		N.S
201323_at	EBNA1BP2	0		N.S		N.S
201327_s_at	CCT6A	0		N.S	-1.09	1.37E-05
201330_at	RARS	11		N.S		N.S
201339_s_at	SCP2	0		N.S	1.10	6.03E-06
201342_at	SNRPC	0		N.S	1.14	3.24E-02

Supplemental Table 2

201346_at	ADIPOR2	0		N.S		N.S
201349_at	SLC9A3R1	0		N.S		N.S
201350_at	FLOT2	0		N.S		N.S
201351_s_at	YME1L1	0		N.S		N.S
201356_at	SF3A1	0	1.2	7.99E-07		N.S
201358_s_at	COPB1	0		N.S		N.S
201360_at	CST3	0		N.S		N.S
201361_at	TMEM109	0		N.S	-1.15	1.15E-05
201363_s_at	IVNS1ABP	0	-1.2	8.09E-04		N.S
201364_s_at	OAZ2	0		N.S		N.S
201366_at	ANXA7	0		N.S		N.S
201368_at	ZFP36L2	0	1.6	3.22E-31		N.S
201371_s_at	CUL3	5		N.S		N.S
201375_s_at	PPP2CB	52		N.S	1.20	2.15E-19
201376_s_at	HNRNPF	0		N.S		N.S
201379_s_at	TPD52L2	0		N.S		N.S
201380_at	CRTAP	0		N.S		N.S
201382_at	CACYBP	3		N.S		N.S
201383_s_at	LOC100133166	0		N.S		N.S
201385_at	DHX15	0		N.S	-1.08	9.70E-03
201387_s_at	UCHL1	0		N.S		N.S
201388_at	PSMD3	0		N.S		N.S
201390_s_at	CSNK2B	0		N.S	1.18	5.78E-05
201391_at	TRAP1	2		N.S		N.S
201392_s_at	IGF2R	3		N.S		N.S
201395_at	RBM5	1		N.S		N.S
201396_s_at	SGTA	0		N.S		N.S
201397_at	PHGDH	0		N.S		N.S
201400_at	PSMB3	1		N.S		N.S
201403_s_at	MGST3	1		N.S		N.S
201405_s_at	COPS6	1		N.S		N.S
201407_s_at	PPP1CB	0		N.S		N.S
201411_s_at	PLEKHB2	0		N.S		N.S
201412_at	LRP10	0		N.S	1.32	7.30E-12
201413_at	HSD17B4	1	-1.1	1.71E-04	-1.17	1.23E-10
201415_at	GSS	3		N.S	1.17	5.05E-07
201417_at	SOX4	1		N.S		N.S
201420_s_at	WDR77	0		N.S		N.S
201422_at	IFI30	0		N.S		N.S
201423_s_at	CUL4A	45		N.S		N.S
201425_at	ALDH2	15		N.S		N.S
201426_s_at	VIM	2		N.S		N.S
201427_s_at	SEPP1	0		N.S		N.S
201429_s_at	RPL37A	0		N.S		N.S
201433_s_at	PTDSS1	0		N.S	-1.14	3.25E-08
201434_at	TTC1	0		N.S		N.S
201437_s_at	EIF4E	18		N.S	-1.19	5.43E-03
201439_at	GBF1	0		N.S		N.S
201441_at	COX6B1	0		N.S		N.S
201444_s_at	ATP6AP2	0		N.S	1.27	3.35E-14
201445_at	CNN3	0		N.S		N.S
201447_at	TIA1	3	-1.1	4.84E-03		N.S
201459_at	RUVBL2	8		N.S		N.S
201460_at	MAPKAPK2	10		N.S	-1.11	2.92E-02
201462_at	SCRN1	0		N.S	-1.26	2.51E-13
201463_s_at	LOC100133665	0		N.S		N.S
201466_s_at	JUN	480	1.8	2.60E-15		N.S
201468_s_at	NQO1	71		N.S	1.10	7.13E-03
201470_at	GSTO1	2		N.S		N.S
201472_at	VBP1	0		N.S	1.08	1.55E-05

Supplemental Table 2

201473_at	JUNB	8	1.3	1.20E-08		N.S
201477_s_at	RRM1	12	1.1	6.53E-04	1.21	5.32E-21
201478_s_at	DKC1	5		N.S	-1.13	7.89E-03
201480_s_at	SUPT5H	0		N.S		N.S
201484_at	SUPT4H1	0	1.1	2.03E-04	1.22	1.76E-15
201486_at	RCN2	0	-1.2	1.21E-10	1.14	4.72E-02
201487_at	CTSC	0		N.S	-1.29	5.95E-18
201489_at	PPIF	1	1.3	1.01E-17		N.S
201491_at	AHSA1	0	-1.1	2.30E-07	-1.32	6.36E-19
201492_s_at	RPL41	0		N.S		N.S
201493_s_at	PUM2	0	-1.2	1.84E-09		N.S
201494_at	PRCP	0		N.S		N.S
201499_s_at	USP7	11		N.S	-1.13	1.18E-07
201500_s_at	PPP1R11	0		N.S		N.S
201502_s_at	NFKBIA	0	1.5	2.36E-22		N.S
201507_at	PFDN1	0		N.S		N.S
201511_at	AAMP	0		N.S		N.S
201512_s_at	TOMM70A	0	-1.1	1.12E-04		N.S
201513_at	TSN	3	1.1	1.97E-02		N.S
201514_s_at	G3BP1	0		N.S		N.S
201516_at	SRM	18		N.S		N.S
201521_s_at	NCBP2	0		N.S		N.S
201526_at	ARF5	0		N.S		N.S
201527_at	ATP6V1F	0		N.S		N.S
201528_at	RPA1	160		N.S		N.S
201531_at	ZFP36	0		N.S	-1.63	2.20E-23
201532_at	PSMA3	0		N.S		N.S
201533_at	CTNNB1	66		N.S		N.S
201534_s_at	UBL3	0		N.S		N.S
201540_at	FHL1	0		N.S		N.S
201541_s_at	ZNHIT1	1		N.S		N.S
201543_s_at	SAR1A	0		N.S		N.S
201546_at	TRIP12	0		N.S		N.S
201553_s_at	LAMP1	0	1.1	1.41E-04	1.20	1.96E-16
201555_at	MCM3	8	1.1	9.52E-03		N.S
201557_at	VAMP2	1	1.4	3.45E-11		N.S
201561_s_at	CLSTN1	0		N.S		N.S
201563_at	SORD	0		N.S	1.28	1.85E-23
201564_s_at	FSCN1	0		N.S	-1.27	5.21E-07
201565_s_at	ID2	8	1.8	4.22E-20		N.S
201567_s_at	GOLGA4	0		N.S	1.20	3.17E-10
201568_at	UQCRQ	0		N.S		N.S
201570_at	SAMM50	0		N.S	1.18	5.77E-15
201574_at	ETF1	1	1.1	1.71E-03		N.S
201576_s_at	GLB1	0		N.S		N.S
201578_at	PODXL	2		N.S	2.74	7.06E-21
201580_s_at	TMX4	0		N.S		N.S
201582_at	SEC23B	0	-1.1	1.08E-02		N.S
201584_s_at	DDX39	0	1.1	3.52E-11	1.07	3.44E-03
201585_s_at	SFPQ	1	1.1	5.15E-03		N.S
201587_s_at	IRAK1	2		N.S		N.S
201588_at	TXNL1	0		N.S		N.S
201591_s_at	NISCH	0		N.S		N.S
201592_at	EIF3H	0		N.S		N.S
201594_s_at	PPP4R1	0		N.S	1.12	1.62E-04
201597_at	COX7A2	0		N.S		N.S
201598_s_at	INPPL1	0		N.S		N.S
201599_at	OAT	17		N.S		N.S
201600_at	PHB2	0		N.S		N.S
201604_s_at	PPP1R12A	0	-1.2	8.02E-03		N.S

201608_s_at	PWP1	0		N.S	-1.09	6.07E-04
201612_at	ALDH9A1	0		N.S		N.S
201613_s_at	AP1G2	0		N.S		N.S
201614_s_at	RUVBL1	7		N.S	-1.18	2.01E-08
201619_at	PRDX3	1		N.S		N.S
201620_at	MBTPS1	0		N.S		N.S
201624_at	DARS	0		N.S	-1.19	3.64E-12
201626_at	INSIG1	0		N.S	-1.62	5.20E-23
201628_s_at	RRAGA	0		N.S		N.S
201629_s_at	ACP1	0		N.S		N.S
201631_s_at	IER3	4	1.6	2.22E-22	1.59	2.32E-18
201632_at	EIF2B1	2		N.S		N.S
201633_s_at	CYB5B	0		N.S		N.S
201637_s_at	FXR1	0		N.S		N.S
201639_s_at	CPSF1	0		N.S		N.S
201641_at	BST2	0		N.S	-1.13	3.41E-02
201642_at	IFNGR2	0		N.S		N.S
201644_at	TSTA3	0		N.S		N.S
201647_s_at	SCARB2	0		N.S		N.S
201648_at	JAK1	3		N.S		N.S
201649_at	UBE2L6	0		N.S	-1.18	1.57E-08
201651_s_at	PACSIN2	0		N.S		N.S
201652_at	COPS5	6		N.S		N.S
201653_at	CNIH	0	-1.1	5.29E-05		N.S
201657_at	ARL1	0		N.S		N.S
201662_s_at	ACSL3	0		N.S	-1.37	3.58E-17
201663_s_at	SMC4	3		N.S		N.S
201666_at	TIMP1	3		N.S		N.S
201672_s_at	USP14	0		N.S		N.S
201673_s_at	GYS1	0		N.S	-1.17	1.70E-02
201677_at	C3orf37	0	1.1	2.86E-02	-1.20	1.24E-04
201682_at	PMPCB	0		N.S		N.S
201684_s_at	TOX4	0		N.S		N.S
201687_s_at	API5	0		N.S		N.S
201689_s_at	TPD52	0	-1.1	3.34E-03	-1.24	5.84E-11
201694_s_at	EGR1	59	-1.5	8.16E-07	-1.81	1.10E-16
201695_s_at	NP	99		N.S		N.S
201696_at	SFRS4	0		N.S	-1.18	1.32E-13
201697_s_at	DNMT1	35		N.S		N.S
201699_at	PSMC6	0		N.S		N.S
201700_at	CCND3	7		N.S	1.14	1.72E-05
201705_at	PSMD7	0		N.S		N.S
201707_at	PEX19	0		N.S		N.S
201709_s_at	NIPSNAP1	0		N.S	-1.13	3.96E-03
201710_at	MYBL2	4	1.1	3.02E-02		N.S
201713_s_at	RANBP2	3		N.S		N.S
201714_at	TUBG1	0		N.S		N.S
201715_s_at	ACIN1	1		N.S		N.S
201716_at	SNX1	0	-1.1	1.18E-05	1.11	1.19E-03
201717_at	MRPL49	0	1.2	1.16E-07	1.77	1.42E-46
201719_s_at	EPB41L2	0		N.S		N.S
201721_s_at	LAPTM5	1		N.S		N.S
201724_s_at	GALNT1	0		N.S		N.S
201725_at	CDC123	0		N.S		N.S
201726_at	ELAVL1	0		N.S	-1.07	1.82E-03
201731_s_at	TPR	8		N.S		N.S
201738_at	EIF1B	0	1.1	4.22E-02	1.18	2.92E-04
201739_at	SGK1	1	2.2	9.23E-25		N.S
201740_at	NDUFS3	1		N.S		N.S
201746_at	TP53	503		N.S		N.S

Supplemental Table 2

201747_s_at	SAFB	0		N.S		N.S
201751_at	JOSD1	0		N.S		N.S
201752_s_at	ADD3	0		N.S	-1.21	7.99E-04
201754_at	COX6C	0		N.S		N.S
201755_at	MCM5	5		N.S		N.S
201756_at	RPA2	39	1.1	5.20E-05	1.15	4.19E-12
201757_at	NDUFS5	0		N.S		N.S
201758_at	TSG101	3		N.S		N.S
201760_s_at	WSB2	0		N.S		N.S
201761_at	MTHFD2	0	1.1	2.26E-03	1.09	2.42E-05
201762_s_at	PSME2	0		N.S		N.S
201763_s_at	DAXX	8		N.S		N.S
201764_at	TMEM106C	0		N.S	1.10	2.83E-03
201768_s_at	CLINT1	0		N.S		N.S
201770_at	SNRPA	0		N.S		N.S
201771_at	SCAMP3	0		N.S		N.S
201772_at	AZIN1	1	-1.2	8.00E-06	-1.18	1.20E-04
201774_s_at	NCAPD2	0		N.S	-1.26	1.83E-05
201777_s_at	KIAA0494	0		N.S		N.S
201780_s_at	RNF13	0		N.S		N.S
201781_s_at	AIP	9	-1.1	3.55E-03	-1.13	4.39E-04
201783_s_at	RELA	57		N.S		N.S
201786_s_at	ADAR	3		N.S		N.S
201788_at	DDX42	1		N.S		N.S
201791_s_at	DHCR7	0		N.S	-1.45	1.82E-16
201795_at	LBR	0	-1.2	1.93E-05	-1.29	6.84E-13
201797_s_at	VARS	0		N.S		N.S
201800_s_at	OSBP	0		N.S		N.S
201802_at	SLC29A1	0		N.S		N.S
201803_at	POLR2B	0		N.S		N.S
201805_at	PRKAG1	0		N.S	1.18	3.18E-11
201807_at	VPS26A	0		N.S	1.08	4.81E-03
201810_s_at	SH3BP5	0		N.S	-1.24	5.79E-09
201812_s_at	C4orf46	0		N.S		N.S
201815_s_at	TBC1D5	0		N.S		N.S
201816_s_at	GBAS	0		N.S		N.S
201817_at	UBE3C	0		N.S		N.S
201818_at	LPCAT1	0	-1.2	3.17E-04	-1.49	1.75E-20
201819_at	SCARB1	0		N.S		N.S
201821_s_at	TIMM17A	0		N.S		N.S
201823_s_at	RNF14	0		N.S		N.S
201826_s_at	SCCPDH	0		N.S	-1.13	2.89E-03
201827_at	SMARCD2	0		N.S		N.S
201830_s_at	NET1	3		N.S		N.S
201833_at	HDAC2	11		N.S		N.S
201834_at	PRKAB1	0	1.4	1.92E-11	2.00	9.48E-35
201837_s_at	SUPT7L	0		N.S	1.15	3.48E-06
201840_at	NEDD8	14		N.S		N.S
201841_s_at	HSPB1	22		N.S		N.S
201845_s_at	RYBP	1	1.1	3.44E-04		N.S
201847_at	LIPA	1		N.S	1.15	2.04E-03
201849_at	BNIP3	6	-1.2	1.10E-09	-1.88	8.80E-21
201851_at	SH3GL1	0		N.S		N.S
201853_s_at	CDC25B	44		N.S	-1.34	2.14E-19
201854_s_at	ATMIN	4		N.S		N.S
201856_s_at	ZFR	0		N.S		N.S
201859_at	SRGN	0		N.S		N.S
201861_s_at	LRRFIP1	0		N.S	-1.24	7.46E-20
201863_at	FAM32A	0		N.S	1.10	3.28E-03
201864_at	GDI1	0		N.S		N.S

Supplemental Table 2

201870_at	TOMM34	0		N.S		N.S
201872_s_at	ABCE1	0		N.S	-1.13	1.47E-07
201874_at	MPZL1	0		N.S		N.S
201881_s_at	ARIH1	0	-1.1	8.29E-07	1.20	2.81E-13
201885_s_at	CYB5R3	0		N.S		N.S
201889_at	FAM3C	0		N.S	-1.15	1.56E-04
201890_at	RRM2	7	1.2	1.47E-09	1.21	1.07E-10
201892_s_at	IMPDH2	0		N.S		N.S
201895_at	ARAF	0		N.S		N.S
201896_s_at	PSRC1	3	-1.7	1.47E-20	-2.25	1.17E-28
201897_s_at	CKS1B	0		N.S		N.S
201899_s_at	UBE2A	7		N.S	1.14	7.44E-16
201900_s_at	AKR1A1	3		N.S		N.S
201903_at	UQCRC1	0		N.S		N.S
201908_at	DVL3	0		N.S	1.26	1.80E-12
201912_s_at	GSPT1	1		N.S	-1.23	3.05E-16
201913_s_at	COASY	0	-1.1	1.60E-03		N.S
201914_s_at	SEC63	1		N.S		N.S
201917_s_at	SLC25A36	0	-1.1	4.19E-03	-1.10	4.05E-02
201920_at	SLC20A1	0	1.2	6.95E-03	1.29	1.14E-15
201921_at	GNG10	0		N.S		N.S
201922_at	TINP1	0		N.S		N.S
201923_at	PRDX4	1		N.S		N.S
201924_at	AFF1	3	-1.2	2.04E-07	-1.11	3.70E-02
201925_s_at	CD55	0	1.2	2.17E-04	-1.62	2.69E-26
201928_at	PKP4	0	-1.2	4.60E-05		N.S
201930_at	MCM6	7	1.1	1.36E-03	-1.11	1.16E-03
201931_at	ETFA	1		N.S		N.S
201932_at	LRRC41	0		N.S	-1.29	2.91E-06
201933_at	CHMP1A	0		N.S		N.S
201934_at	WDR82	0		N.S		N.S
201936_s_at	EIF4G3	0		N.S	-1.22	1.64E-08
201937_s_at	DNPEP	0		N.S		N.S
201938_at	CDK2AP1	1		N.S		N.S
201939_at	PLK2	13	5.3	5.48E-33	4.83	1.41E-30
201944_at	HEXB	1		N.S	1.09	1.21E-04
201947_s_at	CCT2	3		N.S	-1.08	7.95E-05
201948_at	GNL2	1	1.1	1.71E-03		N.S
201952_at	ALCAM	0		N.S	1.17	1.91E-10
201953_at	CIB1	13		N.S		N.S
201954_at	ARPC1B	0		N.S		N.S
201955_at	CCNC	1		N.S		N.S
201956_s_at	GNPAT	0		N.S		N.S
201959_s_at	MYCBP2	0		N.S	-1.16	3.33E-04
201963_at	ACSL1	0		N.S	-1.14	6.75E-03
201964_at	SETX	4	-1.3	9.62E-11		N.S
201966_at	NDUFS2	0		N.S		N.S
201967_at	RBM6	0		N.S	-1.16	6.22E-05
201968_s_at	PGM1	1		N.S	-1.20	7.76E-08
201970_s_at	NASP	0	1.1	3.12E-08	-1.10	1.75E-04
201971_s_at	ATP6V1A	0		N.S		N.S
201973_s_at	C7orf28A	0		N.S		N.S
201975_at	CLIP1	0		N.S		N.S
201977_s_at	KIAA0141	0		N.S		N.S
201985_at	KIAA0196	0		N.S		N.S
201986_at	MED13	0		N.S		N.S
201990_s_at	CREBL2	0		N.S		N.S
201991_s_at	KIF5B	0		N.S		N.S
201994_at	MORF4L2	0	-1.1	4.76E-17	-1.11	1.28E-10
201997_s_at	SPEN	0		N.S		N.S

Supplemental Table 2

201999_s_at	DYNLT1	0	-1.2	1.59E-03		N.S
202001_s_at	NDUFA6	0		N.S		N.S
202007_at	NID1	0		N.S		N.S
202009_at	TWF2	0		N.S		N.S
202010_s_at	ZNF410	0		N.S	1.09	9.48E-04
202012_s_at	EXT2	0		N.S		N.S
202016_at	MEST	0		N.S		N.S
202019_s_at	LANCL1	0		N.S		N.S
202022_at	ALDOC	0		N.S	-1.20	6.23E-05
202024_at	ASNA1	1		N.S		N.S
202026_at	SDHD	0		N.S		N.S
202027_at	TMEM184B	0		N.S		N.S
202028_s_at	RPL38	1		N.S		N.S
202030_at	BCKDK	0		N.S	-1.23	7.73E-07
202033_s_at	RB1CC1	1		N.S		N.S
202038_at	UBE4A	0		N.S		N.S
202040_s_at	KDM5A	1		N.S		N.S
202041_s_at	FIBP	0		N.S		N.S
202042_at	HARS	1		N.S		N.S
202043_s_at	SMS	6		N.S		N.S
202050_s_at	ZMYM4	0	-1.3	1.39E-11		N.S
202053_s_at	ALDH3A2	0		N.S	-1.14	1.28E-03
202055_at	KPNA1	1		N.S	1.41	1.89E-17
202060_at	CTR9	0		N.S		N.S
202064_s_at	SEL1L	0		N.S		N.S
202069_s_at	IDH3A	0		N.S	-1.08	4.18E-02
202071_at	SDC4	1	1.1	4.74E-03		N.S
202072_at	HNRNPL	0		N.S		N.S
202074_s_at	OPTN	0		N.S	1.11	4.39E-05
202075_s_at	PLTP	0		N.S	1.25	3.95E-02
202076_at	BIRC2	4	-1.1	7.28E-04		N.S
202077_at	NDUFAB1	0		N.S		N.S
202078_at	COPS3	0	1.2	1.26E-10	1.09	7.58E-05
202080_s_at	TRAK1	0		N.S		N.S
202081_at	IER2	0		N.S	-1.57	3.00E-25
202083_s_at	SEC14L1	0		N.S	-1.29	2.96E-13
202085_at	TJP2	0		N.S		N.S
202086_at	MX1	4		N.S		N.S
202089_s_at	SLC39A6	0		N.S		N.S
202090_s_at	UQCR	0		N.S		N.S
202092_s_at	ARL2BP	0		N.S	1.23	3.12E-11
202093_s_at	PAF1	4		N.S		N.S
202095_s_at	BIRC5	39		N.S	-1.13	1.78E-04
202096_s_at	TSPO	0		N.S		N.S
202097_at	NUP153	0		N.S		N.S
202100_at	RALB	1		N.S		N.S
202103_at	BRD4	0		N.S		N.S
202104_s_at	SPG7	0		N.S		N.S
202105_at	IGBP1	1		N.S		N.S
202106_at	GOLGA3	0		N.S		N.S
202107_s_at	MCM2	41		N.S		N.S
202108_at	PEPD	0		N.S		N.S
202109_at	ARFIP2	0		N.S		N.S
202110_at	COX7B	0		N.S		N.S
202113_s_at	SNX2	0		N.S	1.21	3.52E-11
202115_s_at	NOC2L	0		N.S		N.S
202116_at	DPF2	0		N.S		N.S
202117_at	ARHGAP1	1		N.S		N.S
202119_s_at	CPNE3	0		N.S		N.S
202121_s_at	CHMP2A	0		N.S		N.S

202122_s_at	M6PRBP1	0		N.S	-1.16	1.05E-03
202123_s_at	ABL1	7		N.S		N.S
202124_s_at	TRAK2	0		N.S	1.18	3.29E-02
202126_at	PRPF4B	0		N.S	-1.16	6.12E-05
202128_at	KIAA0317	0		N.S	1.18	1.19E-05
202129_s_at	RIOK3	0		N.S	1.18	5.84E-04
202135_s_at	ACTR1B	0		N.S		N.S
202136_at	ZMYND11	1	-1.2	5.09E-10		N.S
202139_at	AKR7A2	0		N.S		N.S
202140_s_at	CLK3	0		N.S		N.S
202143_s_at	COPS8	0		N.S		N.S
202144_s_at	ADSL	0		N.S	-1.10	1.92E-11
202145_at	LY6E	0		N.S		N.S
202146_at	IFRD1	1		N.S	1.16	2.93E-02
202148_s_at	PYCR1	0		N.S	-1.15	1.08E-05
202149_at	NEDD9	1		N.S		N.S
202162_s_at	CNOT8	3		N.S		N.S
202166_s_at	PPP1R2	0		N.S	-1.24	3.36E-14
202167_s_at	MMS19	2		N.S	-1.17	2.60E-06
202168_at	TAF9	1	1.1	6.52E-09	1.14	2.06E-10
202170_s_at	AASDHPPT	0		N.S	1.14	2.10E-10
202173_s_at	VEZF1	0	1.2	1.76E-02		N.S
202174_s_at	PCM1	0		N.S		N.S
202179_at	BLMH	0		N.S	1.13	2.54E-04
202180_s_at	MVP	5		N.S		N.S
202181_at	KIAA0247	0		N.S	1.12	2.11E-02
202182_at	KAT2A	1		N.S		N.S
202184_s_at	NUP133	3	-1.1	1.12E-04	-1.12	7.26E-04
202185_at	PLOD3	0		N.S	-1.14	1.36E-03
202187_s_at	PPP2R5A	1		N.S		N.S
202188_at	NUP93	0		N.S		N.S
202190_at	CSTF1	0	-1.2	3.71E-16		N.S
202191_s_at	GAS7	1		N.S		N.S
202193_at	LIMK2	1		N.S	1.33	4.02E-15
202195_s_at	TMED5	0		N.S		N.S
202200_s_at	SRPK1	0	-1.1	1.93E-03	-1.09	1.53E-02
202201_at	BLVRB	0		N.S	1.33	4.44E-08
202205_at	VASP	1		N.S	-1.14	1.36E-02
202209_at	LSM3	0		N.S		N.S
202211_at	ARFGAP3	0		N.S		N.S
202212_at	PES1	1		N.S		N.S
202213_s_at	CUL4B	7		N.S		N.S
202215_s_at	NFYC	1		N.S	1.21	3.78E-12
202217_at	C21orf33	0		N.S		N.S
202218_s_at	FADS2	0		N.S	-1.37	8.62E-12
202220_at	KIAA0907	0	-1.3	1.69E-13		N.S
202221_s_at	EP300	18		N.S		N.S
202223_at	STT3A	1	-1.1	3.10E-07	-1.10	1.16E-02
202225_at	CRK	8		N.S	1.10	4.41E-02
202227_s_at	BRD8	0	-1.1	1.43E-05		N.S
202228_s_at	NPTN	0		N.S		N.S
202230_s_at	CHERP	0		N.S		N.S
202233_s_at	UQCRH	0		N.S		N.S
202234_s_at	SLC16A1	0		N.S	-1.27	4.24E-10
202239_at	PARP4	2		N.S		N.S
202240_at	PLK1	87	-1.2	5.86E-06	-2.87	4.33E-36
202241_at	TRIB1	0		N.S	-1.63	4.83E-14
202243_s_at	PSMB4	0		N.S		N.S
202246_s_at	CDK4	114		N.S	-1.09	1.88E-03
202249_s_at	WDR42A	0		N.S		N.S

202251_at	PRPF3	0		N.S		N.S
202252_at	RAB13	0		N.S		N.S
202254_at	SIPA1L1	0		N.S	-1.22	1.05E-06
202257_s_at	CD2BP2	0		N.S		N.S
202261_at	VPS72	0	-1.1	3.17E-03		N.S
202263_at	CYB5R1	0	1.1	3.82E-02	1.32	1.91E-20
202264_s_at	TOMM40	0		N.S		N.S
202265_at	BMI1	11		N.S	-1.14	1.18E-03
202266_at	TTRAP	1	-1.1	1.88E-04	1.12	5.39E-10
202268_s_at	NAE1	0		N.S		N.S
202270_at	GBP1	0		N.S	-1.42	2.68E-16
202272_s_at	FBXO28	0		N.S		N.S
202274_at	ACTG2	0		N.S		N.S
202275_at	G6PD	28		N.S		N.S
202276_at	SHFM1	4		N.S		N.S
202277_at	SPTLC1	0		N.S	1.13	2.34E-05
202279_at	C14orf2	0		N.S		N.S
202282_at	HSD17B10	0		N.S		N.S
202284_s_at	CDKN1A	1186	2.3	3.28E-28	2.69	7.89E-37
202288_at	FRAP1	0		N.S		N.S
202294_at	STAG1	1		N.S		N.S
202295_s_at	CTSH	1		N.S		N.S
202297_s_at	RER1	0		N.S		N.S
202298_at	NDUFA1	0		N.S		N.S
202300_at	HBXIP	0		N.S		N.S
202301_s_at	RSRC2	0		N.S		N.S
202306_at	POLR2G	0		N.S		N.S
202307_s_at	TAP1	3		N.S	1.33	8.30E-21
202309_at	MTHFD1	1		N.S		N.S
202313_at	PPP2R2A	0	-1.1	6.16E-08	-1.11	2.83E-07
202321_at	GGPS1	0	-1.1	2.00E-03		N.S
202323_s_at	ACBD3	0		N.S	1.28	8.33E-08
202325_s_at	ATP5J	0		N.S		N.S
202329_at	CSK	4	-1.1	2.46E-06	-1.45	5.31E-28
202330_s_at	UNG	55	1.2	2.29E-14	-1.18	1.23E-09
202331_at	BCKDHA	0		N.S		N.S
202336_s_at	PAM	33	-1.2	1.57E-05	-1.34	8.80E-10
202337_at	PMF1	0	1.1	1.30E-06	1.25	1.10E-09
202338_at	TK1	11		N.S	1.14	2.63E-05
202345_s_at	FABP5	2		N.S		N.S
202347_s_at	UBE2K	0		N.S		N.S
202349_at	TOR1A	0	1.1	7.15E-08	1.43	7.71E-39
202351_at	ITGAV	0	-1.2	6.88E-05	1.20	7.39E-09
202352_s_at	PSMD12	0	1.1	3.32E-07		N.S
202355_s_at	GTF2F1	0		N.S		N.S
202359_s_at	SNX19	0	-1.1	8.98E-03		N.S
202360_at	MAML1	1		N.S		N.S
202361_at	SEC24C	0		N.S		N.S
202362_at	RAP1A	0		N.S	-1.12	7.26E-03
202364_at	MXI1	2	-1.2	4.63E-02	-1.31	2.37E-05
202365_at	UNC119B	0		N.S	1.16	1.29E-08
202366_at	ACADS	0		N.S		N.S
202369_s_at	TRAM2	0		N.S	1.39	2.75E-21
202371_at	TCEAL4	0		N.S		N.S
202378_s_at	LEPROT	0		N.S		N.S
202381_at	ADAM9	0		N.S		N.S
202382_s_at	GNPDA1	0		N.S		N.S
202384_s_at	TCOF1	0		N.S		N.S
202386_s_at	KIAA0430	0		N.S		N.S
202388_at	RGS2	0		N.S	-1.69	5.48E-08

Supplemental Table 2

202391_at	BASP1	1		N.S	1.16	1.41E-06
202392_s_at	PISD	0		N.S		N.S
202393_s_at	KLF10	1	1.2	1.78E-07		N.S
202394_s_at	ABCF3	0		N.S		N.S
202395_at	NSF	6		N.S	1.22	9.17E-09
202396_at	TCERG1	0		N.S		N.S
202397_at	NUTF2	0		N.S		N.S
202399_s_at	AP3S2	0		N.S	1.16	2.45E-11
202402_s_at	CARS	6		N.S	1.15	2.66E-02
202406_s_at	TIAL1	10	-1.1	2.82E-05		N.S
202413_s_at	USP1	10	1.2	3.64E-12		N.S
202414_at	ERCC5	29		N.S		N.S
202415_s_at	HSPBP1	0		N.S		N.S
202417_at	KEAP1	13		N.S	1.15	3.08E-06
202418_at	YIF1A	0		N.S		N.S
202419_at	KDSR	0	1.1	9.19E-03	-1.10	4.90E-02
202420_s_at	DHX9	2		N.S		N.S
202421_at	IGSF3	0		N.S		N.S
202422_s_at	ACSL4	0	-1.2	1.10E-02	-1.23	2.00E-04
202424_at	MAP2K2	5		N.S		N.S
202427_s_at	BRP44	0		N.S	1.15	5.71E-03
202431_s_at	MYC	528		N.S	-2.14	7.94E-38
202432_at	PPP3CB	0		N.S	-1.14	4.19E-06
202433_at	SLC35B1	0		N.S	1.18	2.66E-09
202435_s_at	CYP1B1	63		N.S		N.S
202439_s_at	IDS	1		N.S	-1.11	2.52E-03
202446_s_at	PLSCR1	0		N.S	-1.15	3.15E-04
202447_at	DECR1	0		N.S		N.S
202449_s_at	RXRA	0		N.S	1.56	5.64E-21
202450_s_at	CTSK	0		N.S		N.S
202451_at	GTF2H1	1	-1.2	1.37E-10		N.S
202457_s_at	PPP3CA	0		N.S	-1.24	3.27E-06
202459_s_at	LPIN2	0		N.S		N.S
202461_at	EIF2B2	0		N.S	1.11	5.18E-06
202462_s_at	DDX46	0		N.S		N.S
202466_at	POLS	38		N.S	-1.12	3.45E-02
202468_s_at	CTNNAL1	0	1.2	1.55E-13	1.42	2.50E-23
202470_s_at	CPSF6	0		N.S		N.S
202471_s_at	IDH3G	0		N.S		N.S
202472_at	MPI	5		N.S	-1.22	6.55E-04
202474_s_at	HCFC1	11		N.S		N.S
202475_at	TMEM147	0		N.S	-1.16	1.78E-02
202480_s_at	DEDD	0		N.S		N.S
202486_at	AFG3L2	0		N.S		N.S
202487_s_at	H2AFV	0		N.S		N.S
202488_s_at	FXYD3	0		N.S		N.S
202492_at	ATG9A	0		N.S		N.S
202495_at	TBCC	0		N.S		N.S
202496_at	EDC4	0		N.S		N.S
202499_s_at	SLC2A3	2	-2.7	6.86E-28	-2.23	1.57E-26
202502_at	ACADM	0		N.S		N.S
202503_s_at	KIAA0101	2		N.S	1.19	5.12E-09
202505_at	SNRPB2	0		N.S		N.S
202506_at	SSFA2	0	-1.2	1.54E-03		N.S
202510_s_at	TNFAIP2	0		N.S		N.S
202513_s_at	PPP2R5D	0		N.S		N.S
202514_at	DLG1	1	-1.2	1.37E-03		N.S
202518_at	BCL7B	0	1.3	8.04E-09		N.S
202520_s_at	MLH1	173		N.S	1.09	1.58E-04
202521_at	CTCF	0		N.S		N.S

202522_at	PITPNB	0		N.S		N.S
202527_s_at	SMAD4	3		N.S		N.S
202528_at	GALE	8		N.S		N.S
202529_at	PRPSAP1	0		N.S		N.S
202530_at	MAPK14	4	-1.2	2.38E-03	1.17	1.27E-06
202531_at	IRF1	17	1.2	2.52E-04	1.21	5.26E-10
202532_s_at	DHFR	80		N.S	1.10	4.08E-02
202535_at	FADD	52	-1.2	5.95E-06		N.S
202538_s_at	CHMP2B	0		N.S		N.S
202540_s_at	HMGCR	0	-1.5	5.68E-15	-1.25	8.32E-08
202541_at	SCYE1	0	1.2	7.70E-08		N.S
202543_s_at	GMFB	0		N.S	1.13	2.47E-03
202545_at	PRKCD	30		N.S		N.S
202546_at	VAMP8	0		N.S		N.S
202548_s_at	ARHGEF7	0	-1.1	1.70E-02	-1.31	5.98E-13
202550_s_at	VAPB	0		N.S	1.07	2.94E-02
202552_s_at	CRIM1	0		N.S	-1.33	7.78E-05
202553_s_at	SYF2	0		N.S	1.22	8.57E-13
202556_s_at	MCRS1	0		N.S		N.S
202557_at	HSPA13	0		N.S		N.S
202567_at	SNRPD3	0		N.S		N.S
202569_s_at	MARK3	3		N.S		N.S
202573_at	CSNK1G2	0		N.S	1.22	7.52E-15
202578_s_at	DDX19A	0		N.S		N.S
202581_at	HSPA1A	2	-1.5	4.30E-12	1.41	2.59E-07
202583_s_at	RANBP9	1	1.1	8.94E-05		N.S
202584_at	NFX1	0	-1.2	3.56E-03		N.S
202592_at	BLOC1S1	0		N.S		N.S
202593_s_at	GDE1	0		N.S	1.15	2.86E-06
202594_at	LEPROTL1	0	-1.3	1.02E-19		N.S
202600_s_at	NRIP1	0		N.S		N.S
202602_s_at	HTATSF1	0		N.S		N.S
202605_at	GUSB	0	1.1	1.08E-03	1.10	5.78E-06
202609_at	EPS8	0		N.S		N.S
202613_at	CTPS	0	1.1	1.77E-03		N.S
202614_at	SLC30A9	0		N.S		N.S
202620_s_at	PLOD2	0		N.S		N.S
202621_at	IRF3	7		N.S	-1.20	2.42E-03
202622_s_at	ATXN2	0	-1.3	1.39E-11		N.S
202623_at	EAPP	0	1.1	1.85E-05	1.17	2.15E-09
202625_at	LYN	19		N.S	-1.21	6.97E-16
202631_s_at	APPBP2	0	-1.3	1.89E-15	1.15	2.50E-03
202632_at	DPH1	0		N.S		N.S
202633_at	TOPBP1	90		N.S		N.S
202635_s_at	POLR2K	0		N.S		N.S
202636_at	RNF103	0		N.S	-1.14	5.41E-03
202640_s_at	RANBP3	0		N.S		N.S
202643_s_at	TNFAIP3	4	1.4	1.07E-14	1.29	3.30E-13
202645_s_at	MEN1	12	-1.2	3.35E-03		N.S
202647_s_at	NRAS	7	1.1	1.79E-04		N.S
202650_s_at	KIAA0195	0		N.S		N.S
202651_at	LPGAT1	0		N.S	-1.22	4.78E-09
202653_s_at	MARCH7	0		N.S		N.S
202655_at	ARMET	0		N.S		N.S
202657_s_at	SERTAD2	0	1.1	8.68E-04	1.35	1.65E-14
202658_at	PEX11B	0	-1.3	1.05E-20	1.09	4.83E-05
202659_at	PSMB10	0		N.S		N.S
202662_s_at	ITPR2	1		N.S	1.22	2.77E-04
202665_s_at	WIPF1	0		N.S	-1.13	1.70E-02
202666_s_at	ACTL6A	0		N.S		N.S

202667_s_at	SLC39A7	0		N.S	1.16	1.41E-02
202670_at	MAP2K1	11	-1.1	1.61E-02	-1.24	2.69E-17
202672_s_at	ATF3	28	3.1	6.03E-35	2.24	1.14E-29
202673_at	DPM1	0		N.S		N.S
202674_s_at	LMO7	0		N.S		N.S
202677_at	RASA1	0	-1.3	3.74E-13		N.S
202678_at	GTF2A2	0		N.S		N.S
202680_at	GTF2E2	0	-1.1	6.63E-12	-1.41	5.22E-35
202681_at	USP4	0		N.S	-1.09	4.65E-03
202683_s_at	RNMT	0		N.S	-1.16	2.27E-04
202688_at	TNFSF10	109		N.S		N.S
202689_at	RBM15B	0		N.S		N.S
202691_at	SNRPD1	0		N.S		N.S
202693_s_at	STK17A	0		N.S		N.S
202696_at	OXSR1	0		N.S		N.S
202702_at	TRIM26	0	1.1	7.32E-03	1.28	2.50E-13
202703_at	DUSP11	1		N.S	1.14	5.31E-12
202704_at	TOB1	3	1.4	4.94E-13	1.98	3.10E-30
202705_at	CCNB2	11		N.S	-1.35	8.28E-22
202708_s_at	HIST2H2BE	0		N.S		N.S
202709_at	FMOD	1		N.S		N.S
202710_at	BET1	0	-1.2	5.15E-15		N.S
202713_s_at	KIAA0391	0		N.S	-1.14	1.78E-05
202715_at	CAD	65		N.S		N.S
202716_at	PTPN1	2		N.S		N.S
202720_at	TES	3		N.S		N.S
202721_s_at	GFPT1	0		N.S		N.S
202724_s_at	FOXO1	17	1.2	2.57E-05	1.12	3.77E-02
202726_at	LIG1	13		N.S	1.51	6.64E-20
202730_s_at	PDCD4	2		N.S	-1.19	3.40E-07
202732_at	PKIG	0		N.S		N.S
202733_at	P4HA2	0	-1.2	4.16E-08		N.S
202734_at	TRIP10	1		N.S		N.S
202736_s_at	LSM4	0		N.S		N.S
202738_s_at	PHKB	0	-1.1	2.66E-03	-1.33	2.79E-11
202740_at	ACY1	0		N.S		N.S
202742_s_at	PRKACB	1		N.S		N.S
202743_at	PIK3R3	0		N.S	1.30	1.69E-08
202745_at	USP8	0		N.S		N.S
202746_at	ITM2A	0		N.S	1.50	7.34E-19
202748_at	GBP2	0		N.S	-1.32	7.30E-12
202749_at	WRB	0		N.S		N.S
202750_s_at	TFIP11	0		N.S		N.S
202753_at	PSMD6	0		N.S		N.S
202754_at	R3HDM1	0		N.S	-1.11	2.86E-02
202757_at	COBRA1	0		N.S	-1.11	7.86E-03
202758_s_at	RFXANK	0		N.S		N.S
202759_s_at	AKAP2	0		N.S	-1.19	1.60E-07
202760_s_at	PALM2-AKAP2	0		N.S		N.S
202761_s_at	SYNE2	0		N.S	-1.15	4.92E-02
202762_at	ROCK2	2	-1.2	2.70E-14		N.S
202763_at	CASP3	1007		N.S	1.14	7.04E-05
202764_at	STIM1	0		N.S	-1.21	8.63E-04
202767_at	ACP2	1		N.S		N.S
202769_at	CCNG2	8	-1.7	8.63E-19	-1.52	2.99E-12
202771_at	FAM38A	0		N.S	-1.36	3.81E-12
202772_at	HMGCL	0		N.S		N.S
202775_s_at	SFRS8	0		N.S		N.S
202776_at	DNTTIP2	0	1.2	1.54E-12	-1.12	1.14E-06
202777_at	SHOC2	0		N.S		N.S

202779_s_at	LOC731049	0		N.S	-1.55	2.48E-18
202780_at	OXCT1	0		N.S		N.S
202781_s_at	INPP5K	0		N.S		N.S
202783_at	NNT	2		N.S		N.S
202785_at	NDUFA7	0		N.S		N.S
202786_at	STK39	1		N.S		N.S
202787_s_at	MAPKAPK3	0		N.S		N.S
202789_at	PLCG1	0		N.S		N.S
202793_at	LPCAT3	0		N.S	1.21	2.05E-03
202794_at	INPP1	0		N.S	1.93	8.74E-37
202796_at	SYNPO	0		N.S		N.S
202797_at	SACM1L	0	-1.4	4.53E-22		N.S
202798_at	SEC24B	0	-1.2	1.05E-08		N.S
202799_at	CLPP	6		N.S		N.S
202803_s_at	ITGB2	5		N.S	-1.25	1.23E-09
202804_at	ABCC1	2	-1.1	3.71E-02	-1.23	5.31E-11
202808_at	C10orf26	0	-1.1	2.23E-02	1.11	1.63E-04
202809_s_at	INTS3	3		N.S	-1.16	2.23E-03
202810_at	DRG1	0		N.S		N.S
202811_at	STAMBP	0	-1.1	1.61E-06		N.S
202813_at	TARBP1	0		N.S	-1.37	1.12E-12
202820_at	AHR	58		N.S	-1.21	1.07E-07
202824_s_at	TCEB1	0		N.S		N.S
202825_at	SLC25A4	0		N.S		N.S
202829_s_at	VAMP7	0	1.1	3.80E-03	1.19	2.97E-21
202832_at	GCC2	0		N.S		N.S
202837_at	TRAFD1	0		N.S		N.S
202838_at	FUCA1	0	1.2	2.67E-06	1.82	3.49E-25
202839_s_at	NDUFB7	0		N.S		N.S
202840_at	TAF15	1		N.S		N.S
202843_at	DNAJB9	0	1.3	7.76E-06		N.S
202844_s_at	RALBP1	3		N.S		N.S
202847_at	PCK2	0		N.S		N.S
202850_at	ABCD3	0		N.S		N.S
202853_s_at	RYK	1	-1.1	7.13E-06		N.S
202854_at	HPRT1	369		N.S		N.S
202856_s_at	SLC16A3	0	-1.3	8.05E-10	-2.16	3.64E-16
202858_at	U2AF1	0		N.S		N.S
202860_at	DENND4B	0		N.S		N.S
202862_at	FAH	1		N.S		N.S
202868_s_at	POP4	0		N.S	1.10	4.88E-02
202870_s_at	CDC20	33	-1.3	3.22E-09	-2.45	5.54E-34
202871_at	TRAF4	2	1.5	2.72E-18	1.62	9.66E-30
202874_s_at	ATP6V1C1	0	-1.1	6.61E-08	1.19	4.98E-10
202876_s_at	PBX2	0		N.S		N.S
202887_s_at	DDIT4	17	-1.3	2.18E-04	1.32	9.63E-07
202891_at	NIT1	3	-1.2	2.48E-19	1.11	8.22E-03
202892_at	CDC23	2		N.S	1.10	5.33E-03
202899_s_at	SFRS3	0	1.1	1.78E-02		N.S
2028_s_at	E2F1	182	1.2	3.16E-10		N.S
202900_s_at	NUP88	0		N.S	-1.24	8.75E-17
202902_s_at	CTSS	1		N.S		N.S
202904_s_at	LSM5	0		N.S		N.S
202908_at	WFS1	0		N.S	1.39	6.12E-21
202909_at	EPM2AIP1	0		N.S	1.42	6.03E-30
202910_s_at	CD97	0		N.S		N.S
202912_at	ADM	25	-1.8	2.65E-11	-1.40	1.30E-02
202915_s_at	FAM20B	0		N.S		N.S
202919_at	MOBKL3	0	1.2	1.16E-06		N.S
202922_at	GCLC	3	1.2	1.23E-08		N.S

202925_s_at	PLAGL2	1		N.S	1.13	1.58E-03
202926_at	NBAS	0		N.S		N.S
202927_at	PIN1	26		N.S		N.S
202929_s_at	DDT	39		N.S		N.S
202930_s_at	SUCLA2	0		N.S		N.S
202935_s_at	SOX9	2	1.6	7.11E-18		N.S
202939_at	ZMPSTE24	5		N.S	1.12	3.25E-03
202941_at	NDUFV2	0		N.S		N.S
202942_at	ETFB	0		N.S		N.S
202943_s_at	NAGA	0		N.S	-1.12	1.15E-03
202946_s_at	BTBD3	0		N.S		N.S
202947_s_at	GYPC	0		N.S		N.S
202949_s_at	FHL2	4	1.4	1.03E-08	3.34	6.39E-41
202950_at	CRYZ	1		N.S		N.S
202954_at	UBE2C	0		N.S	-1.55	1.38E-17
202957_at	HCLS1	0		N.S		N.S
202958_at	PTPN9	0		N.S		N.S
202960_s_at	MUT	41	-1.1	3.15E-02		N.S
202961_s_at	ATP5J2	0		N.S		N.S
202963_at	RFX5	0		N.S		N.S
202968_s_at	DYRK2	6		N.S	-1.20	1.24E-07
202974_at	MPP1	0		N.S		N.S
202976_s_at	RHOBTB3	0	-1.2	4.98E-06	-1.33	8.07E-07
202980_s_at	SIAH1	2		N.S		N.S
202982_s_at	ACOT1	0	1.1	1.72E-06	1.11	2.00E-02
202983_at	HLTF	5	-1.1	1.93E-05	-1.47	1.24E-23
202984_s_at	BAG5	0	1.1	1.17E-03		N.S
202990_at	PYGL	0		N.S		N.S
202991_at	STARD3	0		N.S		N.S
202996_at	POLD4	1		N.S		N.S
203004_s_at	MEF2D	0		N.S		N.S
203006_at	INPP5A	1		N.S		N.S
203011_at	IMPA1	0	1.1	4.30E-03		N.S
203013_at	ECD	60		N.S		N.S
203017_s_at	SSX2IP	0		N.S		N.S
203022_at	RNASEH2A	0		N.S		N.S
203024_s_at	C5orf15	0	-1.2	3.17E-18		N.S
203025_at	ARD1A	1		N.S		N.S
203026_at	ZBTB5	0	1.1	9.00E-04		N.S
203027_s_at	MVD	6		N.S	-1.22	1.17E-02
203028_s_at	CYBA	6		N.S		N.S
203031_s_at	UROS	0		N.S		N.S
203035_s_at	PIAS3	0		N.S	1.16	3.50E-02
203039_s_at	NDUFS1	0		N.S	1.09	8.07E-04
203040_s_at	HMBS	0		N.S	-1.12	1.09E-09
203041_s_at	LAMP2	0		N.S	1.11	4.78E-03
203043_at	ZBED1	0	1.3	6.99E-07	1.69	3.40E-19
203044_at	CHSY1	0		N.S		N.S
203045_at	NINJ1	1	1.5	3.63E-13	2.33	2.86E-34
203046_s_at	TIMELESS	14		N.S		N.S
203049_s_at	TTC37	0		N.S		N.S
203051_at	BAHD1	0		N.S		N.S
203053_at	BCAS2	1		N.S	1.20	4.43E-12
203054_s_at	TCTA	0		N.S	1.13	8.96E-06
203058_s_at	PAPSS2	0		N.S		N.S
203062_s_at	MDC1	147		N.S		N.S
203064_s_at	FOXK2	0		N.S		N.S
203065_s_at	CAV1	4		N.S		N.S
203067_at	PDHX	0		N.S	1.11	1.04E-06
203072_at	MYO1E	1		N.S	-1.27	5.57E-08

Supplemental Table 2

203073_at	COG2	0		N.S		N.S
203075_at	SMAD2	10		N.S		N.S
203079_s_at	CUL2	4		N.S		N.S
203080_s_at	BAZ2B	0		N.S		N.S
203082_at	BMS1	0		N.S		N.S
203085_s_at	TGFB1	83		N.S		N.S
203089_s_at	HTRA2	3		N.S		N.S
203090_at	SDF2	0		N.S		N.S
203093_s_at	TIMM44	0		N.S		N.S
203094_at	MAD2L1BP	0		N.S		N.S
203095_at	MTIF2	0	-1.1	1.83E-05		N.S
203102_s_at	MGAT2	0		N.S		N.S
203103_s_at	PRPF19	6		N.S		N.S
203105_s_at	DNM1L	0	-1.1	3.63E-07	-1.17	8.34E-06
203106_s_at	VPS41	0		N.S		N.S
203109_at	UBE2M	0		N.S		N.S
203112_s_at	WHSC2	0		N.S		N.S
203113_s_at	EEF1D	1		N.S	1.15	1.12E-05
203114_at	SSSCA1	0		N.S		N.S
203115_at	FECH	0		N.S		N.S
203117_s_at	PAN2	1	-1.2	9.84E-04		N.S
203119_at	CCDC86	0		N.S	-1.16	3.84E-06
203120_at	TP53BP2	5	1.3	1.08E-05		N.S
203124_s_at	SLC11A2	0		N.S	-1.24	2.88E-06
203127_s_at	SPTLC2	0		N.S	-1.14	3.87E-07
203133_at	SEC61B	0		N.S		N.S
203135_at	TBP	27	-1.1	4.94E-02		N.S
203136_at	RABAC1	0		N.S		N.S
203138_at	HAT1	4		N.S	1.10	4.91E-06
203139_at	DAPK1	1	-1.2	1.52E-08		N.S
203141_s_at	AP3B1	0		N.S		N.S
203145_at	SPAG5	1		N.S	-1.16	1.34E-02
203147_s_at	TRIM14	0		N.S	-1.25	2.11E-06
203150_at	RABEPK	0	-1.2	1.07E-05	-1.25	3.43E-14
203152_at	MRPL40	0		N.S		N.S
203153_at	IFIT1	2		N.S	-1.49	4.93E-06
203156_at	AKAP11	0	-1.2	5.31E-12		N.S
203158_s_at	GLS	0	-1.2	2.13E-04	-1.32	1.06E-11
203163_at	KATNB1	0		N.S		N.S
203165_s_at	SLC33A1	0	-1.2	5.54E-07		N.S
203169_at	RGP1	0		N.S		N.S
203171_s_at	RRP8	0	-1.2	1.25E-05		N.S
203173_s_at	C16orf62	0	-1.1	2.81E-03		N.S
203175_at	RHOG	0		N.S		N.S
203176_s_at	TFAM	19	1.2	7.72E-14	1.26	3.31E-18
203178_at	GATM	0		N.S	-1.27	6.87E-07
203179_at	GALT	1	-1.3	9.93E-24	-1.12	2.25E-02
203183_s_at	SMARCD1	0		N.S		N.S
203185_at	RASSF2	1		N.S		N.S
203186_s_at	S100A4	6		N.S		N.S
203188_at	B3GNT1	0	-1.2	1.78E-04	1.24	1.42E-07
203189_s_at	NDUFS8	0		N.S		N.S
203192_at	ABCB6	2		N.S	1.30	7.61E-10
203196_at	ABCC4	0		N.S	-1.24	1.15E-07
203197_s_at	C1orf123	0		N.S		N.S
203198_at	CDK9	7	-1.1	1.48E-02		N.S
203200_s_at	MTRR	3		N.S		N.S
203201_at	PMM2	0	1.3	1.27E-10		N.S
203202_at	KRR1	0		N.S	1.15	2.25E-06
203205_at	KDM4A	1		N.S	1.10	1.70E-02

203206_at	FAM53B	0		N.S		N.S
203208_s_at	MTFR1	1	-1.3	4.22E-06		N.S
203209_at	RFC5	13		N.S		N.S
203212_s_at	MTMR2	0		N.S		N.S
203218_at	MAPK9	0		N.S		N.S
203219_s_at	APRT	60		N.S		N.S
203223_at	RABEP1	0		N.S		N.S
203225_s_at	RFK	0		N.S	1.30	2.09E-08
203226_s_at	TSPAN31	0	-1.1	2.29E-06		N.S
203228_at	PAFAH1B3	0		N.S		N.S
203229_s_at	CLK2	1	-1.1	2.71E-02		N.S
203230_at	DVL1	0		N.S	-1.14	2.24E-03
203235_at	THOP1	0		N.S		N.S
203236_s_at	LGALS9	0		N.S	1.32	1.84E-04
203241_at	UVRAG	0		N.S	-1.17	1.05E-05
203244_at	PEX5	0		N.S	-1.16	4.60E-07
203245_s_at	NCRNA00094	0		N.S		N.S
203246_s_at	TUSC4	0	-1.1	1.62E-02		N.S
203247_s_at	ZNF24	1	1.1	6.35E-04		N.S
203250_at	RBM16	0	1.3	2.49E-14		N.S
203252_at	CDK2AP2	0		N.S		N.S
203253_s_at	HISPPD1	0		N.S	-1.14	2.57E-02
203254_s_at	TLN1	1		N.S		N.S
203258_at	DRAP1	0		N.S		N.S
203260_at	HDDC2	0		N.S		N.S
203261_at	DCTN6	0		N.S	1.18	2.22E-05
203262_s_at	FAM50A	0		N.S		N.S
203263_s_at	ARHGEF9	0	-1.2	9.43E-09		N.S
203266_s_at	MAP2K4	11		N.S	1.17	9.10E-07
203269_at	NSMAF	0		N.S		N.S
203270_at	DTYMK	1		N.S		N.S
203272_s_at	TUSC2	0		N.S	1.21	3.78E-06
203274_at	F8A1	0		N.S	1.32	1.38E-21
203275_at	IRF2	1	-1.2	7.70E-11	-1.10	4.12E-03
203276_at	LMNB1	1		N.S	-1.17	8.60E-04
203277_at	DFFA	0	-1.1	2.65E-02		N.S
203278_s_at	PHF21A	0	-1.2	4.43E-05		N.S
203279_at	EDEM1	1		N.S		N.S
203282_at	GBE1	0		N.S	-1.17	1.24E-13
203286_at	RNF44	0		N.S	-1.21	2.44E-06
203288_at	KIAA0355	0	-1.2	5.63E-08		N.S
203299_s_at	AP1S2	0		N.S	-1.17	3.87E-02
203301_s_at	DMTF1	1		N.S		N.S
203302_at	DCK	9	-1.2	4.22E-08		N.S
203303_at	DYNLT3	0		N.S		N.S
203304_at	BAMBI	5		N.S		N.S
203306_s_at	SLC35A1	0	-1.5	2.45E-23	1.27	1.09E-16
203307_at	GNL1	0		N.S		N.S
203309_s_at	HPS1	0		N.S		N.S
203310_at	STXBP3	0		N.S	1.21	5.24E-09
203311_s_at	ARF6	1	1.2	8.25E-05		N.S
203314_at	GTPBP6	0		N.S		N.S
203315_at	NCK2	3		N.S		N.S
203316_s_at	SNRPE	1		N.S		N.S
203318_s_at	ZNF148	0	-1.1	4.58E-02		N.S
203320_at	SH2B3	0	1.2	1.02E-09		N.S
203321_s_at	ADNP2	0	1.4	1.94E-17		N.S
203330_s_at	STX5	0		N.S	1.19	1.67E-03
203333_at	KIFAP3	0	-1.2	5.87E-06		N.S
203335_at	PHYH	0		N.S	1.17	1.00E-07

Supplemental Table 2

203336_s_at	ITGB1BP1	0		N.S		N.S
203338_at	PPP2R5E	0		N.S		N.S
203339_at	SLC25A12	0		N.S		N.S
203341_at	CEBPZ	0		N.S	-1.14	4.11E-07
203342_at	TIMM17B	0		N.S		N.S
203343_at	UGDH	0		N.S		N.S
203344_s_at	RBBP8	25		N.S		N.S
203350_at	AP1G1	0		N.S		N.S
203351_s_at	ORC4L	0	-1.1	3.12E-06		N.S
203356_at	CAPN7	0		N.S	1.22	1.77E-08
203361_s_at	MYCBP	1		N.S		N.S
203362_s_at	MAD2L1	17		N.S	-1.14	3.98E-03
203363_s_at	KIAA0652	0		N.S		N.S
203366_at	POLG	5		N.S	-1.16	2.29E-04
203367_at	DUSP14	1	1.3	3.40E-10	2.02	5.41E-35
203371_s_at	NDUFB3	0		N.S		N.S
203373_at	SOCS2	1	1.2	3.29E-08	1.56	2.54E-26
203375_s_at	TPP2	1	-1.1	1.36E-03	-1.20	4.03E-10
203377_s_at	CDC40	0		N.S		N.S
203378_at	PCF11	0		N.S		N.S
203379_at	RPS6KA1	2		N.S	1.45	2.72E-20
203384_s_at	GOLGA1	0	-1.2	8.99E-05		N.S
203385_at	DGKA	0		N.S		N.S
203387_s_at	TBC1D4	0		N.S	-1.19	5.96E-05
203391_at	FKBP2	0		N.S		N.S
203396_at	PSMA4	0		N.S		N.S
203401_at	PRPS2	0		N.S	1.07	3.77E-03
203404_at	ARMCX2	0		N.S		N.S
203405_at	PSMG1	0		N.S		N.S
203406_at	MFAP1	0	-1.1	2.22E-06		N.S
203409_at	DDB2	106	1.3	3.92E-13	2.62	5.76E-49
203410_at	AP3M2	0	-1.4	8.76E-11	-1.29	9.95E-12
203411_s_at	LMNA	9		N.S	1.32	1.37E-09
203412_at	LZTR1	0		N.S		N.S
203414_at	MMD	1		N.S		N.S
203415_at	PDCD6	1		N.S		N.S
203416_at	CD53	2		N.S	-1.09	3.25E-03
203420_at	FAM8A1	0	-1.1	5.96E-06		N.S
203422_at	POLD1	8		N.S		N.S
203427_at	ASF1A	11	-1.2	1.03E-06		N.S
203429_s_at	C1orf9	0	1.2	1.23E-03		N.S
203430_at	HEBP2	0		N.S		N.S
203433_at	MTHFS	0		N.S		N.S
203436_at	RPP30	0		N.S		N.S
203437_at	TMEM11	0		N.S		N.S
203445_s_at	CTDSP2	0		N.S		N.S
203449_s_at	TERF1	2	-1.3	5.59E-18		N.S
203454_s_at	ATOX1	0		N.S		N.S
203455_s_at	SAT1	1		N.S		N.S
203457_at	STX7	0	-1.2	7.81E-10	-1.34	1.39E-11
203458_at	SPR	13		N.S		N.S
203459_s_at	VPS16A	0		N.S		N.S
203460_s_at	PSEN1	7		N.S	1.16	3.98E-07
203465_at	MRPL19	0		N.S	-1.15	1.71E-05
203466_at	MPV17	0		N.S		N.S
203468_at	CDK10	0		N.S		N.S
203471_s_at	PLEK	0	1.1	1.17E-02	1.22	3.46E-17
203474_at	IQGAP2	0		N.S	-1.33	2.16E-15
203476_at	TPBG	0		N.S		N.S
203478_at	NDUFC1	0		N.S		N.S

203480_s_at	OTUD4	0	1.1	3.95E-02	-1.17	5.66E-05
203482_at	FAM178A	0	-1.3	7.22E-07		N.S
203484_at	SEC61G	0		N.S		N.S
203487_s_at	ARMC8	0		N.S		N.S
203493_s_at	CEP57	0	-1.2	2.83E-06		N.S
203495_at	LRRC14	0		N.S		N.S
203500_at	GCDH	0		N.S		N.S
203502_at	BPGM	0		N.S		N.S
203508_at	TNFRSF1B	0		N.S	-1.19	1.33E-06
203511_s_at	TRAPPC3	0		N.S		N.S
203513_at	SPG11	0	-1.2	3.10E-06	-1.22	7.28E-07
203514_at	MAP3K3	1		N.S		N.S
203515_s_at	PMVK	0		N.S		N.S
203517_at	MTX2	0		N.S		N.S
203519_s_at	UPF2	1		N.S		N.S
203521_s_at	ZNF318	0	-1.2	5.84E-11	-1.53	1.21E-18
203522_at	CCS	5		N.S		N.S
203523_at	LSP1	0		N.S		N.S
203526_s_at	APC	148		N.S		N.S
203528_at	SEMA4D	0		N.S	-1.31	1.66E-12
203529_at	PPP6C	0	1.1	2.51E-08	1.16	4.71E-19
203530_s_at	STX4	0	-1.1	4.25E-02		N.S
203531_at	CUL5	4		N.S		N.S
203534_at	LSM1	0		N.S		N.S
203537_at	PRPSAP2	0	-1.1	2.79E-02	-1.08	4.01E-03
203538_at	CAMLG	0		N.S	1.15	4.59E-04
203544_s_at	STAM	1	1.2	2.40E-10	1.33	1.33E-23
203545_at	ALG8	0	-1.1	3.00E-06	-1.09	1.63E-04
203546_at	IPO13	0		N.S	1.14	3.12E-04
203550_s_at	C1orf2	0		N.S	-1.24	1.14E-05
203552_at	MAP4K5	0		N.S		N.S
203556_at	ZHX2	0	-1.2	1.04E-05	-1.44	5.31E-12
203557_s_at	PCBD1	0		N.S		N.S
203560_at	GGH	7		N.S		N.S
203562_at	FEZ1	0		N.S	1.85	2.25E-18
203564_at	FANCG	49		N.S		N.S
203565_s_at	MNAT1	0		N.S		N.S
203566_s_at	AGL	0		N.S		N.S
203567_s_at	TRIM38	0		N.S	1.16	6.83E-03
203569_s_at	OFD1	0		N.S		N.S
203572_s_at	TAF6	13		N.S		N.S
203573_s_at	RABGGTA	0		N.S	1.52	1.90E-31
203574_at	NFIL3	1		N.S	-1.41	1.01E-13
203575_at	CSNK2A2	0		N.S	1.12	9.41E-05
203577_at	GTF2H4	0	-1.2	2.73E-09		N.S
203579_s_at	SLC7A6	0		N.S	1.70	4.82E-31
203581_at	RAB4A	0		N.S		N.S
203583_at	UNC50	0		N.S	1.21	8.57E-10
203584_at	TTC35	0	-1.2	2.39E-11		N.S
203588_s_at	TFDP2	1		N.S	-1.13	1.83E-05
203592_s_at	FSTL3	0		N.S		N.S
203593_at	CD2AP	0		N.S		N.S
203599_s_at	WBP4	0		N.S		N.S
203600_s_at	C4orf8	0	-1.1	7.84E-05		N.S
203604_at	ZNF516	0	-1.2	1.47E-02		N.S
203605_at	SRP54	0		N.S	1.09	1.99E-02
203606_at	NDUFS6	0		N.S		N.S
203607_at	INPP5F	0		N.S	-1.33	6.16E-06
203608_at	ALDH5A1	0		N.S		N.S
203611_at	TERF2	28	-1.2	9.09E-11	1.13	1.70E-05

203612_at	BYSL	1		N.S		N.S
203613_s_at	NDUFB6	0		N.S		N.S
203614_at	UTP14C	0	-1.2	2.03E-05	1.19	1.55E-16
203616_at	POLB	21	-1.1	1.09E-03		N.S
203620_s_at	FCHSD2	0	-1.2	2.33E-08		N.S
203621_at	NDUFB5	0		N.S		N.S
203622_s_at	PNO1	0	1.1	6.76E-06	-1.11	1.89E-05
203630_s_at	COG5	0		N.S	-1.15	2.50E-02
203635_at	DSCR3	0		N.S		N.S
203643_at	ERF	1		N.S		N.S
203644_s_at	MON1B	0		N.S		N.S
203647_s_at	FDX1	0		N.S		N.S
203648_at	TATDN2	0		N.S		N.S
203650_at	PROCR	1		N.S	1.99	4.75E-19
203651_at	ZFYVE16	0	-1.2	3.54E-03	1.35	1.66E-11
203652_at	MAP3K11	0		N.S		N.S
203653_s_at	COIL	88		N.S		N.S
203655_at	XRCC1	356		N.S		N.S
203656_at	FIG4	0		N.S		N.S
203658_at	SLC25A20	0	-1.1	4.55E-05		N.S
203659_s_at	TRIM13	0	1.2	1.44E-09	-1.16	5.31E-12
203660_s_at	PCNT	2		N.S	1.14	1.37E-05
203662_s_at	TMOD1	0		N.S		N.S
203665_at	HMOX1	111	1.3	3.39E-13	1.31	2.30E-14
203668_at	MAN2C1	0		N.S		N.S
203674_at	HELZ	0		N.S		N.S
203675_at	NUCB2	0		N.S	-1.15	8.19E-04
203677_s_at	TARBP2	0		N.S		N.S
203678_at	MTMR15	1	-1.2	2.91E-02		N.S
203679_at	TMED1	0	1.3	1.94E-10		N.S
203686_at	MPG	59		N.S		N.S
203688_at	PKD2	0		N.S	1.29	5.84E-15
203689_s_at	FMR1	6	-1.1	3.00E-03		N.S
203693_s_at	E2F3	13		N.S		N.S
203694_s_at	DHX16	0		N.S		N.S
203695_s_at	DFNA5	2		N.S	-1.09	1.23E-02
203701_s_at	TRMT1	0		N.S	-1.13	6.56E-03
203707_at	ZNF263	0	1.2	3.67E-03		N.S
203711_s_at	HIBCH	0		N.S	-1.17	4.52E-03
203712_at	KIAA0020	0		N.S	-1.09	6.53E-03
203715_at	TBCE	0		N.S	-1.29	7.65E-15
203718_at	PNPLA6	0		N.S		N.S
203720_s_at	ERCC1	272		N.S		N.S
203725_at	GADD45A	135	2.1	4.48E-27	2.79	2.54E-32
203728_at	BAK1	35		N.S	1.33	8.48E-18
203729_at	EMP3	0		N.S		N.S
203731_s_at	ZKSCAN5	0		N.S		N.S
203732_at	TRIP4	0		N.S	1.12	1.69E-06
203733_at	DEXI	0		N.S	1.18	1.77E-04
203734_at	FOXJ2	0		N.S		N.S
203737_s_at	PPRC1	0	1.3	3.02E-08	-1.33	2.11E-13
203738_at	C5orf22	0		N.S		N.S
203739_at	ZNF217	2	-1.3	9.79E-13		N.S
203740_at	MPHOSPH6	0		N.S		N.S
203741_s_at	ADCY7	0	-1.3	2.22E-15	-1.28	3.10E-12
203743_s_at	TDG	19	1.2	7.78E-09		N.S
203744_at	HMGB3	1		N.S		N.S
203745_at	HCCS	20		N.S	1.10	1.76E-02
203752_s_at	JUND	5	1.5	1.61E-20		N.S
203755_at	BUB1B	2	-1.4	1.20E-16	-1.40	1.27E-14

Supplemental Table 2

203758_at	CTSO	0	1.2	4.38E-08	1.35	6.20E-20
203762_s_at	DYNC2LI1	0		N.S	-1.15	7.31E-03
203764_at	DLGAP5	0	-1.1	1.63E-03	-1.90	3.50E-30
203765_at	GCA	1		N.S		N.S
203774_at	MTR	15		N.S		N.S
203775_at	SLC25A13	0		N.S	-1.16	3.61E-10
203776_at	GPKOW	0		N.S	1.09	3.38E-02
203777_s_at	RPS6KB2	0		N.S		N.S
203778_at	MANBA	0		N.S		N.S
203781_at	MRPL33	0		N.S		N.S
203787_at	SSBP2	2	-1.1	3.95E-03	-1.29	1.31E-16
203790_s_at	HRSP12	0		N.S		N.S
203791_at	DMXL1	0	-1.2	1.54E-02	-1.25	3.64E-05
203799_at	CD302	0		N.S		N.S
203800_s_at	MRPS14	0		N.S		N.S
203803_at	PCYOX1	0		N.S		N.S
203804_s_at	CROP	46		N.S		N.S
203805_s_at	FANCA	64		N.S		N.S
203810_at	DNAJB4	1	-1.2	2.19E-03	1.58	1.30E-21
203814_s_at	NQO2	3		N.S		N.S
203816_at	DGUOK	0		N.S		N.S
203817_at	GUCY1B3	0	-1.4	3.91E-11	-1.47	1.30E-10
203818_s_at	SF3A3	0	1.1	8.75E-10	1.27	2.95E-25
203820_s_at	IGF2BP3	0	-1.2	5.24E-17	-1.32	1.09E-20
203822_s_at	ELF2	0		N.S	-1.13	7.88E-05
203825_at	BRD3	0	-1.1	2.23E-03		N.S
203827_at	WIPI1	0		N.S	-1.29	1.81E-12
203829_at	ELP4	0		N.S		N.S
203830_at	C17orf75	0		N.S	1.15	1.75E-03
203831_at	R3HDM2	0	-1.1	7.65E-03		N.S
203832_at	SNRPF	1		N.S		N.S
203836_s_at	MAP3K5	4	-1.2	2.81E-04	-1.19	7.14E-04
203840_at	BLZF1	0	-1.2	3.05E-02		N.S
203843_at	RPS6KA3	0	-1.1	6.55E-03		N.S
203845_at	KAT2B	0		N.S		N.S
203846_at	TRIM32	0	1.2	5.02E-04	1.57	4.11E-34
203852_s_at	SMN1	3	1.1	2.02E-02		N.S
203855_at	WDR47	0		N.S	1.13	7.19E-04
203856_at	VRK1	0		N.S		N.S
203857_s_at	PDIA5	0		N.S		N.S
203858_s_at	COX10	0		N.S		N.S
203860_at	PCCA	0		N.S	-1.31	1.71E-14
203868_s_at	VCAM1	0		N.S		N.S
203869_at	USP46	1	1.2	2.77E-06		N.S
203871_at	SENP3	0		N.S		N.S
203879_at	PIK3CD	0	-1.2	3.61E-03	-1.15	4.39E-03
203880_at	COX17	0		N.S		N.S
203881_s_at	DMD	6		N.S		N.S
203883_s_at	RAB11FIP2	0	1.1	1.12E-03		N.S
203885_at	RAB21	0		N.S	1.19	1.74E-13
203890_s_at	DAPK3	1		N.S		N.S
203892_at	WFDC2	0		N.S	1.19	3.34E-02
203894_at	TUBG2	0		N.S		N.S
203897_at	LYRM1	0		N.S	1.32	2.19E-19
203899_s_at	CRCP	0		N.S		N.S
203900_at	KIAA0467	0		N.S		N.S
203903_s_at	HEPH	0		N.S		N.S
203905_at	PARN	1		N.S	1.25	1.94E-14
203909_at	SLC9A6	0		N.S		N.S
203910_at	ARHGAP29	1		N.S		N.S

203916_at	NDST2	0		N.S		N.S
203921_at	CHST2	0		N.S		N.S
203923_s_at	CYBB	6		N.S	-1.53	1.39E-18
203925_at	GCLM	6		N.S	1.18	1.73E-09
203927_at	NFKBIE	0		N.S		N.S
203931_s_at	MRPL12	0		N.S		N.S
203932_at	HLA-DMB	0	1.1	3.01E-02	-1.18	1.67E-05
203933_at	RAB11FIP3	0		N.S		N.S
203935_at	ACVR1	0		N.S	1.30	4.63E-06
203936_s_at	MMP9	1		N.S		N.S
203939_at	NT5E	0		N.S	-1.25	1.68E-02
203941_at	INTS9	0	-1.1	1.52E-02		N.S
203943_at	KIF3B	0		N.S	1.18	6.80E-07
203945_at	ARG2	2		N.S		N.S
203947_at	CSTF3	0	-1.3	1.28E-21	1.39	9.33E-27
203957_at	E2F6	4		N.S		N.S
203958_s_at	ZBTB40	0		N.S		N.S
203960_s_at	HSPB11	0		N.S		N.S
203964_at	NMI	0		N.S	-1.17	1.34E-14
203966_s_at	PPM1A	0	1.1	4.61E-02	1.22	2.47E-15
203967_at	CDC6	40	1.4	1.33E-19		N.S
203970_s_at	PEX3	0		N.S	-1.16	2.75E-07
203971_at	SLC31A1	0	-1.2	4.70E-04	-1.32	1.59E-12
203973_s_at	CEBPD	1	1.3	2.25E-02		N.S
203974_at	HDHD1A	0	-1.1	1.73E-08	-1.12	1.27E-05
203975_s_at	CHAF1A	5	1.3	7.38E-07		N.S
203978_at	NUBP1	0		N.S		N.S
203983_at	TSNAX	1		N.S		N.S
203984_s_at	CASP9	274		N.S		N.S
203985_at	ZNF212	0		N.S		N.S
203987_at	FZD6	0	1.2	8.08E-07	1.52	2.62E-16
203988_s_at	FUT8	0	-1.3	2.45E-16		N.S
203990_s_at	KDM6A	0		N.S		N.S
204001_at	SNAPC3	0	-1.3	2.34E-18	-1.13	1.78E-03
204003_s_at	NUPL2	0	-1.2	2.99E-07		N.S
204004_at	PAWR	0	-1.2	3.23E-07	-1.22	2.59E-06
204015_s_at	DUSP4	2		N.S	-1.26	1.87E-04
204016_at	LARS2	0		N.S		N.S
204019_s_at	SH3YL1	0		N.S	1.12	1.16E-04
204021_s_at	PURA	2	-1.2	4.17E-07	1.12	1.07E-02
204023_at	RFC4	12		N.S		N.S
204025_s_at	PDCD2	0		N.S		N.S
204026_s_at	ZWINT	0		N.S	1.11	1.50E-07
204027_s_at	METTL1	0		N.S		N.S
204028_s_at	RABGAP1	0	-1.2	2.65E-04		N.S
204030_s_at	SCHIP1	0		N.S		N.S
204032_at	BCAR3	0		N.S	-1.40	4.46E-08
204033_at	TRIP13	1		N.S	-1.21	1.10E-12
204034_at	ETHE1	0		N.S	1.16	3.79E-03
204038_s_at	LPAR1	0		N.S		N.S
204044_at	QPRT	0		N.S		N.S
204045_at	TCEAL1	3		N.S		N.S
204054_at	PTEN	87		N.S		N.S
204057_at	IRF8	0	-1.1	9.28E-06	-1.32	2.69E-16
204059_s_at	ME1	0		N.S	1.22	3.04E-10
204061_at	PRKX	0		N.S	1.48	9.09E-26
204064_at	THOC1	2		N.S		N.S
204065_at	CHST10	0		N.S		N.S
204067_at	SUOX	0		N.S		N.S
204068_at	STK3	0	-1.3	2.49E-10		N.S

204070_at	RARRES3	0		N.S		N.S
204071_s_at	TOPORS	2	1.2	9.11E-08		N.S
204076_at	ENTPD4	0		N.S		N.S
204079_at	TPST2	0	-1.1	2.37E-02		N.S
204080_at	TOE1	0	1.1	8.15E-05		N.S
204082_at	PBX3	0	-1.2	6.99E-10		N.S
204085_s_at	CLN5	0		N.S	1.16	5.48E-05
204088_at	P2RX4	0		N.S		N.S
204091_at	PDE6D	0		N.S		N.S
204093_at	CCNH	4		N.S		N.S
204094_s_at	TSC22D2	0	1.3	2.57E-09		N.S
204098_at	RBMX2	0		N.S	1.10	4.22E-02
204102_s_at	EEF2	0		N.S		N.S
204103_at	CCL4	32	1.4	1.03E-03	-1.65	1.86E-14
204106_at	TESK1	0		N.S		N.S
204108_at	NFYA	8		N.S		N.S
204115_at	GNG11	0		N.S		N.S
204116_at	IL2RG	2		N.S	-1.19	2.61E-02
204118_at	CD48	0		N.S		N.S
204120_s_at	ADK	2		N.S		N.S
204125_at	NDUFAF1	0		N.S		N.S
204126_s_at	CDC45L	9		N.S	1.15	3.46E-05
204128_s_at	RFC3	9	1.1	4.95E-03	1.20	1.55E-06
204131_s_at	FOXO3	22	1.3	1.23E-10		N.S
204133_at	RRP9	0		N.S	-1.18	4.97E-05
204135_at	FILIP1L	0		N.S		N.S
204137_at	GPR137B	0	-1.1	2.42E-07	-1.21	8.62E-12
204141_at	TUBB2A	0		N.S		N.S
204142_at	ENOSF1	0		N.S		N.S
204144_s_at	PIGQ	0		N.S		N.S
204145_at	FRG1	0		N.S		N.S
204146_at	RAD51AP1	7		N.S	1.14	5.57E-06
204153_s_at	MFNG	0		N.S		N.S
204156_at	KIAA0999	0		N.S		N.S
204158_s_at	TCIRG1	0		N.S		N.S
204160_s_at	ENPP4	0	-1.1	7.99E-03		N.S
204162_at	NDC80	1	-1.2	5.04E-05	-1.15	1.14E-03
204164_at	SIPA1	0		N.S	-1.58	1.29E-15
204165_at	WASF1	0		N.S		N.S
204168_at	MGST2	0		N.S		N.S
204170_s_at	CKS2	2	-1.3	8.05E-07	-1.40	9.47E-14
204171_at	RPS6KB1	0		N.S		N.S
204172_at	CPOX	0	-1.1	1.05E-04		N.S
204173_at	MYL6B	0		N.S		N.S
204174_at	ALOX5AP	1		N.S	-1.24	4.56E-05
204175_at	ZNF593	0		N.S		N.S
204181_s_at	ZBTB43	0	1.2	1.69E-06	1.12	4.42E-03
204186_s_at	PPID	2		N.S		N.S
204190_at	USPL1	0		N.S	1.23	3.02E-07
204192_at	CD37	1		N.S		N.S
204198_s_at	RUNX3	5		N.S		N.S
204201_s_at	PTPN13	1		N.S		N.S
204203_at	CEBPG	0	1.3	1.56E-14		N.S
204204_at	SLC31A2	0	1.3	6.48E-04	1.34	3.88E-07
204205_at	APOBEC3G	1		N.S	1.43	9.37E-27
204206_at	MNT	30	1.5	5.97E-15	-1.54	2.66E-13
204208_at	RNGTT	0	-1.2	4.08E-07		N.S
204209_at	PCYT1A	0		N.S		N.S
204212_at	ACOT8	0		N.S		N.S
204215_at	C7orf23	0		N.S	-1.24	2.22E-17

204216_s_at	ZC3H14	0		N.S	-1.09	2.44E-02
204218_at	C11orf51	0		N.S		N.S
204219_s_at	PSMC1	2	1.1	1.07E-02		N.S
204220_at	GMFG	0		N.S		N.S
204222_s_at	GLIPR1	0		N.S	-1.26	3.44E-10
204224_s_at	GCH1	0	1.1	9.63E-04	1.80	1.07E-44
204226_at	STAU2	0		N.S	-1.22	1.71E-07
204228_at	PPIH	0		N.S		N.S
204233_s_at	CHKA	1		N.S		N.S
204234_s_at	ZNF195	0	1.2	2.73E-12	1.55	3.03E-30
204241_at	ACOX3	0		N.S		N.S
204243_at	RLF	0	-1.2	4.41E-08	-1.31	5.31E-12
204244_s_at	DBF4	27	-1.1	4.44E-03	-1.20	1.09E-08
204245_s_at	RPP14	0	1.1	4.28E-04	1.10	4.16E-04
204246_s_at	DCTN3	0		N.S		N.S
204247_s_at	CDK5	19		N.S		N.S
204249_s_at	LMO2	2		N.S	-1.25	3.83E-06
204258_at	CHD1	1		N.S	-1.14	2.05E-02
204263_s_at	CPT2	0		N.S	1.28	7.24E-10
204265_s_at	GPSM3	0		N.S	-1.14	3.54E-02
204269_at	PIM2	1		N.S		N.S
204275_at	SOLH	0		N.S		N.S
204276_at	TK2	2		N.S		N.S
204278_s_at	EBAG9	1		N.S	-1.09	1.38E-04
204279_at	PSMB9	0		N.S		N.S
204286_s_at	PMAIP1	31	1.8	5.60E-31	1.60	5.26E-28
204291_at	ZNF518A	0		N.S		N.S
204295_at	SURF1	0		N.S	1.19	2.39E-02
204297_at	PIK3C3	0	-1.2	7.39E-07		N.S
204299_at	FUSIP1	0		N.S		N.S
204300_at	PET112L	0		N.S		N.S
204305_at	MIPEP	0		N.S		N.S
204308_s_at	TECPR2	0	-1.3	7.32E-06	1.29	2.27E-07
204319_s_at	RGS10	0		N.S		N.S
204327_s_at	ZNF202	0	1.2	4.94E-05		N.S
204331_s_at	MRPS12	0		N.S		N.S
204332_s_at	AGA	11		N.S	1.17	5.57E-11
204334_at	KLF7	0	-1.1	5.37E-03	-1.26	3.13E-12
204335_at	CCDC94	0	1.5	3.36E-16		N.S
204336_s_at	RGS19	0	-1.2	6.71E-04	-1.22	2.52E-11
204342_at	SLC25A24	0		N.S		N.S
204346_s_at	RASSF1	10		N.S		N.S
204347_at	AK3L1	0		N.S	-1.41	9.84E-09
204350_s_at	MED7	0	-1.3	2.22E-15		N.S
204352_at	TRAF5	2	-1.3	1.09E-16	-1.65	1.20E-28
204354_at	POT1	45	-1.2	3.52E-08		N.S
204369_at	PIK3CA	4		N.S		N.S
204370_at	CLP1	1	1.3	2.16E-29	1.60	3.38E-49
204372_s_at	KHSRP	0	1.1	8.68E-05		N.S
204373_s_at	CEP350	0	-1.2	1.97E-06		N.S
204382_at	NAT9	0		N.S		N.S
204394_at	SLC43A1	0		N.S	-1.20	1.60E-05
204401_at	KCNN4	0	-1.1	6.65E-03	1.13	3.47E-03
204404_at	SLC12A2	0	1.1	7.13E-07		N.S
204407_at	TTF2	0		N.S		N.S
204408_at	APEX2	4		N.S		N.S
204415_at	IFI6	0		N.S		N.S
204420_at	FOSL1	0	2.4	9.62E-14	3.81	4.94E-24
204423_at	MKLN1	0	-1.1	1.53E-02		N.S
204425_at	ARHGAP4	0		N.S		N.S

204426_at	TMED2	0		N.S		N.S
204430_s_at	SLC2A5	1	1.2	1.31E-07	-1.26	8.27E-09
204432_at	SOX12	0		N.S		N.S
204435_at	NUPL1	0	1.1	3.89E-02		N.S
204436_at	PLEKHO2	0		N.S	1.35	6.98E-21
204439_at	IFI44L	0	-1.2	4.90E-05	-1.29	2.22E-06
204440_at	CD83	1	1.5	8.15E-19	1.12	3.83E-02
204441_s_at	POLA2	0		N.S	1.18	6.73E-07
204444_at	KIF11	1		N.S	-1.19	3.57E-04
204448_s_at	PDCL	3		N.S		N.S
204453_at	ZNF84	0	-1.3	1.62E-10		N.S
204458_at	PLA2G15	0		N.S	1.34	1.51E-12
204459_at	CSTF2	0		N.S		N.S
204460_s_at	RAD1	238		N.S		N.S
204472_at	GEM	7		N.S	-1.20	5.15E-03
204473_s_at	ZNF592	0		N.S		N.S
204474_at	ZNF142	0		N.S		N.S
204477_at	RABIF	0		N.S		N.S
204479_at	OSTF1	0		N.S	-1.12	2.03E-02
204481_at	BRPF1	0	1.4	5.64E-11	1.19	2.07E-03
204483_at	ENO3	0		N.S		N.S
204485_s_at	TOM1L1	0		N.S		N.S
204488_at	DOLK	0		N.S	1.13	1.64E-03
204492_at	ARHGAP11A	0		N.S	-1.55	8.46E-15
204502_at	SAMHD1	0	-1.1	3.92E-02		N.S
204506_at	PPP3R1	0		N.S		N.S
204510_at	CDC7	40		N.S		N.S
204512_at	HIVEP1	0	1.2	8.76E-05	1.13	7.40E-04
204513_s_at	ELMO1	0		N.S	-1.21	1.88E-04
204514_at	DPH2	0		N.S		N.S
204516_at	ATXN7	0	1.2	4.71E-03		N.S
204521_at	C12orf24	0	-1.1	1.86E-02	-1.35	2.21E-16
204523_at	ZNF140	0		N.S	1.15	6.20E-05
204530_s_at	TOX	22		N.S		N.S
204531_s_at	BRCA1	975	-1.1	2.21E-03		N.S
204533_at	CXCL10	1		N.S		N.S
204544_at	HPS5	0		N.S		N.S
204547_at	RAB40B	0		N.S		N.S
204552_at	INPP4A	0	-1.2	4.53E-02	-1.26	3.35E-07
204554_at	PPP1R3D	0		N.S	1.20	6.11E-07
204559_s_at	LSM7	0		N.S		N.S
204562_at	IRF4	0		N.S	-1.35	5.24E-16
204563_at	SELL	5		N.S	-1.37	2.77E-15
204565_at	ACOT13	0		N.S		N.S
204566_at	PPM1D	26	3.0	7.88E-36	3.74	3.41E-51
204568_at	KIAA0831	0	1.2	3.30E-08	1.12	9.35E-04
204569_at	ICK	0		N.S	1.35	5.74E-05
204573_at	CROT	0	-1.2	6.20E-04	1.72	7.52E-30
204576_s_at	CLUAP1	0	-1.3	8.32E-18	1.15	9.11E-07
204593_s_at	SMCR7L	0	1.1	6.68E-09	1.11	1.00E-05
204599_s_at	MRPL28	0		N.S		N.S
204602_at	DKK1	3		N.S		N.S
204603_at	EXO1	41	1.2	2.81E-08	1.15	5.11E-04
204605_at	CGRRF1	0	1.1	4.62E-05	1.50	1.44E-30
204608_at	ASL	4		N.S		N.S
204610_s_at	CCDC85B	0		N.S	-1.30	2.87E-09
204612_at	PKIA	0	-1.2	4.30E-12		N.S
204613_at	PLCG2	1		N.S		N.S
204616_at	UCHL3	0		N.S		N.S
204617_s_at	ACD	1		N.S		N.S

204618_s_at	GABPB1	0	1.2	2.30E-07		N.S
204630_s_at	GOSR1	0		N.S	1.18	1.56E-06
204632_at	RPS6KA4	0		N.S		N.S
204634_at	NEK4	0		N.S		N.S
204635_at	RPS6KA5	0		N.S	1.17	1.21E-03
204638_at	ACP5	0		N.S	-1.17	8.45E-03
204641_at	NEK2	6	-1.2	3.42E-06	-1.65	2.38E-24
204642_at	S1PR1	0		N.S	-1.50	4.22E-12
204646_at	DPYD	0		N.S		N.S
204650_s_at	APBB3	0		N.S		N.S
204651_at	NRF1	2		N.S		N.S
204659_s_at	GFER	0		N.S		N.S
204662_at	CP110	0		N.S	1.78	1.56E-38
204666_s_at	RP5-1000E10.4	0		N.S		N.S
204674_at	LRMP	0	-1.3	2.47E-15	-1.14	3.36E-02
204676_at	TMEM186	0	-1.3	1.83E-09		N.S
204678_s_at	KCNK1	0		N.S		N.S
204683_at	ICAM2	0		N.S	-1.13	2.35E-02
204687_at	DKFZP564O0823	0		N.S		N.S
204688_at	SGCE	4		N.S	-1.15	1.86E-08
204690_at	STX8	0		N.S		N.S
204695_at	CDC25A	314	1.8	1.28E-17		N.S
204698_at	ISG20	0		N.S		N.S
204702_s_at	NFE2L3	0		N.S	-1.19	2.72E-06
204703_at	IFT88	0		N.S		N.S
204706_at	INPP5E	0		N.S		N.S
204709_s_at	KIF23	0	-1.4	3.83E-10	-1.62	1.47E-13
204710_s_at	WIPI2	0		N.S	1.10	9.13E-05
204715_at	PANX1	0	1.2	6.55E-10		N.S
204716_at	CCDC6	2		N.S		N.S
204720_s_at	DNAJC6	0		N.S		N.S
204725_s_at	NCK1	0	-1.2	2.23E-04		N.S
204727_at	WDHD1	0	-1.2	3.23E-02		N.S
204731_at	TGFBR3	0		N.S	1.20	2.33E-02
204735_at	PDE4A	0		N.S	-1.27	4.70E-07
204739_at	CENPC1	0	1.2	4.21E-14		N.S
204740_at	CNKSR1	0		N.S		N.S
204742_s_at	PDS5B	0	-1.2	2.71E-04		N.S
204744_s_at	IARS	0		N.S		N.S
204747_at	IFIT3	0	1.3	3.41E-10	1.16	2.41E-03
204759_at	RCBTB2	0		N.S	1.25	4.69E-07
204761_at	USP6NL	0		N.S	-1.13	2.82E-02
204766_s_at	NUDT1	7		N.S	1.39	2.40E-02
204767_s_at	FEN1	66	1.1	5.46E-04	1.11	1.06E-02
204771_s_at	TTF1	0		N.S		N.S
204774_at	EVI2A	0	-1.4	4.27E-17	-1.68	1.23E-25
204777_s_at	MAL	9		N.S		N.S
204779_s_at	HOXB7	1		N.S		N.S
204781_s_at	FAS	434	1.5	1.09E-26	1.88	2.27E-39
204786_s_at	IFNAR2	0		N.S		N.S
204788_s_at	PPOX	0	-1.1	5.90E-03	-1.14	1.76E-06
204789_at	FMNL1	0		N.S		N.S
204790_at	SMAD7	6		N.S		N.S
204794_at	DUSP2	1		N.S	-1.49	2.77E-30
204795_at	PRR3	0		N.S		N.S
204798_at	MYB	25		N.S	-1.20	2.95E-04
204804_at	TRIM21	0		N.S		N.S
204805_s_at	H1FX	0		N.S	-1.96	6.47E-20
204807_at	TMEM5	0		N.S		N.S
204809_at	CLPX	6		N.S		N.S

204812_at	ZW10	1		N.S		N.S
204813_at	MAPK10	1		N.S		N.S
204821_at	BTN3A3	0	-1.2	2.29E-03	-1.39	7.96E-12
204822_at	TTK	6		N.S	-1.31	1.01E-15
204825_at	MELK	1	-1.1	2.59E-02		N.S
204828_at	RAD9A	4		N.S	1.13	4.05E-02
204831_at	CDK8	4	-1.3	1.36E-14		N.S
204832_s_at	BMPR1A	1	-1.2	8.75E-05		N.S
204834_at	FGL2	0	-1.3	5.41E-05		N.S
204835_at	POLA1	11		N.S		N.S
204836_at	GLDC	0		N.S		N.S
204838_s_at	MLH3	6	-1.5	4.01E-24	-1.27	5.70E-11
204839_at	POP5	0		N.S		N.S
204840_s_at	EEA1	0		N.S	1.31	3.43E-04
204847_at	ZBTB11	0		N.S	1.14	4.35E-04
204849_at	TCFL5	0	-1.2	1.49E-07		N.S
204852_s_at	PTPN7	0		N.S	-1.24	8.29E-08
204853_at	ORC2L	1	-1.3	6.01E-20	-1.16	1.19E-06
204857_at	MAD1L1	5		N.S	1.71	8.85E-21
204862_s_at	NME3	0		N.S	-1.20	1.45E-03
204866_at	PHF16	0		N.S	1.55	2.18E-14
204867_at	GCHFR	0		N.S		N.S
204868_at	ICT1	0		N.S		N.S
204880_at	MGMT	154		N.S		N.S
204881_s_at	UGCG	0	1.1	4.94E-02	-1.18	2.50E-08
204883_s_at	HUS1	115		N.S	-1.22	1.12E-04
204887_s_at	PLK4	5		N.S	-1.17	1.36E-03
204890_s_at	LCK	18		N.S	-1.29	3.98E-13
204905_s_at	EEF1E1	0		N.S		N.S
204909_at	DDX6	1		N.S		N.S
204912_at	IL10RA	1	1.4	5.53E-14	1.15	1.19E-02
204917_s_at	MLLT3	1		N.S	-1.22	4.65E-05
204923_at	SASH3	0		N.S	-1.32	2.76E-14
204937_s_at	ZNF274	0		N.S		N.S
204946_s_at	TOP3A	0		N.S		N.S
204949_at	ICAM3	0		N.S		N.S
204950_at	CARD8	0	-1.2	9.29E-12		N.S
204955_at	SRPX	0		N.S		N.S
204959_at	MNDA	1		N.S	-1.44	1.13E-18
204960_at	PTPRCAP	0		N.S	-1.62	2.76E-21
204961_s_at	LOC648998	0		N.S	-1.17	1.60E-09
204962_s_at	CENPA	4	-1.7	8.16E-27	-2.07	8.88E-36
204968_at	C6orf47	0		N.S		N.S
204970_s_at	MAFG	0		N.S		N.S
204976_s_at	AMMECR1	0	-1.3	5.87E-17		N.S
204977_at	DDX10	0	-1.1	1.62E-13	-1.19	1.39E-13
204978_at	SFRS16	0		N.S		N.S
204985_s_at	TRAPPC6A	0		N.S		N.S
204992_s_at	PFN2	0		N.S	-1.20	4.64E-09
204994_at	MX2	10		N.S	-1.36	3.18E-17
204995_at	CDK5R1	0	-1.2	4.38E-03	-1.58	5.46E-15
205004_at	NKRF	0		N.S		N.S
205010_at	GNL3L	0		N.S		N.S
205012_s_at	HAGH	0		N.S		N.S
205013_s_at	ADORA2A	0	-1.1	2.05E-02		N.S
205027_s_at	MAP3K8	3		N.S		N.S
205034_at	CCNE2	1	1.5	2.46E-15	1.43	7.37E-15
205036_at	LSM6	0		N.S		N.S
205042_at	GNE	0	1.1	1.50E-05	1.36	6.50E-16
205046_at	CENPE	0	-1.3	1.98E-12	-2.06	2.65E-26

Supplemental Table 2

205047_s_at	ASNS	0		N.S	1.19	2.65E-09
205048_s_at	PSPH	0		N.S	1.21	3.68E-05
205049_s_at	CD79A	0		N.S		N.S
205052_at	AUH	2	-1.2	6.02E-13	-1.30	2.66E-19
205053_at	PRIM1	1		N.S		N.S
205055_at	ITGAE	0		N.S		N.S
205060_at	PARG	36	-1.2	2.85E-05		N.S
205061_s_at	EXOSC9	0	1.1	5.29E-08		N.S
205063_at	SIP1	2	-1.3	1.06E-07		N.S
205069_s_at	ARHGAP26	1		N.S		N.S
205070_at	ING3	2	1.2	5.17E-14	1.11	7.57E-06
205078_at	PIGF	0	-1.2	6.94E-03		N.S
205081_at	CRIP1	2		N.S		N.S
205085_at	ORC1L	3	1.3	1.13E-11		N.S
205087_at	RWDD3	0		N.S	1.24	6.04E-05
205089_at	ZNF7	0		N.S	1.13	1.67E-04
205090_s_at	NAGPA	0		N.S		N.S
205094_at	PEX12	0		N.S	1.26	5.83E-15
205097_at	SLC26A2	0		N.S	1.22	3.65E-02
205098_at	CCR1	1	-1.5	6.39E-15	-1.73	7.16E-21
205103_at	C1orf61	0		N.S		N.S
205105_at	MAN2A1	0		N.S		N.S
205107_s_at	EFNA4	0		N.S		N.S
205114_s_at	CCL3	2	1.5	2.89E-15	-1.18	8.56E-03
205126_at	VRK2	0	-1.1	7.90E-07		N.S
205129_at	NPM3	0		N.S		N.S
205132_at	ACTC1	0		N.S		N.S
205133_s_at	HSPE1	0		N.S	-1.25	1.49E-03
205134_s_at	NUFIP1	1		N.S		N.S
205140_at	FPGT	0	-1.3	1.82E-16		N.S
205145_s_at	LOC649851	0		N.S		N.S
205159_at	CSF2RB	0		N.S		N.S
205162_at	ERCC8	16		N.S		N.S
205169_at	RBBP5	2	-1.1	2.48E-03		N.S
205170_at	STAT2	2		N.S		N.S
205171_at	PTPN4	0		N.S	1.15	2.48E-04
205176_s_at	ITGB3BP	0		N.S		N.S
205178_s_at	RBBP6	0	1.2	9.47E-07		N.S
205181_at	ZNF193	0		N.S	1.26	6.13E-04
205188_s_at	SMAD5	1	1.3	4.30E-03	2.26	3.40E-24
205189_s_at	FANCC	59		N.S		N.S
205191_at	RP2	1		N.S		N.S
205192_at	MAP3K14	0		N.S		N.S
205198_s_at	ATP7A	0	-1.2	1.69E-06		N.S
205205_at	RELB	13	1.4	2.80E-15	1.33	7.71E-15
205217_at	TIMM8A	0		N.S		N.S
205218_at	POLR3F	0		N.S	1.20	8.09E-07
205220_at	NIACR2	0	1.6	2.76E-21	2.64	3.23E-41
205222_at	EHHADH	1	-1.4	8.59E-03		N.S
205224_at	SURF2	0		N.S		N.S
205229_s_at	COCH	0		N.S	-1.18	4.03E-05
205231_s_at	EPM2A	1		N.S	1.16	9.02E-04
205235_s_at	KIF20B	1		N.S		N.S
205238_at	TRMT2B	0		N.S		N.S
205241_at	SCO2	0		N.S		N.S
205245_at	PARD6A	0		N.S		N.S
205246_at	PEX13	0		N.S		N.S
205249_at	EGR2	2	1.2	4.30E-02	-1.40	1.88E-09
205256_at	ZBTB39	0		N.S		N.S
205260_s_at	ACYP1	0		N.S		N.S

Supplemental Table 2

205263_at	BCL10	3		N.S	1.18	1.09E-09
205264_at	CD3EAP	1		N.S	-1.41	4.59E-13
205267_at	POU2AF1	0		N.S	-1.15	9.10E-10
205269_at	LCP2	0	-1.3	1.23E-08	-1.66	1.40E-21
205273_s_at	PITRM1	0		N.S		N.S
205283_at	FKTN	0	-1.1	1.41E-03		N.S
205291_at	IL2RB	0		N.S	-1.42	1.79E-15
205292_s_at	HNRNPA2B1	0		N.S	-1.12	8.58E-05
205296_at	RBL1	15		N.S		N.S
205297_s_at	CD79B	0		N.S		N.S
205298_s_at	BTN2A2	0		N.S		N.S
205300_s_at	SNRNP35	0		N.S	-1.14	1.83E-02
205301_s_at	OGG1	351		N.S		N.S
205308_at	FAM164A	0		N.S		N.S
205310_at	FBXO46	0		N.S		N.S
205315_s_at	SNTB2	0		N.S	1.14	5.46E-05
205321_at	EIF2S3	0		N.S		N.S
205322_s_at	MTF1	2		N.S		N.S
205327_s_at	ACVR2A	0	1.2	1.63E-03		N.S
205328_at	CLDN10	0		N.S		N.S
205329_s_at	SNX4	0		N.S	1.10	3.54E-04
205333_s_at	RCE1	0	1.2	1.39E-03		N.S
205335_s_at	SRP19	0		N.S		N.S
205339_at	STIL	0		N.S		N.S
205340_at	ZBTB24	0		N.S		N.S
205345_at	BARD1	59		N.S	-1.15	1.96E-02
205346_at	ST3GAL2	0		N.S		N.S
205349_at	GNA15	0		N.S	1.36	8.19E-08
205352_at	SERPINI1	0	-1.1	1.15E-04		N.S
205354_at	GAMT	4		N.S	1.56	4.23E-18
205355_at	ACADSB	0		N.S		N.S
205356_at	USP13	0		N.S	-1.19	1.35E-09
205361_s_at	PFDN4	0		N.S		N.S
205367_at	SH2B2	0	-1.2	1.47E-05	-1.25	4.41E-07
205372_at	PLAG1	1		N.S		N.S
205376_at	INPP4B	0		N.S		N.S
205393_s_at	CHEK1	16	1.1	6.77E-04		N.S
205401_at	AGPS	0		N.S		N.S
205406_s_at	SPA17	0		N.S		N.S
205408_at	MLLT10	0	-1.3	9.86E-09		N.S
205411_at	STK4	1		N.S		N.S
205412_at	ACAT1	1		N.S		N.S
205414_s_at	RICH2	0		N.S		N.S
205416_s_at	ATXN3	2	-1.1	5.22E-04		N.S
205417_s_at	DAG1	0		N.S		N.S
205419_at	GPR183	0		N.S	-1.32	4.66E-06
205423_at	AP1B1	0		N.S		N.S
205429_s_at	MPP6	0		N.S	-1.31	2.72E-12
205436_s_at	H2AFX	583		N.S		N.S
205443_at	SNAPC1	0	-1.5	6.19E-26	-1.14	7.53E-04
205449_at	SAC3D1	0	1.3	1.73E-09	2.05	3.59E-37
205452_at	PIGB	0		N.S		N.S
205461_at	RAB35	0		N.S		N.S
205463_s_at	PDGFA	0		N.S		N.S
205467_at	CASP10	8		N.S		N.S
205469_s_at	IRF5	7		N.S	1.15	4.02E-04
205474_at	CRLF3	0	-1.2	2.45E-13	-1.23	3.36E-20
205480_s_at	UGP2	0	-1.0	1.34E-02	-1.14	7.79E-19
205483_s_at	ISG15	5		N.S		N.S
205484_at	SIT1	0	-1.4	8.51E-08	-1.97	4.89E-20

Supplemental Table 2

205497_at	ZNF175	0		N.S	1.26	2.03E-05
205504_at	BTK	3		N.S	-1.21	4.21E-09
205512_s_at	AIFM1	19		N.S		N.S
205518_s_at	CMAH	0	-1.4	3.49E-16	-1.32	1.09E-10
205519_at	WDR76	0		N.S		N.S
205521_at	EXOG	0		N.S	1.19	1.82E-05
205526_s_at	KATNA1	0		N.S	1.11	7.79E-08
205527_s_at	GEMIN4	0		N.S	-1.49	3.61E-17
205531_s_at	GLS2	1		N.S	4.79	2.94E-28
205540_s_at	RRAGB	0		N.S		N.S
205541_s_at	GSPT2	0	-1.2	2.59E-11		N.S
205542_at	STEAP1	0		N.S	-1.43	4.26E-09
205543_at	HSPA4L	0	-1.2	4.25E-14		N.S
205544_s_at	CR2	8	-1.2	3.34E-06	-1.73	3.04E-23
205546_s_at	TYK2	1		N.S		N.S
205550_s_at	BRE	10		N.S	-1.12	3.58E-11
205552_s_at	OAS1	0		N.S	-1.49	2.22E-20
205554_s_at	DNASE1L3	1		N.S		N.S
205562_at	RPP38	0		N.S		N.S
205565_s_at	FXN	1		N.S	-1.16	3.55E-04
205569_at	LAMP3	1		N.S	1.58	5.01E-25
205570_at	PIP4K2A	0		N.S	-1.14	3.14E-03
205571_at	LIPT1	0		N.S		N.S
205584_at	ALG13	0	-1.3	7.74E-07	-1.30	1.04E-05
205585_at	ETV6	1	-1.3	1.19E-05		N.S
205588_s_at	FGFR1OP	0	-1.1	4.32E-07		N.S
205590_at	RASGRP1	0	-1.2	3.88E-08		N.S
205594_at	ZNF652	0		N.S		N.S
205596_s_at	SMURF2	2		N.S	1.32	7.30E-25
205599_at	TRAF1	2		N.S		N.S
205603_s_at	DIAPH2	0		N.S	-1.18	6.50E-04
205607_s_at	SCYL3	0	-1.3	1.47E-03	1.17	5.25E-04
205621_at	ALKBH1	11		N.S	1.13	3.93E-05
205628_at	PRIM2	0		N.S		N.S
205633_s_at	ALAS1	0	-1.1	9.06E-04	1.14	7.70E-10
205642_at	CEP110	0	-1.1	1.91E-02	-1.15	3.67E-04
205644_s_at	SNRPG	0		N.S		N.S
205655_at	MDM4	39		N.S		N.S
205659_at	HDAC9	1	-1.5	5.04E-15		N.S
205664_at	KIN	14	-1.2	1.84E-10		N.S
205667_at	WRN	181		N.S		N.S
205668_at	LY75	0	-1.1	1.36E-04		N.S
205671_s_at	HLA-DOB	0		N.S		N.S
205672_at	XPA	332		N.S	1.16	4.19E-06
205677_s_at	DLEU1	0	-1.1	3.09E-02	-1.22	1.06E-07
205681_at	BCL2A1	2		N.S	-1.15	1.52E-02
205684_s_at	DENND4C	0	-1.2	1.94E-02		N.S
205687_at	UBFD1	0	-1.1	1.72E-07	-1.12	4.94E-03
205692_s_at	CD38	18		N.S	-1.22	5.21E-03
205701_at	IPO8	0		N.S		N.S
205704_s_at	ATP6V0A2	0		N.S		N.S
205716_at	SLC25A40	0		N.S		N.S
205718_at	ITGB7	0		N.S		N.S
205733_at	BLM	296		N.S		N.S
205740_s_at	RBM42	0		N.S		N.S
205746_s_at	ADAM17	2		N.S		N.S
205748_s_at	RNF126	0		N.S	-1.15	3.89E-02
205750_at	BPHL	0		N.S		N.S
205756_s_at	F8	2		N.S	1.25	1.00E-14
205761_s_at	DUS4L	0		N.S		N.S

Supplemental Table 2

205770_at	GSR	4		N.S		N.S
205773_at	CPEB3	0		N.S		N.S
205775_at	FAM50B	0	-1.2	4.81E-12		N.S
205781_at	C16orf7	0		N.S		N.S
205788_s_at	ZC3H11A	0	-1.1	1.96E-02		N.S
205790_at	SKAP1	0		N.S	-1.40	9.63E-22
205796_at	TCP11L1	0		N.S	1.49	1.08E-26
205801_s_at	RASGRP3	0	-1.2	3.05E-06		N.S
205804_s_at	TRAF3IP3	0	-1.2	9.49E-03	-1.34	2.10E-21
205807_s_at	TUFT1	0	1.3	4.72E-12	1.40	1.03E-14
205809_s_at	WASL	0		N.S		N.S
205811_at	POLG2	0	-1.4	1.59E-16		N.S
205830_at	CLGN	0		N.S	3.00	3.79E-23
205832_at	CPA4	0		N.S		N.S
205841_at	JAK2	18	-1.6	3.14E-13		N.S
205847_at	PRSS22	0		N.S		N.S
205848_at	GAS2	2		N.S		N.S
205849_s_at	UQCRB	0		N.S		N.S
205851_at	NME6	0		N.S		N.S
205855_at	ZNF197	0		N.S		N.S
205859_at	LY86	0		N.S	-1.12	3.87E-03
205861_at	SPIB	0	-1.2	1.35E-07	-1.30	4.25E-12
205865_at	ARID3A	2		N.S	1.18	3.10E-10
205873_at	PIGL	0		N.S		N.S
205884_at	ITGA4	0	-1.3	2.05E-06	-1.80	2.35E-18
205890_s_at	GABBR1	0		N.S		N.S
205895_s_at	NOLC1	0		N.S		N.S
205901_at	PNOC	0		N.S	-1.22	7.61E-04
205902_at	KCNN3	0	-1.5	9.42E-11	1.58	2.02E-13
205909_at	POLE2	0		N.S		N.S
205917_at	ZNF264	0		N.S	1.24	8.62E-12
205922_at	VNN2	0		N.S		N.S
205928_at	ZNF443	1		N.S		N.S
205930_at	GTF2E1	0	-1.3	5.75E-17		N.S
205932_s_at	MSX1	1		N.S		N.S
205934_at	PLCL1	0		N.S		N.S
205945_at	IL6R	1		N.S		N.S
205953_at	LRIG2	0		N.S		N.S
205955_at	TAF6L	0		N.S		N.S
205963_s_at	DNAJA3	0	-1.1	2.07E-04		N.S
205964_at	ZNF426	0		N.S	1.26	1.08E-02
205965_at	BATF	1		N.S		N.S
205967_at	HIST1H4C	0		N.S		N.S
205978_at	KL	14		N.S	1.33	2.88E-02
205981_s_at	ING2	8	1.3	2.18E-12	-1.15	8.00E-05
205991_s_at	PRRX1	0		N.S		N.S
205992_s_at	IL15	0	1.1	4.72E-04		N.S
205996_s_at	AK2	0		N.S		N.S
206006_s_at	KIAA1009	0		N.S		N.S
206016_at	CCDC22	0		N.S		N.S
206020_at	SOCS6	1		N.S		N.S
206026_s_at	TNFAIP6	1		N.S		N.S
206031_s_at	USP5	0		N.S		N.S
206035_at	REL	29		N.S		N.S
206037_at	CCBL1	0	-1.2	2.50E-05	-1.25	1.65E-07
206038_s_at	NR2C2	0		N.S		N.S
206039_at	RAB33A	0	-1.4	5.83E-16	-1.58	5.16E-24
206044_s_at	BRAF	23		N.S		N.S
206050_s_at	RNH1	1		N.S		N.S
206052_s_at	SLBP	0	1.2	4.57E-19	-1.16	9.81E-10

206055_s_at	SNRPA1	0		N.S		N.S
206059_at	ZNF91	0		N.S		N.S
206061_s_at	DICER1	1	-1.2	9.44E-07		N.S
206074_s_at	HMGA1	7		N.S		N.S
206082_at	HCP5	0	-1.1	5.83E-06	1.20	2.19E-11
206096_at	ZNF35	0	1.2	4.51E-11		N.S
206098_at	ZBTB6	0		N.S		N.S
206102_at	GINS1	0		N.S	1.13	3.00E-04
206106_at	MAPK12	2		N.S	-1.20	2.06E-02
206108_s_at	SFRS6	2	1.2	2.45E-04		N.S
206115_at	EGR3	1	-1.5	7.43E-08	-2.05	2.79E-20
206129_s_at	ARSB	0		N.S		N.S
206132_at	MCC	3		N.S		N.S
206133_at	XAF1	1		N.S		N.S
206134_at	ADAMDEC1	0		N.S	-1.22	1.79E-04
206141_at	MOCS3	0		N.S		N.S
206150_at	CD27	3	-1.1	3.77E-02	-1.39	2.50E-13
206158_s_at	CNBP	0	1.1	2.12E-10		N.S
206181_at	SLAMF1	0	-1.3	8.30E-20		N.S
206182_at	ZNF134	0		N.S		N.S
206184_at	CRKL	1		N.S		N.S
206188_at	ZNF623	0		N.S	1.50	1.89E-19
206194_at	HOXC4	0	-1.1	8.15E-03		N.S
206200_s_at	ANXA11	0		N.S		N.S
206219_s_at	VAV1	1		N.S	-1.19	2.16E-07
206233_at	B4GALT6	0		N.S		N.S
206235_at	LIG4	51		N.S		N.S
206240_s_at	ZNF136	2	1.1	6.09E-04		N.S
206247_at	MICB	7	-1.1	3.56E-02	1.23	3.85E-11
206255_at	BLK	3		N.S	-1.40	5.66E-10
206257_at	CCDC9	0		N.S		N.S
206261_at	ZNF239	0		N.S		N.S
206263_at	FMO4	0		N.S		N.S
206302_s_at	NUDT4	0		N.S		N.S
206308_at	TRDMT1	0		N.S		N.S
206313_at	HLA-DOA	0		N.S		N.S
206314_at	ZNF167	0	-1.2	2.35E-02		N.S
206316_s_at	KNTC1	1		N.S		N.S
206335_at	GALNS	0		N.S		N.S
206337_at	CCR7	0		N.S	1.26	7.72E-15
206357_at	OPA3	0		N.S		N.S
206361_at	GPR44	0		N.S		N.S
206364_at	KIF14	0	-1.3	9.27E-16	-1.64	8.17E-23
206398_s_at	CD19	14		N.S	-1.10	1.96E-02
206412_at	FER	4		N.S		N.S
206440_at	LIN7A	0		N.S		N.S
206445_s_at	PRMT1	12		N.S		N.S
206451_at	TBCCD1	0	-1.4	2.62E-23	1.25	2.36E-13
206478_at	KIAA0125	0		N.S		N.S
206491_s_at	NAPA	4		N.S		N.S
206492_at	FHIT	37		N.S		N.S
206497_at	C7orf44	0		N.S		N.S
206500_s_at	C14orf106	0		N.S	-1.29	1.04E-06
206507_at	ZSCAN12	0		N.S		N.S
206508_at	CD70	2	1.2	6.98E-08	1.63	5.20E-21
206513_at	AIM2	0	-1.2	2.55E-06	-2.03	6.34E-34
206515_at	CYP4F3	0		N.S	2.63	5.10E-34
206533_at	CHRNA5	0		N.S	-1.13	5.93E-03
206536_s_at	XIAP	32		N.S		N.S
206542_s_at	SMARCA2	5		N.S	-1.15	4.04E-02

206550_s_at	NUP155	0		N.S		N.S
206553_at	OAS2	0		N.S	-1.16	7.24E-03
206583_at	ZNF673	0	-1.2	3.13E-04		N.S
206584_at	LY96	1		N.S		N.S
206587_at	CCT6B	0		N.S	1.28	1.61E-14
206588_at	DAZL	0		N.S	1.38	7.70E-07
206592_s_at	AP3D1	0		N.S		N.S
206593_s_at	MED22	0		N.S		N.S
206608_s_at	RPGRIP1	0		N.S		N.S
206613_s_at	TAF1A	0	-1.3	7.10E-08		N.S
206618_at	IL18R1	0	-1.3	1.35E-09	-1.28	2.25E-05
206621_s_at	EIF4H	0		N.S		N.S
206632_s_at	APOBEC3B	0	1.2	6.48E-09	1.74	2.81E-30
206641_at	TNFRSF17	0	1.2	4.55E-06		N.S
206649_s_at	TFE3	0		N.S		N.S
206652_at	ZMYM5	0		N.S		N.S
206654_s_at	POLR3G	0		N.S	-1.36	9.09E-09
206656_s_at	C20orf3	0		N.S		N.S
206659_at	FLJ14082	0		N.S	1.31	2.85E-10
206667_s_at	SCAMP1	0		N.S		N.S
206683_at	ZNF165	0		N.S		N.S
206686_at	PDK1	7		N.S	-1.87	2.78E-15
206687_s_at	PTPN6	7	-1.1	1.33E-04	-1.18	2.15E-06
206688_s_at	CPSF4	0		N.S	1.17	3.49E-14
206693_at	IL7	0		N.S	-1.17	1.59E-02
206704_at	CLCN5	0		N.S		N.S
206729_at	TNFRSF8	0	-1.3	9.35E-15	-1.31	3.55E-12
206734_at	JRKL	0	-1.6	2.32E-16		N.S
206752_s_at	DFFB	4	-1.2	6.25E-07		N.S
206756_at	CHST7	0	1.2	4.03E-08		N.S
206759_at	FCER2	0		N.S		N.S
206766_at	ITGA10	0		N.S		N.S
206788_s_at	CBFB	2		N.S		N.S
206789_s_at	POU2F1	11	-1.2	3.15E-02	-1.17	6.52E-03
206790_s_at	NDUFB1	0		N.S		N.S
206809_s_at	HNRNPA3	1		N.S		N.S
206825_at	OXTR	0	1.1	4.46E-02		N.S
206828_at	TXK	1		N.S	1.31	1.30E-02
206833_s_at	ACYP2	0		N.S		N.S
206845_s_at	RNF40	0		N.S	1.14	4.30E-03
206848_at	FAM36A	0		N.S		N.S
206855_s_at	HYAL2	1		N.S		N.S
206860_s_at	MIOS	0	-1.3	1.02E-18	-1.38	2.29E-22
206861_s_at	CGGBP1	0	1.1	3.21E-05		N.S
206875_s_at	SLK	1		N.S		N.S
206907_at	TNFSF9	2	2.0	5.57E-31	1.63	4.58E-22
206918_s_at	CPNE1	0		N.S		N.S
206925_at	ST8SIA4	0	-1.5	2.56E-18	-1.19	5.39E-03
206928_at	ZNF124	1	1.2	5.08E-09		N.S
206931_at	ZNF141	0		N.S		N.S
206942_s_at	PMCH	0		N.S		N.S
206949_s_at	RUSC1	0		N.S	1.28	6.59E-18
206958_s_at	UPF3A	0		N.S		N.S
206975_at	LTA	8		N.S	-1.61	1.67E-21
206976_s_at	HSPH1	0	-1.2	2.64E-16	-1.23	2.57E-09
206983_at	CCR6	1	-1.6	3.63E-14	-2.15	1.01E-18
206992_s_at	ATP5S	0	1.2	4.81E-05	1.23	6.18E-09
207000_s_at	PPP3CC	0		N.S	-1.24	3.94E-12
207002_s_at	PLAGL1	1	-1.2	6.35E-09		N.S
207038_at	SLC16A6	0		N.S		N.S

207040_s_at	ST13	0		N.S		N.S
207064_s_at	AOC2	0		N.S	1.25	8.38E-06
207071_s_at	ACO1	0		N.S		N.S
207079_s_at	MED6	0		N.S	1.16	1.34E-07
207088_s_at	SLC25A11	0	-1.1	1.55E-02		N.S
207096_at	SAA4	0		N.S		N.S
207103_at	KCND2	0	-1.5	3.59E-17	-1.27	6.84E-05
207113_s_at	TNF	539	1.3	4.62E-10	-1.57	2.30E-16
207121_s_at	MAPK6	0		N.S		N.S
207124_s_at	GNB5	0		N.S		N.S
207127_s_at	HNRNPH3	0		N.S		N.S
207152_at	NTRK2	5		N.S		N.S
207153_s_at	GLMN	0		N.S		N.S
207157_s_at	GNG5	0		N.S		N.S
207158_at	APOBEC1	1		N.S		N.S
207160_at	IL12A	0		N.S	-1.30	2.68E-05
207163_s_at	AKT1	99		N.S		N.S
207170_s_at	LETMD1	0	-1.1	2.27E-02	1.16	1.59E-11
207176_s_at	CD80	3	-1.2	9.89E-07	1.18	3.89E-10
207178_s_at	FRK	2	-1.4	2.27E-04	1.34	7.60E-10
207181_s_at	CASP7	46		N.S		N.S
207186_s_at	BPTF	0		N.S		N.S
207196_s_at	TNIP1	0		N.S	-1.11	3.14E-02
207198_s_at	LIMS1	2		N.S		N.S
207219_at	ZNF643	0	1.4	8.94E-10		N.S
207229_at	KLRA1	0		N.S		N.S
207234_at	RFX3	0		N.S		N.S
207243_s_at	CALM2	0		N.S		N.S
207245_at	UGT2B17	0		N.S	1.38	2.26E-02
207283_at	RPL23AP32	0		N.S		N.S
207305_s_at	KIAA1012	0	-1.2	1.14E-06		N.S
207315_at	CD226	1	-1.1	4.10E-02	-1.27	3.18E-12
207332_s_at	TFRC	1		N.S	1.10	9.40E-03
207338_s_at	ZNF200	0	1.2	3.02E-06	1.20	2.12E-12
207339_s_at	LTB	1		N.S	-1.42	2.60E-09
207347_at	ERCC6	77		N.S		N.S
207350_s_at	VAMP4	0		N.S		N.S
207375_s_at	IL15RA	0		N.S	-1.17	2.88E-03
207386_at	CYP7B1	3		N.S		N.S
207389_at	GP1BA	0		N.S		N.S
207394_at	ZNF137	0		N.S	1.14	1.33E-02
207405_s_at	RAD17	185	-1.1	1.70E-02		N.S
207408_at	SLC22A14	0		N.S		N.S
207417_s_at	ZNF177	0		N.S		N.S
207426_s_at	TNFSF4	2	1.3	1.71E-04	2.03	1.43E-27
207431_s_at	DEGS1	0		N.S	-1.38	3.17E-19
207435_s_at	SRRM2	0		N.S		N.S
207438_s_at	SNUPN	0	-1.1	2.09E-07	-1.13	1.74E-06
207440_at	SLC35A2	0		N.S		N.S
207445_s_at	CCR9	0		N.S		N.S
207469_s_at	PIR	5		N.S		N.S
207480_s_at	MEIS2	0	-1.2	2.44E-05		N.S
207508_at	ATP5G3	1		N.S		N.S
207513_s_at	ZNF189	0	-1.2	5.37E-05	1.18	4.22E-06
207515_s_at	POLR1C	0		N.S		N.S
207519_at	SLC6A4	2		N.S	1.33	5.71E-14
207536_s_at	TNFRSF9	1		N.S		N.S
207540_s_at	SYK	4		N.S		N.S
207541_s_at	EXOSC10	0		N.S		N.S
207543_s_at	P4HA1	0	-1.3	9.63E-19	-2.04	2.75E-24

207545_s_at	NUMB	3		N.S		N.S
207551_s_at	MSL3	0	-1.3	1.11E-18		N.S
207559_s_at	ZMYM3	0	-1.2	2.52E-05	-1.34	4.34E-13
207563_s_at	OGT	23		N.S		N.S
207568_at	CHRNA6	0		N.S	1.23	4.36E-03
207583_at	ABCD2	0		N.S		N.S
207585_s_at	RPL36AL	0		N.S		N.S
207597_at	ADAM18	0		N.S		N.S
207606_s_at	ARHGAP12	0	-1.3	7.55E-08	-1.18	4.96E-02
207610_s_at	EMR2	0		N.S		N.S
207614_s_at	CUL1	17		N.S		N.S
207618_s_at	BCS1L	0		N.S	-1.11	3.81E-05
207621_s_at	PEMT	0		N.S		N.S
207622_s_at	ABCF2	0		N.S		N.S
207624_s_at	RPGR	0	1.2	5.20E-04		N.S
207627_s_at	TFCP2	0	-1.3	2.84E-08		N.S
207628_s_at	WBSCR22	0		N.S		N.S
207641_at	TNFRSF13B	0		N.S	-1.32	7.30E-12
207655_s_at	BLNK	1	-1.1	4.80E-10		N.S
207665_at	ADAM21	0		N.S		N.S
207677_s_at	NCF4	0		N.S	-1.17	8.89E-07
207684_at	TBX6	0		N.S		N.S
207687_at	INHBC	0		N.S		N.S
207707_s_at	SEC13	0		N.S		N.S
207713_s_at	RBCK1	0		N.S		N.S
207727_s_at	MUTYH	29		N.S		N.S
207734_at	LAX1	0	-1.2	1.99E-02		N.S
207735_at	RNF125	0		N.S		N.S
207740_s_at	NUP62	0		N.S		N.S
207746_at	POLQ	9		N.S		N.S
207753_at	ZNF304	0		N.S	1.18	4.59E-04
207761_s_at	METTL7A	0		N.S	1.61	2.13E-20
207777_s_at	SP140	0	-1.2	2.23E-04	-1.40	3.17E-13
207780_at	CYLC2	0		N.S		N.S
207785_s_at	RBPJ	3		N.S		N.S
207805_s_at	PSMD9	0		N.S	1.18	4.38E-06
207809_s_at	ATP6AP1	0		N.S		N.S
207812_s_at	GORASP2	0		N.S	-1.13	3.28E-07
207813_s_at	FDXR	5	1.4	3.73E-15	3.24	7.53E-47
207826_s_at	ID3	1		N.S	1.59	3.03E-14
207830_s_at	PPP1R8	1		N.S	1.09	2.50E-04
207842_s_at	CASC3	0		N.S	1.15	2.88E-05
207845_s_at	ANAPC10	0		N.S		N.S
207855_s_at	CLCC1	0		N.S	1.11	3.76E-02
207856_s_at	LOC150776	0		N.S		N.S
207861_at	CCL22	0		N.S		N.S
207871_s_at	ST7	0		N.S	-1.30	6.76E-18
207891_s_at	HAUS7	0		N.S	-1.16	4.57E-02
207900_at	CCL17	0		N.S		N.S
207904_s_at	LNPEP	0		N.S		N.S
207907_at	TNFSF14	0		N.S		N.S
207908_at	KRT2	0		N.S		N.S
207922_s_at	MAEA	1		N.S	1.12	1.07E-08
207945_s_at	CSNK1D	0		N.S		N.S
207951_at	CSN2	5		N.S		N.S
207957_s_at	PRKCB	0		N.S	-1.27	1.56E-15
207966_s_at	GLG1	0		N.S		N.S
207980_s_at	CITED2	2	1.3	1.94E-08		N.S
207996_s_at	C18orf1	0		N.S	1.81	1.24E-21
208003_s_at	NFAT5	5		N.S		N.S

208018_s_at	HCK	0		N.S		N.S
208021_s_at	RFC1	26		N.S		N.S
208024_s_at	DGCR6	0		N.S		N.S
208035_at	GRM6	0		N.S		N.S
208037_s_at	MADCAM1	0		N.S		N.S
208039_at	SLC9A2	0		N.S		N.S
208047_s_at	NAB1	0		N.S		N.S
208050_s_at	CASP2	11		N.S		N.S
208051_s_at	PAIP1	0	1.2	1.91E-10		N.S
208055_s_at	HERC4	0	-1.4	1.34E-06		N.S
208056_s_at	CBFA2T3	0	-1.3	1.27E-15	-1.16	7.99E-05
208066_s_at	GTF2B	0	1.2	1.33E-15	1.15	1.56E-12
208070_s_at	REV3L	19		N.S	1.46	5.20E-23
208072_s_at	DGKD	0		N.S	-1.28	3.91E-05
208074_s_at	AP2S1	0		N.S		N.S
208089_s_at	TDRD3	0	-1.2	3.02E-08	-1.20	1.10E-05
208091_s_at	ECOP	0		N.S		N.S
208093_s_at	NDEL1	0		N.S		N.S
208095_s_at	SRP72	0	1.1	3.13E-04		N.S
208101_s_at	URM1	0		N.S		N.S
208104_s_at	TSC22D4	0		N.S		N.S
208107_s_at	LOC81691	0		N.S	1.40	1.01E-16
208117_s_at	LAS1L	0		N.S		N.S
208119_s_at	ZNF93	0	-1.2	4.59E-07		N.S
208152_s_at	DDX21	0	1.2	5.31E-12		N.S
208154_at	LOC51336	0		N.S		N.S
208184_s_at	TRAPPC10	0		N.S		N.S
208190_s_at	LSR	0		N.S		N.S
208195_at	TTN	0		N.S		N.S
208217_at	GABRR2	0		N.S		N.S
208249_s_at	TGDS	1		N.S	1.34	2.70E-22
208250_s_at	DMBT1	0		N.S		N.S
208270_s_at	RNPEP	0		N.S	1.22	1.11E-09
208290_s_at	EIF5	0		N.S		N.S
208302_at	HMHB1	0		N.S		N.S
208309_s_at	MALT1	2		N.S		N.S
208319_s_at	RBM3	1	1.1	1.82E-06	1.18	1.23E-08
208328_s_at	MEF2A	0		N.S		N.S
208336_s_at	GPSN2	0		N.S		N.S
208368_s_at	BRCA2	482		N.S		N.S
208382_s_at	DMC1	50		N.S		N.S
208398_s_at	TBPL1	0		N.S		N.S
208405_s_at	CD164	0		N.S	-1.09	1.50E-02
208424_s_at	CIAPIN1	0		N.S	1.20	1.27E-14
208433_s_at	LRP8	0		N.S	-1.19	2.73E-04
208436_s_at	IRF7	4		N.S	-1.20	6.10E-04
208438_s_at	FGR	0		N.S	1.35	4.36E-15
208442_s_at	ATM	2168		N.S	-1.40	1.49E-10
208447_s_at	PRPS1	0		N.S		N.S
208453_s_at	XPNPEP1	0		N.S		N.S
208498_s_at	AMY1A	0		N.S		N.S
208499_s_at	DNAJC3	1		N.S		N.S
208503_s_at	GATAD1	0		N.S	1.15	4.52E-03
208506_at	HIST1H3F	0		N.S		N.S
208511_at	PTTG3	0		N.S		N.S
208524_at	GPR15	0	-1.6	2.98E-17		N.S
208578_at	SCN10A	0		N.S		N.S
208588_at	FKSG2	0		N.S		N.S
208612_at	PDIA3	5		N.S		N.S
208616_s_at	PTP4A2	0		N.S	-1.15	1.16E-16

Supplemental Table 2

208619_at	DDB1	107		N.S		N.S
208620_at	PCBP1	0		N.S	-1.13	1.31E-07
208623_s_at	EZR	1		N.S	-1.17	2.25E-07
208624_s_at	EIF4G1	1		N.S		N.S
208627_s_at	YBX1	11		N.S		N.S
208629_s_at	HADHA	0		N.S		N.S
208632_at	RNF10	0	1.2	2.35E-04	1.15	4.84E-04
208636_at	ACTN1	0		N.S		N.S
208641_s_at	RAC1	27		N.S		N.S
208642_s_at	XRCC5	263		N.S		N.S
208644_at	PARP1	187		N.S		N.S
208645_s_at	RPS14	0		N.S		N.S
208647_at	FDFT1	0	-1.1	1.67E-08	-1.21	1.03E-10
208649_s_at	VCP	6		N.S		N.S
208652_at	PPP2CA	52	1.1	9.96E-05		N.S
208655_at	CCNI	1		N.S		N.S
208659_at	CLIC1	0		N.S		N.S
208660_at	CS	1304		N.S		N.S
208670_s_at	EID1	2		N.S	1.11	4.38E-03
208671_at	SERINC1	0	1.3	2.81E-13		N.S
208675_s_at	DDOST	0		N.S	1.12	2.68E-08
208676_s_at	PA2G4	0		N.S		N.S
208677_s_at	BSG	1		N.S		N.S
208678_at	ATP6V1E1	0		N.S	1.09	2.33E-02
208679_s_at	ARPC2	0		N.S		N.S
208680_at	PRDX1	9		N.S		N.S
208684_at	COPA	1		N.S		N.S
208686_s_at	BRD2	0	1.2	5.19E-07		N.S
208689_s_at	RPN2	2		N.S		N.S
208690_s_at	PDLIM1	0		N.S	1.20	8.77E-15
208692_at	RPS3	8		N.S		N.S
208693_s_at	GARS	0		N.S	1.12	3.04E-05
208695_s_at	RPL39	0		N.S		N.S
208696_at	CCT5	2		N.S	-1.15	2.85E-11
208697_s_at	EIF3E	3		N.S		N.S
208698_s_at	NONO	4		N.S		N.S
208700_s_at	TKT	0		N.S		N.S
208709_s_at	NRD1	0		N.S		N.S
208713_at	HNRNPUL1	0		N.S		N.S
208714_at	NDUFV1	0		N.S		N.S
208715_at	TMCO1	0	1.1	4.42E-02		N.S
208717_at	OXA1L	0		N.S	1.11	4.33E-10
208720_s_at	RBM39	0	1.1	2.98E-02		N.S
208721_s_at	ANAPC5	0		N.S		N.S
208723_at	USP11	2		N.S		N.S
208724_s_at	RAB1A	0	1.1	3.44E-04	1.26	5.71E-25
208726_s_at	EIF2S2	0		N.S		N.S
208736_at	ARPC3	0		N.S		N.S
208737_at	ATP6V1G1	0		N.S		N.S
208741_at	SAP18	0		N.S		N.S
208745_at	ATP5L	0		N.S		N.S
208753_s_at	NAP1L1	0		N.S		N.S
208756_at	EIF3I	0		N.S		N.S
208757_at	TMED9	0		N.S		N.S
208758_at	ATIC	0		N.S	-1.10	2.87E-08
208759_at	NCSTN	0		N.S		N.S
208760_at	UBE2I	0		N.S		N.S
208762_at	SUMO1	12		N.S		N.S
208763_s_at	TSC22D3	1	-1.1	4.34E-02		N.S
208765_s_at	HNRNPR	0		N.S		N.S

208771_s_at	LTA4H	0		N.S		N.S
208777_s_at	PSMD11	0		N.S	1.13	7.94E-08
208784_s_at	KLHDC3	0		N.S		N.S
208785_s_at	MAP1LC3B	0		N.S		N.S
208787_at	MRPL3	0		N.S		N.S
208796_s_at	CCNG1	44	1.2	3.53E-15	1.54	2.55E-39
208799_at	PSMB5	0		N.S		N.S
208808_s_at	HMGB2	20		N.S		N.S
208813_at	GOT1	0	-1.1	1.44E-03		N.S
208819_at	RAB8A	0		N.S		N.S
208820_at	PTK2	15		N.S	-1.34	5.18E-03
208821_at	SNRPB	0	1.1	3.16E-02		N.S
208822_s_at	DAP3	2		N.S		N.S
208827_at	PSMB6	0		N.S		N.S
208828_at	POLE3	0	1.2	4.97E-15		N.S
208829_at	TAPBP	0		N.S		N.S
208830_s_at	SUPT6H	0		N.S		N.S
208833_s_at	ATXN10	0		N.S		N.S
208836_at	ATP1B3	0		N.S		N.S
208837_at	TMED3	0		N.S		N.S
208839_s_at	CAND1	4		N.S		N.S
208841_s_at	G3BP2	0		N.S		N.S
208845_at	VDAC3	0		N.S		N.S
208847_s_at	ADH5	0		N.S		N.S
208849_at	EEF1A1	1		N.S		N.S
208853_s_at	CANX	0		N.S		N.S
208854_s_at	STK24	0		N.S		N.S
208857_s_at	PCMT1	0		N.S	-1.10	1.69E-03
208858_s_at	FAM62A	0		N.S		N.S
208861_s_at	ATRX	3	-1.1	1.70E-07		N.S
208862_s_at	CTNND1	0		N.S	1.47	2.14E-15
208872_s_at	REEP5	0		N.S	-1.13	9.41E-04
208876_s_at	PAK2	8		N.S		N.S
208880_s_at	PRPF6	0		N.S		N.S
208883_at	UBR5	7	-1.2	5.36E-08		N.S
208885_at	LCP1	0		N.S		N.S
208886_at	H1F0	0		N.S	-1.91	7.35E-13
208887_at	EIF3G	0		N.S		N.S
208894_at	HLA-DRA	0		N.S		N.S
208897_s_at	DDX18	0	-1.1	3.14E-06	-1.28	1.89E-18
208900_s_at	TOP1	89		N.S		N.S
208905_at	CYCS	0		N.S		N.S
208906_at	BSCL2	0		N.S	1.41	2.85E-16
208909_at	UQCRFS1	0		N.S		N.S
208910_s_at	C1QBP	1		N.S		N.S
208911_s_at	PDHB	0		N.S	-1.12	3.56E-07
208912_s_at	CNP	2		N.S		N.S
208916_at	SLC1A5	0		N.S		N.S
208920_at	SRI	20		N.S	-1.28	6.05E-10
208922_s_at	NXF1	1		N.S		N.S
208923_at	CYFIP1	0		N.S	-1.13	1.59E-03
208924_at	RNF11	1		N.S		N.S
208925_at	CLDND1	0	-1.2	7.18E-09	1.19	8.18E-08
208926_at	NEU1	0		N.S	1.20	1.38E-07
208927_at	SPOP	0	-1.2	7.30E-12		N.S
208932_at	PPP4C	1		N.S		N.S
208938_at	PRCC	0		N.S		N.S
208941_s_at	SEPHS1	0		N.S		N.S
208942_s_at	SEC62	0		N.S		N.S
208944_at	TGFBR2	4		N.S		N.S

208946_s_at	BECN1	6	1.1	1.04E-03	1.12	3.38E-11
208949_s_at	LGALS3	3		N.S		N.S
208954_s_at	LARP5	0	-1.2	1.15E-04		N.S
208959_s_at	ERP44	0		N.S		N.S
208964_s_at	FADS1	0	-1.1	2.51E-06	-1.24	2.47E-13
208965_s_at	IFI16	7		N.S		N.S
208969_at	NDUFA9	0		N.S		N.S
208971_at	UROD	0		N.S	1.12	3.11E-04
208972_s_at	ATP5G1	0		N.S		N.S
208973_at	ERI3	0		N.S		N.S
208979_at	NCOA6	2	-1.2	1.43E-05		N.S
208980_s_at	UBC	22	1.1	2.85E-02		N.S
208985_s_at	EIF3J	0		N.S		N.S
208986_at	TCF12	0	-1.3	1.33E-16		N.S
208991_at	STAT3	36		N.S		N.S
208995_s_at	PPIG	0		N.S		N.S
208996_s_at	POLR2C	0		N.S		N.S
208998_at	UCP2	1		N.S		N.S
208999_at	SEPT8	0		N.S	1.14	2.18E-04
209001_s_at	ANAPC13	0	1.1	1.34E-04		N.S
209004_s_at	FBXL5	0	-1.2	1.41E-10		N.S
209007_s_at	C1orf63	0		N.S	1.31	2.89E-05
209014_at	MAGED1	0		N.S		N.S
209015_s_at	DNAJB6	0	-1.2	7.90E-08		N.S
209017_s_at	LONP1	0		N.S	-1.15	1.14E-03
209020_at	C20orf111	0	1.2	9.29E-11	1.08	2.26E-02
209023_s_at	STAG2	0		N.S		N.S
209028_s_at	ABI1	2		N.S	-1.19	4.52E-08
209029_at	COPS7A	0		N.S		N.S
209030_s_at	CADM1	0		N.S		N.S
209034_at	PNRC1	0		N.S		N.S
209037_s_at	EHD1	0	1.2	3.14E-06		N.S
209040_s_at	PSMB8	0		N.S		N.S
209042_s_at	UBE2G2	0		N.S		N.S
209043_at	PAPSS1	0		N.S	-1.24	3.15E-15
209046_s_at	GABARAPL2	0		N.S		N.S
209049_s_at	ZMYND8	0	-1.1	7.37E-04		N.S
209050_s_at	RALGDS	3		N.S	1.68	1.05E-23
209055_s_at	CDC5L	4		N.S		N.S
209058_at	EDF1	1		N.S		N.S
209068_at	HNRPDL	0		N.S	-1.28	1.59E-14
209075_s_at	ISCU	1		N.S	1.39	1.91E-19
209076_s_at	WDR45L	0		N.S		N.S
209077_at	TXN2	3		N.S		N.S
209083_at	CORO1A	0		N.S	-1.12	1.42E-04
209084_s_at	RAB28	0	-1.2	8.90E-03		N.S
209088_s_at	UBN1	0		N.S		N.S
209090_s_at	SH3GLB1	0		N.S		N.S
209092_s_at	GLOD4	0		N.S		N.S
209095_at	DLD	26		N.S	1.08	1.30E-04
209096_at	UBE2V2	6		N.S		N.S
209100_at	IFRD2	1		N.S		N.S
209103_s_at	UFD1L	0		N.S	1.15	3.97E-06
209104_s_at	NHP2	1		N.S		N.S
209106_at	NCOA1	0		N.S		N.S
209110_s_at	RGL2	0		N.S		N.S
209111_at	RNF5	0		N.S	-1.16	3.16E-03
209112_at	CDKN1B	115	-1.3	1.53E-10		N.S
209113_s_at	HMG20B	1		N.S		N.S
209115_at	UBA3	0		N.S		N.S

209118_s_at	TUBA1A	1		N.S		N.S
209122_at	ADFP	0		N.S		N.S
209123_at	QDPR	0		N.S		N.S
209124_at	MYD88	9		N.S		N.S
209127_s_at	SART3	0		N.S		N.S
209130_at	SNAP23	0		N.S	-1.17	3.81E-10
209132_s_at	COMMD4	0		N.S		N.S
209135_at	ASPH	0	-1.4	2.55E-04		N.S
209136_s_at	USP10	2		N.S		N.S
209139_s_at	PRKRA	0		N.S	-1.11	1.09E-05
209142_s_at	UBE2G1	0	-1.1	3.31E-03	-1.17	7.30E-12
209143_s_at	CLNS1A	0		N.S		N.S
209146_at	SC4MOL	0	-1.5	1.74E-20	-1.24	8.57E-09
209148_at	RXRB	0		N.S		N.S
209150_s_at	TM9SF1	0		N.S	1.14	9.08E-04
209154_at	TAX1BP3	2		N.S	1.72	3.10E-31
209155_s_at	NT5C2	0		N.S		N.S
209157_at	DNAJA2	0	-1.1	5.16E-05		N.S
209158_s_at	CYTH2	0		N.S		N.S
209162_s_at	PRPF4	0		N.S		N.S
209165_at	AATF	7		N.S	-1.12	6.22E-03
209166_s_at	MAN2B1	0		N.S	1.24	5.97E-11
209171_at	ITPA	0		N.S		N.S
209174_s_at	QRICH1	0		N.S		N.S
209175_at	SEC23IP	0		N.S		N.S
209177_at	NDUFAF3	0		N.S		N.S
209178_at	DHX38	0		N.S		N.S
209187_at	DR1	2	-1.2	1.39E-10	1.17	1.26E-04
209190_s_at	DIAPH1	2		N.S		N.S
209191_at	TUBB6	0		N.S	1.47	1.22E-07
209193_at	PIM1	7	1.7	1.88E-23		N.S
209194_at	CETN2	5		N.S		N.S
209196_at	WDR46	0		N.S		N.S
209198_s_at	SYT11	0		N.S		N.S
209199_s_at	MEF2C	0	-1.2	6.85E-14	-1.39	1.04E-23
209205_s_at	LMO4	0		N.S	1.20	4.34E-05
209206_at	SEC22B	0		N.S		N.S
209208_at	MPDU1	0	1.1	8.40E-04		N.S
209210_s_at	FERMT2	0		N.S		N.S
209213_at	CBR1	0		N.S	-1.18	2.21E-02
209215_at	MFSD10	0		N.S		N.S
209217_s_at	WDR45	0		N.S		N.S
209219_at	RDBP	0		N.S		N.S
209224_s_at	NDUFA2	0		N.S		N.S
209229_s_at	SAPS1	0		N.S	-1.14	5.79E-03
209231_s_at	DCTN5	0		N.S	1.34	1.47E-23
209233_at	EMG1	0		N.S		N.S
209239_at	NFKB1	5		N.S		N.S
209249_s_at	GHITM	0		N.S	1.07	7.05E-05
209252_at	HARS2	0	-1.1	4.07E-02		N.S
209254_at	KLHDC10	0	-1.2	4.75E-06		N.S
209258_s_at	SMC3	20		N.S		N.S
209265_s_at	METTL3	0	-1.4	9.54E-27		N.S
209267_s_at	SLC39A8	0		N.S	-1.23	7.50E-11
209268_at	VPS45	0	-1.1	8.09E-03		N.S
209276_s_at	GLRX	1		N.S	-1.22	1.20E-08
209277_at	TFPI2	0		N.S		N.S
209279_s_at	NSDHL	0		N.S		N.S
209282_at	PRKD2	0		N.S	1.21	1.84E-04
209285_s_at	C3orf63	0	-1.2	8.44E-14	-1.38	4.79E-19

209287_s_at	CDC42EP3	0	1.2	2.81E-05	1.17	1.84E-05
209295_at	TNFRSF10B	74	1.5	2.06E-20	1.65	3.70E-27
209300_s_at	NECAP1	0		N.S		N.S
209302_at	POLR2H	0		N.S		N.S
209303_at	NDUFS4	0		N.S		N.S
209306_s_at	SWAP70	0		N.S		N.S
209308_s_at	BNIP2	0	1.2	1.50E-07	1.17	1.23E-05
209310_s_at	CASP4	3		N.S		N.S
209311_at	BCL2L2	2	-1.2	4.19E-07		N.S
209313_at	GPN1	1		N.S	1.06	3.11E-02
209316_s_at	HBS1L	0		N.S		N.S
209321_s_at	ADCY3	0		N.S		N.S
209323_at	PRKRIR	0		N.S		N.S
209324_s_at	RGS16	0	2.1	5.86E-28	2.16	1.04E-28
209331_s_at	MAX	222	-1.1	1.51E-03		N.S
209336_at	PWP2	0		N.S	-1.16	6.94E-05
209339_at	SIAH2	1		N.S	-1.33	1.60E-15
209340_at	UAP1	0		N.S		N.S
209342_s_at	IKBKB	62		N.S		N.S
209344_at	TPM4	0		N.S	-1.30	5.45E-09
209349_at	RAD50	457		N.S		N.S
209354_at	TNFRSF14	0		N.S		N.S
209358_at	TAF11	0		N.S	1.11	4.07E-06
209363_s_at	MED21	0	-1.2	9.02E-13		N.S
209367_at	STXBP2	0		N.S		N.S
209374_s_at	IGHM	0		N.S		N.S
209375_at	XPC	289	1.2	4.59E-04	2.20	7.82E-39
209377_s_at	HMGN3	0		N.S		N.S
209378_s_at	KIAA1128	0		N.S	1.46	1.17E-16
209383_at	DDIT3	159	1.3	3.35E-05	1.56	1.57E-10
209385_s_at	PROSC	0	-1.1	4.74E-02		N.S
209390_at	TSC1	8	-1.2	1.57E-07		N.S
209391_at	DPM2	0		N.S		N.S
209392_at	ENPP2	1		N.S	-1.32	2.53E-06
209393_s_at	EIF4E2	0		N.S		N.S
209394_at	ASMTL	0	1.1	9.39E-03	1.24	7.62E-07
209398_at	HIST1H1C	0		N.S		N.S
209406_at	BAG2	2	-1.1	8.82E-03	-1.23	1.10E-12
209408_at	KIF2C	2		N.S	-1.16	1.02E-04
209413_at	B4GALT2	0		N.S		N.S
209417_s_at	IFI35	0		N.S		N.S
209421_at	MSH2	207		N.S		N.S
209430_at	BTAF1	0	-1.2	1.54E-03		N.S
209433_s_at	PPAT	0	-1.1	3.11E-04	-1.28	7.18E-15
209435_s_at	ARHGEF2	0	-1.3	5.51E-11	-1.13	3.97E-03
209438_at	PHKA2	0		N.S		N.S
209449_at	LSM2	0		N.S	1.24	3.99E-04
209450_at	OSGEP	0		N.S	-1.09	6.90E-03
209451_at	TANK	16	1.3	6.20E-04	1.35	2.89E-10
209452_s_at	VTI1B	0		N.S		N.S
209456_s_at	FBXW11	0		N.S		N.S
209457_at	DUSP5	1		N.S	-1.45	4.67E-15
209459_s_at	ABAT	0		N.S		N.S
209463_s_at	TAF12	1		N.S	1.19	1.70E-04
209464_at	AURKB	4		N.S	-1.30	2.32E-11
209467_s_at	MKNK1	0		N.S		N.S
209472_at	CCBL2	0		N.S		N.S
209474_s_at	ENTPD1	0		N.S	-1.32	5.47E-15
209475_at	USP15	0		N.S		N.S
209476_at	TMX1	0		N.S		N.S

209477_at	EMD	2		N.S		N.S
209478_at	STRA13	3		N.S		N.S
209479_at	CCDC28A	0		N.S		N.S
209481_at	SNRK	0		N.S	1.24	5.17E-14
209482_at	POP7	0		N.S		N.S
209484_s_at	NSL1	0	-1.2	7.62E-05		N.S
209486_at	UTP3	0		N.S		N.S
209497_s_at	RBM4B	0		N.S	1.20	7.48E-08
209498_at	CEACAM1	0		N.S	3.55	1.00E-29
209503_s_at	PSMC5	0		N.S		N.S
209507_at	RPA3	6		N.S	1.22	2.13E-02
209509_s_at	DPAGT1	0	-1.1	3.23E-08	-1.35	4.55E-23
209510_at	RNF139	0	1.2	7.60E-10	-1.18	1.52E-09
209511_at	POLR2F	0		N.S		N.S
209512_at	HSDL2	0		N.S	1.20	4.70E-06
209514_s_at	RAB27A	0		N.S		N.S
209517_s_at	ASH2L	3		N.S	1.19	4.99E-10
209520_s_at	NCBP1	1	-1.1	8.23E-05	-1.13	4.81E-02
209523_at	TAF2	2		N.S		N.S
209524_at	HDGFRP3	1		N.S		N.S
209531_at	GSTZ1	1		N.S	1.25	4.57E-08
209536_s_at	EHD4	0		N.S	-1.31	1.86E-11
209537_at	EXTL2	1	-1.2	5.31E-12	-1.42	2.65E-24
209538_at	ZNF32	0		N.S		N.S
209539_at	ARHGEF6	1		N.S		N.S
209545_s_at	RIPK2	1		N.S		N.S
209553_at	VPS8	1		N.S		N.S
209556_at	NCDN	0		N.S		N.S
209565_at	RNF113A	0	-1.3	2.43E-12		N.S
209566_at	INSIG2	1	-1.8	5.26E-25	-1.58	5.55E-14
209567_at	RRS1	0		N.S	-1.16	5.01E-04
209568_s_at	RGL1	0	1.1	8.03E-08	1.19	1.66E-12
209571_at	CIR	3		N.S		N.S
209575_at	IL10RB	0		N.S	1.26	3.28E-10
209577_at	PCYT2	0		N.S	-1.20	2.91E-10
209583_s_at	CD200	0	-1.2	3.57E-07	1.09	4.96E-02
209585_s_at	MINPP1	0	-1.2	2.73E-10	1.20	5.31E-10
209593_s_at	TOR1B	0		N.S	1.11	1.29E-02
209595_at	GTF2F2	0		N.S		N.S
209604_s_at	GATA3	3		N.S		N.S
209606_at	CYTIP	0	-1.1	2.03E-06	-1.20	3.30E-15
209608_s_at	ACAT2	0		N.S		N.S
209609_s_at	MRPL9	0		N.S		N.S
209615_s_at	PAK1	8		N.S		N.S
209619_at	CD74	2		N.S		N.S
209620_s_at	ABCB7	1		N.S		N.S
209622_at	STK16	0		N.S		N.S
209624_s_at	MCCC2	0		N.S		N.S
209625_at	PIGH	0		N.S		N.S
209627_s_at	OSBPL3	0		N.S	1.20	4.46E-08
209628_at	NXT2	0		N.S		N.S
209630_s_at	FBXW2	0	-1.1	8.44E-03		N.S
209636_at	NFKB2	0		N.S		N.S
209645_s_at	ALDH1B1	0		N.S		N.S
209647_s_at	SOCS5	1	-1.2	1.30E-04		N.S
209652_s_at	PGF	14		N.S	5.62	3.11E-25
209653_at	KPNA4	0		N.S		N.S
209654_at	KIAA0947	0		N.S		N.S
209657_s_at	HSF2	1		N.S		N.S
209659_s_at	CDC16	3		N.S		N.S

209662_at	CETN3	0	-1.2	4.12E-05		N.S
209665_at	CYB561D2	0		N.S		N.S
209666_s_at	CHUK	25		N.S		N.S
209667_at	CES2	1		N.S	1.68	5.45E-23
209669_s_at	SERBP1	0		N.S	-1.08	1.86E-03
209670_at	TRAC	0		N.S		N.S
209674_at	CRY1	9		N.S		N.S
209678_s_at	PRKCI	0	-1.1	1.43E-03		N.S
209681_at	SLC19A2	3		N.S		N.S
209682_at	CBLB	2	-1.2	1.64E-04	-1.26	5.88E-08
209694_at	PTS	9	1.2	9.89E-04	1.20	1.98E-04
209695_at	PTP4A3	2		N.S	-1.11	1.16E-02
209704_at	MTF2	0		N.S		N.S
209707_at	PIGK	0		N.S		N.S
209708_at	MOXD1	0		N.S		N.S
209709_s_at	HMMR	1	-1.2	9.75E-13	-1.74	8.29E-32
209711_at	SLC35D1	0	1.2	1.07E-03	2.07	6.10E-37
209714_s_at	CDKN3	2		N.S	-1.20	2.55E-02
209722_s_at	SERPINB9	0		N.S		N.S
209724_s_at	ZFP161	0		N.S		N.S
209725_at	UTP20	0		N.S		N.S
209727_at	GM2A	0		N.S	1.36	4.22E-06
209731_at	NTHL1	21		N.S	-1.46	1.36E-16
209732_at	CLEC2B	0		N.S	-1.35	1.95E-03
209748_at	SPAST	0	-1.1	1.56E-02		N.S
209753_s_at	TMPO	1		N.S	-1.13	8.48E-04
209759_s_at	DCI	1		N.S		N.S
209760_at	KIAA0922	0	-1.3	7.87E-13		N.S
209761_s_at	SP110	0		N.S		N.S
209764_at	MGAT3	0		N.S	1.17	1.82E-05
209770_at	BTN3A1	0	-1.4	5.24E-16	-1.35	1.24E-10
209778_at	TRIP11	0	-1.3	1.61E-10		N.S
209780_at	PHTF2	0		N.S		N.S
209786_at	HMGN4	0		N.S	-1.09	5.54E-03
209788_s_at	ERAP1	0		N.S		N.S
209790_s_at	CASP6	20		N.S	1.19	6.21E-09
209795_at	CD69	11		N.S	-2.10	3.13E-23
209796_s_at	CNPY2	0		N.S		N.S
209799_at	PRKAA1	1	1.2	2.06E-04		N.S
209805_at	PMS2	49	-1.2	5.84E-05		N.S
209806_at	HIST1H2BK	0		N.S		N.S
209814_at	ZNF330	0		N.S	-1.09	2.92E-05
209820_s_at	TBL3	0		N.S		N.S
209822_s_at	VLDLR	0	-1.2	1.31E-05	-1.38	5.69E-08
209825_s_at	UCK2	0		N.S	-1.24	2.10E-19
209827_s_at	IL16	2	-1.4	6.73E-23	-1.52	1.10E-26
209829_at	FAM65B	0	-1.8	2.36E-24	-1.50	1.39E-11
209832_s_at	CDT1	46		N.S		N.S
209833_at	CRADD	10		N.S		N.S
209838_at	COPS2	1	1.2	5.08E-08		N.S
209845_at	MKRN1	1		N.S	-1.23	1.78E-12
209849_s_at	RAD51C	51	-1.1	1.05E-05	1.52	5.94E-28
209853_s_at	PSME3	1		N.S		N.S
209861_s_at	METAP2	0		N.S	1.08	6.33E-03
209863_s_at	TP63	45	-1.2	9.35E-06		N.S
209864_at	FRAT2	0		N.S		N.S
209865_at	SLC35A3	0		N.S		N.S
209882_at	RIT1	1	-1.2	3.24E-05		N.S
209883_at	GLT25D2	0		N.S		N.S
209891_at	SPC25	0		N.S		N.S

209894_at	LEPR	0		N.S		N.S
209899_s_at	PUF60	0	1.1	9.52E-04		N.S
209903_s_at	ATR	1057	-1.1	1.64E-04		N.S
209910_at	SLC25A16	0		N.S		N.S
209912_s_at	KIAA0415	0		N.S	1.27	3.73E-04
209916_at	DHTKD1	0		N.S	1.18	1.68E-03
209925_at	OCLN	1		N.S		N.S
209927_s_at	C1orf77	0		N.S		N.S
209928_s_at	MSC	42		N.S	-1.31	2.27E-07
209932_s_at	DUT	8		N.S		N.S
209933_s_at	CD300A	0	-1.3	5.03E-18	-1.73	1.05E-32
209940_at	PARP3	4		N.S		N.S
209943_at	FBXL4	0	-1.4	2.21E-22		N.S
209945_s_at	GSK3B	0		N.S		N.S
209949_at	NCF2	0		N.S		N.S
209953_s_at	CDC37	2		N.S		N.S
209965_s_at	RAD51L3	18		N.S		N.S
209967_s_at	CREM	1		N.S		N.S
209969_s_at	STAT1	31		N.S	-1.27	4.69E-13
209972_s_at	JTV1	0		N.S		N.S
209974_s_at	BUB3	10		N.S		N.S
209989_at	ZNF268	0		N.S	1.23	1.33E-06
209994_s_at	ABCB1	135		N.S		N.S
210004_at	OLR1	1		N.S		N.S
210006_at	ABHD14A	0		N.S		N.S
210010_s_at	SLC25A1	0		N.S		N.S
210024_s_at	UBE2E3	0		N.S		N.S
210027_s_at	APEX1	182		N.S	-1.06	1.74E-02
210028_s_at	ORC3L	0	-1.2	5.27E-06		N.S
210048_at	NAPG	0		N.S		N.S
210052_s_at	TPX2	0	-1.2	1.54E-05	-1.55	4.34E-22
210053_at	TAF5	1	-1.3	3.51E-12		N.S
210054_at	HAUS3	0	1.2	1.45E-08		N.S
210057_at	SMG1	8		N.S		N.S
210058_at	MAPK13	0		N.S	1.18	1.78E-05
210070_s_at	CHKB	1	-1.2	3.94E-04		N.S
210092_at	MAGOH	0		N.S		N.S
210097_s_at	NOL7	0		N.S		N.S
210105_s_at	FYN	4		N.S	-1.18	3.27E-03
210114_at	INVS	2	-1.3	7.30E-12	-1.17	7.14E-04
210115_at	RPL39L	0		N.S		N.S
210117_at	SPAG1	0		N.S	1.20	3.24E-07
210125_s_at	BANF1	1		N.S		N.S
210130_s_at	TM7SF2	0		N.S	1.36	1.14E-09
210137_s_at	DCTD	0		N.S		N.S
210138_at	RGS20	0	-1.2	3.36E-05	1.19	2.38E-02
210144_at	TBC1D22A	0		N.S	-1.34	2.03E-08
210145_at	PLA2G4A	11	-1.6	1.14E-24	-1.23	3.61E-06
210149_s_at	ATP5H	0		N.S		N.S
210154_at	ME2	2	-1.1	6.56E-03	-1.24	3.26E-09
210160_at	PAFAH1B2	0		N.S		N.S
210172_at	SF1	2		N.S		N.S
210176_at	TLR1	0	1.5	3.53E-16	-1.25	4.25E-06
210188_at	GABPA	0		N.S		N.S
210195_s_at	PSG1	1		N.S		N.S
210200_at	WWP2	1		N.S		N.S
210213_s_at	EIF6	0		N.S		N.S
210219_at	SP100	3		N.S		N.S
210235_s_at	PPFIA1	0		N.S		N.S
210241_s_at	TP53TG1	0		N.S	1.68	3.47E-15

Supplemental Table 2

210243_s_at	B4GALT3	0		N.S	-1.17	2.28E-07
210253_at	HTATIP2	1		N.S		N.S
210258_at	RGS13	0		N.S		N.S
210260_s_at	TNFAIP8	0	1.1	1.79E-03	1.11	2.15E-06
210275_s_at	ZFAND5	0	1.2	2.59E-11		N.S
210276_s_at	TRIOBP	0		N.S	1.20	3.79E-05
210278_s_at	AP4S1	0		N.S		N.S
210279_at	GPR18	0		N.S	-1.57	2.24E-06
210284_s_at	MAP3K7IP2	0		N.S	1.21	6.18E-06
210296_s_at	PXMP3	0		N.S		N.S
210301_at	XDH	3		N.S		N.S
210312_s_at	IFT20	0		N.S		N.S
210338_s_at	HSPA8	4		N.S		N.S
210346_s_at	CLK4	1	1.4	2.48E-09	1.58	5.45E-22
210349_at	CAMK4	1		N.S		N.S
210371_s_at	RBBP4	4		N.S		N.S
210377_at	ACSM3	0		N.S		N.S
210378_s_at	SSNA1	0		N.S		N.S
210379_s_at	TLK1	13		N.S	-1.19	5.47E-04
210386_s_at	MTX1	0		N.S		N.S
210396_s_at	BOLA2	0		N.S		N.S
210406_s_at	RAB6A	0		N.S		N.S
210417_s_at	PI4KB	0		N.S	1.21	3.88E-03
210418_s_at	IDH3B	0		N.S	1.08	6.21E-03
210428_s_at	HGS	2		N.S		N.S
210448_s_at	P2RX5	0	-1.1	4.78E-05	-1.71	1.05E-29
210479_s_at	RORA	0		N.S		N.S
210502_s_at	PPIE	1		N.S		N.S
210512_s_at	VEGFA	9		N.S		N.S
210528_at	MR1	3		N.S	1.57	7.39E-08
210531_at	NR2C1	1		N.S		N.S
210538_s_at	BIRC3	2		N.S		N.S
210543_s_at	PRKDC	568		N.S		N.S
210556_at	NFATC3	0		N.S		N.S
210559_s_at	CDC2	572		N.S		N.S
210561_s_at	WSB1	1		N.S		N.S
210573_s_at	POLR3C	0		N.S		N.S
210574_s_at	NUDC	0		N.S	-1.16	3.77E-12
210587_at	INHBE	0		N.S		N.S
210596_at	LOC100129513	0		N.S		N.S
210609_s_at	TP53I3	9		N.S	2.92	4.21E-37
210620_s_at	GTF3C2	0	1.2	3.37E-04		N.S
210625_s_at	AKAP1	0		N.S		N.S
210635_s_at	KLHL20	0		N.S	1.18	6.00E-03
210638_s_at	FBXO9	0		N.S	1.20	4.61E-18
210639_s_at	ATG5	8		N.S	1.13	2.90E-05
210643_at	TNFSF11	3	-1.2	1.25E-05	-1.38	8.15E-14
210649_s_at	ARID1A	0		N.S		N.S
210685_s_at	UBE4B	0		N.S		N.S
210705_s_at	TRIM5	0		N.S	-1.22	5.61E-07
210732_s_at	LGALS8	0		N.S	-1.27	2.87E-07
210733_at	TRAM1	0		N.S		N.S
210740_s_at	ITPK1	0		N.S		N.S
210748_at	LOC100129624	0		N.S		N.S
210752_s_at	MLX	0		N.S		N.S
210758_at	PSIP1	0		N.S	-1.24	4.96E-09
210759_s_at	PSMA1	0		N.S		N.S
210771_at	PPARA	1		N.S		N.S
210774_s_at	NCOA4	4		N.S	1.20	5.86E-17
210785_s_at	C1orf38	0		N.S		N.S

Supplemental Table 2

210786_s_at	FLI1	6	-1.3	1.89E-06	1.26	4.10E-06
210793_s_at	NUP98	3		N.S		N.S
210797_s_at	OASL	0	1.3	1.20E-06		N.S
210811_s_at	DDX49	0		N.S		N.S
210813_s_at	XRCC4	166		N.S		N.S
210817_s_at	CALCOCO2	0		N.S		N.S
210822_at	RPL13P5	0		N.S		N.S
210868_s_at	ELOVL6	0		N.S	-1.54	4.45E-14
210878_s_at	KDM3B	0		N.S		N.S
210889_s_at	FCGR2B	1		N.S		N.S
210892_s_at	GTF2I	0		N.S		N.S
210895_s_at	CD86	7		N.S		N.S
210907_s_at	PDCD10	0		N.S		N.S
210908_s_at	PFDN5	0		N.S		N.S
210910_s_at	POMZP3	0	1.2	5.24E-06	1.60	3.71E-20
210926_at	ACTBL3	0		N.S		N.S
210942_s_at	ST3GAL6	0	-1.4	6.89E-08	-1.23	6.36E-03
210943_s_at	LYST	0		N.S		N.S
210944_s_at	CAPN3	0	-1.3	9.63E-04		N.S
210946_at	PPAP2A	0		N.S	1.18	3.17E-04
210947_s_at	MSH3	29		N.S		N.S
210949_s_at	EIF3C	0		N.S		N.S
210959_s_at	SRD5A1	0		N.S		N.S
210968_s_at	RTN4	0		N.S	-1.24	6.00E-24
210971_s_at	ARNTL	3		N.S		N.S
210976_s_at	PFKM	0		N.S	-1.35	1.15E-17
210978_s_at	TAGLN2	0		N.S		N.S
210983_s_at	MCM7	12	1.2	3.96E-04		N.S
210996_s_at	YWHAE	0		N.S		N.S
211009_s_at	ZNF271	0	-1.2	1.90E-10		N.S
211015_s_at	HSPA4	1		N.S		N.S
211026_s_at	MGLL	0		N.S	-1.27	2.21E-17
211031_s_at	CLIP2	0		N.S		N.S
211033_s_at	PEX7	0		N.S		N.S
211034_s_at	C12orf51	0		N.S	1.17	6.94E-05
211038_s_at	CROCCL1	0		N.S	1.20	1.45E-09
211043_s_at	CLTB	0		N.S		N.S
211048_s_at	PDIA4	0		N.S		N.S
211064_at	ZNF493	0		N.S	-1.27	8.22E-04
211071_s_at	MLLT11	0	-1.1	2.94E-06		N.S
211074_at	FOLR1	1		N.S		N.S
211075_s_at	CD47	1		N.S	-1.48	3.39E-29
211089_s_at	NEK3	0		N.S		N.S
211136_s_at	CLPTM1	0		N.S		N.S
211168_s_at	UPF1	5		N.S		N.S
211200_s_at	EFCAB2	0		N.S		N.S
211212_s_at	ORC5L	0	-1.2	1.33E-09		N.S
211250_s_at	SH3BP2	1		N.S	1.20	5.35E-04
211275_s_at	GYG1	0		N.S		N.S
211276_at	TCEAL2	0		N.S		N.S
211284_s_at	GRN	1		N.S	1.44	3.85E-20
211285_s_at	UBE3A	8		N.S		N.S
211297_s_at	CDK7	19		N.S		N.S
211317_s_at	CFLAR	10	-1.2	1.73E-05	1.17	1.04E-02
211330_s_at	HFE	2		N.S		N.S
211339_s_at	ITK	0		N.S		N.S
211352_s_at	NCOA3	0		N.S	1.18	4.24E-02
211358_s_at	CIZ1	0		N.S		N.S
211364_at	MTAP	0		N.S		N.S
211368_s_at	CASP1	52		N.S		N.S

211391_s_at	PATZ1	0		N.S		N.S
211404_s_at	APLP2	2		N.S		N.S
211406_at	IER3IP1	0		N.S		N.S
211450_s_at	MSH6	69	1.2	1.56E-03	-1.19	1.31E-02
211475_s_at	BAG1	1	1.1	4.28E-08	1.28	7.50E-21
211501_s_at	EIF3B	0		N.S		N.S
211502_s_at	PFTK1	0		N.S		N.S
211505_s_at	STAU1	0		N.S		N.S
211512_s_at	OGFR	0		N.S		N.S
211543_s_at	GRK6	1		N.S	-1.23	2.48E-06
211558_s_at	DHPS	3		N.S	-1.20	8.41E-07
211563_s_at	C19orf2	0		N.S		N.S
211569_s_at	HADH	0		N.S		N.S
211596_s_at	LRIG1	0		N.S	-1.21	1.13E-05
211600_at	PTPRO	0		N.S		N.S
211612_s_at	IL13RA1	0		N.S		N.S
211623_s_at	FBL	0		N.S		N.S
211662_s_at	VDAC2	0		N.S		N.S
211665_s_at	SOS2	1		N.S	1.23	1.03E-03
211671_s_at	NR3C1	1		N.S		N.S
211672_s_at	ARPC4	0		N.S		N.S
211676_s_at	IFNGR1	0	-1.1	9.74E-06	-1.28	2.32E-16
211678_s_at	RNF114	0		N.S		N.S
211684_s_at	DYNC1I2	0		N.S		N.S
211685_s_at	NCALD	0	-1.1	5.11E-04		N.S
211686_s_at	MAK16	0	1.1	1.50E-05	-1.15	5.29E-05
211692_s_at	BBC3	62	2.3	1.58E-31	2.01	7.08E-29
211702_s_at	USP32	0		N.S		N.S
211707_s_at	IQCB1	1	-1.1	1.51E-02		N.S
211715_s_at	BDH1	0		N.S	-1.25	5.39E-10
211717_at	ANKRD40	0		N.S		N.S
211721_s_at	ZNF551	0		N.S		N.S
211725_s_at	BID	86		N.S		N.S
211727_s_at	COX11	2	1.1	1.47E-06	1.20	1.12E-12
211742_s_at	EVI2B	0	-1.1	1.48E-06	-1.33	9.13E-22
211749_s_at	VAMP3	0		N.S		N.S
211752_s_at	NDUFS7	0		N.S		N.S
211753_s_at	RLN1	0	-1.2	1.56E-02		N.S
211754_s_at	SLC25A17	0		N.S		N.S
211755_s_at	ATP5F1	0		N.S		N.S
211763_s_at	UBE2B	4		N.S	1.15	2.46E-06
211764_s_at	UBE2D1	2		N.S		N.S
211767_at	GINS4	1		N.S	1.45	4.65E-12
211774_s_at	MMACHC	0	-1.2	5.99E-10		N.S
211783_s_at	MTA1	6		N.S		N.S
211784_s_at	SFRS1	3		N.S		N.S
211787_s_at	EIF4A1	0		N.S		N.S
211792_s_at	CDKN2C	7		N.S		N.S
211796_s_at	TRBC1	0		N.S	-1.22	1.01E-02
211810_s_at	GALC	2	1.2	5.28E-05		N.S
211819_s_at	SORBS1	0		N.S		N.S
211825_s_at	EWSR1	5		N.S		N.S
211828_s_at	TNIK	0		N.S	-1.30	1.21E-07
211833_s_at	BAX	779		N.S	1.47	6.05E-15
211913_s_at	MERTK	0		N.S		N.S
211926_s_at	MYH9	0		N.S		N.S
211928_at	DYNC1H1	0		N.S	1.22	2.57E-06
211935_at	ARL6IP1	0		N.S		N.S
211936_at	HSPA5	6		N.S		N.S
211938_at	EIF4B	2		N.S		N.S

Supplemental Table 2

211946_s_at	BAT2D1	0		N.S		N.S
211950_at	UBR4	0		N.S		N.S
211955_at	IPO5	0		N.S		N.S
211956_s_at	EIF1	1		N.S		N.S
211960_s_at	RAB7A	0		N.S		N.S
211962_s_at	ZFP36L1	0		N.S		N.S
211963_s_at	ARPC5	0		N.S		N.S
211967_at	TMEM123	0		N.S	-1.11	2.26E-04
211971_s_at	LRPPRC	0		N.S		N.S
211975_at	ARFGAP2	0		N.S		N.S
211985_s_at	CALM1	0		N.S		N.S
211987_at	TOP2B	1		N.S	-1.19	1.44E-07
211989_at	SMARCE1	0		N.S		N.S
211990_at	HLA-DPA1	0		N.S		N.S
211994_at	WNK1	0	-1.1	3.23E-03		N.S
211998_at	H3F3B	0	1.8	5.93E-22		N.S
212005_at	C1orf144	0	1.2	2.18E-02		N.S
212007_at	UBXN4	0		N.S		N.S
212009_s_at	STIP1	1		N.S	-1.28	9.71E-10
212016_s_at	PTBP1	0		N.S		N.S
212017_at	FAM168B	0	1.2	6.70E-07		N.S
212021_s_at	MKI67	96		N.S	-1.18	1.37E-04
212025_s_at	FLII	0		N.S		N.S
212027_at	RBM25	0		N.S		N.S
212032_s_at	PTOV1	0		N.S	-1.18	1.55E-03
212036_s_at	PNN	1		N.S		N.S
212038_s_at	VDAC1	2		N.S	-1.06	4.38E-02
212040_at	TGOLN2	0		N.S		N.S
212041_at	ATP6V0D1	0		N.S	1.11	9.56E-07
212044_s_at	RPL27A	0		N.S		N.S
212047_s_at	RNF167	0		N.S		N.S
212048_s_at	YARS	0		N.S		N.S
212050_at	WIPF2	0		N.S	1.18	6.00E-08
212052_s_at	TBC1D9B	0		N.S	-1.21	5.51E-10
212053_at	PDXDC1	0		N.S		N.S
212055_at	C18orf10	0		N.S		N.S
212057_at	KIAA0182	0	-1.2	1.69E-04		N.S
212058_at	SR140	0		N.S	-1.12	5.88E-04
212059_s_at	TRPC4AP	0		N.S		N.S
212066_s_at	USP34	0		N.S		N.S
212069_s_at	BAT2L	0		N.S		N.S
212072_s_at	CSNK2A1	0		N.S		N.S
212077_at	CALD1	0		N.S		N.S
212078_s_at	MLL	44		N.S		N.S
212082_s_at	MYL6	0		N.S		N.S
212083_at	TEX261	0		N.S		N.S
212087_s_at	ERAL1	0		N.S	1.16	2.04E-04
212088_at	PMPCA	0		N.S		N.S
212090_at	GRINA	0		N.S		N.S
212092_at	PEG10	0		N.S		N.S
212098_at	LOC151162	0		N.S	-1.23	5.56E-05
212099_at	RHOB	15		N.S		N.S
212100_s_at	POLDIP3	0		N.S		N.S
212101_at	KPNA6	0		N.S		N.S
212106_at	FAF2	0		N.S		N.S
212110_at	SLC39A14	0		N.S		N.S
212112_s_at	STX12	0		N.S	1.12	5.55E-03
212115_at	HN1L	0		N.S		N.S
212118_at	TRIM27	0		N.S		N.S
212119_at	RHOQ	0		N.S	-1.21	2.69E-08

212121_at	TCTN3	0		N.S		N.S
212124_at	ZMIZ1	0	-1.1	6.93E-03	-1.13	9.47E-03
212126_at	CBX5	0		N.S	1.10	3.52E-02
212129_at	NIPA2	0		N.S	-1.13	1.02E-06
212131_at	LSM14A	0		N.S		N.S
212140_at	PDS5A	1	-1.1	1.02E-05	-1.15	8.03E-08
212141_at	MCM4	9		N.S		N.S
212144_at	UNC84B	0		N.S	-1.59	2.21E-17
212145_at	MRPS27	0	-1.1	2.43E-02		N.S
212150_at	EFR3A	0		N.S	-1.15	2.92E-04
212155_at	RNF187	0		N.S	-1.10	1.87E-03
212160_at	XPOT	0		N.S	-1.11	1.70E-02
212161_at	AP2A2	0		N.S		N.S
212165_at	TMEM183A	0		N.S		N.S
212166_at	XPO7	0		N.S		N.S
212170_at	RBM12	0		N.S	-1.34	1.66E-10
212177_at	SFRS18	0		N.S		N.S
212186_at	ACACA	0		N.S	-1.20	1.30E-05
212188_at	KCTD12	0	-1.2	2.63E-03	-1.54	1.08E-09
212189_s_at	COG4	1		N.S		N.S
212190_at	SERPINE2	0		N.S		N.S
212193_s_at	LARP1	0		N.S		N.S
212194_s_at	TM9SF4	0		N.S		N.S
212199_at	MRFAP1L1	0	-1.1	3.34E-02	1.08	4.74E-02
212200_at	ANKLE2	0		N.S		N.S
212202_s_at	TMEM87A	0	1.3	1.69E-23	1.11	4.46E-03
212208_at	MED13L	0		N.S		N.S
212211_at	ANKRD17	0	-1.3	1.00E-13		N.S
212217_at	PREPL	0	-1.1	3.00E-02		N.S
212218_s_at	FASN	2		N.S	-1.43	4.22E-17
212219_at	PSME4	1		N.S		N.S
212228_s_at	COQ9	4		N.S		N.S
212231_at	FBXO21	0	1.3	9.30E-18		N.S
212232_at	FNBP4	0		N.S		N.S
212233_at	MAP1B	0		N.S		N.S
212238_at	ASXL1	0		N.S		N.S
212242_at	TUBA4A	0		N.S	-1.19	1.46E-15
212244_at	GCOM1	0	-1.1	2.49E-03		N.S
212245_at	MCFD2	0		N.S		N.S
212247_at	NUP205	0		N.S	-1.13	3.38E-02
212249_at	PIK3R1	0		N.S	1.19	1.61E-02
212251_at	MTDH	0		N.S	-1.23	2.66E-26
212255_s_at	ATP2C1	0	-1.2	6.47E-14	-1.21	1.81E-07
212263_at	QKI	0		N.S	-1.14	5.04E-03
212266_s_at	SFRS5	0	1.2	3.97E-14	-1.32	9.38E-22
212271_at	MAPK1	79		N.S	1.17	4.46E-04
212274_at	LPIN1	0		N.S	-1.24	6.35E-04
212277_at	MTMR4	0		N.S		N.S
212281_s_at	TMEM97	0	1.1	4.07E-05		N.S
212287_at	SUZ12	0		N.S		N.S
212293_at	HIPK1	1		N.S		N.S
212296_at	PSMD14	1		N.S		N.S
212297_at	ATP13A3	0		N.S		N.S
212300_at	TXLNA	0	1.1	1.92E-02	1.22	7.80E-08
212302_at	RTF1	0		N.S		N.S
212308_at	CLASP2	0		N.S		N.S
212310_at	MIA3	0		N.S		N.S
212311_at	KIAA0746	0		N.S	-1.21	9.74E-03
212312_at	BCL2L1	215		N.S	1.33	6.64E-17
212313_at	CHMP7	0		N.S		N.S

212317_at	TNPO3	0		N.S		N.S
212320_at	TUBB	0		N.S		N.S
212323_s_at	VPS13D	0		N.S		N.S
212329_at	SCAP	1		N.S		N.S
212330_at	TFDP1	8		N.S		N.S
212333_at	FAM98A	0		N.S		N.S
212340_at	YIPF6	0		N.S		N.S
212345_s_at	CREB3L2	0		N.S	-1.13	5.35E-03
212348_s_at	KDM1	1		N.S	-1.17	9.60E-04
212350_at	TBC1D1	0		N.S		N.S
212351_at	EIF2B5	0		N.S		N.S
212352_s_at	TMED10	0		N.S		N.S
212355_at	KIAA0323	0		N.S	1.11	1.03E-05
212357_at	FAM168A	0		N.S	1.17	1.85E-05
212360_at	AMPD2	0		N.S	-1.25	8.50E-09
212366_at	ZNF292	0	-1.2	1.30E-06	-1.14	6.64E-03
212371_at	PPPDE1	0		N.S	1.38	9.11E-24
212372_at	MYH10	0		N.S	1.57	2.47E-22
212376_s_at	EP400	3		N.S		N.S
212380_at	FTSJD2	0		N.S		N.S
212381_at	USP24	0		N.S		N.S
212383_at	ATP6V0A1	0		N.S		N.S
212398_at	RDX	0		N.S		N.S
212400_at	FAM102A	0		N.S	1.46	1.67E-12
212401_s_at	CDC2L2	0		N.S		N.S
212402_at	ZC3H13	0	-1.2	1.09E-05		N.S
212403_at	UBE3B	0	-1.1	7.20E-03		N.S
212405_s_at	METTL13	0	-1.2	6.39E-08		N.S
212406_s_at	PCMTD2	0		N.S	1.13	4.72E-03
212408_at	TOR1AIP1	2		N.S	-1.20	1.58E-12
212410_at	EFHA1	0		N.S		N.S
212411_at	IMP4	0		N.S		N.S
212415_at	SEPT6	4		N.S		N.S
212420_at	ELF1	1		N.S		N.S
212422_at	PDCD11	0		N.S		N.S
212430_at	RBM38	1		N.S		N.S
212434_at	GRPEL1	0		N.S	-1.21	5.55E-15
212436_at	TRIM33	0		N.S	1.18	3.25E-10
212437_at	CENPB	3		N.S		N.S
212439_at	IP6K1	1		N.S		N.S
212440_at	SNRNP27	0	1.3	7.96E-15	1.43	4.17E-27
212441_at	KIAA0232	0		N.S	1.22	6.73E-08
212443_at	NBEAL2	0		N.S		N.S
212445_s_at	NEDD4L	0		N.S		N.S
212446_s_at	LASS6	1		N.S		N.S
212447_at	KBTBD2	0	1.2	6.33E-04		N.S
212449_s_at	LYPLA1	0		N.S		N.S
212451_at	SECISBP2L	0		N.S		N.S
212453_at	KIAA1279	0	-1.3	8.66E-16		N.S
212456_at	KIAA0664	0		N.S		N.S
212458_at	SPRED2	0	1.2	5.24E-03	-1.42	2.59E-20
212462_at	MYST4	2		N.S		N.S
212465_at	SETD3	0		N.S		N.S
212467_at	DNAJC13	0	-1.1	1.26E-03	-1.13	1.28E-02
212470_at	SPAG9	0		N.S		N.S
212471_at	AVL9	0		N.S		N.S
212473_s_at	MICAL2	0		N.S		N.S
212476_at	ACAP2	0		N.S	1.18	4.55E-06
212479_s_at	RMND5A	0		N.S		N.S
212483_at	NIPBL	2	-1.1	6.27E-05		N.S

212487_at	GPATCH8	0		N.S		N.S
212491_s_at	DNAJC8	0		N.S		N.S
212499_s_at	FCF1	0		N.S		N.S
212500_at	ADO	4		N.S		N.S
212501_at	CEBPB	1		N.S	1.36	2.80E-07
212505_s_at	KIAA0892	0		N.S		N.S
212507_at	TMEM131	0		N.S	1.21	1.73E-06
212508_at	MOAP1	1	-1.1	2.88E-02		N.S
212509_s_at	MXRA7	0		N.S		N.S
212512_s_at	CARM1	3		N.S	-1.26	2.87E-09
212515_s_at	DDX3X	1	1.2	4.90E-06	-1.16	4.41E-02
212517_at	ATRN	1	-1.4	8.58E-14		N.S
212520_s_at	SMARCA4	10		N.S	-1.16	2.89E-04
212523_s_at	KIAA0146	0		N.S		N.S
212526_at	SPG20	0		N.S		N.S
212527_at	PPPDE2	0		N.S		N.S
212528_at	D15Wsu75e	0		N.S		N.S
212529_at	LSM12	0		N.S		N.S
212530_at	NEK7	0	-1.2	1.13E-11		N.S
212536_at	ATP11B	0		N.S		N.S
212538_at	DOCK9	0		N.S		N.S
212539_at	CHD1L	1		N.S		N.S
212540_at	CDC34	3		N.S	1.10	1.58E-02
212541_at	FLAD1	0		N.S		N.S
212542_s_at	PHIP	91		N.S	1.15	9.00E-05
212543_at	AIM1	0		N.S		N.S
212544_at	ZNHIT3	0		N.S	1.22	1.21E-08
212548_s_at	FRYL	0	-1.4	6.51E-22		N.S
212550_at	STAT5B	3		N.S		N.S
212556_at	SCRIB	1		N.S	1.32	1.52E-16
212557_at	ZNF451	0		N.S	1.26	3.64E-06
212558_at	SPRY1	0		N.S		N.S
212560_at	SORL1	0		N.S		N.S
212561_at	DENND5A	0		N.S	1.24	3.97E-06
212568_s_at	DLAT	0		N.S	-1.16	7.25E-06
212571_at	CHD8	0		N.S		N.S
212572_at	STK38L	0	-1.2	2.94E-03		N.S
212573_at	ENDOD1	0		N.S	-1.43	5.72E-21
212576_at	MGRN1	0		N.S	1.18	2.53E-03
212579_at	SMCHD1	0	-1.1	1.62E-02	-1.16	1.15E-03
212584_at	AQR	0		N.S		N.S
212585_at	OSBPL8	0		N.S		N.S
212586_at	CAST	36		N.S	1.11	6.13E-03
212587_s_at	PTPRC	14		N.S	-1.59	1.55E-26
212589_at	RRAS2	0	-1.1	2.27E-04		N.S
212592_at	IGJ	0		N.S		N.S
212595_s_at	DAZAP2	0	1.1	6.76E-06		N.S
212597_s_at	HMGXB4	0	1.3	7.13E-21	-1.11	3.82E-02
212600_s_at	UQCRC2	0		N.S		N.S
212601_at	ZZEF1	0		N.S		N.S
212603_at	MRPS31	0		N.S		N.S
212608_s_at	NUDT3	0		N.S		N.S
212610_at	PTPN11	14		N.S		N.S
212611_at	DTX4	0		N.S		N.S
212612_at	RCOR1	0		N.S		N.S
212613_at	BTN3A2	0	-1.3	5.11E-11	-1.48	4.67E-16
212614_at	ARID5B	0	-1.5	5.21E-22	-1.27	6.03E-10
212621_at	TMEM194A	0		N.S	1.26	1.00E-07
212623_at	TMEM41B	0	-1.1	4.40E-02	1.11	3.60E-02
212625_at	STX10	0		N.S		N.S

212627_s_at	EXOSC7	0		N.S		N.S
212630_at	EXOC3	0		N.S		N.S
212634_at	KIAA0776	0		N.S		N.S
212637_s_at	WWP1	1		N.S	1.39	3.87E-15
212640_at	PTPLB	0		N.S	-1.13	1.84E-05
212642_s_at	HIVEP2	0	-1.2	5.76E-04	-1.20	3.85E-09
212643_at	MAPK1IP1L	0	1.1	6.51E-08	-1.06	6.56E-05
212646_at	RFTN1	0		N.S	1.10	9.27E-05
212649_at	DHX29	0		N.S		N.S
212653_s_at	EHBP1	0	-1.1	9.58E-05		N.S
212655_at	ZCCHC14	0		N.S		N.S
212656_at	TSFM	0		N.S		N.S
212658_at	LHFPL2	0		N.S		N.S
212665_at	TIPARP	0	1.2	1.28E-05		N.S
212666_at	SMURF1	1	1.4	1.53E-11		N.S
212673_at	METAP1	0		N.S		N.S
212674_s_at	DHX30	0		N.S		N.S
212677_s_at	CEP68	0	-1.2	1.10E-02		N.S
212685_s_at	TBL2	0	-1.2	2.99E-15	-1.28	2.78E-20
212688_at	PIK3CB	0	-1.1	1.63E-03		N.S
212689_s_at	KDM3A	0	-1.5	3.34E-14	-2.19	1.09E-25
212690_at	DDHD2	0		N.S		N.S
212692_s_at	LRBA	0		N.S		N.S
212693_at	MDN1	0		N.S	-1.18	4.58E-06
212694_s_at	PCCB	0		N.S		N.S
212696_s_at	RNF4	2		N.S	-1.13	4.39E-02
212697_at	FAM134C	0	-1.1	1.77E-02		N.S
212698_s_at	SEPT10	0		N.S		N.S
212699_at	SCAMP5	0		N.S		N.S
212704_at	ZCCHC11	0	-1.2	1.39E-05		N.S
212707_s_at	LOC100133005	0		N.S	-1.27	6.43E-04
212708_at	MSL1	1	-1.2	5.71E-06		N.S
212712_at	CAMSAP1	0		N.S		N.S
212716_s_at	EIF3K	0		N.S		N.S
212718_at	PAPOLA	1		N.S	-1.11	1.93E-03
212721_at	SFRS12	0	-1.1	2.47E-03		N.S
212723_at	JMJD6	0	-1.2	1.55E-09	-1.28	3.86E-12
212726_at	PHF2	0		N.S	1.17	1.02E-02
212729_at	DLG3	0		N.S		N.S
212731_at	ANKRD46	0	-1.2	5.86E-04	1.20	2.51E-10
212735_at	KIAA0226	0		N.S		N.S
212739_s_at	NME4	0		N.S		N.S
212742_at	RNF115	0		N.S		N.S
212745_s_at	BBS4	0		N.S	1.64	3.54E-14
212746_s_at	CEP170	0	-1.3	2.77E-03		N.S
212747_at	ANKS1A	0	-1.5	1.52E-18		N.S
212751_at	UBE2N	15		N.S		N.S
212752_at	CLASP1	0		N.S		N.S
212753_at	PCGF3	0		N.S		N.S
212754_s_at	MON2	0		N.S	1.39	1.03E-24
212756_s_at	UBR2	2	-1.2	6.38E-09		N.S
212758_s_at	ZEB1	1	-1.2	5.76E-03		N.S
212763_at	CAMSAP1L1	0		N.S		N.S
212766_s_at	ISG20L2	0		N.S		N.S
212767_at	MTG1	0		N.S		N.S
212771_at	FAM171A1	0		N.S		N.S
212773_s_at	TOMM20	0		N.S		N.S
212774_at	ZNF238	0	-1.3	1.16E-09		N.S
212779_at	KIAA1109	0	-1.2	1.05E-03		N.S
212780_at	SOS1	0	-1.2	5.40E-06		N.S

212784_at	CIC	5		N.S	1.29	7.83E-06
212785_s_at	LARP7	0		N.S		N.S
212786_at	CLEC16A	0		N.S		N.S
212787_at	YLPM1	0		N.S		N.S
212789_at	NCAPD3	0		N.S	-1.11	3.27E-03
212791_at	C1orf216	0		N.S	-1.12	1.03E-02
212794_s_at	KIAA1033	0		N.S	-1.15	1.20E-02
212802_s_at	GAPVD1	0	-1.2	1.64E-07		N.S
212810_s_at	SLC1A4	0		N.S		N.S
212813_at	JAM3	0		N.S		N.S
212815_at	ASCC3	0	-1.1	1.22E-03	1.22	4.89E-08
212817_at	DNAJB5	0	-1.2	1.25E-02	1.45	2.27E-17
212819_at	ASB1	1		N.S		N.S
212820_at	DMXL2	0		N.S		N.S
212824_at	FUBP3	0		N.S		N.S
212825_at	PAXIP1	12	1.1	9.90E-03		N.S
212826_s_at	SLC25A6	0		N.S	1.10	1.05E-04
212830_at	MEGF9	0	-1.2	2.72E-03		N.S
212832_s_at	CKAP5	0	-1.1	1.92E-02	-1.18	5.05E-07
212833_at	SLC25A46	0		N.S	1.15	1.04E-09
212834_at	DDX52	0		N.S	1.11	1.76E-02
212836_at	POLD3	3		N.S		N.S
212837_at	FAM175B	0		N.S		N.S
212838_at	DNMBP	0	-1.2	8.26E-07		N.S
212841_s_at	PPFIBP2	0	-1.2	3.46E-04	-1.16	1.23E-03
212846_at	RRP1B	1		N.S		N.S
212847_at	FUBP1	0		N.S	-1.17	4.27E-03
212851_at	DCUN1D4	0	-1.4	3.06E-12		N.S
212858_at	PAQR4	0		N.S		N.S
212860_at	ZDHHC18	0		N.S		N.S
212861_at	MFSD5	0		N.S		N.S
212862_at	CDS2	0		N.S	1.26	2.31E-19
212867_at	NCOA2	0	-1.1	1.24E-02		N.S
212871_at	MAPKAPK5	2		N.S		N.S
212873_at	HMHA1	0		N.S	-1.30	8.69E-08
212875_s_at	C2CD2	0	-1.4	5.97E-12		N.S
212876_at	B4GALT4	0		N.S		N.S
212877_at	KLC1	0		N.S		N.S
212880_at	WDR7	0		N.S		N.S
212881_at	PIAS4	3		N.S	1.14	1.13E-02
212885_at	MPHOSPH10	0		N.S	-1.11	2.30E-04
212886_at	CCDC69	0		N.S		N.S
212887_at	SEC23A	0	-1.2	1.21E-06		N.S
212891_s_at	GADD45GIP1	1		N.S		N.S
212893_at	ZZZ3	0		N.S		N.S
212894_at	SUPV3L1	0		N.S		N.S
212896_at	SKIV2L2	0		N.S		N.S
212898_at	KIAA0406	0	-1.2	1.43E-04		N.S
212901_s_at	CSTF2T	0		N.S		N.S
212902_at	SEC24A	0		N.S	-1.20	3.03E-06
212904_at	LRRC47	0		N.S	1.11	5.85E-04
212906_at	GRAMD1B	0		N.S		N.S
212907_at	SLC30A1	0	2.2	7.07E-24	2.03	1.78E-31
212908_at	DNAJC16	0	-1.2	8.74E-09	1.12	3.35E-05
212910_at	THAP11	0	-1.3	1.03E-14		N.S
212916_at	PHF8	0		N.S		N.S
212918_at	RECQL	21		N.S		N.S
212919_at	DCP2	0	-1.3	6.37E-11		N.S
212926_at	SMC5	41		N.S		N.S
212928_at	TSPYL4	0	-1.1	1.54E-07		N.S

212929_s_at	FAM21A	0		N.S		N.S
212930_at	ATP2B1	0		N.S		N.S
212931_at	TCF20	1		N.S		N.S
212934_at	UBXN2B	0		N.S		N.S
212936_at	FAM172A	0		N.S	-1.30	1.50E-12
212943_at	KIAA0528	0	-1.2	1.02E-07		N.S
212944_at	SLC5A3	0	-1.5	6.34E-17		N.S
212945_s_at	MGA	2	-1.2	2.68E-02		N.S
212947_at	SLC9A8	0		N.S		N.S
212948_at	CAMTA2	0		N.S		N.S
212949_at	NCAPH	0		N.S	-1.22	1.89E-05
212954_at	DYRK4	0		N.S		N.S
212955_s_at	POLR2I	0		N.S		N.S
212959_s_at	GNPTAB	0		N.S	1.19	3.15E-08
212963_at	TM2D1	0		N.S		N.S
212964_at	HIC2	0	1.2	7.71E-16		N.S
212973_at	RPIA	0		N.S		N.S
212977_at	CXCR7	0		N.S	-1.55	7.24E-17
212978_at	LRRC8B	0	1.4	1.33E-18	-1.19	8.18E-05
212979_s_at	FAM115A	0	-1.3	2.20E-06		N.S
212983_at	HRAS	56		N.S	1.66	2.83E-26
212984_at	ATF2	20	-1.3	8.69E-10		N.S
212986_s_at	TLK2	1		N.S		N.S
212989_at	SGMS1	0		N.S	-1.22	4.81E-05
212990_at	SYNJ1	0	-1.3	1.66E-07	1.26	4.46E-10
212994_at	THOC2	0		N.S		N.S
213000_at	MORC3	0		N.S		N.S
213002_at	MARCKS	2		N.S		N.S
213008_at	FANCI	28		N.S	1.19	2.73E-02
213009_s_at	TRIM37	0		N.S		N.S
213010_at	PRKCDBP	0		N.S		N.S
213011_s_at	TPI1	0		N.S		N.S
213012_at	NEDD4	4		N.S		N.S
213016_at	BBX	0	-1.2	2.05E-05	-1.13	2.66E-03
213017_at	ABHD3	0		N.S	1.22	3.71E-03
213019_at	RANBP6	0		N.S		N.S
213025_at	THUMPD1	0		N.S		N.S
213026_at	ATG12	1		N.S		N.S
213031_s_at	WDR73	0	1.1	3.08E-02	1.19	9.95E-12
213035_at	ANKRD28	0		N.S	-1.20	5.53E-09
213038_at	RNF19B	0	1.6	1.08E-15	2.06	1.45E-29
213039_at	ARHGEF18	0		N.S		N.S
213041_s_at	ATP5D	0		N.S		N.S
213043_s_at	MED24	0		N.S		N.S
213044_at	ROCK1	1	-1.1	1.44E-04		N.S
213046_at	PABPN1	0		N.S		N.S
213048_s_at	SET	960		N.S		N.S
213052_at	PRKAR2A	0		N.S		N.S
213054_at	HAUS5	0		N.S	1.13	4.18E-02
213058_at	TTC28	0		N.S		N.S
213060_s_at	CHI3L2	0		N.S		N.S
213061_s_at	NTAN1	0		N.S	1.17	2.51E-06
213065_at	ZFC3H1	0	-1.4	1.70E-23		N.S
213070_at	PIK3C2A	1	-1.3	2.44E-15	1.13	5.01E-03
213073_at	ZFYVE26	0		N.S		N.S
213076_at	ITPKC	0		N.S	1.51	3.01E-19
213077_at	YTHDC2	0		N.S		N.S
213079_at	TSR2	0		N.S		N.S
213086_s_at	CSNK1A1	0	1.1	6.26E-04	1.15	1.53E-10
213088_s_at	DNAJC9	0	1.3	2.28E-28	1.15	8.62E-12

213090_s_at	TAF4	1	-1.1	4.27E-04		N.S
213097_s_at	DNAJC2	0		N.S		N.S
213098_at	RQCD1	0	-1.1	1.85E-02		N.S
213101_s_at	ACTR3	0		N.S		N.S
213103_at	STARD13	1	-1.5	7.15E-09		N.S
213105_s_at	C16orf42	0		N.S		N.S
213106_at	ATP8A1	0		N.S	-1.55	4.08E-19
213111_at	PIKFYVE	0		N.S		N.S
213113_s_at	SLC43A3	0		N.S	-1.15	4.58E-06
213115_at	ATG4A	0		N.S	1.24	4.84E-11
213117_at	KLHL9	0	-1.3	2.83E-10		N.S
213120_at	UHRF1BP1L	0		N.S		N.S
213122_at	TSPYL5	0		N.S	-1.16	3.01E-02
213126_at	MED8	0		N.S		N.S
213132_s_at	MCAT	4		N.S		N.S
213133_s_at	GCSH	2		N.S		N.S
213137_s_at	PTPN2	0		N.S		N.S
213138_at	ARID5A	0	1.2	2.40E-02	-1.18	7.28E-03
213140_s_at	SS18L1	0		N.S	1.19	1.61E-05
213141_at	PSKH1	0		N.S		N.S
213145_at	FBXL14	0		N.S	-1.18	1.84E-08
213151_s_at	SEPT7	2		N.S	-1.11	7.43E-09
213152_s_at	SFRS2B	0	1.2	3.99E-10	1.22	1.36E-09
213153_at	SETD1B	1		N.S		N.S
213154_s_at	BICD2	0		N.S	1.26	4.41E-19
213160_at	DOCK2	0		N.S		N.S
213161_at	C9orf97	0		N.S		N.S
213168_at	SP3	9		N.S		N.S
213170_at	GPX7	1		N.S		N.S
213172_at	TTC9	0	-1.5	5.85E-08		N.S
213188_s_at	MINA	2	-1.1	2.39E-07	-1.17	1.47E-07
213191_at	TICAM1	0		N.S		N.S
213194_at	ROBO1	0		N.S		N.S
213203_at	SNAPC5	0		N.S	1.21	6.13E-07
213206_at	GOSR2	0		N.S	1.15	5.49E-03
213213_at	DIDO1	1	-1.2	3.86E-03		N.S
213216_at	OTUD3	0		N.S		N.S
213224_s_at	NCRNA00081	0		N.S		N.S
213225_at	PPM1B	0	1.1	1.39E-02		N.S
213226_at	CCNA2	18		N.S	-1.18	6.77E-05
213227_at	PGRMC2	0		N.S		N.S
213237_at	C16orf88	0		N.S		N.S
213238_at	ATP10D	0	-1.5	1.70E-24	-1.16	5.59E-04
213239_at	PIBF1	0	-1.3	1.04E-20	-1.21	2.00E-09
213246_at	C14orf109	0		N.S		N.S
213251_at	SMARCA5	1		N.S		N.S
213253_at	SMC2	8		N.S		N.S
213254_at	TNRC6B	0		N.S		N.S
213256_at	MARCH3	0		N.S	-1.35	8.95E-08
213262_at	SACS	9		N.S		N.S
213266_at	TUBGCP4	0		N.S		N.S
213269_at	ZNF248	0	-1.8	1.61E-20		N.S
213272_s_at	TMEM159	0		N.S		N.S
213274_s_at	CTSB	0		N.S		N.S
213278_at	MTMR9	0	1.1	8.44E-03		N.S
213279_at	DHRS1	0		N.S		N.S
213282_at	APOOL	0		N.S		N.S
213283_s_at	SALL2	0	-1.2	1.52E-02		N.S
213287_s_at	KRT10	1	1.2	3.01E-04		N.S
213293_s_at	TRIM22	0		N.S	1.45	4.75E-21

213294_at	EIF2AK2	0		N.S		N.S
213297_at	RMND5B	0		N.S		N.S
213300_at	ATG2A	0		N.S		N.S
213302_at	PFAS	0		N.S	-1.22	6.62E-07
213304_at	FAM179B	0	-1.2	6.08E-07	1.15	4.77E-05
213305_s_at	PPP2R5C	3		N.S	-1.22	4.20E-09
213310_at	EIF2C2	1		N.S		N.S
213312_at	C6orf162	0	-1.2	2.07E-03		N.S
213318_s_at	BAT3	2		N.S		N.S
213320_at	PRMT3	0		N.S		N.S
213322_at	C6orf130	0		N.S	1.12	1.64E-05
213326_at	VAMP1	0		N.S		N.S
213327_s_at	USP12	1		N.S		N.S
213328_at	NEK1	4	-1.2	4.25E-02		N.S
213333_at	MDH2	0		N.S		N.S
213340_s_at	KIAA0495	0		N.S	1.10	3.20E-02
213341_at	FEM1C	0	1.3	3.97E-15		N.S
213346_at	C13orf27	0		N.S		N.S
213350_at	RPS11	0		N.S		N.S
213351_s_at	TMCC1	0	-1.5	3.70E-12		N.S
213353_at	ABCA5	0		N.S	1.25	1.14E-12
213357_at	GTF2H5	1		N.S		N.S
213361_at	TDRD7	0		N.S		N.S
213365_at	ERI2	0	-1.1	2.81E-02	1.29	4.26E-19
213370_s_at	SFMBT1	0		N.S	1.12	7.26E-03
213372_at	PAQR3	0		N.S		N.S
213373_s_at	CASP8	219	-1.2	8.80E-04		N.S
213376_at	ZBTB1	0		N.S		N.S
213379_at	COQ2	0		N.S		N.S
213387_at	ATAD2B	0		N.S		N.S
213390_at	ZC3H4	0		N.S	1.09	2.50E-02
213391_at	DPY19L4	0	-1.2	2.16E-03	1.29	5.39E-08
213398_s_at	SDR39U1	0		N.S		N.S
213402_at	ZNF787	0		N.S		N.S
213405_at	RAB22A	0		N.S		N.S
213408_s_at	PI4KA	0		N.S		N.S
213409_s_at	RHEB	5	1.3	4.86E-03		N.S
213410_at	C10orf137	0	-1.5	8.70E-19	-1.20	5.78E-05
213414_s_at	RPS19	1		N.S		N.S
213415_at	CLIC2	0		N.S		N.S
213420_at	DHX57	0		N.S		N.S
213427_at	RPP40	0	1.2	8.00E-07		N.S
213436_at	CNR1	0	-1.3	3.00E-04	1.46	2.81E-12
213445_at	ZC3H3	0		N.S		N.S
213446_s_at	IQGAP1	0		N.S		N.S
213447_at	IPW	1		N.S		N.S
213449_at	POP1	0	-1.3	1.14E-08		N.S
213452_at	ZNF184	0		N.S	1.14	2.17E-02
213454_at	APITD1	0		N.S	1.22	2.42E-07
213455_at	FAM114A1	0	-1.2	3.77E-07		N.S
213457_at	MFHAS1	0		N.S		N.S
213461_at	NUDT21	0		N.S		N.S
213469_at	PGAP1	0		N.S	2.05	3.08E-31
213474_at	KCTD7	0	-1.3	1.05E-05		N.S
213475_s_at	ITGAL	3		N.S	-1.23	7.89E-03
213483_at	PPWD1	0	-1.2	6.88E-13	1.11	4.45E-05
213485_s_at	ABCC10	1		N.S		N.S
213494_s_at	YY1	11		N.S		N.S
213497_at	ABTB2	0		N.S	1.16	6.97E-03
213508_at	C14orf147	0	-1.1	4.45E-06	1.18	2.34E-10

Supplemental Table 2

213517_at	PCBP2	0		N.S		N.S
213521_at	PTPN18	0		N.S		N.S
213523_at	CCNE1	6	1.1	1.48E-03		N.S
213526_s_at	LIN37	0	-1.2	2.24E-03	1.26	1.16E-06
213527_s_at	ZNF688	0		N.S		N.S
213528_at	C1orf156	0		N.S		N.S
213531_s_at	RAB3GAP1	0		N.S		N.S
213534_s_at	PASK	0		N.S	-1.08	1.47E-02
213540_at	HSD17B8	0		N.S		N.S
213546_at	DKFZP586I1420	0		N.S		N.S
213548_s_at	CDV3	0		N.S	-1.28	5.66E-03
213549_at	SLC18A2	0		N.S		N.S
213560_at	GADD45B	52	1.3	3.15E-04		N.S
213566_at	RNASE6	0		N.S	-1.61	5.36E-14
213568_at	OSR2	0		N.S	-1.17	1.94E-03
213577_at	SQLE	0		N.S	-1.41	4.68E-12
213587_s_at	ATP6V0E2	0		N.S		N.S
213590_at	LOC100133772	0		N.S		N.S
213593_s_at	TRA2A	0		N.S		N.S
213599_at	OIP5	0		N.S		N.S
213603_s_at	RAC2	0		N.S	-1.13	4.10E-04
213604_at	TCEB3	0	1.2	3.65E-07		N.S
213605_s_at	LOC100134401	0		N.S		N.S
213606_s_at	ARHGDIA	0		N.S		N.S
213608_s_at	SRRD	0		N.S		N.S
213618_at	ARAP2	0	-1.2	3.85E-03		N.S
213622_at	COL9A2	0		N.S		N.S
213623_at	KIF3A	1		N.S		N.S
213625_at	ZKSCAN4	0		N.S	1.29	6.42E-15
213626_at	CBR4	0	-1.2	1.78E-08		N.S
213627_at	MAGED2	1		N.S		N.S
213634_s_at	TRMU	0	-1.2	2.38E-02		N.S
213638_at	PHACTR1	0		N.S	-1.22	4.20E-04
213642_at	RPL27	0		N.S		N.S
213647_at	DNA2	19		N.S		N.S
213650_at	GOLGA8A	0		N.S		N.S
213654_at	TAF5L	0		N.S		N.S
213664_at	SLC1A1	1	-1.4	6.84E-24	-1.28	4.98E-14
213671_s_at	MARS	67		N.S		N.S
213677_s_at	PMS1	27	-1.5	1.28E-33	-1.13	6.25E-05
213679_at	TTC30A	0	-1.4	3.20E-10		N.S
213687_s_at	RPL35A	0		N.S		N.S
213694_at	RSBN1	0	-1.6	6.82E-21	-1.21	1.38E-08
213699_s_at	YWHAQ	0		N.S		N.S
213701_at	C12orf29	0		N.S		N.S
213703_at	LOC150759	0		N.S		N.S
213704_at	RABGGTB	0		N.S		N.S
213705_at	MAT2A	1		N.S		N.S
213738_s_at	ATP5A1	0		N.S		N.S
213742_at	SFRS11	0		N.S		N.S
213743_at	CCNT2	0		N.S		N.S
213746_s_at	FLNA	1		N.S		N.S
213748_at	TRIM66	0		N.S		N.S
213757_at	EIF5A	2		N.S		N.S
213761_at	MDM1	0	-1.2	7.62E-05	1.34	1.56E-12
213779_at	EMID1	0		N.S		N.S
213787_s_at	EBP	63		N.S		N.S
213792_s_at	INSR	1		N.S		N.S
213793_s_at	HOMER1	0		N.S	-1.41	4.26E-18
213794_s_at	NGDN	0	1.1	8.27E-08		N.S

213798_s_at	CAP1	1		N.S	-1.09	1.09E-05
213803_at	KPNB1	0	-1.1	5.07E-07	-1.33	2.77E-17
213804_at	INPP5B	0		N.S		N.S
213810_s_at	AKIRIN2	0		N.S		N.S
213812_s_at	CAMKK2	0		N.S		N.S
213826_s_at	LOC100133109	0		N.S		N.S
213846_at	COX7C	1		N.S		N.S
213850_s_at	SFRS2IP	0		N.S		N.S
213851_at	TMEM110	0		N.S		N.S
213853_at	DNAJC24	0		N.S		N.S
213861_s_at	FAM119B	0		N.S		N.S
213863_s_at	OAZ3	1		N.S		N.S
213872_at	C6orf62	0		N.S	1.17	3.81E-06
213878_at	PYROXD1	0		N.S		N.S
213879_at	SUMO2	5		N.S		N.S
213887_s_at	POLR2E	0		N.S		N.S
213888_s_at	LOC100133233	0	-1.3	1.05E-19	-1.50	9.27E-30
213891_s_at	TCF4	0	-1.2	6.71E-19	-1.35	1.15E-24
213897_s_at	MRPL23	0		N.S		N.S
213900_at	C9orf61	0		N.S		N.S
213906_at	MYBL1	0		N.S	1.46	1.53E-06
213908_at	WHAMML1	0		N.S	-1.83	1.84E-12
213916_at	ZNF20	0	-1.2	6.65E-15		N.S
213919_at	DNAJC4	0		N.S		N.S
213922_at	TTBK2	0		N.S		N.S
213923_at	RAP2B	0	1.2	2.12E-11	1.69	1.63E-36
213927_at	MAP3K9	0	-1.2	7.01E-04		N.S
213934_s_at	ZNF23	0		N.S		N.S
213937_s_at	FTSJ1	0		N.S		N.S
213940_s_at	FNBP1	0		N.S	-1.15	8.66E-17
213951_s_at	PSMC3IP	0	1.2	1.47E-10	1.42	4.52E-20
213954_at	FAM169A	0		N.S	2.16	4.10E-18
213959_s_at	RPGRIP1L	0	-1.4	9.55E-11		N.S
213963_s_at	SAP30	0		N.S		N.S
213970_at	RABL3	0		N.S		N.S
213974_at	ADAMTSL3	0		N.S		N.S
213979_s_at	CTBP1	1		N.S		N.S
213982_s_at	RABGAP1L	0		N.S	-1.32	2.36E-20
213999_at	YIPF4	0		N.S		N.S
214004_s_at	VGLL4	0	-1.3	2.39E-05	-1.30	4.14E-07
214006_s_at	GGCX	0		N.S		N.S
214007_s_at	TWF1	0		N.S		N.S
214011_s_at	NOP16	0		N.S	-1.21	1.19E-03
214022_s_at	IFITM1	0		N.S		N.S
214030_at	CRYBG3	0		N.S		N.S
214036_at	EFNA5	0		N.S		N.S
214039_s_at	LAPTM4B	0		N.S		N.S
214042_s_at	RPL22	0		N.S		N.S
214045_at	LIAS	0		N.S		N.S
214048_at	MBD4	21		N.S		N.S
214051_at	TMSB15B	0		N.S		N.S
214057_at	MCL1	15		N.S		N.S
214060_at	SSBP1	5		N.S	-1.20	1.20E-03
214061_at	WDR67	0	-1.2	4.42E-09	1.25	1.24E-09
214075_at	NENF	0		N.S		N.S
214079_at	DHRS2	0		N.S		N.S
214083_at	LOC100132532	0		N.S		N.S
214086_s_at	PARP2	11	-1.2	8.24E-16		N.S
214096_s_at	SHMT2	0		N.S		N.S
214101_s_at	NPEPPS	0		N.S		N.S

214106_s_at	GMDS	0		N.S	-1.16	2.69E-07
214112_s_at	CXorf40A	0		N.S	1.12	1.61E-04
214113_s_at	RBM8A	0		N.S		N.S
214123_s_at	C4orf10	0		N.S	1.32	8.71E-06
214126_at	MCART1	0		N.S		N.S
214132_at	ATP5C1	0		N.S		N.S
214144_at	POLR2D	0		N.S	1.17	1.34E-04
214148_at	FOXM1	7		N.S	1.20	5.63E-03
214152_at	CCPG1	0		N.S		N.S
214155_s_at	LARP4	0		N.S	1.18	1.99E-02
214167_s_at	RPLP0	0		N.S		N.S
214179_s_at	NFE2L1	0		N.S		N.S
214182_at	LOC100132430	0		N.S		N.S
214186_s_at	HCG26	0		N.S	1.28	8.10E-17
214202_at	PGGT1B	0		N.S		N.S
214218_s_at	XIST	3		N.S		N.S
214221_at	ALMS1	0	-1.2	3.85E-11		N.S
214224_s_at	PIN4	0		N.S		N.S
214231_s_at	KIAA0564	0		N.S		N.S
214264_s_at	C14orf143	0		N.S		N.S
214274_s_at	ACAA1	0		N.S		N.S
214281_s_at	RCHY1	3		N.S		N.S
214290_s_at	HIST2H2AA3	0		N.S		N.S
214291_at	LOC729046	0		N.S		N.S
214305_s_at	SF3B1	1		N.S		N.S
214313_s_at	EIF5B	0		N.S		N.S
214328_s_at	HSP90AA1	0		N.S		N.S
214339_s_at	MAP4K1	0		N.S	-1.24	1.17E-07
214352_s_at	KRAS	13		N.S		N.S
214356_s_at	KIAA0368	0		N.S		N.S
214364_at	MTERFD2	0		N.S	1.17	8.01E-05
214366_s_at	ALOX5	5		N.S	1.31	2.92E-02
214377_s_at	CTRL	2		N.S		N.S
214427_at	NOP2	0		N.S		N.S
214429_at	MTMR6	1		N.S		N.S
214430_at	GLA	23		N.S		N.S
214431_at	GMPS	0		N.S		N.S
214440_at	NAT1	20		N.S	1.12	4.32E-08
214441_at	STX6	1		N.S	1.50	1.28E-23
214446_at	ELL2	0		N.S	1.35	1.13E-12
214447_at	ETS1	7		N.S		N.S
214452_at	BCAT1	0		N.S	-1.26	2.23E-07
214453_s_at	IFI44	2		N.S		N.S
214455_at	HIST1H2BC	0	1.3	4.15E-05	1.37	1.51E-03
214467_at	GPR65	0	-1.4	4.82E-09	-1.41	2.64E-15
214482_at	ZBTB25	0	-1.2	1.11E-04		N.S
214484_s_at	SIGMAR1	0		N.S	-1.16	4.47E-02
214507_s_at	EXOSC2	0		N.S	-1.12	5.56E-03
214513_s_at	CREB1	5	-1.2	1.60E-06		N.S
214519_s_at	RLN2	0		N.S		N.S
214527_s_at	PQBP1	0		N.S		N.S
214529_at	TSHB	0		N.S		N.S
214569_at	IFNA5	0		N.S		N.S
214583_at	RSC1A1	0		N.S		N.S
214585_s_at	VPS52	1		N.S		N.S
214597_at	SSTR2	0		N.S		N.S
214599_at	IVL	0		N.S		N.S
214614_at	MNX1	0	-1.2	7.02E-04		N.S
214615_at	P2RY10	0		N.S		N.S
214617_at	PRF1	1		N.S	1.46	1.31E-10

214626_s_at	GANAB	0		N.S		N.S
214657_s_at	NCRNA00084	0		N.S		N.S
214658_at	TMED7	1		N.S		N.S
214661_s_at	NOP14	0		N.S		N.S
214662_at	WDR43	0	1.2	9.77E-07		N.S
214668_at	C13orf1	0		N.S		N.S
214670_at	ZKSCAN1	0		N.S		N.S
214672_at	TTLL5	0		N.S	-1.16	1.05E-03
214681_at	GK	12		N.S		N.S
214683_s_at	CLK1	0	1.6	3.75E-15	1.50	9.72E-16
214686_at	ZNF266	0		N.S		N.S
214688_at	TLE4	0		N.S		N.S
214690_at	TAF1B	1		N.S		N.S
214696_at	C17orf91	0	1.5	9.04E-19	1.26	1.25E-05
214697_s_at	ROD1	0		N.S	1.39	5.49E-16
214703_s_at	MAN2B2	0		N.S	1.14	3.60E-02
214709_s_at	KTN1	1		N.S	-1.17	1.72E-11
214710_s_at	CCNB1	227	-1.2	4.59E-08	-2.72	2.69E-42
214711_at	GATC	9	-1.2	2.24E-05		N.S
214714_at	ZNF394	0		N.S	-1.15	1.03E-03
214717_at	DKFZp434H1419	0		N.S		N.S
214719_at	SLC46A3	0	-1.2	1.54E-06	-1.18	4.22E-04
214722_at	NOTCH2NL	0	1.2	3.22E-03	1.36	8.42E-11
214729_at	TWISTNB	0		N.S		N.S
214731_at	CTTNBP2NL	0		N.S		N.S
214735_at	IPCEF1	0	-1.2	4.90E-02		N.S
214736_s_at	ADD1	0		N.S		N.S
214739_at	LRCH3	0	-1.3	1.68E-13		N.S
214741_at	ZNF131	0		N.S	1.25	1.07E-07
214742_at	AZI1	0		N.S		N.S
214744_s_at	RPL23	0	1.3	1.31E-15	1.40	5.73E-20
214748_at	N4BP2L2	0	-1.2	7.36E-03	1.29	3.32E-07
214749_s_at	ARMCX6	0		N.S		N.S
214751_at	ZNF468	0	1.3	4.74E-10	1.30	9.95E-12
214759_at	WTAP	1		N.S		N.S
214762_at	ATP6V1G2	0		N.S		N.S
214764_at	RRP15	0	1.3	1.34E-03		N.S
214766_s_at	AHCTF1	0		N.S	-1.13	5.34E-03
214772_at	C11orf41	0		N.S	1.38	4.41E-08
214775_at	N4BP3	0		N.S		N.S
214779_s_at	SGSM3	0		N.S		N.S
214780_s_at	MYO9B	0		N.S		N.S
214785_at	VPS13A	0		N.S		N.S
214787_at	DENND4A	0		N.S		N.S
214790_at	SENP6	2	1.8	2.17E-24	1.27	7.73E-05
214791_at	SP140L	0		N.S		N.S
214801_at	IFRG15	0		N.S		N.S
214804_at	CENPI	0	-1.4	3.97E-14	-1.22	3.06E-04
214813_at	ZNF75D	0	-1.2	7.09E-06		N.S
214814_at	YTHDC1	0		N.S		N.S
214830_at	SLC38A6	0		N.S		N.S
214838_at	SFT2D2	0		N.S		N.S
214843_s_at	USP33	0		N.S		N.S
214844_s_at	DOK5	0		N.S		N.S
214848_at	YWHAZ	0		N.S		N.S
214850_at	LOC100170939	0		N.S		N.S
214857_at	C10orf95	0	-1.2	1.51E-03	-1.21	2.54E-05
214864_s_at	GRHPR	0		N.S		N.S
214876_s_at	TUBGCP5	0	1.2	3.62E-03		N.S
214878_at	ZNF37A	0		N.S	1.31	1.42E-02

214882_s_at	SFRS2	2	1.1	6.44E-16		N.S
214895_s_at	ADAM10	1		N.S		N.S
214918_at	HNRNPM	0		N.S		N.S
214919_s_at	ANKHD1-EIF4EBP3	0	-1.2	1.28E-06		N.S
214923_at	ATP6V1D	0		N.S		N.S
214931_s_at	SRPK2	0		N.S		N.S
214941_s_at	PRPF40A	0		N.S		N.S
214943_s_at	RBM34	0		N.S	1.18	4.50E-02
214948_s_at	TMF1	0		N.S		N.S
214949_at	hCG_1795560	0		N.S		N.S
214953_s_at	APP	39		N.S		N.S
214962_s_at	NUP160	0	1.7	1.92E-25	1.33	1.26E-09
214975_s_at	MTMR1	0		N.S		N.S
214984_at	LOC440345	0		N.S		N.S
214992_s_at	DNASE2	1		N.S		N.S
214997_at	C9orf126	0		N.S		N.S
215001_s_at	GLUL	0		N.S		N.S
215011_at	SNHG3	0	-1.3	1.10E-08	-1.22	1.43E-06
215023_s_at	PEX1	0		N.S		N.S
215029_at	C1orf108	0		N.S		N.S
215030_at	GRSF1	0	-1.1	5.81E-03	-1.17	2.22E-05
215068_s_at	FBXL18	0		N.S		N.S
215071_s_at	HIST1H2AC	1		N.S		N.S
215075_s_at	GRB2	8		N.S	-1.10	1.12E-04
215084_s_at	LRRC42	0		N.S		N.S
215087_at	C15orf39	0		N.S	-1.34	4.23E-15
215088_s_at	SDHC	2		N.S		N.S
215089_s_at	RBM10	0		N.S		N.S
215091_s_at	GTF3A	0		N.S		N.S
215096_s_at	ESD	3		N.S		N.S
215100_at	C6orf105	0		N.S		N.S
215109_at	KIAA0492	0		N.S		N.S
215111_s_at	TSC22D1	1	-1.3	4.92E-07		N.S
215123_at	LOC642778	0		N.S		N.S
215127_s_at	RBMS1	1		N.S		N.S
215134_at	PI4K2A	0		N.S	1.18	1.97E-06
215136_s_at	EXOSC8	0		N.S		N.S
215143_at	DPY19L2P2	0		N.S		N.S
215148_s_at	APBA3	0		N.S		N.S
215150_at	YOD1	0		N.S		N.S
215170_s_at	CEP152	0		N.S		N.S
215190_at	EIF3M	0		N.S		N.S
215191_at	FBXL11	0		N.S		N.S
215210_s_at	DLST	0		N.S		N.S
215218_s_at	WDR62	0		N.S		N.S
215223_s_at	SOD2	49		N.S		N.S
215228_at	NHLH2	0		N.S	1.77	5.47E-13
215241_at	ANO3	1		N.S	2.10	8.78E-15
215263_at	ZXDA	0		N.S		N.S
215285_s_at	PHTF1	0	1.1	2.20E-02	1.37	1.53E-19
215307_at	ZNF529	0		N.S		N.S
215338_s_at	NKTR	0	-1.4	1.05E-12	1.15	8.09E-04
215343_at	CCDC88C	0	-1.1	6.15E-03	1.28	1.39E-12
215366_at	SNX13	0		N.S		N.S
215380_s_at	GGCT	0		N.S	1.15	5.97E-06
215385_at	FTO	3		N.S		N.S
215399_s_at	OS9	0		N.S		N.S
215416_s_at	STOML2	0		N.S		N.S
215424_s_at	SNW1	1		N.S		N.S
215425_at	BTG3	3		N.S		N.S

215440_s_at	BEX4	0		N.S		N.S
215463_at	OR7E24	0		N.S		N.S
215465_at	ABCA12	0		N.S	1.77	2.68E-13
215470_at	GTF2H2B	0		N.S		N.S
215482_s_at	EIF2B4	0		N.S		N.S
215499_at	MAP2K3	2		N.S	1.25	1.14E-15
215501_s_at	DUSP10	2		N.S	-1.38	1.10E-16
215509_s_at	BUB1	28		N.S	-1.77	6.27E-19
215521_at	PHC3	0		N.S		N.S
215536_at	HLA-DQB2	0		N.S		N.S
215548_s_at	SCFD1	0		N.S		N.S
215577_at	UBE2E1	0		N.S		N.S
215596_s_at	RNF160	0	-1.2	8.65E-06		N.S
215602_at	FGD2	0		N.S		N.S
215631_s_at	BRMS1	0		N.S	1.13	1.14E-04
215684_s_at	ASCC2	0		N.S		N.S
215694_at	SPATA5L1	0		N.S		N.S
215696_s_at	SEC16A	0		N.S		N.S
215707_s_at	PRNP	3		N.S		N.S
215718_s_at	PHF3	0		N.S		N.S
215728_s_at	ACOT7	0		N.S	1.12	4.82E-03
215734_at	C19orf36	0		N.S		N.S
215739_s_at	TUBGCP3	0		N.S		N.S
215743_at	NMT2	1		N.S	1.31	4.35E-05
215747_s_at	RCC1	2		N.S		N.S
215749_s_at	GORASP1	0		N.S		N.S
215767_at	ZNF804A	0	-1.5	4.33E-14	-1.25	1.12E-04
215780_s_at	hCG_1644608	0		N.S		N.S
215785_s_at	CYFIP2	0		N.S	1.19	2.51E-02
215792_s_at	DNAJC11	0		N.S		N.S
215854_at	FBXO22	0	1.4	2.89E-12	1.40	7.89E-11
215884_s_at	UBQLN2	0	-1.1	2.52E-05		N.S
215905_s_at	SNRNP40	0		N.S		N.S
215919_s_at	MRPS11	0		N.S		N.S
215930_s_at	CTAGE5	0		N.S		N.S
215942_s_at	GTSE1	4	-1.2	2.58E-05	-1.30	6.44E-07
215947_s_at	FAM136A	0		N.S		N.S
215954_s_at	C19orf29	0		N.S		N.S
215980_s_at	IGHMBP2	0		N.S		N.S
215983_s_at	UBXN8	0		N.S		N.S
215984_s_at	ARFRP1	0	-1.3	6.83E-10		N.S
215985_at	NCRNA00171	0	-1.3	1.13E-07		N.S
216006_at	RAPGEFL1	0		N.S		N.S
216020_at	IFIH1	0	1.4	1.08E-17		N.S
216026_s_at	POLE	28		N.S		N.S
216028_at	DKFZP564C152	0		N.S		N.S
216032_s_at	ERGIC3	0		N.S	1.10	3.37E-03
216060_s_at	DAAM1	0	-1.2	7.25E-03		N.S
216088_s_at	PSMA7	1		N.S		N.S
216114_at	NCKIPSD	0		N.S		N.S
216147_at	SEPT11	0		N.S		N.S
216177_at	LOC391132	0		N.S		N.S
216194_s_at	TBCB	0		N.S		N.S
216199_s_at	MAP3K4	3	-1.2	1.02E-06		N.S
216218_s_at	PLCL2	0		N.S	1.42	1.22E-12
216226_at	TAF4B	0		N.S	-1.20	3.96E-04
216231_s_at	B2M	2		N.S		N.S
216232_s_at	GCN1L1	0		N.S	-1.17	2.37E-06
216241_s_at	TCEA1	0		N.S		N.S
216247_at	RPS20	0		N.S	1.24	2.05E-05

216248_s_at	NR4A2	5		N.S	-1.52	9.92E-05
216250_s_at	LPXN	0		N.S	1.13	2.43E-13
216251_s_at	TTLL12	0		N.S	-1.26	4.41E-09
216261_at	ITGB3	0		N.S		N.S
216262_s_at	TGIF2	0		N.S		N.S
216266_s_at	ARFGEF1	0		N.S	1.19	5.73E-05
216267_s_at	TMEM115	0		N.S	1.19	7.89E-03
216305_s_at	C2orf3	0		N.S		N.S
216326_s_at	HDAC3	5		N.S		N.S
216338_s_at	YIPF3	0		N.S		N.S
216348_at	RPS17P5	0		N.S		N.S
216383_at	RPL18A	0		N.S		N.S
216396_s_at	EI24	8	1.3	1.18E-23	1.93	1.34E-48
216397_s_at	BOP1	1		N.S		N.S
216409_at	ACSL6	0		N.S		N.S
216411_s_at	GALK2	0		N.S		N.S
216437_at	EPC1	1		N.S		N.S
216438_s_at	TMSB4X	0		N.S		N.S
216520_s_at	TPT1	0		N.S		N.S
216521_s_at	BRCC3	4		N.S		N.S
216547_at	LOC127406	0		N.S		N.S
216563_at	ANKRD12	0		N.S		N.S
216574_s_at	hCG_2024410	0		N.S		N.S
216591_s_at	hCG_1776980	0		N.S		N.S
216602_s_at	FARSA	0		N.S		N.S
216607_s_at	CYP51A1	0		N.S	-1.40	7.30E-11
216609_at	TXN	4		N.S		N.S
216640_s_at	PDIA6	0		N.S		N.S
216650_at	LOC283412	0		N.S		N.S
216678_at	IFT122	0		N.S		N.S
216699_s_at	KLK1	0		N.S		N.S
216705_s_at	ADA	78		N.S	1.20	9.63E-03
216783_at	LOC283677	0		N.S		N.S
216806_at	RPSA	0		N.S		N.S
216834_at	RGS1	1		N.S	-1.39	4.67E-13
216835_s_at	DOK1	1	-1.2	8.52E-06	-1.14	8.19E-03
216862_s_at	MTCP1NB	0		N.S		N.S
216863_s_at	MORC2	0	-1.3	1.04E-09	-1.17	2.21E-03
216899_s_at	SKAP2	0		N.S		N.S
216902_s_at	LOC653390	0		N.S		N.S
216903_s_at	CBARA1	0		N.S		N.S
216942_s_at	CD58	0		N.S		N.S
216944_s_at	ITPR1	3		N.S	-1.37	1.42E-13
216952_s_at	LMNB2	0		N.S		N.S
216961_s_at	RPAIN	0		N.S		N.S
216969_s_at	KIF22	0		N.S		N.S
216993_s_at	COL11A2	8		N.S		N.S
216996_s_at	FASTKD2	0		N.S		N.S
217019_at	RPS4X	0		N.S		N.S
217028_at	CXCR4	5	1.3	1.49E-04		N.S
217042_at	RDH11	0	-1.2	3.17E-02	-1.18	4.09E-02
217043_s_at	MFN1	1		N.S	-1.18	2.06E-05
217047_s_at	FAM13A	0	-1.4	3.25E-11	-1.33	2.17E-06
217094_s_at	ITCH	9		N.S		N.S
217100_s_at	UBXN7	0	-1.2	1.69E-08		N.S
217104_at	ST20	0		N.S		N.S
217118_s_at	C22orf9	0		N.S	-1.24	5.73E-17
217122_s_at	RP11-345P4.4	0	-1.3	3.57E-12		N.S
217125_at	UBBP2	0		N.S		N.S
217127_at	CTH	1		N.S	1.23	1.19E-02

217139_at	LOC100133724	0		N.S		N.S
217144_at	LOC648390	0		N.S		N.S
217168_s_at	HERPUD1	0		N.S	-1.18	3.29E-12
217176_s_at	ZFX	0		N.S		N.S
217188_s_at	C14orf1	0		N.S		N.S
217211_at	ACTBP9	0		N.S		N.S
217266_at	RPL15P22	0		N.S		N.S
217286_s_at	NDRG3	0		N.S	-1.31	3.84E-16
217289_s_at	SLC37A4	0		N.S		N.S
217299_s_at	NBN	271	-1.1	1.37E-06	-1.14	1.32E-04
217310_s_at	FOXJ3	0		N.S		N.S
217317_s_at	HERC2P2	0	-1.6	8.77E-19		N.S
217336_at	RPS10	0		N.S		N.S
217340_at	RPL21P68	0		N.S		N.S
217346_at	LOC128192	0		N.S		N.S
217365_at	PRAMEF11	0		N.S		N.S
217368_at	ATP5G2	0		N.S		N.S
217379_at	RPL10	0		N.S		N.S
217383_at	PGK1	18		N.S		N.S
217388_s_at	KYNU	0	-1.1	8.94E-04	-1.22	1.88E-25
217403_s_at	ZNF227	0	1.2	2.77E-04	1.21	1.40E-03
217408_at	MRPS18B	0		N.S		N.S
217427_s_at	HIRA	1		N.S		N.S
217445_s_at	GART	2		N.S	-1.21	7.41E-03
217448_s_at	LOC285412	0		N.S		N.S
217457_s_at	RAP1GDS1	0		N.S	-1.18	2.10E-04
217465_at	NCKAP1	0		N.S		N.S
217477_at	PIP5K1B	0		N.S		N.S
217478_s_at	HLA-DMA	0		N.S		N.S
217494_s_at	PTENP1	0		N.S		N.S
217496_s_at	IDE	81		N.S		N.S
217501_at	CIAO1	0		N.S		N.S
217503_at	STK17B	0	-1.2	4.61E-08	-1.25	2.38E-09
217504_at	ABCA6	0		N.S		N.S
217506_at	LOC339290	0		N.S		N.S
217523_at	CD44	35	1.3	6.14E-03		N.S
217527_s_at	NFATC2IP	0	1.1	2.60E-02		N.S
217540_at	FAM55C	0		N.S		N.S
217544_at	LOC729806	0		N.S		N.S
217549_at	NCKAP1L	0		N.S		N.S
217559_at	RPL10L	0		N.S		N.S
217599_s_at	MDFIC	0	-1.2	8.96E-06		N.S
217602_at	PPIA	0		N.S		N.S
217608_at	SFRS12IP1	0		N.S		N.S
217627_at	ZNF573	0	-1.4	5.47E-14		N.S
217645_at	COX16	0		N.S		N.S
217663_at	ZNF234	0		N.S		N.S
217677_at	PLEKHA2	0		N.S	-1.33	4.82E-08
217682_at	C16orf72	0	1.5	1.32E-19		N.S
217692_at	MAGOH2	0		N.S		N.S
217716_s_at	SEC61A1	0		N.S	1.53	2.53E-19
217718_s_at	YWHAB	0		N.S		N.S
217719_at	EIF3L	0		N.S		N.S
217720_at	CHCHD2	0		N.S		N.S
217722_s_at	NGRN	0		N.S		N.S
217726_at	COPZ1	0		N.S		N.S
217728_at	S100A6	4		N.S		N.S
217729_s_at	AES	19		N.S		N.S
217730_at	TMBIM1	0		N.S	1.46	1.53E-15
217731_s_at	ITM2B	0		N.S	1.64	1.41E-14

217733_s_at	TMSB10	0		N.S		N.S
217734_s_at	WDR6	0		N.S		N.S
217736_s_at	EIF2AK1	33		N.S		N.S
217739_s_at	NAMPT	2	1.1	3.67E-05		N.S
217742_s_at	WAC	1		N.S		N.S
217743_s_at	TMEM30A	0		N.S	1.43	2.69E-24
217744_s_at	PERP	7		N.S	1.30	4.48E-06
217745_s_at	NAT13	0		N.S		N.S
217746_s_at	PDCD6IP	1		N.S		N.S
217747_s_at	RPS9	1		N.S		N.S
217748_at	ADIPOR1	0		N.S	1.12	6.24E-06
217749_at	COPG	0		N.S		N.S
217750_s_at	UBE2Z	0		N.S		N.S
217751_at	GSTK1	0		N.S		N.S
217752_s_at	CNDP2	0		N.S		N.S
217753_s_at	RPS26	0		N.S		N.S
217754_at	DDX56	0		N.S		N.S
217755_at	HN1	0		N.S	-1.18	2.41E-04
217758_s_at	TM9SF3	0		N.S		N.S
217759_at	TRIM44	0		N.S		N.S
217761_at	ADI1	0	1.1	1.19E-05	1.26	1.38E-18
217763_s_at	RAB31	0		N.S	-1.29	5.59E-03
217765_at	NRBP1	0		N.S		N.S
217766_s_at	TMEM50A	0		N.S		N.S
217768_at	C14orf166	0		N.S		N.S
217769_s_at	POMP	3		N.S		N.S
217772_s_at	MTCH2	0		N.S		N.S
217773_s_at	NDUFA4	0		N.S		N.S
217774_s_at	HSPC152	0		N.S		N.S
217777_s_at	PTPLAD1	0		N.S		N.S
217778_at	SLC39A1	0		N.S		N.S
217779_s_at	LOC100132235	0		N.S		N.S
217780_at	C19orf56	0		N.S		N.S
217781_s_at	ZFP106	0		N.S		N.S
217782_s_at	GPS1	0		N.S		N.S
217783_s_at	YPEL5	0	1.1	3.04E-05		N.S
217786_at	PRMT5	5		N.S		N.S
217788_s_at	GALNT2	0		N.S		N.S
217789_at	SNX6	0		N.S		N.S
217790_s_at	SSR3	0		N.S		N.S
217791_s_at	ALDH18A1	0		N.S		N.S
217792_at	SNX5	0	-1.2	5.31E-11		N.S
217794_at	PRR13	0		N.S		N.S
217795_s_at	TMEM43	0		N.S		N.S
217796_s_at	NPLOC4	0		N.S		N.S
217797_at	UFC1	0		N.S		N.S
217800_s_at	NDFIP1	0		N.S	1.22	2.60E-04
217801_at	ATP5E	0		N.S		N.S
217803_at	GOLPH3	0		N.S		N.S
217805_at	ILF3	0		N.S		N.S
217806_s_at	POLDIP2	0		N.S		N.S
217807_s_at	GLTSCR2	0		N.S	1.11	3.79E-05
217808_s_at	MAPKAP1	0		N.S		N.S
217809_at	BZW2	0		N.S		N.S
217811_at	SELT	0		N.S		N.S
217812_at	YTHDF2	0	1.1	5.81E-03		N.S
217813_s_at	SPIN1	0		N.S		N.S
217814_at	CCDC47	0		N.S		N.S
217815_at	SUPT16H	4		N.S		N.S
217816_s_at	PCNP	1		N.S	1.19	1.20E-17

Supplemental Table 2

217819_at	GOLGA7	0		N.S	1.07	2.90E-03
217822_at	WBP11	0		N.S		N.S
217826_s_at	UBE2J1	0	-1.1	3.25E-02	-1.34	4.63E-14
217827_s_at	SPG21	0		N.S	-1.16	1.02E-12
217828_at	SLTM	0		N.S		N.S
217829_s_at	USP39	0		N.S		N.S
217830_s_at	NSFL1C	0	1.2	5.08E-05		N.S
217833_at	SYNCRIP	0	1.3	5.02E-15	-1.17	4.25E-05
217836_s_at	YY1AP1	2	1.1	2.06E-05		N.S
217837_s_at	VPS24	0	1.3	2.84E-16	1.57	3.27E-39
217838_s_at	EVL	1		N.S	-1.27	1.79E-11
217840_at	DDX41	0		N.S	-1.10	3.49E-02
217841_s_at	PPME1	0	-1.4	2.76E-13	-1.21	2.79E-03
217843_s_at	MED4	3		N.S	1.28	5.03E-13
217846_at	QARS	0		N.S		N.S
217848_s_at	PPA1	0		N.S		N.S
217850_at	GNL3	1	1.1	4.46E-09	-1.17	7.83E-11
217851_s_at	SLMO2	0		N.S		N.S
217852_s_at	ARL8B	0	1.1	1.07E-09	1.18	2.52E-11
217853_at	TNS3	0		N.S		N.S
217858_s_at	ARMCX3	0	-1.3	6.65E-15		N.S
217860_at	LOC732160	0		N.S		N.S
217861_s_at	PREB	0		N.S	1.31	6.59E-18
217862_at	PIAS1	3		N.S	-1.13	7.64E-04
217866_at	CPSF7	0	1.1	2.05E-02		N.S
217868_s_at	METTL9	0		N.S		N.S
217869_at	HSD17B12	0		N.S	1.13	5.77E-11
217870_s_at	CMPK1	0		N.S		N.S
217871_s_at	MIF	10		N.S		N.S
217872_at	PIH1D1	0		N.S		N.S
217873_at	CAB39	0		N.S		N.S
217874_at	SUCLG1	0		N.S		N.S
217877_s_at	GPBP1L1	0		N.S		N.S
217878_s_at	CDC27	2		N.S		N.S
217882_at	TMEM111	0	1.2	3.84E-07	1.13	4.11E-06
217883_at	MMADHC	0	1.1	8.78E-03		N.S
217884_at	NAT10	2		N.S	-1.14	2.35E-04
217885_at	IPO9	0		N.S		N.S
217886_at	EPS15	0	-1.1	1.41E-02		N.S
217888_s_at	ARFGAP1	0		N.S		N.S
217892_s_at	LIMA1	0	-1.1	1.48E-06		N.S
217893_s_at	AKIRIN1	0		N.S		N.S
217894_at	KCTD3	0		N.S		N.S
217895_at	PTCD3	0	-1.1	4.44E-05	-1.14	1.19E-06
217896_s_at	NIP30	0		N.S		N.S
217898_at	C15orf24	0		N.S		N.S
217899_at	TMEM214	0		N.S		N.S
217900_at	IARS2	0		N.S		N.S
217901_at	DSG2	0	-1.1	2.68E-02	1.11	3.11E-04
217902_s_at	HERC2	3	-1.1	3.49E-04		N.S
217903_at	STRN4	0		N.S		N.S
217905_at	C10orf119	1	1.1	9.08E-04		N.S
217906_at	KLHDC2	0	1.1	1.93E-05	1.29	3.47E-30
217907_at	MRPL18	0		N.S	1.16	4.48E-04
217908_s_at	IQWD1	0		N.S		N.S
217911_s_at	BAG3	0		N.S		N.S
217912_at	DUS1L	0		N.S		N.S
217913_at	VPS4A	0		N.S		N.S
217914_at	TPCN1	0		N.S	-1.21	2.31E-02
217915_s_at	RSL24D1	0	1.1	3.33E-02		N.S

Supplemental Table 2

217918_at	DYNLRB1	0		N.S		N.S
217919_s_at	MRPL42	0		N.S		N.S
217923_at	PEF1	0		N.S		N.S
217924_at	C6orf106	0		N.S		N.S
217926_at	C19orf53	0		N.S		N.S
217927_at	SPCS1	0		N.S		N.S
217928_s_at	SAPS3	0	1.1	5.06E-03		N.S
217930_s_at	TOLLIP	0		N.S	1.20	1.33E-07
217931_at	CNPY3	0		N.S	-1.31	3.20E-02
217932_at	MRPS7	0		N.S		N.S
217933_s_at	LAP3	0		N.S		N.S
217935_s_at	UQCC	0	-1.2	2.57E-10	1.23	1.59E-15
217936_at	ARHGAP5	0		N.S		N.S
217938_s_at	KCMF1	0		N.S	-1.06	3.68E-02
217939_s_at	AFTPH	0	-1.2	3.55E-08		N.S
217940_s_at	CARKD	0		N.S		N.S
217941_s_at	ERBB2IP	0		N.S		N.S
217942_at	MRPS35	0		N.S		N.S
217943_s_at	MAP7D1	0		N.S		N.S
217944_at	POMGNT1	0		N.S		N.S
217945_at	BTBD1	0		N.S		N.S
217946_s_at	SAE1	3		N.S		N.S
217947_at	CMTM6	0	1.1	1.35E-07	1.23	2.15E-21
217949_s_at	VKORC1	0		N.S		N.S
217950_at	NOSIP	0		N.S		N.S
217955_at	BCL2L13	0		N.S		N.S
217956_s_at	ENOPH1	0		N.S	-1.19	3.76E-18
217957_at	C16orf80	0		N.S		N.S
217959_s_at	TRAPPC4	0		N.S		N.S
217960_s_at	TOMM22	0		N.S		N.S
217961_at	SLC25A38	0		N.S		N.S
217962_at	NOP10	0		N.S		N.S
217963_s_at	NGFRAP1	0		N.S		N.S
217964_at	TTC19	0		N.S		N.S
217965_s_at	SAP30BP	0		N.S		N.S
217966_s_at	FAM129A	1		N.S	1.40	5.02E-18
217968_at	TSSC1	0		N.S		N.S
217969_at	C11orf2	0		N.S		N.S
217970_s_at	CNOT6	1		N.S	1.14	6.14E-04
217971_at	MAPKSP1	0		N.S	1.12	8.33E-05
217972_at	CHCHD3	0		N.S	-1.11	1.53E-07
217973_at	DCXR	0		N.S		N.S
217974_at	TM7SF3	0		N.S	2.07	2.25E-33
217975_at	WBP5	0		N.S		N.S
217976_s_at	DYNC1LI1	0		N.S		N.S
217977_at	SEPX1	0	-1.1	5.30E-04	-1.32	1.11E-07
217978_s_at	UBE2Q1	0		N.S	1.11	2.12E-05
217980_s_at	MRPL16	0		N.S		N.S
217981_s_at	FXC1	0		N.S		N.S
217982_s_at	MORF4L1	3		N.S		N.S
217984_at	RNASET2	0		N.S	-1.84	1.23E-27
217986_s_at	BAZ1A	0	-1.2	7.35E-05		N.S
217987_at	ASNSD1	0	1.2	6.43E-20		N.S
217988_at	CCNB1IP1	0		N.S	1.23	1.12E-24
217989_at	HSD17B11	0		N.S	-1.22	1.33E-07
217990_at	GMPR2	0		N.S		N.S
217992_s_at	EFHD2	0		N.S		N.S
217993_s_at	MAT2B	0	-1.1	7.08E-03		N.S
217995_at	SQRDL	0		N.S		N.S
217997_at	PHLDA1	0		N.S		N.S

218001_at	MRPS2	0	1.1	2.01E-02	-1.11	4.98E-02
218003_s_at	FKBP3	1		N.S		N.S
218005_at	ZNF22	0		N.S	-1.13	2.19E-11
218007_s_at	RPS27L	2	1.1	5.00E-02	1.41	4.36E-12
218008_at	C7orf42	0		N.S		N.S
218009_s_at	PRC1	3	-1.3	8.06E-15	-1.48	8.04E-21
218011_at	UBL5	0		N.S		N.S
218014_at	NUP85	0		N.S		N.S
218016_s_at	POLR3E	0	-1.2	2.17E-09	-1.16	1.95E-06
218017_s_at	HGSNAT	0		N.S		N.S
218018_at	PDXK	1		N.S		N.S
218020_s_at	ZFAND3	0		N.S	1.12	5.74E-03
218021_at	DHRS4	0		N.S		N.S
218022_at	VRK3	0		N.S		N.S
218023_s_at	FAM53C	0	1.2	1.72E-11		N.S
218024_at	BRP44L	0		N.S		N.S
218025_s_at	PECI	0		N.S		N.S
218026_at	CCDC56	0		N.S		N.S
218027_at	MRPL15	1		N.S		N.S
218032_at	SNN	0		N.S		N.S
218034_at	FIS1	1		N.S		N.S
218035_s_at	RBM47	0	-1.1	1.39E-02		N.S
218039_at	NUSAP1	0		N.S		N.S
218040_at	PRPF38B	0	-1.2	7.69E-04		N.S
218042_at	COPS4	0		N.S		N.S
218043_s_at	AZI2	0		N.S		N.S
218046_s_at	MRPS16	0		N.S		N.S
218047_at	OSBPL9	0		N.S		N.S
218048_at	COMMD3	0		N.S		N.S
218049_s_at	MRPL13	0		N.S		N.S
218050_at	UFM1	0	1.2	1.19E-11	1.21	1.62E-09
218051_s_at	NT5DC2	0		N.S		N.S
218052_s_at	ATP13A1	0		N.S		N.S
218055_s_at	WDR41	0		N.S		N.S
218056_at	BFAR	1	-1.2	5.17E-11	1.16	5.65E-09
218058_at	CXXC1	1		N.S	-1.33	2.38E-12
218059_at	ZNF706	0		N.S		N.S
218061_at	MEA1	0		N.S		N.S
218065_s_at	TMEM9B	0		N.S	1.18	4.70E-12
218066_at	SLC12A7	0		N.S		N.S
218067_s_at	ARGLU1	0		N.S		N.S
218068_s_at	ZNF672	0		N.S	1.12	3.53E-02
218069_at	DCTPP1	0		N.S		N.S
218070_s_at	GMPPA	0		N.S		N.S
218071_s_at	MKRN2	0	1.1	2.30E-04		N.S
218072_at	COMMD9	0		N.S	1.14	2.10E-02
218073_s_at	TMEM48	0		N.S		N.S
218074_at	FAM96B	0		N.S		N.S
218075_at	AAAS	1		N.S		N.S
218076_s_at	ARHGAP17	0		N.S	-1.30	7.99E-15
218077_s_at	ZDHHC3	0		N.S		N.S
218079_s_at	GGNBP2	1	1.3	1.88E-20		N.S
218082_s_at	UBP1	0		N.S		N.S
218083_at	PTGES2	0		N.S	-1.13	3.00E-02
218085_at	CHMP5	0		N.S		N.S
218088_s_at	RRAGC	0		N.S		N.S
218089_at	C20orf4	0	1.1	6.80E-05		N.S
218090_s_at	BRWD2	0		N.S		N.S
218093_s_at	ANKRD10	0	-1.1	8.12E-06		N.S
218095_s_at	TMEM165	0	-1.2	2.43E-13		N.S

218096_at	AGPAT5	0		N.S	-1.21	1.39E-15
218097_s_at	CUEDC2	0		N.S		N.S
218099_at	TEX2	0		N.S		N.S
218100_s_at	IFT57	0		N.S	-1.21	1.89E-10
218101_s_at	NDUFC2	1		N.S		N.S
218102_at	DERA	0		N.S	1.11	4.83E-04
218103_at	FTSJ3	0		N.S		N.S
218104_at	TEX10	0		N.S	-1.15	2.33E-07
218105_s_at	MRPL4	0		N.S		N.S
218106_s_at	MRPS10	0	1.1	7.24E-10	1.13	1.26E-10
218107_at	WDR26	0	-1.1	2.75E-07		N.S
218108_at	UBR7	0		N.S		N.S
218109_s_at	MFSD1	0		N.S	1.13	2.50E-05
218110_at	XAB2	4		N.S		N.S
218111_s_at	CMAS	0		N.S		N.S
218112_at	MRPS34	0		N.S		N.S
218115_at	ASF1B	6		N.S	1.24	8.49E-15
218116_at	C9orf78	0		N.S	1.13	1.29E-03
218117_at	RBX1	13		N.S		N.S
218118_s_at	TIMM23	0		N.S		N.S
218122_s_at	SENP2	0		N.S	1.13	2.25E-04
218123_at	C21orf59	0		N.S	-1.29	1.41E-17
218124_at	RETSAT	0		N.S	1.99	1.45E-35
218125_s_at	CCDC25	0		N.S		N.S
218126_at	FAM82A2	0		N.S		N.S
218129_s_at	NFYB	2		N.S		N.S
218130_at	C17orf62	0	-1.1	3.78E-05	-1.23	2.85E-12
218131_s_at	GATAD2A	0		N.S		N.S
218132_s_at	TSEN34	0		N.S		N.S
218133_s_at	NIF3L1	0	-1.2	8.08E-18	-1.15	1.83E-13
218134_s_at	RBM22	0	1.1	1.02E-03		N.S
218135_at	ERGIC2	0		N.S		N.S
218137_s_at	SMAP1	0		N.S	1.09	1.28E-03
218138_at	MKKS	0	-1.2	8.17E-16		N.S
218139_s_at	MUDENG	0	-1.2	2.13E-12	1.21	2.26E-14
218142_s_at	CRBN	0		N.S		N.S
218143_s_at	SCAMP2	0		N.S		N.S
218145_at	TRIB3	1		N.S	-1.11	2.34E-02
218147_s_at	GLT8D1	0	-1.3	1.99E-19		N.S
218149_s_at	ZNF395	0	-1.3	3.53E-16	-2.51	1.97E-26
218150_at	ARL5A	0		N.S		N.S
218152_at	HMG20A	0	-1.2	9.31E-14		N.S
218153_at	CARS2	0		N.S	-1.16	2.28E-07
218154_at	GSDMD	0		N.S		N.S
218158_s_at	APPL1	0	-1.1	1.84E-02	-1.16	1.46E-04
218159_at	DDRGK1	1		N.S		N.S
218160_at	NDUFA8	0		N.S		N.S
218161_s_at	CLN6	0		N.S		N.S
218163_at	MCTS1	4		N.S		N.S
218164_at	SPATA20	0		N.S		N.S
218165_at	C1orf149	0		N.S		N.S
218166_s_at	RSF1	0	-1.3	2.90E-15		N.S
218167_at	AMZ2	0		N.S	1.23	1.21E-14
218168_s_at	CABC1	1		N.S	1.75	7.52E-32
218170_at	ISOC1	0		N.S		N.S
218171_at	VPS4B	0		N.S	1.15	1.19E-06
218172_s_at	DERL1	0	1.1	7.67E-03		N.S
218174_s_at	C10orf57	0		N.S		N.S
218176_at	MAGEF1	0	-1.2	2.07E-05	1.13	6.52E-03
218178_s_at	CHMP1B	0	1.1	5.37E-07		N.S

218179_s_at	C4orf41	0	-1.2	1.12E-04		N.S
218181_s_at	MAP4K4	0		N.S	1.55	1.92E-10
218183_at	C16orf5	0		N.S	2.08	2.57E-12
218184_at	TULP4	0	-1.1	8.09E-04	1.10	9.63E-03
218185_s_at	ARMC1	0		N.S	1.11	1.17E-02
218187_s_at	C8orf33	0	1.2	1.73E-05		N.S
218188_s_at	TIMM13	0		N.S		N.S
218189_s_at	NANS	0		N.S		N.S
218190_s_at	UCRC	0		N.S		N.S
218191_s_at	LMBRD1	0	-1.2	7.26E-17		N.S
218192_at	IP6K2	1	1.2	7.83E-11	1.20	4.64E-12
218193_s_at	GOLT1B	0	1.2	5.91E-09	1.15	1.35E-09
218194_at	REXO2	0		N.S		N.S
218195_at	C6orf211	0		N.S		N.S
218196_at	OSTM1	0		N.S	1.17	2.05E-02
218197_s_at	OXR1	4		N.S		N.S
218198_at	DHX32	0	-1.2	6.63E-12		N.S
218201_at	NDUFB2	0		N.S		N.S
218203_at	ALG5	0	1.2	9.29E-12		N.S
218204_s_at	FYCO1	0	-1.3	5.92E-08		N.S
218205_s_at	MKNK2	0		N.S		N.S
218208_at	LOC100131178	0		N.S		N.S
218209_s_at	RPRD1A	0	-1.4	1.37E-18		N.S
218210_at	FN3KRP	0		N.S	1.08	3.46E-02
218211_s_at	MLPH	0		N.S		N.S
218212_s_at	MOCS2	0		N.S	1.28	4.06E-08
218213_s_at	C11orf10	0		N.S		N.S
218214_at	C12orf44	0	-1.1	4.65E-02	-1.13	8.94E-04
218215_s_at	NR1H2	0		N.S		N.S
218217_at	SCPEP1	0		N.S		N.S
218218_at	APPL2	0		N.S		N.S
218219_s_at	LANCL2	0	1.3	1.88E-21	1.24	1.11E-10
218220_at	C12orf10	0		N.S		N.S
218221_at	ARNT	8		N.S		N.S
218223_s_at	PLEKHO1	0	-1.1	4.15E-02	-1.33	3.03E-20
218224_at	PNMA1	0		N.S	-1.10	1.34E-04
218225_at	ECSIT	0		N.S		N.S
218226_s_at	NDUFB4	0		N.S		N.S
218227_at	NUBP2	0		N.S		N.S
218228_s_at	TNKS2	0		N.S		N.S
218229_s_at	POGK	0		N.S		N.S
218230_at	ARFIP1	0	-1.2	1.93E-07	1.19	2.77E-07
218231_at	NAGK	0	-1.2	4.74E-14		N.S
218233_s_at	PRICKLE4	0		N.S		N.S
218235_s_at	UTP11L	0		N.S		N.S
218236_s_at	PRKD3	0	-1.1	1.59E-02	-1.23	7.43E-06
218237_s_at	SLC38A1	1		N.S	-1.16	4.09E-06
218239_s_at	GTPBP4	0	1.1	2.46E-03		N.S
218241_at	GOLGA5	1		N.S		N.S
218242_s_at	SUV420H1	0		N.S	1.37	8.62E-12
218243_at	RUFY1	0		N.S		N.S
218244_at	NOL8	0		N.S		N.S
218247_s_at	MEX3C	0		N.S		N.S
218248_at	FAM111A	0		N.S		N.S
218249_at	ZDHHC6	0	-1.1	5.14E-08		N.S
218250_s_at	CNOT7	1		N.S	-1.06	2.42E-02
218251_at	MID1IP1	0		N.S		N.S
218252_at	CKAP2	1	-1.1	8.50E-05	1.14	7.58E-04
218253_s_at	LGTN	0		N.S		N.S
218254_s_at	SAR1B	0		N.S		N.S

218255_s_at	FBRS	0		N.S		N.S
218257_s_at	UGCGL1	0		N.S		N.S
218258_at	POLR1D	0		N.S		N.S
218259_at	MKL2	0	-1.2	3.18E-10		N.S
218260_at	DDA1	1		N.S		N.S
218263_s_at	ZBED5	0	-1.3	6.63E-12		N.S
218264_at	BCCIP	9		N.S		N.S
218265_at	SECISBP2	1		N.S		N.S
218267_at	CINP	1		N.S		N.S
218268_at	TBC1D15	0		N.S		N.S
218269_at	RNASEN	2	-1.2	5.25E-07		N.S
218270_at	MRPL24	0		N.S		N.S
218271_s_at	PARL	7		N.S	1.09	2.90E-04
218273_s_at	PPM2C	0		N.S		N.S
218275_at	SLC25A10	0		N.S		N.S
218276_s_at	SAV1	0		N.S		N.S
218277_s_at	DHX40	0		N.S		N.S
218278_at	LOC649169	0		N.S		N.S
218281_at	MRPL48	0		N.S		N.S
218282_at	EDEM2	0		N.S		N.S
218283_at	SS18L2	0		N.S		N.S
218284_at	SMAD3	13		N.S		N.S
218285_s_at	BDH2	0		N.S		N.S
218286_s_at	RNF7	3		N.S		N.S
218287_s_at	EIF2C1	0		N.S		N.S
218288_s_at	CCDC90B	0		N.S	2.03	2.98E-45
218289_s_at	UBA5	0		N.S		N.S
218290_at	PLEKHJ1	0		N.S		N.S
218291_at	ROBLD3	0		N.S	1.24	2.28E-02
218294_s_at	NUP50	1		N.S		N.S
218297_at	C10orf97	0		N.S		N.S
218298_s_at	C14orf159	0		N.S		N.S
218301_at	RNPEPL1	0		N.S		N.S
218302_at	PSENEN	0		N.S		N.S
218304_s_at	OSBPL11	0		N.S		N.S
218305_at	IPO4	0		N.S	-1.13	1.54E-03
218306_s_at	HERC1	0		N.S	-1.20	1.76E-06
218307_at	RSAD1	0		N.S		N.S
218308_at	TACC3	0		N.S	-1.33	1.79E-09
218310_at	RABGEF1	0		N.S		N.S
218311_at	MAP4K3	1		N.S		N.S
218314_s_at	C11orf57	0	1.1	2.37E-03		N.S
218315_s_at	CDK5RAP1	1		N.S		N.S
218316_at	TIMM9	0		N.S		N.S
218318_s_at	NLK	0	-1.2	5.25E-06		N.S
218319_at	PELI1	0	1.1	1.86E-02	-1.14	6.95E-05
218320_s_at	NDUFB11	0		N.S		N.S
218322_s_at	ACSL5	0		N.S		N.S
218324_s_at	SPATS2	0	-1.2	4.11E-11		N.S
218326_s_at	LGR4	0		N.S		N.S
218327_s_at	SNAP29	0		N.S		N.S
218328_at	COQ4	0		N.S		N.S
218330_s_at	NAV2	0	-1.2	6.59E-07		N.S
218331_s_at	C10orf18	0		N.S		N.S
218333_at	DERL2	0		N.S	1.15	7.33E-03
218334_at	THOC7	0		N.S		N.S
218336_at	PFDN2	0		N.S		N.S
218337_at	FAM160B2	0		N.S		N.S
218339_at	MRPL22	0		N.S		N.S
218340_s_at	UBA6	0	-1.2	5.19E-04		N.S

218341_at	PPCS	0		N.S		N.S
218343_s_at	GTF3C3	0		N.S		N.S
218344_s_at	RCOR3	0		N.S		N.S
218346_s_at	SESN1	6	1.8	8.69E-18	5.28	1.41E-46
218347_at	TYW1	0	-1.1	1.44E-02		N.S
218348_s_at	ZC3H7A	0	1.2	3.93E-10	1.48	1.03E-34
218349_s_at	ZWILCH	1		N.S		N.S
218350_s_at	GMNN	18	1.3	4.47E-18		N.S
218351_at	COMMD8	0		N.S		N.S
218352_at	RCBTB1	0		N.S		N.S
218354_at	TRAPPC2L	0		N.S		N.S
218355_at	KIF4A	1		N.S	-1.16	2.64E-03
218357_s_at	TIMM8B	0		N.S		N.S
218358_at	CRELD2	0		N.S	1.16	4.49E-04
218361_at	GOLPH3L	0	-1.5	1.31E-22		N.S
218362_s_at	DIS3	0	1.2	5.05E-03		N.S
218363_at	EXD2	0	-1.1	1.07E-02		N.S
218364_at	LRRFIP2	0		N.S	1.10	1.93E-02
218365_s_at	DARS2	0		N.S	-1.18	1.37E-06
218370_s_at	S100PBP	0	-1.8	9.57E-27		N.S
218372_at	MED9	0	1.1	1.03E-03		N.S
218373_at	AKTIP	0		N.S		N.S
218374_s_at	C12orf4	0		N.S		N.S
218375_at	NUDT9	0	-1.1	1.16E-04	-1.18	2.96E-07
218376_s_at	MICAL1	0		N.S	-1.18	4.01E-04
218377_s_at	RWDD2B	0	-1.1	2.26E-04	1.10	2.05E-04
218378_s_at	PRKRIP1	0		N.S	1.21	3.28E-04
218379_at	RBM7	0	-1.1	4.50E-05		N.S
218381_s_at	U2AF2	0		N.S		N.S
218383_at	HAUS4	0		N.S		N.S
218384_at	CARHSP1	0		N.S		N.S
218385_at	MRPS18A	0		N.S		N.S
218388_at	PGLS	1		N.S		N.S
218389_s_at	APH1A	0		N.S		N.S
218391_at	SNF8	0		N.S		N.S
218393_s_at	SMU1	0		N.S		N.S
218394_at	ROGDI	0		N.S		N.S
218395_at	ACTR6	0		N.S		N.S
218396_at	VPS13C	0		N.S		N.S
218397_at	FANCL	18	-1.3	1.31E-13		N.S
218399_s_at	CDCA4	0	1.3	1.30E-15	-1.13	4.94E-03
218400_at	OAS3	0		N.S		N.S
218401_s_at	ZNF281	0	1.1	3.00E-02		N.S
218403_at	TRIAP1	1	1.5	1.39E-29	2.15	2.22E-44
218404_at	SNX10	0	-1.1	5.56E-09	-1.17	6.67E-09
218405_at	ABT1	0		N.S		N.S
218408_at	TIMM10	0		N.S		N.S
218411_s_at	MBIP	0		N.S		N.S
218414_s_at	NDE1	0	-1.3	1.64E-12		N.S
218415_at	VPS33B	0		N.S	1.35	6.63E-12
218419_s_at	TMUB2	0		N.S		N.S
218420_s_at	C13orf23	0		N.S		N.S
218421_at	CERK	0		N.S	-1.28	1.84E-13
218422_s_at	RBM26	0		N.S		N.S
218424_s_at	STEAP3	1		N.S		N.S
218426_s_at	RNF216	0	-1.2	2.07E-03		N.S
218427_at	SDCCAG3	0		N.S	-1.23	3.39E-09
218428_s_at	REV1	111		N.S		N.S
218429_s_at	C19orf66	0		N.S		N.S
218430_s_at	RFX7	0		N.S	1.66	4.44E-20

218431_at	C14orf133	0	-1.3	9.52E-16		N.S
218432_at	FBXO3	0		N.S		N.S
218433_at	PANK3	0		N.S		N.S
218434_s_at	AACS	0		N.S		N.S
218435_at	DNAJC15	0		N.S		N.S
218436_at	SIL1	0		N.S		N.S
218437_s_at	LZTFL1	0		N.S	-1.24	4.03E-02
218439_s_at	COMMD10	0		N.S		N.S
218440_at	MCCC1	0		N.S		N.S
218441_s_at	RPAP1	0		N.S		N.S
218443_s_at	DAZAP1	0		N.S	-1.12	1.67E-04
218446_s_at	FAM18B	0		N.S		N.S
218447_at	C16orf61	0		N.S		N.S
218448_at	C20orf11	0		N.S		N.S
218449_at	UFSP2	0		N.S		N.S
218452_at	SMARCAL1	6	-1.3	8.77E-08		N.S
218455_at	NFS1	0	-1.1	7.86E-06	-1.13	4.96E-07
218456_at	CAPRIN2	0	-1.2	1.19E-06	1.16	1.90E-06
218458_at	GMCL1	0	-1.3	3.73E-10		N.S
218459_at	TOR3A	0	1.2	1.48E-13	1.15	2.03E-07
218460_at	HEATR2	0		N.S	-1.22	1.56E-12
218461_at	GPN3	0		N.S	1.09	1.34E-02
218462_at	BXDC5	0		N.S		N.S
218463_s_at	MUS81	71	-1.2	1.03E-13	-1.11	9.78E-05
218465_at	TMEM33	0	-1.1	5.91E-03		N.S
218467_at	PSMG2	0		N.S		N.S
218470_at	YARS2	0		N.S		N.S
218471_s_at	BBS1	0		N.S	1.23	3.38E-10
218473_s_at	GLT25D1	0		N.S		N.S
218474_s_at	KCTD5	0		N.S	-1.11	4.12E-03
218477_at	TMEM14A	0		N.S		N.S
218478_s_at	ZCCHC8	0	-1.2	1.24E-08		N.S
218479_s_at	XPO4	0		N.S		N.S
218481_at	EXOSC5	0		N.S		N.S
218482_at	ENY2	0		N.S		N.S
218483_s_at	C11orf60	0		N.S		N.S
218487_at	ALAD	6		N.S		N.S
218488_at	EIF2B3	0	-1.1	2.27E-06		N.S
218490_s_at	ZNF302	0	-1.3	1.66E-15	1.14	1.41E-04
218491_s_at	THYN1	0		N.S		N.S
218492_s_at	THAP7	0		N.S		N.S
218493_at	SNRNP25	0		N.S		N.S
218494_s_at	SLC2A4RG	0		N.S		N.S
218495_at	UXT	1		N.S		N.S
218496_at	RNASEH1	0	1.3	1.93E-13		N.S
218498_s_at	ERO1L	0		N.S	-1.45	1.59E-13
218499_at	RP6-213H19.1	0		N.S	-1.22	3.05E-19
218501_at	ARHGEF3	0	1.3	8.32E-07	1.36	2.08E-14
218503_at	KIAA1797	0		N.S	-1.30	4.15E-12
218507_at	C7orf68	0	-1.6	1.68E-15	-1.64	1.19E-14
218508_at	DCP1A	0		N.S		N.S
218511_s_at	PNPO	0	-1.1	2.86E-05		N.S
218512_at	WDR12	1		N.S	-1.07	3.18E-03
218513_at	C4orf43	0		N.S		N.S
218514_at	C17orf71	0		N.S		N.S
218515_at	C21orf66	0		N.S		N.S
218516_s_at	IMPAD1	0		N.S		N.S
218517_at	PHF17	0		N.S		N.S
218518_at	FAM13B	0		N.S	1.22	1.59E-09
218519_at	SLC35A5	0		N.S	1.18	4.86E-07

218520_at	TBK1	0		N.S	1.22	1.61E-15
218521_s_at	UBE2W	0		N.S		N.S
218524_at	E4F1	0		N.S		N.S
218525_s_at	HIF1AN	1	-1.1	3.70E-02		N.S
218526_s_at	RANGRF	0		N.S	-1.16	6.19E-03
218527_at	APTX	20		N.S	1.62	3.87E-37
218528_s_at	RNF38	0		N.S		N.S
218529_at	CD320	0		N.S		N.S
218531_at	TMEM134	0		N.S		N.S
218532_s_at	FAM134B	0	-1.3	2.60E-02		N.S
218533_s_at	UCKL1	0		N.S		N.S
218534_s_at	AGGF1	1	-1.2	6.50E-11	1.11	4.16E-04
218535_s_at	RIOK2	0		N.S		N.S
218536_at	MRS2	0	-1.1	5.96E-04		N.S
218539_at	FBXO34	0		N.S		N.S
218542_at	CEP55	0	-1.1	1.40E-06	-1.36	4.63E-16
218543_s_at	PARP12	0		N.S	-1.16	7.02E-04
218544_s_at	RCL1	0	1.1	4.46E-02		N.S
218545_at	CCDC91	0		N.S		N.S
218547_at	DHDDS	0		N.S	1.18	4.36E-08
218549_s_at	FAM82B	0		N.S	1.10	1.30E-04
218550_s_at	LRRC20	0	-1.2	5.38E-03		N.S
218552_at	ECHDC2	0		N.S		N.S
218555_at	ANAPC2	0		N.S		N.S
218556_at	ORMDL2	0		N.S		N.S
218557_at	NIT2	1		N.S		N.S
218558_s_at	MRPL39	0		N.S	1.31	1.91E-23
218561_s_at	LYRM4	0		N.S		N.S
218562_s_at	TMEM57	0	1.4	7.92E-09	1.75	6.26E-31
218563_at	NDUFA3	0		N.S		N.S
218564_at	RFWD3	1	1.2	3.16E-06		N.S
218565_at	C9orf114	0		N.S		N.S
218566_s_at	CHORDC1	0	-1.3	2.55E-21	-1.14	3.18E-02
218568_at	AGK	0		N.S		N.S
218570_at	KBTBD4	0		N.S		N.S
218571_s_at	CHMP4A	0		N.S		N.S
218573_at	MAGEH1	0		N.S		N.S
218574_s_at	LMCD1	0	-1.2	1.73E-03		N.S
218575_at	ANAPC1	0		N.S		N.S
218576_s_at	DUSP12	0	1.2	2.60E-07		N.S
218577_at	LRRC40	0	-1.1	1.54E-02	1.13	4.56E-02
218578_at	CDC73	3	-1.2	1.72E-06		N.S
218579_s_at	DHX35	0		N.S		N.S
218581_at	ABHD4	0		N.S	1.43	1.03E-21
218582_at	MARCH5	0		N.S		N.S
218583_s_at	DCUN1D1	0	-1.1	1.11E-04	1.14	5.46E-10
218584_at	TCTN1	0		N.S		N.S
218585_s_at	DTL	6	1.2	6.47E-10		N.S
218586_at	C20orf20	0		N.S	-1.25	1.85E-09
218587_s_at	KTELC1	0		N.S	1.32	6.77E-16
218588_s_at	FAM114A2	0	-1.1	2.76E-03		N.S
218589_at	P2RY5	0		N.S	1.37	4.44E-02
218590_at	C10orf2	1		N.S	-1.22	3.41E-09
218591_s_at	NOL10	0		N.S		N.S
218592_s_at	CECR5	0		N.S	-1.32	5.55E-19
218593_at	RBM28	0		N.S		N.S
218594_at	HEATR1	0		N.S		N.S
218597_s_at	CISD1	0		N.S		N.S
218598_at	RINT1	1		N.S		N.S
218599_at	REC8	6		N.S		N.S

218602_s_at	HAUS6	0		N.S		N.S
218603_at	HECA	0		N.S		N.S
218604_at	LEMD3	0		N.S		N.S
218605_at	TFB2M	0	1.2	1.31E-08		N.S
218606_at	ZDHHC7	0		N.S		N.S
218607_s_at	SDAD1	0		N.S		N.S
218609_s_at	NUDT2	0		N.S		N.S
218610_s_at	CPPED1	0		N.S		N.S
218611_at	IER5	2	1.6	4.45E-34	1.46	3.20E-33
218614_at	C12orf35	0	-1.2	8.25E-05		N.S
218615_s_at	TMEM39A	0		N.S		N.S
218616_at	INTS12	0		N.S		N.S
218617_at	TRIT1	0		N.S	-1.11	1.80E-06
218618_s_at	FNDC3B	0	-1.2	9.50E-03		N.S
218619_s_at	SUV39H1	6		N.S		N.S
218622_at	NUP37	0		N.S		N.S
218624_s_at	MGC2752	0		N.S	1.13	1.35E-02
218626_at	EIF4ENIF1	0	-1.2	2.71E-10		N.S
218627_at	DRAM	2	1.4	1.22E-08	2.08	9.20E-35
218628_at	CCDC53	0		N.S		N.S
218630_at	MKS1	0	-1.1	2.10E-02		N.S
218632_at	HECTD3	0		N.S		N.S
218634_at	PHLDA3	0	1.5	1.85E-22	2.42	2.46E-38
218636_s_at	MAN1B1	0		N.S		N.S
218637_at	IMPACT	1509		N.S		N.S
218640_s_at	PLEKHF2	0	-1.2	2.15E-06		N.S
218641_at	LOC65998	0		N.S		N.S
218642_s_at	CHCHD7	0	1.4	1.57E-23	1.24	2.15E-15
218643_s_at	CRIPT	0		N.S		N.S
218645_at	ZNF277	0		N.S	1.14	3.86E-06
218646_at	C4orf27	0		N.S		N.S
218647_s_at	YRDC	0	1.3	6.63E-12	-1.17	2.02E-05
218648_at	CRTC3	0		N.S		N.S
218650_at	DGCR8	1		N.S		N.S
218652_s_at	PIGG	0		N.S		N.S
218653_at	SLC25A15	0		N.S	1.24	7.47E-08
218654_s_at	MRPS33	0		N.S		N.S
218655_s_at	CCDC49	0		N.S		N.S
218656_s_at	LHFP	0		N.S	1.36	4.62E-06
218658_s_at	ACTR8	0		N.S	1.16	2.46E-03
218659_at	ASXL2	0	-1.3	7.87E-15		N.S
218661_at	NAT15	0		N.S		N.S
218663_at	NCAPG	0		N.S		N.S
218664_at	MECR	0		N.S		N.S
218666_s_at	STX17	0		N.S		N.S
218667_at	PJA1	0	-1.1	2.16E-04		N.S
218669_at	RAP2C	0		N.S		N.S
218670_at	PUS1	0		N.S	-1.17	1.58E-03
218671_s_at	ATPIF1	0		N.S		N.S
218672_at	SCNM1	0		N.S		N.S
218673_s_at	ATG7	3		N.S		N.S
218674_at	C5orf44	0		N.S	1.21	1.09E-02
218676_s_at	PCTP	0		N.S		N.S
218679_s_at	VPS28	0		N.S		N.S
218681_s_at	SDF2L1	0		N.S		N.S
218682_s_at	SLC4A1AP	0		N.S	1.09	3.56E-03
218683_at	PTBP2	0		N.S		N.S
218684_at	LRRC8D	0	-1.2	8.57E-09	-1.23	3.06E-17
218685_s_at	SMUG1	18	-1.3	3.25E-10	1.22	3.34E-07
218688_at	DAK	1		N.S		N.S

Supplemental Table 2

218689_at	FANCF	18	-1.3	7.51E-09		N.S
218692_at	GOLSYN	0		N.S	-1.34	6.23E-09
218693_at	TSPAN15	0		N.S		N.S
218694_at	ARMCX1	0		N.S		N.S
218696_at	EIF2AK3	2		N.S		N.S
218698_at	APIP	1		N.S		N.S
218699_at	RAB7L1	0	-1.2	1.77E-06		N.S
218701_at	LACTB2	0		N.S		N.S
218703_at	SEC22A	0	-1.2	1.11E-08	1.38	2.41E-22
218705_s_at	SNX24	0		N.S		N.S
218706_s_at	GRAMD3	0		N.S	1.43	5.03E-15
218708_at	NXT1	0	1.2	2.58E-08	-1.21	1.78E-06
218709_s_at	IFT52	0		N.S		N.S
218710_at	TTC27	0		N.S		N.S
218712_at	C1orf109	0		N.S		N.S
218713_at	NARG2	0		N.S		N.S
218715_at	UTP6	0	-1.1	2.30E-10		N.S
218719_s_at	GINS3	1	1.4	1.11E-18		N.S
218721_s_at	C1orf27	0		N.S		N.S
218722_s_at	CCDC51	0	-1.3	1.93E-16		N.S
218723_s_at	C13orf15	0		N.S	-1.56	3.80E-16
218726_at	HJURP	1	-1.5	6.45E-18	-1.35	1.06E-11
218728_s_at	CNIH4	0		N.S		N.S
218729_at	LXN	0		N.S		N.S
218732_at	PTRH2	0		N.S	-1.31	1.53E-10
218733_at	MSL2	0		N.S	1.21	3.52E-05
218735_s_at	ZNF544	0		N.S		N.S
218738_s_at	RNF138	0	1.1	1.12E-05		N.S
218739_at	ABHD5	0		N.S		N.S
218740_s_at	CDK5RAP3	3		N.S		N.S
218741_at	CENPM	0		N.S		N.S
218743_at	CHMP6	0		N.S		N.S
218746_at	TAPBPL	0		N.S	1.24	6.01E-10
218748_s_at	EXOC5	0		N.S		N.S
218751_s_at	FBXW7	8		N.S	1.58	2.40E-21
218753_at	XKR8	0		N.S		N.S
218754_at	NOL9	0		N.S		N.S
218755_at	KIF20A	0	-1.6	7.08E-26	-1.67	9.71E-17
218756_s_at	DHRS11	0		N.S		N.S
218757_s_at	UPF3B	0		N.S	1.14	1.76E-05
218760_at	COQ6	0		N.S		N.S
218761_at	RNF111	0		N.S	1.21	9.83E-10
218763_at	STX18	0	-1.1	9.08E-04		N.S
218764_at	PRKCH	0		N.S		N.S
218766_s_at	WARS2	0		N.S	1.11	5.76E-04
218767_at	REXO4	0		N.S		N.S
218768_at	NUP107	0		N.S		N.S
218769_s_at	ANKRA2	0	1.2	4.30E-07	2.39	2.19E-39
218770_s_at	TMEM39B	0		N.S		N.S
218771_at	PANK4	0		N.S		N.S
218774_at	DCPS	0		N.S		N.S
218776_s_at	TMEM62	0		N.S		N.S
218777_at	REEP4	0		N.S		N.S
218781_at	SMC6	34	-1.1	3.11E-06	1.08	1.89E-02
218782_s_at	ATAD2	1		N.S		N.S
218788_s_at	SMYD3	0		N.S	-1.17	1.57E-07
218789_s_at	C11orf71	0		N.S	1.11	1.90E-02
218791_s_at	C15orf29	0	-1.3	1.02E-22		N.S
218794_s_at	TXNL4B	0	1.1	3.63E-06	1.12	8.87E-07
218795_at	ACP6	0		N.S		N.S

Supplemental Table 2

218797_s_at	SIRT7	4		N.S	1.13	2.42E-02
218799_at	GPN2	0		N.S		N.S
218801_at	UGCGL2	0		N.S		N.S
218802_at	CCDC109B	0		N.S		N.S
218803_at	CHFR	6		N.S		N.S
218809_at	PANK2	0		N.S	1.16	2.32E-11
218810_at	ZC3H12A	0	1.4	6.10E-18		N.S
218812_s_at	ORAI2	0		N.S	-1.19	5.67E-05
218813_s_at	SH3GLB2	0		N.S		N.S
218817_at	SPCS3	0		N.S		N.S
218823_s_at	KCTD9	0	1.1	7.17E-05	1.11	1.26E-02
218826_at	SLC35F2	0	1.2	1.43E-09		N.S
218827_s_at	CEP192	0	-1.1	2.51E-02		N.S
218830_at	RPL26L1	0		N.S		N.S
218833_at	ZAK	7		N.S		N.S
218836_at	RPP21	0		N.S		N.S
218837_s_at	UBE2D4	0		N.S		N.S
218838_s_at	TTC31	0		N.S		N.S
218840_s_at	NADSYN1	0		N.S	1.27	7.30E-12
218841_at	ASB8	0		N.S	1.10	1.73E-02
218842_at	RPAP3	1		N.S		N.S
218845_at	DUSP22	1	-1.1	7.49E-07		N.S
218846_at	MED23	0		N.S	1.36	5.40E-17
218848_at	THOC6	0		N.S		N.S
218850_s_at	LIMD1	0	-1.2	1.17E-07	-1.18	2.20E-04
218852_at	PPP2R3C	0	1.2	5.97E-12	1.09	8.84E-05
218853_s_at	MOSPD1	0	1.2	1.69E-07	1.60	1.43E-38
218854_at	DSE	5		N.S	1.49	3.85E-33
218859_s_at	ESF1	0		N.S		N.S
218860_at	NOC4L	0		N.S		N.S
218866_s_at	POLR3K	0		N.S		N.S
218867_s_at	C12orf49	0		N.S		N.S
218868_at	ACTR3B	0	-1.1	6.70E-03		N.S
218870_at	ARHGAP15	0		N.S	-1.48	1.31E-30
218872_at	TESC	0		N.S		N.S
218873_at	GON4L	0	-1.1	3.30E-02		N.S
218875_s_at	FBXO5	6		N.S		N.S
218877_s_at	TRMT11	0		N.S		N.S
218878_s_at	SIRT1	80	1.3	6.61E-10		N.S
218879_s_at	MTHFSD	0		N.S		N.S
218882_s_at	WDR3	0	-1.1	2.71E-04	-1.24	8.97E-09
218883_s_at	MLF1IP	0	1.3	3.04E-25	1.33	2.62E-23
218884_s_at	GUF1	0		N.S	1.11	2.47E-02
218886_at	PAK1IP1	0		N.S	-1.25	1.76E-05
218887_at	MRPL2	0		N.S		N.S
218888_s_at	NETO2	0		N.S		N.S
218889_at	NOC3L	0	-1.2	1.99E-11	-1.13	2.87E-03
218893_at	ISOC2	0		N.S		N.S
218894_s_at	MAGOHB	0		N.S		N.S
218895_at	GPATCH3	0		N.S		N.S
218896_s_at	C17orf85	0		N.S		N.S
218897_at	TMEM177	0	-1.2	1.23E-06		N.S
218898_at	FAM57A	0		N.S	-1.38	2.42E-13
218902_at	NOTCH1	12		N.S	1.66	2.94E-24
218903_s_at	OBFC2B	0		N.S		N.S
218904_s_at	C9orf40	0	-1.4	6.00E-24		N.S
218905_at	INTS8	0	-1.1	1.33E-03	-1.17	3.39E-10
218907_s_at	LRRC61	0		N.S		N.S
218909_at	RPS6KC1	0	-1.2	7.73E-07		N.S
218911_at	YEATS4	0	1.2	2.53E-09		N.S

218912_at	GCC1	0	1.1	4.57E-03		N.S
218913_s_at	GMIP	0		N.S		N.S
218916_at	ZNF768	0		N.S		N.S
218919_at	ZFAND1	0		N.S		N.S
218920_at	FLJ10404	0		N.S		N.S
218924_s_at	CTBS	1		N.S		N.S
218926_at	MYNN	0		N.S	1.12	2.04E-03
218927_s_at	CHST12	0	-1.2	2.13E-05	-1.42	9.17E-15
218929_at	CDKN2AIP	1		N.S	1.17	1.11E-03
218930_s_at	TMEM106B	0	-1.2	2.02E-05		N.S
218932_at	ZNHIT6	0		N.S		N.S
218936_s_at	CCDC59	0	1.2	7.33E-04		N.S
218937_at	ZNF434	0		N.S		N.S
218938_at	FBXL15	0		N.S		N.S
218940_at	C14orf138	0	1.2	1.70E-07		N.S
218945_at	C16orf68	0		N.S		N.S
218946_at	NFU1	0		N.S		N.S
218947_s_at	MTPAP	0		N.S		N.S
218949_s_at	QRSL1	0	-1.1	5.04E-11	-1.09	1.45E-04
218951_s_at	PLCXD1	0	-1.2	2.09E-02	-1.38	9.95E-12
218956_s_at	PTCD1	0		N.S		N.S
218957_s_at	PAAF1	0	-1.2	1.21E-14		N.S
218961_s_at	PNKP	8		N.S	-1.12	4.50E-03
218962_s_at	TMEM168	0		N.S	2.01	1.01E-40
218966_at	MYO5C	0		N.S		N.S
218967_s_at	PTER	2		N.S		N.S
218968_s_at	ZFP64	0		N.S		N.S
218969_at	Magmas	0		N.S		N.S
218970_s_at	CUTC	0		N.S	-1.21	6.34E-16
218971_s_at	WDR91	0	-1.2	1.16E-08		N.S
218972_at	TTC17	0	-1.2	1.46E-08		N.S
218973_at	EFTUD1	0		N.S	-1.16	2.94E-04
218974_at	SOBP	4		N.S	-1.34	1.53E-09
218977_s_at	TRNAU1AP	0		N.S	1.18	1.13E-02
218979_at	RMI1	8		N.S	1.16	7.22E-06
218981_at	ACN9	0		N.S		N.S
218982_s_at	MRPS17	0		N.S		N.S
218983_at	C1RL	0	-1.1	1.23E-02	1.22	3.77E-05
218984_at	PUS7	0		N.S	-1.21	2.90E-09
218986_s_at	DDX60	0		N.S	-1.47	1.27E-18
218987_at	ATF7IP	0		N.S		N.S
218988_at	SLC35E3	0	1.2	2.27E-03	1.82	5.06E-27
218992_at	C9orf46	0		N.S		N.S
218993_at	RNMTL1	0		N.S		N.S
218996_at	TFPT	0		N.S		N.S
218997_at	POLR1E	0	1.1	1.41E-03		N.S
218998_at	C9orf6	0		N.S		N.S
218999_at	TMEM140	0	-1.3	1.50E-03	-1.20	1.16E-02
219001_s_at	WDR32	0		N.S		N.S
219002_at	FASTKD1	0	-1.2	4.25E-10		N.S
219003_s_at	MANEA	0		N.S		N.S
219004_s_at	C21orf45	0		N.S		N.S
219006_at	NDUFAF4	0		N.S	-1.19	4.49E-04
219007_at	NUP43	0	-1.1	2.25E-07		N.S
219012_s_at	C11orf30	0	-1.3	5.12E-07		N.S
219013_at	GALNT11	0		N.S		N.S
219014_at	PLAC8	0		N.S		N.S
219016_at	FASTKD5	0		N.S		N.S
219021_at	RNF121	0		N.S	1.19	5.69E-04
219022_at	C12orf43	0		N.S		N.S

219023_at	C4orf16	0		N.S		N.S
219025_at	CD248	0		N.S		N.S
219027_s_at	MYO9A	0		N.S		N.S
219029_at	C5orf28	0		N.S		N.S
219030_at	TPRKB	0		N.S		N.S
219031_s_at	NIP7	1	1.3	2.58E-10		N.S
219033_at	PARP8	0	-1.2	1.39E-07	-1.17	1.15E-03
219034_at	PARP16	0		N.S		N.S
219035_s_at	RNF34	0		N.S		N.S
219038_at	MORC4	0		N.S		N.S
219041_s_at	REPIN1	0		N.S	-1.25	3.24E-10
219043_s_at	LOC285359	0		N.S		N.S
219045_at	RHOF	0		N.S	-1.17	2.91E-05
219052_at	HPS6	0		N.S		N.S
219053_s_at	VPS37C	0		N.S		N.S
219055_at	SRBD1	0	-1.2	5.65E-09		N.S
219060_at	WDYHV1	0		N.S		N.S
219061_s_at	LAGE3	0		N.S		N.S
219062_s_at	ZCCHC2	0	-1.3	1.20E-08		N.S
219063_at	C1orf35	0		N.S		N.S
219065_s_at	MEMO1	0		N.S		N.S
219066_at	PPCDC	0	1.1	1.28E-04		N.S
219067_s_at	NSMCE4A	0		N.S		N.S
219069_at	ANKRD49	0		N.S		N.S
219074_at	TMEM184C	0		N.S	1.22	1.74E-08
219076_s_at	PXMP2	0		N.S		N.S
219077_s_at	WWOX	12		N.S		N.S
219078_at	GPATCH2	0		N.S		N.S
219079_at	CYB5R4	0		N.S		N.S
219080_s_at	CTPS2	0		N.S	-1.15	7.07E-08
219086_at	ZNF839	0		N.S		N.S
219089_s_at	ZNF576	0		N.S		N.S
219092_s_at	IPPK	0		N.S		N.S
219098_at	MYBBP1A	0		N.S		N.S
219099_at	C12orf5	1	1.6	3.42E-27	2.24	8.92E-44
219100_at	OBFC1	0		N.S		N.S
219104_at	RNF141	0		N.S		N.S
219109_at	SPAG16	0		N.S	-1.14	3.56E-02
219110_at	GAR1	0		N.S		N.S
219111_s_at	DDX54	0		N.S		N.S
219112_at	RAPGEF6	0	-1.2	1.32E-03		N.S
219116_s_at	DCUN1D2	0	1.2	2.52E-03		N.S
219117_s_at	FKBP11	0		N.S		N.S
219119_at	LSM8	0		N.S		N.S
219120_at	C2orf44	0	-1.2	4.56E-02		N.S
219122_s_at	THG1L	0		N.S		N.S
219123_at	ZNF232	0	-1.4	6.09E-23		N.S
219124_at	C8orf41	0	-1.1	2.88E-05		N.S
219125_s_at	RAG1AP1	0		N.S		N.S
219126_at	PHF10	0	-1.2	2.77E-16	1.12	9.98E-08
219128_at	C2orf42	0		N.S		N.S
219129_s_at	SAP30L	0		N.S	1.13	4.70E-02
219130_at	CCDC76	0	-1.2	1.27E-09		N.S
219131_at	UBIAD1	0	1.3	6.89E-17	-1.23	1.53E-09
219133_at	OXSM	0	-1.3	4.20E-25		N.S
219137_s_at	MFF	0		N.S		N.S
219142_at	RASL11B	0		N.S		N.S
219143_s_at	RPP25	0		N.S	-1.28	3.89E-13
219146_at	C17orf42	0	-1.2	5.77E-05	1.12	3.47E-03
219147_s_at	C9orf95	0	-1.1	2.12E-05	-1.27	2.91E-13

219148_at	PBK	4		N.S		N.S
219155_at	PITPNC1	0		N.S	-1.11	5.46E-03
219156_at	SYNJ2BP	0	-1.2	9.40E-04		N.S
219157_at	KLHL2	0	-1.2	2.13E-10		N.S
219158_s_at	NARG1	0		N.S		N.S
219159_s_at	SLAMF7	0	-1.2	1.74E-05		N.S
219162_s_at	MRPL11	0		N.S		N.S
219163_at	ZNF562	0		N.S		N.S
219164_s_at	ATG2B	0	-1.3	1.93E-07		N.S
219165_at	PDLIM2	0		N.S		N.S
219166_at	C14orf104	0	-1.4	1.50E-16	-1.19	2.38E-06
219169_s_at	TFB1M	0	-1.3	4.46E-16	-1.12	1.86E-04
219174_at	IFT74	0		N.S		N.S
219175_s_at	SLC41A3	0		N.S		N.S
219176_at	C2orf47	0		N.S		N.S
219177_at	BXDC2	0		N.S	-1.24	5.26E-05
219178_at	QTRTD1	0		N.S		N.S
219180_s_at	PEX26	0		N.S		N.S
219189_at	FBXL6	0		N.S		N.S
219192_at	UBAP2	0		N.S		N.S
219193_at	WDR70	0		N.S		N.S
219198_at	GTF3C4	0	1.2	9.27E-03		N.S
219200_at	FASTKD3	0		N.S		N.S
219201_s_at	TWSG1	0		N.S		N.S
219203_at	FAM158A	0		N.S	-1.17	1.93E-02
219205_at	SRR	1		N.S		N.S
219207_at	EDC3	0		N.S	1.15	7.78E-03
219210_s_at	RAB8B	0	-1.2	2.30E-07		N.S
219211_at	USP18	0	1.2	6.34E-03		N.S
219212_at	HSPA14	0	1.2	2.69E-16	1.14	8.33E-06
219214_s_at	NT5C	0		N.S		N.S
219215_s_at	SLC39A4	0		N.S		N.S
219216_at	ETAA1	0		N.S		N.S
219217_at	NARS2	0	-1.2	7.89E-11	-1.21	2.38E-14
219219_at	TMEM160	0		N.S		N.S
219221_at	ZBTB38	0	-1.5	3.97E-10	-1.28	4.83E-06
219228_at	ZNF331	0	-1.1	1.92E-06	-1.14	1.44E-03
219231_at	TGS1	0	1.3	2.78E-05	1.48	2.11E-16
219232_s_at	EGLN3	1		N.S	-1.70	1.69E-12
219235_s_at	PHACTR4	0		N.S	1.25	2.19E-11
219237_s_at	DNAJB14	0		N.S	1.15	1.50E-02
219238_at	PIGV	0		N.S		N.S
219239_s_at	ZNF654	0		N.S	1.50	2.99E-18
219240_s_at	C10orf88	0		N.S	1.15	2.29E-06
219242_at	CEP63	1		N.S		N.S
219244_s_at	MRPL46	0		N.S		N.S
219248_at	THUMPD2	0		N.S		N.S
219252_s_at	GEMIN8	0	-1.2	9.54E-04	-1.35	7.30E-12
219253_at	TMEM185B	0	1.2	1.19E-11	1.23	2.43E-09
219256_s_at	SH3TC1	0		N.S	1.14	5.92E-03
219258_at	TIPIN	8	1.3	5.24E-11	-1.18	1.13E-06
219259_at	SEMA4A	0		N.S		N.S
219260_s_at	C17orf81	0		N.S		N.S
219262_at	SUV39H2	0	-1.2	2.80E-07		N.S
219267_at	GLTP	0		N.S	1.29	9.13E-21
219269_at	HMBOX1	0		N.S		N.S
219274_at	TSPAN12	0	-1.6	5.05E-21		N.S
219275_at	PDCD5	1		N.S		N.S
219279_at	DOCK10	0	-1.2	5.02E-10	-1.37	1.18E-16
219280_at	BRWD1	0	-1.3	1.01E-02		N.S

Supplemental Table 2

219281_at	MSRA	1		N.S		N.S
219282_s_at	TRPV2	0		N.S		N.S
219283_at	C1GALT1C1	0	-1.1	8.15E-04	-1.19	9.37E-10
219284_at	HSPBAP1	0	-1.3	2.71E-08	1.30	1.84E-13
219286_s_at	RBM15	0	1.4	4.01E-24	-1.21	1.13E-09
219287_at	KCNMB4	0		N.S		N.S
219288_at	C3orf14	0		N.S		N.S
219289_at	HEATR3	0	-1.1	2.05E-05	-1.14	6.81E-06
219291_at	DTWD1	0	-1.2	1.84E-05	1.24	2.69E-06
219292_at	THAP1	0		N.S	-1.15	1.28E-05
219293_s_at	OLA1	0		N.S		N.S
219294_at	CENPQ	0		N.S		N.S
219296_at	ZDHHC13	0	-1.2	4.44E-03	1.17	9.92E-05
219297_at	WDR44	0		N.S		N.S
219299_at	TRMT12	0		N.S		N.S
219303_at	RNF219	0		N.S		N.S
219304_s_at	PDGFD	0		N.S		N.S
219306_at	KIF15	0	-1.1	2.06E-05	-1.18	6.91E-06
219311_at	CEP76	0		N.S	1.23	1.12E-05
219312_s_at	ZBTB10	0	1.6	3.60E-17		N.S
219317_at	POLI	34		N.S		N.S
219320_at	MYO19	0		N.S		N.S
219321_at	MPP5	0		N.S		N.S
219322_s_at	WDR8	0		N.S	1.57	2.20E-34
219324_at	NOL12	0		N.S		N.S
219325_s_at	ELAC1	0		N.S	1.23	8.15E-05
219326_s_at	B3GNT2	0		N.S	-1.26	2.02E-07
219329_s_at	C2orf28	0		N.S		N.S
219330_at	VANGL1	0		N.S		N.S
219334_s_at	OBFC2A	0		N.S	1.53	9.72E-09
219335_at	ARMCX5	0		N.S		N.S
219336_s_at	ASCC1	0		N.S	1.20	5.88E-08
219338_s_at	LRRC49	0	-1.1	3.73E-02		N.S
219342_at	CASD1	0		N.S		N.S
219343_at	CDC37L1	0		N.S	1.19	1.10E-07
219345_at	BOLA1	0	-1.4	6.98E-15		N.S
219347_at	NUDT15	0	1.6	6.58E-26	1.61	1.41E-29
219348_at	USE1	0		N.S		N.S
219350_s_at	DIABLO	24		N.S	1.10	5.28E-05
219351_at	TRAPPC2	0		N.S		N.S
219352_at	HERC6	0		N.S	-1.30	1.67E-06
219353_at	NHLRC2	0		N.S		N.S
219357_at	GTPBP1	0		N.S		N.S
219361_s_at	AEN	3	2.1	5.16E-34	1.80	1.51E-32
219363_s_at	MTERFD1	0	-1.4	1.99E-27	-1.14	5.18E-08
219366_at	AVEN	4		N.S		N.S
219368_at	NAP1L2	0		N.S		N.S
219371_s_at	KLF2	1	-1.9	3.14E-19	-1.93	2.84E-23
219372_at	IFT81	0	-1.1	6.85E-05	-1.22	2.17E-06
219374_s_at	ALG9	0		N.S	1.09	1.43E-03
219375_at	CEPT1	0	-1.3	6.10E-19	1.15	5.13E-05
219376_at	ZNF322B	0		N.S	1.48	3.64E-17
219378_at	NARG1L	0		N.S		N.S
219382_at	SERTAD3	0	-1.2	3.50E-03		N.S
219384_s_at	ADAT1	0		N.S	1.20	1.44E-09
219387_at	CCDC88A	1		N.S		N.S
219390_at	FKBP14	0		N.S		N.S
219394_at	PGS1	0		N.S		N.S
219397_at	COQ10B	0	1.2	5.23E-19		N.S
219405_at	TRIM68	0		N.S	1.36	6.32E-17

219406_at	C1orf50	0		N.S		N.S
219409_at	SNIP1	2	1.2	3.60E-07		N.S
219410_at	TMEM45A	0	-1.2	2.13E-05	-1.57	3.16E-14
219412_at	RAB38	0		N.S	1.45	4.46E-04
219421_at	TTC33	0	-1.4	7.77E-17	1.21	2.32E-06
219424_at	EBI3	0		N.S		N.S
219426_at	EIF2C3	0	-1.2	6.40E-05		N.S
219428_s_at	PXMP4	0		N.S		N.S
219429_at	FA2H	0		N.S		N.S
219431_at	ARHGAP10	0		N.S		N.S
219433_at	BCOR	1	1.2	3.03E-04		N.S
219435_at	C17orf68	0		N.S	-1.17	3.39E-04
219437_s_at	ANKRD11	1		N.S		N.S
219439_at	C1GALT1	0	-1.1	7.68E-04		N.S
219441_s_at	LRRK1	0	-1.3	2.05E-06	-1.19	3.28E-02
219443_at	TASP1	0		N.S		N.S
219444_at	BCORL1	1		N.S		N.S
219446_at	RIC8B	0	-1.4	1.44E-17		N.S
219447_s_at	SLC35C2	0		N.S		N.S
219449_s_at	TMEM70	0		N.S		N.S
219458_s_at	NSUN3	0		N.S		N.S
219459_at	POLR3B	0	-1.1	3.64E-03		N.S
219462_at	TMEM53	0		N.S		N.S
219467_at	GIN1	0	-1.3	7.58E-16		N.S
219471_at	C13orf18	0	-1.3	5.51E-11		N.S
219472_at	CENPO	0		N.S		N.S
219473_at	GDAP2	0		N.S		N.S
219479_at	KDELC1	0	-1.2	3.74E-14	1.14	3.61E-04
219481_at	TTC13	0		N.S		N.S
219484_at	HCFC2	0	-1.1	4.72E-02	1.10	4.29E-02
219485_s_at	PSMD10	2		N.S		N.S
219487_at	BBS10	0	-1.6	4.52E-12		N.S
219489_s_at	NXN	1		N.S		N.S
219490_s_at	DCLRE1B	13		N.S		N.S
219492_at	CHIC2	0		N.S	1.14	4.83E-04
219493_at	SHCBP1	0		N.S		N.S
219494_at	RAD54B	10	-1.7	2.53E-23		N.S
219495_s_at	ZNF180	0		N.S		N.S
219496_at	ANKRD57	0		N.S		N.S
219497_s_at	BCL11A	1	-1.9	4.65E-15		N.S
219501_at	ENOX1	0		N.S		N.S
219502_at	NEIL3	7	-1.3	4.46E-12		N.S
219504_s_at	RPAP2	0		N.S	1.46	3.24E-07
219506_at	C1orf54	0		N.S		N.S
219507_at	RSRC1	0		N.S		N.S
219512_at	DSN1	0		N.S	1.20	1.71E-05
219515_at	PRDM10	0		N.S		N.S
219517_at	ELL3	0	-1.1	2.72E-03	-1.29	1.04E-07
219520_s_at	WWC3	0		N.S		N.S
219526_at	C14orf169	0	1.2	6.04E-11		N.S
219530_at	PALB2	20		N.S	1.27	5.97E-12
219531_at	CEP72	0	-1.2	7.05E-03	-1.26	1.28E-06
219538_at	WDR5B	0	-1.3	1.14E-07	1.29	8.68E-13
219539_at	GEMIN6	0		N.S		N.S
219540_at	ZNF267	0		N.S		N.S
219543_at	PBLD	0		N.S	1.61	1.06E-25
219544_at	C13orf34	1	-1.4	2.46E-24	-1.34	1.61E-19
219548_at	ZNF16	0	1.1	3.83E-03		N.S
219549_s_at	RTN3	0		N.S		N.S
219551_at	EAF2	2		N.S	-1.18	2.21E-07

219557_s_at	NRIP3	0		N.S		N.S
219559_at	SLC17A9	0		N.S		N.S
219563_at	C14orf139	0	-1.2	2.93E-08	1.20	4.17E-15
219565_at	CYP20A1	0	1.4	2.06E-11	1.35	2.88E-10
219567_s_at	DEM1	1		N.S		N.S
219570_at	KIF16B	0		N.S		N.S
219571_s_at	ZNF12	0		N.S		N.S
219575_s_at	COG8	0	1.1	1.25E-03		N.S
219577_s_at	ABCA7	0		N.S		N.S
219581_at	TSEN2	0	-1.2	1.34E-06	-1.29	1.53E-11
219582_at	OGFRL1	0		N.S		N.S
219583_s_at	SPATA7	0	-1.4	1.01E-08	1.41	7.39E-18
219584_at	PLA1A	0		N.S	1.31	4.29E-08
219588_s_at	NCAPG2	0		N.S		N.S
219593_at	SLC15A3	0		N.S		N.S
219594_at	NINJ2	0		N.S		N.S
219596_at	THAP10	0	-1.2	5.10E-06	1.36	1.13E-11
219598_s_at	RWDD1	0		N.S		N.S
219600_s_at	TMEM50B	0	-1.1	2.15E-05		N.S
219602_s_at	FAM38B	0		N.S	-1.30	3.19E-02
219603_s_at	ZNF226	0		N.S		N.S
219613_s_at	SIRT6	5		N.S		N.S
219617_at	C2orf34	0		N.S		N.S
219618_at	IRAK4	0		N.S		N.S
219624_at	BAG4	0	1.2	6.76E-04		N.S
219627_at	ZNF767	0		N.S	1.41	4.74E-17
219628_at	ZMAT3	0	1.3	2.09E-12	1.51	3.87E-25
219629_at	FAM118A	0		N.S		N.S
219633_at	TTPAL	0		N.S	1.17	1.38E-05
219634_at	CHST11	0		N.S		N.S
219635_at	ZNF606	0		N.S		N.S
219636_s_at	ARMC9	0		N.S		N.S
219644_at	CCDC41	0	-1.3	8.22E-13	1.21	2.31E-05
219646_at	DEF8	0		N.S		N.S
219648_at	MREG	0		N.S	1.10	7.30E-03
219649_at	ALG6	0	-1.1	4.97E-09		N.S
219650_at	ERCC6L	0		N.S		N.S
219653_at	LSM14B	0		N.S		N.S
219662_at	C2orf49	0	-1.2	3.37E-09		N.S
219667_s_at	BANK1	0		N.S	-1.38	1.13E-06
219673_at	MCM9	0	1.3	4.78E-11	1.20	2.67E-07
219675_s_at	UXS1	0		N.S	1.14	5.53E-08
219676_at	ZSCAN16	0		N.S	1.41	1.28E-13
219680_at	NLRX1	0		N.S	1.30	4.05E-12
219681_s_at	RAB11FIP1	0	1.6	7.62E-21	-1.40	4.75E-14
219683_at	FZD3	0	-1.4	4.60E-08	-1.19	2.44E-02
219688_at	BBS7	0		N.S	1.38	9.29E-12
219690_at	TMEM149	0		N.S		N.S
219691_at	SAMD9	0		N.S	-1.28	7.08E-06
219696_at	DENND1B	0		N.S		N.S
219698_s_at	METTL4	0	-1.2	1.45E-10		N.S
219703_at	MNS1	0		N.S		N.S
219706_at	C20orf29	0		N.S		N.S
219711_at	ZNF586	0		N.S		N.S
219715_s_at	TDP1	36	-1.1	7.99E-10	-1.21	2.23E-19
219717_at	C4orf30	0		N.S		N.S
219720_s_at	C14orf118	0		N.S		N.S
219724_s_at	KIAA0748	0		N.S		N.S
219731_at	FLJ34077	0	-1.1	4.45E-05		N.S
219740_at	VASH2	0	-1.2	3.52E-04		N.S

219751_at	SETD6	0		N.S	-1.11	2.22E-02
219753_at	STAG3	1		N.S		N.S
219754_at	RBM41	0		N.S	1.34	1.23E-08
219757_s_at	C14orf101	0	-1.2	2.40E-04		N.S
219759_at	ERAP2	0		N.S		N.S
219762_s_at	RPL36	1		N.S		N.S
219763_at	DENND1A	0		N.S		N.S
219765_at	ZNF329	0		N.S	1.26	2.29E-04
219767_s_at	CRYZL1	0		N.S	1.14	3.26E-06
219770_at	GTDC1	0	-1.2	2.22E-07		N.S
219774_at	CCDC93	0		N.S		N.S
219777_at	GIMAP6	0	-1.4	1.43E-13		N.S
219785_s_at	FBXO31	3		N.S	1.14	4.63E-02
219787_s_at	ECT2	6		N.S	-1.40	5.11E-13
219793_at	SNX16	0		N.S		N.S
219798_s_at	MEPCE	0	1.2	8.97E-06		N.S
219800_s_at	THNSL1	0	-1.4	3.05E-03		N.S
219801_at	ZNF34	0		N.S		N.S
219805_at	CXorf56	0		N.S		N.S
219806_s_at	C11orf75	0	-1.2	1.54E-03	-1.49	2.46E-21
219809_at	WDR55	0		N.S		N.S
219812_at	PVRIG	0	-1.1	9.09E-03	-1.98	1.17E-25
219816_s_at	RBM23	0		N.S	1.10	2.41E-02
219818_s_at	GPATCH1	0		N.S		N.S
219819_s_at	MRPS28	0		N.S		N.S
219821_s_at	GFOD1	0	-1.3	3.05E-16	-1.49	2.12E-26
219822_at	MTRF1	2	-1.3	3.21E-13		N.S
219833_s_at	EFHC1	0		N.S		N.S
219834_at	ALS2CR8	1	-1.4	3.86E-05		N.S
219841_at	AICDA	36		N.S	-1.19	5.92E-07
219843_at	IPP	4		N.S	1.36	1.43E-09
219848_s_at	ZNF432	0		N.S		N.S
219849_at	ZNF671	0		N.S		N.S
219854_at	ZNF14	0		N.S	1.17	1.34E-02
219858_s_at	MFSD6	0		N.S		N.S
219860_at	LY6G5C	0		N.S		N.S
219861_at	DNAJC17	0		N.S		N.S
219862_s_at	NARF	0		N.S	-1.30	5.52E-14
219863_at	HERC5	1		N.S	1.17	7.74E-04
219870_at	ATF7IP2	0	-1.7	1.37E-14		N.S
219874_at	SLC12A8	0		N.S	-1.34	2.78E-13
219878_s_at	KLF13	1		N.S		N.S
219885_at	SLFN12	0		N.S		N.S
219889_at	FRAT1	0		N.S		N.S
219891_at	PGPEP1	0		N.S		N.S
219892_at	TM6SF1	0		N.S		N.S
219901_at	FGD6	0		N.S		N.S
219904_at	ZSCAN5A	0		N.S		N.S
219910_at	FICD	0	1.1	2.45E-04		N.S
219913_s_at	CRNKL1	0		N.S		N.S
219915_s_at	SLC16A10	0		N.S		N.S
219917_at	ZCCHC4	0	-1.2	3.95E-05		N.S
219918_s_at	ASPM	5	-1.3	6.63E-12	-1.86	2.98E-23
219920_s_at	GMPPB	0		N.S		N.S
219923_at	TRIM45	0		N.S		N.S
219924_s_at	ZMYM6	0	-1.2	8.62E-07		N.S
219929_s_at	ZFYVE21	0	-1.2	1.77E-04		N.S
219931_s_at	KLHL12	0		N.S	1.22	2.23E-03
219933_at	GLRX2	0		N.S		N.S
219938_s_at	PSTPIP2	0		N.S	2.55	3.20E-41

Supplemental Table 2

219939_s_at	CSDE1	1		N.S		N.S
219940_s_at	PCID2	0		N.S	-1.11	8.05E-07
219941_at	TMEM19	0		N.S		N.S
219951_s_at	C20orf12	0		N.S		N.S
219956_at	GALNT6	0		N.S		N.S
219959_at	MOCOS	0		N.S		N.S
219960_s_at	UCHL5	0		N.S	-1.12	7.72E-10
219967_at	MRM1	0	-1.2	9.88E-04		N.S
219971_at	IL21R	2		N.S	1.30	3.37E-10
219972_s_at	C14orf135	0	-1.2	2.74E-03		N.S
219976_at	HOOK1	0		N.S		N.S
219979_s_at	C11orf73	0		N.S		N.S
219980_at	C4orf29	0		N.S		N.S
219982_s_at	SERF1A	0		N.S	1.13	3.04E-02
219988_s_at	RNF220	0	1.2	2.90E-07		N.S
219990_at	E2F8	2	1.3	2.15E-07		N.S
219996_at	ASB7	0		N.S		N.S
219997_s_at	COPS7B	0		N.S		N.S
219999_at	MAN2A2	0		N.S		N.S
220007_at	METTL8	0		N.S		N.S
220011_at	C1orf135	0	1.2	4.10E-10		N.S
220012_at	ERO1LB	0	1.2	4.35E-06		N.S
220015_at	CASZ1	0		N.S		N.S
220018_at	CBLL1	0	1.2	3.37E-05		N.S
220019_s_at	ZNF224	0		N.S		N.S
220035_at	NUP210	0		N.S		N.S
220036_s_at	LMBR1L	0		N.S		N.S
220046_s_at	CCNL1	0	1.2	2.51E-09		N.S
220050_at	C9orf9	0		N.S		N.S
220052_s_at	TINF2	5		N.S		N.S
220054_at	IL23A	0		N.S		N.S
220058_at	C17orf39	0		N.S		N.S
220059_at	STAP1	0		N.S	-1.30	1.22E-23
220060_s_at	C12orf48	0		N.S		N.S
220063_at	GSTCD	0		N.S		N.S
220066_at	NOD2	0	-1.4	1.56E-12	-1.22	1.99E-05
220073_s_at	PLEKHG6	0		N.S	1.75	9.71E-15
220079_s_at	USP48	1		N.S	-1.22	2.38E-02
220085_at	HELLS	0		N.S		N.S
220089_at	L2HGDH	0		N.S		N.S
220091_at	SLC2A6	0		N.S		N.S
220094_s_at	CCDC90A	0	1.1	4.85E-02		N.S
220099_s_at	LUC7L2	0	1.1	1.28E-06	1.14	1.91E-08
220103_s_at	MRPS18C	0		N.S		N.S
220104_at	ZC3HAV1	0	1.3	7.40E-16	1.39	3.46E-18
220118_at	ZBTB32	0	-1.5	4.32E-13	-1.60	1.61E-16
220121_at	LINS1	0		N.S		N.S
220122_at	MCTP1	0		N.S	-1.31	2.14E-06
220127_s_at	FBXL12	0	1.1	2.18E-10	1.09	1.49E-07
220132_s_at	CLEC2D	15		N.S	-1.58	1.53E-12
220145_at	MAP9	1		N.S		N.S
220146_at	TLR7	1	-1.1	1.93E-02	-1.43	1.53E-19
220147_s_at	FAM60A	0		N.S	-1.22	6.89E-14
220155_s_at	BRD9	0		N.S		N.S
220158_at	LGALS14	0		N.S		N.S
220159_at	ABCA11P	0	-1.4	8.81E-08	1.26	2.05E-05
220161_s_at	EPB41L4B	0		N.S	1.18	3.65E-02
220167_s_at	LOC729355	0		N.S		N.S
220169_at	TMEM156	0	-1.3	2.97E-07	-1.32	8.27E-06
220172_at	C2orf37	0		N.S		N.S

220175_s_at	CBWD1	0		N.S	1.15	1.13E-02
220176_at	NUBPL	0		N.S		N.S
220183_s_at	NUDT6	1	-1.3	1.31E-10		N.S
220189_s_at	MGAT4B	0		N.S		N.S
220195_at	MBD5	0	-1.3	8.78E-16	-1.22	6.13E-05
220199_s_at	AIDA	7		N.S		N.S
220200_s_at	SETD8	5		N.S		N.S
220201_at	RC3H2	0		N.S		N.S
220215_at	ZNF669	0		N.S		N.S
220230_s_at	CYB5R2	0		N.S		N.S
220235_s_at	C1orf103	0		N.S	1.28	3.37E-13
220236_at	PDPR	0		N.S		N.S
220238_s_at	KLHL7	0		N.S	1.27	7.91E-07
220241_at	TMCO3	0		N.S		N.S
220244_at	LOH3CR2A	0		N.S		N.S
220250_at	ZNF286A	0		N.S		N.S
220251_at	C1orf107	0		N.S		N.S
220255_at	FANCE	16	1.2	1.91E-05		N.S
220261_s_at	ZDHHC4	0		N.S		N.S
220285_at	FAM108B1	0		N.S		N.S
220287_at	ADAMTS9	0		N.S		N.S
220288_at	MYO15A	0		N.S		N.S
220305_at	MAVS	0	-1.3	1.68E-15	1.19	4.19E-07
220319_s_at	MYLIP	0		N.S	-1.16	6.71E-04
220320_at	DOK3	0		N.S		N.S
220329_s_at	RMND1	0		N.S		N.S
220330_s_at	SAMSN1	0		N.S	-1.17	3.53E-05
220346_at	MTHFD2L	0		N.S		N.S
220355_s_at	PBRM1	0		N.S		N.S
220358_at	BATF3	0		N.S	-1.25	4.88E-05
220367_s_at	SAP130	2		N.S		N.S
220368_s_at	SMEK1	0	1.2	9.25E-03	1.42	1.16E-17
220370_s_at	USP36	0	1.3	1.11E-06		N.S
220371_s_at	SLC12A9	0		N.S		N.S
220375_s_at	H2AFY	0		N.S		N.S
220386_s_at	EML4	0		N.S		N.S
220387_s_at	HHLA3	0		N.S		N.S
220390_at	AGBL2	0		N.S		N.S
220391_at	ZBTB3	0		N.S		N.S
220399_at	NCRNA00115	0		N.S		N.S
220417_s_at	THAP4	0		N.S		N.S
220419_s_at	USP25	0		N.S	-1.24	2.72E-11
220446_s_at	CHST4	0		N.S		N.S
220459_at	MCM3APAS	0	-1.5	4.58E-11	-1.22	2.68E-02
220466_at	CCDC15	0	-1.2	5.18E-07	1.36	1.40E-16
220467_at	FLJ21272	0		N.S		N.S
220476_s_at	C1orf183	0	1.6	5.53E-08	2.94	1.41E-26
220477_s_at	C20orf30	0		N.S		N.S
220488_s_at	BCAS3	0		N.S		N.S
220494_s_at	C14orf43	0		N.S	-1.50	9.94E-20
220495_s_at	TXNDC15	0		N.S	1.14	5.69E-08
220500_s_at	RABL2A	0		N.S		N.S
220525_s_at	AUP1	0		N.S		N.S
220547_s_at	FAM35A	0		N.S		N.S
220550_at	FBXO4	1		N.S		N.S
220553_s_at	PRPF39	0		N.S		N.S
220565_at	CCR10	0		N.S	-1.18	3.80E-03
220566_at	PIK3R5	0		N.S		N.S
220572_at	DKFZp547G183	0		N.S		N.S
220577_at	GVIN1	0	-1.4	1.66E-18		N.S

220580_at	BICC1	0		N.S		N.S
220586_at	CHD9	0		N.S		N.S
220587_s_at	GBL	0		N.S		N.S
220588_at	BCAS4	0		N.S	1.22	1.43E-03
220590_at	ITFG2	0		N.S		N.S
220597_s_at	ARL6IP4	0		N.S		N.S
220603_s_at	MCTP2	0	-1.2	2.69E-05	-1.29	1.59E-04
220605_s_at	SIRT2	6		N.S		N.S
220608_s_at	ZNF770	0		N.S		N.S
220623_s_at	TSGA10	0		N.S	1.41	3.18E-05
220643_s_at	FAIM	2		N.S		N.S
220647_s_at	CHCHD8	0		N.S		N.S
220651_s_at	MCM10	6	1.2	1.51E-04		N.S
220658_s_at	ARNTL2	0		N.S		N.S
220661_s_at	ZNF692	0	-1.3	8.23E-11		N.S
220682_s_at	KLHL5	0		N.S		N.S
220688_s_at	MRTO4	0	1.2	2.97E-07	-1.12	1.75E-02
220690_s_at	DHRS7B	0	-1.2	1.92E-11	1.19	2.27E-08
220703_at	C10orf110	0		N.S		N.S
220721_at	ZNF614	0		N.S		N.S
220731_s_at	NECAP2	0		N.S	1.14	2.76E-03
220735_s_at	SENP7	0	-1.3	7.17E-07		N.S
220739_s_at	CNNM3	0		N.S		N.S
220740_s_at	SLC12A6	0		N.S		N.S
220741_s_at	PPA2	1		N.S		N.S
220746_s_at	UIMC1	1		N.S		N.S
220750_s_at	LEPRE1	0		N.S		N.S
220753_s_at	CRYL1	0		N.S		N.S
220755_s_at	C6orf48	0		N.S	1.23	7.07E-06
220757_s_at	UBXN6	0		N.S		N.S
220761_s_at	TAOK3	0		N.S	-1.19	7.12E-08
220768_s_at	CSNK1G3	0		N.S		N.S
220770_s_at	C5orf54	0	-2.1	4.93E-16	1.42	6.87E-15
220773_s_at	GPHN	0		N.S		N.S
220775_s_at	UEVLD	0	-1.2	6.03E-07		N.S
220776_at	KCNJ14	0		N.S		N.S
220788_s_at	IRF9	1		N.S		N.S
220789_s_at	TBRG4	0		N.S		N.S
220797_at	METT10D	0		N.S		N.S
220800_s_at	TMOD3	0		N.S		N.S
220826_at	TCP10L	0		N.S		N.S
220840_s_at	C1orf112	0	-1.1	3.58E-04		N.S
220864_s_at	NDUFA13	0		N.S		N.S
220865_s_at	PDSS1	0		N.S	-1.07	5.73E-03
220885_s_at	CENPJ	2		N.S		N.S
220890_s_at	DDX47	0		N.S		N.S
220892_s_at	PSAT1	0		N.S	1.16	3.49E-03
220924_s_at	SLC38A2	0		N.S		N.S
220925_at	MAK10	0		N.S	1.11	3.45E-04
220926_s_at	EDEM3	0	-1.3	1.89E-04		N.S
220931_at	MGC5590	0		N.S		N.S
220933_s_at	ZCCHC6	0	-1.2	3.38E-09		N.S
220934_s_at	TMEM223	0	-1.2	2.17E-07		N.S
220935_s_at	CDK5RAP2	3		N.S		N.S
220937_s_at	ST6GALNAC4	0		N.S	-1.17	7.63E-03
220939_s_at	DPP8	0		N.S		N.S
220940_at	ANKRD36B	0		N.S		N.S
220941_s_at	C21orf91	0	1.2	1.74E-04	1.13	6.18E-04
220943_s_at	C2orf56	0	-1.3	4.05E-06	-1.26	1.14E-03
220944_at	PGLYRP4	0		N.S		N.S

220946_s_at	SETD2	0		N.S		N.S
220947_s_at	TBC1D10B	0		N.S		N.S
220948_s_at	ATP1A1	0		N.S	1.12	1.04E-08
220949_s_at	C7orf49	0	-1.2	1.54E-16	1.09	3.61E-04
220953_s_at	MTMR12	0	1.2	1.19E-02		N.S
220956_s_at	EGLN2	0		N.S	-1.15	7.83E-04
220957_at	CTAGE1	0		N.S		N.S
220964_s_at	RAB1B	0		N.S		N.S
220980_s_at	ADPGK	0		N.S		N.S
220985_s_at	RNF170	0	-1.2	3.92E-02		N.S
220987_s_at	C11orf17	0		N.S		N.S
220988_s_at	C1QTNF3	0		N.S		N.S
220990_s_at	MIR21	0	-1.2	2.63E-08		N.S
220991_s_at	RNF32	0	-1.2	1.06E-02		N.S
220992_s_at	C1orf25	0	-1.5	1.14E-22		N.S
220993_s_at	GPR63	0		N.S		N.S
221002_s_at	TSPAN14	0		N.S	1.30	3.63E-14
221004_s_at	ITM2C	0		N.S		N.S
221007_s_at	FIP1L1	0		N.S	-1.09	5.94E-03
221011_s_at	LBH	1	-1.1	4.71E-04	-1.26	4.05E-06
221012_s_at	TRIM8	0		N.S		N.S
221014_s_at	RAB33B	0		N.S	1.40	3.23E-16
221020_s_at	SLC25A32	0	1.1	1.96E-03	-1.17	2.17E-07
221021_s_at	CTNNBL1	0		N.S		N.S
221027_s_at	PLA2G12A	0		N.S		N.S
221031_s_at	APOLD1	0	-1.6	4.80E-05		N.S
221036_s_at	APH1B	0		N.S		N.S
221038_at	UTP15	0		N.S		N.S
221039_s_at	ASAP1	0		N.S		N.S
221044_s_at	TRIM34	0		N.S	-1.27	1.27E-14
221046_s_at	GTPBP8	0		N.S		N.S
221059_s_at	COTL1	0		N.S		N.S
221069_s_at	CCDC44	0		N.S	1.19	2.73E-16
221079_s_at	METTL2A	0		N.S		N.S
221080_s_at	DENND1C	0		N.S		N.S
221081_s_at	DENND2D	0	-1.4	3.46E-20		N.S
221087_s_at	APOL3	0	-1.1	1.72E-02	-1.29	3.54E-08
221090_s_at	OGFOD1	0		N.S	1.11	2.80E-03
221094_s_at	ELP3	2	-1.1	1.13E-05		N.S
221096_s_at	TMCO6	0	-1.2	3.26E-06	-1.11	2.17E-02
221103_s_at	WDR52	0	-1.3	1.49E-09		N.S
221104_s_at	NIPSNAP3B	0		N.S		N.S
221139_s_at	CSAD	0		N.S		N.S
221142_s_at	PECR	0		N.S		N.S
221188_s_at	CIDEB	0		N.S		N.S
221190_s_at	C18orf8	0	-1.1	4.61E-08		N.S
221193_s_at	ZCCHC10	0		N.S	1.16	3.47E-04
221194_s_at	RNFT1	0		N.S	1.19	2.62E-05
221203_s_at	YEATS2	0	-1.6	1.01E-25	-1.18	1.12E-04
221207_s_at	NBEA	0		N.S	-1.42	1.69E-05
221208_s_at	C11orf61	0	-1.2	5.33E-04	1.22	3.92E-06
221210_s_at	NPL	6		N.S		N.S
221211_s_at	C21orf7	0		N.S		N.S
221213_s_at	ZNF280D	0	-1.6	5.92E-10		N.S
221214_s_at	NELF	0		N.S		N.S
221216_s_at	SCMH1	0		N.S		N.S
221218_s_at	TPK1	1		N.S	-1.18	4.76E-09
221219_s_at	KLHDC4	0		N.S		N.S
221221_s_at	KLHL3	0		N.S		N.S
221222_s_at	C1orf56	0		N.S	1.32	6.92E-08

Supplemental Table 2

221229_s_at	TRMT61B	0		N.S		N.S
221230_s_at	ARID4B	1	-1.2	2.44E-05		N.S
221235_s_at	LOC644617	0	1.1	1.77E-02	1.21	3.57E-07
221238_at	NSBP1	0		N.S		N.S
221244_s_at	PDPK1	0		N.S		N.S
221245_s_at	FZD5	0		N.S	1.31	1.18E-08
221247_s_at	WBSCR16	0		N.S		N.S
221249_s_at	FAM117A	0		N.S		N.S
221253_s_at	TXNDC5	0		N.S	-1.24	2.29E-08
221255_s_at	TMEM93	0		N.S		N.S
221256_s_at	HDHD3	0	-1.2	8.51E-06		N.S
221258_s_at	KIF18A	0	-1.4	3.43E-19	-1.30	4.73E-13
221260_s_at	CSRNP2	0		N.S	1.30	2.87E-16
221263_s_at	SF3B5	0		N.S		N.S
221264_s_at	LOC100128223	0		N.S	-1.15	5.84E-03
221265_s_at	C15orf44	0	-1.1	3.44E-02	1.10	3.15E-04
221267_s_at	FAM108A1	0		N.S	-1.10	1.76E-02
221268_s_at	SGPP1	0		N.S	1.20	4.63E-05
221269_s_at	SH3BGRL3	0		N.S		N.S
221277_s_at	PUS3	0		N.S	1.12	3.84E-07
221286_s_at	MGC29506	0		N.S	-1.33	1.07E-06
221381_s_at	MORF4	2		N.S		N.S
221423_s_at	YIPF5	0		N.S	1.19	9.82E-11
221425_s_at	ISCA1	0	1.1	8.24E-03		N.S
221428_s_at	TBL1XR1	0		N.S		N.S
221430_s_at	RNF146	0	-1.2	3.01E-03	1.21	4.92E-04
221434_s_at	C14orf156	0		N.S		N.S
221436_s_at	CDCA3	0	-1.2	1.27E-03	-1.47	1.62E-15
221437_s_at	MRPS15	0		N.S		N.S
221449_s_at	ITFG1	0		N.S	1.18	1.07E-04
221452_s_at	TMEM14B	0		N.S		N.S
221464_at	OR1D2	0		N.S		N.S
221466_at	P2RY4	0		N.S		N.S
221471_at	SERINC3	0	-1.1	2.60E-03		N.S
221474_at	MYL12B	0		N.S		N.S
221475_s_at	RPL15	0		N.S		N.S
221479_s_at	BNIP3L	2		N.S	-1.24	4.01E-07
221483_s_at	ARPP19	0	1.1	1.01E-02	1.18	4.32E-15
221484_at	B4GALT5	0		N.S	-1.22	3.25E-11
221486_at	ENSA	1	-1.2	2.36E-09	-1.20	4.74E-07
221488_s_at	CUTA	0		N.S		N.S
221492_s_at	ATG3	0		N.S		N.S
221493_at	TSPYL1	0		N.S		N.S
221495_s_at	TCF25	0		N.S		N.S
221502_at	KPNA3	0		N.S	-1.11	1.35E-03
221504_s_at	ATP6V1H	0		N.S		N.S
221505_at	ANP32E	0		N.S	-1.09	5.73E-03
221506_s_at	TNPO2	0		N.S		N.S
221509_at	DENR	0		N.S		N.S
221513_s_at	UTP14A	0		N.S		N.S
221515_s_at	LCMT1	0		N.S		N.S
221517_s_at	MED17	1		N.S		N.S
221518_s_at	USP47	1		N.S		N.S
221520_s_at	CDCA8	0	-1.2	1.50E-06	-1.34	3.20E-16
221521_s_at	GINS2	0		N.S	-1.13	1.39E-03
221522_at	ANKRD27	0	-1.2	6.11E-14	-1.24	2.99E-11
221524_s_at	RRAGD	0		N.S	-1.27	8.04E-05
221532_s_at	WDR61	0		N.S		N.S
221534_at	C11orf68	0		N.S		N.S
221535_at	LSG1	0		N.S		N.S

221539_at	EIF4EBP1	10		N.S		N.S
221542_s_at	ERLIN2	0		N.S		N.S
221548_s_at	ILKAP	0		N.S		N.S
221549_at	GRWD1	1		N.S	-1.22	1.40E-04
221550_at	COX15	0		N.S	1.27	1.70E-10
221553_at	MAGT1	0		N.S		N.S
221558_s_at	LEF1	1		N.S		N.S
221559_s_at	MIS12	1	1.2	2.05E-08		N.S
221561_at	SOAT1	0		N.S		N.S
221568_s_at	LIN7C	0		N.S		N.S
221569_at	AHI1	0	-1.2	1.35E-07		N.S
221570_s_at	METTL5	0		N.S		N.S
221571_at	TRAF3	0		N.S		N.S
221575_at	SCLY	0	-1.1	2.99E-03	-1.14	3.49E-08
221580_s_at	TAF1D	0		N.S		N.S
221582_at	HIST3H2A	0		N.S		N.S
221586_s_at	E2F5	8	-1.3	1.83E-14	-1.15	1.70E-02
221587_s_at	C19orf24	0		N.S		N.S
221591_s_at	FAM64A	0		N.S	-1.44	2.21E-20
221593_s_at	RPL31	0		N.S		N.S
221594_at	C7orf64	0		N.S		N.S
221595_at	DKFZP564O0523	0	1.4	4.65E-05		N.S
221597_s_at	TMEM208	0		N.S		N.S
221600_s_at	C11orf67	0		N.S		N.S
221602_s_at	FAIM3	0		N.S		N.S
221610_s_at	STAP2	0		N.S		N.S
221614_s_at	RPH3AL	0		N.S		N.S
221616_s_at	TAF9B	0		N.S		N.S
221619_s_at	MTCH1	1	1.1	4.35E-02	1.09	2.58E-05
221620_s_at	APOO	1		N.S		N.S
221621_at	C17orf86	0		N.S	-1.16	3.16E-03
221622_s_at	TMEM126B	0		N.S	-1.15	9.76E-05
221626_at	ZNF506	0		N.S	1.21	2.67E-03
221632_s_at	WDR4	0		N.S		N.S
221634_at	RPL23AP7	0		N.S	1.40	5.25E-10
221637_s_at	C11orf48	0		N.S		N.S
221638_s_at	STX16	0		N.S		N.S
221640_s_at	LRDD	17		N.S	2.35	2.52E-21
221641_s_at	ACOT9	0		N.S		N.S
221643_s_at	RERE	0		N.S		N.S
221645_s_at	ZNF83	0		N.S		N.S
221647_s_at	RIC8A	0		N.S		N.S
221649_s_at	PPAN	0	-1.2	1.76E-08	-1.32	5.12E-18
221650_s_at	MED18	0		N.S		N.S
221652_s_at	C12orf11	0	-1.2	3.23E-18	-1.33	3.07E-21
221657_s_at	ASB6	0		N.S		N.S
221666_s_at	PYCARD	1		N.S		N.S
221669_s_at	ACAD8	0		N.S	1.17	4.28E-05
221673_s_at	CSNK1G1	0		N.S	1.41	3.80E-16
221675_s_at	CHPT1	0		N.S	-1.12	2.36E-02
221676_s_at	CORO1C	0		N.S		N.S
221677_s_at	DONSON	2	1.1	7.22E-03	1.11	1.30E-04
221680_s_at	ETV7	0		N.S	1.22	1.04E-02
221683_s_at	CEP290	0		N.S		N.S
221685_s_at	CCDC99	0	-1.2	1.49E-13		N.S
221688_s_at	IMP3	0		N.S		N.S
221689_s_at	PIGP	0		N.S		N.S
221692_s_at	MRPL34	0		N.S		N.S
221699_s_at	DDX50	0		N.S		N.S
221700_s_at	UBA52	0		N.S		N.S

221702_s_at	TM2D3	0		N.S		N.S
221703_at	BRIP1	25		N.S	1.37	5.40E-08
221704_s_at	VPS37B	0		N.S	1.45	6.11E-13
221708_s_at	UNC45A	0		N.S		N.S
221711_s_at	C19orf62	4		N.S		N.S
221712_s_at	WDR74	0		N.S		N.S
221725_at	WASF2	0		N.S	1.11	6.44E-03
221727_at	SUB1	2	1.3	6.63E-11	-1.29	7.98E-08
221737_at	GNA12	0		N.S		N.S
221738_at	KIAA1219	0		N.S	1.39	2.48E-14
221739_at	C19orf10	0		N.S		N.S
221741_s_at	YTHDF1	0		N.S		N.S
221744_at	WDR68	0	-1.2	7.20E-04		N.S
221746_at	UBL4A	0		N.S		N.S
221749_at	YTHDF3	0		N.S		N.S
221750_at	HMGCS1	0	-1.3	2.85E-11	-1.39	4.45E-14
221751_at	SLC2A3P1	0		N.S	-1.16	5.10E-05
221753_at	SSH1	0		N.S		N.S
221758_at	ARMC6	0		N.S		N.S
221760_at	MAN1A1	0		N.S		N.S
221761_at	ADSS	0	-1.1	3.47E-05	-1.14	9.39E-03
221763_at	JMJD1C	0		N.S	-1.27	7.23E-11
221766_s_at	FAM46A	0	1.5	1.01E-13	1.84	9.17E-20
221770_at	RPE	52	-1.4	2.45E-11		N.S
221771_s_at	MPHOSPH8	0	-1.1	8.68E-04		N.S
221776_s_at	BRD7	1		N.S		N.S
221780_s_at	DDX27	0		N.S		N.S
221782_at	DNAJC10	0		N.S		N.S
221786_at	C6orf120	0		N.S		N.S
221791_s_at	CCDC72	0		N.S		N.S
221800_s_at	C17orf70	0		N.S		N.S
221803_s_at	NRBF2	0	1.2	4.64E-12		N.S
221804_s_at	FAM45A	0		N.S		N.S
221806_s_at	SETD5	0		N.S		N.S
221808_at	RAB9A	0		N.S		N.S
221813_at	FBXO42	0	-1.2	7.60E-07		N.S
221816_s_at	PHF11	0		N.S		N.S
221817_at	DOLPP1	0		N.S		N.S
221821_s_at	C12orf41	0	1.3	3.01E-22	1.18	2.85E-15
221823_at	C5orf30	0		N.S	-1.50	4.79E-25
221824_s_at	MARCH8	0		N.S	1.29	4.86E-10
221825_at	ANGEL2	0		N.S	1.29	7.69E-17
221829_s_at	TNPO1	0		N.S	-1.13	7.44E-04
221840_at	PTPRE	0		N.S	1.40	7.84E-17
221845_s_at	CLPB	1		N.S		N.S
221847_at	LOC100129361	0		N.S		N.S
221853_s_at	NOMO1	0		N.S		N.S
221858_at	TBC1D12	0		N.S		N.S
221864_at	ORAI3	0		N.S	1.86	2.37E-28
221865_at	C9orf91	0		N.S	-1.11	4.98E-03
221871_s_at	TFG	1		N.S		N.S
221873_at	ZNF143	1		N.S	-1.27	7.06E-13
221881_s_at	CLIC4	2		N.S		N.S
221882_s_at	TMEM8	0		N.S		N.S
221896_s_at	HIGD1A	0		N.S	1.16	3.12E-11
221897_at	TRIM52	0	-1.2	6.18E-05		N.S
221904_at	FAM131A	0	-1.3	2.56E-13		N.S
221905_at	CYLD	0		N.S		N.S
221912_s_at	CCDC28B	0		N.S		N.S
221915_s_at	RANBP1	1		N.S		N.S

221918_at	PCTK2	0		N.S		N.S
221922_at	GPSM2	0	-1.4	1.61E-13	-1.25	3.66E-04
221925_s_at	CSPP1	0		N.S		N.S
221927_s_at	ABHD11	0		N.S		N.S
221931_s_at	SEH1L	0		N.S		N.S
221932_s_at	GLRX5	0		N.S		N.S
221935_s_at	C3orf64	0	1.2	2.60E-03		N.S
221940_at	RPUSD2	0		N.S		N.S
221957_at	PDK3	0		N.S		N.S
221960_s_at	RAB2A	0		N.S		N.S
221962_s_at	UBE2H	0		N.S		N.S
221965_at	MPHOSPH9	0		N.S		N.S
221969_at	PAX5	2		N.S		N.S
221970_s_at	NOL11	0		N.S		N.S
221972_s_at	SDF4	0		N.S		N.S
221978_at	HLA-F	0		N.S		N.S
221983_at	FAM134A	0		N.S		N.S
221985_at	KLHL24	0		N.S		N.S
221987_s_at	TSR1	0		N.S	-1.22	1.30E-06
221992_at	PDXDC2	0		N.S		N.S
221995_s_at	MRP63	0		N.S		N.S
221997_s_at	MRPL52	0		N.S		N.S
222000_at	C1orf174	0	-1.1	2.21E-04	-1.17	1.96E-10
222006_at	LETM1	0		N.S		N.S
222010_at	TCP1	0		N.S	-1.11	5.97E-12
222019_at	PFDN6	0		N.S		N.S
222027_at	NUCKS1	0		N.S		N.S
222028_at	ZNF45	0		N.S	1.11	1.06E-03
222030_at	SIVA1	2		N.S		N.S
222031_at	LOC286434	0		N.S		N.S
222034_at	GNB2L1	3		N.S	-1.29	1.35E-07
222038_s_at	UTP18	0		N.S		N.S
222039_at	KIF18B	0	-1.2	8.51E-09	-1.14	4.34E-04
222040_at	HNRNPA1	0	1.3	8.64E-07		N.S
222051_s_at	LOC100131637	0		N.S		N.S
222052_at	C19orf54	0		N.S		N.S
222064_s_at	AARSD1	0		N.S		N.S
222077_s_at	RACGAP1	0	-1.2	1.43E-10	-1.26	7.26E-10
222088_s_at	SLC2A14	0	-1.9	6.74E-26	-1.75	5.80E-22
222103_at	ATF1	7	1.1	8.18E-04		N.S
222105_s_at	NKIRAS2	0		N.S	1.16	2.93E-03
222118_at	CENPN	0	1.3	4.36E-15		N.S
222119_s_at	FBXO11	0	-1.1	2.80E-04		N.S
222125_s_at	P4HTM	0		N.S	1.11	1.29E-02
222127_s_at	EXOC1	0	-1.2	1.51E-09	1.12	1.75E-06
222128_at	NSUN6	0		N.S	1.26	7.91E-03
222130_s_at	FTSJ2	0	1.2	2.44E-09	1.47	4.66E-27
222139_at	KIAA1466	0	-1.3	4.13E-05		N.S
222140_s_at	GPR89A	0		N.S		N.S
222143_s_at	MTMR14	0		N.S		N.S
222147_s_at	ACTR5	0		N.S		N.S
222148_s_at	RHOT1	0	-1.3	1.59E-21		N.S
222150_s_at	PION	9		N.S		N.S
222154_s_at	LOC26010	0		N.S	-1.16	5.94E-06
222155_s_at	GPR172A	0		N.S		N.S
222175_s_at	MED15	0		N.S		N.S
222193_at	C2orf43	0	-1.2	6.27E-05		N.S
222199_s_at	BIN3	1		N.S		N.S
222200_s_at	BSDC1	0		N.S	1.32	8.04E-10
222201_s_at	CASP8AP2	1		N.S		N.S

222203_s_at	RDH14	0		N.S		N.S
222204_s_at	RRN3	0		N.S		N.S
222208_s_at	POLR2J4	0		N.S		N.S
222209_s_at	TMEM135	0		N.S		N.S
222212_s_at	LASS2	1		N.S		N.S
222216_s_at	MRPL17	0		N.S	-1.27	5.77E-06
222228_s_at	ALKBH4	0		N.S		N.S
222230_s_at	ACTR10	0	-1.1	1.93E-03	1.27	1.50E-20
222231_s_at	LRRC59	0	1.1	5.88E-06		N.S
222233_s_at	DCLRE1C	42		N.S		N.S
222235_s_at	CSGALNACT2	0	1.2	2.04E-04		N.S
222239_s_at	INTS6	0	-1.3	4.42E-06		N.S
222243_s_at	TOB2	1		N.S	1.14	2.28E-04
222244_s_at	TUG1	0	-1.2	2.82E-04		N.S
222250_s_at	INTS7	0		N.S		N.S
222262_s_at	ETNK1	0	-1.2	4.22E-05	1.15	5.13E-03
222263_at	SLC35E1	0		N.S		N.S
222264_at	HNRNPUL2	0		N.S		N.S
222267_at	TMEM209	0		N.S		N.S
222270_at	SMEK2	0	1.6	1.99E-11		N.S
222273_at	PAPOLG	0		N.S		N.S
222275_at	MRPS30	1	-1.3	1.23E-13	-1.19	1.37E-07
222279_at	RP3-377H14.5	0		N.S		N.S
222305_at	HK2	0		N.S		N.S
222310_at	SFRS15	0		N.S		N.S
222316_at	USO1	0		N.S		N.S
222326_at	PDE4B	0		N.S	1.55	4.72E-07
222344_at	C5orf13	0		N.S	-1.29	1.96E-03
222360_at	DPH5	0		N.S		N.S
222366_at	ADNP	1		N.S		N.S
222369_at	NAT11	0	-1.3	3.86E-13	1.18	2.81E-05
222376_at	HACE1	0	-1.2	7.18E-03		N.S
266_s_at	CD24	8		N.S	-1.24	1.42E-02
31845_at	ELF4	4		N.S		N.S
32032_at	DGCR14	0		N.S		N.S
32091_at	SLC25A44	0		N.S		N.S
32811_at	MYO1C	0		N.S		N.S
32836_at	AGPAT1	0		N.S		N.S
33322_i_at	SFN	49		N.S		N.S
33494_at	ETFDH	0		N.S		N.S
33760_at	PEX14	0	-1.1	6.99E-06		N.S
33814_at	PAK4	0		N.S		N.S
33850_at	MAP4	7		N.S		N.S
34210_at	CD52	0		N.S		N.S
34221_at	HMGXB3	0		N.S		N.S
34260_at	TELO2	0		N.S		N.S
34408_at	RTN2	0		N.S		N.S
34858_at	KCTD2	0		N.S		N.S
34868_at	SMG5	1		N.S		N.S
35160_at	LDB1	0		N.S	1.34	1.88E-13
35265_at	FXR2	0		N.S		N.S
35626_at	SGSH	0		N.S		N.S
35666_at	SEMA3F	0		N.S		N.S
35671_at	GTF3C1	0		N.S		N.S
36019_at	STK19	0	1.1	2.62E-02	1.13	2.74E-10
36030_at	IFFO1	0	-1.1	4.10E-06	1.15	9.47E-10
36084_at	CUL7	2		N.S		N.S
36475_at	GCAT	5		N.S		N.S
36545_s_at	SFI1	0		N.S	-1.33	2.18E-12
36552_at	C2CD3	0	-1.1	1.52E-03		N.S

Supplemental Table 2

36711_at	MAFF	2	1.4	4.19E-07	-1.80	3.64E-21
36829_at	PER1	15		N.S		N.S
36865_at	ANGEL1	0		N.S	-1.12	2.13E-02
36907_at	MVK	2		N.S		N.S
36920_at	MTM1	0		N.S		N.S
36994_at	ATP6V0C	0		N.S		N.S
37012_at	CAPZB	0		N.S		N.S
37028_at	PPP1R15A	63	1.5	3.37E-13		N.S
37079_at	YDD19	0		N.S		N.S
37170_at	BMP2K	0		N.S		N.S
37226_at	BNIP1	1	1.2	3.71E-11		N.S
37232_at	KIAA0586	0		N.S		N.S
37254_at	ZNF133	0	-1.2	2.50E-08	1.15	1.33E-06
37278_at	TAZ	2		N.S		N.S
37462_i_at	SF3A2	0		N.S		N.S
37512_at	HSD17B6	0		N.S		N.S
37802_r_at	FAM63B	0		N.S		N.S
37860_at	ZNF337	0		N.S	1.45	7.88E-21
37872_at	JRK	0		N.S		N.S
37966_at	PARVB	0		N.S		N.S
38290_at	RGS14	0	-1.2	4.33E-09	-1.23	2.91E-13
38398_at	MADD	1		N.S		N.S
38447_at	ADRBK1	0		N.S		N.S
38892_at	KIAA0240	0		N.S		N.S
39248_at	AQP3	0	1.3	2.57E-04	1.34	4.10E-05
39318_at	TCL1A	1		N.S	-1.67	4.17E-13
39729_at	PRDX2	1		N.S		N.S
39817_s_at	C6orf108	0		N.S		N.S
40149_at	SH2B1	0		N.S		N.S
40255_at	DDX28	0		N.S		N.S
40359_at	RASSF7	0	-1.3	1.46E-11		N.S
40420_at	STK10	0		N.S	1.24	1.16E-05
40446_at	PHF1	2	1.1	6.42E-04	1.22	1.37E-15
40465_at	DDX23	0		N.S		N.S
41160_at	MBD3	0		N.S		N.S
41220_at	SEPT9	1		N.S	-1.12	2.15E-02
41387_r_at	KDM6B	0	1.2	2.29E-04	-1.22	3.19E-08
41512_at	BRAP	6		N.S	1.12	6.35E-03
41577_at	PPP1R16B	0		N.S	-1.38	1.13E-14
41858_at	FRAG1	2		N.S		N.S
43544_at	MED16	0		N.S		N.S
43977_at	TMEM161A	0		N.S	-1.12	2.28E-06
44065_at	C12orf52	0		N.S	-1.22	1.83E-12
44669_at	LOC644096	0		N.S	1.13	1.61E-04
44702_at	SYDE1	0		N.S		N.S
44783_s_at	HEY1	1	-1.2	1.41E-12	-1.52	8.49E-26
45687_at	PRR14	0		N.S	1.26	4.42E-09
46167_at	TTC4	0		N.S		N.S
46270_at	UBAP1	0		N.S		N.S
46665_at	SEMA4C	0		N.S		N.S
47083_at	C7orf26	0		N.S		N.S
47571_at	ZNF236	0		N.S		N.S
47608_at	TJAP1	0		N.S	1.17	4.18E-10
48531_at	TNIP2	1	1.2	9.57E-20	1.16	7.63E-09
48659_at	MIIP	0		N.S		N.S
49306_at	RASSF4	0	-1.2	1.96E-07		N.S
49327_at	SIRT3	2		N.S		N.S
49485_at	PRDM4	0		N.S		N.S
50221_at	TFEB	0		N.S		N.S
50277_at	GGA1	0		N.S		N.S

Supplemental Table 2

50374_at	C17orf90	0	-1.2	2.51E-08		N.S
51158_at	FAM174B	0		N.S		N.S
51774_s_at	LOC222070	0		N.S		N.S
52078_at	TMEM222	0	1.2	1.65E-06	1.16	4.13E-02
52164_at	C11orf24	0		N.S	1.62	7.76E-41
52169_at	STRADA	2		N.S	1.27	1.48E-17
52741_at	TRMT61A	0		N.S		N.S
53076_at	B4GALT7	0		N.S		N.S
53912_at	SNX11	0		N.S		N.S
53987_at	RANBP10	0		N.S	1.33	2.71E-14
54037_at	HPS4	0		N.S		N.S
54051_at	PKNOX1	0		N.S		N.S
54970_at	ZMIZ2	0		N.S		N.S
55065_at	MARK4	0		N.S	1.25	1.81E-04
55081_at	MICALL1	0		N.S	1.68	1.66E-25
55662_at	C10orf76	0		N.S		N.S
55692_at	ELMO2	0		N.S		N.S
55705_at	C19orf22	0		N.S		N.S
55872_at	ZNF512B	0		N.S		N.S
56256_at	SIDT2	0		N.S		N.S
56919_at	WDR48	0		N.S		N.S
57082_at	LDLRAP1	0		N.S		N.S
57163_at	ELOVL1	0		N.S		N.S
57539_at	ZGPAT	0		N.S		N.S
58308_at	TRIM62	0		N.S		N.S
58696_at	EXOSC4	0	-1.1	1.59E-02		N.S
60528_at	JMJD7-PLA2G4B	2		N.S		N.S
63009_at	SHQ1	0		N.S	-1.14	3.81E-02
632_at	GSK3A	0		N.S		N.S
635_s_at	PPP2R5B	1		N.S		N.S
64371_at	SFRS14	0	-1.2	1.57E-02		N.S
64486_at	CORO1B	0		N.S		N.S
64488_at	IRGQ	0		N.S	1.43	4.01E-22
64883_at	MOSPD2	0	-1.2	6.07E-06		N.S
64900_at	FLJ22167	0		N.S		N.S
65133_i_at	INO80B	0		N.S		N.S
65585_at	FAM86B1	0		N.S		N.S
65588_at	LOC388796	0	1.3	5.73E-15		N.S
65630_at	TMEM80	0		N.S		N.S
65770_at	RHOT2	0		N.S		N.S
78383_at	LOC100129250	0		N.S		N.S
89948_at	PCIF1	0	1.4	1.62E-14		N.S
90610_at	LRCH4	0		N.S		N.S
91703_at	EHBP1L1	0		N.S		N.S
91816_f_at	MEX3D	0		N.S		N.S

Supplemental Table 3. Properties of coexpression networks using different correlation thresholds

Correlation Threshold	No. of connections	No. of genes	Max connec.	Average no. of connections	Clustering Coefficient	Scale free topology criterion	Gamma
DMSO treatment							
0.1	14,792,155	6775	5371	4366.7	N.D.	0.36	1.93
0.2	8,352,322	6775	3974	2465.6	N.D.	0.16	0.41
0.3	4,164,502	6775	2755	1229.4	N.D.	0.02	0.07
0.4	1,828,730	6636	1886	551.15	0.508	0.52	0.55
0.5	688,693	5939	1186	231.92	0.534	0.73	0.81
0.6	212,298	4354	662	97.519	0.549	0.80	0.97
0.7	51,316	2476	322	41.451	0.569	0.84	1.04
0.8	10,044	894	140	22.47	0.629	0.61	0.87
0.9	1,634	194	53	16.845	0.701	0.39	0.55
Tunicamycin treatment							
0.1	14,583,140	6775	5364	4305	N.D.	0.33	1.77
0.2	8,071,030	6775	4010	2382.6	N.D.	0.11	0.31
0.3	3,949,687	6774	2846	1166.1	N.D.	0.06	0.14
0.4	1,709,749	6625	1945	516.15	0.507	0.58	0.60
0.5	639,833	5810	1264	220.25	0.531	0.77	0.84
0.6	197,019	4205	738	93.707	0.555	0.81	0.96
0.7	45,239	2317	333	39.05	0.577	0.86	1.08
0.8	7,526	859	113	17.523	0.606	0.72	1.00
0.9	1,222	168	48	14.548	0.672	0.46	0.64
Before treatment with ionizing radiation							
0.1	11,971,044	5975	4867	4007	N.D.	0.32	2.03
0.2	7,135,543	5975	3818	2388.5	N.D.	0.11	0.39
0.3	3,771,686	5974	2868	1262.7	N.D.	0.00	0.02
0.4	1,734,379	5946	2043	583.38	0.473	0.49	0.51
0.5	667,903	5588	1313	239.05	0.476	0.75	0.81
0.6	203,949	4401	683	92.683	0.489	0.79	0.98
0.7	49,866	2582	280	38.626	0.514	0.79	0.98
0.8	10,104	886	135	22.808	0.564	0.60	0.84
0.9	1,964	177	62	22.192	0.710	0.33	0.47
2 hours after exposure to ionizing radiation							
0.1	11,948,429	5975	4971	3999.5	N.D.	0.27	1.78

0.2	7,088,905	5975	3956	2372.9	N.D.	0.09	0.33
0.3	3,694,167	5975	3019	1236.5	N.D.	0.01	0.05
0.4	1,642,560	5946	2176	552.49	0.470	0.54	0.54
0.5	590,946	5557	1356	212.69	0.465	0.77	0.86
0.6	159,749	4325	695	73.872	0.471	0.84	1.08
0.7	32,088	2405	238	26.684	0.478	0.83	1.16
0.8	6,082	745	91	16.328	0.528	0.56	0.89
0.9	1,518	126	59	24.095	0.750	0.20	0.33
6 hours after exposure to ionizing radiation							
0.1	12,020,500	5975	4974	4023.6	N.D.	0.30	1.81
0.2	7,212,953	5975	4015	2414.4	N.D.	0.07	0.29
0.3	3,850,900	5975	3113	1289	N.D.	0.02	0.08
0.4	1,791,232	5944	2274	602.7	0.490	0.54	0.55
0.5	694,654	5544	1488	250.6	0.495	0.75	0.79
0.6	212,679	4249	817	100.11	0.506	0.81	0.95
0.7	51,516	2367	346	43.529	0.521	0.77	0.99
0.8	11,312	816	155	27.725	0.590	0.48	0.72
0.9	2,122	186	62	22.817	0.715	0.33	0.49

Supplemental Table 4. SVM modeling results upon endoplasmic reticulum stress

Index	Affymetrix ID	Gene Symbol	R^2 - DMSO	R^2 - Tunicamycin	difference in R^2	P value
1	1007_s_at	DDR1	0.51408	0.56016	-0.04608	0.708
2	1053_at	RFC2	0.65607	0.55899	0.09708	0.02
3	117_at	HSPA6	0.10616	0.19385	-0.08769	0.695
4	1405_i_at	CCL5	0.51823	0.61403	-0.0958	0.856
5	1487_at	ESRRA	0.42301	0.2996	0.12341	0.142
6	1729_at	TRADD	0.67147	0.57887	0.0926	0.087
7	1861_at	BAD	0.39274	0.50362	-0.11088	0.83
8	200000_s_at	PRPF8	0.86198	0.63379	0.22819	0.002
9	200001_at	CAPNS1	0.66879	0.64745	0.02134	0.458
10	200002_at	RPL35	0.84752	0.84348	0.00404	0.459
11	200003_s_at	RPL28	0.89465	0.82263	0.07202	0.042
12	200004_at	EIF4G2	0.83579	0.81979	0.016	0.377
13	200005_at	EIF3D	0.69352	0.7245	-0.03098	0.661
14	200006_at	PARK7	0.91682	0.89209	0.02473	0.12
15	200007_at	SRP14	0.80917	0.68124	0.12793	0.078
16	200009_at	GDI2	0.91084	0.85723	0.05361	0.017
17	200010_at	RPL11	0.9031	0.89275	0.01035	0.297
18	200013_at	RPL24	0.8891	0.84131	0.04779	0.027
19	200015_s_at	SEPT2	0.48984	0.47643	0.01341	0.466
20	200017_at	RPS27A	0.8725	0.87642	-0.00392	0.574
21	200018_at	RPS13	0.85926	0.85904	0.00022	0.499
22	200019_s_at	FAU	0.94444	0.89298	0.05146	0.008
23	200020_at	TARDBP	0.67773	0.66497	0.01276	0.396
24	200021_at	CFL1	0.83206	0.76204	0.07002	0.055
25	200024_at	RPS5	0.89733	0.88853	0.0088	0.39
26	200026_at	RPL34	0.88405	0.8627	0.02135	0.151
27	200027_at	NARS	0.52706	0.3578	0.16926	0.016
28	200029_at	RPL19	0.88243	0.86597	0.01646	0.251
29	200030_s_at	SLC25A3	0.82077	0.86185	-0.04108	0.907
30	200032_s_at	RPL9	0.85588	0.85493	0.00095	0.494
31	200033_at	DDX5	0.68108	0.60742	0.07366	0.095
32	200034_s_at	RPL6	0.86836	0.85682	0.01154	0.398
33	200035_at	DULLARD	0.50182	0.34495	0.15687	0.105
34	200036_s_at	RPL10A	0.89438	0.87812	0.01626	0.324
35	200038_s_at	RPL17	0.86811	0.84045	0.02766	0.156
36	200039_s_at	PSMB2	0.82091	0.741	0.07991	0.011
37	200040_at	KHDRBS1	0.79505	0.65479	0.14026	0.021
38	200041_s_at	BAT1	0.72571	0.66758	0.05813	0.224
39	200042_at	C22orf28	0.78919	0.624	0.16519	0.061
40	200043_at	ERH	0.86077	0.80348	0.05729	0.042
41	200044_at	SFRS9	0.64834	0.53963	0.10871	0.124
42	200045_at	ABCF1	0.35955	0.57501	-0.21546	0.988
43	200046_at	DAD1	0.63788	0.65345	-0.01557	0.611
44	200048_s_at	JTB	0.84175	0.85574	-0.01399	0.608
45	200049_at	MYST2	0.20302	0.2424	-0.03938	0.639
46	200050_at	ZNF146	0.48696	0.54317	-0.05621	0.673
47	200051_at	SART1	0.76146	0.59216	0.1693	0.002
48	200052_s_at	ILF2	0.82941	0.85261	-0.0232	0.766
49	200053_at	SPAG7	0.55275	0.63644	-0.08369	0.788
50	200054_at	ZNF259	0.62407	0.50187	0.1222	0.105
51	200055_at	TAF10	0.73768	0.80004	-0.06236	0.82
52	200056_s_at	C1D	0.46871	0.64257	-0.17386	0.995

53	200059_s_at	RHOA	0.54113	0.62415	-0.08302	0.746
54	200060_s_at	RNPS1	0.45965	0.30019	0.15946	0.02
55	200061_s_at	RPS24	0.81467	0.81208	0.00259	0.473
56	200062_s_at	RPL30	0.90102	0.89542	0.0056	0.413
57	200063_s_at	NPM1	0.88207	0.87301	0.00906	0.371
58	200064_at	HSP90AB1	0.69698	0.37635	0.32063	<0.001
59	200065_s_at	ARF1	0.46512	0.53394	-0.06882	0.687
60	200066_at	IK	0.54161	0.54553	-0.00392	0.531
61	200071_at	SMNDC1	0.76443	0.74704	0.01739	0.402
62	200073_s_at	HNRNPD	0.53104	0.33868	0.19236	0.01
63	200074_s_at	RPL14	0.77539	0.75135	0.02404	0.279
64	200077_s_at	OAZ1	0.89729	0.86203	0.03526	0.169
65	200078_s_at	ATP6V0B	0.74714	0.82068	-0.07354	0.944
66	200080_s_at	H3F3A	0.87975	0.84153	0.03822	0.155
67	200082_s_at	RPS7	0.88733	0.88661	0.00072	0.503
68	200084_at	C11orf58	0.75032	0.70626	0.04406	0.238
69	200085_s_at	TCEB2	0.90168	0.86668	0.035	0.1
70	200089_s_at	RPL4	0.84068	0.87664	-0.03596	0.849
71	200090_at	FNTA	0.68572	0.78289	-0.09717	0.922
72	200091_s_at	RPS25	0.89014	0.89659	-0.00645	0.613
73	200092_s_at	RPL37	0.9278	0.88953	0.03827	0.029
74	200093_s_at	HINT1	0.89527	0.84123	0.05404	0.165
75	200096_s_at	ATP6V0E1	0.38658	0.56071	-0.17413	0.972
76	200099_s_at	RPS3A	0.87902	0.88919	-0.01017	0.675
77	200593_s_at	HNRNPU	0.84747	0.72224	0.12523	<0.001
78	200596_s_at	EIF3A	0.94857	0.89389	0.05468	0.025
79	200599_s_at	HSP90B1	0.66989	0.47296	0.19693	0.062
80	200600_at	MSN	0.73922	0.69357	0.04565	0.202
81	200601_at	ACTN4	0.4857	0.26762	0.21808	0.012
82	200604_s_at	PRKAR1A	0.13614	0.26596	-0.12982	0.846
83	200607_s_at	RAD21	0.92936	0.89765	0.03171	0.04
84	200609_s_at	WDR1	0.64677	0.70549	-0.05872	0.772
85	200610_s_at	NCL	0.64056	0.48449	0.15607	0.048
86	200613_at	AP2M1	0.60625	0.6375	-0.03125	0.642
87	200615_s_at	AP2B1	0.58464	0.50638	0.07826	0.144
88	200616_s_at	MLEC	0.78068	0.73884	0.04184	0.337
89	200618_at	LASP1	0.72775	0.64102	0.08673	0.125
90	200619_at	SF3B2	0.67775	0.63798	0.03977	0.353
91	200620_at	TMEM59	0.69785	0.78994	-0.09209	0.811
92	200621_at	CSRP1	0.57671	0.543	0.03371	0.35
93	200623_s_at	CALM3	0.65419	0.70273	-0.04854	0.802
94	200626_s_at	MATR3	0.31695	0.23565	0.0813	0.118
95	200627_at	PTGES3	0.87755	0.83939	0.03816	0.2
96	200629_at	WARS	0.66229	0.54763	0.11466	0.126
97	200632_s_at	NDRG1	0.18473	0.18741	-0.00268	0.522
98	200633_at	UBB	0.91224	0.85864	0.0536	0.003
99	200634_at	PFN1	0.86475	0.81094	0.05381	0.014
100	200642_at	SOD1	0.72825	0.70659	0.02166	0.369
101	200644_at	MARCKSL1	0.58376	0.65698	-0.07322	0.83
102	200645_at	GABARAP	0.60558	0.547	0.05858	0.342
103	200649_at	NUCB1	0.67818	0.62938	0.0488	0.317
104	200650_s_at	LDHA	0.86036	0.66743	0.19293	<0.001
105	200652_at	SSR2	0.75461	0.70076	0.05385	0.069
106	200654_at	P4HB	0.82755	0.81756	0.00999	0.479

107	200657_at	SLC25A5	0.86085	0.85524	0.00561	0.437
108	200658_s_at	PHB	0.84551	0.89303	-0.04752	0.994
109	200660_at	S100A11	0.30163	0.55299	-0.25136	0.987
110	200661_at	CTSA	0.4302	0.57386	-0.14366	0.927
111	200663_at	CD63	0.59255	0.65229	-0.05974	0.736
112	200665_s_at	SPARC	0.36164	0.56425	-0.20261	0.971
113	200666_s_at	DNAJB1	0.66056	0.2009	0.45966	<0.001
114	200668_s_at	UBE2D3	0.78433	0.82877	-0.04444	0.831
115	200670_at	XBP1	0.61397	0.59586	0.01811	0.448
116	200673_at	LAPTM4A	0.60026	0.52405	0.07621	0.198
117	200674_s_at	RPL32	0.90772	0.91389	-0.00617	0.641
118	200675_at	CD81	0.50837	0.64166	-0.13329	0.939
119	200677_at	PTTG1IP	0.67873	0.54092	0.13781	0.039
120	200681_at	GLO1	0.81182	0.74719	0.06463	0.094
121	200682_s_at	UBE2L3	0.59186	0.583	0.00886	0.46
122	200687_s_at	SF3B3	0.79829	0.65697	0.14132	<0.001
123	200692_s_at	HSPA9	0.79487	0.70935	0.08552	0.135
124	200695_at	PPP2R1A	0.71236	0.49387	0.21849	0.001
125	200696_s_at	GSN	0.32288	0.40307	-0.08019	0.759
126	200697_at	HK1	0.22148	0.18038	0.0411	0.368
127	200699_at	KDELR2	0.18625	0.19226	-0.00601	0.53
128	200701_at	NPC2	0.78464	0.78188	0.00276	0.485
129	200702_s_at	DDX24	0.83863	0.86853	-0.0299	0.693
130	200703_at	DYNLL1	0.90776	0.74311	0.16465	0.01
131	200705_s_at	EEF1B2	0.87233	0.92267	-0.05034	0.944
132	200706_s_at	LITAF	0.76216	0.67777	0.08439	0.051
133	200707_at	PRKCSH	0.61417	0.71789	-0.10372	0.925
134	200708_at	GOT2	0.70466	0.51296	0.1917	0.011
135	200709_at	FKBP1A	0.68888	0.54756	0.14132	0.01
136	200710_at	ACADVL	0.55005	0.59322	-0.04317	0.762
137	200713_s_at	MAPRE1	0.44301	0.1544	0.28861	<0.001
138	200718_s_at	SKP1	0.71228	0.64565	0.06663	0.047
139	200720_s_at	ACTR1A	0.29993	0.22289	0.07704	0.27
140	200722_s_at	CAPRIN1	0.48992	0.57166	-0.08174	0.87
141	200726_at	PPP1CC	0.72944	0.8067	-0.07726	0.879
142	200729_s_at	ACTR2	0.56656	0.62354	-0.05698	0.739
143	200733_s_at	PTP4A1	0.70233	0.7639	-0.06157	0.844
144	200734_s_at	ARF3	0.77049	0.71364	0.05685	0.258
145	200736_s_at	GPX1	0.66874	0.72412	-0.05538	0.736
146	200740_s_at	SUMO3	0.62304	0.62156	0.00148	0.505
147	200741_s_at	RPS27	0.89613	0.87282	0.02331	0.197
148	200743_s_at	TPP1	0.70723	0.67728	0.02995	0.337
149	200746_s_at	GNB1	0.80997	0.742	0.06797	0.095
150	200748_s_at	FTH1	0.77886	0.80853	-0.02967	0.62
151	200749_at	RAN	0.46732	0.60486	-0.13754	0.872
152	200751_s_at	HNRNPC	0.7878	0.79513	-0.00733	0.566
153	200752_s_at	CAPN1	0.58965	0.63929	-0.04964	0.751
154	200755_s_at	CALU	0.27446	0.15449	0.11997	0.04
155	200761_s_at	ARL6IP5	0.68831	0.7774	-0.08909	0.878
156	200762_at	DPYSL2	0.52252	0.547	-0.02448	0.64
157	200763_s_at	RPLP1	0.85472	0.86396	-0.00924	0.616
158	200766_at	CTSD	0.19852	0.17147	0.02705	0.421
159	200767_s_at	FAM120A	0.91067	0.89763	0.01304	0.32
160	200770_s_at	LAMC1	0.39685	0.43517	-0.03832	0.63

161	200775_s_at	HNRNPK	0.83748	0.6955	0.14198	<0.001
162	200776_s_at	BZW1	0.67533	0.61513	0.0602	0.166
163	200779_at	ATF4	0.75567	0.72397	0.0317	0.401
164	200782_at	ANXA5	0.31511	0.64385	-0.32874	0.997
165	200783_s_at	STMN1	0.48547	0.55916	-0.07369	0.773
166	200786_at	PSMB7	0.56661	0.70685	-0.14024	0.81
167	200788_s_at	PEA15	0.65148	0.70134	-0.04986	0.773
168	200789_at	ECH1	0.52348	0.64996	-0.12648	0.909
169	200790_at	ODC1	0.78529	0.72174	0.06355	0.068
170	200792_at	XRCC6	0.70909	0.47375	0.23534	0.001
171	200793_s_at	ACO2	0.74137	0.42662	0.31475	0.005
172	200802_at	SARS	0.75411	0.7622	-0.00809	0.546
173	200804_at	TMBIM6	0.79494	0.73611	0.05883	0.264
174	200805_at	LMAN2	0.69862	0.71749	-0.01887	0.61
175	200806_s_at	HSPD1	0.8278	0.86697	-0.03917	0.816
176	200808_s_at	ZYX	0.67136	0.68946	-0.0181	0.603
177	200811_at	CIRBP	0.54142	0.72754	-0.18612	0.985
178	200812_at	CCT7	0.79372	0.8111	-0.01738	0.637
179	200814_at	PSME1	0.85867	0.88993	-0.03126	0.918
180	200815_s_at	PAFAH1B1	0.61073	0.57818	0.03255	0.304
181	200818_at	ATP5O	0.69402	0.67447	0.01955	0.418
182	200819_s_at	RPS15	0.90874	0.89665	0.01209	0.26
183	200820_at	PSMD8	0.79151	0.80502	-0.01351	0.589
184	200824_at	GSTP1	0.40536	0.59003	-0.18467	0.958
185	200825_s_at	HYOU1	0.55867	0.50709	0.05158	0.216
186	200826_at	SNRPD2	0.91659	0.84394	0.07265	<0.001
187	200827_at	PLOD1	0.7501	0.5344	0.2157	0.001
188	200828_s_at	ZNF207	0.72571	0.69316	0.03255	0.301
189	200830_at	PSMD2	0.4063	0.43392	-0.02762	0.62
190	200833_s_at	hCG_1757335	0.68686	0.75419	-0.06733	0.803
191	200834_s_at	RPS21	0.92194	0.90085	0.02109	0.199
192	200837_at	BCAP31	0.54841	0.69361	-0.1452	0.894
193	200840_at	KARS	0.66603	0.59515	0.07088	0.269
194	200842_s_at	EPRS	0.89679	0.88581	0.01098	0.396
195	200845_s_at	PRDX6	0.52506	0.53581	-0.01075	0.568
196	200846_s_at	PPP1CA	0.75591	0.77162	-0.01571	0.587
197	200847_s_at	TMEM66	0.66909	0.55604	0.11305	0.096
198	200851_s_at	KIAA0174	0.50045	0.48062	0.01983	0.432
199	200853_at	H2AFZ	0.86634	0.81803	0.04831	0.096
200	200854_at	NCOR1	0.56229	0.56755	-0.00526	0.541
201	200855_at	C20orf191	0.16758	0.086227	0.081353	0.267
202	200860_s_at	CNOT1	0.6593	0.65081	0.00849	0.449
203	200862_at	DHCR24	0.51029	0.34554	0.16475	<0.001
204	200863_s_at	RAB11A	0.73491	0.73742	-0.00251	0.533
205	200870_at	STRAP	0.6949	0.70667	-0.01177	0.567
206	200871_s_at	PSAP	0.57686	0.70402	-0.12716	0.901
207	200873_s_at	CCT8	0.8381	0.74342	0.09468	0.1
208	200875_s_at	NOP56	0.89583	0.65649	0.23934	<0.001
209	200877_at	CCT4	0.85247	0.63068	0.22179	<0.001
210	200881_s_at	DNAJA1	0.78128	0.51838	0.2629	<0.001
211	200882_s_at	PSMD4	0.88029	0.87421	0.00608	0.405
212	200885_at	RHOC	0.69214	0.7347	-0.04256	0.682
213	200886_s_at	PGAM1	0.74517	0.67097	0.0742	0.093
214	200891_s_at	SSR1	0.64353	0.57269	0.07084	0.235

215	200892_s_at	TRA2B	0.78847	0.72133	0.06714	0.138
216	200894_s_at	FKBP4	0.59169	0.43721	0.15448	0.001
217	200900_s_at	M6PR	0.88677	0.847	0.03977	0.098
218	200902_at	SEP15	0.65806	0.78824	-0.13018	0.94
219	200903_s_at	AHCY	0.83078	0.77543	0.05535	0.182
220	200904_at	HLA-E	0.77678	0.64124	0.13554	0.029
221	200908_s_at	RPLP2	0.81646	0.87171	-0.05525	0.938
222	200910_at	CCT3	0.73215	0.45159	0.28056	<0.001
223	200911_s_at	TACC1	0.59512	0.6705	-0.07538	0.782
224	200912_s_at	EIF4A2	0.68292	0.49064	0.19228	0.003
225	200913_at	PPM1G	0.80059	0.68521	0.11538	<0.001
226	200918_s_at	SRPR	0.35916	0.30815	0.05101	0.273
227	200919_at	PHC2	0.33475	0.3228	0.01195	0.449
228	200920_s_at	BTG1	0.63217	0.61985	0.01232	0.475
229	200922_at	KDELR1	0.71645	0.76852	-0.05207	0.777
230	200924_s_at	SLC3A2	0.352	0.17256	0.17944	0.041
231	200925_at	COX6A1	0.74524	0.82074	-0.0755	0.877
232	200926_at	RPS23	0.90084	0.89549	0.00535	0.386
233	200928_s_at	RAB14	0.19847	0.28129	-0.08282	0.755
234	200932_s_at	DCTN2	0.64214	0.70962	-0.06748	0.788
235	200934_at	DEK	0.72681	0.75317	-0.02636	0.614
236	200935_at	CALR	0.40936	0.28864	0.12072	0.049
237	200936_at	RPL8	0.91842	0.91259	0.00583	0.411
238	200937_s_at	RPL5	0.83966	0.8012	0.03846	0.111
239	200941_at	HSBP1	0.23893	0.24775	-0.00882	0.547
240	200944_s_at	HMGN1	0.93636	0.88847	0.04789	0.024
241	200945_s_at	SEC31A	0.62714	0.45026	0.17688	<0.001
242	200947_s_at	GLUD1	0.4344	0.51124	-0.07684	0.723
243	200948_at	MLF2	0.37597	0.46089	-0.08492	0.791
244	200951_s_at	CCND2	0.76628	0.73287	0.03341	0.327
245	200955_at	IMMT	0.74415	0.64902	0.09513	0.038
246	200957_s_at	SSRP1	0.82776	0.74208	0.08568	0.07
247	200958_s_at	SDCBP	0.42181	0.64733	-0.22552	0.987
248	200959_at	FUS	0.80908	0.61773	0.19135	<0.001
249	200961_at	SEPHS2	0.57408	0.57478	-0.0007	0.499
250	200964_at	UBA1	0.48382	0.60502	-0.1212	0.884
251	200965_s_at	ABLIM1	0.5214	0.55953	-0.03813	0.68
252	200967_at	PPIB	0.88105	0.75405	0.127	0.09
253	200971_s_at	SERP1	0.75518	0.76161	-0.00643	0.538
254	200973_s_at	TSPAN3	0.68197	0.67864	0.00333	0.495
255	200975_at	PPT1	0.52831	0.69887	-0.17056	0.817
256	200977_s_at	TAX1BP1	0.70346	0.7619	-0.05844	0.774
257	200978_at	MDH1	0.81582	0.74122	0.0746	0.022
258	200980_s_at	PDHA1	0.57114	0.77066	-0.19952	0.998
259	200982_s_at	ANXA6	0.28737	0.30847	-0.0211	0.588
260	200984_s_at	CD59	0.54823	0.53978	0.00845	0.405
261	200989_at	HIF1A	0.51625	0.50959	0.00666	0.488
262	200990_at	TRIM28	0.66505	0.52093	0.14412	0.019
263	200991_s_at	SNX17	0.75454	0.61105	0.14349	0.081
264	200994_at	IPO7	0.49371	0.6045	-0.11079	0.861
265	200997_at	RBM4	0.66049	0.7221	-0.06161	0.836
266	200998_s_at	CKAP4	0.40892	0.47608	-0.06716	0.693
267	201000_at	AARS	0.64721	0.25686	0.39035	<0.001
268	201002_s_at	TMEM189-UBE2V1	0.23658	0.46334	-0.22676	0.995

269	201004_at	SSR4	0.7269	0.66386	0.06304	0.177
270	201005_at	CD9	0.44707	0.59025	-0.14318	0.914
271	201007_at	HADHB	0.36849	0.57088	-0.20239	0.963
272	201008_s_at	TXNIP	0.55605	0.48747	0.06858	0.22
273	201011_at	RPN1	0.71579	0.72684	-0.01105	0.571
274	201012_at	ANXA1	0.19254	0.47445	-0.28191	0.992
275	201017_at	EIF1AX	0.21175	0.36745	-0.1557	0.935
276	201019_s_at	EIF1AP1	0.52163	0.5869	-0.06527	0.761
277	201020_at	YWHAH	0.79781	0.67946	0.11835	<0.001
278	201021_s_at	DSTN	0.10423	0.37749	-0.27326	0.963
279	201023_at	TAF7	0.34173	0.45655	-0.11482	0.778
280	201028_s_at	CD99	0.25207	0.5057	-0.25363	0.987
281	201031_s_at	HNRNPH1	0.80375	0.72474	0.07901	0.081
282	201032_at	BLCAP	0.54431	0.4786	0.06571	0.272
283	201037_at	PFKP	0.4729	0.23119	0.24171	<0.001
284	201039_s_at	RAD23A	0.79004	0.65808	0.13196	0.063
285	201041_s_at	DUSP1	0.32991	0.22384	0.10607	0.199
286	201049_s_at	RPS18	0.82216	0.84783	-0.02567	0.815
287	201050_at	PLD3	0.50844	0.623	-0.11456	0.949
288	201051_at	ANP32A	0.63469	0.55753	0.07716	0.249
289	201053_s_at	PSMF1	0.70616	0.64104	0.06512	0.154
290	201055_s_at	HNRNPA0	0.63426	0.44506	0.1892	0.002
291	201056_at	GOLGB1	0.57044	0.18976	0.38068	<0.001
292	201063_at	RCN1	0.78855	0.73191	0.05664	0.147
293	201064_s_at	PABPC4	0.56898	0.66495	-0.09597	0.87
294	201066_at	CYC1	0.8313	0.86315	-0.03185	0.843
295	201068_s_at	PSMC2	0.74385	0.67003	0.07382	0.202
296	201074_at	SMARCC1	0.75324	0.65313	0.10011	0.026
297	201077_s_at	NHP2L1	0.80754	0.80286	0.00468	0.472
298	201078_at	TM9SF2	0.63452	0.52585	0.10867	0.228
299	201079_at	SYNGR2	0.7066	0.6851	0.0215	0.355
300	201081_s_at	PIP4K2B	0.071219	0.044148	0.027071	0.422
301	201085_s_at	SON	0.91375	0.93635	-0.0226	0.772
302	201087_at	PXN	0.23337	0.18289	0.05048	0.37
303	201088_at	KPNA2	0.77803	0.71413	0.0639	0.129
304	201089_at	ATP6V1B2	0.6978	0.6164	0.0814	0.182
305	201091_s_at	CBX3	0.86496	0.86725	-0.00229	0.553
306	201092_at	RBBP7	0.64832	0.64116	0.00716	0.469
307	201094_at	RPS29	0.84493	0.87365	-0.02872	0.833
308	201095_at	DAP	0.22446	0.2126	0.01186	0.484
309	201097_s_at	ARF4	0.45291	0.59451	-0.1416	0.934
310	201098_at	COPB2	0.82475	0.63577	0.18898	<0.001
311	201099_at	USP9X	0.29175	0.38673	-0.09498	0.831
312	201101_s_at	BCLAF1	0.93107	0.88806	0.04301	0.001
313	201102_s_at	PFKL	0.82373	0.62818	0.19555	<0.001
314	201106_at	GPX4	0.59649	0.48655	0.10994	0.142
315	201112_s_at	CSE1L	0.83794	0.71796	0.11998	<0.001
316	201113_at	TUFM	0.82549	0.81893	0.00656	0.476
317	201115_at	POLD2	0.34154	0.29478	0.04676	0.293
318	201118_at	PGD	0.65613	0.70231	-0.04618	0.795
319	201119_s_at	COX8A	0.65435	0.64092	0.01343	0.457
320	201121_s_at	PGRMC1	0.76166	0.68457	0.07709	0.049
321	201126_s_at	MGAT1	0.37184	0.34912	0.02272	0.424
322	201128_s_at	ACLY	0.65633	0.38415	0.27218	<0.001

323	201129_at	SFRS7	0.45939	0.49086	-0.03147	0.698
324	201132_at	HNRNPH2	0.47484	0.53138	-0.05654	0.684
325	201133_s_at	PJA2	0.61111	0.67482	-0.06371	0.821
326	201135_at	ECHS1	0.70021	0.75428	-0.05407	0.777
327	201136_at	PLP2	0.27777	0.48319	-0.20542	0.915
328	201137_s_at	HLA-DPB1	0.1838	0.32873	-0.14493	0.919
329	201139_s_at	SSB	0.91409	0.87483	0.03926	0.148
330	201141_at	GPNMB	0.16341	0.31698	-0.15357	0.888
331	201144_s_at	EIF2S1	0.75359	0.63331	0.12028	0.089
332	201145_at	HAX1	0.69879	0.79859	-0.0998	0.853
333	201146_at	NFE2L2	0.63136	0.5836	0.04776	0.319
334	201155_s_at	MFN2	0.3787	0.35935	0.01935	0.431
335	201156_s_at	RAB5C	0.66826	0.75602	-0.08776	0.892
336	201158_at	NMT1	0.60463	0.51328	0.09135	0.207
337	201165_s_at	PUM1	0.60916	0.60613	0.00303	0.515
338	201170_s_at	BHLHE40	0.70622	0.78792	-0.0817	0.906
339	201174_s_at	TERF2IP	0.65921	0.52407	0.13514	0.012
340	201175_at	TMX2	0.3773	0.45922	-0.08192	0.761
341	201176_s_at	ARCN1	0.70425	0.66294	0.04131	0.192
342	201177_s_at	UBA2	0.86472	0.89037	-0.02565	0.788
343	201178_at	FBXO7	0.48942	0.54111	-0.05169	0.711
344	201180_s_at	GNAI3	0.5953	0.56331	0.03199	0.377
345	201182_s_at	CHD4	0.81048	0.8868	-0.07632	0.989
346	201186_at	LRPAP1	0.60331	0.5823	0.02101	0.399
347	201189_s_at	ITPR3	0.60758	0.39384	0.21374	0.031
348	201191_at	PITPNA	0.69363	0.62371	0.06992	0.189
349	201193_at	IDH1	0.5867	0.43042	0.15628	0.068
350	201194_at	SEPW1	0.7205	0.60219	0.11831	0.061
351	201195_s_at	SLC7A5	0.69894	0.54672	0.15222	0.062
352	201197_at	AMD1	0.76927	0.58685	0.18242	<0.001
353	201198_s_at	PSMD1	0.88272	0.84916	0.03356	0.322
354	201200_at	CREG1	0.24937	0.45128	-0.20191	0.988
355	201201_at	CSTB	0.24994	0.52193	-0.27199	0.977
356	201204_s_at	RRBP1	0.72116	0.52114	0.20002	<0.001
357	201209_at	HDAC1	0.88283	0.78962	0.09321	0.042
358	201212_at	LGMN	0.28504	0.40221	-0.11717	0.856
359	201214_s_at	PPP1R7	0.66222	0.57308	0.08914	0.179
360	201215_at	PLS3	0.073344	0.38248	-0.309136	0.987
361	201216_at	ERP29	0.76626	0.83585	-0.06959	0.782
362	201221_s_at	SNRNP70	0.79667	0.64903	0.14764	0.02
363	201223_s_at	RAD23B	0.52757	0.71773	-0.19016	0.895
364	201224_s_at	SRRM1	0.8765	0.90597	-0.02947	0.665
365	201226_at	NDUFB8	0.61186	0.70964	-0.09778	0.782
366	201228_s_at	ARIH2	0.093977	0.20362	-0.109643	0.781
367	201231_s_at	ENO1	0.73876	0.60459	0.13417	0.001
368	201232_s_at	PSMD13	0.54668	0.53113	0.01555	0.445
369	201234_at	ILK	0.59163	0.71424	-0.12261	0.888
370	201235_s_at	BTG2	0.63488	0.5871	0.04778	0.262
371	201238_s_at	CAPZA2	0.60952	0.46847	0.14105	0.097
372	201240_s_at	LOC653566	0.82154	0.7812	0.04034	0.362
373	201241_at	DDX1	0.78319	0.7919	-0.00871	0.577
374	201243_s_at	ATP1B1	0.74616	0.79611	-0.04995	0.799
375	201244_s_at	RAF1	0.27056	0.56399	-0.29343	0.997
376	201246_s_at	OTUB1	0.43473	0.36905	0.06568	0.291

377	201248_s_at	SREBF2	0.42377	0.42236	0.00141	0.485
378	201250_s_at	SLC2A1	0.18731	0.30461	-0.1173	0.809
379	201252_at	PSMC4	0.66473	0.78189	-0.11716	0.913
380	201253_s_at	CDIPT	0.51835	0.57846	-0.06011	0.72
381	201256_at	COX7A2L	0.52522	0.63138	-0.10616	0.843
382	201258_at	RPS16	0.8458	0.79598	0.04982	0.083
383	201260_s_at	SYPL1	0.51124	0.63199	-0.12075	0.857
384	201263_at	TARS	0.90956	0.65063	0.25893	<0.001
385	201266_at	TXNRD1	0.66215	0.40425	0.2579	<0.001
386	201267_s_at	PSMC3	0.90089	0.92663	-0.02574	0.917
387	201268_at	NME1	0.89272	0.84391	0.04881	0.081
388	201271_s_at	RALY	0.81582	0.77605	0.03977	0.196
389	201272_at	AKR1B1	0.536	0.68233	-0.14633	0.878
390	201273_s_at	SRP9	0.80617	0.76978	0.03639	0.259
391	201274_at	PSMA5	0.68222	0.77681	-0.09459	0.981
392	201275_at	FDPS	0.79882	0.46729	0.33153	<0.001
393	201276_at	RAB5B	0.83784	0.70127	0.13657	0.032
394	201277_s_at	HNRNPAB	0.87939	0.71582	0.16357	<0.001
395	201281_at	ADRM1	0.68308	0.6233	0.05978	0.173
396	201282_at	OGDH	0.54439	0.54554	-0.00115	0.477
397	201284_s_at	APEH	0.34516	0.35683	-0.01167	0.546
398	201288_at	ARHGDIB	0.87164	0.79559	0.07605	0.044
399	201290_at	SEC11A	0.55877	0.55038	0.00839	0.505
400	201291_s_at	TOP2A	0.73479	0.69073	0.04406	0.244
401	201299_s_at	MOBKL1B	0.91476	0.91655	-0.00179	0.541
402	201302_at	ANXA4	0.61999	0.70125	-0.08126	0.843
403	201303_at	EIF4A3	0.62347	0.65358	-0.03011	0.68
404	201304_at	NDUFA5	0.66112	0.78438	-0.12326	0.974
405	201306_s_at	ANP32B	0.8333	0.86302	-0.02972	0.703
406	201312_s_at	SH3BGRL	0.61003	0.64629	-0.03626	0.669
407	201313_at	ENO2	0.27174	0.29073	-0.01899	0.589
408	201314_at	STK25	0.37273	0.24803	0.1247	0.182
409	201316_at	PSMA2	0.81571	0.6564	0.15931	0.005
410	201319_at	MYL12A	0.53716	0.56315	-0.02599	0.593
411	201321_s_at	SMARCC2	0.70612	0.74033	-0.03421	0.716
412	201322_at	ATP5B	0.64225	0.77335	-0.1311	0.957
413	201323_at	EBNA1BP2	0.76719	0.76096	0.00623	0.488
414	201327_s_at	CCT6A	0.66822	0.4545	0.21372	<0.001
415	201330_at	RARS	0.20394	0.042519	0.161421	0.087
416	201332_s_at	STAT6	0.38714	0.52542	-0.13828	0.892
417	201339_s_at	SCP2	0.2777	0.25609	0.02161	0.435
418	201341_at	ENC1	0.52734	0.58184	-0.0545	0.728
419	201342_at	SNRPC	0.74421	0.68147	0.06274	0.173
420	201346_at	ADIPOR2	0.5409	0.37526	0.16564	0.014
421	201349_at	SLC9A3R1	0.119	0.28982	-0.17082	0.935
422	201350_at	FLOT2	0.73922	0.81879	-0.07957	0.91
423	201351_s_at	YME1L1	0.81496	0.70826	0.1067	<0.001
424	201354_s_at	BAZ2A	0.4631	0.34365	0.11945	0.136
425	201356_at	SF3A1	0.52022	0.47237	0.04785	0.426
426	201358_s_at	COPB1	0.67023	0.74416	-0.07393	0.885
427	201360_at	CST3	0.62177	0.67855	-0.05678	0.751
428	201361_at	TMEM109	0.71263	0.75005	-0.03742	0.67
429	201363_s_at	IVNS1ABP	0.4209	0.41518	0.00572	0.495
430	201364_s_at	OAZ2	0.097466	0.23272	-0.135254	0.922

431	201366_at	ANXA7	0.54827	0.59774	-0.04947	0.692
432	201368_at	ZFP36L2	0.48702	0.58589	-0.09887	0.752
433	201371_s_at	CUL3	0.82942	0.7169	0.11252	0.014
434	201375_s_at	PPP2CB	0.56365	0.52673	0.03692	0.299
435	201376_s_at	HNRNPF	0.77051	0.49819	0.27232	<0.001
436	201379_s_at	TPD52L2	0.37374	0.46018	-0.08644	0.857
437	201380_at	CRTAP	0.27189	0.48753	-0.21564	0.972
438	201382_at	CACYBP	0.14705	0.16955	-0.0225	0.584
439	201383_s_at	LOC100133166	0.52628	0.6227	-0.09642	0.844
440	201385_at	DHX15	0.69445	0.46633	0.22812	<0.001
441	201387_s_at	UCHL1	0.31187	0.4369	-0.12503	0.816
442	201388_at	PSMD3	0.70556	0.63774	0.06782	0.21
443	201390_s_at	CSNK2B	0.69212	0.72674	-0.03462	0.667
444	201391_at	TRAP1	0.80228	0.67182	0.13046	0.028
445	201392_s_at	IGF2R	0.69122	0.83737	-0.14615	0.995
446	201395_at	RBM5	0.64382	0.76921	-0.12539	0.93
447	201396_s_at	SGTA	0.35015	0.25706	0.09309	0.244
448	201397_at	PHGDH	0.61027	0.48791	0.12236	0.044
449	201400_at	PSMB3	0.87945	0.85231	0.02714	0.143
450	201403_s_at	MGST3	0.60218	0.70831	-0.10613	0.885
451	201405_s_at	COPS6	0.73686	0.80112	-0.06426	0.843
452	201407_s_at	PPP1CB	0.87358	0.89013	-0.01655	0.713
453	201411_s_at	PLEKHB2	0.7964	0.81	-0.0136	0.59
454	201412_at	LRP10	0.77144	0.70703	0.06441	0.158
455	201413_at	HSD17B4	0.78856	0.554	0.23456	<0.001
456	201415_at	GSS	0.51212	0.59333	-0.08121	0.775
457	201417_at	SOX4	0.40631	0.51058	-0.10427	0.903
458	201419_at	BAP1	0.56367	0.43104	0.13263	0.166
459	201420_s_at	WDR77	0.52532	0.48927	0.03605	0.421
460	201422_at	IFI30	0.47065	0.6674	-0.19675	0.99
461	201423_s_at	CUL4A	0.4798	0.54526	-0.06546	0.747
462	201425_at	ALDH2	0.3389	0.46164	-0.12274	0.829
463	201426_s_at	VIM	0.45642	0.59796	-0.14154	0.913
464	201427_s_at	SEPP1	0.082015	0.33924	-0.257225	0.993
465	201429_s_at	RPL37A	0.8638	0.87067	-0.00687	0.609
466	201433_s_at	PTDSS1	0.57336	0.45274	0.12062	0.076
467	201434_at	TTC1	0.66552	0.65574	0.00978	0.496
468	201437_s_at	EIF4E	0.78522	0.78036	0.00486	0.501
469	201439_at	GBF1	0.87019	0.79276	0.07743	0.004
470	201441_at	COX6B1	0.85944	0.70574	0.1537	<0.001
471	201444_s_at	ATP6AP2	0.86461	0.71006	0.15455	0.001
472	201445_at	CNN3	0.36442	0.51029	-0.14587	0.888
473	201447_at	TIA1	0.3132	0.50796	-0.19476	0.943
474	201459_at	RUVBL2	0.79317	0.80451	-0.01134	0.627
475	201460_at	MAPKAPK2	0.30623	0.35281	-0.04658	0.662
476	201462_at	SCRN1	0.087876	0.15219	-0.064314	0.723
477	201463_s_at	LOC100133665	0.82584	0.81397	0.01187	0.43
478	201466_s_at	JUN	0.54597	0.52791	0.01806	0.47
479	201468_s_at	NQO1	0.54172	0.7556	-0.21388	0.99
480	201469_s_at	SHC1	0.16789	0.28279	-0.1149	0.899
481	201470_at	GSTO1	0.80932	0.77719	0.03213	0.239
482	201472_at	VBP1	0.42624	0.48378	-0.05754	0.683
483	201473_at	JUNB	0.55912	0.66471	-0.10559	0.875
484	201477_s_at	RRM1	0.81266	0.74663	0.06603	0.114

485	201478_s_at	DKC1	0.75631	0.70617	0.05014	0.034
486	201480_s_at	SUPT5H	0.13959	0.14128	-0.00169	0.502
487	201484_at	SUPT4H1	0.44741	0.6042	-0.15679	0.927
488	201486_at	RCN2	0.62606	0.69747	-0.07141	0.885
489	201487_at	CTSC	0.24644	0.42364	-0.1772	0.896
490	201489_at	PPIF	0.59125	0.57435	0.0169	0.425
491	201491_at	AHSA1	0.78888	0.66062	0.12826	0.005
492	201492_s_at	RPL41	0.85633	0.86187	-0.00554	0.604
493	201493_s_at	PUM2	0.74366	0.73619	0.00747	0.484
494	201494_at	PRCP	0.60526	0.59676	0.0085	0.463
495	201499_s_at	USP7	0.48739	0.49448	-0.00709	0.542
496	201500_s_at	PPP1R11	0.54424	0.6511	-0.10686	0.791
497	201502_s_at	NFKBIA	0.80605	0.84753	-0.04148	0.837
498	201507_at	PFDN1	0.32152	0.40334	-0.08182	0.8
499	201511_at	AAMP	0.50432	0.6128	-0.10848	0.881
500	201512_s_at	TOMM70A	0.54859	0.61218	-0.06359	0.741
501	201513_at	TSN	0.632	0.65013	-0.01813	0.61
502	201514_s_at	G3BP1	0.87769	0.76672	0.11097	0.001
503	201516_at	SRM	0.79949	0.81351	-0.01402	0.626
504	201521_s_at	NCBP2	0.15225	0.19338	-0.04113	0.685
505	201526_at	ARF5	0.35726	0.55227	-0.19501	0.929
506	201527_at	ATP6V1F	0.7228	0.75934	-0.03654	0.697
507	201528_at	RPA1	0.78432	0.62562	0.1587	0.003
508	201531_at	ZFP36	0.56028	0.56278	-0.0025	0.506
509	201532_at	PSMA3	0.75733	0.80205	-0.04472	0.837
510	201533_at	CTNNB1	0.18542	0.34825	-0.16283	0.901
511	201534_s_at	UBL3	0.61881	0.62435	-0.00554	0.535
512	201536_at	DUSP3	0.1737	0.072748	0.100952	0.279
513	201540_at	FHL1	0.043405	0.25148	-0.208075	0.989
514	201541_s_at	ZNHIT1	0.25607	0.4595	-0.20343	0.929
515	201543_s_at	SAR1A	0.72339	0.74861	-0.02522	0.632
516	201546_at	TRIP12	0.67386	0.68259	-0.00873	0.548
517	201553_s_at	LAMP1	0.76678	0.77893	-0.01215	0.557
518	201555_at	MCM3	0.80243	0.81014	-0.00771	0.594
519	201557_at	VAMP2	0.37656	0.39845	-0.02189	0.599
520	201561_s_at	CLSTN1	0.73932	0.70956	0.02976	0.332
521	201563_at	SORD	0.53453	0.5755	-0.04097	0.703
522	201564_s_at	FSCN1	0.34416	0.58275	-0.23859	0.991
523	201565_s_at	ID2	0.77643	0.83365	-0.05722	0.879
524	201567_s_at	GOLGA4	0.69049	0.58175	0.10874	0.013
525	201568_at	UQCRQ	0.82457	0.76479	0.05978	0.026
526	201570_at	SAMM50	0.59958	0.60961	-0.01003	0.536
527	201574_at	ETF1	0.86946	0.79266	0.0768	0.019
528	201576_s_at	GLB1	0.47909	0.41115	0.06794	0.235
529	201580_s_at	TMX4	0.70755	0.70588	0.00167	0.48
530	201582_at	SEC23B	0.37986	0.37395	0.00591	0.466
531	201584_s_at	DDX39	0.78708	0.7822	0.00488	0.478
532	201585_s_at	SFPQ	0.59265	0.62477	-0.03212	0.687
533	201587_s_at	IRAK1	0.77378	0.74808	0.0257	0.355
534	201588_at	TXNL1	0.86195	0.8632	-0.00125	0.517
535	201591_s_at	NISCH	0.73115	0.70159	0.02956	0.325
536	201592_at	EIF3H	0.86372	0.88504	-0.02132	0.777
537	201594_s_at	PPP4R1	0.15354	0.31785	-0.16431	0.898
538	201597_at	COX7A2	0.6321	0.50895	0.12315	0.063

539	201598_s_at	INPPL1	0.30022	0.18462	0.1156	0.17
540	201599_at	OAT	0.071315	0.26462	-0.193305	0.924
541	201600_at	PHB2	0.86862	0.91913	-0.05051	0.934
542	201604_s_at	PPP1R12A	0.42922	0.44416	-0.01494	0.565
543	201608_s_at	PWP1	0.84879	0.76713	0.08166	0.031
544	201612_at	ALDH9A1	0.35479	0.47092	-0.11613	0.762
545	201613_s_at	AP1G2	0.49633	0.51884	-0.02251	0.596
546	201614_s_at	RUVBL1	0.721	0.65798	0.06302	0.19
547	201619_at	PRDX3	0.72786	0.67255	0.05531	0.159
548	201620_at	MBTPS1	0.55777	0.6037	-0.04593	0.706
549	201622_at	SND1	0.73694	0.68994	0.047	0.229
550	201624_at	DARS	0.83396	0.83674	-0.00278	0.542
551	201626_at	INSIG1	0.30767	0.023573	0.284097	<0.001
552	201628_s_at	RRAGA	0.30636	0.44202	-0.13566	0.872
553	201629_s_at	ACP1	0.6823	0.79368	-0.11138	0.935
554	201631_s_at	IER3	0.80752	0.73772	0.0698	0.093
555	201632_at	EIF2B1	0.074639	0.3488	-0.274161	0.946
556	201633_s_at	CYB5B	0.45151	0.41183	0.03968	0.379
557	201637_s_at	FXR1	0.77935	0.70108	0.07827	0.127
558	201639_s_at	CPSF1	0.50591	0.48708	0.01883	0.429
559	201641_at	BST2	0.71512	0.79119	-0.07607	0.904
560	201642_at	IFNGR2	0.59295	0.54031	0.05264	0.297
561	201644_at	TSTA3	0.32397	0.52617	-0.2022	0.953
562	201645_at	TNC	0.22229	0.18986	0.03243	0.408
563	201647_s_at	SCARB2	0.42496	0.6078	-0.18284	0.942
564	201648_at	JAK1	0.39093	0.47803	-0.0871	0.735
565	201649_at	UBE2L6	0.79795	0.79989	-0.00194	0.524
566	201651_s_at	PACSIN2	0.43169	0.53699	-0.1053	0.793
567	201652_at	COPS5	0.71239	0.7732	-0.06081	0.884
568	201653_at	CNIH	0.83479	0.7641	0.07069	0.173
569	201657_at	ARL1	0.21832	0.37569	-0.15737	0.974
570	201662_s_at	ACSL3	0.83326	0.76144	0.07182	0.119
571	201663_s_at	SMC4	0.69602	0.73084	-0.03482	0.733
572	201666_at	TIMP1	0.42106	0.49185	-0.07079	0.653
573	201672_s_at	USP14	0.39719	0.466	-0.06881	0.76
574	201673_s_at	GYS1	0.62883	0.4923	0.13653	0.064
575	201677_at	C3orf37	0.31167	0.26517	0.0465	0.348
576	201682_at	PMPCB	0.66089	0.72576	-0.06487	0.772
577	201684_s_at	TOX4	0.42116	0.10497	0.31619	<0.001
578	201687_s_at	API5	0.62908	0.65291	-0.02383	0.607
579	201689_s_at	TPD52	0.77012	0.85077	-0.08065	0.904
580	201694_s_at	EGR1	0.50325	0.23657	0.26668	0.005
581	201695_s_at	NP	0.38321	0.37715	0.00606	0.503
582	201696_at	SFRS4	0.34581	0.46012	-0.11431	0.809
583	201697_s_at	DNMT1	0.74592	0.67882	0.0671	0.078
584	201699_at	PSMC6	0.80963	0.67398	0.13565	0.002
585	201700_at	CCND3	0.61395	0.72445	-0.1105	0.943
586	201702_s_at	PPP1R10	0.83836	0.8386	-0.00024	0.543
587	201704_at	ENTPD6	0.5902	0.51136	0.07884	0.188
588	201705_at	PSMD7	0.81675	0.77268	0.04407	0.243
589	201707_at	PEX19	0.42374	0.1981	0.22564	0.016
590	201709_s_at	NIPSNAP1	0.60611	0.55896	0.04715	0.331
591	201710_at	MYBL2	0.69092	0.71081	-0.01989	0.618
592	201713_s_at	RANBP2	0.58383	0.63592	-0.05209	0.719

593	201714_at	TUBG1	0.65345	0.73679	-0.08334	0.942
594	201715_s_at	ACIN1	0.64445	0.54132	0.10313	0.094
595	201716_at	SNX1	0.44478	0.20048	0.2443	<0.001
596	201717_at	MRPL49	0.35597	0.45811	-0.10214	0.797
597	201719_s_at	EPB41L2	0.58083	0.57026	0.01057	0.472
598	201721_s_at	LAPTM5	0.61316	0.66612	-0.05296	0.732
599	201724_s_at	GALNT1	0.30352	0.26778	0.03574	0.329
600	201725_at	CDC123	0.56129	0.66748	-0.10619	0.971
601	201726_at	ELAVL1	0.62716	0.64578	-0.01862	0.673
602	201728_s_at	KIAA0100	0.82977	0.86283	-0.03306	0.706
603	201731_s_at	TPR	0.34191	0.37158	-0.02967	0.613
604	201732_s_at	CLCN3	0.75726	0.61543	0.14183	0.003
605	201738_at	EIF1B	0.78088	0.71779	0.06309	0.148
606	201739_at	SGK1	0.22608	0.37737	-0.15129	0.892
607	201740_at	NDUFS3	0.79815	0.82655	-0.0284	0.752
608	201746_at	TP53	0.81472	0.70019	0.11453	0.016
609	201747_s_at	SAFB	0.54056	0.40928	0.13128	0.057
610	201751_at	JOSD1	0.50258	0.56418	-0.0616	0.748
611	201752_s_at	ADD3	0.68926	0.69075	-0.00149	0.528
612	201754_at	COX6C	0.73211	0.61406	0.11805	0.019
613	201755_at	MCM5	0.81428	0.73607	0.07821	0.009
614	201756_at	RPA2	0.8023	0.72396	0.07834	0.164
615	201757_at	NDUFS5	0.27688	0.28108	-0.0042	0.508
616	201758_at	TSG101	0.49034	0.72474	-0.2344	0.99
617	201760_s_at	WSB2	0.55613	0.62643	-0.0703	0.813
618	201761_at	MTHFD2	0.70402	0.57705	0.12697	0.098
619	201762_s_at	PSME2	0.75854	0.75476	0.00378	0.472
620	201763_s_at	DAXX	0.11893	0.13109	-0.01216	0.55
621	201764_at	TMEM106C	0.69274	0.60639	0.08635	0.045
622	201768_s_at	CLINT1	0.71491	0.7037	0.01121	0.443
623	201770_at	SNRPA	0.90083	0.81885	0.08198	0.049
624	201771_at	SCAMP3	0.73633	0.65497	0.08136	0.062
625	201772_at	AZIN1	0.78164	0.7749	0.00674	0.468
626	201774_s_at	NCAPD2	0.76114	0.77116	-0.01002	0.572
627	201777_s_at	KIAA0494	0.76657	0.76706	-0.00049	0.539
628	201780_s_at	RNF13	0.67543	0.71938	-0.04395	0.758
629	201781_s_at	AIP	0.69205	0.78426	-0.09221	0.951
630	201783_s_at	RELA	0.62312	0.58946	0.03366	0.371
631	201786_s_at	ADAR	0.90063	0.88171	0.01892	0.317
632	201788_at	DDX42	0.12442	0.14012	-0.0157	0.577
633	201791_s_at	DHCR7	0.72608	0.38406	0.34202	<0.001
634	201795_at	LBR	0.75339	0.74282	0.01057	0.462
635	201797_s_at	VARS	0.60787	0.58909	0.01878	0.426
636	201800_s_at	OSBP	0.58549	0.4202	0.16529	0.022
637	201802_at	SLC29A1	0.39298	0.18998	0.203	<0.001
638	201803_at	POLR2B	0.36889	0.63538	-0.26649	0.995
639	201805_at	PRKAG1	0.62326	0.63064	-0.00738	0.546
640	201806_s_at	ATXN2L	0.56418	0.62035	-0.05617	0.81
641	201807_at	VPS26A	0.50326	0.48907	0.01419	0.424
642	201810_s_at	SH3BP5	0.24011	0.47425	-0.23414	0.958
643	201812_s_at	C4orf46	0.79309	0.78246	0.01063	0.413
644	201815_s_at	TBC1D5	0.54835	0.32433	0.22402	0.017
645	201816_s_at	GBAS	0.48441	0.6697	-0.18529	0.939
646	201817_at	UBE3C	0.23426	0.26931	-0.03505	0.634

647	201818_at	LPCAT1	0.32707	0.40876	-0.08169	0.817
648	201819_at	SCARB1	0.3901	0.6012	-0.2111	0.969
649	201821_s_at	TIMM17A	0.47697	0.53923	-0.06226	0.748
650	201823_s_at	RNF14	0.58323	0.50372	0.07951	0.18
651	201826_s_at	SCCPDH	0.018049	0.039606	-0.021557	0.56
652	201827_at	SMARCD2	0.55019	0.51318	0.03701	0.379
653	201830_s_at	NET1	0.75049	0.73431	0.01618	0.372
654	201833_at	HDAC2	0.79888	0.76041	0.03847	0.217
655	201834_at	PRKAB1	0.31383	0.35386	-0.04003	0.658
656	201837_s_at	SUPT7L	0.19982	0.24628	-0.04646	0.845
657	201840_at	NEDD8	0.25716	0.41895	-0.16179	0.975
658	201841_s_at	HSPB1	0.48917	0.62469	-0.13552	0.955
659	201845_s_at	RYBP	0.62206	0.69845	-0.07639	0.816
660	201847_at	LIPA	0.42104	0.37324	0.0478	0.31
661	201849_at	BNIP3	0.44638	0.39413	0.05225	0.337
662	201850_at	CAPG	0.52265	0.59984	-0.07719	0.723
663	201851_at	SH3GL1	0.26618	0.22919	0.03699	0.356
664	201853_s_at	CDC25B	0.54249	0.50593	0.03656	0.374
665	201854_s_at	ATMIN	0.30214	0.38031	-0.07817	0.727
666	201856_s_at	ZFR	0.8375	0.81399	0.02351	0.291
667	201859_at	SRGN	0.64356	0.73016	-0.0866	0.846
668	201861_s_at	LRRFIP1	0.88182	0.87711	0.00471	0.436
669	201863_at	FAM32A	0.80017	0.7113	0.08887	0.041
670	201864_at	GDI1	0.48813	0.36421	0.12392	0.148
671	201868_s_at	TBL1X	0.60069	0.66495	-0.06426	0.858
672	201870_at	TOMM34	0.27231	0.070683	0.201627	0.026
673	201872_s_at	ABCE1	0.84716	0.78005	0.06711	0.167
674	201874_at	MPZL1	0.18299	0.24312	-0.06013	0.722
675	201881_s_at	ARIH1	0.16701	0.23111	-0.0641	0.689
676	201885_s_at	CYB5R3	0.59356	0.48623	0.10733	0.156
677	201889_at	FAM3C	0.48088	0.62911	-0.14823	0.92
678	201890_at	RRM2	0.80081	0.77786	0.02295	0.367
679	201892_s_at	IMPDH2	0.78447	0.71023	0.07424	0.112
680	201895_at	ARAF	0.63327	0.39547	0.2378	0.009
681	201896_s_at	PSRC1	0.52306	0.62042	-0.09736	0.802
682	201897_s_at	CKS1B	0.9088	0.86468	0.04412	0.236
683	201899_s_at	UBE2A	0.65985	0.68533	-0.02548	0.606
684	201900_s_at	AKR1A1	0.74129	0.68195	0.05934	0.168
685	201903_at	UQCRC1	0.84134	0.87295	-0.03161	0.809
686	201908_at	DVL3	0.53162	0.4388	0.09282	0.186
687	201912_s_at	GSPT1	0.83749	0.76702	0.07047	0.055
688	201913_s_at	COASY	0.52732	0.50251	0.02481	0.438
689	201914_s_at	SEC63	0.84979	0.85854	-0.00875	0.535
690	201917_s_at	SLC25A36	0.89368	0.90115	-0.00747	0.623
691	201920_at	SLC20A1	0.64869	0.43398	0.21471	0.003
692	201921_at	GNG10	0.40501	0.44619	-0.04118	0.741
693	201922_at	TINP1	0.55583	0.57513	-0.0193	0.572
694	201923_at	PRDX4	0.845	0.84898	-0.00398	0.544
695	201924_at	AFF1	0.52878	0.71198	-0.1832	0.954
696	201925_s_at	CD55	0.45169	0.62513	-0.17344	0.964
697	201928_at	PKP4	0.044181	0.1654	-0.121219	0.711
698	201930_at	MCM6	0.90138	0.79927	0.10211	<0.001
699	201931_at	ETFA	0.49725	0.75741	-0.26016	0.995
700	201932_at	LRRC41	0.49826	0.55344	-0.05518	0.685

701	201933_at	CHMP1A	0.11198	0.12923	-0.01725	0.578
702	201934_at	WDR82	0.75434	0.61878	0.13556	0.121
703	201936_s_at	EIF4G3	0.37921	0.26406	0.11515	0.188
704	201937_s_at	DNPEP	0.75465	0.56598	0.18867	0.008
705	201938_at	CDK2AP1	0.2869	0.30987	-0.02297	0.588
706	201944_at	HEXB	0.3413	0.51454	-0.17324	0.852
707	201945_at	FURIN	0.56526	0.56169	0.00357	0.5
708	201947_s_at	CCT2	0.76967	0.68474	0.08493	0.039
709	201948_at	GNL2	0.70103	0.40689	0.29414	<0.001
710	201952_at	ALCAM	0.50708	0.56218	-0.0551	0.699
711	201953_at	CIB1	0.40739	0.55163	-0.14424	0.901
712	201954_at	ARPC1B	0.54771	0.55291	-0.0052	0.53
713	201955_at	CCNC	0.93044	0.86919	0.06125	0.007
714	201956_s_at	GNPAT	0.85749	0.85203	0.00546	0.453
715	201957_at	PPP1R12B	0.25639	0.12296	0.13343	0.142
716	201959_s_at	MYCBP2	0.77559	0.88415	-0.10856	0.977
717	201961_s_at	RNF41	0.33326	0.48796	-0.1547	0.979
718	201963_at	ACSL1	0.65402	0.78943	-0.13541	0.984
719	201964_at	SETX	0.65585	0.59895	0.0569	0.277
720	201966_at	NDUFS2	0.66046	0.76911	-0.10865	0.939
721	201967_at	RBM6	0.4586	0.41134	0.04726	0.353
722	201968_s_at	PGM1	0.38063	0.41084	-0.03021	0.622
723	201970_s_at	NASP	0.87715	0.7981	0.07905	<0.001
724	201971_s_at	ATP6V1A	0.86099	0.76405	0.09694	0.002
725	201973_s_at	C7orf28A	0.47479	0.61279	-0.138	0.907
726	201975_at	CLIP1	0.64111	0.59507	0.04604	0.215
727	201977_s_at	KIAA0141	0.29116	0.32683	-0.03567	0.597
728	201985_at	KIAA0196	0.40712	0.027386	0.379734	<0.001
729	201986_at	MED13	0.70374	0.72186	-0.01812	0.594
730	201990_s_at	CREBL2	0.73176	0.62529	0.10647	0.1
731	201991_s_at	KIF5B	0.8623	0.80473	0.05757	0.094
732	201994_at	MORF4L2	0.68224	0.42043	0.26181	<0.001
733	201997_s_at	SPEN	0.23736	0.30752	-0.07016	0.709
734	201999_s_at	DYNLT1	0.76567	0.75932	0.00635	0.465
735	202001_s_at	NDUFA6	0.37737	0.35134	0.02603	0.429
736	202007_at	NID1	0.50405	0.62554	-0.12149	0.846
737	202009_at	TWF2	0.63021	0.40211	0.2281	0.026
738	202010_s_at	ZNF410	0.61069	0.70096	-0.09027	0.856
739	202012_s_at	EXT2	0.64743	0.7188	-0.07137	0.818
740	202016_at	MEST	3.15E-05	0.1601	-0.160068512	0.894
741	202019_s_at	LANCL1	0.59408	0.70816	-0.11408	0.869
742	202022_at	ALDOC	0.58612	0.61157	-0.02545	0.608
743	202024_at	ASNA1	0.78078	0.83294	-0.05216	0.809
744	202026_at	SDHD	0.84255	0.85922	-0.01667	0.59
745	202027_at	TMEM184B	0.076963	0.089837	-0.012874	0.56
746	202028_s_at	RPL38	0.56411	0.71748	-0.15337	0.978
747	202030_at	BCKDK	0.092221	0.45794	-0.365719	1
748	202033_s_at	RB1CC1	0.52445	0.68528	-0.16083	0.952
749	202038_at	UBE4A	0.46289	0.3346	0.12829	0.179
750	202039_at	MYO18A	0.75821	0.71516	0.04305	0.337
751	202040_s_at	KDM5A	0.73279	0.76186	-0.02907	0.698
752	202041_s_at	FIBP	0.53471	0.62084	-0.08613	0.793
753	202042_at	HARS	0.81459	0.67891	0.13568	0.024
754	202043_s_at	SMS	0.75758	0.74476	0.01282	0.412

755	202045_s_at	GRLF1	0.38956	0.52721	-0.13765	0.907
756	202048_s_at	CBX6	0.50973	0.72907	-0.21934	0.99
757	202050_s_at	ZMYM4	0.61268	0.6618	-0.04912	0.759
758	202053_s_at	ALDH3A2	0.45392	0.48378	-0.02986	0.573
759	202055_at	KPNA1	0.36241	0.29682	0.06559	0.292
760	202060_at	CTR9	0.76695	0.67861	0.08834	0.099
761	202064_s_at	SEL1L	0.28248	0.13767	0.14481	0.009
762	202069_s_at	IDH3A	0.73152	0.74597	-0.01445	0.566
763	202071_at	SDC4	0.59471	0.65951	-0.0648	0.756
764	202072_at	HNRNPL	0.47881	0.59126	-0.11245	0.907
765	202074_s_at	OPTN	0.42025	0.42348	-0.00323	0.523
766	202075_s_at	PLTP	0.044903	0.24221	-0.197307	0.98
767	202076_at	BIRC2	0.76317	0.69101	0.07216	0.124
768	202077_at	NDUFAB1	0.8563	0.83074	0.02556	0.135
769	202078_at	COPS3	0.89244	0.86012	0.03232	0.125
770	202080_s_at	TRAK1	0.63148	0.6818	-0.05032	0.762
771	202081_at	IER2	0.41859	0.34026	0.07833	0.171
772	202083_s_at	SEC14L1	0.42779	0.57117	-0.14338	0.912
773	202085_at	TJP2	0.63763	0.59907	0.03856	0.326
774	202086_at	MX1	0.64256	0.56502	0.07754	0.177
775	202089_s_at	SLC39A6	0.9069	0.83856	0.06834	0.006
776	202090_s_at	UQCR	0.77919	0.65262	0.12657	0.027
777	202092_s_at	ARL2BP	0.79225	0.68238	0.10987	0.039
778	202093_s_at	PAF1	0.66122	0.4711	0.19012	0.006
779	202095_s_at	BIRC5	0.67952	0.57884	0.10068	0.075
780	202096_s_at	TSPO	0.26624	0.4828	-0.21656	0.949
781	202097_at	NUP153	0.51718	0.47529	0.04189	0.323
782	202100_at	RALB	0.42597	0.63144	-0.20547	0.976
783	202103_at	BRD4	0.57472	0.52197	0.05275	0.294
784	202104_s_at	SPG7	0.50807	0.56019	-0.05212	0.721
785	202105_at	IGBP1	0.72519	0.66257	0.06262	0.143
786	202106_at	GOLGA3	0.31101	0.46138	-0.15037	0.937
787	202107_s_at	MCM2	0.82929	0.74997	0.07932	0.002
788	202108_at	PEPD	0.26772	0.19027	0.07745	0.232
789	202109_at	ARFIP2	0.63343	0.29388	0.33955	0.001
790	202110_at	COX7B	0.82388	0.63505	0.18883	<0.001
791	202111_at	SLC4A2	0.36081	0.44577	-0.08496	0.808
792	202113_s_at	SNX2	0.75764	0.75361	0.00403	0.506
793	202115_s_at	NOC2L	0.43922	0.04259	0.39663	<0.001
794	202116_at	DPF2	0.48844	0.46474	0.0237	0.396
795	202117_at	ARHGAP1	0.56505	0.28117	0.28388	0.01
796	202119_s_at	CPNE3	0.59904	0.54102	0.05802	0.21
797	202121_s_at	CHMP2A	0.74714	0.79981	-0.05267	0.808
798	202122_s_at	M6PRBP1	0.44987	0.48539	-0.03552	0.655
799	202123_s_at	ABL1	0.33834	0.46786	-0.12952	0.914
800	202124_s_at	TRAK2	0.6887	0.54853	0.14017	0.016
801	202126_at	PRPF4B	0.46604	0.40884	0.0572	0.136
802	202128_at	KIAA0317	0.64496	0.68531	-0.04035	0.698
803	202129_s_at	RIOK3	0.84066	0.84485	-0.00419	0.561
804	202135_s_at	ACTR1B	0.69344	0.60347	0.08997	0.105
805	202136_at	ZMYND11	0.77384	0.69643	0.07741	0.107
806	202139_at	AKR7A2	0.52109	0.58722	-0.06613	0.719
807	202140_s_at	CLK3	0.61891	0.45425	0.16466	0.044
808	202143_s_at	COPS8	0.68359	0.70483	-0.02124	0.611

809	202144_s_at	ADSL	0.8586	0.78118	0.07742	<0.001
810	202145_at	LY6E	0.69738	0.72293	-0.02555	0.698
811	202146_at	IFRD1	0.30142	0.42175	-0.12033	0.971
812	202148_s_at	PYCR1	0.19593	0.22596	-0.03003	0.649
813	202149_at	NEDD9	0.76939	0.73817	0.03122	0.302
814	202151_s_at	UBAC1	0.32467	0.15341	0.17126	0.026
815	202161_at	PKN1	0.40889	0.49232	-0.08343	0.794
816	202162_s_at	CNOT8	0.26397	0.21079	0.05318	0.36
817	202166_s_at	PPP1R2	0.48416	0.5376	-0.05344	0.675
818	202167_s_at	MMS19	0.66286	0.44401	0.21885	0.005
819	202168_at	TAF9	0.60952	0.67905	-0.06953	0.791
820	202170_s_at	AASDHPPT	0.72784	0.66787	0.05997	0.207
821	202173_s_at	VEZF1	0.86123	0.83787	0.02336	0.307
822	202174_s_at	PCM1	0.48935	0.48882	0.00053	0.495
823	202175_at	CHPF	0.43042	0.42473	0.00569	0.463
824	202179_at	BLMH	0.74443	0.65463	0.0898	0.064
825	202180_s_at	MVP	0.67946	0.57964	0.09982	0.154
826	202181_at	KIAA0247	0.74854	0.68822	0.06032	0.138
827	202182_at	KAT2A	0.30854	0.14775	0.16079	0.047
828	202184_s_at	NUP133	0.83312	0.60497	0.22815	<0.001
829	202185_at	PLOD3	0.51009	0.35688	0.15321	0.086
830	202187_s_at	PPP2R5A	0.34005	0.37215	-0.0321	0.606
831	202188_at	NUP93	0.58026	0.5644	0.01586	0.43
832	202190_at	CSTF1	0.54979	0.40571	0.14408	0.024
833	202191_s_at	GAS7	0.43168	0.58628	-0.1546	0.914
834	202193_at	LIMK2	0.32821	0.21958	0.10863	0.162
835	202195_s_at	TMED5	0.48486	0.451	0.03386	0.362
836	202200_s_at	SRPK1	0.71384	0.56993	0.14391	<0.001
837	202201_at	BLVRB	0.60725	0.62472	-0.01747	0.55
838	202204_s_at	AMFR	0.38263	0.48745	-0.10482	0.828
839	202205_at	VASP	0.55214	0.48751	0.06463	0.285
840	202209_at	LSM3	0.82509	0.78514	0.03995	0.068
841	202211_at	ARFGAP3	0.65754	0.53052	0.12702	<0.001
842	202212_at	PES1	0.62257	0.45039	0.17218	0.01
843	202213_s_at	CUL4B	0.53771	0.61996	-0.08225	0.791
844	202215_s_at	NFYC	0.21871	0.31755	-0.09884	0.817
845	202217_at	C21orf33	0.74148	0.74131	0.00017	0.488
846	202218_s_at	FADS2	0.45123	0.4282	0.02303	0.4
847	202220_at	KIAA0907	0.41752	0.18207	0.23545	0.012
848	202221_s_at	EP300	0.57802	0.50749	0.07053	0.298
849	202223_at	STT3A	0.46175	0.44441	0.01734	0.389
850	202225_at	CRK	0.36727	0.31217	0.0551	0.33
851	202227_s_at	BRD8	0.63045	0.56009	0.07036	0.263
852	202228_s_at	NPTN	0.57808	0.55307	0.02501	0.431
853	202230_s_at	CHERP	0.69013	0.62683	0.0633	0.106
854	202233_s_at	UQCRH	0.59028	0.71128	-0.121	0.933
855	202234_s_at	SLC16A1	0.62763	0.53257	0.09506	0.106
856	202239_at	PARP4	0.029153	0.16295	-0.133797	0.878
857	202240_at	PLK1	0.65092	0.76051	-0.10959	0.956
858	202241_at	TRIB1	0.66576	0.77389	-0.10813	0.908
859	202243_s_at	PSMB4	0.89958	0.90399	-0.00441	0.573
860	202246_s_at	CDK4	0.84697	0.88297	-0.036	0.835
861	202249_s_at	WDR42A	0.52317	0.38846	0.13471	0.113
862	202251_at	PRPF3	0.45249	0.49684	-0.04435	0.685

863	202252_at	RAB13	0.36335	0.50176	-0.13841	0.785
864	202253_s_at	DNM2	0.55405	0.46259	0.09146	0.214
865	202254_at	SIPA1L1	0.5837	0.66649	-0.08279	0.784
866	202257_s_at	CD2BP2	0.59479	0.46676	0.12803	0.107
867	202261_at	VPS72	0.65143	0.56831	0.08312	0.236
868	202263_at	CYB5R1	0.78415	0.73224	0.05191	0.188
869	202264_s_at	TOMM40	0.75137	0.54897	0.2024	<0.001
870	202265_at	BMI1	0.66329	0.4947	0.16859	0.025
871	202266_at	TTRAP	0.44103	0.48751	-0.04648	0.674
872	202268_s_at	NAE1	0.61587	0.69337	-0.0775	0.972
873	202270_at	GBP1	0.46289	0.42952	0.03337	0.426
874	202272_s_at	FBXO28	0.43056	0.44422	-0.01366	0.559
875	202275_at	G6PD	0.51909	0.48268	0.03641	0.385
876	202276_at	SHFM1	0.53742	0.66061	-0.12319	0.93
877	202277_at	SPTLC1	0.58536	0.54879	0.03657	0.373
878	202279_at	C14orf2	0.59639	0.5209	0.07549	0.171
879	202282_at	HSD17B10	0.46049	0.61671	-0.15622	0.882
880	202284_s_at	CDKN1A	0.8063	0.80731	-0.00101	0.517
881	202288_at	FRAP1	0.28365	0.33826	-0.05461	0.712
882	202294_at	STAG1	0.11547	0.25908	-0.14361	0.915
883	202295_s_at	CTSH	0.11112	0.3559	-0.24478	0.973
884	202297_s_at	RER1	0.60539	0.33426	0.27113	<0.001
885	202298_at	NDUFA1	0.68759	0.56562	0.12197	0.073
886	202300_at	HBXIP	0.6973	0.69975	-0.00245	0.53
887	202301_s_at	RSRC2	0.88338	0.81357	0.06981	0.023
888	202306_at	POLR2G	0.64311	0.73786	-0.09475	0.877
889	202307_s_at	TAP1	0.70271	0.68055	0.02216	0.42
890	202308_at	SREBF1	0.60399	0.56531	0.03868	0.354
891	202309_at	MTHFD1	0.075105	0.097408	-0.022303	0.61
892	202313_at	PPP2R2A	0.69423	0.56941	0.12482	0.049
893	202321_at	GGPS1	0.38356	0.45106	-0.0675	0.726
894	202323_s_at	ACBD3	0.77558	0.8025	-0.02692	0.73
895	202325_s_at	ATP5J	0.74417	0.60305	0.14112	0.016
896	202328_s_at	PKD1	0.27647	0.23231	0.04416	0.375
897	202329_at	CSK	0.66699	0.42496	0.24203	0.007
898	202330_s_at	UNG	0.6349	0.65715	-0.02225	0.747
899	202331_at	BCKDHA	0.40096	0.50039	-0.09943	0.8
900	202336_s_at	PAM	0.45195	0.52599	-0.07404	0.788
901	202337_at	PMF1	0.5019	0.51071	-0.00881	0.572
902	202338_at	TK1	0.77756	0.75489	0.02267	0.334
903	202339_at	SYMPK	0.63296	0.48689	0.14607	0.031
904	202344_at	HSF1	0.20164	0.18414	0.0175	0.433
905	202345_s_at	FABP5	0.68446	0.59073	0.09373	0.011
906	202347_s_at	UBE2K	0.59397	0.43048	0.16349	0.002
907	202349_at	TOR1A	0.083193	0.18683	-0.103637	0.735
908	202351_at	ITGAV	0.5051	0.42194	0.08316	0.265
909	202352_s_at	PSMD12	0.75938	0.66882	0.09056	0.186
910	202355_s_at	GTF2F1	0.35282	0.4021	-0.04928	0.654
911	202359_s_at	SNX19	0.4813	0.40485	0.07645	0.222
912	202360_at	MAML1	0.62379	0.75886	-0.13507	0.952
913	202361_at	SEC24C	0.52286	0.49833	0.02453	0.412
914	202362_at	RAP1A	0.4952	0.67719	-0.18199	0.978
915	202364_at	MXI1	0.65368	0.64598	0.0077	0.483
916	202365_at	UNC119B	0.66262	0.61094	0.05168	0.326

917	202366_at	ACADS	0.1431	0.23809	-0.09499	0.762
918	202367_at	CUX1	0.62023	0.59765	0.02258	0.411
919	202369_s_at	TRAM2	0.52735	0.58704	-0.05969	0.785
920	202371_at	TCEAL4	0.17997	0.3912	-0.21123	0.866
921	202378_s_at	LEPROT	0.67661	0.75731	-0.0807	0.951
922	202381_at	ADAM9	0.66847	0.62606	0.04241	0.316
923	202382_s_at	GNPDA1	0.57234	0.70081	-0.12847	0.88
924	202383_at	KDM5C	0.40512	0.40691	-0.00179	0.503
925	202384_s_at	TCOF1	0.78446	0.46382	0.32064	<0.001
926	202386_s_at	KIAA0430	0.48777	0.44292	0.04485	0.334
927	202388_at	RGS2	0.59321	0.63438	-0.04117	0.642
928	202390_s_at	HTT	0.28889	0.54056	-0.25167	0.99
929	202391_at	BASP1	0.4516	0.51624	-0.06464	0.785
930	202392_s_at	PISD	0.17654	0.18001	-0.00347	0.519
931	202393_s_at	KLF10	0.53207	0.52091	0.01116	0.446
932	202394_s_at	ABCF3	0.4257	0.4755	-0.0498	0.655
933	202395_at	NSF	0.72742	0.70421	0.02321	0.381
934	202396_at	TCERG1	0.6657	0.55656	0.10914	0.074
935	202397_at	NUTF2	0.70856	0.50179	0.20677	0.008
936	202399_s_at	AP3S2	0.4686	0.5153	-0.0467	0.738
937	202402_s_at	CARS	0.2167	0.1774	0.0393	0.322
938	202406_s_at	TIAL1	0.68119	0.51214	0.16905	0.015
939	202413_s_at	USP1	0.92487	0.88461	0.04026	0.189
940	202414_at	ERCC5	0.38089	0.53314	-0.15225	0.997
941	202415_s_at	HSPBP1	0.61629	0.69578	-0.07949	0.78
942	202417_at	KEAP1	0.70031	0.65933	0.04098	0.293
943	202418_at	YIF1A	0.68637	0.78402	-0.09765	0.901
944	202419_at	KDSR	0.45835	0.54965	-0.0913	0.806
945	202420_s_at	DHX9	0.66884	0.4141	0.25474	<0.001
946	202421_at	IGSF3	0.54952	0.6788	-0.12928	0.884
947	202422_s_at	ACSL4	0.73782	0.75069	-0.01287	0.587
948	202424_at	MAP2K2	0.73818	0.79466	-0.05648	0.887
949	202427_s_at	BRP44	0.66857	0.78292	-0.11435	0.965
950	202431_s_at	MYC	0.6325	0.43898	0.19352	0.014
951	202432_at	PPP3CB	0.53517	0.46683	0.06834	0.239
952	202433_at	SLC35B1	0.70149	0.67342	0.02807	0.386
953	202435_s_at	CYP1B1	0.49596	0.5328	-0.03684	0.672
954	202439_s_at	IDS	0.4474	0.79488	-0.34748	1
955	202444_s_at	ERLIN1	0.48317	0.41687	0.0663	0.205
956	202446_s_at	PLSCR1	0.7924	0.7177	0.0747	0.16
957	202447_at	DECR1	0.47004	0.4398	0.03024	0.384
958	202449_s_at	RXRA	0.42612	0.31776	0.10836	0.196
959	202450_s_at	CTSK	0.32164	0.36177	-0.04013	0.618
960	202451_at	GTF2H1	0.70802	0.56034	0.14768	0.066
961	202457_s_at	PPP3CA	0.611	0.61065	0.00035	0.504
962	202459_s_at	LPIN2	0.6222	0.5024	0.1198	0.068
963	202461_at	EIF2B2	0.32827	0.55029	-0.22202	0.977
964	202462_s_at	DDX46	0.55624	0.39445	0.16179	0.033
965	202466_at	POLS	0.47787	0.28043	0.19744	0.024
966	202468_s_at	CTNNAL1	0.60675	0.56357	0.04318	0.34
967	202470_s_at	CPSF6	0.66069	0.57228	0.08841	0.16
968	202471_s_at	IDH3G	0.77047	0.83551	-0.06504	0.883
969	202472_at	MPI	0.5113	0.35322	0.15808	0.066
970	202474_s_at	HCFC1	0.49381	0.49073	0.00308	0.485

971	202475_at	TMEM147	0.64706	0.64554	0.00152	0.506
972	202480_s_at	DEDD	0.25257	0.10991	0.14266	0.12
973	202486_at	AFG3L2	0.39206	0.37747	0.01459	0.484
974	202487_s_at	H2AFV	0.74487	0.83776	-0.09289	0.884
975	202488_s_at	FXYD3	0.5666	0.30397	0.26263	0.019
976	202490_at	IKBKAP	0.16545	0.047238	0.118212	0.151
977	202492_at	ATG9A	0.47408	0.38177	0.09231	0.163
978	202495_at	TBCC	0.65287	0.60594	0.04693	0.298
979	202496_at	EDC4	0.2393	0.30035	-0.06105	0.732
980	202499_s_at	SLC2A3	0.65487	0.64758	0.00729	0.481
981	202500_at	DNAJB2	0.44371	0.44405	-0.00034	0.491
982	202502_at	ACADM	0.41125	0.49247	-0.08122	0.752
983	202503_s_at	KIAA0101	0.84604	0.78571	0.06033	0.068
984	202505_at	SNRPB2	0.80788	0.80045	0.00743	0.465
985	202506_at	SSFA2	0.74037	0.81524	-0.07487	0.876
986	202510_s_at	TNFAIP2	0.67533	0.72196	-0.04663	0.767
987	202513_s_at	PPP2R5D	0.040397	0.015454	0.024943	0.452
988	202514_at	DLG1	0.39221	0.32107	0.07114	0.317
989	202518_at	BCL7B	0.57854	0.52966	0.04888	0.329
990	202520_s_at	MLH1	0.62058	0.61438	0.0062	0.497
991	202521_at	CTCF	0.80893	0.66058	0.14835	<0.001
992	202522_at	PITPNB	0.66439	0.68717	-0.02278	0.591
993	202527_s_at	SMAD4	0.68564	0.57937	0.10627	0.033
994	202528_at	GALE	0.28447	0.069474	0.214996	0.049
995	202529_at	PRPSAP1	0.41698	0.28415	0.13283	0.197
996	202530_at	MAPK14	0.64446	0.70181	-0.05735	0.724
997	202531_at	IRF1	0.65561	0.54718	0.10843	0.238
998	202532_s_at	DHFR	0.88422	0.80094	0.08328	0.001
999	202535_at	FADD	0.50719	0.46248	0.04471	0.151
1000	202538_s_at	CHMP2B	0.40862	0.2727	0.13592	0.171
1001	202540_s_at	HMGCR	0.61502	0.59503	0.01999	0.438
1002	202541_at	SCYE1	0.57236	0.46615	0.10621	0.153
1003	202543_s_at	GMFB	0.75564	0.73236	0.02328	0.396
1004	202545_at	PRKCD	0.61889	0.42606	0.19283	0.004
1005	202546_at	VAMP8	0.71611	0.72016	-0.00405	0.522
1006	202548_s_at	ARHGEF7	0.71376	0.64059	0.07317	0.153
1007	202550_s_at	VAPB	0.087502	0.0812	0.006302	0.49
1008	202552_s_at	CRIM1	0.59694	0.70698	-0.11004	0.88
1009	202553_s_at	SYF2	0.67414	0.50458	0.16956	0.006
1010	202556_s_at	MCRS1	0.57553	0.70989	-0.13436	0.883
1011	202557_at	HSPA13	0.61388	0.58377	0.03011	0.302
1012	202567_at	SNRPD3	0.82837	0.75353	0.07484	0.06
1013	202569_s_at	MARK3	0.40796	0.32677	0.08119	0.254
1014	202573_at	CSNK1G2	0.32681	0.44364	-0.11683	0.881
1015	202578_s_at	DDX19A	0.73458	0.6731	0.06148	0.27
1016	202581_at	HSPA1A	0.59965	0.40822	0.19143	0.025
1017	202583_s_at	RANBP9	0.7803	0.66092	0.11938	0.129
1018	202584_at	NFX1	0.35574	0.35756	-0.00182	0.497
1019	202586_at	POLR2L	0.22395	0.43267	-0.20872	0.966
1020	202587_s_at	AK1	0.26644	0.41106	-0.14462	0.908
1021	202592_at	BLOC1S1	0.45588	0.56721	-0.11133	0.851
1022	202593_s_at	GDE1	0.69637	0.58147	0.1149	0.044
1023	202594_at	LEPROTL1	0.58349	0.67546	-0.09197	0.841
1024	202600_s_at	NRIP1	0.404	0.44034	-0.03634	0.68

1025	202602_s_at	HTATSF1	0.69698	0.73933	-0.04235	0.716
1026	202605_at	GUSB	0.80368	0.75712	0.04656	0.173
1027	202609_at	EPS8	0.6079	0.77989	-0.17199	0.978
1028	202613_at	CTPS	0.68439	0.61212	0.07227	0.006
1029	202614_at	SLC30A9	0.49849	0.36224	0.13625	0.09
1030	202617_s_at	MECP2	0.10317	0.14289	-0.03972	0.626
1031	202620_s_at	PLOD2	0.07878	0.31865	-0.23987	0.979
1032	202621_at	IRF3	0.41523	0.52941	-0.11418	0.853
1033	202622_s_at	ATXN2	0.40091	0.45365	-0.05274	0.663
1034	202623_at	EAPP	0.76854	0.73887	0.02967	0.343
1035	202624_s_at	CABIN1	0.74942	0.723	0.02642	0.325
1036	202625_at	LYN	0.52255	0.64122	-0.11867	0.852
1037	202631_s_at	APPBP2	0.70796	0.67838	0.02958	0.305
1038	202632_at	DPH1	0.35277	0.54191	-0.18914	0.937
1039	202633_at	TOPBP1	0.74433	0.75007	-0.00574	0.567
1040	202635_s_at	POLR2K	0.82274	0.67235	0.15039	<0.001
1041	202636_at	RNF103	0.54129	0.63546	-0.09417	0.771
1042	202640_s_at	RANBP3	0.48226	0.48136	0.0009	0.488
1043	202643_s_at	TNFAIP3	0.80861	0.81625	-0.00764	0.59
1044	202645_s_at	MEN1	0.46433	0.54449	-0.08016	0.791
1045	202647_s_at	NRAS	0.38807	0.54293	-0.15486	0.85
1046	202650_s_at	KIAA0195	0.52426	0.6037	-0.07944	0.864
1047	202651_at	LPGAT1	0.31203	0.55092	-0.23889	0.986
1048	202653_s_at	MARCH7	0.63927	0.75442	-0.11515	0.873
1049	202655_at	ARMET	0.70489	0.51685	0.18804	0.017
1050	202657_s_at	SERTAD2	0.73309	0.69689	0.0362	0.351
1051	202658_at	PEX11B	0.56772	0.66702	-0.0993	0.832
1052	202659_at	PSMB10	0.82405	0.75137	0.07268	0.223
1053	202662_s_at	ITPR2	0.40141	0.51994	-0.11853	0.898
1054	202665_s_at	WIPF1	0.65329	0.73876	-0.08547	0.878
1055	202666_s_at	ACTL6A	0.80974	0.85363	-0.04389	0.819
1056	202667_s_at	SLC39A7	0.42878	0.35687	0.07191	0.145
1057	202670_at	MAP2K1	0.33193	0.53338	-0.20145	0.957
1058	202672_s_at	ATF3	0.77673	0.66902	0.10771	0.048
1059	202673_at	DPM1	0.56767	0.5974	-0.02973	0.597
1060	202677_at	RASA1	0.58272	0.67137	-0.08865	0.86
1061	202678_at	GTF2A2	0.68884	0.59682	0.09202	0.165
1062	202680_at	GTF2E2	0.77349	0.78346	-0.00997	0.548
1063	202681_at	USP4	0.40511	0.4993	-0.09419	0.85
1064	202683_s_at	RNMT	0.61576	0.5474	0.06836	0.198
1065	202688_at	TNFSF10	0.64157	0.54294	0.09863	0.201
1066	202689_at	RBM15B	0.1466	0.37418	-0.22758	0.984
1067	202691_at	SNRPD1	0.81408	0.82271	-0.00863	0.606
1068	202693_s_at	STK17A	0.53579	0.76174	-0.22595	0.997
1069	202696_at	OXSR1	0.64338	0.68613	-0.04275	0.696
1070	202702_at	TRIM26	0.37876	0.424	-0.04524	0.616
1071	202703_at	DUSP11	0.40373	0.52294	-0.11921	0.806
1072	202704_at	TOB1	0.61782	0.61942	-0.0016	0.538
1073	202705_at	CCNB2	0.66209	0.79294	-0.13085	0.872
1074	202708_s_at	HIST2H2BE	0.46529	0.33692	0.12837	0.116
1075	202709_at	FMOD	0.19912	0.10477	0.09435	0.219
1076	202710_at	BET1	0.4675	0.60095	-0.13345	0.926
1077	202713_s_at	KIAA0391	0.36762	0.48339	-0.11577	0.867
1078	202715_at	CAD	0.68201	0.37475	0.30726	<0.001

1079	202716_at	PTPN1	0.38853	0.51979	-0.13126	0.893
1080	202720_at	TES	0.5233	0.43325	0.09005	0.198
1081	202721_s_at	GFPT1	0.37604	0.24827	0.12777	0.029
1082	202724_s_at	FOXO1	0.12419	0.22631	-0.10212	0.764
1083	202725_at	POLR2A	0.21894	0.49319	-0.27425	1
1084	202726_at	LIG1	0.56946	0.53663	0.03283	0.294
1085	202730_s_at	PDCD4	0.6501	0.55664	0.09346	0.088
1086	202732_at	PKIG	0.58987	0.65055	-0.06068	0.803
1087	202733_at	P4HA2	0.45748	0.62	-0.16252	0.97
1088	202734_at	TRIP10	0.63921	0.51518	0.12403	0.148
1089	202736_s_at	LSM4	0.74622	0.84685	-0.10063	0.956
1090	202738_s_at	PHKB	0.77669	0.73598	0.04071	0.242
1091	202740_at	ACY1	0.72458	0.68752	0.03706	0.328
1092	202742_s_at	PRKACB	0.6653	0.67117	-0.00587	0.53
1093	202743_at	PIK3R3	0.39005	0.50819	-0.11814	0.829
1094	202745_at	USP8	0.58344	0.64538	-0.06194	0.767
1095	202746_at	ITM2A	0.054366	0.26272	-0.208354	0.891
1096	202748_at	GBP2	0.34427	0.42281	-0.07854	0.751
1097	202749_at	WRB	0.30879	0.58049	-0.2717	0.972
1098	202750_s_at	TFIP11	0.16293	0.26467	-0.10174	0.802
1099	202753_at	PSMD6	0.85318	0.87304	-0.01986	0.678
1100	202754_at	R3HDM1	0.54587	0.47192	0.07395	0.248
1101	202757_at	COBRA1	0.21756	0.36288	-0.14532	0.874
1102	202758_s_at	RFXANK	0.38862	0.24174	0.14688	0.07
1103	202759_s_at	AKAP2	0.62203	0.64614	-0.02411	0.609
1104	202760_s_at	PALM2-AKAP2	0.54294	0.5869	-0.04396	0.657
1105	202761_s_at	SYNE2	0.68149	0.73699	-0.0555	0.737
1106	202762_at	ROCK2	0.11983	0.41576	-0.29593	0.988
1107	202763_at	CASP3	0.54642	0.57298	-0.02656	0.603
1108	202764_at	STIM1	0.27462	0.29606	-0.02144	0.586
1109	202765_s_at	FBN1	0.65662	0.78792	-0.1313	0.982
1110	202767_at	ACP2	0.47347	0.53284	-0.05937	0.73
1111	202769_at	CCNG2	0.78092	0.52071	0.26021	0.002
1112	202771_at	FAM38A	0.53277	0.51794	0.01483	0.433
1113	202772_at	HMGCL	0.68139	0.58395	0.09744	0.055
1114	202775_s_at	SFRS8	0.37961	0.23467	0.14494	0.101
1115	202776_at	DNTTIP2	0.30609	0.23801	0.06808	0.362
1116	202777_at	SHOC2	0.44055	0.56342	-0.12287	0.943
1117	202779_s_at	LOC731049	0.78	0.83498	-0.05498	0.833
1118	202780_at	OXCT1	0.70461	0.70503	-0.00042	0.524
1119	202781_s_at	INPP5K	0.3034	0.00081544	0.30258456	0.005
1120	202783_at	NNT	0.23651	0.38498	-0.14847	0.91
1121	202785_at	NDUFA7	0.81191	0.64022	0.17169	0.005
1122	202786_at	STK39	0.13511	0.23576	-0.10065	0.74
1123	202787_s_at	MAPKAPK3	0.46143	0.42665	0.03478	0.425
1124	202789_at	PLCG1	0.52319	0.50588	0.01731	0.405
1125	202793_at	LPCAT3	0.36489	0.30579	0.0591	0.29
1126	202794_at	INPP1	0.55439	0.54542	0.00897	0.465
1127	202796_at	SYNPO	0.70215	0.70262	-0.00047	0.538
1128	202797_at	SACM1L	0.8858	0.77602	0.10978	0.004
1129	202798_at	SEC24B	0.50511	0.51675	-0.01164	0.541
1130	202799_at	CLPP	0.80723	0.7841	0.02313	0.313
1131	202803_s_at	ITGB2	0.45798	0.43386	0.02412	0.409
1132	202804_at	ABCC1	0.51178	0.53961	-0.02783	0.607

1133	202808_at	C10orf26	0.4026	0.53509	-0.13249	0.883
1134	202809_s_at	INTS3	0.49156	0.42948	0.06208	0.212
1135	202810_at	DRG1	0.8761	0.89897	-0.02287	0.694
1136	202811_at	STAMBP	0.59508	0.65934	-0.06426	0.815
1137	202812_at	GAA	0.25058	0.37705	-0.12647	0.845
1138	202813_at	TARBP1	0.61182	0.75155	-0.13973	0.961
1139	202815_s_at	HEXIM1	0.34873	0.48257	-0.13384	0.886
1140	202820_at	AHR	0.357	0.55401	-0.19701	0.961
1141	202824_s_at	TCEB1	0.88508	0.8648	0.02028	0.372
1142	202825_at	SLC25A4	0.47673	0.52623	-0.0495	0.666
1143	202829_s_at	VAMP7	0.59049	0.70866	-0.11817	0.881
1144	202832_at	GCC2	0.56341	0.54977	0.01364	0.391
1145	202837_at	TRAFD1	0.66974	0.49448	0.17526	0.02
1146	202838_at	FUCA1	0.66875	0.72389	-0.05514	0.772
1147	202839_s_at	NDUFB7	0.68826	0.53911	0.14915	0.005
1148	202840_at	TAF15	0.53581	0.30174	0.23407	0.028
1149	202843_at	DNAJB9	0.67886	0.44468	0.23418	<0.001
1150	202844_s_at	RALBP1	0.75709	0.80946	-0.05237	0.796
1151	202847_at	PCK2	0.63639	0.34943	0.28696	<0.001
1152	202850_at	ABCD3	0.44958	0.51135	-0.06177	0.748
1153	202853_s_at	RYK	0.44389	0.6304	-0.18651	0.966
1154	202854_at	HPRT1	0.78105	0.66945	0.1116	<0.001
1155	202856_s_at	SLC16A3	0.6169	0.67857	-0.06167	0.738
1156	202858_at	U2AF1	0.44659	0.59869	-0.1521	0.884
1157	202860_at	DENND4B	0.36621	0.63495	-0.26874	0.992
1158	202862_at	FAH	0.39342	0.46656	-0.07314	0.749
1159	202868_s_at	POP4	0.8425	0.75098	0.09152	0.074
1160	202870_s_at	CDC20	0.87552	0.80705	0.06847	0.083
1161	202871_at	TRAF4	0.39126	0.54634	-0.15508	0.944
1162	202874_s_at	ATP6V1C1	0.73351	0.66229	0.07122	0.072
1163	202876_s_at	PBX2	0.6246	0.60103	0.02357	0.372
1164	202887_s_at	DDIT4	0.35657	0.20199	0.15458	0.043
1165	202891_at	NIT1	0.6885	0.67893	0.00957	0.475
1166	202892_at	CDC23	0.61368	0.55318	0.0605	0.308
1167	202897_at	SIRPA	0.39161	0.4071	-0.01549	0.562
1168	202899_s_at	SFRS3	0.84474	0.85522	-0.01048	0.666
1169	2028_s_at	E2F1	0.59952	0.53564	0.06388	0.17
1170	202900_s_at	NUP88	0.78369	0.76211	0.02158	0.406
1171	202902_s_at	CTSS	0.76524	0.70967	0.05557	0.199
1172	202904_s_at	LSM5	0.74187	0.57914	0.16273	0.023
1173	202908_at	WFS1	0.055366	0.2198	-0.164434	0.997
1174	202909_at	EPM2AIP1	0.54865	0.78627	-0.23762	0.994
1175	202910_s_at	CD97	0.48624	0.39497	0.09127	0.184
1176	202912_at	ADM	0.58226	0.57863	0.00363	0.477
1177	202915_s_at	FAM20B	0.61366	0.55292	0.06074	0.252
1178	202919_at	MOBKL3	0.23476	0.18431	0.05045	0.332
1179	202922_at	GCLC	0.45666	0.37461	0.08205	0.262
1180	202925_s_at	PLAGL2	0.47044	0.3734	0.09704	0.121
1181	202926_at	NBAS	0.24218	0.1183	0.12388	0.097
1182	202927_at	PIN1	0.71573	0.5279	0.18783	<0.001
1183	202929_s_at	DDT	0.63685	0.71388	-0.07703	0.88
1184	202930_s_at	SUCLA2	0.76028	0.83535	-0.07507	0.917
1185	202935_s_at	SOX9	0.59212	0.64333	-0.05121	0.708
1186	202939_at	ZMPSTE24	0.4928	0.4171	0.0757	0.264

1187	202941_at	NDUFV2	0.89591	0.8281	0.06781	0.064
1188	202942_at	ETFB	0.587	0.68053	-0.09353	0.88
1189	202943_s_at	NAGA	0.54895	0.67829	-0.12934	0.858
1190	202945_at	FPGS	0.073781	0.074278	-0.000497	0.483
1191	202946_s_at	BTBD3	0.18648	0.31404	-0.12756	0.836
1192	202947_s_at	GYPC	0.65818	0.66264	-0.00446	0.53
1193	202949_s_at	FHL2	0.53472	0.65327	-0.11855	0.859
1194	202950_at	CRYZ	0.43535	0.38102	0.05433	0.296
1195	202954_at	UBE2C	0.78293	0.7846	-0.00167	0.526
1196	202957_at	HCLS1	0.5446	0.58422	-0.03962	0.646
1197	202958_at	PTPN9	0.29307	0.31842	-0.02535	0.617
1198	202960_s_at	MUT	0.22922	0.33793	-0.10871	0.815
1199	202961_s_at	ATP5J2	0.71393	0.53857	0.17536	0.004
1200	202962_at	KIF13B	0.38128	0.36714	0.01414	0.449
1201	202963_at	RFX5	0.52111	0.4931	0.02801	0.398
1202	202967_at	GSTA4	0.32271	0.39974	-0.07703	0.732
1203	202968_s_at	DYRK2	0.41538	0.60893	-0.19355	0.966
1204	202974_at	MPP1	0.35211	0.48313	-0.13102	0.871
1205	202976_s_at	RHOBTB3	0.12641	0.47055	-0.34414	0.994
1206	202980_s_at	SIAH1	0.29945	0.40377	-0.10432	0.803
1207	202982_s_at	ACOT1	0.029315	0.22951	-0.200195	0.833
1208	202983_at	HLTF	0.46491	0.58442	-0.11951	0.89
1209	202984_s_at	BAG5	0.69764	0.70204	-0.0044	0.521
1210	202987_at	TRAF3IP2	0.27799	0.29101	-0.01302	0.588
1211	202991_at	STARD3	0.57046	0.30543	0.26503	0.006
1212	202996_at	POLD4	0.67771	0.70529	-0.02758	0.622
1213	203004_s_at	MEF2D	0.58352	0.7149	-0.13138	0.956
1214	203006_at	INPP5A	0.63039	0.64308	-0.01269	0.578
1215	203010_at	STAT5A	0.78481	0.72607	0.05874	0.261
1216	203011_at	IMPA1	0.49256	0.44769	0.04487	0.34
1217	203013_at	ECD	0.67382	0.66377	0.01005	0.472
1218	203017_s_at	SSX2IP	0.44088	0.3432	0.09768	0.168
1219	203022_at	RNASEH2A	0.74065	0.69052	0.05013	0.181
1220	203024_s_at	C5orf15	0.53748	0.61277	-0.07529	0.731
1221	203025_at	ARD1A	0.42503	0.55643	-0.1314	0.922
1222	203026_at	ZBTB5	0.61632	0.49225	0.12407	0.016
1223	203027_s_at	MVD	0.40238	0.28823	0.11415	0.167
1224	203028_s_at	CYBA	0.85335	0.81749	0.03586	0.198
1225	203031_s_at	UROS	0.29994	0.42305	-0.12311	0.818
1226	203035_s_at	PIAS3	0.49513	0.31666	0.17847	0.058
1227	203038_at	PTPRK	0.50747	0.59113	-0.08366	0.777
1228	203039_s_at	NDUFS1	0.779	0.72817	0.05083	0.266
1229	203040_s_at	HMBS	0.35482	0.40409	-0.04927	0.681
1230	203041_s_at	LAMP2	0.43342	0.44917	-0.01575	0.551
1231	203043_at	ZBED1	0.44943	0.48846	-0.03903	0.642
1232	203044_at	CHSY1	0.23262	0.52639	-0.29377	0.994
1233	203046_s_at	TIMELESS	0.49783	0.41917	0.07866	0.139
1234	203049_s_at	TTC37	0.78697	0.83931	-0.05234	0.878
1235	203050_at	TP53BP1	0.36946	0.41006	-0.0406	0.633
1236	203051_at	BAHD1	0.16175	0.10964	0.05211	0.319
1237	203053_at	BCAS2	0.65662	0.63202	0.0246	0.358
1238	203054_s_at	TCTA	0.44541	0.24675	0.19866	0.056
1239	203055_s_at	ARHGEF1	0.73543	0.55213	0.1833	0.005
1240	203058_s_at	PAPSS2	0.14969	0.3862	-0.23651	0.978

1241	203062_s_at	MDC1	0.50789	0.44377	0.06412	0.333
1242	203064_s_at	FOXK2	0.25815	0.44878	-0.19063	0.961
1243	203065_s_at	CAV1	0.26653	0.3745	-0.10797	0.799
1244	203067_at	PDHX	0.48107	0.40754	0.07353	0.257
1245	203068_at	KLHL21	0.14563	0.074142	0.071488	0.213
1246	203069_at	SV2A	0.19821	0.28898	-0.09077	0.704
1247	203072_at	MYO1E	0.13188	0.28002	-0.14814	0.885
1248	203073_at	COG2	0.35054	0.086217	0.264323	0.001
1249	203075_at	SMAD2	0.5363	0.54061	-0.00431	0.531
1250	203079_s_at	CUL2	0.31799	0.38099	-0.063	0.744
1251	203080_s_at	BAZ2B	0.44634	0.52226	-0.07592	0.789
1252	203081_at	CTNNBIP1	0.19279	0.13719	0.0556	0.352
1253	203082_at	BMS1	0.52989	0.56531	-0.03542	0.674
1254	203085_s_at	TGFB1	0.66619	0.53581	0.13038	0.016
1255	203089_s_at	HTRA2	0.66776	0.71174	-0.04398	0.731
1256	203090_at	SDF2	0.61302	0.74482	-0.1318	0.975
1257	203093_s_at	TIMM44	0.49211	0.29529	0.19682	0.033
1258	203094_at	MAD2L1BP	0.67105	0.58157	0.08948	0.112
1259	203095_at	MTIF2	0.56472	0.28572	0.279	<0.001
1260	203099_s_at	CDYL	0.19598	0.21763	-0.02165	0.583
1261	203102_s_at	MGAT2	0.47584	0.56926	-0.09342	0.993
1262	203103_s_at	PRPF19	0.66932	0.50062	0.1687	0.016
1263	203105_s_at	DNM1L	0.35156	0.40647	-0.05491	0.8
1264	203106_s_at	VPS41	0.42365	0.56995	-0.1463	0.888
1265	203109_at	UBE2M	0.80464	0.79863	0.00601	0.467
1266	203112_s_at	WHSC2	0.55449	0.51471	0.03978	0.36
1267	203113_s_at	EEF1D	0.85386	0.79419	0.05967	0.198
1268	203114_at	SSSCA1	0.75173	0.68261	0.06912	0.192
1269	203115_at	FECH	0.3232	0.41545	-0.09225	0.797
1270	203117_s_at	PAN2	0.62092	0.69991	-0.07899	0.842
1271	203118_at	PCSK7	0.32251	0.44673	-0.12422	0.851
1272	203119_at	CCDC86	0.78802	0.26837	0.51965	<0.001
1273	203120_at	TP53BP2	0.26661	0.49067	-0.22406	0.965
1274	203122_at	TTC15	0.61258	0.42164	0.19094	0.036
1275	203124_s_at	SLC11A2	0.26114	0.23651	0.02463	0.449
1276	203126_at	IMPA2	0.39783	0.51429	-0.11646	0.777
1277	203127_s_at	SPTLC2	0.36559	0.489	-0.12341	0.863
1278	203133_at	SEC61B	0.35799	0.48035	-0.12236	0.734
1279	203135_at	TBP	0.56269	0.29446	0.26823	0.007
1280	203136_at	RABAC1	0.55189	0.66075	-0.10886	0.888
1281	203138_at	HAT1	0.81224	0.70058	0.11166	0.001
1282	203139_at	DAPK1	0.27188	0.46338	-0.1915	0.913
1283	203141_s_at	AP3B1	0.78115	0.82018	-0.03903	0.752
1284	203143_s_at	KIAA0040	0.60824	0.53408	0.07416	0.275
1285	203145_at	SPAG5	0.85241	0.80194	0.05047	0.007
1286	203147_s_at	TRIM14	0.8017	0.8287	-0.027	0.689
1287	203150_at	RABEPK	0.69477	0.66335	0.03142	0.314
1288	203152_at	MRPL40	0.82447	0.87107	-0.0466	0.846
1289	203153_at	IFIT1	0.74639	0.64126	0.10513	0.046
1290	203155_at	SETDB1	0.26904	0.27636	-0.00732	0.524
1291	203156_at	AKAP11	0.38033	0.4119	-0.03157	0.629
1292	203158_s_at	GLS	0.7352	0.69961	0.03559	0.21
1293	203163_at	KATNB1	0.49599	0.5115	-0.01551	0.554
1294	203165_s_at	SLC33A1	0.46669	0.48963	-0.02294	0.684

1295	203169_at	RGP1	0.54346	0.62815	-0.08469	0.804
1296	203171_s_at	RRP8	0.46619	0.30748	0.15871	0.034
1297	203173_s_at	C16orf62	0.53903	0.60358	-0.06455	0.729
1298	203175_at	RHOG	0.61882	0.63533	-0.01651	0.574
1299	203176_s_at	TFAM	0.78636	0.83876	-0.0524	0.924
1300	203178_at	GATM	0.55205	0.59095	-0.0389	0.634
1301	203179_at	GALT	0.20945	0.40912	-0.19967	0.94
1302	203183_s_at	SMARCD1	0.34396	0.40024	-0.05628	0.712
1303	203185_at	RASSF2	0.39139	0.3332	0.05819	0.303
1304	203186_s_at	S100A4	0.59104	0.60634	-0.0153	0.622
1305	203188_at	B3GNT1	0.27537	0.23391	0.04146	0.385
1306	203189_s_at	NDUFS8	0.69076	0.71371	-0.02295	0.607
1307	203192_at	ABCB6	0.44269	0.55	-0.10731	0.78
1308	203196_at	ABCC4	0.44258	0.50511	-0.06253	0.7
1309	203197_s_at	C1orf123	0.46009	0.70454	-0.24445	0.997
1310	203198_at	CDK9	0.76953	0.76409	0.00544	0.467
1311	203200_s_at	MTRR	0.21371	0.22419	-0.01048	0.534
1312	203201_at	PMM2	0.53398	0.57451	-0.04053	0.635
1313	203202_at	KRR1	0.5741	0.45718	0.11692	0.163
1314	203205_at	KDM4A	0.29691	0.25845	0.03846	0.213
1315	203206_at	FAM53B	0.27183	0.13215	0.13968	0.075
1316	203208_s_at	MTFR1	0.33449	0.31903	0.01546	0.462
1317	203209_at	RFC5	0.70549	0.70861	-0.00312	0.536
1318	203212_s_at	MTMR2	0.48769	0.49127	-0.00358	0.505
1319	203218_at	MAPK9	0.39722	0.26298	0.13424	0.123
1320	203219_s_at	APRT	0.49343	0.55559	-0.06216	0.654
1321	203223_at	RABEP1	0.19166	0.36653	-0.17487	0.922
1322	203225_s_at	RFK	0.44533	0.20536	0.23997	0.005
1323	203226_s_at	TSPAN31	0.76002	0.65069	0.10933	0.091
1324	203228_at	PAFAH1B3	0.70526	0.76659	-0.06133	0.895
1325	203229_s_at	CLK2	0.60998	0.67358	-0.0636	0.758
1326	203230_at	DVL1	0.51309	0.60575	-0.09266	0.882
1327	203231_s_at	ATXN1	0.3574	0.49104	-0.13364	0.894
1328	203233_at	IL4R	0.56647	0.51525	0.05122	0.286
1329	203234_at	UPP1	0.65191	0.74007	-0.08816	0.928
1330	203235_at	THOP1	0.37597	0.46829	-0.09232	0.831
1331	203236_s_at	LGALS9	0.50905	0.58137	-0.07232	0.796
1332	203241_at	UVRAG	0.46472	0.43358	0.03114	0.348
1333	203244_at	PEX5	0.34333	0.21948	0.12385	0.119
1334	203245_s_at	NCRNA00094	0.43648	0.3373	0.09918	0.214
1335	203246_s_at	TUSC4	0.57515	0.6891	-0.11395	0.902
1336	203247_s_at	ZNF24	0.76391	0.76093	0.00298	0.533
1337	203250_at	RBM16	0.71914	0.71631	0.00283	0.508
1338	203252_at	CDK2AP2	0.63758	0.55655	0.08103	0.178
1339	203253_s_at	HISPPD1	0.52493	0.72121	-0.19628	1
1340	203254_s_at	TLN1	0.80905	0.80493	0.00412	0.472
1341	203258_at	DRAP1	0.48124	0.53227	-0.05103	0.66
1342	203260_at	HDDC2	0.00013014	0.070234	-0.07010386	0.752
1343	203261_at	DCTN6	0.78917	0.72969	0.05948	0.198
1344	203262_s_at	FAM50A	0.76691	0.7957	-0.02879	0.723
1345	203263_s_at	ARHGEF9	0.35782	0.47005	-0.11223	0.859
1346	203266_s_at	MAP2K4	0.28989	0.46508	-0.17519	0.935
1347	203268_s_at	DRG2	0.19304	0.12001	0.07303	0.277
1348	203269_at	NSMAF	0.58351	0.4336	0.14991	0.052

1349	203270_at	DTYMK	0.70602	0.68468	0.02134	0.391
1350	203271_s_at	UNC119	0.63499	0.67564	-0.04065	0.735
1351	203272_s_at	TUSC2	0.68354	0.58714	0.0964	0.025
1352	203274_at	F8A1	0.25465	0.34789	-0.09324	0.783
1353	203275_at	IRF2	0.64274	0.70129	-0.05855	0.724
1354	203276_at	LMNB1	0.90929	0.82075	0.08854	0.002
1355	203277_at	DFFA	0.4215	0.2234	0.1981	0.003
1356	203278_s_at	PHF21A	0.56888	0.61902	-0.05014	0.696
1357	203279_at	EDEM1	0.33368	0.32241	0.01127	0.47
1358	203280_at	SAFB2	0.28586	0.2165	0.06936	0.271
1359	203281_s_at	UBA7	0.48542	0.45585	0.02957	0.361
1360	203282_at	GBE1	0.45672	0.67474	-0.21802	0.97
1361	203286_at	RNF44	0.73903	0.67296	0.06607	0.23
1362	203288_at	KIAA0355	0.21487	0.24982	-0.03495	0.643
1363	203289_s_at	C16orf35	0.63188	0.5587	0.07318	0.181
1364	203292_s_at	VPS11	0.13801	0.14189	-0.00388	0.512
1365	203298_s_at	JARID2	0.50388	0.51514	-0.01126	0.53
1366	203299_s_at	AP1S2	0.57622	0.65286	-0.07664	0.913
1367	203301_s_at	DMTF1	0.63174	0.66177	-0.03003	0.623
1368	203302_at	DCK	0.61466	0.71699	-0.10233	0.917
1369	203303_at	DYNLT3	0.67999	0.55421	0.12578	0.059
1370	203304_at	BAMBI	0.38256	0.7273	-0.34474	1
1371	203306_s_at	SLC35A1	0.25478	0.34096	-0.08618	0.813
1372	203307_at	GNL1	0.64965	0.61475	0.0349	0.283
1373	203309_s_at	HPS1	0.32525	0.35594	-0.03069	0.63
1374	203310_at	STXBP3	0.83376	0.84603	-0.01227	0.633
1375	203311_s_at	ARF6	0.68629	0.5913	0.09499	0.264
1376	203313_s_at	TGIF1	0.20102	0.44458	-0.24356	0.991
1377	203314_at	GTPBP6	0.3552	0.36019	-0.00499	0.521
1378	203315_at	NCK2	0.45296	0.49882	-0.04586	0.658
1379	203316_s_at	SNRPE	0.94635	0.86668	0.07967	<0.001
1380	203318_s_at	ZNF148	0.87162	0.92117	-0.04955	0.976
1381	203320_at	SH2B3	0.39947	0.4466	-0.04713	0.668
1382	203321_s_at	ADNP2	0.6516	0.80052	-0.14892	0.971
1383	203330_s_at	STX5	0.49748	0.1938	0.30368	<0.001
1384	203331_s_at	INPP5D	0.58844	0.71058	-0.12214	0.869
1385	203333_at	KIFAP3	0.49255	0.45935	0.0332	0.384
1386	203334_at	DHX8	0.40093	0.34364	0.05729	0.297
1387	203335_at	PHYH	0.347	0.46173	-0.11473	0.861
1388	203336_s_at	ITGB1BP1	0.3336	0.45375	-0.12015	0.861
1389	203338_at	PPP2R5E	0.47143	0.45255	0.01888	0.419
1390	203339_at	SLC25A12	0.24169	0.36273	-0.12104	0.821
1391	203341_at	CEBPZ	0.60098	0.48713	0.11385	0.134
1392	203342_at	TIMM17B	0.56043	0.61672	-0.05629	0.732
1393	203343_at	UGDH	0.59741	0.6162	-0.01879	0.56
1394	203344_s_at	RBBP8	0.78458	0.746	0.03858	0.306
1395	203350_at	AP1G1	0.5285	0.42396	0.10454	0.104
1396	203351_s_at	ORC4L	0.73268	0.62585	0.10683	0.041
1397	203356_at	CAPN7	0.72306	0.57754	0.14552	0.071
1398	203361_s_at	MYCBP	0.17659	0.07297	0.10362	0.229
1399	203362_s_at	MAD2L1	0.84498	0.7612	0.08378	0.014
1400	203363_s_at	KIAA0652	0.62709	0.50417	0.12292	<0.001
1401	203366_at	POLG	0.70611	0.60712	0.09899	0.132
1402	203367_at	DUSP14	0.055645	0.21742	-0.161775	0.889

1403	203368_at	CRELD1	0.16094	0.16328	-0.00234	0.517
1404	203371_s_at	NDUFB3	0.56962	0.41745	0.15217	0.018
1405	203373_at	SOCS2	0.46535	0.59958	-0.13423	0.902
1406	203375_s_at	TPP2	0.79449	0.67057	0.12392	0.086
1407	203377_s_at	CDC40	0.75979	0.78705	-0.02726	0.684
1408	203378_at	PCF11	0.32457	0.48258	-0.15801	0.957
1409	203379_at	RPS6KA1	0.45092	0.4054	0.04552	0.346
1410	203384_s_at	GOLGA1	0.66947	0.60183	0.06764	0.218
1411	203385_at	DGKA	0.75141	0.64911	0.1023	0.039
1412	203387_s_at	TBC1D4	0.25738	0.45274	-0.19536	0.904
1413	203388_at	ARRB2	0.1962	0.036629	0.159571	0.005
1414	203391_at	FKBP2	0.54141	0.54653	-0.00512	0.534
1415	203396_at	PSMA4	0.59428	0.51158	0.0827	0.208
1416	203397_s_at	GALNT3	0.36944	0.46668	-0.09724	0.833
1417	203401_at	PRPS2	0.76467	0.69621	0.06846	0.046
1418	203404_at	ARMCX2	0.01318	0.17064	-0.15746	0.914
1419	203405_at	PSMG1	0.6396	0.67851	-0.03891	0.689
1420	203406_at	MFAP1	0.40832	0.45187	-0.04355	0.688
1421	203409_at	DDB2	0.6996	0.72961	-0.03001	0.683
1422	203410_at	AP3M2	0.51179	0.6857	-0.17391	0.94
1423	203411_s_at	LMNA	0.69472	0.75957	-0.06485	0.876
1424	203412_at	LZTR1	0.49126	0.49984	-0.00858	0.529
1425	203414_at	MMD	0.63064	0.60513	0.02551	0.383
1426	203415_at	PDCD6	0.67013	0.68575	-0.01562	0.627
1427	203416_at	CD53	0.73192	0.63782	0.0941	0.084
1428	203420_at	FAM8A1	0.65849	0.54576	0.11273	0.09
1429	203422_at	POLD1	0.84273	0.66194	0.18079	<0.001
1430	203427_at	ASF1A	0.68946	0.67756	0.0119	0.427
1431	203429_s_at	C1orf9	0.70624	0.7376	-0.03136	0.719
1432	203430_at	HEBP2	0.57683	0.57208	0.00475	0.516
1433	203433_at	MTHFS	0.37379	0.50579	-0.132	0.923
1434	203436_at	RPP30	0.75751	0.80563	-0.04812	0.731
1435	203437_at	TMEM11	0.83954	0.72445	0.11509	0.067
1436	203438_at	STC2	0.18399	0.3189	-0.13491	0.958
1437	203444_s_at	MTA2	0.53242	0.48478	0.04764	0.325
1438	203445_s_at	CTDSP2	0.54827	0.47941	0.06886	0.249
1439	203449_s_at	TERF1	0.40361	0.55921	-0.1556	0.942
1440	203450_at	CBY1	0.45855	0.57985	-0.1213	0.956
1441	203452_at	B3GAT3	0.053672	0.042361	0.011311	0.442
1442	203454_s_at	ATOX1	0.71311	0.71322	-0.00011	0.505
1443	203455_s_at	SAT1	0.52464	0.65922	-0.13458	0.851
1444	203456_at	PRAF2	0.54811	0.62939	-0.08128	0.855
1445	203457_at	STX7	0.18113	0.19427	-0.01314	0.552
1446	203458_at	SPR	0.67445	0.49905	0.1754	0.015
1447	203459_s_at	VPS16A	0.21695	0.4131	-0.19615	0.929
1448	203460_s_at	PSEN1	0.85103	0.86624	-0.01521	0.65
1449	203464_s_at	EPN2	0.2258	0.18395	0.04185	0.449
1450	203465_at	MRPL19	0.24636	0.37013	-0.12377	0.881
1451	203466_at	MPV17	0.58614	0.57855	0.00759	0.463
1452	203467_at	PMM1	0.48398	0.51999	-0.03601	0.69
1453	203468_at	CDK10	0.13845	0.16688	-0.02843	0.589
1454	203471_s_at	PLEK	0.58744	0.45573	0.13171	0.082
1455	203474_at	IQGAP2	0.47831	0.62044	-0.14213	0.878
1456	203476_at	TPBG	0.3179	0.43016	-0.11226	0.819

1457	203478_at	NDUFC1	0.46627	0.59953	-0.13326	0.933
1458	203480_s_at	OTUD4	0.10665	0.2612	-0.15455	0.777
1459	203482_at	FAM178A	0.33824	0.51828	-0.18004	0.926
1460	203484_at	SEC61G	0.85505	0.82203	0.03302	0.259
1461	203487_s_at	ARMC8	0.69549	0.61848	0.07701	0.183
1462	203493_s_at	CEP57	0.77337	0.75827	0.0151	0.4
1463	203495_at	LRRC14	0.4392	0.39626	0.04294	0.338
1464	203496_s_at	MED1	0.79615	0.80616	-0.01001	0.625
1465	203500_at	GCDH	0.30557	0.22337	0.0822	0.234
1466	203502_at	BPGM	0.42103	0.50849	-0.08746	0.8
1467	203508_at	TNFRSF1B	0.51315	0.55126	-0.03811	0.632
1468	203511_s_at	TRAPPC3	0.75545	0.81315	-0.0577	0.783
1469	203513_at	SPG11	0.43968	0.50305	-0.06337	0.718
1470	203514_at	MAP3K3	0.42312	0.45469	-0.03157	0.609
1471	203515_s_at	PMVK	0.65756	0.6694	-0.01184	0.539
1472	203517_at	MTX2	0.52925	0.63584	-0.10659	0.896
1473	203519_s_at	UPF2	0.18344	0.0096717	0.1737683	0.048
1474	203521_s_at	ZNF318	0.56629	0.63799	-0.0717	0.818
1475	203522_at	CCS	0.71178	0.76785	-0.05607	0.866
1476	203523_at	LSP1	0.67589	0.64062	0.03527	0.329
1477	203524_s_at	MPST	0.57663	0.64829	-0.07166	0.766
1478	203526_s_at	APC	0.47166	0.60063	-0.12897	0.92
1479	203528_at	SEMA4D	0.20642	0.37246	-0.16604	0.905
1480	203529_at	PPP6C	0.6358	0.69961	-0.06381	0.713
1481	203530_s_at	STX4	0.67928	0.76353	-0.08425	0.849
1482	203531_at	CUL5	0.59202	0.57411	0.01791	0.422
1483	203534_at	LSM1	0.51194	0.60999	-0.09805	0.78
1484	203537_at	PRPSAP2	0.48172	0.38338	0.09834	0.098
1485	203538_at	CAMLG	0.62742	0.49135	0.13607	0.111
1486	203542_s_at	KLF9	0.38827	0.51811	-0.12984	0.914
1487	203544_s_at	STAM	0.70954	0.74169	-0.03215	0.658
1488	203545_at	ALG8	0.24335	0.46973	-0.22638	0.946
1489	203546_at	IPO13	0.62827	0.52869	0.09958	0.163
1490	203550_s_at	C1orf2	0.5616	0.45639	0.10521	0.122
1491	203552_at	MAP4K5	0.56914	0.69932	-0.13018	0.953
1492	203556_at	ZHX2	0.52108	0.60319	-0.08211	0.882
1493	203557_s_at	PCBD1	0.80647	0.76696	0.03951	0.214
1494	203560_at	GGH	0.6787	0.75332	-0.07462	0.866
1495	203561_at	FCGR2A	0.072556	0.26237	-0.189814	0.913
1496	203562_at	FEZ1	0.48945	0.6401	-0.15065	0.919
1497	203564_at	FANCG	0.55538	0.59221	-0.03683	0.68
1498	203565_s_at	MNAT1	0.28537	0.34734	-0.06197	0.673
1499	203566_s_at	AGL	0.73334	0.75682	-0.02348	0.642
1500	203567_s_at	TRIM38	0.74105	0.71466	0.02639	0.346
1501	203569_s_at	OFD1	0.5997	0.51734	0.08236	0.14
1502	203572_s_at	TAF6	0.13884	0.35111	-0.21227	0.982
1503	203573_s_at	RABGGTA	0.53467	0.59222	-0.05755	0.717
1504	203574_at	NFIL3	0.4863	0.48125	0.00505	0.51
1505	203575_at	CSNK2A2	0.32674	0.30104	0.0257	0.424
1506	203577_at	GTF2H4	0.50026	0.57132	-0.07106	0.757
1507	203579_s_at	SLC7A6	0.66792	0.63001	0.03791	0.357
1508	203581_at	RAB4A	0.31965	0.45467	-0.13502	0.976
1509	203583_at	UNC50	0.81878	0.76773	0.05105	0.24
1510	203584_at	TTC35	0.55626	0.63984	-0.08358	0.864

1511	203588_s_at	TFDP2	0.31712	0.4782	-0.16108	0.909
1512	203590_at	DYNC1LI2	0.80138	0.76329	0.03809	0.237
1513	203592_s_at	FSTL3	0.67101	0.57174	0.09927	0.26
1514	203593_at	CD2AP	0.6688	0.75275	-0.08395	0.897
1515	203596_s_at	IFIT5	0.57202	0.5417	0.03032	0.379
1516	203599_s_at	WBP4	0.46098	0.51412	-0.05314	0.751
1517	203600_s_at	C4orf8	0.51425	0.58789	-0.07364	0.767
1518	203604_at	ZNF516	0.1122	0.25148	-0.13928	0.903
1519	203605_at	SRP54	0.38386	0.3901	-0.00624	0.542
1520	203606_at	NDUFS6	0.89962	0.85519	0.04443	0.094
1521	203607_at	INPP5F	0.70303	0.79216	-0.08913	0.963
1522	203608_at	ALDH5A1	0.27335	0.49812	-0.22477	0.966
1523	203611_at	TERF2	0.24577	0.59124	-0.34547	1
1524	203612_at	BYSL	0.59323	0.34377	0.24946	<0.001
1525	203613_s_at	NDUFB6	0.56601	0.53693	0.02908	0.394
1526	203614_at	UTP14C	0.79747	0.79423	0.00324	0.475
1527	203616_at	POLB	0.57607	0.67385	-0.09778	0.916
1528	203620_s_at	FCHSD2	0.59501	0.69992	-0.10491	0.908
1529	203621_at	NDUFB5	0.64114	0.8133	-0.17216	0.915
1530	203622_s_at	PNO1	0.74601	0.61998	0.12603	0.064
1531	203630_s_at	COG5	0.085919	0.26586	-0.179941	0.945
1532	203634_s_at	CPT1A	0.37336	0.34751	0.02585	0.429
1533	203635_at	DSCR3	0.36672	0.34557	0.02115	0.413
1534	203643_at	ERF	0.56561	0.28522	0.28039	<0.001
1535	203644_s_at	MON1B	0.53494	0.60628	-0.07134	0.738
1536	203647_s_at	FDX1	0.56888	0.65153	-0.08265	0.807
1537	203648_at	TATDN2	0.31736	0.35649	-0.03913	0.653
1538	203651_at	ZFYVE16	0.12512	0.20768	-0.08256	0.778
1539	203652_at	MAP3K11	0.74102	0.75459	-0.01357	0.592
1540	203653_s_at	COIL	0.77246	0.75374	0.01872	0.392
1541	203655_at	XRCC1	0.23278	0.29681	-0.06403	0.728
1542	203656_at	FIG4	0.77626	0.70826	0.068	0.09
1543	203658_at	SLC25A20	0.28175	0.40401	-0.12226	0.833
1544	203659_s_at	TRIM13	0.55094	0.48098	0.06996	0.194
1545	203660_s_at	PCNT	0.57198	0.5785	-0.00652	0.552
1546	203662_s_at	TMOD1	0.4144	0.52158	-0.10718	0.824
1547	203665_at	HMOX1	0.27597	0.51849	-0.24252	0.973
1548	203668_at	MAN2C1	0.59238	0.60447	-0.01209	0.573
1549	203669_s_at	DGAT1	0.17422	0.29721	-0.12299	0.836
1550	203671_at	TPMT	0.39836	0.39318	0.00518	0.497
1551	203674_at	HELZ	0.47383	0.44291	0.03092	0.379
1552	203675_at	NUCB2	0.66286	0.62958	0.03328	0.353
1553	203677_s_at	TARBP2	0.75491	0.58575	0.16916	0.019
1554	203678_at	MTMR15	0.3817	0.37413	0.00757	0.472
1555	203679_at	TMED1	0.44055	0.58617	-0.14562	0.867
1556	203683_s_at	VEGFB	0.56674	0.48555	0.08119	0.122
1557	203684_s_at	BCL2	0.41131	0.2101	0.20121	0.039
1558	203686_at	MPG	0.64766	0.47005	0.17761	0.04
1559	203688_at	PKD2	0.49906	0.5171	-0.01804	0.576
1560	203689_s_at	FMR1	0.48693	0.43449	0.05244	0.36
1561	203693_s_at	E2F3	0.76315	0.64333	0.11982	0.015
1562	203694_s_at	DHX16	0.36711	0.32051	0.0466	0.388
1563	203695_s_at	DFNA5	0.53107	0.57064	-0.03957	0.698
1564	203701_s_at	TRMT1	0.59152	0.489	0.10252	0.138

1565	203702_s_at	TTLL4	0.27672	0.31224	-0.03552	0.625
1566	203705_s_at	FZD7	0.14658	0.32882	-0.18224	0.936
1567	203707_at	ZNF263	0.13725	0.33404	-0.19679	0.941
1568	203709_at	PHKG2	0.43218	0.46901	-0.03683	0.659
1569	203711_s_at	HIBCH	0.23762	0.37587	-0.13825	0.892
1570	203712_at	KIAA0020	0.80556	0.76894	0.03662	0.359
1571	203713_s_at	LLGL2	0.030925	0.043264	-0.012339	0.55
1572	203715_at	TBCE	0.36936	0.35024	0.01912	0.427
1573	203718_at	PNPLA6	0.78627	0.70079	0.08548	0.059
1574	203720_s_at	ERCC1	0.59958	0.6387	-0.03912	0.728
1575	203722_at	ALDH4A1	0.43227	0.42183	0.01044	0.485
1576	203723_at	ITPKB	0.61827	0.68986	-0.07159	0.725
1577	203725_at	GADD45A	0.87387	0.83106	0.04281	0.077
1578	203727_at	SKIV2L	0.56905	0.47833	0.09072	0.186
1579	203728_at	BAK1	0.58748	0.66	-0.07252	0.768
1580	203729_at	EMP3	0.43586	0.59042	-0.15456	0.943
1581	203731_s_at	ZKSCAN5	0.028364	0.075745	-0.047381	0.629
1582	203732_at	TRIP4	0.46262	0.46197	0.00065	0.512
1583	203733_at	DEXI	0.51015	0.69365	-0.1835	0.958
1584	203734_at	FOXJ2	0.20667	0.22752	-0.02085	0.611
1585	203737_s_at	PPRC1	0.73113	0.51271	0.21842	<0.001
1586	203738_at	C5orf22	0.4822	0.54028	-0.05808	0.693
1587	203739_at	ZNF217	0.51917	0.58425	-0.06508	0.654
1588	203740_at	MPHOSPH6	0.35179	0.29913	0.05266	0.328
1589	203741_s_at	ADCY7	0.42629	0.39211	0.03418	0.387
1590	203743_s_at	TDG	0.75004	0.46996	0.28008	<0.001
1591	203744_at	HMGB3	0.38251	0.48019	-0.09768	0.916
1592	203745_at	HCCS	0.393	0.45221	-0.05921	0.703
1593	203752_s_at	JUND	0.71341	0.63122	0.08219	0.209
1594	203755_at	BUB1B	0.79231	0.73419	0.05812	0.184
1595	203758_at	CTSO	0.60375	0.54216	0.06159	0.231
1596	203762_s_at	DYNC2LI1	0.19088	0.33141	-0.14053	0.873
1597	203764_at	DLGAP5	0.6272	0.68998	-0.06278	0.824
1598	203765_at	GCA	0.64165	0.63427	0.00738	0.477
1599	203774_at	MTR	0.65277	0.59496	0.05781	0.294
1600	203775_at	SLC25A13	0.78651	0.79319	-0.00668	0.523
1601	203776_at	GPKOW	0.64631	0.54899	0.09732	0.162
1602	203777_s_at	RPS6KB2	0.15547	0.22852	-0.07305	0.74
1603	203778_at	MANBA	0.30076	0.23357	0.06719	0.295
1604	203781_at	MRPL33	0.4601	0.4898	-0.0297	0.605
1605	203782_s_at	POLRMT	0.60044	0.56284	0.0376	0.316
1606	203787_at	SSBP2	0.69769	0.72198	-0.02429	0.661
1607	203790_s_at	HRSP12	0.65789	0.75356	-0.09567	0.931
1608	203791_at	DMXL1	0.84705	0.84399	0.00306	0.485
1609	203795_s_at	BCL7A	0.49924	0.60725	-0.10801	0.841
1610	203799_at	CD302	0.44378	0.50055	-0.05677	0.671
1611	203800_s_at	MRPS14	0.71641	0.79251	-0.0761	0.908
1612	203803_at	PCYOX1	0.67669	0.71629	-0.0396	0.678
1613	203804_s_at	CROP	0.71027	0.6657	0.04457	0.24
1614	203805_s_at	FANCA	0.42624	0.48935	-0.06311	0.752
1615	203810_at	DNAJB4	0.4168	0.31405	0.10275	0.168
1616	203814_s_at	NQO2	0.060062	0.27539	-0.215328	0.96
1617	203815_at	GSTT1	0.0014301	0.12881	-0.1273799	0.867
1618	203816_at	DGUOK	0.28664	0.58304	-0.2964	0.996

1619	203817_at	GUCY1B3	0.20961	0.43552	-0.22591	0.98
1620	203818_s_at	SF3A3	0.75877	0.71682	0.04195	0.195
1621	203820_s_at	IGF2BP3	0.28675	0.45875	-0.172	0.861
1622	203822_s_at	ELF2	0.55116	0.3802	0.17096	0.041
1623	203825_at	BRD3	0.43855	0.54395	-0.1054	0.797
1624	203826_s_at	PITPNM1	0.51869	0.5081	0.01059	0.457
1625	203827_at	WIPI1	0.4732	0.23464	0.23856	<0.001
1626	203829_at	ELP4	0.39422	0.62038	-0.22616	0.929
1627	203830_at	C17orf75	0.55318	0.56541	-0.01223	0.549
1628	203831_at	R3HDM2	0.28187	0.4054	-0.12353	0.932
1629	203832_at	SNRPF	0.64179	0.66362	-0.02183	0.665
1630	203836_s_at	MAP3K5	0.7723	0.81032	-0.03802	0.75
1631	203840_at	BLZF1	0.54915	0.67401	-0.12486	0.982
1632	203843_at	RPS6KA3	0.47302	0.27069	0.20233	0.026
1633	203845_at	KAT2B	0.26453	0.33834	-0.07381	0.796
1634	203846_at	TRIM32	0.70594	0.56079	0.14515	0.109
1635	203852_s_at	SMN1	0.31033	0.48167	-0.17134	0.944
1636	203855_at	WDR47	0.70651	0.62994	0.07657	0.219
1637	203856_at	VRK1	0.77049	0.7505	0.01999	0.41
1638	203857_s_at	PDIA5	0.43277	0.53132	-0.09855	0.861
1639	203858_s_at	COX10	0.071924	0.0005096	0.0714144	0.237
1640	203860_at	PCCA	0.33302	0.36459	-0.03157	0.636
1641	203866_at	NLE1	0.040002	0.092913	-0.052911	0.696
1642	203868_s_at	VCAM1	0.54178	0.64395	-0.10217	0.896
1643	203869_at	USP46	0.72569	0.72775	-0.00206	0.512
1644	203871_at	SENP3	0.53599	0.64554	-0.10955	0.896
1645	203879_at	PIK3CD	0.66997	0.64215	0.02782	0.345
1646	203880_at	COX17	0.27831	0.43582	-0.15751	0.923
1647	203881_s_at	DMD	0.41044	0.57883	-0.16839	0.895
1648	203883_s_at	RAB11FIP2	0.67524	0.76013	-0.08489	0.918
1649	203885_at	RAB21	0.62648	0.61267	0.01381	0.418
1650	203890_s_at	DAPK3	0.56426	0.77565	-0.21139	0.998
1651	203892_at	WFDC2	0.45149	0.55567	-0.10418	0.864
1652	203894_at	TUBG2	0.47407	0.48446	-0.01039	0.542
1653	203897_at	LYRM1	0.65879	0.6823	-0.02351	0.599
1654	203899_s_at	CRCP	0.84906	0.72875	0.12031	0.001
1655	203900_at	KIAA0467	0.41743	0.56724	-0.14981	0.933
1656	203901_at	MAP3K7IP1	0.48422	0.28729	0.19693	0.023
1657	203903_s_at	HEPH	0.37029	0.54639	-0.1761	0.904
1658	203905_at	PARN	0.20108	0.30941	-0.10833	0.822
1659	203907_s_at	IQSEC1	0.32706	0.41216	-0.0851	0.829
1660	203909_at	SLC9A6	0.69881	0.64011	0.0587	0.279
1661	203912_s_at	DNASE1L1	0.26278	0.40215	-0.13937	0.899
1662	203916_at	NDST2	0.59645	0.41261	0.18384	0.008
1663	203919_at	TCEA2	0.53919	0.65916	-0.11997	0.894
1664	203920_at	NR1H3	0.4006	0.36915	0.03145	0.407
1665	203921_at	CHST2	0.71389	0.74311	-0.02922	0.723
1666	203923_s_at	CYBB	0.40361	0.60494	-0.20133	0.937
1667	203925_at	GCLM	0.70752	0.64385	0.06367	0.296
1668	203927_at	NFKBIE	0.76593	0.75661	0.00932	0.452
1669	203931_s_at	MRPL12	0.5617	0.32857	0.23313	0.005
1670	203932_at	HLA-DMB	0.53124	0.5468	-0.01556	0.566
1671	203933_at	RAB11FIP3	0.28178	0.27307	0.00871	0.482
1672	203935_at	ACVR1	0.39452	0.37498	0.01954	0.442

1673	203936_s_at	MMP9	0.0014479	0.061803	-0.0603551	0.669
1674	203938_s_at	TAF1C	0.20961	0.28337	-0.07376	0.721
1675	203939_at	NT5E	0.2982	0.47654	-0.17834	0.853
1676	203941_at	INTS9	0.54951	0.59311	-0.0436	0.646
1677	203942_s_at	MARK2	0.55796	0.63654	-0.07858	0.817
1678	203943_at	KIF3B	0.69531	0.70042	-0.00511	0.55
1679	203945_at	ARG2	0.65058	0.51217	0.13841	0.035
1680	203947_at	CSTF3	0.5637	0.52752	0.03618	0.369
1681	203952_at	ATF6	0.013622	0.19925	-0.185628	0.961
1682	203955_at	KIAA0649	0.4951	0.44318	0.05192	0.29
1683	203957_at	E2F6	0.63536	0.64847	-0.01311	0.564
1684	203958_s_at	ZBTB40	0.63358	0.73554	-0.10196	0.945
1685	203960_s_at	HSPB11	0.52307	0.41498	0.10809	0.114
1686	203964_at	NMI	0.7646	0.72295	0.04165	0.295
1687	203965_at	USP20	0.24331	0.28421	-0.0409	0.649
1688	203966_s_at	PPM1A	0.59325	0.66127	-0.06802	0.834
1689	203967_at	CDC6	0.75912	0.57825	0.18087	0.016
1690	203970_s_at	PEX3	0.30275	0.45427	-0.15152	0.885
1691	203971_at	SLC31A1	0.47786	0.33903	0.13883	0.087
1692	203973_s_at	CEBPD	0.63928	0.62932	0.00996	0.493
1693	203974_at	HDHD1A	0.23496	0.5561	-0.32114	0.998
1694	203975_s_at	CHAF1A	0.86939	0.85991	0.00948	0.391
1695	203978_at	NUBP1	0.66906	0.75292	-0.08386	0.911
1696	203983_at	TSNAX	0.61366	0.65161	-0.03795	0.637
1697	203984_s_at	CASP9	0.67452	0.75736	-0.08284	0.943
1698	203985_at	ZNF212	0.15639	0.31291	-0.15652	0.866
1699	203987_at	FZD6	0.4879	0.49608	-0.00818	0.581
1700	203988_s_at	FUT8	0.4503	0.45481	-0.00451	0.51
1701	203990_s_at	KDM6A	0.74713	0.77388	-0.02675	0.676
1702	204001_at	SNAPC3	0.31246	0.21629	0.09617	0.199
1703	204003_s_at	NUPL2	0.31258	0.25638	0.0562	0.297
1704	204004_at	PAWR	0.48833	0.52615	-0.03782	0.665
1705	204008_at	DNAL4	0.085431	0.098413	-0.012982	0.518
1706	204015_s_at	DUSP4	0.75253	0.72321	0.02932	0.296
1707	204016_at	LARS2	0.46602	0.42516	0.04086	0.356
1708	204019_s_at	SH3YL1	0.23728	0.27719	-0.03991	0.611
1709	204021_s_at	PURA	0.58939	0.69867	-0.10928	0.866
1710	204023_at	RFC4	0.88102	0.7871	0.09392	<0.001
1711	204025_s_at	PDCD2	0.43908	0.59397	-0.15489	0.95
1712	204026_s_at	ZWINT	0.78559	0.71991	0.06568	0.062
1713	204027_s_at	METTL1	0.47766	0.35658	0.12108	0.081
1714	204028_s_at	RABGAP1	0.73286	0.6977	0.03516	0.309
1715	204030_s_at	SCHIP1	0.10781	0.40498	-0.29717	0.998
1716	204032_at	BCAR3	0.48839	0.57859	-0.0902	0.823
1717	204033_at	TRIP13	0.90741	0.61793	0.28948	0.001
1718	204034_at	ETHE1	0.54381	0.59619	-0.05238	0.747
1719	204044_at	QPRT	0.19517	0.2898	-0.09463	0.98
1720	204045_at	TCEAL1	0.49038	0.36095	0.12943	0.116
1721	204049_s_at	PHACTR2	0.454	0.59811	-0.14411	0.973
1722	204054_at	PTEN	0.40081	0.38612	0.01469	0.453
1723	204057_at	IRF8	0.54082	0.58659	-0.04577	0.652
1724	204059_s_at	ME1	0.014293	0.093843	-0.07955	0.924
1725	204061_at	PRKX	0.1303	0.34817	-0.21787	0.973
1726	204064_at	THOC1	0.51572	0.39455	0.12117	0.064

1727	204065_at	CHST10	0.35434	0.3247	0.02964	0.467
1728	204067_at	SUOX	0.23176	0.22015	0.01161	0.466
1729	204068_at	STK3	0.56033	0.65582	-0.09549	0.836
1730	204070_at	RARRES3	0.45956	0.47165	-0.01209	0.559
1731	204071_s_at	TOPORS	0.40991	0.33948	0.07043	0.213
1732	204074_s_at	KIAA0562	0.84897	0.86558	-0.01661	0.678
1733	204076_at	ENTPD4	0.54461	0.35783	0.18678	0.034
1734	204079_at	TPST2	0.2894	0.31339	-0.02399	0.579
1735	204080_at	TOE1	0.63878	0.60749	0.03129	0.317
1736	204081_at	NRGN	0.60927	0.55878	0.05049	0.419
1737	204082_at	PBX3	0.70022	0.76097	-0.06075	0.907
1738	204085_s_at	CLN5	0.23056	0.25885	-0.02829	0.606
1739	204087_s_at	SLC5A6	0.58267	0.67361	-0.09094	0.828
1740	204088_at	P2RX4	0.56062	0.48788	0.07274	0.242
1741	204091_at	PDE6D	0.67184	0.70484	-0.033	0.694
1742	204093_at	CCNH	0.74649	0.755	-0.00851	0.55
1743	204094_s_at	TSC22D2	0.63995	0.66847	-0.02852	0.665
1744	204096_s_at	ELL	0.15261	0.33624	-0.18363	0.938
1745	204098_at	RBMX2	0.31042	0.48334	-0.17292	0.889
1746	204102_s_at	EEF2	0.91262	0.87612	0.0365	0.069
1747	204103_at	CCL4	0.58972	0.61196	-0.02224	0.543
1748	204104_at	SNAPC2	0.20312	0.14257	0.06055	0.328
1749	204106_at	TESK1	0.35545	0.3877	-0.03225	0.611
1750	204108_at	NFYA	0.64546	0.48274	0.16272	0.038
1751	204113_at	CUGBP1	0.58058	0.66327	-0.08269	0.819
1752	204115_at	GNG11	0.13363	0.40179	-0.26816	0.993
1753	204116_at	IL2RG	0.70585	0.74434	-0.03849	0.656
1754	204117_at	PREP	0.24894	0.24122	0.00772	0.485
1755	204118_at	CD48	0.70273	0.84088	-0.13815	0.909
1756	204120_s_at	ADK	0.71643	0.78534	-0.06891	0.842
1757	204123_at	LIG3	0.061935	0.037968	0.023967	0.418
1758	204125_at	NDUFAF1	0.025883	0.13683	-0.110947	0.838
1759	204126_s_at	CDC45L	0.65762	0.55558	0.10204	0.077
1760	204128_s_at	RFC3	0.83693	0.68355	0.15338	<0.001
1761	204131_s_at	FOXO3	0.39408	0.63218	-0.2381	0.979
1762	204133_at	RRP9	0.75994	0.52165	0.23829	<0.001
1763	204135_at	FILIP1L	0.60635	0.64636	-0.04001	0.663
1764	204137_at	GPR137B	0.74588	0.61523	0.13065	0.013
1765	204140_at	TPST1	0.39517	0.49529	-0.10012	0.725
1766	204141_at	TUBB2A	0.2157	0.37627	-0.16057	0.946
1767	204142_at	ENOSF1	0.18673	0.27341	-0.08668	0.809
1768	204144_s_at	PIGQ	0.10163	0.075359	0.026271	0.416
1769	204145_at	FRG1	0.21481	0.3574	-0.14259	0.895
1770	204146_at	RAD51AP1	0.68491	0.72097	-0.03606	0.715
1771	204149_s_at	GSTM4	0.12985	0.40486	-0.27501	0.985
1772	204153_s_at	MFNG	0.73446	0.71244	0.02202	0.376
1773	204156_at	KIAA0999	0.73147	0.6486	0.08287	0.144
1774	204158_s_at	TCIRG1	0.73404	0.75696	-0.02292	0.682
1775	204160_s_at	ENPP4	0.49099	0.53109	-0.0401	0.674
1776	204162_at	NDC80	0.83029	0.83267	-0.00238	0.547
1777	204164_at	SIPA1	0.85386	0.72191	0.13195	0.02
1778	204165_at	WASF1	0.26627	0.4062	-0.13993	0.828
1779	204168_at	MGST2	0.37706	0.59229	-0.21523	0.965
1780	204169_at	IMPDH1	0.32354	0.13613	0.18741	0.031

1781	204170_s_at	CKS2	0.84623	0.78937	0.05686	0.127
1782	204171_at	RPS6KB1	0.62521	0.67891	-0.0537	0.751
1783	204172_at	CPOX	0.24367	0.36895	-0.12528	0.792
1784	204173_at	MYL6B	0.62459	0.62377	0.00082	0.524
1785	204174_at	ALOX5AP	0.20318	0.37646	-0.17328	0.971
1786	204175_at	ZNF593	0.49632	0.47227	0.02405	0.408
1787	204178_s_at	RBM14	0.69719	0.45689	0.2403	<0.001
1788	204181_s_at	ZBTB43	0.66375	0.68748	-0.02373	0.651
1789	204183_s_at	ADRBK2	0.29741	0.52775	-0.23034	0.968
1790	204186_s_at	PPID	0.78583	0.68141	0.10442	0.122
1791	204190_at	USPL1	0.020744	0.13332	-0.112576	0.812
1792	204191_at	IFNAR1	0.50209	0.086214	0.415876	0.001
1793	204192_at	CD37	0.67125	0.68066	-0.00941	0.584
1794	204198_s_at	RUNX3	0.65393	0.74938	-0.09545	0.884
1795	204201_s_at	PTPN13	0.35287	0.48226	-0.12939	0.825
1796	204203_at	CEBPG	0.7463	0.59659	0.14971	0.032
1797	204205_at	APOBEC3G	0.49192	0.58432	-0.0924	0.847
1798	204206_at	MNT	0.018074	0.1292	-0.111126	0.866
1799	204208_at	RNGTT	0.22826	0.37639	-0.14813	0.901
1800	204209_at	PCYT1A	0.3316	0.46926	-0.13766	0.914
1801	204212_at	ACOT8	0.36862	0.33196	0.03666	0.375
1802	204215_at	C7orf23	0.45012	0.61109	-0.16097	0.956
1803	204216_s_at	ZC3H14	0.085558	0.080929	0.004629	0.504
1804	204218_at	C11orf51	0.42615	0.41761	0.00854	0.462
1805	204219_s_at	PSMC1	0.6493	0.44164	0.20766	0.015
1806	204220_at	GMFG	0.75962	0.77695	-0.01733	0.697
1807	204222_s_at	GLIPR1	0.004297	0.19674	-0.192443	0.982
1808	204224_s_at	GCH1	0.51868	0.5358	-0.01712	0.603
1809	204225_at	HDAC4	0.3078	0.38722	-0.07942	0.762
1810	204226_at	STAU2	0.69036	0.68572	0.00464	0.509
1811	204228_at	PPIH	0.62162	0.73152	-0.1099	0.904
1812	204232_at	FCER1G	0.0082019	0.12982	-0.1216181	0.843
1813	204233_s_at	CHKA	0.29935	0.4415	-0.14215	0.847
1814	204234_s_at	ZNF195	0.66179	0.69158	-0.02979	0.653
1815	204241_at	ACOX3	0.036701	0.26034	-0.223639	0.912
1816	204243_at	RLF	0.60781	0.60659	0.00122	0.512
1817	204244_s_at	DBF4	0.73711	0.72486	0.01225	0.404
1818	204245_s_at	RPP14	0.7616	0.75145	0.01015	0.409
1819	204246_s_at	DCTN3	0.79691	0.7533	0.04361	0.196
1820	204247_s_at	CDK5	0.74259	0.6163	0.12629	0.04
1821	204249_s_at	LMO2	0.71285	0.57505	0.1378	0.059
1822	204250_s_at	CEP164	0.03635	0.048468	-0.012118	0.565
1823	204258_at	CHD1	0.57683	0.64867	-0.07184	0.778
1824	204263_s_at	CPT2	0.70845	0.70799	0.00046	0.497
1825	204265_s_at	GPSM3	0.68266	0.58231	0.10035	0.155
1826	204269_at	PIM2	0.62068	0.47729	0.14339	0.013
1827	204275_at	SOLH	0.69417	0.45892	0.23525	0.006
1828	204276_at	TK2	0.29181	0.30386	-0.01205	0.555
1829	204278_s_at	EBAG9	0.70088	0.70155	-0.00067	0.521
1830	204279_at	PSMB9	0.65574	0.7582	-0.10246	0.901
1831	204286_s_at	PMAIP1	0.74355	0.7964	-0.05285	0.815
1832	204291_at	ZNF518A	0.55145	0.53686	0.01459	0.423
1833	204294_at	AMT	0.16936	0.2543	-0.08494	0.785
1834	204295_at	SURF1	0.46841	0.53863	-0.07022	0.784

1835	204296_at	DCTN1	0.62371	0.79472	-0.17101	0.996
1836	204297_at	PIK3C3	0.17238	0.33833	-0.16595	0.876
1837	204299_at	FUSIP1	0.7209	0.6767	0.0442	0.201
1838	204300_at	PET112L	0.47754	0.23876	0.23878	0.006
1839	204305_at	MIPEP	0.1993	0.32	-0.1207	0.83
1840	204306_s_at	CD151	0.26174	0.36969	-0.10795	0.783
1841	204308_s_at	TECPR2	0.62528	0.66824	-0.04296	0.674
1842	204319_s_at	RGS10	0.71267	0.68645	0.02622	0.398
1843	204327_s_at	ZNF202	0.20076	0.046275	0.154485	0.1
1844	204331_s_at	MRPS12	0.87591	0.88825	-0.01234	0.603
1845	204332_s_at	AGA	0.37341	0.26192	0.11149	0.074
1846	204334_at	KLF7	0.60183	0.51175	0.09008	0.179
1847	204335_at	CCDC94	0.4434	0.5841	-0.1407	0.929
1848	204336_s_at	RGS19	0.42765	0.40782	0.01983	0.447
1849	204340_at	TMEM187	0.091882	0.20498	-0.113098	0.844
1850	204341_at	TRIM16	0.11075	0.11003	0.00072	0.481
1851	204342_at	SLC25A24	0.30284	0.51165	-0.20881	0.964
1852	204346_s_at	RASSF1	0.43515	0.13373	0.30142	0.007
1853	204347_at	AK3L1	0.51924	0.60412	-0.08488	0.808
1854	204350_s_at	MED7	0.59389	0.58029	0.0136	0.449
1855	204352_at	TRAF5	0.71133	0.674	0.03733	0.296
1856	204354_at	POT1	0.4788	0.5011	-0.0223	0.598
1857	204369_at	PIK3CA	0.54525	0.57908	-0.03383	0.651
1858	204370_at	CLP1	0.51702	0.61529	-0.09827	0.819
1859	204372_s_at	KHSRP	0.60272	0.6795	-0.07678	0.851
1860	204373_s_at	CEP350	0.72311	0.83323	-0.11012	0.963
1861	204375_at	CLSTN3	0.47778	0.44063	0.03715	0.365
1862	204377_s_at	VPRBP	0.070652	0.097947	-0.027295	0.601
1863	204378_at	BCAS1	0.12419	0.10094	0.02325	0.447
1864	204382_at	NAT9	0.53421	0.46389	0.07032	0.235
1865	204394_at	SLC43A1	0.69869	0.55173	0.14696	0.053
1866	204401_at	KCNN4	0.41747	0.48821	-0.07074	0.749
1867	204404_at	SLC12A2	0.6733	0.64678	0.02652	0.348
1868	204407_at	TTF2	0.63475	0.52253	0.11222	0.16
1869	204408_at	APEX2	0.099971	0.014484	0.085487	0.193
1870	204411_at	KIF21B	0.31645	0.21057	0.10588	0.19
1871	204413_at	TRAF2	0.017651	0.11019	-0.092539	0.748
1872	204415_at	IFI6	0.78124	0.80371	-0.02247	0.688
1873	204421_s_at	FGF2	0.54157	0.50734	0.03423	0.35
1874	204423_at	MKLN1	0.52199	0.60545	-0.08346	0.748
1875	204425_at	ARHGAP4	0.52654	0.58797	-0.06143	0.786
1876	204426_at	TMED2	0.72185	0.76252	-0.04067	0.763
1877	204430_s_at	SLC2A5	0.36319	0.5252	-0.16201	0.933
1878	204432_at	SOX12	0.57372	0.47946	0.09426	0.185
1879	204433_s_at	SPATA2	0.26263	0.16784	0.09479	0.206
1880	204435_at	NUPL1	0.42928	0.5174	-0.08812	0.816
1881	204436_at	PLEKHO2	0.57898	0.54561	0.03337	0.362
1882	204439_at	IFI44L	0.66835	0.67674	-0.00839	0.604
1883	204440_at	CD83	0.71577	0.74536	-0.02959	0.634
1884	204441_s_at	POLA2	0.44863	0.23849	0.21014	0.008
1885	204444_at	KIF11	0.58364	0.53363	0.05001	0.203
1886	204448_s_at	PDCL	0.22782	0.17929	0.04853	0.338
1887	204453_at	ZNF84	0.34297	0.36127	-0.0183	0.586
1888	204458_at	PLA2G15	0.35763	0.37098	-0.01335	0.548

1889	204459_at	CSTF2	0.36127	0.4998	-0.13853	0.971
1890	204460_s_at	RAD1	0.82994	0.79624	0.0337	0.301
1891	204472_at	GEM	0.50988	0.57077	-0.06089	0.749
1892	204473_s_at	ZNF592	0.1004	0.18076	-0.08036	0.739
1893	204474_at	ZNF142	0.51699	0.65581	-0.13882	0.939
1894	204477_at	RABIF	0.48376	0.38224	0.10152	0.147
1895	204479_at	OSTF1	0.42512	0.52453	-0.09941	0.775
1896	204481_at	BRPF1	0.16831	0.11345	0.05486	0.308
1897	204483_at	ENO3	0.19566	0.28367	-0.08801	0.777
1898	204484_at	PIK3C2B	0.45217	0.49084	-0.03867	0.643
1899	204485_s_at	TOM1L1	0.48507	0.49722	-0.01215	0.557
1900	204488_at	DOLK	0.37543	0.33758	0.03785	0.372
1901	204492_at	ARHGAP11A	0.5833	0.52687	0.05643	0.298
1902	204502_at	SAMHD1	0.61299	0.57674	0.03625	0.323
1903	204504_s_at	HIRIP3	0.54417	0.51017	0.034	0.349
1904	204506_at	PPP3R1	0.32793	0.51968	-0.19175	0.961
1905	204510_at	CDC7	0.46317	0.4775	-0.01433	0.611
1906	204511_at	FARP2	0.4277	0.40258	0.02512	0.412
1907	204512_at	HIVEP1	0.55684	0.47377	0.08307	0.17
1908	204513_s_at	ELMO1	0.35665	0.35909	-0.00244	0.502
1909	204514_at	DPH2	0.33972	0.11662	0.2231	<0.001
1910	204516_at	ATXN7	0.71634	0.72124	-0.0049	0.525
1911	204521_at	C12orf24	0.71446	0.73916	-0.0247	0.756
1912	204522_at	DOM3Z	0.028846	0.13957	-0.110724	0.847
1913	204523_at	ZNF140	0.39098	0.50822	-0.11724	0.857
1914	204531_s_at	BRCA1	0.60956	0.54497	0.06459	0.267
1915	204533_at	CXCL10	0.13975	0.35157	-0.21182	1
1916	204544_at	HPS5	0.63784	0.71758	-0.07974	0.837
1917	204545_at	PEX6	0.1382	0.32884	-0.19064	0.922
1918	204546_at	KIAA0513	0.31624	0.3369	-0.02066	0.6
1919	204547_at	RAB40B	0.39118	0.34512	0.04606	0.38
1920	204552_at	INPP4A	0.43372	0.5648	-0.13108	0.944
1921	204554_at	PPP1R3D	0.35333	0.43996	-0.08663	0.77
1922	204558_at	RAD54L	0.68532	0.56656	0.11876	0.025
1923	204559_s_at	LSM7	0.87799	0.88049	-0.0025	0.559
1924	204562_at	IRF4	0.56314	0.48346	0.07968	0.24
1925	204563_at	SELL	0.3272	0.52144	-0.19424	0.963
1926	204565_at	ACOT13	0.51045	0.62654	-0.11609	0.867
1927	204566_at	PPM1D	0.77845	0.79702	-0.01857	0.648
1928	204568_at	KIAA0831	0.55502	0.63581	-0.08079	0.835
1929	204569_at	ICK	0.28874	0.49639	-0.20765	0.957
1930	204573_at	CROT	0.64932	0.68368	-0.03436	0.642
1931	204576_s_at	CLUAP1	0.13189	0.13081	0.00108	0.497
1932	204578_at	HISPPD2A	0.00013506	0.12606	-0.12592494	0.923
1933	204588_s_at	SLC7A7	0.19704	0.33285	-0.13581	0.877
1934	204593_s_at	SMCR7L	0.51239	0.42482	0.08757	0.176
1935	204599_s_at	MRPL28	0.42273	0.20925	0.21348	0.034
1936	204603_at	EXO1	0.8376	0.65409	0.18351	0.004
1937	204605_at	CGRRF1	0.59302	0.44556	0.14746	0.048
1938	204608_at	ASL	0.78747	0.8067	-0.01923	0.627
1939	204610_s_at	CCDC85B	0.71649	0.48298	0.23351	<0.001
1940	204612_at	PKIA	0.33396	0.29534	0.03862	0.334
1941	204613_at	PLCG2	0.79146	0.75373	0.03773	0.258
1942	204616_at	UCHL3	0.46416	0.66799	-0.20383	0.974

1943	204617_s_at	ACD	0.64709	0.73767	-0.09058	0.897
1944	204618_s_at	GABPB1	0.38976	0.40871	-0.01895	0.585
1945	204630_s_at	GOSR1	0.61935	0.76886	-0.14951	0.938
1946	204632_at	RPS6KA4	0.27405	0.027363	0.246687	<0.001
1947	204634_at	NEK4	0.69187	0.5873	0.10457	0.124
1948	204635_at	RPS6KA5	0.31871	0.48969	-0.17098	0.936
1949	204638_at	ACP5	0.3178	0.33651	-0.01871	0.573
1950	204641_at	NEK2	0.76628	0.76047	0.00581	0.486
1951	204642_at	S1PR1	0.18183	0.2303	-0.04847	0.648
1952	204646_at	DPYD	1.54E-05	0.18244	-0.182424625	0.892
1953	204649_at	TROAP	0.35212	0.50872	-0.1566	0.911
1954	204650_s_at	APBB3	0.18025	0.16819	0.01206	0.45
1955	204651_at	NRF1	0.65998	0.63566	0.02432	0.404
1956	204659_s_at	GFER	0.18124	0.16052	0.02072	0.431
1957	204662_at	CP110	0.25048	0.19225	0.05823	0.319
1958	204666_s_at	RP5-1000E10.4	0.7129	0.67734	0.03556	0.345
1959	204668_at	RNF24	0.48188	0.36456	0.11732	0.187
1960	204672_s_at	ANKRD6	0.32907	0.48135	-0.15228	0.889
1961	204674_at	LRMP	0.38073	0.45519	-0.07446	0.796
1962	204676_at	TMEM186	0.025114	0.071865	-0.046751	0.632
1963	204678_s_at	KCNK1	0.34644	0.37467	-0.02823	0.608
1964	204683_at	ICAM2	0.24399	0.23343	0.01056	0.476
1965	204687_at	DKFZP564O0823	0.27878	0.30718	-0.0284	0.572
1966	204688_at	SGCE	0.20995	0.43609	-0.22614	0.989
1967	204690_at	STX8	0.55391	0.6465	-0.09259	0.813
1968	204695_at	CDC25A	0.58287	0.50363	0.07924	0.094
1969	204698_at	ISG20	0.66769	0.73156	-0.06387	0.794
1970	204702_s_at	NFE2L3	0.56906	0.60205	-0.03299	0.657
1971	204703_at	IFT88	0.18479	0.39447	-0.20968	0.956
1972	204706_at	INPP5E	0.23113	0.33579	-0.10466	0.772
1973	204709_s_at	KIF23	0.74245	0.66247	0.07998	0.106
1974	204710_s_at	WIPI2	0.70853	0.75917	-0.05064	0.72
1975	204711_at	KIAA0753	0.44946	0.23953	0.20993	0.03
1976	204715_at	PANX1	0.50251	0.35591	0.1466	0.077
1977	204716_at	CCDC6	0.43866	0.43341	0.00525	0.454
1978	204717_s_at	SLC29A2	0.15241	0.05724	0.09517	0.246
1979	204720_s_at	DNAJC6	0.42642	0.53131	-0.10489	0.848
1980	204725_s_at	NCK1	0.50379	0.39985	0.10394	0.142
1981	204727_at	WDHD1	0.48058	0.49348	-0.0129	0.584
1982	204730_at	RIMS3	0.55887	0.70743	-0.14856	0.96
1983	204731_at	TGFBR3	0.71045	0.73383	-0.02338	0.59
1984	204735_at	PDE4A	0.4611	0.57574	-0.11464	0.823
1985	204739_at	CENPC1	0.46918	0.41421	0.05497	0.303
1986	204740_at	CNKSR1	0.030455	0.12288	-0.092425	0.828
1987	204742_s_at	PDS5B	0.35817	0.344	0.01417	0.452
1988	204744_s_at	IARS	0.72603	0.6085	0.11753	0.116
1989	204747_at	IFIT3	0.7302	0.7028	0.0274	0.322
1990	204757_s_at	C2CD2L	0.059183	0.22103	-0.161847	0.839
1991	204759_at	RCBTB2	0.45451	0.5923	-0.13779	0.881
1992	204761_at	USP6NL	0.50675	0.55961	-0.05286	0.699
1993	204765_at	ARHGEF5	0.31241	0.46569	-0.15328	0.894
1994	204766_s_at	NUDT1	0.52262	0.57629	-0.05367	0.77
1995	204767_s_at	FEN1	0.74992	0.48387	0.26605	<0.001
1996	204770_at	TAP2	0.00010529	0.052743	-0.05263771	0.712

1997	204771_s_at	TTF1	0.3409	0.59206	-0.25116	0.97
1998	204773_at	IL11RA	0.14883	0.31521	-0.16638	0.89
1999	204774_at	EVI2A	0.5468	0.70901	-0.16221	0.947
2000	204777_s_at	MAL	0.63506	0.68712	-0.05206	0.726
2001	204779_s_at	HOXB7	0.42134	0.48651	-0.06517	0.801
2002	204781_s_at	FAS	0.76867	0.64459	0.12408	0.003
2003	204786_s_at	IFNAR2	0.65798	0.47893	0.17905	0.042
2004	204788_s_at	PPOX	0.27869	0.32576	-0.04707	0.667
2005	204789_at	FMNL1	0.73286	0.73268	0.00018	0.502
2006	204790_at	SMAD7	0.60423	0.54663	0.0576	0.296
2007	204793_at	GPRASP1	0.11849	0.14521	-0.02672	0.578
2008	204794_at	DUSP2	0.6175	0.52663	0.09087	0.174
2009	204795_at	PRR3	0.70919	0.58569	0.1235	0.084
2010	204798_at	MYB	0.49919	0.51509	-0.0159	0.567
2011	204804_at	TRIM21	0.3941	0.30168	0.09242	0.205
2012	204805_s_at	H1FX	0.098953	0.27441	-0.175457	0.931
2013	204807_at	TMEM5	0.66329	0.59952	0.06377	0.22
2014	204809_at	CLPX	0.55209	0.77939	-0.2273	0.996
2015	204812_at	ZW10	0.39323	0.44075	-0.04752	0.68
2016	204813_at	MAPK10	0.31212	0.33315	-0.02103	0.569
2017	204817_at	ESPL1	0.58793	0.61304	-0.02511	0.629
2018	204821_at	BTN3A3	0.43241	0.28246	0.14995	0.029
2019	204822_at	TTK	0.50634	0.5073	-0.00096	0.515
2020	204824_at	ENDOG	0.44221	0.13044	0.31177	<0.001
2021	204825_at	MELK	0.73114	0.75713	-0.02599	0.699
2022	204827_s_at	CCNF	0.83434	0.68105	0.15329	<0.001
2023	204828_at	RAD9A	0.69441	0.72436	-0.02995	0.621
2024	204831_at	CDK8	0.78724	0.56577	0.22147	0.004
2025	204832_s_at	BMPR1A	0.24355	0.26447	-0.02092	0.556
2026	204834_at	FGL2	0.56877	0.7099	-0.14113	0.951
2027	204835_at	POLA1	0.7043	0.47361	0.23069	0.011
2028	204836_at	GLDC	0.43268	0.56724	-0.13456	0.92
2029	204838_s_at	MLH3	0.0066201	0.078754	-0.0721339	0.753
2030	204839_at	POP5	0.56448	0.44646	0.11802	0.162
2031	204840_s_at	EEA1	0.84741	0.75667	0.09074	0.019
2032	204847_at	ZBTB11	0.71906	0.61575	0.10331	0.088
2033	204849_at	TCFL5	0.46662	0.53731	-0.07069	0.718
2034	204852_s_at	PTPN7	0.28369	0.456	-0.17231	0.914
2035	204853_at	ORC2L	0.53077	0.17729	0.35348	<0.001
2036	204857_at	MAD1L1	0.56646	0.65435	-0.08789	0.843
2037	204858_s_at	TYMP	0.29255	0.34425	-0.0517	0.674
2038	204861_s_at	NAIP	0.10127	0.26486	-0.16359	0.876
2039	204862_s_at	NME3	0.63324	0.61974	0.0135	0.454
2040	204863_s_at	IL6ST	0.79897	0.86858	-0.06961	0.939
2041	204866_at	PHF16	0.68697	0.72739	-0.04042	0.649
2042	204867_at	GCHFR	0.47846	0.64362	-0.16516	0.947
2043	204868_at	ICT1	0.69538	0.70797	-0.01259	0.585
2044	204871_at	MTERF	0.44163	0.59664	-0.15501	0.953
2045	204880_at	MGMT	0.2424	0.4572	-0.2148	0.906
2046	204881_s_at	UGCG	0.69964	0.76345	-0.06381	0.819
2047	204883_s_at	HUS1	0.26777	0.41039	-0.14262	0.88
2048	204887_s_at	PLK4	0.70303	0.60088	0.10215	0.021
2049	204890_s_at	LCK	0.34331	0.50236	-0.15905	0.919
2050	204902_s_at	ATG4B	0.35038	0.23937	0.11101	0.172

2051	204905_s_at	EEF1E1	0.79886	0.70949	0.08937	0.023
2052	204909_at	DDX6	0.8133	0.81288	0.00042	0.524
2053	204912_at	IL10RA	0.49294	0.61422	-0.12128	0.903
2054	204917_s_at	MLLT3	0.4908	0.26544	0.22536	0.012
2055	204919_at	PRR4	0.11782	0.063229	0.054591	0.315
2056	204923_at	SASH3	0.62367	0.544	0.07967	0.238
2057	204928_s_at	SLC10A3	0.060146	0.064679	-0.004533	0.54
2058	204932_at	TNFRSF11B	0.5097	0.52455	-0.01485	0.562
2059	204936_at	MAP4K2	0.67554	0.48903	0.18651	0.031
2060	204937_s_at	ZNF274	0.41839	0.61566	-0.19727	0.963
2061	204946_s_at	TOP3A	0.49847	0.51432	-0.01585	0.591
2062	204949_at	ICAM3	0.77987	0.76727	0.0126	0.395
2063	204950_at	CARD8	0.22045	0.24188	-0.02143	0.601
2064	204959_at	MNDA	0.26831	0.46956	-0.20125	0.965
2065	204960_at	PTPRCAP	0.64518	0.71952	-0.07434	0.867
2066	204961_s_at	LOC648998	0.72353	0.76399	-0.04046	0.728
2067	204962_s_at	CENPA	0.88796	0.82503	0.06293	0.202
2068	204968_at	C6orf47	0.082922	0.122	-0.039078	0.633
2069	204970_s_at	MAFG	0.48917	0.72715	-0.23798	0.986
2070	204971_at	CSTA	0.36369	0.50395	-0.14026	0.924
2071	204976_s_at	AMMECR1	0.55967	0.45001	0.10966	0.105
2072	204977_at	DDX10	0.68677	0.56548	0.12129	0.023
2073	204978_at	SFRS16	0.60221	0.50994	0.09227	0.189
2074	204979_s_at	SH3BGR	0.0019613	0.074234	-0.0722727	0.745
2075	204981_at	SLC22A18	0.4659	0.32092	0.14498	0.101
2076	204982_at	GIT2	0.71586	0.65501	0.06085	0.149
2077	204985_s_at	TRAPPC6A	0.69655	0.69127	0.00528	0.491
2078	204992_s_at	PFN2	0.4768	0.63035	-0.15355	0.976
2079	204994_at	MX2	0.441	0.48722	-0.04622	0.653
2080	204995_at	CDK5R1	0.68733	0.72674	-0.03941	0.733
2081	205002_at	AHDC1	0.51627	0.47902	0.03725	0.369
2082	205003_at	DOCK4	0.2366	0.41491	-0.17831	0.91
2083	205004_at	NKRF	0.31184	0.091934	0.219906	0.007
2084	205010_at	GNL3L	0.78499	0.79282	-0.00783	0.561
2085	205012_s_at	HAGH	0.45402	0.33486	0.11916	0.151
2086	205013_s_at	ADORA2A	0.42101	0.40992	0.01109	0.465
2087	205020_s_at	ARL4A	0.2703	0.36189	-0.09159	0.774
2088	205025_at	ZBTB48	0.43423	0.52398	-0.08975	0.834
2089	205027_s_at	MAP3K8	0.71114	0.62722	0.08392	0.146
2090	205034_at	CCNE2	0.6109	0.50335	0.10755	0.032
2091	205035_at	CTDP1	0.12903	0.15398	-0.02495	0.568
2092	205036_at	LSM6	0.37249	0.3867	-0.01421	0.559
2093	205042_at	GNE	0.26666	0.28262	-0.01596	0.544
2094	205046_at	CENPE	0.76659	0.68676	0.07983	0.19
2095	205047_s_at	ASNS	0.63929	0.56664	0.07265	0.244
2096	205048_s_at	PSPH	0.0028731	0.16483	-0.1619569	0.957
2097	205049_s_at	CD79A	0.64999	0.67602	-0.02603	0.615
2098	205052_at	AUH	0.32627	0.37773	-0.05146	0.667
2099	205053_at	PRIM1	0.63572	0.69427	-0.05855	0.713
2100	205055_at	ITGAE	0.43581	0.30151	0.1343	0.058
2101	205060_at	PARG	0.19389	0.14495	0.04894	0.349
2102	205061_s_at	EXOSC9	0.69135	0.40282	0.28853	<0.001
2103	205063_at	SIP1	0.66845	0.74546	-0.07701	0.859
2104	205069_s_at	ARHGAP26	0.1272	0.10843	0.01877	0.538

2105	205070_at	ING3	0.78387	0.73449	0.04938	0.182
2106	205074_at	SLC22A5	0.30378	0.45424	-0.15046	0.952
2107	205078_at	PIGF	0.42171	0.48201	-0.0603	0.682
2108	205081_at	CRIP1	0.61158	0.62704	-0.01546	0.58
2109	205085_at	ORC1L	0.79244	0.74835	0.04409	0.215
2110	205087_at	RWDD3	0.65241	0.54162	0.11079	0.071
2111	205089_at	ZNF7	0.5382	0.55007	-0.01187	0.538
2112	205090_s_at	NAGPA	0.57145	0.57082	0.00063	0.509
2113	205094_at	PEX12	0.48879	0.52614	-0.03735	0.663
2114	205097_at	SLC26A2	0.73546	0.69085	0.04461	0.252
2115	205098_at	CCR1	0.22642	0.43778	-0.21136	0.937
2116	205101_at	CIITA	0.49378	0.53583	-0.04205	0.641
2117	205105_at	MAN2A1	0.54321	0.71786	-0.17465	0.952
2118	205107_s_at	EFNA4	0.32565	0.2043	0.12135	0.215
2119	205114_s_at	CCL3	0.4943	0.61325	-0.11895	0.832
2120	205124_at	LOC729991-MEF2B	0.55329	0.64716	-0.09387	0.898
2121	205126_at	VRK2	0.69502	0.65016	0.04486	0.242
2122	205129_at	NPM3	0.6736	0.66619	0.00741	0.451
2123	205132_at	ACTC1	0.0021085	0.01782	-0.0157115	0.59
2124	205133_s_at	HSPE1	0.71826	0.61848	0.09978	0.046
2125	205134_s_at	NUFIP1	0.65246	0.67161	-0.01915	0.614
2126	205140_at	FPGT	0.60846	0.61345	-0.00499	0.542
2127	205141_at	ANG	0.42956	0.39261	0.03695	0.366
2128	205145_s_at	LOC649851	0.47625	0.49463	-0.01838	0.591
2129	205158_at	RNASE4	0.27081	0.10573	0.16508	0.036
2130	205159_at	CSF2RB	0.40613	0.44326	-0.03713	0.625
2131	205162_at	ERCC8	0.093124	0.2518	-0.158676	0.914
2132	205168_at	DDR2	0.21978	0.45173	-0.23195	0.995
2133	205169_at	RBBP5	0.76188	0.75903	0.00285	0.478
2134	205170_at	STAT2	0.69848	0.79294	-0.09446	0.972
2135	205171_at	PTPN4	0.59434	0.61831	-0.02397	0.609
2136	205174_s_at	QPCT	0.14584	0.3903	-0.24446	0.981
2137	205176_s_at	ITGB3BP	0.31698	0.4789	-0.16192	0.926
2138	205178_s_at	RBBP6	0.81886	0.7836	0.03526	0.203
2139	205181_at	ZNF193	0.36277	0.24547	0.1173	0.179
2140	205188_s_at	SMAD5	0.3648	0.4483	-0.0835	0.82
2141	205189_s_at	FANCC	0.3685	0.30885	0.05965	0.339
2142	205190_at	PLS1	0.13604	0.25003	-0.11399	0.791
2143	205191_at	RP2	0.6244	0.55067	0.07373	0.238
2144	205192_at	MAP3K14	0.30515	0.39377	-0.08862	0.721
2145	205196_s_at	AP1S1	0.7488	0.70164	0.04716	0.223
2146	205198_s_at	ATP7A	0.13104	0.24676	-0.11572	0.868
2147	205205_at	RELB	0.56166	0.49825	0.06341	0.244
2148	205210_at	TGFBRAP1	0.7443	0.57707	0.16723	0.09
2149	205211_s_at	RIN1	0.14268	0.075036	0.067644	0.325
2150	205212_s_at	ACAP1	0.57503	0.40439	0.17064	0.02
2151	205215_at	RNF2	0.23465	0.13365	0.101	0.24
2152	205217_at	TIMM8A	0.37697	0.081169	0.295801	<0.001
2153	205218_at	POLR3F	0.27809	0.48925	-0.21116	0.985
2154	205220_at	NIACR2	0.39738	0.37087	0.02651	0.438
2155	205222_at	EHHADH	0.039571	0.17268	-0.133109	0.849
2156	205223_at	DEPDC5	0.14072	0.28572	-0.145	0.865
2157	205224_at	SURF2	0.48009	0.35813	0.12196	0.107
2158	205229_s_at	COCH	0.21275	0.35088	-0.13813	0.885

2159	205231_s_at	EPM2A	0.08922	0.25379	-0.16457	0.888
2160	205233_s_at	PAFAH2	0.21272	0.34609	-0.13337	0.886
2161	205235_s_at	KIF20B	0.22295	0.47937	-0.25642	0.995
2162	205238_at	TRMT2B	0.53398	0.65764	-0.12366	0.945
2163	205241_at	SCO2	0.69277	0.63068	0.06209	0.236
2164	205245_at	PARD6A	0.47219	0.64642	-0.17423	0.98
2165	205246_at	PEX13	0.76036	0.33768	0.42268	<0.001
2166	205247_at	NOTCH4	0.16853	0.23801	-0.06948	0.668
2167	205248_at	DOPEY2	0.60972	0.6335	-0.02378	0.575
2168	205249_at	EGR2	0.49993	0.61661	-0.11668	0.862
2169	205252_at	ZNF174	0.23284	0.34959	-0.11675	0.816
2170	205256_at	ZBTB39	0.10986	0.24033	-0.13047	0.845
2171	205260_s_at	ACYP1	0.78941	0.66177	0.12764	0.003
2172	205263_at	BCL10	0.42076	0.58079	-0.16003	0.931
2173	205264_at	CD3EAP	0.38604	0.18975	0.19629	<0.001
2174	205267_at	POU2AF1	0.78258	0.70506	0.07752	0.052
2175	205269_at	LCP2	0.66845	0.70738	-0.03893	0.707
2176	205273_s_at	PITRM1	0.24288	0.42628	-0.1834	0.926
2177	205283_at	FKTN	0.2024	0.14628	0.05612	0.307
2178	205291_at	IL2RB	0.45538	0.67484	-0.21946	0.987
2179	205292_s_at	HNRNPA2B1	0.93415	0.84985	0.0843	<0.001
2180	205296_at	RBL1	0.52661	0.54814	-0.02153	0.636
2181	205297_s_at	CD79B	0.75986	0.77926	-0.0194	0.617
2182	205298_s_at	BTN2A2	0.37952	0.32964	0.04988	0.326
2183	205300_s_at	SNRNP35	0.4444	0.20459	0.23981	0.008
2184	205301_s_at	OGG1	0.50422	0.42663	0.07759	0.159
2185	205307_s_at	KMO	0.081072	0.20408	-0.123008	0.816
2186	205308_at	FAM164A	0.43553	0.49724	-0.06171	0.725
2187	205310_at	FBXO46	0.58152	0.61893	-0.03741	0.644
2188	205312_at	SPI1	0.14492	0.2443	-0.09938	0.775
2189	205313_at	HNF1B	0.78099	0.78567	-0.00468	0.566
2190	205315_s_at	SNTB2	0.83496	0.80485	0.03011	0.3
2191	205317_s_at	SLC15A2	0.30056	0.35206	-0.0515	0.676
2192	205321_at	EIF2S3	0.66867	0.72993	-0.06126	0.765
2193	205322_s_at	MTF1	0.68765	0.63708	0.05057	0.311
2194	205327_s_at	ACVR2A	0.12128	0.18107	-0.05979	0.725
2195	205329_s_at	SNX4	0.67581	0.74214	-0.06633	0.799
2196	205333_s_at	RCE1	0.095032	0.26678	-0.171748	0.923
2197	205335_s_at	SRP19	0.35727	0.20747	0.1498	<0.001
2198	205339_at	STIL	0.61748	0.66518	-0.0477	0.762
2199	205340_at	ZBTB24	0.17316	0.30039	-0.12723	0.942
2200	205345_at	BARD1	0.61855	0.72687	-0.10832	0.941
2201	205346_at	ST3GAL2	0.72905	0.44745	0.2816	<0.001
2202	205347_s_at	TMSB15A	0.063967	0.10353	-0.039563	0.644
2203	205349_at	GNA15	0.39092	0.51181	-0.12089	0.827
2204	205352_at	SERPINI1	0.63533	0.6713	-0.03597	0.676
2205	205354_at	GAMT	0.22639	0.38717	-0.16078	0.933
2206	205355_at	ACADSB	0.74327	0.58101	0.16226	0.029
2207	205356_at	USP13	0.59885	0.72463	-0.12578	0.959
2208	205361_s_at	PFDN4	0.64974	0.68773	-0.03799	0.683
2209	205367_at	SH2B2	0.7688	0.78971	-0.02091	0.639
2210	205372_at	PLAG1	0.38551	0.50511	-0.1196	0.848
2211	205373_at	CTNNA2	0.44679	0.54267	-0.09588	0.846
2212	205376_at	INPP4B	0.25455	0.46388	-0.20933	0.979

2213	205393_s_at	CHEK1	0.76916	0.66391	0.10525	0.007
2214	205400_at	WAS	0.33725	0.34322	-0.00597	0.538
2215	205401_at	AGPS	0.69112	0.5817	0.10942	0.095
2216	205406_s_at	SPA17	0.40654	0.50761	-0.10107	0.818
2217	205407_at	RECK	0.44653	0.4953	-0.04877	0.66
2218	205408_at	MLLT10	0.7466	0.75177	-0.00517	0.57
2219	205411_at	STK4	0.58145	0.61165	-0.0302	0.61
2220	205412_at	ACAT1	0.64902	0.733	-0.08398	0.807
2221	205414_s_at	RICH2	0.30409	0.43997	-0.13588	0.814
2222	205416_s_at	ATXN3	0.33626	0.30583	0.03043	0.423
2223	205417_s_at	DAG1	0.607	0.41418	0.19282	0.019
2224	205419_at	GPR183	0.61933	0.81527	-0.19594	0.997
2225	205423_at	AP1B1	0.3533	0.26003	0.09327	0.182
2226	205425_at	HIP1	0.23502	0.47756	-0.24254	0.974
2227	205427_at	ZNF354A	0.29895	0.25086	0.04809	0.375
2228	205429_s_at	MPP6	0.27214	0.12268	0.14946	0.059
2229	205433_at	BCHE	0.13029	0.40474	-0.27445	0.985
2230	205436_s_at	H2AFX	0.75175	0.71137	0.04038	0.182
2231	205437_at	ZNF211	0.39739	0.39563	0.00176	0.507
2232	205441_at	OCEL1	0.2298	0.33599	-0.10619	0.805
2233	205443_at	SNAPC1	0.50075	0.45835	0.0424	0.363
2234	205449_at	SAC3D1	0.61643	0.54486	0.07157	0.145
2235	205452_at	PIGB	0.31517	0.47654	-0.16137	0.917
2236	205453_at	HOXB2	0.26607	0.4647	-0.19863	0.949
2237	205461_at	RAB35	0.72351	0.44305	0.28046	0.007
2238	205462_s_at	HPCAL1	0.38463	0.35681	0.02782	0.415
2239	205463_s_at	PDGFA	0.26642	0.47679	-0.21037	0.959
2240	205467_at	CASP10	0.1906	0.38822	-0.19762	0.941
2241	205469_s_at	IRF5	0.22289	0.43001	-0.20712	0.944
2242	205474_at	CRLF3	0.60925	0.71388	-0.10463	0.895
2243	205480_s_at	UGP2	0.24449	0.15413	0.09036	0.153
2244	205483_s_at	ISG15	0.76536	0.68644	0.07892	0.102
2245	205484_at	SIT1	0.24298	0.40469	-0.16171	0.871
2246	205486_at	TESK2	0.20969	0.4149	-0.20521	0.956
2247	205497_at	ZNF175	0.46163	0.41627	0.04536	0.37
2248	205500_at	C5	0.048684	0.275	-0.226316	0.868
2249	205504_at	BTK	0.72237	0.69052	0.03185	0.381
2250	205505_at	GCNT1	0.5195	0.61231	-0.09281	0.773
2251	205511_at	FLJ10038	0.73596	0.81074	-0.07478	0.926
2252	205512_s_at	AIFM1	0.53409	0.70781	-0.17372	0.959
2253	205518_s_at	CMAH	0.24937	0.43924	-0.18987	0.978
2254	205519_at	WDR76	0.46167	0.22341	0.23826	0.006
2255	205521_at	EXOG	0.4691	0.30143	0.16767	0.106
2256	205526_s_at	KATNA1	0.49032	0.59496	-0.10464	0.85
2257	205527_s_at	GEMIN4	0.72214	0.50737	0.21477	<0.001
2258	205536_at	VAV2	0.3039	0.30586	-0.00196	0.514
2259	205539_at	AVIL	0.36714	0.18395	0.18319	0.081
2260	205540_s_at	RRAGB	0.51342	0.44532	0.0681	0.266
2261	205541_s_at	GSPT2	0.44346	0.30425	0.13921	0.1
2262	205542_at	STEAP1	0.71532	0.83597	-0.12065	0.971
2263	205543_at	HSPA4L	0.71796	0.62113	0.09683	0.183
2264	205544_s_at	CR2	0.32538	0.53396	-0.20858	0.962
2265	205546_s_at	TYK2	0.64985	0.55425	0.0956	0.094
2266	205547_s_at	TAGLN	0.3944	0.24049	0.15391	0.079

2267	205550_s_at	BRE	0.58428	0.3503	0.23398	0.027
2268	205551_at	SV2B	0.51011	0.4718	0.03831	0.374
2269	205552_s_at	OAS1	0.75835	0.73037	0.02798	0.312
2270	205554_s_at	DNASE1L3	0.35654	0.55654	-0.2	0.947
2271	205558_at	TRAF6	0.39286	0.089741	0.303119	0.005
2272	205562_at	RPP38	0.12809	0.12735	0.00074	0.498
2273	205565_s_at	FXN	0.34251	0.53705	-0.19454	0.955
2274	205569_at	LAMP3	0.73428	0.71975	0.01453	0.429
2275	205570_at	PIP4K2A	0.54761	0.47633	0.07128	0.228
2276	205571_at	LIPT1	0.24235	0.40857	-0.16622	0.898
2277	205584_at	ALG13	0.28615	0.53995	-0.2538	0.993
2278	205585_at	ETV6	0.74831	0.80102	-0.05271	0.799
2279	205588_s_at	FGFR1OP	0.80076	0.75189	0.04887	0.217
2280	205590_at	RASGRP1	0.48066	0.65409	-0.17343	0.97
2281	205594_at	ZNF652	0.42892	0.43877	-0.00985	0.558
2282	205596_s_at	SMURF2	0.35693	0.41233	-0.0554	0.668
2283	205599_at	TRAF1	0.73657	0.80641	-0.06984	0.834
2284	205603_s_at	DIAPH2	0.13019	0.31446	-0.18427	0.89
2285	205607_s_at	SCYL3	0.26037	0.24207	0.0183	0.45
2286	205621_at	ALKBH1	0.3296	0.32038	0.00922	0.453
2287	205628_at	PRIM2	0.64	0.67439	-0.03439	0.756
2288	205633_s_at	ALAS1	0.53449	0.45127	0.08322	0.211
2289	205642_at	CEP110	0.57665	0.53905	0.0376	0.386
2290	205644_s_at	SNRPG	0.94213	0.86553	0.0766	0.001
2291	205647_at	RAD52	0.032882	0.13874	-0.105858	0.802
2292	205652_s_at	TTLL1	0.48157	0.45474	0.02683	0.411
2293	205657_at	HAAO	0.14788	0.18248	-0.0346	0.639
2294	205658_s_at	SNAPC4	0.50726	0.53234	-0.02508	0.609
2295	205659_at	HDAC9	0.0275	0.28402	-0.25652	0.988
2296	205664_at	KIN	0.41911	0.40219	0.01692	0.441
2297	205667_at	WRN	0.036365	0.0015843	0.0347807	0.381
2298	205668_at	LY75	0.70335	0.65381	0.04954	0.263
2299	205671_s_at	HLA-DOB	0.7664	0.7211	0.0453	0.211
2300	205672_at	XPA	0.37941	0.31788	0.06153	0.262
2301	205677_s_at	DLEU1	0.53297	0.60635	-0.07338	0.826
2302	205681_at	BCL2A1	0.70108	0.66723	0.03385	0.327
2303	205684_s_at	DENND4C	0.695	0.73513	-0.04013	0.687
2304	205687_at	UBFD1	0.37006	0.36614	0.00392	0.471
2305	205691_at	SYNGR3	0.21364	0.32054	-0.1069	0.845
2306	205692_s_at	CD38	0.73231	0.76582	-0.03351	0.753
2307	205704_s_at	ATP6V0A2	0.15461	0.11414	0.04047	0.221
2308	205705_at	ANKRD26	0.27321	0.12748	0.14573	0.11
2309	205707_at	IL17RA	0.37701	0.44655	-0.06954	0.711
2310	205708_s_at	TRPM2	0.10043	0.33485	-0.23442	0.965
2311	205716_at	SLC25A40	0.40875	0.15836	0.25039	0.025
2312	205718_at	ITGB7	0.68273	0.75865	-0.07592	0.874
2313	205733_at	BLM	0.82074	0.34552	0.47522	<0.001
2314	205740_s_at	RBM42	0.47895	0.28578	0.19317	0.06
2315	205746_s_at	ADAM17	0.15874	0.26731	-0.10857	0.844
2316	205748_s_at	RNF126	0.62709	0.47171	0.15538	0.007
2317	205750_at	BPHL	0.20993	0.40876	-0.19883	0.95
2318	205756_s_at	F8	0.36049	0.329	0.03149	0.378
2319	205761_s_at	DUS4L	0.52116	0.46501	0.05615	0.318
2320	205770_at	GSR	0.6264	0.67772	-0.05132	0.753

2321	205773_at	CPEB3	0.38522	0.62082	-0.2356	0.987
2322	205774_at	F12	0.46348	0.50407	-0.04059	0.666
2323	205775_at	FAM50B	0.0028523	0.26716	-0.2643077	0.97
2324	205780_at	BIK	0.36385	0.53869	-0.17484	0.915
2325	205781_at	C16orf7	0.22852	0.13769	0.09083	0.207
2326	205788_s_at	ZC3H11A	0.75576	0.57048	0.18528	<0.001
2327	205790_at	SKAP1	0.35323	0.50705	-0.15382	0.902
2328	205796_at	TCP11L1	0.25203	0.44037	-0.18834	0.898
2329	205801_s_at	RASGRP3	0.45461	0.48119	-0.02658	0.618
2330	205804_s_at	TRAF3IP3	0.41712	0.22486	0.19226	0.01
2331	205807_s_at	TUFT1	0.15335	0.26704	-0.11369	0.813
2332	205809_s_at	WASL	0.89301	0.85761	0.0354	0.067
2333	205811_at	POLG2	0.59409	0.53979	0.0543	0.274
2334	205830_at	CLGN	0.14074	0.24239	-0.10165	0.778
2335	205832_at	CPA4	0.1611	0.1829	-0.0218	0.558
2336	205839_s_at	BZRAP1	0.57411	0.59939	-0.02528	0.589
2337	205841_at	JAK2	0.37568	0.34539	0.03029	0.431
2338	205848_at	GAS2	0.58188	0.72458	-0.1427	0.979
2339	205849_s_at	UQCRB	0.76526	0.70934	0.05592	0.178
2340	205851_at	NME6	0.39467	0.47535	-0.08068	0.737
2341	205855_at	ZNF197	0.19344	0.23461	-0.04117	0.622
2342	205859_at	LY86	0.18158	0.35284	-0.17126	0.895
2343	205861_at	SPIB	0.50022	0.42337	0.07685	0.184
2344	205865_at	ARID3A	0.46186	0.56303	-0.10117	0.832
2345	205873_at	PIGL	0.39605	0.40949	-0.01344	0.553
2346	205877_s_at	ZC3H7B	0.22506	0.15592	0.06914	0.266
2347	205881_at	ZNF74	0.054447	0.060829	-0.006382	0.542
2348	205884_at	ITGA4	0.6769	0.53493	0.14197	0.027
2349	205890_s_at	GABBR1	0.4544	0.64954	-0.19514	0.974
2350	205895_s_at	NOLC1	0.7488	0.58062	0.16818	<0.001
2351	205901_at	PNOC	0.62205	0.71857	-0.09652	0.867
2352	205902_at	KCNN3	0.52572	0.59438	-0.06866	0.791
2353	205909_at	POLE2	0.90923	0.69231	0.21692	<0.001
2354	205917_at	ZNF264	0.6436	0.69246	-0.04886	0.772
2355	205922_at	VNN2	0.069835	0.40119	-0.331355	1
2356	205928_at	ZNF443	0.55543	0.55233	0.0031	0.482
2357	205930_at	GTF2E1	0.19124	0.01825	0.17299	0.002
2358	205932_s_at	MSX1	0.40344	0.49749	-0.09405	0.859
2359	205933_at	SETBP1	0.56646	0.6971	-0.13064	0.814
2360	205945_at	IL6R	0.48169	0.54057	-0.05888	0.739
2361	205953_at	LRIG2	0.696	0.67056	0.02544	0.363
2362	205955_at	TAF6L	0.33976	0.37822	-0.03846	0.626
2363	205963_s_at	DNAJA3	0.76312	0.61524	0.14788	0.001
2364	205964_at	ZNF426	0.098302	0.18935	-0.091048	0.778
2365	205965_at	BATF	0.73234	0.71615	0.01619	0.422
2366	205966_at	TAF13	0.6324	0.54569	0.08671	0.179
2367	205967_at	HIST1H4C	0.51712	0.55782	-0.0407	0.659
2368	205978_at	KL	0.085899	0.40542	-0.319521	0.995
2369	205981_s_at	ING2	0.68225	0.58674	0.09551	0.165
2370	205990_s_at	WNT5A	0.085676	0.33155	-0.245874	0.997
2371	205992_s_at	IL15	0.55615	0.51383	0.04232	0.234
2372	205994_at	ELK4	0.2986	0.345	-0.0464	0.651
2373	205996_s_at	AK2	0.53711	0.5252	0.01191	0.435
2374	206006_s_at	KIAA1009	0.33052	0.18072	0.1498	0.101

2375	206016_at	CCDC22	0.66489	0.76479	-0.0999	0.832
2376	206020_at	SOCS6	0.17212	0.28109	-0.10897	0.872
2377	206026_s_at	TNFAIP6	0.3714	0.48728	-0.11588	0.845
2378	206031_s_at	USP5	0.32688	0.18423	0.14265	0.135
2379	206034_at	SERPINB8	0.35656	0.21124	0.14532	0.072
2380	206035_at	REL	0.51717	0.41265	0.10452	0.232
2381	206037_at	CCBL1	0.50069	0.37003	0.13066	0.076
2382	206038_s_at	NR2C2	0.76071	0.76525	-0.00454	0.535
2383	206039_at	RAB33A	0.59408	0.58815	0.00593	0.502
2384	206044_s_at	BRAF	0.53113	0.65972	-0.12859	0.969
2385	206045_s_at	NOL4	0.22978	0.49288	-0.2631	0.997
2386	206050_s_at	RNH1	0.58874	0.69436	-0.10562	0.777
2387	206052_s_at	SLBP	0.90198	0.69261	0.20937	<0.001
2388	206053_at	ZNF510	0.22987	0.094557	0.135313	0.153
2389	206055_s_at	SNRPA1	0.6835	0.71953	-0.03603	0.72
2390	206059_at	ZNF91	0.55419	0.69526	-0.14107	0.899
2391	206061_s_at	DICER1	0.86243	0.86829	-0.00586	0.588
2392	206074_s_at	HMGA1	0.6658	0.64179	0.02401	0.387
2393	206082_at	HCP5	0.48052	0.41776	0.06276	0.323
2394	206096_at	ZNF35	0.29048	0.43964	-0.14916	0.893
2395	206098_at	ZBTB6	0.48535	0.6501	-0.16475	0.945
2396	206102_at	GINS1	0.70371	0.57476	0.12895	0.002
2397	206106_at	MAPK12	0.16448	0.13904	0.02544	0.419
2398	206108_s_at	SFRS6	0.31871	0.62598	-0.30727	0.999
2399	206110_at	HIST1H3J	0.32436	0.46089	-0.13653	0.882
2400	206115_at	EGR3	0.23983	0.51836	-0.27853	0.987
2401	206129_s_at	ARSB	0.29726	0.27014	0.02712	0.426
2402	206133_at	XAF1	0.78061	0.78358	-0.00297	0.544
2403	206141_at	MOCS3	0.23029	0.22075	0.00954	0.5
2404	206150_at	CD27	0.3501	0.49077	-0.14067	0.853
2405	206158_s_at	CNBP	0.75433	0.7043	0.05003	0.236
2406	206181_at	SLAMF1	0.72793	0.69247	0.03546	0.344
2407	206182_at	ZNF134	0.00016831	0.14677	-0.14660169	0.843
2408	206183_s_at	HERC3	0.073912	0.061288	0.012624	0.458
2409	206184_at	CRKL	0.43037	0.46765	-0.03728	0.636
2410	206188_at	ZNF623	0.70439	0.70766	-0.00327	0.542
2411	206194_at	HOXC4	0.037636	0.16463	-0.126994	0.978
2412	206200_s_at	ANXA11	0.73051	0.77125	-0.04074	0.744
2413	206219_s_at	VAV1	0.72439	0.76308	-0.03869	0.737
2414	206233_at	B4GALT6	0.63485	0.67597	-0.04112	0.704
2415	206235_at	LIG4	0.62846	0.62419	0.00427	0.465
2416	206238_s_at	YAF2	0.54732	0.33191	0.21541	0.013
2417	206240_s_at	ZNF136	0.58275	0.60978	-0.02703	0.637
2418	206241_at	KPNA5	0.08648	0.23046	-0.14398	0.871
2419	206247_at	MICB	0.41043	0.42424	-0.01381	0.55
2420	206255_at	BLK	0.75159	0.64865	0.10294	0.072
2421	206257_at	CCDC9	0.25655	0.24707	0.00948	0.47
2422	206261_at	ZNF239	0.035672	0.18844	-0.152768	0.882
2423	206263_at	FMO4	0.17624	0.35721	-0.18097	0.915
2424	206278_at	PTAFR	0.38223	0.39732	-0.01509	0.582
2425	206279_at	PRKY	0.044825	0.28678	-0.241955	0.992
2426	206302_s_at	NUDT4	0.64075	0.70539	-0.06464	0.745
2427	206308_at	TRDMT1	0.33852	0.35729	-0.01877	0.558
2428	206313_at	HLA-DOA	0.1398	0.22282	-0.08302	0.804

2429	206314_at	ZNF167	0.16697	0.091646	0.075324	0.179
2430	206316_s_at	KNTC1	0.65282	0.51915	0.13367	0.031
2431	206335_at	GALNS	0.44639	0.54819	-0.1018	0.863
2432	206337_at	CCR7	0.69465	0.80325	-0.1086	0.941
2433	206352_s_at	PEX10	0.51889	0.52003	-0.00114	0.53
2434	206357_at	OPA3	0.4806	0.44262	0.03798	0.356
2435	206364_at	KIF14	0.88737	0.84568	0.04169	0.176
2436	206398_s_at	CD19	0.42033	0.44045	-0.02012	0.553
2437	206412_at	FER	0.36532	0.17339	0.19193	0.051
2438	206437_at	S1PR4	0.22884	0.40482	-0.17598	0.926
2439	206440_at	LIN7A	0.4597	0.62125	-0.16155	0.973
2440	206445_s_at	PRMT1	0.85919	0.84881	0.01038	0.411
2441	206451_at	TBCCD1	0.65551	0.72353	-0.06802	0.826
2442	206478_at	KIAA0125	0.076863	0.2873	-0.210437	0.971
2443	206491_s_at	NAPA	0.77608	0.80447	-0.02839	0.738
2444	206492_at	FHIT	0.23882	0.47527	-0.23645	0.986
2445	206495_s_at	HINFP	0.29673	0.15429	0.14244	0.133
2446	206497_at	C7orf44	0.20376	0.24516	-0.0414	0.633
2447	206500_s_at	C14orf106	0.91945	0.89692	0.02253	0.221
2448	206507_at	ZSCAN12	0.012358	0.038185	-0.025827	0.602
2449	206508_at	CD70	0.75511	0.67405	0.08106	0.152
2450	206512_at	ZRSR1	0.029633	0.082615	-0.052982	0.719
2451	206513_at	AIM2	0.53505	0.4037	0.13135	0.205
2452	206515_at	CYP4F3	0.43089	0.46554	-0.03465	0.627
2453	206521_s_at	GTF2A1	0.58602	0.56444	0.02158	0.415
2454	206530_at	RAB30	0.58905	0.55416	0.03489	0.367
2455	206533_at	CHRNA5	0.084387	0.20094	-0.116553	0.824
2456	206536_s_at	XIAP	0.61318	0.47913	0.13405	0.066
2457	206540_at	GLB1L	0.1182	0.20296	-0.08476	0.73
2458	206542_s_at	SMARCA2	0.71626	0.76291	-0.04665	0.844
2459	206550_s_at	NUP155	0.68028	0.49983	0.18045	<0.001
2460	206553_at	OAS2	0.5704	0.46204	0.10836	0.094
2461	206566_at	SLC7A1	0.22053	0.5115	-0.29097	1
2462	206583_at	ZNF673	0.33821	0.20024	0.13797	0.068
2463	206584_at	LY96	0.57222	0.29426	0.27796	0.002
2464	206586_at	CNR2	0.58172	0.53926	0.04246	0.338
2465	206587_at	CCT6B	0.29166	0.26423	0.02743	0.405
2466	206589_at	GFI1	0.65129	0.67406	-0.02277	0.622
2467	206592_s_at	AP3D1	0.31029	0.32964	-0.01935	0.609
2468	206593_s_at	MED22	0.39057	0.36271	0.02786	0.382
2469	206613_s_at	TAF1A	0.51501	0.44402	0.07099	0.259
2470	206618_at	IL18R1	0.59737	0.70148	-0.10411	0.884
2471	206620_at	GRAP	0.58039	0.49145	0.08894	0.161
2472	206621_s_at	EIF4H	0.52095	0.45945	0.0615	0.247
2473	206632_s_at	APOBEC3B	0.078289	0.20328	-0.124991	0.802
2474	206636_at	RASA2	0.79087	0.73902	0.05185	0.241
2475	206641_at	TNFRSF17	0.542	0.5957	-0.0537	0.756
2476	206648_at	ZNF571	0.37306	0.39799	-0.02493	0.602
2477	206649_s_at	TFE3	0.5413	0.50921	0.03209	0.376
2478	206650_at	IQCC	0.18355	0.15054	0.03301	0.401
2479	206652_at	ZMYM5	0.87504	0.8647	0.01034	0.401
2480	206654_s_at	POLR3G	0.68048	0.4384	0.24208	0.003
2481	206656_s_at	C20orf3	0.32736	0.24824	0.07912	0.25
2482	206659_at	FLJ14082	0.48288	0.53408	-0.0512	0.674

2483	206661_at	DBF4B	0.63452	0.69386	-0.05934	0.736
2484	206667_s_at	SCAMP1	0.73841	0.73582	0.00259	0.509
2485	206683_at	ZNF165	0.042176	0.086761	-0.044585	0.874
2486	206686_at	PDK1	0.18743	0.30744	-0.12001	0.881
2487	206687_s_at	PTPN6	0.83767	0.79968	0.03799	0.256
2488	206688_s_at	CPSF4	0.22544	0.3382	-0.11276	0.825
2489	206693_at	IL7	0.77716	0.80825	-0.03109	0.706
2490	206703_at	CHRNB1	0.20919	0.14794	0.06125	0.288
2491	206704_at	CLCN5	0.25196	0.24384	0.00812	0.458
2492	206708_at	FOXN2	0.61329	0.55295	0.06034	0.361
2493	206724_at	CBX4	0.51184	0.28586	0.22598	0.004
2494	206729_at	TNFRSF8	0.49233	0.57625	-0.08392	0.785
2495	206734_at	JRKL	0.20339	0.18549	0.0179	0.455
2496	206752_s_at	DFFB	0.53091	0.3623	0.16861	0.053
2497	206756_at	CHST7	0.55496	0.70258	-0.14762	0.917
2498	206759_at	FCER2	0.34326	0.49388	-0.15062	0.895
2499	206766_at	ITGA10	0.33898	0.297	0.04198	0.403
2500	206788_s_at	CBFB	0.73714	0.72426	0.01288	0.421
2501	206789_s_at	POU2F1	0.76666	0.82938	-0.06272	0.86
2502	206790_s_at	NDUFB1	0.69248	0.67381	0.01867	0.404
2503	206809_s_at	HNRNPA3	0.62999	0.58366	0.04633	0.325
2504	206818_s_at	CNNM2	0.43852	0.21912	0.2194	0.044
2505	206825_at	OXTR	0.76979	0.8037	-0.03391	0.7
2506	206828_at	TXK	0.31417	0.55168	-0.23751	0.992
2507	206833_s_at	ACYP2	0.54617	0.6444	-0.09823	0.922
2508	206838_at	TBX19	0.51306	0.50391	0.00915	0.456
2509	206845_s_at	RNF40	0.27063	0.39856	-0.12793	0.876
2510	206848_at	FAM36A	0.90377	0.89179	0.01198	0.318
2511	206853_s_at	MAP3K7	0.14396	0.30367	-0.15971	0.921
2512	206855_s_at	HYAL2	0.11555	0.28277	-0.16722	0.97
2513	206858_s_at	HOXC6	0.10833	0.23389	-0.12556	0.886
2514	206860_s_at	MIOS	0.36202	0.39559	-0.03357	0.646
2515	206861_s_at	CGGBP1	0.29816	0.28486	0.0133	0.462
2516	206875_s_at	SLK	0.54775	0.51562	0.03213	0.416
2517	206907_at	TNFSF9	0.69201	0.66664	0.02537	0.372
2518	206918_s_at	CPNE1	0.19872	0.35221	-0.15349	0.931
2519	206925_at	ST8SIA4	0.52509	0.6946	-0.16951	0.975
2520	206928_at	ZNF124	0.65529	0.64639	0.0089	0.482
2521	206931_at	ZNF141	0.51306	0.50291	0.01015	0.462
2522	206949_s_at	RUSC1	0.2879	0.25776	0.03014	0.367
2523	206956_at	BGLAP	0.11107	0.19305	-0.08198	0.791
2524	206958_s_at	UPF3A	0.43721	0.45513	-0.01792	0.591
2525	206967_at	CCNT1	0.57419	0.50274	0.07145	0.301
2526	206975_at	LTA	0.58824	0.58089	0.00735	0.453
2527	206976_s_at	HSPH1	0.78507	0.24593	0.53914	<0.001
2528	206983_at	CCR6	0.75725	0.76918	-0.01193	0.585
2529	206992_s_at	ATP5S	0.63397	0.6291	0.00487	0.477
2530	207000_s_at	PPP3CC	0.54109	0.58442	-0.04333	0.638
2531	207002_s_at	PLAGL1	0.39161	0.52054	-0.12893	0.879
2532	207006_s_at	CCDC106	0.40097	0.50288	-0.10191	0.819
2533	207030_s_at	CSRP2	0.53519	0.63131	-0.09612	0.789
2534	207038_at	SLC16A6	0.78115	0.75227	0.02888	0.307
2535	207040_s_at	ST13	0.6982	0.68935	0.00885	0.434
2536	207046_at	HIST2H4A	0.27621	0.43471	-0.1585	0.97

2537	207061_at	ERN1	0.40885	0.37163	0.03722	0.385
2538	207064_s_at	AOC2	0.085085	0.072622	0.012463	0.446
2539	207071_s_at	ACO1	0.31936	0.3779	-0.05854	0.668
2540	207076_s_at	ASS1	0.32447	0.50379	-0.17932	0.958
2541	207079_s_at	MED6	0.877	0.87057	0.00643	0.444
2542	207088_s_at	SLC25A11	0.59793	0.75397	-0.15604	0.942
2543	207103_at	KCND2	0.32025	0.49963	-0.17938	0.938
2544	207113_s_at	TNF	0.6112	0.65144	-0.04024	0.666
2545	207121_s_at	MAPK6	0.69969	0.62566	0.07403	0.089
2546	207124_s_at	GNB5	0.68151	0.56731	0.1142	0.074
2547	207125_at	ZNF225	0.10618	0.094724	0.011456	0.474
2548	207127_s_at	HNRNPH3	0.6405	0.66289	-0.02239	0.605
2549	207128_s_at	ZNF223	0.22277	0.24258	-0.01981	0.579
2550	207143_at	CDK6	0.67908	0.64517	0.03391	0.604
2551	207153_s_at	GLMN	0.77201	0.59148	0.18053	<0.001
2552	207156_at	HIST1H2AG	0.51199	0.36169	0.1503	0.091
2553	207157_s_at	GNG5	0.83517	0.76035	0.07482	0.044
2554	207160_at	IL12A	0.47377	0.55318	-0.07941	0.75
2555	207163_s_at	AKT1	0.65154	0.55195	0.09959	0.02
2556	207170_s_at	LETMD1	0.58221	0.46754	0.11467	0.14
2557	207176_s_at	CD80	0.8205	0.81807	0.00243	0.479
2558	207178_s_at	FRK	0.32186	0.4676	-0.14574	0.905
2559	207181_s_at	CASP7	0.65827	0.71778	-0.05951	0.767
2560	207183_at	GPR19	0.25543	0.34316	-0.08773	0.774
2561	207186_s_at	BPTF	0.78656	0.86059	-0.07403	0.912
2562	207196_s_at	TNIP1	0.73482	0.70828	0.02654	0.386
2563	207198_s_at	LIMS1	0.64358	0.67946	-0.03588	0.693
2564	207219_at	ZNF643	0.23651	0.27117	-0.03466	0.608
2565	207232_s_at	DZIP3	0.36881	0.27821	0.0906	0.22
2566	207234_at	RFX3	0.060695	0.1138	-0.053105	0.665
2567	207243_s_at	CALM2	0.88591	0.8605	0.02541	0.124
2568	207245_at	UGT2B17	0.0069844	0.065911	-0.0589266	0.724
2569	207283_at	RPL23AP32	0.51118	0.60717	-0.09599	0.864
2570	207286_at	CEP135	0.51931	0.5148	0.00451	0.496
2571	207291_at	PRRG4	0.41138	0.43926	-0.02788	0.612
2572	207305_s_at	KIAA1012	0.53837	0.53042	0.00795	0.482
2573	207315_at	CD226	0.29225	0.40095	-0.1087	0.812
2574	207332_s_at	TFRC	0.76104	0.45958	0.30146	<0.001
2575	207338_s_at	ZNF200	0.28538	0.33734	-0.05196	0.697
2576	207339_s_at	LTB	0.66878	0.77527	-0.10649	0.881
2577	207347_at	ERCC6	0.61066	0.64103	-0.03037	0.618
2578	207350_s_at	VAMP4	0.27529	0.23216	0.04313	0.37
2579	207375_s_at	IL15RA	0.0558	0.21258	-0.15678	0.888
2580	207386_at	CYP7B1	0.33847	0.46133	-0.12286	0.841
2581	207394_at	ZNF137	0.17809	0.3065	-0.12841	0.872
2582	207396_s_at	ALG3	0.41204	0.66794	-0.2559	0.988
2583	207399_at	BFSP2	0.17545	0.36303	-0.18758	0.907
2584	207405_s_at	RAD17	0.69727	0.81394	-0.11667	0.955
2585	207417_s_at	ZNF177	0.19275	0.16602	0.02673	0.413
2586	207426_s_at	TNFSF4	0.61064	0.74068	-0.13004	0.95
2587	207431_s_at	DEGS1	0.71488	0.53675	0.17813	0.039
2588	207433_at	IL10	0.59714	0.76026	-0.16312	0.95
2589	207435_s_at	SRRM2	0.71998	0.76469	-0.04471	0.763
2590	207438_s_at	SNUPN	0.25057	0.36198	-0.11141	0.849

2591	207440_at	SLC35A2	0.29525	0.22683	0.06842	0.277
2592	207446_at	TLR6	0.29011	0.23105	0.05906	0.282
2593	207469_s_at	PIR	0.52312	0.60085	-0.07773	0.832
2594	207480_s_at	MEIS2	0.32918	0.46018	-0.131	0.864
2595	207492_at	NGLY1	0.7708	0.80926	-0.03846	0.84
2596	207508_at	ATP5G3	0.81063	0.59305	0.21758	<0.001
2597	207513_s_at	ZNF189	0.66468	0.64846	0.01622	0.436
2598	207515_s_at	POLR1C	0.87961	0.80295	0.07666	0.003
2599	207518_at	DGKE	0.12726	0.022999	0.104261	0.179
2600	207520_at	TROVE2	0.59413	0.61693	-0.0228	0.589
2601	207525_s_at	GIPC1	0.22382	0.35252	-0.1287	0.846
2602	207528_s_at	SLC7A11	0.24566	0.54147	-0.29581	1
2603	207536_s_at	TNFRSF9	0.57113	0.55811	0.01302	0.453
2604	207540_s_at	SYK	0.37024	0.50834	-0.1381	0.9
2605	207541_s_at	EXOSC10	0.6739	0.4594	0.2145	0.001
2606	207543_s_at	P4HA1	0.59107	0.48345	0.10762	0.151
2607	207545_s_at	NUMB	0.56387	0.75537	-0.1915	0.999
2608	207551_s_at	MSL3	0.55351	0.51765	0.03586	0.38
2609	207556_s_at	DGKZ	0.65672	0.70448	-0.04776	0.756
2610	207559_s_at	ZMYM3	0.29356	0.31737	-0.02381	0.6
2611	207563_s_at	OGT	0.69034	0.74741	-0.05707	0.815
2612	207583_at	ABCD2	0.48461	0.47375	0.01086	0.455
2613	207585_s_at	RPL36AL	0.69426	0.64046	0.0538	0.268
2614	207604_s_at	SLC4A7	0.60563	0.43906	0.16657	0.057
2615	207606_s_at	ARHGAP12	0.48757	0.49107	-0.0035	0.52
2616	207610_s_at	EMR2	0.091804	0.21027	-0.118466	0.82
2617	207614_s_at	CUL1	0.66159	0.46962	0.19197	<0.001
2618	207618_s_at	BCS1L	0.61636	0.59128	0.02508	0.394
2619	207621_s_at	PEMT	0.6845	0.67941	0.00509	0.48
2620	207622_s_at	ABCF2	0.58808	0.46973	0.11835	0.015
2621	207624_s_at	RPGR	0.47299	0.53465	-0.06166	0.748
2622	207627_s_at	TFCP2	0.3926	0.41553	-0.02293	0.573
2623	207628_s_at	WBSCR22	0.61071	0.62346	-0.01275	0.571
2624	207641_at	TNFRSF13B	0.27959	0.25075	0.02884	0.41
2625	207643_s_at	TNFRSF1A	0.16034	0.26782	-0.10748	0.846
2626	207655_s_at	BLNK	0.57248	0.70259	-0.13011	0.918
2627	207677_s_at	NCF4	0.089627	0.37148	-0.281853	0.994
2628	207687_at	INHBC	0.28185	0.20305	0.0788	0.357
2629	207707_s_at	SEC13	0.64041	0.77394	-0.13353	0.981
2630	207711_at	C20orf117	0.33573	0.43845	-0.10272	0.833
2631	207713_s_at	RBCK1	0.75046	0.55731	0.19315	0.017
2632	207714_s_at	SERPINH1	0.31934	0.36912	-0.04978	0.677
2633	207722_s_at	BTBD2	0.37343	0.54034	-0.16691	0.892
2634	207727_s_at	MUTYH	0.53373	0.50687	0.02686	0.41
2635	207734_at	LAX1	0.44748	0.49971	-0.05223	0.699
2636	207735_at	RNF125	0.39826	0.48767	-0.08941	0.8
2637	207740_s_at	NUP62	0.85898	0.52537	0.33361	<0.001
2638	207746_at	POLQ	0.74161	0.7338	0.00781	0.476
2639	207753_at	ZNF304	0.70825	0.56731	0.14094	0.013
2640	207761_s_at	METTL7A	0.38134	0.50765	-0.12631	0.821
2641	207765_s_at	KIAA1539	0.38219	0.22592	0.15627	0.062
2642	207777_s_at	SP140	0.47726	0.40665	0.07061	0.24
2643	207785_s_at	RBPJ	0.40216	0.48211	-0.07995	0.772
2644	207786_at	CYP2R1	0.093832	0.20686	-0.113028	0.736

2645	207805_s_at	PSMD9	0.74919	0.71088	0.03831	0.33
2646	207809_s_at	ATP6AP1	0.49783	0.46701	0.03082	0.378
2647	207812_s_at	GORASP2	0.73498	0.49822	0.23676	<0.001
2648	207813_s_at	FDXR	0.79877	0.77935	0.01942	0.365
2649	207819_s_at	ABCB4	0.66436	0.5775	0.08686	0.105
2650	207824_s_at	MAZ	0.62669	0.67486	-0.04817	0.727
2651	207826_s_at	ID3	0.69872	0.7061	-0.00738	0.564
2652	207830_s_at	PPP1R8	0.91781	0.85115	0.06666	0.103
2653	207839_s_at	C9orf127	0.15932	0.19041	-0.03109	0.605
2654	207842_s_at	CASC3	0.47911	0.33379	0.14532	0.103
2655	207845_s_at	ANAPC10	0.731	0.85648	-0.12548	0.998
2656	207855_s_at	CLCC1	0.56011	0.54863	0.01148	0.441
2657	207856_s_at	LOC150776	0.74998	0.52437	0.22561	<0.001
2658	207861_at	CCL22	0.53317	0.65658	-0.12341	0.879
2659	207871_s_at	ST7	0.74027	0.76772	-0.02745	0.662
2660	207877_s_at	NVL	0.44238	0.17276	0.26962	0.002
2661	207891_s_at	HAUS7	0.68663	0.6769	0.00973	0.457
2662	207900_at	CCL17	0.40082	0.52993	-0.12911	0.828
2663	207901_at	IL12B	0.366	0.57576	-0.20976	0.979
2664	207904_s_at	LNPEP	0.42464	0.40027	0.02437	0.431
2665	207907_at	TNFSF14	0.68881	0.73354	-0.04473	0.767
2666	207917_at	NUDT13	0.47837	0.31214	0.16623	0.066
2667	207922_s_at	MAEA	0.50219	0.21866	0.28353	0.013
2668	207945_s_at	CSNK1D	0.38794	0.33537	0.05257	0.346
2669	207957_s_at	PRKCB	0.73114	0.76194	-0.0308	0.725
2670	207966_s_at	GLG1	0.84457	0.86654	-0.02197	0.681
2671	207980_s_at	CITED2	0.12028	0.21564	-0.09536	0.986
2672	207996_s_at	C18orf1	0.45636	0.56039	-0.10403	0.885
2673	208003_s_at	NFAT5	0.89001	0.93313	-0.04312	0.99
2674	208018_s_at	HCK	0.54759	0.59974	-0.05215	0.702
2675	208021_s_at	RFC1	0.70729	0.65592	0.05137	0.277
2676	208024_s_at	DGCR6	0.28704	0.43039	-0.14335	0.903
2677	208047_s_at	NAB1	0.45034	0.47058	-0.02024	0.567
2678	208050_s_at	CASP2	0.6254	0.63686	-0.01146	0.578
2679	208051_s_at	PAIP1	0.72586	0.60249	0.12337	0.061
2680	208055_s_at	HERC4	0.23583	0.31497	-0.07914	0.768
2681	208056_s_at	CBFA2T3	0.49054	0.52162	-0.03108	0.616
2682	208066_s_at	GTF2B	0.63701	0.73057	-0.09356	0.909
2683	208070_s_at	REV3L	0.48327	0.57977	-0.0965	0.851
2684	208072_s_at	DGKD	0.48204	0.54999	-0.06795	0.727
2685	208074_s_at	AP2S1	0.68725	0.7859	-0.09865	0.979
2686	208076_at	HIST1H4D	0.2154	0.12917	0.08623	0.236
2687	208081_s_at	ZNF442	0.40711	0.41938	-0.01227	0.529
2688	208089_s_at	TDRD3	0.23898	0.15628	0.0827	0.301
2689	208091_s_at	ECOP	0.61738	0.58876	0.02862	0.375
2690	208093_s_at	NDEL1	0.42504	0.36754	0.0575	0.282
2691	208094_s_at	CCDC130	0.41287	0.37396	0.03891	0.404
2692	208095_s_at	SRP72	0.73606	0.7843	-0.04824	0.857
2693	208101_s_at	URM1	0.60362	0.40858	0.19504	0.024
2694	208104_s_at	TSC22D4	0.38124	0.43006	-0.04882	0.687
2695	208107_s_at	LOC81691	0.54217	0.55822	-0.01605	0.574
2696	208117_s_at	LAS1L	0.55075	0.46983	0.08092	0.17
2697	208119_s_at	ZNF93	0.20088	0.39634	-0.19546	0.892
2698	208152_s_at	DDX21	0.6471	0.57232	0.07478	0.124

2699	208154_at	LOC51336	0.63199	0.57961	0.05238	0.322
2700	208165_s_at	PRSS16	0.076136	0.32507	-0.248934	0.987
2701	208184_s_at	TRAPPC10	0.19503	0.30871	-0.11368	0.829
2702	208190_s_at	LSR	0.05704	0.3206	-0.26356	0.98
2703	208194_s_at	STAM2	0.17213	0.26732	-0.09519	0.774
2704	208195_at	TTN	0.26593	0.39779	-0.13186	0.88
2705	208249_s_at	TGDS	0.39773	0.24108	0.15665	<0.001
2706	208270_s_at	RNPEP	0.43701	0.34445	0.09256	0.227
2707	208290_s_at	EIF5	0.67939	0.29835	0.38104	<0.001
2708	208302_at	HMHB1	0.3327	0.45499	-0.12229	0.794
2709	208309_s_at	MALT1	0.31276	0.44202	-0.12926	0.829
2710	208319_s_at	RBM3	0.59011	0.32882	0.26129	0.001
2711	208328_s_at	MEF2A	0.72161	0.83651	-0.1149	0.969
2712	208336_s_at	GPSN2	0.84665	0.83434	0.01231	0.376
2713	208361_s_at	POLR3D	0.23649	0.32691	-0.09042	0.765
2714	208368_s_at	BRCA2	0.7257	0.73024	-0.00454	0.524
2715	208382_s_at	DMC1	0.32301	0.42437	-0.10136	0.838
2716	208398_s_at	TBPL1	0.55736	0.6086	-0.05124	0.711
2717	208405_s_at	CD164	0.79554	0.88429	-0.08875	0.934
2718	208424_s_at	CIAPIN1	0.70733	0.54148	0.16585	0.018
2719	208433_s_at	LRP8	0.57108	0.59894	-0.02786	0.685
2720	208436_s_at	IRF7	0.69942	0.59286	0.10656	0.043
2721	208438_s_at	FGR	0.2583	0.3931	-0.1348	0.766
2722	208442_s_at	ATM	0.35895	0.41792	-0.05897	0.736
2723	208447_s_at	PRPS1	0.82092	0.71141	0.10951	0.03
2724	208453_s_at	XPNPEP1	0.54272	0.57828	-0.03556	0.621
2725	208460_at	GJC1	0.028385	0.1488	-0.120415	0.877
2726	208498_s_at	AMY1A	0.25421	0.38031	-0.1261	0.852
2727	208499_s_at	DNAJC3	0.48553	0.5345	-0.04897	0.689
2728	208503_s_at	GATAD1	0.73265	0.77076	-0.03811	0.716
2729	208506_at	HIST1H3F	0.33037	0.24825	0.08212	0.198
2730	208511_at	PTTG3	0.16573	0.30676	-0.14103	0.887
2731	208515_at	HIST1H2BM	0.46535	0.41978	0.04557	0.343
2732	208524_at	GPR15	0.60285	0.6094	-0.00655	0.542
2733	208534_s_at	RASA4	0.32223	0.36644	-0.04421	0.664
2734	208553_at	HIST1H1E	0.79003	0.47958	0.31045	<0.001
2735	208576_s_at	HIST1H3B	0.29822	0.24994	0.04828	0.351
2736	208588_at	FKSG2	0.12734	0.36295	-0.23561	0.892
2737	208612_at	PDIA3	0.64722	0.49374	0.15348	0.012
2738	208613_s_at	FLNB	0.29555	0.41558	-0.12003	0.777
2739	208616_s_at	PTP4A2	0.75009	0.72864	0.02145	0.385
2740	208619_at	DDB1	0.69908	0.30877	0.39031	<0.001
2741	208620_at	PCBP1	0.45623	0.50374	-0.04751	0.671
2742	208623_s_at	EZR	0.7773	0.78985	-0.01255	0.607
2743	208624_s_at	EIF4G1	0.91356	0.91899	-0.00543	0.584
2744	208626_s_at	VAT1	0.51018	0.44177	0.06841	0.284
2745	208627_s_at	YBX1	0.71444	0.76048	-0.04604	0.688
2746	208629_s_at	HADHA	0.81188	0.89184	-0.07996	0.976
2747	208632_at	RNF10	0.090094	0.19729	-0.107196	0.881
2748	208633_s_at	MACF1	0.75282	0.81514	-0.06232	0.922
2749	208636_at	ACTN1	0.65609	0.70303	-0.04694	0.718
2750	208641_s_at	RAC1	0.75824	0.78373	-0.02549	0.643
2751	208642_s_at	XRCC5	0.86982	0.57288	0.29694	<0.001
2752	208644_at	PARP1	0.15624	0.30486	-0.14862	0.798

2753	208645_s_at	RPS14	0.91217	0.88973	0.02244	0.104
2754	208647_at	FDFT1	0.81352	0.38074	0.43278	<0.001
2755	208649_s_at	VCP	0.53247	0.42337	0.1091	0.052
2756	208652_at	PPP2CA	0.55455	0.4939	0.06065	0.332
2757	208655_at	CCNI	0.51916	0.58286	-0.0637	0.748
2758	208659_at	CLIC1	0.86669	0.80948	0.05721	0.031
2759	208660_at	CS	0.41909	0.37287	0.04622	0.358
2760	208664_s_at	TTC3	0.87626	0.90522	-0.02896	0.844
2761	208670_s_at	EID1	0.79012	0.74283	0.04729	0.11
2762	208671_at	SERINC1	0.58828	0.45356	0.13472	0.014
2763	208675_s_at	DDOST	0.47751	0.51694	-0.03943	0.677
2764	208676_s_at	PA2G4	0.55789	0.54747	0.01042	0.483
2765	208677_s_at	BSG	0.64458	0.54646	0.09812	0.136
2766	208678_at	ATP6V1E1	0.79709	0.78787	0.00922	0.426
2767	208679_s_at	ARPC2	0.61648	0.55316	0.06332	0.191
2768	208680_at	PRDX1	0.83447	0.64312	0.19135	<0.001
2769	208684_at	COPA	0.67802	0.78409	-0.10607	0.993
2770	208686_s_at	BRD2	0.32475	0.51151	-0.18676	0.949
2771	208689_s_at	RPN2	0.81753	0.76747	0.05006	0.248
2772	208690_s_at	PDLIM1	0.7663	0.79596	-0.02966	0.731
2773	208692_at	RPS3	0.85341	0.86328	-0.00987	0.634
2774	208693_s_at	GARS	0.78384	0.50851	0.27533	0.003
2775	208695_s_at	RPL39	0.89871	0.88358	0.01513	0.239
2776	208696_at	CCT5	0.80527	0.68415	0.12112	0.005
2777	208697_s_at	EIF3E	0.86366	0.86852	-0.00486	0.592
2778	208698_s_at	NONO	0.67403	0.77615	-0.10212	0.888
2779	208700_s_at	TKT	0.50375	0.72103	-0.21728	0.998
2780	208709_s_at	NRD1	0.6951	0.72325	-0.02815	0.688
2781	208713_at	HNRNPUL1	0.36888	0.54904	-0.18016	0.963
2782	208714_at	NDUFV1	0.71977	0.82741	-0.10764	0.947
2783	208715_at	TMCO1	0.47481	0.46661	0.0082	0.495
2784	208717_at	OXA1L	0.31209	0.55138	-0.23929	0.973
2785	208719_s_at	DDX17	0.42403	0.56293	-0.1389	0.897
2786	208720_s_at	RBM39	0.82973	0.6187	0.21103	<0.001
2787	208721_s_at	ANAPC5	0.85974	0.81804	0.0417	0.228
2788	208723_at	USP11	0.51982	0.5857	-0.06588	0.846
2789	208724_s_at	RAB1A	0.59978	0.47839	0.12139	0.035
2790	208726_s_at	EIF2S2	0.85097	0.64407	0.2069	0.004
2791	208736_at	ARPC3	0.87798	0.8708	0.00718	0.462
2792	208737_at	ATP6V1G1	0.38978	0.43667	-0.04689	0.661
2793	208741_at	SAP18	0.21669	0.1709	0.04579	0.381
2794	208745_at	ATP5L	0.65135	0.65741	-0.00606	0.523
2795	208753_s_at	NAP1L1	0.78575	0.73856	0.04719	0.256
2796	208756_at	EIF3I	0.61175	0.82284	-0.21109	1
2797	208757_at	TMED9	0.28066	0.57318	-0.29252	0.995
2798	208758_at	ATIC	0.73426	0.72724	0.00702	0.437
2799	208759_at	NCSTN	0.18286	0.29575	-0.11289	0.853
2800	208760_at	UBE2I	0.3141	0.49718	-0.18308	0.949
2801	208762_at	SUMO1	0.67991	0.70312	-0.02321	0.614
2802	208763_s_at	TSC22D3	0.20018	0.25071	-0.05053	0.715
2803	208765_s_at	HNRNPR	0.62743	0.54777	0.07966	0.174
2804	208771_s_at	LTA4H	0.43855	0.59913	-0.16058	0.889
2805	208777_s_at	PSMD11	0.51453	0.65693	-0.1424	0.974
2806	208784_s_at	KLHDC3	0.49559	0.45482	0.04077	0.365

2807	208785_s_at	MAP1LC3B	0.30339	0.017415	0.285975	<0.001
2808	208787_at	MRPL3	0.91907	0.823	0.09607	<0.001
2809	208796_s_at	CCNG1	0.38849	0.70566	-0.31717	1
2810	208799_at	PSMB5	0.76714	0.80367	-0.03653	0.772
2811	208807_s_at	CHD3	0.39159	0.43228	-0.04069	0.635
2812	208808_s_at	HMGB2	0.75455	0.81224	-0.05769	0.674
2813	208813_at	GOT1	0.71034	0.43332	0.27702	<0.001
2814	208819_at	RAB8A	0.6737	0.67947	-0.00577	0.537
2815	208820_at	PTK2	0.74044	0.75752	-0.01708	0.618
2816	208821_at	SNRPB	0.89269	0.87788	0.01481	0.29
2817	208822_s_at	DAP3	0.82284	0.82745	-0.00461	0.544
2818	208827_at	PSMB6	0.89579	0.93333	-0.03754	0.944
2819	208828_at	POLE3	0.71971	0.55024	0.16947	0.025
2820	208829_at	TAPBP	0.74437	0.74463	-0.00026	0.499
2821	208830_s_at	SUPT6H	0.55596	0.6444	-0.08844	0.789
2822	208833_s_at	ATXN10	0.32208	0.34292	-0.02084	0.589
2823	208836_at	ATP1B3	0.69561	0.68412	0.01149	0.442
2824	208837_at	TMED3	0.58011	0.33462	0.24549	0.012
2825	208839_s_at	CAND1	0.6225	0.59913	0.02337	0.391
2826	208841_s_at	G3BP2	0.62349	0.61669	0.0068	0.47
2827	208845_at	VDAC3	0.92773	0.92237	0.00536	0.481
2828	208847_s_at	ADH5	0.32609	0.49936	-0.17327	0.867
2829	208853_s_at	CANX	0.81105	0.7795	0.03155	0.379
2830	208854_s_at	STK24	0.36485	0.16048	0.20437	<0.001
2831	208857_s_at	PCMT1	0.75437	0.8006	-0.04623	0.727
2832	208858_s_at	FAM62A	0.58524	0.55119	0.03405	0.355
2833	208861_s_at	ATRX	0.77464	0.54072	0.23392	0.001
2834	208862_s_at	CTNND1	0.11998	0.27535	-0.15537	0.94
2835	208872_s_at	REEP5	0.66953	0.81045	-0.14092	0.922
2836	208876_s_at	PAK2	0.62978	0.58729	0.04249	0.351
2837	208880_s_at	PRPF6	0.24457	0.4389	-0.19433	0.948
2838	208883_at	UBR5	0.23535	0.19227	0.04308	0.495
2839	208885_at	LCP1	0.58096	0.58176	-0.0008	0.515
2840	208886_at	H1F0	0.26756	0.41855	-0.15099	0.94
2841	208887_at	EIF3G	0.81008	0.74947	0.06061	0.123
2842	208894_at	HLA-DRA	0.56149	0.62348	-0.06199	0.736
2843	208897_s_at	DDX18	0.68583	0.46894	0.21689	0.014
2844	208900_s_at	TOP1	0.85005	0.8903	-0.04025	0.766
2845	208905_at	CYCS	0.87522	0.77694	0.09828	0.03
2846	208906_at	BSCL2	0.23758	0.37954	-0.14196	0.891
2847	208909_at	UQCRFS1	0.85353	0.83592	0.01761	0.38
2848	208910_s_at	C1QBP	0.80928	0.84233	-0.03305	0.82
2849	208911_s_at	PDHB	0.69294	0.74917	-0.05623	0.835
2850	208912_s_at	CNP	0.33989	0.3124	0.02749	0.407
2851	208916_at	SLC1A5	0.70768	0.49796	0.20972	0.016
2852	208920_at	SRI	0.31804	0.43354	-0.1155	0.854
2853	208922_s_at	NXF1	0.64887	0.56892	0.07995	0.209
2854	208923_at	CYFIP1	0.5492	0.60688	-0.05768	0.687
2855	208924_at	RNF11	0.6716	0.75794	-0.08634	0.831
2856	208925_at	CLDND1	0.63375	0.49715	0.1366	0.072
2857	208926_at	NEU1	0.40093	0.55446	-0.15353	0.857
2858	208927_at	SPOP	0.73572	0.75515	-0.01943	0.666
2859	208928_at	POR	0.51332	0.5199	-0.00658	0.522
2860	208932_at	PPP4C	0.66094	0.66204	-0.0011	0.533

2861	208938_at	PRCC	0.64905	0.65865	-0.0096	0.559
2862	208941_s_at	SEPHS1	0.67991	0.6441	0.03581	0.298
2863	208942_s_at	SEC62	0.78114	0.80596	-0.02482	0.63
2864	208944_at	TGFBR2	0.49707	0.68371	-0.18664	0.988
2865	208946_s_at	BECN1	0.51099	0.3141	0.19689	0.053
2866	208949_s_at	LGALS3	0.4834	0.63646	-0.15306	0.959
2867	208954_s_at	LARP5	0.14393	0.25508	-0.11115	0.806
2868	208959_s_at	ERP44	0.62205	0.61865	0.0034	0.493
2869	208964_s_at	FADS1	0.49243	0.45687	0.03556	0.356
2870	208965_s_at	IFI16	0.8845	0.90098	-0.01648	0.734
2871	208969_at	NDUFA9	0.87287	0.87586	-0.00299	0.544
2872	208971_at	UROD	0.31522	0.54429	-0.22907	0.976
2873	208972_s_at	ATP5G1	0.74869	0.69167	0.05702	0.069
2874	208973_at	ERI3	0.50116	0.47605	0.02511	0.396
2875	208979_at	NCOA6	0.27368	0.42426	-0.15058	0.901
2876	208980_s_at	UBC	0.48746	0.38139	0.10607	0.252
2877	208985_s_at	EIF3J	0.63896	0.50409	0.13487	0.107
2878	208986_at	TCF12	0.55491	0.39804	0.15687	0.022
2879	208987_s_at	KDM2A	0.75223	0.77788	-0.02565	0.704
2880	208991_at	STAT3	0.6708	0.71346	-0.04266	0.726
2881	208995_s_at	PPIG	0.82565	0.79235	0.0333	0.309
2882	208996_s_at	POLR2C	0.66731	0.66049	0.00682	0.488
2883	208998_at	UCP2	0.59889	0.55075	0.04814	0.346
2884	208999_at	SEPT8	0.44124	0.52625	-0.08501	0.811
2885	209001_s_at	ANAPC13	0.38645	0.62687	-0.24042	0.986
2886	209002_s_at	CALCOCO1	0.75626	0.58456	0.1717	0.04
2887	209004_s_at	FBXL5	0.56826	0.64038	-0.07212	0.764
2888	209007_s_at	C1orf63	0.75052	0.66847	0.08205	0.125
2889	209014_at	MAGED1	0.54937	0.34407	0.2053	0.014
2890	209015_s_at	DNAJB6	0.44927	0.38683	0.06244	0.302
2891	209017_s_at	LONP1	0.70107	0.53612	0.16495	0.011
2892	209018_s_at	PINK1	0.24975	0.18669	0.06306	0.337
2893	209020_at	C20orf111	0.66666	0.5557	0.11096	0.133
2894	209023_s_at	STAG2	0.91426	0.91029	0.00397	0.414
2895	209028_s_at	ABI1	0.68969	0.77687	-0.08718	0.924
2896	209029_at	COPS7A	0.28862	0.29937	-0.01075	0.539
2897	209030_s_at	CADM1	0.23652	0.38487	-0.14835	0.893
2898	209034_at	PNRC1	0.64165	0.46278	0.17887	0.045
2899	209037_s_at	EHD1	0.48883	0.64983	-0.161	0.895
2900	209040_s_at	PSMB8	0.76524	0.80559	-0.04035	0.839
2901	209042_s_at	UBE2G2	0.41957	0.68142	-0.26185	0.976
2902	209043_at	PAPSS1	0.3786	0.40033	-0.02173	0.575
2903	209046_s_at	GABARAPL2	0.56215	0.62136	-0.05921	0.756
2904	209049_s_at	ZMYND8	0.24428	0.58203	-0.33775	0.997
2905	209050_s_at	RALGDS	0.57426	0.63044	-0.05618	0.755
2906	209052_s_at	WHSC1	0.76895	0.71372	0.05523	0.172
2907	209055_s_at	CDC5L	0.95045	0.88358	0.06687	<0.001
2908	209058_at	EDF1	0.72046	0.72771	-0.00725	0.485
2909	209068_at	HNRPDL	0.4316	0.64237	-0.21077	0.966
2910	209075_s_at	ISCU	0.7529	0.77661	-0.02371	0.663
2911	209076_s_at	WDR45L	0.35592	0.14614	0.20978	0.028
2912	209077_at	TXN2	0.49677	0.49231	0.00446	0.482
2913	209083_at	CORO1A	0.84561	0.80876	0.03685	0.285
2914	209084_s_at	RAB28	0.1814	0.27418	-0.09278	0.79

2915	209088_s_at	UBN1	0.934	0.95232	-0.01832	0.812
2916	209090_s_at	SH3GLB1	0.43305	0.41648	0.01657	0.462
2917	209092_s_at	GLOD4	0.55574	0.40559	0.15015	0.056
2918	209095_at	DLD	0.77197	0.67232	0.09965	0.123
2919	209096_at	UBE2V2	0.57429	0.43817	0.13612	0.036
2920	209100_at	IFRD2	0.79764	0.61762	0.18002	<0.001
2921	209102_s_at	HBP1	0.50004	0.37475	0.12529	<0.001
2922	209103_s_at	UFD1L	0.80141	0.8163	-0.01489	0.638
2923	209104_s_at	NHP2	0.91396	0.8756	0.03836	0.084
2924	209106_at	NCOA1	0.76033	0.6824	0.07793	0.237
2925	209110_s_at	RGL2	0.6263	0.58619	0.04011	0.355
2926	209111_at	RNF5	0.61624	0.6792	-0.06296	0.77
2927	209112_at	CDKN1B	0.67126	0.59604	0.07522	0.032
2928	209113_s_at	HMG20B	0.33269	0.28216	0.05053	0.326
2929	209115_at	UBA3	0.71288	0.76585	-0.05297	0.835
2930	209117_at	WBP2	0.58118	0.58941	-0.00823	0.544
2931	209118_s_at	TUBA1A	0.80725	0.7577	0.04955	0.132
2932	209122_at	ADFP	0.16611	0.32305	-0.15694	0.931
2933	209123_at	QDPR	0.2777	0.39474	-0.11704	0.774
2934	209124_at	MYD88	0.61174	0.32322	0.28852	<0.001
2935	209127_s_at	SART3	0.87604	0.90907	-0.03303	0.874
2936	209130_at	SNAP23	0.72879	0.73695	-0.00816	0.58
2937	209132_s_at	COMMD4	0.50716	0.72559	-0.21843	0.997
2938	209135_at	ASPH	0.49819	0.46493	0.03326	0.471
2939	209136_s_at	USP10	0.92307	0.9156	0.00747	0.408
2940	209139_s_at	PRKRA	0.51155	0.65276	-0.14121	0.922
2941	209142_s_at	UBE2G1	0.80631	0.73778	0.06853	0.034
2942	209143_s_at	CLNS1A	0.65931	0.59734	0.06197	0.151
2943	209146_at	SC4MOL	0.64755	0.27283	0.37472	<0.001
2944	209148_at	RXRB	0.32728	0.2981	0.02918	0.421
2945	209150_s_at	TM9SF1	0.27907	0.43461	-0.15554	0.972
2946	209154_at	TAX1BP3	0.54839	0.65004	-0.10165	0.791
2947	209155_s_at	NT5C2	0.32993	0.39448	-0.06455	0.757
2948	209157_at	DNAJA2	0.50439	0.37964	0.12475	0.146
2949	209158_s_at	CYTH2	0.53566	0.5043	0.03136	0.384
2950	209162_s_at	PRPF4	0.84381	0.80849	0.03532	0.29
2951	209163_at	CYB561	0.3535	0.4778	-0.1243	0.855
2952	209165_at	AATF	0.60333	0.55946	0.04387	0.339
2953	209166_s_at	MAN2B1	0.38875	0.44268	-0.05393	0.682
2954	209171_at	ITPA	0.55371	0.61299	-0.05928	0.686
2955	209174_s_at	QRICH1	0.66759	0.60304	0.06455	0.244
2956	209175_at	SEC23IP	0.29229	0.38364	-0.09135	0.803
2957	209177_at	NDUFAF3	0.52009	0.67263	-0.15254	0.893
2958	209178_at	DHX38	0.55071	0.57753	-0.02682	0.606
2959	209179_s_at	MBOAT7	0.21904	0.17965	0.03939	0.379
2960	209187_at	DR1	0.36045	0.61001	-0.24956	0.992
2961	209190_s_at	DIAPH1	0.59157	0.5635	0.02807	0.385
2962	209191_at	TUBB6	0.21736	0.44533	-0.22797	0.986
2963	209193_at	PIM1	0.012995	0.10605	-0.093055	0.761
2964	209194_at	CETN2	0.59046	0.59226	-0.0018	0.516
2965	209196_at	WDR46	0.30154	0.079754	0.221786	0.03
2966	209198_s_at	SYT11	0.54136	0.58548	-0.04412	0.652
2967	209199_s_at	MEF2C	0.78172	0.73277	0.04895	0.207
2968	209205_s_at	LMO4	0.60163	0.6204	-0.01877	0.595

2969	209206_at	SEC22B	0.63984	0.51718	0.12266	<0.001
2970	209208_at	MPDU1	0.7385	0.85857	-0.12007	0.928
2971	209210_s_at	FERMT2	0.40835	0.54846	-0.14011	0.946
2972	209213_at	CBR1	0.26048	0.4585	-0.19802	0.963
2973	209215_at	MFSD10	0.53876	0.56787	-0.02911	0.615
2974	209217_s_at	WDR45	0.20554	0.22482	-0.01928	0.676
2975	209219_at	RDBP	0.83911	0.81384	0.02527	0.342
2976	209221_s_at	OSBPL2	0.20433	0.11837	0.08596	0.277
2977	209224_s_at	NDUFA2	0.84403	0.67277	0.17126	<0.001
2978	209229_s_at	SAPS1	0.56261	0.56899	-0.00638	0.539
2979	209231_s_at	DCTN5	0.4943	0.61084	-0.11654	0.847
2980	209233_at	EMG1	0.84485	0.85158	-0.00673	0.535
2981	209234_at	KIF1B	0.44083	0.47123	-0.0304	0.622
2982	209239_at	NFKB1	0.51425	0.40094	0.11331	0.141
2983	209249_s_at	GHITM	0.75652	0.78769	-0.03117	0.617
2984	209252_at	HARS2	0.40734	0.47469	-0.06735	0.671
2985	209254_at	KLHDC10	0.27859	0.24725	0.03134	0.409
2986	209258_s_at	SMC3	0.83944	0.90381	-0.06437	0.987
2987	209265_s_at	METTL3	0.73627	0.7094	0.02687	0.401
2988	209267_s_at	SLC39A8	0.63915	0.61356	0.02559	0.441
2989	209268_at	VPS45	0.64881	0.46606	0.18275	0.042
2990	209275_s_at	CLN3	0.31601	0.31624	-0.00023	0.475
2991	209276_s_at	GLRX	0.6541	0.80805	-0.15395	0.988
2992	209279_s_at	NSDHL	0.54951	0.56325	-0.01374	0.556
2993	209282_at	PRKD2	0.46164	0.45338	0.00826	0.485
2994	209285_s_at	C3orf63	0.65741	0.5836	0.07381	0.199
2995	209287_s_at	CDC42EP3	0.70522	0.61935	0.08587	0.063
2996	209295_at	TNFRSF10B	0.85429	0.75966	0.09463	<0.001
2997	209300_s_at	NECAP1	0.25473	0.29011	-0.03538	0.615
2998	209301_at	CA2	0.51843	0.51463	0.0038	0.493
2999	209302_at	POLR2H	0.82551	0.812	0.01351	0.387
3000	209303_at	NDUFS4	0.57083	0.7072	-0.13637	0.92
3001	209306_s_at	SWAP70	0.25585	0.51044	-0.25459	0.975
3002	209308_s_at	BNIP2	0.60899	0.62871	-0.01972	0.577
3003	209310_s_at	CASP4	0.30469	0.33536	-0.03067	0.575
3004	209311_at	BCL2L2	0.20705	0.37304	-0.16599	0.793
3005	209313_at	GPN1	0.32596	0.28524	0.04072	0.35
3006	209316_s_at	HBS1L	0.32416	0.43122	-0.10706	0.802
3007	209321_s_at	ADCY3	0.27466	0.42932	-0.15466	0.88
3008	209323_at	PRKRIR	0.61844	0.63049	-0.01205	0.549
3009	209324_s_at	RGS16	0.61503	0.52583	0.0892	0.099
3010	209331_s_at	MAX	0.24315	0.18566	0.05749	0.315
3011	209333_at	ULK1	0.23506	0.15116	0.0839	0.255
3012	209336_at	PWP2	0.44401	0.035205	0.408805	<0.001
3013	209339_at	SIAH2	0.69642	0.55136	0.14506	0.012
3014	209340_at	UAP1	0.6189	0.59272	0.02618	0.372
3015	209342_s_at	IKBKB	0.43547	0.44341	-0.00794	0.556
3016	209344_at	TPM4	0.68277	0.77749	-0.09472	0.926
3017	209349_at	RAD50	0.59259	0.6409	-0.04831	0.733
3018	209352_s_at	SIN3B	0.28494	0.37968	-0.09474	0.814
3019	209354_at	TNFRSF14	0.67035	0.64878	0.02157	0.409
3020	209358_at	TAF11	0.27683	0.45763	-0.1808	0.927
3021	209361_s_at	PCBP4	0.35726	0.39937	-0.04211	0.622
3022	209363_s_at	MED21	0.28208	0.33583	-0.05375	0.755

3023	209367_at	STXBP2	0.47248	0.58127	-0.10879	0.823
3024	209374_s_at	IGHM	0.12045	0.3737	-0.25325	0.997
3025	209375_at	XPC	0.77933	0.76654	0.01279	0.434
3026	209377_s_at	HMGN3	0.41748	0.39726	0.02022	0.416
3027	209378_s_at	KIAA1128	0.34072	0.40523	-0.06451	0.721
3028	209383_at	DDIT3	0.6381	0.044795	0.593305	<0.001
3029	209385_s_at	PROSC	0.80334	0.86873	-0.06539	0.881
3030	209390_at	TSC1	0.59874	0.52673	0.07201	0.225
3031	209391_at	DPM2	0.50354	0.58911	-0.08557	0.781
3032	209392_at	ENPP2	0.41111	0.65326	-0.24215	0.992
3033	209393_s_at	EIF4E2	0.63801	0.62182	0.01619	0.42
3034	209394_at	ASMTL	0.18394	0.30793	-0.12399	0.798
3035	209398_at	HIST1H1C	0.46922	0.66169	-0.19247	0.993
3036	209403_at	LOC653498	0.53486	0.7142	-0.17934	0.963
3037	209405_s_at	FAM3A	0.15265	0.24631	-0.09366	0.77
3038	209406_at	BAG2	0.58923	0.5005	0.08873	0.006
3039	209407_s_at	DEAF1	0.34175	0.45762	-0.11587	0.883
3040	209408_at	KIF2C	0.7581	0.6065	0.1516	<0.001
3041	209411_s_at	GGA3	0.77877	0.8232	-0.04443	0.877
3042	209413_at	B4GALT2	0.37393	0.38234	-0.00841	0.526
3043	209417_s_at	IFI35	0.82349	0.71847	0.10502	0.021
3044	209421_at	MSH2	0.73288	0.65048	0.0824	0.14
3045	209427_at	SMTN	0.23061	0.21395	0.01666	0.46
3046	209428_s_at	ZFPL1	0.57463	0.59016	-0.01553	0.569
3047	209430_at	BTAF1	0.40699	0.39061	0.01638	0.463
3048	209432_s_at	CREB3	0.58256	0.41829	0.16427	0.016
3049	209433_s_at	PPAT	0.46649	0.54736	-0.08087	0.754
3050	209435_s_at	ARHGEF2	0.63572	0.56961	0.06611	0.258
3051	209438_at	PHKA2	0.19005	0.33546	-0.14541	0.848
3052	209449_at	LSM2	0.45867	0.6456	-0.18693	0.972
3053	209450_at	OSGEP	0.40685	0.67453	-0.26768	0.983
3054	209451_at	TANK	0.81775	0.78847	0.02928	0.284
3055	209452_s_at	VTI1B	0.52995	0.51441	0.01554	0.442
3056	209453_at	SLC9A1	0.31079	0.32514	-0.01435	0.59
3057	209456_s_at	FBXW11	0.50107	0.61197	-0.1109	0.869
3058	209457_at	DUSP5	0.6321	0.55174	0.08036	0.175
3059	209459_s_at	ABAT	0.61219	0.61266	-0.00047	0.531
3060	209463_s_at	TAF12	0.62086	0.74598	-0.12512	0.954
3061	209464_at	AURKB	0.75341	0.76034	-0.00693	0.554
3062	209467_s_at	MKNK1	0.31654	0.34154	-0.025	0.594
3063	209468_at	LRP5	0.37215	0.27432	0.09783	0.227
3064	209472_at	CCBL2	0.42066	0.53041	-0.10975	0.817
3065	209474_s_at	ENTPD1	0.33594	0.46557	-0.12963	0.793
3066	209475_at	USP15	0.4272	0.43171	-0.00451	0.498
3067	209476_at	TMX1	0.82164	0.90453	-0.08289	0.988
3068	209477_at	EMD	0.45815	0.47181	-0.01366	0.555
3069	209478_at	STRA13	0.5012	0.71384	-0.21264	1
3070	209479_at	CCDC28A	0.34983	0.43417	-0.08434	0.754
3071	209481_at	SNRK	0.28828	0.34339	-0.05511	0.675
3072	209482_at	POP7	0.78639	0.77789	0.0085	0.444
3073	209484_s_at	NSL1	0.41625	0.42393	-0.00768	0.54
3074	209486_at	UTP3	0.23263	0.24527	-0.01264	0.518
3075	209497_s_at	RBM4B	0.21368	0.37527	-0.16159	0.946
3076	209501_at	CDR2	0.44639	0.42634	0.02005	0.419

3077	209503_s_at	PSMC5	0.69791	0.75588	-0.05797	0.77
3078	209507_at	RPA3	0.76921	0.62646	0.14275	0.035
3079	209509_s_at	DPAGT1	0.58055	0.54176	0.03879	0.349
3080	209510_at	RNF139	0.64911	0.67286	-0.02375	0.581
3081	209511_at	POLR2F	0.59359	0.46037	0.13322	0.024
3082	209512_at	HSDL2	0.40914	0.50679	-0.09765	0.815
3083	209514_s_at	RAB27A	0.20777	0.37734	-0.16957	0.92
3084	209516_at	SMYD5	0.40714	0.29911	0.10803	0.266
3085	209517_s_at	ASH2L	0.69686	0.56255	0.13431	0.082
3086	209520_s_at	NCBP1	0.42014	0.47575	-0.05561	0.688
3087	209523_at	TAF2	0.8075	0.81355	-0.00605	0.528
3088	209524_at	HDGFRP3	0.29377	0.47837	-0.1846	0.938
3089	209531_at	GSTZ1	0.55996	0.66394	-0.10398	0.889
3090	209532_at	PLAA	0.51479	0.54059	-0.0258	0.591
3091	209536_s_at	EHD4	0.18935	0.29441	-0.10506	0.83
3092	209537_at	EXTL2	0.42489	0.48558	-0.06069	0.679
3093	209538_at	ZNF32	0.06838	0.27376	-0.20538	0.915
3094	209539_at	ARHGEF6	0.27076	0.48396	-0.2132	0.975
3095	209545_s_at	RIPK2	0.73776	0.75219	-0.01443	0.594
3096	209546_s_at	APOL1	0.37425	0.40094	-0.02669	0.582
3097	209553_at	VPS8	0.55955	0.55002	0.00953	0.468
3098	209556_at	NCDN	0.20923	0.14031	0.06892	0.267
3099	209565_at	RNF113A	0.7552	0.85529	-0.10009	0.953
3100	209566_at	INSIG2	0.57441	0.54043	0.03398	0.329
3101	209567_at	RRS1	0.61055	0.29526	0.31529	<0.001
3102	209568_s_at	RGL1	0.38074	0.57347	-0.19273	0.969
3103	209571_at	CIR	0.42217	0.33805	0.08412	0.221
3104	209575_at	IL10RB	0.61934	0.66772	-0.04838	0.798
3105	209577_at	PCYT2	0.25649	0.035702	0.220788	0.036
3106	209581_at	PLA2G16	0.022112	0.17912	-0.157008	0.894
3107	209583_s_at	CD200	0.45222	0.61928	-0.16706	0.905
3108	209585_s_at	MINPP1	0.49427	0.37167	0.1226	0.121
3109	209593_s_at	TOR1B	0.27162	0.33055	-0.05893	0.688
3110	209595_at	GTF2F2	0.37949	0.31284	0.06665	0.298
3111	209604_s_at	GATA3	0.38979	0.5239	-0.13411	0.889
3112	209605_at	TST	0.49479	0.55244	-0.05765	0.726
3113	209606_at	CYTIP	0.3349	0.59218	-0.25728	0.982
3114	209608_s_at	ACAT2	0.57896	0.6856	-0.10664	0.888
3115	209609_s_at	MRPL9	0.879	0.8474	0.0316	0.219
3116	209615_s_at	PAK1	0.55472	0.53317	0.02155	0.446
3117	209619_at	CD74	0.79026	0.7841	0.00616	0.509
3118	209620_s_at	ABCB7	0.59514	0.51674	0.0784	0.103
3119	209622_at	STK16	0.51787	0.36398	0.15389	0.061
3120	209624_s_at	MCCC2	0.51114	0.40814	0.103	0.164
3121	209625_at	PIGH	0.4556	0.46104	-0.00544	0.548
3122	209627_s_at	OSBPL3	0.457	0.46211	-0.00511	0.521
3123	209628_at	NXT2	0.5463	0.48709	0.05921	0.45
3124	209630_s_at	FBXW2	0.55749	0.51526	0.04223	0.44
3125	209636_at	NFKB2	0.61791	0.55983	0.05808	0.257
3126	209645_s_at	ALDH1B1	0.16742	0.036617	0.130803	0.118
3127	209647_s_at	SOCS5	0.45855	0.4118	0.04675	0.338
3128	209653_at	KPNA4	0.58819	0.5093	0.07889	0.192
3129	209654_at	KIAA0947	0.46654	0.41579	0.05075	0.321
3130	209657_s_at	HSF2	0.31542	0.21804	0.09738	0.149

3131	209659_s_at	CDC16	0.56633	0.61315	-0.04682	0.694
3132	209662_at	CETN3	0.63683	0.71054	-0.07371	0.855
3133	209665_at	CYB561D2	0.18253	0.34196	-0.15943	0.882
3134	209666_s_at	CHUK	0.78054	0.70347	0.07707	0.147
3135	209667_at	CES2	0.52474	0.52607	-0.00133	0.53
3136	209669_s_at	SERBP1	0.71569	0.64138	0.07431	0.073
3137	209670_at	TRAC	0.31127	0.38448	-0.07321	0.766
3138	209674_at	CRY1	0.0050328	0.24116	-0.2361272	0.913
3139	209678_s_at	PRKCI	0.61113	0.68962	-0.07849	0.852
3140	209680_s_at	KIFC1	0.63119	0.59586	0.03533	0.261
3141	209681_at	SLC19A2	0.53627	0.69149	-0.15522	0.945
3142	209682_at	CBLB	0.48192	0.71949	-0.23757	0.994
3143	209694_at	PTS	0.76539	0.8058	-0.04041	0.715
3144	209695_at	PTP4A3	0.31569	0.38242	-0.06673	0.708
3145	209704_at	MTF2	0.69961	0.48786	0.21175	0.001
3146	209707_at	PIGK	0.50022	0.42919	0.07103	0.276
3147	209709_s_at	HMMR	0.73425	0.73913	-0.00488	0.564
3148	209711_at	SLC35D1	0.34698	0.34234	0.00464	0.492
3149	209714_s_at	CDKN3	0.77189	0.79862	-0.02673	0.658
3150	209724_s_at	ZFP161	0.15434	0.15321	0.00113	0.509
3151	209725_at	UTP20	0.62749	0.58996	0.03753	0.301
3152	209726_at	CA11	0.38787	0.42272	-0.03485	0.637
3153	209727_at	GM2A	0.16515	0.10516	0.05999	0.333
3154	209731_at	NTHL1	0.62208	0.46615	0.15593	0.016
3155	209732_at	CLEC2B	0.082926	0.21966	-0.136734	0.864
3156	209739_s_at	PNPLA4	0.0098981	0.17789	-0.1679919	0.885
3157	209748_at	SPAST	0.83057	0.75998	0.07059	0.123
3158	209753_s_at	TMPO	0.67666	0.61632	0.06034	0.152
3159	209759_s_at	DCI	0.48704	0.45593	0.03111	0.396
3160	209760_at	KIAA0922	0.554	0.54595	0.00805	0.467
3161	209761_s_at	SP110	0.71364	0.77277	-0.05913	0.781
3162	209764_at	MGAT3	0.10134	0.20659	-0.10525	0.796
3163	209770_at	BTN3A1	0.080491	0.01933	0.061161	0.297
3164	209778_at	TRIP11	0.37364	0.28641	0.08723	0.262
3165	209780_at	PHTF2	0.79269	0.61576	0.17693	<0.001
3166	209786_at	HMGN4	0.4782	0.51286	-0.03466	0.63
3167	209788_s_at	ERAP1	0.00049738	0.13891	-0.13841262	0.91
3168	209790_s_at	CASP6	0.32116	0.42846	-0.1073	0.844
3169	209795_at	CD69	0.56654	0.51351	0.05303	0.295
3170	209796_s_at	CNPY2	0.73222	0.87174	-0.13952	0.956
3171	209799_at	PRKAA1	0.025454	0.20484	-0.179386	0.973
3172	209805_at	PMS2	0.1297	0.30574	-0.17604	0.919
3173	209806_at	HIST1H2BK	0.33846	0.52234	-0.18388	0.982
3174	209814_at	ZNF330	0.54982	0.36733	0.18249	0.012
3175	209820_s_at	TBL3	0.7344	0.36835	0.36605	<0.001
3176	209822_s_at	VLDLR	0.12766	0.15173	-0.02407	0.636
3177	209825_s_at	UCK2	0.83521	0.74443	0.09078	0.029
3178	209827_s_at	IL16	0.55892	0.52288	0.03604	0.314
3179	209829_at	FAM65B	0.58248	0.57742	0.00506	0.479
3180	209832_s_at	CDT1	0.73396	0.66656	0.0674	0.091
3181	209833_at	CRADD	0.40915	0.35517	0.05398	0.327
3182	209837_at	AP4M1	0.15511	0.15555	-0.00044	0.52
3183	209838_at	COPS2	0.075902	0.18851	-0.112608	0.81
3184	209845_at	MKRN1	0.3693	0.30512	0.06418	0.366

3185	209849_s_at	RAD51C	0.69692	0.69492	0.002	0.51
3186	209853_s_at	PSME3	0.82663	0.65236	0.17427	0.019
3187	209861_s_at	METAP2	0.82926	0.81408	0.01518	0.388
3188	209863_s_at	TP63	0.25065	0.39769	-0.14704	0.864
3189	209864_at	FRAT2	0.4879	0.45019	0.03771	0.389
3190	209865_at	SLC35A3	0.75203	0.81778	-0.06575	0.938
3191	209879_at	SELPLG	0.50695	0.52292	-0.01597	0.595
3192	209882_at	RIT1	0.53669	0.45052	0.08617	0.197
3193	209883_at	GLT25D2	0.27061	0.40525	-0.13464	0.828
3194	209891_at	SPC25	0.76427	0.77485	-0.01058	0.663
3195	209893_s_at	FUT4	0.69803	0.64774	0.05029	0.331
3196	209894_at	LEPR	0.095107	0.34618	-0.251073	0.972
3197	209899_s_at	PUF60	0.72174	0.46101	0.26073	<0.001
3198	209903_s_at	ATR	0.53666	0.32366	0.213	0.013
3199	209910_at	SLC25A16	0.15436	0.2542	-0.09984	0.86
3200	209912_s_at	KIAA0415	0.4031	0.52029	-0.11719	0.861
3201	209916_at	DHTKD1	0.56563	0.64512	-0.07949	0.837
3202	209925_at	OCLN	0.61678	0.68138	-0.0646	0.753
3203	209926_at	LOC729991	0.26588	0.13406	0.13182	0.102
3204	209927_s_at	C1orf77	0.65829	0.53088	0.12741	0.057
3205	209928_s_at	MSC	0.5919	0.6431	-0.0512	0.734
3206	209929_s_at	IKBKG	0.4373	0.26456	0.17274	0.053
3207	209932_s_at	DUT	0.79724	0.79592	0.00132	0.521
3208	209933_s_at	CD300A	0.40426	0.4714	-0.06714	0.693
3209	209940_at	PARP3	0.19101	0.26651	-0.0755	0.74
3210	209941_at	RIPK1	0.18703	0.17246	0.01457	0.43
3211	209943_at	FBXL4	0.4596	0.43018	0.02942	0.422
3212	209945_s_at	GSK3B	0.57972	0.63597	-0.05625	0.73
3213	209949_at	NCF2	0.79926	0.8421	-0.04284	0.828
3214	209953_s_at	CDC37	0.59706	0.64393	-0.04687	0.723
3215	209962_at	EPOR	0.45561	0.18282	0.27279	0.036
3216	209965_s_at	RAD51L3	0.15593	0.14	0.01593	0.465
3217	209967_s_at	CREM	0.50341	0.52472	-0.02131	0.562
3218	209969_s_at	STAT1	0.66511	0.60486	0.06025	0.322
3219	209972_s_at	JTV1	0.80727	0.79912	0.00815	0.407
3220	209973_at	NFKBIL1	0.55666	0.23374	0.32292	<0.001
3221	209974_s_at	BUB3	0.83854	0.81173	0.02681	0.224
3222	209989_at	ZNF268	0.40797	0.38698	0.02099	0.465
3223	209994_s_at	ABCB1	0.49133	0.58172	-0.09039	0.814
3224	210006_at	ABHD14A	0.51338	0.61758	-0.1042	0.876
3225	210010_s_at	SLC25A1	0.29346	0.4076	-0.11414	0.89
3226	210022_at	PCGF1	0.59094	0.51597	0.07497	0.237
3227	210024_s_at	UBE2E3	0.22709	0.34304	-0.11595	0.858
3228	210027_s_at	APEX1	0.68196	0.75588	-0.07392	0.937
3229	210028_s_at	ORC3L	0.31211	0.37028	-0.05817	0.662
3230	210041_s_at	PGM3	0.53651	0.50619	0.03032	0.327
3231	210045_at	IDH2	0.28576	0.41333	-0.12757	0.829
3232	210048_at	NAPG	0.39657	0.58742	-0.19085	0.96
3233	210052_s_at	TPX2	0.8933	0.84845	0.04485	0.104
3234	210053_at	TAF5	0.79285	0.71437	0.07848	0.062
3235	210054_at	HAUS3	0.33331	0.49833	-0.16502	0.914
3236	210057_at	SMG1	0.89959	0.90336	-0.00377	0.567
3237	210058_at	MAPK13	0.62545	0.76577	-0.14032	0.933
3238	210070_s_at	CHKB	0.47288	0.55867	-0.08579	0.803

3239	210075_at	MARCH2	0.44373	0.51909	-0.07536	0.791
3240	210092_at	MAGOH	0.85826	0.85743	0.00083	0.52
3241	210097_s_at	NOL7	0.75733	0.7489	0.00843	0.447
3242	210105_s_at	FYN	0.10438	0.25928	-0.1549	0.916
3243	210109_at	C7orf54	0.71247	0.62415	0.08832	0.511
3244	210114_at	INVS	0.13843	0.23612	-0.09769	0.789
3245	210115_at	RPL39L	0.24908	0.43004	-0.18096	0.925
3246	210117_at	SPAG1	0.54464	0.65309	-0.10845	0.899
3247	210125_s_at	BANF1	0.60543	0.51098	0.09445	0.167
3248	210128_s_at	LTB4R	0.29906	0.18863	0.11043	0.178
3249	210130_s_at	TM7SF2	0.51967	0.49503	0.02464	0.443
3250	210137_s_at	DCTD	0.78751	0.76027	0.02724	0.289
3251	210138_at	RGS20	0.30745	0.55333	-0.24588	0.975
3252	210144_at	TBC1D22A	0.28388	0.40968	-0.1258	0.819
3253	210145_at	PLA2G4A	0.10972	0.32882	-0.2191	0.96
3254	210149_s_at	ATP5H	0.87753	0.84011	0.03742	0.152
3255	210151_s_at	DYRK3	0.2386	0.23326	0.00534	0.497
3256	210152_at	LILRB4	0.36259	0.27612	0.08647	0.177
3257	210154_at	ME2	0.72196	0.66423	0.05773	0.185
3258	210160_at	PAFAH1B2	0.21925	0.16901	0.05024	0.344
3259	210169_at	SEC14L5	0.18714	0.31523	-0.12809	0.823
3260	210172_at	SF1	0.77599	0.83899	-0.063	0.86
3261	210176_at	TLR1	0.42127	0.46933	-0.04806	0.645
3262	210188_at	GABPA	0.59692	0.33253	0.26439	0.003
3263	210200_at	WWP2	0.27166	0.27157	9E-05	0.553
3264	210202_s_at	BIN1	0.18675	0.31504	-0.12829	0.877
3265	210205_at	B3GALT4	0.32771	0.24022	0.08749	0.231
3266	210206_s_at	DDX11	0.73707	0.46775	0.26932	0.001
3267	210213_s_at	EIF6	0.85875	0.80052	0.05823	0.032
3268	210214_s_at	BMPR2	0.6881	0.82629	-0.13819	0.96
3269	210219_at	SP100	0.65989	0.58951	0.07038	0.266
3270	210220_at	FZD2	0.36703	0.35428	0.01275	0.462
3271	210235_s_at	PPFIA1	0.44648	0.62585	-0.17937	0.978
3272	210241_s_at	TP53TG1	0.55416	0.59719	-0.04303	0.619
3273	210243_s_at	B4GALT3	0.3233	0.29685	0.02645	0.368
3274	210247_at	SYN2	0.21173	0.16641	0.04532	0.352
3275	210253_at	HTATIP2	0.29235	0.18048	0.11187	0.201
3276	210258_at	RGS13	0.56595	0.61024	-0.04429	0.755
3277	210260_s_at	TNFAIP8	0.35023	0.55484	-0.20461	0.974
3278	210269_s_at	SFRS17A	0.4379	0.3094	0.1285	0.123
3279	210275_s_at	ZFAND5	0.47192	0.4062	0.06572	0.259
3280	210276_s_at	TRIOBP	0.50608	0.52957	-0.02349	0.591
3281	210278_s_at	AP4S1	0.34211	0.36945	-0.02734	0.556
3282	210279_at	GPR18	0.22191	0.41204	-0.19013	0.946
3283	210280_at	MPZ	0.172	0.20565	-0.03365	0.592
3284	210281_s_at	ZMYM2	0.83979	0.82186	0.01793	0.347
3285	210284_s_at	MAP3K7IP2	0.81443	0.72134	0.09309	0.048
3286	210296_s_at	PXMP3	0.73977	0.69146	0.04831	0.22
3287	210312_s_at	IFT20	0.68658	0.71387	-0.02729	0.725
3288	210338_s_at	HSPA8	0.77635	0.39051	0.38584	<0.001
3289	210346_s_at	CLK4	0.71408	0.68812	0.02596	0.398
3290	210349_at	CAMK4	0.24355	0.51004	-0.26649	0.996
3291	210371_s_at	RBBP4	0.66817	0.62903	0.03914	0.309
3292	210377_at	ACSM3	0.69643	0.80533	-0.1089	0.968

3293	210378_s_at	SSNA1	0.30048	0.33214	-0.03166	0.599
3294	210379_s_at	TLK1	0.41395	0.61612	-0.20217	0.969
3295	210386_s_at	MTX1	0.65129	0.78853	-0.13724	0.956
3296	210396_s_at	BOLA2	0.23088	0.37042	-0.13954	0.867
3297	210406_s_at	RAB6A	0.75148	0.76828	-0.0168	0.715
3298	210415_s_at	ODF2	0.049047	0.11707	-0.068023	0.727
3299	210416_s_at	CHEK2	0.28318	0.45363	-0.17045	0.936
3300	210417_s_at	PI4KB	0.36161	0.48345	-0.12184	0.887
3301	210418_s_at	IDH3B	0.82693	0.76945	0.05748	0.126
3302	210428_s_at	HGS	0.48368	0.69953	-0.21585	0.992
3303	210448_s_at	P2RX5	0.37414	0.46199	-0.08785	0.755
3304	210450_at	LOC90925	0.44705	0.40986	0.03719	0.364
3305	210474_s_at	CDC2L1	0.26869	0.27694	-0.00825	0.519
3306	210479_s_at	RORA	0.532	0.54537	-0.01337	0.574
3307	210480_s_at	MYO6	0.62235	0.67449	-0.05214	0.726
3308	210502_s_at	PPIE	0.79152	0.80361	-0.01209	0.608
3309	210512_s_at	VEGFA	0.29097	0.093558	0.197412	0.022
3310	210528_at	MR1	0.63091	0.62545	0.00546	0.472
3311	210531_at	NR2C1	0.13234	0.10417	0.02817	0.407
3312	210538_s_at	BIRC3	0.48883	0.47563	0.0132	0.444
3313	210543_s_at	PRKDC	0.74943	0.75755	-0.00812	0.556
3314	210556_at	NFATC3	0.64073	0.69887	-0.05814	0.704
3315	210559_s_at	CDC2	0.82407	0.84571	-0.02164	0.774
3316	210561_s_at	WSB1	0.6107	0.58918	0.02152	0.406
3317	210573_s_at	POLR3C	0.84538	0.51604	0.32934	<0.001
3318	210574_s_at	NUDC	0.80912	0.77265	0.03647	0.166
3319	210587_at	INHBE	0.11996	0.2304	-0.11044	0.851
3320	210589_s_at	GBAP	0.1805	0.27454	-0.09404	0.703
3321	210596_at	LOC100129513	0.75611	0.85061	-0.0945	0.883
3322	210609_s_at	TP53I3	0.601	0.62678	-0.02578	0.602
3323	210620_s_at	GTF3C2	0.34443	0.31559	0.02884	0.416
3324	210625_s_at	AKAP1	0.75282	0.54111	0.21171	<0.001
3325	210627_s_at	MOGS	0.39048	0.26059	0.12989	0.025
3326	210631_at	NF1	0.21084	0.23097	-0.02013	0.589
3327	210635_s_at	KLHL20	0.65432	0.42188	0.23244	0.019
3328	210638_s_at	FBXO9	0.31276	0.44662	-0.13386	0.883
3329	210639_s_at	ATG5	0.76077	0.69647	0.0643	0.321
3330	210643_at	TNFSF11	0.53299	0.48166	0.05133	0.29
3331	210649_s_at	ARID1A	0.80903	0.75936	0.04967	0.214
3332	210656_at	EED	0.16137	0.38792	-0.22655	0.954
3333	210685_s_at	UBE4B	0.36309	0.42201	-0.05892	0.684
3334	210701_at	CFDP1	0.44819	0.4773	-0.02911	0.632
3335	210705_s_at	TRIM5	0.56518	0.59997	-0.03479	0.674
3336	210715_s_at	SPINT2	0.32579	0.45369	-0.1279	0.796
3337	210718_s_at	ARL17P1	0.20993	0.46229	-0.25236	0.967
3338	210720_s_at	NECAB3	0.332	0.38514	-0.05314	0.633
3339	210732_s_at	LGALS8	0.51545	0.55951	-0.04406	0.642
3340	210733_at	TRAM1	0.40232	0.54238	-0.14006	0.939
3341	210740_s_at	ITPK1	0.36491	0.54238	-0.17747	0.921
3342	210752_s_at	MLX	0.32615	0.31978	0.00637	0.49
3343	210758_at	PSIP1	0.48141	0.452	0.02941	0.376
3344	210759_s_at	PSMA1	0.81648	0.74841	0.06807	0.142
3345	210771_at	PPARA	0.21772	0.29475	-0.07703	0.782
3346	210774_s_at	NCOA4	0.61051	0.67532	-0.06481	0.77

3347	210785_s_at	C1orf38	0.75009	0.6939	0.05619	0.251
3348	210786_s_at	FLI1	0.72804	0.69452	0.03352	0.355
3349	210793_s_at	NUP98	0.50569	0.42181	0.08388	0.184
3350	210797_s_at	OASL	0.56535	0.30252	0.26283	0.001
3351	210807_s_at	SLC16A7	0.28224	0.55709	-0.27485	0.97
3352	210811_s_at	DDX49	0.52715	0.34248	0.18467	0.02
3353	210813_s_at	XRCC4	0.11294	0.11533	-0.00239	0.503
3354	210817_s_at	CALCOCO2	0.54586	0.56246	-0.0166	0.57
3355	210822_at	RPL13P5	0.49979	0.37689	0.1229	0.114
3356	210830_s_at	PON2	0.73908	0.80966	-0.07058	0.887
3357	210868_s_at	ELOVL6	0.6399	0.62155	0.01835	0.449
3358	210878_s_at	KDM3B	0.51469	0.45725	0.05744	0.263
3359	210889_s_at	FCGR2B	0.14656	0.3956	-0.24904	0.958
3360	210892_s_at	GTF2I	0.47881	0.46002	0.01879	0.426
3361	210895_s_at	CD86	0.70135	0.72739	-0.02604	0.606
3362	210907_s_at	PDCD10	0.69455	0.68533	0.00922	0.46
3363	210908_s_at	PFDN5	0.8635	0.86504	-0.00154	0.554
3364	210910_s_at	POMZP3	0.022786	0.11414	-0.091354	0.844
3365	210926_at	ACTBL3	0.40804	0.49119	-0.08315	0.807
3366	210942_s_at	ST3GAL6	0.61542	0.65914	-0.04372	0.706
3367	210943_s_at	LYST	0.70601	0.69328	0.01273	0.441
3368	210944_s_at	CAPN3	0.38603	0.41674	-0.03071	0.604
3369	210946_at	PPAP2A	0.26339	0.33289	-0.0695	0.715
3370	210947_s_at	MSH3	0.13308	0.35698	-0.2239	0.967
3371	210949_s_at	EIF3C	0.29494	0.46358	-0.16864	0.931
3372	210959_s_at	SRD5A1	0.64416	0.45617	0.18799	0.053
3373	210968_s_at	RTN4	0.69764	0.64788	0.04976	0.22
3374	210971_s_at	ARNTL	0.66265	0.65372	0.00893	0.479
3375	210976_s_at	PFKM	0.55481	0.41871	0.1361	0.044
3376	210978_s_at	TAGLN2	0.5204	0.72479	-0.20439	0.991
3377	210983_s_at	MCM7	0.79523	0.75005	0.04518	0.021
3378	210996_s_at	YWHAE	0.86895	0.81738	0.05157	0.207
3379	211009_s_at	ZNF271	0.3219	0.38705	-0.06515	0.704
3380	211010_s_at	NCR3	0.34842	0.50384	-0.15542	0.928
3381	211012_s_at	LOC161527	0.40266	0.42384	-0.02118	0.576
3382	211015_s_at	HSPA4	0.85856	0.68721	0.17135	0.003
3383	211026_s_at	MGLL	0.58654	0.71316	-0.12662	0.895
3384	211028_s_at	KHK	0.51212	0.22107	0.29105	0.008
3385	211031_s_at	CLIP2	0.67363	0.51686	0.15677	0.054
3386	211033_s_at	PEX7	0.60662	0.59942	0.0072	0.494
3387	211034_s_at	C12orf51	0.66378	0.6796	-0.01582	0.617
3388	211038_s_at	CROCCL1	0.11052	0.34136	-0.23084	0.944
3389	211043_s_at	CLTB	0.6383	0.7171	-0.0788	0.86
3390	211048_s_at	PDIA4	0.66647	0.50602	0.16045	0.033
3391	211052_s_at	TBCD	0.18464	0.37721	-0.19257	0.938
3392	211059_s_at	GOLGA2	0.54023	0.41703	0.1232	0.119
3393	211064_at	ZNF493	0.13502	0.1982	-0.06318	0.719
3394	211071_s_at	MLLT11	0.33291	0.4736	-0.14069	0.877
3395	211074_at	FOLR1	0.41218	0.50558	-0.0934	0.833
3396	211075_s_at	CD47	0.344	0.48354	-0.13954	0.868
3397	211089_s_at	NEK3	0.61018	0.71445	-0.10427	0.858
3398	211113_s_at	ABCG1	0.069124	0.24363	-0.174506	0.945
3399	211136_s_at	CLPTM1	0.82069	0.84139	-0.0207	0.696
3400	211141_s_at	CNOT3	0.38028	0.39892	-0.01864	0.608

3401	211168_s_at	UPF1	0.27001	0.24293	0.02708	0.402
3402	211178_s_at	PSTPIP1	0.29449	0.39138	-0.09689	0.824
3403	211212_s_at	ORC5L	0.83616	0.78353	0.05263	0.125
3404	211250_s_at	SH3BP2	0.68348	0.48871	0.19477	0.014
3405	211275_s_at	GYG1	0.1503	0.46533	-0.31503	0.98
3406	211284_s_at	GRN	0.58911	0.6272	-0.03809	0.666
3407	211285_s_at	UBE3A	0.40218	0.55153	-0.14935	0.901
3408	211297_s_at	CDK7	0.55933	0.44685	0.11248	0.166
3409	211310_at	EZH1	0.79881	0.59513	0.20368	0.031
3410	211317_s_at	CFLAR	0.80314	0.80942	-0.00628	0.581
3411	211330_s_at	HFE	0.202	0.26198	-0.05998	0.706
3412	211339_s_at	ITK	0.10176	0.25533	-0.15357	0.927
3413	211352_s_at	NCOA3	0.94016	0.9132	0.02696	0.145
3414	211358_s_at	CIZ1	0.29837	0.38043	-0.08206	0.765
3415	211364_at	MTAP	0.046928	0.1739	-0.126972	0.84
3416	211368_s_at	CASP1	0.26763	0.45478	-0.18715	0.934
3417	211373_s_at	PSEN2	0.16863	0.27924	-0.11061	0.824
3418	211391_s_at	PATZ1	0.4611	0.49234	-0.03124	0.64
3419	211404_s_at	APLP2	0.51924	0.5855	-0.06626	0.745
3420	211406_at	IER3IP1	0.82909	0.81401	0.01508	0.39
3421	211450_s_at	MSH6	0.85992	0.72549	0.13443	<0.001
3422	211475_s_at	BAG1	0.63203	0.65119	-0.01916	0.6
3423	211501_s_at	EIF3B	0.81459	0.74087	0.07372	0.073
3424	211502_s_at	PFTK1	0.37306	0.5512	-0.17814	0.938
3425	211505_s_at	STAU1	0.55678	0.69642	-0.13964	0.943
3426	211512_s_at	OGFR	0.29272	0.26185	0.03087	0.41
3427	211538_s_at	HSPA2	0.17562	0.054518	0.121102	0.194
3428	211543_s_at	GRK6	0.67429	0.74135	-0.06706	0.885
3429	211558_s_at	DHPS	0.59783	0.68801	-0.09018	0.893
3430	211563_s_at	C19orf2	0.52478	0.39079	0.13399	0.099
3431	211569_s_at	HADH	0.64877	0.61401	0.03476	0.327
3432	211572_s_at	SLC23A2	0.50819	0.5712	-0.06301	0.782
3433	211574_s_at	CD46	0.59857	0.51031	0.08826	0.283
3434	211593_s_at	MAST2	0.67068	0.43563	0.23505	<0.001
3435	211596_s_at	LRIG1	0.18227	0.36581	-0.18354	0.918
3436	211600_at	PTPRO	0.74143	0.81519	-0.07376	0.87
3437	211612_s_at	IL13RA1	0.4466	0.56178	-0.11518	0.759
3438	211623_s_at	FBL	0.79764	0.78714	0.0105	0.429
3439	211662_s_at	VDAC2	0.80985	0.78356	0.02629	0.323
3440	211665_s_at	SOS2	0.6313	0.70457	-0.07327	0.809
3441	211671_s_at	NR3C1	0.61459	0.54574	0.06885	0.258
3442	211672_s_at	ARPC4	0.68509	0.72713	-0.04204	0.676
3443	211676_s_at	IFNGR1	0.69079	0.66384	0.02695	0.355
3444	211678_s_at	RNF114	0.72578	0.74791	-0.02213	0.652
3445	211684_s_at	DYNC1I2	0.47616	0.47798	-0.00182	0.564
3446	211685_s_at	NCALD	0.58658	0.62869	-0.04211	0.656
3447	211686_s_at	MAK16	0.80662	0.71994	0.08668	0.029
3448	211692_s_at	BBC3	0.32682	0.42166	-0.09484	0.801
3449	211702_s_at	USP32	0.55272	0.45889	0.09383	0.204
3450	211704_s_at	SPIN2A	0.35955	0.1874	0.17215	0.088
3451	211707_s_at	IQCB1	0.36388	0.5268	-0.16292	0.942
3452	211708_s_at	SCD	0.67595	0.61156	0.06439	0.209
3453	211715_s_at	BDH1	0.61534	0.63295	-0.01761	0.574
3454	211717_at	ANKRD40	0.40245	0.23358	0.16887	0.098

3455	211721_s_at	ZNF551	0.75607	0.72255	0.03352	0.279
3456	211725_s_at	BID	0.55537	0.51236	0.04301	0.336
3457	211727_s_at	COX11	0.55457	0.63606	-0.08149	0.852
3458	211742_s_at	EVI2B	0.58289	0.71675	-0.13386	0.885
3459	211749_s_at	VAMP3	0.59431	0.58796	0.00635	0.507
3460	211752_s_at	NDUFS7	0.55409	0.64742	-0.09333	0.837
3461	211753_s_at	RLN1	0.32829	0.24124	0.08705	0.227
3462	211754_s_at	SLC25A17	0.4292	0.55411	-0.12491	0.905
3463	211755_s_at	ATP5F1	0.72644	0.70542	0.02102	0.334
3464	211763_s_at	UBE2B	0.64855	0.60047	0.04808	0.177
3465	211764_s_at	UBE2D1	0.60383	0.68062	-0.07679	0.805
3466	211767_at	GINS4	0.69281	0.58463	0.10818	0.022
3467	211773_s_at	ZKSCAN3	0.099379	0.1376	-0.038221	0.636
3468	211774_s_at	MMACHC	0.2045	0.17444	0.03006	0.41
3469	211783_s_at	MTA1	0.72425	0.62813	0.09612	0.119
3470	211784_s_at	SFRS1	0.89809	0.69373	0.20436	<0.001
3471	211787_s_at	EIF4A1	0.90749	0.85877	0.04872	0.015
3472	211792_s_at	CDKN2C	0.56581	0.61269	-0.04688	0.663
3473	211796_s_at	TRBC1	0.48127	0.51723	-0.03596	0.666
3474	211810_s_at	GALC	0.025419	0.036589	-0.01117	0.581
3475	211812_s_at	B3GALNT1	0.33009	0.44533	-0.11524	0.846
3476	211825_s_at	EWSR1	0.69316	0.55926	0.1339	0.106
3477	211828_s_at	TNIK	0.45487	0.52432	-0.06945	0.738
3478	211833_s_at	BAX	0.85713	0.85859	-0.00146	0.535
3479	211855_s_at	SLC25A14	0.49036	0.48827	0.00209	0.488
3480	211913_s_at	MERTK	0.22366	0.20479	0.01887	0.427
3481	211926_s_at	MYH9	0.51598	0.62208	-0.1061	0.885
3482	211928_at	DYNC1H1	0.62623	0.60931	0.01692	0.445
3483	211935_at	ARL6IP1	0.33585	0.44422	-0.10837	0.83
3484	211936_at	HSPA5	0.61874	0.34533	0.27341	0.003
3485	211938_at	EIF4B	0.75276	0.7792	-0.02644	0.624
3486	211946_s_at	BAT2D1	0.72358	0.68801	0.03557	0.357
3487	211950_at	UBR4	0.63154	0.59958	0.03196	0.377
3488	211955_at	IPO5	0.66358	0.76894	-0.10536	0.969
3489	211956_s_at	EIF1	0.90959	0.90174	0.00785	0.452
3490	211960_s_at	RAB7A	0.26611	0.37236	-0.10625	0.924
3491	211962_s_at	ZFP36L1	0.54646	0.60098	-0.05452	0.673
3492	211963_s_at	ARPC5	0.22057	0.35048	-0.12991	0.875
3493	211967_at	TMEM123	0.76211	0.69879	0.06332	0.261
3494	211971_s_at	LRPPRC	0.71085	0.70485	0.006	0.492
3495	211975_at	ARFGAP2	0.678	0.65669	0.02131	0.395
3496	211985_s_at	CALM1	0.52954	0.28861	0.24093	0.009
3497	211987_at	TOP2B	0.55703	0.5945	-0.03747	0.631
3498	211989_at	SMARCE1	0.70874	0.66394	0.0448	0.278
3499	211990_at	HLA-DPA1	0.6537	0.59159	0.06211	0.275
3500	211994_at	WNK1	0.42711	0.5556	-0.12849	0.917
3501	211998_at	H3F3B	0.36366	0.58025	-0.21659	0.983
3502	212005_at	C1orf144	0.61285	0.61169	0.00116	0.504
3503	212007_at	UBXN4	0.87937	0.88779	-0.00842	0.641
3504	212009_s_at	STIP1	0.79427	0.61016	0.18411	<0.001
3505	212016_s_at	PTBP1	0.86036	0.83967	0.02069	0.318
3506	212017_at	FAM168B	0.5053	0.38985	0.11545	0.116
3507	212019_at	RSL1D1	0.58092	0.59351	-0.01259	0.574
3508	212021_s_at	MKI67	0.65944	0.62153	0.03791	0.326

3509	212025_s_at	FLII	0.17228	0.27515	-0.10287	0.748
3510	212027_at	RBM25	0.70697	0.83481	-0.12784	0.982
3511	212032_s_at	PTOV1	0.4805	0.33179	0.14871	0.057
3512	212036_s_at	PNN	0.57738	0.70709	-0.12971	0.968
3513	212038_s_at	VDAC1	0.7756	0.6113	0.1643	0.003
3514	212040_at	TGOLN2	0.79915	0.76709	0.03206	0.313
3515	212041_at	ATP6V0D1	0.64591	0.6746	-0.02869	0.687
3516	212044_s_at	RPL27A	0.79765	0.82859	-0.03094	0.767
3517	212047_s_at	RNF167	0.23602	0.23621	-0.00019	0.495
3518	212048_s_at	YARS	0.73352	0.62822	0.1053	0.152
3519	212050_at	WIPF2	0.59627	0.66176	-0.06549	0.805
3520	212052_s_at	TBC1D9B	0.28654	0.26679	0.01975	0.45
3521	212053_at	PDXDC1	0.53326	0.46068	0.07258	0.199
3522	212055_at	C18orf10	0.39307	0.37764	0.01543	0.42
3523	212057_at	KIAA0182	0.59836	0.61222	-0.01386	0.575
3524	212058_at	SR140	0.73716	0.60564	0.13152	0.04
3525	212059_s_at	TRPC4AP	0.39258	0.18415	0.20843	0.01
3526	212066_s_at	USP34	0.37299	0.4309	-0.05791	0.719
3527	212069_s_at	BAT2L	0.26655	0.31414	-0.04759	0.64
3528	212072_s_at	CSNK2A1	0.60307	0.60454	-0.00147	0.532
3529	212078_s_at	MLL	0.91768	0.94716	-0.02948	0.962
3530	212082_s_at	MYL6	0.71072	0.70532	0.0054	0.49
3531	212083_at	TEX261	0.54557	0.57799	-0.03242	0.642
3532	212087_s_at	ERAL1	0.75354	0.7225	0.03104	0.32
3533	212088_at	PMPCA	0.6385	0.58916	0.04934	0.291
3534	212090_at	GRINA	0.24154	0.28441	-0.04287	0.647
3535	212092_at	PEG10	0.77528	0.74648	0.0288	0.329
3536	212098_at	LOC151162	0.77492	0.77538	-0.00046	0.528
3537	212099_at	RHOB	0.13907	0.20828	-0.06921	0.753
3538	212100_s_at	POLDIP3	0.44046	0.28309	0.15737	0.053
3539	212101_at	KPNA6	0.67029	0.6205	0.04979	0.329
3540	212106_at	FAF2	0.90877	0.79019	0.11858	0.005
3541	212110_at	SLC39A14	0.60264	0.37628	0.22636	0.013
3542	212112_s_at	STX12	0.19259	0.2001	-0.00751	0.527
3543	212115_at	HN1L	0.62174	0.58173	0.04001	0.315
3544	212118_at	TRIM27	0.24575	0.24799	-0.00224	0.514
3545	212119_at	RHOQ	0.77961	0.81056	-0.03095	0.703
3546	212121_at	TCTN3	0.56286	0.47918	0.08368	0.244
3547	212124_at	ZMIZ1	0.36046	0.48741	-0.12695	0.843
3548	212126_at	CBX5	0.74954	0.58654	0.163	0.003
3549	212127_at	RANGAP1	0.12509	0.12236	0.00273	0.459
3550	212129_at	NIPA2	0.61522	0.66615	-0.05093	0.698
3551	212131_at	LSM14A	0.82891	0.78972	0.03919	0.219
3552	212140_at	PDS5A	0.61345	0.47643	0.13702	0.065
3553	212141_at	MCM4	0.83193	0.77514	0.05679	0.016
3554	212144_at	UNC84B	0.32895	0.2948	0.03415	0.364
3555	212145_at	MRPS27	0.58119	0.55137	0.02982	0.372
3556	212146_at	PLEKHM2	0.33119	0.29382	0.03737	0.377
3557	212150_at	EFR3A	0.40413	0.38778	0.01635	0.423
3558	212153_at	POGZ	0.33135	0.55485	-0.2235	0.986
3559	212155_at	RNF187	0.56512	0.44636	0.11876	0.009
3560	212156_at	VPS39	0.3071	0.49692	-0.18982	0.961
3561	212160_at	XPOT	0.76729	0.46168	0.30561	<0.001
3562	212161_at	AP2A2	0.11098	0.1705	-0.05952	0.732

3563	212162_at	KIDINS220	0.78359	0.86362	-0.08003	0.958
3564	212165_at	TMEM183A	0.55661	0.61944	-0.06283	0.749
3565	212166_at	XPO7	0.343	0.25169	0.09131	0.229
3566	212169_at	FKBP9	0.15192	0.35671	-0.20479	0.958
3567	212170_at	RBM12	0.42866	0.15827	0.27039	<0.001
3568	212177_at	SFRS18	0.8549	0.8994	-0.0445	0.908
3569	212186_at	ACACA	0.50344	0.395	0.10844	0.016
3570	212188_at	KCTD12	0.67164	0.62348	0.04816	0.303
3571	212189_s_at	COG4	0.29473	0.32372	-0.02899	0.578
3572	212190_at	SERPINE2	0.066649	0.20698	-0.140331	0.906
3573	212193_s_at	LARP1	0.90861	0.88143	0.02718	0.162
3574	212194_s_at	TM9SF4	0.50065	0.57939	-0.07874	0.794
3575	212199_at	MRFAP1L1	0.4716	0.56001	-0.08841	0.723
3576	212200_at	ANKLE2	0.14719	0.21849	-0.0713	0.69
3577	212202_s_at	TMEM87A	0.50209	0.58112	-0.07903	0.866
3578	212208_at	MED13L	0.65741	0.78552	-0.12811	0.926
3579	212211_at	ANKRD17	0.69246	0.7305	-0.03804	0.72
3580	212217_at	PREPL	0.67469	0.593	0.08169	0.133
3581	212218_s_at	FASN	0.69837	0.33378	0.36459	<0.001
3582	212219_at	PSME4	0.31738	0.47966	-0.16228	0.915
3583	212228_s_at	COQ9	0.65365	0.67022	-0.01657	0.635
3584	212231_at	FBXO21	0.37713	0.26994	0.10719	0.109
3585	212232_at	FNBP4	0.56192	0.61505	-0.05313	0.769
3586	212233_at	MAP1B	0.0074592	0.16325	-0.1557908	0.941
3587	212238_at	ASXL1	0.61478	0.65056	-0.03578	0.644
3588	212242_at	TUBA4A	0.81245	0.72421	0.08824	0.133
3589	212244_at	GCOM1	0.44211	0.68797	-0.24586	0.989
3590	212245_at	MCFD2	0.43358	0.35333	0.08025	0.196
3591	212247_at	NUP205	0.63565	0.55866	0.07699	0.058
3592	212249_at	PIK3R1	0.82907	0.8548	-0.02573	0.714
3593	212251_at	MTDH	0.73203	0.82152	-0.08949	0.962
3594	212255_s_at	ATP2C1	0.29084	0.37263	-0.08179	0.736
3595	212260_at	GIGYF2	0.58375	0.39665	0.1871	0.007
3596	212263_at	QKI	0.49596	0.37387	0.12209	0.125
3597	212266_s_at	SFRS5	0.71208	0.75278	-0.0407	0.779
3598	212268_at	SERPINB1	0.57708	0.59748	-0.0204	0.576
3599	212271_at	MAPK1	0.42262	0.47058	-0.04796	0.742
3600	212274_at	LPIN1	0.59537	0.65981	-0.06444	0.73
3601	212277_at	MTMR4	0.62957	0.5443	0.08527	0.197
3602	212281_s_at	TMEM97	0.58911	0.46787	0.12124	<0.001
3603	212287_at	SUZ12	0.80093	0.71588	0.08505	0.037
3604	212293_at	HIPK1	0.097212	0.0032096	0.0940024	0.163
3605	212296_at	PSMD14	0.89079	0.80157	0.08922	0.005
3606	212297_at	ATP13A3	0.85065	0.80477	0.04588	0.07
3607	212300_at	TXLNA	0.60576	0.3976	0.20816	0.014
3608	212302_at	RTF1	0.25676	0.11746	0.1393	0.117
3609	212308_at	CLASP2	0.51172	0.5609	-0.04918	0.685
3610	212310_at	MIA3	0.25513	0.23678	0.01835	0.447
3611	212311_at	KIAA0746	0.50924	0.60963	-0.10039	0.847
3612	212312_at	BCL2L1	0.58885	0.70806	-0.11921	0.916
3613	212313_at	CHMP7	0.31898	0.43708	-0.1181	0.863
3614	212317_at	TNPO3	0.46203	0.4519	0.01013	0.474
3615	212320_at	TUBB	0.65656	0.47067	0.18589	<0.001
3616	212323_s_at	VPS13D	0.3124	0.27863	0.03377	0.394

3617	212329_at	SCAP	0.24719	0.4989	-0.25171	0.993
3618	212330_at	TFDP1	0.80483	0.73555	0.06928	0.001
3619	212332_at	RBL2	0.76773	0.80291	-0.03518	0.674
3620	212333_at	FAM98A	0.6321	0.51637	0.11573	0.1
3621	212338_at	MYO1D	0.62179	0.55533	0.06646	0.532
3622	212340_at	YIPF6	0.099235	0.18904	-0.089805	0.807
3623	212345_s_at	CREB3L2	0.71948	0.65602	0.06346	0.103
3624	212348_s_at	KDM1	0.56072	0.5612	-0.00048	0.497
3625	212350_at	TBC1D1	0.51031	0.61947	-0.10916	0.876
3626	212351_at	EIF2B5	0.16746	0.36697	-0.19951	0.969
3627	212352_s_at	TMED10	0.67999	0.55572	0.12427	0.077
3628	212355_at	KIAA0323	0.5474	0.49992	0.04748	0.291
3629	212357_at	FAM168A	0.51166	0.31432	0.19734	0.061
3630	212360_at	AMPD2	0.64603	0.63494	0.01109	0.452
3631	212366_at	ZNF292	0.7661	0.75132	0.01478	0.427
3632	212371_at	PPPDE1	0.65179	0.67579	-0.024	0.597
3633	212372_at	MYH10	0.11314	0.35886	-0.24572	0.974
3634	212374_at	FEM1B	0.21378	0.20005	0.01373	0.461
3635	212376_s_at	EP400	0.23816	0.2609	-0.02274	0.612
3636	212380_at	FTSJD2	0.44951	0.53371	-0.0842	0.796
3637	212381_at	USP24	0.40093	0.41815	-0.01722	0.565
3638	212383_at	ATP6V0A1	0.48596	0.51835	-0.03239	0.635
3639	212398_at	RDX	0.77778	0.84289	-0.06511	0.922
3640	212400_at	FAM102A	0.71742	0.62697	0.09045	0.141
3641	212401_s_at	CDC2L2	0.47736	0.71003	-0.23267	0.97
3642	212402_at	ZC3H13	0.40973	0.32762	0.08211	0.243
3643	212403_at	UBE3B	0.38142	0.51865	-0.13723	0.949
3644	212405_s_at	METTL13	0.32164	0.17213	0.14951	0.089
3645	212406_s_at	PCMTD2	0.22282	0.22341	-0.00059	0.543
3646	212408_at	TOR1AIP1	0.5725	0.59221	-0.01971	0.578
3647	212410_at	EFHA1	0.72154	0.78188	-0.06034	0.78
3648	212411_at	IMP4	0.62486	0.023232	0.601628	<0.001
3649	212415_at	SEPT6	0.1793	0.35619	-0.17689	0.908
3650	212420_at	ELF1	0.92465	0.93646	-0.01181	0.709
3651	212422_at	PDCD11	0.59164	0.65381	-0.06217	0.733
3652	212430_at	RBM38	0.48305	0.60251	-0.11946	0.951
3653	212434_at	GRPEL1	0.51191	0.20299	0.30892	<0.001
3654	212436_at	TRIM33	0.52619	0.36661	0.15958	0.071
3655	212437_at	CENPB	0.39468	0.27471	0.11997	0.065
3656	212439_at	IP6K1	0.16637	0.25549	-0.08912	0.792
3657	212440_at	SNRNP27	0.44659	0.56528	-0.11869	0.849
3658	212441_at	KIAA0232	0.54778	0.39104	0.15674	0.034
3659	212443_at	NBEAL2	0.55113	0.62922	-0.07809	0.842
3660	212445_s_at	NEDD4L	0.46102	0.54753	-0.08651	0.75
3661	212446_s_at	LASS6	0.43818	0.55079	-0.11261	0.901
3662	212447_at	KBTBD2	0.32105	0.28926	0.03179	0.404
3663	212449_s_at	LYPLA1	0.8321	0.85576	-0.02366	0.738
3664	212451_at	SECISBP2L	0.83887	0.84334	-0.00447	0.552
3665	212453_at	KIAA1279	0.39923	0.25639	0.14284	0.161
3666	212456_at	KIAA0664	0.65677	0.60501	0.05176	0.23
3667	212458_at	SPRED2	0.38709	0.45415	-0.06706	0.747
3668	212462_at	MYST4	0.38776	0.51275	-0.12499	0.887
3669	212465_at	SETD3	0.41611	0.59191	-0.1758	0.956
3670	212467_at	DNAJC13	0.56024	0.53701	0.02323	0.397

3671	212470_at	SPAG9	0.33013	0.37847	-0.04834	0.69
3672	212471_at	AVL9	0.066662	0.26933	-0.202668	0.964
3673	212473_s_at	MICAL2	0.08976	0.32999	-0.24023	0.978
3674	212476_at	ACAP2	0.81886	0.86902	-0.05016	0.893
3675	212479_s_at	RMND5A	0.6257	0.61998	0.00572	0.481
3676	212480_at	CYTSA	0.20496	0.24156	-0.0366	0.612
3677	212483_at	NIPBL	0.57257	0.59096	-0.01839	0.582
3678	212484_at	FAM89B	0.29706	0.37949	-0.08243	0.712
3679	212487_at	GPATCH8	0.90511	0.88074	0.02437	0.207
3680	212491_s_at	DNAJC8	0.80463	0.78942	0.01521	0.38
3681	212499_s_at	FCF1	0.72835	0.77497	-0.04662	0.845
3682	212500_at	ADO	0.43848	0.4728	-0.03432	0.61
3683	212501_at	CEBPB	0.31064	0.42865	-0.11801	0.983
3684	212505_s_at	KIAA0892	0.65489	0.55118	0.10371	0.13
3685	212507_at	TMEM131	0.72412	0.77979	-0.05567	0.747
3686	212508_at	MOAP1	0.44541	0.50782	-0.06241	0.692
3687	212509_s_at	MXRA7	0.15315	0.14366	0.00949	0.485
3688	212510_at	GPD1L	0.51451	0.33484	0.17967	0.042
3689	212512_s_at	CARM1	0.79911	0.7376	0.06151	0.164
3690	212515_s_at	DDX3X	0.75566	0.72175	0.03391	0.373
3691	212516_at	ARAP1	0.51379	0.50835	0.00544	0.478
3692	212517_at	ATRN	0.44528	0.36756	0.07772	0.211
3693	212518_at	PIP5K1C	0.42546	0.6095	-0.18404	0.971
3694	212520_s_at	SMARCA4	0.85153	0.85256	-0.00103	0.523
3695	212523_s_at	KIAA0146	0.48282	0.44992	0.0329	0.389
3696	212526_at	SPG20	0.26316	0.41244	-0.14928	0.864
3697	212527_at	PPPDE2	0.59533	0.77775	-0.18242	0.999
3698	212528_at	D15Wsu75e	0.31755	0.49147	-0.17392	0.878
3699	212529_at	LSM12	0.81764	0.80363	0.01401	0.384
3700	212530_at	NEK7	0.63805	0.69465	-0.0566	0.841
3701	212536_at	ATP11B	0.49681	0.53148	-0.03467	0.652
3702	212538_at	DOCK9	0.45276	0.56396	-0.1112	0.834
3703	212539_at	CHD1L	0.55807	0.54971	0.00836	0.498
3704	212540_at	CDC34	0.64998	0.70284	-0.05286	0.748
3705	212541_at	FLAD1	0.67087	0.43135	0.23952	0.007
3706	212542_s_at	PHIP	0.65697	0.54348	0.11349	0.21
3707	212543_at	AIM1	0.5699	0.60756	-0.03766	0.701
3708	212544_at	ZNHIT3	0.46751	0.63093	-0.16342	0.974
3709	212548_s_at	FRYL	0.4534	0.31334	0.14006	0.092
3710	212550_at	STAT5B	0.74768	0.59997	0.14771	0.045
3711	212553_at	RPRD2	0.77305	0.68369	0.08936	0.026
3712	212556_at	SCRIB	0.76056	0.67708	0.08348	0.127
3713	212557_at	ZNF451	0.29523	0.35948	-0.06425	0.721
3714	212558_at	SPRY1	0.49148	0.41091	0.08057	0.253
3715	212560_at	SORL1	0.5418	0.7309	-0.1891	0.981
3716	212561_at	DENND5A	0.7795	0.69621	0.08329	0.036
3717	212568_s_at	DLAT	0.80708	0.71092	0.09616	0.031
3718	212571_at	CHD8	0.87403	0.8811	-0.00707	0.558
3719	212572_at	STK38L	0.73989	0.67921	0.06068	0.186
3720	212573_at	ENDOD1	0.81026	0.8127	-0.00244	0.521
3721	212576_at	MGRN1	0.7	0.73136	-0.03136	0.687
3722	212579_at	SMCHD1	0.82596	0.7481	0.07786	0.044
3723	212584_at	AQR	0.76872	0.6638	0.10492	0.123
3724	212585_at	OSBPL8	0.6121	0.66272	-0.05062	0.67

3725	212586_at	CAST	0.27468	0.47502	-0.20034	0.922
3726	212587_s_at	PTPRC	0.72379	0.77282	-0.04903	0.753
3727	212589_at	RRAS2	0.49059	0.61714	-0.12655	0.88
3728	212592_at	IGJ	0.44142	0.61929	-0.17787	0.992
3729	212595_s_at	DAZAP2	0.23297	0.32764	-0.09467	0.799
3730	212597_s_at	HMGXB4	0.50564	0.57566	-0.07002	0.875
3731	212600_s_at	UQCRC2	0.66809	0.75485	-0.08676	0.882
3732	212601_at	ZZEF1	0.56617	0.27452	0.29165	0.002
3733	212603_at	MRPS31	0.67473	0.49727	0.17746	0.025
3734	212608_s_at	NUDT3	0.14295	0.28319	-0.14024	0.899
3735	212610_at	PTPN11	0.69818	0.69611	0.00207	0.495
3736	212611_at	DTX4	0.70752	0.77664	-0.06912	0.852
3737	212612_at	RCOR1	0.36756	0.42685	-0.05929	0.661
3738	212613_at	BTN3A2	0.10715	0.27803	-0.17088	0.896
3739	212614_at	ARID5B	0.46337	0.62216	-0.15879	0.797
3740	212621_at	TMEM194A	0.56698	0.59889	-0.03191	0.65
3741	212623_at	TMEM41B	0.27861	0.26499	0.01362	0.446
3742	212625_at	STX10	0.59528	0.61458	-0.0193	0.626
3743	212627_s_at	EXOSC7	0.65148	0.74484	-0.09336	0.85
3744	212630_at	EXOC3	0.33296	0.50427	-0.17131	0.924
3745	212634_at	KIAA0776	0.93781	0.90338	0.03443	0.123
3746	212637_s_at	WWP1	0.69709	0.71174	-0.01465	0.594
3747	212640_at	PTPLB	0.61096	0.58361	0.02735	0.425
3748	212642_s_at	HIVEP2	0.65922	0.65004	0.00918	0.439
3749	212643_at	MAPK1IP1L	0.53986	0.56778	-0.02792	0.645
3750	212646_at	RFTN1	0.6162	0.64258	-0.02638	0.652
3751	212647_at	RRAS	0.41107	0.37466	0.03641	0.359
3752	212649_at	DHX29	0.87799	0.8877	-0.00971	0.636
3753	212653_s_at	EHBP1	0.55273	0.67547	-0.12274	0.871
3754	212655_at	ZCCHC14	0.18163	0.41636	-0.23473	0.971
3755	212656_at	TSFM	0.38	0.62426	-0.24426	1
3756	212658_at	LHFPL2	0.56398	0.68257	-0.11859	0.929
3757	212663_at	FKBP15	0.51168	0.62767	-0.11599	0.765
3758	212664_at	TUBB4	0.54568	0.47556	0.07012	0.32
3759	212665_at	TIPARP	0.52994	0.50135	0.02859	0.435
3760	212666_at	SMURF1	0.42967	0.4287	0.00097	0.538
3761	212673_at	METAP1	0.60149	0.55805	0.04344	0.299
3762	212674_s_at	DHX30	0.55967	0.41935	0.14032	0.037
3763	212677_s_at	CEP68	0.69185	0.63026	0.06159	0.199
3764	212685_s_at	TBL2	0.70678	0.5922	0.11458	0.001
3765	212688_at	PIK3CB	0.55468	0.50404	0.05064	0.329
3766	212689_s_at	KDM3A	0.66091	0.64752	0.01339	0.471
3767	212690_at	DDHD2	0.4462	0.54314	-0.09694	0.775
3768	212692_s_at	LRBA	0.60598	0.6161	-0.01012	0.542
3769	212693_at	MDN1	0.71647	0.5373	0.17917	0.001
3770	212694_s_at	PCCB	0.50452	0.6564	-0.15188	0.937
3771	212696_s_at	RNF4	0.43979	0.49713	-0.05734	0.706
3772	212697_at	FAM134C	0.6699	0.51645	0.15345	0.017
3773	212698_s_at	SEPT10	0.24619	0.45683	-0.21064	0.933
3774	212699_at	SCAMP5	0.19391	0.27244	-0.07853	0.743
3775	212704_at	ZCCHC11	0.63945	0.67259	-0.03314	0.683
3776	212707_s_at	LOC100133005	0.35688	0.38149	-0.02461	0.585
3777	212708_at	MSL1	0.38217	0.20998	0.17219	0.059
3778	212712_at	CAMSAP1	0.21502	0.35497	-0.13995	0.895

3779	212716_s_at	EIF3K	0.67507	0.75111	-0.07604	0.853
3780	212718_at	PAPOLA	0.66618	0.63132	0.03486	0.358
3781	212721_at	SFRS12	0.6554	0.36181	0.29359	0.002
3782	212723_at	JMJD6	0.41018	0.26037	0.14981	0.072
3783	212726_at	PHF2	0.35137	0.32927	0.0221	0.451
3784	212729_at	DLG3	0.35339	0.48926	-0.13587	0.939
3785	212731_at	ANKRD46	0.60996	0.52463	0.08533	0.25
3786	212735_at	KIAA0226	0.35484	0.57935	-0.22451	0.98
3787	212738_at	ARHGAP19	0.7564	0.65155	0.10485	0.024
3788	212739_s_at	NME4	0.43824	0.42347	0.01477	0.479
3789	212742_at	RNF115	0.86438	0.71841	0.14597	<0.001
3790	212745_s_at	BBS4	0.64295	0.67244	-0.02949	0.66
3791	212746_s_at	CEP170	0.65863	0.55457	0.10406	0.053
3792	212747_at	ANKS1A	0.53193	0.45841	0.07352	0.233
3793	212751_at	UBE2N	0.59504	0.51586	0.07918	0.174
3794	212752_at	CLASP1	0.49042	0.32962	0.1608	0.077
3795	212753_at	PCGF3	0.55884	0.26256	0.29628	<0.001
3796	212754_s_at	MON2	0.79556	0.74897	0.04659	0.193
3797	212756_s_at	UBR2	0.19917	0.34864	-0.14947	0.889
3798	212758_s_at	ZEB1	0.921	0.91143	0.00957	0.393
3799	212763_at	CAMSAP1L1	0.84828	0.86233	-0.01405	0.626
3800	212766_s_at	ISG20L2	0.80956	0.75606	0.0535	0.218
3801	212767_at	MTG1	0.1097	0.29234	-0.18264	0.928
3802	212770_at	TLE3	0.4128	0.50165	-0.08885	0.781
3803	212773_s_at	TOMM20	0.6329	0.63619	-0.00329	0.535
3804	212774_at	ZNF238	0.51511	0.57961	-0.0645	0.723
3805	212779_at	KIAA1109	0.50566	0.335	0.17066	0.015
3806	212780_at	SOS1	0.23273	0.13411	0.09862	0.172
3807	212784_at	CIC	0.11624	0.026224	0.090016	0.244
3808	212785_s_at	LARP7	0.58811	0.64186	-0.05375	0.712
3809	212786_at	CLEC16A	0.41643	0.26357	0.15286	0.141
3810	212787_at	YLPM1	0.31344	0.1984	0.11504	0.087
3811	212789_at	NCAPD3	0.77662	0.50319	0.27343	<0.001
3812	212791_at	C1orf216	0.39296	0.3172	0.07576	0.131
3813	212794_s_at	KIAA1033	0.89842	0.91687	-0.01845	0.823
3814	212801_at	CIT	0.69538	0.67417	0.02121	0.381
3815	212802_s_at	GAPVD1	0.25016	0.27434	-0.02418	0.604
3816	212810_s_at	SLC1A4	0.080185	0.23077	-0.150585	0.986
3817	212813_at	JAM3	0.021144	0.15143	-0.130286	0.864
3818	212815_at	ASCC3	0.57009	0.60411	-0.03402	0.647
3819	212816_s_at	CBS	0.40488	0.30835	0.09653	0.181
3820	212817_at	DNAJB5	0.47994	0.3446	0.13534	0.077
3821	212819_at	ASB1	0.45356	0.20833	0.24523	0.01
3822	212820_at	DMXL2	0.60951	0.7068	-0.09729	0.804
3823	212822_at	HEG1	0.56457	0.5333	0.03127	0.406
3824	212824_at	FUBP3	0.66835	0.66801	0.00034	0.502
3825	212825_at	PAXIP1	0.57899	0.39989	0.1791	<0.001
3826	212826_s_at	SLC25A6	0.50155	0.63494	-0.13339	0.879
3827	212828_at	SYNJ2	0.12083	0.31992	-0.19909	0.944
3828	212830_at	MEGF9	0.029523	0.081474	-0.051951	0.7
3829	212832_s_at	CKAP5	0.67573	0.54766	0.12807	0.072
3830	212833_at	SLC25A46	0.5159	0.51389	0.00201	0.506
3831	212834_at	DDX52	0.33175	0.29291	0.03884	0.372
3832	212836_at	POLD3	0.674	0.44859	0.22541	<0.001

3833	212837_at	FAM175B	0.53894	0.59734	-0.0584	0.797
3834	212838_at	DNMBP	0.81882	0.83848	-0.01966	0.64
3835	212841_s_at	PPFIBP2	0.64266	0.53364	0.10902	0.133
3836	212846_at	RRP1B	0.74507	0.61255	0.13252	<0.001
3837	212847_at	FUBP1	0.80809	0.82628	-0.01819	0.595
3838	212851_at	DCUN1D4	0.23778	0.38658	-0.1488	0.83
3839	212856_at	GRAMD4	0.2381	0.24705	-0.00895	0.547
3840	212858_at	PAQR4	0.53594	0.56932	-0.03338	0.7
3841	212860_at	ZDHHC18	0.28308	0.15083	0.13225	0.147
3842	212861_at	MFSD5	0.73001	0.70768	0.02233	0.306
3843	212862_at	CDS2	0.61215	0.52356	0.08859	0.154
3844	212866_at	R3HCC1	0.34694	0.41838	-0.07144	0.754
3845	212867_at	NCOA2	0.80168	0.84173	-0.04005	0.77
3846	212871_at	MAPKAPK5	0.59021	0.35399	0.23622	0.008
3847	212873_at	HMHA1	0.52611	0.55467	-0.02856	0.605
3848	212875_s_at	C2CD2	0.15025	0.31585	-0.1656	0.905
3849	212876_at	B4GALT4	0.24278	0.23605	0.00673	0.477
3850	212877_at	KLC1	0.42768	0.34455	0.08313	0.224
3851	212880_at	WDR7	0.70624	0.57579	0.13045	0.09
3852	212881_at	PIAS4	0.039762	0.15607	-0.116308	0.85
3853	212885_at	MPHOSPH10	0.75863	0.7588	-0.00017	0.508
3854	212886_at	CCDC69	0.56694	0.67439	-0.10745	0.89
3855	212887_at	SEC23A	0.73894	0.63388	0.10506	0.011
3856	212890_at	SLC38A10	0.61956	0.45615	0.16341	0.006
3857	212891_s_at	GADD45GIP1	0.7572	0.69858	0.05862	0.145
3858	212892_at	ZNF282	0.52483	0.44798	0.07685	0.2
3859	212893_at	ZZZ3	0.4127	0.44868	-0.03598	0.631
3860	212894_at	SUPV3L1	0.50954	0.18781	0.32173	0.001
3861	212896_at	SKIV2L2	0.81007	0.79255	0.01752	0.394
3862	212898_at	KIAA0406	0.81604	0.72995	0.08609	0.059
3863	212901_s_at	CSTF2T	0.3303	0.41103	-0.08073	0.782
3864	212902_at	SEC24A	0.8327	0.85512	-0.02242	0.615
3865	212904_at	LRRC47	0.61863	0.61109	0.00754	0.466
3866	212906_at	GRAMD1B	0.6345	0.72062	-0.08612	0.881
3867	212907_at	SLC30A1	0.71164	0.71238	-0.00074	0.522
3868	212908_at	DNAJC16	0.59918	0.56989	0.02929	0.339
3869	212910_at	THAP11	0.62933	0.61036	0.01897	0.409
3870	212912_at	RPS6KA2	0.14362	0.22092	-0.0773	0.794
3871	212913_at	C6orf26	0.40064	0.54514	-0.1445	0.904
3872	212914_at	CBX7	0.52039	0.15514	0.36525	<0.001
3873	212916_at	PHF8	0.0092274	0.067944	-0.0587166	0.663
3874	212918_at	RECQL	0.31395	0.401	-0.08705	0.77
3875	212919_at	DCP2	0.39644	0.39738	-0.00094	0.52
3876	212926_at	SMC5	0.83229	0.90166	-0.06937	0.992
3877	212928_at	TSPYL4	0.48823	0.50275	-0.01452	0.573
3878	212929_s_at	FAM21A	0.49107	0.43309	0.05798	0.286
3879	212930_at	ATP2B1	0.39062	0.51854	-0.12792	0.84
3880	212931_at	TCF20	0.27173	0.43781	-0.16608	0.908
3881	212934_at	UBXN2B	0.32478	0.37843	-0.05365	0.672
3882	212936_at	FAM172A	0.34165	0.45959	-0.11794	0.848
3883	212943_at	KIAA0528	0.41174	0.36514	0.0466	0.347
3884	212944_at	SLC5A3	0.28539	0.2607	0.02469	0.395
3885	212945_s_at	MGA	0.33332	0.14936	0.18396	0.023
3886	212947_at	SLC9A8	0.25405	0.28738	-0.03333	0.608

3887	212948_at	CAMTA2	0.33196	0.14196	0.19	0.056
3888	212949_at	NCAPH	0.77962	0.82794	-0.04832	0.958
3889	212954_at	DYRK4	0.63365	0.38817	0.24548	<0.001
3890	212955_s_at	POLR2I	0.61087	0.61775	-0.00688	0.519
3891	212956_at	TBC1D9	0.14756	0.36188	-0.21432	0.96
3892	212959_s_at	GNPTAB	0.2986	0.39972	-0.10112	0.829
3893	212963_at	TM2D1	0.72541	0.78756	-0.06215	0.913
3894	212964_at	HIC2	0.10933	0.20694	-0.09761	0.823
3895	212968_at	RFNG	0.29658	0.45935	-0.16277	0.879
3896	212973_at	RPIA	0.45995	0.54165	-0.0817	0.786
3897	212974_at	DENND3	0.49796	0.65475	-0.15679	0.931
3898	212977_at	CXCR7	0.63769	0.70443	-0.06674	0.759
3899	212978_at	LRRC8B	0.16412	0.32224	-0.15812	0.902
3900	212979_s_at	FAM115A	0.55799	0.59032	-0.03233	0.63
3901	212983_at	HRAS	0.62599	0.41486	0.21113	<0.001
3902	212984_at	ATF2	0.61972	0.82395	-0.20423	1
3903	212986_s_at	TLK2	0.6669	0.69211	-0.02521	0.629
3904	212989_at	SGMS1	0.66757	0.64085	0.02672	0.368
3905	212990_at	SYNJ1	0.20527	0.26233	-0.05706	0.692
3906	212994_at	THOC2	0.87822	0.89585	-0.01763	0.682
3907	213000_at	MORC3	0.72303	0.60877	0.11426	0.12
3908	213002_at	MARCKS	0.46489	0.70129	-0.2364	0.999
3909	213008_at	FANCI	0.75824	0.70428	0.05396	0.124
3910	213009_s_at	TRIM37	0.64723	0.70804	-0.06081	0.738
3911	213010_at	PRKCDBP	0.45371	0.56234	-0.10863	0.891
3912	213011_s_at	TPI1	0.66489	0.64245	0.02244	0.386
3913	213012_at	NEDD4	0.19876	0.3453	-0.14654	0.844
3914	213016_at	BBX	0.74178	0.76463	-0.02285	0.659
3915	213017_at	ABHD3	0.76179	0.61609	0.1457	0.033
3916	213019_at	RANBP6	0.67937	0.58207	0.0973	0.118
3917	213025_at	THUMPD1	0.75267	0.77761	-0.02494	0.616
3918	213026_at	ATG12	0.53963	0.4856	0.05403	0.321
3919	213028_at	NFRKB	0.21065	0.075006	0.135644	0.224
3920	213031_s_at	WDR73	0.52084	0.47795	0.04289	0.357
3921	213035_at	ANKRD28	0.38575	0.42566	-0.03991	0.637
3922	213038_at	RNF19B	0.70702	0.78434	-0.07732	0.824
3923	213039_at	ARHGEF18	0.32677	0.35882	-0.03205	0.63
3924	213041_s_at	ATP5D	0.66291	0.6725	-0.00959	0.574
3925	213043_s_at	MED24	0.57832	0.50724	0.07108	0.184
3926	213044_at	ROCK1	0.52455	0.44606	0.07849	0.068
3927	213045_at	MAST3	0.64931	0.60239	0.04692	0.3
3928	213046_at	PABPN1	0.8525	0.83001	0.02249	0.232
3929	213048_s_at	SET	0.67579	0.76833	-0.09254	0.908
3930	213052_at	PRKAR2A	0.40789	0.32558	0.08231	0.224
3931	213054_at	HAUS5	0.09364	0.15092	-0.05728	0.68
3932	213058_at	TTC28	0.33328	0.48203	-0.14875	0.877
3933	213060_s_at	CHI3L2	0.0020192	0.16796	-0.1659408	0.928
3934	213061_s_at	NTAN1	0.36377	0.42935	-0.06558	0.654
3935	213065_at	ZFC3H1	0.42114	0.58581	-0.16467	0.945
3936	213070_at	PIK3C2A	0.092422	0.25972	-0.167298	0.873
3937	213073_at	ZFYVE26	0.66408	0.64291	0.02117	0.402
3938	213076_at	ITPKC	0.48024	0.35075	0.12949	0.117
3939	213077_at	YTHDC2	0.45904	0.41743	0.04161	0.341
3940	213079_at	TSR2	0.36092	0.32299	0.03793	0.402

3941	213081_at	ZBTB22	0.10236	0.041258	0.061102	0.319
3942	213082_s_at	SLC35D2	0.11239	0.22107	-0.10868	0.883
3943	213086_s_at	CSNK1A1	0.35583	0.4034	-0.04757	0.69
3944	213088_s_at	DNAJC9	0.81872	0.51288	0.30584	<0.001
3945	213089_at	LOC100272216	0.71414	0.78588	-0.07174	0.831
3946	213090_s_at	TAF4	0.35125	0.28785	0.0634	0.226
3947	213097_s_at	DNAJC2	0.74268	0.51839	0.22429	0.001
3948	213098_at	RQCD1	0.34666	0.35268	-0.00602	0.566
3949	213101_s_at	ACTR3	0.85685	0.80474	0.05211	0.05
3950	213103_at	STARD13	0.47053	0.65065	-0.18012	0.958
3951	213105_s_at	C16orf42	0.36173	0.17359	0.18814	0.038
3952	213106_at	ATP8A1	0.42214	0.54036	-0.11822	0.855
3953	213111_at	PIKFYVE	0.52691	0.60143	-0.07452	0.784
3954	213113_s_at	SLC43A3	0.2615	0.39342	-0.13192	0.851
3955	213115_at	ATG4A	0.31393	0.37192	-0.05799	0.741
3956	213117_at	KLHL9	0.38919	0.54543	-0.15624	0.924
3957	213119_at	SLC36A1	0.30302	0.29152	0.0115	0.473
3958	213122_at	TSPYL5	0.01423	0.12754	-0.11331	0.904
3959	213126_at	MED8	0.28299	0.29155	-0.00856	0.547
3960	213130_at	ZNF473	0.0385	0.058785	-0.020285	0.553
3961	213132_s_at	MCAT	0.34294	0.30912	0.03382	0.333
3962	213133_s_at	GCSH	0.53431	0.53039	0.00392	0.487
3963	213137_s_at	PTPN2	0.60532	0.62311	-0.01779	0.566
3964	213138_at	ARID5A	0.44684	0.48516	-0.03832	0.656
3965	213140_s_at	SS18L1	0.40651	0.62128	-0.21477	0.969
3966	213141_at	PSKH1	0.30944	0.11042	0.19902	0.068
3967	213145_at	FBXL14	0.49031	0.48651	0.0038	0.468
3968	213151_s_at	SEPT7	0.61889	0.64236	-0.02347	0.631
3969	213152_s_at	SFRS2B	0.5087	0.55208	-0.04338	0.66
3970	213153_at	SETD1B	0.34851	0.28549	0.06302	0.332
3971	213154_s_at	BICD2	0.35265	0.471	-0.11835	0.861
3972	213160_at	DOCK2	0.35619	0.26315	0.09304	0.233
3973	213161_at	C9orf97	0.55083	0.51192	0.03891	0.384
3974	213168_at	SP3	0.75483	0.49747	0.25736	0.001
3975	213170_at	GPX7	0.14546	0.44691	-0.30145	0.989
3976	213172_at	TTC9	0.43735	0.51562	-0.07827	0.821
3977	213173_at	PCNX	0.43763	0.48225	-0.04462	0.684
3978	213185_at	KIAA0556	0.41218	0.482	-0.06982	0.723
3979	213188_s_at	MINA	0.61896	0.45583	0.16313	0.098
3980	213190_at	COG7	0.16641	0.17584	-0.00943	0.541
3981	213191_at	TICAM1	0.079241	0.14961	-0.070369	0.689
3982	213192_at	THAP3	0.12516	0.2394	-0.11424	0.854
3983	213194_at	ROBO1	0.13986	0.39319	-0.25333	0.995
3984	213203_at	SNAPC5	0.29857	0.45904	-0.16047	0.949
3985	213204_at	CUL9	0.58118	0.6217	-0.04052	0.729
3986	213205_s_at	RAD54L2	0.23897	0.33136	-0.09239	0.798
3987	213206_at	GOSR2	0.60275	0.58171	0.02104	0.427
3988	213213_at	DIDO1	0.25304	0.435	-0.18196	0.934
3989	213216_at	OTUD3	0.052789	0.1743	-0.121511	0.794
3990	213218_at	ZNF187	0.14083	0.3925	-0.25167	0.99
3991	213221_s_at	SIK2	0.023417	0.14385	-0.120433	0.867
3992	213224_s_at	NCRNA00081	0.40004	0.40934	-0.0093	0.576
3993	213225_at	PPM1B	0.6735	0.74918	-0.07568	0.907
3994	213226_at	CCNA2	0.88265	0.79052	0.09213	<0.001

3995	213227_at	PGRMC2	0.47486	0.51403	-0.03917	0.712
3996	213237_at	C16orf88	0.5821	0.29507	0.28703	<0.001
3997	213238_at	ATP10D	0.66438	0.55133	0.11305	0.142
3998	213239_at	PIBF1	0.37432	0.21092	0.1634	0.08
3999	213244_at	SCAMP4	0.60172	0.66933	-0.06761	0.872
4000	213246_at	C14orf109	0.48874	0.64411	-0.15537	0.98
4001	213251_at	SMARCA5	0.66848	0.72469	-0.05621	0.739
4002	213252_at	SH3PXD2A	0.42191	0.41009	0.01182	0.448
4003	213253_at	SMC2	0.56693	0.63352	-0.06659	0.667
4004	213254_at	TNRC6B	0.68465	0.72793	-0.04328	0.734
4005	213256_at	MARCH3	0.67446	0.6203	0.05416	0.204
4006	213259_s_at	SARM1	0.2962	0.2262	0.07	0.29
4007	213261_at	LBA1	0.51812	0.63167	-0.11355	0.93
4008	213262_at	SACS	0.79032	0.61089	0.17943	0.001
4009	213266_at	TUBGCP4	0.31703	0.33435	-0.01732	0.574
4010	213267_at	DOPEY1	0.13524	0.24268	-0.10744	0.861
4011	213269_at	ZNF248	0.30126	0.23187	0.06939	0.249
4012	213272_s_at	TMEM159	0.61596	0.51219	0.10377	0.102
4013	213274_s_at	CTSB	0.15675	0.29881	-0.14206	0.82
4014	213278_at	MTMR9	0.64949	0.635	0.01449	0.479
4015	213279_at	DHRS1	0.1704	0.35357	-0.18317	0.923
4016	213282_at	APOOL	0.36201	0.45768	-0.09567	0.755
4017	213283_s_at	SALL2	0.12535	0.24895	-0.1236	0.875
4018	213287_s_at	KRT10	0.45872	0.62132	-0.1626	0.993
4019	213293_s_at	TRIM22	0.83867	0.73074	0.10793	0.035
4020	213294_at	EIF2AK2	0.64664	0.54624	0.1004	0.13
4021	213297_at	RMND5B	0.60317	0.66083	-0.05766	0.831
4022	213298_at	NFIC	0.46764	0.64397	-0.17633	0.962
4023	213300_at	ATG2A	0.30554	0.5142	-0.20866	0.953
4024	213302_at	PFAS	0.81213	0.72871	0.08342	0.037
4025	213304_at	FAM179B	0.65313	0.52204	0.13109	0.033
4026	213305_s_at	PPP2R5C	0.73938	0.71046	0.02892	0.326
4027	213310_at	EIF2C2	0.47291	0.47492	-0.00201	0.487
4028	213312_at	C6orf162	0.26398	0.086647	0.177333	0.011
4029	213318_s_at	BAT3	0.76149	0.5243	0.23719	0.002
4030	213320_at	PRMT3	0.81633	0.59676	0.21957	<0.001
4031	213322_at	C6orf130	0.26454	0.38062	-0.11608	0.831
4032	213324_at	SRC	0.39606	0.37081	0.02525	0.424
4033	213326_at	VAMP1	0.33082	0.48011	-0.14929	0.898
4034	213327_s_at	USP12	0.53489	0.44371	0.09118	0.164
4035	213328_at	NEK1	0.90809	0.90278	0.00531	0.432
4036	213333_at	MDH2	0.48636	0.43088	0.05548	0.266
4037	213340_s_at	KIAA0495	0.26028	0.38073	-0.12045	0.877
4038	213341_at	FEM1C	0.57997	0.57215	0.00782	0.469
4039	213346_at	C13orf27	0.72799	0.70133	0.02666	0.411
4040	213350_at	RPS11	0.73952	0.76934	-0.02982	0.718
4041	213351_s_at	TMCC1	0.48201	0.48994	-0.00793	0.549
4042	213353_at	ABCA5	0.68599	0.63569	0.0503	0.237
4043	213357_at	GTF2H5	0.42294	0.56567	-0.14273	0.956
4044	213361_at	TDRD7	0.4622	0.61116	-0.14896	0.927
4045	213365_at	ERI2	0.55734	0.53161	0.02573	0.423
4046	213370_s_at	SFMBT1	0.2122	0.31844	-0.10624	0.819
4047	213372_at	PAQR3	0.54745	0.68175	-0.1343	0.948
4048	213373_s_at	CASP8	0.64897	0.74469	-0.09572	0.841

4049	213376_at	ZBTB1	0.41643	0.51671	-0.10028	0.923
4050	213379_at	COQ2	0.51335	0.59548	-0.08213	0.779
4051	213383_at	SAPS2	0.25324	0.42606	-0.17282	0.923
4052	213387_at	ATAD2B	0.50282	0.30145	0.20137	0.04
4053	213390_at	ZC3H4	0.32174	0.32632	-0.00458	0.527
4054	213391_at	DPY19L4	0.15307	0.082375	0.070695	0.271
4055	213392_at	IQCK	0.1126	0.34715	-0.23455	0.976
4056	213394_at	MAPKBP1	0.64185	0.70769	-0.06584	0.756
4057	213398_s_at	SDR39U1	0.30251	0.43071	-0.1282	0.855
4058	213402_at	ZNF787	0.28186	0.32457	-0.04271	0.644
4059	213403_at	MFSD9	0.42605	0.10444	0.32161	<0.001
4060	213405_at	RAB22A	0.63765	0.62394	0.01371	0.461
4061	213408_s_at	PI4KA	0.52976	0.66165	-0.13189	0.955
4062	213409_s_at	RHEB	0.67035	0.70295	-0.0326	0.731
4063	213410_at	C10orf137	0.57411	0.52623	0.04788	0.332
4064	213414_s_at	RPS19	0.90337	0.86756	0.03581	0.016
4065	213415_at	CLIC2	0.35548	0.54214	-0.18666	0.908
4066	213420_at	DHX57	0.41361	0.41063	0.00298	0.489
4067	213427_at	RPP40	0.38802	0.52497	-0.13695	0.885
4068	213430_at	RUFY3	0.22436	0.44792	-0.22356	0.963
4069	213433_at	ARL3	0.19353	0.2318	-0.03827	0.641
4070	213434_at	STX2	0.33232	0.46529	-0.13297	0.908
4071	213436_at	CNR1	0.59848	0.70931	-0.11083	0.912
4072	213445_at	ZC3H3	0.19796	0.17616	0.0218	0.442
4073	213446_s_at	IQGAP1	0.94223	0.91508	0.02715	0.11
4074	213447_at	IPW	0.5497	0.53272	0.01698	0.46
4075	213449_at	POP1	0.61543	0.39713	0.2183	0.008
4076	213452_at	ZNF184	0.45972	0.54866	-0.08894	0.819
4077	213454_at	APITD1	0.64546	0.54625	0.09921	0.093
4078	213455_at	FAM114A1	0.11576	0.061154	0.054606	0.333
4079	213457_at	MFHAS1	0.64399	0.67774	-0.03375	0.655
4080	213461_at	NUDT21	0.65036	0.66399	-0.01363	0.609
4081	213469_at	PGAP1	0.41479	0.39216	0.02263	0.48
4082	213471_at	NPHP4	0.20651	0.18752	0.01899	0.479
4083	213474_at	KCTD7	0.15792	0.24866	-0.09074	0.78
4084	213475_s_at	ITGAL	0.45773	0.54957	-0.09184	0.798
4085	213478_at	RP1-21O18.1	0.55345	0.60772	-0.05427	0.7
4086	213483_at	PPWD1	0.39832	0.1988	0.19952	0.004
4087	213485_s_at	ABCC10	0.55605	0.51626	0.03979	0.401
4088	213489_at	MAPRE2	0.36552	0.25275	0.11277	0.206
4089	213494_s_at	YY1	0.47348	0.6413	-0.16782	0.93
4090	213508_at	C14orf147	0.50566	0.38889	0.11677	0.16
4091	213517_at	PCBP2	0.89752	0.90987	-0.01235	0.689
4092	213520_at	RECQL4	0.39568	0.22095	0.17473	0.034
4093	213521_at	PTPN18	0.45539	0.3789	0.07649	0.229
4094	213523_at	CCNE1	0.65773	0.40495	0.25278	<0.001
4095	213524_s_at	G0S2	0.40205	0.51265	-0.1106	0.849
4096	213526_s_at	LIN37	0.64545	0.52692	0.11853	0.014
4097	213527_s_at	ZNF688	0.65646	0.5958	0.06066	0.105
4098	213528_at	C1orf156	0.54222	0.57708	-0.03486	0.669
4099	213531_s_at	RAB3GAP1	0.37271	0.016955	0.355755	<0.001
4100	213534_s_at	PASK	0.43474	0.6421	-0.20736	0.97
4101	213540_at	HSD17B8	0.51822	0.39951	0.11871	0.135
4102	213546_at	DKFZP586I1420	0.37751	0.45191	-0.0744	0.771

4103	213548_s_at	CDV3	0.68698	0.72285	-0.03587	0.708
4104	213549_at	SLC18A2	0.68298	0.65646	0.02652	0.31
4105	213552_at	GLCE	0.60566	0.56193	0.04373	0.33
4106	213555_at	RWDD2A	0.60165	0.50761	0.09404	0.012
4107	213556_at	LOC390940	0.12481	0.18367	-0.05886	0.698
4108	213557_at	CRKRS	0.35342	0.28839	0.06503	0.287
4109	213560_at	GADD45B	0.59361	0.62955	-0.03594	0.74
4110	213566_at	RNASE6	0.4565	0.6753	-0.2188	0.98
4111	213568_at	OSR2	0.48108	0.53659	-0.05551	0.711
4112	213577_at	SQLE	0.81504	0.80278	0.01226	0.42
4113	213587_s_at	ATP6V0E2	0.24028	0.33976	-0.09948	0.848
4114	213590_at	LOC100133772	0.25961	0.18499	0.07462	0.31
4115	213593_s_at	TRA2A	0.77339	0.86465	-0.09126	0.954
4116	213599_at	OIP5	0.76873	0.82376	-0.05503	0.812
4117	213603_s_at	RAC2	0.76378	0.73057	0.03321	0.297
4118	213604_at	TCEB3	0.67374	0.34923	0.32451	<0.001
4119	213605_s_at	LOC100134401	0.67782	0.73726	-0.05944	0.827
4120	213606_s_at	ARHGDIA	0.77101	0.46395	0.30706	<0.001
4121	213608_s_at	SRRD	0.55024	0.60276	-0.05252	0.656
4122	213618_at	ARAP2	0.62248	0.59561	0.02687	0.415
4123	213622_at	COL9A2	0.70586	0.54731	0.15855	0.021
4124	213623_at	KIF3A	0.65127	0.69064	-0.03937	0.712
4125	213625_at	ZKSCAN4	0.14868	0.22429	-0.07561	0.746
4126	213626_at	CBR4	0.60246	0.5952	0.00726	0.479
4127	213627_at	MAGED2	0.20537	0.34424	-0.13887	0.976
4128	213634_s_at	TRMU	0.33444	0.27777	0.05667	0.24
4129	213638_at	PHACTR1	0.61688	0.61113	0.00575	0.514
4130	213642_at	RPL27	0.78016	0.83435	-0.05419	0.924
4131	213647_at	DNA2	0.68729	0.48263	0.20466	<0.001
4132	213650_at	GOLGA8A	0.78572	0.73897	0.04675	0.236
4133	213654_at	TAF5L	0.29793	0.40764	-0.10971	0.846
4134	213660_s_at	TOP3B	0.51392	0.47267	0.04125	0.335
4135	213664_at	SLC1A1	0.24977	0.4769	-0.22713	1
4136	213671_s_at	MARS	0.78759	0.51169	0.2759	0.001
4137	213677_s_at	PMS1	0.68484	0.51821	0.16663	0.061
4138	213679_at	TTC30A	0.34263	0.19205	0.15058	0.068
4139	213681_at	CYHR1	0.014361	0.096725	-0.082364	0.728
4140	213687_s_at	RPL35A	0.85185	0.84287	0.00898	0.418
4141	213694_at	RSBN1	0.53533	0.39894	0.13639	0.12
4142	213699_s_at	YWHAQ	0.82439	0.79602	0.02837	0.328
4143	213701_at	C12orf29	0.43309	0.40001	0.03308	0.475
4144	213703_at	LOC150759	0.18644	0.55526	-0.36882	1
4145	213704_at	RABGGTB	0.59814	0.48177	0.11637	0.091
4146	213705_at	MAT2A	0.8767	0.7319	0.1448	<0.001
4147	213736_at	COX5B	0.72303	0.81985	-0.09682	0.987
4148	213738_s_at	ATP5A1	0.85057	0.7901	0.06047	0.062
4149	213742_at	SFRS11	0.63659	0.80286	-0.16627	0.993
4150	213743_at	CCNT2	0.41571	0.26749	0.14822	0.103
4151	213746_s_at	FLNA	0.18121	0.28763	-0.10642	0.812
4152	213748_at	TRIM66	0.67202	0.71049	-0.03847	0.738
4153	213757_at	EIF5A	0.26081	0.39159	-0.13078	0.891
4154	213761_at	MDM1	0.52446	0.55655	-0.03209	0.693
4155	213763_at	HIPK2	0.70959	0.76732	-0.05773	0.851
4156	213779_at	EMID1	0.5283	0.64212	-0.11382	0.853

4157	213787_s_at	EBP	0.69535	0.75957	-0.06422	0.812
4158	213792_s_at	INSR	0.35185	0.36872	-0.01687	0.567
4159	213793_s_at	HOMER1	0.55978	0.52322	0.03656	0.471
4160	213794_s_at	NGDN	0.65098	0.73741	-0.08643	0.895
4161	213798_s_at	CAP1	0.62811	0.62076	0.00735	0.464
4162	213799_s_at	PTPRA	0.76228	0.74278	0.0195	0.371
4163	213803_at	KPNB1	0.58308	0.69755	-0.11447	0.921
4164	213804_at	INPP5B	0.10167	0.28171	-0.18004	0.941
4165	213810_s_at	AKIRIN2	0.60807	0.46594	0.14213	0.07
4166	213812_s_at	CAMKK2	0.2743	0.4072	-0.1329	0.83
4167	213820_s_at	STARD5	0.16533	0.23133	-0.066	0.735
4168	213826_s_at	LOC100133109	0.78599	0.79575	-0.00976	0.59
4169	213846_at	COX7C	0.74311	0.70069	0.04242	0.212
4170	213850_s_at	SFRS2IP	0.76257	0.89043	-0.12786	0.997
4171	213851_at	TMEM110	0.38543	0.3703	0.01513	0.464
4172	213853_at	DNAJC24	0.37638	0.21672	0.15966	0.093
4173	213861_s_at	FAM119B	0.39937	0.44066	-0.04129	0.638
4174	213872_at	C6orf62	0.77362	0.62399	0.14963	<0.001
4175	213878_at	PYROXD1	0.18826	0.12488	0.06338	0.272
4176	213879_at	SUMO2	0.56658	0.65627	-0.08969	0.846
4177	213887_s_at	POLR2E	0.51719	0.65952	-0.14233	0.931
4178	213888_s_at	LOC100133233	0.20508	0.35305	-0.14797	0.891
4179	213891_s_at	TCF4	0.33906	0.40983	-0.07077	0.744
4180	213897_s_at	MRPL23	0.69167	0.7502	-0.05853	0.812
4181	213906_at	MYBL1	0.2385	0.41574	-0.17724	0.918
4182	213908_at	WHAMML1	0.30156	0.45406	-0.1525	0.865
4183	213916_at	ZNF20	0.40692	0.37085	0.03607	0.381
4184	213919_at	DNAJC4	0.3206	0.27113	0.04947	0.337
4185	213922_at	TTBK2	0.65489	0.74794	-0.09305	0.903
4186	213923_at	RAP2B	0.415	0.4817	-0.0667	0.708
4187	213927_at	MAP3K9	0.14329	0.17464	-0.03135	0.595
4188	213934_s_at	ZNF23	0.19243	0.13778	0.05465	0.364
4189	213937_s_at	FTSJ1	0.55201	0.42257	0.12944	0.019
4190	213938_at	ERC2	0.014813	0.024238	-0.009425	0.562
4191	213940_s_at	FNBP1	0.82992	0.85921	-0.02929	0.73
4192	213951_s_at	PSMC3IP	0.88492	0.74856	0.13636	<0.001
4193	213959_s_at	RPGRIP1L	0.29164	0.29341	-0.00177	0.54
4194	213963_s_at	SAP30	0.75085	0.73027	0.02058	0.302
4195	213970_at	RABL3	0.31686	0.43364	-0.11678	0.779
4196	213979_s_at	CTBP1	0.53099	0.67597	-0.14498	0.938
4197	213982_s_at	RABGAP1L	0.61067	0.73858	-0.12791	0.978
4198	214004_s_at	VGLL4	0.66012	0.68203	-0.02191	0.619
4199	214006_s_at	GGCX	0.49597	0.58663	-0.09066	0.841
4200	214007_s_at	TWF1	0.73697	0.7371	-0.00013	0.514
4201	214011_s_at	NOP16	0.6484	0.68688	-0.03848	0.698
4202	214022_s_at	IFITM1	0.66491	0.69834	-0.03343	0.673
4203	214030_at	CRYBG3	0.30853	0.38997	-0.08144	0.77
4204	214036_at	EFNA5	0.40421	0.60303	-0.19882	0.933
4205	214039_s_at	LAPTM4B	0.15821	0.38295	-0.22474	0.961
4206	214042_s_at	RPL22	0.81803	0.82663	-0.0086	0.588
4207	214045_at	LIAS	0.7802	0.7623	0.0179	0.406
4208	214048_at	MBD4	0.48568	0.63644	-0.15076	0.955
4209	214051_at	TMSB15B	0.22483	0.38633	-0.1615	0.896
4210	214054_at	DOK2	0.069927	0.1407	-0.070773	0.631

4211	214057_at	MCL1	0.86719	0.85874	0.00845	0.427
4212	214060_at	SSBP1	0.55879	0.61003	-0.05124	0.704
4213	214061_at	WDR67	0.58332	0.52251	0.06081	0.266
4214	214075_at	NENF	0.54092	0.58453	-0.04361	0.683
4215	214079_at	DHRS2	0.26346	0.35034	-0.08688	0.785
4216	214083_at	LOC100132532	0.63322	0.49214	0.14108	0.082
4217	214086_s_at	PARP2	0.61177	0.44901	0.16276	0.064
4218	214096_s_at	SHMT2	0.77563	0.60843	0.1672	0.052
4219	214101_s_at	NPEPPS	0.52475	0.5902	-0.06545	0.8
4220	214106_s_at	GMDS	0.23559	0.41954	-0.18395	0.934
4221	214112_s_at	CXorf40A	0.75784	0.58085	0.17699	0.001
4222	214113_s_at	RBM8A	0.61897	0.66808	-0.04911	0.696
4223	214116_at	BTD	0.23227	0.34477	-0.1125	0.846
4224	214126_at	MCART1	0.52008	0.17351	0.34657	<0.001
4225	214132_at	ATP5C1	0.40015	0.406	-0.00585	0.537
4226	214144_at	POLR2D	0.103	0.155	-0.052	0.698
4227	214148_at	FOXM1	0.34951	0.3605	-0.01099	0.545
4228	214152_at	CCPG1	0.62294	0.57557	0.04737	0.29
4229	214153_at	ELOVL5	0.48001	0.422	0.05801	0.283
4230	214155_s_at	LARP4	0.67595	0.6692	0.00675	0.459
4231	214167_s_at	RPLP0	0.87105	0.87706	-0.00601	0.582
4232	214179_s_at	NFE2L1	0.56191	0.21179	0.35012	<0.001
4233	214182_at	LOC100132430	0.79124	0.84654	-0.0553	0.907
4234	214186_s_at	HCG26	0.15527	0.21912	-0.06385	0.729
4235	214202_at	PGGT1B	0.23805	0.22597	0.01208	0.474
4236	214221_at	ALMS1	0.2399	0.28936	-0.04946	0.667
4237	214224_s_at	PIN4	0.22451	0.2594	-0.03489	0.628
4238	214231_s_at	KIAA0564	0.1715	0.23131	-0.05981	0.695
4239	214264_s_at	C14orf143	0.26901	0.48924	-0.22023	0.95
4240	214274_s_at	ACAA1	0.79294	0.77041	0.02253	0.339
4241	214281_s_at	RCHY1	0.49983	0.4679	0.03193	0.373
4242	214287_s_at	CDC2L5	0.50296	0.62022	-0.11726	0.887
4243	214290_s_at	HIST2H2AA3	0.36321	0.55969	-0.19648	0.979
4244	214291_at	LOC729046	0.43446	0.52829	-0.09383	0.824
4245	214305_s_at	SF3B1	0.88519	0.82005	0.06514	0.027
4246	214313_s_at	EIF5B	0.64604	0.63115	0.01489	0.437
4247	214328_s_at	HSP90AA1	0.8706	0.72878	0.14182	0.002
4248	214339_s_at	MAP4K1	0.57585	0.68589	-0.11004	0.88
4249	214352_s_at	KRAS	0.91877	0.8732	0.04557	0.032
4250	214356_s_at	KIAA0368	0.4448	0.43759	0.00721	0.472
4251	214364_at	MTERFD2	0.18495	0.29983	-0.11488	0.846
4252	214366_s_at	ALOX5	0.33904	0.39694	-0.0579	0.687
4253	214374_s_at	PPFIBP1	0.70362	0.63497	0.06865	0.18
4254	214377_s_at	CTRL	0.042201	0.17238	-0.130179	0.836
4255	214422_at	LOC131185	0.70806	0.69847	0.00959	0.484
4256	214427_at	NOP2	0.72354	0.46258	0.26096	<0.001
4257	214429_at	MTMR6	0.20599	0.296	-0.09001	0.8
4258	214430_at	GLA	0.56012	0.7293	-0.16918	0.966
4259	214431_at	GMPS	0.93931	0.82967	0.10964	<0.001
4260	214440_at	NAT1	0.45348	0.5786	-0.12512	0.928
4261	214441_at	STX6	0.10231	0.11086	-0.00855	0.52
4262	214446_at	ELL2	0.69275	0.72789	-0.03514	0.676
4263	214447_at	ETS1	0.57803	0.60073	-0.0227	0.626
4264	214452_at	BCAT1	0.369	0.51835	-0.14935	0.91

4265	214453_s_at	IFI44	0.65813	0.64257	0.01556	0.433
4266	214455_at	HIST1H2BC	0.47598	0.51621	-0.04023	0.706
4267	214467_at	GPR65	0.35237	0.31392	0.03845	0.322
4268	214469_at	HIST1H2AE	0.45382	0.53499	-0.08117	0.827
4269	214472_at	HIST1H2AD	0.41388	0.43507	-0.02119	0.582
4270	214474_at	PRKAB2	0.66929	0.78253	-0.11324	0.875
4271	214481_at	HIST1H2AM	0.33084	0.35547	-0.02463	0.593
4272	214482_at	ZBTB25	0.494	0.33154	0.16246	0.087
4273	214484_s_at	SIGMAR1	0.50191	0.35752	0.14439	0.086
4274	214502_at	HIST1H2BJ	0.28953	0.37247	-0.08294	0.742
4275	214507_s_at	EXOSC2	0.76233	0.607	0.15533	0.007
4276	214513_s_at	CREB1	0.635	0.39422	0.24078	0.008
4277	214519_s_at	RLN2	0.13417	0.35345	-0.21928	0.958
4278	214527_s_at	PQBP1	0.81039	0.83544	-0.02505	0.682
4279	214539_at	SERPINB10	0.13761	0.43522	-0.29761	0.998
4280	214554_at	HIST1H2AL	0.35101	0.43979	-0.08878	0.818
4281	214572_s_at	INSL3	0.24165	0.18223	0.05942	0.348
4282	214583_at	RSC1A1	0.51376	0.54292	-0.02916	0.582
4283	214585_s_at	VPS52	0.20472	0.33417	-0.12945	0.889
4284	214597_at	SSTR2	0.54695	0.67659	-0.12964	0.911
4285	214614_at	MNX1	0.76254	0.83613	-0.07359	0.942
4286	214615_at	P2RY10	0.36265	0.44595	-0.0833	0.813
4287	214617_at	PRF1	0.11544	0.21582	-0.10038	0.803
4288	214626_s_at	GANAB	0.59007	0.40462	0.18545	0.006
4289	214657_s_at	NCRNA00084	0.41888	0.4994	-0.08052	0.798
4290	214658_at	TMED7	0.68686	0.78383	-0.09697	0.942
4291	214661_s_at	NOP14	0.64918	0.56908	0.0801	0.101
4292	214662_at	WDR43	0.63839	0.32451	0.31388	<0.001
4293	214670_at	ZKSCAN1	0.71927	0.73959	-0.02032	0.67
4294	214672_at	TTLL5	0.81074	0.63795	0.17279	<0.001
4295	214674_at	USP19	0.034383	0.086148	-0.051765	0.683
4296	214681_at	GK	0.25513	0.1164	0.13873	0.172
4297	214683_s_at	CLK1	0.6196	0.35389	0.26571	0.006
4298	214686_at	ZNF266	0.33301	0.35915	-0.02614	0.569
4299	214688_at	TLE4	0.24726	0.21104	0.03622	0.418
4300	214690_at	TAF1B	0.49322	0.55549	-0.06227	0.768
4301	214694_at	LOC729143	0.23041	0.36796	-0.13755	0.869
4302	214696_at	C17orf91	0.75806	0.60459	0.15347	<0.001
4303	214697_s_at	ROD1	0.74137	0.66949	0.07188	0.22
4304	214703_s_at	MAN2B2	0.39038	0.37703	0.01335	0.455
4305	214709_s_at	KTN1	0.64216	0.78248	-0.14032	0.983
4306	214710_s_at	CCNB1	0.8848	0.76407	0.12073	<0.001
4307	214711_at	GATC	0.4899	0.41757	0.07233	0.204
4308	214714_at	ZNF394	0.64931	0.49733	0.15198	0.075
4309	214717_at	DKFZp434H1419	0.16146	0.22939	-0.06793	0.685
4310	214719_at	SLC46A3	0.69156	0.60511	0.08645	0.184
4311	214722_at	NOTCH2NL	0.47741	0.68544	-0.20803	0.97
4312	214729_at	TWISTNB	0.77847	0.50541	0.27306	0.002
4313	214731_at	CTTNBP2NL	0.73909	0.74232	-0.00323	0.549
4314	214733_s_at	YIPF1	0.57686	0.66613	-0.08927	0.889
4315	214735_at	IPCEF1	0.76764	0.76142	0.00622	0.49
4316	214736_s_at	ADD1	0.63629	0.47634	0.15995	0.04
4317	214739_at	LRCH3	0.58359	0.47985	0.10374	0.157
4318	214741_at	ZNF131	0.31977	0.45665	-0.13688	0.848

4319	214742_at	AZI1	0.4673	0.39905	0.06825	0.25
4320	214744_s_at	RPL23	0.59561	0.56253	0.03308	0.369
4321	214747_at	ZBED4	0.41037	0.4426	-0.03223	0.622
4322	214748_at	N4BP2L2	0.53254	0.37418	0.15836	0.084
4323	214749_s_at	ARMCX6	0.7515	0.73294	0.01856	0.411
4324	214751_at	ZNF468	0.53853	0.56583	-0.0273	0.635
4325	214755_at	UAP1L1	0.37663	0.4819	-0.10527	0.751
4326	214757_at	PMS2L2	0.78603	0.81649	-0.03046	0.706
4327	214759_at	WTAP	0.068003	0.039082	0.028921	0.366
4328	214762_at	ATP6V1G2	0.26667	0.3223	-0.05563	0.697
4329	214764_at	RRP15	0.38802	0.32754	0.06048	0.248
4330	214765_s_at	NAAA	0.088133	0.41678	-0.328647	0.994
4331	214766_s_at	AHCTF1	0.3252	0.38705	-0.06185	0.688
4332	214772_at	C11orf41	0.22057	0.39186	-0.17129	0.886
4333	214778_at	MEGF8	0.70867	0.73587	-0.0272	0.722
4334	214779_s_at	SGSM3	0.41087	0.32505	0.08582	0.229
4335	214780_s_at	MYO9B	0.392	0.50425	-0.11225	0.856
4336	214785_at	VPS13A	0.55106	0.64563	-0.09457	0.875
4337	214787_at	DENND4A	0.60932	0.7129	-0.10358	0.935
4338	214790_at	SENP6	0.40599	0.42008	-0.01409	0.57
4339	214791_at	SP140L	0.37078	0.27304	0.09774	0.155
4340	214801_at	IFRG15	0.74353	0.56378	0.17975	0.007
4341	214804_at	CENPI	0.73408	0.53963	0.19445	<0.001
4342	214806_at	BICD1	0.37377	0.61723	-0.24346	0.971
4343	214813_at	ZNF75D	0.044656	0.016572	0.028084	0.387
4344	214814_at	YTHDC1	0.59674	0.72683	-0.13009	0.933
4345	214828_s_at	RRP7A	0.67484	0.60145	0.07339	0.215
4346	214830_at	SLC38A6	0.37369	0.39187	-0.01818	0.597
4347	214838_at	SFT2D2	0.56499	0.52555	0.03944	0.32
4348	214843_s_at	USP33	0.82147	0.86882	-0.04735	0.789
4349	214848_at	YWHAZ	0.023316	0.0063941	0.0169219	0.437
4350	214849_at	KCTD20	0.48315	0.55743	-0.07428	0.708
4351	214850_at	LOC100170939	0.48389	0.62021	-0.13632	0.913
4352	214857_at	C10orf95	0.48253	0.49862	-0.01609	0.567
4353	214861_at	KDM4C	0.30439	0.44089	-0.1365	0.85
4354	214864_s_at	GRHPR	0.66078	0.63692	0.02386	0.394
4355	214876_s_at	TUBGCP5	0.25927	0.13525	0.12402	0.129
4356	214878_at	ZNF37A	0.60599	0.6672	-0.06121	0.743
4357	214881_s_at	UBTF	0.78154	0.79362	-0.01208	0.597
4358	214882_s_at	SFRS2	0.76262	0.46861	0.29401	<0.001
4359	214895_s_at	ADAM10	0.70005	0.6855	0.01455	0.428
4360	214910_s_at	APOM	0.35579	0.48983	-0.13404	0.855
4361	214918_at	HNRNPM	0.44506	0.46023	-0.01517	0.583
4362	214919_s_at	ANKHD1-EIF4EBP3	0.5224	0.44503	0.07737	0.219
4363	214931_s_at	SRPK2	0.44183	0.26246	0.17937	0.053
4364	214941_s_at	PRPF40A	0.8216	0.83286	-0.01126	0.629
4365	214943_s_at	RBM34	0.65477	0.70676	-0.05199	0.77
4366	214948_s_at	TMF1	0.18682	0.37451	-0.18769	0.981
4367	214949_at	hCG_1795560	0.41492	0.52392	-0.109	0.847
4368	214953_s_at	APP	0.028668	0.26116	-0.232492	0.982
4369	214958_s_at	TMC6	0.72501	0.81185	-0.08684	0.918
4370	214962_s_at	NUP160	0.0033981	0.20738	-0.2039819	0.805
4371	214965_at	SPATA2L	0.56381	0.55848	0.00533	0.493
4372	214972_at	MGEA5	0.60735	0.77767	-0.17032	0.898

4373	214975_s_at	MTMR1	0.36258	0.48602	-0.12344	0.837
4374	214976_at	RPL13	0.28093	0.061627	0.219303	0.113
4375	214982_at	SNRNP200	0.38528	0.4041	-0.01882	0.561
4376	214991_s_at	PIGO	0.11825	0.27553	-0.15728	0.923
4377	214992_s_at	DNASE2	0.60521	0.63738	-0.03217	0.622
4378	214994_at	APOBEC3F	0.5851	0.5468	0.0383	0.365
4379	214997_at	C9orf126	0.39296	0.39064	0.00232	0.525
4380	215001_s_at	GLUL	0.67245	0.64157	0.03088	0.343
4381	215006_at	EZH2	0.59329	0.56443	0.02886	0.399
4382	215011_at	SNHG3	0.59667	0.48288	0.11379	0.112
4383	215023_s_at	PEX1	0.4768	0.44432	0.03248	0.444
4384	215024_at	C7orf28B	0.81294	0.85873	-0.04579	0.918
4385	215029_at	C1orf108	0.76484	0.85824	-0.0934	0.991
4386	215030_at	GRSF1	0.51196	0.62065	-0.10869	0.902
4387	215043_s_at	LOC653188	0.10863	0.3344	-0.22577	0.953
4388	215046_at	C2orf67	0.7097	0.64816	0.06154	0.18
4389	215058_at	DENND5B	0.50169	0.58891	-0.08722	0.828
4390	215068_s_at	FBXL18	0.44609	0.53362	-0.08753	0.793
4391	215071_s_at	HIST1H2AC	0.47644	0.5103	-0.03386	0.677
4392	215075_s_at	GRB2	0.58792	0.67814	-0.09022	0.798
4393	215084_s_at	LRRC42	0.45875	0.3998	0.05895	0.38
4394	215087_at	C15orf39	0.29752	0.33658	-0.03906	0.655
4395	215088_s_at	SDHC	0.59293	0.66444	-0.07151	0.753
4396	215089_s_at	RBM10	0.50785	0.53702	-0.02917	0.65
4397	215091_s_at	GTF3A	0.75064	0.76922	-0.01858	0.64
4398	215096_s_at	ESD	0.84218	0.84536	-0.00318	0.528
4399	215109_at	KIAA0492	0.29608	0.48587	-0.18979	0.942
4400	215111_s_at	TSC22D1	0.20711	0.28135	-0.07424	0.742
4401	215123_at	LOC642778	0.66169	0.72939	-0.0677	0.887
4402	215127_s_at	RBMS1	0.33542	0.49534	-0.15992	0.92
4403	215134_at	PI4K2A	0.21232	0.10598	0.10634	0.14
4404	215136_s_at	EXOSC8	0.87759	0.74291	0.13468	0.066
4405	215143_at	DPY19L2P2	0.46225	0.40548	0.05677	0.285
4406	215148_s_at	APBA3	0.64471	0.5184	0.12631	0.026
4407	215159_s_at	NADK	0.10448	0.21127	-0.10679	0.853
4408	215170_s_at	CEP152	0.63925	0.72974	-0.09049	0.895
4409	215190_at	EIF3M	0.40739	0.55553	-0.14814	0.938
4410	215191_at	FBXL11	0.62243	0.71739	-0.09496	0.906
4411	215201_at	REPS1	0.41277	0.51095	-0.09818	0.849
4412	215210_s_at	DLST	0.57603	0.58099	-0.00496	0.532
4413	215218_s_at	WDR62	0.071366	0.18918	-0.117814	0.824
4414	215223_s_at	SOD2	0.32708	0.40344	-0.07636	0.781
4415	215224_at	SNORA21	0.61786	0.57762	0.04024	0.315
4416	215271_at	TNN	0.41237	0.52788	-0.11551	0.836
4417	215285_s_at	PHTF1	0.55463	0.61166	-0.05703	0.75
4418	215307_at	ZNF529	0.38342	0.42425	-0.04083	0.672
4419	215318_at	CG012	0.32581	0.61571	-0.2899	0.991
4420	215338_s_at	NKTR	0.89689	0.90662	-0.00973	0.645
4421	215343_at	CCDC88C	0.18907	0.50071	-0.31164	0.998
4422	215351_at	RTCD1	0.17367	0.35768	-0.18401	0.967
4423	215354_s_at	PELP1	0.48602	0.29178	0.19424	0.021
4424	215378_at	ANKHD1	0.20908	0.40944	-0.20036	0.965
4425	215380_s_at	GGCT	0.81551	0.69245	0.12306	0.022
4426	215390_at	C9orf5	0.3213	0.39145	-0.07015	0.774

4427	215399_s_at	OS9	0.39558	0.10331	0.29227	<0.001
4428	215416_s_at	STOML2	0.7672	0.82405	-0.05685	0.871
4429	215424_s_at	SNW1	0.21337	0.064881	0.148489	0.159
4430	215429_s_at	ZNF428	0.41232	0.50029	-0.08797	0.827
4431	215440_s_at	BEX4	0.0073238	0.14524	-0.1379162	0.855
4432	215465_at	ABCA12	0.42195	0.51423	-0.09228	0.838
4433	215470_at	GTF2H2B	0.6134	0.76309	-0.14969	0.983
4434	215482_s_at	EIF2B4	0.44002	0.44106	-0.00104	0.487
4435	215485_s_at	ICAM1	0.59981	0.43179	0.16802	0.019
4436	215499_at	MAP2K3	0.27105	0.51633	-0.24528	0.953
4437	215501_s_at	DUSP10	0.44359	0.5298	-0.08621	0.799
4438	215505_s_at	STRN3	0.35509	0.57063	-0.21554	0.977
4439	215509_s_at	BUB1	0.70901	0.73938	-0.03037	0.72
4440	215543_s_at	LARGE	0.25728	0.40957	-0.15229	0.914
4441	215545_at	ERCC3	0.61662	0.67081	-0.05419	0.768
4442	215548_s_at	SCFD1	0.51356	0.38774	0.12582	<0.001
4443	215577_at	UBE2E1	0.78102	0.83065	-0.04963	0.856
4444	215596_s_at	RNF160	0.49891	0.54971	-0.0508	0.741
4445	215602_at	FGD2	0.5942	0.66079	-0.06659	0.788
4446	215606_s_at	ERC1	0.30907	0.48628	-0.17721	0.938
4447	215631_s_at	BRMS1	0.53147	0.56643	-0.03496	0.61
4448	215639_at	SH2D3C	0.59113	0.51952	0.07161	0.197
4449	215641_at	SEC24D	0.49975	0.76943	-0.26968	1
4450	215648_at	NUDCD3	0.62845	0.64183	-0.01338	0.607
4451	215684_s_at	ASCC2	0.84527	0.85685	-0.01158	0.664
4452	215694_at	SPATA5L1	0.56723	0.40122	0.16601	0.026
4453	215696_s_at	SEC16A	0.52121	0.42403	0.09718	0.274
4454	215707_s_at	PRNP	0.23681	0.28081	-0.044	0.635
4455	215711_s_at	WEE1	0.78769	0.59376	0.19393	<0.001
4456	215718_s_at	PHF3	0.62809	0.682	-0.05391	0.834
4457	215728_s_at	ACOT7	0.68126	0.71243	-0.03117	0.671
4458	215732_s_at	DTX2	0.48407	0.28016	0.20391	0.043
4459	215734_at	C19orf36	0.59265	0.64073	-0.04808	0.688
4460	215739_s_at	TUBGCP3	0.52591	0.47223	0.05368	0.3
4461	215743_at	NMT2	0.32358	0.41302	-0.08944	0.824
4462	215747_s_at	RCC1	0.46112	0.36179	0.09933	0.168
4463	215749_s_at	GORASP1	0.68831	0.60095	0.08736	0.155
4464	215750_at	KIAA1659	0.72986	0.83763	-0.10777	0.973
4465	215767_at	ZNF804A	0.10293	0.26376	-0.16083	0.925
4466	215779_s_at	HIST1H2BG	0.48758	0.5754	-0.08782	0.876
4467	215780_s_at	hCG_1644608	0.38072	0.34513	0.03559	0.409
4468	215785_s_at	CYFIP2	0.62516	0.56278	0.06238	0.264
4469	215792_s_at	DNAJC11	0.41667	0.36718	0.04949	0.339
4470	215836_s_at	PCDHGA1	0.30208	0.42497	-0.12289	0.854
4471	215854_at	FBXO22	0.51572	0.56194	-0.04622	0.65
4472	215884_s_at	UBQLN2	0.58876	0.47137	0.11739	0.109
4473	215905_s_at	SNRNP40	0.79351	0.8825	-0.08899	0.966
4474	215910_s_at	FNDC3A	0.41026	0.50601	-0.09575	0.944
4475	215919_s_at	MRPS11	0.057897	0.060021	-0.002124	0.524
4476	215925_s_at	CD72	0.69231	0.72334	-0.03103	0.678
4477	215930_s_at	CTAGE5	0.31547	0.37091	-0.05544	0.806
4478	215931_s_at	ARFGEF2	0.42284	0.31496	0.10788	0.241
4479	215938_s_at	PLA2G6	0.1334	0.20575	-0.07235	0.78
4480	215942_s_at	GTSE1	0.86372	0.83609	0.02763	0.176

4481	215947_s_at	FAM136A	0.56711	0.38819	0.17892	0.005
4482	215954_s_at	C19orf29	0.37112	0.15194	0.21918	0.057
4483	215967_s_at	LY9	0.26509	0.40198	-0.13689	0.923
4484	215983_s_at	UBXN8	0.43441	0.61025	-0.17584	0.923
4485	215984_s_at	ARFRP1	0.41213	0.31512	0.09701	0.198
4486	215985_at	NCRNA00171	0.40762	0.42145	-0.01383	0.537
4487	216006_at	RAPGEFL1	0.50628	0.55262	-0.04634	0.701
4488	216020_at	IFIH1	0.46695	0.29697	0.16998	0.066
4489	216026_s_at	POLE	0.33722	0.4583	-0.12108	0.874
4490	216028_at	DKFZP564C152	0.15275	0.24407	-0.09132	0.786
4491	216032_s_at	ERGIC3	0.77924	0.84342	-0.06418	0.884
4492	216060_s_at	DAAM1	0.76303	0.73382	0.02921	0.396
4493	216088_s_at	PSMA7	0.69167	0.68418	0.00749	0.45
4494	216114_at	NCKIPSD	0.15671	0.038364	0.118346	0.226
4495	216177_at	LOC391132	0.55532	0.615	-0.05968	0.792
4496	216194_s_at	TBCB	0.78838	0.85707	-0.06869	0.92
4497	216199_s_at	MAP3K4	0.61439	0.65838	-0.04399	0.701
4498	216218_s_at	PLCL2	0.78095	0.80908	-0.02813	0.7
4499	216226_at	TAF4B	0.59915	0.44908	0.15007	0.071
4500	216231_s_at	B2M	0.82841	0.85829	-0.02988	0.826
4501	216232_s_at	GCN1L1	0.55458	0.64677	-0.09219	0.801
4502	216241_s_at	TCEA1	0.8199	0.81594	0.00396	0.5
4503	216247_at	RPS20	0.24893	0.023841	0.225089	0.051
4504	216248_s_at	NR4A2	0.28886	0.20367	0.08519	0.223
4505	216250_s_at	LPXN	0.28892	0.37507	-0.08615	0.766
4506	216251_s_at	TTLL12	0.47465	0.41012	0.06453	0.128
4507	216262_s_at	TGIF2	0.69253	0.51691	0.17562	0.004
4508	216266_s_at	ARFGEF1	0.61573	0.545	0.07073	0.177
4509	216267_s_at	TMEM115	0.38844	0.16605	0.22239	0.019
4510	216278_at	KIAA0256	0.44665	0.59579	-0.14914	0.967
4511	216288_at	CYSLTR1	0.37224	0.41227	-0.04003	0.633
4512	216305_s_at	C2orf3	0.25795	0.36235	-0.1044	0.815
4513	216326_s_at	HDAC3	0.61933	0.62639	-0.00706	0.554
4514	216338_s_at	YIPF3	0.42602	0.69083	-0.26481	0.994
4515	216347_s_at	PPP1R13B	0.39742	0.46812	-0.0707	0.768
4516	216348_at	RPS17P5	0.71576	0.79518	-0.07942	0.941
4517	216361_s_at	MYST3	0.63367	0.62562	0.00805	0.472
4518	216383_at	RPL18A	0.6623	0.6123	0.05	0.253
4519	216389_s_at	WDR23	0.25364	0.24515	0.00849	0.472
4520	216396_s_at	EI24	0.77645	0.67404	0.10241	0.101
4521	216397_s_at	BOP1	0.75226	0.48292	0.26934	<0.001
4522	216411_s_at	GALK2	0.34837	0.25097	0.0974	0.213
4523	216421_at	GTSF1L	0.24813	0.17508	0.07305	0.319
4524	216438_s_at	TMSB4X	0.87453	0.89482	-0.02029	0.79
4525	216520_s_at	TPT1	0.90514	0.89263	0.01251	0.244
4526	216521_s_at	BRCC3	0.9285	0.91458	0.01392	0.262
4527	216531_at	YY2	0.67469	0.50046	0.17423	0.012
4528	216547_at	LOC127406	0.48663	0.54635	-0.05972	0.745
4529	216555_at	C22orf30	0.84461	0.77484	0.06977	0.123
4530	216563_at	ANKRD12	0.79002	0.83499	-0.04497	0.812
4531	216574_s_at	hCG_2024410	0.6525	0.64513	0.00737	0.47
4532	216591_s_at	hCG_1776980	0.83276	0.87395	-0.04119	0.84
4533	216602_s_at	FARSA	0.80759	0.73687	0.07072	0.037
4534	216607_s_at	CYP51A1	0.7496	0.75009	-0.00049	0.536

4535	216609_at	TXN	0.89693	0.93707	-0.04014	0.989
4536	216627_s_at	B4GALT1	0.23122	0.31978	-0.08856	0.665
4537	216640_s_at	PDIA6	0.58638	0.41749	0.16889	<0.001
4538	216650_at	LOC283412	0.05359	0.13257	-0.07898	0.767
4539	216678_at	IFT122	0.50543	0.38571	0.11972	0.1
4540	216699_s_at	KLK1	0.26475	0.21428	0.05047	0.344
4541	216705_s_at	ADA	0.278	0.32074	-0.04274	0.696
4542	216783_at	LOC283677	0.53477	0.55894	-0.02417	0.615
4543	216806_at	RPSA	0.48316	0.48122	0.00194	0.496
4544	216834_at	RGS1	0.34518	0.52484	-0.17966	0.962
4545	216835_s_at	DOK1	0.5303	0.53363	-0.00333	0.532
4546	216836_s_at	ERBB2	0.32978	0.31751	0.01227	0.453
4547	216860_s_at	GDF11	0.35035	0.26725	0.0831	0.243
4548	216862_s_at	MTCP1NB	0.34084	0.55638	-0.21554	0.942
4549	216863_s_at	MORC2	0.4635	0.28761	0.17589	0.02
4550	216873_s_at	ATP8B2	0.50044	0.39609	0.10435	0.179
4551	216899_s_at	SKAP2	0.74122	0.7342	0.00702	0.476
4552	216902_s_at	LOC653390	0.48341	0.4385	0.04491	0.33
4553	216903_s_at	CBARA1	0.54498	0.52034	0.02464	0.398
4554	216942_s_at	CD58	0.86612	0.87944	-0.01332	0.712
4555	216944_s_at	ITPR1	0.55354	0.67352	-0.11998	0.899
4556	216952_s_at	LMNB2	0.74103	0.7467	-0.00567	0.591
4557	216961_s_at	RPAIN	0.65196	0.36274	0.28922	0.011
4558	216969_s_at	KIF22	0.49955	0.27171	0.22784	0.037
4559	216993_s_at	COL11A2	0.45257	0.35231	0.10026	0.224
4560	216996_s_at	FASTKD2	0.75489	0.53547	0.21942	0.006
4561	217019_at	RPS4X	0.062675	0.13275	-0.070075	0.723
4562	217025_s_at	DBN1	0.44263	0.50142	-0.05879	0.699
4563	217028_at	CXCR4	0.57856	0.6983	-0.11974	0.938
4564	217042_at	RDH11	0.63947	0.43656	0.20291	0.031
4565	217043_s_at	MFN1	0.56037	0.50742	0.05295	0.286
4566	217047_s_at	FAM13A	0.72067	0.79214	-0.07147	0.787
4567	217094_s_at	ITCH	0.63358	0.63192	0.00166	0.488
4568	217100_s_at	UBXN7	0.84811	0.91446	-0.06635	0.964
4569	217104_at	ST20	0.00032826	0.13605	-0.13572174	0.797
4570	217118_s_at	C22orf9	0.41653	0.46916	-0.05263	0.73
4571	217122_s_at	RP11-345P4.4	0.70328	0.65305	0.05023	0.277
4572	217124_at	IQCE	0.088978	0.20155	-0.112572	0.816
4573	217125_at	UBBP2	0.26198	0.28288	-0.0209	0.596
4574	217127_at	CTH	0.59894	0.55887	0.04007	0.344
4575	217144_at	LOC648390	0.70975	0.73393	-0.02418	0.637
4576	217168_s_at	HERPUD1	0.51936	0.34903	0.17033	0.005
4577	217176_s_at	ZFX	0.44596	0.43033	0.01563	0.451
4578	217188_s_at	C14orf1	0.5127	0.64529	-0.13259	0.935
4579	217211_at	ACTBP9	0.17003	0.18582	-0.01579	0.535
4580	217266_at	RPL15P22	0.51651	0.59001	-0.0735	0.812
4581	217286_s_at	NDRG3	0.6202	0.59551	0.02469	0.392
4582	217289_s_at	SLC37A4	0.39145	0.26137	0.13008	0.229
4583	217299_s_at	NBN	0.75879	0.71178	0.04701	0.26
4584	217310_s_at	FOXJ3	0.11078	0.20599	-0.09521	0.766
4585	217317_s_at	HERC2P2	0.43917	0.64138	-0.20221	0.941
4586	217336_at	RPS10	0.44748	0.27015	0.17733	0.023
4587	217340_at	RPL21P68	0.26998	0.12728	0.1427	0.173
4588	217346_at	LOC128192	0.41806	0.38849	0.02957	0.373

4589	217365_at	PRAMEF11	0.21295	0.060606	0.152344	0.111
4590	217368_at	ATP5G2	0.2275	0.17875	0.04875	0.351
4591	217379_at	RPL10	0.6395	0.69941	-0.05991	0.763
4592	217383_at	PGK1	0.45659	0.48309	-0.0265	0.635
4593	217388_s_at	KYNU	0.63723	0.63501	0.00222	0.51
4594	217403_s_at	ZNF227	0.56	0.58119	-0.02119	0.583
4595	217408_at	MRPS18B	0.68969	0.76105	-0.07136	0.829
4596	217427_s_at	HIRA	0.49138	0.21903	0.27235	0.005
4597	217445_s_at	GART	0.76456	0.69598	0.06858	0.13
4598	217448_s_at	LOC285412	0.80337	0.64415	0.15922	0.002
4599	217457_s_at	RAP1GDS1	0.30044	0.35872	-0.05828	0.673
4600	217478_s_at	HLA-DMA	0.6448	0.61007	0.03473	0.319
4601	217494_s_at	PTENP1	0.39173	0.47593	-0.0842	0.764
4602	217496_s_at	IDE	0.49984	0.51569	-0.01585	0.604
4603	217501_at	CIAO1	0.34537	0.32956	0.01581	0.472
4604	217503_at	STK17B	0.64721	0.59932	0.04789	0.293
4605	217504_at	ABCA6	0.55935	0.65787	-0.09852	0.887
4606	217506_at	LOC339290	0.049122	0.25882	-0.209698	0.923
4607	217523_at	CD44	0.5422	0.73978	-0.19758	0.989
4608	217527_s_at	NFATC2IP	0.45454	0.67131	-0.21677	1
4609	217538_at	SGSM2	0.67791	0.61686	0.06105	0.172
4610	217539_at	C18orf25	0.35549	0.24986	0.10563	0.15
4611	217540_at	FAM55C	0.5188	0.38428	0.13452	0.141
4612	217544_at	LOC729806	0.61861	0.52814	0.09047	0.083
4613	217549_at	NCKAP1L	0.75648	0.81423	-0.05775	0.847
4614	217555_at	SMC1A	0.14718	0.0088123	0.1383677	0.088
4615	217559_at	RPL10L	0.314	0.18939	0.12461	0.167
4616	217588_at	CATSPER2	0.20126	0.45798	-0.25672	0.944
4617	217591_at	SKIL	0.70843	0.64476	0.06367	0.476
4618	217599_s_at	MDFIC	0.91617	0.87898	0.03719	0.067
4619	217608_at	SFRS12IP1	0.72019	0.61225	0.10794	0.055
4620	217609_at	LRRC23	0.55377	0.65991	-0.10614	0.838
4621	217612_at	TIMM50	0.23238	0.083527	0.148853	0.132
4622	217627_at	ZNF573	0.23238	0.42567	-0.19329	0.957
4623	217645_at	COX16	0.26956	0.22203	0.04753	0.368
4624	217663_at	ZNF234	0.12019	0.052839	0.067351	0.292
4625	217667_at	LOC729799	0.54461	0.53846	0.00615	0.482
4626	217677_at	PLEKHA2	0.71684	0.56376	0.15308	0.059
4627	217682_at	C16orf72	0.24061	0.24663	-0.00602	0.519
4628	217716_s_at	SEC61A1	0.55085	0.53099	0.01986	0.344
4629	217718_s_at	YWHAB	0.81566	0.77892	0.03674	0.255
4630	217719_at	EIF3L	0.88388	0.90429	-0.02041	0.843
4631	217720_at	CHCHD2	0.80115	0.76342	0.03773	0.306
4632	217722_s_at	NGRN	0.69025	0.66806	0.02219	0.402
4633	217726_at	COPZ1	0.49031	0.59164	-0.10133	0.907
4634	217728_at	S100A6	0.40941	0.50329	-0.09388	0.764
4635	217729_s_at	AES	0.41867	0.55029	-0.13162	0.881
4636	217730_at	TMBIM1	0.48294	0.58151	-0.09857	0.852
4637	217731_s_at	ITM2B	0.63656	0.53235	0.10421	0.137
4638	217733_s_at	TMSB10	0.83675	0.77703	0.05972	0.047
4639	217734_s_at	WDR6	0.75293	0.48177	0.27116	<0.001
4640	217736_s_at	EIF2AK1	0.84236	0.76865	0.07371	0.023
4641	217739_s_at	NAMPT	0.55042	0.57443	-0.02401	0.601
4642	217742_s_at	WAC	0.50435	0.36937	0.13498	0.038

4643	217743_s_at	TMEM30A	0.78764	0.64047	0.14717	0.014
4644	217744_s_at	PERP	0.4544	0.53363	-0.07923	0.714
4645	217745_s_at	NAT13	0.64119	0.61222	0.02897	0.316
4646	217746_s_at	PDCD6IP	0.15999	0.33643	-0.17644	0.925
4647	217747_s_at	RPS9	0.84313	0.88248	-0.03935	0.685
4648	217748_at	ADIPOR1	0.42163	0.28139	0.14024	0.1
4649	217749_at	COPG	0.8319	0.74409	0.08781	<0.001
4650	217750_s_at	UBE2Z	0.76725	0.80158	-0.03433	0.709
4651	217751_at	GSTK1	0.78035	0.66747	0.11288	0.103
4652	217752_s_at	CNDP2	0.4947	0.59856	-0.10386	0.843
4653	217753_s_at	RPS26	0.66385	0.46449	0.19936	0.025
4654	217754_at	DDX56	0.66699	0.45574	0.21125	<0.001
4655	217755_at	HN1	0.75025	0.76075	-0.0105	0.599
4656	217758_s_at	TM9SF3	0.43888	0.37713	0.06175	0.177
4657	217759_at	TRIM44	0.19711	0.29724	-0.10013	0.771
4658	217761_at	ADI1	0.11221	0.30107	-0.18886	0.944
4659	217763_s_at	RAB31	0.43437	0.53469	-0.10032	0.846
4660	217765_at	NRBP1	0.50867	0.37343	0.13524	0.056
4661	217766_s_at	TMEM50A	0.39473	0.43613	-0.0414	0.636
4662	217768_at	C14orf166	0.86248	0.83093	0.03155	0.16
4663	217769_s_at	POMP	0.75088	0.77481	-0.02393	0.693
4664	217770_at	PIGT	0.3672	0.17987	0.18733	0.109
4665	217771_at	GOLM1	0.14898	0.39145	-0.24247	0.944
4666	217772_s_at	MTCH2	0.82763	0.82698	0.00065	0.523
4667	217773_s_at	NDUFA4	0.71407	0.54068	0.17339	0.006
4668	217774_s_at	HSPC152	0.46628	0.57668	-0.1104	0.82
4669	217777_s_at	PTPLAD1	0.85689	0.90146	-0.04457	0.932
4670	217778_at	SLC39A1	0.40547	0.29485	0.11062	0.17
4671	217779_s_at	LOC100132235	0.51351	0.50952	0.00399	0.503
4672	217780_at	C19orf56	0.7291	0.79177	-0.06267	0.805
4673	217781_s_at	ZFP106	0.42534	0.52548	-0.10014	0.841
4674	217782_s_at	GPS1	0.42441	0.27995	0.14446	0.092
4675	217783_s_at	YPEL5	0.81288	0.76268	0.0502	0.129
4676	217784_at	YKT6	0.7478	0.58155	0.16625	<0.001
4677	217786_at	PRMT5	0.77063	0.75307	0.01756	0.417
4678	217788_s_at	GALNT2	0.41722	0.54559	-0.12837	0.873
4679	217789_at	SNX6	0.61623	0.65539	-0.03916	0.707
4680	217790_s_at	SSR3	0.34912	0.4294	-0.08028	0.808
4681	217791_s_at	ALDH18A1	0.4869	0.46753	0.01937	0.345
4682	217792_at	SNX5	0.34988	0.44336	-0.09348	0.79
4683	217794_at	PRR13	0.43669	0.55632	-0.11963	0.845
4684	217795_s_at	TMEM43	0.37264	0.38471	-0.01207	0.55
4685	217796_s_at	NPLOC4	0.4002	0.54626	-0.14606	0.942
4686	217797_at	UFC1	0.80541	0.8132	-0.00779	0.577
4687	217800_s_at	NDFIP1	0.44184	0.65279	-0.21095	0.992
4688	217801_at	ATP5E	0.86934	0.83617	0.03317	0.361
4689	217803_at	GOLPH3	0.64607	0.6326	0.01347	0.443
4690	217805_at	ILF3	0.81066	0.56949	0.24117	<0.001
4691	217806_s_at	POLDIP2	0.5382	0.31807	0.22013	0.012
4692	217807_s_at	GLTSCR2	0.82907	0.85863	-0.02956	0.77
4693	217808_s_at	MAPKAP1	0.63015	0.58102	0.04913	0.272
4694	217809_at	BZW2	0.61412	0.68272	-0.0686	0.832
4695	217811_at	SELT	0.7541	0.87852	-0.12442	0.91
4696	217812_at	YTHDF2	0.84673	0.77433	0.0724	0.105

4697	217813_s_at	SPIN1	0.64923	0.65445	-0.00522	0.546
4698	217814_at	CCDC47	0.65705	0.50518	0.15187	0.066
4699	217815_at	SUPT16H	0.62357	0.75312	-0.12955	0.999
4700	217816_s_at	PCNP	0.44142	0.53527	-0.09385	0.697
4701	217819_at	GOLGA7	0.58088	0.67368	-0.0928	0.823
4702	217822_at	WBP11	0.73407	0.62136	0.11271	0.085
4703	217826_s_at	UBE2J1	0.92057	0.90778	0.01279	0.334
4704	217827_s_at	SPG21	0.45171	0.48765	-0.03594	0.64
4705	217828_at	SLTM	0.35192	0.23239	0.11953	0.148
4706	217829_s_at	USP39	0.47956	0.257	0.22256	0.02
4707	217830_s_at	NSFL1C	0.35899	0.43112	-0.07213	0.739
4708	217833_at	SYNCRIP	0.80577	0.63914	0.16663	<0.001
4709	217836_s_at	YY1AP1	0.32629	0.49009	-0.1638	0.963
4710	217837_s_at	VPS24	0.65672	0.62406	0.03266	0.322
4711	217838_s_at	EVL	0.47055	0.64503	-0.17448	0.928
4712	217840_at	DDX41	0.52508	0.57981	-0.05473	0.713
4713	217841_s_at	PPME1	0.71095	0.63112	0.07983	0.15
4714	217843_s_at	MED4	0.41306	0.62951	-0.21645	0.977
4715	217844_at	CTDSP1	0.6315	0.69373	-0.06223	0.839
4716	217846_at	QARS	0.77486	0.74389	0.03097	0.355
4717	217847_s_at	THRAP3	0.3161	0.57457	-0.25847	0.993
4718	217848_s_at	PPA1	0.77928	0.73078	0.0485	0.262
4719	217849_s_at	CDC42BPB	0.37555	0.33068	0.04487	0.372
4720	217850_at	GNL3	0.6741	0.35531	0.31879	0.003
4721	217851_s_at	SLMO2	0.786	0.77601	0.00999	0.436
4722	217852_s_at	ARL8B	0.65005	0.73885	-0.0888	0.825
4723	217853_at	TNS3	0.011754	0.24069	-0.228936	0.999
4724	217858_s_at	ARMCX3	0.70176	0.46559	0.23617	<0.001
4725	217860_at	LOC732160	0.29398	0.36416	-0.07018	0.753
4726	217861_s_at	PREB	0.67247	0.54636	0.12611	<0.001
4727	217862_at	PIAS1	0.91783	0.91881	-0.00098	0.536
4728	217866_at	CPSF7	0.16005	0.13764	0.02241	0.417
4729	217868_s_at	METTL9	0.79884	0.82592	-0.02708	0.703
4730	217869_at	HSD17B12	0.34815	0.48506	-0.13691	0.831
4731	217870_s_at	CMPK1	0.57632	0.60033	-0.02401	0.619
4732	217871_s_at	MIF	0.88404	0.86074	0.0233	0.359
4733	217872_at	PIH1D1	0.68479	0.80913	-0.12434	0.981
4734	217873_at	CAB39	0.23933	0.42012	-0.18079	0.92
4735	217874_at	SUCLG1	0.70418	0.6141	0.09008	0.119
4736	217876_at	GTF3C5	0.36933	0.30416	0.06517	0.248
4737	217877_s_at	GPBP1L1	0.47696	0.35781	0.11915	0.056
4738	217878_s_at	CDC27	0.86505	0.90705	-0.042	0.92
4739	217882_at	TMEM111	0.79716	0.81441	-0.01725	0.613
4740	217883_at	MMADHC	0.81291	0.83417	-0.02126	0.736
4741	217884_at	NAT10	0.25111	0.38634	-0.13523	0.93
4742	217885_at	IPO9	0.62953	0.48242	0.14711	0.07
4743	217886_at	EPS15	0.43035	0.39023	0.04012	0.346
4744	217888_s_at	ARFGAP1	0.44093	0.38507	0.05586	0.263
4745	217889_s_at	CYBRD1	0.46137	0.49607	-0.0347	0.691
4746	217892_s_at	LIMA1	0.49109	0.51855	-0.02746	0.607
4747	217893_s_at	AKIRIN1	0.51924	0.68846	-0.16922	0.956
4748	217894_at	KCTD3	0.34788	0.29966	0.04822	0.342
4749	217895_at	PTCD3	0.69986	0.70203	-0.00217	0.517
4750	217896_s_at	NIP30	0.47694	0.55713	-0.08019	0.805

4751	217898_at	C15orf24	0.54377	0.67991	-0.13614	0.915
4752	217899_at	TMEM214	0.5008	0.39513	0.10567	0.069
4753	217900_at	IARS2	0.5344	0.6104	-0.076	0.838
4754	217901_at	DSG2	0.21918	0.39106	-0.17188	0.854
4755	217902_s_at	HERC2	0.30935	0.45629	-0.14694	0.881
4756	217903_at	STRN4	0.72282	0.59215	0.13067	0.022
4757	217905_at	C10orf119	0.051736	0.097159	-0.045423	0.684
4758	217906_at	KLHDC2	0.52518	0.62801	-0.10283	0.952
4759	217907_at	MRPL18	0.61432	0.50946	0.10486	0.043
4760	217908_s_at	IQWD1	0.64485	0.55272	0.09213	0.132
4761	217911_s_at	BAG3	0.84783	0.83209	0.01574	0.357
4762	217912_at	DUS1L	0.60428	0.74439	-0.14011	0.948
4763	217913_at	VPS4A	0.46443	0.30008	0.16435	0.072
4764	217914_at	TPCN1	0.4072	0.53371	-0.12651	0.9
4765	217915_s_at	RSL24D1	0.66393	0.6711	-0.00717	0.518
4766	217918_at	DYNLRB1	0.61144	0.65851	-0.04707	0.742
4767	217919_s_at	MRPL42	0.6619	0.67289	-0.01099	0.568
4768	217923_at	PEF1	0.77457	0.73411	0.04046	0.317
4769	217924_at	C6orf106	0.6394	0.66314	-0.02374	0.621
4770	217926_at	C19orf53	0.39647	0.50823	-0.11176	0.822
4771	217927_at	SPCS1	0.82514	0.83873	-0.01359	0.657
4772	217928_s_at	SAPS3	0.36449	0.42254	-0.05805	0.726
4773	217929_s_at	KIAA0319L	0.41406	0.47163	-0.05757	0.732
4774	217930_s_at	TOLLIP	0.50489	0.48035	0.02454	0.429
4775	217931_at	CNPY3	0.59183	0.61835	-0.02652	0.585
4776	217932_at	MRPS7	0.59615	0.76695	-0.1708	0.985
4777	217933_s_at	LAP3	0.71864	0.56995	0.14869	0.001
4778	217935_s_at	UQCC	0.24111	0.030114	0.210996	0.058
4779	217936_at	ARHGAP5	0.56385	0.58762	-0.02377	0.613
4780	217938_s_at	KCMF1	0.67725	0.70297	-0.02572	0.651
4781	217939_s_at	AFTPH	0.61392	0.60091	0.01301	0.412
4782	217940_s_at	CARKD	0.20778	0.31202	-0.10424	0.702
4783	217941_s_at	ERBB2IP	0.7743	0.70782	0.06648	0.183
4784	217942_at	MRPS35	0.6865	0.64834	0.03816	0.32
4785	217943_s_at	MAP7D1	0.12711	0.33651	-0.2094	0.945
4786	217944_at	POMGNT1	0.13838	0.042823	0.095557	0.265
4787	217945_at	BTBD1	0.46203	0.46596	-0.00393	0.514
4788	217946_s_at	SAE1	0.66468	0.51162	0.15306	0.032
4789	217947_at	CMTM6	0.59844	0.659	-0.06056	0.791
4790	217949_s_at	VKORC1	0.64673	0.68853	-0.0418	0.75
4791	217950_at	NOSIP	0.78446	0.71428	0.07018	0.071
4792	217955_at	BCL2L13	0.53734	0.50253	0.03481	0.359
4793	217956_s_at	ENOPH1	0.83098	0.75735	0.07363	0.201
4794	217957_at	C16orf80	0.73701	0.77229	-0.03528	0.724
4795	217959_s_at	TRAPPC4	0.30551	0.48108	-0.17557	0.919
4796	217960_s_at	TOMM22	0.8799	0.88359	-0.00369	0.566
4797	217961_at	SLC25A38	0.45374	0.53056	-0.07682	0.742
4798	217962_at	NOP10	0.84373	0.81731	0.02642	0.412
4799	217963_s_at	NGFRAP1	0.049845	0.31034	-0.260495	0.961
4800	217964_at	TTC19	0.18721	0.46362	-0.27641	0.993
4801	217965_s_at	SAP30BP	0.72764	0.74956	-0.02192	0.644
4802	217966_s_at	FAM129A	0.28747	0.49231	-0.20484	0.947
4803	217968_at	TSSC1	0.17153	0.35931	-0.18778	0.931
4804	217969_at	C11orf2	0.78736	0.7409	0.04646	0.118

4805	217970_s_at	CNOT6	0.51481	0.33011	0.1847	0.046
4806	217971_at	MAPKSP1	0.57176	0.5692	0.00256	0.502
4807	217972_at	CHCHD3	0.82611	0.80213	0.02398	0.234
4808	217973_at	DCXR	0.3793	0.55431	-0.17501	0.938
4809	217974_at	TM7SF3	0.56519	0.62593	-0.06074	0.739
4810	217975_at	WBP5	0.23614	0.44397	-0.20783	0.965
4811	217976_s_at	DYNC1LI1	0.76926	0.77618	-0.00692	0.589
4812	217977_at	SEPX1	0.43762	0.37391	0.06371	0.283
4813	217978_s_at	UBE2Q1	0.7296	0.76359	-0.03399	0.721
4814	217980_s_at	MRPL16	0.7034	0.72482	-0.02142	0.643
4815	217981_s_at	FXC1	0.10748	0.13439	-0.02691	0.577
4816	217982_s_at	MORF4L1	0.64985	0.67267	-0.02282	0.603
4817	217984_at	RNASET2	0.40449	0.63221	-0.22772	0.981
4818	217986_s_at	BAZ1A	0.72801	0.51143	0.21658	<0.001
4819	217987_at	ASNSD1	0.45119	0.22329	0.2279	0.034
4820	217988_at	CCNB1IP1	0.59621	0.39316	0.20305	0.011
4821	217989_at	HSD17B11	0.018811	0.17584	-0.157029	0.988
4822	217990_at	GMPR2	0.50049	0.56564	-0.06515	0.741
4823	217992_s_at	EFHD2	0.42498	0.35953	0.06545	0.195
4824	217993_s_at	MAT2B	0.59714	0.53601	0.06113	0.248
4825	217995_at	SQRDL	0.55452	0.64834	-0.09382	0.827
4826	217997_at	PHLDA1	0.72325	0.6734	0.04985	0.3
4827	218001_at	MRPS2	0.59899	0.51366	0.08533	0.107
4828	218003_s_at	FKBP3	0.87785	0.7551	0.12275	<0.001
4829	218005_at	ZNF22	0.58194	0.66446	-0.08252	0.813
4830	218007_s_at	RPS27L	0.53309	0.70671	-0.17362	0.972
4831	218008_at	C7orf42	0.47879	0.5505	-0.07171	0.804
4832	218009_s_at	PRC1	0.59761	0.71883	-0.12122	0.952
4833	218011_at	UBL5	0.82608	0.64018	0.1859	<0.001
4834	218012_at	TSPYL2	0.34572	0.11449	0.23123	0.003
4835	218014_at	NUP85	0.82115	0.62095	0.2002	<0.001
4836	218016_s_at	POLR3E	0.57185	0.44205	0.1298	0.015
4837	218017_s_at	HGSNAT	0.62477	0.7042	-0.07943	0.875
4838	218018_at	PDXK	0.39289	0.25666	0.13623	0.086
4839	218020_s_at	ZFAND3	0.74395	0.49357	0.25038	<0.001
4840	218021_at	DHRS4	0.047659	0.31661	-0.268951	0.939
4841	218022_at	VRK3	0.31507	0.56072	-0.24565	0.994
4842	218023_s_at	FAM53C	0.11582	0.081729	0.034091	0.386
4843	218024_at	BRP44L	0.46862	0.48482	-0.0162	0.555
4844	218025_s_at	PECI	0.11095	0.19472	-0.08377	0.74
4845	218026_at	CCDC56	0.6561	0.68826	-0.03216	0.614
4846	218027_at	MRPL15	0.89391	0.89678	-0.00287	0.544
4847	218030_at	GIT1	0.15877	0.16221	-0.00344	0.508
4848	218032_at	SNN	0.77629	0.72994	0.04635	0.309
4849	218034_at	FIS1	0.74395	0.79971	-0.05576	0.862
4850	218035_s_at	RBM47	0.41169	0.55275	-0.14106	0.934
4851	218039_at	NUSAP1	0.65592	0.44088	0.21504	0.056
4852	218040_at	PRPF38B	0.64217	0.60802	0.03415	0.35
4853	218042_at	COPS4	0.73807	0.72283	0.01524	0.445
4854	218043_s_at	AZI2	0.79718	0.82245	-0.02527	0.67
4855	218046_s_at	MRPS16	0.4609	0.70148	-0.24058	0.989
4856	218047_at	OSBPL9	0.44958	0.56239	-0.11281	0.884
4857	218048_at	COMMD3	0.70094	0.77465	-0.07371	0.876
4858	218049_s_at	MRPL13	0.8234	0.8753	-0.0519	0.864

4859	218050_at	UFM1	0.57294	0.61836	-0.04542	0.805
4860	218051_s_at	NT5DC2	0.37081	0.39143	-0.02062	0.597
4861	218052_s_at	ATP13A1	0.66756	0.55391	0.11365	0.09
4862	218055_s_at	WDR41	0.027855	0.31881	-0.290955	0.995
4863	218056_at	BFAR	0.41676	0.42158	-0.00482	0.527
4864	218058_at	CXXC1	0.71043	0.73844	-0.02801	0.655
4865	218059_at	ZNF706	0.82739	0.8508	-0.02341	0.698
4866	218060_s_at	C16orf57	0.14155	0.049314	0.092236	0.154
4867	218061_at	MEA1	0.8863	0.82881	0.05749	0.085
4868	218065_s_at	TMEM9B	0.53168	0.54361	-0.01193	0.563
4869	218066_at	SLC12A7	0.31679	0.44653	-0.12974	0.895
4870	218067_s_at	ARGLU1	0.26235	0.30965	-0.0473	0.651
4871	218068_s_at	ZNF672	0.44647	0.36274	0.08373	0.222
4872	218069_at	DCTPP1	0.76678	0.54753	0.21925	<0.001
4873	218070_s_at	GMPPA	0.61029	0.55677	0.05352	0.22
4874	218071_s_at	MKRN2	0.78945	0.78025	0.0092	0.393
4875	218072_at	COMMD9	0.25141	0.4385	-0.18709	0.964
4876	218073_s_at	TMEM48	0.84374	0.79174	0.052	0.197
4877	218074_at	FAM96B	0.82471	0.8429	-0.01819	0.681
4878	218075_at	AAAS	0.35963	0.48604	-0.12641	0.889
4879	218076_s_at	ARHGAP17	0.3518	0.59306	-0.24126	0.951
4880	218077_s_at	ZDHHC3	0.45648	0.34597	0.11051	0.182
4881	218079_s_at	GGNBP2	0.57843	0.28715	0.29128	0.004
4882	218081_at	C20orf27	0.3559	0.26949	0.08641	0.235
4883	218082_s_at	UBP1	0.77122	0.57153	0.19969	0.001
4884	218083_at	PTGES2	0.43161	0.44233	-0.01072	0.567
4885	218085_at	CHMP5	0.69048	0.74007	-0.04959	0.81
4886	218088_s_at	RRAGC	0.78541	0.83044	-0.04503	0.829
4887	218089_at	C20orf4	0.13851	0.048879	0.089631	0.272
4888	218090_s_at	BRWD2	0.66118	0.6445	0.01668	0.39
4889	218093_s_at	ANKRD10	0.60798	0.72967	-0.12169	0.932
4890	218095_s_at	TMEM165	0.64163	0.75443	-0.1128	0.898
4891	218096_at	AGPAT5	0.81738	0.85417	-0.03679	0.76
4892	218097_s_at	CUEDC2	0.72878	0.79051	-0.06173	0.798
4893	218099_at	TEX2	0.27177	0.29574	-0.02397	0.62
4894	218100_s_at	IFT57	0.21168	0.39207	-0.18039	0.933
4895	218101_s_at	NDUFC2	0.73488	0.80593	-0.07105	0.826
4896	218102_at	DERA	0.60748	0.63171	-0.02423	0.629
4897	218103_at	FTSJ3	0.54403	0.59769	-0.05366	0.731
4898	218104_at	TEX10	0.5364	0.45809	0.07831	0.165
4899	218105_s_at	MRPL4	0.89472	0.8675	0.02722	0.154
4900	218106_s_at	MRPS10	0.30445	0.51461	-0.21016	0.955
4901	218107_at	WDR26	0.25195	0.39804	-0.14609	0.95
4902	218108_at	UBR7	0.59868	0.6322	-0.03352	0.673
4903	218109_s_at	MFSD1	0.49375	0.43067	0.06308	0.272
4904	218110_at	XAB2	0.70081	0.67014	0.03067	0.372
4905	218111_s_at	CMAS	0.23553	0.48837	-0.25284	0.947
4906	218112_at	MRPS34	0.60077	0.63097	-0.0302	0.668
4907	218115_at	ASF1B	0.71208	0.72484	-0.01276	0.667
4908	218116_at	C9orf78	0.4397	0.22014	0.21956	0.01
4909	218117_at	RBX1	0.64668	0.72652	-0.07984	0.927
4910	218118_s_at	TIMM23	0.75219	0.7074	0.04479	0.304
4911	218121_at	HMOX2	0.40142	0.12517	0.27625	<0.001
4912	218122_s_at	SENP2	0.74227	0.69806	0.04421	0.263

4913	218123_at	C21orf59	0.47385	0.46752	0.00633	0.467
4914	218124_at	RETSAT	0.32985	0.48356	-0.15371	0.894
4915	218125_s_at	CCDC25	0.28492	0.18116	0.10376	0.143
4916	218126_at	FAM82A2	0.1915	0.32433	-0.13283	0.881
4917	218129_s_at	NFYB	0.47201	0.45633	0.01568	0.465
4918	218130_at	C17orf62	0.47603	0.60223	-0.1262	0.886
4919	218131_s_at	GATAD2A	0.76703	0.57709	0.18994	0.007
4920	218132_s_at	TSEN34	0.71865	0.55795	0.1607	0.024
4921	218133_s_at	NIF3L1	0.67283	0.76227	-0.08944	0.897
4922	218134_s_at	RBM22	0.7181	0.46264	0.25546	<0.001
4923	218135_at	ERGIC2	0.57007	0.65493	-0.08486	0.833
4924	218137_s_at	SMAP1	0.1508	0.47772	-0.32692	1
4925	218138_at	MKKS	0.39558	0.50422	-0.10864	0.859
4926	218139_s_at	MUDENG	0.59342	0.71338	-0.11996	0.926
4927	218141_at	UBE2O	0.60428	0.59458	0.0097	0.45
4928	218142_s_at	CRBN	0.70573	0.52294	0.18279	0.014
4929	218143_s_at	SCAMP2	0.72896	0.70186	0.0271	0.413
4930	218144_s_at	INF2	0.29023	0.37003	-0.0798	0.76
4931	218145_at	TRIB3	0.50748	0.36306	0.14442	0.084
4932	218147_s_at	GLT8D1	0.4596	0.56376	-0.10416	0.863
4933	218148_at	CENPT	0.49054	0.56168	-0.07114	0.73
4934	218149_s_at	ZNF395	0.62228	0.60686	0.01542	0.451
4935	218150_at	ARL5A	0.77784	0.82105	-0.04321	0.7
4936	218152_at	HMG20A	0.2017	0.43818	-0.23648	0.969
4937	218153_at	CARS2	0.49718	0.24881	0.24837	<0.001
4938	218154_at	GSDMD	0.32348	0.22625	0.09723	0.258
4939	218158_s_at	APPL1	0.063313	0.19061	-0.127297	0.916
4940	218159_at	DDRGK1	0.048829	0.058159	-0.00933	0.552
4941	218160_at	NDUFA8	0.53659	0.70772	-0.17113	0.944
4942	218161_s_at	CLN6	0.56772	0.4212	0.14652	0.055
4943	218163_at	MCTS1	0.78167	0.81611	-0.03444	0.725
4944	218164_at	SPATA20	0.026333	0.12388	-0.097547	0.828
4945	218165_at	C1orf149	0.41635	0.29722	0.11913	0.214
4946	218166_s_at	RSF1	0.78954	0.73895	0.05059	0.251
4947	218167_at	AMZ2	0.45886	0.55232	-0.09346	0.892
4948	218168_s_at	CABC1	0.52096	0.45581	0.06515	0.27
4949	218170_at	ISOC1	0.79578	0.78938	0.0064	0.469
4950	218171_at	VPS4B	0.78347	0.7249	0.05857	0.174
4951	218172_s_at	DERL1	0.41458	0.35983	0.05475	0.252
4952	218174_s_at	C10orf57	0.24479	0.37689	-0.1321	0.904
4953	218175_at	CCDC92	0.38967	0.54737	-0.1577	0.795
4954	218176_at	MAGEF1	0.28086	0.32796	-0.0471	0.653
4955	218178_s_at	CHMP1B	0.53365	0.50212	0.03153	0.369
4956	218179_s_at	C4orf41	0.54193	0.66142	-0.11949	0.91
4957	218184_at	TULP4	0.30423	0.35144	-0.04721	0.646
4958	218185_s_at	ARMC1	0.87517	0.8012	0.07397	0.052
4959	218187_s_at	C8orf33	0.45525	0.49838	-0.04313	0.679
4960	218188_s_at	TIMM13	0.51316	0.50932	0.00384	0.515
4961	218189_s_at	NANS	0.67664	0.5867	0.08994	0.161
4962	218190_s_at	UCRC	0.73903	0.79842	-0.05939	0.897
4963	218191_s_at	LMBRD1	0.32341	0.35614	-0.03273	0.654
4964	218192_at	IP6K2	0.72002	0.68191	0.03811	0.323
4965	218193_s_at	GOLT1B	0.62176	0.54128	0.08048	0.037
4966	218194_at	REXO2	0.32301	0.64034	-0.31733	0.991

4967	218195_at	C6orf211	0.82819	0.87232	-0.04413	0.868
4968	218196_at	OSTM1	0.4082	0.5915	-0.1833	0.981
4969	218197_s_at	OXR1	0.82479	0.75918	0.06561	0.067
4970	218198_at	DHX32	0.65356	0.65596	-0.0024	0.538
4971	218199_s_at	NOL6	0.23803	0.075772	0.162258	0.022
4972	218201_at	NDUFB2	0.16907	0.26647	-0.0974	0.875
4973	218203_at	ALG5	0.70765	0.73828	-0.03063	0.661
4974	218204_s_at	FYCO1	0.25901	0.34317	-0.08416	0.764
4975	218205_s_at	MKNK2	0.5837	0.55641	0.02729	0.38
4976	218208_at	LOC100131178	0.46791	0.52463	-0.05672	0.702
4977	218209_s_at	RPRD1A	0.61541	0.58052	0.03489	0.333
4978	218210_at	FN3KRP	0.35297	0.31117	0.0418	0.324
4979	218211_s_at	MLPH	0.12133	0.044115	0.077215	0.276
4980	218212_s_at	MOCS2	0.40127	0.31155	0.08972	0.274
4981	218213_s_at	C11orf10	0.74873	0.77425	-0.02552	0.642
4982	218214_at	C12orf44	0.42772	0.57753	-0.14981	0.916
4983	218215_s_at	NR1H2	0.39354	0.48284	-0.0893	0.729
4984	218217_at	SCPEP1	0.30033	0.36083	-0.0605	0.676
4985	218218_at	APPL2	0.61826	0.63841	-0.02015	0.616
4986	218219_s_at	LANCL2	0.48606	0.58628	-0.10022	0.801
4987	218220_at	C12orf10	0.73378	0.69281	0.04097	0.199
4988	218221_at	ARNT	0.22834	0.16924	0.0591	0.309
4989	218223_s_at	PLEKHO1	0.4942	0.51242	-0.01822	0.588
4990	218224_at	PNMA1	0.40424	0.50602	-0.10178	0.766
4991	218225_at	ECSIT	0.51873	0.61769	-0.09896	0.818
4992	218226_s_at	NDUFB4	0.76	0.62731	0.13269	0.019
4993	218227_at	NUBP2	0.014835	0.12096	-0.106125	0.78
4994	218228_s_at	TNKS2	0.62904	0.624	0.00504	0.463
4995	218229_s_at	POGK	0.55405	0.64497	-0.09092	0.809
4996	218230_at	ARFIP1	0.39285	0.35166	0.04119	0.374
4997	218231_at	NAGK	0.51664	0.49175	0.02489	0.395
4998	218233_s_at	PRICKLE4	0.88176	0.67726	0.2045	<0.001
4999	218235_s_at	UTP11L	0.64436	0.78096	-0.1366	0.977
5000	218236_s_at	PRKD3	0.17501	0.35827	-0.18326	0.967
5001	218237_s_at	SLC38A1	0.69802	0.67334	0.02468	0.4
5002	218239_s_at	GTPBP4	0.75197	0.66499	0.08698	<0.001
5003	218241_at	GOLGA5	0.6703	0.64721	0.02309	0.33
5004	218242_s_at	SUV420H1	0.63153	0.64637	-0.01484	0.568
5005	218243_at	RUFY1	0.2074	0.2083	-0.0009	0.505
5006	218244_at	NOL8	0.65148	0.6338	0.01768	0.414
5007	218247_s_at	MEX3C	0.73192	0.77457	-0.04265	0.746
5008	218248_at	FAM111A	0.29196	0.4028	-0.11084	0.845
5009	218249_at	ZDHHC6	0.49817	0.40919	0.08898	0.112
5010	218250_s_at	CNOT7	0.44865	0.42396	0.02469	0.415
5011	218251_at	MID1IP1	0.48992	0.55322	-0.0633	0.767
5012	218252_at	CKAP2	0.55917	0.34842	0.21075	0.001
5013	218253_s_at	LGTN	0.83641	0.82592	0.01049	0.424
5014	218254_s_at	SAR1B	0.78181	0.80791	-0.0261	0.691
5015	218255_s_at	FBRS	0.23329	0.30504	-0.07175	0.696
5016	218257_s_at	UGCGL1	0.56174	0.65236	-0.09062	0.838
5017	218258_at	POLR1D	0.42231	0.41557	0.00674	0.481
5018	218259_at	MKL2	0.52579	0.58081	-0.05502	0.742
5019	218260_at	DDA1	0.67481	0.7239	-0.04909	0.802
5020	218263_s_at	ZBED5	0.48834	0.56259	-0.07425	0.757

5021	218264_at	BCCIP	0.45157	0.35838	0.09319	0.228
5022	218265_at	SECISBP2	0.44476	0.58372	-0.13896	0.961
5023	218267_at	CINP	0.061964	0.1863	-0.124336	0.887
5024	218268_at	TBC1D15	0.10649	0.25389	-0.1474	0.905
5025	218269_at	RNASEN	0.4959	0.3813	0.1146	0.142
5026	218270_at	MRPL24	0.14139	0.12897	0.01242	0.48
5027	218271_s_at	PARL	0.69931	0.73268	-0.03337	0.752
5028	218272_at	TTC38	0.23076	0.26443	-0.03367	0.611
5029	218273_s_at	PPM2C	0.75376	0.81748	-0.06372	0.86
5030	218274_s_at	ANKZF1	0.35137	0.26267	0.0887	0.231
5031	218275_at	SLC25A10	0.54541	0.45943	0.08598	0.133
5032	218276_s_at	SAV1	0.64838	0.76604	-0.11766	0.826
5033	218277_s_at	DHX40	0.38057	0.46402	-0.08345	0.913
5034	218278_at	LOC649169	0.5512	0.54079	0.01041	0.464
5035	218281_at	MRPL48	0.57485	0.72846	-0.15361	0.949
5036	218282_at	EDEM2	0.52423	0.46815	0.05608	0.097
5037	218283_at	SS18L2	0.42172	0.40894	0.01278	0.442
5038	218284_at	SMAD3	0.10411	0.38767	-0.28356	0.999
5039	218285_s_at	BDH2	0.47578	0.49438	-0.0186	0.554
5040	218286_s_at	RNF7	0.23383	0.28445	-0.05062	0.681
5041	218287_s_at	EIF2C1	0.44914	0.30989	0.13925	0.104
5042	218288_s_at	CCDC90B	0.76892	0.72803	0.04089	0.107
5043	218289_s_at	UBA5	0.46652	0.48587	-0.01935	0.645
5044	218290_at	PLEKHJ1	0.63317	0.56319	0.06998	0.156
5045	218291_at	ROBLD3	0.75925	0.77646	-0.01721	0.632
5046	218294_s_at	NUP50	0.67037	0.48019	0.19018	0.015
5047	218297_at	C10orf97	0.047972	0.047676	0.000296	0.497
5048	218298_s_at	C14orf159	0.23343	0.29275	-0.05932	0.692
5049	218300_at	C16orf53	0.68654	0.69477	-0.00823	0.526
5050	218301_at	RNPEPL1	0.1689	0.33796	-0.16906	0.908
5051	218302_at	PSENEN	0.6163	0.64497	-0.02867	0.659
5052	218304_s_at	OSBPL11	0.55159	0.49993	0.05166	0.269
5053	218305_at	IPO4	0.57622	0.49773	0.07849	0.172
5054	218306_s_at	HERC1	0.80196	0.76675	0.03521	0.3
5055	218307_at	RSAD1	0.64102	0.61139	0.02963	0.343
5056	218308_at	TACC3	0.75014	0.59562	0.15452	0.002
5057	218310_at	RABGEF1	0.32632	0.40788	-0.08156	0.77
5058	218311_at	MAP4K3	0.65691	0.5238	0.13311	0.323
5059	218314_s_at	C11orf57	0.16782	0.072975	0.094845	0.204
5060	218315_s_at	CDK5RAP1	0.62567	0.57765	0.04802	0.269
5061	218316_at	TIMM9	0.71608	0.60867	0.10741	0.074
5062	218318_s_at	NLK	0.84492	0.75467	0.09025	0.028
5063	218319_at	PELI1	0.58682	0.73029	-0.14347	0.996
5064	218320_s_at	NDUFB11	0.76726	0.84145	-0.07419	0.965
5065	218322_s_at	ACSL5	0.70923	0.69458	0.01465	0.449
5066	218324_s_at	SPATS2	0.84395	0.78069	0.06326	0.029
5067	218326_s_at	LGR4	0.040912	0.15333	-0.112418	0.799
5068	218327_s_at	SNAP29	0.044211	0.10404	-0.059829	0.729
5069	218328_at	COQ4	0.52761	0.57859	-0.05098	0.701
5070	218330_s_at	NAV2	0.58533	0.7205	-0.13517	0.938
5071	218331_s_at	C10orf18	0.62559	0.47791	0.14768	0.019
5072	218333_at	DERL2	0.7676	0.7343	0.0333	0.355
5073	218334_at	THOC7	0.8083	0.84095	-0.03265	0.679
5074	218336_at	PFDN2	0.85029	0.83256	0.01773	0.394

5075	218337_at	FAM160B2	0.12197	0.28698	-0.16501	0.912
5076	218339_at	MRPL22	0.81434	0.81078	0.00356	0.508
5077	218340_s_at	UBA6	0.19818	0.32996	-0.13178	0.848
5078	218341_at	PPCS	0.43274	0.37744	0.0553	0.328
5079	218342_s_at	ERMP1	0.48919	0.35489	0.1343	0.06
5080	218343_s_at	GTF3C3	0.3776	0.34592	0.03168	0.387
5081	218344_s_at	RCOR3	0.31589	0.30181	0.01408	0.468
5082	218346_s_at	SESN1	0.70753	0.70596	0.00157	0.498
5083	218347_at	TYW1	0.50453	0.4081	0.09643	0.2
5084	218348_s_at	ZC3H7A	0.59665	0.52744	0.06921	0.187
5085	218349_s_at	ZWILCH	0.91753	0.88013	0.0374	0.02
5086	218350_s_at	GMNN	0.81119	0.7226	0.08859	0.002
5087	218351_at	COMMD8	0.49669	0.75729	-0.2606	0.911
5088	218352_at	RCBTB1	0.59661	0.62393	-0.02732	0.631
5089	218354_at	TRAPPC2L	0.59511	0.56315	0.03196	0.343
5090	218355_at	KIF4A	0.62561	0.68643	-0.06082	0.737
5091	218357_s_at	TIMM8B	0.81934	0.70802	0.11132	0.097
5092	218358_at	CRELD2	0.48848	0.45146	0.03702	0.345
5093	218361_at	GOLPH3L	0.53714	0.46872	0.06842	0.03
5094	218362_s_at	DIS3	0.33705	0.53271	-0.19566	0.938
5095	218363_at	EXD2	0.19145	0.16483	0.02662	0.381
5096	218364_at	LRRFIP2	0.23818	0.34611	-0.10793	0.818
5097	218365_s_at	DARS2	0.47089	0.29022	0.18067	0.064
5098	218370_s_at	S100PBP	0.52626	0.43094	0.09532	0.202
5099	218372_at	MED9	0.18492	0.081174	0.103746	0.25
5100	218373_at	AKTIP	0.58469	0.36604	0.21865	0.001
5101	218374_s_at	C12orf4	0.54777	0.62155	-0.07378	0.736
5102	218375_at	NUDT9	0.54457	0.59681	-0.05224	0.706
5103	218376_s_at	MICAL1	0.57074	0.71844	-0.1477	0.972
5104	218377_s_at	RWDD2B	0.38894	0.40265	-0.01371	0.553
5105	218378_s_at	PRKRIP1	0.62023	0.63573	-0.0155	0.579
5106	218379_at	RBM7	0.38236	0.36207	0.02029	0.445
5107	218381_s_at	U2AF2	0.55652	0.58705	-0.03053	0.629
5108	218383_at	HAUS4	0.69661	0.77555	-0.07894	0.807
5109	218384_at	CARHSP1	0.88093	0.8619	0.01903	0.318
5110	218385_at	MRPS18A	0.72757	0.68256	0.04501	0.238
5111	218388_at	PGLS	0.52693	0.53972	-0.01279	0.544
5112	218389_s_at	APH1A	0.65701	0.77207	-0.11506	0.948
5113	218390_s_at	C10orf84	0.18169	0.079836	0.101854	0.167
5114	218391_at	SNF8	0.64013	0.54789	0.09224	0.181
5115	218393_s_at	SMU1	0.14978	0.37278	-0.223	0.957
5116	218394_at	ROGDI	0.39487	0.31251	0.08236	0.256
5117	218395_at	ACTR6	0.54124	0.52992	0.01132	0.469
5118	218396_at	VPS13C	0.44434	0.57307	-0.12873	0.935
5119	218397_at	FANCL	0.77991	0.80169	-0.02178	0.728
5120	218399_s_at	CDCA4	0.6409	0.54795	0.09295	0.043
5121	218400_at	OAS3	0.74262	0.75139	-0.00877	0.561
5122	218401_s_at	ZNF281	0.44321	0.47882	-0.03561	0.626
5123	218403_at	TRIAP1	0.86825	0.766	0.10225	0.022
5124	218404_at	SNX10	0.64449	0.64771	-0.00322	0.547
5125	218405_at	ABT1	0.72817	0.69271	0.03546	0.369
5126	218408_at	TIMM10	0.83281	0.80627	0.02654	0.352
5127	218409_s_at	DNAJC1	0.31934	0.21272	0.10662	0.068
5128	218411_s_at	MBIP	0.6536	0.66195	-0.00835	0.575

5129	218412_s_at	GTF2IRD1	0.17841	0.35043	-0.17202	0.924
5130	218414_s_at	NDE1	0.61107	0.61451	-0.00344	0.54
5131	218415_at	VPS33B	0.38523	0.49383	-0.1086	0.847
5132	218419_s_at	TMUB2	0.21711	0.36791	-0.1508	0.942
5133	218420_s_at	C13orf23	0.29282	0.15009	0.14273	0.069
5134	218421_at	CERK	0.39358	0.48373	-0.09015	0.846
5135	218422_s_at	RBM26	0.51187	0.35344	0.15843	0.112
5136	218424_s_at	STEAP3	0.43913	0.3602	0.07893	0.268
5137	218426_s_at	RNF216	0.59368	0.39024	0.20344	0.021
5138	218427_at	SDCCAG3	0.048125	0.12912	-0.080995	0.769
5139	218428_s_at	REV1	0.36063	0.32709	0.03354	0.395
5140	218429_s_at	C19orf66	0.74033	0.64778	0.09255	0.102
5141	218430_s_at	RFX7	0.86359	0.85956	0.00403	0.465
5142	218431_at	C14orf133	0.65728	0.56171	0.09557	0.023
5143	218432_at	FBXO3	0.5939	0.61465	-0.02075	0.617
5144	218433_at	PANK3	0.20493	0.22837	-0.02344	0.591
5145	218434_s_at	AACS	0.38592	0.40667	-0.02075	0.591
5146	218435_at	DNAJC15	0.25808	0.37629	-0.11821	0.866
5147	218436_at	SIL1	0.42594	0.39472	0.03122	0.345
5148	218437_s_at	LZTFL1	0.68223	0.69017	-0.00794	0.553
5149	218439_s_at	COMMD10	0.31085	0.34334	-0.03249	0.602
5150	218440_at	MCCC1	0.31866	0.47742	-0.15876	0.899
5151	218441_s_at	RPAP1	0.29444	0.1483	0.14614	0.054
5152	218443_s_at	DAZAP1	0.92551	0.92555	-4E-05	0.527
5153	218444_at	ALG12	0.25483	0.074232	0.180598	0.031
5154	218446_s_at	FAM18B	0.52988	0.66214	-0.13226	0.991
5155	218447_at	C16orf61	0.57807	0.61966	-0.04159	0.681
5156	218448_at	C20orf11	0.54187	0.37457	0.1673	0.09
5157	218449_at	UFSP2	0.10047	0.23813	-0.13766	0.905
5158	218452_at	SMARCAL1	0.46715	0.59294	-0.12579	0.935
5159	218455_at	NFS1	0.15603	0.15891	-0.00288	0.511
5160	218456_at	CAPRIN2	0.58363	0.63586	-0.05223	0.807
5161	218457_s_at	DNMT3A	0.58051	0.77014	-0.18963	0.984
5162	218458_at	GMCL1	0.74552	0.69946	0.04606	0.308
5163	218459_at	TOR3A	0.49213	0.44298	0.04915	0.383
5164	218460_at	HEATR2	0.55649	0.50954	0.04695	0.163
5165	218461_at	GPN3	0.79995	0.70672	0.09323	0.085
5166	218462_at	BXDC5	0.79861	0.83318	-0.03457	0.726
5167	218463_s_at	MUS81	0.36611	0.39832	-0.03221	0.645
5168	218464_s_at	C17orf63	0.49943	0.57413	-0.0747	0.763
5169	218465_at	TMEM33	0.62933	0.56404	0.06529	0.234
5170	218466_at	TBC1D17	0.59421	0.61096	-0.01675	0.61
5171	218467_at	PSMG2	0.81816	0.79929	0.01887	0.392
5172	218470_at	YARS2	0.42928	0.54631	-0.11703	0.879
5173	218471_s_at	BBS1	0.56669	0.46117	0.10552	0.138
5174	218473_s_at	GLT25D1	0.31475	0.33778	-0.02303	0.656
5175	218474_s_at	KCTD5	0.5222	0.48395	0.03825	0.384
5176	218476_at	POMT1	0.23822	0.35099	-0.11277	0.869
5177	218477_at	TMEM14A	0.58573	0.71204	-0.12631	0.921
5178	218478_s_at	ZCCHC8	0.50593	0.23158	0.27435	0.004
5179	218479_s_at	XPO4	0.3268	0.16839	0.15841	0.041
5180	218480_at	AGBL5	0.089466	0.21826	-0.128794	0.889
5181	218481_at	EXOSC5	0.58586	0.71643	-0.13057	0.924
5182	218482_at	ENY2	0.50892	0.3829	0.12602	0.02

5183	218483_s_at	C11orf60	0.19363	0.42202	-0.22839	0.99
5184	218487_at	ALAD	0.44323	0.48916	-0.04593	0.706
5185	218488_at	EIF2B3	0.74123	0.72858	0.01265	0.472
5186	218490_s_at	ZNF302	0.14742	0.32039	-0.17297	0.933
5187	218491_s_at	THYN1	0.71583	0.77415	-0.05832	0.812
5188	218492_s_at	THAP7	0.55795	0.57066	-0.01271	0.559
5189	218493_at	SNRNP25	0.77342	0.77676	-0.00334	0.532
5190	218494_s_at	SLC2A4RG	0.77819	0.70122	0.07697	0.055
5191	218495_at	UXT	0.89388	0.91734	-0.02346	0.729
5192	218496_at	RNASEH1	0.29686	0.42662	-0.12976	0.868
5193	218498_s_at	ERO1L	0.3379	0.44627	-0.10837	0.83
5194	218499_at	RP6-213H19.1	0.60464	0.6231	-0.01846	0.579
5195	218500_at	C8orf55	0.55951	0.25359	0.30592	0.001
5196	218501_at	ARHGEF3	0.46631	0.60175	-0.13544	0.902
5197	218503_at	KIAA1797	0.18068	0.25104	-0.07036	0.727
5198	218507_at	C7orf68	0.74551	0.6774	0.06811	0.216
5199	218508_at	DCP1A	0.71178	0.62071	0.09107	0.076
5200	218511_s_at	PNPO	0.55427	0.52852	0.02575	0.416
5201	218512_at	WDR12	0.85427	0.8057	0.04857	0.136
5202	218513_at	C4orf43	0.42774	0.51479	-0.08705	0.825
5203	218514_at	C17orf71	0.16138	0.19492	-0.03354	0.624
5204	218515_at	C21orf66	0.24851	0.5446	-0.29609	0.998
5205	218516_s_at	IMPAD1	0.21057	0.29018	-0.07961	0.708
5206	218517_at	PHF17	0.65441	0.59883	0.05558	0.277
5207	218518_at	FAM13B	0.77318	0.70876	0.06442	0.214
5208	218519_at	SLC35A5	0.42194	0.5005	-0.07856	0.831
5209	218520_at	TBK1	0.41919	0.26295	0.15624	0.071
5210	218521_s_at	UBE2W	0.84268	0.89135	-0.04867	0.866
5211	218522_s_at	MAP1S	0.57031	0.6305	-0.06019	0.793
5212	218524_at	E4F1	0.32467	0.56294	-0.23827	0.97
5213	218525_s_at	HIF1AN	0.26179	0.15261	0.10918	0.198
5214	218526_s_at	RANGRF	0.7463	0.734	0.0123	0.465
5215	218527_at	APTX	0.51016	0.37745	0.13271	0.096
5216	218528_s_at	RNF38	0.40128	0.29438	0.1069	0.166
5217	218529_at	CD320	0.65807	0.64575	0.01232	0.431
5218	218530_at	FHOD1	0.67413	0.72071	-0.04658	0.728
5219	218531_at	TMEM134	0.54626	0.47692	0.06934	0.209
5220	218532_s_at	FAM134B	0.26823	0.38456	-0.11633	0.807
5221	218533_s_at	UCKL1	0.23387	0.36344	-0.12957	0.89
5222	218534_s_at	AGGF1	0.55608	0.5469	0.00918	0.49
5223	218535_s_at	RIOK2	0.4986	0.68649	-0.18789	0.973
5224	218536_at	MRS2	0.28027	0.37831	-0.09804	0.941
5225	218537_at	HCFC1R1	0.3462	0.27832	0.06788	0.336
5226	218539_at	FBXO34	0.28197	0.25606	0.02591	0.399
5227	218542_at	CEP55	0.75186	0.7691	-0.01724	0.635
5228	218543_s_at	PARP12	0.6217	0.67796	-0.05626	0.708
5229	218544_s_at	RCL1	0.44289	0.31142	0.13147	0.101
5230	218545_at	CCDC91	0.38956	0.4622	-0.07264	0.755
5231	218547_at	DHDDS	0.51861	0.45947	0.05914	0.266
5232	218549_s_at	FAM82B	0.70085	0.81346	-0.11261	0.945
5233	218550_s_at	LRRC20	0.33684	0.13243	0.20441	<0.001
5234	218552_at	ECHDC2	0.12297	0.26987	-0.1469	0.898
5235	218554_s_at	ASH1L	0.82269	0.72175	0.10094	0.028
5236	218555_at	ANAPC2	0.47392	0.57187	-0.09795	0.826

5237	218556_at	ORMDL2	0.41061	0.7387	-0.32809	0.999
5238	218557_at	NIT2	0.822	0.81119	0.01081	0.418
5239	218558_s_at	MRPL39	0.74982	0.79855	-0.04873	0.855
5240	218561_s_at	LYRM4	0.61024	0.68008	-0.06984	0.812
5241	218562_s_at	TMEM57	0.3364	0.322	0.0144	0.44
5242	218563_at	NDUFA3	0.56495	0.5968	-0.03185	0.679
5243	218564_at	RFWD3	0.75481	0.80679	-0.05198	0.768
5244	218565_at	C9orf114	0.14134	0.15448	-0.01314	0.564
5245	218566_s_at	CHORDC1	0.92077	0.74344	0.17733	0.024
5246	218568_at	AGK	0.49687	0.45522	0.04165	0.345
5247	218570_at	KBTBD4	0.5258	0.43949	0.08631	0.205
5248	218571_s_at	CHMP4A	0.44234	0.49314	-0.0508	0.655
5249	218573_at	MAGEH1	0.4249	0.2938	0.1311	0.135
5250	218574_s_at	LMCD1	0.24716	0.33745	-0.09029	0.744
5251	218575_at	ANAPC1	0.43329	0.51882	-0.08553	0.773
5252	218576_s_at	DUSP12	0.71224	0.57783	0.13441	0.083
5253	218577_at	LRRC40	0.84437	0.84419	0.00018	0.536
5254	218578_at	CDC73	0.59421	0.72078	-0.12657	0.915
5255	218579_s_at	DHX35	0.03016	0.048418	-0.018258	0.585
5256	218581_at	ABHD4	0.45464	0.26344	0.1912	0.004
5257	218582_at	MARCH5	0.43514	0.43384	0.0013	0.503
5258	218583_s_at	DCUN1D1	0.55203	0.36466	0.18737	0.023
5259	218584_at	TCTN1	0.3096	0.35674	-0.04714	0.644
5260	218585_s_at	DTL	0.79085	0.63923	0.15162	<0.001
5261	218586_at	C20orf20	0.5973	0.52141	0.07589	0.178
5262	218587_s_at	KTELC1	0.73185	0.76012	-0.02827	0.665
5263	218588_s_at	FAM114A2	0.37638	0.49502	-0.11864	0.882
5264	218590_at	C10orf2	0.58805	0.3856	0.20245	<0.001
5265	218592_s_at	CECR5	0.45092	0.39211	0.05881	0.29
5266	218593_at	RBM28	0.36442	0.5406	-0.17618	0.933
5267	218594_at	HEATR1	0.74402	0.45847	0.28555	<0.001
5268	218596_at	TBC1D13	0.41548	0.16522	0.25026	0.016
5269	218597_s_at	CISD1	0.66076	0.62869	0.03207	0.366
5270	218598_at	RINT1	0.71973	0.77585	-0.05612	0.836
5271	218599_at	REC8	0.26278	0.41446	-0.15168	0.879
5272	218600_at	LIMD2	0.71914	0.71785	0.00129	0.53
5273	218602_s_at	HAUS6	0.76854	0.79132	-0.02278	0.692
5274	218603_at	HECA	0.50652	0.53834	-0.03182	0.64
5275	218604_at	LEMD3	0.47228	0.33437	0.13791	0.175
5276	218605_at	TFB2M	0.56567	0.58849	-0.02282	0.607
5277	218606_at	ZDHHC7	0.26493	0.27711	-0.01218	0.563
5278	218607_s_at	SDAD1	0.19783	0.46719	-0.26936	0.979
5279	218608_at	ATP13A2	0.36717	0.46264	-0.09547	0.829
5280	218609_s_at	NUDT2	0.053371	0.19639	-0.143019	0.872
5281	218610_s_at	CPPED1	0.23545	0.2207	0.01475	0.453
5282	218611_at	IER5	0.79986	0.77033	0.02953	0.253
5283	218612_s_at	TSSC4	0.75887	0.70514	0.05373	0.221
5284	218614_at	C12orf35	0.31498	0.55427	-0.23929	0.961
5285	218615_s_at	TMEM39A	0.35002	0.35465	-0.00463	0.564
5286	218616_at	INTS12	0.73292	0.66134	0.07158	0.116
5287	218617_at	TRIT1	0.22677	0.29655	-0.06978	0.698
5288	218618_s_at	FNDC3B	0.74129	0.7651	-0.02381	0.651
5289	218619_s_at	SUV39H1	0.75194	0.69184	0.0601	0.156
5290	218620_s_at	HEMK1	0.32291	0.42544	-0.10253	0.8

5291	218622_at	NUP37	0.80198	0.8415	-0.03952	0.742
5292	218624_s_at	MGC2752	0.32308	0.32669	-0.00361	0.506
5293	218626_at	EIF4ENIF1	0.41331	0.43314	-0.01983	0.583
5294	218627_at	DRAM	0.58425	0.66137	-0.07712	0.816
5295	218628_at	CCDC53	0.59967	0.69777	-0.0981	0.842
5296	218630_at	MKS1	0.26081	0.22798	0.03283	0.39
5297	218631_at	AVPI1	0.27884	0.27669	0.00215	0.508
5298	218632_at	HECTD3	0.74995	0.69406	0.05589	0.181
5299	218634_at	PHLDA3	0.77959	0.78287	-0.00328	0.524
5300	218636_s_at	MAN1B1	0.28038	0.19185	0.08853	0.233
5301	218637_at	IMPACT	0.2155	0.41013	-0.19463	0.903
5302	218639_s_at	ZXDC	0.28443	0.37566	-0.09123	0.779
5303	218640_s_at	PLEKHF2	0.79614	0.75057	0.04557	0.185
5304	218641_at	LOC65998	0.46245	0.33896	0.12349	0.177
5305	218642_s_at	CHCHD7	0.54936	0.50384	0.04552	0.34
5306	218643_s_at	CRIPT	0.61016	0.63346	-0.0233	0.623
5307	218645_at	ZNF277	0.60266	0.53056	0.0721	0.283
5308	218646_at	C4orf27	0.68503	0.71859	-0.03356	0.747
5309	218647_s_at	YRDC	0.66421	0.56335	0.10086	0.161
5310	218648_at	CRTC3	0.37309	0.43053	-0.05744	0.66
5311	218650_at	DGCR8	0.38259	0.37854	0.00405	0.515
5312	218652_s_at	PIGG	0.68791	0.71977	-0.03186	0.659
5313	218653_at	SLC25A15	0.58006	0.45131	0.12875	0.039
5314	218654_s_at	MRPS33	0.79521	0.81022	-0.01501	0.648
5315	218655_s_at	CCDC49	0.53006	0.23481	0.29525	0.001
5316	218656_s_at	LHFP	0.23693	0.46502	-0.22809	0.97
5317	218658_s_at	ACTR8	0.30555	0.20911	0.09644	0.19
5318	218659_at	ASXL2	0.50813	0.46725	0.04088	0.33
5319	218661_at	NAT15	0.2987	0.30223	-0.00353	0.589
5320	218663_at	NCAPG	0.66146	0.70674	-0.04528	0.887
5321	218664_at	MECR	0.47678	0.59363	-0.11685	0.946
5322	218666_s_at	STX17	0.51487	0.53898	-0.02411	0.61
5323	218667_at	PJA1	0.26028	0.33287	-0.07259	0.77
5324	218669_at	RAP2C	0.55608	0.6485	-0.09242	0.784
5325	218670_at	PUS1	0.43637	0.37022	0.06615	0.307
5326	218671_s_at	ATPIF1	0.47251	0.51711	-0.0446	0.676
5327	218672_at	SCNM1	0.78431	0.73931	0.045	0.15
5328	218673_s_at	ATG7	0.39492	0.34435	0.05057	0.334
5329	218674_at	C5orf44	0.67812	0.74502	-0.0669	0.84
5330	218676_s_at	PCTP	0.49272	0.58003	-0.08731	0.777
5331	218679_s_at	VPS28	0.62363	0.65628	-0.03265	0.675
5332	218681_s_at	SDF2L1	0.72461	0.717	0.00761	0.479
5333	218682_s_at	SLC4A1AP	0.54511	0.52675	0.01836	0.454
5334	218683_at	PTBP2	0.67967	0.72054	-0.04087	0.71
5335	218684_at	LRRC8D	0.39904	0.47618	-0.07714	0.859
5336	218685_s_at	SMUG1	0.26651	0.31837	-0.05186	0.685
5337	218688_at	DAK	0.4889	0.47443	0.01447	0.437
5338	218689_at	FANCF	0.33999	0.44441	-0.10442	0.813
5339	218692_at	GOLSYN	0.3853	0.46147	-0.07617	0.725
5340	218694_at	ARMCX1	0.17863	0.34954	-0.17091	0.906
5341	218696_at	EIF2AK3	0.61095	0.62681	-0.01586	0.676
5342	218698_at	APIP	0.57976	0.58036	-0.0006	0.507
5343	218699_at	RAB7L1	0.64174	0.67659	-0.03485	0.654
5344	218701_at	LACTB2	0.61761	0.68229	-0.06468	0.78

5345	218703_at	SEC22A	0.49693	0.32287	0.17406	0.025
5346	218705_s_at	SNX24	0.63432	0.65762	-0.0233	0.64
5347	218706_s_at	GRAMD3	0.40469	0.33505	0.06964	0.54
5348	218708_at	NXT1	0.83953	0.82638	0.01315	0.432
5349	218709_s_at	IFT52	0.52394	0.49982	0.02412	0.42
5350	218710_at	TTC27	0.4621	0.37538	0.08672	0.192
5351	218712_at	C1orf109	0.36016	0.61102	-0.25086	0.985
5352	218713_at	NARG2	0.35884	0.49344	-0.1346	0.908
5353	218715_at	UTP6	0.74131	0.71657	0.02474	0.383
5354	218719_s_at	GINS3	0.67422	0.65703	0.01719	0.378
5355	218721_s_at	C1orf27	0.59818	0.67083	-0.07265	0.785
5356	218722_s_at	CCDC51	0.51842	0.64093	-0.12251	0.943
5357	218723_s_at	C13orf15	0.75444	0.76534	-0.0109	0.575
5358	218725_at	SLC25A22	0.18964	0.20862	-0.01898	0.548
5359	218726_at	HJURP	0.83217	0.72371	0.10846	0.034
5360	218728_s_at	CNIH4	0.53466	0.70365	-0.16899	0.917
5361	218729_at	LXN	0.32472	0.45573	-0.13101	0.861
5362	218732_at	PTRH2	0.6634	0.47497	0.18843	0.025
5363	218733_at	MSL2	0.53929	0.43709	0.1022	0.037
5364	218735_s_at	ZNF544	0.29555	0.24789	0.04766	0.34
5365	218738_s_at	RNF138	0.8055	0.67161	0.13389	0.015
5366	218739_at	ABHD5	0.68234	0.54029	0.14205	0.024
5367	218740_s_at	CDK5RAP3	0.70894	0.73891	-0.02997	0.701
5368	218741_at	CENPM	0.73914	0.83011	-0.09097	0.929
5369	218742_at	NARFL	0.07308	0.19806	-0.12498	0.823
5370	218743_at	CHMP6	0.26143	0.25013	0.0113	0.468
5371	218746_at	TAPBPL	0.23288	0.2301	0.00278	0.481
5372	218748_s_at	EXOC5	0.89922	0.88847	0.01075	0.433
5373	218751_s_at	FBXW7	0.54191	0.66543	-0.12352	0.919
5374	218752_at	ZMAT5	0.58265	0.59676	-0.01411	0.588
5375	218753_at	XKR8	0.34312	0.38553	-0.04241	0.65
5376	218754_at	NOL9	0.65595	0.59149	0.06446	0.235
5377	218755_at	KIF20A	0.60965	0.64685	-0.0372	0.632
5378	218756_s_at	DHRS11	0.14769	0.21136	-0.06367	0.699
5379	218757_s_at	UPF3B	0.33941	0.39179	-0.05238	0.642
5380	218758_s_at	RRP1	0.34925	0.19439	0.15486	0.09
5381	218759_at	DVL2	0.23575	0.16575	0.07	0.288
5382	218760_at	COQ6	0.10643	0.24812	-0.14169	0.889
5383	218761_at	RNF111	0.54	0.50158	0.03842	0.362
5384	218762_at	ZNF574	0.1278	0.11534	0.01246	0.457
5385	218763_at	STX18	0.6183	0.71462	-0.09632	0.875
5386	218764_at	PRKCH	0.52212	0.63864	-0.11652	0.937
5387	218766_s_at	WARS2	0.45345	0.33543	0.11802	0.129
5388	218767_at	REXO4	0.35429	0.49251	-0.13822	0.896
5389	218768_at	NUP107	0.86157	0.79149	0.07008	0.036
5390	218769_s_at	ANKRA2	0.67556	0.69568	-0.02012	0.594
5391	218770_s_at	TMEM39B	0.41752	0.54454	-0.12702	0.943
5392	218771_at	PANK4	0.2162	0.10287	0.11333	0.066
5393	218774_at	DCPS	0.47937	0.63436	-0.15499	0.947
5394	218776_s_at	TMEM62	0.26881	0.3771	-0.10829	0.856
5395	218777_at	REEP4	0.26547	0.067214	0.198256	0.047
5396	218781_at	SMC6	0.56967	0.26594	0.30373	<0.001
5397	218782_s_at	ATAD2	0.87389	0.79854	0.07535	0.005
5398	218785_s_at	RABL5	0.14456	0.18426	-0.0397	0.625

5399	218786_at	NT5DC3	0.076898	0.12979	-0.052892	0.673
5400	218788_s_at	SMYD3	0.50865	0.67062	-0.16197	0.932
5401	218789_s_at	C11orf71	0.45651	0.38302	0.07349	0.227
5402	218791_s_at	C15orf29	0.18928	0.43062	-0.24134	0.973
5403	218794_s_at	TXNL4B	0.22275	0.50383	-0.28108	0.993
5404	218795_at	ACP6	0.083561	0.28786	-0.204299	0.908
5405	218797_s_at	SIRT7	0.44854	0.6064	-0.15786	0.989
5406	218798_at	KRI1	0.11308	0.19086	-0.07778	0.717
5407	218799_at	GPN2	0.59345	0.60016	-0.00671	0.553
5408	218800_at	SRD5A3	0.083642	0.19456	-0.110918	0.721
5409	218801_at	UGCGL2	0.0096706	0.057047	-0.0473764	0.666
5410	218802_at	CCDC109B	0.38433	0.6151	-0.23077	0.983
5411	218803_at	CHFR	0.69018	0.66507	0.02511	0.392
5412	218809_at	PANK2	0.44742	0.51176	-0.06434	0.779
5413	218810_at	ZC3H12A	0.49695	0.45711	0.03984	0.376
5414	218812_s_at	ORAI2	0.69148	0.70352	-0.01204	0.59
5415	218813_s_at	SH3GLB2	0.44877	0.39532	0.05345	0.267
5416	218817_at	SPCS3	0.68426	0.72827	-0.04401	0.687
5417	218823_s_at	KCTD9	0.65535	0.71061	-0.05526	0.722
5418	218826_at	SLC35F2	0.27944	0.45307	-0.17363	0.919
5419	218827_s_at	CEP192	0.0022772	0.057629	-0.0553518	0.659
5420	218830_at	RPL26L1	0.73913	0.62691	0.11222	0.004
5421	218833_at	ZAK	0.23949	0.21756	0.02193	0.46
5422	218836_at	RPP21	0.61534	0.59812	0.01722	0.42
5423	218837_s_at	UBE2D4	0.24626	0.37819	-0.13193	0.843
5424	218838_s_at	TTC31	0.21205	0.57802	-0.36597	0.998
5425	218840_s_at	NADSYN1	0.77834	0.67482	0.10352	0.051
5426	218841_at	ASB8	0.31016	0.31985	-0.00969	0.565
5427	218842_at	RPAP3	0.73867	0.6756	0.06307	0.108
5428	218844_at	ACSF2	0.32359	0.34574	-0.02215	0.588
5429	218845_at	DUSP22	0.34977	0.44492	-0.09515	0.776
5430	218846_at	MED23	0.51805	0.569	-0.05095	0.719
5431	218848_at	THOC6	0.63544	0.66115	-0.02571	0.622
5432	218850_s_at	LIMD1	0.84955	0.86972	-0.02017	0.72
5433	218851_s_at	WDR33	0.27341	0.036551	0.236859	0.025
5434	218852_at	PPP2R3C	0.38406	0.51355	-0.12949	0.884
5435	218853_s_at	MOSPD1	0.65978	0.59636	0.06342	0.175
5436	218854_at	DSE	0.49984	0.60781	-0.10797	0.87
5437	218855_at	GPR175	0.13916	0.1041	0.03506	0.306
5438	218858_at	DEPDC6	0.022817	0.22626	-0.203443	0.972
5439	218859_s_at	ESF1	0.85013	0.85852	-0.00839	0.539
5440	218860_at	NOC4L	0.42751	0.47434	-0.04683	0.671
5441	218861_at	RNF25	0.38289	0.35456	0.02833	0.418
5442	218866_s_at	POLR3K	0.66021	0.73313	-0.07292	0.846
5443	218867_s_at	C12orf49	0.18942	0.21283	-0.02341	0.595
5444	218868_at	ACTR3B	0.44739	0.42007	0.02732	0.372
5445	218869_at	MLYCD	0.11851	0.285	-0.16649	0.886
5446	218870_at	ARHGAP15	0.3477	0.6704	-0.3227	1
5447	218872_at	TESC	0.32178	0.55722	-0.23544	0.958
5448	218873_at	GON4L	0.50052	0.3345	0.16602	0.076
5449	218874_s_at	C6orf134	0.043742	0.12048	-0.076738	0.783
5450	218875_s_at	FBXO5	0.69953	0.62416	0.07537	0.315
5451	218877_s_at	TRMT11	0.30773	0.32416	-0.01643	0.564
5452	218878_s_at	SIRT1	0.74516	0.6415	0.10366	0.075

5453	218879_s_at	MTHFSD	0.41185	0.43113	-0.01928	0.552
5454	218882_s_at	WDR3	0.65428	0.31704	0.33724	<0.001
5455	218883_s_at	MLF1IP	0.49562	0.538	-0.04238	0.674
5456	218884_s_at	GUF1	0.30202	0.56074	-0.25872	0.991
5457	218886_at	PAK1IP1	0.18516	0.28096	-0.0958	0.87
5458	218887_at	MRPL2	0.32776	0.57566	-0.2479	0.986
5459	218888_s_at	NETO2	0.4989	0.14334	0.35556	0.019
5460	218889_at	NOC3L	0.61433	0.29178	0.32255	<0.001
5461	218893_at	ISOC2	0.44074	0.30308	0.13766	0.333
5462	218894_s_at	MAGOHB	0.6367	0.61117	0.02553	0.384
5463	218895_at	GPATCH3	0.15581	0.050615	0.105195	0.168
5464	218896_s_at	C17orf85	0.33293	0.26428	0.06865	0.313
5465	218897_at	TMEM177	0.45193	0.15394	0.29799	<0.001
5466	218898_at	FAM57A	0.53584	0.59254	-0.0567	0.771
5467	218900_at	CNNM4	0.24333	0.49883	-0.2555	0.985
5468	218902_at	NOTCH1	0.37119	0.4167	-0.04551	0.662
5469	218903_s_at	OBFC2B	0.30285	0.22617	0.07668	0.299
5470	218904_s_at	C9orf40	0.62515	0.29628	0.32887	<0.001
5471	218905_at	INTS8	0.19134	0.27468	-0.08334	0.727
5472	218907_s_at	LRRC61	0.18681	0.15502	0.03179	0.375
5473	218908_at	ASPSCR1	0.23897	0.37166	-0.13269	0.878
5474	218909_at	RPS6KC1	0.56323	0.2344	0.32883	<0.001
5475	218911_at	YEATS4	0.60623	0.77396	-0.16773	0.975
5476	218912_at	GCC1	0.089687	0.00093456	0.08875244	0.147
5477	218913_s_at	GMIP	0.73242	0.62449	0.10793	0.031
5478	218914_at	C1orf66	0.3227	0.22098	0.10172	0.203
5479	218916_at	ZNF768	0.50837	0.56887	-0.0605	0.703
5480	218919_at	ZFAND1	0.42315	0.23615	0.187	0.018
5481	218920_at	FLJ10404	0.49609	0.39697	0.09912	0.193
5482	218922_s_at	LASS4	0.30287	0.25542	0.04745	0.37
5483	218924_s_at	CTBS	0.62837	0.60791	0.02046	0.451
5484	218926_at	MYNN	0.48012	0.44995	0.03017	0.395
5485	218927_s_at	CHST12	0.62727	0.72069	-0.09342	0.789
5486	218928_s_at	SLC37A1	0.078301	0.29952	-0.221219	0.964
5487	218929_at	CDKN2AIP	0.64172	0.54166	0.10006	0.089
5488	218930_s_at	TMEM106B	0.65121	0.52768	0.12353	0.163
5489	218932_at	ZNHIT6	0.39148	0.4346	-0.04312	0.654
5490	218935_at	EHD3	0.51401	0.53229	-0.01828	0.59
5491	218936_s_at	CCDC59	0.77749	0.70731	0.07018	0.081
5492	218937_at	ZNF434	0.25999	0.49404	-0.23405	0.979
5493	218938_at	FBXL15	0.50183	0.58335	-0.08152	0.781
5494	218940_at	C14orf138	0.36424	0.39803	-0.03379	0.609
5495	218942_at	PIP4K2C	0.69876	0.67582	0.02294	0.374
5496	218943_s_at	DDX58	0.45942	0.42679	0.03263	0.392
5497	218945_at	C16orf68	0.29273	0.29741	-0.00468	0.541
5498	218946_at	NFU1	0.60601	0.70863	-0.10262	0.876
5499	218947_s_at	MTPAP	0.69704	0.64553	0.05151	0.233
5500	218949_s_at	QRSL1	0.72349	0.61851	0.10498	0.069
5501	218951_s_at	PLCXD1	0.4013	0.3756	0.0257	0.42
5502	218953_s_at	PCYOX1L	0.33801	0.47181	-0.1338	0.85
5503	218954_s_at	BRF2	0.77431	0.61422	0.16009	0.012
5504	218956_s_at	PTCD1	0.46854	0.50568	-0.03714	0.64
5505	218957_s_at	PAAF1	0.16684	0.32831	-0.16147	0.92
5506	218958_at	C19orf60	0.34108	0.29546	0.04562	0.339

5507	218961_s_at	PNKP	0.48065	0.6124	-0.13175	0.874
5508	218962_s_at	TMEM168	0.47232	0.62559	-0.15327	0.94
5509	218964_at	ARID3B	0.312	0.28509	0.02691	0.425
5510	218965_s_at	TUT1	0.2867	0.34585	-0.05915	0.693
5511	218966_at	MYO5C	0.28754	0.46749	-0.17995	0.92
5512	218967_s_at	PTER	0.71761	0.69204	0.02557	0.37
5513	218968_s_at	ZFP64	0.25744	0.22166	0.03578	0.382
5514	218969_at	Magmas	0.5856	0.72121	-0.13561	0.959
5515	218970_s_at	CUTC	0.39593	0.46947	-0.07354	0.789
5516	218971_s_at	WDR91	0.59376	0.54833	0.04543	0.3
5517	218972_at	TTC17	0.2908	0.088891	0.201909	0.019
5518	218973_at	EFTUD1	0.20007	0.29138	-0.09131	0.784
5519	218974_at	SOBP	0.36487	0.51095	-0.14608	0.915
5520	218976_at	DNAJC12	0.26448	0.32671	-0.06223	0.691
5521	218977_s_at	TRNAU1AP	0.23216	0.37631	-0.14415	0.911
5522	218979_at	RMI1	0.75941	0.70801	0.0514	0.159
5523	218981_at	ACN9	0.63145	0.65897	-0.02752	0.693
5524	218982_s_at	MRPS17	0.75123	0.61535	0.13588	0.042
5525	218983_at	C1RL	0.30968	0.43686	-0.12718	0.842
5526	218984_at	PUS7	0.7005	0.44353	0.25697	<0.001
5527	218985_at	SLC2A8	0.35272	0.26198	0.09074	0.195
5528	218986_s_at	DDX60	0.7375	0.76652	-0.02902	0.635
5529	218987_at	ATF7IP	0.65996	0.57301	0.08695	0.111
5530	218988_at	SLC35E3	0.51484	0.50872	0.00612	0.471
5531	218991_at	HEATR6	0.059152	0.12277	-0.063618	0.714
5532	218992_at	C9orf46	0.6085	0.70499	-0.09649	0.834
5533	218993_at	RNMTL1	0.78184	0.56531	0.21653	<0.001
5534	218994_s_at	STAG3L4	0.32366	0.35113	-0.02747	0.608
5535	218996_at	TFPT	0.51997	0.56844	-0.04847	0.7
5536	218997_at	POLR1E	0.21349	0.3616	-0.14811	0.916
5537	218998_at	C9orf6	0.14526	0.22852	-0.08326	0.831
5538	218999_at	TMEM140	0.7454	0.73758	0.00782	0.448
5539	219001_s_at	WDR32	0.56234	0.63495	-0.07261	0.816
5540	219002_at	FASTKD1	0.50681	0.29524	0.21157	0.021
5541	219003_s_at	MANEA	0.67975	0.76348	-0.08373	0.86
5542	219004_s_at	C21orf45	0.76748	0.71886	0.04862	0.116
5543	219006_at	NDUFAF4	0.82004	0.78451	0.03553	0.275
5544	219007_at	NUP43	0.63785	0.55087	0.08698	0.203
5545	219009_at	C14orf93	0.091967	0.096953	-0.004986	0.519
5546	219010_at	C1orf106	0.5969	0.75904	-0.16214	0.992
5547	219012_s_at	C11orf30	0.14652	0.17186	-0.02534	0.587
5548	219013_at	GALNT11	0.13803	0.34559	-0.20756	0.99
5549	219014_at	PLAC8	0.49926	0.58132	-0.08206	0.817
5550	219016_at	FASTKD5	0.28224	0.22195	0.06029	0.301
5551	219020_at	HS1BP3	0.14673	0.36435	-0.21762	0.963
5552	219021_at	RNF121	0.48998	0.37782	0.11216	0.179
5553	219022_at	C12orf43	0.19084	0.29926	-0.10842	0.847
5554	219023_at	C4orf16	0.80908	0.65539	0.15369	0.003
5555	219027_s_at	MYO9A	0.41891	0.23622	0.18269	0.046
5556	219029_at	C5orf28	0.64575	0.50492	0.14083	0.002
5557	219030_at	TPRKB	0.46442	0.53523	-0.07081	0.743
5558	219031_s_at	NIP7	0.49466	0.39304	0.10162	0.163
5559	219033_at	PARP8	0.45257	0.53999	-0.08742	0.891
5560	219034_at	PARP16	0.2642	0.14952	0.11468	0.184

5561	219035_s_at	RNF34	0.40086	0.58675	-0.18589	0.968
5562	219036_at	CEP70	0.56677	0.61778	-0.05101	0.74
5563	219040_at	CORO7	0.64853	0.49287	0.15566	0.026
5564	219041_s_at	REPIN1	0.52735	0.44399	0.08336	0.103
5565	219043_s_at	LOC285359	0.54205	0.45436	0.08769	0.097
5566	219045_at	RHOF	0.65472	0.63476	0.01996	0.389
5567	219047_s_at	ZNF668	0.057796	0.054451	0.003345	0.575
5568	219048_at	PIGN	0.10592	0.03628	0.06964	0.285
5569	219052_at	HPS6	0.057348	0.19784	-0.140492	0.938
5570	219053_s_at	VPS37C	0.39403	0.38838	0.00565	0.473
5571	219055_at	SRBD1	0.27524	0.41914	-0.1439	0.897
5572	219060_at	WDYHV1	0.36846	0.39127	-0.02281	0.603
5573	219061_s_at	LAGE3	0.13332	0.2786	-0.14528	0.891
5574	219062_s_at	ZCCHC2	0.75178	0.71217	0.03961	0.256
5575	219063_at	C1orf35	0.36897	0.095419	0.273551	0.002
5576	219065_s_at	MEMO1	0.53764	0.58704	-0.0494	0.714
5577	219066_at	PPCDC	0.53605	0.67127	-0.13522	0.924
5578	219067_s_at	NSMCE4A	0.66221	0.75892	-0.09671	0.93
5579	219069_at	ANKRD49	0.52795	0.48626	0.04169	0.33
5580	219070_s_at	MOSPD3	0.58122	0.41455	0.16667	0.063
5581	219072_at	BCL7C	0.50581	0.4255	0.08031	0.245
5582	219074_at	TMEM184C	0.46452	0.28896	0.17556	0.062
5583	219076_s_at	PXMP2	0.16691	0.19264	-0.02573	0.603
5584	219077_s_at	WWOX	0.27279	0.43697	-0.16418	0.9
5585	219078_at	GPATCH2	0.34923	0.41875	-0.06952	0.724
5586	219079_at	CYB5R4	0.41399	0.28703	0.12696	0.142
5587	219080_s_at	CTPS2	0.53576	0.53547	0.00029	0.54
5588	219084_at	NSD1	0.15203	0.047546	0.104484	0.222
5589	219086_at	ZNF839	0.14296	0.1219	0.02106	0.437
5590	219089_s_at	ZNF576	0.057191	0.29961	-0.242419	0.986
5591	219092_s_at	IPPK	0.14458	0.02438	0.1202	0.175
5592	219096_at	ARMC7	0.31224	0.31402	-0.00178	0.54
5593	219098_at	MYBBP1A	0.5293	0.26618	0.26312	0.003
5594	219099_at	C12orf5	0.74764	0.71082	0.03682	0.305
5595	219100_at	OBFC1	0.48583	0.56894	-0.08311	0.815
5596	219104_at	RNF141	0.18869	0.2183	-0.02961	0.616
5597	219109_at	SPAG16	0.46155	0.5681	-0.10655	0.885
5598	219110_at	GAR1	0.8456	0.77272	0.07288	0.01
5599	219111_s_at	DDX54	0.19147	0.30682	-0.11535	0.84
5600	219112_at	RAPGEF6	0.6582	0.58801	0.07019	0.151
5601	219116_s_at	DCUN1D2	0.44047	0.27426	0.16621	0.054
5602	219117_s_at	FKBP11	0.62586	0.41443	0.21143	0.008
5603	219119_at	LSM8	0.2282	0.49152	-0.26332	0.865
5604	219120_at	C2orf44	0.26329	0.084113	0.179177	0.035
5605	219122_s_at	THG1L	0.40395	0.35733	0.04662	0.37
5606	219123_at	ZNF232	0.36474	0.26928	0.09546	0.168
5607	219124_at	C8orf41	0.38689	0.29309	0.0938	0.186
5608	219125_s_at	RAG1AP1	0.6084	0.3344	0.274	<0.001
5609	219126_at	PHF10	0.42355	0.43925	-0.0157	0.602
5610	219128_at	C2orf42	0.65606	0.43627	0.21979	0.024
5611	219129_s_at	SAP30L	0.58708	0.61352	-0.02644	0.636
5612	219130_at	CCDC76	0.35535	0.16037	0.19498	0.047
5613	219131_at	UBIAD1	0.6454	0.38474	0.26066	<0.001
5614	219133_at	OXSM	0.49099	0.67824	-0.18725	0.963

5615	219137_s_at	MFF	0.58661	0.50045	0.08616	0.219
5616	219143_s_at	RPP25	0.56791	0.42687	0.14104	0.072
5617	219146_at	C17orf42	0.19285	0.28762	-0.09477	0.814
5618	219147_s_at	C9orf95	0.19904	0.42005	-0.22101	0.978
5619	219148_at	PBK	0.68552	0.77327	-0.08775	0.901
5620	219150_s_at	ADAP1	0.34149	0.36212	-0.02063	0.599
5621	219155_at	PITPNC1	0.46175	0.66483	-0.20308	0.921
5622	219156_at	SYNJ2BP	0.65803	0.66131	-0.00328	0.522
5623	219157_at	KLHL2	0.29831	0.21682	0.08149	0.186
5624	219158_s_at	NARG1	0.9267	0.92806	-0.00136	0.515
5625	219159_s_at	SLAMF7	0.78103	0.8569	-0.07587	0.969
5626	219162_s_at	MRPL11	0.85336	0.77205	0.08131	0.006
5627	219163_at	ZNF562	0.75203	0.81155	-0.05952	0.894
5628	219164_s_at	ATG2B	0.43282	0.57342	-0.1406	0.97
5629	219165_at	PDLIM2	0.15803	0.34172	-0.18369	0.918
5630	219166_at	C14orf104	0.60581	0.56942	0.03639	0.348
5631	219169_s_at	TFB1M	0.14242	0.31472	-0.1723	0.895
5632	219174_at	IFT74	0.30258	0.24826	0.05432	0.318
5633	219175_s_at	SLC41A3	0.24851	0.19657	0.05194	0.182
5634	219176_at	C2orf47	0.65341	0.50218	0.15123	0.009
5635	219177_at	BXDC2	0.38536	0.46071	-0.07535	0.768
5636	219178_at	QTRTD1	0.55504	0.26443	0.29061	<0.001
5637	219180_s_at	PEX26	0.49783	0.52555	-0.02772	0.609
5638	219187_at	FKBPL	0.016494	0.087697	-0.071203	0.701
5639	219189_at	FBXL6	0.48168	0.40412	0.07756	0.161
5640	219190_s_at	EIF2C4	0.21597	0.24821	-0.03224	0.613
5641	219191_s_at	BIN2	0.61764	0.55139	0.06625	0.224
5642	219192_at	UBAP2	0.7546	0.68136	0.07324	0.201
5643	219193_at	WDR70	0.25666	0.24788	0.00878	0.461
5644	219198_at	GTF3C4	0.49998	0.46017	0.03981	0.357
5645	219199_at	AFF4	0.23534	0.30532	-0.06998	0.727
5646	219200_at	FASTKD3	0.31853	0.19189	0.12664	0.123
5647	219201_s_at	TWSG1	0.43625	0.53723	-0.10098	0.838
5648	219202_at	RHBDF2	0.041985	0.37633	-0.334345	0.994
5649	219203_at	FAM158A	0.20283	0.37039	-0.16756	0.933
5650	219205_at	SRR	0.0096347	0.092768	-0.0831333	0.748
5651	219207_at	EDC3	0.11146	0.11418	-0.00272	0.506
5652	219210_s_at	RAB8B	0.60202	0.48585	0.11617	0.14
5653	219211_at	USP18	0.57475	0.55895	0.0158	0.418
5654	219212_at	HSPA14	0.55445	0.57264	-0.01819	0.625
5655	219213_at	JAM2	0.50401	0.56249	-0.05848	0.714
5656	219214_s_at	NT5C	0.44752	0.46586	-0.01834	0.573
5657	219215_s_at	SLC39A4	0.0056961	0.073956	-0.0682599	0.777
5658	219216_at	ETAA1	0.043084	0.133	-0.089916	0.731
5659	219217_at	NARS2	0.54387	0.54432	-0.00045	0.526
5660	219219_at	TMEM160	0.3053	0.20969	0.09561	0.205
5661	219221_at	ZBTB38	0.82294	0.85068	-0.02774	0.768
5662	219228_at	ZNF331	0.35186	0.40094	-0.04908	0.759
5663	219231_at	TGS1	0.46396	0.35565	0.10831	0.14
5664	219232_s_at	EGLN3	0.25823	0.32717	-0.06894	0.715
5665	219235_s_at	PHACTR4	0.35194	0.56981	-0.21787	0.977
5666	219237_s_at	DNAJB14	0.89703	0.89589	0.00114	0.488
5667	219238_at	PIGV	0.63916	0.17851	0.46065	<0.001
5668	219239_s_at	ZNF654	0.88722	0.87478	0.01244	0.363

5669	219240_s_at	C10orf88	0.30776	0.23159	0.07617	0.244
5670	219242_at	CEP63	0.17411	0.12665	0.04746	0.351
5671	219244_s_at	MRPL46	0.714	0.73426	-0.02026	0.631
5672	219246_s_at	OGFOD2	0.30596	0.49357	-0.18761	0.951
5673	219248_at	THUMPD2	0.46516	0.27901	0.18615	0.07
5674	219252_s_at	GEMIN8	0.24267	0.33171	-0.08904	0.76
5675	219253_at	TMEM185B	0.49972	0.40379	0.09593	0.169
5676	219254_at	C17orf101	0.41225	0.35817	0.05408	0.306
5677	219256_s_at	SH3TC1	0.58371	0.60641	-0.0227	0.606
5678	219258_at	TIPIN	0.78017	0.5418	0.23837	<0.001
5679	219259_at	SEMA4A	0.25496	0.53956	-0.2846	0.991
5680	219260_s_at	C17orf81	0.28393	0.58597	-0.30204	0.998
5681	219262_at	SUV39H2	0.75321	0.58709	0.16612	0.017
5682	219264_s_at	LOC100134089	0.53091	0.30808	0.22283	0.035
5683	219266_at	ZNF350	0.16436	0.25787	-0.09351	0.786
5684	219267_at	GLTP	0.57987	0.55355	0.02632	0.424
5685	219269_at	HMBOX1	0.3478	0.41743	-0.06963	0.754
5686	219270_at	CHAC1	0.34218	0.066079	0.276101	<0.001
5687	219274_at	TSPAN12	0.51172	0.65241	-0.14069	0.927
5688	219275_at	PDCD5	0.6755	0.80307	-0.12757	0.993
5689	219279_at	DOCK10	0.40669	0.49646	-0.08977	0.771
5690	219280_at	BRWD1	0.20885	0.22501	-0.01616	0.57
5691	219281_at	MSRA	0.14156	0.36742	-0.22586	0.966
5692	219282_s_at	TRPV2	0.5758	0.64246	-0.06666	0.778
5693	219283_at	C1GALT1C1	0.41824	0.49527	-0.07703	0.726
5694	219284_at	HSPBAP1	0.27142	0.16999	0.10143	0.242
5695	219286_s_at	RBM15	0.70332	0.64978	0.05354	0.231
5696	219287_at	KCNMB4	0.38772	0.39466	-0.00694	0.562
5697	219288_at	C3orf14	0.00060304	0.14171	-0.14110696	0.882
5698	219289_at	HEATR3	0.6988	0.59744	0.10136	0.005
5699	219291_at	DTWD1	0.32511	0.24897	0.07614	0.3
5700	219292_at	THAP1	0.80244	0.3731	0.42934	<0.001
5701	219293_s_at	OLA1	0.70843	0.79602	-0.08759	0.848
5702	219294_at	CENPQ	0.4677	0.6671	-0.1994	0.954
5703	219296_at	ZDHHC13	0.83055	0.77827	0.05228	0.116
5704	219297_at	WDR44	0.52596	0.35023	0.17573	0.041
5705	219298_at	ECHDC3	0.17449	0.28399	-0.1095	0.698
5706	219299_at	TRMT12	0.23688	0.14616	0.09072	0.249
5707	219303_at	RNF219	0.024481	0.16891	-0.144429	0.896
5708	219304_s_at	PDGFD	0.50415	0.60466	-0.10051	0.808
5709	219306_at	KIF15	0.80771	0.6873	0.12041	0.003
5710	219311_at	CEP76	0.60079	0.49293	0.10786	0.098
5711	219312_s_at	ZBTB10	0.42734	0.53482	-0.10748	0.822
5712	219317_at	POLI	0.48539	0.4582	0.02719	0.389
5713	219320_at	MYO19	0.43367	0.48868	-0.05501	0.72
5714	219321_at	MPP5	0.5327	0.44379	0.08891	0.185
5715	219322_s_at	WDR8	0.38883	0.48825	-0.09942	0.823
5716	219324_at	NOL12	0.71556	0.68245	0.03311	0.333
5717	219325_s_at	ELAC1	0.1607	0.15541	0.00529	0.502
5718	219326_s_at	B3GNT2	0.80585	0.7993	0.00655	0.474
5719	219329_s_at	C2orf28	0.8057	0.90057	-0.09487	0.945
5720	219330_at	VANGL1	0.20365	0.3491	-0.14545	0.838
5721	219334_s_at	OBFC2A	0.27268	0.36101	-0.08833	0.793
5722	219335_at	ARMCX5	0.0017392	0.049018	-0.0472788	0.666

5723	219336_s_at	ASCC1	0.58075	0.4288	0.15195	0.024
5724	219337_at	C1orf159	0.0025949	0.0021492	0.0004457	0.531
5725	219338_s_at	LRRC49	0.25833	0.44704	-0.18871	0.909
5726	219342_at	CASD1	0.017725	0.11129	-0.093565	0.781
5727	219343_at	CDC37L1	0.65148	0.57981	0.07167	0.226
5728	219345_at	BOLA1	0.48057	0.36317	0.1174	0.214
5729	219347_at	NUDT15	0.76531	0.63578	0.12953	0.013
5730	219348_at	USE1	0.53361	0.39148	0.14213	0.046
5731	219349_s_at	EXOC2	0.11713	0.11476	0.00237	0.499
5732	219350_s_at	DIABLO	0.77975	0.75623	0.02352	0.374
5733	219351_at	TRAPPC2	0.27739	0.28461	-0.00722	0.532
5734	219352_at	HERC6	0.6996	0.70573	-0.00613	0.541
5735	219353_at	NHLRC2	0.55479	0.60938	-0.05459	0.702
5736	219354_at	KLHL26	0.13525	0.2029	-0.06765	0.699
5737	219357_at	GTPBP1	0.16193	0.057676	0.104254	0.219
5738	219358_s_at	ADAP2	0.12506	0.21135	-0.08629	0.788
5739	219359_at	ATHL1	0.1668	0.42445	-0.25765	0.988
5740	219361_s_at	AEN	0.56428	0.61142	-0.04714	0.678
5741	219363_s_at	MTERFD1	0.57344	0.53324	0.0402	0.267
5742	219366_at	AVEN	0.40868	0.5227	-0.11402	0.842
5743	219368_at	NAP1L2	0.03394	0.20366	-0.16972	0.97
5744	219371_s_at	KLF2	0.65769	0.70375	-0.04606	0.742
5745	219372_at	IFT81	0.055436	0.16585	-0.110414	0.789
5746	219373_at	DPM3	0.69929	0.67915	0.02014	0.356
5747	219374_s_at	ALG9	0.34325	0.45658	-0.11333	0.853
5748	219375_at	CEPT1	0.37521	0.53494	-0.15973	0.919
5749	219376_at	ZNF322B	0.26549	0.5097	-0.24421	0.988
5750	219378_at	NARG1L	0.28779	0.36849	-0.0807	0.799
5751	219381_at	C5orf42	0.25376	0.2746	-0.02084	0.624
5752	219382_at	SERTAD3	0.10277	0.039341	0.063429	0.342
5753	219384_s_at	ADAT1	0.39071	0.56316	-0.17245	0.972
5754	219387_at	CCDC88A	0.81513	0.81053	0.0046	0.478
5755	219390_at	FKBP14	0.64181	0.38451	0.2573	<0.001
5756	219394_at	PGS1	0.27499	0.51759	-0.2426	0.987
5757	219397_at	COQ10B	0.43855	0.49916	-0.06061	0.745
5758	219398_at	CIDEC	7.88E-07	0.00076429	-0.000763502	0.488
5759	219400_at	CNTNAP1	0.36532	0.2038	0.16152	0.075
5760	219401_at	XYLT2	0.20728	0.24829	-0.04101	0.685
5761	219405_at	TRIM68	0.18153	0.31104	-0.12951	0.781
5762	219406_at	C1orf50	0.50816	0.38653	0.12163	0.17
5763	219408_at	PRMT7	0.068341	0.31083	-0.242489	0.987
5764	219409_at	SNIP1	0.042995	0.24742	-0.204425	0.967
5765	219410_at	TMEM45A	0.43441	0.51468	-0.08027	0.726
5766	219411_at	ELMO3	0.29874	0.22916	0.06958	0.294
5767	219412_at	RAB38	0.62524	0.64508	-0.01984	0.571
5768	219413_at	ACBD4	0.46245	0.2504	0.21205	0.029
5769	219417_s_at	C17orf59	0.27649	0.11844	0.15805	0.035
5770	219420_s_at	C1orf163	0.79216	0.75055	0.04161	0.243
5771	219421_at	TTC33	0.64717	0.69013	-0.04296	0.787
5772	219424_at	EBI3	0.77096	0.81825	-0.04729	0.782
5773	219426_at	EIF2C3	0.48992	0.54116	-0.05124	0.79
5774	219428_s_at	PXMP4	0.49437	0.43297	0.0614	0.255
5775	219429_at	FA2H	0.45005	0.42377	0.02628	0.425
5776	219431_at	ARHGAP10	0.29954	0.3371	-0.03756	0.632

5777	219433_at	BCOR	0.37922	0.58644	-0.20722	0.961
5778	219435_at	C17orf68	0.34381	0.28749	0.05632	0.32
5779	219437_s_at	ANKRD11	0.91665	0.89063	0.02602	0.204
5780	219439_at	C1GALT1	0.73227	0.79996	-0.06769	0.818
5781	219441_s_at	LRRK1	0.48695	0.48154	0.00541	0.486
5782	219442_at	C16orf67	0.63674	0.59227	0.04447	0.316
5783	219443_at	TASP1	0.47742	0.4461	0.03132	0.401
5784	219444_at	BCORL1	0.10758	0.2217	-0.11412	0.823
5785	219445_at	GLTSCR1	0.33824	0.16459	0.17365	0.071
5786	219446_at	RIC8B	0.29789	0.28349	0.0144	0.497
5787	219447_s_at	SLC35C2	0.26348	0.14697	0.11651	0.143
5788	219449_s_at	TMEM70	0.78769	0.79519	-0.0075	0.555
5789	219451_at	MSRB2	0.58423	0.51896	0.06527	0.23
5790	219453_at	KLHL36	0.08865	0.11753	-0.02888	0.581
5791	219458_s_at	NSUN3	0.54688	0.43794	0.10894	0.093
5792	219459_at	POLR3B	0.50676	0.53804	-0.03128	0.633
5793	219460_s_at	TMEM127	0.69961	0.4888	0.21081	0.003
5794	219462_at	TMEM53	0.38359	0.2623	0.12129	0.176
5795	219467_at	GIN1	0.42697	0.30902	0.11795	0.142
5796	219471_at	C13orf18	0.52255	0.57042	-0.04787	0.66
5797	219472_at	CENPO	0.33194	0.34055	-0.00861	0.598
5798	219473_at	GDAP2	0.30634	0.16655	0.13979	0.136
5799	219477_s_at	THSD1	0.17931	0.047454	0.131856	0.195
5800	219479_at	KDELC1	0.15114	0.23421	-0.08307	0.747
5801	219481_at	TTC13	0.29635	0.28721	0.00914	0.515
5802	219483_s_at	PORCN	0.46519	0.19459	0.2706	0.001
5803	219484_at	HCFC2	0.22478	0.3369	-0.11212	0.853
5804	219485_s_at	PSMD10	0.75497	0.74419	0.01078	0.457
5805	219486_at	DUS2L	0.00014115	0.0011082	-0.00096705	0.5
5806	219487_at	BBS10	0.2822	0.29018	-0.00798	0.525
5807	219489_s_at	NXN	0.21736	0.03435	0.18301	0.076
5808	219490_s_at	DCLRE1B	0.66849	0.4595	0.20899	0.009
5809	219492_at	CHIC2	0.70162	0.62969	0.07193	0.151
5810	219493_at	SHCBP1	0.8462	0.8199	0.0263	0.226
5811	219494_at	RAD54B	0.36775	0.28641	0.08134	0.261
5812	219495_s_at	ZNF180	0.027084	0.085191	-0.058107	0.674
5813	219496_at	ANKRD57	0.11446	0.29178	-0.17732	0.91
5814	219497_s_at	BCL11A	0.59717	0.67047	-0.0733	0.82
5815	219499_at	SEC61A2	0.41928	0.44706	-0.02778	0.626
5816	219501_at	ENOX1	0.27523	0.62693	-0.3517	0.986
5817	219502_at	NEIL3	0.43262	0.32854	0.10408	0.091
5818	219504_s_at	RPAP2	0.25349	0.40358	-0.15009	0.9
5819	219505_at	CECR1	0.56878	0.54678	0.022	0.412
5820	219506_at	C1orf54	0.011633	0.20587	-0.194237	0.947
5821	219507_at	RSRC1	0.87979	0.85264	0.02715	0.251
5822	219512_at	DSN1	0.70339	0.5938	0.10959	0.056
5823	219513_s_at	SH2D3A	0.15283	0.16709	-0.01426	0.563
5824	219515_at	PRDM10	0.25121	0.060048	0.191162	0.012
5825	219517_at	ELL3	0.53399	0.65016	-0.11617	0.879
5826	219520_s_at	WWC3	0.69092	0.73939	-0.04847	0.817
5827	219522_at	FJX1	0.56965	0.44088	0.12877	0.035
5828	219526_at	C14orf169	0.61464	0.57491	0.03973	0.314
5829	219530_at	PALB2	0.40653	0.26318	0.14335	0.038
5830	219531_at	CEP72	0.26583	0.29646	-0.03063	0.633

5831	219538_at	WDR5B	0.37904	0.19714	0.1819	0.069
5832	219539_at	GEMIN6	0.71986	0.56585	0.15401	0.019
5833	219540_at	ZNF267	0.80233	0.74434	0.05799	0.209
5834	219541_at	LIME1	0.69209	0.67198	0.02011	0.46
5835	219543_at	PBLD	0.45993	0.37032	0.08961	0.149
5836	219544_at	C13orf34	0.49918	0.43328	0.0659	0.233
5837	219548_at	ZNF16	0.28392	0.30653	-0.02261	0.571
5838	219549_s_at	RTN3	0.53718	0.54244	-0.00526	0.529
5839	219551_at	EAF2	0.48302	0.56528	-0.08226	0.751
5840	219559_at	SLC17A9	0.624	0.67467	-0.05067	0.763
5841	219560_at	C22orf29	0.2516	0.31962	-0.06802	0.736
5842	219563_at	C14orf139	0.44839	0.37353	0.07486	0.205
5843	219565_at	CYP20A1	0.065967	0.0090279	0.0569391	0.313
5844	219567_s_at	DEM1	0.24699	0.27928	-0.03229	0.593
5845	219570_at	KIF16B	0.18278	0.28071	-0.09793	0.83
5846	219571_s_at	ZNF12	0.84269	0.87578	-0.03309	0.841
5847	219575_s_at	COG8	0.65238	0.30969	0.34269	0.001
5848	219576_at	MAP7D3	0.73276	0.67453	0.05823	0.39
5849	219577_s_at	ABCA7	0.4458	0.61004	-0.16424	0.952
5850	219581_at	TSEN2	0.22979	0.094605	0.135185	0.091
5851	219582_at	OGFRL1	0.55218	0.65618	-0.104	0.857
5852	219583_s_at	SPATA7	0.36758	0.37756	-0.00998	0.566
5853	219584_at	PLA1A	0.80976	0.86163	-0.05187	0.885
5854	219588_s_at	NCAPG2	0.62101	0.62153	-0.00052	0.531
5855	219593_at	SLC15A3	0.42461	0.46687	-0.04226	0.643
5856	219594_at	NINJ2	0.10492	0.32612	-0.2212	0.992
5857	219595_at	ZNF26	0.14116	0.34945	-0.20829	0.928
5858	219596_at	THAP10	0.043822	0.29625	-0.252428	0.99
5859	219598_s_at	RWDD1	0.76486	0.6471	0.11776	0.051
5860	219600_s_at	TMEM50B	0.4929	0.4324	0.0605	0.243
5861	219602_s_at	FAM38B	0.25903	0.41841	-0.15938	0.898
5862	219603_s_at	ZNF226	0.31397	0.37471	-0.06074	0.675
5863	219609_at	WDR25	0.28851	0.29582	-0.00731	0.561
5864	219613_s_at	SIRT6	0.19968	0.29412	-0.09444	0.785
5865	219617_at	C2orf34	0.25874	0.36829	-0.10955	0.827
5866	219618_at	IRAK4	0.5724	0.51428	0.05812	0.269
5867	219622_at	RAB20	0.32036	0.53598	-0.21562	0.967
5868	219624_at	BAG4	0.37101	0.33632	0.03469	0.424
5869	219625_s_at	COL4A3BP	0.73742	0.74101	-0.00359	0.544
5870	219627_at	ZNF767	0.40805	0.3206	0.08745	0.217
5871	219628_at	ZMAT3	0.73589	0.79799	-0.0621	0.827
5872	219629_at	FAM118A	0.23941	0.3045	-0.06509	0.716
5873	219632_s_at	TRPV1	0.037289	0.17557	-0.138281	0.873
5874	219633_at	TTPAL	0.56561	0.5947	-0.02909	0.618
5875	219634_at	CHST11	0.72338	0.69284	0.03054	0.337
5876	219635_at	ZNF606	0.27629	0.25253	0.02376	0.433
5877	219636_s_at	ARMC9	0.24148	0.3597	-0.11822	0.658
5878	219640_at	CLDN15	0.26523	0.25928	0.00595	0.486
5879	219641_at	DET1	0.3464	0.42063	-0.07423	0.755
5880	219644_at	CCDC41	0.72481	0.6718	0.05301	0.206
5881	219646_at	DEF8	0.36826	0.47176	-0.1035	0.843
5882	219648_at	MREG	0.81894	0.80382	0.01512	0.434
5883	219649_at	ALG6	0.37372	0.40058	-0.02686	0.582
5884	219650_at	ERCC6L	0.67852	0.38524	0.29328	<0.001

5885	219653_at	LSM14B	0.27964	0.30235	-0.02271	0.576
5886	219657_s_at	KLF3	0.33311	0.408	-0.07489	0.726
5887	219662_at	C2orf49	0.514	0.58484	-0.07084	0.77
5888	219665_at	NUDT18	0.67988	0.6153	0.06458	0.276
5889	219667_s_at	BANK1	0.40872	0.47766	-0.06894	0.697
5890	219673_at	MCM9	0.42202	0.45293	-0.03091	0.626
5891	219675_s_at	UXS1	0.57076	0.40139	0.16937	0.025
5892	219676_at	ZSCAN16	0.24261	0.37757	-0.13496	0.85
5893	219680_at	NLRX1	0.23795	0.17631	0.06164	0.321
5894	219681_s_at	RAB11FIP1	0.53945	0.57776	-0.03831	0.649
5895	219683_at	FZD3	0.4654	0.58359	-0.11819	0.771
5896	219684_at	RTP4	0.69925	0.72694	-0.02769	0.619
5897	219688_at	BBS7	0.24838	0.20939	0.03899	0.37
5898	219690_at	TMEM149	0.40099	0.40375	-0.00276	0.523
5899	219691_at	SAMD9	0.39876	0.3132	0.08556	0.188
5900	219696_at	DENND1B	0.32983	0.45254	-0.12271	0.849
5901	219698_s_at	METTL4	0.33463	0.31857	0.01606	0.432
5902	219702_at	PLAC1	0.12044	0.076628	0.043812	0.336
5903	219703_at	MNS1	0.39272	0.47943	-0.08671	0.832
5904	219705_at	QSER1	0.1811	0.18244	-0.00134	0.509
5905	219706_at	C20orf29	0.13235	0.29133	-0.15898	0.891
5906	219711_at	ZNF586	0.20716	0.30599	-0.09883	0.783
5907	219713_at	SHPK	0.23489	0.10789	0.127	0.163
5908	219715_s_at	TDP1	0.34832	0.32434	0.02398	0.393
5909	219716_at	APOL6	0.44219	0.50713	-0.06494	0.727
5910	219717_at	C4orf30	0.89561	0.89404	0.00157	0.5
5911	219718_at	FGGY	0.25585	0.30302	-0.04717	0.675
5912	219720_s_at	C14orf118	0.36656	0.2022	0.16436	0.103
5913	219724_s_at	KIAA0748	0.26153	0.21317	0.04836	0.339
5914	219731_at	FLJ34077	0.24785	0.35143	-0.10358	0.802
5915	219733_s_at	SLC27A5	0.38074	0.372	0.00874	0.453
5916	219740_at	VASH2	0.54139	0.6403	-0.09891	0.837
5917	219742_at	PRR7	0.16258	0.11245	0.05013	0.354
5918	219751_at	SETD6	0.61885	0.46775	0.1511	0.057
5919	219753_at	STAG3	0.47068	0.56885	-0.09817	0.841
5920	219754_at	RBM41	0.46564	0.56489	-0.09925	0.799
5921	219757_s_at	C14orf101	0.83189	0.86157	-0.02968	0.809
5922	219758_at	TTC26	0.0025081	0.058236	-0.0557279	0.679
5923	219759_at	ERAP2	0.028596	0.18868	-0.160084	0.924
5924	219762_s_at	RPL36	0.86423	0.85809	0.00614	0.428
5925	219763_at	DENND1A	0.18157	0.30169	-0.12012	0.815
5926	219765_at	ZNF329	0.27475	0.41921	-0.14446	0.92
5927	219767_s_at	CRYZL1	0.47565	0.61568	-0.14003	0.831
5928	219770_at	GTDC1	0.30404	0.53955	-0.23551	0.948
5929	219774_at	CCDC93	0.20265	0.22673	-0.02408	0.568
5930	219777_at	GIMAP6	0.7255	0.79149	-0.06599	0.85
5931	219783_at	C2orf18	0.37807	0.21784	0.16023	0.116
5932	219785_s_at	FBXO31	0.52063	0.39853	0.1221	0.071
5933	219787_s_at	ECT2	0.73304	0.68518	0.04786	0.207
5934	219788_at	PILRA	0.44881	0.597	-0.14819	0.944
5935	219793_at	SNX16	0.34352	0.32137	0.02215	0.457
5936	219797_at	MGAT4A	0.29909	0.33677	-0.03768	0.662
5937	219798_s_at	MEPCE	0.21505	0.231	-0.01595	0.545
5938	219800_s_at	THNSL1	0.33047	0.16226	0.16821	0.033

5939	219801_at	ZNF34	0.32708	0.24377	0.08331	0.225
5940	219805_at	CXorf56	0.44817	0.3613	0.08687	0.216
5941	219806_s_at	C11orf75	0.23202	0.31296	-0.08094	0.752
5942	219809_at	WDR55	0.16912	0.099064	0.070056	0.295
5943	219812_at	PVRIG	0.19353	0.45262	-0.25909	0.994
5944	219814_at	MBNL3	0.37938	0.38826	-0.00888	0.55
5945	219816_s_at	RBM23	0.22929	0.36288	-0.13359	0.79
5946	219817_at	C12orf47	0.39745	0.15883	0.23862	0.024
5947	219818_s_at	GPATCH1	0.43936	0.43173	0.00763	0.46
5948	219819_s_at	MRPS28	0.87683	0.84795	0.02888	0.229
5949	219821_s_at	GFOD1	0.19601	0.18206	0.01395	0.436
5950	219822_at	MTRF1	0.27021	0.26467	0.00554	0.461
5951	219828_at	C9orf86	0.18842	0.33525	-0.14683	0.914
5952	219831_at	CDKL3	0.1661	0.29233	-0.12623	0.854
5953	219833_s_at	EFHC1	0.52064	0.54637	-0.02573	0.627
5954	219834_at	ALS2CR8	0.45254	0.49132	-0.03878	0.643
5955	219838_at	TTC23	0.049134	0.23426	-0.185126	0.934
5956	219841_at	AICDA	0.38943	0.44041	-0.05098	0.722
5957	219842_at	ARL15	0.32447	0.52976	-0.20529	0.993
5958	219843_at	IPP	0.43403	0.493	-0.05897	0.698
5959	219848_s_at	ZNF432	0.24911	0.1882	0.06091	0.334
5960	219849_at	ZNF671	0.44363	0.29291	0.15072	0.083
5961	219854_at	ZNF14	0.64493	0.69816	-0.05323	0.759
5962	219858_s_at	MFSD6	0.83307	0.85505	-0.02198	0.675
5963	219860_at	LY6G5C	0.048357	0.2178	-0.169443	0.825
5964	219861_at	DNAJC17	0.54429	0.58993	-0.04564	0.697
5965	219862_s_at	NARF	0.43224	0.33929	0.09295	0.217
5966	219863_at	HERC5	0.67801	0.75904	-0.08103	0.806
5967	219865_at	HSPC157	0.11363	0.33298	-0.21935	0.948
5968	219868_s_at	ANKFY1	0.36573	0.34513	0.0206	0.422
5969	219870_at	ATF7IP2	0.2649	0.34672	-0.08182	0.798
5970	219874_at	SLC12A8	0.59813	0.6378	-0.03967	0.718
5971	219876_s_at	GOLGA2L1	0.32571	0.28071	0.045	0.382
5972	219878_s_at	KLF13	0.89357	0.89544	-0.00187	0.534
5973	219885_at	SLFN12	0.067323	0.15391	-0.086587	0.705
5974	219888_at	SPAG4	0.19712	0.25044	-0.05332	0.669
5975	219889_at	FRAT1	0.1864	0.39231	-0.20591	0.974
5976	219891_at	PGPEP1	0.17274	0.22032	-0.04758	0.685
5977	219892_at	TM6SF1	0.26493	0.46151	-0.19658	0.965
5978	219901_at	FGD6	0.37564	0.43687	-0.06123	0.724
5979	219904_at	ZSCAN5A	0.35755	0.2865	0.07105	0.3
5980	219905_at	ERMAP	0.078248	0.35424	-0.275992	0.978
5981	219906_at	FLJ10213	0.57483	0.63695	-0.06212	0.763
5982	219910_at	FICD	0.73638	0.54893	0.18745	<0.001
5983	219911_s_at	LOC100134295	0.68751	0.67847	0.00904	0.469
5984	219913_s_at	CRNKL1	0.35194	0.4968	-0.14486	0.871
5985	219915_s_at	SLC16A10	0.41786	0.36837	0.04949	0.401
5986	219917_at	ZCCHC4	0.17709	0.16182	0.01527	0.465
5987	219918_s_at	ASPM	0.66236	0.67765	-0.01529	0.569
5988	219920_s_at	GMPPB	0.59439	0.49031	0.10408	0.037
5989	219922_s_at	LTBP3	0.13809	0.33646	-0.19837	0.926
5990	219923_at	TRIM45	0.24916	0.22531	0.02385	0.452
5991	219924_s_at	ZMYM6	0.70299	0.80245	-0.09946	0.904
5992	219929_s_at	ZFYVE21	0.49335	0.51808	-0.02473	0.593

5993	219931_s_at	KLHL12	0.47354	0.15639	0.31715	0.003
5994	219933_at	GLRX2	0.60549	0.49208	0.11341	0.125
5995	219938_s_at	PSTPIP2	0.46649	0.56594	-0.09945	0.829
5996	219939_s_at	CSDE1	0.74826	0.74077	0.00749	0.447
5997	219940_s_at	PCID2	0.77505	0.65321	0.12184	0.029
5998	219941_at	TMEM19	0.69405	0.73969	-0.04564	0.73
5999	219944_at	CLIP4	0.39292	0.50194	-0.10902	0.813
6000	219947_at	CLEC4A	0.067358	0.25659	-0.189232	0.952
6001	219951_s_at	C20orf12	0.16686	0.34891	-0.18205	0.911
6002	219952_s_at	MCOLN1	0.11399	0.096538	0.017452	0.421
6003	219956_at	GALNT6	0.37172	0.44877	-0.07705	0.729
6004	219957_at	RUFY2	0.68253	0.78602	-0.10349	0.973
6005	219959_at	MOCOS	0.030851	0.15124	-0.120389	0.834
6006	219960_s_at	UCHL5	0.7271	0.75253	-0.02543	0.637
6007	219961_s_at	NCRNA00153	0.42733	0.51076	-0.08343	0.866
6008	219967_at	MRM1	0.27801	0.11853	0.15948	0.047
6009	219968_at	ZNF589	0.17751	0.31678	-0.13927	0.916
6010	219969_at	CXorf15	0.59536	0.62329	-0.02793	0.652
6011	219971_at	IL21R	0.48331	0.3274	0.15591	0.036
6012	219972_s_at	C14orf135	0.50292	0.60292	-0.1	0.845
6013	219976_at	HOOK1	0.26988	0.45443	-0.18455	0.867
6014	219979_s_at	C11orf73	0.54751	0.67684	-0.12933	0.956
6015	219980_at	C4orf29	0.57635	0.552	0.02435	0.405
6016	219982_s_at	SERF1A	0.20211	0.34681	-0.1447	0.882
6017	219986_s_at	ACAD10	0.40579	0.3375	0.06829	0.326
6018	219988_s_at	RNF220	0.63522	0.58963	0.04559	0.35
6019	219990_at	E2F8	0.84222	0.74329	0.09893	0.015
6020	219994_at	APBB1IP	0.3724	0.56501	-0.19261	0.97
6021	219996_at	ASB7	0.086981	0.096324	-0.009343	0.506
6022	219997_s_at	COPS7B	0.52432	0.40779	0.11653	0.135
6023	219998_at	HSPC159	0.64461	0.61976	0.02485	0.439
6024	219999_at	MAN2A2	0.23776	0.40239	-0.16463	0.904
6025	220002_at	KIF26B	0.34289	0.37156	-0.02867	0.636
6026	220007_at	METTL8	0.73326	0.71718	0.01608	0.44
6027	220011_at	C1orf135	0.51345	0.55395	-0.0405	0.757
6028	220012_at	ERO1LB	0.52348	0.51713	0.00635	0.474
6029	220015_at	CASZ1	0.4871	0.33345	0.15365	0.055
6030	220018_at	CBLL1	0.71193	0.76742	-0.05549	0.8
6031	220019_s_at	ZNF224	0.41875	0.37488	0.04387	0.348
6032	220020_at	XPNPEP3	0.32366	0.44725	-0.12359	0.853
6033	220028_at	ACVR2B	0.34064	0.27639	0.06425	0.317
6034	220034_at	IRAK3	0.28721	0.48197	-0.19476	0.94
6035	220035_at	NUP210	0.76653	0.77949	-0.01296	0.575
6036	220036_s_at	LMBR1L	0.35644	0.28062	0.07582	0.268
6037	220038_at	C8orf44	0.29995	0.4943	-0.19435	0.979
6038	220041_at	PIGZ	0.028961	0.10187	-0.072909	0.687
6039	220046_s_at	CCNL1	0.85272	0.89018	-0.03746	0.886
6040	220050_at	C9orf9	0.52885	0.37456	0.15429	0.085
6041	220052_s_at	TINF2	0.47736	0.59083	-0.11347	0.854
6042	220054_at	IL23A	0.20602	0.34674	-0.14072	0.932
6043	220058_at	C17orf39	0.23542	0.3019	-0.06648	0.692
6044	220059_at	STAP1	0.46645	0.64073	-0.17428	0.972
6045	220060_s_at	C12orf48	0.79491	0.72846	0.06645	0.14
6046	220063_at	GSTCD	0.60422	0.64611	-0.04189	0.748

6047	220066_at	NOD2	0.1857	0.27217	-0.08647	0.717
6048	220068_at	VPREB3	0.67624	0.74045	-0.06421	0.839
6049	220079_s_at	USP48	0.37852	0.50464	-0.12612	0.888
6050	220085_at	HELLS	0.78652	0.83716	-0.05064	0.822
6051	220086_at	IKZF5	0.21968	0.21952	0.00016	0.502
6052	220089_at	L2HGDH	0.333	0.1042	0.2288	0.03
6053	220091_at	SLC2A6	0.55798	0.66754	-0.10956	0.875
6054	220094_s_at	CCDC90A	0.44787	0.65893	-0.21106	0.973
6055	220099_s_at	LUC7L2	0.28605	0.30066	-0.01461	0.557
6056	220103_s_at	MRPS18C	0.18054	0.055187	0.125353	0.125
6057	220104_at	ZC3HAV1	0.10734	0.23022	-0.12288	0.951
6058	220118_at	ZBTB32	0.42064	0.32596	0.09468	0.09
6059	220121_at	LINS1	0.3434	0.076651	0.266749	0.1
6060	220122_at	MCTP1	0.33206	0.36023	-0.02817	0.61
6061	220123_at	SLC35F5	0.06979	0.17309	-0.1033	0.841
6062	220127_s_at	FBXL12	0.38379	0.37365	0.01014	0.459
6063	220132_s_at	CLEC2D	0.67527	0.74144	-0.06617	0.819
6064	220145_at	MAP9	0.51899	0.53831	-0.01932	0.584
6065	220146_at	TLR7	0.23509	0.17782	0.05727	0.279
6066	220147_s_at	FAM60A	0.742	0.7646	-0.0226	0.696
6067	220148_at	ALDH8A1	0.13526	0.31169	-0.17643	0.878
6068	220153_at	ENTPD7	0.13928	0.31852	-0.17924	0.935
6069	220155_s_at	BRD9	0.20062	0.30955	-0.10893	0.875
6070	220158_at	LGALS14	0.57109	0.67923	-0.10814	0.893
6071	220159_at	ABCA11P	0.20018	0.45847	-0.25829	0.98
6072	220161_s_at	EPB41L4B	0.30668	0.3975	-0.09082	0.768
6073	220169_at	TMEM156	0.27427	0.48309	-0.20882	0.978
6074	220172_at	C2orf37	0.40892	0.44904	-0.04012	0.667
6075	220175_s_at	CBWD1	0.74496	0.67817	0.06679	0.12
6076	220176_at	NUBPL	0.66864	0.66284	0.0058	0.478
6077	220178_at	C19orf28	0.19831	0.20668	-0.00837	0.498
6078	220182_at	SLC25A23	0.058079	0.096007	-0.037928	0.634
6079	220183_s_at	NUDT6	0.21965	0.48461	-0.26496	0.986
6080	220189_s_at	MGAT4B	0.74683	0.61618	0.13065	0.008
6081	220195_at	MBD5	0.61011	0.42378	0.18633	0.004
6082	220199_s_at	AIDA	0.62109	0.54063	0.08046	0.211
6083	220200_s_at	SETD8	0.67279	0.61189	0.0609	0.306
6084	220201_at	RC3H2	0.63111	0.43781	0.1933	0.045
6085	220212_s_at	THADA	0.076234	0.26885	-0.192616	0.954
6086	220214_at	ZNF215	0.017436	0.081352	-0.063916	0.726
6087	220215_at	ZNF669	0.80392	0.73085	0.07307	0.12
6088	220219_s_at	LOC100133503	0.68882	0.53468	0.15414	0.009
6089	220223_at	ATAD5	0.92695	0.78652	0.14043	<0.001
6090	220230_s_at	CYB5R2	0.12364	0.40862	-0.28498	0.98
6091	220235_s_at	C1orf103	0.62212	0.52346	0.09866	0.024
6092	220236_at	PDPR	0.074673	0.049258	0.025415	0.423
6093	220238_s_at	KLHL7	0.588	0.66756	-0.07956	0.909
6094	220241_at	TMCO3	0.50895	0.31928	0.18967	0.014
6095	220244_at	LOH3CR2A	0.2364	0.35814	-0.12174	0.845
6096	220246_at	CAMK1D	0.2676	0.3828	-0.1152	0.75
6097	220250_at	ZNF286A	0.42199	0.48572	-0.06373	0.713
6098	220251_at	C1orf107	0.11987	0.15584	-0.03597	0.62
6099	220255_at	FANCE	0.65426	0.647	0.00726	0.477
6100	220260_at	TBC1D19	0.37624	0.31366	0.06258	0.225

6101	220261_s_at	ZDHHC4	0.26953	0.22699	0.04254	0.349
6102	220278_at	KDM4D	0.15182	0.080199	0.071621	0.293
6103	220285_at	FAM108B1	0.62429	0.65937	-0.03508	0.661
6104	220287_at	ADAMTS9	0.10729	0.23724	-0.12995	0.811
6105	220288_at	MYO15A	0.15804	0.20394	-0.0459	0.642
6106	220305_at	MAVS	0.073381	0.33913	-0.265749	0.984
6107	220311_at	N6AMT1	0.51976	0.44739	0.07237	0.227
6108	220315_at	PARP11	0.26434	0.21998	0.04436	0.318
6109	220319_s_at	MYLIP	0.34012	0.29841	0.04171	0.381
6110	220329_s_at	RMND1	0.36223	0.30652	0.05571	0.29
6111	220330_s_at	SAMSN1	0.53859	0.68653	-0.14794	0.919
6112	220346_at	MTHFD2L	0.27481	0.29238	-0.01757	0.588
6113	220349_s_at	ENGASE	0.20706	0.35932	-0.15226	0.895
6114	220353_at	FAM86C	0.24131	0.19739	0.04392	0.391
6115	220355_s_at	PBRM1	0.76789	0.8043	-0.03641	0.787
6116	220358_at	BATF3	0.2224	0.51931	-0.29691	0.999
6117	220367_s_at	SAP130	0.56416	0.58212	-0.01796	0.592
6118	220368_s_at	SMEK1	0.78611	0.82412	-0.03801	0.752
6119	220370_s_at	USP36	0.5016	0.6134	-0.1118	0.887
6120	220371_s_at	SLC12A9	0.27371	0.19798	0.07573	0.288
6121	220372_at	DNAJC28	0.26084	0.10016	0.16068	0.063
6122	220375_s_at	H2AFY	0.25839	0.23773	0.02066	0.436
6123	220386_s_at	EML4	0.73734	0.62007	0.11727	0.038
6124	220387_s_at	HHLA3	0.55387	0.65409	-0.10022	0.838
6125	220390_at	AGBL2	0.25629	0.28588	-0.02959	0.596
6126	220391_at	ZBTB3	0.080639	0.16303	-0.082391	0.75
6127	220399_at	NCRNA00115	0.36255	0.29016	0.07239	0.316
6128	220417_s_at	THAP4	0.54861	0.45022	0.09839	0.029
6129	220419_s_at	USP25	0.53276	0.5653	-0.03254	0.608
6130	220444_at	ZNF557	0.13041	0.17177	-0.04136	0.656
6131	220446_s_at	CHST4	0.29429	0.29906	-0.00477	0.525
6132	220450_at	SMAD1	0.52062	0.70824	-0.18762	0.867
6133	220458_at	FLJ10246	0.57906	0.68272	-0.10366	0.861
6134	220459_at	MCM3APAS	0.62147	0.34585	0.27562	<0.001
6135	220465_at	LOC80054	0.4855	0.47109	0.01441	0.45
6136	220466_at	CCDC15	0.59444	0.67629	-0.08185	0.835
6137	220467_at	FLJ21272	0.58789	0.76696	-0.17907	0.996
6138	220470_at	BET1L	0.42833	0.40463	0.0237	0.444
6139	220477_s_at	C20orf30	0.65989	0.82226	-0.16237	0.928
6140	220482_s_at	SERGEF	0.020166	0.0014213	0.0187447	0.412
6141	220484_at	MCOLN3	0.26535	0.40412	-0.13877	0.834
6142	220488_s_at	BCAS3	0.49633	0.54457	-0.04824	0.672
6143	220491_at	HAMP	0.22866	0.24294	-0.01428	0.559
6144	220494_s_at	C14orf43	0.56872	0.62137	-0.05265	0.701
6145	220495_s_at	TXNDC15	0.52116	0.45729	0.06387	0.197
6146	220500_s_at	RABL2A	0.41064	0.3919	0.01874	0.447
6147	220525_s_at	AUP1	0.82206	0.85608	-0.03402	0.768
6148	220534_at	TRIM48	0.12256	0.21017	-0.08761	0.746
6149	220547_s_at	FAM35A	0.49712	0.4713	0.02582	0.411
6150	220550_at	FBXO4	0.25846	0.16041	0.09805	0.363
6151	220553_s_at	PRPF39	0.77765	0.74011	0.03754	0.348
6152	220565_at	CCR10	0.51073	0.62285	-0.11212	0.805
6153	220566_at	PIK3R5	0.55667	0.45472	0.10195	0.106
6154	220572_at	DKFZp547G183	0.62597	0.47195	0.15402	0.041

6155	220577_at	GVIN1	0.64234	0.73491	-0.09257	0.893
6156	220586_at	CHD9	0.46887	0.50037	-0.0315	0.638
6157	220587_s_at	GBL	0.52512	0.45191	0.07321	0.263
6158	220588_at	BCAS4	0.41263	0.63411	-0.22148	0.97
6159	220590_at	ITFG2	0.71743	0.76883	-0.0514	0.857
6160	220597_s_at	ARL6IP4	0.62179	0.36952	0.25227	0.049
6161	220600_at	C3orf75	0.32668	0.39773	-0.07105	0.778
6162	220602_s_at	LOC388152	0.073054	0.16071	-0.087656	0.724
6163	220603_s_at	MCTP2	0.72473	0.78905	-0.06432	0.899
6164	220605_s_at	SIRT2	0.27617	0.319	-0.04283	0.622
6165	220606_s_at	C17orf48	0.63109	0.68758	-0.05649	0.727
6166	220608_s_at	ZNF770	0.39047	0.46586	-0.07539	0.767
6167	220609_at	LOC202181	0.57457	0.42583	0.14874	0.072
6168	220615_s_at	FAR2	0.63163	0.70141	-0.06978	0.773
6169	220631_at	OSGEPL1	0.48696	0.19374	0.29322	0.001
6170	220633_s_at	HP1BP3	0.28274	0.2799	0.00284	0.516
6171	220643_s_at	FAIM	0.58236	0.6802	-0.09784	0.855
6172	220647_s_at	CHCHD8	0.73257	0.53817	0.1944	<0.001
6173	220651_s_at	MCM10	0.74072	0.54714	0.19358	<0.001
6174	220658_s_at	ARNTL2	0.6011	0.73689	-0.13579	0.957
6175	220661_s_at	ZNF692	0.45094	0.42523	0.02571	0.408
6176	220668_s_at	DNMT3B	0.12159	0.15693	-0.03534	0.62
6177	220671_at	CCRN4L	0.31602	0.20628	0.10974	0.221
6178	220682_s_at	KLHL5	0.83487	0.81665	0.01822	0.371
6179	220685_at	FAM120C	0.095483	0.045108	0.050375	0.341
6180	220688_s_at	MRTO4	0.77996	0.67635	0.10361	0.023
6181	220690_s_at	DHRS7B	0.37544	0.59135	-0.21591	0.962
6182	220703_at	C10orf110	0.23367	0.058715	0.174955	0.096
6183	220712_at	C8orf60	0.49263	0.45212	0.04051	0.305
6184	220721_at	ZNF614	0.031787	0.026208	0.005579	0.482
6185	220731_s_at	NECAP2	0.73777	0.74568	-0.00791	0.562
6186	220734_s_at	GLTPD1	0.45855	0.47071	-0.01216	0.555
6187	220735_s_at	SENP7	0.48277	0.60268	-0.11991	0.931
6188	220739_s_at	CNNM3	0.66874	0.73279	-0.06405	0.776
6189	220740_s_at	SLC12A6	0.58081	0.62002	-0.03921	0.711
6190	220741_s_at	PPA2	0.56418	0.71069	-0.14651	0.945
6191	220746_s_at	UIMC1	0.38579	0.40166	-0.01587	0.568
6192	220748_s_at	ZNF580	0.67714	0.6005	0.07664	0.161
6193	220750_s_at	LEPRE1	0.3532	0.2372	0.116	0.254
6194	220753_s_at	CRYL1	0.32255	0.49448	-0.17193	0.89
6195	220755_s_at	C6orf48	0.72927	0.62577	0.1035	0.071
6196	220757_s_at	UBXN6	0.41033	0.36841	0.04192	0.374
6197	220761_s_at	TAOK3	0.47061	0.42454	0.04607	0.308
6198	220762_s_at	GNB1L	0.49649	0.3275	0.16899	0.127
6199	220768_s_at	CSNK1G3	0.72168	0.69459	0.02709	0.331
6200	220770_s_at	C5orf54	0.35284	0.23597	0.11687	0.179
6201	220773_s_at	GPHN	0.52996	0.45701	0.07295	0.228
6202	220774_at	DYM	0.59146	0.66556	-0.0741	0.834
6203	220775_s_at	UEVLD	0.71118	0.55748	0.1537	0.018
6204	220776_at	KCNJ14	0.14095	0.046478	0.094472	0.209
6205	220788_s_at	IRF9	0.31416	0.35711	-0.04295	0.649
6206	220789_s_at	TBRG4	0.3255	0.20035	0.12515	0.06
6207	220797_at	METT10D	0.66869	0.70604	-0.03735	0.672
6208	220800_s_at	TMOD3	0.70154	0.7307	-0.02916	0.696

6209	220840_s_at	C1orf112	0.66742	0.52536	0.14206	0.029
6210	220864_s_at	NDUFA13	0.87698	0.81885	0.05813	0.017
6211	220865_s_at	PDSS1	0.68968	0.69359	-0.00391	0.538
6212	220885_s_at	CENPJ	0.88977	0.86598	0.02379	0.209
6213	220890_s_at	DDX47	0.58719	0.62782	-0.04063	0.736
6214	220892_s_at	PSAT1	0.81656	0.5017	0.31486	<0.001
6215	220917_s_at	WDR19	0.4961	0.58292	-0.08682	0.828
6216	220924_s_at	SLC38A2	0.33168	0.46355	-0.13187	0.956
6217	220925_at	MAK10	0.8338	0.79735	0.03645	0.259
6218	220926_s_at	EDEM3	0.53799	0.66081	-0.12282	0.999
6219	220933_s_at	ZCCHC6	0.60118	0.59772	0.00346	0.487
6220	220934_s_at	TMEM223	0.75393	0.71976	0.03417	0.321
6221	220935_s_at	CDK5RAP2	0.66197	0.43404	0.22793	0.019
6222	220936_s_at	H2AFJ	0.26814	0.22117	0.04697	0.463
6223	220937_s_at	ST6GALNAC4	0.487	0.60088	-0.11388	0.929
6224	220939_s_at	DPP8	0.49688	0.4862	0.01068	0.49
6225	220940_at	ANKRD36B	0.88344	0.92311	-0.03967	0.888
6226	220941_s_at	C21orf91	0.61414	0.53036	0.08378	0.172
6227	220943_s_at	C2orf56	0.41144	0.45534	-0.0439	0.666
6228	220944_at	PGLYRP4	0.11408	0.2508	-0.13672	0.886
6229	220946_s_at	SETD2	0.88555	0.87388	0.01167	0.355
6230	220947_s_at	TBC1D10B	0.58123	0.65533	-0.0741	0.839
6231	220948_s_at	ATP1A1	0.57068	0.67698	-0.1063	0.875
6232	220949_s_at	C7orf49	0.68838	0.55485	0.13353	0.093
6233	220953_s_at	MTMR12	0.26194	0.24454	0.0174	0.458
6234	220954_s_at	PILRB	0.58787	0.65132	-0.06345	0.856
6235	220956_s_at	EGLN2	0.28688	0.43405	-0.14717	0.908
6236	220964_s_at	RAB1B	0.4859	0.39242	0.09348	0.227
6237	220973_s_at	SHARPIN	0.24092	0.17986	0.06106	0.331
6238	220980_s_at	ADPGK	0.21294	0.33468	-0.12174	0.836
6239	220984_s_at	SLCO5A1	0.49807	0.49248	0.00559	0.496
6240	220985_s_at	RNF170	0.52719	0.6777	-0.15051	0.943
6241	220987_s_at	C11orf17	0.58002	0.5894	-0.00938	0.533
6242	220988_s_at	C1QTNF3	0.1814	0.17752	0.00388	0.499
6243	220990_s_at	MIR21	0.58185	0.652	-0.07015	0.846
6244	220991_s_at	RNF32	0.32049	0.39631	-0.07582	0.866
6245	220992_s_at	C1orf25	0.25568	0.47783	-0.22215	0.978
6246	220993_s_at	GPR63	0.27049	0.21861	0.05188	0.387
6247	221002_s_at	TSPAN14	0.31351	0.18077	0.13274	0.159
6248	221004_s_at	ITM2C	0.33068	0.42323	-0.09255	0.806
6249	221006_s_at	SNX27	0.81506	0.76026	0.0548	0.14
6250	221007_s_at	FIP1L1	0.43862	0.43784	0.00078	0.516
6251	221011_s_at	LBH	0.63631	0.69901	-0.0627	0.774
6252	221012_s_at	TRIM8	0.53964	0.36735	0.17229	0.081
6253	221014_s_at	RAB33B	0.42157	0.478	-0.05643	0.752
6254	221015_s_at	CDADC1	0.044684	0.052317	-0.007633	0.554
6255	221020_s_at	SLC25A32	0.81518	0.69252	0.12266	0.022
6256	221021_s_at	CTNNBL1	0.56674	0.10924	0.4575	<0.001
6257	221027_s_at	PLA2G12A	0.70493	0.79537	-0.09044	0.973
6258	221031_s_at	APOLD1	0.56418	0.57415	-0.00997	0.567
6259	221036_s_at	APH1B	0.28313	0.38532	-0.10219	0.791
6260	221039_s_at	ASAP1	0.56992	0.60279	-0.03287	0.598
6261	221041_s_at	SLC17A5	0.57655	0.4098	0.16675	0.048
6262	221042_s_at	CLMN	0.35621	0.51517	-0.15896	0.838

6263	221044_s_at	TRIM34	0.36698	0.47063	-0.10365	0.852
6264	221046_s_at	GTPBP8	0.46665	0.63784	-0.17119	0.963
6265	221050_s_at	GTPBP2	0.38419	0.34921	0.03498	0.386
6266	221053_s_at	TDRKH	0.023748	0.28049	-0.256742	1
6267	221058_s_at	CKLF	0.51883	0.5919	-0.07307	0.815
6268	221059_s_at	COTL1	0.37099	0.50061	-0.12962	0.89
6269	221069_s_at	CCDC44	0.56441	0.37632	0.18809	0.062
6270	221073_s_at	NOD1	0.24052	0.23459	0.00593	0.457
6271	221079_s_at	METTL2A	0.74702	0.78385	-0.03683	0.671
6272	221080_s_at	DENND1C	0.81241	0.7461	0.06631	0.19
6273	221081_s_at	DENND2D	0.33065	0.31694	0.01371	0.427
6274	221087_s_at	APOL3	0.19729	0.25993	-0.06264	0.687
6275	221090_s_at	OGFOD1	0.72324	0.62271	0.10053	0.183
6276	221092_at	IKZF3	0.88801	0.8844	0.00361	0.478
6277	221094_s_at	ELP3	0.25524	0.31078	-0.05554	0.68
6278	221096_s_at	TMCO6	0.39339	0.29385	0.09954	0.208
6279	221103_s_at	WDR52	0.24159	0.38483	-0.14324	0.826
6280	221104_s_at	NIPSNAP3B	0.26069	0.30796	-0.04727	0.636
6281	221135_s_at	ASTE1	0.37586	0.19827	0.17759	0.071
6282	221139_s_at	CSAD	0.57039	0.74272	-0.17233	0.997
6283	221142_s_at	PECR	0.48767	0.39003	0.09764	0.207
6284	221187_s_at	FUZ	0.32624	0.28688	0.03936	0.396
6285	221188_s_at	CIDEB	0.30753	0.27783	0.0297	0.438
6286	221189_s_at	TARS2	0.58207	0.42849	0.15358	0.02
6287	221190_s_at	C18orf8	0.67186	0.63141	0.04045	0.157
6288	221193_s_at	ZCCHC10	0.72852	0.69775	0.03077	0.386
6289	221194_s_at	RNFT1	0.43843	0.54324	-0.10481	0.784
6290	221203_s_at	YEATS2	0.59897	0.65505	-0.05608	0.773
6291	221207_s_at	NBEA	0.47152	0.65659	-0.18507	0.963
6292	221208_s_at	C11orf61	0.6786	0.75017	-0.07157	0.853
6293	221210_s_at	NPL	0.62413	0.60072	0.02341	0.403
6294	221211_s_at	C21orf7	0.0057293	0.18656	-0.1808307	0.902
6295	221213_s_at	ZNF280D	0.26069	0.21525	0.04544	0.348
6296	221214_s_at	NELF	0.35484	0.34312	0.01172	0.453
6297	221216_s_at	SCMH1	0.6317	0.50189	0.12981	0.049
6298	221218_s_at	TPK1	0.52382	0.59848	-0.07466	0.785
6299	221219_s_at	KLHDC4	0.038016	0.14079	-0.102774	0.868
6300	221220_s_at	SCYL2	0.88785	0.89406	-0.00621	0.579
6301	221221_s_at	KLHL3	0.46281	0.43887	0.02394	0.451
6302	221222_s_at	C1orf56	0.35171	0.14815	0.20356	0.05
6303	221229_s_at	TRMT61B	0.19144	0.13768	0.05376	0.344
6304	221230_s_at	ARID4B	0.78809	0.77597	0.01212	0.427
6305	221235_s_at	LOC644617	0.66006	0.39137	0.26869	<0.001
6306	221238_at	NSBP1	0.45294	0.35888	0.09406	0.286
6307	221244_s_at	PDPK1	0.40032	0.40396	-0.00364	0.509
6308	221245_s_at	FZD5	0.59199	0.63114	-0.03915	0.744
6309	221247_s_at	WBSCR16	0.57353	0.58062	-0.00709	0.508
6310	221248_s_at	WHSC1L1	0.22256	0.38859	-0.16603	0.938
6311	221249_s_at	FAM117A	0.74951	0.67278	0.07673	0.131
6312	221253_s_at	TXNDC5	0.51626	0.71811	-0.20185	0.991
6313	221255_s_at	TMEM93	0.70179	0.82534	-0.12355	0.947
6314	221256_s_at	HDHD3	0.011071	0.17177	-0.160699	0.891
6315	221258_s_at	KIF18A	0.63286	0.49983	0.13303	0.011
6316	221260_s_at	CSRNP2	0.76304	0.58681	0.17623	0.002

6317	221262_s_at	SLC2A11	0.4662	0.36895	0.09725	0.575
6318	221263_s_at	SF3B5	0.82724	0.69019	0.13705	0.024
6319	221264_s_at	LOC100128223	0.66898	0.74393	-0.07495	0.882
6320	221265_s_at	C15orf44	0.28616	0.51044	-0.22428	0.954
6321	221267_s_at	FAM108A1	0.65348	0.57243	0.08105	0.036
6322	221268_s_at	SGPP1	0.77547	0.74345	0.03202	0.424
6323	221269_s_at	SH3BGRL3	0.60562	0.50935	0.09627	0.176
6324	221270_s_at	QTRT1	0.3829	0.46513	-0.08223	0.791
6325	221274_s_at	LMAN2L	0.57124	0.5146	0.05664	0.266
6326	221277_s_at	PUS3	0.23879	0.31367	-0.07488	0.762
6327	221286_s_at	MGC29506	0.66711	0.53887	0.12824	0.043
6328	221293_s_at	DEF6	0.32106	0.53554	-0.21448	0.954
6329	221306_at	GPR27	0.41834	0.42468	-0.00634	0.532
6330	221326_s_at	TUBD1	0.29228	0.29175	0.00053	0.506
6331	221381_s_at	MORF4	0.36193	0.53527	-0.17334	0.892
6332	221423_s_at	YIPF5	0.8397	0.68474	0.15496	0.007
6333	221425_s_at	ISCA1	0.50887	0.42922	0.07965	0.195
6334	221427_s_at	CCNL2	0.72419	0.83094	-0.10675	0.981
6335	221428_s_at	TBL1XR1	0.6822	0.72894	-0.04674	0.762
6336	221430_s_at	RNF146	0.67312	0.45306	0.22006	0.003
6337	221432_s_at	SLC25A28	0.23507	0.10028	0.13479	0.162
6338	221434_s_at	C14orf156	0.87529	0.64573	0.22956	<0.001
6339	221436_s_at	CDCA3	0.73895	0.77776	-0.03881	0.755
6340	221437_s_at	MRPS15	0.84974	0.84973	1E-05	0.512
6341	221449_s_at	ITFG1	0.76482	0.5001	0.26472	0.001
6342	221452_s_at	TMEM14B	0.6672	0.61785	0.04935	0.31
6343	221471_at	SERINC3	0.59309	0.42193	0.17116	0.011
6344	221474_at	MYL12B	0.76987	0.73414	0.03573	0.239
6345	221475_s_at	RPL15	0.87614	0.85997	0.01617	0.29
6346	221479_s_at	BNIP3L	0.70495	0.64555	0.0594	0.219
6347	221483_s_at	ARPP19	0.54706	0.63736	-0.0903	0.878
6348	221484_at	B4GALT5	0.3346	0.4542	-0.1196	0.873
6349	221486_at	ENSA	0.41078	0.41568	-0.0049	0.509
6350	221488_s_at	CUTA	0.73496	0.67452	0.06044	0.176
6351	221489_s_at	SPRY4	0.51556	0.65412	-0.13856	0.984
6352	221492_s_at	ATG3	0.61713	0.6718	-0.05467	0.82
6353	221493_at	TSPYL1	0.61559	0.62723	-0.01164	0.546
6354	221495_s_at	TCF25	0.52807	0.67629	-0.14822	0.956
6355	221502_at	KPNA3	0.70169	0.31441	0.38728	<0.001
6356	221504_s_at	ATP6V1H	0.45918	0.53264	-0.07346	0.812
6357	221505_at	ANP32E	0.78591	0.78774	-0.00183	0.527
6358	221506_s_at	TNPO2	0.58117	0.67071	-0.08954	0.868
6359	221509_at	DENR	0.59534	0.42599	0.16935	0.01
6360	221513_s_at	UTP14A	0.48799	0.529	-0.04101	0.633
6361	221515_s_at	LCMT1	0.73958	0.7028	0.03678	0.279
6362	221517_s_at	MED17	0.57779	0.2727	0.30509	0.003
6363	221518_s_at	USP47	0.39477	0.48323	-0.08846	0.89
6364	221520_s_at	CDCA8	0.71929	0.70586	0.01343	0.456
6365	221521_s_at	GINS2	0.84094	0.61281	0.22813	<0.001
6366	221522_at	ANKRD27	0.26024	0.31433	-0.05409	0.686
6367	221524_s_at	RRAGD	0.63197	0.76168	-0.12971	0.958
6368	221532_s_at	WDR61	0.56212	0.71949	-0.15737	0.931
6369	221534_at	C11orf68	0.54458	0.4935	0.05108	0.392
6370	221535_at	LSG1	0.068793	0.3159	-0.247107	0.984

6371	221539_at	EIF4EBP1	0.6535	0.64615	0.00735	0.488
6372	221542_s_at	ERLIN2	0.13287	0.29069	-0.15782	0.896
6373	221548_s_at	ILKAP	0.67575	0.7744	-0.09865	0.93
6374	221549_at	GRWD1	0.4411	0.20773	0.23337	0.001
6375	221550_at	COX15	0.43462	0.34455	0.09007	0.248
6376	221553_at	MAGT1	0.7704	0.83521	-0.06481	0.783
6377	221556_at	CDC14B	0.49427	0.41145	0.08282	0.131
6378	221558_s_at	LEF1	0.036116	0.28152	-0.245404	0.981
6379	221559_s_at	MIS12	0.50829	0.28545	0.22284	0.001
6380	221561_at	SOAT1	0.5034	0.73501	-0.23161	0.893
6381	221565_s_at	CALHM2	0.29908	0.61662	-0.31754	0.987
6382	221568_s_at	LIN7C	0.39208	0.33807	0.05401	0.329
6383	221569_at	AHI1	0.27726	0.42615	-0.14889	0.858
6384	221570_s_at	METTL5	0.71932	0.66645	0.05287	0.23
6385	221571_at	TRAF3	0.53622	0.55377	-0.01755	0.612
6386	221573_at	C7orf25	0.069	0.14915	-0.08015	0.759
6387	221575_at	SCLY	0.53902	0.27237	0.26665	0.004
6388	221580_s_at	TAF1D	0.43779	0.28974	0.14805	0.023
6389	221582_at	HIST3H2A	0.21553	0.28515	-0.06962	0.941
6390	221586_s_at	E2F5	0.55546	0.51987	0.03559	0.393
6391	221587_s_at	C19orf24	0.11222	0.076183	0.036037	0.455
6392	221591_s_at	FAM64A	0.27107	0.41363	-0.14256	0.917
6393	221593_s_at	RPL31	0.64986	0.65701	-0.00715	0.574
6394	221595_at	DKFZP564O0523	0.39481	0.32908	0.06573	0.271
6395	221597_s_at	TMEM208	0.58742	0.58482	0.0026	0.533
6396	221598_s_at	LOC100131612	0.53796	0.56001	-0.02205	0.574
6397	221600_s_at	C11orf67	0.33888	0.39489	-0.05601	0.668
6398	221602_s_at	FAIM3	0.4637	0.55188	-0.08818	0.833
6399	221610_s_at	STAP2	0.0006892	0.14539	-0.1447008	0.895
6400	221616_s_at	TAF9B	0.39	0.42787	-0.03787	0.651
6401	221619_s_at	MTCH1	0.82617	0.69491	0.13126	<0.001
6402	221620_s_at	APOO	0.67862	0.69611	-0.01749	0.611
6403	221621_at	C17orf86	0.21766	0.16819	0.04947	0.345
6404	221622_s_at	TMEM126B	0.86239	0.81166	0.05073	0.139
6405	221626_at	ZNF506	0.34007	0.31163	0.02844	0.442
6406	221632_s_at	WDR4	0.2311	0.11587	0.11523	0.141
6407	221634_at	RPL23AP7	0.13781	0.28862	-0.15081	0.886
6408	221636_s_at	MOSC2	0.011249	0.21575	-0.204501	0.974
6409	221637_s_at	C11orf48	0.79479	0.77868	0.01611	0.426
6410	221638_s_at	STX16	0.6081	0.59119	0.01691	0.59
6411	221640_s_at	LRDD	0.28298	0.30468	-0.0217	0.6
6412	221641_s_at	ACOT9	0.6071	0.64202	-0.03492	0.695
6413	221643_s_at	RERE	0.59451	0.52807	0.06644	0.345
6414	221645_s_at	ZNF83	0.31452	0.36581	-0.05129	0.685
6415	221646_s_at	ZDHHC11	0.0528	0.13516	-0.08236	0.772
6416	221647_s_at	RIC8A	0.58779	0.61801	-0.03022	0.636
6417	221649_s_at	PPAN	0.62932	0.54893	0.08039	0.129
6418	221650_s_at	MED18	0.5092	0.60051	-0.09131	0.865
6419	221652_s_at	C12orf11	0.50398	0.57099	-0.06701	0.79
6420	221657_s_at	ASB6	0.18711	0.10586	0.08125	0.237
6421	221666_s_at	PYCARD	0.51273	0.63572	-0.12299	0.868
6422	221669_s_at	ACAD8	0.48316	0.55385	-0.07069	0.819
6423	221673_s_at	CSNK1G1	0.79992	0.87863	-0.07871	0.969
6424	221675_s_at	CHPT1	0.37561	0.42766	-0.05205	0.667

6425	221676_s_at	CORO1C	0.59481	0.64039	-0.04558	0.743
6426	221677_s_at	DONSON	0.77983	0.71951	0.06032	0.192
6427	221681_s_at	DSPP	0.030212	0.026691	0.003521	0.458
6428	221683_s_at	CEP290	0.91741	0.85501	0.0624	0.013
6429	221685_s_at	CCDC99	0.66934	0.5171	0.15224	0.109
6430	221688_s_at	IMP3	0.36172	0.37455	-0.01283	0.568
6431	221689_s_at	PIGP	0.47714	0.67045	-0.19331	0.993
6432	221692_s_at	MRPL34	0.70268	0.68928	0.0134	0.419
6433	221699_s_at	DDX50	0.42174	0.534	-0.11226	0.886
6434	221700_s_at	UBA52	0.84925	0.85062	-0.00137	0.524
6435	221701_s_at	STRA6	0.36385	0.33385	0.03	0.415
6436	221702_s_at	TM2D3	0.56651	0.60527	-0.03876	0.652
6437	221703_at	BRIP1	0.52045	0.47452	0.04593	0.343
6438	221704_s_at	VPS37B	0.39314	0.49643	-0.10329	0.826
6439	221708_s_at	UNC45A	0.038856	0.00010157	0.03875443	0.384
6440	221711_s_at	C19orf62	0.56827	0.70058	-0.13231	0.963
6441	221712_s_at	WDR74	0.6638	0.50611	0.15769	0.001
6442	221725_at	WASF2	0.44394	0.37929	0.06465	0.24
6443	221727_at	SUB1	0.45629	0.46423	-0.00794	0.559
6444	221732_at	CANT1	0.79837	0.75478	0.04359	0.154
6445	221734_at	PRRC1	0.75027	0.78355	-0.03328	0.756
6446	221737_at	GNA12	0.28063	0.55042	-0.26979	0.978
6447	221738_at	KIAA1219	0.66882	0.64549	0.02333	0.374
6448	221739_at	C19orf10	0.72327	0.78458	-0.06131	0.794
6449	221741_s_at	YTHDF1	0.65375	0.50771	0.14604	0.064
6450	221744_at	WDR68	0.64245	0.74139	-0.09894	0.897
6451	221746_at	UBL4A	0.1154	0.082819	0.032581	0.378
6452	221749_at	YTHDF3	0.78888	0.79018	-0.0013	0.515
6453	221750_at	HMGCS1	0.60961	0.049666	0.559944	<0.001
6454	221751_at	SLC2A3P1	0.63651	0.67845	-0.04194	0.647
6455	221753_at	SSH1	0.82319	0.84982	-0.02663	0.721
6456	221758_at	ARMC6	0.051118	0.02566	0.025458	0.407
6457	221759_at	G6PC3	0.38283	0.59232	-0.20949	0.962
6458	221760_at	MAN1A1	0.66693	0.57692	0.09001	0.157
6459	221761_at	ADSS	0.82399	0.57351	0.25048	0.007
6460	221763_at	JMJD1C	0.55789	0.58969	-0.0318	0.787
6461	221766_s_at	FAM46A	0.78105	0.78688	-0.00583	0.562
6462	221770_at	RPE	0.60173	0.47229	0.12944	0.117
6463	221771_s_at	MPHOSPH8	0.57775	0.71238	-0.13463	0.941
6464	221772_s_at	PPP2R2D	0.082336	0.10264	-0.020304	0.563
6465	221776_s_at	BRD7	0.77795	0.77627	0.00168	0.51
6466	221778_at	JHDM1D	0.5433	0.29928	0.24402	0.011
6467	221780_s_at	DDX27	0.70907	0.78924	-0.08017	0.792
6468	221782_at	DNAJC10	0.28751	0.40192	-0.11441	0.945
6469	221786_at	C6orf120	0.33548	0.32931	0.00617	0.499
6470	221791_s_at	CCDC72	0.80696	0.79049	0.01647	0.347
6471	221799_at	CSGLCA-T	0.62873	0.23851	0.39022	<0.001
6472	221800_s_at	C17orf70	0.55671	0.53613	0.02058	0.423
6473	221803_s_at	NRBF2	0.69683	0.70264	-0.00581	0.557
6474	221804_s_at	FAM45A	0.52765	0.56887	-0.04122	0.636
6475	221806_s_at	SETD5	0.63651	0.69415	-0.05764	0.78
6476	221807_s_at	TRABD	0.36733	0.37903	-0.0117	0.536
6477	221808_at	RAB9A	0.67297	0.77134	-0.09837	0.958
6478	221813_at	FBXO42	0.62092	0.52501	0.09591	0.181

6479	221816_s_at	PHF11	0.82064	0.85521	-0.03457	0.777
6480	221817_at	DOLPP1	0.34648	0.38506	-0.03858	0.719
6481	221818_at	INTS5	0.045734	0.17921	-0.133476	0.86
6482	221821_s_at	C12orf41	0.36257	0.15459	0.20798	0.024
6483	221823_at	C5orf30	0.48774	0.49813	-0.01039	0.553
6484	221824_s_at	MARCH8	0.48146	0.44094	0.04052	0.379
6485	221825_at	ANGEL2	0.56333	0.47546	0.08787	0.118
6486	221829_s_at	TNPO1	0.64613	0.83883	-0.1927	0.979
6487	221831_at	LUZP1	0.2589	0.31285	-0.05395	0.695
6488	221833_at	LONP2	0.29232	0.56175	-0.26943	0.98
6489	221840_at	PTPRE	0.68361	0.65022	0.03339	0.309
6490	221843_s_at	KIAA1609	0.26881	0.24043	0.02838	0.409
6491	221845_s_at	CLPB	0.60024	0.69463	-0.09439	0.816
6492	221847_at	LOC100129361	0.34118	0.28708	0.0541	0.331
6493	221851_at	C19orf72	0.5207	0.56661	-0.04591	0.715
6494	221853_s_at	NOMO1	0.28758	0.41838	-0.1308	0.828
6495	221858_at	TBC1D12	0.43298	0.51997	-0.08699	0.782
6496	221864_at	ORAI3	0.71328	0.59866	0.11462	0.027
6497	221865_at	C9orf91	0.70249	0.6508	0.05169	0.189
6498	221867_at	N4BP1	0.39256	0.46044	-0.06788	0.777
6499	221873_at	ZNF143	0.6081	0.21011	0.39799	<0.001
6500	221876_at	ZNF783	0.084806	0.24373	-0.158924	0.901
6501	221879_at	CALML4	0.64091	0.45065	0.19026	0.016
6502	221881_s_at	CLIC4	0.30267	0.38415	-0.08148	0.802
6503	221882_s_at	TMEM8	0.27148	0.4219	-0.15042	0.9
6504	221888_at	CC2D1A	0.09844	0.21631	-0.11787	0.826
6505	221896_s_at	HIGD1A	0.72536	0.73075	-0.00539	0.533
6506	221897_at	TRIM52	0.049914	0.28883	-0.238916	0.945
6507	221904_at	FAM131A	0.21049	0.41995	-0.20946	0.97
6508	221905_at	CYLD	0.61359	0.6842	-0.07061	0.768
6509	221909_at	RNFT2	0.023087	0.28517	-0.262083	0.974
6510	221912_s_at	CCDC28B	0.67645	0.68279	-0.00634	0.531
6511	221915_s_at	RANBP1	0.6077	0.56823	0.03947	0.344
6512	221918_at	PCTK2	0.37975	0.49061	-0.11086	0.79
6513	221920_s_at	SLC25A37	0.77704	0.62656	0.15048	0.045
6514	221922_at	GPSM2	0.42708	0.3987	0.02838	0.417
6515	221925_s_at	CSPP1	0.17756	0.16625	0.01131	0.481
6516	221927_s_at	ABHD11	0.54186	0.14456	0.3973	<0.001
6517	221931_s_at	SEH1L	0.85177	0.75703	0.09474	0.004
6518	221932_s_at	GLRX5	0.65578	0.70531	-0.04953	0.75
6519	221934_s_at	DALRD3	0.4071	0.49271	-0.08561	0.779
6520	221935_s_at	C3orf64	0.24624	0.3332	-0.08696	0.789
6521	221937_at	AP1GBP1	0.34012	0.26539	0.07473	0.207
6522	221940_at	RPUSD2	0.24459	0.13532	0.10927	0.228
6523	221957_at	PDK3	0.42627	0.36311	0.06316	0.283
6524	221960_s_at	RAB2A	0.69277	0.69861	-0.00584	0.524
6525	221962_s_at	UBE2H	0.36022	0.471	-0.11078	0.875
6526	221965_at	MPHOSPH9	0.28839	0.51546	-0.22707	0.944
6527	221969_at	PAX5	0.65425	0.6561	-0.00185	0.54
6528	221970_s_at	NOL11	0.71204	0.76423	-0.05219	0.899
6529	221972_s_at	SDF4	0.48587	0.4682	0.01767	0.447
6530	221978_at	HLA-F	0.51791	0.58095	-0.06304	0.732
6531	221983_at	FAM134A	0.31422	0.34404	-0.02982	0.58
6532	221985_at	KLHL24	0.63246	0.63836	-0.0059	0.552

6533	221987_s_at	TSR1	0.68169	0.5509	0.13079	0.054
6534	221988_at	C19orf42	0.15677	0.42634	-0.26957	0.995
6535	221995_s_at	MRP63	0.79912	0.78019	0.01893	0.37
6536	221997_s_at	MRPL52	0.6461	0.79173	-0.14563	0.989
6537	222000_at	C1orf174	0.55343	0.64115	-0.08772	0.833
6538	222006_at	LETM1	0.7117	0.44703	0.26467	<0.001
6539	222010_at	TCP1	0.70246	0.60537	0.09709	0.057
6540	222016_s_at	ZNF323	0.11885	0.24023	-0.12138	0.825
6541	222018_at	NACA	0.44418	0.62862	-0.18444	0.967
6542	222019_at	PFDN6	0.23938	0.25521	-0.01583	0.552
6543	222027_at	NUCKS1	0.44925	0.53359	-0.08434	0.759
6544	222028_at	ZNF45	0.28882	0.33665	-0.04783	0.67
6545	222030_at	SIVA1	0.22533	0.29323	-0.0679	0.683
6546	222031_at	LOC286434	0.40877	0.41595	-0.00718	0.585
6547	222034_at	GNB2L1	0.65295	0.55378	0.09917	0.147
6548	222039_at	KIF18B	0.52373	0.46701	0.05672	0.137
6549	222040_at	HNRNPA1	0.67575	0.71595	-0.0402	0.785
6550	222046_at	SRRT	0.08953	0.25451	-0.16498	0.922
6551	222048_at	CRYBB2P1	0.28622	0.12101	0.16521	0.443
6552	222052_at	C19orf54	0.5742	0.43685	0.13735	0.092
6553	222064_s_at	AARSD1	0.55243	0.58027	-0.02784	0.605
6554	222071_s_at	SLCO4C1	0.71818	0.69841	0.01977	0.409
6555	222077_s_at	RACGAP1	0.37925	0.47551	-0.09626	0.791
6556	222088_s_at	SLC2A14	0.59235	0.58569	0.00666	0.461
6557	222103_at	ATF1	0.63326	0.60385	0.02941	0.477
6558	222105_s_at	NKIRAS2	0.76076	0.67902	0.08174	0.097
6559	222118_at	CENPN	0.8374	0.75058	0.08682	0.005
6560	222119_s_at	FBXO11	0.48012	0.63255	-0.15243	0.918
6561	222120_at	ZNF764	0.21846	0.10754	0.11092	0.228
6562	222125_s_at	P4HTM	0.51322	0.52282	-0.0096	0.571
6563	222127_s_at	EXOC1	0.10088	0.35452	-0.25364	0.928
6564	222128_at	NSUN6	0.2444	0.38551	-0.14111	0.922
6565	222130_s_at	FTSJ2	0.13548	0.079977	0.055503	0.257
6566	222138_s_at	WDR13	0.64994	0.70705	-0.05711	0.81
6567	222139_at	KIAA1466	0.3434	0.44696	-0.10356	0.831
6568	222140_s_at	GPR89A	0.48377	0.58062	-0.09685	0.953
6569	222143_s_at	MTMR14	0.69285	0.59215	0.1007	0.197
6570	222147_s_at	ACTR5	0.23464	0.13715	0.09749	0.188
6571	222148_s_at	RHOT1	0.02677	0.12249	-0.09572	0.776
6572	222150_s_at	PION	0.14089	0.44677	-0.30588	0.998
6573	222154_s_at	LOC26010	0.46388	0.55639	-0.09251	0.836
6574	222155_s_at	GPR172A	0.55258	0.48721	0.06537	0.203
6575	222175_s_at	MED15	0.23805	0.45151	-0.21346	0.879
6576	222186_at	ZFAND6	0.7386	0.85098	-0.11238	0.986
6577	222190_s_at	C16orf58	0.25379	0.1	0.15379	0.051
6578	222193_at	C2orf43	0.36318	0.51348	-0.1503	0.915
6579	222199_s_at	BIN3	0.56367	0.53979	0.02388	0.369
6580	222200_s_at	BSDC1	0.39667	0.26955	0.12712	0.084
6581	222201_s_at	CASP8AP2	0.562	0.5068	0.0552	0.24
6582	222203_s_at	RDH14	0.71294	0.57466	0.13828	0.011
6583	222204_s_at	RRN3	0.65345	0.6462	0.00725	0.471
6584	222208_s_at	POLR2J4	0.723	0.76639	-0.04339	0.783
6585	222209_s_at	TMEM135	0.43903	0.38323	0.0558	0.334
6586	222212_s_at	LASS2	0.52916	0.67713	-0.14797	0.869

6587	222214_at	SUZ12P	0.76968	0.78282	-0.01314	0.596
6588	222216_s_at	MRPL17	0.69328	0.61363	0.07965	0.167
6589	222217_s_at	SLC27A3	0.39894	0.49911	-0.10017	0.782
6590	222228_s_at	ALKBH4	0.34364	0.21473	0.12891	0.207
6591	222230_s_at	ACTR10	0.50744	0.57531	-0.06787	0.844
6592	222231_s_at	LRRC59	0.72845	0.65854	0.06991	0.074
6593	222233_s_at	DCLRE1C	0.77688	0.73599	0.04089	0.301
6594	222235_s_at	CSGALNACT2	0.26465	0.58955	-0.3249	1
6595	222238_s_at	POLM	0.32759	0.37078	-0.04319	0.635
6596	222239_s_at	INTS6	0.84941	0.79544	0.05397	0.197
6597	222243_s_at	TOB2	0.78	0.78257	-0.00257	0.544
6598	222244_s_at	TUG1	0.67452	0.50148	0.17304	0.055
6599	222250_s_at	INTS7	0.35517	0.53043	-0.17526	0.963
6600	222251_s_at	GMEB2	0.60006	0.44158	0.15848	0.046
6601	222262_s_at	ETNK1	0.23753	0.35707	-0.11954	0.897
6602	222263_at	SLC35E1	0.70854	0.61351	0.09503	0.12
6603	222264_at	HNRNPUL2	0.24503	0.16984	0.07519	0.303
6604	222270_at	SMEK2	0.30544	0.054638	0.250802	0.025
6605	222273_at	PAPOLG	0.24116	0.37375	-0.13259	0.869
6606	222275_at	MRPS30	0.25226	0.25319	-0.00093	0.529
6607	222276_at	METTL2B	0.084431	0.08959	-0.005159	0.512
6608	222279_at	RP3-377H14.5	0.28144	0.20968	0.07176	0.31
6609	222283_at	ZNF480	0.72994	0.64817	0.08177	0.075
6610	222305_at	HK2	0.18959	0.10792	0.08167	0.194
6611	222307_at	LOC282997	0.43885	0.31116	0.12769	0.164
6612	222310_at	SFRS15	0.71524	0.67461	0.04063	0.385
6613	222313_at	CNOT2	0.77098	0.76929	0.00169	0.517
6614	222316_at	USO1	0.54082	0.66399	-0.12317	0.984
6615	222318_at	ZNF324B	0.06406	0.080393	-0.016333	0.588
6616	222326_at	PDE4B	0.72365	0.61114	0.11251	0.115
6617	222336_at	C4orf34	0.58846	0.58019	0.00827	0.494
6618	222344_at	C5orf13	0.44355	0.41666	0.02689	0.395
6619	222350_at	BCL9	0.059377	0.026129	0.033248	0.405
6620	222351_at	PPP2R1B	0.42944	0.32736	0.10208	0.185
6621	222354_at	F11R	0.066102	0.13229	-0.066188	0.706
6622	222360_at	DPH5	0.27685	0.21242	0.06443	0.294
6623	222366_at	ADNP	0.90079	0.90438	-0.00359	0.591
6624	222369_at	NAT11	0.53144	0.48374	0.0477	0.28
6625	222376_at	HACE1	0.4939	0.4606	0.0333	0.388
6626	266_s_at	CD24	0.32905	0.46715	-0.1381	0.831
6627	31845_at	ELF4	0.2757	0.42632	-0.15062	0.905
6628	32032_at	DGCR14	0.66502	0.53931	0.12571	0.12
6629	32091_at	SLC25A44	0.64584	0.51675	0.12909	0.055
6630	32811_at	MYO1C	0.45366	0.61248	-0.15882	0.953
6631	32836_at	AGPAT1	0.45995	0.45142	0.00853	0.482
6632	33322_i_at	SFN	0.66063	0.5651	0.09553	0.106
6633	33494_at	ETFDH	0.26727	0.32488	-0.05761	0.705
6634	33760_at	PEX14	0.38378	0.49754	-0.11376	0.888
6635	33814_at	PAK4	0.26739	0.32541	-0.05802	0.701
6636	33850_at	MAP4	0.094782	0.12867	-0.033888	0.594
6637	34210_at	CD52	0.73583	0.69635	0.03948	0.289
6638	34221_at	HMGXB3	0.086776	0.15001	-0.063234	0.708
6639	34260_at	TELO2	0.61235	0.36564	0.24671	0.002
6640	34406_at	PACS2	0.16056	0.25342	-0.09286	0.836

6641	34408_at	RTN2	0.21058	0.31919	-0.10861	0.787
6642	34478_at	RAB11B	0.47777	0.2684	0.20937	0.055
6643	34697_at	LRP6	0.33861	0.53312	-0.19451	0.971
6644	34858_at	KCTD2	0.33837	0.46874	-0.13037	0.881
6645	34868_at	SMG5	0.27288	0.33887	-0.06599	0.702
6646	35160_at	LDB1	0.74938	0.59818	0.1512	0.03
6647	35265_at	FXR2	0.53797	0.4173	0.12067	0.191
6648	35626_at	SGSH	0.53522	0.44096	0.09426	0.195
6649	35671_at	GTF3C1	0.69828	0.53662	0.16166	0.064
6650	36019_at	STK19	0.69929	0.63359	0.0657	0.23
6651	36030_at	IFFO1	0.39439	0.44518	-0.05079	0.714
6652	36084_at	CUL7	0.40781	0.49683	-0.08902	0.763
6653	36475_at	GCAT	0.17992	0.26628	-0.08636	0.792
6654	36545_s_at	SFI1	0.61725	0.58011	0.03714	0.378
6655	36552_at	C2CD3	0.29498	0.41575	-0.12077	0.82
6656	36711_at	MAFF	0.63261	0.65142	-0.01881	0.596
6657	36829_at	PER1	0.35484	0.065051	0.289789	0.005
6658	36865_at	ANGEL1	0.57552	0.63986	-0.06434	0.743
6659	36907_at	MVK	0.41327	0.29046	0.12281	0.184
6660	36920_at	MTM1	0.42128	0.53312	-0.11184	0.918
6661	36994_at	ATP6V0C	0.55189	0.6397	-0.08781	0.8
6662	37012_at	CAPZB	0.58441	0.57395	0.01046	0.47
6663	37028_at	PPP1R15A	0.62322	0.15218	0.47104	<0.001
6664	37079_at	YDD19	0.5526	0.6466	-0.094	0.91
6665	37152_at	PPARD	0.65333	0.65829	-0.00496	0.523
6666	37170_at	BMP2K	0.57038	0.57952	-0.00914	0.556
6667	37226_at	BNIP1	0.65217	0.66901	-0.01684	0.594
6668	37232_at	KIAA0586	0.38959	0.27173	0.11786	0.181
6669	37254_at	ZNF133	0.13841	0.30801	-0.1696	0.905
6670	37278_at	TAZ	0.3012	0.43212	-0.13092	0.927
6671	37462_i_at	SF3A2	0.65824	0.53154	0.1267	0.085
6672	37512_at	HSD17B6	0.28741	0.39978	-0.11237	0.829
6673	37802_r_at	FAM63B	0.1754	0.15325	0.02215	0.495
6674	37860_at	ZNF337	0.2916	0.3869	-0.0953	0.821
6675	37872_at	JRK	0.68182	0.59625	0.08557	0.164
6676	37966_at	PARVB	0.62274	0.59131	0.03143	0.384
6677	38290_at	RGS14	0.46558	0.5513	-0.08572	0.796
6678	38398_at	MADD	0.25712	0.14851	0.10861	0.174
6679	38447_at	ADRBK1	0.50602	0.51847	-0.01245	0.585
6680	38892_at	KIAA0240	0.42961	0.25945	0.17016	0.032
6681	39248_at	AQP3	0.52601	0.68735	-0.16134	0.979
6682	39318_at	TCL1A	0.32146	0.46483	-0.14337	0.879
6683	39729_at	PRDX2	0.26216	0.32242	-0.06026	0.679
6684	39817_s_at	C6orf108	0.51017	0.58021	-0.07004	0.717
6685	40020_at	CELSR3	0.076559	0.13235	-0.055791	0.676
6686	40149_at	SH2B1	0.34904	0.46427	-0.11523	0.809
6687	40255_at	DDX28	0.42626	0.37082	0.05544	0.323
6688	40273_at	SPHK2	0.49988	0.44361	0.05627	0.298
6689	40359_at	RASSF7	0.14625	0.099872	0.046378	0.337
6690	40420_at	STK10	0.34564	0.414	-0.06836	0.746
6691	40446_at	PHF1	0.69362	0.49506	0.19856	<0.001
6692	40465_at	DDX23	0.87339	0.7747	0.09869	0.092
6693	40829_at	WDTC1	0.79155	0.74418	0.04737	0.217
6694	41160_at	MBD3	0.7667	0.68303	0.08367	0.032

6695	41220_at	SEPT9	0.72748	0.66444	0.06304	0.3
6696	41387_r_at	KDM6B	0.67353	0.39957	0.27396	<0.001
6697	41397_at	ZNF821	0.37515	0.23729	0.13786	0.079
6698	41512_at	BRAP	0.66917	0.62483	0.04434	0.259
6699	41577_at	PPP1R16B	0.52696	0.56967	-0.04271	0.708
6700	41657_at	STK11	0.29659	0.48966	-0.19307	0.941
6701	41858_at	FRAG1	0.40653	0.26901	0.13752	0.08
6702	43544_at	MED16	0.42798	0.45919	-0.03121	0.61
6703	43934_at	GPR137	0.28552	0.2323	0.05322	0.338
6704	43977_at	TMEM161A	0.3979	0.21981	0.17809	0.038
6705	44065_at	C12orf52	0.42625	0.56391	-0.13766	0.891
6706	44669_at	LOC644096	0.45279	0.52536	-0.07257	0.756
6707	44702_at	SYDE1	0.43159	0.30683	0.12476	0.148
6708	44783_s_at	HEY1	0.71497	0.77448	-0.05951	0.733
6709	45687_at	PRR14	0.6344	0.58251	0.05189	0.273
6710	46167_at	TTC4	0.55199	0.40661	0.14538	0.051
6711	46270_at	UBAP1	0.4234	0.46902	-0.04562	0.663
6712	46665_at	SEMA4C	0.57166	0.50209	0.06957	0.162
6713	47083_at	C7orf26	0.43288	0.35534	0.07754	0.261
6714	47571_at	ZNF236	0.30312	0.31637	-0.01325	0.549
6715	47608_at	TJAP1	0.2976	0.48599	-0.18839	0.95
6716	48117_at	CCDC101	0.01467	0.13153	-0.11686	0.878
6717	48531_at	TNIP2	0.71666	0.69041	0.02625	0.4
6718	48659_at	MIIP	0.75681	0.68026	0.07655	0.069
6719	49306_at	RASSF4	0.74897	0.71729	0.03168	0.276
6720	49327_at	SIRT3	0.45153	0.29522	0.15631	0.018
6721	49485_at	PRDM4	0.40967	0.30282	0.10685	0.188
6722	50221_at	TFEB	0.738	0.65468	0.08332	0.083
6723	50277_at	GGA1	0.37684	0.42158	-0.04474	0.623
6724	50374_at	C17orf90	0.82516	0.75057	0.07459	0.192
6725	51158_at	FAM174B	0.3685	0.45441	-0.08591	0.806
6726	51774_s_at	LOC222070	0.40035	0.3734	0.02695	0.459
6727	52078_at	TMEM222	0.49126	0.50966	-0.0184	0.576
6728	52164_at	C11orf24	0.60748	0.66102	-0.05354	0.687
6729	52169_at	STRADA	0.34311	0.52611	-0.183	0.921
6730	52741_at	TRMT61A	0.27853	0.25465	0.02388	0.37
6731	52940_at	SIGIRR	0.37425	0.47591	-0.10166	0.853
6732	53076_at	B4GALT7	0.56224	0.54077	0.02147	0.423
6733	53912_at	SNX11	0.42234	0.25046	0.17188	0.056
6734	53987_at	RANBP10	0.13618	0.23833	-0.10215	0.757
6735	54037_at	HPS4	0.37915	0.38623	-0.00708	0.566
6736	54051_at	PKNOX1	0.38713	0.2123	0.17483	0.063
6737	54970_at	ZMIZ2	0.83215	0.82788	0.00427	0.456
6738	55065_at	MARK4	0.56738	0.66026	-0.09288	0.801
6739	55081_at	MICALL1	0.36478	0.3308	0.03398	0.394
6740	55616_at	PERLD1	0.53012	0.38078	0.14934	0.101
6741	55662_at	C10orf76	0.43469	0.43071	0.00398	0.505
6742	55692_at	ELMO2	0.45764	0.27426	0.18338	0.05
6743	55705_at	C19orf22	0.54157	0.52642	0.01515	0.456
6744	55872_at	ZNF512B	0.34856	0.47561	-0.12705	0.86
6745	56256_at	SIDT2	0.71731	0.68925	0.02806	0.327
6746	56919_at	WDR48	0.20428	0.43814	-0.23386	0.991
6747	57082_at	LDLRAP1	0.4319	0.62286	-0.19096	0.926
6748	57163_at	ELOVL1	0.28726	0.18758	0.09968	0.221

6749	57539_at	ZGPAT	0.63039	0.59062	0.03977	0.379
6750	58308_at	TRIM62	0.21596	0.23416	-0.0182	0.572
6751	58696_at	EXOSC4	0.72666	0.56899	0.15767	0.012
6752	59625_at	NOL3	0.36726	0.29884	0.06842	0.232
6753	60528_at	JMJD7-PLA2G4B	0.47335	0.35568	0.11767	0.12
6754	63009_at	SHQ1	0.67394	0.52575	0.14819	0.013
6755	632_at	GSK3A	0.44783	0.51374	-0.06591	0.729
6756	635_s_at	PPP2R5B	0.49985	0.36758	0.13227	0.118
6757	64371_at	SFRS14	0.74456	0.72183	0.02273	0.366
6758	64486_at	CORO1B	0.24306	0.43273	-0.18967	0.929
6759	64488_at	IRGQ	0.61046	0.33999	0.27047	0.005
6760	64883_at	MOSPD2	0.35046	0.29808	0.05238	0.311
6761	64900_at	FLJ22167	0.43252	0.55138	-0.11886	0.91
6762	65086_at	YIPF2	0.28203	0.32061	-0.03858	0.658
6763	65133_i_at	INO80B	0.63373	0.28986	0.34387	0.002
6764	65585_at	FAM86B1	0.25126	0.33595	-0.08469	0.791
6765	65588_at	LOC388796	4.99E-05	0.098392	-0.098342089	0.857
6766	65630_at	TMEM80	0.16704	0.27238	-0.10534	0.818
6767	65770_at	RHOT2	0.70949	0.64015	0.06934	0.154
6768	77508_r_at	LOC100133585	0.32773	0.35629	-0.02856	0.611
6769	78047_s_at	LOC729580	0.6275	0.43331	0.19419	0.016
6770	78383_at	LOC100129250	0.46249	0.46239	1E-04	0.496
6771	87100_at	ABHD2	0.60257	0.62209	-0.01952	0.608
6772	89948_at	PCIF1	0.094399	0.14037	-0.045971	0.676
6773	90610_at	LRCH4	0.45297	0.53629	-0.08332	0.8
6774	91703_at	EHBP1L1	0.77303	0.77012	0.00291	0.476
6775	91816_f_at	MEX3D	0.52536	0.44616	0.0792	0.111

Supplemental Table 5. SVM modeling results upon ionizing radiation stress

Index	Affymetrix ID	Gene Symbol	R^2 - 0 hour	R^2 - 2 hour	R^2 - 6 hour	difference in R^2 between 0 and 6 hr	P value
1	1007_s_at	DDR1	0.43371	0.5866	0.54544	-0.11173	0.782
2	1053_at	RFC2	0.67271	0.48489	0.63612	0.03659	0.361
3	1405_i_at	CCL5	0.36622	0.57544	0.5039	-0.13768	0.872
4	1487_at	ESRRA	0.36634	0.19822	0.47399	-0.10765	0.88
5	1729_at	TRADD	0.47494	0.45656	0.29426	0.18068	0.083
6	1861_at	BAD	0.45824	0.45391	0.4254	0.03284	0.403
7	200000_s_at	PRPF8	0.89564	0.89306	0.83624	0.0594	0.15
8	200001_at	CAPNS1	0.79518	0.80918	0.848	-0.05282	0.806
9	200002_at	RPL35	0.90268	0.89815	0.90657	-0.00389	0.593
10	200003_s_at	RPL28	0.93999	0.90924	0.92643	0.01356	0.181
11	200004_at	EIF4G2	0.78441	0.78782	0.76245	0.02196	0.385
12	200005_at	EIF3D	0.5245	0.57792	0.6508	-0.1263	0.78
13	200006_at	PARK7	0.85801	0.89315	0.92361	-0.0656	0.991
14	200007_at	SRP14	0.79089	0.82677	0.87469	-0.0838	0.908
15	200009_at	GDI2	0.49884	0.64033	0.73118	-0.23234	0.964
16	200010_at	RPL11	0.91326	0.92611	0.94377	-0.03051	0.973
17	200013_at	RPL24	0.91816	0.89746	0.93287	-0.01471	0.816
18	200015_s_at	SEPT2	0.56242	0.54324	0.42267	0.13975	0.058
19	200017_at	RPS27A	0.93662	0.92217	0.94197	-0.00535	0.639
20	200018_at	RPS13	0.9332	0.91481	0.93037	0.00283	0.363
21	200019_s_at	FAU	0.95724	0.92868	0.95049	0.00675	0.179
22	200020_at	TARDBP	0.74729	0.58747	0.72791	0.01938	0.427
23	200021_at	CFL1	0.9145	0.89664	0.90508	0.00942	0.362
24	200024_at	RPS5	0.89573	0.89436	0.93594	-0.04021	0.973
25	200026_at	RPL34	0.88512	0.90815	0.87468	0.01044	0.414
26	200027_at	NARS	0.64691	0.50009	0.4963	0.15061	0.07
27	200029_at	RPL19	0.93655	0.93137	0.93685	-0.0003	0.501
28	200030_s_at	SLC25A3	0.90173	0.92322	0.9235	-0.02177	0.821
29	200032_s_at	RPL9	0.94256	0.93319	0.93993	0.00263	0.449
30	200033_at	DDX5	0.79244	0.76393	0.66898	0.12346	0.046
31	200034_s_at	RPL6	0.91342	0.93439	0.92337	-0.00995	0.72
32	200035_at	DULLARD	0.44677	0.43962	0.47905	-0.03228	0.617
33	200036_s_at	RPL10A	0.90329	0.91728	0.9366	-0.03331	0.976
34	200038_s_at	RPL17	0.92058	0.92909	0.93876	-0.01818	0.857
35	200039_s_at	PSMB2	0.85679	0.84344	0.83604	0.02075	0.396
36	200040_at	KHDRBS1	0.69302	0.55185	0.48072	0.2123	0.035
37	200041_s_at	BAT1	0.5434	0.5107	0.65627	-0.11287	0.821
38	200042_at	C22orf28	0.65126	0.69161	0.57586	0.0754	0.32
39	200043_at	ERH	0.82376	0.86237	0.89116	-0.0674	0.958
40	200044_at	SFRS9	0.81827	0.84936	0.85457	-0.0363	0.699
41	200045_at	ABCF1	0.66649	0.82578	0.72859	-0.0621	0.733
42	200046_at	DAD1	0.8316	0.80833	0.82683	0.00477	0.467
43	200048_s_at	JTB	0.80563	0.86456	0.89958	-0.09395	0.909
44	200049_at	MYST2	0.77841	0.7385	0.76467	0.01374	0.445
45	200050_at	ZNF146	0.67763	0.41111	0.59109	0.08654	0.12
46	200051_at	SART1	0.44708	0.65378	0.46967	-0.02259	0.558
47	200052_s_at	ILF2	0.40377	0.52882	0.51709	-0.11332	0.712
48	200053_at	SPAG7	0.79396	0.76161	0.78306	0.0109	0.433
49	200054_at	ZNF259	0.46936	0.54443	0.50928	-0.03992	0.598
50	200055_at	TAF10	0.88663	0.86284	0.8893	-0.00267	0.512

51	200056_s_at	C1D	0.71362	0.73911	0.72699	-0.01337	0.531
52	200059_s_at	RHOA	0.51059	0.75414	0.80946	-0.29887	0.976
53	200060_s_at	RNPS1	0.42642	0.63154	0.67343	-0.24701	0.991
54	200061_s_at	RPS24	0.93742	0.92451	0.93268	0.00474	0.42
55	200062_s_at	RPL30	0.95499	0.94114	0.9377	0.01729	0.024
56	200063_s_at	NPM1	0.92798	0.93167	0.93499	-0.00701	0.623
57	200064_at	HSP90AB1	0.87828	0.79366	0.77526	0.10302	0.001
58	200065_s_at	ARF1	0.54439	0.57782	0.52333	0.02106	0.451
59	200066_at	IK	0.36201	0.40122	0.55959	-0.19758	0.91
60	200071_at	SMNDC1	0.60558	0.59266	0.44937	0.15621	0.084
61	200073_s_at	HNRNPD	0.73975	0.68391	0.55806	0.18169	0.109
62	200074_s_at	RPL14	0.91568	0.92447	0.94329	-0.02761	0.859
63	200077_s_at	OAZ1	0.9034	0.89493	0.91943	-0.01603	0.704
64	200078_s_at	ATP6V0B	0.51031	0.65955	0.61216	-0.10185	0.758
65	200080_s_at	H3F3A	0.82818	0.76325	0.79595	0.03223	0.245
66	200082_s_at	RPS7	0.93987	0.94271	0.94758	-0.00771	0.769
67	200084_at	C11orf58	0.51147	0.35771	0.27787	0.2336	0.066
68	200085_s_at	TCEB2	0.91688	0.88524	0.92401	-0.00713	0.612
69	200089_s_at	RPL4	0.91788	0.9306	0.93703	-0.01915	0.778
70	200090_at	FNTA	0.18426	0.29333	0.31392	-0.12966	0.825
71	200091_s_at	RPS25	0.92407	0.90598	0.93543	-0.01136	0.79
72	200092_s_at	RPL37	0.94868	0.93848	0.93581	0.01287	0.142
73	200093_s_at	HINT1	0.94098	0.92161	0.92047	0.02051	0.122
74	200096_s_at	ATP6V0E1	0.74198	0.79686	0.82537	-0.08339	0.91
75	200099_s_at	RPS3A	0.9395	0.93853	0.93448	0.00502	0.366
76	200593_s_at	HNRNPU	0.76761	0.74101	0.76314	0.00447	0.478
77	200596_s_at	EIF3A	0.83261	0.805	0.86193	-0.02932	0.704
78	200599_s_at	HSP90B1	0.66034	0.67934	0.59618	0.06416	0.272
79	200600_at	MSN	0.74393	0.80228	0.78105	-0.03712	0.679
80	200601_at	ACTN4	0.40517	0.68094	0.61871	-0.21354	0.989
81	200604_s_at	PRKAR1A	0.84127	0.81321	0.87856	-0.03729	0.804
82	200607_s_at	RAD21	0.76513	0.75878	0.71911	0.04602	0.318
83	200609_s_at	WDR1	0.8771	0.73592	0.76797	0.10913	0.06
84	200610_s_at	NCL	0.27871	0.5168	0.44151	-0.1628	0.85
85	200613_at	AP2M1	0.66431	0.53122	0.6919	-0.02759	0.62
86	200615_s_at	AP2B1	0.82346	0.85483	0.81002	0.01344	0.459
87	200616_s_at	MLEC	0.55895	0.61298	0.4497	0.10925	0.224
88	200618_at	LASP1	0.58214	0.57228	0.52371	0.05843	0.283
89	200619_at	SF3B2	0.60875	0.71205	0.73632	-0.12757	0.899
90	200620_at	TMEM59	0.47865	0.71989	0.66402	-0.18537	0.985
91	200621_at	CSRP1	0.29273	0.43074	0.45329	-0.16056	0.954
92	200623_s_at	CALM3	0.38803	0.52003	0.50358	-0.11555	0.767
93	200626_s_at	MATR3	0.67174	0.4731	0.53302	0.13872	0.01
94	200627_at	PTGES3	0.66133	0.8415	0.74842	-0.08709	0.819
95	200629_at	WARS	0.57513	0.62438	0.69472	-0.11959	0.881
96	200632_s_at	NDRG1	0.25279	0.28005	0.20926	0.04353	0.455
97	200633_at	UBB	0.92397	0.93877	0.94926	-0.02529	0.881
98	200634_at	PFN1	0.88801	0.79279	0.89235	-0.00434	0.557
99	200642_at	SOD1	0.8213	0.86874	0.8882	-0.0669	0.892
100	200644_at	MARCKSL1	0.37932	0.54356	0.53459	-0.15527	0.912
101	200645_at	GABARAP	0.81453	0.82986	0.8707	-0.05617	0.782
102	200649_at	NUCB1	0.59914	0.6519	0.6141	-0.01496	0.573
103	200650_s_at	LDHA	0.9063	0.8724	0.81499	0.09131	0.024

104	200652_at	SSR2	0.80227	0.81985	0.83584	-0.03357	0.732
105	200654_at	P4HB	0.53837	0.58924	0.44643	0.09194	0.185
106	200657_at	SLC25A5	0.86044	0.84042	0.8855	-0.02506	0.723
107	200658_s_at	PHB	0.77004	0.79409	0.82185	-0.05181	0.764
108	200660_at	S100A11	0.31747	0.39596	0.44408	-0.12661	0.818
109	200661_at	CTSA	0.62086	0.7371	0.73726	-0.1164	0.876
110	200663_at	CD63	0.70974	0.76081	0.83086	-0.12112	0.897
111	200665_s_at	SPARC	0.15333	0.41168	0.27057	-0.11724	0.773
112	200666_s_at	DNAJB1	0.47088	0.53062	0.57065	-0.09977	0.794
113	200668_s_at	UBE2D3	0.66055	0.6545	0.48643	0.17412	0.12
114	200670_at	XBP1	0.62457	0.72944	0.6338	-0.00923	0.547
115	200673_at	LAPTM4A	0.78795	0.67701	0.68729	0.10066	0.115
116	200674_s_at	RPL32	0.94438	0.93777	0.94384	0.00054	0.487
117	200675_at	CD81	0.52283	0.70968	0.64023	-0.1174	0.852
118	200677_at	PTTG1IP	0.63106	0.7249	0.68384	-0.05278	0.729
119	200681_at	GLO1	0.49794	0.5774	0.53321	-0.03527	0.605
120	200682_s_at	UBE2L3	0.8213	0.8214	0.87424	-0.05294	0.825
121	200687_s_at	SF3B3	0.80853	0.80048	0.77581	0.03272	0.299
122	200692_s_at	HSPA9	0.78744	0.80537	0.79002	-0.00258	0.495
123	200695_at	PPP2R1A	0.52215	0.48123	0.42695	0.0952	0.269
124	200696_s_at	GSN	0.18891	0.35755	0.28064	-0.09173	0.761
125	200697_at	HK1	0.50428	0.6846	0.65893	-0.15465	0.901
126	200699_at	KDELR2	0.31653	0.49912	0.5081	-0.19157	0.886
127	200701_at	NPC2	0.75494	0.727	0.80573	-0.05079	0.717
128	200702_s_at	DDX24	0.2644	0.24942	0.44148	-0.17708	0.891
129	200703_at	DYNLL1	0.81106	0.83747	0.72639	0.08467	0.154
130	200705_s_at	EEF1B2	0.87992	0.92413	0.93462	-0.0547	0.989
131	200706_s_at	LITAF	0.57502	0.60062	0.62141	-0.04639	0.674
132	200707_at	PRKCSH	0.72678	0.72761	0.69203	0.03475	0.36
133	200708_at	GOT2	0.70647	0.67898	0.66953	0.03694	0.366
134	200709_at	FKBP1A	0.37016	0.33324	0.46085	-0.09069	0.68
135	200710_at	ACADVL	0.49118	0.56699	0.54397	-0.05279	0.629
136	200713_s_at	MAPRE1	0.63949	0.61568	0.66321	-0.02372	0.581
137	200718_s_at	SKP1	0.83754	0.82604	0.88527	-0.04773	0.821
138	200720_s_at	ACTR1A	0.41901	0.5584	0.42568	-0.00667	0.538
139	200722_s_at	CAPRIN1	0.78952	0.79488	0.76921	0.02031	0.356
140	200726_at	PPP1CC	0.6771	0.63182	0.62445	0.05265	0.294
141	200729_s_at	ACTR2	0.68858	0.71196	0.68726	0.00132	0.491
142	200733_s_at	PTP4A1	0.65804	0.57516	0.63632	0.02172	0.397
143	200734_s_at	ARF3	0.3941	0.44635	0.53782	-0.14372	0.864
144	200736_s_at	GPX1	0.73264	0.81383	0.83446	-0.10182	0.952
145	200740_s_at	SUMO3	0.74373	0.77886	0.64726	0.09647	0.071
146	200741_s_at	RPS27	0.93494	0.93525	0.92706	0.00788	0.333
147	200743_s_at	TPP1	0.77742	0.8314	0.80437	-0.02695	0.717
148	200746_s_at	GNB1	0.77172	0.7154	0.69154	0.08018	0.143
149	200748_s_at	FTH1	0.66299	0.73595	0.84725	-0.18426	0.973
150	200749_at	RAN	0.70279	0.70144	0.64867	0.05412	0.33
151	200751_s_at	HNRNPC	0.60734	0.61185	0.67064	-0.0633	0.683
152	200755_s_at	CALU	0.33697	0.30943	0.51207	-0.1751	0.837
153	200761_s_at	ARL6IP5	0.35615	0.49792	0.36718	-0.01103	0.563
154	200762_at	DPYSL2	0.4394	0.45416	0.49139	-0.05199	0.663
155	200763_s_at	RPLP1	0.94797	0.92887	0.94873	-0.00076	0.52
156	200766_at	CTSD	0.34225	0.35805	0.22324	0.11901	0.178

157	200767_s_at	FAM120A	0.65577	0.53863	0.55543	0.10034	0.236
158	200770_s_at	LAMC1	0.6319	0.58898	0.65394	-0.02204	0.524
159	200775_s_at	HNRNPK	0.77096	0.82212	0.75284	0.01812	0.43
160	200776_s_at	BZW1	0.42707	0.43682	0.42822	-0.00115	0.5
161	200779_at	ATF4	0.60722	0.61518	0.65332	-0.0461	0.702
162	200782_at	ANXA5	0.25737	0.43237	0.35537	-0.098	0.807
163	200783_s_at	STMN1	0.35532	0.38144	0.31214	0.04318	0.379
164	200786_at	PSMB7	0.75641	0.78495	0.86274	-0.10633	0.898
165	200788_s_at	PEA15	0.37983	0.47659	0.42861	-0.04878	0.65
166	200789_at	ECH1	0.47643	0.65829	0.67061	-0.19418	0.812
167	200790_at	ODC1	0.60699	0.66844	0.53294	0.07405	0.278
168	200792_at	XRCC6	0.86182	0.83166	0.80014	0.06168	0.236
169	200793_s_at	ACO2	0.76774	0.72238	0.73687	0.03087	0.381
170	200802_at	SARS	0.78614	0.61669	0.711	0.07514	0.162
171	200804_at	TMBIM6	0.70981	0.68289	0.66186	0.04795	0.338
172	200805_at	LMAN2	0.23507	0.38729	0.58135	-0.34628	0.998
173	200806_s_at	HSPD1	0.87301	0.85412	0.83752	0.03549	0.259
174	200808_s_at	ZYX	0.50178	0.7112	0.6757	-0.17392	0.95
175	200811_at	CIRBP	0.33403	0.25398	0.24897	0.08506	0.296
176	200812_at	CCT7	0.75535	0.66183	0.6784	0.07695	0.164
177	200814_at	PSME1	0.80297	0.82478	0.8498	-0.04683	0.789
178	200815_s_at	PAFAH1B1	0.44713	0.62097	0.5349	-0.08777	0.729
179	200818_at	ATP5O	0.85382	0.84903	0.88742	-0.0336	0.765
180	200819_s_at	RPS15	0.95845	0.92114	0.93486	0.02359	0.004
181	200820_at	PSMD8	0.8175	0.81314	0.85363	-0.03613	0.715
182	200824_at	GSTP1	0.59814	0.55279	0.626	-0.02786	0.61
183	200825_s_at	HYOU1	0.52142	0.56054	0.56367	-0.04225	0.672
184	200826_at	SNRPD2	0.94002	0.92177	0.93433	0.00569	0.439
185	200827_at	PLOD1	0.72126	0.68092	0.49785	0.22341	0.002
186	200828_s_at	ZNF207	0.64735	0.38921	0.42666	0.22069	0.003
187	200830_at	PSMD2	0.65481	0.77284	0.74372	-0.08891	0.781
188	200833_s_at	hCG_1757335	0.57944	0.66825	0.52226	0.05718	0.347
189	200834_s_at	RPS21	0.93214	0.90814	0.92063	0.01151	0.187
190	200837_at	BCAP31	0.41494	0.48497	0.60194	-0.187	0.894
191	200840_at	KARS	0.80665	0.84581	0.77732	0.02933	0.427
192	200842_s_at	EPRS	0.67177	0.6315	0.69773	-0.02596	0.598
193	200845_s_at	PRDX6	0.35678	0.41177	0.31055	0.04623	0.382
194	200846_s_at	PPP1CA	0.72231	0.73576	0.78066	-0.05835	0.786
195	200847_s_at	TMEM66	0.52813	0.61188	0.65589	-0.12776	0.907
196	200851_s_at	KIAA0174	0.73452	0.62287	0.53767	0.19685	0.041
197	200853_at	H2AFZ	0.89489	0.89639	0.8792	0.01569	0.329
198	200854_at	NCOR1	0.69925	0.75608	0.70256	-0.00331	0.51
199	200855_at	C20orf191	0.34539	0.36951	0.45903	-0.11364	0.821
200	200860_s_at	CNOT1	0.78825	0.83634	0.73419	0.05406	0.172
201	200862_at	DHCR24	0.55114	0.61292	0.46972	0.08142	0.271
202	200863_s_at	RAB11A	0.70621	0.78221	0.78066	-0.07445	0.775
203	200870_at	STRAP	0.26751	0.26236	0.3997	-0.13219	0.827
204	200871_s_at	PSAP	0.66765	0.65979	0.54142	0.12623	0.131
205	200873_s_at	CCT8	0.66467	0.83066	0.75377	-0.0891	0.86
206	200875_s_at	NOP56	0.90533	0.88499	0.89899	0.00634	0.455
207	200877_at	CCT4	0.7275	0.80301	0.73334	-0.00584	0.559
208	200881_s_at	DNAJA1	0.56739	0.50276	0.28207	0.28532	0.01
209	200882_s_at	PSMD4	0.79301	0.8382	0.79656	-0.00355	0.516

210	200885_at	RHOC	0.64913	0.77818	0.73786	-0.08873	0.825
211	200886_s_at	PGAM1	0.80579	0.79044	0.64135	0.16444	0.039
212	200891_s_at	SSR1	0.58364	0.64698	0.57075	0.01289	0.454
213	200892_s_at	TRA2B	0.55425	0.70748	0.48982	0.06443	0.322
214	200894_s_at	FKBP4	0.61105	0.66073	0.62357	-0.01252	0.54
215	200900_s_at	M6PR	0.41511	0.5737	0.44599	-0.03088	0.607
216	200902_at	SEP15	0.67028	0.71529	0.73219	-0.06191	0.753
217	200903_s_at	AHCY	0.72914	0.6565	0.66946	0.05968	0.211
218	200904_at	HLA-E	0.56881	0.60608	0.50224	0.06657	0.202
219	200908_s_at	RPLP2	0.691	0.71243	0.77772	-0.08672	0.898
220	200910_at	CCT3	0.81612	0.81803	0.71375	0.10237	0.153
221	200911_s_at	TACC1	0.68325	0.77846	0.70901	-0.02576	0.653
222	200912_s_at	EIF4A2	0.81595	0.80622	0.73818	0.07777	0.127
223	200913_at	PPM1G	0.61314	0.60666	0.56116	0.05198	0.341
224	200918_s_at	SRPR	0.35617	0.48971	0.46364	-0.10747	0.85
225	200919_at	PHC2	0.52399	0.47678	0.4659	0.05809	0.34
226	200920_s_at	BTG1	0.5731	0.50206	0.60329	-0.03019	0.595
227	200922_at	KDELR1	0.29443	0.22066	0.25781	0.03662	0.428
228	200924_s_at	SLC3A2	0.46196	0.1932	0.23271	0.22925	0.016
229	200925_at	COX6A1	0.94934	0.93442	0.95365	-0.00431	0.593
230	200926_at	RPS23	0.93296	0.94264	0.93982	-0.00686	0.678
231	200928_s_at	RAB14	0.3814	0.16901	0.15341	0.22799	0.052
232	200932_s_at	DCTN2	0.36226	0.54398	0.44335	-0.08109	0.741
233	200934_at	DEK	0.75904	0.68809	0.74767	0.01137	0.463
234	200935_at	CALR	0.48405	0.39302	0.34618	0.13787	0.123
235	200936_at	RPL8	0.91767	0.91393	0.93793	-0.02026	0.872
236	200937_s_at	RPL5	0.92338	0.9203	0.94145	-0.01807	0.827
237	200941_at	HSBP1	0.45525	0.32688	0.41617	0.03908	0.409
238	200944_s_at	HMGN1	0.8126	0.84746	0.83792	-0.02532	0.69
239	200945_s_at	SEC31A	0.59722	0.70924	0.65698	-0.05976	0.794
240	200947_s_at	GLUD1	0.60057	0.58278	0.5605	0.04007	0.344
241	200948_at	MLF2	0.63381	0.694	0.70038	-0.06657	0.725
242	200951_s_at	CCND2	0.56308	0.60161	0.63309	-0.07001	0.804
243	200955_at	IMMT	0.73451	0.7233	0.68688	0.04763	0.325
244	200957_s_at	SSRP1	0.78723	0.77038	0.77166	0.01557	0.444
245	200958_s_at	SDCBP	0.55993	0.61073	0.54849	0.01144	0.468
246	200959_at	FUS	0.52133	0.7951	0.77392	-0.25259	0.939
247	200961_at	SEPHS2	0.67388	0.72022	0.80808	-0.1342	0.927
248	200964_at	UBA1	0.62317	0.77465	0.70408	-0.08091	0.815
249	200965_s_at	ABLIM1	0.53787	0.61524	0.48457	0.0533	0.372
250	200967_at	PPIB	0.71207	0.7367	0.76174	-0.04967	0.747
251	200971_s_at	SERP1	0.78613	0.76304	0.76198	0.02415	0.355
252	200973_s_at	TSPAN3	0.43666	0.42474	0.39769	0.03897	0.397
253	200975_at	PPT1	0.10392	0.18802	0.2821	-0.17818	0.863
254	200977_s_at	TAX1BP1	0.59437	0.38365	0.23385	0.36052	0.002
255	200978_at	MDH1	0.82765	0.77523	0.86895	-0.0413	0.812
256	200980_s_at	PDHA1	0.78339	0.6874	0.55493	0.22846	0.007
257	200982_s_at	ANXA6	0.6435	0.7283	0.67433	-0.03083	0.633
258	200984_s_at	CD59	0.43468	0.60653	0.62714	-0.19246	0.973
259	200989_at	HIF1A	0.45597	0.45014	0.43859	0.01738	0.476
260	200990_at	TRIM28	0.77825	0.87826	0.85865	-0.0804	0.966
261	200991_s_at	SNX17	0.62058	0.54613	0.60603	0.01455	0.451
262	200994_at	IPO7	0.33474	0.54229	0.37917	-0.04443	0.647

263	200997_at	RBM4	0.66345	0.65347	0.67042	-0.00697	0.533
264	200998_s_at	CKAP4	0.24401	0.42215	0.47928	-0.23527	0.98
265	201000_at	AARS	0.77868	0.79163	0.78434	-0.00566	0.535
266	201002_s_at	TMEM189-UBE2V1	0.51969	0.49654	0.46835	0.05134	0.348
267	201004_at	SSR4	0.81589	0.80382	0.89743	-0.08154	0.859
268	201007_at	HADHB	0.50433	0.43082	0.44307	0.06126	0.337
269	201008_s_at	TXNIP	0.33097	0.31765	0.54781	-0.21684	0.939
270	201011_at	RPN1	0.5316	0.54576	0.52452	0.00708	0.479
271	201012_at	ANXA1	0.14238	0.33311	0.29688	-0.1545	0.818
272	201017_at	EIF1AX	0.25021	0.35334	0.2488	0.00141	0.488
273	201019_s_at	EIF1AP1	0.66904	0.71566	0.64505	0.02399	0.414
274	201020_at	YWHAH	0.57687	0.64755	0.58623	-0.00936	0.513
275	201021_s_at	DSTN	0.3827	0.60687	0.60655	-0.22385	0.965
276	201023_at	TAF7	0.45913	0.31856	0.53561	-0.07648	0.737
277	201028_s_at	CD99	0.15287	0.50816	0.44461	-0.29174	0.971
278	201031_s_at	HNRNPH1	0.72185	0.67666	0.68242	0.03943	0.403
279	201032_at	BLCAP	0.53961	0.52313	0.45909	0.08052	0.231
280	201037_at	PFKP	0.6298	0.63537	0.48643	0.14337	0.063
281	201039_s_at	RAD23A	0.54282	0.60764	0.48024	0.06258	0.254
282	201041_s_at	DUSP1	0.65046	0.37531	0.3461	0.30436	0.004
283	201049_s_at	RPS18	0.94141	0.936	0.94348	-0.00207	0.565
284	201051_at	ANP32A	0.58264	0.6271	0.56876	0.01388	0.455
285	201053_s_at	PSMF1	0.51359	0.57976	0.64743	-0.13384	0.839
286	201055_s_at	HNRNPA0	0.10772	0.18749	0.12476	-0.01704	0.586
287	201056_at	GOLGB1	0.3694	0.68911	0.44063	-0.07123	0.686
288	201063_at	RCN1	0.57679	0.56079	0.56409	0.0127	0.446
289	201064_s_at	PABPC4	0.74249	0.70055	0.72656	0.01593	0.465
290	201066_at	CYC1	0.73013	0.74382	0.82257	-0.09244	0.943
291	201068_s_at	PSMC2	0.35392	0.5204	0.65274	-0.29882	0.958
292	201074_at	SMARCC1	0.67981	0.67786	0.6469	0.03291	0.354
293	201077_s_at	NHP2L1	0.92216	0.88321	0.91132	0.01084	0.4
294	201078_at	TM9SF2	0.71599	0.71977	0.72882	-0.01283	0.601
295	201079_at	SYNGR2	0.27479	0.54152	0.67675	-0.40196	1
296	201085_s_at	SON	0.51786	0.61307	0.56946	-0.0516	0.64
297	201088_at	KPNA2	0.70912	0.59043	0.17003	0.53909	<0.001
298	201089_at	ATP6V1B2	0.83268	0.86172	0.8814	-0.04872	0.799
299	201091_s_at	CBX3	0.79478	0.69727	0.82605	-0.03127	0.7
300	201092_at	RBBP7	0.70225	0.61217	0.57747	0.12478	0.143
301	201094_at	RPS29	0.94704	0.93047	0.91136	0.03568	0.001
302	201095_at	DAP	0.26895	0.53539	0.42092	-0.15197	0.847
303	201097_s_at	ARF4	0.089199	0.36202	0.3407	-0.251501	0.935
304	201098_at	COPB2	0.77095	0.78786	0.68742	0.08353	0.14
305	201099_at	USP9X	0.43451	0.55735	0.36935	0.06516	0.43
306	201101_s_at	BCLAF1	0.7398	0.76134	0.81671	-0.07691	0.835
307	201102_s_at	PFKL	0.77068	0.73155	0.70006	0.07062	0.314
308	201106_at	GPX4	0.82682	0.67967	0.75016	0.07666	0.15
309	201112_s_at	CSE1L	0.7242	0.71366	0.69139	0.03281	0.335
310	201113_at	TUFM	0.7296	0.74636	0.75091	-0.02131	0.64
311	201115_at	POLD2	0.58751	0.74625	0.64511	-0.0576	0.783
312	201118_at	PGD	0.5915	0.62805	0.56931	0.02219	0.439
313	201119_s_at	COX8A	0.91598	0.88076	0.90984	0.00614	0.46
314	201121_s_at	PGRMC1	0.6146	0.51076	0.48202	0.13258	0.072
315	201126_s_at	MGAT1	0.65052	0.72395	0.66054	-0.01002	0.56

316	201128_s_at	ACLY	0.72623	0.67916	0.71288	0.01335	0.454
317	201129_at	SFRS7	0.87294	0.85983	0.70046	0.17248	0.013
318	201132_at	HNRNPH2	0.58419	0.50277	0.53771	0.04648	0.347
319	201133_s_at	PJA2	0.87308	0.77512	0.88235	-0.00927	0.575
320	201135_at	ECHS1	0.78334	0.80601	0.86416	-0.08082	0.949
321	201136_at	PLP2	0.27288	0.46026	0.3939	-0.12102	0.86
322	201137_s_at	HLA-DPB1	0.43874	0.50457	0.4576	-0.01886	0.581
323	201139_s_at	SSB	0.76663	0.7347	0.70383	0.0628	0.138
324	201141_at	GPNMB	0.21176	0.33616	0.24902	-0.03726	0.66
325	201144_s_at	EIF2S1	0.64922	0.69215	0.68728	-0.03806	0.651
326	201145_at	HAX1	0.62308	0.68444	0.70671	-0.08363	0.719
327	201146_at	NFE2L2	0.593	0.62144	0.64535	-0.05235	0.671
328	201155_s_at	MFN2	0.17162	0.49101	0.32236	-0.15074	0.878
329	201156_s_at	RAB5C	0.63587	0.574	0.62557	0.0103	0.501
330	201158_at	NMT1	0.65276	0.67784	0.59555	0.05721	0.323
331	201165_s_at	PUM1	0.59273	0.40285	0.48296	0.10977	0.282
332	201170_s_at	BHLHE40	0.79327	0.62186	0.6272	0.16607	0.067
333	201174_s_at	TERF2IP	0.44018	0.51181	0.40464	0.03554	0.438
334	201175_at	TMX2	0.51154	0.61409	0.55779	-0.04625	0.62
335	201176_s_at	ARCN1	0.33674	0.28555	0.36678	-0.03004	0.563
336	201177_s_at	UBA2	0.68611	0.63705	0.73532	-0.04921	0.787
337	201178_at	FBXO7	0.48008	0.27235	0.4929	-0.01282	0.57
338	201180_s_at	GNAI3	0.57511	0.61869	0.68071	-0.1056	0.885
339	201182_s_at	CHD4	0.50351	0.41438	0.3522	0.15131	0.104
340	201186_at	LRPAP1	0.55965	0.55292	0.58013	-0.02048	0.585
341	201191_at	PITPNA	0.42131	0.62706	0.47841	-0.0571	0.65
342	201193_at	IDH1	0.43479	0.56574	0.45263	-0.01784	0.583
343	201194_at	SEPW1	0.68314	0.64261	0.58985	0.09329	0.201
344	201195_s_at	SLC7A5	0.54606	0.44472	0.39094	0.15512	0.11
345	201197_at	AMD1	0.67814	0.62485	0.63043	0.04771	0.242
346	201198_s_at	PSMD1	0.769	0.73285	0.74835	0.02065	0.38
347	201200_at	CREG1	0.40157	0.33094	0.28408	0.11749	0.163
348	201201_at	CSTB	0.5235	0.61645	0.66844	-0.14494	0.847
349	201204_s_at	RRBP1	0.52577	0.57942	0.46862	0.05715	0.305
350	201209_at	HDAC1	0.55418	0.63099	0.5843	-0.03012	0.612
351	201212_at	LGMN	0.11444	0.29201	0.13253	-0.01809	0.559
352	201214_s_at	PPP1R7	0.69002	0.5896	0.75866	-0.06864	0.728
353	201215_at	PLS3	0.069312	0.18087	0.19566	-0.126348	0.785
354	201216_at	ERP29	0.57998	0.7765	0.74536	-0.16538	0.905
355	201221_s_at	SNRNP70	0.53692	0.66094	0.57184	-0.03492	0.609
356	201223_s_at	RAD23B	0.74034	0.57905	0.52035	0.21999	0.004
357	201224_s_at	SRRM1	0.74133	0.63305	0.7036	0.03773	0.285
358	201226_at	NDUFB8	0.83921	0.80637	0.87334	-0.03413	0.666
359	201228_s_at	ARIH2	0.34829	0.16213	0.28913	0.05916	0.373
360	201231_s_at	ENO1	0.90813	0.8936	0.85141	0.05672	0.156
361	201232_s_at	PSMD13	0.60656	0.65527	0.64507	-0.03851	0.668
362	201234_at	ILK	0.44378	0.48228	0.54793	-0.10415	0.812
363	201235_s_at	BTG2	0.75594	0.56628	0.62314	0.1328	0.12
364	201238_s_at	CAPZA2	0.75376	0.78367	0.76622	-0.01246	0.584
365	201240_s_at	LOC653566	0.69272	0.755	0.65037	0.04235	0.394
366	201241_at	DDX1	0.44272	0.50133	0.56889	-0.12617	0.822
367	201243_s_at	ATP1B1	0.7937	0.78213	0.69297	0.10073	0.116
368	201244_s_at	RAF1	0.85457	0.83607	0.77778	0.07679	0.093

369	201246_s_at	OTUB1	0.11002	0.28701	0.33148	-0.22146	0.926
370	201248_s_at	SREBF2	0.49619	0.3972	0.21593	0.28026	0.024
371	201250_s_at	SLC2A1	0.22545	0.14082	0.11403	0.11142	0.22
372	201252_at	PSMC4	0.83791	0.7277	0.73174	0.10617	0.036
373	201253_s_at	CDIPT	0.0039997	0.065293	0.16746	-0.1634603	0.881
374	201256_at	COX7A2L	0.81403	0.81935	0.8192	-0.00517	0.549
375	201258_at	RPS16	0.93327	0.92619	0.9317	0.00157	0.478
376	201260_s_at	SYPL1	0.31947	0.44586	0.39512	-0.07565	0.72
377	201263_at	TARS	0.54138	0.58601	0.5487	-0.00732	0.506
378	201266_at	TXNRD1	0.82299	0.68103	0.66775	0.15524	0.034
379	201267_s_at	PSMC3	0.74102	0.8328	0.80341	-0.06239	0.761
380	201268_at	NME1	0.88377	0.9124	0.93774	-0.05397	0.994
381	201271_s_at	RALY	0.62055	0.67797	0.79121	-0.17066	0.982
382	201272_at	AKR1B1	0.70092	0.62607	0.6226	0.07832	0.222
383	201273_s_at	SRP9	0.7592	0.73591	0.75351	0.00569	0.472
384	201274_at	PSMA5	0.7315	0.76764	0.87521	-0.14371	0.948
385	201275_at	FDPS	0.61405	0.66585	0.69157	-0.07752	0.733
386	201276_at	RAB5B	0.61529	0.62887	0.60885	0.00644	0.48
387	201277_s_at	HNRNPAB	0.6514	0.76392	0.77507	-0.12367	0.964
388	201281_at	ADRM1	0.74412	0.77368	0.83386	-0.08974	0.992
389	201282_at	OGDH	0.50306	0.6317	0.4509	0.05216	0.338
390	201284_s_at	APEH	0.69529	0.70384	0.72228	-0.02699	0.645
391	201288_at	ARHGDIB	0.7399	0.78556	0.80891	-0.06901	0.823
392	201290_at	SEC11A	0.89826	0.90629	0.8841	0.01416	0.342
393	201291_s_at	TOP2A	0.85538	0.83466	0.72996	0.12542	0.072
394	201299_s_at	MOBKL1B	0.79298	0.85153	0.83076	-0.03778	0.835
395	201302_at	ANXA4	0.71198	0.66732	0.65924	0.05274	0.344
396	201303_at	EIF4A3	0.65504	0.63141	0.72555	-0.07051	0.659
397	201304_at	NDUFA5	0.67605	0.71388	0.65013	0.02592	0.419
398	201306_s_at	ANP32B	0.87844	0.84135	0.84871	0.02973	0.332
399	201312_s_at	SH3BGRL	0.78003	0.86358	0.78937	-0.00934	0.567
400	201313_at	ENO2	0.68104	0.76109	0.56483	0.11621	0.239
401	201314_at	STK25	0.45446	0.39057	0.47443	-0.01997	0.556
402	201316_at	PSMA2	0.72975	0.61265	0.39723	0.33252	0.001
403	201319_at	MYL12A	0.40447	0.44276	0.55202	-0.14755	0.881
404	201321_s_at	SMARCC2	0.60167	0.80759	0.72653	-0.12486	0.903
405	201322_at	ATP5B	0.8807	0.88395	0.88434	-0.00364	0.545
406	201323_at	EBNA1BP2	0.85908	0.86111	0.84514	0.01394	0.392
407	201327_s_at	CCT6A	0.60582	0.58298	0.63454	-0.02872	0.637
408	201330_at	RARS	0.13001	0.15856	0.075039	0.054971	0.37
409	201339_s_at	SCP2	0.75293	0.8074	0.68613	0.0668	0.137
410	201342_at	SNRPC	0.80914	0.78118	0.78693	0.02221	0.394
411	201346_at	ADIPOR2	0.41237	0.34122	0.46372	-0.05135	0.696
412	201349_at	SLC9A3R1	0.5376	0.72645	0.46591	0.07169	0.246
413	201350_at	FLOT2	0.56498	0.71946	0.67134	-0.10636	0.782
414	201351_s_at	YME1L1	0.76137	0.70356	0.73305	0.02832	0.364
415	201356_at	SF3A1	0.8602	0.81365	0.81919	0.04101	0.259
416	201358_s_at	COPB1	0.69872	0.65556	0.64379	0.05493	0.278
417	201360_at	CST3	0.54821	0.481	0.51063	0.03758	0.383
418	201361_at	TMEM109	0.4835	0.4447	0.40248	0.08102	0.245
419	201363_s_at	IVNS1ABP	0.62873	0.70096	0.65718	-0.02845	0.615
420	201364_s_at	OAZ2	0.57543	0.52266	0.37277	0.20266	0.135
421	201366_at	ANXA7	0.85864	0.84056	0.732	0.12664	0.04

422	201368_at	ZFP36L2	0.19195	0.24034	0.22306	-0.03111	0.601
423	201371_s_at	CUL3	0.73921	0.68971	0.75698	-0.01777	0.59
424	201375_s_at	PPP2CB	0.71946	0.50759	0.52585	0.19361	0.023
425	201376_s_at	HNRNPF	0.52244	0.49555	0.51669	0.00575	0.498
426	201379_s_at	TPD52L2	0.42105	0.40629	0.2882	0.13285	0.228
427	201380_at	CRTAP	0.34931	0.4851	0.50661	-0.1573	0.814
428	201382_at	CACYBP	0.17893	0.34782	0.17035	0.00858	0.486
429	201383_s_at	LOC100133166	0.66935	0.51837	0.5996	0.06975	0.278
430	201385_at	DHX15	0.74723	0.66344	0.63451	0.11272	0.141
431	201387_s_at	UCHL1	0.08265	0.18702	0.17665	-0.094	0.75
432	201388_at	PSMD3	0.58186	0.54369	0.43869	0.14317	0.142
433	201390_s_at	CSNK2B	0.87607	0.83318	0.91882	-0.04275	0.746
434	201391_at	TRAP1	0.4396	0.45349	0.47895	-0.03935	0.634
435	201392_s_at	IGF2R	0.4385	0.48232	0.56017	-0.12167	0.866
436	201395_at	RBM5	0.58766	0.67749	0.66252	-0.07486	0.794
437	201396_s_at	SGTA	0.46834	0.40932	0.55316	-0.08482	0.795
438	201397_at	PHGDH	0.39067	0.6207	0.59563	-0.20496	0.978
439	201400_at	PSMB3	0.87534	0.85235	0.90777	-0.03243	0.775
440	201403_s_at	MGST3	0.61717	0.72425	0.73563	-0.11846	0.843
441	201405_s_at	COPS6	0.55403	0.60361	0.71595	-0.16192	0.881
442	201407_s_at	PPP1CB	0.80937	0.81991	0.8124	-0.00303	0.568
443	201411_s_at	PLEKHB2	0.21586	0.34336	0.29727	-0.08141	0.69
444	201412_at	LRP10	0.8014	0.72868	0.79005	0.01135	0.456
445	201413_at	HSD17B4	0.41507	0.32362	0.30773	0.10734	0.199
446	201415_at	GSS	0.319	0.53418	0.51042	-0.19142	0.928
447	201417_at	SOX4	0.21586	0.4037	0.49199	-0.27613	0.918
448	201420_s_at	WDR77	0.67658	0.61252	0.64313	0.03345	0.395
449	201422_at	IFI30	0.72412	0.72661	0.78816	-0.06404	0.743
450	201423_s_at	CUL4A	0.7427	0.74871	0.65805	0.08465	0.156
451	201425_at	ALDH2	0.2696	0.47109	0.444	-0.1744	0.739
452	201426_s_at	VIM	0.55157	0.70279	0.66416	-0.11259	0.948
453	201427_s_at	SEPP1	0.119	0.32938	0.28632	-0.16732	0.883
454	201429_s_at	RPL37A	0.96503	0.94701	0.94773	0.0173	0.126
455	201433_s_at	PTDSS1	0.54895	0.59884	0.51934	0.02961	0.382
456	201434_at	TTC1	0.4693	0.43418	0.3768	0.0925	0.244
457	201437_s_at	EIF4E	0.82932	0.81687	0.83303	-0.00371	0.521
458	201439_at	GBF1	0.64884	0.67425	0.61469	0.03415	0.378
459	201441_at	COX6B1	0.91807	0.87161	0.91621	0.00186	0.482
460	201444_s_at	ATP6AP2	0.73566	0.7188	0.5975	0.13816	0.026
461	201445_at	CNN3	0.1433	0.32182	0.32661	-0.18331	0.897
462	201447_at	TIA1	0.5638	0.63759	0.57975	-0.01595	0.545
463	201459_at	RUVBL2	0.73323	0.81854	0.74728	-0.01405	0.571
464	201460_at	MAPKAPK2	0.44183	0.46354	0.55117	-0.10934	0.826
465	201462_at	SCRN1	0.095306	0.2279	0.25175	-0.156444	0.791
466	201463_s_at	LOC100133665	0.70963	0.78201	0.8	-0.09037	0.859
467	201466_s_at	JUN	0.61349	0.33343	0.58218	0.03131	0.395
468	201468_s_at	NQO1	0.42163	0.59243	0.51579	-0.09416	0.722
469	201470_at	GSTO1	0.82103	0.83488	0.86807	-0.04704	0.75
470	201472_at	VBP1	0.74746	0.74694	0.63474	0.11272	0.139
471	201473_at	JUNB	0.60708	0.39349	0.42725	0.17983	0.085
472	201477_s_at	RRM1	0.64726	0.54299	0.55031	0.09695	0.125
473	201478_s_at	DKC1	0.45737	0.58575	0.50544	-0.04807	0.661
474	201480_s_at	SUPT5H	0.71415	0.73554	0.69757	0.01658	0.454

475	201484_at	SUPT4H1	0.49242	0.52778	0.54225	-0.04983	0.605
476	201486_at	RCN2	0.85287	0.67727	0.69503	0.15784	0.021
477	201487_at	CTSC	0.21875	0.43645	0.43271	-0.21396	0.949
478	201489_at	PPIF	0.39552	0.54275	0.52959	-0.13407	0.817
479	201491_at	AHSA1	0.67026	0.72544	0.39401	0.27625	0.017
480	201492_s_at	RPL41	0.92143	0.93548	0.9205	0.00093	0.513
481	201493_s_at	PUM2	0.85716	0.83381	0.87245	-0.01529	0.617
482	201494_at	PRCP	0.41063	0.52603	0.43053	-0.0199	0.564
483	201499_s_at	USP7	0.68019	0.42835	0.411	0.26919	0.021
484	201500_s_at	PPP1R11	0.72409	0.79201	0.79126	-0.06717	0.844
485	201502_s_at	NFKBIA	0.55819	0.34043	0.46814	0.09005	0.304
486	201507_at	PFDN1	0.10647	0.014379	0.095426	0.011044	0.461
487	201511_at	AAMP	0.29819	0.36624	0.30925	-0.01106	0.55
488	201512_s_at	TOMM70A	0.46373	0.59093	0.52926	-0.06553	0.695
489	201513_at	TSN	0.74193	0.63004	0.59534	0.14659	0.121
490	201514_s_at	G3BP1	0.64568	0.59073	0.51944	0.12624	0.148
491	201516_at	SRM	0.71464	0.76524	0.80553	-0.09089	0.825
492	201521_s_at	NCBP2	0.28889	0.48335	0.43422	-0.14533	0.879
493	201526_at	ARF5	0.62074	0.64406	0.74384	-0.1231	0.821
494	201527_at	ATP6V1F	0.60355	0.65033	0.71134	-0.10779	0.758
495	201528_at	RPA1	0.53027	0.59794	0.71865	-0.18838	0.918
496	201531_at	ZFP36	0.35587	0.43024	0.268	0.08787	0.248
497	201532_at	PSMA3	0.71274	0.74242	0.77865	-0.06591	0.789
498	201533_at	CTNNB1	0.57891	0.50903	0.58596	-0.00705	0.511
499	201534_s_at	UBL3	0.24235	0.20729	0.36362	-0.12127	0.783
500	201540_at	FHL1	0.066072	0.21686	0.15014	-0.084068	0.761
501	201541_s_at	ZNHIT1	0.72197	0.76498	0.8158	-0.09383	0.836
502	201543_s_at	SAR1A	0.61945	0.55932	0.56027	0.05918	0.287
503	201546_at	TRIP12	0.84942	0.85287	0.804	0.04542	0.187
504	201553_s_at	LAMP1	0.68194	0.5338	0.52575	0.15619	0.005
505	201555_at	MCM3	0.71579	0.73374	0.64378	0.07201	0.226
506	201557_at	VAMP2	0.53325	0.53694	0.46795	0.0653	0.303
507	201561_s_at	CLSTN1	0.53304	0.58802	0.55069	-0.01765	0.529
508	201563_at	SORD	0.16344	0.38483	0.27657	-0.11313	0.803
509	201564_s_at	FSCN1	0.57685	0.76689	0.62891	-0.05206	0.702
510	201565_s_at	ID2	0.62386	0.61476	0.62913	-0.00527	0.534
511	201567_s_at	GOLGA4	0.66184	0.66844	0.77828	-0.11644	0.898
512	201568_at	UQCRQ	0.88246	0.86462	0.89817	-0.01571	0.616
513	201570_at	SAMM50	0.21064	0.40613	0.36656	-0.15592	0.873
514	201574_at	ETF1	0.81712	0.70851	0.6239	0.19322	0.006
515	201576_s_at	GLB1	0.67812	0.67379	0.6234	0.05472	0.346
516	201578_at	PODXL	0.013771	0.12802	0.085738	-0.071967	0.705
517	201580_s_at	TMX4	0.25925	0.40528	0.41866	-0.15941	0.889
518	201582_at	SEC23B	0.62587	0.64656	0.58996	0.03591	0.382
519	201584_s_at	DDX39	0.76583	0.80753	0.77172	-0.00589	0.545
520	201585_s_at	SFPQ	0.67391	0.65568	0.55199	0.12192	0.096
521	201587_s_at	IRAK1	0.82205	0.66552	0.66162	0.16043	0.015
522	201588_at	TXNL1	0.73671	0.70546	0.79526	-0.05855	0.716
523	201591_s_at	NISCH	0.75188	0.76182	0.73754	0.01434	0.426
524	201592_at	EIF3H	0.8834	0.91409	0.91463	-0.03123	0.892
525	201594_s_at	PPP4R1	0.57358	0.52238	0.60863	-0.03505	0.572
526	201597_at	COX7A2	0.91702	0.88005	0.90068	0.01634	0.274
527	201598_s_at	INPPL1	0.22434	0.30611	0.15882	0.06552	0.309

528	201599_at	OAT	0.085959	0.30355	0.20819	-0.122231	0.85
529	201600_at	PHB2	0.77131	0.79862	0.84182	-0.07051	0.92
530	201604_s_at	PPP1R12A	0.80789	0.72462	0.77964	0.02825	0.365
531	201608_s_at	PWP1	0.27827	0.34281	0.31968	-0.04141	0.65
532	201612_at	ALDH9A1	0.50765	0.56773	0.61465	-0.107	0.735
533	201613_s_at	AP1G2	0.53654	0.57657	0.63001	-0.09347	0.842
534	201614_s_at	RUVBL1	0.73669	0.73407	0.67129	0.0654	0.208
535	201619_at	PRDX3	0.41699	0.42921	0.4329	-0.01591	0.527
536	201620_at	MBTPS1	0.42017	0.5039	0.50985	-0.08968	0.787
537	201624_at	DARS	0.59925	0.496	0.41499	0.18426	0.082
538	201626_at	INSIG1	0.32971	0.57058	0.4397	-0.10999	0.792
539	201628_s_at	RRAGA	0.46463	0.50318	0.51903	-0.0544	0.692
540	201629_s_at	ACP1	0.50695	0.60541	0.58317	-0.07622	0.743
541	201631_s_at	IER3	0.54357	0.42299	0.46363	0.07994	0.316
542	201632_at	EIF2B1	0.42835	0.42736	0.36549	0.06286	0.37
543	201633_s_at	CYB5B	0.45589	0.57667	0.49195	-0.03606	0.659
544	201637_s_at	FXR1	0.80405	0.70375	0.71188	0.09217	0.195
545	201639_s_at	CPSF1	0.49926	0.62646	0.48032	0.01894	0.464
546	201641_at	BST2	0.69949	0.69595	0.74764	-0.04815	0.753
547	201642_at	IFNGR2	0.24364	0.25301	0.38292	-0.13928	0.863
548	201644_at	TSTA3	0.60315	0.57423	0.71851	-0.11536	0.892
549	201647_s_at	SCARB2	0.27192	0.43538	0.31874	-0.04682	0.637
550	201648_at	JAK1	0.66081	0.64796	0.6436	0.01721	0.485
551	201649_at	UBE2L6	0.68512	0.72826	0.70783	-0.02271	0.623
552	201651_s_at	PACSIN2	0.64035	0.69617	0.71972	-0.07937	0.772
553	201652_at	COPS5	0.79679	0.73845	0.51503	0.28176	0.02
554	201653_at	CNIH	0.73876	0.82079	0.74177	-0.00301	0.507
555	201657_at	ARL1	0.59564	0.59708	0.53107	0.06457	0.272
556	201662_s_at	ACSL3	0.52887	0.68822	0.72921	-0.20034	0.986
557	201663_s_at	SMC4	0.74626	0.7867	0.79531	-0.04905	0.685
558	201666_at	TIMP1	0.42177	0.46455	0.592	-0.17023	0.913
559	201672_s_at	USP14	0.39044	0.19125	0.34739	0.04305	0.371
560	201673_s_at	GYS1	0.76216	0.76626	0.74542	0.01674	0.429
561	201677_at	C3orf37	0.46671	0.28752	0.19462	0.27209	0.001
562	201682_at	PMPCB	0.71544	0.69342	0.57008	0.14536	0.133
563	201684_s_at	TOX4	0.59285	0.54212	0.68629	-0.09344	0.77
564	201687_s_at	API5	0.7848	0.75403	0.76081	0.02399	0.39
565	201689_s_at	TPD52	0.68689	0.75692	0.75149	-0.0646	0.765
566	201694_s_at	EGR1	0.2802	0.29127	0.51346	-0.23326	0.933
567	201695_s_at	NP	0.21878	0.53824	0.32712	-0.10834	0.811
568	201696_at	SFRS4	0.68449	0.75995	0.79418	-0.10969	0.893
569	201697_s_at	DNMT1	0.84435	0.78799	0.68564	0.15871	0.005
570	201699_at	PSMC6	0.8253	0.75111	0.51577	0.30953	<0.001
571	201700_at	CCND3	0.41679	0.45172	0.5447	-0.12791	0.87
572	201705_at	PSMD7	0.6577	0.64338	0.59679	0.06091	0.239
573	201707_at	PEX19	0.60696	0.47058	0.5024	0.10456	0.272
574	201709_s_at	NIPSNAP1	0.49792	0.52792	0.547	-0.04908	0.652
575	201710_at	MYBL2	0.55405	0.56391	0.55283	0.00122	0.498
576	201713_s_at	RANBP2	0.78992	0.76935	0.75718	0.03274	0.35
577	201714_at	TUBG1	0.70659	0.72207	0.65529	0.0513	0.203
578	201715_s_at	ACIN1	0.52864	0.40297	0.32997	0.19867	0.059
579	201716_at	SNX1	0.31042	0.47195	0.52257	-0.21215	0.925
580	201717_at	MRPL49	0.6991	0.54082	0.36114	0.33796	<0.001

581	201719_s_at	EPB41L2	0.33313	0.50562	0.46701	-0.13388	0.814
582	201721_s_at	LAPTM5	0.7871	0.78296	0.84484	-0.05774	0.796
583	201724_s_at	GALNT1	0.33514	0.42569	0.41545	-0.08031	0.692
584	201725_at	CDC123	0.45718	0.64362	0.67729	-0.22011	0.937
585	201726_at	ELAVL1	0.69134	0.76442	0.72946	-0.03812	0.637
586	201731_s_at	TPR	0.69336	0.85952	0.74942	-0.05606	0.726
587	201738_at	EIF1B	0.83879	0.82617	0.79783	0.04096	0.326
588	201739_at	SGK1	0.057015	0.1402	0.059345	-0.00233	0.546
589	201740_at	NDUFS3	0.82576	0.8492	0.84245	-0.01669	0.609
590	201746_at	TP53	0.72987	0.71044	0.67342	0.05645	0.237
591	201747_s_at	SAFB	0.52705	0.48608	0.55748	-0.03043	0.578
592	201751_at	JOSD1	0.70746	0.6799	0.60026	0.1072	0.2
593	201752_s_at	ADD3	0.32445	0.45318	0.51777	-0.19332	0.932
594	201754_at	COX6C	0.88136	0.8991	0.9202	-0.03884	0.925
595	201755_at	MCM5	0.82717	0.87287	0.84426	-0.01709	0.639
596	201756_at	RPA2	0.44725	0.54911	0.47966	-0.03241	0.621
597	201757_at	NDUFS5	0.82884	0.81557	0.84984	-0.021	0.628
598	201758_at	TSG101	0.20083	0.31188	0.28572	-0.08489	0.73
599	201760_s_at	WSB2	0.33395	0.55664	0.51209	-0.17814	0.85
600	201761_at	MTHFD2	0.49417	0.41426	0.34752	0.14665	0.074
601	201762_s_at	PSME2	0.86552	0.82041	0.9049	-0.03938	0.785
602	201763_s_at	DAXX	0.17595	0.1759	0.20961	-0.03366	0.586
603	201764_at	TMEM106C	0.48361	0.54305	0.5109	-0.02729	0.644
604	201768_s_at	CLINT1	0.28458	0.38521	0.43463	-0.15005	0.788
605	201770_at	SNRPA	0.78479	0.76304	0.77707	0.00772	0.46
606	201771_at	SCAMP3	0.68087	0.69511	0.69683	-0.01596	0.63
607	201772_at	AZIN1	0.66011	0.80784	0.76086	-0.10075	0.797
608	201774_s_at	NCAPD2	0.79784	0.75024	0.73281	0.06503	0.294
609	201777_s_at	KIAA0494	0.46592	0.3042	0.4819	-0.01598	0.562
610	201780_s_at	RNF13	0.58271	0.59499	0.60835	-0.02564	0.579
611	201781_s_at	AIP	0.56717	0.65847	0.63093	-0.06376	0.67
612	201783_s_at	RELA	0.47312	0.61464	0.63651	-0.16339	0.955
613	201786_s_at	ADAR	0.74325	0.75358	0.64975	0.0935	0.119
614	201788_at	DDX42	0.6119	0.67233	0.61187	3E-05	0.538
615	201791_s_at	DHCR7	0.30019	0.39457	0.35663	-0.05644	0.664
616	201795_at	LBR	0.74231	0.78595	0.73999	0.00232	0.519
617	201797_s_at	VARS	0.81023	0.81438	0.74079	0.06944	0.173
618	201800_s_at	OSBP	0.68558	0.45839	0.51544	0.17014	0.062
619	201802_at	SLC29A1	0.47062	0.60715	0.4613	0.00932	0.435
620	201803_at	POLR2B	0.79268	0.70749	0.62436	0.16832	0.048
621	201805_at	PRKAG1	0.44637	0.48329	0.46214	-0.01577	0.558
622	201807_at	VPS26A	0.58464	0.73805	0.57389	0.01075	0.454
623	201810_s_at	SH3BP5	0.51904	0.5327	0.56074	-0.0417	0.639
624	201812_s_at	C4orf46	0.87146	0.85073	0.85855	0.01291	0.359
625	201815_s_at	TBC1D5	0.55485	0.54009	0.60586	-0.05101	0.69
626	201816_s_at	GBAS	0.65699	0.56537	0.62457	0.03242	0.421
627	201817_at	UBE3C	0.54074	0.64621	0.633	-0.09226	0.788
628	201818_at	LPCAT1	0.60823	0.63446	0.55544	0.05279	0.309
629	201819_at	SCARB1	0.74339	0.8028	0.74722	-0.00383	0.555
630	201821_s_at	TIMM17A	0.64849	0.74858	0.58502	0.06347	0.277
631	201823_s_at	RNF14	0.72299	0.64237	0.61667	0.10632	0.147
632	201826_s_at	SCCPDH	0.48216	0.60112	0.50602	-0.02386	0.556
633	201827_at	SMARCD2	0.42071	0.26904	0.45875	-0.03804	0.627

634	201830_s_at	NET1	0.60652	0.80962	0.79549	-0.18897	0.99
635	201833_at	HDAC2	0.57286	0.53756	0.52314	0.04972	0.294
636	201834_at	PRKAB1	0.44956	0.36432	0.25122	0.19834	0.036
637	201837_s_at	SUPT7L	0.57473	0.3342	0.46518	0.10955	0.254
638	201840_at	NEDD8	0.78574	0.79449	0.8699	-0.08416	0.762
639	201841_s_at	HSPB1	0.68027	0.77455	0.74805	-0.06778	0.873
640	201845_s_at	RYBP	0.63262	0.55102	0.59452	0.0381	0.356
641	201847_at	LIPA	0.68622	0.7398	0.531	0.15522	0.162
642	201849_at	BNIP3	0.74653	0.67829	0.11687	0.62966	<0.001
643	201851_at	SH3GL1	0.48381	0.39875	0.3027	0.18111	0.126
644	201853_s_at	CDC25B	0.68612	0.68912	0.57295	0.11317	0.159
645	201854_s_at	ATMIN	0.74626	0.64969	0.64491	0.10135	0.154
646	201856_s_at	ZFR	0.7861	0.78921	0.76916	0.01694	0.402
647	201859_at	SRGN	0.75584	0.84011	0.86022	-0.10438	0.976
648	201861_s_at	LRRFIP1	0.329	0.52981	0.55329	-0.22429	0.971
649	201863_at	FAM32A	0.58505	0.59605	0.66494	-0.07989	0.753
650	201864_at	GDI1	0.58999	0.63262	0.62998	-0.03999	0.65
651	201870_at	TOMM34	0.35089	0.46798	0.47779	-0.1269	0.835
652	201872_s_at	ABCE1	0.7238	0.75036	0.83674	-0.11294	0.934
653	201874_at	MPZL1	0.52261	0.38703	0.38134	0.14127	0.134
654	201881_s_at	ARIH1	0.55687	0.523	0.56846	-0.01159	0.559
655	201885_s_at	CYB5R3	0.62595	0.71254	0.6572	-0.03125	0.628
656	201889_at	FAM3C	0.74143	0.66034	0.71362	0.02781	0.388
657	201890_at	RRM2	0.54245	0.6398	0.69833	-0.15588	0.91
658	201892_s_at	IMPDH2	0.56658	0.63812	0.7121	-0.14552	0.938
659	201895_at	ARAF	0.70698	0.61675	0.64849	0.05849	0.262
660	201896_s_at	PSRC1	0.50316	0.30743	0.043786	0.459374	<0.001
661	201897_s_at	CKS1B	0.85596	0.88549	0.81721	0.03875	0.267
662	201899_s_at	UBE2A	0.72529	0.66004	0.47886	0.24643	0.005
663	201900_s_at	AKR1A1	0.52487	0.70103	0.7073	-0.18243	0.959
664	201903_at	UQCRC1	0.60154	0.49656	0.58374	0.0178	0.413
665	201908_at	DVL3	0.51439	0.55585	0.55711	-0.04272	0.634
666	201912_s_at	GSPT1	0.47751	0.5003	0.31508	0.16243	0.045
667	201913_s_at	COASY	0.5933	0.40993	0.52242	0.07088	0.285
668	201914_s_at	SEC63	0.78161	0.73249	0.73364	0.04797	0.311
669	201917_s_at	SLC25A36	0.56355	0.51063	0.62298	-0.05943	0.675
670	201920_at	SLC20A1	0.51333	0.71245	0.3907	0.12263	0.236
671	201921_at	GNG10	0.35884	0.50177	0.41249	-0.05365	0.622
672	201922_at	TINP1	0.59796	0.68415	0.73703	-0.13907	0.919
673	201923_at	PRDX4	0.77116	0.84223	0.80503	-0.03387	0.65
674	201924_at	AFF1	0.60123	0.53848	0.66322	-0.06199	0.784
675	201925_s_at	CD55	0.42941	0.47108	0.42272	0.00669	0.496
676	201928_at	PKP4	0.51561	0.21183	0.050726	0.464884	0.016
677	201930_at	MCM6	0.62755	0.63628	0.71095	-0.0834	0.728
678	201931_at	ETFA	0.54991	0.6699	0.68202	-0.13211	0.856
679	201932_at	LRRC41	0.57836	0.79861	0.68215	-0.10379	0.86
680	201933_at	CHMP1A	0.53762	0.52873	0.40374	0.13388	0.062
681	201934_at	WDR82	0.6616	0.57997	0.68371	-0.02211	0.63
682	201936_s_at	EIF4G3	0.65712	0.66185	0.63155	0.02557	0.322
683	201937_s_at	DNPEP	0.69088	0.78224	0.77834	-0.08746	0.909
684	201938_at	CDK2AP1	0.20242	0.40102	0.4744	-0.27198	0.968
685	201939_at	PLK2	0.34551	0.36666	0.27662	0.06889	0.311
686	201944_at	HEXB	0.51191	0.46541	0.44495	0.06696	0.241

687	201947_s_at	CCT2	0.81674	0.83754	0.80253	0.01421	0.456
688	201948_at	GNL2	0.87189	0.80564	0.78576	0.08613	0.071
689	201952_at	ALCAM	0.21908	0.38085	0.2798	-0.06072	0.705
690	201953_at	CIB1	0.41445	0.653	0.60826	-0.19381	0.893
691	201954_at	ARPC1B	0.64571	0.70844	0.7486	-0.10289	0.861
692	201955_at	CCNC	0.72722	0.78054	0.76856	-0.04134	0.66
693	201956_s_at	GNPAT	0.67813	0.42523	0.5012	0.17693	0.019
694	201959_s_at	MYCBP2	0.71242	0.7821	0.71946	-0.00704	0.563
695	201963_at	ACSL1	0.6521	0.65562	0.60941	0.04269	0.335
696	201964_at	SETX	0.53112	0.49507	0.23259	0.29853	0.045
697	201966_at	NDUFS2	0.65715	0.59169	0.61807	0.03908	0.359
698	201967_at	RBM6	0.3089	0.53724	0.42114	-0.11224	0.75
699	201968_s_at	PGM1	0.80912	0.78431	0.54184	0.26728	<0.001
700	201970_s_at	NASP	0.81568	0.78696	0.78901	0.02667	0.401
701	201971_s_at	ATP6V1A	0.65313	0.67443	0.74177	-0.08864	0.82
702	201973_s_at	C7orf28A	0.26015	0.48645	0.32218	-0.06203	0.639
703	201975_at	CLIP1	0.47843	0.50777	0.46815	0.01028	0.486
704	201977_s_at	KIAA0141	0.53044	0.27703	0.46332	0.06712	0.28
705	201985_at	KIAA0196	0.70807	0.66942	0.70017	0.0079	0.5
706	201986_at	MED13	0.74365	0.72165	0.70382	0.03983	0.337
707	201990_s_at	CREBL2	0.69524	0.74083	0.63097	0.06427	0.281
708	201991_s_at	KIF5B	0.57799	0.57981	0.52955	0.04844	0.332
709	201994_at	MORF4L2	0.076084	0.11925	0.099136	-0.023052	0.519
710	201997_s_at	SPEN	0.63031	0.77732	0.67076	-0.04045	0.663
711	201999_s_at	DYNLT1	0.80612	0.74995	0.71405	0.09207	0.149
712	202001_s_at	NDUFA6	0.82025	0.81431	0.85185	-0.0316	0.617
713	202007_at	NID1	0.37155	0.60213	0.62434	-0.25279	0.749
714	202009_at	TWF2	0.24167	0.24236	0.36094	-0.11927	0.823
715	202010_s_at	ZNF410	0.48274	0.28777	0.16473	0.31801	0.011
716	202012_s_at	EXT2	0.58484	0.51203	0.57068	0.01416	0.458
717	202016_at	MEST	0.013002	0.04831	0.045583	-0.032581	0.836
718	202019_s_at	LANCL1	0.61884	0.64568	0.59715	0.02169	0.435
719	202022_at	ALDOC	0.79954	0.77907	0.67266	0.12688	0.041
720	202024_at	ASNA1	0.63534	0.59645	0.64386	-0.00852	0.542
721	202026_at	SDHD	0.83159	0.84085	0.7787	0.05289	0.208
722	202027_at	TMEM184B	0.23483	0.35734	0.29379	-0.05896	0.633
723	202028_s_at	RPL38	0.77398	0.75092	0.7859	-0.01192	0.569
724	202030_at	BCKDK	0.55641	0.60739	0.53267	0.02374	0.397
725	202033_s_at	RB1CC1	0.8638	0.78668	0.72657	0.13723	0.006
726	202038_at	UBE4A	0.65793	0.65542	0.67892	-0.02099	0.573
727	202040_s_at	KDM5A	0.51855	0.43309	0.31362	0.20493	0.076
728	202041_s_at	FIBP	0.62433	0.61138	0.624	0.00033	0.489
729	202042_at	HARS	0.6585	0.61946	0.65103	0.00747	0.48
730	202043_s_at	SMS	0.58	0.65138	0.60975	-0.02975	0.582
731	202050_s_at	ZMYM4	0.70992	0.51476	0.68511	0.02481	0.434
732	202053_s_at	ALDH3A2	0.20269	0.26458	0.1618	0.04089	0.499
733	202055_at	KPNA1	0.34715	0.46011	0.5815	-0.23435	0.944
734	202060_at	CTR9	0.50905	0.53098	0.52017	-0.01112	0.588
735	202064_s_at	SEL1L	0.44712	0.52947	0.53367	-0.08655	0.768
736	202069_s_at	IDH3A	0.7195	0.77178	0.7395	-0.02	0.613
737	202071_at	SDC4	0.53694	0.68378	0.62798	-0.09104	0.789
738	202072_at	HNRNPL	0.56815	0.41822	0.42434	0.14381	0.169
739	202074_s_at	OPTN	0.53034	0.48116	0.48049	0.04985	0.336

740	202075_s_at	PLTP	0.4014	0.37094	0.50745	-0.10605	0.789
741	202076_at	BIRC2	0.74907	0.70479	0.74742	0.00165	0.527
742	202077_at	NDUFAB1	0.91254	0.89298	0.9151	-0.00256	0.581
743	202078_at	COPS3	0.624	0.70547	0.7061	-0.0821	0.752
744	202080_s_at	TRAK1	0.65554	0.48976	0.47956	0.17598	0.146
745	202081_at	IER2	0.51621	0.21026	0.32788	0.18833	0.056
746	202083_s_at	SEC14L1	0.31674	0.40396	0.41796	-0.10122	0.776
747	202085_at	TJP2	0.82589	0.81684	0.77502	0.05087	0.175
748	202086_at	MX1	0.7037	0.76073	0.6613	0.0424	0.325
749	202089_s_at	SLC39A6	0.80781	0.84612	0.79396	0.01385	0.446
750	202090_s_at	UQCR	0.84368	0.76341	0.87295	-0.02927	0.637
751	202092_s_at	ARL2BP	0.63091	0.51932	0.51582	0.11509	0.218
752	202093_s_at	PAF1	0.24646	0.27508	0.16661	0.07985	0.297
753	202095_s_at	BIRC5	0.6982	0.73007	0.43972	0.25848	0.003
754	202096_s_at	TSPO	0.35704	0.25973	0.21579	0.14125	0.196
755	202097_at	NUP153	0.89837	0.76963	0.84865	0.04972	0.163
756	202100_at	RALB	0.26435	0.49297	0.4383	-0.17395	0.885
757	202103_at	BRD4	0.59596	0.60013	0.54828	0.04768	0.322
758	202104_s_at	SPG7	0.62081	0.63385	0.67109	-0.05028	0.765
759	202105_at	IGBP1	0.17168	0.30981	0.25039	-0.07871	0.674
760	202106_at	GOLGA3	0.36296	0.38452	0.28992	0.07304	0.349
761	202107_s_at	MCM2	0.71886	0.76934	0.73261	-0.01375	0.576
762	202108_at	PEPD	0.048814	0.089639	0.036183	0.012631	0.476
763	202109_at	ARFIP2	0.60423	0.6287	0.61853	-0.0143	0.591
764	202110_at	COX7B	0.89311	0.86214	0.87069	0.02242	0.272
765	202113_s_at	SNX2	0.68771	0.75559	0.63175	0.05596	0.325
766	202115_s_at	NOC2L	0.41759	0.45062	0.39347	0.02412	0.475
767	202116_at	DPF2	0.44064	0.27029	0.32878	0.11186	0.183
768	202117_at	ARHGAP1	0.82247	0.81458	0.82295	-0.00048	0.527
769	202119_s_at	CPNE3	0.66416	0.69712	0.6675	-0.00334	0.525
770	202121_s_at	CHMP2A	0.82439	0.81334	0.8785	-0.05411	0.737
771	202122_s_at	M6PRBP1	0.41878	0.44383	0.36537	0.05341	0.272
772	202123_s_at	ABL1	0.62082	0.53743	0.54655	0.07427	0.248
773	202124_s_at	TRAK2	0.31639	0.48149	0.49606	-0.17967	0.909
774	202126_at	PRPF4B	0.71124	0.44682	0.59903	0.11221	0.114
775	202128_at	KIAA0317	0.82843	0.81075	0.76596	0.06247	0.244
776	202129_s_at	RIOK3	0.74792	0.69831	0.74679	0.00113	0.502
777	202135_s_at	ACTR1B	0.59691	0.74565	0.69875	-0.10184	0.796
778	202136_at	ZMYND11	0.15301	0.16978	0.25829	-0.10528	0.709
779	202139_at	AKR7A2	0.68988	0.72413	0.75002	-0.06014	0.759
780	202140_s_at	CLK3	0.26595	0.19012	0.29599	-0.03004	0.569
781	202143_s_at	COPS8	0.40115	0.4443	0.3312	0.06995	0.317
782	202144_s_at	ADSL	0.77618	0.84499	0.81364	-0.03746	0.756
783	202145_at	LY6E	0.81018	0.70597	0.71339	0.09679	0.038
784	202146_at	IFRD1	0.53811	0.5366	0.3931	0.14501	0.158
785	202148_s_at	PYCR1	0.34284	0.49787	0.45812	-0.11528	0.779
786	202149_at	NEDD9	0.70488	0.72745	0.69356	0.01132	0.478
787	202162_s_at	CNOT8	0.60919	0.64062	0.59076	0.01843	0.44
788	202166_s_at	PPP1R2	0.71324	0.58237	0.73517	-0.02193	0.64
789	202167_s_at	MMS19	0.82583	0.65619	0.58297	0.24286	0.008
790	202168_at	TAF9	0.6962	0.73923	0.72428	-0.02808	0.688
791	202170_s_at	AASDHPPT	0.6905	0.74875	0.73059	-0.04009	0.653
792	202173_s_at	VEZF1	0.4785	0.73237	0.70229	-0.22379	0.897

793	202174_s_at	PCM1	0.6539	0.75761	0.74603	-0.09213	0.848
794	202179_at	BLMH	0.77641	0.66433	0.66948	0.10693	0.109
795	202180_s_at	MVP	0.70586	0.79701	0.83763	-0.13177	0.996
796	202181_at	KIAA0247	0.57391	0.59241	0.52487	0.04904	0.362
797	202182_at	KAT2A	0.37347	0.38901	0.45026	-0.07679	0.708
798	202184_s_at	NUP133	0.6898	0.7061	0.66498	0.02482	0.378
799	202185_at	PLOD3	0.62906	0.67999	0.50667	0.12239	0.065
800	202187_s_at	PPP2R5A	0.18752	0.32778	0.20213	-0.01461	0.55
801	202188_at	NUP93	0.48702	0.4734	0.49032	-0.0033	0.522
802	202190_at	CSTF1	0.50466	0.66953	0.49574	0.00892	0.457
803	202191_s_at	GAS7	0.16983	0.43504	0.33227	-0.16244	0.833
804	202193_at	LIMK2	0.58784	0.81345	0.54425	0.04359	0.354
805	202195_s_at	TMED5	0.4228	0.56675	0.69064	-0.26784	0.989
806	202200_s_at	SRPK1	0.81487	0.8561	0.7856	0.02927	0.325
807	202201_at	BLVRB	0.70239	0.54626	0.72707	-0.02468	0.606
808	202205_at	VASP	0.50011	0.50581	0.49849	0.00162	0.502
809	202209_at	LSM3	0.85819	0.85565	0.84862	0.00957	0.419
810	202211_at	ARFGAP3	0.6166	0.63424	0.60554	0.01106	0.442
811	202212_at	PES1	0.61847	0.72423	0.52368	0.09479	0.109
812	202213_s_at	CUL4B	0.76505	0.76355	0.74278	0.02227	0.386
813	202215_s_at	NFYC	0.54596	0.62692	0.49702	0.04894	0.294
814	202217_at	C21orf33	0.84266	0.83055	0.70659	0.13607	0.004
815	202218_s_at	FADS2	0.58411	0.43382	0.34562	0.23849	0.062
816	202220_at	KIAA0907	0.63938	0.33669	0.63907	0.00031	0.49
817	202221_s_at	EP300	0.61326	0.55066	0.66697	-0.05371	0.653
818	202223_at	STT3A	0.69642	0.63278	0.70094	-0.00452	0.542
819	202225_at	CRK	0.49737	0.04901	0.43851	0.05886	0.37
820	202227_s_at	BRD8	0.60828	0.37416	0.36897	0.23931	0.062
821	202228_s_at	NPTN	0.54519	0.70979	0.61563	-0.07044	0.702
822	202230_s_at	CHERP	0.79923	0.80755	0.75293	0.0463	0.291
823	202233_s_at	UQCRH	0.75982	0.83715	0.86431	-0.10449	0.919
824	202234_s_at	SLC16A1	0.61997	0.578	0.52066	0.09931	0.092
825	202239_at	PARP4	0.28585	0.4541	0.50592	-0.22007	0.923
826	202240_at	PLK1	0.66398	0.71854	0.23354	0.43044	<0.001
827	202241_at	TRIB1	0.61781	0.50356	0.5669	0.05091	0.304
828	202243_s_at	PSMB4	0.83852	0.8298	0.88916	-0.05064	0.897
829	202246_s_at	CDK4	0.53088	0.67158	0.68197	-0.15109	0.8
830	202249_s_at	WDR42A	0.24479	0.42705	0.32481	-0.08002	0.637
831	202251_at	PRPF3	0.62013	0.62802	0.50275	0.11738	0.246
832	202252_at	RAB13	0.54378	0.55221	0.5046	0.03918	0.394
833	202254_at	SIPA1L1	0.47102	0.35758	0.31549	0.15553	0.225
834	202257_s_at	CD2BP2	0.32181	0.40679	0.32938	-0.00757	0.513
835	202261_at	VPS72	0.58661	0.46394	0.48918	0.09743	0.141
836	202263_at	CYB5R1	0.3772	0.30018	0.49425	-0.11705	0.787
837	202264_s_at	TOMM40	0.51242	0.65564	0.65538	-0.14296	0.984
838	202265_at	BMI1	0.40489	0.48956	0.51836	-0.11347	0.831
839	202266_at	TTRAP	0.57896	0.66874	0.64773	-0.06877	0.733
840	202268_s_at	NAE1	0.51054	0.357	0.34953	0.16101	0.174
841	202270_at	GBP1	0.48641	0.47959	0.52401	-0.0376	0.581
842	202272_s_at	FBXO28	0.66412	0.65736	0.75791	-0.09379	0.752
843	202274_at	ACTG2	0.032483	0.082611	0.095633	-0.06315	0.674
844	202275_at	G6PD	0.45641	0.66977	0.58223	-0.12582	0.914
845	202276_at	SHFM1	0.84966	0.79484	0.84021	0.00945	0.416

846	202277_at	SPTLC1	0.77524	0.81477	0.82877	-0.05353	0.733
847	202279_at	C14orf2	0.80883	0.83784	0.84865	-0.03982	0.673
848	202282_at	HSD17B10	0.70867	0.64235	0.68285	0.02582	0.438
849	202284_s_at	CDKN1A	0.67422	0.43108	0.34322	0.331	0.001
850	202288_at	FRAP1	0.44159	0.51279	0.4028	0.03879	0.393
851	202294_at	STAG1	0.43604	0.50717	0.38681	0.04923	0.435
852	202295_s_at	CTSH	0.023073	0.42486	0.59929	-0.576217	1
853	202297_s_at	RER1	0.73885	0.55975	0.50812	0.23073	0.006
854	202298_at	NDUFA1	0.85732	0.81779	0.8497	0.00762	0.465
855	202300_at	HBXIP	0.82117	0.83729	0.83921	-0.01804	0.586
856	202301_s_at	RSRC2	0.37654	0.42596	0.45256	-0.07602	0.747
857	202306_at	POLR2G	0.81761	0.85565	0.86162	-0.04401	0.803
858	202307_s_at	TAP1	0.64457	0.71861	0.73661	-0.09204	0.841
859	202309_at	MTHFD1	0.61588	0.54412	0.41736	0.19852	0.033
860	202313_at	PPP2R2A	0.49185	0.58526	0.59418	-0.10233	0.932
861	202321_at	GGPS1	0.16637	0.25349	0.39294	-0.22657	0.954
862	202323_s_at	ACBD3	0.24603	0.19117	0.18759	0.05844	0.346
863	202325_s_at	ATP5J	0.90568	0.89755	0.92219	-0.01651	0.654
864	202329_at	CSK	0.68824	0.76784	0.69111	-0.00287	0.52
865	202330_s_at	UNG	0.62829	0.6863	0.64366	-0.01537	0.583
866	202331_at	BCKDHA	0.42301	0.39744	0.32391	0.0991	0.249
867	202336_s_at	PAM	0.62765	0.56098	0.41481	0.21284	0.018
868	202337_at	PMF1	0.58004	0.59608	0.62681	-0.04677	0.658
869	202338_at	TK1	0.78288	0.58889	0.59795	0.18493	0.026
870	202345_s_at	FABP5	0.52397	0.63608	0.67022	-0.14625	0.852
871	202347_s_at	UBE2K	0.66832	0.76104	0.62328	0.04504	0.371
872	202349_at	TOR1A	0.78074	0.56806	0.70953	0.07121	0.246
873	202351_at	ITGAV	0.52484	0.47219	0.61168	-0.08684	0.744
874	202352_s_at	PSMD12	0.79653	0.65012	0.47898	0.31755	<0.001
875	202355_s_at	GTF2F1	0.37367	0.48314	0.38288	-0.00921	0.49
876	202359_s_at	SNX19	0.44602	0.64614	0.64197	-0.19595	0.938
877	202360_at	MAML1	0.4478	0.49649	0.26205	0.18575	0.09
878	202361_at	SEC24C	0.81485	0.7066	0.6754	0.13945	0.013
879	202362_at	RAP1A	0.42562	0.46314	0.50508	-0.07946	0.672
880	202364_at	MXI1	0.38787	0.46747	0.42703	-0.03916	0.603
881	202365_at	UNC119B	0.44913	0.26417	0.1806	0.26853	0.021
882	202366_at	ACADS	0.0072058	0.30225	0.19473	-0.1875242	0.826
883	202369_s_at	TRAM2	0.26878	0.5108	0.57331	-0.30453	0.995
884	202371_at	TCEAL4	0.59357	0.63892	0.62727	-0.0337	0.614
885	202378_s_at	LEPROT	0.35798	0.38331	0.40743	-0.04945	0.674
886	202381_at	ADAM9	0.67804	0.75059	0.75102	-0.07298	0.898
887	202382_s_at	GNPDA1	0.40064	0.29938	0.43659	-0.03595	0.631
888	202384_s_at	TCOF1	0.71684	0.73091	0.48673	0.23011	0.048
889	202386_s_at	KIAA0430	0.62853	0.53561	0.5486	0.07993	0.224
890	202388_at	RGS2	0.39626	0.27642	0.24001	0.15625	0.18
891	202391_at	BASP1	0.31471	0.39318	0.43286	-0.11815	0.826
892	202392_s_at	PISD	0.70256	0.59954	0.63994	0.06262	0.167
893	202393_s_at	KLF10	0.54927	0.62161	0.65549	-0.10622	0.856
894	202394_s_at	ABCF3	0.5041	0.50446	0.55932	-0.05522	0.717
895	202395_at	NSF	0.4804	0.6592	0.65978	-0.17938	0.796
896	202396_at	TCERG1	0.84865	0.8057	0.74733	0.10132	0.059
897	202397_at	NUTF2	0.7679	0.75966	0.73696	0.03094	0.456
898	202399_s_at	AP3S2	0.060544	0.3023	0.38159	-0.321046	0.979

899	202402_s_at	CARS	0.57317	0.43272	0.27474	0.29843	0.005
900	202406_s_at	TIAL1	0.70799	0.67592	0.6256	0.08239	0.174
901	202413_s_at	USP1	0.77427	0.7112	0.71828	0.05599	0.153
902	202414_at	ERCC5	0.74433	0.73187	0.66282	0.08151	0.162
903	202415_s_at	HSPBP1	0.74091	0.73972	0.70786	0.03305	0.344
904	202417_at	KEAP1	0.44133	0.59546	0.60353	-0.1622	0.915
905	202418_at	YIF1A	0.71744	0.64588	0.63995	0.07749	0.27
906	202419_at	KDSR	0.56312	0.29657	0.34034	0.22278	0.046
907	202420_s_at	DHX9	0.73518	0.75694	0.67475	0.06043	0.23
908	202421_at	IGSF3	0.25127	0.50936	0.4305	-0.17923	0.883
909	202422_s_at	ACSL4	0.77612	0.80433	0.8258	-0.04968	0.776
910	202424_at	MAP2K2	0.63685	0.64935	0.67665	-0.0398	0.672
911	202427_s_at	BRP44	0.67859	0.72975	0.73484	-0.05625	0.738
912	202431_s_at	MYC	0.70536	0.57618	0.64012	0.06524	0.262
913	202432_at	PPP3CB	0.3799	0.37174	0.44605	-0.06615	0.674
914	202433_at	SLC35B1	0.4081	0.61557	0.62362	-0.21552	0.944
915	202435_s_at	CYP1B1	0.11001	0.27802	0.26929	-0.15928	0.909
916	202439_s_at	IDS	0.34823	0.59142	0.53587	-0.18764	0.968
917	202446_s_at	PLSCR1	0.67463	0.73741	0.68737	-0.01274	0.556
918	202447_at	DECR1	0.71293	0.83075	0.72565	-0.01272	0.533
919	202449_s_at	RXRA	0.38307	0.49282	0.39821	-0.01514	0.496
920	202450_s_at	CTSK	0.12429	0.29082	0.33901	-0.21472	0.853
921	202451_at	GTF2H1	0.49622	0.31067	0.41963	0.07659	0.26
922	202457_s_at	PPP3CA	0.31029	0.39464	0.31801	-0.00772	0.538
923	202459_s_at	LPIN2	0.66877	0.65105	0.64142	0.02735	0.401
924	202461_at	EIF2B2	0.10971	0.31327	0.34506	-0.23535	0.945
925	202462_s_at	DDX46	0.65017	0.69373	0.58082	0.06935	0.301
926	202466_at	POLS	0.50218	0.37946	0.24451	0.25767	0.056
927	202468_s_at	CTNNAL1	0.66811	0.71527	0.61442	0.05369	0.316
928	202470_s_at	CPSF6	0.42925	0.31891	0.36918	0.06007	0.326
929	202471_s_at	IDH3G	0.69287	0.65073	0.60168	0.09119	0.094
930	202472_at	MPI	0.61824	0.56172	0.38953	0.22871	0.015
931	202474_s_at	HCFC1	0.35638	0.34248	0.23482	0.12156	0.21
932	202475_at	TMEM147	0.86783	0.85781	0.82696	0.04087	0.192
933	202480_s_at	DEDD	0.43186	0.22394	0.2242	0.20766	0.077
934	202486_at	AFG3L2	0.2932	0.13861	0.20987	0.08333	0.282
935	202487_s_at	H2AFV	0.81965	0.82161	0.79979	0.01986	0.383
936	202488_s_at	FXYD3	0.72757	0.76964	0.68568	0.04189	0.338
937	202492_at	ATG9A	0.35156	0.39889	0.28224	0.06932	0.301
938	202495_at	TBCC	0.76684	0.62246	0.65681	0.11003	0.102
939	202496_at	EDC4	0.62406	0.57583	0.63999	-0.01593	0.558
940	202499_s_at	SLC2A3	0.55676	0.07311	0.2176	0.33916	<0.001
941	202502_at	ACADM	0.37258	0.6625	0.49773	-0.12515	0.75
942	202503_s_at	KIAA0101	0.72492	0.69292	0.75691	-0.03199	0.656
943	202505_at	SNRPB2	0.72326	0.78951	0.8229	-0.09964	0.881
944	202506_at	SSFA2	0.79537	0.80221	0.80983	-0.01446	0.622
945	202510_s_at	TNFAIP2	0.66833	0.66613	0.63226	0.03607	0.357
946	202513_s_at	PPP2R5D	0.12669	0.42702	0.24885	-0.12216	0.784
947	202514_at	DLG1	0.54788	0.50785	0.60396	-0.05608	0.704
948	202518_at	BCL7B	0.58562	0.61903	0.4762	0.10942	0.278
949	202520_s_at	MLH1	0.64587	0.74235	0.62311	0.02276	0.378
950	202521_at	CTCF	0.53117	0.6135	0.62275	-0.09158	0.734
951	202522_at	PITPNB	0.57994	0.63937	0.34184	0.2381	0.001

952	202527_s_at	SMAD4	0.85986	0.86697	0.88681	-0.02695	0.822
953	202528_at	GALE	0.39711	0.47923	0.45423	-0.05712	0.646
954	202529_at	PRPSAP1	0.54124	0.31482	0.48052	0.06072	0.289
955	202530_at	MAPK14	0.65869	0.56296	0.42861	0.23008	0.055
956	202531_at	IRF1	0.45382	0.61101	0.68252	-0.2287	0.989
957	202532_s_at	DHFR	0.63941	0.56751	0.60856	0.03085	0.382
958	202535_at	FADD	0.44634	0.079915	0.33494	0.1114	0.27
959	202538_s_at	CHMP2B	0.56084	0.45245	0.38165	0.17919	0.041
960	202540_s_at	HMGCR	0.56589	0.50633	0.56012	0.00577	0.519
961	202541_at	SCYE1	0.80684	0.6612	0.72019	0.08665	0.125
962	202543_s_at	GMFB	0.68502	0.73063	0.73949	-0.05447	0.766
963	202545_at	PRKCD	0.66633	0.58035	0.55535	0.11098	0.095
964	202546_at	VAMP8	0.69503	0.7183	0.75828	-0.06325	0.687
965	202548_s_at	ARHGEF7	0.81407	0.80421	0.7202	0.09387	0.134
966	202550_s_at	VAPB	0.14385	0.16661	0.16834	-0.02449	0.579
967	202552_s_at	CRIM1	0.35221	0.60561	0.54154	-0.18933	0.954
968	202553_s_at	SYF2	0.74119	0.67547	0.64531	0.09588	0.214
969	202556_s_at	MCRS1	0.21435	0.30325	0.37329	-0.15894	0.769
970	202557_at	HSPA13	0.56691	0.6995	0.73322	-0.16631	0.994
971	202567_at	SNRPD3	0.90975	0.88206	0.9049	0.00485	0.451
972	202569_s_at	MARK3	0.67215	0.53	0.72024	-0.04809	0.714
973	202573_at	CSNK1G2	0.29324	0.12	0.36324	-0.07	0.688
974	202578_s_at	DDX19A	0.73369	0.66581	0.6251	0.10859	0.023
975	202581_at	HSPA1A	0.5949	0.49354	0.54921	0.04569	0.344
976	202583_s_at	RANBP9	0.72599	0.67533	0.68989	0.0361	0.377
977	202584_at	NFX1	0.55828	0.57163	0.55539	0.00289	0.478
978	202592_at	BLOC1S1	0.73292	0.77226	0.83921	-0.10629	0.86
979	202593_s_at	GDE1	0.54947	0.43493	0.40138	0.14809	0.142
980	202594_at	LEPROTL1	0.72549	0.62738	0.71951	0.00598	0.466
981	202600_s_at	NRIP1	0.35072	0.47791	0.43745	-0.08673	0.746
982	202602_s_at	HTATSF1	0.78126	0.64263	0.7148	0.06646	0.179
983	202605_at	GUSB	0.32446	0.44592	0.4471	-0.12264	0.799
984	202609_at	EPS8	0.70735	0.78947	0.74805	-0.0407	0.714
985	202613_at	CTPS	0.70538	0.7206	0.73703	-0.03165	0.59
986	202614_at	SLC30A9	0.73241	0.67195	0.74016	-0.00775	0.544
987	202620_s_at	PLOD2	0.18778	0.38277	0.38684	-0.19906	0.908
988	202621_at	IRF3	0.74704	0.74328	0.68092	0.06612	0.111
989	202622_s_at	ATXN2	0.54506	0.39553	0.46952	0.07554	0.3
990	202623_at	EAPP	0.60744	0.61286	0.4674	0.14004	0.128
991	202625_at	LYN	0.60537	0.61681	0.54832	0.05705	0.244
992	202631_s_at	APPBP2	0.53115	0.42967	0.58241	-0.05126	0.764
993	202632_at	DPH1	0.62776	0.70428	0.61937	0.00839	0.482
994	202633_at	TOPBP1	0.83691	0.74491	0.72984	0.10707	0.004
995	202635_s_at	POLR2K	0.84565	0.83773	0.89039	-0.04474	0.737
996	202636_at	RNF103	0.52603	0.49499	0.41176	0.11427	0.193
997	202640_s_at	RANBP3	0.68388	0.59945	0.66105	0.02283	0.427
998	202643_s_at	TNFAIP3	0.72737	0.72262	0.77047	-0.0431	0.659
999	202645_s_at	MEN1	0.8065	0.74146	0.7021	0.1044	0.045
1000	202647_s_at	NRAS	0.69033	0.74604	0.77462	-0.08429	0.765
1001	202650_s_at	KIAA0195	0.57274	0.46205	0.47502	0.09772	0.27
1002	202651_at	LPGAT1	0.54816	0.55145	0.54149	0.00667	0.538
1003	202653_s_at	MARCH7	0.86379	0.67296	0.73287	0.13092	0.001
1004	202655_at	ARMET	0.53058	0.64364	0.62869	-0.09811	0.774

1005	202657_s_at	SERTAD2	0.35621	0.16168	0.22476	0.13145	0.12
1006	202658_at	PEX11B	0.63997	0.64137	0.62449	0.01548	0.442
1007	202659_at	PSMB10	0.80692	0.80047	0.87489	-0.06797	0.838
1008	202662_s_at	ITPR2	0.38953	0.47499	0.52924	-0.13971	0.882
1009	202665_s_at	WIPF1	0.23111	0.24538	0.30564	-0.07453	0.718
1010	202666_s_at	ACTL6A	0.66274	0.61065	0.62439	0.03835	0.386
1011	202667_s_at	SLC39A7	0.61418	0.41965	0.403	0.21118	0.028
1012	202670_at	MAP2K1	0.77279	0.67947	0.76345	0.00934	0.489
1013	202672_s_at	ATF3	0.72946	0.086371	0.30096	0.4285	<0.001
1014	202673_at	DPM1	0.74727	0.78878	0.78793	-0.04066	0.639
1015	202674_s_at	LMO7	0.30113	0.42247	0.26327	0.03786	0.391
1016	202677_at	RASA1	0.45802	0.5798	0.59706	-0.13904	0.923
1017	202678_at	GTF2A2	0.91928	0.9068	0.93598	-0.0167	0.703
1018	202680_at	GTF2E2	0.43079	0.48991	0.15091	0.27988	0.01
1019	202681_at	USP4	0.25093	0.3651	0.31984	-0.06891	0.721
1020	202683_s_at	RNMT	0.36689	0.49186	0.26609	0.1008	0.289
1021	202688_at	TNFSF10	0.54898	0.55284	0.56047	-0.01149	0.587
1022	202689_at	RBM15B	0.3228	0.22905	0.32952	-0.00672	0.509
1023	202691_at	SNRPD1	0.70834	0.62073	0.53864	0.1697	0.07
1024	202693_s_at	STK17A	0.67159	0.61378	0.51705	0.15454	0.06
1025	202696_at	OXSR1	0.88964	0.85356	0.87198	0.01766	0.408
1026	202702_at	TRIM26	0.60327	0.69694	0.59226	0.01101	0.453
1027	202703_at	DUSP11	0.54992	0.61563	0.57075	-0.02083	0.558
1028	202704_at	TOB1	0.24	0.55698	0.31208	-0.07208	0.819
1029	202705_at	CCNB2	0.75421	0.82387	0.52148	0.23273	0.031
1030	202708_s_at	HIST2H2BE	0.42051	0.49425	0.37028	0.05023	0.429
1031	202709_at	FMOD	0.28462	0.34281	0.3767	-0.09208	0.627
1032	202710_at	BET1	0.61726	0.52024	0.38642	0.23084	0.014
1033	202713_s_at	KIAA0391	0.25764	0.044183	0.34723	-0.08959	0.714
1034	202715_at	CAD	0.79722	0.81743	0.79201	0.00521	0.475
1035	202716_at	PTPN1	0.049484	0.22728	0.17112	-0.121636	0.776
1036	202720_at	TES	0.36739	0.59986	0.53126	-0.16387	0.844
1037	202721_s_at	GFPT1	0.56428	0.53223	0.60303	-0.03875	0.666
1038	202724_s_at	FOXO1	0.045745	0.4376	0.38619	-0.340445	0.997
1039	202726_at	LIG1	0.42416	0.20204	0.25505	0.16911	0.076
1040	202730_s_at	PDCD4	0.57617	0.72195	0.58135	-0.00518	0.553
1041	202732_at	PKIG	0.49132	0.5578	0.46174	0.02958	0.405
1042	202733_at	P4HA2	0.6448	0.54937	0.24031	0.40449	0.002
1043	202734_at	TRIP10	0.66783	0.7584	0.45914	0.20869	0.018
1044	202736_s_at	LSM4	0.6833	0.809	0.83067	-0.14737	0.956
1045	202738_s_at	PHKB	0.65543	0.60794	0.65706	-0.00163	0.524
1046	202740_at	ACY1	0.61523	0.76203	0.74142	-0.12619	0.906
1047	202742_s_at	PRKACB	0.40208	0.61114	0.60259	-0.20051	0.918
1048	202743_at	PIK3R3	0.38965	0.49688	0.49386	-0.10421	0.806
1049	202745_at	USP8	0.77321	0.76647	0.80253	-0.02932	0.64
1050	202746_at	ITM2A	0.16188	0.25044	0.32883	-0.16695	0.838
1051	202748_at	GBP2	0.27371	0.34303	0.42175	-0.14804	0.918
1052	202749_at	WRB	0.24758	0.40265	0.28856	-0.04098	0.633
1053	202750_s_at	TFIP11	0.50569	0.25591	0.29203	0.21366	0.043
1054	202753_at	PSMD6	0.7246	0.67234	0.77669	-0.05209	0.794
1055	202754_at	R3HDM1	0.79454	0.78022	0.76626	0.02828	0.352
1056	202757_at	COBRA1	0.61416	0.75849	0.76026	-0.1461	0.93
1057	202758_s_at	RFXANK	0.46623	0.51614	0.56426	-0.09803	0.723

1058	202759_s_at	AKAP2	0.23066	0.3601	0.42412	-0.19346	0.867
1059	202760_s_at	PALM2-AKAP2	0.12732	0.26683	0.29063	-0.16331	0.848
1060	202761_s_at	SYNE2	0.68866	0.74295	0.68747	0.00119	0.496
1061	202762_at	ROCK2	0.14605	0.40171	0.24629	-0.10024	0.73
1062	202763_at	CASP3	0.73935	0.31875	0.61549	0.12386	0.136
1063	202764_at	STIM1	0.55663	0.3428	0.30837	0.24826	0.026
1064	202767_at	ACP2	0.57061	0.55942	0.59073	-0.02012	0.569
1065	202769_at	CCNG2	0.66486	0.4598	0.55155	0.11331	0.166
1066	202771_at	FAM38A	0.54852	0.59475	0.53151	0.01701	0.456
1067	202772_at	HMGCL	0.81453	0.74376	0.61183	0.2027	0.005
1068	202775_s_at	SFRS8	0.57256	0.52464	0.50604	0.06652	0.352
1069	202776_at	DNTTIP2	0.7949	0.47488	0.59073	0.20417	0.036
1070	202777_at	SHOC2	0.63842	0.76401	0.72867	-0.09025	0.813
1071	202779_s_at	LOC731049	0.85161	0.84996	0.45762	0.39399	0.002
1072	202780_at	OXCT1	0.35823	0.34208	0.55456	-0.19633	0.896
1073	202781_s_at	INPP5K	0.60682	0.43676	0.49867	0.10815	0.222
1074	202783_at	NNT	0.55647	0.66281	0.7304	-0.17393	0.949
1075	202785_at	NDUFA7	0.86117	0.84222	0.85779	0.00338	0.49
1076	202786_at	STK39	0.14356	0.10826	0.33144	-0.18788	0.835
1077	202787_s_at	MAPKAPK3	0.40067	0.23896	0.41905	-0.01838	0.55
1078	202789_at	PLCG1	0.24893	0.262	0.12401	0.12492	0.196
1079	202793_at	LPCAT3	0.28547	0.49771	0.46226	-0.17679	0.903
1080	202794_at	INPP1	0.41958	0.52428	0.37188	0.0477	0.38
1081	202796_at	SYNPO	0.38716	0.50623	0.38786	-0.0007	0.502
1082	202797_at	SACM1L	0.63909	0.44626	0.47211	0.16698	0.158
1083	202798_at	SEC24B	0.65136	0.67236	0.65605	-0.00469	0.545
1084	202799_at	CLPP	0.88046	0.85502	0.8729	0.00756	0.46
1085	202803_s_at	ITGB2	0.60611	0.69183	0.5438	0.06231	0.304
1086	202804_at	ABCC1	0.77528	0.69597	0.64307	0.13221	0.136
1087	202808_at	C10orf26	0.61186	0.6173	0.53568	0.07618	0.276
1088	202809_s_at	INTS3	0.79896	0.88481	0.81284	-0.01388	0.614
1089	202810_at	DRG1	0.63146	0.67428	0.76561	-0.13415	0.901
1090	202811_at	STAMBP	0.60953	0.65072	0.74633	-0.1368	0.968
1091	202813_at	TARBP1	0.64606	0.62823	0.6021	0.04396	0.255
1092	202820_at	AHR	0.44537	0.71026	0.46394	-0.01857	0.568
1093	202824_s_at	TCEB1	0.92366	0.86696	0.91084	0.01282	0.369
1094	202825_at	SLC25A4	0.42773	0.57304	0.40563	0.0221	0.446
1095	202829_s_at	VAMP7	0.51274	0.69094	0.68543	-0.17269	0.91
1096	202832_at	GCC2	0.64807	0.58804	0.46281	0.18526	0.035
1097	202837_at	TRAFD1	0.42189	0.41899	0.44004	-0.01815	0.603
1098	202838_at	FUCA1	0.4657	0.58515	0.61562	-0.14992	0.906
1099	202839_s_at	NDUFB7	0.85307	0.85551	0.90072	-0.04765	0.793
1100	202840_at	TAF15	0.24386	0.41479	0.47238	-0.22852	0.929
1101	202843_at	DNAJB9	0.68833	0.59487	0.6311	0.05723	0.351
1102	202844_s_at	RALBP1	0.72809	0.73297	0.71055	0.01754	0.417
1103	202847_at	PCK2	0.46275	0.39526	0.30617	0.15658	0.128
1104	202850_at	ABCD3	0.70017	0.75982	0.69604	0.00413	0.497
1105	202853_s_at	RYK	0.3294	0.57272	0.45543	-0.12603	0.849
1106	202854_at	HPRT1	0.58412	0.54872	0.58899	-0.00487	0.503
1107	202856_s_at	SLC16A3	0.74472	0.72577	0.38647	0.35825	0.001
1108	202858_at	U2AF1	0.67958	0.64153	0.73992	-0.06034	0.802
1109	202860_at	DENND4B	0.473	0.59254	0.51463	-0.04163	0.658
1110	202862_at	FAH	0.24078	0.36834	0.39963	-0.15885	0.836

1111	202868_s_at	POP4	0.76769	0.80241	0.72378	0.04391	0.263
1112	202870_s_at	CDC20	0.81422	0.78609	0.39893	0.41529	0.002
1113	202871_at	TRAF4	0.29789	0.5151	0.31861	-0.02072	0.545
1114	202874_s_at	ATP6V1C1	0.32224	0.36236	0.53067	-0.20843	0.963
1115	202876_s_at	PBX2	0.081303	0.1875	0.16956	-0.088257	0.771
1116	202887_s_at	DDIT4	0.6139	0.23294	0.41282	0.20108	0.029
1117	202891_at	NIT1	0.5367	0.64188	0.54707	-0.01037	0.56
1118	202892_at	CDC23	0.51747	0.41593	0.49548	0.02199	0.445
1119	202899_s_at	SFRS3	0.71743	0.80005	0.69493	0.0225	0.422
1120	2028_s_at	E2F1	0.5159	0.58297	0.44929	0.06661	0.298
1121	202900_s_at	NUP88	0.60528	0.66438	0.62159	-0.01631	0.604
1122	202902_s_at	CTSS	0.43852	0.55233	0.54533	-0.10681	0.863
1123	202904_s_at	LSM5	0.88086	0.85925	0.88003	0.00083	0.489
1124	202908_at	WFS1	0.35598	0.38073	0.42462	-0.06864	0.71
1125	202909_at	EPM2AIP1	0.68037	0.75985	0.66972	0.01065	0.452
1126	202910_s_at	CD97	0.52859	0.42841	0.37584	0.15275	0.096
1127	202912_at	ADM	0.27348	0.27138	0.24211	0.03137	0.424
1128	202915_s_at	FAM20B	0.35868	0.46526	0.44524	-0.08656	0.755
1129	202919_at	MOBKL3	0.34854	0.18364	0.32081	0.02773	0.433
1130	202922_at	GCLC	0.42112	0.34568	0.34797	0.07315	0.367
1131	202925_s_at	PLAGL2	0.39219	0.49241	0.38849	0.0037	0.516
1132	202926_at	NBAS	0.17675	0.25186	0.45843	-0.28168	0.959
1133	202927_at	PIN1	0.53999	0.62853	0.71959	-0.1796	0.912
1134	202929_s_at	DDT	0.80937	0.84439	0.82127	-0.0119	0.542
1135	202930_s_at	SUCLA2	0.83242	0.76748	0.75763	0.07479	0.145
1136	202935_s_at	SOX9	0.73634	0.75171	0.75755	-0.02121	0.641
1137	202939_at	ZMPSTE24	0.77472	0.72166	0.45556	0.31916	0.022
1138	202941_at	NDUFV2	0.94445	0.90445	0.90235	0.0421	0.049
1139	202942_at	ETFB	0.74808	0.76054	0.84704	-0.09896	0.957
1140	202943_s_at	NAGA	0.61964	0.48531	0.59163	0.02801	0.425
1141	202946_s_at	BTBD3	0.14931	0.36374	0.28405	-0.13474	0.816
1142	202947_s_at	GYPC	0.53126	0.72651	0.65485	-0.12359	0.819
1143	202949_s_at	FHL2	0.5472	0.56391	0.57512	-0.02792	0.704
1144	202950_at	CRYZ	0.37206	0.45485	0.30091	0.07115	0.338
1145	202954_at	UBE2C	0.82111	0.87666	0.59557	0.22554	0.048
1146	202957_at	HCLS1	0.61342	0.6401	0.65589	-0.04247	0.67
1147	202958_at	PTPN9	0.30305	0.36527	0.4318	-0.12875	0.82
1148	202960_s_at	MUT	0.53116	0.51088	0.56788	-0.03672	0.599
1149	202961_s_at	ATP5J2	0.88355	0.83738	0.89514	-0.01159	0.594
1150	202963_at	RFX5	0.47593	0.50513	0.45728	0.01865	0.421
1151	202968_s_at	DYRK2	0.18828	0.11746	0.242	-0.05372	0.698
1152	202974_at	MPP1	0.20924	0.50029	0.40435	-0.19511	0.932
1153	202976_s_at	RHOBTB3	0.29457	0.4099	0.50806	-0.21349	0.934
1154	202980_s_at	SIAH1	0.44535	0.2991	0.51234	-0.06699	0.72
1155	202982_s_at	ACOT1	0.17704	0.32397	0.21547	-0.03843	0.59
1156	202983_at	HLTF	0.63267	0.62922	0.63275	-8E-05	0.521
1157	202984_s_at	BAG5	0.59792	0.62926	0.6547	-0.05678	0.664
1158	202990_at	PYGL	0.038165	0.16126	0.17269	-0.134525	0.856
1159	202991_at	STARD3	0.37699	0.41579	0.55734	-0.18035	0.913
1160	202996_at	POLD4	0.69541	0.7098	0.66663	0.02878	0.339
1161	203004_s_at	MEF2D	0.099908	0.050531	0.10537	-0.005462	0.51
1162	203006_at	INPP5A	0.28679	0.29005	0.37195	-0.08516	0.756
1163	203011_at	IMPA1	0.71755	0.72219	0.71838	-0.00083	0.524

1164	203013_at	ECD	0.67255	0.69121	0.72921	-0.05666	0.713
1165	203017_s_at	SSX2IP	0.61694	0.56382	0.48798	0.12896	0.074
1166	203022_at	RNASEH2A	0.77989	0.8278	0.82279	-0.0429	0.833
1167	203024_s_at	C5orf15	0.46827	0.44632	0.60982	-0.14155	0.87
1168	203025_at	ARD1A	0.8171	0.81431	0.82801	-0.01091	0.537
1169	203026_at	ZBTB5	0.71774	0.59809	0.67269	0.04505	0.32
1170	203027_s_at	MVD	0.39578	0.31053	0.21894	0.17684	0.067
1171	203028_s_at	CYBA	0.86269	0.85065	0.87519	-0.0125	0.601
1172	203031_s_at	UROS	0.219	0.36169	0.51782	-0.29882	0.933
1173	203035_s_at	PIAS3	0.20183	0.33437	0.2405	-0.03867	0.652
1174	203039_s_at	NDUFS1	0.63919	0.56095	0.55967	0.07952	0.192
1175	203040_s_at	HMBS	0.55302	0.71974	0.62828	-0.07526	0.696
1176	203041_s_at	LAMP2	0.51426	0.64416	0.5959	-0.08164	0.724
1177	203043_at	ZBED1	0.44102	0.695	0.55124	-0.11022	0.795
1178	203044_at	CHSY1	0.51779	0.59668	0.56522	-0.04743	0.636
1179	203045_at	NINJ1	0.44333	0.58182	0.63168	-0.18835	0.999
1180	203046_s_at	TIMELESS	0.53191	0.73789	0.67528	-0.14337	0.91
1181	203049_s_at	TTC37	0.82273	0.82317	0.78327	0.03946	0.231
1182	203051_at	BAHD1	0.3737	0.40202	0.20483	0.16887	0.143
1183	203053_at	BCAS2	0.69233	0.70529	0.63236	0.05997	0.247
1184	203054_s_at	TCTA	0.45523	0.59905	0.62805	-0.17282	0.856
1185	203058_s_at	PAPSS2	0.039256	0.27802	0.16489	-0.125634	0.748
1186	203062_s_at	MDC1	0.44347	0.6824	0.68977	-0.2463	0.962
1187	203064_s_at	FOXK2	0.42583	0.59171	0.59227	-0.16644	0.787
1188	203065_s_at	CAV1	0.15419	0.40337	0.31965	-0.16546	0.883
1189	203067_at	PDHX	0.57729	0.55797	0.75318	-0.17589	0.893
1190	203072_at	MYO1E	0.64239	0.5946	0.48582	0.15657	0.124
1191	203073_at	COG2	0.7234	0.59861	0.70656	0.01684	0.426
1192	203075_at	SMAD2	0.7449	0.80165	0.7553	-0.0104	0.576
1193	203079_s_at	CUL2	0.73117	0.75865	0.75529	-0.02412	0.629
1194	203080_s_at	BAZ2B	0.59967	0.6382	0.68294	-0.08327	0.716
1195	203082_at	BMS1	0.37256	0.56018	0.42005	-0.04749	0.604
1196	203085_s_at	TGFB1	0.77997	0.81139	0.77172	0.00825	0.445
1197	203089_s_at	HTRA2	0.50719	0.4048	0.49161	0.01558	0.49
1198	203090_at	SDF2	0.77591	0.71501	0.70783	0.06808	0.193
1199	203093_s_at	TIMM44	0.38517	0.36508	0.44815	-0.06298	0.642
1200	203094_at	MAD2L1BP	0.56903	0.42599	0.37113	0.1979	0.017
1201	203095_at	MTIF2	0.67637	0.62204	0.59806	0.07831	0.309
1202	203102_s_at	MGAT2	0.72269	0.73439	0.61061	0.11208	0.173
1203	203103_s_at	PRPF19	0.49691	0.49115	0.53203	-0.03512	0.613
1204	203105_s_at	DNM1L	0.59451	0.63771	0.62934	-0.03483	0.655
1205	203106_s_at	VPS41	0.40524	0.53006	0.39943	0.00581	0.519
1206	203109_at	UBE2M	0.64316	0.59682	0.65055	-0.00739	0.534
1207	203112_s_at	WHSC2	0.49966	0.423	0.58922	-0.08956	0.754
1208	203113_s_at	EEF1D	0.9187	0.87484	0.92773	-0.00903	0.612
1209	203114_at	SSSCA1	0.74739	0.71086	0.78698	-0.03959	0.722
1210	203115_at	FECH	0.2817	0.29341	0.34491	-0.06321	0.648
1211	203117_s_at	PAN2	0.56092	0.56266	0.53385	0.02707	0.425
1212	203119_at	CCDC86	0.44854	0.41103	0.46919	-0.02065	0.559
1213	203120_at	TP53BP2	0.24468	0.42721	0.28282	-0.03814	0.592
1214	203124_s_at	SLC11A2	0.26667	0.10094	0.13618	0.13049	0.262
1215	203127_s_at	SPTLC2	0.19475	0.29869	0.32261	-0.12786	0.808
1216	203133_at	SEC61B	0.81964	0.71293	0.84693	-0.02729	0.657

1217	203135_at	TBP	0.17916	0.32026	0.27498	-0.09582	0.75
1218	203136_at	RABAC1	0.57523	0.70844	0.74461	-0.16938	0.871
1219	203138_at	HAT1	0.75109	0.62268	0.57269	0.1784	0.026
1220	203139_at	DAPK1	0.25699	0.45851	0.50202	-0.24503	0.921
1221	203141_s_at	AP3B1	0.67433	0.72768	0.70251	-0.02818	0.658
1222	203145_at	SPAG5	0.82172	0.80139	0.78614	0.03558	0.367
1223	203147_s_at	TRIM14	0.17857	0.20748	0.31886	-0.14029	0.888
1224	203150_at	RABEPK	0.597	0.58048	0.61463	-0.01763	0.554
1225	203152_at	MRPL40	0.91722	0.91428	0.92217	-0.00495	0.616
1226	203153_at	IFIT1	0.67506	0.72043	0.62816	0.0469	0.203
1227	203156_at	AKAP11	0.63057	0.7061	0.75024	-0.11967	0.893
1228	203158_s_at	GLS	0.53263	0.65092	0.60027	-0.06764	0.761
1229	203163_at	KATNB1	0.28373	0.45332	0.33963	-0.0559	0.666
1230	203165_s_at	SLC33A1	0.73932	0.74456	0.7751	-0.03578	0.659
1231	203169_at	RGP1	0.13567	0.086974	0.19835	-0.06268	0.663
1232	203171_s_at	RRP8	0.28732	0.26727	0.4505	-0.16318	0.874
1233	203173_s_at	C16orf62	0.37358	0.46711	0.39374	-0.02016	0.562
1234	203175_at	RHOG	0.58954	0.67172	0.66383	-0.07429	0.783
1235	203176_s_at	TFAM	0.34057	0.62165	0.58095	-0.24038	0.953
1236	203178_at	GATM	0.38239	0.53061	0.53685	-0.15446	0.837
1237	203179_at	GALT	0.21209	0.23969	0.22786	-0.01577	0.537
1238	203183_s_at	SMARCD1	0.33782	0.25815	0.11661	0.22121	0.077
1239	203185_at	RASSF2	0.22156	0.50859	0.5678	-0.34624	0.977
1240	203186_s_at	S100A4	0.61054	0.55205	0.54322	0.06732	0.308
1241	203188_at	B3GNT1	0.12641	0.082893	0.022773	0.103637	0.272
1242	203189_s_at	NDUFS8	0.6994	0.82324	0.83149	-0.13209	0.88
1243	203192_at	ABCB6	0.44791	0.47322	0.43616	0.01175	0.475
1244	203196_at	ABCC4	0.34475	0.41572	0.3868	-0.04205	0.623
1245	203197_s_at	C1orf123	0.53351	0.50539	0.54867	-0.01516	0.536
1246	203198_at	CDK9	0.6261	0.66899	0.64303	-0.01693	0.586
1247	203200_s_at	MTRR	0.35616	0.28394	0.16667	0.18949	0.116
1248	203201_at	PMM2	0.54439	0.46196	0.52023	0.02416	0.419
1249	203202_at	KRR1	0.73409	0.79817	0.6473	0.08679	0.115
1250	203205_at	KDM4A	0.46401	0.43756	0.43566	0.02835	0.432
1251	203206_at	FAM53B	0.18232	0.1494	0.091434	0.090886	0.332
1252	203208_s_at	MTFR1	0.38317	0.32653	0.29969	0.08348	0.296
1253	203209_at	RFC5	0.57453	0.49133	0.50987	0.06466	0.318
1254	203212_s_at	MTMR2	0.40634	0.45394	0.51505	-0.10871	0.742
1255	203218_at	MAPK9	0.3117	0.25479	0.353	-0.0413	0.593
1256	203219_s_at	APRT	0.86836	0.82277	0.83294	0.03542	0.276
1257	203223_at	RABEP1	0.35362	0.43161	0.42102	-0.0674	0.745
1258	203225_s_at	RFK	0.14143	0.29335	0.25497	-0.11354	0.822
1259	203226_s_at	TSPAN31	0.64638	0.75269	0.73238	-0.086	0.766
1260	203228_at	PAFAH1B3	0.45956	0.23025	0.43974	0.01982	0.47
1261	203229_s_at	CLK2	0.71175	0.43567	0.63924	0.07251	0.231
1262	203230_at	DVL1	0.46378	0.61232	0.54937	-0.08559	0.708
1263	203235_at	THOP1	0.72588	0.78004	0.78598	-0.0601	0.689
1264	203236_s_at	LGALS9	0.54609	0.49775	0.51438	0.03171	0.463
1265	203241_at	UVRAG	0.80662	0.65858	0.63117	0.17545	0.033
1266	203244_at	PEX5	0.24211	0.22891	0.22836	0.01375	0.435
1267	203245_s_at	NCRNA00094	0.079557	0.32401	0.26633	-0.186773	0.909
1268	203246_s_at	TUSC4	0.36427	0.21458	0.24621	0.11806	0.24
1269	203247_s_at	ZNF24	0.46568	0.43414	0.4268	0.03888	0.406

1270	203250_at	RBM16	0.877	0.79577	0.87804	-0.00104	0.538
1271	203252_at	CDK2AP2	0.65226	0.72843	0.64173	0.01053	0.465
1272	203253_s_at	HISPPD1	0.8307	0.70644	0.79205	0.03865	0.272
1273	203254_s_at	TLN1	0.39819	0.4652	0.2355	0.16269	0.17
1274	203258_at	DRAP1	0.66352	0.5401	0.67161	-0.00809	0.515
1275	203260_at	HDDC2	0.0021126	0.12139	0.023771	-0.0216584	0.555
1276	203261_at	DCTN6	0.81675	0.65845	0.74313	0.07362	0.26
1277	203262_s_at	FAM50A	0.74018	0.77101	0.75242	-0.01224	0.585
1278	203263_s_at	ARHGEF9	0.41257	0.4798	0.38898	0.02359	0.434
1279	203266_s_at	MAP2K4	0.62194	0.65279	0.52948	0.09246	0.259
1280	203269_at	NSMAF	0.2728	0.40048	0.39142	-0.11862	0.784
1281	203270_at	DTYMK	0.70361	0.75399	0.70679	-0.00318	0.504
1282	203272_s_at	TUSC2	0.62222	0.69746	0.75272	-0.1305	0.944
1283	203274_at	F8A1	0.43872	0.58597	0.56433	-0.12561	0.852
1284	203275_at	IRF2	0.5466	0.56693	0.62278	-0.07618	0.765
1285	203276_at	LMNB1	0.49893	0.64158	0.55378	-0.05485	0.714
1286	203277_at	DFFA	0.59731	0.39591	0.45719	0.14012	0.146
1287	203278_s_at	PHF21A	0.6086	0.28388	0.39228	0.21632	0.035
1288	203279_at	EDEM1	0.54478	0.59284	0.52634	0.01844	0.489
1289	203282_at	GBE1	0.65955	0.77097	0.76033	-0.10078	0.797
1290	203286_at	RNF44	0.61609	0.50901	0.59259	0.0235	0.461
1291	203288_at	KIAA0355	0.18335	0.28412	0.23337	-0.05002	0.588
1292	203299_s_at	AP1S2	0.20455	0.35021	0.45614	-0.25159	0.972
1293	203301_s_at	DMTF1	0.40439	0.40433	0.41948	-0.01509	0.557
1294	203302_at	DCK	0.54235	0.46735	0.59457	-0.05222	0.712
1295	203303_at	DYNLT3	0.78239	0.6859	0.5262	0.25619	0.024
1296	203304_at	BAMBI	0.40613	0.40135	0.38893	0.0172	0.455
1297	203306_s_at	SLC35A1	0.48521	0.54399	0.531	-0.04579	0.74
1298	203307_at	GNL1	0.46239	0.50419	0.47628	-0.01389	0.512
1299	203309_s_at	HPS1	0.0035756	0.086309	0.11257	-0.1089944	0.739
1300	203310_at	STXBP3	0.82046	0.83064	0.8565	-0.03604	0.606
1301	203311_s_at	ARF6	0.59476	0.52316	0.66427	-0.06951	0.727
1302	203314_at	GTPBP6	0.34466	0.51019	0.57421	-0.22955	0.972
1303	203315_at	NCK2	0.38004	0.4089	0.38182	-0.00178	0.51
1304	203316_s_at	SNRPE	0.93402	0.91576	0.92188	0.01214	0.371
1305	203318_s_at	ZNF148	0.61175	0.47451	0.50154	0.11021	0.197
1306	203320_at	SH2B3	0.53542	0.37924	0.51901	0.01641	0.473
1307	203321_s_at	ADNP2	0.35818	0.31241	0.58778	-0.2296	0.943
1308	203330_s_at	STX5	0.52464	0.44116	0.34601	0.17863	0.152
1309	203333_at	KIFAP3	0.66553	0.67959	0.7115	-0.04597	0.696
1310	203335_at	PHYH	0.47827	0.47564	0.39109	0.08718	0.293
1311	203336_s_at	ITGB1BP1	0.31967	0.48175	0.55579	-0.23612	0.939
1312	203338_at	PPP2R5E	0.79992	0.73299	0.84518	-0.04526	0.797
1313	203339_at	SLC25A12	0.29251	0.30668	0.25094	0.04157	0.393
1314	203341_at	CEBPZ	0.78804	0.62538	0.69796	0.09008	0.149
1315	203342_at	TIMM17B	0.541	0.36155	0.56831	-0.02731	0.56
1316	203343_at	UGDH	0.43253	0.54417	0.45565	-0.02312	0.566
1317	203344_s_at	RBBP8	0.7025	0.66546	0.65262	0.04988	0.314
1318	203350_at	AP1G1	0.74769	0.81977	0.85987	-0.11218	0.952
1319	203351_s_at	ORC4L	0.70161	0.50397	0.63316	0.06845	0.258
1320	203356_at	CAPN7	0.73679	0.6725	0.79355	-0.05676	0.736
1321	203361_s_at	MYCBP	0.23783	0.23245	0.2802	-0.04237	0.626
1322	203362_s_at	MAD2L1	0.84321	0.79938	0.79774	0.04547	0.294

1323	203363_s_at	KIAA0652	0.42815	0.39945	0.3428	0.08535	0.289
1324	203366_at	POLG	0.42118	0.46623	0.56783	-0.14665	0.927
1325	203367_at	DUSP14	0.2648	0.32084	0.24636	0.01844	0.453
1326	203371_s_at	NDUFB3	0.90782	0.8712	0.89889	0.00893	0.363
1327	203373_at	SOCS2	0.29526	0.48371	0.45214	-0.15688	0.925
1328	203375_s_at	TPP2	0.8423	0.75116	0.72933	0.11297	0.006
1329	203377_s_at	CDC40	0.66365	0.67012	0.68658	-0.02293	0.634
1330	203378_at	PCF11	0.58167	0.5655	0.69646	-0.11479	0.868
1331	203379_at	RPS6KA1	0.80059	0.75183	0.70663	0.09396	0.126
1332	203384_s_at	GOLGA1	0.34369	0.50905	0.32671	0.01698	0.435
1333	203385_at	DGKA	0.60161	0.22378	0.52647	0.07514	0.323
1334	203387_s_at	TBC1D4	0.27775	0.46084	0.42873	-0.15098	0.87
1335	203391_at	FKBP2	0.40972	0.57452	0.53646	-0.12674	0.823
1336	203396_at	PSMA4	0.75916	0.87512	0.85143	-0.09227	0.923
1337	203401_at	PRPS2	0.53855	0.4146	0.43985	0.0987	0.272
1338	203404_at	ARMCX2	0.124	0.36287	0.32398	-0.19998	0.86
1339	203405_at	PSMG1	0.76519	0.75335	0.71371	0.05148	0.296
1340	203406_at	MFAP1	0.79418	0.77775	0.62446	0.16972	0.063
1341	203409_at	DDB2	0.59879	0.49312	0.21454	0.38425	<0.001
1342	203410_at	AP3M2	0.68362	0.57786	0.70214	-0.01852	0.567
1343	203411_s_at	LMNA	0.63907	0.77816	0.75827	-0.1192	0.91
1344	203412_at	LZTR1	0.34308	0.49415	0.43872	-0.09564	0.742
1345	203414_at	MMD	0.60067	0.53877	0.61978	-0.01911	0.602
1346	203415_at	PDCD6	0.64784	0.72709	0.67493	-0.02709	0.621
1347	203416_at	CD53	0.57085	0.72836	0.64639	-0.07554	0.721
1348	203420_at	FAM8A1	0.58958	0.65368	0.76112	-0.17154	0.95
1349	203422_at	POLD1	0.73516	0.68271	0.74677	-0.01161	0.505
1350	203427_at	ASF1A	0.65696	0.64537	0.68613	-0.02917	0.614
1351	203429_s_at	C1orf9	0.74346	0.80341	0.85011	-0.10665	0.916
1352	203430_at	HEBP2	0.49134	0.52913	0.513	-0.02166	0.548
1353	203433_at	MTHFS	0.53956	0.58238	0.61796	-0.0784	0.716
1354	203436_at	RPP30	0.70842	0.72462	0.76158	-0.05316	0.616
1355	203437_at	TMEM11	0.7447	0.71006	0.76633	-0.02163	0.615
1356	203445_s_at	CTDSP2	0.41637	0.57566	0.60371	-0.18734	0.904
1357	203449_s_at	TERF1	0.54061	0.45	0.42635	0.11426	0.169
1358	203454_s_at	ATOX1	0.69852	0.64194	0.75365	-0.05513	0.737
1359	203455_s_at	SAT1	0.58208	0.70465	0.69949	-0.11741	0.818
1360	203457_at	STX7	0.028895	0.2405	0.28947	-0.260575	0.954
1361	203458_at	SPR	0.51445	0.63472	0.51776	-0.00331	0.515
1362	203459_s_at	VPS16A	0.36987	0.54493	0.49869	-0.12882	0.806
1363	203460_s_at	PSEN1	0.6967	0.71581	0.73127	-0.03457	0.673
1364	203465_at	MRPL19	0.40703	0.48731	0.43596	-0.02893	0.58
1365	203466_at	MPV17	0.72342	0.62053	0.70506	0.01836	0.377
1366	203468_at	CDK10	0.15825	0.10761	0.083157	0.075093	0.389
1367	203471_s_at	PLEK	0.64547	0.65426	0.59202	0.05345	0.319
1368	203474_at	IQGAP2	0.099109	0.51117	0.38721	-0.288101	0.879
1369	203476_at	TPBG	0.20546	0.33954	0.30337	-0.09791	0.816
1370	203478_at	NDUFC1	0.79005	0.76268	0.83726	-0.04721	0.723
1371	203480_s_at	OTUD4	0.55143	0.59556	0.55856	-0.00713	0.545
1372	203482_at	FAM178A	0.25	0.43203	0.37422	-0.12422	0.826
1373	203484_at	SEC61G	0.85211	0.84281	0.86744	-0.01533	0.62
1374	203487_s_at	ARMC8	0.33801	0.22422	0.17886	0.15915	0.124
1375	203493_s_at	CEP57	0.88294	0.86791	0.88647	-0.00353	0.533

1376	203495_at	LRRC14	0.17332	0.19136	0.31559	-0.14227	0.853
1377	203500_at	GCDH	0.48052	0.55944	0.43651	0.04401	0.415
1378	203502_at	BPGM	0.43162	0.44423	0.47465	-0.04303	0.627
1379	203508_at	TNFRSF1B	0.22146	0.22808	0.36745	-0.14599	0.879
1380	203511_s_at	TRAPPC3	0.70537	0.70003	0.74677	-0.0414	0.603
1381	203513_at	SPG11	0.7747	0.84742	0.81749	-0.04279	0.813
1382	203514_at	MAP3K3	0.62939	0.72658	0.6415	-0.01211	0.512
1383	203515_s_at	PMVK	0.82684	0.81654	0.79056	0.03628	0.3
1384	203517_at	MTX2	0.77079	0.7355	0.62932	0.14147	0.108
1385	203519_s_at	UPF2	0.6085	0.63238	0.70875	-0.10025	0.763
1386	203521_s_at	ZNF318	0.48576	0.60866	0.37024	0.11552	0.196
1387	203522_at	CCS	0.25068	0.14124	0.25561	-0.00493	0.516
1388	203523_at	LSP1	0.51998	0.69419	0.72283	-0.20285	0.93
1389	203526_s_at	APC	0.62223	0.43371	0.43118	0.19105	0.045
1390	203528_at	SEMA4D	0.33352	0.46447	0.48798	-0.15446	0.865
1391	203529_at	PPP6C	0.4004	0.6111	0.4649	-0.0645	0.693
1392	203530_s_at	STX4	0.55123	0.5348	0.57531	-0.02408	0.605
1393	203531_at	CUL5	0.56915	0.64246	0.53513	0.03402	0.405
1394	203534_at	LSM1	0.64146	0.67116	0.6345	0.00696	0.481
1395	203537_at	PRPSAP2	0.21349	0.42739	0.25862	-0.04513	0.626
1396	203538_at	CAMLG	0.72533	0.71613	0.8676	-0.14227	0.883
1397	203544_s_at	STAM	0.68292	0.73059	0.7272	-0.04428	0.611
1398	203545_at	ALG8	0.57513	0.63897	0.46723	0.1079	0.198
1399	203546_at	IPO13	0.62452	0.53923	0.56694	0.05758	0.29
1400	203550_s_at	C1orf2	0.39007	0.56567	0.40395	-0.01388	0.543
1401	203552_at	MAP4K5	0.50161	0.73358	0.66916	-0.16755	0.92
1402	203556_at	ZHX2	0.33819	0.40163	0.40029	-0.0621	0.694
1403	203557_s_at	PCBD1	0.76351	0.71886	0.62717	0.13634	0.002
1404	203560_at	GGH	0.58819	0.74025	0.77485	-0.18666	0.945
1405	203562_at	FEZ1	0.40598	0.26122	0.31293	0.09305	0.244
1406	203564_at	FANCG	0.67726	0.74112	0.72353	-0.04627	0.802
1407	203565_s_at	MNAT1	0.19139	0.16624	0.18766	0.00373	0.49
1408	203566_s_at	AGL	0.81887	0.8363	0.78962	0.02925	0.299
1409	203567_s_at	TRIM38	0.20145	0.55046	0.46403	-0.26258	0.922
1410	203569_s_at	OFD1	0.53405	0.54075	0.4528	0.08125	0.338
1411	203572_s_at	TAF6	0.15512	0.17213	0.33327	-0.17815	0.939
1412	203573_s_at	RABGGTA	0.63842	0.63197	0.63071	0.00771	0.499
1413	203574_at	NFIL3	0.25733	0.23183	0.11657	0.14076	0.125
1414	203575_at	CSNK2A2	0.42035	0.41991	0.41599	0.00436	0.497
1415	203577_at	GTF2H4	0.16917	0.15054	0.25403	-0.08486	0.712
1416	203579_s_at	SLC7A6	0.721	0.66621	0.47804	0.24296	0.012
1417	203581_at	RAB4A	0.55386	0.55036	0.59581	-0.04195	0.685
1418	203583_at	UNC50	0.88022	0.72785	0.82729	0.05293	0.203
1419	203584_at	TTC35	0.67716	0.60189	0.61639	0.06077	0.299
1420	203588_s_at	TFDP2	0.45769	0.48583	0.47641	-0.01872	0.53
1421	203592_s_at	FSTL3	0.36854	0.54747	0.50992	-0.14138	0.876
1422	203593_at	CD2AP	0.87331	0.8396	0.88849	-0.01518	0.712
1423	203599_s_at	WBP4	0.65682	0.6099	0.50667	0.15015	0.088
1424	203600_s_at	C4orf8	0.15174	0.4648	0.44137	-0.28963	0.994
1425	203604_at	ZNF516	0.19567	0.22347	0.34241	-0.14674	0.834
1426	203605_at	SRP54	0.33024	0.38249	0.38252	-0.05228	0.656
1427	203606_at	NDUFS6	0.94128	0.87814	0.90436	0.03692	0.135
1428	203607_at	INPP5F	0.63728	0.69794	0.69124	-0.05396	0.657

1429	203608_at	ALDH5A1	0.22684	0.45256	0.38953	-0.16269	0.875
1430	203611_at	TERF2	0.48454	0.58211	0.69448	-0.20994	0.982
1431	203612_at	BYSL	0.50371	0.666	0.5922	-0.08849	0.822
1432	203613_s_at	NDUFB6	0.81674	0.86788	0.89528	-0.07854	0.863
1433	203614_at	UTP14C	0.46512	0.079767	0.22461	0.24051	0.015
1434	203616_at	POLB	0.66464	0.80473	0.74471	-0.08007	0.857
1435	203620_s_at	FCHSD2	0.76804	0.8201	0.79625	-0.02821	0.693
1436	203621_at	NDUFB5	0.81437	0.88464	0.78939	0.02498	0.34
1437	203622_s_at	PNO1	0.69979	0.57487	0.58098	0.11881	0.144
1438	203630_s_at	COG5	0.47169	0.38332	0.55537	-0.08368	0.807
1439	203635_at	DSCR3	0.6467	0.65522	0.50764	0.13906	0.092
1440	203643_at	ERF	0.55206	0.25284	0.53495	0.01711	0.438
1441	203644_s_at	MON1B	0.27389	0.32125	0.38429	-0.1104	0.79
1442	203647_s_at	FDX1	0.66978	0.54563	0.49257	0.17721	0.043
1443	203648_at	TATDN2	0.62958	0.4226	0.41185	0.21773	0.061
1444	203650_at	PROCR	0.068734	0.2464	0.15898	-0.090246	0.766
1445	203651_at	ZFYVE16	0.40636	0.36822	0.4927	-0.08634	0.753
1446	203652_at	MAP3K11	0.65876	0.61778	0.52121	0.13755	0.093
1447	203653_s_at	COIL	0.67738	0.51557	0.68683	-0.00945	0.547
1448	203655_at	XRCC1	0.10829	0.25403	0.31645	-0.20816	0.894
1449	203656_at	FIG4	0.54719	0.57148	0.64116	-0.09397	0.841
1450	203658_at	SLC25A20	0.49664	0.4239	0.43755	0.05909	0.382
1451	203659_s_at	TRIM13	0.49982	0.25633	0.40699	0.09283	0.311
1452	203660_s_at	PCNT	0.57748	0.53919	0.64296	-0.06548	0.783
1453	203662_s_at	TMOD1	0.34247	0.47607	0.36986	-0.02739	0.601
1454	203665_at	HMOX1	0.41871	0.36088	0.44373	-0.02502	0.589
1455	203668_at	MAN2C1	0.57881	0.53325	0.52979	0.04902	0.356
1456	203674_at	HELZ	0.62728	0.63146	0.56261	0.06467	0.288
1457	203675_at	NUCB2	0.73825	0.75722	0.67752	0.06073	0.262
1458	203677_s_at	TARBP2	0.54972	0.41971	0.6245	-0.07478	0.78
1459	203678_at	MTMR15	0.63424	0.65688	0.5702	0.06404	0.401
1460	203679_at	TMED1	0.36227	0.50608	0.54402	-0.18175	0.929
1461	203686_at	MPG	0.5732	0.65713	0.70193	-0.12873	0.833
1462	203688_at	PKD2	0.50465	0.45851	0.52771	-0.02306	0.621
1463	203689_s_at	FMR1	0.73678	0.71232	0.78579	-0.04901	0.72
1464	203693_s_at	E2F3	0.52317	0.46373	0.49155	0.03162	0.396
1465	203694_s_at	DHX16	0.68623	0.65605	0.71127	-0.02504	0.625
1466	203695_s_at	DFNA5	0.40186	0.5839	0.59949	-0.19763	0.97
1467	203701_s_at	TRMT1	0.46157	0.45471	0.49123	-0.02966	0.604
1468	203707_at	ZNF263	0.70722	0.65812	0.63103	0.07619	0.288
1469	203711_s_at	HIBCH	0.15803	0.31505	0.22596	-0.06793	0.653
1470	203712_at	KIAA0020	0.48583	0.52778	0.40785	0.07798	0.307
1471	203715_at	TBCE	0.21641	0.24657	0.16706	0.04935	0.414
1472	203718_at	PNPLA6	0.64816	0.63924	0.55041	0.09775	0.21
1473	203720_s_at	ERCC1	0.55925	0.55694	0.51298	0.04627	0.355
1474	203725_at	GADD45A	0.8079	0.42897	0.26472	0.54318	<0.001
1475	203728_at	BAK1	0.43322	0.35477	0.32374	0.10948	0.127
1476	203729_at	EMP3	0.59089	0.52587	0.71858	-0.12769	0.839
1477	203731_s_at	ZKSCAN5	0.097573	0.22888	0.21635	-0.118777	0.784
1478	203732_at	TRIP4	0.32892	0.45381	0.27391	0.05501	0.348
1479	203733_at	DEXI	0.57991	0.32214	0.29028	0.28963	<0.001
1480	203734_at	FOXJ2	0.22431	0.24434	0.20105	0.02326	0.438
1481	203737_s_at	PPRC1	0.33824	0.4867	0.417	-0.07876	0.715

1482	203738_at	C5orf22	0.74818	0.59513	0.82804	-0.07986	0.847
1483	203739_at	ZNF217	0.65274	0.50733	0.52526	0.12748	0.175
1484	203740_at	MPHOSPH6	0.24619	0.3943	0.50415	-0.25796	0.96
1485	203741_s_at	ADCY7	0.59235	0.54282	0.60277	-0.01042	0.555
1486	203743_s_at	TDG	0.76459	0.68904	0.66118	0.10341	0.153
1487	203744_at	HMGB3	0.36203	0.39793	0.36422	-0.00219	0.483
1488	203745_at	HCCS	0.53497	0.61716	0.50497	0.03	0.377
1489	203752_s_at	JUND	0.5805	0.57198	0.6641	-0.0836	0.767
1490	203755_at	BUB1B	0.86351	0.73132	0.80339	0.06012	0.157
1491	203758_at	CTSO	0.62678	0.62255	0.34062	0.28616	0.001
1492	203762_s_at	DYNC2LI1	0.3483	0.38393	0.42998	-0.08168	0.739
1493	203764_at	DLGAP5	0.7999	0.80276	0.57775	0.22215	0.02
1494	203765_at	GCA	0.76375	0.71015	0.66954	0.09421	0.125
1495	203774_at	MTR	0.63519	0.74259	0.74931	-0.11412	0.85
1496	203775_at	SLC25A13	0.57853	0.68848	0.64733	-0.0688	0.728
1497	203776_at	GPKOW	0.6912	0.62419	0.55177	0.13943	0.006
1498	203777_s_at	RPS6KB2	0.04457	0.21012	0.083226	-0.038656	0.596
1499	203778_at	MANBA	0.3951	0.41284	0.30563	0.08947	0.322
1500	203781_at	MRPL33	0.81946	0.81261	0.8504	-0.03094	0.684
1501	203787_at	SSBP2	0.5961	0.64521	0.57857	0.01753	0.451
1502	203790_s_at	HRSP12	0.54029	0.54194	0.56277	-0.02248	0.569
1503	203791_at	DMXL1	0.61652	0.61906	0.67691	-0.06039	0.759
1504	203799_at	CD302	0.15841	0.43195	0.36238	-0.20397	0.929
1505	203800_s_at	MRPS14	0.86037	0.80852	0.76559	0.09478	0.035
1506	203803_at	PCYOX1	0.488	0.54899	0.62137	-0.13337	0.901
1507	203804_s_at	CROP	0.8019	0.71101	0.78342	0.01848	0.461
1508	203805_s_at	FANCA	0.47854	0.61378	0.61794	-0.1394	0.881
1509	203810_at	DNAJB4	0.40209	0.38966	0.34998	0.05211	0.372
1510	203814_s_at	NQO2	0.24131	0.40248	0.4439	-0.20259	0.959
1511	203816_at	DGUOK	0.80964	0.63938	0.77505	0.03459	0.366
1512	203817_at	GUCY1B3	0.33817	0.51969	0.49758	-0.15941	0.9
1513	203818_s_at	SF3A3	0.65997	0.62985	0.61319	0.04678	0.352
1514	203820_s_at	IGF2BP3	0.19473	0.44353	0.35571	-0.16098	0.93
1515	203822_s_at	ELF2	0.31055	0.06716	0.39654	-0.08599	0.693
1516	203825_at	BRD3	0.15635	0.24212	0.33434	-0.17799	0.92
1517	203827_at	WIPI1	0.4149	0.54889	0.35965	0.05525	0.359
1518	203829_at	ELP4	0.18777	0.26425	0.16098	0.02679	0.475
1519	203830_at	C17orf75	0.48345	0.32625	0.1984	0.28505	0.032
1520	203831_at	R3HDM2	0.020909	0.042852	0.17751	-0.156601	0.904
1521	203832_at	SNRPF	0.89673	0.86553	0.89437	0.00236	0.486
1522	203836_s_at	MAP3K5	0.68758	0.56909	0.64697	0.04061	0.353
1523	203840_at	BLZF1	0.67955	0.63079	0.53455	0.145	0.076
1524	203843_at	RPS6KA3	0.46171	0.48422	0.53976	-0.07805	0.746
1525	203845_at	KAT2B	0.43226	0.54502	0.49077	-0.05851	0.661
1526	203846_at	TRIM32	0.61343	0.51958	0.73463	-0.1212	0.884
1527	203852_s_at	SMN1	0.49895	0.46296	0.49581	0.00314	0.498
1528	203855_at	WDR47	0.67647	0.71632	0.67565	0.00082	0.524
1529	203856_at	VRK1	0.76916	0.84598	0.79923	-0.03007	0.679
1530	203857_s_at	PDIA5	0.6347	0.51235	0.6713	-0.0366	0.689
1531	203858_s_at	COX10	0.5478	0.42281	0.42081	0.12699	0.175
1532	203860_at	PCCA	0.39147	0.28235	0.47374	-0.08227	0.78
1533	203868_s_at	VCAM1	0.31787	0.4715	0.55201	-0.23414	0.965
1534	203869_at	USP46	0.61235	0.73643	0.58657	0.02578	0.442

1535	203871_at	SENP3	0.41676	0.37632	0.40164	0.01512	0.48
1536	203879_at	PIK3CD	0.58272	0.72955	0.61565	-0.03293	0.649
1537	203880_at	COX17	0.77876	0.74942	0.82161	-0.04285	0.667
1538	203881_s_at	DMD	0.66738	0.71604	0.68469	-0.01731	0.577
1539	203883_s_at	RAB11FIP2	0.39275	0.55394	0.56336	-0.17061	0.904
1540	203885_at	RAB21	0.64736	0.7123	0.6585	-0.01114	0.571
1541	203890_s_at	DAPK3	0.52354	0.32612	0.2275	0.29604	0.125
1542	203892_at	WFDC2	0.27001	0.48622	0.36393	-0.09392	0.792
1543	203894_at	TUBG2	0.21625	0.25525	0.1641	0.05215	0.387
1544	203897_at	LYRM1	0.60488	0.58723	0.55731	0.04757	0.349
1545	203899_s_at	CRCP	0.38134	0.3794	0.18371	0.19763	0.085
1546	203900_at	KIAA0467	0.18729	0.30436	0.18957	-0.00228	0.502
1547	203903_s_at	HEPH	0.3005	0.4981	0.38829	-0.08779	0.703
1548	203905_at	PARN	0.88553	0.6758	0.54951	0.33602	<0.001
1549	203909_at	SLC9A6	0.54126	0.52238	0.61888	-0.07762	0.697
1550	203910_at	ARHGAP29	0.16949	0.18888	0.10993	0.05956	0.332
1551	203916_at	NDST2	0.62627	0.73644	0.5244	0.10187	0.215
1552	203921_at	CHST2	0.66608	0.68922	0.62091	0.04517	0.257
1553	203923_s_at	CYBB	0.36167	0.70501	0.5529	-0.19123	0.981
1554	203925_at	GCLM	0.61925	0.65716	0.71084	-0.09159	0.802
1555	203927_at	NFKBIE	0.35463	0.55245	0.54573	-0.1911	0.934
1556	203931_s_at	MRPL12	0.79737	0.73885	0.78919	0.00818	0.446
1557	203932_at	HLA-DMB	0.41266	0.6691	0.55934	-0.14668	0.918
1558	203933_at	RAB11FIP3	0.40113	0.50374	0.54109	-0.13996	0.822
1559	203935_at	ACVR1	0.19347	0.19117	0.1038	0.08967	0.235
1560	203936_s_at	MMP9	0.44322	0.34086	0.46455	-0.02133	0.54
1561	203939_at	NT5E	0.24514	0.31545	0.37764	-0.1325	0.782
1562	203941_at	INTS9	0.6036	0.52621	0.59217	0.01143	0.444
1563	203943_at	KIF3B	0.81396	0.7857	0.78265	0.03131	0.331
1564	203945_at	ARG2	0.61568	0.55864	0.45058	0.1651	0.08
1565	203947_at	CSTF3	0.64086	0.49532	0.24888	0.39198	0.002
1566	203957_at	E2F6	0.33391	0.22015	0.23246	0.10145	0.227
1567	203958_s_at	ZBTB40	0.44759	0.28626	0.37271	0.07488	0.283
1568	203960_s_at	HSPB11	0.8608	0.82893	0.81726	0.04354	0.258
1569	203964_at	NMI	0.45362	0.60362	0.55743	-0.10381	0.821
1570	203966_s_at	PPM1A	0.15564	0.36983	0.17707	-0.02143	0.545
1571	203967_at	CDC6	0.64629	0.68476	0.64383	0.00246	0.518
1572	203970_s_at	PEX3	0.66713	0.6601	0.51612	0.15101	0.056
1573	203971_at	SLC31A1	0.35548	0.43748	0.20711	0.14837	0.179
1574	203973_s_at	CEBPD	0.32556	0.22617	0.31033	0.01523	0.489
1575	203974_at	HDHD1A	0.027908	0.24574	0.18583	-0.157922	0.898
1576	203975_s_at	CHAF1A	0.5667	0.29024	0.55502	0.01168	0.475
1577	203978_at	NUBP1	0.6641	0.71121	0.67416	-0.01006	0.556
1578	203983_at	TSNAX	0.75227	0.60019	0.62064	0.13163	0.104
1579	203984_s_at	CASP9	0.32602	0.19973	0.31978	0.00624	0.485
1580	203985_at	ZNF212	0.21977	0.22271	0.32883	-0.10906	0.771
1581	203987_at	FZD6	0.32198	0.48005	0.47831	-0.15633	0.87
1582	203988_s_at	FUT8	0.44168	0.49681	0.46028	-0.0186	0.609
1583	203990_s_at	KDM6A	0.33305	0.49862	0.43906	-0.10601	0.763
1584	204001_at	SNAPC3	0.47944	0.58717	0.64302	-0.16358	0.898
1585	204003_s_at	NUPL2	0.50552	0.36047	0.3692	0.13632	0.078
1586	204004_at	PAWR	0.52621	0.51714	0.53813	-0.01192	0.536
1587	204015_s_at	DUSP4	0.56032	0.58668	0.59381	-0.03349	0.599

1588	204016_at	LARS2	0.4298	0.41514	0.31845	0.11135	0.179
1589	204019_s_at	SH3YL1	0.30127	0.50567	0.43404	-0.13277	0.83
1590	204021_s_at	PURA	0.67539	0.30915	0.5497	0.12569	0.071
1591	204023_at	RFC4	0.72089	0.83136	0.73673	-0.01584	0.572
1592	204025_s_at	PDCD2	0.68259	0.58951	0.44293	0.23966	0.018
1593	204026_s_at	ZWINT	0.62989	0.7239	0.64485	-0.01496	0.549
1594	204027_s_at	METTL1	0.59582	0.62934	0.64311	-0.04729	0.695
1595	204028_s_at	RABGAP1	0.34523	0.41214	0.23925	0.10598	0.248
1596	204030_s_at	SCHIP1	0.25007	0.4583	0.51286	-0.26279	0.976
1597	204032_at	BCAR3	0.39394	0.51705	0.52625	-0.13231	0.853
1598	204033_at	TRIP13	0.72204	0.72827	0.69928	0.02276	0.411
1599	204034_at	ETHE1	0.74247	0.79245	0.75099	-0.00852	0.576
1600	204038_s_at	LPAR1	0.26711	0.39571	0.20171	0.0654	0.358
1601	204044_at	QPRT	0.33398	0.56784	0.44488	-0.1109	0.773
1602	204045_at	TCEAL1	0.56595	0.51986	0.45814	0.10781	0.071
1603	204054_at	PTEN	0.36489	0.48658	0.59046	-0.22557	0.921
1604	204057_at	IRF8	0.45762	0.63116	0.55546	-0.09784	0.809
1605	204059_s_at	ME1	0.02878	0.36456	0.24575	-0.21697	0.852
1606	204061_at	PRKX	0.44012	0.49122	0.45809	-0.01797	0.547
1607	204064_at	THOC1	0.48848	0.63353	0.61613	-0.12765	0.744
1608	204065_at	CHST10	0.5283	0.6722	0.54391	-0.01561	0.522
1609	204067_at	SUOX	0.14451	0.18415	0.24554	-0.10103	0.691
1610	204068_at	STK3	0.71632	0.59683	0.63618	0.08014	0.203
1611	204070_at	RARRES3	0.47597	0.52935	0.53869	-0.06272	0.727
1612	204071_s_at	TOPORS	0.76024	0.67532	0.71377	0.04647	0.209
1613	204076_at	ENTPD4	0.6194	0.55919	0.55677	0.06263	0.273
1614	204079_at	TPST2	0.3048	0.39036	0.47369	-0.16889	0.879
1615	204080_at	TOE1	0.53837	0.69231	0.60339	-0.06502	0.702
1616	204082_at	PBX3	0.63261	0.78148	0.74642	-0.11381	0.925
1617	204085_s_at	CLN5	0.63055	0.51788	0.2992	0.33135	0.013
1618	204088_at	P2RX4	0.36846	0.41246	0.34228	0.02618	0.398
1619	204091_at	PDE6D	0.73006	0.75356	0.67606	0.054	0.248
1620	204093_at	CCNH	0.68754	0.6958	0.5699	0.11764	0.217
1621	204094_s_at	TSC22D2	0.64817	0.67577	0.64399	0.00418	0.486
1622	204098_at	RBMX2	0.2168	0.35213	0.29192	-0.07512	0.739
1623	204102_s_at	EEF2	0.91734	0.88573	0.90376	0.01358	0.312
1624	204103_at	CCL4	0.42684	0.36296	0.49022	-0.06338	0.683
1625	204106_at	TESK1	0.50378	0.47946	0.39287	0.11091	0.217
1626	204108_at	NFYA	0.49308	0.45595	0.51385	-0.02077	0.539
1627	204115_at	GNG11	0.08341	0.30597	0.20725	-0.12384	0.848
1628	204116_at	IL2RG	0.8049	0.8051	0.82498	-0.02008	0.601
1629	204118_at	CD48	0.61086	0.81214	0.68617	-0.07531	0.744
1630	204120_s_at	ADK	0.70211	0.8025	0.85058	-0.14847	0.97
1631	204125_at	NDUFAF1	0.44035	0.38184	0.44728	-0.00693	0.52
1632	204126_s_at	CDC45L	0.67756	0.63905	0.61832	0.05924	0.282
1633	204128_s_at	RFC3	0.68633	0.72973	0.76677	-0.08044	0.838
1634	204131_s_at	FOXO3	0.084162	0.30032	0.30111	-0.216948	0.906
1635	204133_at	RRP9	0.59361	0.56006	0.63478	-0.04117	0.619
1636	204135_at	FILIP1L	0.50441	0.63877	0.56412	-0.05971	0.664
1637	204137_at	GPR137B	0.55725	0.55306	0.44958	0.10767	0.237
1638	204141_at	TUBB2A	0.16744	0.35423	0.40484	-0.2374	0.892
1639	204142_at	ENOSF1	0.17344	0.16742	0.11147	0.06197	0.384
1640	204144_s_at	PIGQ	0.21836	0.33158	0.1021	0.11626	0.275

1641	204145_at	FRG1	0.71203	0.78656	0.77705	-0.06502	0.798
1642	204146_at	RAD51AP1	0.59709	0.71318	0.66881	-0.07172	0.744
1643	204153_s_at	MFNG	0.433	0.4075	0.3181	0.1149	0.314
1644	204156_at	KIAA0999	0.44483	0.40228	0.49124	-0.04641	0.61
1645	204158_s_at	TCIRG1	0.54442	0.58921	0.45223	0.09219	0.155
1646	204160_s_at	ENPP4	0.57383	0.71303	0.60204	-0.02821	0.635
1647	204162_at	NDC80	0.70268	0.64029	0.59992	0.10276	0.119
1648	204164_at	SIPA1	0.36698	0.52962	0.2603	0.10668	0.179
1649	204165_at	WASF1	0.46114	0.60264	0.44803	0.01311	0.479
1650	204168_at	MGST2	0.31022	0.46016	0.44574	-0.13552	0.812
1651	204170_s_at	CKS2	0.74809	0.72573	0.58851	0.15958	0.145
1652	204171_at	RPS6KB1	0.59367	0.5655	0.54055	0.05312	0.3
1653	204172_at	CPOX	0.39673	0.44023	0.43781	-0.04108	0.631
1654	204173_at	MYL6B	0.69282	0.77075	0.79404	-0.10122	0.863
1655	204174_at	ALOX5AP	0.27714	0.46818	0.52682	-0.24968	0.951
1656	204175_at	ZNF593	0.80288	0.79721	0.83629	-0.03341	0.635
1657	204181_s_at	ZBTB43	0.29544	0.18708	0.28712	0.00832	0.468
1658	204186_s_at	PPID	0.79361	0.75968	0.7142	0.07941	0.172
1659	204190_at	USPL1	0.37773	0.2399	0.22517	0.15256	0.152
1660	204192_at	CD37	0.63405	0.51373	0.57836	0.05569	0.273
1661	204198_s_at	RUNX3	0.8167	0.90281	0.86041	-0.04371	0.811
1662	204201_s_at	PTPN13	0.17458	0.3567	0.34291	-0.16833	0.885
1663	204203_at	CEBPG	0.63725	0.58656	0.59838	0.03887	0.307
1664	204204_at	SLC31A2	0.11745	0.15995	0.14262	-0.02517	0.572
1665	204205_at	APOBEC3G	0.33634	0.52006	0.41673	-0.08039	0.691
1666	204206_at	MNT	0.257	0.21094	0.23457	0.02243	0.46
1667	204208_at	RNGTT	0.18864	0.42864	0.4898	-0.30116	0.974
1668	204209_at	PCYT1A	0.55326	0.37099	0.56399	-0.01073	0.582
1669	204212_at	ACOT8	0.64036	0.54585	0.44272	0.19764	0.068
1670	204215_at	C7orf23	0.42529	0.62459	0.5498	-0.12451	0.736
1671	204216_s_at	ZC3H14	0.53496	0.52889	0.52628	0.00868	0.466
1672	204218_at	C11orf51	0.70225	0.65536	0.78591	-0.08366	0.778
1673	204219_s_at	PSMC1	0.77114	0.74775	0.80505	-0.03391	0.623
1674	204220_at	GMFG	0.72616	0.83428	0.80032	-0.07416	0.81
1675	204222_s_at	GLIPR1	0.26165	0.31758	0.43462	-0.17297	0.86
1676	204224_s_at	GCH1	0.4824	0.71648	0.49136	-0.00896	0.52
1677	204226_at	STAU2	0.79267	0.75966	0.65711	0.13556	0.04
1678	204228_at	PPIH	0.80259	0.78272	0.83705	-0.03446	0.699
1679	204233_s_at	CHKA	0.27412	0.51709	0.27896	-0.00484	0.506
1680	204234_s_at	ZNF195	0.49797	0.32604	0.37229	0.12568	0.141
1681	204241_at	ACOX3	0.37735	0.35305	0.30991	0.06744	0.335
1682	204243_at	RLF	0.50775	0.023037	0.28957	0.21818	0.03
1683	204244_s_at	DBF4	0.81647	0.72432	0.80676	0.00971	0.48
1684	204245_s_at	RPP14	0.2078	0.22583	0.1865	0.0213	0.456
1685	204246_s_at	DCTN3	0.88036	0.89299	0.88023	0.00013	0.533
1686	204247_s_at	CDK5	0.49981	0.57914	0.66015	-0.16034	0.923
1687	204249_s_at	LMO2	0.59227	0.65135	0.72091	-0.12864	0.907
1688	204258_at	CHD1	0.75369	0.82129	0.83335	-0.07966	0.824
1689	204263_s_at	CPT2	0.63794	0.44191	0.68254	-0.0446	0.652
1690	204265_s_at	GPSM3	0.60929	0.6743	0.56955	0.03974	0.299
1691	204269_at	PIM2	0.42143	0.57586	0.46787	-0.04644	0.613
1692	204275_at	SOLH	0.48626	0.35456	0.29719	0.18907	0.106
1693	204276_at	TK2	0.19801	0.33075	0.35698	-0.15897	0.896

1694	204278_s_at	EBAG9	0.5308	0.62522	0.49856	0.03224	0.379
1695	204279_at	PSMB9	0.75808	0.6123	0.64603	0.11205	0.022
1696	204286_s_at	PMAIP1	0.60629	0.45422	0.60244	0.00385	0.501
1697	204291_at	ZNF518A	0.73723	0.6529	0.65208	0.08515	0.188
1698	204295_at	SURF1	0.59898	0.55125	0.69605	-0.09707	0.836
1699	204297_at	PIK3C3	0.38987	0.34664	0.44741	-0.05754	0.646
1700	204299_at	FUSIP1	0.91209	0.73439	0.77055	0.14154	0.015
1701	204300_at	PET112L	0.11242	0.17855	0.19901	-0.08659	0.748
1702	204305_at	MIPEP	0.3559	0.3409	0.22228	0.13362	0.214
1703	204308_s_at	TECPR2	0.3778	0.53021	0.55699	-0.17919	0.969
1704	204319_s_at	RGS10	0.69486	0.75632	0.77291	-0.07805	0.796
1705	204327_s_at	ZNF202	0.36605	0.31894	0.3533	0.01275	0.457
1706	204331_s_at	MRPS12	0.86526	0.90061	0.93137	-0.06611	0.892
1707	204332_s_at	AGA	0.12221	0.49537	0.38364	-0.26143	0.94
1708	204334_at	KLF7	0.49379	0.42	0.34696	0.14683	0.126
1709	204335_at	CCDC94	0.13911	0.34803	0.22774	-0.08863	0.775
1710	204336_s_at	RGS19	0.39949	0.49769	0.46527	-0.06578	0.609
1711	204342_at	SLC25A24	0.10643	0.35489	0.47771	-0.37128	0.999
1712	204346_s_at	RASSF1	0.50801	0.4123	0.41422	0.09379	0.213
1713	204347_at	AK3L1	0.62627	0.54113	0.20173	0.42454	<0.001
1714	204350_s_at	MED7	0.63349	0.56993	0.58489	0.0486	0.338
1715	204352_at	TRAF5	0.53947	0.45222	0.5326	0.00687	0.477
1716	204354_at	POT1	0.62932	0.71144	0.72013	-0.09081	0.825
1717	204369_at	PIK3CA	0.61226	0.80146	0.72985	-0.11759	0.933
1718	204370_at	CLP1	0.39934	0.33834	0.29244	0.1069	0.199
1719	204372_s_at	KHSRP	0.58689	0.67759	0.65137	-0.06448	0.773
1720	204373_s_at	CEP350	0.82059	0.79433	0.83206	-0.01147	0.559
1721	204382_at	NAT9	0.23967	0.22227	0.24963	-0.00996	0.53
1722	204394_at	SLC43A1	0.4684	0.4463	0.36334	0.10506	0.242
1723	204401_at	KCNN4	0.56335	0.523	0.51434	0.04901	0.352
1724	204404_at	SLC12A2	0.35838	0.31088	0.22082	0.13756	0.232
1725	204407_at	TTF2	0.72682	0.77891	0.69694	0.02988	0.404
1726	204408_at	APEX2	0.3836	0.46627	0.14954	0.23406	0.047
1727	204415_at	IFI6	0.70985	0.62995	0.64619	0.06366	0.263
1728	204420_at	FOSL1	0.53705	0.58638	0.58808	-0.05103	0.687
1729	204423_at	MKLN1	0.56644	0.57251	0.6627	-0.09626	0.774
1730	204425_at	ARHGAP4	0.096167	0.12913	0.052468	0.043699	0.391
1731	204426_at	TMED2	0.56486	0.72915	0.73577	-0.17091	1
1732	204430_s_at	SLC2A5	0.27315	0.43378	0.48996	-0.21681	0.961
1733	204432_at	SOX12	0.20004	0.35867	0.24705	-0.04701	0.64
1734	204435_at	NUPL1	0.54817	0.74459	0.58155	-0.03338	0.615
1735	204436_at	PLEKHO2	0.7701	0.70758	0.58659	0.18351	0.037
1736	204439_at	IFI44L	0.612	0.70658	0.66754	-0.05554	0.712
1737	204440_at	CD83	0.65761	0.71997	0.76617	-0.10856	0.854
1738	204441_s_at	POLA2	0.70422	0.76328	0.66932	0.0349	0.331
1739	204444_at	KIF11	0.74676	0.70283	0.65203	0.09473	0.195
1740	204448_s_at	PDCL	0.076121	0.21474	0.28329	-0.207169	0.886
1741	204453_at	ZNF84	0.3033	0.45338	0.37282	-0.06952	0.684
1742	204458_at	PLA2G15	0.1436	0.12229	0.27306	-0.12946	0.866
1743	204459_at	CSTF2	0.56527	0.63063	0.61967	-0.0544	0.649
1744	204460_s_at	RAD1	0.36889	0.39701	0.3342	0.03469	0.397
1745	204472_at	GEM	0.40095	0.30423	0.39479	0.00616	0.526
1746	204473_s_at	ZNF592	0.38023	0.25463	0.2063	0.17393	0.136

1747	204474_at	ZNF142	0.39306	0.48526	0.4616	-0.06854	0.709
1748	204477_at	RABIF	0.037511	0.091267	0.062046	-0.024535	0.568
1749	204479_at	OSTF1	0.56692	0.75524	0.62175	-0.05483	0.721
1750	204481_at	BRPF1	0.25946	0.41944	0.31015	-0.05069	0.606
1751	204483_at	ENO3	0.38571	0.21352	0.12194	0.26377	0.053
1752	204485_s_at	TOM1L1	0.28768	0.29309	0.26143	0.02625	0.423
1753	204488_at	DOLK	0.16802	0.35479	0.30321	-0.13519	0.879
1754	204492_at	ARHGAP11A	0.57447	0.39612	0.15527	0.4192	<0.001
1755	204502_at	SAMHD1	0.39528	0.28635	0.31089	0.08439	0.248
1756	204506_at	PPP3R1	0.29561	0.15328	0.25989	0.03572	0.389
1757	204510_at	CDC7	0.5516	0.46112	0.48995	0.06165	0.291
1758	204512_at	HIVEP1	0.48581	0.55947	0.48844	-0.00263	0.498
1759	204513_s_at	ELMO1	0.71425	0.79141	0.70106	0.01319	0.473
1760	204514_at	DPH2	0.31308	0.33478	0.47828	-0.1652	0.897
1761	204516_at	ATXN7	0.85062	0.78087	0.71371	0.13691	0.005
1762	204521_at	C12orf24	0.49337	0.71048	0.5999	-0.10653	0.769
1763	204523_at	ZNF140	0.5904	0.37211	0.58301	0.00739	0.505
1764	204530_s_at	TOX	0.25407	0.3307	0.3461	-0.09203	0.763
1765	204531_s_at	BRCA1	0.48325	0.33954	0.42981	0.05344	0.329
1766	204533_at	CXCL10	0.12469	0.36543	0.19447	-0.06978	0.676
1767	204544_at	HPS5	0.48409	0.71272	0.54698	-0.06289	0.69
1768	204547_at	RAB40B	0.43315	0.38724	0.39603	0.03712	0.44
1769	204552_at	INPP4A	0.37152	0.2888	0.35011	0.02141	0.414
1770	204554_at	PPP1R3D	0.33114	0.28663	0.29539	0.03575	0.425
1771	204559_s_at	LSM7	0.79916	0.83367	0.87813	-0.07897	0.805
1772	204562_at	IRF4	0.66831	0.68551	0.5452	0.12311	0.188
1773	204563_at	SELL	0.59609	0.616	0.63925	-0.04316	0.673
1774	204565_at	ACOT13	0.79579	0.77265	0.72871	0.06708	0.228
1775	204566_at	PPM1D	0.36872	0.51795	0.5099	-0.14118	0.863
1776	204568_at	KIAA0831	0.24706	0.14063	0.26413	-0.01707	0.543
1777	204569_at	ICK	0.26314	0.32524	0.3699	-0.10676	0.73
1778	204573_at	CROT	0.57362	0.5366	0.35734	0.21628	0.01
1779	204576_s_at	CLUAP1	0.26364	0.32825	0.26686	-0.00322	0.547
1780	204593_s_at	SMCR7L	0.36145	0.23544	0.25105	0.1104	0.232
1781	204599_s_at	MRPL28	0.4449	0.65878	0.64409	-0.19919	0.934
1782	204602_at	DKK1	0.0021616	0.0011913	0.066401	-0.0642394	0.62
1783	204603_at	EXO1	0.73449	0.60063	0.51929	0.2152	0.069
1784	204605_at	CGRRF1	0.26774	0.46066	0.31593	-0.04819	0.673
1785	204608_at	ASL	0.75265	0.69239	0.61974	0.13291	0.055
1786	204610_s_at	CCDC85B	0.75306	0.79652	0.68434	0.06872	0.235
1787	204612_at	PKIA	0.40345	0.45274	0.37391	0.02954	0.424
1788	204613_at	PLCG2	0.72637	0.6182	0.58093	0.14544	0.022
1789	204616_at	UCHL3	0.62415	0.66895	0.76922	-0.14507	0.833
1790	204617_s_at	ACD	0.4342	0.68363	0.60934	-0.17514	0.883
1791	204618_s_at	GABPB1	0.46159	0.56222	0.67174	-0.21015	0.994
1792	204630_s_at	GOSR1	0.71076	0.61693	0.60974	0.10102	0.087
1793	204632_at	RPS6KA4	0.30484	0.52632	0.50254	-0.1977	0.942
1794	204634_at	NEK4	0.77447	0.75143	0.78498	-0.01051	0.54
1795	204635_at	RPS6KA5	0.29295	0.15743	0.37933	-0.08638	0.743
1796	204638_at	ACP5	0.13199	0.38831	0.32188	-0.18989	0.794
1797	204641_at	NEK2	0.74398	0.74705	0.6464	0.09758	0.229
1798	204642_at	S1PR1	0.43959	0.29699	0.26456	0.17503	0.087
1799	204646_at	DPYD	0.054012	0.26366	0.21492	-0.160908	0.883

1800	204650_s_at	APBB3	0.36121	0.32005	0.23861	0.1226	0.186
1801	204651_at	NRF1	0.49576	0.44248	0.25177	0.24399	0.018
1802	204659_s_at	GFER	0.74496	0.52063	0.78831	-0.04335	0.713
1803	204662_at	CP110	0.017869	0.36978	0.18896	-0.171091	0.918
1804	204666_s_at	RP5-1000E10.4	0.52623	0.65716	0.67168	-0.14545	0.895
1805	204674_at	LRMP	0.048765	0.24722	0.28153	-0.232765	0.939
1806	204676_at	TMEM186	0.18473	0.25988	0.45379	-0.26906	0.964
1807	204678_s_at	KCNK1	0.13396	0.38903	0.3696	-0.23564	0.778
1808	204683_at	ICAM2	0.15292	0.21694	0.20082	-0.0479	0.664
1809	204687_at	DKFZP564O0823	0.40699	0.52146	0.46131	-0.05432	0.684
1810	204688_at	SGCE	0.070733	0.34048	0.3452	-0.274467	0.954
1811	204690_at	STX8	0.53424	0.60125	0.5407	-0.00646	0.523
1812	204695_at	CDC25A	0.5821	0.56748	0.48196	0.10014	0.155
1813	204698_at	ISG20	0.54574	0.61128	0.70316	-0.15742	0.901
1814	204702_s_at	NFE2L3	0.71258	0.64636	0.71289	-0.00031	0.493
1815	204703_at	IFT88	0.17798	0.15831	0.13248	0.0455	0.374
1816	204706_at	INPP5E	0.53321	0.44393	0.51705	0.01616	0.428
1817	204709_s_at	KIF23	0.68849	0.35342	0.37304	0.31545	0.009
1818	204710_s_at	WIPI2	0.75376	0.69342	0.74077	0.01299	0.461
1819	204715_at	PANX1	0.37929	0.48826	0.51884	-0.13955	0.806
1820	204716_at	CCDC6	0.57385	0.58187	0.504	0.06985	0.294
1821	204720_s_at	DNAJC6	0.12246	0.29711	0.21744	-0.09498	0.79
1822	204725_s_at	NCK1	0.52186	0.4131	0.53083	-0.00897	0.564
1823	204727_at	WDHD1	0.62267	0.72082	0.58512	0.03755	0.403
1824	204731_at	TGFBR3	0.49961	0.63078	0.65816	-0.15855	0.872
1825	204735_at	PDE4A	0.42472	0.51537	0.37335	0.05137	0.355
1826	204739_at	CENPC1	0.67881	0.47178	0.55941	0.1194	0.181
1827	204740_at	CNKSR1	0.10691	0.2248	0.064117	0.042793	0.392
1828	204742_s_at	PDS5B	0.61876	0.71868	0.55402	0.06474	0.35
1829	204744_s_at	IARS	0.70507	0.71841	0.68601	0.01906	0.42
1830	204747_at	IFIT3	0.63633	0.64513	0.62647	0.00986	0.474
1831	204759_at	RCBTB2	0.5149	0.61475	0.58587	-0.07097	0.683
1832	204761_at	USP6NL	0.60451	0.65452	0.63877	-0.03426	0.648
1833	204766_s_at	NUDT1	0.50016	0.52865	0.71135	-0.21119	0.925
1834	204767_s_at	FEN1	0.44706	0.57423	0.55253	-0.10547	0.807
1835	204771_s_at	TTF1	0.78783	0.71056	0.69617	0.09166	0.098
1836	204774_at	EVI2A	0.66403	0.77349	0.73711	-0.07308	0.801
1837	204777_s_at	MAL	0.72725	0.73931	0.70043	0.02682	0.406
1838	204779_s_at	HOXB7	0.61837	0.42414	0.455	0.16337	0.094
1839	204781_s_at	FAS	0.54935	0.25036	0.042918	0.506432	<0.001
1840	204786_s_at	IFNAR2	0.53953	0.4551	0.50813	0.0314	0.406
1841	204788_s_at	PPOX	0.52204	0.39711	0.47337	0.04867	0.319
1842	204789_at	FMNL1	0.43706	0.44409	0.39783	0.03923	0.374
1843	204790_at	SMAD7	0.12415	0.35554	0.33911	-0.21496	0.964
1844	204794_at	DUSP2	0.46232	0.45731	0.51056	-0.04824	0.724
1845	204795_at	PRR3	0.45321	0.45647	0.51006	-0.05685	0.639
1846	204798_at	MYB	0.46483	0.66656	0.63389	-0.16906	0.97
1847	204804_at	TRIM21	0.58435	0.47763	0.53825	0.0461	0.357
1848	204805_s_at	H1FX	0.40959	0.36846	0.23348	0.17611	0.09
1849	204807_at	TMEM5	0.73479	0.61466	0.6097	0.12509	0.056
1850	204809_at	CLPX	0.72991	0.82274	0.66232	0.06759	0.156
1851	204812_at	ZW10	0.66907	0.71384	0.79115	-0.12208	0.903
1852	204813_at	MAPK10	0.11991	0.25882	0.19519	-0.07528	0.646

1853	204821_at	BTN3A3	0.74506	0.45701	0.55376	0.1913	0.019
1854	204822_at	TTK	0.7786	0.65742	0.67332	0.10528	0.069
1855	204825_at	MELK	0.63182	0.60817	0.56219	0.06963	0.263
1856	204828_at	RAD9A	0.44038	0.6133	0.58498	-0.1446	0.88
1857	204831_at	CDK8	0.82033	0.8199	0.85302	-0.03269	0.698
1858	204832_s_at	BMPR1A	0.31022	0.14924	0.33641	-0.02619	0.568
1859	204834_at	FGL2	0.024438	0.26985	0.33791	-0.313472	0.995
1860	204835_at	POLA1	0.64043	0.56518	0.62148	0.01895	0.405
1861	204836_at	GLDC	0.30984	0.48304	0.3025	0.00734	0.47
1862	204838_s_at	MLH3	0.00057343	0.17204	0.15751	-0.15693657	0.846
1863	204839_at	POP5	0.75584	0.80567	0.80809	-0.05225	0.657
1864	204840_s_at	EEA1	0.47384	0.48036	0.51226	-0.03842	0.598
1865	204847_at	ZBTB11	0.71159	0.4441	0.70567	0.00592	0.444
1866	204849_at	TCFL5	0.22899	0.31793	0.30903	-0.08004	0.733
1867	204852_s_at	PTPN7	0.65152	0.47011	0.56824	0.08328	0.245
1868	204853_at	ORC2L	0.68903	0.39009	0.46345	0.22558	0.052
1869	204857_at	MAD1L1	0.55654	0.53999	0.65926	-0.10272	0.807
1870	204862_s_at	NME3	0.67063	0.50741	0.70569	-0.03506	0.591
1871	204866_at	PHF16	0.4847	0.56602	0.65139	-0.16669	0.924
1872	204867_at	GCHFR	0.69109	0.69662	0.74497	-0.05388	0.708
1873	204868_at	ICT1	0.79306	0.7605	0.75053	0.04253	0.308
1874	204880_at	MGMT	0.1067	0.42744	0.3687	-0.262	0.941
1875	204881_s_at	UGCG	0.45497	0.56666	0.54827	-0.0933	0.768
1876	204883_s_at	HUS1	0.41673	0.42018	0.24299	0.17374	0.196
1877	204887_s_at	PLK4	0.7975	0.8596	0.83777	-0.04027	0.7
1878	204890_s_at	LCK	0.37287	0.47275	0.46097	-0.0881	0.809
1879	204905_s_at	EEF1E1	0.76237	0.8469	0.83231	-0.06994	0.858
1880	204909_at	DDX6	0.27761	0.19615	0.23933	0.03828	0.402
1881	204912_at	IL10RA	0.52114	0.5791	0.4808	0.04034	0.409
1882	204917_s_at	MLLT3	0.35719	0.39039	0.48282	-0.12563	0.777
1883	204923_at	SASH3	0.61218	0.60229	0.54956	0.06262	0.304
1884	204937_s_at	ZNF274	0.24675	0.33541	0.31807	-0.07132	0.658
1885	204946_s_at	TOP3A	0.48937	0.36069	0.3284	0.16097	0.122
1886	204949_at	ICAM3	0.4989	0.62538	0.51087	-0.01197	0.533
1887	204950_at	CARD8	0.18788	0.15384	0.22417	-0.03629	0.628
1888	204955_at	SRPX	0.13729	0.1677	0.088971	0.048319	0.379
1889	204959_at	MNDA	0.25925	0.55764	0.5446	-0.28535	0.989
1890	204960_at	PTPRCAP	0.59368	0.73574	0.68602	-0.09234	0.786
1891	204961_s_at	LOC648998	0.71538	0.63608	0.65101	0.06437	0.169
1892	204962_s_at	CENPA	0.70141	0.61347	0.30325	0.39816	<0.001
1893	204968_at	C6orf47	0.56891	0.23745	0.33045	0.23846	0.06
1894	204970_s_at	MAFG	0.091677	0.23562	0.19582	-0.104143	0.754
1895	204976_s_at	AMMECR1	0.39044	0.46301	0.55625	-0.16581	0.888
1896	204977_at	DDX10	0.58899	0.47806	0.5951	-0.00611	0.537
1897	204978_at	SFRS16	0.56252	0.43761	0.29953	0.26299	0.045
1898	204985_s_at	TRAPPC6A	0.83986	0.79111	0.82231	0.01755	0.364
1899	204992_s_at	PFN2	0.34546	0.52244	0.52367	-0.17821	0.89
1900	204994_at	MX2	0.73242	0.76329	0.69902	0.0334	0.357
1901	204995_at	CDK5R1	0.63031	0.71535	0.71477	-0.08446	0.829
1902	205004_at	NKRF	0.45129	0.27839	0.31324	0.13805	0.206
1903	205010_at	GNL3L	0.40941	0.21196	0.27051	0.1389	0.147
1904	205012_s_at	HAGH	0.21441	0.15157	0.41467	-0.20026	0.888
1905	205013_s_at	ADORA2A	0.22795	0.57344	0.37683	-0.14888	0.856

1906	205027_s_at	MAP3K8	0.55794	0.71936	0.70948	-0.15154	0.876
1907	205034_at	CCNE2	0.38695	0.57004	0.57583	-0.18888	0.966
1908	205036_at	LSM6	0.8246	0.78824	0.82166	0.00294	0.468
1909	205042_at	GNE	0.046193	0.35853	0.39166	-0.345467	0.998
1910	205046_at	CENPE	0.68801	0.7343	0.18933	0.49868	<0.001
1911	205047_s_at	ASNS	0.282	0.46801	0.32281	-0.04081	0.631
1912	205048_s_at	PSPH	0.0069856	0.071201	0.033055	-0.0260694	0.573
1913	205049_s_at	CD79A	0.70022	0.76871	0.70891	-0.00869	0.544
1914	205052_at	AUH	0.46298	0.53483	0.28931	0.17367	0.092
1915	205053_at	PRIM1	0.49726	0.6993	0.59751	-0.10025	0.825
1916	205055_at	ITGAE	0.51926	0.74507	0.74745	-0.22819	0.958
1917	205060_at	PARG	0.69018	0.51659	0.6683	0.02188	0.414
1918	205061_s_at	EXOSC9	0.68294	0.51472	0.67479	0.00815	0.454
1919	205063_at	SIP1	0.86101	0.77728	0.85239	0.00862	0.439
1920	205069_s_at	ARHGAP26	0.027531	0.18654	0.084543	-0.057012	0.619
1921	205070_at	ING3	0.22572	0.28291	0.38022	-0.1545	0.836
1922	205078_at	PIGF	0.6926	0.75237	0.45494	0.23766	0.001
1923	205081_at	CRIP1	0.60603	0.65095	0.7126	-0.10657	0.771
1924	205085_at	ORC1L	0.75582	0.64952	0.5808	0.17502	0.018
1925	205087_at	RWDD3	0.58621	0.68634	0.65422	-0.06801	0.777
1926	205089_at	ZNF7	0.46986	0.43271	0.3545	0.11536	0.204
1927	205090_s_at	NAGPA	0.34744	0.39067	0.50801	-0.16057	0.862
1928	205094_at	PEX12	0.41245	0.51549	0.54407	-0.13162	0.858
1929	205097_at	SLC26A2	0.67274	0.38688	0.53594	0.1368	0.232
1930	205098_at	CCR1	0.083861	0.33067	0.25983	-0.175969	0.928
1931	205103_at	C1orf61	0.091404	0.41052	0.35116	-0.259756	0.984
1932	205105_at	MAN2A1	0.61673	0.73328	0.75172	-0.13499	0.816
1933	205107_s_at	EFNA4	0.20229	0.13329	0.16382	0.03847	0.453
1934	205114_s_at	CCL3	0.38463	0.46438	0.47822	-0.09359	0.752
1935	205126_at	VRK2	0.61568	0.74204	0.54124	0.07444	0.175
1936	205129_at	NPM3	0.70147	0.71226	0.77739	-0.07592	0.74
1937	205132_at	ACTC1	0.067839	0.11503	0.22782	-0.159981	0.839
1938	205133_s_at	HSPE1	0.77161	0.76238	0.70976	0.06185	0.277
1939	205134_s_at	NUFIP1	0.63522	0.6541	0.57629	0.05893	0.242
1940	205140_at	FPGT	0.66495	0.63077	0.68769	-0.02274	0.634
1941	205145_s_at	LOC649851	0.55261	0.516	0.44675	0.10586	0.164
1942	205159_at	CSF2RB	0.28076	0.43545	0.47232	-0.19156	0.922
1943	205162_at	ERCC8	0.18099	0.3034	0.17014	0.01085	0.489
1944	205169_at	RBBP5	0.46884	0.51169	0.44592	0.02292	0.442
1945	205170_at	STAT2	0.73546	0.7793	0.77476	-0.0393	0.697
1946	205171_at	PTPN4	0.060722	0.15641	0.31009	-0.249368	0.946
1947	205176_s_at	ITGB3BP	0.57532	0.76723	0.65645	-0.08113	0.812
1948	205178_s_at	RBBP6	0.65122	0.60385	0.65785	-0.00663	0.523
1949	205181_at	ZNF193	0.34667	0.22177	0.070902	0.275768	0.023
1950	205188_s_at	SMAD5	0.42809	0.20202	0.25439	0.1737	0.109
1951	205189_s_at	FANCC	0.38877	0.50108	0.35532	0.03345	0.403
1952	205191_at	RP2	0.802	0.73284	0.86967	-0.06767	0.769
1953	205192_at	MAP3K14	0.50249	0.45473	0.57322	-0.07073	0.695
1954	205198_s_at	ATP7A	0.44759	0.53696	0.46091	-0.01332	0.548
1955	205205_at	RELB	0.52118	0.67351	0.45741	0.06377	0.265
1956	205217_at	TIMM8A	0.53033	0.60593	0.48721	0.04312	0.396
1957	205218_at	POLR3F	0.54343	0.46989	0.45758	0.08585	0.271
1958	205220_at	NIACR2	0.27037	0.35069	0.27667	-0.0063	0.555

1959	205222_at	EHHADH	0.019245	0.027469	0.18891	-0.169665	0.85
1960	205224_at	SURF2	0.63206	0.66372	0.72532	-0.09326	0.771
1961	205229_s_at	COCH	0.47235	0.59608	0.52091	-0.04856	0.657
1962	205231_s_at	EPM2A	0.39368	0.21975	0.22146	0.17222	0.11
1963	205235_s_at	KIF20B	0.45666	0.50188	0.6284	-0.17174	0.887
1964	205238_at	TRMT2B	0.37755	0.18582	0.22361	0.15394	0.222
1965	205241_at	SCO2	0.60643	0.53902	0.6635	-0.05707	0.655
1966	205245_at	PARD6A	0.21302	0.1685	0.31823	-0.10521	0.78
1967	205246_at	PEX13	0.26857	0.30331	0.23697	0.0316	0.413
1968	205249_at	EGR2	0.5102	0.54495	0.49069	0.01951	0.444
1969	205256_at	ZBTB39	0.25023	0.16206	0.26474	-0.01451	0.53
1970	205260_s_at	ACYP1	0.49115	0.50626	0.53645	-0.0453	0.62
1971	205263_at	BCL10	0.49877	0.50181	0.45231	0.04646	0.351
1972	205264_at	CD3EAP	0.20043	0.18823	0.13296	0.06747	0.366
1973	205267_at	POU2AF1	0.62285	0.60823	0.5585	0.06435	0.213
1974	205269_at	LCP2	0.67646	0.68556	0.739	-0.06254	0.803
1975	205273_s_at	PITRM1	0.49473	0.54854	0.61571	-0.12098	0.815
1976	205283_at	FKTN	0.7017	0.44418	0.3957	0.306	<0.001
1977	205291_at	IL2RB	0.6219	0.62781	0.65238	-0.03048	0.599
1978	205292_s_at	HNRNPA2B1	0.64344	0.84366	0.82961	-0.18617	0.991
1979	205296_at	RBL1	0.76223	0.66617	0.62306	0.13917	0.171
1980	205297_s_at	CD79B	0.52687	0.58715	0.45288	0.07399	0.225
1981	205298_s_at	BTN2A2	0.095306	0.27565	0.2548	-0.159494	0.832
1982	205300_s_at	SNRNP35	0.47292	0.55646	0.62513	-0.15221	0.893
1983	205301_s_at	OGG1	0.080207	0.069832	0.072562	0.007645	0.51
1984	205308_at	FAM164A	0.44087	0.4339	0.41662	0.02425	0.444
1985	205310_at	FBXO46	0.31999	0.4071	0.53877	-0.21878	0.963
1986	205315_s_at	SNTB2	0.28643	0.31982	0.35361	-0.06718	0.659
1987	205321_at	EIF2S3	0.7016	0.60012	0.59503	0.10657	0.258
1988	205322_s_at	MTF1	0.38509	0.25772	0.19313	0.19196	0.091
1989	205327_s_at	ACVR2A	0.21156	0.18532	0.23765	-0.02609	0.542
1990	205328_at	CLDN10	0.42765	0.306	0.36437	0.06328	0.365
1991	205329_s_at	SNX4	0.56552	0.46075	0.53	0.03552	0.392
1992	205333_s_at	RCE1	0.60719	0.55816	0.44417	0.16302	0.102
1993	205335_s_at	SRP19	0.83175	0.8175	0.78449	0.04726	0.271
1994	205339_at	STIL	0.74472	0.63908	0.75227	-0.00755	0.549
1995	205340_at	ZBTB24	0.10303	0.049398	0.23517	-0.13214	0.803
1996	205345_at	BARD1	0.5584	0.5597	0.46559	0.09281	0.226
1997	205346_at	ST3GAL2	0.73843	0.65451	0.5218	0.21663	0.016
1998	205349_at	GNA15	0.1798	0.40729	0.23682	-0.05702	0.653
1999	205352_at	SERPINI1	0.52684	0.44073	0.25803	0.26881	0.01
2000	205354_at	GAMT	0.38076	0.62707	0.53936	-0.1586	0.895
2001	205355_at	ACADSB	0.46833	0.65082	0.49966	-0.03133	0.613
2002	205356_at	USP13	0.26426	0.29552	0.46928	-0.20502	0.93
2003	205361_s_at	PFDN4	0.89583	0.89469	0.89627	-0.00044	0.494
2004	205367_at	SH2B2	0.85259	0.76129	0.77136	0.08123	0.179
2005	205372_at	PLAG1	0.26288	0.30291	0.32264	-0.05976	0.738
2006	205376_at	INPP4B	0.40677	0.5226	0.47391	-0.06714	0.687
2007	205393_s_at	CHEK1	0.75679	0.70272	0.67785	0.07894	0.155
2008	205401_at	AGPS	0.64208	0.7259	0.7156	-0.07352	0.747
2009	205406_s_at	SPA17	0.54629	0.54911	0.49768	0.04861	0.357
2010	205408_at	MLLT10	0.74752	0.66653	0.70907	0.03845	0.341
2011	205411_at	STK4	0.14953	0.27606	0.47207	-0.32254	0.945

2012	205412_at	ACAT1	0.62038	0.77629	0.64653	-0.02615	0.593
2013	205414_s_at	RICH2	0.1321	0.30905	0.34668	-0.21458	0.92
2014	205416_s_at	ATXN3	0.33384	0.29466	0.48605	-0.15221	0.895
2015	205417_s_at	DAG1	0.66306	0.58126	0.51197	0.15109	0.099
2016	205419_at	GPR183	0.54935	0.60529	0.61723	-0.06788	0.688
2017	205423_at	AP1B1	0.65801	0.64705	0.55106	0.10695	0.18
2018	205429_s_at	MPP6	0.31896	0.31869	0.25166	0.0673	0.305
2019	205436_s_at	H2AFX	0.80869	0.82413	0.82512	-0.01643	0.58
2020	205443_at	SNAPC1	0.42369	0.35718	0.37406	0.04963	0.424
2021	205449_at	SAC3D1	0.71987	0.79856	0.75921	-0.03934	0.668
2022	205452_at	PIGB	0.67124	0.65383	0.66666	0.00458	0.494
2023	205461_at	RAB35	0.69251	0.28043	0.2872	0.40531	0.293
2024	205463_s_at	PDGFA	0.0080979	0.091571	0.13416	-0.1260621	0.815
2025	205467_at	CASP10	0.51459	0.20395	0.3876	0.12699	0.181
2026	205469_s_at	IRF5	0.19194	0.36114	0.34647	-0.15453	0.854
2027	205474_at	CRLF3	0.68368	0.56664	0.60887	0.07481	0.27
2028	205480_s_at	UGP2	0.55028	0.54512	0.59611	-0.04583	0.692
2029	205483_s_at	ISG15	0.67744	0.68656	0.68707	-0.00963	0.515
2030	205484_at	SIT1	0.31193	0.26765	0.2185	0.09343	0.256
2031	205497_at	ZNF175	0.33278	0.36488	0.32087	0.01191	0.456
2032	205504_at	BTK	0.43558	0.18019	0.23298	0.2026	0.028
2033	205512_s_at	AIFM1	0.63211	0.5748	0.52038	0.11173	0.233
2034	205518_s_at	CMAH	0.39389	0.35046	0.44021	-0.04632	0.629
2035	205519_at	WDR76	0.53938	0.45227	0.28389	0.25549	0.005
2036	205521_at	EXOG	0.40691	0.30769	0.26659	0.14032	0.2
2037	205526_s_at	KATNA1	0.20788	0.32922	0.37582	-0.16794	0.874
2038	205527_s_at	GEMIN4	0.76058	0.66659	0.54658	0.214	<0.001
2039	205531_s_at	GLS2	0.26463	0.19642	0.18549	0.07914	0.268
2040	205540_s_at	RRAGB	0.45808	0.4587	0.37206	0.08602	0.28
2041	205541_s_at	GSPT2	0.39455	0.48614	0.4456	-0.05105	0.661
2042	205542_at	STEAP1	0.79285	0.80696	0.7731	0.01975	0.392
2043	205543_at	HSPA4L	0.59729	0.70352	0.65179	-0.0545	0.736
2044	205544_s_at	CR2	0.80003	0.81207	0.75948	0.04055	0.351
2045	205546_s_at	TYK2	0.72393	0.81383	0.64052	0.08341	0.172
2046	205550_s_at	BRE	0.28025	0.4222	0.36436	-0.08411	0.742
2047	205552_s_at	OAS1	0.71301	0.7543	0.75889	-0.04588	0.779
2048	205554_s_at	DNASE1L3	0.39873	0.44594	0.45649	-0.05776	0.703
2049	205562_at	RPP38	0.56929	0.24414	0.5398	0.02949	0.414
2050	205565_s_at	FXN	0.54156	0.67144	0.61678	-0.07522	0.723
2051	205569_at	LAMP3	0.70441	0.77585	0.63284	0.07157	0.21
2052	205570_at	PIP4K2A	0.12064	0.20927	0.10852	0.01212	0.498
2053	205571_at	LIPT1	0.29065	0.29051	0.23966	0.05099	0.368
2054	205584_at	ALG13	0.39679	0.35536	0.47727	-0.08048	0.72
2055	205585_at	ETV6	0.5756	0.48251	0.40803	0.16757	0.058
2056	205588_s_at	FGFR1OP	0.40031	0.52558	0.3619	0.03841	0.399
2057	205590_at	RASGRP1	0.59014	0.6254	0.54706	0.04308	0.397
2058	205594_at	ZNF652	0.1409	0.22127	0.18782	-0.04692	0.628
2059	205596_s_at	SMURF2	0.44066	0.57254	0.68771	-0.24705	0.937
2060	205599_at	TRAF1	0.64998	0.74327	0.6331	0.01688	0.459
2061	205603_s_at	DIAPH2	0.11812	0.45978	0.34997	-0.23185	0.829
2062	205607_s_at	SCYL3	0.37146	0.41831	0.36472	0.00674	0.496
2063	205621_at	ALKBH1	0.5691	0.6079	0.57998	-0.01088	0.546
2064	205628_at	PRIM2	0.73222	0.6171	0.49139	0.24083	0.003

2065	205633_s_at	ALAS1	0.42858	0.48533	0.48423	-0.05565	0.688
2066	205642_at	CEP110	0.39031	0.41427	0.43473	-0.04442	0.629
2067	205644_s_at	SNRPG	0.93107	0.87993	0.86939	0.06168	0.044
2068	205655_at	MDM4	0.39447	0.37381	0.33571	0.05876	0.348
2069	205659_at	HDAC9	0.021597	0.18913	0.094758	-0.073161	0.715
2070	205664_at	KIN	0.75838	0.27627	0.47023	0.28815	0.011
2071	205667_at	WRN	0.25808	0.21927	0.3093	-0.05122	0.66
2072	205668_at	LY75	0.42834	0.46882	0.50535	-0.07701	0.74
2073	205671_s_at	HLA-DOB	0.65044	0.72833	0.68248	-0.03204	0.611
2074	205672_at	XPA	0.21059	0.27718	0.27522	-0.06463	0.67
2075	205677_s_at	DLEU1	0.37924	0.37146	0.41656	-0.03732	0.595
2076	205681_at	BCL2A1	0.69486	0.61923	0.70248	-0.00762	0.569
2077	205684_s_at	DENND4C	0.48917	0.67273	0.64676	-0.15759	0.898
2078	205687_at	UBFD1	0.67739	0.61016	0.64692	0.03047	0.334
2079	205692_s_at	CD38	0.56441	0.70133	0.69948	-0.13507	0.928
2080	205701_at	IPO8	0.3241	0.34703	0.28273	0.04137	0.41
2081	205704_s_at	ATP6V0A2	0.15444	0.37826	0.27534	-0.1209	0.81
2082	205716_at	SLC25A40	0.65932	0.62985	0.60896	0.05036	0.327
2083	205718_at	ITGB7	0.72411	0.68758	0.68642	0.03769	0.341
2084	205733_at	BLM	0.68872	0.5759	0.57785	0.11087	0.128
2085	205740_s_at	RBM42	0.79125	0.76207	0.83428	-0.04303	0.748
2086	205746_s_at	ADAM17	0.11301	0.16293	0.26552	-0.15251	0.845
2087	205748_s_at	RNF126	0.69084	0.49976	0.34205	0.34879	0.001
2088	205750_at	BPHL	0.28279	0.39542	0.29499	-0.0122	0.567
2089	205756_s_at	F8	0.017701	0.16038	0.068842	-0.051141	0.646
2090	205761_s_at	DUS4L	0.45029	0.31475	0.35231	0.09798	0.205
2091	205770_at	GSR	0.63586	0.53857	0.55316	0.0827	0.167
2092	205773_at	CPEB3	0.47358	0.56983	0.59225	-0.11867	0.813
2093	205775_at	FAM50B	0.57032	0.33866	0.22399	0.34633	0.004
2094	205781_at	C16orf7	0.013197	0.052506	0.00045692	0.01274008	0.479
2095	205788_s_at	ZC3H11A	0.84526	0.82285	0.83977	0.00549	0.472
2096	205790_at	SKAP1	0.071195	0.30678	0.11091	-0.039715	0.623
2097	205796_at	TCP11L1	0.47047	0.43559	0.55909	-0.08862	0.816
2098	205801_s_at	RASGRP3	0.23942	0.31892	0.34343	-0.10401	0.762
2099	205804_s_at	TRAF3IP3	0.26386	0.17825	0.36075	-0.09689	0.73
2100	205807_s_at	TUFT1	0.084132	0.1869	0.17329	-0.089158	0.71
2101	205809_s_at	WASL	0.34818	0.32668	0.21415	0.13403	0.171
2102	205811_at	POLG2	0.50685	0.1001	0.33927	0.16758	0.158
2103	205830_at	CLGN	0.030385	0.303	0.35163	-0.321245	0.996
2104	205832_at	CPA4	0.11644	0.089679	0.095492	0.020948	0.493
2105	205841_at	JAK2	0.30276	0.37647	0.50915	-0.20639	0.934
2106	205847_at	PRSS22	0.46339	0.33443	0.43165	0.03174	0.437
2107	205848_at	GAS2	0.4247	0.58987	0.57783	-0.15313	0.94
2108	205849_s_at	UQCRB	0.93737	0.9112	0.90606	0.03131	0.166
2109	205851_at	NME6	0.24484	0.46633	0.28677	-0.04193	0.599
2110	205855_at	ZNF197	0.30381	0.23119	0.25913	0.04468	0.388
2111	205859_at	LY86	0.50168	0.61968	0.54053	-0.03885	0.605
2112	205861_at	SPIB	0.62189	0.6678	0.56279	0.0591	0.309
2113	205865_at	ARID3A	0.33185	0.42562	0.47293	-0.14108	0.835
2114	205873_at	PIGL	0.23111	0.14769	0.15362	0.07749	0.345
2115	205884_at	ITGA4	0.64612	0.59932	0.54855	0.09757	0.067
2116	205890_s_at	GABBR1	0.57086	0.6334	0.66492	-0.09406	0.823
2117	205895_s_at	NOLC1	0.69252	0.68545	0.70319	-0.01067	0.543

2118	205901_at	PNOC	0.37169	0.53954	0.58616	-0.21447	0.948
2119	205902_at	KCNN3	0.42254	0.48623	0.62133	-0.19879	0.844
2120	205909_at	POLE2	0.75972	0.8132	0.7651	-0.00538	0.521
2121	205917_at	ZNF264	0.15747	0.35859	0.26193	-0.10446	0.779
2122	205922_at	VNN2	0.2517	0.35204	0.32749	-0.07579	0.71
2123	205928_at	ZNF443	0.3307	0.55541	0.71385	-0.38315	1
2124	205930_at	GTF2E1	0.35602	0.39064	0.48898	-0.13296	0.805
2125	205932_s_at	MSX1	0.52534	0.62409	0.56578	-0.04044	0.664
2126	205934_at	PLCL1	0.20688	0.31258	0.17219	0.03469	0.467
2127	205945_at	IL6R	0.22756	0.44336	0.41185	-0.18429	0.869
2128	205953_at	LRIG2	0.48038	0.42511	0.22886	0.25152	0.057
2129	205955_at	TAF6L	0.1242	0.08507	0.024514	0.099686	0.26
2130	205963_s_at	DNAJA3	0.75451	0.70968	0.73624	0.01827	0.408
2131	205964_at	ZNF426	0.21499	0.27583	0.21259	0.0024	0.455
2132	205965_at	BATF	0.71109	0.67691	0.72213	-0.01104	0.545
2133	205967_at	HIST1H4C	0.55026	0.65051	0.53739	0.01287	0.469
2134	205978_at	KL	0.064821	0.37468	0.42397	-0.359149	0.994
2135	205981_s_at	ING2	0.451	0.54229	0.34861	0.10239	0.215
2136	205991_s_at	PRRX1	0.17317	0.35008	0.3254	-0.15223	0.847
2137	205992_s_at	IL15	0.5789	0.49652	0.46582	0.11308	0.226
2138	205996_s_at	AK2	0.70241	0.64339	0.56536	0.13705	0.061
2139	206006_s_at	KIAA1009	0.2349	0.20631	0.31475	-0.07985	0.724
2140	206016_at	CCDC22	0.64744	0.60729	0.61263	0.03481	0.392
2141	206020_at	SOCS6	0.46213	0.23285	0.22864	0.23349	0.068
2142	206026_s_at	TNFAIP6	0.29677	0.36371	0.39594	-0.09917	0.799
2143	206031_s_at	USP5	0.20538	0.27536	0.28705	-0.08167	0.708
2144	206035_at	REL	0.39128	0.59331	0.55508	-0.1638	0.901
2145	206037_at	CCBL1	0.37802	0.28795	0.28473	0.09329	0.215
2146	206038_s_at	NR2C2	0.39845	0.16577	0.13074	0.26771	0.025
2147	206039_at	RAB33A	0.49087	0.6217	0.51334	-0.02247	0.579
2148	206044_s_at	BRAF	0.51648	0.43567	0.55984	-0.04336	0.654
2149	206050_s_at	RNH1	0.64618	0.63214	0.57545	0.07073	0.265
2150	206052_s_at	SLBP	0.81519	0.63671	0.70889	0.1063	0.016
2151	206055_s_at	SNRPA1	0.55284	0.68823	0.68598	-0.13314	0.833
2152	206059_at	ZNF91	0.57948	0.67313	0.65134	-0.07186	0.71
2153	206061_s_at	DICER1	0.52751	0.70544	0.71592	-0.18841	0.96
2154	206074_s_at	HMGA1	0.79051	0.80837	0.81531	-0.0248	0.63
2155	206082_at	HCP5	0.44196	0.38583	0.52607	-0.08411	0.727
2156	206096_at	ZNF35	0.1425	0.12747	0.19898	-0.05648	0.674
2157	206098_at	ZBTB6	0.55895	0.58936	0.75629	-0.19734	0.983
2158	206102_at	GINS1	0.68688	0.81403	0.74476	-0.05788	0.752
2159	206106_at	MAPK12	0.14634	0.18969	0.099048	0.047292	0.469
2160	206108_s_at	SFRS6	0.1934	0.15856	0.15135	0.04205	0.396
2161	206115_at	EGR3	0.17551	0.11654	0.13448	0.04103	0.398
2162	206129_s_at	ARSB	0.37372	0.1449	0.13416	0.23956	0.074
2163	206132_at	MCC	0.67046	0.54703	0.41396	0.2565	0.026
2164	206133_at	XAF1	0.48818	0.61389	0.60321	-0.11503	0.85
2165	206134_at	ADAMDEC1	0.34215	0.3179	0.37195	-0.0298	0.587
2166	206141_at	MOCS3	0.56029	0.51989	0.49813	0.06216	0.281
2167	206150_at	CD27	0.28637	0.38413	0.38836	-0.10199	0.77
2168	206158_s_at	CNBP	0.67053	0.77844	0.73566	-0.06513	0.804
2169	206181_at	SLAMF1	0.6538	0.55801	0.64126	0.01254	0.469
2170	206182_at	ZNF134	0.19451	0.13525	0.21071	-0.0162	0.602

2171	206184_at	CRKL	0.66148	0.57382	0.63512	0.02636	0.392
2172	206188_at	ZNF623	0.73962	0.36635	0.65729	0.08233	0.126
2173	206194_at	HOXC4	0.1304	0.36025	0.33662	-0.20622	0.942
2174	206200_s_at	ANXA11	0.76389	0.74414	0.7151	0.04879	0.297
2175	206219_s_at	VAV1	0.69977	0.5321	0.49016	0.20961	0.054
2176	206233_at	B4GALT6	0.7019	0.76461	0.72834	-0.02644	0.638
2177	206235_at	LIG4	0.364	0.56296	0.59157	-0.22757	0.926
2178	206240_s_at	ZNF136	0.48796	0.23803	0.44638	0.04158	0.364
2179	206247_at	MICB	0.48908	0.52068	0.62238	-0.1333	0.815
2180	206255_at	BLK	0.49434	0.45967	0.4107	0.08364	0.365
2181	206257_at	CCDC9	0.36367	0.43624	0.27503	0.08864	0.322
2182	206261_at	ZNF239	0.040163	0.029777	0.077072	-0.036909	0.62
2183	206263_at	FMO4	0.17174	0.29814	0.21261	-0.04087	0.597
2184	206302_s_at	NUDT4	0.66743	0.76105	0.76353	-0.0961	0.836
2185	206308_at	TRDMT1	0.38095	0.43055	0.33653	0.04442	0.399
2186	206313_at	HLA-DOA	0.69041	0.60903	0.63188	0.05853	0.329
2187	206314_at	ZNF167	0.014008	0.081915	0.10211	-0.088102	0.76
2188	206316_s_at	KNTC1	0.66378	0.74584	0.76271	-0.09893	0.843
2189	206335_at	GALNS	0.33997	0.24022	0.2324	0.10757	0.182
2190	206337_at	CCR7	0.82276	0.84576	0.8177	0.00506	0.487
2191	206357_at	OPA3	0.27868	0.27073	0.24079	0.03789	0.39
2192	206361_at	GPR44	0.041975	0.088944	0.21852	-0.176545	0.877
2193	206364_at	KIF14	0.69368	0.74461	0.49197	0.20171	0.047
2194	206398_s_at	CD19	0.53776	0.6071	0.60708	-0.06932	0.742
2195	206412_at	FER	0.26714	0.11868	0.25919	0.00795	0.475
2196	206440_at	LIN7A	0.55458	0.59482	0.48941	0.06517	0.293
2197	206445_s_at	PRMT1	0.61864	0.74808	0.71677	-0.09813	0.805
2198	206451_at	TBCCD1	0.64225	0.59308	0.45858	0.18367	0.065
2199	206478_at	KIAA0125	0.0023056	0.14776	0.1777	-0.1753944	0.946
2200	206491_s_at	NAPA	0.67	0.501	0.54349	0.12651	0.228
2201	206492_at	FHIT	0.49727	0.35295	0.29433	0.20294	0.068
2202	206497_at	C7orf44	0.28623	0.047086	0.10142	0.18481	0.127
2203	206500_s_at	C14orf106	0.41464	0.47941	0.53919	-0.12455	0.859
2204	206507_at	ZSCAN12	0.19942	0.26742	0.24676	-0.04734	0.646
2205	206508_at	CD70	0.63146	0.64409	0.69214	-0.06068	0.673
2206	206513_at	AIM2	0.36911	0.56007	0.53488	-0.16577	0.945
2207	206515_at	CYP4F3	0.3079	0.30092	0.32086	-0.01296	0.565
2208	206533_at	CHRNA5	0.16273	0.21623	0.1856	-0.02287	0.611
2209	206536_s_at	XIAP	0.20666	0.29234	0.11788	0.08878	0.329
2210	206542_s_at	SMARCA2	0.75778	0.73056	0.80755	-0.04977	0.774
2211	206550_s_at	NUP155	0.7525	0.67997	0.69809	0.05441	0.288
2212	206553_at	OAS2	0.69763	0.72653	0.72491	-0.02728	0.613
2213	206583_at	ZNF673	0.42665	0.18663	0.35669	0.06996	0.325
2214	206584_at	LY96	0.67602	0.66961	0.72555	-0.04953	0.693
2215	206587_at	CCT6B	0.39132	0.44325	0.48141	-0.09009	0.804
2216	206588_at	DAZL	0.43751	0.46345	0.46781	-0.0303	0.623
2217	206592_s_at	AP3D1	0.62686	0.71466	0.76696	-0.1401	0.835
2218	206593_s_at	MED22	0.76996	0.67753	0.54595	0.22401	0.007
2219	206608_s_at	RPGRIP1	0.15903	0.27786	0.062744	0.096286	0.334
2220	206613_s_at	TAF1A	0.42952	0.19032	0.34137	0.08815	0.32
2221	206618_at	IL18R1	0.71612	0.65144	0.69667	0.01945	0.429
2222	206621_s_at	EIF4H	0.70477	0.49648	0.45328	0.25149	0.013
2223	206632_s_at	APOBEC3B	0.32896	0.49075	0.4832	-0.15424	0.879

2224	206641_at	TNFRSF17	0.50675	0.64662	0.63349	-0.12674	0.782
2225	206649_s_at	TFE3	0.25121	0.31777	0.3862	-0.13499	0.843
2226	206652_at	ZMYM5	0.19319	0.15115	0.20228	-0.00909	0.53
2227	206654_s_at	POLR3G	0.58312	0.59128	0.42874	0.15438	0.106
2228	206656_s_at	C20orf3	0.51635	0.4218	0.47025	0.0461	0.407
2229	206659_at	FLJ14082	0.18087	0.24239	0.23876	-0.05789	0.647
2230	206667_s_at	SCAMP1	0.60353	0.63246	0.67002	-0.06649	0.839
2231	206683_at	ZNF165	0.12608	0.16272	0.097874	0.028206	0.398
2232	206686_at	PDK1	0.72484	0.61036	0.30476	0.42008	<0.001
2233	206687_s_at	PTPN6	0.47164	0.51469	0.56234	-0.0907	0.745
2234	206688_s_at	CPSF4	0.60658	0.61987	0.58525	0.02133	0.434
2235	206693_at	IL7	0.77942	0.7073	0.72228	0.05714	0.228
2236	206704_at	CLCN5	0.052334	0.086424	0.11412	-0.061786	0.624
2237	206729_at	TNFRSF8	0.35436	0.66188	0.48552	-0.13116	0.776
2238	206734_at	JRKL	0.50714	0.42128	0.55959	-0.05245	0.733
2239	206752_s_at	DFFB	0.1704	0.11093	0.19368	-0.02328	0.571
2240	206756_at	CHST7	0.27776	0.23686	0.33169	-0.05393	0.705
2241	206759_at	FCER2	0.50632	0.62619	0.6453	-0.13898	0.841
2242	206766_at	ITGA10	0.45535	0.30727	0.25588	0.19947	0.134
2243	206788_s_at	CBFB	0.67116	0.76355	0.69401	-0.02285	0.624
2244	206789_s_at	POU2F1	0.34097	0.27654	0.28851	0.05246	0.376
2245	206790_s_at	NDUFB1	0.82417	0.80378	0.80907	0.0151	0.418
2246	206809_s_at	HNRNPA3	0.76274	0.69502	0.47747	0.28527	0.007
2247	206825_at	OXTR	0.58701	0.73865	0.71969	-0.13268	0.916
2248	206828_at	TXK	0.13949	0.11268	0.20039	-0.0609	0.62
2249	206833_s_at	ACYP2	0.52567	0.58582	0.56895	-0.04328	0.608
2250	206845_s_at	RNF40	0.64755	0.69531	0.63173	0.01582	0.436
2251	206848_at	FAM36A	0.38589	0.30139	0.33077	0.05512	0.302
2252	206855_s_at	HYAL2	0.31927	0.48461	0.36678	-0.04751	0.622
2253	206860_s_at	MIOS	0.75157	0.51691	0.59849	0.15308	0.061
2254	206861_s_at	CGGBP1	0.39125	0.29481	0.1578	0.23345	0.059
2255	206875_s_at	SLK	0.50275	0.32202	0.51842	-0.01567	0.555
2256	206907_at	TNFSF9	0.32631	0.085261	0.21752	0.10879	0.178
2257	206918_s_at	CPNE1	0.14839	0.32254	0.19177	-0.04338	0.622
2258	206925_at	ST8SIA4	0.52633	0.42209	0.57057	-0.04424	0.696
2259	206928_at	ZNF124	0.09301	0.12874	0.23172	-0.13871	0.809
2260	206931_at	ZNF141	0.57283	0.55964	0.64987	-0.07704	0.764
2261	206942_s_at	PMCH	0.55818	0.60297	0.43755	0.12063	0.389
2262	206949_s_at	RUSC1	0.74084	0.68177	0.72402	0.01682	0.425
2263	206958_s_at	UPF3A	0.51182	0.48088	0.43834	0.07348	0.345
2264	206975_at	LTA	0.51011	0.67016	0.63402	-0.12391	0.847
2265	206976_s_at	HSPH1	0.73346	0.79296	0.58168	0.15178	0.051
2266	206983_at	CCR6	0.27547	0.40181	0.39684	-0.12137	0.729
2267	206992_s_at	ATP5S	0.53013	0.43986	0.48853	0.0416	0.394
2268	207000_s_at	PPP3CC	0.45361	0.39087	0.30366	0.14995	0.046
2269	207002_s_at	PLAGL1	0.42739	0.48209	0.37804	0.04935	0.41
2270	207038_at	SLC16A6	0.53589	0.51026	0.34714	0.18875	0.023
2271	207040_s_at	ST13	0.65152	0.68738	0.68072	-0.0292	0.674
2272	207064_s_at	AOC2	0.17918	0.227	0.13208	0.0471	0.373
2273	207071_s_at	ACO1	0.44063	0.45761	0.51881	-0.07818	0.763
2274	207079_s_at	MED6	0.54818	0.64589	0.45994	0.08824	0.198
2275	207088_s_at	SLC25A11	0.75107	0.70939	0.78226	-0.03119	0.659
2276	207096_at	SAA4	0.23597	0.34354	0.32843	-0.09246	0.747

2277	207103_at	KCND2	0.32671	0.43957	0.49223	-0.16552	0.835
2278	207113_s_at	TNF	0.36931	0.56093	0.38377	-0.01446	0.485
2279	207121_s_at	MAPK6	0.77799	0.69568	0.7344	0.04359	0.174
2280	207124_s_at	GNB5	0.21737	0.33754	0.36853	-0.15116	0.852
2281	207127_s_at	HNRNPH3	0.59165	0.68754	0.28063	0.31102	0.001
2282	207152_at	NTRK2	0.4391	0.35675	0.30981	0.12929	0.224
2283	207153_s_at	GLMN	0.60326	0.64575	0.60226	0.001	0.47
2284	207157_s_at	GNG5	0.87443	0.87741	0.89351	-0.01908	0.62
2285	207158_at	APOBEC1	0.17038	0.35529	0.34241	-0.17203	0.844
2286	207160_at	IL12A	0.11826	0.43979	0.2313	-0.11304	0.67
2287	207163_s_at	AKT1	0.68574	0.73805	0.58192	0.10382	0.172
2288	207170_s_at	LETMD1	0.58744	0.6999	0.64251	-0.05507	0.704
2289	207176_s_at	CD80	0.67868	0.53969	0.51238	0.1663	0.021
2290	207178_s_at	FRK	0.51955	0.34625	0.46101	0.05854	0.341
2291	207181_s_at	CASP7	0.48059	0.51443	0.52786	-0.04727	0.648
2292	207186_s_at	BPTF	0.50243	0.36479	0.47374	0.02869	0.425
2293	207196_s_at	TNIP1	0.77408	0.87505	0.78567	-0.01159	0.603
2294	207198_s_at	LIMS1	0.17082	0.38686	0.28673	-0.11591	0.788
2295	207219_at	ZNF643	0.0018704	0.14194	0.0577	-0.0558296	0.608
2296	207229_at	KLRA1	0.21013	0.20922	0.24655	-0.03642	0.592
2297	207234_at	RFX3	0.12397	0.16276	0.15964	-0.03567	0.598
2298	207243_s_at	CALM2	0.81312	0.86216	0.87416	-0.06104	0.89
2299	207245_at	UGT2B17	0.034476	0.13459	0.095823	-0.061347	0.599
2300	207283_at	RPL23AP32	0.41913	0.40549	0.42909	-0.00996	0.516
2301	207305_s_at	KIAA1012	0.72974	0.68237	0.73734	-0.0076	0.569
2302	207315_at	CD226	0.30224	0.50808	0.42711	-0.12487	0.842
2303	207332_s_at	TFRC	0.5665	0.51102	0.61272	-0.04622	0.723
2304	207338_s_at	ZNF200	0.36417	0.18242	0.12391	0.24026	0.034
2305	207339_s_at	LTB	0.59539	0.67923	0.65342	-0.05803	0.689
2306	207347_at	ERCC6	0.32722	0.46051	0.38906	-0.06184	0.693
2307	207350_s_at	VAMP4	0.31752	0.3567	0.36429	-0.04677	0.61
2308	207375_s_at	IL15RA	0.085819	0.41984	0.36591	-0.280091	0.929
2309	207386_at	CYP7B1	0.042615	0.1984	0.26891	-0.226295	0.945
2310	207389_at	GP1BA	0.23485	0.30573	0.22682	0.00803	0.49
2311	207394_at	ZNF137	0.018364	0.15669	0.0034187	0.0149453	0.464
2312	207405_s_at	RAD17	0.79107	0.69998	0.75843	0.03264	0.399
2313	207408_at	SLC22A14	0.16486	0.11775	0.091185	0.073675	0.329
2314	207417_s_at	ZNF177	0.078105	0.040023	0.019188	0.058917	0.348
2315	207426_s_at	TNFSF4	0.39507	0.58715	0.45022	-0.05515	0.647
2316	207431_s_at	DEGS1	0.19605	0.46102	0.44488	-0.24883	0.966
2317	207435_s_at	SRRM2	0.58273	0.6591	0.65768	-0.07495	0.692
2318	207438_s_at	SNUPN	0.17826	0.44418	0.36265	-0.18439	0.885
2319	207440_at	SLC35A2	0.010423	0.055149	0.089131	-0.078708	0.669
2320	207445_s_at	CCR9	0.14819	0.21164	0.28738	-0.13919	0.804
2321	207469_s_at	PIR	0.45899	0.58973	0.51636	-0.05737	0.706
2322	207480_s_at	MEIS2	0.018311	0.26995	0.30441	-0.286099	0.983
2323	207508_at	ATP5G3	0.8882	0.76096	0.84329	0.04491	0.104
2324	207513_s_at	ZNF189	0.13062	0.46911	0.43315	-0.30253	0.996
2325	207515_s_at	POLR1C	0.73658	0.71692	0.70135	0.03523	0.28
2326	207519_at	SLC6A4	0.73979	0.66789	0.60048	0.13931	0.095
2327	207536_s_at	TNFRSF9	0.34348	0.56161	0.38951	-0.04603	0.623
2328	207540_s_at	SYK	0.25924	0.36747	0.42007	-0.16083	0.881
2329	207541_s_at	EXOSC10	0.25774	0.324	0.30747	-0.04973	0.623

2330	207543_s_at	P4HA1	0.8307	0.76929	0.53326	0.29744	0.002
2331	207545_s_at	NUMB	0.84171	0.7364	0.79555	0.04616	0.179
2332	207551_s_at	MSL3	0.41496	0.62817	0.54486	-0.1299	0.894
2333	207559_s_at	ZMYM3	0.52003	0.35941	0.41462	0.10541	0.166
2334	207563_s_at	OGT	0.62433	0.70166	0.77036	-0.14603	0.957
2335	207568_at	CHRNA6	0.27729	0.2701	0.2086	0.06869	0.276
2336	207583_at	ABCD2	0.335	0.33517	0.40617	-0.07117	0.716
2337	207585_s_at	RPL36AL	0.82456	0.82221	0.83377	-0.00921	0.576
2338	207597_at	ADAM18	0.26108	0.21767	0.18794	0.07314	0.422
2339	207606_s_at	ARHGAP12	0.48083	0.57129	0.5656	-0.08477	0.841
2340	207610_s_at	EMR2	0.24197	0.17084	0.23538	0.00659	0.481
2341	207614_s_at	CUL1	0.67166	0.64984	0.61112	0.06054	0.317
2342	207618_s_at	BCS1L	0.50199	0.65995	0.77995	-0.27796	0.995
2343	207621_s_at	PEMT	0.5545	0.62975	0.58689	-0.03239	0.616
2344	207622_s_at	ABCF2	0.72879	0.66022	0.67509	0.0537	0.359
2345	207624_s_at	RPGR	0.15139	0.2385	0.39571	-0.24432	0.984
2346	207627_s_at	TFCP2	0.66529	0.48575	0.55169	0.1136	0.103
2347	207628_s_at	WBSCR22	0.65712	0.82365	0.67601	-0.01889	0.604
2348	207641_at	TNFRSF13B	0.20737	0.38958	0.33969	-0.13232	0.859
2349	207655_s_at	BLNK	0.62585	0.55395	0.56967	0.05618	0.34
2350	207665_at	ADAM21	0.18123	0.38813	0.19757	-0.01634	0.543
2351	207677_s_at	NCF4	0.00021269	0.2556	0.16132	-0.16110731	0.868
2352	207684_at	TBX6	0.24895	0.18446	0.26973	-0.02078	0.584
2353	207687_at	INHBC	0.37085	0.40627	0.38992	-0.01907	0.551
2354	207707_s_at	SEC13	0.22165	0.44536	0.42178	-0.20013	0.91
2355	207713_s_at	RBCK1	0.31057	0.4442	0.16847	0.1421	0.235
2356	207727_s_at	MUTYH	0.33469	0.11999	0.32193	0.01276	0.466
2357	207734_at	LAX1	0.27001	0.3979	0.36041	-0.0904	0.719
2358	207735_at	RNF125	0.3139	0.39421	0.36522	-0.05132	0.655
2359	207740_s_at	NUP62	0.80884	0.80565	0.81417	-0.00533	0.56
2360	207746_at	POLQ	0.33802	0.47627	0.39883	-0.06081	0.648
2361	207753_at	ZNF304	0.70312	0.69363	0.64053	0.06259	0.279
2362	207761_s_at	METTL7A	0.35042	0.32955	0.25915	0.09127	0.301
2363	207777_s_at	SP140	0.43182	0.67799	0.57598	-0.14416	0.892
2364	207780_at	CYLC2	0.29575	0.19098	0.18745	0.1083	0.295
2365	207785_s_at	RBPJ	0.71646	0.72017	0.68804	0.02842	0.429
2366	207805_s_at	PSMD9	0.66237	0.77837	0.71565	-0.05328	0.676
2367	207809_s_at	ATP6AP1	0.74253	0.7316	0.71093	0.0316	0.341
2368	207812_s_at	GORASP2	0.66913	0.61696	0.58763	0.0815	0.181
2369	207813_s_at	FDXR	0.55095	0.70665	0.54798	0.00297	0.509
2370	207826_s_at	ID3	0.65576	0.64674	0.63582	0.01994	0.45
2371	207830_s_at	PPP1R8	0.44075	0.33949	0.48883	-0.04808	0.65
2372	207842_s_at	CASC3	0.52186	0.72838	0.72026	-0.1984	0.927
2373	207845_s_at	ANAPC10	0.80289	0.70144	0.77362	0.02927	0.346
2374	207855_s_at	CLCC1	0.60868	0.52877	0.58467	0.02401	0.39
2375	207856_s_at	LOC150776	0.39606	0.52	0.44723	-0.05117	0.671
2376	207861_at	CCL22	0.35963	0.52339	0.46745	-0.10782	0.846
2377	207871_s_at	ST7	0.48258	0.52937	0.40233	0.08025	0.308
2378	207891_s_at	HAUS7	0.65722	0.51937	0.3999	0.25732	0.018
2379	207900_at	CCL17	0.45059	0.44282	0.43762	0.01297	0.465
2380	207904_s_at	LNPEP	0.22464	0.41787	0.50587	-0.28123	0.979
2381	207907_at	TNFSF14	0.42517	0.58257	0.53982	-0.11465	0.815
2382	207908_at	KRT2	0.083524	0.30512	0.23512	-0.151596	0.725

2383	207922_s_at	MAEA	0.67419	0.72666	0.72012	-0.04593	0.69
2384	207945_s_at	CSNK1D	0.77672	0.69267	0.63471	0.14201	0.08
2385	207951_at	CSN2	0.376	0.34555	0.33118	0.04482	0.409
2386	207957_s_at	PRKCB	0.69579	0.68996	0.64485	0.05094	0.303
2387	207966_s_at	GLG1	0.61996	0.66161	0.64797	-0.02801	0.586
2388	207980_s_at	CITED2	0.35573	0.36555	0.31952	0.03621	0.403
2389	207996_s_at	C18orf1	0.45469	0.3903	0.56672	-0.11203	0.779
2390	208003_s_at	NFAT5	0.52756	0.62726	0.59163	-0.06407	0.681
2391	208018_s_at	HCK	0.35049	0.51624	0.52374	-0.17325	0.915
2392	208021_s_at	RFC1	0.57067	0.46732	0.42026	0.15041	0.169
2393	208024_s_at	DGCR6	0.43457	0.49142	0.47534	-0.04077	0.616
2394	208035_at	GRM6	0.31246	0.38491	0.41191	-0.09945	0.77
2395	208037_s_at	MADCAM1	0.10964	0.25581	0.031804	0.077836	0.393
2396	208039_at	SLC9A2	0.50598	0.40273	0.31017	0.19581	0.074
2397	208047_s_at	NAB1	0.67692	0.71536	0.72092	-0.044	0.801
2398	208050_s_at	CASP2	0.68408	0.58591	0.38792	0.29616	0.007
2399	208051_s_at	PAIP1	0.70138	0.4878	0.66767	0.03371	0.337
2400	208055_s_at	HERC4	0.68223	0.51578	0.67413	0.0081	0.464
2401	208056_s_at	CBFA2T3	0.26291	0.26254	0.24413	0.01878	0.471
2402	208066_s_at	GTF2B	0.48698	0.53432	0.45225	0.03473	0.401
2403	208070_s_at	REV3L	0.6896	0.62942	0.64315	0.04645	0.313
2404	208072_s_at	DGKD	0.55094	0.4726	0.37669	0.17425	0.056
2405	208074_s_at	AP2S1	0.80699	0.85975	0.87244	-0.06545	0.744
2406	208089_s_at	TDRD3	0.45049	0.45913	0.437	0.01349	0.435
2407	208091_s_at	ECOP	0.5261	0.65872	0.55615	-0.03005	0.643
2408	208093_s_at	NDEL1	0.26616	0.17642	0.12595	0.14021	0.255
2409	208095_s_at	SRP72	0.46829	0.67228	0.49443	-0.02614	0.593
2410	208101_s_at	URM1	0.44223	0.67333	0.69951	-0.25728	0.989
2411	208104_s_at	TSC22D4	0.28609	0.20022	0.10872	0.17737	0.093
2412	208107_s_at	LOC81691	0.39307	0.40397	0.43207	-0.039	0.601
2413	208117_s_at	LAS1L	0.68094	0.5776	0.47761	0.20333	0.065
2414	208119_s_at	ZNF93	0.1158	0.07071	0.29707	-0.18127	0.891
2415	208152_s_at	DDX21	0.79659	0.69297	0.59109	0.2055	0.005
2416	208154_at	LOC51336	0.1301	0.28205	0.30891	-0.17881	0.91
2417	208184_s_at	TRAPPC10	0.3297	0.45116	0.4554	-0.1257	0.695
2418	208190_s_at	LSR	0.15837	0.3387	0.15744	0.00093	0.526
2419	208195_at	TTN	0.085628	0.13746	0.13159	-0.045962	0.623
2420	208217_at	GABRR2	0.11679	0.27222	0.37964	-0.26285	0.898
2421	208249_s_at	TGDS	0.23136	0.29471	0.11377	0.11759	0.063
2422	208250_s_at	DMBT1	0.29959	0.163	0.27715	0.02244	0.482
2423	208270_s_at	RNPEP	0.5522	0.6952	0.58225	-0.03005	0.621
2424	208290_s_at	EIF5	0.3345	0.36492	0.24721	0.08729	0.25
2425	208302_at	HMHB1	0.29964	0.30743	0.20934	0.0903	0.478
2426	208309_s_at	MALT1	0.46603	0.63037	0.54236	-0.07633	0.712
2427	208319_s_at	RBM3	0.81957	0.81536	0.85137	-0.0318	0.79
2428	208328_s_at	MEF2A	0.70045	0.64449	0.64657	0.05388	0.268
2429	208336_s_at	GPSN2	0.66775	0.77399	0.7761	-0.10835	0.913
2430	208368_s_at	BRCA2	0.57079	0.65974	0.51039	0.0604	0.255
2431	208382_s_at	DMC1	0.13403	0.32294	0.13337	0.00066	0.503
2432	208398_s_at	TBPL1	0.78081	0.73182	0.70113	0.07968	0.195
2433	208405_s_at	CD164	0.48596	0.54227	0.53782	-0.05186	0.668
2434	208424_s_at	CIAPIN1	0.58529	0.37369	0.49721	0.08808	0.158
2435	208433_s_at	LRP8	0.34313	0.45927	0.52252	-0.17939	0.903

2436	208436_s_at	IRF7	0.63365	0.69643	0.63128	0.00237	0.53
2437	208438_s_at	FGR	0.32567	0.38838	0.36951	-0.04384	0.625
2438	208442_s_at	ATM	0.7032	0.65653	0.63745	0.06575	0.329
2439	208447_s_at	PRPS1	0.65319	0.68768	0.62671	0.02648	0.397
2440	208453_s_at	XPNPEP1	0.78118	0.6678	0.59991	0.18127	0.015
2441	208498_s_at	AMY1A	0.34448	0.12357	0.18789	0.15659	0.137
2442	208499_s_at	DNAJC3	0.36886	0.23504	0.2587	0.11016	0.244
2443	208503_s_at	GATAD1	0.54826	0.41124	0.45603	0.09223	0.226
2444	208506_at	HIST1H3F	0.51006	0.34479	0.3531	0.15696	0.094
2445	208511_at	PTTG3	0.62132	0.77011	0.65717	-0.03585	0.617
2446	208524_at	GPR15	0.46738	0.57519	0.6927	-0.22532	0.945
2447	208578_at	SCN10A	0.42913	0.17389	0.25291	0.17622	0.156
2448	208588_at	FKSG2	0.36998	0.36929	0.43333	-0.06335	0.668
2449	208612_at	PDIA3	0.5676	0.63081	0.70347	-0.13587	0.894
2450	208616_s_at	PTP4A2	0.73196	0.74356	0.61801	0.11395	0.234
2451	208619_at	DDB1	0.79224	0.70221	0.65114	0.1411	0.025
2452	208620_at	PCBP1	0.64698	0.76401	0.74901	-0.10203	0.873
2453	208623_s_at	EZR	0.64544	0.65258	0.58608	0.05936	0.277
2454	208624_s_at	EIF4G1	0.57505	0.62144	0.63659	-0.06154	0.679
2455	208627_s_at	YBX1	0.91438	0.83132	0.87646	0.03792	0.174
2456	208629_s_at	HADHA	0.51357	0.64303	0.54252	-0.02895	0.623
2457	208632_at	RNF10	0.33594	0.36238	0.30864	0.0273	0.447
2458	208636_at	ACTN1	0.40423	0.5222	0.50859	-0.10436	0.756
2459	208641_s_at	RAC1	0.81193	0.85848	0.8476	-0.03567	0.666
2460	208642_s_at	XRCC5	0.84562	0.81689	0.84229	0.00333	0.478
2461	208644_at	PARP1	0.75105	0.69613	0.66583	0.08522	0.207
2462	208645_s_at	RPS14	0.93805	0.93856	0.94325	-0.0052	0.666
2463	208647_at	FDFT1	0.51619	0.6937	0.55965	-0.04346	0.668
2464	208649_s_at	VCP	0.85896	0.81966	0.79963	0.05933	0.134
2465	208652_at	PPP2CA	0.66568	0.59724	0.53374	0.13194	0.052
2466	208655_at	CCNI	0.64409	0.72824	0.67283	-0.02874	0.59
2467	208659_at	CLIC1	0.85882	0.79536	0.87256	-0.01374	0.606
2468	208660_at	CS	0.61652	0.7116	0.73098	-0.11446	0.867
2469	208670_s_at	EID1	0.52254	0.59332	0.53628	-0.01374	0.535
2470	208671_at	SERINC1	0.82462	0.7286	0.73914	0.08548	0.139
2471	208675_s_at	DDOST	0.54971	0.50912	0.57129	-0.02158	0.584
2472	208676_s_at	PA2G4	0.61577	0.76723	0.76069	-0.14492	0.926
2473	208677_s_at	BSG	0.44139	0.61519	0.54902	-0.10763	0.817
2474	208678_at	ATP6V1E1	0.64846	0.52736	0.7378	-0.08934	0.834
2475	208679_s_at	ARPC2	0.55938	0.711	0.78312	-0.22374	0.968
2476	208680_at	PRDX1	0.79876	0.85626	0.89204	-0.09328	0.972
2477	208684_at	COPA	0.68847	0.75318	0.72347	-0.035	0.632
2478	208686_s_at	BRD2	0.27622	0.61304	0.41829	-0.14207	0.707
2479	208689_s_at	RPN2	0.60211	0.6745	0.64787	-0.04576	0.678
2480	208690_s_at	PDLIM1	0.71111	0.75624	0.8432	-0.13209	0.929
2481	208692_at	RPS3	0.92507	0.9377	0.93761	-0.01254	0.754
2482	208693_s_at	GARS	0.48244	0.57585	0.41849	0.06395	0.296
2483	208695_s_at	RPL39	0.9268	0.91588	0.9201	0.0067	0.339
2484	208696_at	CCT5	0.79397	0.79566	0.72959	0.06438	0.274
2485	208697_s_at	EIF3E	0.79587	0.84843	0.87548	-0.07961	0.932
2486	208698_s_at	NONO	0.82456	0.7975	0.883	-0.05844	0.965
2487	208700_s_at	TKT	0.71054	0.74347	0.82857	-0.11803	0.901
2488	208709_s_at	NRD1	0.64222	0.69782	0.69782	-0.0556	0.73

2489	208713_at	HNRNPUL1	0.25977	0.34668	0.28557	-0.0258	0.592
2490	208714_at	NDUFV1	0.81816	0.67446	0.71153	0.10663	0.045
2491	208715_at	TMCO1	0.47408	0.33984	0.32412	0.14996	0.181
2492	208717_at	OXA1L	0.42936	0.46883	0.53169	-0.10233	0.781
2493	208720_s_at	RBM39	0.76732	0.62583	0.53862	0.2287	0.022
2494	208721_s_at	ANAPC5	0.68332	0.71447	0.6887	-0.00538	0.555
2495	208723_at	USP11	0.69116	0.66835	0.60275	0.08841	0.124
2496	208724_s_at	RAB1A	0.47832	0.51064	0.60386	-0.12554	0.774
2497	208726_s_at	EIF2S2	0.7901	0.81324	0.82559	-0.03549	0.732
2498	208736_at	ARPC3	0.85619	0.86037	0.89436	-0.03817	0.748
2499	208737_at	ATP6V1G1	0.716	0.64837	0.74131	-0.02531	0.586
2500	208741_at	SAP18	0.23002	0.43338	0.28647	-0.05645	0.624
2501	208745_at	ATP5L	0.80791	0.80586	0.77754	0.03037	0.289
2502	208753_s_at	NAP1L1	0.59527	0.64151	0.60841	-0.01314	0.567
2503	208756_at	EIF3I	0.53789	0.67544	0.60168	-0.06379	0.738
2504	208757_at	TMED9	0.44267	0.69285	0.58383	-0.14116	0.894
2505	208758_at	ATIC	0.58564	0.67295	0.72347	-0.13783	0.874
2506	208759_at	NCSTN	0.37677	0.43485	0.28678	0.08999	0.328
2507	208760_at	UBE2I	0.57129	0.66864	0.57558	-0.00429	0.533
2508	208762_at	SUMO1	0.8623	0.82998	0.74681	0.11549	0.031
2509	208763_s_at	TSC22D3	0.70944	0.68906	0.59129	0.11815	0.031
2510	208765_s_at	HNRNPR	0.78213	0.69986	0.76484	0.01729	0.396
2511	208771_s_at	LTA4H	0.53594	0.53	0.4902	0.04574	0.394
2512	208777_s_at	PSMD11	0.57099	0.58947	0.49864	0.07235	0.311
2513	208784_s_at	KLHDC3	0.61682	0.56704	0.52972	0.0871	0.263
2514	208785_s_at	MAP1LC3B	0.41668	0.51221	0.52548	-0.1088	0.812
2515	208787_at	MRPL3	0.63614	0.76962	0.81581	-0.17967	0.989
2516	208796_s_at	CCNG1	0.47788	0.48838	0.36246	0.11542	0.193
2517	208799_at	PSMB5	0.765	0.78579	0.81869	-0.05369	0.72
2518	208808_s_at	HMGB2	0.72926	0.79386	0.72863	0.00063	0.509
2519	208813_at	GOT1	0.82603	0.59091	0.68348	0.14255	0.018
2520	208819_at	RAB8A	0.62899	0.69332	0.73274	-0.10375	0.771
2521	208820_at	PTK2	0.57703	0.7362	0.7161	-0.13907	0.969
2522	208821_at	SNRPB	0.81005	0.86188	0.88099	-0.07094	0.906
2523	208822_s_at	DAP3	0.34932	0.36617	0.16788	0.18144	0.085
2524	208827_at	PSMB6	0.8117	0.86145	0.90462	-0.09292	0.997
2525	208828_at	POLE3	0.58187	0.4828	0.48817	0.0937	0.164
2526	208829_at	TAPBP	0.84462	0.83996	0.78575	0.05887	0.138
2527	208830_s_at	SUPT6H	0.50469	0.41638	0.43713	0.06756	0.29
2528	208833_s_at	ATXN10	0.34671	0.48189	0.5492	-0.20249	0.978
2529	208836_at	ATP1B3	0.52114	0.63686	0.57543	-0.05429	0.678
2530	208837_at	TMED3	0.031163	0.0286	0.12435	-0.093187	0.767
2531	208839_s_at	CAND1	0.8599	0.88129	0.78677	0.07313	0.132
2532	208841_s_at	G3BP2	0.62437	0.67565	0.71676	-0.09239	0.789
2533	208845_at	VDAC3	0.67631	0.69226	0.72864	-0.05233	0.784
2534	208847_s_at	ADH5	0.36363	0.48368	0.37401	-0.01038	0.553
2535	208849_at	EEF1A1	0.645	0.63399	0.66407	-0.01907	0.557
2536	208853_s_at	CANX	0.82459	0.88218	0.8667	-0.04211	0.728
2537	208854_s_at	STK24	0.67446	0.65557	0.65955	0.01491	0.447
2538	208857_s_at	PCMT1	0.88171	0.80278	0.6977	0.18401	0.006
2539	208858_s_at	FAM62A	0.51165	0.63759	0.58493	-0.07328	0.719
2540	208861_s_at	ATRX	0.5279	0.61753	0.61252	-0.08462	0.805
2541	208862_s_at	CTNND1	0.17076	0.3209	0.26452	-0.09376	0.756

2542	208872_s_at	REEP5	0.58598	0.74686	0.61703	-0.03105	0.589
2543	208876_s_at	PAK2	0.30684	0.50611	0.64321	-0.33637	0.998
2544	208880_s_at	PRPF6	0.29629	0.32842	0.33551	-0.03922	0.626
2545	208883_at	UBR5	0.56359	0.62016	0.50272	0.06087	0.321
2546	208885_at	LCP1	0.87533	0.84902	0.84348	0.03185	0.184
2547	208886_at	H1F0	0.16432	0.32834	0.30803	-0.14371	0.84
2548	208887_at	EIF3G	0.77754	0.73644	0.79873	-0.02119	0.705
2549	208894_at	HLA-DRA	0.8352	0.8998	0.87681	-0.04161	0.869
2550	208897_s_at	DDX18	0.82806	0.85092	0.81817	0.00989	0.458
2551	208900_s_at	TOP1	0.42397	0.31439	0.37324	0.05073	0.376
2552	208905_at	CYCS	0.61264	0.75554	0.84094	-0.2283	0.999
2553	208906_at	BSCL2	0.14125	0.14185	0.22587	-0.08462	0.788
2554	208909_at	UQCRFS1	0.84171	0.89617	0.90356	-0.06185	0.954
2555	208910_s_at	C1QBP	0.62127	0.73538	0.69298	-0.07171	0.738
2556	208911_s_at	PDHB	0.29439	0.44812	0.45058	-0.15619	0.857
2557	208912_s_at	CNP	0.18694	0.34039	0.33473	-0.14779	0.858
2558	208916_at	SLC1A5	0.65454	0.53837	0.35899	0.29555	<0.001
2559	208920_at	SRI	0.48498	0.44495	0.4646	0.02038	0.463
2560	208922_s_at	NXF1	0.74948	0.60106	0.7512	-0.00172	0.496
2561	208923_at	CYFIP1	0.70575	0.72109	0.58604	0.11971	0.187
2562	208924_at	RNF11	0.80844	0.70964	0.70185	0.10659	0.043
2563	208925_at	CLDND1	0.65008	0.56275	0.69829	-0.04821	0.693
2564	208926_at	NEU1	0.50976	0.42275	0.54117	-0.03141	0.592
2565	208927_at	SPOP	0.53979	0.41052	0.5486	-0.00881	0.531
2566	208932_at	PPP4C	0.41526	0.20864	0.3893	0.02596	0.441
2567	208938_at	PRCC	0.47344	0.55058	0.58061	-0.10717	0.752
2568	208941_s_at	SEPHS1	0.61097	0.68135	0.59219	0.01878	0.43
2569	208942_s_at	SEC62	0.83733	0.81057	0.78827	0.04906	0.287
2570	208944_at	TGFBR2	0.20967	0.35156	0.33511	-0.12544	0.815
2571	208946_s_at	BECN1	0.70049	0.63031	0.47713	0.22336	0.028
2572	208949_s_at	LGALS3	0.35781	0.46544	0.47639	-0.11858	0.776
2573	208954_s_at	LARP5	0.43231	0.38012	0.1818	0.25051	0.028
2574	208959_s_at	ERP44	0.50802	0.69183	0.60212	-0.0941	0.752
2575	208964_s_at	FADS1	0.23072	0.295	0.21947	0.01125	0.494
2576	208965_s_at	IFI16	0.49202	0.66986	0.61399	-0.12197	0.821
2577	208969_at	NDUFA9	0.50882	0.69099	0.72535	-0.21653	0.964
2578	208971_at	UROD	0.45648	0.56994	0.66199	-0.20551	0.947
2579	208972_s_at	ATP5G1	0.87542	0.87019	0.85044	0.02498	0.25
2580	208973_at	ERI3	0.55071	0.65387	0.6746	-0.12389	0.883
2581	208979_at	NCOA6	0.67404	0.76521	0.74449	-0.07045	0.781
2582	208980_s_at	UBC	0.85617	0.84692	0.78966	0.06651	0.146
2583	208985_s_at	EIF3J	0.8131	0.76793	0.70329	0.10981	0.161
2584	208986_at	TCF12	0.5826	0.64532	0.68702	-0.10442	0.852
2585	208991_at	STAT3	0.71545	0.7543	0.83825	-0.1228	0.888
2586	208995_s_at	PPIG	0.7531	0.77365	0.72128	0.03182	0.338
2587	208996_s_at	POLR2C	0.62403	0.5777	0.4144	0.20963	0.084
2588	208998_at	UCP2	0.64223	0.72911	0.7348	-0.09257	0.883
2589	208999_at	SEPT8	0.68435	0.71782	0.72758	-0.04323	0.641
2590	209001_s_at	ANAPC13	0.48489	0.33509	0.49462	-0.00973	0.517
2591	209004_s_at	FBXL5	0.83238	0.63843	0.80729	0.02509	0.329
2592	209007_s_at	C1orf63	0.75435	0.37712	0.3383	0.41605	<0.001
2593	209014_at	MAGED1	0.52538	0.57011	0.56354	-0.03816	0.629
2594	209015_s_at	DNAJB6	0.55957	0.66086	0.54565	0.01392	0.477

2595	209017_s_at	LONP1	0.84283	0.83679	0.74438	0.09845	0.141
2596	209020_at	C20orf111	0.51386	0.43659	0.41924	0.09462	0.204
2597	209023_s_at	STAG2	0.81271	0.69229	0.84062	-0.02791	0.69
2598	209028_s_at	ABI1	0.82054	0.81587	0.76733	0.05321	0.114
2599	209029_at	COPS7A	0.36882	0.22879	0.34608	0.02274	0.451
2600	209030_s_at	CADM1	0.031235	0.14032	0.2374	-0.206165	0.917
2601	209034_at	PNRC1	0.7173	0.65701	0.52661	0.19069	0.073
2602	209037_s_at	EHD1	0.42831	0.5205	0.37441	0.0539	0.299
2603	209040_s_at	PSMB8	0.47321	0.59302	0.53846	-0.06525	0.722
2604	209042_s_at	UBE2G2	0.51001	0.27644	0.32092	0.18909	0.02
2605	209043_at	PAPSS1	0.34224	0.4765	0.57146	-0.22922	0.904
2606	209046_s_at	GABARAPL2	0.73349	0.8226	0.78293	-0.04944	0.748
2607	209049_s_at	ZMYND8	0.1245	0.16651	0.062412	0.062088	0.418
2608	209050_s_at	RALGDS	0.85204	0.74781	0.64897	0.20307	0.001
2609	209055_s_at	CDC5L	0.70748	0.75592	0.78194	-0.07446	0.804
2610	209058_at	EDF1	0.88515	0.84403	0.91173	-0.02658	0.711
2611	209068_at	HNRPDL	0.76142	0.83243	0.80923	-0.04781	0.831
2612	209075_s_at	ISCU	0.70592	0.7173	0.61788	0.08804	0.281
2613	209076_s_at	WDR45L	0.27837	0.29966	0.055387	0.222983	0.071
2614	209077_at	TXN2	0.58814	0.63358	0.73145	-0.14331	0.962
2615	209083_at	CORO1A	0.72483	0.78645	0.78363	-0.0588	0.757
2616	209084_s_at	RAB28	0.55313	0.34423	0.53458	0.01855	0.42
2617	209088_s_at	UBN1	0.48466	0.51354	0.50766	-0.023	0.577
2618	209090_s_at	SH3GLB1	0.74578	0.73642	0.6637	0.08208	0.141
2619	209092_s_at	GLOD4	0.2694	0.3939	0.20118	0.06822	0.326
2620	209095_at	DLD	0.66049	0.73354	0.66265	-0.00216	0.507
2621	209096_at	UBE2V2	0.75181	0.63027	0.67947	0.07234	0.201
2622	209100_at	IFRD2	0.70816	0.65729	0.67415	0.03401	0.339
2623	209103_s_at	UFD1L	0.7622	0.81705	0.81013	-0.04793	0.745
2624	209104_s_at	NHP2	0.89255	0.88961	0.91632	-0.02377	0.769
2625	209106_at	NCOA1	0.31444	0.5802	0.4004	-0.08596	0.736
2626	209110_s_at	RGL2	0.44151	0.23464	0.2001	0.24141	0.026
2627	209111_at	RNF5	0.67418	0.66114	0.64793	0.02625	0.419
2628	209112_at	CDKN1B	0.75523	0.66861	0.67063	0.0846	0.222
2629	209113_s_at	HMG20B	0.41911	0.27513	0.219	0.20011	0.104
2630	209115_at	UBA3	0.74436	0.69157	0.74779	-0.00343	0.534
2631	209118_s_at	TUBA1A	0.52671	0.69796	0.66023	-0.13352	0.794
2632	209122_at	ADFP	0.35047	0.3875	0.36455	-0.01408	0.599
2633	209123_at	QDPR	0.015172	0.23086	0.29108	-0.275908	0.968
2634	209124_at	MYD88	0.497	0.48799	0.36871	0.12829	0.103
2635	209127_s_at	SART3	0.48236	0.50292	0.63644	-0.15408	0.889
2636	209130_at	SNAP23	0.82387	0.7742	0.8567	-0.03283	0.675
2637	209132_s_at	COMMD4	0.75594	0.7324	0.78295	-0.02701	0.615
2638	209135_at	ASPH	0.76863	0.65579	0.59611	0.17252	0.104
2639	209136_s_at	USP10	0.78066	0.78847	0.80093	-0.02027	0.682
2640	209139_s_at	PRKRA	0.31915	0.38972	0.20764	0.11151	0.191
2641	209142_s_at	UBE2G1	0.78519	0.78832	0.7649	0.02029	0.391
2642	209143_s_at	CLNS1A	0.73778	0.70129	0.60357	0.13421	0.047
2643	209146_at	SC4MOL	0.34201	0.34744	0.24938	0.09263	0.34
2644	209148_at	RXRB	0.1783	0.10879	0.26191	-0.08361	0.757
2645	209150_s_at	TM9SF1	0.66836	0.75023	0.79594	-0.12758	0.879
2646	209154_at	TAX1BP3	0.45413	0.64243	0.50724	-0.05311	0.747
2647	209155_s_at	NT5C2	0.44393	0.55373	0.43508	0.00885	0.452

2648	209157_at	DNAJA2	0.76121	0.7879	0.67328	0.08793	0.195
2649	209158_s_at	CYTH2	0.57269	0.45498	0.25524	0.31745	0.01
2650	209162_s_at	PRPF4	0.66608	0.75345	0.69331	-0.02723	0.617
2651	209165_at	AATF	0.59834	0.63489	0.69928	-0.10094	0.793
2652	209166_s_at	MAN2B1	0.78176	0.67149	0.66251	0.11925	0.078
2653	209171_at	ITPA	0.51241	0.58102	0.68878	-0.17637	0.914
2654	209174_s_at	QRICH1	0.69758	0.64902	0.58578	0.1118	0.139
2655	209175_at	SEC23IP	0.83439	0.85643	0.84607	-0.01168	0.555
2656	209177_at	NDUFAF3	0.77562	0.70319	0.75967	0.01595	0.403
2657	209178_at	DHX38	0.59465	0.51399	0.45567	0.13898	0.127
2658	209187_at	DR1	0.7103	0.68929	0.66552	0.04478	0.215
2659	209190_s_at	DIAPH1	0.67325	0.70337	0.68583	-0.01258	0.572
2660	209191_at	TUBB6	0.17833	0.36316	0.30167	-0.12334	0.78
2661	209193_at	PIM1	0.10513	0.15781	0.20896	-0.10383	0.69
2662	209194_at	CETN2	0.64703	0.69823	0.70853	-0.0615	0.684
2663	209196_at	WDR46	0.52702	0.48663	0.51635	0.01067	0.467
2664	209198_s_at	SYT11	0.097517	0.46109	0.39936	-0.301843	0.992
2665	209199_s_at	MEF2C	0.84376	0.70471	0.69023	0.15353	0.064
2666	209205_s_at	LMO4	0.55139	0.67238	0.59969	-0.0483	0.695
2667	209206_at	SEC22B	0.74879	0.61361	0.58062	0.16817	0.049
2668	209208_at	MPDU1	0.63165	0.64235	0.70277	-0.07112	0.737
2669	209210_s_at	FERMT2	0.23562	0.4743	0.49611	-0.26049	0.972
2670	209213_at	CBR1	0.36624	0.34586	0.40822	-0.04198	0.64
2671	209215_at	MFSD10	0.57005	0.66605	0.61767	-0.04762	0.674
2672	209217_s_at	WDR45	0.34991	0.24596	0.37285	-0.02294	0.579
2673	209219_at	RDBP	0.7748	0.69661	0.70503	0.06977	0.146
2674	209224_s_at	NDUFA2	0.91432	0.89153	0.90146	0.01286	0.397
2675	209229_s_at	SAPS1	0.54871	0.6151	0.47291	0.0758	0.232
2676	209231_s_at	DCTN5	0.56354	0.62176	0.61576	-0.05222	0.684
2677	209233_at	EMG1	0.8002	0.8221	0.833	-0.0328	0.667
2678	209239_at	NFKB1	0.83037	0.79154	0.74986	0.08051	0.151
2679	209249_s_at	GHITM	0.61398	0.66445	0.70685	-0.09287	0.76
2680	209252_at	HARS2	0.40332	0.27095	0.47462	-0.0713	0.758
2681	209254_at	KLHDC10	0.15153	0.030424	0.10705	0.04448	0.422
2682	209258_s_at	SMC3	0.42141	0.55588	0.59748	-0.17607	0.928
2683	209265_s_at	METTL3	0.61441	0.20696	0.4063	0.20811	0.067
2684	209267_s_at	SLC39A8	0.49589	0.61271	0.50985	-0.01396	0.542
2685	209268_at	VPS45	0.56091	0.49284	0.59283	-0.03192	0.606
2686	209276_s_at	GLRX	0.54319	0.73439	0.63744	-0.09425	0.761
2687	209277_at	TFPI2	0.39938	0.54859	0.47931	-0.07993	0.743
2688	209279_s_at	NSDHL	0.50371	0.52306	0.29326	0.21045	0.049
2689	209282_at	PRKD2	0.78428	0.60346	0.60946	0.17482	0.011
2690	209285_s_at	C3orf63	0.37749	0.45963	0.46271	-0.08522	0.747
2691	209287_s_at	CDC42EP3	0.31958	0.41751	0.58233	-0.26275	0.94
2692	209295_at	TNFRSF10B	0.88599	0.74153	0.57818	0.30781	0.001
2693	209300_s_at	NECAP1	0.38237	0.4296	0.64888	-0.26651	0.976
2694	209302_at	POLR2H	0.89074	0.8837	0.84495	0.04579	0.26
2695	209303_at	NDUFS4	0.78831	0.80434	0.84296	-0.05465	0.732
2696	209306_s_at	SWAP70	0.4469	0.65517	0.66187	-0.21497	0.972
2697	209308_s_at	BNIP2	0.83598	0.68086	0.65122	0.18476	0.06
2698	209310_s_at	CASP4	0.3206	0.37696	0.32263	-0.00203	0.504
2699	209311_at	BCL2L2	0.36574	0.033289	0.29104	0.0747	0.581
2700	209313_at	GPN1	0.43785	0.35801	0.36434	0.07351	0.32

2701	209316_s_at	HBS1L	0.48676	0.42651	0.4702	0.01656	0.466
2702	209321_s_at	ADCY3	0.49235	0.69226	0.5582	-0.06585	0.691
2703	209323_at	PRKRIR	0.58529	0.6203	0.54717	0.03812	0.355
2704	209324_s_at	RGS16	0.38022	0.30625	0.39349	-0.01327	0.598
2705	209331_s_at	MAX	0.45011	0.43789	0.51912	-0.06901	0.685
2706	209336_at	PWP2	0.80826	0.72875	0.60689	0.20137	0.002
2707	209339_at	SIAH2	0.38034	0.55379	0.46277	-0.08243	0.755
2708	209340_at	UAP1	0.40783	0.63919	0.57735	-0.16952	0.912
2709	209342_s_at	IKBKB	0.34044	0.46843	0.386	-0.04556	0.57
2710	209344_at	TPM4	0.2117	0.39786	0.43625	-0.22455	0.949
2711	209349_at	RAD50	0.098198	0.37331	0.32702	-0.228822	0.932
2712	209354_at	TNFRSF14	0.52056	0.3278	0.50551	0.01505	0.458
2713	209358_at	TAF11	0.62725	0.50159	0.39153	0.23572	0.039
2714	209363_s_at	MED21	0.48599	0.35217	0.37932	0.10667	0.177
2715	209367_at	STXBP2	0.62143	0.68581	0.62826	-0.00683	0.555
2716	209374_s_at	IGHM	0.03947	0.25086	0.21809	-0.17862	0.896
2717	209375_at	XPC	0.57523	0.73427	0.53027	0.04496	0.356
2718	209377_s_at	HMGN3	0.77236	0.78103	0.80166	-0.0293	0.691
2719	209378_s_at	KIAA1128	0.15552	0.31784	0.12208	0.03344	0.421
2720	209383_at	DDIT3	0.035322	0.29816	0.36164	-0.326318	0.973
2721	209385_s_at	PROSC	0.57051	0.54713	0.49257	0.07794	0.216
2722	209390_at	TSC1	0.29555	0.48871	0.46109	-0.16554	0.853
2723	209391_at	DPM2	0.82235	0.66708	0.78796	0.03439	0.282
2724	209392_at	ENPP2	0.63297	0.70572	0.73578	-0.10281	0.86
2725	209393_s_at	EIF4E2	0.69615	0.66742	0.68459	0.01156	0.453
2726	209394_at	ASMTL	0.24801	0.28014	0.33058	-0.08257	0.685
2727	209398_at	HIST1H1C	0.68839	0.73117	0.72773	-0.03934	0.671
2728	209406_at	BAG2	0.63455	0.66181	0.58946	0.04509	0.327
2729	209408_at	KIF2C	0.78209	0.76381	0.77029	0.0118	0.442
2730	209413_at	B4GALT2	0.19003	0.30185	0.38204	-0.19201	0.867
2731	209417_s_at	IFI35	0.63985	0.6415	0.68037	-0.04052	0.692
2732	209421_at	MSH2	0.80065	0.77936	0.67062	0.13003	0.021
2733	209430_at	BTAF1	0.83785	0.70639	0.77398	0.06387	0.095
2734	209433_s_at	PPAT	0.29393	0.5359	0.51738	-0.22345	0.939
2735	209435_s_at	ARHGEF2	0.39599	0.42067	0.30954	0.08645	0.338
2736	209438_at	PHKA2	0.098319	0.19231	0.31499	-0.216671	0.918
2737	209449_at	LSM2	0.84999	0.81001	0.79026	0.05973	0.15
2738	209450_at	OSGEP	0.22643	0.30108	0.27791	-0.05148	0.657
2739	209451_at	TANK	0.7657	0.51723	0.83409	-0.06839	0.735
2740	209452_s_at	VTI1B	0.76239	0.78874	0.7471	0.01529	0.393
2741	209456_s_at	FBXW11	0.69012	0.58516	0.6806	0.00952	0.48
2742	209457_at	DUSP5	0.75095	0.54728	0.57006	0.18089	0.004
2743	209459_s_at	ABAT	0.51602	0.59471	0.55318	-0.03716	0.609
2744	209463_s_at	TAF12	0.45702	0.44382	0.36052	0.0965	0.233
2745	209464_at	AURKB	0.74567	0.7266	0.69051	0.05516	0.279
2746	209467_s_at	MKNK1	0.51879	0.41779	0.47227	0.04652	0.344
2747	209472_at	CCBL2	0.50963	0.57743	0.52478	-0.01515	0.572
2748	209474_s_at	ENTPD1	0.56087	0.44701	0.50986	0.05101	0.33
2749	209475_at	USP15	0.39055	0.54418	0.56266	-0.17211	0.862
2750	209476_at	TMX1	0.50271	0.58362	0.63631	-0.1336	0.785
2751	209477_at	EMD	0.50754	0.71739	0.71216	-0.20462	0.98
2752	209478_at	STRA13	0.7061	0.8335	0.78777	-0.08167	0.789
2753	209479_at	CCDC28A	0.354	0.45378	0.32735	0.02665	0.432

2754	209481_at	SNRK	0.53153	0.51897	0.52698	0.00455	0.501
2755	209482_at	POP7	0.8633	0.85506	0.89987	-0.03657	0.704
2756	209484_s_at	NSL1	0.77363	0.59917	0.67882	0.09481	0.107
2757	209486_at	UTP3	0.58037	0.50588	0.28112	0.29925	0.04
2758	209497_s_at	RBM4B	0.41959	0.36807	0.5476	-0.12801	0.906
2759	209498_at	CEACAM1	0.53692	0.41544	0.25429	0.28263	0.006
2760	209503_s_at	PSMC5	0.7482	0.81765	0.79324	-0.04504	0.654
2761	209507_at	RPA3	0.85511	0.87859	0.86772	-0.01261	0.573
2762	209509_s_at	DPAGT1	0.5407	0.55972	0.41502	0.12568	0.192
2763	209510_at	RNF139	0.077247	0.024484	0.14362	-0.066373	0.704
2764	209511_at	POLR2F	0.70547	0.72111	0.79814	-0.09267	0.827
2765	209512_at	HSDL2	0.60873	0.65609	0.63154	-0.02281	0.611
2766	209514_s_at	RAB27A	0.42185	0.45041	0.43193	-0.01008	0.538
2767	209517_s_at	ASH2L	0.64108	0.67402	0.68266	-0.04158	0.622
2768	209520_s_at	NCBP1	0.696	0.6946	0.72238	-0.02638	0.59
2769	209523_at	TAF2	0.83263	0.79473	0.81485	0.01778	0.4
2770	209524_at	HDGFRP3	0.46203	0.44735	0.43185	0.03018	0.408
2771	209531_at	GSTZ1	0.54262	0.47032	0.47844	0.06418	0.312
2772	209536_s_at	EHD4	0.58925	0.42318	0.49536	0.09389	0.234
2773	209537_at	EXTL2	0.41875	0.415	0.33785	0.0809	0.25
2774	209538_at	ZNF32	0.28182	0.34134	0.49498	-0.21316	0.937
2775	209539_at	ARHGEF6	0.15025	0.32366	0.23182	-0.08157	0.7
2776	209545_s_at	RIPK2	0.30574	0.28023	0.28202	0.02372	0.451
2777	209553_at	VPS8	0.65326	0.57651	0.56578	0.08748	0.3
2778	209556_at	NCDN	0.26639	0.29054	0.29819	-0.0318	0.573
2779	209565_at	RNF113A	0.42071	0.30703	0.45465	-0.03394	0.63
2780	209566_at	INSIG2	0.74084	0.33713	0.27414	0.4667	<0.001
2781	209567_at	RRS1	0.69519	0.50697	0.48584	0.20935	0.034
2782	209568_s_at	RGL1	0.10212	0.31456	0.24321	-0.14109	0.839
2783	209571_at	CIR	0.43227	0.60065	0.58356	-0.15129	0.931
2784	209575_at	IL10RB	0.5708	0.64605	0.53839	0.03241	0.392
2785	209577_at	PCYT2	0.16403	0.44022	0.21182	-0.04779	0.598
2786	209583_s_at	CD200	0.20957	0.41183	0.45771	-0.24814	0.967
2787	209585_s_at	MINPP1	0.51807	0.53095	0.59209	-0.07402	0.732
2788	209593_s_at	TOR1B	0.38804	0.40806	0.3226	0.06544	0.33
2789	209595_at	GTF2F2	0.69051	0.56616	0.55835	0.13216	0.145
2790	209604_s_at	GATA3	0.34241	0.31954	0.35406	-0.01165	0.522
2791	209606_at	CYTIP	0.37504	0.4705	0.34473	0.03031	0.416
2792	209608_s_at	ACAT2	0.3218	0.55521	0.54594	-0.22414	0.951
2793	209609_s_at	MRPL9	0.73921	0.81799	0.68285	0.05636	0.264
2794	209615_s_at	PAK1	0.46005	0.38166	0.55049	-0.09044	0.702
2795	209619_at	CD74	0.87615	0.83405	0.84137	0.03478	0.255
2796	209620_s_at	ABCB7	0.25095	0.51197	0.51023	-0.25928	0.955
2797	209622_at	STK16	0.40927	0.35434	0.35629	0.05298	0.359
2798	209624_s_at	MCCC2	0.77277	0.7553	0.75824	0.01453	0.411
2799	209625_at	PIGH	0.26699	0.16959	0.22278	0.04421	0.391
2800	209627_s_at	OSBPL3	0.47554	0.68278	0.5204	-0.04486	0.656
2801	209628_at	NXT2	0.62717	0.62707	0.59504	0.03213	0.492
2802	209630_s_at	FBXW2	0.78491	0.72721	0.73951	0.0454	0.274
2803	209636_at	NFKB2	0.50834	0.6505	0.58671	-0.07837	0.706
2804	209645_s_at	ALDH1B1	0.17159	0.10421	0.17731	-0.00572	0.522
2805	209647_s_at	SOCS5	0.48212	0.59223	0.55719	-0.07507	0.799
2806	209652_s_at	PGF	0.0092209	0.17411	0.15712	-0.1478991	0.925

2807	209653_at	KPNA4	0.24058	0.40226	0.49839	-0.25781	0.931
2808	209654_at	KIAA0947	0.80661	0.83348	0.7806	0.02601	0.331
2809	209657_s_at	HSF2	0.6853	0.61917	0.48625	0.19905	0.009
2810	209659_s_at	CDC16	0.4411	0.28557	0.31819	0.12291	0.189
2811	209662_at	CETN3	0.82139	0.80121	0.73951	0.08188	0.172
2812	209665_at	CYB561D2	0.56924	0.47278	0.56913	0.00011	0.507
2813	209666_s_at	CHUK	0.8083	0.70702	0.75441	0.05389	0.317
2814	209667_at	CES2	0.351	0.12414	0.19546	0.15554	0.091
2815	209669_s_at	SERBP1	0.58847	0.64153	0.55407	0.0344	0.419
2816	209670_at	TRAC	0.45058	0.39142	0.40064	0.04994	0.405
2817	209674_at	CRY1	0.30411	0.41429	0.33053	-0.02642	0.57
2818	209678_s_at	PRKCI	0.31788	0.47741	0.42538	-0.1075	0.742
2819	209681_at	SLC19A2	0.42633	0.57561	0.61924	-0.19291	0.982
2820	209682_at	CBLB	0.54543	0.72067	0.61945	-0.07402	0.735
2821	209694_at	PTS	0.64935	0.69122	0.72188	-0.07253	0.75
2822	209695_at	PTP4A3	0.21296	0.36312	0.30118	-0.08822	0.763
2823	209704_at	MTF2	0.75047	0.707	0.79074	-0.04027	0.733
2824	209707_at	PIGK	0.63105	0.54157	0.68919	-0.05814	0.662
2825	209708_at	MOXD1	0.21197	0.47363	0.42204	-0.21007	0.945
2826	209709_s_at	HMMR	0.74956	0.82305	0.70586	0.0437	0.395
2827	209711_at	SLC35D1	0.50335	0.56559	0.43706	0.06629	0.285
2828	209714_s_at	CDKN3	0.80693	0.86099	0.7368	0.07013	0.229
2829	209722_s_at	SERPINB9	0.63582	0.67057	0.58854	0.04728	0.391
2830	209724_s_at	ZFP161	0.40243	0.20553	0.10371	0.29872	0.021
2831	209725_at	UTP20	0.74244	0.73953	0.68404	0.0584	0.301
2832	209727_at	GM2A	0.67914	0.66632	0.63755	0.04159	0.343
2833	209731_at	NTHL1	0.66729	0.60816	0.57121	0.09608	0.256
2834	209732_at	CLEC2B	0.14101	0.31923	0.33045	-0.18944	0.899
2835	209748_at	SPAST	0.75612	0.69148	0.70202	0.0541	0.241
2836	209753_s_at	TMPO	0.65404	0.76163	0.75546	-0.10142	0.895
2837	209759_s_at	DCI	0.4766	0.45537	0.55488	-0.07828	0.75
2838	209760_at	KIAA0922	0.80446	0.79712	0.7188	0.08566	0.091
2839	209761_s_at	SP110	0.53124	0.58841	0.3912	0.14004	0.113
2840	209764_at	MGAT3	0.22321	0.39297	0.31684	-0.09363	0.784
2841	209770_at	BTN3A1	0.4162	0.48522	0.47648	-0.06028	0.667
2842	209778_at	TRIP11	0.43208	0.45521	0.50196	-0.06988	0.69
2843	209780_at	PHTF2	0.79278	0.8026	0.7975	-0.00472	0.525
2844	209786_at	HMGN4	0.6352	0.75646	0.78155	-0.14635	0.913
2845	209788_s_at	ERAP1	0.059959	0.18584	0.17173	-0.111771	0.774
2846	209790_s_at	CASP6	0.17032	0.35889	0.1944	-0.02408	0.569
2847	209795_at	CD69	0.45091	0.12199	0.35098	0.09993	0.203
2848	209796_s_at	CNPY2	0.76298	0.81394	0.82983	-0.06685	0.754
2849	209799_at	PRKAA1	0.1775	0.2493	0.1688	0.0087	0.495
2850	209805_at	PMS2	0.56494	0.16412	0.29921	0.26573	0.009
2851	209806_at	HIST1H2BK	0.49171	0.60278	0.59524	-0.10353	0.741
2852	209814_at	ZNF330	0.20246	0.47745	0.20406	-0.0016	0.514
2853	209820_s_at	TBL3	0.61687	0.54292	0.52103	0.09584	0.249
2854	209822_s_at	VLDLR	0.06235	0.20055	0.22477	-0.16242	0.907
2855	209825_s_at	UCK2	0.75198	0.8129	0.73131	0.02067	0.421
2856	209827_s_at	IL16	0.52736	0.39777	0.52255	0.00481	0.493
2857	209829_at	FAM65B	0.33902	0.43495	0.46212	-0.1231	0.704
2858	209832_s_at	CDT1	0.28237	0.43252	0.47337	-0.191	0.931
2859	209833_at	CRADD	0.48361	0.40917	0.43249	0.05112	0.354

2860	209838_at	COPS2	0.32192	0.39936	0.32262	-0.0007	0.519
2861	209845_at	MKRN1	0.61837	0.51972	0.49606	0.12231	0.168
2862	209849_s_at	RAD51C	0.39905	0.4339	0.26299	0.13606	0.045
2863	209853_s_at	PSME3	0.73587	0.50011	0.52015	0.21572	0.025
2864	209861_s_at	METAP2	0.65079	0.69586	0.70813	-0.05734	0.735
2865	209863_s_at	TP63	0.267	0.31239	0.37216	-0.10516	0.76
2866	209864_at	FRAT2	0.12648	0.28067	0.40016	-0.27368	0.947
2867	209865_at	SLC35A3	0.42585	0.30923	0.40621	0.01964	0.448
2868	209882_at	RIT1	0.70905	0.60244	0.50827	0.20078	0.007
2869	209883_at	GLT25D2	0.20476	0.113	0.19871	0.00605	0.473
2870	209891_at	SPC25	0.67615	0.75243	0.68324	-0.00709	0.522
2871	209894_at	LEPR	0.26912	0.23013	0.16515	0.10397	0.254
2872	209899_s_at	PUF60	0.62968	0.64047	0.64331	-0.01363	0.563
2873	209903_s_at	ATR	0.78135	0.78586	0.80731	-0.02596	0.669
2874	209910_at	SLC25A16	0.33545	0.28031	0.23572	0.09973	0.259
2875	209912_s_at	KIAA0415	0.46039	0.2556	0.2886	0.17179	0.109
2876	209916_at	DHTKD1	0.6743	0.76156	0.66065	0.01365	0.434
2877	209925_at	OCLN	0.43635	0.67373	0.62014	-0.18379	0.83
2878	209927_s_at	C1orf77	0.50757	0.5671	0.30656	0.20101	0.071
2879	209928_s_at	MSC	0.38759	0.47436	0.57801	-0.19042	0.936
2880	209932_s_at	DUT	0.82998	0.84547	0.89452	-0.06454	0.836
2881	209933_s_at	CD300A	0.51271	0.59696	0.45853	0.05418	0.334
2882	209940_at	PARP3	0.24589	0.13822	0.091477	0.154413	0.16
2883	209943_at	FBXL4	0.32096	0.34161	0.3874	-0.06644	0.689
2884	209945_s_at	GSK3B	0.3452	0.27158	0.39092	-0.04572	0.632
2885	209949_at	NCF2	0.72809	0.78661	0.75336	-0.02527	0.657
2886	209953_s_at	CDC37	0.56205	0.63168	0.55773	0.00432	0.508
2887	209965_s_at	RAD51L3	0.59554	0.53651	0.62215	-0.02661	0.594
2888	209967_s_at	CREM	0.56424	0.63297	0.66633	-0.10209	0.804
2889	209969_s_at	STAT1	0.67074	0.70411	0.62028	0.05046	0.215
2890	209972_s_at	JTV1	0.51137	0.51302	0.45869	0.05268	0.389
2891	209974_s_at	BUB3	0.60261	0.61957	0.50698	0.09563	0.183
2892	209989_at	ZNF268	0.65008	0.56209	0.55655	0.09353	0.21
2893	209994_s_at	ABCB1	0.37148	0.41423	0.306	0.06548	0.404
2894	210004_at	OLR1	0.12452	0.14387	0.099904	0.024616	0.423
2895	210006_at	ABHD14A	0.55678	0.64159	0.667	-0.11022	0.81
2896	210010_s_at	SLC25A1	0.50407	0.48514	0.27698	0.22709	0.05
2897	210024_s_at	UBE2E3	0.61389	0.45182	0.54131	0.07258	0.268
2898	210027_s_at	APEX1	0.47784	0.54999	0.6527	-0.17486	0.91
2899	210028_s_at	ORC3L	0.39775	0.45669	0.30802	0.08973	0.238
2900	210048_at	NAPG	0.30182	0.35692	0.44237	-0.14055	0.872
2901	210052_s_at	TPX2	0.83893	0.84886	0.76463	0.0743	0.237
2902	210053_at	TAF5	0.63438	0.60258	0.6553	-0.02092	0.593
2903	210054_at	HAUS3	0.69294	0.60711	0.76212	-0.06918	0.802
2904	210057_at	SMG1	0.46712	0.60791	0.56695	-0.09983	0.871
2905	210058_at	MAPK13	0.57734	0.50197	0.4729	0.10444	0.247
2906	210070_s_at	CHKB	0.32738	0.35401	0.23584	0.09154	0.274
2907	210092_at	MAGOH	0.82409	0.72979	0.79663	0.02746	0.356
2908	210097_s_at	NOL7	0.90452	0.91128	0.89833	0.00619	0.415
2909	210105_s_at	FYN	0.14232	0.41586	0.4636	-0.32128	0.974
2910	210114_at	INVS	0.34415	0.44302	0.35117	-0.00702	0.509
2911	210115_at	RPL39L	0.29717	0.65016	0.45442	-0.15725	0.904
2912	210117_at	SPAG1	0.50815	0.75081	0.62518	-0.11703	0.767

2913	210125_s_at	BANF1	0.66846	0.64416	0.68988	-0.02142	0.582
2914	210130_s_at	TM7SF2	0.30332	0.54015	0.36152	-0.0582	0.682
2915	210137_s_at	DCTD	0.48154	0.60067	0.63915	-0.15761	0.914
2916	210138_at	RGS20	0.045386	0.3364	0.23271	-0.187324	0.912
2917	210144_at	TBC1D22A	0.4107	0.44004	0.30872	0.10198	0.243
2918	210145_at	PLA2G4A	0.13411	0.20892	0.15856	-0.02445	0.583
2919	210149_s_at	ATP5H	0.88498	0.8815	0.86782	0.01716	0.312
2920	210154_at	ME2	0.74185	0.70816	0.67218	0.06967	0.217
2921	210160_at	PAFAH1B2	0.16248	0.2701	0.34443	-0.18195	0.896
2922	210172_at	SF1	0.27798	0.41736	0.30616	-0.02818	0.575
2923	210176_at	TLR1	0.80753	0.58071	0.62659	0.18094	0.018
2924	210188_at	GABPA	0.38884	0.47251	0.45199	-0.06315	0.776
2925	210195_s_at	PSG1	0.32574	0.1612	0.20314	0.1226	0.235
2926	210200_at	WWP2	0.020608	0.3568	0.14225	-0.121642	0.738
2927	210213_s_at	EIF6	0.84699	0.83783	0.86168	-0.01469	0.582
2928	210219_at	SP100	0.51347	0.33654	0.13977	0.3737	0.003
2929	210235_s_at	PPFIA1	0.34965	0.46678	0.34798	0.00167	0.499
2930	210241_s_at	TP53TG1	0.76426	0.68476	0.62783	0.13643	0.106
2931	210243_s_at	B4GALT3	0.51638	0.581	0.55862	-0.04224	0.609
2932	210253_at	HTATIP2	0.086103	0.47095	0.25356	-0.167457	0.846
2933	210258_at	RGS13	0.53528	0.63709	0.63273	-0.09745	0.817
2934	210260_s_at	TNFAIP8	0.4272	0.47133	0.41404	0.01316	0.472
2935	210275_s_at	ZFAND5	0.33174	0.54	0.54453	-0.21279	0.942
2936	210276_s_at	TRIOBP	0.42885	0.48783	0.64133	-0.21248	0.982
2937	210278_s_at	AP4S1	0.55315	0.38907	0.38236	0.17079	0.165
2938	210279_at	GPR18	0.07325	0.24327	0.12901	-0.05576	0.679
2939	210284_s_at	MAP3K7IP2	0.72456	0.82871	0.785	-0.06044	0.838
2940	210296_s_at	PXMP3	0.62292	0.73494	0.60366	0.01926	0.412
2941	210301_at	XDH	0.39358	0.34003	0.45453	-0.06095	0.634
2942	210312_s_at	IFT20	0.56545	0.60705	0.66524	-0.09979	0.799
2943	210338_s_at	HSPA8	0.71799	0.69398	0.59817	0.11982	0.108
2944	210346_s_at	CLK4	0.62396	0.17873	0.32445	0.29951	<0.001
2945	210349_at	CAMK4	0.4	0.3759	0.43766	-0.03766	0.583
2946	210371_s_at	RBBP4	0.57343	0.46816	0.43763	0.1358	0.021
2947	210377_at	ACSM3	0.65486	0.63483	0.46352	0.19134	0.02
2948	210378_s_at	SSNA1	0.73613	0.78353	0.76409	-0.02796	0.611
2949	210379_s_at	TLK1	0.35365	0.43014	0.43979	-0.08614	0.762
2950	210386_s_at	MTX1	0.79712	0.79881	0.82944	-0.03232	0.648
2951	210396_s_at	BOLA2	0.4538	0.4431	0.4801	-0.0263	0.573
2952	210406_s_at	RAB6A	0.56759	0.60931	0.7096	-0.14201	0.886
2953	210417_s_at	PI4KB	0.67571	0.57244	0.53355	0.14216	0.069
2954	210418_s_at	IDH3B	0.76833	0.67893	0.70394	0.06439	0.262
2955	210428_s_at	HGS	0.77528	0.8319	0.77616	-0.00088	0.503
2956	210448_s_at	P2RX5	0.096318	0.41877	0.22234	-0.126022	0.893
2957	210479_s_at	RORA	0.09031	0.31052	0.33345	-0.24314	0.962
2958	210502_s_at	PPIE	0.38842	0.53434	0.41536	-0.02694	0.568
2959	210512_s_at	VEGFA	0.56853	0.43961	0.28624	0.28229	0.017
2960	210528_at	MR1	0.28232	0.35926	0.257	0.02532	0.499
2961	210531_at	NR2C1	0.37776	0.14816	0.14106	0.2367	0.063
2962	210538_s_at	BIRC3	0.51711	0.59768	0.66563	-0.14852	0.914
2963	210543_s_at	PRKDC	0.82065	0.80494	0.81019	0.01046	0.419
2964	210556_at	NFATC3	0.1285	0.2883	0.24456	-0.11606	0.726
2965	210559_s_at	CDC2	0.74843	0.78745	0.67401	0.07442	0.187

2966	210561_s_at	WSB1	0.45361	0.57212	0.48912	-0.03551	0.636
2967	210573_s_at	POLR3C	0.35841	0.30567	0.22834	0.13007	0.165
2968	210574_s_at	NUDC	0.77095	0.85986	0.80786	-0.03691	0.662
2969	210587_at	INHBE	0.44624	0.26043	0.23345	0.21279	0.163
2970	210596_at	LOC100129513	0.58616	0.66056	0.62055	-0.03439	0.657
2971	210609_s_at	TP53I3	0.61429	0.55124	0.56144	0.05285	0.271
2972	210620_s_at	GTF3C2	0.76916	0.61637	0.38672	0.38244	<0.001
2973	210625_s_at	AKAP1	0.53109	0.39329	0.38207	0.14902	0.138
2974	210635_s_at	KLHL20	0.5933	0.41737	0.57384	0.01946	0.457
2975	210638_s_at	FBXO9	0.52187	0.44919	0.40439	0.11748	0.184
2976	210639_s_at	ATG5	0.43358	0.48564	0.39423	0.03935	0.383
2977	210643_at	TNFSF11	0.44183	0.58805	0.56037	-0.11854	0.814
2978	210649_s_at	ARID1A	0.45497	0.50849	0.46757	-0.0126	0.552
2979	210685_s_at	UBE4B	0.67779	0.70687	0.55116	0.12663	0.088
2980	210705_s_at	TRIM5	0.59044	0.60828	0.60406	-0.01362	0.574
2981	210732_s_at	LGALS8	0.4295	0.46416	0.22403	0.20547	0.184
2982	210733_at	TRAM1	0.69026	0.66924	0.67877	0.01149	0.444
2983	210740_s_at	ITPK1	0.71971	0.57242	0.70507	0.01464	0.475
2984	210748_at	LOC100129624	0.51595	0.45938	0.50714	0.00881	0.477
2985	210752_s_at	MLX	0.57815	0.46324	0.36071	0.21744	0.04
2986	210758_at	PSIP1	0.52532	0.45415	0.61271	-0.08739	0.774
2987	210759_s_at	PSMA1	0.82309	0.85929	0.90128	-0.07819	0.932
2988	210771_at	PPARA	0.29802	0.10268	0.32073	-0.02271	0.572
2989	210774_s_at	NCOA4	0.44955	0.66115	0.57549	-0.12594	0.777
2990	210785_s_at	C1orf38	0.48263	0.56654	0.42482	0.05781	0.347
2991	210786_s_at	FLI1	0.75721	0.6977	0.77623	-0.01902	0.62
2992	210793_s_at	NUP98	0.67607	0.76256	0.61656	0.05951	0.33
2993	210797_s_at	OASL	0.61599	0.54234	0.44222	0.17377	0.002
2994	210811_s_at	DDX49	0.43768	0.49728	0.56669	-0.12901	0.805
2995	210813_s_at	XRCC4	0.23562	0.29673	0.28311	-0.04749	0.63
2996	210817_s_at	CALCOCO2	0.20948	0.20655	0.31518	-0.1057	0.745
2997	210822_at	RPL13P5	0.11507	0.25318	0.2544	-0.13933	0.725
2998	210868_s_at	ELOVL6	0.037011	0.23404	0.23966	-0.202649	0.932
2999	210878_s_at	KDM3B	0.76669	0.8638	0.86604	-0.09935	0.838
3000	210889_s_at	FCGR2B	0.42444	0.65845	0.49847	-0.07403	0.739
3001	210892_s_at	GTF2I	0.81044	0.78364	0.8337	-0.02326	0.675
3002	210895_s_at	CD86	0.50419	0.59572	0.57258	-0.06839	0.787
3003	210907_s_at	PDCD10	0.69999	0.73044	0.76686	-0.06687	0.697
3004	210908_s_at	PFDN5	0.87199	0.87316	0.89608	-0.02409	0.67
3005	210910_s_at	POMZP3	0.017784	0.11002	0.10833	-0.090546	0.809
3006	210926_at	ACTBL3	0.41608	0.61197	0.6692	-0.25312	0.976
3007	210942_s_at	ST3GAL6	0.3217	0.47087	0.53909	-0.21739	0.941
3008	210943_s_at	LYST	0.15434	0.1765	0.24576	-0.09142	0.702
3009	210944_s_at	CAPN3	0.22028	0.28787	0.37252	-0.15224	0.64
3010	210946_at	PPAP2A	0.4092	0.42793	0.41975	-0.01055	0.515
3011	210947_s_at	MSH3	0.4332	0.64403	0.57425	-0.14105	0.843
3012	210949_s_at	EIF3C	0.52311	0.55335	0.52424	-0.00113	0.487
3013	210959_s_at	SRD5A1	0.55195	0.58553	0.58092	-0.02897	0.62
3014	210968_s_at	RTN4	0.40637	0.66357	0.52636	-0.11999	0.852
3015	210971_s_at	ARNTL	0.4365	0.34923	0.46121	-0.02471	0.584
3016	210976_s_at	PFKM	0.67255	0.6886	0.42134	0.25121	0.003
3017	210978_s_at	TAGLN2	0.67041	0.72284	0.61474	0.05567	0.302
3018	210983_s_at	MCM7	0.83354	0.8071	0.76781	0.06573	0.115

3019	210996_s_at	YWHAE	0.3596	0.53382	0.49964	-0.14004	0.928
3020	211009_s_at	ZNF271	0.42282	0.41239	0.48157	-0.05875	0.642
3021	211015_s_at	HSPA4	0.79223	0.70999	0.71147	0.08076	0.11
3022	211026_s_at	MGLL	0.55859	0.5998	0.58997	-0.03138	0.615
3023	211031_s_at	CLIP2	0.51979	0.72067	0.68966	-0.16987	0.914
3024	211033_s_at	PEX7	0.37862	0.4147	0.26701	0.11161	0.197
3025	211034_s_at	C12orf51	0.5497	0.60911	0.66602	-0.11632	0.87
3026	211038_s_at	CROCCL1	0.38279	0.34131	0.29242	0.09037	0.228
3027	211043_s_at	CLTB	0.67364	0.61677	0.7475	-0.07386	0.905
3028	211048_s_at	PDIA4	0.41792	0.52762	0.55858	-0.14066	0.872
3029	211064_at	ZNF493	0.35792	0.36963	0.29888	0.05904	0.352
3030	211071_s_at	MLLT11	0.26765	0.50088	0.46747	-0.19982	0.933
3031	211074_at	FOLR1	0.43174	0.47838	0.51308	-0.08134	0.667
3032	211075_s_at	CD47	0.57453	0.69098	0.70588	-0.13135	0.834
3033	211089_s_at	NEK3	0.28174	0.15746	0.14316	0.13858	0.268
3034	211136_s_at	CLPTM1	0.78889	0.73076	0.77463	0.01426	0.519
3035	211168_s_at	UPF1	0.35407	0.49106	0.54873	-0.19466	0.947
3036	211200_s_at	EFCAB2	0.093423	0.27843	0.21701	-0.123587	0.784
3037	211212_s_at	ORC5L	0.5971	0.59598	0.61932	-0.02222	0.562
3038	211250_s_at	SH3BP2	0.35655	0.42546	0.33133	0.02522	0.412
3039	211275_s_at	GYG1	0.57456	0.67153	0.56692	0.00764	0.507
3040	211276_at	TCEAL2	0.23107	0.25959	0.18658	0.04449	0.391
3041	211284_s_at	GRN	0.51635	0.60925	0.59838	-0.08203	0.744
3042	211285_s_at	UBE3A	0.66514	0.74795	0.66751	-0.00237	0.492
3043	211297_s_at	CDK7	0.66698	0.62377	0.55884	0.10814	0.21
3044	211317_s_at	CFLAR	0.56304	0.41595	0.56034	0.0027	0.51
3045	211330_s_at	HFE	0.19546	0.17336	0.061871	0.133589	0.195
3046	211339_s_at	ITK	0.21057	0.28687	0.23934	-0.02877	0.599
3047	211352_s_at	NCOA3	0.57278	0.67634	0.64472	-0.07194	0.748
3048	211358_s_at	CIZ1	0.45239	0.51361	0.47441	-0.02202	0.564
3049	211364_at	MTAP	0.18303	0.42218	0.26995	-0.08692	0.756
3050	211368_s_at	CASP1	0.47529	0.59722	0.57585	-0.10056	0.766
3051	211391_s_at	PATZ1	0.44748	0.20135	0.2541	0.19338	0.096
3052	211404_s_at	APLP2	0.22864	0.28501	0.10855	0.12009	0.16
3053	211406_at	IER3IP1	0.65248	0.73167	0.69773	-0.04525	0.679
3054	211450_s_at	MSH6	0.80542	0.80775	0.75925	0.04617	0.281
3055	211475_s_at	BAG1	0.6493	0.59071	0.71613	-0.06683	0.771
3056	211501_s_at	EIF3B	0.73377	0.75629	0.68099	0.05278	0.275
3057	211502_s_at	PFTK1	0.00078507	0.029921	0.08518	-0.08439493	0.673
3058	211505_s_at	STAU1	0.48655	0.45723	0.47649	0.01006	0.476
3059	211512_s_at	OGFR	0.44415	0.0040203	0.36477	0.07938	0.303
3060	211543_s_at	GRK6	0.56363	0.28104	0.33367	0.22996	0.048
3061	211558_s_at	DHPS	0.62705	0.59342	0.36208	0.26497	0.007
3062	211563_s_at	C19orf2	0.35942	0.38593	0.37887	-0.01945	0.607
3063	211569_s_at	HADH	0.42593	0.54494	0.51701	-0.09108	0.805
3064	211596_s_at	LRIG1	0.15032	0.31083	0.27926	-0.12894	0.821
3065	211600_at	PTPRO	0.10726	0.28677	0.2743	-0.16704	0.877
3066	211612_s_at	IL13RA1	0.18751	0.35514	0.26763	-0.08012	0.684
3067	211623_s_at	FBL	0.72696	0.81122	0.83936	-0.1124	0.897
3068	211662_s_at	VDAC2	0.7576	0.81768	0.81945	-0.06185	0.907
3069	211665_s_at	SOS2	0.63929	0.62755	0.62859	0.0107	0.444
3070	211671_s_at	NR3C1	0.76222	0.79986	0.71169	0.05053	0.279
3071	211672_s_at	ARPC4	0.6417	0.53152	0.59039	0.05131	0.32

3072	211676_s_at	IFNGR1	0.64819	0.77107	0.77333	-0.12514	0.952
3073	211678_s_at	RNF114	0.54172	0.36811	0.41084	0.13088	0.127
3074	211684_s_at	DYNC1I2	0.55389	0.52863	0.57245	-0.01856	0.564
3075	211685_s_at	NCALD	0.25658	0.46702	0.29058	-0.034	0.603
3076	211686_s_at	MAK16	0.68252	0.69147	0.6784	0.00412	0.466
3077	211692_s_at	BBC3	0.53687	0.21634	0.49535	0.04152	0.39
3078	211702_s_at	USP32	0.54281	0.49065	0.57739	-0.03458	0.629
3079	211707_s_at	IQCB1	0.29467	0.44297	0.34077	-0.0461	0.631
3080	211715_s_at	BDH1	0.44112	0.44832	0.2903	0.15082	0.169
3081	211717_at	ANKRD40	0.26392	0.31773	0.37102	-0.1071	0.715
3082	211721_s_at	ZNF551	0.49557	0.45717	0.40073	0.09484	0.286
3083	211725_s_at	BID	0.55323	0.64379	0.64519	-0.09196	0.71
3084	211727_s_at	COX11	0.56759	0.54565	0.512	0.05559	0.335
3085	211742_s_at	EVI2B	0.41351	0.49705	0.48165	-0.06814	0.703
3086	211749_s_at	VAMP3	0.50654	0.61214	0.56335	-0.05681	0.691
3087	211752_s_at	NDUFS7	0.8353	0.73611	0.77555	0.05975	0.196
3088	211753_s_at	RLN1	0.20933	0.28406	0.17503	0.0343	0.462
3089	211754_s_at	SLC25A17	0.23424	0.4989	0.4406	-0.20636	0.857
3090	211755_s_at	ATP5F1	0.70764	0.78818	0.87993	-0.17229	0.995
3091	211763_s_at	UBE2B	0.87831	0.77733	0.71278	0.16553	<0.001
3092	211764_s_at	UBE2D1	0.69988	0.44541	0.59434	0.10554	0.186
3093	211767_at	GINS4	0.038684	0.29547	0.17123	-0.132546	0.826
3094	211774_s_at	MMACHC	0.39904	0.34661	0.23026	0.16878	0.115
3095	211783_s_at	MTA1	0.63825	0.72542	0.68029	-0.04204	0.664
3096	211784_s_at	SFRS1	0.74631	0.85402	0.66122	0.08509	0.189
3097	211787_s_at	EIF4A1	0.9125	0.91191	0.91302	-0.00052	0.519
3098	211792_s_at	CDKN2C	0.33449	0.40422	0.38657	-0.05208	0.658
3099	211796_s_at	TRBC1	0.49491	0.59603	0.56252	-0.06761	0.663
3100	211810_s_at	GALC	0.37349	0.33228	0.30225	0.07124	0.351
3101	211819_s_at	SORBS1	0.44442	0.099722	0.047736	0.396684	0.001
3102	211825_s_at	EWSR1	0.40195	0.40312	0.58179	-0.17984	0.953
3103	211828_s_at	TNIK	0.72749	0.65567	0.70611	0.02138	0.476
3104	211833_s_at	BAX	0.62074	0.67673	0.62358	-0.00284	0.533
3105	211913_s_at	MERTK	0.26175	0.36923	0.22645	0.0353	0.443
3106	211926_s_at	MYH9	0.76375	0.79262	0.78612	-0.02237	0.629
3107	211928_at	DYNC1H1	0.708	0.69308	0.6892	0.0188	0.405
3108	211935_at	ARL6IP1	0.65509	0.58687	0.45531	0.19978	0.091
3109	211936_at	HSPA5	0.55562	0.60912	0.52695	0.02867	0.426
3110	211938_at	EIF4B	0.64022	0.6739	0.65552	-0.0153	0.567
3111	211946_s_at	BAT2D1	0.83682	0.7481	0.71901	0.11781	0.057
3112	211950_at	UBR4	0.77222	0.78332	0.74101	0.03121	0.372
3113	211955_at	IPO5	0.89282	0.79955	0.78979	0.10303	0.109
3114	211956_s_at	EIF1	0.95739	0.93577	0.93853	0.01886	0.099
3115	211960_s_at	RAB7A	0.46562	0.55573	0.35844	0.10718	0.089
3116	211962_s_at	ZFP36L1	0.61329	0.55822	0.67581	-0.06252	0.659
3117	211963_s_at	ARPC5	0.28174	0.53425	0.42434	-0.1426	0.872
3118	211967_at	TMEM123	0.39783	0.51971	0.48593	-0.0881	0.831
3119	211971_s_at	LRPPRC	0.63384	0.64645	0.66596	-0.03212	0.619
3120	211975_at	ARFGAP2	0.69034	0.70358	0.69039	-5E-05	0.536
3121	211985_s_at	CALM1	0.65359	0.74648	0.7639	-0.11031	0.823
3122	211987_at	TOP2B	0.78355	0.7836	0.79212	-0.00857	0.555
3123	211989_at	SMARCE1	0.73073	0.72596	0.67423	0.0565	0.2
3124	211990_at	HLA-DPA1	0.83336	0.90072	0.9162	-0.08284	0.905

3125	211994_at	WNK1	0.44823	0.52266	0.54938	-0.10115	0.79
3126	211998_at	H3F3B	0.25482	0.034406	0.16716	0.08766	0.322
3127	212005_at	C1orf144	0.59134	0.47789	0.56503	0.02631	0.549
3128	212007_at	UBXN4	0.37237	0.29598	0.25545	0.11692	0.19
3129	212009_s_at	STIP1	0.78518	0.69485	0.76907	0.01611	0.378
3130	212016_s_at	PTBP1	0.84833	0.67415	0.65727	0.19106	0.052
3131	212017_at	FAM168B	0.83964	0.81137	0.62996	0.20968	0.006
3132	212021_s_at	MKI67	0.68956	0.66762	0.56574	0.12382	0.204
3133	212025_s_at	FLII	0.65964	0.64662	0.54739	0.11225	0.242
3134	212027_at	RBM25	0.67272	0.48781	0.54925	0.12347	0.125
3135	212032_s_at	PTOV1	0.3297	0.29573	0.30768	0.02202	0.446
3136	212036_s_at	PNN	0.56564	0.19154	0.29703	0.26861	0.01
3137	212038_s_at	VDAC1	0.42317	0.54286	0.57356	-0.15039	0.896
3138	212040_at	TGOLN2	0.71173	0.68007	0.62446	0.08727	0.237
3139	212041_at	ATP6V0D1	0.6807	0.66544	0.73081	-0.05011	0.731
3140	212044_s_at	RPL27A	0.6337	0.73751	0.72868	-0.09498	0.92
3141	212047_s_at	RNF167	0.36573	0.36379	0.23996	0.12577	0.185
3142	212048_s_at	YARS	0.72717	0.65918	0.50782	0.21935	0.007
3143	212050_at	WIPF2	0.74978	0.65021	0.71273	0.03705	0.312
3144	212052_s_at	TBC1D9B	0.52782	0.51373	0.4901	0.03772	0.407
3145	212053_at	PDXDC1	0.61578	0.63606	0.60945	0.00633	0.484
3146	212055_at	C18orf10	0.26546	0.6484	0.54322	-0.27776	0.953
3147	212057_at	KIAA0182	0.49755	0.35498	0.49222	0.00533	0.484
3148	212058_at	SR140	0.75446	0.73108	0.79536	-0.0409	0.704
3149	212059_s_at	TRPC4AP	0.54505	0.57983	0.45189	0.09316	0.305
3150	212066_s_at	USP34	0.49496	0.63266	0.58765	-0.09269	0.813
3151	212069_s_at	BAT2L	0.40799	0.61969	0.69081	-0.28282	0.987
3152	212072_s_at	CSNK2A1	0.66493	0.50345	0.38071	0.28422	0.005
3153	212077_at	CALD1	0.16472	0.22669	0.42757	-0.26285	0.974
3154	212078_s_at	MLL	0.41891	0.33238	0.24443	0.17448	0.092
3155	212082_s_at	MYL6	0.91231	0.91237	0.92966	-0.01735	0.701
3156	212083_at	TEX261	0.71603	0.69436	0.69861	0.01742	0.437
3157	212087_s_at	ERAL1	0.60952	0.71316	0.65561	-0.04609	0.705
3158	212088_at	PMPCA	0.66197	0.46237	0.54295	0.11902	0.102
3159	212090_at	GRINA	0.62394	0.72596	0.54943	0.07451	0.21
3160	212092_at	PEG10	0.29983	0.50417	0.47579	-0.17596	0.859
3161	212098_at	LOC151162	0.7144	0.77641	0.77261	-0.05821	0.784
3162	212099_at	RHOB	0.518	0.4769	0.51801	-1E-05	0.548
3163	212100_s_at	POLDIP3	0.6867	0.62256	0.58895	0.09775	0.227
3164	212101_at	KPNA6	0.85582	0.82024	0.77447	0.08135	0.035
3165	212106_at	FAF2	0.12521	0.038526	0.13117	-0.00596	0.499
3166	212110_at	SLC39A14	0.64536	0.64993	0.47887	0.16649	0.139
3167	212112_s_at	STX12	0.33473	0.33674	0.1847	0.15003	0.151
3168	212115_at	HN1L	0.72682	0.74712	0.68124	0.04558	0.266
3169	212118_at	TRIM27	0.74357	0.62454	0.42969	0.31388	0.016
3170	212119_at	RHOQ	0.53262	0.59669	0.50224	0.03038	0.411
3171	212121_at	TCTN3	0.64879	0.52748	0.60965	0.03914	0.36
3172	212124_at	ZMIZ1	0.40897	0.45198	0.50506	-0.09609	0.764
3173	212126_at	CBX5	0.55252	0.64932	0.56966	-0.01714	0.572
3174	212129_at	NIPA2	0.68304	0.44212	0.42306	0.25998	0.004
3175	212131_at	LSM14A	0.69943	0.62626	0.62976	0.06967	0.186
3176	212140_at	PDS5A	0.65047	0.6537	0.63552	0.01495	0.427
3177	212141_at	MCM4	0.87262	0.83746	0.78999	0.08263	0.056

3178	212144_at	UNC84B	0.39555	0.1266	0.073422	0.322128	0.016
3179	212145_at	MRPS27	0.66943	0.5199	0.60421	0.06522	0.218
3180	212150_at	EFR3A	0.62977	0.69312	0.62501	0.00476	0.481
3181	212155_at	RNF187	0.50469	0.58493	0.55796	-0.05327	0.71
3182	212160_at	XPOT	0.86138	0.81456	0.80119	0.06019	0.115
3183	212161_at	AP2A2	0.047667	0.19733	0.19944	-0.151773	0.744
3184	212165_at	TMEM183A	0.5176	0.34893	0.29604	0.22156	0.016
3185	212166_at	XPO7	0.46865	0.35622	0.51418	-0.04553	0.616
3186	212170_at	RBM12	0.59668	0.44636	0.3059	0.29078	0.061
3187	212177_at	SFRS18	0.74199	0.72575	0.81875	-0.07676	0.872
3188	212186_at	ACACA	0.41812	0.41283	0.40087	0.01725	0.473
3189	212188_at	KCTD12	0.55352	0.63413	0.46643	0.08709	0.311
3190	212189_s_at	COG4	0.57624	0.60319	0.62832	-0.05208	0.72
3191	212190_at	SERPINE2	0.32524	0.49012	0.46171	-0.13647	0.906
3192	212193_s_at	LARP1	0.80639	0.76615	0.75475	0.05164	0.277
3193	212194_s_at	TM9SF4	0.6109	0.53848	0.51735	0.09355	0.2
3194	212199_at	MRFAP1L1	0.24169	0.27664	0.27578	-0.03409	0.631
3195	212200_at	ANKLE2	0.73105	0.72363	0.57786	0.15319	0.045
3196	212202_s_at	TMEM87A	0.57066	0.31004	0.3987	0.17196	0.064
3197	212208_at	MED13L	0.59666	0.45706	0.52814	0.06852	0.293
3198	212211_at	ANKRD17	0.71652	0.70218	0.81417	-0.09765	0.889
3199	212217_at	PREPL	0.7795	0.77824	0.75101	0.02849	0.32
3200	212218_s_at	FASN	0.55377	0.49508	0.47074	0.08303	0.22
3201	212219_at	PSME4	0.69498	0.78699	0.66713	0.02785	0.354
3202	212228_s_at	COQ9	0.27686	0.15493	0.23964	0.03722	0.396
3203	212231_at	FBXO21	0.41546	0.43867	0.53977	-0.12431	0.864
3204	212232_at	FNBP4	0.62702	0.59444	0.51529	0.11173	0.169
3205	212233_at	MAP1B	0.05553	0.20041	0.23815	-0.18262	0.935
3206	212238_at	ASXL1	0.66806	0.63289	0.60182	0.06624	0.283
3207	212242_at	TUBA4A	0.68013	0.7439	0.67656	0.00357	0.49
3208	212244_at	GCOM1	0.59791	0.63454	0.6297	-0.03179	0.631
3209	212245_at	MCFD2	0.65673	0.66709	0.66079	-0.00406	0.523
3210	212247_at	NUP205	0.77759	0.79269	0.7745	0.00309	0.51
3211	212249_at	PIK3R1	0.6529	0.75947	0.72647	-0.07357	0.797
3212	212251_at	MTDH	0.66354	0.72583	0.61509	0.04845	0.277
3213	212255_s_at	ATP2C1	0.54685	0.71677	0.66531	-0.11846	0.846
3214	212263_at	QKI	0.59654	0.65589	0.62499	-0.02845	0.649
3215	212266_s_at	SFRS5	0.29208	0.43147	0.17171	0.12037	0.157
3216	212271_at	MAPK1	0.66968	0.78838	0.70972	-0.04004	0.656
3217	212274_at	LPIN1	0.33209	0.48507	0.38777	-0.05568	0.657
3218	212277_at	MTMR4	0.70023	0.83054	0.73638	-0.03615	0.684
3219	212281_s_at	TMEM97	0.29356	0.57227	0.389	-0.09544	0.731
3220	212287_at	SUZ12	0.80137	0.78573	0.75321	0.04816	0.269
3221	212293_at	HIPK1	0.45724	0.32108	0.34231	0.11493	0.234
3222	212296_at	PSMD14	0.56628	0.72565	0.73252	-0.16624	0.914
3223	212297_at	ATP13A3	0.82214	0.8551	0.85543	-0.03329	0.683
3224	212300_at	TXLNA	0.77084	0.83968	0.70597	0.06487	0.238
3225	212302_at	RTF1	0.46165	0.47549	0.48716	-0.02551	0.604
3226	212308_at	CLASP2	0.66737	0.60058	0.63295	0.03442	0.404
3227	212310_at	MIA3	0.43087	0.39895	0.28296	0.14791	0.106
3228	212311_at	KIAA0746	0.30097	0.30918	0.28007	0.0209	0.454
3229	212312_at	BCL2L1	0.72039	0.71818	0.69006	0.03033	0.375
3230	212313_at	CHMP7	0.50987	0.42492	0.40924	0.10063	0.323

3231	212317_at	TNPO3	0.67949	0.47167	0.58712	0.09237	0.222
3232	212320_at	TUBB	0.80473	0.76701	0.73272	0.07201	0.205
3233	212323_s_at	VPS13D	0.0061728	0.26891	0.22585	-0.2196772	0.794
3234	212329_at	SCAP	0.65116	0.57404	0.62312	0.02804	0.445
3235	212330_at	TFDP1	0.77685	0.75142	0.70865	0.0682	0.205
3236	212333_at	FAM98A	0.72536	0.72607	0.52026	0.2051	0.033
3237	212340_at	YIPF6	1.09E-006	0.17447	0.16385	-0.163848913	0.803
3238	212345_s_at	CREB3L2	0.59093	0.68366	0.62501	-0.03408	0.615
3239	212348_s_at	KDM1	0.526	0.45941	0.44065	0.08535	0.301
3240	212350_at	TBC1D1	0.38778	0.53037	0.54545	-0.15767	0.87
3241	212351_at	EIF2B5	0.63902	0.63346	0.56575	0.07327	0.26
3242	212352_s_at	TMED10	0.72442	0.78699	0.82278	-0.09836	0.937
3243	212355_at	KIAA0323	0.64196	0.64202	0.61339	0.02857	0.387
3244	212357_at	FAM168A	0.49052	0.49661	0.42344	0.06708	0.328
3245	212360_at	AMPD2	0.81982	0.78471	0.54029	0.27953	<0.001
3246	212366_at	ZNF292	0.68893	0.69871	0.80956	-0.12063	0.86
3247	212371_at	PPPDE1	0.69706	0.75992	0.63608	0.06098	0.267
3248	212372_at	MYH10	0.038705	0.29231	0.1121	-0.073395	0.773
3249	212376_s_at	EP400	0.34576	0.41093	0.50909	-0.16333	0.78
3250	212380_at	FTSJD2	0.67918	0.67236	0.63713	0.04205	0.388
3251	212381_at	USP24	0.5786	0.44846	0.62604	-0.04744	0.692
3252	212383_at	ATP6V0A1	0.36252	0.44418	0.36778	-0.00526	0.499
3253	212398_at	RDX	0.8185	0.84388	0.83289	-0.01439	0.602
3254	212400_at	FAM102A	0.56938	0.45126	0.39916	0.17022	0.093
3255	212401_s_at	CDC2L2	0.086287	0.097984	0.19793	-0.111643	0.774
3256	212402_at	ZC3H13	0.68997	0.74989	0.67861	0.01136	0.439
3257	212403_at	UBE3B	0.34497	0.41847	0.48688	-0.14191	0.865
3258	212405_s_at	METTL13	0.40031	0.45992	0.54802	-0.14771	0.846
3259	212406_s_at	PCMTD2	0.69713	0.62469	0.66352	0.03361	0.416
3260	212408_at	TOR1AIP1	0.80049	0.702	0.74432	0.05617	0.208
3261	212410_at	EFHA1	0.7672	0.73752	0.73269	0.03451	0.386
3262	212411_at	IMP4	0.68404	0.7554	0.71707	-0.03303	0.696
3263	212415_at	SEPT6	0.6136	0.72752	0.61593	-0.00233	0.502
3264	212420_at	ELF1	0.52161	0.58462	0.54256	-0.02095	0.599
3265	212422_at	PDCD11	0.76761	0.81529	0.71643	0.05118	0.278
3266	212430_at	RBM38	0.23	0.49742	0.53262	-0.30262	0.95
3267	212434_at	GRPEL1	0.63578	0.58996	0.39445	0.24133	0.027
3268	212436_at	TRIM33	0.22773	0.44803	0.5217	-0.29397	0.958
3269	212437_at	CENPB	0.3841	0.37104	0.49601	-0.11191	0.864
3270	212439_at	IP6K1	0.59091	0.64481	0.64903	-0.05812	0.733
3271	212440_at	SNRNP27	0.51496	0.72836	0.71993	-0.20497	0.948
3272	212441_at	KIAA0232	0.54839	0.49071	0.31992	0.22847	0.036
3273	212443_at	NBEAL2	0.64261	0.74004	0.61552	0.02709	0.443
3274	212445_s_at	NEDD4L	0.27591	0.37081	0.33817	-0.06226	0.697
3275	212446_s_at	LASS6	0.19415	0.39144	0.2843	-0.09015	0.707
3276	212447_at	KBTBD2	0.35892	0.58072	0.31916	0.03976	0.4
3277	212449_s_at	LYPLA1	0.84325	0.84032	0.84544	-0.00219	0.522
3278	212451_at	SECISBP2L	0.30099	0.30344	0.34313	-0.04214	0.668
3279	212453_at	KIAA1279	0.52877	0.6728	0.54299	-0.01422	0.519
3280	212456_at	KIAA0664	0.85289	0.74843	0.76857	0.08432	0.141
3281	212458_at	SPRED2	0.21866	0.23025	0.36329	-0.14463	0.869
3282	212462_at	MYST4	0.4956	0.45406	0.53717	-0.04157	0.675
3283	212465_at	SETD3	0.2829	0.22838	0.26103	0.02187	0.44

3284	212467_at	DNAJC13	0.79693	0.83719	0.76749	0.02944	0.374
3285	212470_at	SPAG9	0.66012	0.68247	0.67136	-0.01124	0.541
3286	212471_at	AVL9	0.20369	0.32104	0.24804	-0.04435	0.61
3287	212473_s_at	MICAL2	0.1367	0.22489	0.19287	-0.05617	0.682
3288	212476_at	ACAP2	0.87816	0.84639	0.82289	0.05527	0.212
3289	212479_s_at	RMND5A	0.41024	0.34839	0.53117	-0.12093	0.792
3290	212483_at	NIPBL	0.49854	0.63686	0.594	-0.09546	0.792
3291	212487_at	GPATCH8	0.54446	0.45011	0.47366	0.0708	0.32
3292	212491_s_at	DNAJC8	0.75658	0.73368	0.71954	0.03704	0.338
3293	212499_s_at	FCF1	0.76315	0.78127	0.8182	-0.05505	0.701
3294	212500_at	ADO	0.61136	0.61025	0.49525	0.11611	0.125
3295	212501_at	CEBPB	0.23681	0.31829	0.21934	0.01747	0.461
3296	212505_s_at	KIAA0892	0.49717	0.40075	0.28223	0.21494	0.054
3297	212507_at	TMEM131	0.53347	0.74137	0.60195	-0.06848	0.738
3298	212508_at	MOAP1	0.67831	0.29795	0.60597	0.07234	0.311
3299	212509_s_at	MXRA7	0.042981	0.15951	0.13031	-0.087329	0.809
3300	212512_s_at	CARM1	0.72333	0.79479	0.7539	-0.03057	0.653
3301	212515_s_at	DDX3X	0.55078	0.51448	0.45941	0.09137	0.211
3302	212517_at	ATRN	0.34111	0.26818	0.32302	0.01809	0.423
3303	212520_s_at	SMARCA4	0.49179	0.60474	0.56099	-0.0692	0.691
3304	212523_s_at	KIAA0146	0.22633	0.38843	0.33528	-0.10895	0.793
3305	212526_at	SPG20	0.19581	0.38342	0.45679	-0.26098	0.984
3306	212527_at	PPPDE2	0.67061	0.55029	0.63096	0.03965	0.346
3307	212528_at	D15Wsu75e	0.58685	0.58547	0.3525	0.23435	0.016
3308	212529_at	LSM12	0.44446	0.41514	0.39149	0.05297	0.378
3309	212530_at	NEK7	0.69654	0.75432	0.75653	-0.05999	0.791
3310	212536_at	ATP11B	0.66665	0.57239	0.70539	-0.03874	0.648
3311	212538_at	DOCK9	0.29009	0.33378	0.41918	-0.12909	0.808
3312	212539_at	CHD1L	0.68698	0.52965	0.54541	0.14157	0.146
3313	212540_at	CDC34	0.37496	0.50208	0.51941	-0.14445	0.895
3314	212541_at	FLAD1	0.30885	0.28159	0.28323	0.02562	0.421
3315	212542_s_at	PHIP	0.75439	0.75206	0.7127	0.04169	0.279
3316	212543_at	AIM1	0.44038	0.43207	0.36885	0.07153	0.326
3317	212544_at	ZNHIT3	0.5684	0.70387	0.77188	-0.20348	0.938
3318	212548_s_at	FRYL	0.61459	0.18184	0.37689	0.2377	0.06
3319	212550_at	STAT5B	0.2527	0.22456	0.095128	0.157572	0.14
3320	212556_at	SCRIB	0.82063	0.77767	0.60636	0.21427	0.022
3321	212557_at	ZNF451	0.5964	0.5678	0.68968	-0.09328	0.849
3322	212558_at	SPRY1	0.58582	0.3818	0.51919	0.06663	0.327
3323	212560_at	SORL1	0.53489	0.7172	0.67694	-0.14205	0.905
3324	212561_at	DENND5A	0.5715	0.77418	0.71805	-0.14655	0.925
3325	212568_s_at	DLAT	0.8346	0.83122	0.85775	-0.02315	0.736
3326	212571_at	CHD8	0.68119	0.5488	0.51819	0.163	0.072
3327	212572_at	STK38L	0.66465	0.67864	0.64706	0.01759	0.448
3328	212573_at	ENDOD1	0.55458	0.59418	0.58137	-0.02679	0.618
3329	212576_at	MGRN1	0.49803	0.66933	0.47575	0.02228	0.444
3330	212579_at	SMCHD1	0.84697	0.79875	0.79114	0.05583	0.141
3331	212584_at	AQR	0.87653	0.85926	0.83173	0.0448	0.117
3332	212585_at	OSBPL8	0.83564	0.82023	0.82082	0.01482	0.461
3333	212586_at	CAST	0.46954	0.67306	0.68615	-0.21661	0.97
3334	212587_s_at	PTPRC	0.60663	0.69461	0.66294	-0.05631	0.74
3335	212589_at	RRAS2	0.42401	0.39032	0.41955	0.00446	0.487
3336	212592_at	IGJ	0.59455	0.66915	0.74457	-0.15002	0.932

3337	212595_s_at	DAZAP2	0.42153	0.48629	0.48476	-0.06323	0.701
3338	212597_s_at	HMGXB4	0.61161	0.46581	0.54849	0.06312	0.426
3339	212600_s_at	UQCRC2	0.57973	0.62566	0.55046	0.02927	0.423
3340	212601_at	ZZEF1	0.064118	0.10561	0.13346	-0.069342	0.656
3341	212603_at	MRPS31	0.31889	0.40991	0.32923	-0.01034	0.536
3342	212608_s_at	NUDT3	0.040558	0.29122	0.19209	-0.151532	0.772
3343	212610_at	PTPN11	0.49139	0.50884	0.57865	-0.08726	0.723
3344	212611_at	DTX4	0.59944	0.68573	0.65905	-0.05961	0.721
3345	212612_at	RCOR1	0.43367	0.49079	0.46739	-0.03372	0.622
3346	212613_at	BTN3A2	0.4729	0.63531	0.48485	-0.01195	0.573
3347	212614_at	ARID5B	0.42639	0.5284	0.34476	0.08163	0.25
3348	212621_at	TMEM194A	0.61642	0.41015	0.56377	0.05265	0.304
3349	212623_at	TMEM41B	0.5662	0.75143	0.64185	-0.07565	0.731
3350	212625_at	STX10	0.49266	0.34952	0.27986	0.2128	0.052
3351	212627_s_at	EXOSC7	0.26779	0.29262	0.23428	0.03351	0.399
3352	212630_at	EXOC3	0.72399	0.67479	0.6641	0.05989	0.34
3353	212634_at	KIAA0776	0.80196	0.79179	0.82587	-0.02391	0.647
3354	212637_s_at	WWP1	0.83662	0.78631	0.83085	0.00577	0.448
3355	212640_at	PTPLB	0.53701	0.41367	0.40232	0.13469	0.127
3356	212642_s_at	HIVEP2	0.2008	0.34689	0.42401	-0.22321	0.928
3357	212643_at	MAPK1IP1L	0.54727	0.52837	0.62428	-0.07701	0.746
3358	212646_at	RFTN1	0.67245	0.75948	0.6533	0.01915	0.432
3359	212649_at	DHX29	0.59524	0.59217	0.66247	-0.06723	0.671
3360	212653_s_at	EHBP1	0.70982	0.71787	0.74688	-0.03706	0.711
3361	212655_at	ZCCHC14	0.067835	0.27681	0.28304	-0.215205	0.882
3362	212656_at	TSFM	0.40772	0.40703	0.28639	0.12133	0.198
3363	212658_at	LHFPL2	0.42861	0.53381	0.44668	-0.01807	0.552
3364	212665_at	TIPARP	0.24296	0.37491	0.42476	-0.1818	0.883
3365	212666_at	SMURF1	0.28779	0.30503	0.27564	0.01215	0.468
3366	212673_at	METAP1	0.35158	0.37355	0.33276	0.01882	0.463
3367	212674_s_at	DHX30	0.78629	0.76597	0.653	0.13329	0.032
3368	212677_s_at	CEP68	0.72775	0.42816	0.37071	0.35704	<0.001
3369	212685_s_at	TBL2	0.48389	0.45936	0.47867	0.00522	0.497
3370	212688_at	PIK3CB	0.59404	0.50256	0.54309	0.05095	0.339
3371	212689_s_at	KDM3A	0.76617	0.16979	0.045022	0.721148	<0.001
3372	212690_at	DDHD2	0.71373	0.75478	0.62001	0.09372	0.164
3373	212692_s_at	LRBA	0.54177	0.71788	0.71003	-0.16826	0.979
3374	212693_at	MDN1	0.64098	0.64939	0.50514	0.13584	0.152
3375	212694_s_at	PCCB	0.56225	0.58028	0.49171	0.07054	0.257
3376	212696_s_at	RNF4	0.57434	0.58784	0.44542	0.12892	0.117
3377	212697_at	FAM134C	0.73824	0.67739	0.75269	-0.01445	0.566
3378	212698_s_at	SEPT10	0.036871	0.33519	0.23131	-0.194439	0.913
3379	212699_at	SCAMP5	0.36238	0.44539	0.39159	-0.02921	0.587
3380	212704_at	ZCCHC11	0.38124	0.65963	0.53284	-0.1516	0.886
3381	212707_s_at	LOC100133005	0.37101	0.14171	0.30532	0.06569	0.308
3382	212708_at	MSL1	0.57495	0.77336	0.79099	-0.21604	1
3383	212712_at	CAMSAP1	0.33694	0.48916	0.38918	-0.05224	0.609
3384	212716_s_at	EIF3K	0.86221	0.83429	0.90242	-0.04021	0.791
3385	212718_at	PAPOLA	0.81593	0.68844	0.70162	0.11431	0.16
3386	212721_at	SFRS12	0.62827	0.60013	0.6505	-0.02223	0.58
3387	212723_at	JMJD6	0.46242	0.42542	0.2981	0.16432	0.132
3388	212726_at	PHF2	0.40544	0.41266	0.53678	-0.13134	0.873
3389	212729_at	DLG3	0.35842	0.55123	0.22278	0.13564	0.229

3390	212731_at	ANKRD46	0.48615	0.63683	0.45	0.03615	0.42
3391	212735_at	KIAA0226	0.08435	0.13601	0.097332	-0.012982	0.548
3392	212739_s_at	NME4	0.61458	0.68853	0.68564	-0.07106	0.714
3393	212742_at	RNF115	0.68775	0.68524	0.49051	0.19724	0.049
3394	212745_s_at	BBS4	0.52926	0.31297	0.19227	0.33699	0.026
3395	212746_s_at	CEP170	0.80059	0.68738	0.7935	0.00709	0.452
3396	212747_at	ANKS1A	0.39798	0.39993	0.4807	-0.08272	0.705
3397	212751_at	UBE2N	0.16275	0.15181	0.17984	-0.01709	0.568
3398	212752_at	CLASP1	0.49762	0.57095	0.56709	-0.06947	0.674
3399	212753_at	PCGF3	0.46198	0.60075	0.46426	-0.00228	0.513
3400	212754_s_at	MON2	0.6476	0.79333	0.57124	0.07636	0.143
3401	212756_s_at	UBR2	0.36135	0.39836	0.49759	-0.13624	0.819
3402	212758_s_at	ZEB1	0.23075	0.4109	0.32106	-0.09031	0.74
3403	212763_at	CAMSAP1L1	0.49131	0.46174	0.34336	0.14795	0.15
3404	212766_s_at	ISG20L2	0.71085	0.58069	0.71129	-0.00044	0.515
3405	212767_at	MTG1	0.42208	0.564	0.52134	-0.09926	0.778
3406	212771_at	FAM171A1	0.12298	0.36897	0.30684	-0.18386	0.901
3407	212773_s_at	TOMM20	0.71762	0.80804	0.80933	-0.09171	0.915
3408	212774_at	ZNF238	0.33274	0.48096	0.38524	-0.0525	0.659
3409	212779_at	KIAA1109	0.58829	0.60209	0.67612	-0.08783	0.833
3410	212780_at	SOS1	0.49096	0.31235	0.5017	-0.01074	0.518
3411	212784_at	CIC	0.60482	0.59239	0.57221	0.03261	0.442
3412	212785_s_at	LARP7	0.41561	0.37133	0.32654	0.08907	0.271
3413	212786_at	CLEC16A	0.57332	0.37134	0.27005	0.30327	0.008
3414	212787_at	YLPM1	0.57289	0.6312	0.74602	-0.17313	0.966
3415	212789_at	NCAPD3	0.72797	0.68525	0.53092	0.19705	0.032
3416	212791_at	C1orf216	0.30664	0.32528	0.47338	-0.16674	0.894
3417	212794_s_at	KIAA1033	0.50725	0.67674	0.64708	-0.13983	0.918
3418	212802_s_at	GAPVD1	0.72151	0.67767	0.62146	0.10005	0.159
3419	212810_s_at	SLC1A4	0.5791	0.61732	0.48853	0.09057	0.297
3420	212813_at	JAM3	0.10603	0.13186	0.10942	-0.00339	0.504
3421	212815_at	ASCC3	0.69142	0.68579	0.4178	0.27362	<0.001
3422	212817_at	DNAJB5	0.13911	0.17513	0.49252	-0.35341	0.986
3423	212819_at	ASB1	0.35026	0.50363	0.56044	-0.21018	0.908
3424	212820_at	DMXL2	0.66866	0.68408	0.70451	-0.03585	0.64
3425	212824_at	FUBP3	0.75658	0.68932	0.80658	-0.05	0.816
3426	212825_at	PAXIP1	0.83302	0.82123	0.78212	0.0509	0.124
3427	212826_s_at	SLC25A6	0.51147	0.52539	0.54742	-0.03595	0.621
3428	212830_at	MEGF9	0.12047	0.17206	0.14193	-0.02146	0.541
3429	212832_s_at	CKAP5	0.68951	0.71337	0.64454	0.04497	0.331
3430	212833_at	SLC25A46	0.6108	0.5579	0.52415	0.08665	0.226
3431	212834_at	DDX52	0.65743	0.38556	0.5913	0.06613	0.246
3432	212836_at	POLD3	0.66006	0.69563	0.59949	0.06057	0.251
3433	212837_at	FAM175B	0.12833	0.55351	0.37727	-0.24894	0.924
3434	212838_at	DNMBP	0.74704	0.76775	0.73333	0.01371	0.455
3435	212841_s_at	PPFIBP2	0.46005	0.36244	0.45952	0.00053	0.507
3436	212846_at	RRP1B	0.74508	0.80228	0.74129	0.00379	0.492
3437	212847_at	FUBP1	0.82608	0.61826	0.6903	0.13578	0.021
3438	212851_at	DCUN1D4	0.28235	0.16696	0.4472	-0.16485	0.853
3439	212858_at	PAQR4	0.55863	0.48174	0.19462	0.36401	0.007
3440	212860_at	ZDHHC18	0.4518	0.59699	0.51009	-0.05829	0.683
3441	212861_at	MFSD5	0.13055	0.29506	0.36679	-0.23624	0.917
3442	212862_at	CDS2	0.33124	0.47371	0.46256	-0.13132	0.858

3443	212867_at	NCOA2	0.79919	0.83309	0.79144	0.00775	0.461
3444	212871_at	MAPKAPK5	0.1478	0.16207	0.19526	-0.04746	0.61
3445	212873_at	HMHA1	0.6534	0.49704	0.43379	0.21961	0.011
3446	212875_s_at	C2CD2	0.11166	0.29097	0.22148	-0.10982	0.791
3447	212876_at	B4GALT4	0.36805	0.29208	0.29256	0.07549	0.327
3448	212877_at	KLC1	0.31126	0.23774	0.37443	-0.06317	0.682
3449	212880_at	WDR7	0.47852	0.4906	0.46403	0.01449	0.472
3450	212881_at	PIAS4	0.13608	0.067257	0.1875	-0.05142	0.641
3451	212885_at	MPHOSPH10	0.44503	0.40745	0.35457	0.09046	0.173
3452	212886_at	CCDC69	0.66896	0.76628	0.7156	-0.04664	0.708
3453	212887_at	SEC23A	0.8387	0.77856	0.82024	0.01846	0.378
3454	212891_s_at	GADD45GIP1	0.74058	0.75273	0.83926	-0.09868	0.824
3455	212893_at	ZZZ3	0.68184	0.6142	0.72102	-0.03918	0.667
3456	212894_at	SUPV3L1	0.24693	0.31374	0.39611	-0.14918	0.845
3457	212896_at	SKIV2L2	0.9082	0.87293	0.82942	0.07878	0.149
3458	212898_at	KIAA0406	0.69751	0.7341	0.77357	-0.07606	0.727
3459	212901_s_at	CSTF2T	0.22071	0.16061	0.22921	-0.0085	0.54
3460	212902_at	SEC24A	0.62711	0.71401	0.70023	-0.07312	0.785
3461	212904_at	LRRC47	0.62581	0.64034	0.61654	0.00927	0.468
3462	212906_at	GRAMD1B	0.55349	0.60391	0.64398	-0.09049	0.86
3463	212907_at	SLC30A1	0.22614	0.31451	0.3195	-0.09336	0.706
3464	212908_at	DNAJC16	0.46035	0.63486	0.53548	-0.07513	0.764
3465	212910_at	THAP11	0.45592	0.2323	0.47174	-0.01582	0.543
3466	212916_at	PHF8	0.30909	0.12791	0.20148	0.10761	0.223
3467	212918_at	RECQL	0.62257	0.77893	0.78795	-0.16538	0.979
3468	212919_at	DCP2	0.61497	0.52076	0.48407	0.1309	0.155
3469	212926_at	SMC5	0.58614	0.60236	0.58631	-0.00017	0.501
3470	212928_at	TSPYL4	0.48399	0.47433	0.55075	-0.06676	0.717
3471	212929_s_at	FAM21A	0.5732	0.63687	0.59091	-0.01771	0.564
3472	212930_at	ATP2B1	0.60047	0.50539	0.60045	2E-05	0.511
3473	212931_at	TCF20	0.45589	0.43523	0.61354	-0.15765	0.86
3474	212934_at	UBXN2B	0.23692	0.55781	0.26624	-0.02932	0.602
3475	212936_at	FAM172A	0.73774	0.57223	0.48204	0.2557	<0.001
3476	212943_at	KIAA0528	0.76414	0.71281	0.69614	0.068	0.129
3477	212944_at	SLC5A3	0.62226	0.40041	0.56439	0.05787	0.301
3478	212945_s_at	MGA	0.3077	0.56586	0.53208	-0.22438	0.876
3479	212947_at	SLC9A8	0.40912	0.39398	0.31566	0.09346	0.313
3480	212948_at	CAMTA2	0.60262	0.61365	0.40917	0.19345	0.071
3481	212949_at	NCAPH	0.79341	0.6509	0.58034	0.21307	0.023
3482	212954_at	DYRK4	0.44202	0.46524	0.42624	0.01578	0.461
3483	212955_s_at	POLR2I	0.84648	0.80866	0.85778	-0.0113	0.622
3484	212959_s_at	GNPTAB	0.35514	0.6578	0.50814	-0.153	0.834
3485	212963_at	TM2D1	0.34137	0.5412	0.49498	-0.15361	0.931
3486	212964_at	HIC2	0.061457	0.019019	0.071685	-0.010228	0.495
3487	212973_at	RPIA	0.68612	0.55427	0.37319	0.31293	<0.001
3488	212977_at	CXCR7	0.53619	0.64971	0.66761	-0.13142	0.833
3489	212978_at	LRRC8B	0.4084	0.27062	0.36187	0.04653	0.353
3490	212979_s_at	FAM115A	0.30004	0.19956	0.29679	0.00325	0.461
3491	212983_at	HRAS	0.64789	0.68674	0.62756	0.02033	0.378
3492	212984_at	ATF2	0.7983	0.84131	0.8793	-0.081	0.874
3493	212986_s_at	TLK2	0.71136	0.58259	0.62765	0.08371	0.203
3494	212989_at	SGMS1	0.39544	0.5633	0.58627	-0.19083	0.957
3495	212990_at	SYNJ1	0.57245	0.33115	0.44585	0.1266	0.163

3496	212994_at	THOC2	0.68275	0.67078	0.63191	0.05084	0.309
3497	213000_at	MORC3	0.74032	0.73389	0.7234	0.01692	0.416
3498	213002_at	MARCKS	0.51392	0.66626	0.59221	-0.07829	0.706
3499	213008_at	FANCI	0.80785	0.80133	0.79858	0.00927	0.459
3500	213009_s_at	TRIM37	0.78669	0.77919	0.79748	-0.01079	0.566
3501	213010_at	PRKCDBP	0.26909	0.52817	0.48114	-0.21205	0.925
3502	213011_s_at	TPI1	0.68931	0.67154	0.62153	0.06778	0.333
3503	213012_at	NEDD4	0.26674	0.46954	0.44928	-0.18254	0.89
3504	213016_at	BBX	0.57755	0.63102	0.54997	0.02758	0.394
3505	213017_at	ABHD3	0.75171	0.68988	0.60158	0.15013	0.033
3506	213019_at	RANBP6	0.76916	0.72304	0.72317	0.04599	0.326
3507	213025_at	THUMPD1	0.78261	0.76672	0.77223	0.01038	0.462
3508	213026_at	ATG12	0.63497	0.55928	0.69909	-0.06412	0.77
3509	213031_s_at	WDR73	0.67323	0.47757	0.51978	0.15345	0.096
3510	213035_at	ANKRD28	0.043151	0.21806	0.069389	-0.026238	0.564
3511	213038_at	RNF19B	0.74796	0.79178	0.73334	0.01462	0.441
3512	213039_at	ARHGEF18	0.50252	0.45188	0.38084	0.12168	0.184
3513	213041_s_at	ATP5D	0.71922	0.65436	0.68094	0.03828	0.381
3514	213043_s_at	MED24	0.75096	0.73142	0.75599	-0.00503	0.522
3515	213044_at	ROCK1	0.77536	0.7569	0.74385	0.03151	0.342
3516	213046_at	PABPN1	0.42292	0.35757	0.32419	0.09873	0.302
3517	213048_s_at	SET	0.51801	0.64804	0.65601	-0.138	0.852
3518	213052_at	PRKAR2A	0.67236	0.67892	0.68063	-0.00827	0.518
3519	213054_at	HAUS5	0.22555	0.14665	0.17587	0.04968	0.37
3520	213058_at	TTC28	0.098433	0.20892	0.14543	-0.046997	0.625
3521	213060_s_at	CHI3L2	0.032735	0.17895	0.2278	-0.195065	0.975
3522	213061_s_at	NTAN1	0.81104	0.76171	0.7408	0.07024	0.161
3523	213065_at	ZFC3H1	0.61756	0.57115	0.64792	-0.03036	0.645
3524	213070_at	PIK3C2A	0.78111	0.62529	0.77183	0.00928	0.452
3525	213073_at	ZFYVE26	0.62199	0.7732	0.72264	-0.10065	0.814
3526	213076_at	ITPKC	0.48994	0.46811	0.43411	0.05583	0.273
3527	213077_at	YTHDC2	0.67596	0.67637	0.66129	0.01467	0.453
3528	213079_at	TSR2	0.28216	0.34834	0.3657	-0.08354	0.7
3529	213086_s_at	CSNK1A1	0.69781	0.66928	0.49805	0.19976	0.009
3530	213088_s_at	DNAJC9	0.54922	0.64748	0.66731	-0.11809	0.812
3531	213090_s_at	TAF4	0.48295	0.5216	0.49077	-0.00782	0.557
3532	213097_s_at	DNAJC2	0.79748	0.7552	0.66835	0.12913	0.07
3533	213098_at	RQCD1	0.12622	0.37218	0.30766	-0.18144	0.87
3534	213101_s_at	ACTR3	0.86685	0.87337	0.87273	-0.00588	0.562
3535	213103_at	STARD13	0.37967	0.31584	0.41085	-0.03118	0.59
3536	213105_s_at	C16orf42	0.80202	0.76079	0.73553	0.06649	0.229
3537	213106_at	ATP8A1	0.50147	0.48862	0.53076	-0.02929	0.585
3538	213111_at	PIKFYVE	0.77965	0.80648	0.78656	-0.00691	0.605
3539	213113_s_at	SLC43A3	0.64315	0.7516	0.49357	0.14958	0.114
3540	213115_at	ATG4A	0.29278	0.2905	0.45553	-0.16275	0.913
3541	213117_at	KLHL9	0.39278	0.29293	0.4376	-0.04482	0.658
3542	213120_at	UHRF1BP1L	0.42369	0.31533	0.28422	0.13947	0.175
3543	213122_at	TSPYL5	0.0031528	0.083661	0.045066	-0.0419132	0.597
3544	213126_at	MED8	0.018354	0.092713	0.040873	-0.022519	0.562
3545	213132_s_at	MCAT	0.28597	0.29441	0.33068	-0.04471	0.632
3546	213133_s_at	GCSH	0.58802	0.64116	0.68936	-0.10134	0.797
3547	213137_s_at	PTPN2	0.54312	0.53733	0.60807	-0.06495	0.711
3548	213138_at	ARID5A	0.30345	0.41742	0.35678	-0.05333	0.645

3549	213140_s_at	SS18L1	0.54343	0.42146	0.48792	0.05551	0.259
3550	213141_at	PSKH1	0.050478	0.024104	0.09068	-0.040202	0.673
3551	213145_at	FBXL14	0.4535	0.5024	0.46425	-0.01075	0.527
3552	213151_s_at	SEPT7	0.38676	0.53438	0.57795	-0.19119	0.958
3553	213152_s_at	SFRS2B	0.33042	0.37563	0.25652	0.0739	0.303
3554	213153_at	SETD1B	0.45928	0.39405	0.47628	-0.017	0.585
3555	213154_s_at	BICD2	0.30483	0.48597	0.33266	-0.02783	0.565
3556	213160_at	DOCK2	0.61389	0.66209	0.55382	0.06007	0.324
3557	213161_at	C9orf97	0.67991	0.74768	0.69422	-0.01431	0.562
3558	213168_at	SP3	0.75105	0.79861	0.8279	-0.07685	0.819
3559	213170_at	GPX7	0.043821	0.21725	0.2683	-0.224479	0.959
3560	213172_at	TTC9	0.45851	0.34861	0.37811	0.0804	0.308
3561	213188_s_at	MINA	0.6583	0.57612	0.51597	0.14233	0.061
3562	213191_at	TICAM1	0.0086809	0.035399	0.20275	-0.1940691	0.763
3563	213194_at	ROBO1	0.20092	0.24079	0.27902	-0.0781	0.72
3564	213203_at	SNAPC5	0.74989	0.66849	0.69399	0.0559	0.301
3565	213206_at	GOSR2	0.66766	0.60621	0.69053	-0.02287	0.609
3566	213213_at	DIDO1	0.24062	0.17137	0.17021	0.07041	0.349
3567	213216_at	OTUD3	0.14387	0.1134	0.21953	-0.07566	0.58
3568	213224_s_at	NCRNA00081	0.72044	0.72168	0.66846	0.05198	0.293
3569	213225_at	PPM1B	0.79661	0.619	0.70291	0.0937	0.128
3570	213226_at	CCNA2	0.78054	0.74127	0.72316	0.05738	0.268
3571	213227_at	PGRMC2	0.50731	0.40522	0.56048	-0.05317	0.7
3572	213237_at	C16orf88	0.508	0.43033	0.31113	0.19687	0.124
3573	213238_at	ATP10D	0.71464	0.58686	0.73042	-0.01578	0.597
3574	213239_at	PIBF1	0.62696	0.43999	0.61524	0.01172	0.471
3575	213246_at	C14orf109	0.45061	0.53122	0.59327	-0.14266	0.808
3576	213251_at	SMARCA5	0.74502	0.72736	0.76452	-0.0195	0.618
3577	213253_at	SMC2	0.74685	0.77353	0.82408	-0.07723	0.915
3578	213254_at	TNRC6B	0.50143	0.40652	0.34029	0.16114	0.134
3579	213256_at	MARCH3	0.42206	0.56071	0.46272	-0.04066	0.673
3580	213262_at	SACS	0.79501	0.80355	0.66976	0.12525	0.073
3581	213266_at	TUBGCP4	0.33609	0.21796	0.18446	0.15163	0.178
3582	213269_at	ZNF248	0.33761	0.13593	0.11935	0.21826	0.058
3583	213272_s_at	TMEM159	0.68616	0.47645	0.48546	0.2007	0.039
3584	213274_s_at	CTSB	0.48594	0.35046	0.30827	0.17767	0.089
3585	213278_at	MTMR9	0.30033	0.36864	0.42116	-0.12083	0.875
3586	213279_at	DHRS1	0.32759	0.48461	0.3519	-0.02431	0.598
3587	213282_at	APOOL	0.47197	0.63961	0.54248	-0.07051	0.729
3588	213283_s_at	SALL2	0.40719	0.43877	0.41967	-0.01248	0.553
3589	213287_s_at	KRT10	0.77167	0.80936	0.84691	-0.07524	0.711
3590	213293_s_at	TRIM22	0.66311	0.68966	0.41116	0.25195	0.004
3591	213294_at	EIF2AK2	0.7032	0.84353	0.80969	-0.10649	0.874
3592	213297_at	RMND5B	0.69845	0.64451	0.69994	-0.00149	0.541
3593	213300_at	ATG2A	0.079103	0.089175	0.1325	-0.053397	0.612
3594	213302_at	PFAS	0.73783	0.72296	0.63988	0.09795	0.17
3595	213304_at	FAM179B	0.65605	0.70796	0.78462	-0.12857	0.862
3596	213305_s_at	PPP2R5C	0.74264	0.81321	0.61004	0.1326	0.01
3597	213310_at	EIF2C2	0.32148	0.51118	0.27477	0.04671	0.36
3598	213312_at	C6orf162	0.61425	0.62135	0.46502	0.14923	0.066
3599	213318_s_at	BAT3	0.73643	0.82462	0.78312	-0.04669	0.688
3600	213320_at	PRMT3	0.82345	0.8525	0.79629	0.02716	0.343
3601	213322_at	C6orf130	0.50117	0.54827	0.5023	-0.00113	0.497

3602	213326_at	VAMP1	0.3652	0.28218	0.38066	-0.01546	0.553
3603	213327_s_at	USP12	0.58726	0.69134	0.61078	-0.02352	0.616
3604	213328_at	NEK1	0.22824	0.28298	0.081165	0.147075	0.152
3605	213333_at	MDH2	0.5826	0.60549	0.54417	0.03843	0.396
3606	213340_s_at	KIAA0495	0.57693	0.4452	0.41376	0.16317	0.106
3607	213341_at	FEM1C	0.48915	0.42316	0.4999	-0.01075	0.529
3608	213346_at	C13orf27	0.45254	0.39226	0.43014	0.0224	0.477
3609	213350_at	RPS11	0.59569	0.59237	0.66207	-0.06638	0.735
3610	213351_s_at	TMCC1	0.1928	0.091358	0.13872	0.05408	0.617
3611	213353_at	ABCA5	0.60931	0.63378	0.66024	-0.05093	0.728
3612	213357_at	GTF2H5	0.79843	0.76039	0.8056	-0.00717	0.532
3613	213361_at	TDRD7	0.66934	0.73074	0.56225	0.10709	0.133
3614	213365_at	ERI2	0.61808	0.57926	0.47626	0.14182	0.138
3615	213370_s_at	SFMBT1	0.25122	0.31524	0.26514	-0.01392	0.568
3616	213372_at	PAQR3	0.67634	0.63202	0.71402	-0.03768	0.652
3617	213373_s_at	CASP8	0.56471	0.45553	0.61651	-0.0518	0.7
3618	213376_at	ZBTB1	0.65088	0.67668	0.58551	0.06537	0.235
3619	213379_at	COQ2	0.57922	0.55447	0.50901	0.07021	0.247
3620	213387_at	ATAD2B	0.20596	0.28462	0.32255	-0.11659	0.756
3621	213390_at	ZC3H4	0.43483	0.39974	0.31849	0.11634	0.152
3622	213391_at	DPY19L4	0.28476	0.36869	0.38627	-0.10151	0.797
3623	213398_s_at	SDR39U1	0.35746	0.40734	0.4458	-0.08834	0.695
3624	213402_at	ZNF787	0.10434	0.27755	0.096971	0.007369	0.485
3625	213405_at	RAB22A	0.6872	0.76948	0.73369	-0.04649	0.763
3626	213408_s_at	PI4KA	0.61336	0.69332	0.61353	-0.00017	0.48
3627	213409_s_at	RHEB	0.77044	0.72198	0.68174	0.0887	0.204
3628	213410_at	C10orf137	0.60413	0.54021	0.76993	-0.1658	0.883
3629	213414_s_at	RPS19	0.94731	0.92231	0.93847	0.00884	0.253
3630	213415_at	CLIC2	0.43539	0.52075	0.4203	0.01509	0.482
3631	213420_at	DHX57	0.44873	0.43291	0.32068	0.12805	0.2
3632	213427_at	RPP40	0.65867	0.68991	0.70198	-0.04331	0.652
3633	213436_at	CNR1	0.52483	0.61436	0.54609	-0.02126	0.563
3634	213445_at	ZC3H3	0.29259	0.40823	0.35838	-0.06579	0.674
3635	213446_s_at	IQGAP1	0.43863	0.57975	0.49665	-0.05802	0.711
3636	213447_at	IPW	0.28423	0.53741	0.44412	-0.15989	0.923
3637	213449_at	POP1	0.52999	0.44619	0.37278	0.15721	0.106
3638	213452_at	ZNF184	0.54283	0.60048	0.67113	-0.1283	0.898
3639	213454_at	APITD1	0.59815	0.68272	0.65129	-0.05314	0.761
3640	213455_at	FAM114A1	0.30679	0.49017	0.21425	0.09254	0.309
3641	213457_at	MFHAS1	0.53415	0.62296	0.43023	0.10392	0.221
3642	213461_at	NUDT21	0.69214	0.79108	0.78343	-0.09129	0.86
3643	213469_at	PGAP1	0.46846	0.45326	0.4633	0.00516	0.477
3644	213474_at	KCTD7	0.17412	0.045413	0.093969	0.080151	0.373
3645	213475_s_at	ITGAL	0.46276	0.54354	0.57442	-0.11166	0.777
3646	213483_at	PPWD1	0.61601	0.44435	0.3486	0.26741	0.004
3647	213485_s_at	ABCC10	0.54604	0.58137	0.53828	0.00776	0.452
3648	213494_s_at	YY1	0.39686	0.48428	0.36704	0.02982	0.424
3649	213497_at	ABTB2	0.42287	0.54347	0.57373	-0.15086	0.942
3650	213508_at	C14orf147	0.30724	0.48517	0.5203	-0.21306	0.91
3651	213517_at	PCBP2	0.32455	0.25237	0.39196	-0.06741	0.673
3652	213521_at	PTPN18	0.57255	0.45609	0.39406	0.17849	0.125
3653	213523_at	CCNE1	0.49695	0.43652	0.48663	0.01032	0.468
3654	213526_s_at	LIN37	0.65646	0.37981	0.71605	-0.05959	0.723

3655	213527_s_at	ZNF688	0.62511	0.61592	0.50894	0.11617	0.155
3656	213528_at	C1orf156	0.31406	0.34093	0.22329	0.09077	0.297
3657	213531_s_at	RAB3GAP1	0.10935	0.37146	0.25338	-0.14403	0.874
3658	213534_s_at	PASK	0.10081	0.23909	0.30906	-0.20825	0.933
3659	213540_at	HSD17B8	0.62482	0.5507	0.61225	0.01257	0.459
3660	213546_at	DKFZP586I1420	0.34351	0.41116	0.27594	0.06757	0.323
3661	213548_s_at	CDV3	0.82871	0.79053	0.79754	0.03117	0.351
3662	213549_at	SLC18A2	0.56238	0.40931	0.35233	0.21005	0.081
3663	213560_at	GADD45B	0.53851	0.58212	0.53803	0.00048	0.511
3664	213566_at	RNASE6	0.39154	0.62705	0.60799	-0.21645	0.981
3665	213568_at	OSR2	0.34046	0.37378	0.44686	-0.1064	0.715
3666	213577_at	SQLE	0.61144	0.51183	0.46323	0.14821	0.076
3667	213587_s_at	ATP6V0E2	0.31734	0.50984	0.47192	-0.15458	0.855
3668	213590_at	LOC100133772	0.037353	0.25713	0.24836	-0.211007	0.942
3669	213593_s_at	TRA2A	0.16629	0.41716	0.31017	-0.14388	0.805
3670	213599_at	OIP5	0.77261	0.78599	0.50119	0.27142	<0.001
3671	213603_s_at	RAC2	0.73071	0.72209	0.6896	0.04111	0.343
3672	213604_at	TCEB3	0.55409	0.56256	0.60926	-0.05517	0.69
3673	213605_s_at	LOC100134401	0.14199	0.3668	0.36343	-0.22144	0.946
3674	213606_s_at	ARHGDIA	0.67806	0.38136	0.39296	0.2851	0.118
3675	213608_s_at	SRRD	0.51461	0.50862	0.47148	0.04313	0.388
3676	213618_at	ARAP2	0.39124	0.69718	0.5614	-0.17016	0.931
3677	213622_at	COL9A2	0.52496	0.65331	0.58543	-0.06047	0.696
3678	213623_at	KIF3A	0.35508	0.41501	0.41996	-0.06488	0.679
3679	213625_at	ZKSCAN4	0.50035	0.35271	0.335	0.16535	0.121
3680	213626_at	CBR4	0.60013	0.72536	0.68158	-0.08145	0.832
3681	213627_at	MAGED2	0.31498	0.39093	0.23348	0.0815	0.329
3682	213634_s_at	TRMU	0.33699	0.16057	0.30488	0.03211	0.439
3683	213638_at	PHACTR1	0.547	0.68029	0.60472	-0.05772	0.726
3684	213642_at	RPL27	0.69817	0.73611	0.74891	-0.05074	0.761
3685	213647_at	DNA2	0.63973	0.69147	0.829	-0.18927	0.967
3686	213650_at	GOLGA8A	0.56216	0.40027	0.4211	0.14106	0.121
3687	213654_at	TAF5L	0.010798	0.24859	0.17035	-0.159552	0.828
3688	213664_at	SLC1A1	0.42008	0.45629	0.41159	0.00849	0.492
3689	213671_s_at	MARS	0.67714	0.74318	0.66861	0.00853	0.465
3690	213677_s_at	PMS1	0.58656	0.39667	0.52419	0.06237	0.316
3691	213679_at	TTC30A	0.34488	0.49316	0.3579	-0.01302	0.541
3692	213687_s_at	RPL35A	0.93026	0.93563	0.92602	0.00424	0.4
3693	213694_at	RSBN1	0.47341	0.33709	0.44437	0.02904	0.413
3694	213699_s_at	YWHAQ	0.62919	0.73364	0.64839	-0.0192	0.567
3695	213701_at	C12orf29	0.58941	0.62413	0.62633	-0.03692	0.621
3696	213703_at	LOC150759	0.069048	0.23744	0.21988	-0.150832	0.868
3697	213704_at	RABGGTB	0.69131	0.74496	0.6739	0.01741	0.448
3698	213705_at	MAT2A	0.80537	0.78119	0.75118	0.05419	0.232
3699	213738_s_at	ATP5A1	0.91289	0.91138	0.91479	-0.0019	0.556
3700	213742_at	SFRS11	0.29061	0.19592	0.19497	0.09564	0.254
3701	213743_at	CCNT2	0.55277	0.61092	0.66747	-0.1147	0.924
3702	213746_s_at	FLNA	0.493	0.6901	0.64152	-0.14852	0.907
3703	213748_at	TRIM66	0.36267	0.29972	0.41325	-0.05058	0.648
3704	213757_at	EIF5A	0.34958	0.22244	0.40429	-0.05471	0.636
3705	213761_at	MDM1	0.77558	0.73504	0.75941	0.01617	0.4
3706	213779_at	EMID1	0.52403	0.58204	0.64683	-0.1228	0.873
3707	213787_s_at	EBP	0.51157	0.68142	0.56686	-0.05529	0.613

3708	213792_s_at	INSR	0.12738	0.37874	0.34336	-0.21598	0.864
3709	213793_s_at	HOMER1	0.31221	0.54927	0.48459	-0.17238	0.901
3710	213794_s_at	NGDN	0.63468	0.60981	0.62508	0.0096	0.494
3711	213798_s_at	CAP1	0.61719	0.4848	0.62149	-0.0043	0.511
3712	213803_at	KPNB1	0.65718	0.80725	0.66351	-0.00633	0.561
3713	213804_at	INPP5B	0.31218	0.38024	0.48959	-0.17741	0.888
3714	213810_s_at	AKIRIN2	0.4803	0.51847	0.50294	-0.02264	0.589
3715	213812_s_at	CAMKK2	0.2841	0.3969	0.41611	-0.13201	0.826
3716	213826_s_at	LOC100133109	0.65893	0.62475	0.62389	0.03504	0.375
3717	213846_at	COX7C	0.70406	0.68579	0.74944	-0.04538	0.634
3718	213850_s_at	SFRS2IP	0.75868	0.53524	0.69508	0.0636	0.293
3719	213851_at	TMEM110	0.095694	0.19306	0.32743	-0.231736	0.936
3720	213853_at	DNAJC24	0.2442	0.16037	0.18299	0.06121	0.33
3721	213861_s_at	FAM119B	0.31326	0.37986	0.2788	0.03446	0.416
3722	213863_s_at	OAZ3	0.28748	0.3606	0.48319	-0.19571	0.946
3723	213872_at	C6orf62	0.61683	0.53398	0.58057	0.03626	0.385
3724	213878_at	PYROXD1	0.592	0.55334	0.6198	-0.0278	0.62
3725	213879_at	SUMO2	0.79511	0.81684	0.80742	-0.01231	0.601
3726	213887_s_at	POLR2E	0.72221	0.66966	0.75611	-0.0339	0.652
3727	213888_s_at	LOC100133233	0.39347	0.52656	0.45057	-0.0571	0.667
3728	213891_s_at	TCF4	0.19575	0.28316	0.34253	-0.14678	0.867
3729	213897_s_at	MRPL23	0.82456	0.81657	0.88594	-0.06138	0.833
3730	213900_at	C9orf61	0.25862	0.25111	0.27388	-0.01526	0.542
3731	213906_at	MYBL1	0.098627	0.16725	0.35323	-0.254603	0.976
3732	213908_at	WHAMML1	0.2834	0.1066	0.35472	-0.07132	0.729
3733	213916_at	ZNF20	0.32617	0.29932	0.23052	0.09565	0.283
3734	213919_at	DNAJC4	0.59526	0.37699	0.4877	0.10756	0.156
3735	213922_at	TTBK2	0.47054	0.17763	0.40154	0.069	0.354
3736	213923_at	RAP2B	0.29971	0.46387	0.28925	0.01046	0.469
3737	213927_at	MAP3K9	0.27835	0.54395	0.52299	-0.24464	0.888
3738	213934_s_at	ZNF23	0.099087	0.043586	0.013586	0.085501	0.282
3739	213937_s_at	FTSJ1	0.31411	0.35494	0.51947	-0.20536	0.905
3740	213940_s_at	FNBP1	0.28074	0.62459	0.45659	-0.17585	0.968
3741	213951_s_at	PSMC3IP	0.8164	0.5001	0.53269	0.28371	0.001
3742	213954_at	FAM169A	0.025109	0.15196	0.25777	-0.232661	0.952
3743	213959_s_at	RPGRIP1L	0.43444	0.47873	0.38839	0.04605	0.376
3744	213963_s_at	SAP30	0.2876	0.41875	0.33866	-0.05106	0.632
3745	213970_at	RABL3	0.005478	0.084251	0.017242	-0.011764	0.524
3746	213974_at	ADAMTSL3	0.35641	0.22555	0.26786	0.08855	0.281
3747	213979_s_at	CTBP1	0.18194	0.14602	0.2928	-0.11086	0.738
3748	213982_s_at	RABGAP1L	0.59017	0.68226	0.6761	-0.08593	0.814
3749	213999_at	YIPF4	0.55285	0.7503	0.68792	-0.13507	0.836
3750	214004_s_at	VGLL4	0.26077	0.14651	0.20638	0.05439	0.378
3751	214006_s_at	GGCX	0.56275	0.55772	0.62965	-0.0669	0.696
3752	214007_s_at	TWF1	0.45972	0.51739	0.47335	-0.01363	0.569
3753	214011_s_at	NOP16	0.85737	0.78657	0.758	0.09937	0.019
3754	214022_s_at	IFITM1	0.81559	0.77812	0.77714	0.03845	0.281
3755	214030_at	CRYBG3	0.52287	0.65425	0.54107	-0.0182	0.587
3756	214036_at	EFNA5	0.17997	0.32474	0.34567	-0.1657	0.91
3757	214039_s_at	LAPTM4B	0.10343	0.19988	0.30758	-0.20415	0.965
3758	214042_s_at	RPL22	0.8231	0.80623	0.85405	-0.03095	0.673
3759	214045_at	LIAS	0.061455	0.16118	0.077222	-0.015767	0.564
3760	214048_at	MBD4	0.57115	0.56712	0.65549	-0.08434	0.796

3761	214051_at	TMSB15B	0.32997	0.41072	0.38342	-0.05345	0.699
3762	214057_at	MCL1	0.59757	0.75038	0.68623	-0.08866	0.769
3763	214060_at	SSBP1	0.21789	0.10347	0.19201	0.02588	0.434
3764	214061_at	WDR67	0.6836	0.41385	0.5498	0.1338	0.077
3765	214075_at	NENF	0.3259	0.29819	0.43015	-0.10425	0.792
3766	214079_at	DHRS2	0.22535	0.28799	0.31284	-0.08749	0.761
3767	214083_at	LOC100132532	0.50306	0.70299	0.69508	-0.19202	0.972
3768	214086_s_at	PARP2	0.44566	0.20535	0.45668	-0.01102	0.556
3769	214096_s_at	SHMT2	0.74756	0.73573	0.72952	0.01804	0.443
3770	214101_s_at	NPEPPS	0.73857	0.71665	0.67	0.06857	0.199
3771	214106_s_at	GMDS	0.13895	0.21508	0.33558	-0.19663	0.929
3772	214112_s_at	CXorf40A	0.63271	0.47414	0.56291	0.0698	0.251
3773	214113_s_at	RBM8A	0.61946	0.7121	0.62774	-0.00828	0.536
3774	214123_s_at	C4orf10	0.55415	0.59084	0.35311	0.20104	0.065
3775	214126_at	MCART1	0.075915	0.14425	0.081285	-0.00537	0.526
3776	214132_at	ATP5C1	0.19404	0.15344	0.076773	0.117267	0.228
3777	214144_at	POLR2D	0.33485	0.34248	0.32848	0.00637	0.473
3778	214148_at	FOXM1	0.12463	0.11395	0.10935	0.01528	0.524
3779	214152_at	CCPG1	0.7193	0.71329	0.79232	-0.07302	0.807
3780	214155_s_at	LARP4	0.84163	0.84752	0.87511	-0.03348	0.723
3781	214167_s_at	RPLP0	0.95077	0.9427	0.94601	0.00476	0.332
3782	214179_s_at	NFE2L1	0.62752	0.57108	0.48374	0.14378	0.052
3783	214182_at	LOC100132430	0.52455	0.54035	0.41907	0.10548	0.209
3784	214186_s_at	HCG26	0.33289	0.43622	0.45379	-0.1209	0.807
3785	214202_at	PGGT1B	0.38469	0.27575	0.3927	-0.00801	0.506
3786	214218_s_at	XIST	8.27E-005	0.29272	0.1566	-0.156517317	0.88
3787	214221_at	ALMS1	0.34916	0.33133	0.23414	0.11502	0.174
3788	214224_s_at	PIN4	0.66549	0.74126	0.7425	-0.07701	0.716
3789	214231_s_at	KIAA0564	0.37481	0.3414	0.47125	-0.09644	0.719
3790	214264_s_at	C14orf143	0.60516	0.68734	0.70254	-0.09738	0.784
3791	214274_s_at	ACAA1	0.59871	0.68382	0.65763	-0.05892	0.705
3792	214281_s_at	RCHY1	0.46701	0.5369	0.39945	0.06756	0.36
3793	214290_s_at	HIST2H2AA3	0.49803	0.68722	0.72527	-0.22724	0.969
3794	214291_at	LOC729046	0.020586	0.016465	0.036215	-0.015629	0.536
3795	214305_s_at	SF3B1	0.18903	0.25268	0.37016	-0.18113	0.92
3796	214313_s_at	EIF5B	0.74493	0.68012	0.73589	0.00904	0.464
3797	214328_s_at	HSP90AA1	0.86649	0.88863	0.90116	-0.03467	0.733
3798	214339_s_at	MAP4K1	0.7555	0.68492	0.72984	0.02566	0.343
3799	214352_s_at	KRAS	0.61125	0.66877	0.6304	-0.01915	0.593
3800	214356_s_at	KIAA0368	0.34025	0.59342	0.53327	-0.19302	0.943
3801	214364_at	MTERFD2	0.52398	0.32606	0.43697	0.08701	0.261
3802	214366_s_at	ALOX5	0.25456	0.29737	0.35622	-0.10166	0.8
3803	214377_s_at	CTRL	0.17921	0.19514	0.11992	0.05929	0.323
3804	214427_at	NOP2	0.70432	0.71713	0.66842	0.0359	0.292
3805	214429_at	MTMR6	0.6999	0.71656	0.69066	0.00924	0.484
3806	214430_at	GLA	0.4677	0.60798	0.62571	-0.15801	0.878
3807	214431_at	GMPS	0.73445	0.60823	0.50637	0.22808	0.001
3808	214440_at	NAT1	0.74058	0.48118	0.5747	0.16588	0.038
3809	214441_at	STX6	0.50335	0.47964	0.38806	0.11529	0.158
3810	214446_at	ELL2	0.64267	0.72265	0.70883	-0.06616	0.793
3811	214447_at	ETS1	0.43447	0.51785	0.57638	-0.14191	0.902
3812	214452_at	BCAT1	0.48386	0.48357	0.38852	0.09534	0.247
3813	214453_s_at	IFI44	0.60296	0.69446	0.58641	0.01655	0.446

3814	214455_at	HIST1H2BC	0.54257	0.59926	0.50436	0.03821	0.396
3815	214467_at	GPR65	0.31917	0.25185	0.47951	-0.16034	0.851
3816	214482_at	ZBTB25	0.48074	0.27098	0.28649	0.19425	0.14
3817	214484_s_at	SIGMAR1	0.11516	0.15108	0.28173	-0.16657	0.868
3818	214507_s_at	EXOSC2	0.73657	0.57458	0.42715	0.30942	<0.001
3819	214513_s_at	CREB1	0.45879	0.40451	0.56013	-0.10134	0.805
3820	214519_s_at	RLN2	0.43329	0.57345	0.56621	-0.13292	0.834
3821	214527_s_at	PQBP1	0.64422	0.68391	0.69868	-0.05446	0.684
3822	214529_at	TSHB	0.23519	0.086386	0.078823	0.156367	0.19
3823	214569_at	IFNA5	0.23134	0.26867	0.21513	0.01621	0.443
3824	214583_at	RSC1A1	0.65393	0.46308	0.55721	0.09672	0.276
3825	214585_s_at	VPS52	0.52834	0.49064	0.47275	0.05559	0.325
3826	214597_at	SSTR2	0.51672	0.50446	0.62581	-0.10909	0.828
3827	214599_at	IVL	0.48035	0.48522	0.53128	-0.05093	0.646
3828	214614_at	MNX1	0.77606	0.67359	0.73216	0.0439	0.258
3829	214615_at	P2RY10	0.52184	0.49985	0.43926	0.08258	0.259
3830	214617_at	PRF1	0.21111	0.3421	0.43133	-0.22022	0.904
3831	214626_s_at	GANAB	0.47755	0.3439	0.34706	0.13049	0.211
3832	214657_s_at	NCRNA00084	0.34007	0.46193	0.49182	-0.15175	0.902
3833	214658_at	TMED7	0.82172	0.78308	0.81459	0.00713	0.444
3834	214661_s_at	NOP14	0.55076	0.56007	0.62613	-0.07537	0.682
3835	214662_at	WDR43	0.72938	0.71723	0.71081	0.01857	0.446
3836	214668_at	C13orf1	0.4629	0.44488	0.52744	-0.06454	0.678
3837	214670_at	ZKSCAN1	0.83753	0.75522	0.72045	0.11708	0.038
3838	214672_at	TTLL5	0.52324	0.39216	0.48239	0.04085	0.5
3839	214681_at	GK	0.59182	0.41697	0.48926	0.10256	0.196
3840	214683_s_at	CLK1	0.72275	0.4806	0.56806	0.15469	0.07
3841	214686_at	ZNF266	0.19451	0.39987	0.48857	-0.29406	0.979
3842	214688_at	TLE4	0.099091	0.15772	0.14263	-0.043539	0.664
3843	214690_at	TAF1B	0.18921	0.24529	0.26098	-0.07177	0.743
3844	214696_at	C17orf91	0.81356	0.66252	0.60935	0.20421	0.03
3845	214697_s_at	ROD1	0.68369	0.77027	0.62419	0.0595	0.159
3846	214703_s_at	MAN2B2	0.24847	0.42261	0.26659	-0.01812	0.525
3847	214709_s_at	KTN1	0.60321	0.43821	0.61663	-0.01342	0.617
3848	214710_s_at	CCNB1	0.78926	0.77383	0.18585	0.60341	<0.001
3849	214711_at	GATC	0.28765	0.25002	0.19372	0.09393	0.235
3850	214714_at	ZNF394	0.64987	0.21158	0.50856	0.14131	0.159
3851	214717_at	DKFZp434H1419	0.074046	0.19091	0.14755	-0.073504	0.663
3852	214719_at	SLC46A3	0.48502	0.4973	0.61225	-0.12723	0.828
3853	214722_at	NOTCH2NL	0.12787	0.33856	0.33386	-0.20599	0.91
3854	214729_at	TWISTNB	0.26879	0.30197	0.43758	-0.16879	0.918
3855	214731_at	CTTNBP2NL	0.16238	0.227	0.46567	-0.30329	0.986
3856	214735_at	IPCEF1	0.60167	0.72413	0.6933	-0.09163	0.802
3857	214736_s_at	ADD1	0.58012	0.7205	0.66829	-0.08817	0.773
3858	214739_at	LRCH3	0.56316	0.62494	0.67376	-0.1106	0.83
3859	214741_at	ZNF131	0.39842	0.41168	0.42133	-0.02291	0.577
3860	214742_at	AZI1	0.14277	0.32095	0.18781	-0.04504	0.607
3861	214744_s_at	RPL23	0.19805	0.51127	0.62432	-0.42627	1
3862	214748_at	N4BP2L2	0.45991	0.47092	0.12226	0.33765	0.003
3863	214749_s_at	ARMCX6	0.7509	0.7917	0.76904	-0.01814	0.566
3864	214751_at	ZNF468	0.65358	0.6059	0.71606	-0.06248	0.671
3865	214759_at	WTAP	0.62052	0.67239	0.66769	-0.04717	0.62
3866	214762_at	ATP6V1G2	0.019628	0.12409	0.10953	-0.089902	0.66

3867	214764_at	RRP15	0.71158	0.63611	0.65835	0.05323	0.31
3868	214766_s_at	AHCTF1	0.57606	0.33824	0.28814	0.28792	0.002
3869	214772_at	C11orf41	0.31233	0.58899	0.37978	-0.06745	0.699
3870	214775_at	N4BP3	0.13008	0.144	0.075714	0.054366	0.37
3871	214779_s_at	SGSM3	0.15808	0.25745	0.34407	-0.18599	0.908
3872	214780_s_at	MYO9B	0.46516	0.51684	0.36114	0.10402	0.211
3873	214785_at	VPS13A	0.63085	0.67535	0.67371	-0.04286	0.717
3874	214787_at	DENND4A	0.70668	0.60154	0.60139	0.10529	0.188
3875	214790_at	SENP6	0.39381	0.18513	0.14158	0.25223	0.023
3876	214791_at	SP140L	0.33346	0.35101	0.30219	0.03127	0.407
3877	214801_at	IFRG15	0.66529	0.63532	0.59399	0.0713	0.259
3878	214804_at	CENPI	0.63326	0.56418	0.57978	0.05348	0.291
3879	214813_at	ZNF75D	0.45434	0.35461	0.29818	0.15616	0.13
3880	214814_at	YTHDC1	0.25828	0.15558	0.16581	0.09247	0.33
3881	214830_at	SLC38A6	0.1834	0.53111	0.19992	-0.01652	0.548
3882	214838_at	SFT2D2	0.44178	0.60226	0.53487	-0.09309	0.755
3883	214843_s_at	USP33	0.66494	0.53794	0.73051	-0.06557	0.804
3884	214844_s_at	DOK5	0.058493	0.2429	0.43538	-0.376887	0.973
3885	214848_at	YWHAZ	0.02745	0.076808	0.16162	-0.13417	0.785
3886	214850_at	LOC100170939	0.073456	0.19413	0.20923	-0.135774	0.783
3887	214857_at	C10orf95	0.2414	0.35943	0.39494	-0.15354	0.902
3888	214864_s_at	GRHPR	0.53727	0.582	0.63757	-0.1003	0.757
3889	214876_s_at	TUBGCP5	0.57713	0.51967	0.35756	0.21957	0.03
3890	214878_at	ZNF37A	0.13555	0.27898	0.29247	-0.15692	0.872
3891	214882_s_at	SFRS2	0.7706	0.81739	0.66811	0.10249	0.223
3892	214895_s_at	ADAM10	0.77089	0.78108	0.75395	0.01694	0.462
3893	214918_at	HNRNPM	0.25717	0.30495	0.33446	-0.07729	0.723
3894	214919_s_at	ANKHD1-EIF4EBP3	0.49814	0.50954	0.48486	0.01328	0.473
3895	214923_at	ATP6V1D	0.50973	0.48544	0.41145	0.09828	0.251
3896	214931_s_at	SRPK2	0.4162	0.60278	0.61001	-0.19381	0.846
3897	214941_s_at	PRPF40A	0.43639	0.56649	0.61289	-0.1765	0.971
3898	214943_s_at	RBM34	0.76353	0.67172	0.6356	0.12793	0.068
3899	214948_s_at	TMF1	0.47904	0.51018	0.46301	0.01603	0.452
3900	214949_at	hCG_1795560	0.66391	0.62344	0.652	0.01191	0.462
3901	214953_s_at	APP	0.016489	0.076013	0.12983	-0.113341	0.858
3902	214962_s_at	NUP160	0.26328	0.00018406	0.044707	0.218573	0.027
3903	214975_s_at	MTMR1	0.64949	0.6502	0.67531	-0.02582	0.62
3904	214984_at	LOC440345	0.0043134	0.070891	0.08169	-0.0773766	0.726
3905	214992_s_at	DNASE2	0.47531	0.46383	0.49138	-0.01607	0.571
3906	214997_at	C9orf126	0.42213	0.20081	0.24819	0.17394	0.183
3907	215001_s_at	GLUL	0.48517	0.55152	0.65344	-0.16827	0.897
3908	215011_at	SNHG3	0.16794	0.30259	0.24932	-0.08138	0.75
3909	215023_s_at	PEX1	0.38093	0.52385	0.43198	-0.05105	0.715
3910	215029_at	C1orf108	0.21394	0.14179	0.19498	0.01896	0.46
3911	215030_at	GRSF1	0.6303	0.60773	0.51591	0.11439	0.118
3912	215068_s_at	FBXL18	0.41711	0.16505	0.31102	0.10609	0.281
3913	215071_s_at	HIST1H2AC	0.31673	0.42623	0.28166	0.03507	0.441
3914	215075_s_at	GRB2	0.46217	0.5631	0.54699	-0.08482	0.813
3915	215084_s_at	LRRC42	0.35472	0.40347	0.3338	0.02092	0.486
3916	215087_at	C15orf39	0.2834	0.22723	0.25141	0.03199	0.397
3917	215088_s_at	SDHC	0.59552	0.59428	0.52826	0.06726	0.287
3918	215089_s_at	RBM10	0.70455	0.80037	0.64511	0.05944	0.324
3919	215091_s_at	GTF3A	0.66192	0.69083	0.66294	-0.00102	0.526

3920	215096_s_at	ESD	0.79561	0.83166	0.8557	-0.06009	0.935
3921	215100_at	C6orf105	0.2616	0.42235	0.37398	-0.11238	0.72
3922	215109_at	KIAA0492	0.42567	0.33597	0.3484	0.07727	0.289
3923	215111_s_at	TSC22D1	0.099602	0.26182	0.38958	-0.289978	0.956
3924	215123_at	LOC642778	0.29703	0.31965	0.27663	0.0204	0.43
3925	215127_s_at	RBMS1	0.42079	0.45179	0.54087	-0.12008	0.817
3926	215134_at	PI4K2A	0.0078511	0.07688	0.037509	-0.0296579	0.576
3927	215136_s_at	EXOSC8	0.81761	0.87168	0.86024	-0.04263	0.825
3928	215143_at	DPY19L2P2	0.44557	0.26734	0.33891	0.10666	0.186
3929	215148_s_at	APBA3	0.75655	0.79195	0.70804	0.04851	0.231
3930	215150_at	YOD1	0.3895	0.43047	0.50479	-0.11529	0.865
3931	215170_s_at	CEP152	0.25187	0.35354	0.26439	-0.01252	0.558
3932	215190_at	EIF3M	0.3347	0.34527	0.13951	0.19519	0.166
3933	215191_at	FBXL11	0.22607	0.1852	0.37318	-0.14711	0.855
3934	215210_s_at	DLST	0.29957	0.29152	0.32331	-0.02374	0.549
3935	215218_s_at	WDR62	0.080489	0.066369	0.048818	0.031671	0.405
3936	215223_s_at	SOD2	0.35881	0.24748	0.19775	0.16106	0.091
3937	215228_at	NHLH2	0.04339	0.023458	0.0045155	0.0388745	0.418
3938	215241_at	ANO3	0.43115	0.48854	0.52495	-0.0938	0.721
3939	215263_at	ZXDA	0.17542	0.011765	0.033052	0.142368	0.191
3940	215285_s_at	PHTF1	0.21159	0.45055	0.42876	-0.21717	0.951
3941	215307_at	ZNF529	0.085019	0.30105	0.0734	0.011619	0.468
3942	215338_s_at	NKTR	0.32364	0.059905	0.20976	0.11388	0.202
3943	215343_at	CCDC88C	0.34814	0.4161	0.27386	0.07428	0.274
3944	215366_at	SNX13	0.67904	0.58764	0.52595	0.15309	0.094
3945	215380_s_at	GGCT	0.78616	0.76832	0.82228	-0.03612	0.659
3946	215385_at	FTO	0.047166	0.22068	0.093459	-0.046293	0.617
3947	215399_s_at	OS9	0.53746	0.64208	0.65642	-0.11896	0.889
3948	215416_s_at	STOML2	0.69894	0.75598	0.74402	-0.04508	0.709
3949	215424_s_at	SNW1	0.32408	0.19613	0.19399	0.13009	0.211
3950	215425_at	BTG3	0.36023	0.29483	0.13116	0.22907	0.084
3951	215440_s_at	BEX4	0.080771	0.25132	0.20789	-0.127119	0.857
3952	215463_at	OR7E24	0.14387	0.031566	0.16554	-0.02167	0.561
3953	215465_at	ABCA12	0.30445	0.41799	0.46927	-0.16482	0.909
3954	215470_at	GTF2H2B	0.11112	0.29191	0.15816	-0.04704	0.593
3955	215482_s_at	EIF2B4	0.2672	0.3619	0.35207	-0.08487	0.734
3956	215499_at	MAP2K3	0.49577	0.25681	0.24791	0.24786	0.033
3957	215501_s_at	DUSP10	0.5291	0.67347	0.71387	-0.18477	0.971
3958	215509_s_at	BUB1	0.80081	0.76825	0.75678	0.04403	0.346
3959	215521_at	PHC3	0.057103	0.062122	0.033296	0.023807	0.46
3960	215536_at	HLA-DQB2	0.11434	0.25249	0.34316	-0.22882	0.965
3961	215548_s_at	SCFD1	0.75564	0.69043	0.57007	0.18557	0.011
3962	215577_at	UBE2E1	0.34287	0.47079	0.24555	0.09732	0.239
3963	215596_s_at	RNF160	0.72716	0.74804	0.81104	-0.08388	0.879
3964	215602_at	FGD2	0.11373	0.13975	0.088922	0.024808	0.461
3965	215631_s_at	BRMS1	0.79334	0.73601	0.79357	-0.00023	0.528
3966	215684_s_at	ASCC2	0.6245	0.5122	0.64251	-0.01801	0.553
3967	215694_at	SPATA5L1	0.030182	0.1558	0.18474	-0.154558	0.843
3968	215696_s_at	SEC16A	0.81341	0.81311	0.78248	0.03093	0.39
3969	215707_s_at	PRNP	0.16258	0.39819	0.31007	-0.14749	0.86
3970	215718_s_at	PHF3	0.45625	0.60667	0.68331	-0.22706	0.938
3971	215728_s_at	ACOT7	0.54211	0.66612	0.47562	0.06649	0.358
3972	215734_at	C19orf36	0.50606	0.52304	0.48854	0.01752	0.466

3973	215739_s_at	TUBGCP3	0.62969	0.54338	0.64887	-0.01918	0.578
3974	215743_at	NMT2	0.16902	0.18784	0.22276	-0.05374	0.641
3975	215747_s_at	RCC1	0.55636	0.52824	0.54433	0.01203	0.464
3976	215749_s_at	GORASP1	0.51903	0.46546	0.40093	0.1181	0.274
3977	215767_at	ZNF804A	0.24644	0.34467	0.27465	-0.02821	0.604
3978	215780_s_at	hCG_1644608	0.5235	0.54924	0.48018	0.04332	0.486
3979	215785_s_at	CYFIP2	0.60099	0.64665	0.69652	-0.09553	0.755
3980	215792_s_at	DNAJC11	0.64714	0.70038	0.74541	-0.09827	0.86
3981	215854_at	FBXO22	0.086498	0.41767	0.1554	-0.068902	0.597
3982	215884_s_at	UBQLN2	0.69515	0.70654	0.78175	-0.0866	0.838
3983	215905_s_at	SNRNP40	0.40354	0.49814	0.48969	-0.08615	0.714
3984	215919_s_at	MRPS11	0.1444	0.21497	0.2115	-0.0671	0.644
3985	215930_s_at	CTAGE5	0.4332	0.4693	0.47069	-0.03749	0.629
3986	215942_s_at	GTSE1	0.70815	0.59042	0.44231	0.26584	0.01
3987	215947_s_at	FAM136A	0.69285	0.75559	0.61717	0.07568	0.238
3988	215954_s_at	C19orf29	0.24716	0.40218	0.39996	-0.1528	0.819
3989	215980_s_at	IGHMBP2	0.056988	0.14782	0.10047	-0.043482	0.585
3990	215983_s_at	UBXN8	0.63983	0.44682	0.50808	0.13175	0.132
3991	215984_s_at	ARFRP1	0.43183	0.34544	0.20455	0.22728	0.06
3992	215985_at	NCRNA00171	0.36313	0.2899	0.28648	0.07665	0.268
3993	216006_at	RAPGEFL1	0.1187	0.23932	0.18484	-0.06614	0.629
3994	216020_at	IFIH1	0.40715	0.55234	0.3256	0.08155	0.38
3995	216026_s_at	POLE	0.40512	0.37316	0.38795	0.01717	0.446
3996	216028_at	DKFZP564C152	0.0058348	0.10405	0.05918	-0.0533452	0.694
3997	216032_s_at	ERGIC3	0.65641	0.75242	0.72371	-0.0673	0.803
3998	216060_s_at	DAAM1	0.70504	0.69062	0.67134	0.0337	0.391
3999	216088_s_at	PSMA7	0.8756	0.86247	0.8826	-0.007	0.585
4000	216114_at	NCKIPSD	0.25922	0.17915	0.21827	0.04095	0.406
4001	216147_at	SEPT11	0.064472	0.21348	0.075311	-0.010839	0.515
4002	216177_at	LOC391132	0.61569	0.66099	0.77105	-0.15536	0.923
4003	216194_s_at	TBCB	0.60037	0.54259	0.70354	-0.10317	0.778
4004	216199_s_at	MAP3K4	0.40209	0.60045	0.47141	-0.06932	0.74
4005	216218_s_at	PLCL2	0.85253	0.75233	0.7402	0.11233	0.106
4006	216226_at	TAF4B	0.58617	0.58697	0.54553	0.04064	0.344
4007	216231_s_at	B2M	0.9302	0.93209	0.93974	-0.00954	0.64
4008	216232_s_at	GCN1L1	0.62702	0.65508	0.62723	-0.00021	0.503
4009	216241_s_at	TCEA1	0.34181	0.47404	0.57089	-0.22908	0.89
4010	216247_at	RPS20	0.15663	0.29227	0.15756	-0.00093	0.537
4011	216248_s_at	NR4A2	0.26159	0.013567	0.010351	0.251239	0.059
4012	216250_s_at	LPXN	0.38956	0.45881	0.55036	-0.1608	0.836
4013	216251_s_at	TTLL12	0.66245	0.71663	0.64045	0.022	0.42
4014	216261_at	ITGB3	0.084777	0.072259	0.087194	-0.002417	0.514
4015	216262_s_at	TGIF2	0.30163	0.42275	0.22554	0.07609	0.342
4016	216266_s_at	ARFGEF1	0.67493	0.76207	0.82246	-0.14753	0.974
4017	216267_s_at	TMEM115	0.47175	0.16901	0.36005	0.1117	0.245
4018	216305_s_at	C2orf3	0.4634	0.60366	0.55123	-0.08783	0.707
4019	216326_s_at	HDAC3	0.29344	0.41081	0.3393	-0.04586	0.626
4020	216338_s_at	YIPF3	0.72841	0.67379	0.71596	0.01245	0.419
4021	216348_at	RPS17P5	0.88701	0.87108	0.88785	-0.00084	0.53
4022	216383_at	RPL18A	0.28326	0.4936	0.46944	-0.18618	0.862
4023	216396_s_at	EI24	0.39768	0.65906	0.24954	0.14814	0.118
4024	216397_s_at	BOP1	0.74152	0.57725	0.53978	0.20174	0.007
4025	216409_at	ACSL6	0.14935	0.12214	0.10657	0.04278	0.576

4026	216411_s_at	GALK2	0.2324	0.089706	0.073954	0.158446	0.34
4027	216437_at	EPC1	0.15148	0.13379	0.23465	-0.08317	0.623
4028	216438_s_at	TMSB4X	0.93913	0.94244	0.93988	-0.00075	0.539
4029	216520_s_at	TPT1	0.95236	0.94593	0.94286	0.0095	0.292
4030	216521_s_at	BRCC3	0.54368	0.62114	0.63001	-0.08633	0.802
4031	216547_at	LOC127406	0.33427	0.60334	0.62474	-0.29047	0.987
4032	216563_at	ANKRD12	0.68317	0.70974	0.77581	-0.09264	0.844
4033	216574_s_at	hCG_2024410	0.22333	0.0032074	0.0062663	0.2170637	0.228
4034	216591_s_at	hCG_1776980	0.59159	0.77228	0.57423	0.01736	0.402
4035	216602_s_at	FARSA	0.85295	0.8924	0.87327	-0.02032	0.757
4036	216607_s_at	CYP51A1	0.7425	0.78394	0.67192	0.07058	0.232
4037	216609_at	TXN	0.75114	0.77994	0.81495	-0.06381	0.821
4038	216640_s_at	PDIA6	0.46838	0.51065	0.61181	-0.14343	0.886
4039	216650_at	LOC283412	0.48076	0.4854	0.36652	0.11424	0.23
4040	216678_at	IFT122	0.31799	0.50549	0.43452	-0.11653	0.82
4041	216699_s_at	KLK1	0.53239	0.55525	0.54571	-0.01332	0.555
4042	216705_s_at	ADA	0.51862	0.4595	0.3611	0.15752	0.108
4043	216783_at	LOC283677	0.45446	0.37831	0.37742	0.07704	0.277
4044	216806_at	RPSA	0.3968	0.47238	0.64879	-0.25199	0.972
4045	216834_at	RGS1	0.34635	0.28026	0.2095	0.13685	0.194
4046	216835_s_at	DOK1	0.29944	0.35101	0.3504	-0.05096	0.652
4047	216862_s_at	MTCP1NB	0.79342	0.775	0.78561	0.00781	0.441
4048	216863_s_at	MORC2	0.55434	0.52459	0.51467	0.03967	0.416
4049	216899_s_at	SKAP2	0.76608	0.65463	0.70376	0.06232	0.236
4050	216902_s_at	LOC653390	0.55858	0.64117	0.63365	-0.07507	0.796
4051	216903_s_at	CBARA1	0.50421	0.60039	0.47625	0.02796	0.408
4052	216942_s_at	CD58	0.4791	0.4812	0.5167	-0.0376	0.659
4053	216944_s_at	ITPR1	0.67613	0.62041	0.5607	0.11543	0.119
4054	216952_s_at	LMNB2	0.60246	0.58375	0.55954	0.04292	0.379
4055	216961_s_at	RPAIN	0.59148	0.61897	0.6505	-0.05902	0.681
4056	216969_s_at	KIF22	0.60466	0.50425	0.48932	0.11534	0.23
4057	216993_s_at	COL11A2	0.59359	0.38766	0.34144	0.25215	0.039
4058	216996_s_at	FASTKD2	0.74895	0.74864	0.65055	0.0984	0.223
4059	217019_at	RPS4X	0.26032	0.33447	0.17059	0.08973	0.21
4060	217028_at	CXCR4	0.44412	0.48527	0.40922	0.0349	0.46
4061	217042_at	RDH11	0.31923	0.17055	0.46077	-0.14154	0.864
4062	217043_s_at	MFN1	0.66018	0.64659	0.64725	0.01293	0.429
4063	217047_s_at	FAM13A	0.84156	0.75975	0.74869	0.09287	0.086
4064	217094_s_at	ITCH	0.53161	0.40095	0.50871	0.0229	0.415
4065	217100_s_at	UBXN7	0.8068	0.82043	0.80893	-0.00213	0.535
4066	217104_at	ST20	0.034423	0.13427	0.16241	-0.127987	0.754
4067	217118_s_at	C22orf9	0.18169	0.2644	0.26887	-0.08718	0.747
4068	217122_s_at	RP11-345P4.4	0.559	0.56319	0.61377	-0.05477	0.739
4069	217125_at	UBBP2	0.52872	0.45215	0.47528	0.05344	0.354
4070	217127_at	CTH	0.2253	0.55877	0.32538	-0.10008	0.733
4071	217139_at	LOC100133724	0.16551	0.094627	0.19776	-0.03225	0.582
4072	217144_at	LOC648390	0.68248	0.6848	0.66848	0.014	0.41
4073	217168_s_at	HERPUD1	0.20028	0.38123	0.25887	-0.05859	0.628
4074	217176_s_at	ZFX	0.16393	0.53052	0.40594	-0.24201	0.903
4075	217188_s_at	C14orf1	0.29797	0.48176	0.3619	-0.06393	0.638
4076	217211_at	ACTBP9	0.3665	0.5424	0.4413	-0.0748	0.694
4077	217266_at	RPL15P22	0.68798	0.73227	0.74841	-0.06043	0.776
4078	217286_s_at	NDRG3	0.38717	0.34877	0.37149	0.01568	0.482

4079	217289_s_at	SLC37A4	0.67609	0.54821	0.41476	0.26133	0.008
4080	217299_s_at	NBN	0.61997	0.74039	0.77139	-0.15142	0.975
4081	217310_s_at	FOXJ3	0.45637	0.54046	0.51899	-0.06262	0.732
4082	217317_s_at	HERC2P2	0.41467	0.42278	0.52645	-0.11178	0.818
4083	217336_at	RPS10	0.33223	0.35458	0.46642	-0.13419	0.762
4084	217340_at	RPL21P68	0.38314	0.25462	0.47106	-0.08792	0.714
4085	217346_at	LOC128192	0.49931	0.43346	0.40531	0.094	0.212
4086	217365_at	PRAMEF11	0.26158	0.44267	0.42209	-0.16051	0.806
4087	217368_at	ATP5G2	0.4296	0.3632	0.28292	0.14668	0.181
4088	217379_at	RPL10	0.71154	0.74983	0.81281	-0.10127	0.944
4089	217383_at	PGK1	0.49149	0.6368	0.58511	-0.09362	0.768
4090	217388_s_at	KYNU	0.59059	0.56508	0.59996	-0.00937	0.581
4091	217403_s_at	ZNF227	0.32744	0.53966	0.41479	-0.08735	0.755
4092	217408_at	MRPS18B	0.74821	0.67996	0.71331	0.0349	0.354
4093	217427_s_at	HIRA	0.14455	0.14915	0.27774	-0.13319	0.823
4094	217445_s_at	GART	0.79649	0.79656	0.78712	0.00937	0.417
4095	217448_s_at	LOC285412	0.49269	0.47688	0.486	0.00669	0.49
4096	217457_s_at	RAP1GDS1	0.34004	0.31875	0.30913	0.03091	0.418
4097	217465_at	NCKAP1	0.58661	0.31289	0.47082	0.11579	0.24
4098	217477_at	PIP5K1B	0.43779	0.40797	0.57893	-0.14114	0.843
4099	217478_s_at	HLA-DMA	0.58968	0.75262	0.70777	-0.11809	0.85
4100	217494_s_at	PTENP1	0.6513	0.74168	0.69742	-0.04612	0.691
4101	217496_s_at	IDE	0.81785	0.75304	0.75009	0.06776	0.205
4102	217501_at	CIAO1	0.32228	0.33745	0.34344	-0.02116	0.557
4103	217503_at	STK17B	0.30363	0.28759	0.39275	-0.08912	0.757
4104	217504_at	ABCA6	0.59645	0.63346	0.58701	0.00944	0.462
4105	217506_at	LOC339290	0.33715	0.4039	0.23251	0.10464	0.283
4106	217523_at	CD44	0.32729	0.48288	0.53537	-0.20808	0.985
4107	217527_s_at	NFATC2IP	0.68645	0.71417	0.76675	-0.0803	0.862
4108	217540_at	FAM55C	0.66479	0.5545	0.53156	0.13323	0.052
4109	217544_at	LOC729806	0.44308	0.50562	0.53471	-0.09163	0.773
4110	217549_at	NCKAP1L	0.48216	0.48051	0.35053	0.13163	0.193
4111	217559_at	RPL10L	0.48036	0.50133	0.49074	-0.01038	0.577
4112	217599_s_at	MDFIC	0.43242	0.49248	0.59292	-0.1605	0.939
4113	217602_at	PPIA	0.50963	0.3421	0.31888	0.19075	0.087
4114	217608_at	SFRS12IP1	0.367	0.4727	0.51016	-0.14316	0.84
4115	217627_at	ZNF573	0.061469	0.36006	0.1676	-0.106131	0.799
4116	217645_at	COX16	0.27005	0.39251	0.27003	2E-05	0.505
4117	217663_at	ZNF234	0.01754	0.018288	0.036545	-0.019005	0.58
4118	217677_at	PLEKHA2	0.57027	0.65744	0.51262	0.05765	0.359
4119	217682_at	C16orf72	0.45442	0.23474	0.37903	0.07539	0.263
4120	217692_at	MAGOH2	0.74285	0.73653	0.70874	0.03411	0.359
4121	217716_s_at	SEC61A1	0.8333	0.6563	0.76873	0.06457	0.227
4122	217718_s_at	YWHAB	0.75051	0.68159	0.75103	-0.00052	0.513
4123	217719_at	EIF3L	0.59359	0.68374	0.71936	-0.12577	0.92
4124	217720_at	CHCHD2	0.89783	0.8775	0.9341	-0.03627	0.875
4125	217722_s_at	NGRN	0.62047	0.63935	0.65121	-0.03074	0.632
4126	217726_at	COPZ1	0.39652	0.2423	0.2432	0.15332	0.08
4127	217728_at	S100A6	0.35289	0.26606	0.42988	-0.07699	0.719
4128	217729_s_at	AES	0.53993	0.53732	0.53748	0.00245	0.489
4129	217730_at	TMBIM1	0.30195	0.38517	0.26311	0.03884	0.393
4130	217731_s_at	ITM2B	0.55946	0.69351	0.55252	0.00694	0.458
4131	217733_s_at	TMSB10	0.79697	0.83708	0.86085	-0.06388	0.829

4132	217734_s_at	WDR6	0.70205	0.75066	0.67011	0.03194	0.375
4133	217736_s_at	EIF2AK1	0.71196	0.70294	0.72376	-0.0118	0.542
4134	217739_s_at	NAMPT	0.71589	0.69204	0.57142	0.14447	0.175
4135	217742_s_at	WAC	0.45434	0.45411	0.31749	0.13685	0.48
4136	217743_s_at	TMEM30A	0.8341	0.80763	0.71951	0.11459	0.169
4137	217744_s_at	PERP	0.33539	0.46405	0.42261	-0.08722	0.722
4138	217745_s_at	NAT13	0.73277	0.5536	0.54292	0.18985	0.018
4139	217746_s_at	PDCD6IP	0.77935	0.71824	0.62775	0.1516	0.064
4140	217747_s_at	RPS9	0.94537	0.92101	0.95903	-0.01366	0.905
4141	217748_at	ADIPOR1	0.4781	0.48538	0.49075	-0.01265	0.523
4142	217749_at	COPG	0.75314	0.74167	0.71253	0.04061	0.291
4143	217750_s_at	UBE2Z	0.83536	0.82105	0.7507	0.08466	0.084
4144	217751_at	GSTK1	0.68203	0.76664	0.69651	-0.01448	0.595
4145	217752_s_at	CNDP2	0.30239	0.58897	0.51571	-0.21332	0.935
4146	217753_s_at	RPS26	0.6589	0.69465	0.78927	-0.13037	0.953
4147	217754_at	DDX56	0.44831	0.53583	0.63166	-0.18335	0.861
4148	217755_at	HN1	0.81981	0.86821	0.87901	-0.0592	0.681
4149	217758_s_at	TM9SF3	0.71892	0.7593	0.81036	-0.09144	0.857
4150	217759_at	TRIM44	0.6809	0.72843	0.70054	-0.01964	0.595
4151	217761_at	ADI1	0.11916	0.32915	0.34587	-0.22671	0.915
4152	217763_s_at	RAB31	0.3947	0.57008	0.55925	-0.16455	0.855
4153	217765_at	NRBP1	0.32767	0.41003	0.33211	-0.00444	0.501
4154	217766_s_at	TMEM50A	0.38993	0.4563	0.46593	-0.076	0.762
4155	217768_at	C14orf166	0.82351	0.82678	0.87137	-0.04786	0.879
4156	217769_s_at	POMP	0.88428	0.8839	0.89403	-0.00975	0.654
4157	217772_s_at	MTCH2	0.44447	0.53126	0.5363	-0.09183	0.715
4158	217773_s_at	NDUFA4	0.91689	0.86836	0.8835	0.03339	0.176
4159	217774_s_at	HSPC152	0.83391	0.74434	0.78705	0.04686	0.27
4160	217777_s_at	PTPLAD1	0.55272	0.49834	0.53632	0.0164	0.443
4161	217778_at	SLC39A1	0.55886	0.29012	0.29356	0.2653	0.008
4162	217779_s_at	LOC100132235	0.53255	0.37405	0.52892	0.00363	0.522
4163	217780_at	C19orf56	0.85492	0.83059	0.8684	-0.01348	0.57
4164	217781_s_at	ZFP106	0.44513	0.47158	0.56292	-0.11779	0.765
4165	217782_s_at	GPS1	0.78033	0.72951	0.67497	0.10536	0.036
4166	217783_s_at	YPEL5	0.78584	0.73399	0.63534	0.1505	<0.001
4167	217786_at	PRMT5	0.66891	0.50848	0.71121	-0.0423	0.728
4168	217788_s_at	GALNT2	0.40624	0.57273	0.51275	-0.10651	0.875
4169	217789_at	SNX6	0.50828	0.39695	0.407	0.10128	0.193
4170	217790_s_at	SSR3	0.5895	0.55701	0.54923	0.04027	0.383
4171	217791_s_at	ALDH18A1	0.81863	0.76044	0.77749	0.04114	0.268
4172	217792_at	SNX5	0.35753	0.53407	0.5718	-0.21427	0.921
4173	217794_at	PRR13	0.59683	0.62079	0.62698	-0.03015	0.609
4174	217795_s_at	TMEM43	0.42922	0.43614	0.42132	0.0079	0.484
4175	217796_s_at	NPLOC4	0.68471	0.41378	0.43583	0.24888	0.006
4176	217797_at	UFC1	0.65116	0.7995	0.7468	-0.09564	0.83
4177	217800_s_at	NDFIP1	0.27294	0.49987	0.50511	-0.23217	0.923
4178	217801_at	ATP5E	0.87544	0.86429	0.87578	-0.00034	0.506
4179	217803_at	GOLPH3	0.59006	0.4854	0.6152	-0.02514	0.609
4180	217805_at	ILF3	0.66529	0.70288	0.59381	0.07148	0.234
4181	217806_s_at	POLDIP2	0.41063	0.5222	0.36712	0.04351	0.394
4182	217807_s_at	GLTSCR2	0.83304	0.79852	0.83456	-0.00152	0.523
4183	217808_s_at	MAPKAP1	0.65171	0.67069	0.71352	-0.06181	0.707
4184	217809_at	BZW2	0.4477	0.6177	0.56837	-0.12067	0.86

4185	217811_at	SELT	0.60435	0.73737	0.74336	-0.13901	0.854
4186	217812_at	YTHDF2	0.53887	0.30144	0.40398	0.13489	0.192
4187	217813_s_at	SPIN1	0.25281	0.47845	0.51432	-0.26151	0.92
4188	217814_at	CCDC47	0.32863	0.35581	0.38655	-0.05792	0.626
4189	217815_at	SUPT16H	0.73119	0.79835	0.83272	-0.10153	0.888
4190	217816_s_at	PCNP	0.41163	0.37838	0.29078	0.12085	0.169
4191	217819_at	GOLGA7	0.48645	0.55331	0.6938	-0.20735	0.96
4192	217822_at	WBP11	0.74123	0.79931	0.69879	0.04244	0.297
4193	217826_s_at	UBE2J1	0.74541	0.8113	0.80121	-0.0558	0.739
4194	217827_s_at	SPG21	0.5369	0.55991	0.61369	-0.07679	0.8
4195	217828_at	SLTM	0.77931	0.72402	0.73185	0.04746	0.312
4196	217829_s_at	USP39	0.4673	0.28545	0.38485	0.08245	0.294
4197	217830_s_at	NSFL1C	0.23577	0.33591	0.34873	-0.11296	0.743
4198	217833_at	SYNCRIP	0.65483	0.49477	0.42711	0.22772	<0.001
4199	217836_s_at	YY1AP1	0.43711	0.37281	0.34419	0.09292	0.168
4200	217837_s_at	VPS24	0.23458	0.42251	0.43955	-0.20497	0.949
4201	217838_s_at	EVL	0.48389	0.51144	0.53781	-0.05392	0.616
4202	217840_at	DDX41	0.64342	0.63349	0.44492	0.1985	0.005
4203	217841_s_at	PPME1	0.71315	0.66561	0.51189	0.20126	0.059
4204	217843_s_at	MED4	0.81264	0.82658	0.73583	0.07681	0.195
4205	217846_at	QARS	0.88649	0.68996	0.75351	0.13298	0.045
4206	217848_s_at	PPA1	0.55479	0.73398	0.70166	-0.14687	0.949
4207	217850_at	GNL3	0.71202	0.64565	0.54536	0.16666	<0.001
4208	217851_s_at	SLMO2	0.79994	0.84851	0.86367	-0.06373	0.8
4209	217852_s_at	ARL8B	0.64392	0.66539	0.59433	0.04959	0.335
4210	217853_at	TNS3	0.033331	0.15146	0.080158	-0.046827	0.754
4211	217858_s_at	ARMCX3	0.73568	0.70751	0.70965	0.02603	0.413
4212	217860_at	LOC732160	0.40292	0.55848	0.37028	0.03264	0.421
4213	217861_s_at	PREB	0.57787	0.5444	0.40494	0.17293	0.009
4214	217862_at	PIAS1	0.60676	0.51124	0.52024	0.08652	0.183
4215	217866_at	CPSF7	0.71956	0.76019	0.74845	-0.02889	0.657
4216	217868_s_at	METTL9	0.63325	0.56285	0.44355	0.1897	0.005
4217	217869_at	HSD17B12	0.064431	0.14181	0.14841	-0.083979	0.706
4218	217870_s_at	CMPK1	0.64475	0.67508	0.62481	0.01994	0.415
4219	217871_s_at	MIF	0.89347	0.82904	0.85191	0.04156	0.231
4220	217872_at	PIH1D1	0.53988	0.60121	0.64523	-0.10535	0.75
4221	217873_at	CAB39	0.56947	0.52508	0.52764	0.04183	0.353
4222	217874_at	SUCLG1	0.67546	0.75476	0.74356	-0.0681	0.693
4223	217877_s_at	GPBP1L1	0.71929	0.56883	0.64511	0.07418	0.29
4224	217878_s_at	CDC27	0.56721	0.60051	0.56374	0.00347	0.507
4225	217882_at	TMEM111	0.58132	0.7452	0.82421	-0.24289	0.972
4226	217883_at	MMADHC	0.53427	0.69234	0.51032	0.02395	0.427
4227	217884_at	NAT10	0.82584	0.81651	0.75785	0.06799	0.205
4228	217885_at	IPO9	0.70362	0.72069	0.76961	-0.06599	0.763
4229	217886_at	EPS15	0.73422	0.76391	0.75556	-0.02134	0.63
4230	217888_s_at	ARFGAP1	0.41176	0.45833	0.61877	-0.20701	0.939
4231	217892_s_at	LIMA1	0.31604	0.39575	0.47785	-0.16181	0.873
4232	217893_s_at	AKIRIN1	0.69974	0.54989	0.66288	0.03686	0.362
4233	217894_at	KCTD3	0.44494	0.43227	0.51782	-0.07288	0.775
4234	217895_at	PTCD3	0.4519	0.25263	0.20791	0.24399	0.033
4235	217896_s_at	NIP30	0.4845	0.37235	0.40149	0.08301	0.277
4236	217898_at	C15orf24	0.68051	0.82896	0.82073	-0.14022	0.907
4237	217899_at	TMEM214	0.52385	0.56636	0.67112	-0.14727	0.889

4238	217900_at	IARS2	0.63902	0.75562	0.7337	-0.09468	0.856
4239	217901_at	DSG2	0.13351	0.32325	0.33296	-0.19945	0.918
4240	217902_s_at	HERC2	0.69196	0.66243	0.56386	0.1281	0.117
4241	217903_at	STRN4	0.40547	0.56704	0.40334	0.00213	0.511
4242	217905_at	C10orf119	0.27637	0.28562	0.28741	-0.01104	0.543
4243	217906_at	KLHDC2	0.37877	0.37693	0.30537	0.0734	0.264
4244	217907_at	MRPL18	0.85389	0.69671	0.70206	0.15183	<0.001
4245	217908_s_at	IQWD1	0.78687	0.78106	0.7201	0.06677	0.178
4246	217911_s_at	BAG3	0.48049	0.54484	0.63121	-0.15072	0.865
4247	217912_at	DUS1L	0.64905	0.52517	0.58787	0.06118	0.312
4248	217913_at	VPS4A	0.40422	0.19911	0.30107	0.10315	0.248
4249	217914_at	TPCN1	0.48148	0.23205	0.12249	0.35899	0.016
4250	217915_s_at	RSL24D1	0.62312	0.80864	0.70762	-0.0845	0.81
4251	217918_at	DYNLRB1	0.82351	0.81958	0.83497	-0.01146	0.542
4252	217919_s_at	MRPL42	0.53735	0.61147	0.53503	0.00232	0.52
4253	217923_at	PEF1	0.57696	0.67145	0.66022	-0.08326	0.809
4254	217924_at	C6orf106	0.56809	0.60709	0.69647	-0.12838	0.895
4255	217926_at	C19orf53	0.80877	0.76555	0.82001	-0.01124	0.564
4256	217927_at	SPCS1	0.81599	0.85952	0.91052	-0.09453	0.912
4257	217928_s_at	SAPS3	0.39502	0.4373	0.30415	0.09087	0.289
4258	217930_s_at	TOLLIP	0.48884	0.49637	0.48836	0.00048	0.528
4259	217931_at	CNPY3	0.50267	0.47556	0.49309	0.00958	0.469
4260	217932_at	MRPS7	0.67381	0.73206	0.73726	-0.06345	0.698
4261	217933_s_at	LAP3	0.61345	0.63441	0.66102	-0.04757	0.715
4262	217935_s_at	UQCC	0.49034	0.53115	0.52959	-0.03925	0.655
4263	217936_at	ARHGAP5	0.31809	0.37426	0.47393	-0.15584	0.906
4264	217938_s_at	KCMF1	0.58033	0.56254	0.47347	0.10686	0.224
4265	217939_s_at	AFTPH	0.73664	0.66056	0.80186	-0.06522	0.769
4266	217940_s_at	CARKD	0.56626	0.61788	0.4754	0.09086	0.268
4267	217941_s_at	ERBB2IP	0.84864	0.83193	0.84866	-2E-05	0.514
4268	217942_at	MRPS35	0.64445	0.55505	0.58153	0.06292	0.304
4269	217943_s_at	MAP7D1	0.67457	0.63564	0.62259	0.05198	0.334
4270	217944_at	POMGNT1	0.61124	0.27432	0.35735	0.25389	0.043
4271	217945_at	BTBD1	0.61331	0.66967	0.60766	0.00565	0.485
4272	217946_s_at	SAE1	0.58447	0.43656	0.54639	0.03808	0.35
4273	217947_at	CMTM6	0.42413	0.53035	0.44794	-0.02381	0.615
4274	217949_s_at	VKORC1	0.86017	0.80384	0.79143	0.06874	0.205
4275	217950_at	NOSIP	0.75472	0.59744	0.65579	0.09893	0.141
4276	217955_at	BCL2L13	0.41808	0.5957	0.67188	-0.2538	0.97
4277	217956_s_at	ENOPH1	0.51651	0.63971	0.56314	-0.04663	0.721
4278	217957_at	C16orf80	0.68034	0.6623	0.57765	0.10269	0.173
4279	217959_s_at	TRAPPC4	0.23693	0.48144	0.47234	-0.23541	0.97
4280	217960_s_at	TOMM22	0.71508	0.66387	0.72519	-0.01011	0.555
4281	217961_at	SLC25A38	0.03933	0.2386	0.378	-0.33867	0.945
4282	217962_at	NOP10	0.83469	0.81697	0.8474	-0.01271	0.57
4283	217963_s_at	NGFRAP1	0.015192	0.28097	0.21488	-0.199688	0.924
4284	217964_at	TTC19	0.37589	0.36874	0.31741	0.05848	0.308
4285	217965_s_at	SAP30BP	0.68726	0.42625	0.47655	0.21071	0.003
4286	217966_s_at	FAM129A	0.47401	0.49092	0.45402	0.01999	0.426
4287	217968_at	TSSC1	0.54513	0.53267	0.51293	0.0322	0.39
4288	217969_at	C11orf2	0.76201	0.63779	0.67232	0.08969	0.146
4289	217970_s_at	CNOT6	0.69122	0.6633	0.72303	-0.03181	0.604
4290	217971_at	MAPKSP1	0.51078	0.4348	0.21789	0.29289	0.027

4291	217972_at	CHCHD3	0.75433	0.63986	0.48249	0.27184	<0.001
4292	217973_at	DCXR	0.72791	0.75812	0.73755	-0.00964	0.56
4293	217974_at	TM7SF3	0.3323	0.36102	0.33128	0.00102	0.475
4294	217975_at	WBP5	0.17577	0.24437	0.32637	-0.1506	0.872
4295	217976_s_at	DYNC1LI1	0.55561	0.39773	0.38867	0.16694	0.181
4296	217977_at	SEPX1	0.56231	0.62626	0.418	0.14431	0.209
4297	217978_s_at	UBE2Q1	0.58623	0.53291	0.64044	-0.05421	0.67
4298	217980_s_at	MRPL16	0.80957	0.7721	0.75885	0.05072	0.201
4299	217981_s_at	FXC1	0.46344	0.36668	0.49114	-0.0277	0.62
4300	217982_s_at	MORF4L1	0.51158	0.60212	0.46921	0.04237	0.431
4301	217984_at	RNASET2	0.64981	0.65643	0.42592	0.22389	<0.001
4302	217986_s_at	BAZ1A	0.70907	0.65704	0.74077	-0.0317	0.633
4303	217987_at	ASNSD1	0.56369	0.26329	0.3776	0.18609	0.102
4304	217988_at	CCNB1IP1	0.57865	0.75198	0.68395	-0.1053	0.846
4305	217989_at	HSD17B11	0.028351	0.32191	0.25697	-0.228619	0.942
4306	217990_at	GMPR2	0.43833	0.64235	0.4265	0.01183	0.46
4307	217992_s_at	EFHD2	0.44345	0.38211	0.41097	0.03248	0.447
4308	217993_s_at	MAT2B	0.7136	0.71033	0.50323	0.21037	0.053
4309	217995_at	SQRDL	0.33398	0.5047	0.39263	-0.05865	0.674
4310	217997_at	PHLDA1	0.73618	0.77503	0.81733	-0.08115	0.888
4311	218001_at	MRPS2	0.55223	0.65071	0.59381	-0.04158	0.654
4312	218003_s_at	FKBP3	0.66631	0.77163	0.73145	-0.06514	0.731
4313	218005_at	ZNF22	0.31997	0.46001	0.40734	-0.08737	0.736
4314	218007_s_at	RPS27L	0.85704	0.82001	0.76032	0.09672	0.056
4315	218008_at	C7orf42	0.52505	0.49136	0.48108	0.04397	0.292
4316	218009_s_at	PRC1	0.79696	0.66556	0.76316	0.0338	0.399
4317	218011_at	UBL5	0.72184	0.72602	0.76952	-0.04768	0.697
4318	218014_at	NUP85	0.69675	0.73375	0.61435	0.0824	0.203
4319	218016_s_at	POLR3E	0.56375	0.70774	0.52741	0.03634	0.414
4320	218017_s_at	HGSNAT	0.74341	0.6814	0.72286	0.02055	0.429
4321	218018_at	PDXK	0.13061	0.3566	0.26859	-0.13798	0.789
4322	218020_s_at	ZFAND3	0.70352	0.71561	0.74524	-0.04172	0.734
4323	218021_at	DHRS4	0.32405	0.4217	0.47769	-0.15364	0.848
4324	218022_at	VRK3	0.39084	0.42042	0.20519	0.18565	0.113
4325	218023_s_at	FAM53C	0.65498	0.47565	0.55888	0.0961	0.258
4326	218024_at	BRP44L	0.46298	0.61729	0.65232	-0.18934	0.905
4327	218025_s_at	PECI	0.27833	0.17711	0.081951	0.196379	0.639
4328	218026_at	CCDC56	0.82776	0.86667	0.86893	-0.04117	0.735
4329	218027_at	MRPL15	0.79131	0.78438	0.82852	-0.03721	0.619
4330	218032_at	SNN	0.61581	0.70102	0.73754	-0.12173	0.908
4331	218034_at	FIS1	0.8434	0.7555	0.81376	0.02964	0.336
4332	218035_s_at	RBM47	0.31765	0.31192	0.36537	-0.04772	0.643
4333	218039_at	NUSAP1	0.82033	0.79335	0.76719	0.05314	0.246
4334	218040_at	PRPF38B	0.78324	0.66962	0.55178	0.23146	0.037
4335	218042_at	COPS4	0.61246	0.66351	0.5637	0.04876	0.311
4336	218043_s_at	AZI2	0.79977	0.76476	0.79517	0.0046	0.481
4337	218046_s_at	MRPS16	0.77132	0.7638	0.74891	0.02241	0.377
4338	218047_at	OSBPL9	0.56094	0.56089	0.7025	-0.14156	0.847
4339	218048_at	COMMD3	0.62827	0.69669	0.70979	-0.08152	0.78
4340	218049_s_at	MRPL13	0.87931	0.85129	0.80938	0.06993	0.047
4341	218050_at	UFM1	0.74693	0.46546	0.5756	0.17133	0.043
4342	218051_s_at	NT5DC2	0.53299	0.63385	0.52123	0.01176	0.48
4343	218052_s_at	ATP13A1	0.81547	0.65098	0.652	0.16347	0.023

4344	218055_s_at	WDR41	0.12705	0.22944	0.24381	-0.11676	0.764
4345	218056_at	BFAR	0.7036	0.55648	0.54041	0.16319	0.036
4346	218058_at	CXXC1	0.7222	0.69176	0.63591	0.08629	0.197
4347	218059_at	ZNF706	0.87144	0.80295	0.77364	0.0978	0.096
4348	218061_at	MEA1	0.87728	0.84414	0.8726	0.00468	0.473
4349	218065_s_at	TMEM9B	0.68632	0.63648	0.66912	0.0172	0.429
4350	218066_at	SLC12A7	0.47007	0.55245	0.47819	-0.00812	0.567
4351	218067_s_at	ARGLU1	0.84403	0.62134	0.72501	0.11902	0.035
4352	218068_s_at	ZNF672	0.68638	0.78314	0.71755	-0.03117	0.629
4353	218069_at	DCTPP1	0.80902	0.86102	0.83911	-0.03009	0.712
4354	218070_s_at	GMPPA	0.64515	0.60354	0.65318	-0.00803	0.534
4355	218071_s_at	MKRN2	0.67368	0.55705	0.56228	0.1114	0.138
4356	218072_at	COMMD9	0.39243	0.38256	0.39055	0.00188	0.501
4357	218073_s_at	TMEM48	0.61566	0.69574	0.6218	-0.00614	0.537
4358	218074_at	FAM96B	0.93321	0.88471	0.88116	0.05205	0.024
4359	218075_at	AAAS	0.31567	0.34252	0.33663	-0.02096	0.585
4360	218076_s_at	ARHGAP17	0.72277	0.7636	0.68722	0.03555	0.359
4361	218077_s_at	ZDHHC3	0.50508	0.48631	0.42598	0.0791	0.311
4362	218079_s_at	GGNBP2	0.25555	0.20825	0.18284	0.07271	0.324
4363	218082_s_at	UBP1	0.81024	0.82162	0.82934	-0.0191	0.615
4364	218083_at	PTGES2	0.30295	0.44787	0.33611	-0.03316	0.568
4365	218085_at	CHMP5	0.80339	0.83367	0.83149	-0.0281	0.645
4366	218088_s_at	RRAGC	0.47009	0.5073	0.58824	-0.11815	0.824
4367	218089_at	C20orf4	0.61456	0.58036	0.60845	0.00611	0.479
4368	218090_s_at	BRWD2	0.72544	0.70965	0.61694	0.1085	0.119
4369	218093_s_at	ANKRD10	0.61507	0.54824	0.45224	0.16283	0.097
4370	218095_s_at	TMEM165	0.78726	0.6845	0.71195	0.07531	0.184
4371	218096_at	AGPAT5	0.73248	0.74336	0.78011	-0.04763	0.755
4372	218097_s_at	CUEDC2	0.74743	0.80603	0.73007	0.01736	0.438
4373	218099_at	TEX2	0.41725	0.34449	0.37228	0.04497	0.369
4374	218100_s_at	IFT57	0.56436	0.64276	0.66323	-0.09887	0.784
4375	218101_s_at	NDUFC2	0.79981	0.8032	0.8342	-0.03439	0.71
4376	218102_at	DERA	0.42866	0.67015	0.533	-0.10434	0.725
4377	218103_at	FTSJ3	0.54912	0.6416	0.69857	-0.14945	0.862
4378	218104_at	TEX10	0.61335	0.52417	0.45049	0.16286	0.155
4379	218105_s_at	MRPL4	0.76268	0.70209	0.80742	-0.04474	0.73
4380	218106_s_at	MRPS10	0.22568	0.29125	0.37386	-0.14818	0.891
4381	218107_at	WDR26	0.70402	0.70486	0.51624	0.18778	0.003
4382	218108_at	UBR7	0.69976	0.67713	0.62919	0.07057	0.278
4383	218109_s_at	MFSD1	0.58368	0.64718	0.53815	0.04553	0.344
4384	218110_at	XAB2	0.3568	0.37978	0.35988	-0.00308	0.517
4385	218111_s_at	CMAS	0.52661	0.52649	0.51698	0.00963	0.493
4386	218112_at	MRPS34	0.68139	0.74061	0.70789	-0.0265	0.629
4387	218115_at	ASF1B	0.71512	0.74338	0.75006	-0.03494	0.712
4388	218116_at	C9orf78	0.46762	0.6307	0.6412	-0.17358	0.954
4389	218117_at	RBX1	0.91559	0.87999	0.89545	0.02014	0.244
4390	218118_s_at	TIMM23	0.67123	0.68678	0.70484	-0.03361	0.597
4391	218122_s_at	SENP2	0.71815	0.74577	0.66672	0.05143	0.326
4392	218123_at	C21orf59	0.5791	0.66385	0.47229	0.10681	0.124
4393	218124_at	RETSAT	0.38108	0.51979	0.30539	0.07569	0.268
4394	218125_s_at	CCDC25	0.33542	0.11466	0.20128	0.13414	0.099
4395	218126_at	FAM82A2	0.48334	0.63217	0.59642	-0.11308	0.845
4396	218129_s_at	NFYB	0.61964	0.59888	0.62317	-0.00353	0.534

4397	218130_at	C17orf62	0.75746	0.62602	0.66937	0.08809	0.177
4398	218131_s_at	GATAD2A	0.3334	0.56431	0.27451	0.05889	0.346
4399	218132_s_at	TSEN34	0.56748	0.56462	0.32924	0.23824	0.027
4400	218133_s_at	NIF3L1	0.37752	0.28601	0.32594	0.05158	0.371
4401	218134_s_at	RBM22	0.67197	0.51758	0.70962	-0.03765	0.682
4402	218135_at	ERGIC2	0.32765	0.39082	0.16496	0.16269	0.156
4403	218137_s_at	SMAP1	0.32968	0.62775	0.63164	-0.30196	0.971
4404	218138_at	MKKS	0.353	0.26289	0.36925	-0.01625	0.54
4405	218139_s_at	MUDENG	0.71678	0.67278	0.6817	0.03508	0.439
4406	218142_s_at	CRBN	0.54963	0.39599	0.4859	0.06373	0.311
4407	218143_s_at	SCAMP2	0.4645	0.50295	0.58891	-0.12441	0.818
4408	218145_at	TRIB3	0.061614	0.20683	0.16067	-0.099056	0.741
4409	218147_s_at	GLT8D1	0.71179	0.69144	0.59422	0.11757	0.076
4410	218149_s_at	ZNF395	0.74607	0.60747	0.061263	0.684807	<0.001
4411	218150_at	ARL5A	0.67156	0.64398	0.44572	0.22584	0.03
4412	218152_at	HMG20A	0.53047	0.73452	0.75336	-0.22289	0.981
4413	218153_at	CARS2	0.6538	0.72156	0.68612	-0.03232	0.62
4414	218154_at	GSDMD	0.35693	0.40761	0.47758	-0.12065	0.747
4415	218158_s_at	APPL1	0.62602	0.61849	0.67762	-0.0516	0.678
4416	218159_at	DDRGK1	0.51655	0.61189	0.70848	-0.19193	0.942
4417	218160_at	NDUFA8	0.76433	0.68878	0.76436	-3E-05	0.499
4418	218161_s_at	CLN6	0.39733	0.43029	0.24051	0.15682	0.179
4419	218163_at	MCTS1	0.87671	0.89997	0.88404	-0.00733	0.58
4420	218164_at	SPATA20	0.1629	0.27867	0.27696	-0.11406	0.808
4421	218165_at	C1orf149	0.28843	0.21851	0.17624	0.11219	0.22
4422	218166_s_at	RSF1	0.61728	0.65246	0.70576	-0.08848	0.816
4423	218167_at	AMZ2	0.46819	0.61154	0.56292	-0.09473	0.787
4424	218168_s_at	CABC1	0.37922	0.49511	0.48305	-0.10383	0.896
4425	218170_at	ISOC1	0.85505	0.81875	0.7589	0.09615	0.018
4426	218171_at	VPS4B	0.8143	0.79371	0.79237	0.02193	0.378
4427	218172_s_at	DERL1	0.75275	0.60392	0.60233	0.15042	0.009
4428	218174_s_at	C10orf57	0.23453	0.21144	0.44545	-0.21092	0.844
4429	218176_at	MAGEF1	0.14641	0.19785	0.13034	0.01607	0.43
4430	218178_s_at	CHMP1B	0.67306	0.68644	0.57658	0.09648	0.143
4431	218179_s_at	C4orf41	0.87627	0.75988	0.86313	0.01314	0.379
4432	218181_s_at	MAP4K4	0.48034	0.44475	0.5559	-0.07556	0.659
4433	218183_at	C16orf5	0.30137	0.13938	0.20356	0.09781	0.289
4434	218184_at	TULP4	0.39879	0.61982	0.57806	-0.17927	0.925
4435	218185_s_at	ARMC1	0.76293	0.7374	0.70192	0.06101	0.25
4436	218187_s_at	C8orf33	0.65808	0.43439	0.41731	0.24077	0.009
4437	218188_s_at	TIMM13	0.79072	0.75693	0.74951	0.04121	0.31
4438	218189_s_at	NANS	0.75924	0.78099	0.77825	-0.01901	0.585
4439	218190_s_at	UCRC	0.83351	0.8446	0.85504	-0.02153	0.612
4440	218191_s_at	LMBRD1	0.66944	0.57867	0.51809	0.15135	0.094
4441	218192_at	IP6K2	0.68322	0.52057	0.55206	0.13116	0.09
4442	218193_s_at	GOLT1B	0.56229	0.58827	0.60716	-0.04487	0.705
4443	218194_at	REXO2	0.34789	0.51798	0.49069	-0.1428	0.877
4444	218195_at	C6orf211	0.77532	0.86038	0.79506	-0.01974	0.668
4445	218196_at	OSTM1	0.47308	0.46027	0.41913	0.05395	0.377
4446	218197_s_at	OXR1	0.75993	0.82057	0.78173	-0.0218	0.599
4447	218198_at	DHX32	0.62366	0.49531	0.41103	0.21263	0.029
4448	218201_at	NDUFB2	0.82295	0.82711	0.85591	-0.03296	0.655
4449	218203_at	ALG5	0.67629	0.76536	0.71235	-0.03606	0.689

4450	218204_s_at	FYCO1	0.32552	0.37857	0.37688	-0.05136	0.633
4451	218205_s_at	MKNK2	0.44048	0.45841	0.45781	-0.01733	0.54
4452	218208_at	LOC100131178	0.099171	0.22091	0.22451	-0.125339	0.763
4453	218209_s_at	RPRD1A	0.39299	0.29637	0.29318	0.09981	0.225
4454	218210_at	FN3KRP	0.48812	0.38363	0.5616	-0.07348	0.693
4455	218211_s_at	MLPH	0.35302	0.38297	0.52185	-0.16883	0.829
4456	218212_s_at	MOCS2	0.58861	0.49565	0.5465	0.04211	0.418
4457	218213_s_at	C11orf10	0.84435	0.81426	0.83889	0.00546	0.478
4458	218214_at	C12orf44	0.38874	0.42162	0.44166	-0.05292	0.61
4459	218215_s_at	NR1H2	0.5348	0.51793	0.58576	-0.05096	0.725
4460	218217_at	SCPEP1	0.60765	0.69687	0.73034	-0.12269	0.83
4461	218218_at	APPL2	0.15687	0.18821	0.34814	-0.19127	0.905
4462	218219_s_at	LANCL2	0.40578	0.21043	0.49367	-0.08789	0.746
4463	218220_at	C12orf10	0.48742	0.58169	0.5802	-0.09278	0.731
4464	218221_at	ARNT	0.53098	0.63569	0.69096	-0.15998	0.938
4465	218223_s_at	PLEKHO1	0.62288	0.63584	0.64726	-0.02438	0.62
4466	218224_at	PNMA1	0.55126	0.60742	0.55366	-0.0024	0.562
4467	218225_at	ECSIT	0.45726	0.61166	0.55622	-0.09896	0.767
4468	218226_s_at	NDUFB4	0.82594	0.79621	0.87716	-0.05122	0.753
4469	218227_at	NUBP2	0.35546	0.52142	0.39902	-0.04356	0.58
4470	218228_s_at	TNKS2	0.7219	0.71918	0.72642	-0.00452	0.541
4471	218229_s_at	POGK	0.5924	0.66025	0.59543	-0.00303	0.504
4472	218230_at	ARFIP1	0.71496	0.46095	0.70226	0.0127	0.453
4473	218231_at	NAGK	0.43694	0.48534	0.44385	-0.00691	0.55
4474	218233_s_at	PRICKLE4	0.88931	0.90046	0.9318	-0.04249	0.949
4475	218235_s_at	UTP11L	0.68279	0.77857	0.71102	-0.02823	0.619
4476	218236_s_at	PRKD3	0.24741	0.55735	0.44879	-0.20138	0.918
4477	218237_s_at	SLC38A1	0.64985	0.60576	0.43289	0.21696	0.016
4478	218239_s_at	GTPBP4	0.55724	0.50831	0.36888	0.18836	0.018
4479	218241_at	GOLGA5	0.4433	0.70391	0.65715	-0.21385	0.901
4480	218242_s_at	SUV420H1	0.61112	0.48916	0.50567	0.10545	0.145
4481	218243_at	RUFY1	0.5134	0.40387	0.33367	0.17973	0.15
4482	218244_at	NOL8	0.7223	0.63048	0.52104	0.20126	0.071
4483	218247_s_at	MEX3C	0.7038	0.77101	0.78387	-0.08007	0.773
4484	218248_at	FAM111A	0.59237	0.6597	0.53276	0.05961	0.312
4485	218249_at	ZDHHC6	0.47685	0.38223	0.58748	-0.11063	0.832
4486	218250_s_at	CNOT7	0.33334	0.48624	0.43786	-0.10452	0.787
4487	218251_at	MID1IP1	0.5778	0.55344	0.62294	-0.04514	0.687
4488	218252_at	CKAP2	0.61377	0.70945	0.70654	-0.09277	0.807
4489	218253_s_at	LGTN	0.453	0.48543	0.60355	-0.15055	0.916
4490	218254_s_at	SAR1B	0.91393	0.91907	0.88288	0.03105	0.162
4491	218255_s_at	FBRS	0.2771	0.39364	0.52242	-0.24532	0.943
4492	218257_s_at	UGCGL1	0.66527	0.55606	0.58768	0.07759	0.26
4493	218258_at	POLR1D	0.65941	0.78801	0.72745	-0.06804	0.629
4494	218259_at	MKL2	0.45055	0.26811	0.64845	-0.1979	0.93
4495	218260_at	DDA1	0.63188	0.59333	0.58453	0.04735	0.35
4496	218263_s_at	ZBED5	0.7401	0.61113	0.68388	0.05622	0.285
4497	218264_at	BCCIP	0.38577	0.44849	0.52367	-0.1379	0.878
4498	218265_at	SECISBP2	0.25707	0.39355	0.27969	-0.02262	0.566
4499	218267_at	CINP	0.4017	0.45212	0.55046	-0.14876	0.8
4500	218268_at	TBC1D15	0.42297	0.50292	0.44004	-0.01707	0.582
4501	218269_at	RNASEN	0.79856	0.74844	0.64272	0.15584	0.004
4502	218270_at	MRPL24	0.56739	0.6354	0.59492	-0.02753	0.59

4503	218271_s_at	PARL	0.40194	0.57273	0.65868	-0.25674	0.934
4504	218273_s_at	PPM2C	0.44262	0.45975	0.50386	-0.06124	0.697
4505	218275_at	SLC25A10	0.52882	0.40821	0.41335	0.11547	0.241
4506	218276_s_at	SAV1	0.26964	0.51496	0.3805	-0.11086	0.731
4507	218277_s_at	DHX40	0.67809	0.71748	0.68799	-0.0099	0.559
4508	218278_at	LOC649169	0.35182	0.080031	0.12065	0.23117	0.036
4509	218281_at	MRPL48	0.45303	0.68869	0.63256	-0.17953	0.919
4510	218282_at	EDEM2	0.47326	0.43124	0.54909	-0.07583	0.759
4511	218283_at	SS18L2	0.84691	0.87959	0.81147	0.03544	0.302
4512	218284_at	SMAD3	0.20706	0.25207	0.20919	-0.00213	0.544
4513	218285_s_at	BDH2	0.48436	0.65296	0.55411	-0.06975	0.7
4514	218286_s_at	RNF7	0.74841	0.5918	0.62427	0.12414	0.053
4515	218287_s_at	EIF2C1	0.49759	0.56335	0.48755	0.01004	0.481
4516	218288_s_at	CCDC90B	0.60035	0.35629	0.42935	0.171	0.08
4517	218289_s_at	UBA5	0.66852	0.36001	0.45352	0.215	0.057
4518	218290_at	PLEKHJ1	0.54976	0.60622	0.61418	-0.06442	0.639
4519	218291_at	ROBLD3	0.80179	0.77324	0.7742	0.02759	0.368
4520	218294_s_at	NUP50	0.76907	0.67531	0.61039	0.15868	0.046
4521	218297_at	C10orf97	0.26498	0.19749	0.33621	-0.07123	0.697
4522	218298_s_at	C14orf159	0.37852	0.33368	0.43413	-0.05561	0.672
4523	218301_at	RNPEPL1	0.2409	0.18146	0.22071	0.02019	0.439
4524	218302_at	PSENEN	0.7542	0.66465	0.69587	0.05833	0.325
4525	218304_s_at	OSBPL11	0.45897	0.66256	0.65122	-0.19225	0.934
4526	218305_at	IPO4	0.69241	0.72692	0.69198	0.00043	0.491
4527	218306_s_at	HERC1	0.51452	0.61208	0.67076	-0.15624	0.918
4528	218307_at	RSAD1	0.20724	0.2046	0.087115	0.120125	0.271
4529	218308_at	TACC3	0.62098	0.5482	0.2923	0.32868	0.011
4530	218310_at	RABGEF1	0.63269	0.57265	0.5828	0.04989	0.32
4531	218311_at	MAP4K3	0.27604	0.54255	0.38283	-0.10679	0.75
4532	218314_s_at	C11orf57	0.74748	0.65609	0.50031	0.24717	0.019
4533	218315_s_at	CDK5RAP1	0.5216	0.33916	0.45327	0.06833	0.357
4534	218316_at	TIMM9	0.74437	0.58575	0.69876	0.04561	0.307
4535	218318_s_at	NLK	0.40946	0.53375	0.49213	-0.08267	0.751
4536	218319_at	PELI1	0.41493	0.33971	0.6731	-0.25817	0.962
4537	218320_s_at	NDUFB11	0.85992	0.87044	0.87847	-0.01855	0.605
4538	218322_s_at	ACSL5	0.75715	0.62564	0.58434	0.17281	0.037
4539	218324_s_at	SPATS2	0.62018	0.60789	0.61709	0.00309	0.492
4540	218326_s_at	LGR4	0.018503	0.1135	0.064328	-0.045825	0.649
4541	218327_s_at	SNAP29	0.48899	0.30391	0.3762	0.11279	0.251
4542	218328_at	COQ4	0.29798	0.49587	0.41422	-0.11624	0.792
4543	218330_s_at	NAV2	0.55647	0.58107	0.54127	0.0152	0.454
4544	218331_s_at	C10orf18	0.75794	0.73795	0.76626	-0.00832	0.579
4545	218333_at	DERL2	0.62946	0.62914	0.60468	0.02478	0.441
4546	218334_at	THOC7	0.84934	0.81735	0.85719	-0.00785	0.529
4547	218336_at	PFDN2	0.88763	0.87334	0.88257	0.00506	0.463
4548	218337_at	FAM160B2	0.67725	0.56138	0.43635	0.2409	0.009
4549	218339_at	MRPL22	0.89491	0.87229	0.87315	0.02176	0.327
4550	218340_s_at	UBA6	0.2562	0.40483	0.41406	-0.15786	0.76
4551	218341_at	PPCS	0.2449	0.3089	0.35794	-0.11304	0.752
4552	218343_s_at	GTF3C3	0.64549	0.69099	0.63463	0.01086	0.467
4553	218344_s_at	RCOR3	0.4917	0.57964	0.5672	-0.0755	0.687
4554	218346_s_at	SESN1	0.45148	0.53728	0.1405	0.31098	<0.001
4555	218347_at	TYW1	0.42217	0.4637	0.46792	-0.04575	0.66

4556	218348_s_at	ZC3H7A	0.71479	0.65064	0.63882	0.07597	0.205
4557	218349_s_at	ZWILCH	0.81924	0.79469	0.79915	0.02009	0.347
4558	218350_s_at	GMNN	0.78223	0.83318	0.75688	0.02535	0.384
4559	218351_at	COMMD8	0.7454	0.66555	0.53807	0.20733	0.161
4560	218352_at	RCBTB1	0.84045	0.81339	0.74939	0.09106	0.039
4561	218354_at	TRAPPC2L	0.78091	0.59923	0.68654	0.09437	0.17
4562	218355_at	KIF4A	0.67628	0.77952	0.67461	0.00167	0.505
4563	218357_s_at	TIMM8B	0.80208	0.79288	0.82934	-0.02726	0.715
4564	218358_at	CRELD2	0.24883	0.53334	0.48125	-0.23242	0.915
4565	218361_at	GOLPH3L	0.65772	0.39464	0.70024	-0.04252	0.663
4566	218362_s_at	DIS3	0.77803	0.53713	0.53985	0.23818	0.001
4567	218363_at	EXD2	0.37098	0.36128	0.54011	-0.16913	0.883
4568	218364_at	LRRFIP2	0.11369	0.35418	0.19072	-0.07703	0.714
4569	218365_s_at	DARS2	0.70477	0.59088	0.62231	0.08246	0.221
4570	218370_s_at	S100PBP	0.61774	0.59246	0.66277	-0.04503	0.672
4571	218372_at	MED9	0.04642	0.095079	0.039837	0.006583	0.464
4572	218373_at	AKTIP	0.40247	0.33483	0.3088	0.09367	0.273
4573	218374_s_at	C12orf4	0.60456	0.47533	0.73876	-0.1342	0.877
4574	218375_at	NUDT9	0.61336	0.51461	0.2615	0.35186	0.005
4575	218376_s_at	MICAL1	0.58864	0.72328	0.55874	0.0299	0.377
4576	218377_s_at	RWDD2B	0.54043	0.50815	0.31927	0.22116	0.014
4577	218378_s_at	PRKRIP1	0.46923	0.26906	0.31123	0.158	0.084
4578	218379_at	RBM7	0.6557	0.60011	0.68708	-0.03138	0.687
4579	218381_s_at	U2AF2	0.64854	0.6173	0.63069	0.01785	0.449
4580	218383_at	HAUS4	0.52766	0.62079	0.60893	-0.08127	0.73
4581	218384_at	CARHSP1	0.80214	0.77068	0.76221	0.03993	0.367
4582	218385_at	MRPS18A	0.65813	0.66486	0.75995	-0.10182	0.818
4583	218388_at	PGLS	0.81606	0.79715	0.82905	-0.01299	0.537
4584	218389_s_at	APH1A	0.62016	0.45185	0.50648	0.11368	0.269
4585	218391_at	SNF8	0.61121	0.53401	0.71074	-0.09953	0.843
4586	218393_s_at	SMU1	0.40055	0.37876	0.40496	-0.00441	0.512
4587	218394_at	ROGDI	0.25823	0.24667	0.37835	-0.12012	0.779
4588	218395_at	ACTR6	0.79919	0.70252	0.5908	0.20839	0.005
4589	218396_at	VPS13C	0.7149	0.71727	0.69014	0.02476	0.381
4590	218397_at	FANCL	0.81325	0.62639	0.68886	0.12439	0.036
4591	218399_s_at	CDCA4	0.53358	0.51956	0.52555	0.00803	0.449
4592	218400_at	OAS3	0.72953	0.75634	0.75098	-0.02145	0.598
4593	218401_s_at	ZNF281	0.68417	0.66741	0.68695	-0.00278	0.491
4594	218403_at	TRIAP1	0.51479	0.52338	0.40532	0.10947	0.156
4595	218404_at	SNX10	0.66557	0.67272	0.68949	-0.02392	0.679
4596	218405_at	ABT1	0.24816	0.43325	0.48092	-0.23276	0.979
4597	218408_at	TIMM10	0.78951	0.73082	0.75882	0.03069	0.354
4598	218411_s_at	MBIP	0.32728	0.29069	0.47328	-0.146	0.879
4599	218414_s_at	NDE1	0.38426	0.34862	0.41607	-0.03181	0.627
4600	218415_at	VPS33B	0.53238	0.40703	0.36873	0.16365	0.037
4601	218419_s_at	TMUB2	0.28758	0.051543	0.2727	0.01488	0.484
4602	218420_s_at	C13orf23	0.60624	0.61351	0.56355	0.04269	0.363
4603	218421_at	CERK	0.48188	0.43069	0.18797	0.29391	0.008
4604	218422_s_at	RBM26	0.70085	0.73524	0.59542	0.10543	0.11
4605	218424_s_at	STEAP3	0.40096	0.38014	0.25687	0.14409	0.168
4606	218426_s_at	RNF216	0.62175	0.4763	0.57811	0.04364	0.359
4607	218427_at	SDCCAG3	0.3891	0.3701	0.29422	0.09488	0.247
4608	218428_s_at	REV1	0.56626	0.52202	0.44254	0.12372	0.176

4609	218429_s_at	C19orf66	0.42937	0.47251	0.51931	-0.08994	0.801
4610	218430_s_at	RFX7	0.426	0.55027	0.54237	-0.11637	0.848
4611	218431_at	C14orf133	0.81724	0.61884	0.75084	0.0664	0.163
4612	218432_at	FBXO3	0.60287	0.64791	0.71419	-0.11132	0.899
4613	218433_at	PANK3	0.0039458	0.016456	0.097535	-0.0935892	0.74
4614	218434_s_at	AACS	0.28364	0.44524	0.43123	-0.14759	0.867
4615	218435_at	DNAJC15	0.3014	0.48585	0.52971	-0.22831	0.883
4616	218436_at	SIL1	0.36487	0.45038	0.53006	-0.16519	0.859
4617	218437_s_at	LZTFL1	0.57956	0.51147	0.62836	-0.0488	0.659
4618	218439_s_at	COMMD10	0.13195	0.28064	0.25438	-0.12243	0.769
4619	218440_at	MCCC1	0.5324	0.5194	0.5086	0.0238	0.45
4620	218441_s_at	RPAP1	0.46084	0.46874	0.33	0.13084	0.218
4621	218443_s_at	DAZAP1	0.51257	0.63805	0.6362	-0.12363	0.872
4622	218446_s_at	FAM18B	0.84898	0.7993	0.71259	0.13639	0.013
4623	218447_at	C16orf61	0.73699	0.78809	0.84361	-0.10662	0.772
4624	218448_at	C20orf11	0.2346	0.41315	0.27341	-0.03881	0.619
4625	218449_at	UFSP2	0.13817	0.17302	0.13087	0.0073	0.504
4626	218452_at	SMARCAL1	0.48502	0.32598	0.28418	0.20084	0.061
4627	218455_at	NFS1	0.24473	0.30396	0.37784	-0.13311	0.812
4628	218456_at	CAPRIN2	0.28854	0.24196	0.44348	-0.15494	0.934
4629	218458_at	GMCL1	0.65268	0.69252	0.76326	-0.11058	0.957
4630	218459_at	TOR3A	0.3522	0.23285	0.39121	-0.03901	0.672
4631	218460_at	HEATR2	0.53972	0.56663	0.55435	-0.01463	0.579
4632	218461_at	GPN3	0.39559	0.39979	0.43979	-0.0442	0.596
4633	218462_at	BXDC5	0.76678	0.76264	0.63679	0.12999	0.148
4634	218463_s_at	MUS81	0.55812	0.47015	0.33661	0.22151	0.026
4635	218465_at	TMEM33	0.61496	0.44924	0.64145	-0.02649	0.59
4636	218467_at	PSMG2	0.83968	0.90177	0.88435	-0.04467	0.824
4637	218470_at	YARS2	0.41018	0.45204	0.41116	-0.00098	0.475
4638	218471_s_at	BBS1	0.68533	0.67883	0.62737	0.05796	0.234
4639	218473_s_at	GLT25D1	0.56116	0.57354	0.27324	0.28792	0.051
4640	218474_s_at	KCTD5	0.55525	0.68771	0.63257	-0.07732	0.8
4641	218477_at	TMEM14A	0.62005	0.56448	0.61802	0.00203	0.482
4642	218478_s_at	ZCCHC8	0.21425	0.53996	0.37329	-0.15904	0.838
4643	218479_s_at	XPO4	0.57227	0.43341	0.40211	0.17016	0.12
4644	218481_at	EXOSC5	0.47034	0.58422	0.68113	-0.21079	0.937
4645	218482_at	ENY2	0.8958	0.8565	0.88363	0.01217	0.446
4646	218483_s_at	C11orf60	0.33967	0.20115	0.35309	-0.01342	0.509
4647	218487_at	ALAD	0.31344	0.24006	0.35053	-0.03709	0.584
4648	218488_at	EIF2B3	0.62898	0.52158	0.43011	0.19887	0.027
4649	218490_s_at	ZNF302	0.55535	0.50795	0.52667	0.02868	0.433
4650	218491_s_at	THYN1	0.61299	0.71264	0.62783	-0.01484	0.558
4651	218492_s_at	THAP7	0.33664	0.33364	0.18866	0.14798	0.152
4652	218493_at	SNRNP25	0.68148	0.77131	0.80447	-0.12299	0.912
4653	218494_s_at	SLC2A4RG	0.15838	0.17637	0.29399	-0.13561	0.824
4654	218495_at	UXT	0.9099	0.90665	0.91587	-0.00597	0.581
4655	218496_at	RNASEH1	0.67037	0.35742	0.32808	0.34229	0.001
4656	218498_s_at	ERO1L	0.49494	0.50737	0.49338	0.00156	0.495
4657	218499_at	RP6-213H19.1	0.52168	0.58476	0.59435	-0.07267	0.793
4658	218501_at	ARHGEF3	0.36839	0.51459	0.39957	-0.03118	0.527
4659	218503_at	KIAA1797	0.50242	0.48425	0.53261	-0.03019	0.601
4660	218507_at	C7orf68	0.61625	0.29045	0.24361	0.37264	0.007
4661	218508_at	DCP1A	0.47428	0.67028	0.6161	-0.14182	0.939

4662	218511_s_at	PNPO	0.25215	0.3834	0.46945	-0.2173	0.972
4663	218512_at	WDR12	0.70157	0.48738	0.59104	0.11053	0.189
4664	218513_at	C4orf43	0.86966	0.74986	0.75392	0.11574	0.005
4665	218514_at	C17orf71	0.69731	0.74255	0.69241	0.0049	0.487
4666	218515_at	C21orf66	0.28836	0.36528	0.31925	-0.03089	0.558
4667	218516_s_at	IMPAD1	0.0021007	0.069981	0.14418	-0.1420793	0.682
4668	218517_at	PHF17	0.27479	0.30707	0.27173	0.00306	0.525
4669	218518_at	FAM13B	0.63696	0.41994	0.45414	0.18282	0.072
4670	218519_at	SLC35A5	0.59576	0.57043	0.54363	0.05213	0.263
4671	218520_at	TBK1	0.39651	0.37091	0.36745	0.02906	0.416
4672	218521_s_at	UBE2W	0.29901	0.56774	0.5391	-0.24009	0.991
4673	218524_at	E4F1	0.46383	0.56169	0.53094	-0.06711	0.692
4674	218525_s_at	HIF1AN	0.26353	0.45876	0.29819	-0.03466	0.628
4675	218526_s_at	RANGRF	0.80281	0.80199	0.80793	-0.00512	0.469
4676	218527_at	APTX	0.33189	0.51181	0.24431	0.08758	0.161
4677	218528_s_at	RNF38	0.77828	0.81264	0.77432	0.00396	0.487
4678	218529_at	CD320	0.48683	0.49266	0.56312	-0.07629	0.681
4679	218531_at	TMEM134	0.61388	0.53512	0.66161	-0.04773	0.704
4680	218532_s_at	FAM134B	0.013947	0.078512	0.22866	-0.214713	0.962
4681	218533_s_at	UCKL1	0.54424	0.32818	0.41795	0.12629	0.099
4682	218534_s_at	AGGF1	0.77372	0.71973	0.76457	0.00915	0.465
4683	218535_s_at	RIOK2	0.38683	0.045461	0.21308	0.17375	0.116
4684	218536_at	MRS2	0.63408	0.62445	0.60412	0.02996	0.402
4685	218539_at	FBXO34	0.47785	0.54107	0.49309	-0.01524	0.566
4686	218542_at	CEP55	0.86839	0.78268	0.77692	0.09147	0.103
4687	218543_s_at	PARP12	0.74375	0.77867	0.70064	0.04311	0.368
4688	218544_s_at	RCL1	0.45893	0.50552	0.52523	-0.0663	0.651
4689	218545_at	CCDC91	0.74897	0.63201	0.65121	0.09776	0.172
4690	218547_at	DHDDS	0.35839	0.42524	0.32224	0.03615	0.408
4691	218549_s_at	FAM82B	0.77384	0.85681	0.7382	0.03564	0.317
4692	218550_s_at	LRRC20	0.54928	0.47384	0.46173	0.08755	0.229
4693	218552_at	ECHDC2	0.17034	0.22112	0.2601	-0.08976	0.701
4694	218555_at	ANAPC2	0.36655	0.45504	0.34973	0.01682	0.421
4695	218556_at	ORMDL2	0.66511	0.73171	0.78033	-0.11522	0.835
4696	218557_at	NIT2	0.61949	0.66917	0.74947	-0.12998	0.849
4697	218558_s_at	MRPL39	0.34087	0.55798	0.53138	-0.19051	0.965
4698	218561_s_at	LYRM4	0.71655	0.68646	0.82889	-0.11234	0.879
4699	218562_s_at	TMEM57	0.41998	0.53239	0.41247	0.00751	0.473
4700	218563_at	NDUFA3	0.83329	0.74766	0.79657	0.03672	0.356
4701	218564_at	RFWD3	0.3965	0.42386	0.528	-0.1315	0.809
4702	218565_at	C9orf114	0.69492	0.66039	0.68883	0.00609	0.453
4703	218566_s_at	CHORDC1	0.88267	0.75113	0.40425	0.47842	<0.001
4704	218568_at	AGK	0.54746	0.60416	0.4591	0.08836	0.245
4705	218570_at	KBTBD4	0.66452	0.41408	0.49507	0.16945	0.032
4706	218571_s_at	CHMP4A	0.80327	0.63706	0.65933	0.14394	0.168
4707	218573_at	MAGEH1	0.21605	0.29163	0.3245	-0.10845	0.774
4708	218574_s_at	LMCD1	0.21696	0.077669	0.30962	-0.09266	0.743
4709	218575_at	ANAPC1	0.64485	0.64399	0.48953	0.15532	0.109
4710	218576_s_at	DUSP12	0.38448	0.2938	0.18582	0.19866	0.096
4711	218577_at	LRRC40	0.76529	0.81819	0.70408	0.06121	0.288
4712	218578_at	CDC73	0.73683	0.77808	0.77	-0.03317	0.674
4713	218579_s_at	DHX35	0.59546	0.52501	0.46303	0.13243	0.186
4714	218581_at	ABHD4	0.48084	0.53913	0.47422	0.00662	0.499

4715	218582_at	MARCH5	0.60217	0.53974	0.53412	0.06805	0.251
4716	218583_s_at	DCUN1D1	0.40917	0.57346	0.45401	-0.04484	0.638
4717	218584_at	TCTN1	0.24341	0.28425	0.15366	0.08975	0.252
4718	218585_s_at	DTL	0.41233	0.53023	0.46747	-0.05514	0.673
4719	218586_at	C20orf20	0.4159	0.46921	0.43595	-0.02005	0.602
4720	218587_s_at	KTELC1	0.40584	0.40886	0.31205	0.09379	0.234
4721	218588_s_at	FAM114A2	0.48737	0.59156	0.68896	-0.20159	0.92
4722	218589_at	P2RY5	0.061374	0.21359	0.23972	-0.178346	0.921
4723	218590_at	C10orf2	0.18008	0.51419	0.32036	-0.14028	0.905
4724	218591_s_at	NOL10	0.2968	0.19635	0.07875	0.21805	0.112
4725	218592_s_at	CECR5	0.46095	0.59024	0.27516	0.18579	0.057
4726	218593_at	RBM28	0.61731	0.66733	0.65423	-0.03692	0.604
4727	218594_at	HEATR1	0.76968	0.77363	0.78568	-0.016	0.58
4728	218597_s_at	CISD1	0.84635	0.83886	0.8414	0.00495	0.486
4729	218598_at	RINT1	0.6709	0.76515	0.61558	0.05532	0.228
4730	218599_at	REC8	0.31407	0.47807	0.42099	-0.10692	0.724
4731	218602_s_at	HAUS6	0.64286	0.71655	0.75883	-0.11597	0.884
4732	218603_at	HECA	0.25563	0.52411	0.57013	-0.3145	0.986
4733	218604_at	LEMD3	0.17618	0.32143	0.31095	-0.13477	0.85
4734	218605_at	TFB2M	0.70141	0.50763	0.40342	0.29799	0.007
4735	218606_at	ZDHHC7	0.39733	0.37958	0.31653	0.0808	0.335
4736	218607_s_at	SDAD1	0.40933	0.67855	0.53337	-0.12404	0.827
4737	218609_s_at	NUDT2	0.58477	0.54827	0.58056	0.00421	0.493
4738	218610_s_at	CPPED1	0.14	0.20008	0.16	-0.02	0.544
4739	218611_at	IER5	0.6901	0.084467	0.22032	0.46978	<0.001
4740	218614_at	C12orf35	0.52153	0.46165	0.52032	0.00121	0.521
4741	218615_s_at	TMEM39A	0.44623	0.081749	0.13193	0.3143	0.016
4742	218616_at	INTS12	0.29775	0.27099	0.19822	0.09953	0.256
4743	218617_at	TRIT1	0.19555	0.40827	0.41113	-0.21558	0.966
4744	218618_s_at	FNDC3B	0.60952	0.58114	0.74471	-0.13519	0.909
4745	218619_s_at	SUV39H1	0.56982	0.56372	0.53602	0.0338	0.401
4746	218622_at	NUP37	0.52787	0.63188	0.53964	-0.01177	0.541
4747	218624_s_at	MGC2752	0.19308	0.28668	0.35187	-0.15879	0.881
4748	218626_at	EIF4ENIF1	0.59557	0.66639	0.58994	0.00563	0.513
4749	218627_at	DRAM	0.56253	0.54419	0.45873	0.1038	0.226
4750	218628_at	CCDC53	0.55117	0.41771	0.49678	0.05439	0.33
4751	218630_at	MKS1	0.40337	0.2521	0.20483	0.19854	0.121
4752	218632_at	HECTD3	0.54005	0.63095	0.68967	-0.14962	0.953
4753	218634_at	PHLDA3	0.71693	0.75506	0.71682	0.00011	0.553
4754	218636_s_at	MAN1B1	0.36152	0.34812	0.30308	0.05844	0.354
4755	218637_at	IMPACT	0.47981	0.50673	0.43742	0.04239	0.367
4756	218640_s_at	PLEKHF2	0.59748	0.7287	0.70727	-0.10979	0.889
4757	218641_at	LOC65998	0.22745	0.37191	0.28048	-0.05303	0.619
4758	218642_s_at	CHCHD7	0.80771	0.68728	0.53364	0.27407	<0.001
4759	218643_s_at	CRIPT	0.6057	0.44859	0.41586	0.18984	0.041
4760	218645_at	ZNF277	0.66756	0.80798	0.75717	-0.08961	0.769
4761	218646_at	C4orf27	0.43647	0.4855	0.3525	0.08397	0.258
4762	218647_s_at	YRDC	0.63643	0.60338	0.56883	0.0676	0.213
4763	218648_at	CRTC3	0.76335	0.59536	0.45495	0.3084	0.008
4764	218650_at	DGCR8	0.547	0.68087	0.55663	-0.00963	0.507
4765	218652_s_at	PIGG	0.3793	0.47381	0.627	-0.2477	0.988
4766	218653_at	SLC25A15	0.32202	0.40213	0.1457	0.17632	0.129
4767	218654_s_at	MRPS33	0.86724	0.7873	0.7937	0.07354	0.114

4768	218655_s_at	CCDC49	0.2962	0.37304	0.36211	-0.06591	0.65
4769	218656_s_at	LHFP	0.33926	0.4212	0.42677	-0.08751	0.76
4770	218658_s_at	ACTR8	0.25435	0.30065	0.21632	0.03803	0.413
4771	218659_at	ASXL2	0.5673	0.57787	0.64016	-0.07286	0.719
4772	218661_at	NAT15	0.20215	0.36532	0.36149	-0.15934	0.818
4773	218663_at	NCAPG	0.80233	0.80703	0.71141	0.09092	0.121
4774	218664_at	MECR	0.34553	0.25522	0.21829	0.12724	0.213
4775	218666_s_at	STX17	0.037011	0.21132	0.18849	-0.151479	0.849
4776	218667_at	PJA1	0.18228	0.3835	0.28719	-0.10491	0.769
4777	218669_at	RAP2C	0.57954	0.55874	0.63308	-0.05354	0.703
4778	218670_at	PUS1	0.25819	0.39074	0.22046	0.03773	0.384
4779	218671_s_at	ATPIF1	0.8753	0.83264	0.8423	0.033	0.229
4780	218672_at	SCNM1	0.81134	0.86637	0.87531	-0.06397	0.883
4781	218673_s_at	ATG7	0.54751	0.3408	0.4766	0.07091	0.263
4782	218674_at	C5orf44	0.73491	0.6747	0.72293	0.01198	0.447
4783	218676_s_at	PCTP	0.43098	0.37413	0.40481	0.02617	0.409
4784	218679_s_at	VPS28	0.53138	0.56825	0.65879	-0.12741	0.819
4785	218681_s_at	SDF2L1	0.45672	0.59878	0.58425	-0.12753	0.805
4786	218682_s_at	SLC4A1AP	0.50106	0.63494	0.53956	-0.0385	0.64
4787	218683_at	PTBP2	0.61928	0.61006	0.75546	-0.13618	0.877
4788	218684_at	LRRC8D	0.70831	0.59196	0.59485	0.11346	0.141
4789	218685_s_at	SMUG1	0.090603	0.141	0.080762	0.009841	0.504
4790	218688_at	DAK	0.6981	0.62488	0.62683	0.07127	0.278
4791	218689_at	FANCF	0.30906	0.028841	0.18144	0.12762	0.17
4792	218692_at	GOLSYN	0.25532	0.35401	0.277	-0.02168	0.568
4793	218693_at	TSPAN15	0.15741	0.099597	0.10907	0.04834	0.414
4794	218694_at	ARMCX1	0.14725	0.26797	0.30493	-0.15768	0.855
4795	218696_at	EIF2AK3	0.55053	0.58234	0.69001	-0.13948	0.898
4796	218698_at	APIP	0.52814	0.37411	0.42296	0.10518	0.214
4797	218699_at	RAB7L1	0.62722	0.60008	0.62974	-0.00252	0.58
4798	218701_at	LACTB2	0.54874	0.66324	0.58424	-0.0355	0.596
4799	218703_at	SEC22A	0.23149	0.21878	0.24907	-0.01758	0.587
4800	218705_s_at	SNX24	0.39645	0.5408	0.42749	-0.03104	0.623
4801	218706_s_at	GRAMD3	0.51859	0.46067	0.40814	0.11045	0.169
4802	218708_at	NXT1	0.78019	0.76284	0.69742	0.08277	0.176
4803	218709_s_at	IFT52	0.15464	0.25518	0.29585	-0.14121	0.821
4804	218710_at	TTC27	0.17968	0.32756	0.32039	-0.14071	0.83
4805	218712_at	C1orf109	0.79674	0.72671	0.77054	0.0262	0.334
4806	218713_at	NARG2	0.42347	0.51981	0.49505	-0.07158	0.687
4807	218715_at	UTP6	0.41348	0.64684	0.72981	-0.31633	0.997
4808	218719_s_at	GINS3	0.3413	0.34871	0.17652	0.16478	0.127
4809	218721_s_at	C1orf27	0.51752	0.63955	0.53373	-0.01621	0.552
4810	218722_s_at	CCDC51	0.4042	0.26121	0.40076	0.00344	0.473
4811	218723_s_at	C13orf15	0.45704	0.54906	0.42535	0.03169	0.396
4812	218726_at	HJURP	0.69457	0.34855	0.55556	0.13901	0.135
4813	218728_s_at	CNIH4	0.80652	0.78674	0.83108	-0.02456	0.592
4814	218729_at	LXN	0.3594	0.40406	0.43161	-0.07221	0.695
4815	218732_at	PTRH2	0.74948	0.78197	0.73381	0.01567	0.447
4816	218733_at	MSL2	0.59699	0.56598	0.56851	0.02848	0.401
4817	218735_s_at	ZNF544	0.48472	0.31339	0.5107	-0.02598	0.552
4818	218738_s_at	RNF138	0.81427	0.75229	0.81085	0.00342	0.444
4819	218739_at	ABHD5	0.29699	0.39204	0.39748	-0.10049	0.73
4820	218740_s_at	CDK5RAP3	0.65754	0.60634	0.57462	0.08292	0.267

4821	218741_at	CENPM	0.67617	0.59944	0.65727	0.0189	0.469
4822	218743_at	CHMP6	0.14731	0.37894	0.44189	-0.29458	0.97
4823	218746_at	TAPBPL	0.10874	0.13197	0.22321	-0.11447	0.823
4824	218748_s_at	EXOC5	0.79494	0.85032	0.84607	-0.05113	0.861
4825	218751_s_at	FBXW7	0.54531	0.56409	0.4537	0.09161	0.271
4826	218753_at	XKR8	0.71619	0.72951	0.77248	-0.05629	0.779
4827	218754_at	NOL9	0.7171	0.7756	0.79759	-0.08049	0.779
4828	218755_at	KIF20A	0.68377	0.43742	0.23173	0.45204	<0.001
4829	218756_s_at	DHRS11	0.28506	0.33167	0.42478	-0.13972	0.847
4830	218757_s_at	UPF3B	0.7474	0.7083	0.61573	0.13167	0.036
4831	218760_at	COQ6	0.40907	0.25899	0.24594	0.16313	0.104
4832	218761_at	RNF111	0.66747	0.52756	0.61679	0.05068	0.325
4833	218763_at	STX18	0.44567	0.27264	0.30329	0.14238	0.147
4834	218764_at	PRKCH	0.25878	0.33904	0.38128	-0.1225	0.8
4835	218766_s_at	WARS2	0.12216	0.20039	0.28232	-0.16016	0.837
4836	218767_at	REXO4	0.42912	0.48132	0.52527	-0.09615	0.77
4837	218768_at	NUP107	0.70821	0.70501	0.69863	0.00958	0.467
4838	218769_s_at	ANKRA2	0.56962	0.65868	0.52158	0.04804	0.322
4839	218770_s_at	TMEM39B	0.60504	0.5946	0.56112	0.04392	0.327
4840	218771_at	PANK4	0.33196	0.37472	0.41133	-0.07937	0.681
4841	218774_at	DCPS	0.55668	0.62891	0.58222	-0.02554	0.601
4842	218776_s_at	TMEM62	0.45422	0.26474	0.25948	0.19474	0.158
4843	218777_at	REEP4	0.49615	0.55574	0.54305	-0.0469	0.626
4844	218781_at	SMC6	0.56201	0.61143	0.65664	-0.09463	0.718
4845	218782_s_at	ATAD2	0.59456	0.69142	0.66586	-0.0713	0.739
4846	218788_s_at	SMYD3	0.35858	0.35047	0.40919	-0.05061	0.652
4847	218789_s_at	C11orf71	0.13897	0.17393	0.11044	0.02853	0.439
4848	218791_s_at	C15orf29	0.32262	0.43398	0.24112	0.0815	0.279
4849	218794_s_at	TXNL4B	0.64416	0.55032	0.58179	0.06237	0.339
4850	218795_at	ACP6	0.17997	0.32947	0.34049	-0.16052	0.813
4851	218797_s_at	SIRT7	0.2498	0.46862	0.5402	-0.2904	0.98
4852	218799_at	GPN2	0.46422	0.6203	0.59672	-0.1325	0.883
4853	218801_at	UGCGL2	0.14649	0.12004	0.17153	-0.02504	0.581
4854	218802_at	CCDC109B	0.52263	0.61198	0.57258	-0.04995	0.683
4855	218803_at	CHFR	0.32792	0.51566	0.34845	-0.02053	0.559
4856	218809_at	PANK2	0.45245	0.51298	0.20093	0.25152	0.024
4857	218810_at	ZC3H12A	0.60778	0.41454	0.40988	0.1979	0.064
4858	218812_s_at	ORAI2	0.45356	0.52307	0.43532	0.01824	0.457
4859	218813_s_at	SH3GLB2	0.36077	0.22773	0.1631	0.19767	0.373
4860	218817_at	SPCS3	0.50751	0.63641	0.60618	-0.09867	0.862
4861	218823_s_at	KCTD9	0.63219	0.59283	0.61261	0.01958	0.47
4862	218826_at	SLC35F2	0.18745	0.20819	0.17637	0.01108	0.481
4863	218827_s_at	CEP192	0.33394	0.30922	0.3882	-0.05426	0.704
4864	218830_at	RPL26L1	0.74035	0.78023	0.83641	-0.09606	0.792
4865	218833_at	ZAK	0.20495	0.12767	0.12857	0.07638	0.274
4866	218836_at	RPP21	0.78835	0.80524	0.82542	-0.03707	0.775
4867	218837_s_at	UBE2D4	0.59549	0.62522	0.53621	0.05928	0.293
4868	218838_s_at	TTC31	0.72705	0.56476	0.65981	0.06724	0.21
4869	218840_s_at	NADSYN1	0.78007	0.64208	0.6829	0.09717	0.05
4870	218841_at	ASB8	0.50934	0.38784	0.34737	0.16197	0.1
4871	218842_at	RPAP3	0.69707	0.6451	0.74727	-0.0502	0.71
4872	218845_at	DUSP22	0.31705	0.35773	0.41247	-0.09542	0.803
4873	218846_at	MED23	0.59208	0.75419	0.78315	-0.19107	0.921

4874	218848_at	THOC6	0.51956	0.45649	0.39954	0.12002	0.144
4875	218850_s_at	LIMD1	0.61092	0.57242	0.47122	0.1397	0.131
4876	218852_at	PPP2R3C	0.22456	0.21209	0.24215	-0.01759	0.558
4877	218853_s_at	MOSPD1	0.70626	0.54466	0.33772	0.36854	0.002
4878	218854_at	DSE	0.29681	0.49418	0.3117	-0.01489	0.546
4879	218859_s_at	ESF1	0.71166	0.52662	0.64057	0.07109	0.259
4880	218860_at	NOC4L	0.63381	0.56213	0.59957	0.03424	0.37
4881	218866_s_at	POLR3K	0.75676	0.77385	0.73707	0.01969	0.424
4882	218867_s_at	C12orf49	0.46799	0.2422	0.21379	0.2542	0.03
4883	218868_at	ACTR3B	0.73294	0.78371	0.62055	0.11239	0.234
4884	218870_at	ARHGAP15	0.17238	0.19562	0.24485	-0.07247	0.726
4885	218872_at	TESC	0.20378	0.47787	0.54506	-0.34128	0.995
4886	218873_at	GON4L	0.61013	0.35966	0.34011	0.27002	0.021
4887	218875_s_at	FBXO5	0.83387	0.71685	0.78511	0.04876	0.258
4888	218877_s_at	TRMT11	0.6556	0.51517	0.66369	-0.00809	0.533
4889	218878_s_at	SIRT1	0.62804	0.52842	0.56525	0.06279	0.259
4890	218879_s_at	MTHFSD	0.066912	0.22492	0.10936	-0.042448	0.589
4891	218882_s_at	WDR3	0.62359	0.72143	0.60954	0.01405	0.478
4892	218883_s_at	MLF1IP	0.30309	0.37938	0.39536	-0.09227	0.775
4893	218884_s_at	GUF1	0.60911	0.5971	0.60487	0.00424	0.471
4894	218886_at	PAK1IP1	0.45618	0.46965	0.48555	-0.02937	0.605
4895	218887_at	MRPL2	0.66857	0.69867	0.6991	-0.03053	0.646
4896	218888_s_at	NETO2	0.59006	0.73217	0.65468	-0.06462	0.776
4897	218889_at	NOC3L	0.84751	0.75249	0.78564	0.06187	0.208
4898	218893_at	ISOC2	0.59954	0.39925	0.44072	0.15882	0.596
4899	218894_s_at	MAGOHB	0.74827	0.72591	0.73412	0.01415	0.434
4900	218895_at	GPATCH3	0.36187	0.23055	0.083655	0.278215	0.043
4901	218896_s_at	C17orf85	0.26899	0.25992	0.42597	-0.15698	0.884
4902	218897_at	TMEM177	0.36548	0.34094	0.53774	-0.17226	0.855
4903	218898_at	FAM57A	0.52094	0.50418	0.20884	0.3121	0.009
4904	218902_at	NOTCH1	0.49924	0.64297	0.50606	-0.00682	0.518
4905	218903_s_at	OBFC2B	0.36156	0.41766	0.39766	-0.0361	0.586
4906	218904_s_at	C9orf40	0.39678	0.57154	0.52662	-0.12984	0.803
4907	218905_at	INTS8	0.36924	0.37634	0.32617	0.04307	0.415
4908	218907_s_at	LRRC61	0.19779	0.090039	0.25563	-0.05784	0.661
4909	218909_at	RPS6KC1	0.58118	0.55439	0.58407	-0.00289	0.505
4910	218911_at	YEATS4	0.65477	0.64152	0.51738	0.13739	0.127
4911	218912_at	GCC1	0.55639	0.50437	0.50613	0.05026	0.297
4912	218913_s_at	GMIP	0.44583	0.57408	0.45688	-0.01105	0.538
4913	218916_at	ZNF768	0.063809	0.19319	0.18795	-0.124141	0.786
4914	218919_at	ZFAND1	0.60388	0.59416	0.63672	-0.03284	0.608
4915	218920_at	FLJ10404	0.51957	0.59552	0.59621	-0.07664	0.748
4916	218924_s_at	CTBS	0.691	0.71907	0.69288	-0.00188	0.501
4917	218926_at	MYNN	0.42771	0.4573	0.28926	0.13845	0.117
4918	218927_s_at	CHST12	0.48796	0.68671	0.55889	-0.07093	0.71
4919	218929_at	CDKN2AIP	0.65772	0.71335	0.61046	0.04726	0.288
4920	218930_s_at	TMEM106B	0.69053	0.67472	0.74174	-0.05121	0.767
4921	218932_at	ZNHIT6	0.55108	0.62529	0.58832	-0.03724	0.651
4922	218936_s_at	CCDC59	0.8288	0.80163	0.82965	-0.00085	0.532
4923	218937_at	ZNF434	0.62976	0.34278	0.29839	0.33137	0.01
4924	218938_at	FBXL15	0.15396	0.31107	0.24355	-0.08959	0.698
4925	218940_at	C14orf138	0.70059	0.547	0.63444	0.06615	0.284
4926	218945_at	C16orf68	0.16019	0.21125	0.20637	-0.04618	0.654

4927	218946_at	NFU1	0.73658	0.65953	0.69677	0.03981	0.328
4928	218947_s_at	MTPAP	0.64242	0.67687	0.5759	0.06652	0.267
4929	218949_s_at	QRSL1	0.31275	0.13465	0.38125	-0.0685	0.657
4930	218951_s_at	PLCXD1	0.35906	0.32748	0.42195	-0.06289	0.68
4931	218956_s_at	PTCD1	0.35448	0.47091	0.51615	-0.16167	0.879
4932	218957_s_at	PAAF1	0.41392	0.34702	0.36701	0.04691	0.353
4933	218961_s_at	PNKP	0.40176	0.39657	0.51338	-0.11162	0.842
4934	218962_s_at	TMEM168	0.63779	0.61973	0.53292	0.10487	0.153
4935	218966_at	MYO5C	0.49745	0.4883	0.40577	0.09168	0.235
4936	218967_s_at	PTER	0.58462	0.68764	0.65078	-0.06616	0.733
4937	218968_s_at	ZFP64	0.35864	0.44205	0.29798	0.06066	0.311
4938	218969_at	Magmas	0.52118	0.51924	0.59789	-0.07671	0.756
4939	218970_s_at	CUTC	0.40205	0.64415	0.53559	-0.13354	0.848
4940	218971_s_at	WDR91	0.61915	0.55027	0.43737	0.18178	0.084
4941	218972_at	TTC17	0.34839	0.53595	0.51418	-0.16579	0.878
4942	218973_at	EFTUD1	0.64444	0.4885	0.54026	0.10418	0.163
4943	218974_at	SOBP	0.38442	0.39266	0.33399	0.05043	0.356
4944	218977_s_at	TRNAU1AP	0.65037	0.39359	0.54441	0.10596	0.15
4945	218979_at	RMI1	0.53198	0.5794	0.60912	-0.07714	0.783
4946	218981_at	ACN9	0.60413	0.69093	0.60905	-0.00492	0.523
4947	218982_s_at	MRPS17	0.82645	0.812	0.81639	0.01006	0.44
4948	218983_at	C1RL	0.33938	0.54519	0.62397	-0.28459	0.993
4949	218984_at	PUS7	0.81536	0.83472	0.76999	0.04537	0.257
4950	218986_s_at	DDX60	0.58248	0.72635	0.72623	-0.14375	0.865
4951	218987_at	ATF7IP	0.54343	0.47256	0.57832	-0.03489	0.616
4952	218988_at	SLC35E3	0.49874	0.50418	0.27289	0.22585	0.028
4953	218992_at	C9orf46	0.57808	0.53181	0.49485	0.08323	0.23
4954	218993_at	RNMTL1	0.69687	0.59593	0.65461	0.04226	0.345
4955	218996_at	TFPT	0.56317	0.57792	0.59729	-0.03412	0.625
4956	218997_at	POLR1E	0.37867	0.47389	0.31189	0.06678	0.29
4957	218998_at	C9orf6	0.2338	0.23969	0.18514	0.04866	0.388
4958	218999_at	TMEM140	0.72635	0.63277	0.67185	0.0545	0.31
4959	219001_s_at	WDR32	0.65799	0.40083	0.59686	0.06113	0.293
4960	219002_at	FASTKD1	0.38441	0.32576	0.30915	0.07526	0.305
4961	219003_s_at	MANEA	0.73391	0.69304	0.74776	-0.01385	0.642
4962	219004_s_at	C21orf45	0.74409	0.64629	0.76292	-0.01883	0.597
4963	219006_at	NDUFAF4	0.83557	0.83868	0.76097	0.0746	0.261
4964	219007_at	NUP43	0.46139	0.30752	0.16419	0.2972	0.023
4965	219012_s_at	C11orf30	0.20458	0.28644	0.14903	0.05555	0.353
4966	219013_at	GALNT11	0.090397	0.18326	0.34784	-0.257443	0.959
4967	219014_at	PLAC8	0.64052	0.54113	0.47681	0.16371	0.066
4968	219016_at	FASTKD5	0.56334	0.54906	0.50864	0.0547	0.331
4969	219021_at	RNF121	0.40173	0.34023	0.069984	0.331746	0.012
4970	219022_at	C12orf43	0.36708	0.40243	0.4011	-0.03402	0.602
4971	219023_at	C4orf16	0.92081	0.87253	0.83791	0.0829	0.067
4972	219025_at	CD248	0.45217	0.4471	0.29486	0.15731	0.172
4973	219027_s_at	MYO9A	0.31046	0.26421	0.36685	-0.05639	0.656
4974	219029_at	C5orf28	0.63182	0.49019	0.53067	0.10115	0.161
4975	219030_at	TPRKB	0.8595	0.8695	0.84016	0.01934	0.404
4976	219031_s_at	NIP7	0.80079	0.62037	0.5943	0.20649	<0.001
4977	219033_at	PARP8	0.45196	0.58733	0.51439	-0.06243	0.781
4978	219034_at	PARP16	0.42645	0.22588	0.25826	0.16819	0.15
4979	219035_s_at	RNF34	0.63538	0.26889	0.48249	0.15289	0.132

4980	219038_at	MORC4	0.23716	0.38583	0.2896	-0.05244	0.6
4981	219041_s_at	REPIN1	0.58537	0.36453	0.60639	-0.02102	0.589
4982	219043_s_at	LOC285359	0.64457	0.76676	0.78235	-0.13778	0.895
4983	219045_at	RHOF	0.24357	0.24833	0.25307	-0.0095	0.507
4984	219052_at	HPS6	0.18378	0.31797	0.31764	-0.13386	0.804
4985	219053_s_at	VPS37C	0.52351	0.53496	0.47894	0.04457	0.376
4986	219055_at	SRBD1	0.42198	0.33465	0.38902	0.03296	0.386
4987	219060_at	WDYHV1	0.39607	0.46487	0.51184	-0.11577	0.812
4988	219061_s_at	LAGE3	0.64559	0.61237	0.61323	0.03236	0.403
4989	219062_s_at	ZCCHC2	0.35417	0.23115	0.39667	-0.0425	0.609
4990	219063_at	C1orf35	0.29583	0.1457	0.43833	-0.1425	0.819
4991	219065_s_at	MEMO1	0.8128	0.81771	0.7726	0.0402	0.181
4992	219066_at	PPCDC	0.38048	0.55772	0.4566	-0.07612	0.681
4993	219067_s_at	NSMCE4A	0.72309	0.71689	0.53128	0.19181	0.012
4994	219069_at	ANKRD49	0.36169	0.30906	0.3969	-0.03521	0.619
4995	219074_at	TMEM184C	0.50215	0.49424	0.33868	0.16347	0.094
4996	219076_s_at	PXMP2	0.66845	0.70161	0.72275	-0.0543	0.698
4997	219077_s_at	WWOX	0.056645	0.13016	0.39151	-0.334865	0.978
4998	219078_at	GPATCH2	0.28262	0.060386	0.093986	0.188634	0.116
4999	219079_at	CYB5R4	0.35546	0.43834	0.54993	-0.19447	0.931
5000	219080_s_at	CTPS2	0.25685	0.47267	0.47574	-0.21889	0.954
5001	219086_at	ZNF839	0.10083	0.1011	0.25628	-0.15545	0.873
5002	219089_s_at	ZNF576	0.14716	0.010316	0.15603	-0.00887	0.554
5003	219092_s_at	IPPK	0.16158	0.25685	0.30323	-0.14165	0.774
5004	219098_at	MYBBP1A	0.55361	0.60364	0.59946	-0.04585	0.642
5005	219099_at	C12orf5	0.3049	0.48528	0.30565	-0.00075	0.53
5006	219100_at	OBFC1	0.5708	0.51991	0.44384	0.12696	0.126
5007	219104_at	RNF141	0.079496	0.061801	0.11345	-0.033954	0.579
5008	219109_at	SPAG16	0.48594	0.54564	0.38138	0.10456	0.318
5009	219110_at	GAR1	0.80124	0.83085	0.8049	-0.00366	0.521
5010	219111_s_at	DDX54	0.50892	0.4297	0.24475	0.26417	0.038
5011	219112_at	RAPGEF6	0.59961	0.54862	0.76897	-0.16936	0.931
5012	219116_s_at	DCUN1D2	0.27452	0.28443	0.28322	-0.0087	0.53
5013	219117_s_at	FKBP11	0.59172	0.76511	0.71002	-0.1183	0.895
5014	219119_at	LSM8	0.43029	0.56245	0.54117	-0.11088	0.753
5015	219120_at	C2orf44	0.35552	0.40236	0.45311	-0.09759	0.696
5016	219122_s_at	THG1L	0.22914	0.33784	0.23529	-0.00615	0.509
5017	219123_at	ZNF232	0.088747	0.015676	0.174	-0.085253	0.76
5018	219124_at	C8orf41	0.1729	0.19535	0.29854	-0.12564	0.79
5019	219125_s_at	RAG1AP1	0.50107	0.65005	0.62611	-0.12504	0.776
5020	219126_at	PHF10	0.32435	0.35132	0.50798	-0.18363	0.909
5021	219128_at	C2orf42	0.45063	0.28475	0.31399	0.13664	0.12
5022	219129_s_at	SAP30L	0.54715	0.41224	0.53298	0.01417	0.466
5023	219130_at	CCDC76	0.76019	0.66649	0.49299	0.2672	0.003
5024	219131_at	UBIAD1	0.70572	0.45746	0.25755	0.44817	0.001
5025	219133_at	OXSM	0.011094	0.13025	0.047122	-0.036028	0.593
5026	219137_s_at	MFF	0.58213	0.40012	0.41527	0.16686	0.085
5027	219142_at	RASL11B	0.1148	0.14323	0.26579	-0.15099	0.743
5028	219143_s_at	RPP25	0.16064	0.10929	0.24067	-0.08003	0.749
5029	219146_at	C17orf42	0.17945	0.22638	0.20071	-0.02126	0.541
5030	219147_s_at	C9orf95	0.27522	0.50429	0.36894	-0.09372	0.741
5031	219148_at	PBK	0.68609	0.7409	0.63754	0.04855	0.316
5032	219155_at	PITPNC1	0.29894	0.52032	0.52387	-0.22493	0.954

5033	219156_at	SYNJ2BP	0.38501	0.3492	0.2876	0.09741	0.23
5034	219157_at	KLHL2	0.5727	0.67969	0.62666	-0.05396	0.667
5035	219158_s_at	NARG1	0.6761	0.65861	0.62506	0.05104	0.34
5036	219159_s_at	SLAMF7	0.58286	0.57378	0.63205	-0.04919	0.686
5037	219162_s_at	MRPL11	0.86608	0.8762	0.88791	-0.02183	0.673
5038	219163_at	ZNF562	0.53773	0.30543	0.49116	0.04657	0.368
5039	219164_s_at	ATG2B	0.5336	0.54716	0.54568	-0.01208	0.54
5040	219165_at	PDLIM2	0.22682	0.39646	0.30386	-0.07704	0.673
5041	219166_at	C14orf104	0.5094	0.64208	0.54949	-0.04009	0.652
5042	219169_s_at	TFB1M	0.20987	0.29539	0.13962	0.07025	0.31
5043	219174_at	IFT74	0.14435	0.27058	0.2573	-0.11295	0.746
5044	219175_s_at	SLC41A3	0.40511	0.47805	0.41738	-0.01227	0.571
5045	219176_at	C2orf47	0.75263	0.69372	0.54836	0.20427	0.015
5046	219177_at	BXDC2	0.65762	0.54963	0.46983	0.18779	0.04
5047	219178_at	QTRTD1	0.80863	0.73172	0.77823	0.0304	0.313
5048	219180_s_at	PEX26	0.38741	0.41646	0.34832	0.03909	0.418
5049	219189_at	FBXL6	0.39055	0.45219	0.22224	0.16831	0.137
5050	219192_at	UBAP2	0.24443	0.19834	0.29618	-0.05175	0.658
5051	219193_at	WDR70	0.22598	0.31024	0.387	-0.16102	0.859
5052	219198_at	GTF3C4	0.41372	0.47818	0.4494	-0.03568	0.603
5053	219200_at	FASTKD3	0.6031	0.26109	0.50906	0.09404	0.216
5054	219201_s_at	TWSG1	0.48422	0.55834	0.62427	-0.14005	0.851
5055	219203_at	FAM158A	0.67382	0.49492	0.60615	0.06767	0.317
5056	219205_at	SRR	0.015488	0.26737	0.1573	-0.141812	0.862
5057	219207_at	EDC3	0.11576	0.15484	0.39605	-0.28029	0.956
5058	219210_s_at	RAB8B	0.4306	0.4737	0.43867	-0.00807	0.556
5059	219211_at	USP18	0.43925	0.47353	0.44761	-0.00836	0.559
5060	219212_at	HSPA14	0.64734	0.5658	0.60477	0.04257	0.393
5061	219214_s_at	NT5C	0.6037	0.44026	0.56536	0.03834	0.39
5062	219215_s_at	SLC39A4	0.2729	0.39646	0.3582	-0.0853	0.655
5063	219216_at	ETAA1	0.24951	0.45325	0.41662	-0.16711	0.906
5064	219217_at	NARS2	0.60156	0.47122	0.60195	-0.00039	0.479
5065	219219_at	TMEM160	0.7638	0.77885	0.88149	-0.11769	0.93
5066	219221_at	ZBTB38	0.4893	0.69447	0.61822	-0.12892	0.875
5067	219228_at	ZNF331	0.125	0.11652	0.11575	0.00925	0.475
5068	219231_at	TGS1	0.46056	0.73558	0.63451	-0.17395	0.883
5069	219232_s_at	EGLN3	0.33159	0.44664	0.52779	-0.1962	0.926
5070	219235_s_at	PHACTR4	0.66804	0.60623	0.62405	0.04399	0.354
5071	219237_s_at	DNAJB14	0.67432	0.77135	0.80611	-0.13179	0.917
5072	219238_at	PIGV	0.46009	0.48441	0.26928	0.19081	0.039
5073	219239_s_at	ZNF654	0.64751	0.42468	0.42856	0.21895	0.04
5074	219240_s_at	C10orf88	0.13394	0.18966	0.22756	-0.09362	0.742
5075	219242_at	CEP63	0.47391	0.3411	0.21221	0.2617	0.05
5076	219244_s_at	MRPL46	0.7792	0.77356	0.78533	-0.00613	0.553
5077	219248_at	THUMPD2	0.47546	0.12768	0.15827	0.31719	0.01
5078	219252_s_at	GEMIN8	0.37164	0.42875	0.28931	0.08233	0.273
5079	219253_at	TMEM185B	0.20465	0.1557	0.11289	0.09176	0.232
5080	219256_s_at	SH3TC1	0.34324	0.52601	0.54889	-0.20565	0.955
5081	219258_at	TIPIN	0.85782	0.75363	0.7753	0.08252	0.049
5082	219259_at	SEMA4A	0.29605	0.43356	0.30188	-0.00583	0.551
5083	219260_s_at	C17orf81	0.29485	0.42605	0.32513	-0.03028	0.585
5084	219262_at	SUV39H2	0.47451	0.60531	0.6359	-0.16139	0.955
5085	219267_at	GLTP	0.37186	0.48159	0.40561	-0.03375	0.619

5086	219269_at	HMBOX1	0.02403	0.35372	0.17423	-0.1502	0.763
5087	219274_at	TSPAN12	0.61314	0.65213	0.60362	0.00952	0.484
5088	219275_at	PDCD5	0.82545	0.85952	0.86247	-0.03702	0.751
5089	219279_at	DOCK10	0.66217	0.61237	0.64863	0.01354	0.439
5090	219280_at	BRWD1	0.23176	0.067139	0.23753	-0.00577	0.51
5091	219281_at	MSRA	0.18051	0.41792	0.29073	-0.11022	0.755
5092	219282_s_at	TRPV2	0.77702	0.78632	0.81966	-0.04264	0.802
5093	219283_at	C1GALT1C1	0.53573	0.66793	0.56585	-0.03012	0.564
5094	219284_at	HSPBAP1	0.46044	0.42057	0.16118	0.29926	0.014
5095	219286_s_at	RBM15	0.4485	0.1157	0.24265	0.20585	0.091
5096	219287_at	KCNMB4	0.10496	0.16923	0.15303	-0.04807	0.633
5097	219288_at	C3orf14	0.21063	0.23634	0.23153	-0.0209	0.557
5098	219289_at	HEATR3	0.37206	0.53587	0.52468	-0.15262	0.869
5099	219291_at	DTWD1	0.42405	0.4737	0.57605	-0.152	0.94
5100	219292_at	THAP1	0.62057	0.63532	0.72996	-0.10939	0.868
5101	219293_s_at	OLA1	0.14309	0.48026	0.50011	-0.35702	0.978
5102	219294_at	CENPQ	0.33422	0.444	0.50634	-0.17212	0.843
5103	219296_at	ZDHHC13	0.46334	0.25385	0.56838	-0.10504	0.829
5104	219297_at	WDR44	0.6373	0.72837	0.66561	-0.02831	0.639
5105	219299_at	TRMT12	0.13569	0.11786	0.29189	-0.1562	0.903
5106	219303_at	RNF219	0.53873	0.48182	0.52065	0.01808	0.451
5107	219304_s_at	PDGFD	0.45707	0.62438	0.58547	-0.1284	0.832
5108	219306_at	KIF15	0.71846	0.63033	0.54744	0.17102	0.066
5109	219311_at	CEP76	0.04041	0.12195	0.11667	-0.07626	0.668
5110	219312_s_at	ZBTB10	0.41079	0.5165	0.49147	-0.08068	0.682
5111	219317_at	POLI	0.44792	0.36887	0.30447	0.14345	0.071
5112	219320_at	MYO19	0.089374	0.12253	0.2882	-0.198826	0.813
5113	219321_at	MPP5	0.54106	0.53719	0.53964	0.00142	0.521
5114	219322_s_at	WDR8	0.12611	0.27213	0.1961	-0.06999	0.71
5115	219324_at	NOL12	0.62911	0.75811	0.60768	0.02143	0.391
5116	219325_s_at	ELAC1	0.54707	0.0085256	0.22706	0.32001	0.004
5117	219326_s_at	B3GNT2	0.74203	0.71512	0.72557	0.01646	0.37
5118	219329_s_at	C2orf28	0.66549	0.70789	0.79962	-0.13413	0.925
5119	219330_at	VANGL1	0.12769	0.22099	0.1822	-0.05451	0.679
5120	219334_s_at	OBFC2A	0.29191	0.21296	0.35973	-0.06782	0.725
5121	219335_at	ARMCX5	0.03546	0.076705	0.18549	-0.15003	0.66
5122	219336_s_at	ASCC1	0.30347	0.43702	0.48778	-0.18431	0.943
5123	219338_s_at	LRRC49	0.2132	0.17487	0.12207	0.09113	0.223
5124	219342_at	CASD1	0.52854	0.74375	0.68232	-0.15378	0.917
5125	219343_at	CDC37L1	0.59158	0.55449	0.53457	0.05701	0.314
5126	219345_at	BOLA1	0.58309	0.24526	0.41174	0.17135	0.106
5127	219347_at	NUDT15	0.35983	0.43349	0.42398	-0.06415	0.745
5128	219348_at	USE1	0.6938	0.58855	0.68821	0.00559	0.473
5129	219350_s_at	DIABLO	0.32219	0.45467	0.47851	-0.15632	0.782
5130	219351_at	TRAPPC2	0.31733	0.5527	0.52646	-0.20913	0.836
5131	219352_at	HERC6	0.72883	0.65085	0.68255	0.04628	0.255
5132	219353_at	NHLRC2	0.66304	0.46084	0.65755	0.00549	0.5
5133	219357_at	GTPBP1	0.56675	0.62373	0.6111	-0.04435	0.649
5134	219361_s_at	AEN	0.5942	0.33738	0.46741	0.12679	0.177
5135	219363_s_at	MTERFD1	0.80685	0.45026	0.66414	0.14271	0.16
5136	219366_at	AVEN	0.44533	0.45991	0.518	-0.07267	0.682
5137	219368_at	NAP1L2	0.011197	0.10947	0.11609	-0.104893	0.75
5138	219371_s_at	KLF2	0.68764	0.51567	0.59635	0.09129	0.24

5139	219372_at	IFT81	0.57121	0.34088	0.49794	0.07327	0.274
5140	219374_s_at	ALG9	0.38551	0.39139	0.48428	-0.09877	0.778
5141	219375_at	CEPT1	0.39437	0.5006	0.40458	-0.01021	0.534
5142	219376_at	ZNF322B	0.18408	0.40332	0.38916	-0.20508	0.931
5143	219378_at	NARG1L	0.65865	0.70282	0.75741	-0.09876	0.886
5144	219382_at	SERTAD3	0.30665	0.13584	0.16052	0.14613	0.164
5145	219384_s_at	ADAT1	0.74564	0.67723	0.71161	0.03403	0.336
5146	219387_at	CCDC88A	0.18383	0.46611	0.48206	-0.29823	0.989
5147	219390_at	FKBP14	0.019889	0.22319	0.33578	-0.315891	0.758
5148	219394_at	PGS1	0.30215	0.25532	0.42269	-0.12054	0.802
5149	219397_at	COQ10B	0.49169	0.31872	0.30499	0.1867	0.149
5150	219405_at	TRIM68	0.30521	0.4232	0.35702	-0.05181	0.649
5151	219406_at	C1orf50	0.49627	0.51653	0.49536	0.00091	0.517
5152	219409_at	SNIP1	0.40735	0.32937	0.25969	0.14766	0.136
5153	219410_at	TMEM45A	0.74033	0.60017	0.39851	0.34182	<0.001
5154	219412_at	RAB38	0.41963	0.58486	0.55093	-0.1313	0.863
5155	219421_at	TTC33	0.61498	0.6642	0.74492	-0.12994	0.942
5156	219424_at	EBI3	0.4983	0.60555	0.57502	-0.07672	0.726
5157	219426_at	EIF2C3	0.32957	0.18451	0.3593	-0.02973	0.606
5158	219428_s_at	PXMP4	0.53337	0.56079	0.59587	-0.0625	0.676
5159	219429_at	FA2H	0.34384	0.38386	0.29052	0.05332	0.35
5160	219431_at	ARHGAP10	0.38929	0.41618	0.28573	0.10356	0.22
5161	219433_at	BCOR	0.5524	0.75133	0.6472	-0.0948	0.787
5162	219435_at	C17orf68	0.55275	0.56828	0.34688	0.20587	0.025
5163	219437_s_at	ANKRD11	0.46281	0.33171	0.37607	0.08674	0.267
5164	219439_at	C1GALT1	0.57995	0.66264	0.60806	-0.02811	0.641
5165	219441_s_at	LRRK1	0.3427	0.15872	0.13883	0.20387	0.068
5166	219443_at	TASP1	0.31722	0.24812	0.30618	0.01104	0.487
5167	219444_at	BCORL1	0.19647	0.17645	0.23181	-0.03534	0.597
5168	219446_at	RIC8B	0.48652	0.34246	0.40191	0.08461	0.283
5169	219447_s_at	SLC35C2	0.3078	0.423	0.4083	-0.1005	0.659
5170	219449_s_at	TMEM70	0.6546	0.67804	0.69119	-0.03659	0.637
5171	219458_s_at	NSUN3	0.20909	0.20105	0.14043	0.06866	0.327
5172	219459_at	POLR3B	0.56392	0.57099	0.51114	0.05278	0.332
5173	219462_at	TMEM53	0.28739	0.30744	0.32588	-0.03849	0.617
5174	219467_at	GIN1	0.2105	0.16388	0.16324	0.04726	0.421
5175	219471_at	C13orf18	0.57425	0.6207	0.58059	-0.00634	0.522
5176	219472_at	CENPO	0.43776	0.23523	0.44355	-0.00579	0.548
5177	219473_at	GDAP2	0.11073	0.18159	0.25005	-0.13932	0.775
5178	219479_at	KDELC1	0.066722	0.12113	0.27959	-0.212868	0.917
5179	219481_at	TTC13	0.34363	0.24549	0.40062	-0.05699	0.675
5180	219484_at	HCFC2	0.31816	0.13242	0.29525	0.02291	0.445
5181	219485_s_at	PSMD10	0.69345	0.74757	0.66107	0.03238	0.312
5182	219487_at	BBS10	0.35663	0.47456	0.50338	-0.14675	0.858
5183	219489_s_at	NXN	0.23204	0.22688	0.16258	0.06946	0.321
5184	219490_s_at	DCLRE1B	0.3287	0.39075	0.18972	0.13898	0.208
5185	219492_at	CHIC2	0.75432	0.45018	0.51242	0.2419	0.012
5186	219493_at	SHCBP1	0.83297	0.7717	0.87641	-0.04344	0.772
5187	219494_at	RAD54B	0.6023	0.47773	0.65323	-0.05093	0.675
5188	219495_s_at	ZNF180	0.42242	0.52277	0.50112	-0.0787	0.726
5189	219496_at	ANKRD57	0.0049265	0.20665	0.13644	-0.1315135	0.827
5190	219497_s_at	BCL11A	0.49851	0.5986	0.64515	-0.14664	0.918
5191	219501_at	ENOX1	0.23509	0.28232	0.20928	0.02581	0.435

5192	219502_at	NEIL3	0.38018	0.42568	0.51158	-0.1314	0.894
5193	219504_s_at	RPAP2	0.051223	0.27275	0.26899	-0.217767	0.77
5194	219506_at	C1orf54	0.15047	0.3536	0.35433	-0.20386	0.917
5195	219507_at	RSRC1	0.25193	0.29791	0.244	0.00793	0.503
5196	219512_at	DSN1	0.23496	0.10353	0.12232	0.11264	0.221
5197	219515_at	PRDM10	0.22228	0.30936	0.45724	-0.23496	0.922
5198	219517_at	ELL3	0.48709	0.65615	0.60029	-0.1132	0.894
5199	219520_s_at	WWC3	0.37097	0.45428	0.43651	-0.06554	0.679
5200	219526_at	C14orf169	0.60814	0.59174	0.62785	-0.01971	0.581
5201	219530_at	PALB2	0.6082	0.6384	0.59469	0.01351	0.456
5202	219531_at	CEP72	0.29902	0.33701	0.1888	0.11022	0.216
5203	219538_at	WDR5B	0.086272	0.15034	0.078561	0.007711	0.508
5204	219539_at	GEMIN6	0.66451	0.52088	0.57832	0.08619	0.238
5205	219540_at	ZNF267	0.6931	0.53743	0.56869	0.12441	0.045
5206	219543_at	PBLD	0.57711	0.43963	0.3759	0.20121	0.033
5207	219544_at	C13orf34	0.60924	0.35593	0.49082	0.11842	0.22
5208	219548_at	ZNF16	0.55318	0.20218	0.33164	0.22154	0.077
5209	219549_s_at	RTN3	0.66448	0.5788	0.53037	0.13411	0.158
5210	219551_at	EAF2	0.4336	0.69209	0.49872	-0.06512	0.623
5211	219557_s_at	NRIP3	0.3185	0.36763	0.31573	0.00277	0.491
5212	219559_at	SLC17A9	0.10709	0.13871	0.15887	-0.05178	0.643
5213	219563_at	C14orf139	0.50927	0.3773	0.56149	-0.05222	0.671
5214	219565_at	CYP20A1	0.18628	0.33698	0.064744	0.121536	0.184
5215	219567_s_at	DEM1	0.00056442	0.12225	0.07308	-0.07251558	0.695
5216	219570_at	KIF16B	0.046637	0.20671	0.22637	-0.179733	0.826
5217	219571_s_at	ZNF12	0.67641	0.5433	0.54379	0.13262	0.147
5218	219575_s_at	COG8	0.46176	0.59742	0.54475	-0.08299	0.665
5219	219577_s_at	ABCA7	0.1841	0.18439	0.15982	0.02428	0.456
5220	219581_at	TSEN2	0.41623	0.22863	0.3495	0.06673	0.327
5221	219582_at	OGFRL1	0.456	0.54878	0.54876	-0.09276	0.732
5222	219583_s_at	SPATA7	0.22485	0.1679	0.12626	0.09859	0.287
5223	219584_at	PLA1A	0.87796	0.86938	0.85047	0.02749	0.306
5224	219588_s_at	NCAPG2	0.69628	0.73205	0.68506	0.01122	0.473
5225	219593_at	SLC15A3	0.40854	0.47285	0.39524	0.0133	0.453
5226	219594_at	NINJ2	0.20703	0.40863	0.32566	-0.11863	0.751
5227	219596_at	THAP10	0.36048	0.25524	0.34895	0.01153	0.506
5228	219598_s_at	RWDD1	0.692	0.74075	0.7485	-0.0565	0.692
5229	219600_s_at	TMEM50B	0.36579	0.53656	0.55389	-0.1881	0.88
5230	219602_s_at	FAM38B	0.23769	0.36966	0.53086	-0.29317	0.981
5231	219603_s_at	ZNF226	0.3055	0.61679	0.70602	-0.40052	0.918
5232	219613_s_at	SIRT6	0.33165	0.60855	0.48879	-0.15714	0.857
5233	219617_at	C2orf34	0.36723	0.20404	0.32874	0.03849	0.384
5234	219618_at	IRAK4	0.5055	0.46304	0.48116	0.02434	0.444
5235	219624_at	BAG4	0.12956	0.23923	0.25449	-0.12493	0.805
5236	219627_at	ZNF767	0.44162	0.2844	0.28541	0.15621	0.082
5237	219628_at	ZMAT3	0.48234	0.66442	0.38612	0.09622	0.285
5238	219629_at	FAM118A	0.13507	0.21904	0.23204	-0.09697	0.75
5239	219633_at	TTPAL	0.47389	0.64536	0.47716	-0.00327	0.51
5240	219634_at	CHST11	0.63907	0.59474	0.6169	0.02217	0.426
5241	219635_at	ZNF606	0.10671	0.041677	0.1383	-0.03159	0.546
5242	219636_s_at	ARMC9	0.18632	0.17391	0.1887	-0.00238	0.603
5243	219644_at	CCDC41	0.6059	0.39607	0.55636	0.04954	0.228
5244	219646_at	DEF8	0.34794	0.51077	0.47584	-0.1279	0.869

5245	219648_at	MREG	0.6554	0.69674	0.54909	0.10631	0.182
5246	219649_at	ALG6	0.3323	0.39915	0.40262	-0.07032	0.658
5247	219650_at	ERCC6L	0.25415	0.45321	0.49155	-0.2374	0.936
5248	219653_at	LSM14B	0.16944	0.0025379	0.11672	0.05272	0.35
5249	219662_at	C2orf49	0.55469	0.24477	0.59102	-0.03633	0.633
5250	219667_s_at	BANK1	0.18806	0.38534	0.2945	-0.10644	0.763
5251	219673_at	MCM9	0.63006	0.60957	0.61944	0.01062	0.473
5252	219675_s_at	UXS1	0.29393	0.24595	0.24638	0.04755	0.401
5253	219676_at	ZSCAN16	0.76141	0.57056	0.72039	0.04102	0.276
5254	219680_at	NLRX1	0.5237	0.3991	0.5328	-0.0091	0.552
5255	219681_s_at	RAB11FIP1	0.39281	0.40469	0.48195	-0.08914	0.803
5256	219683_at	FZD3	0.44991	0.55015	0.49948	-0.04957	0.659
5257	219688_at	BBS7	0.50871	0.52749	0.44542	0.06329	0.261
5258	219690_at	TMEM149	0.27439	0.32528	0.46191	-0.18752	0.868
5259	219691_at	SAMD9	0.52062	0.68858	0.59779	-0.07717	0.741
5260	219696_at	DENND1B	0.05013	0.23109	0.24973	-0.1996	0.963
5261	219698_s_at	METTL4	0.39279	0.44451	0.4842	-0.09141	0.791
5262	219703_at	MNS1	0.46751	0.50891	0.51665	-0.04914	0.659
5263	219706_at	C20orf29	0.29482	0.3216	0.31388	-0.01906	0.586
5264	219711_at	ZNF586	0.14075	0.33238	0.40057	-0.25982	0.906
5265	219715_s_at	TDP1	0.45372	0.47978	0.43609	0.01763	0.469
5266	219717_at	C4orf30	0.33498	0.42546	0.55701	-0.22203	0.944
5267	219720_s_at	C14orf118	0.48173	0.24142	0.13176	0.34997	0.04
5268	219724_s_at	KIAA0748	0.042062	0.068872	0.090424	-0.048362	0.617
5269	219731_at	FLJ34077	0.29536	0.33808	0.24844	0.04692	0.399
5270	219740_at	VASH2	0.49738	0.59635	0.49113	0.00625	0.499
5271	219751_at	SETD6	0.43148	0.45215	0.2977	0.13378	0.144
5272	219753_at	STAG3	0.5284	0.46245	0.48529	0.04311	0.37
5273	219754_at	RBM41	0.38075	0.51335	0.36488	0.01587	0.435
5274	219757_s_at	C14orf101	0.33307	0.39062	0.39401	-0.06094	0.698
5275	219759_at	ERAP2	0.048534	0.22456	0.23379	-0.185256	0.923
5276	219762_s_at	RPL36	0.9421	0.87903	0.92129	0.02081	0.179
5277	219763_at	DENND1A	0.49872	0.62794	0.47115	0.02757	0.412
5278	219765_at	ZNF329	0.19452	0.33484	0.2281	-0.03358	0.52
5279	219767_s_at	CRYZL1	0.33522	0.35233	0.23926	0.09596	0.231
5280	219770_at	GTDC1	0.061165	0.15111	0.23345	-0.172285	0.917
5281	219774_at	CCDC93	0.024612	0.071002	0.16734	-0.142728	0.722
5282	219777_at	GIMAP6	0.49677	0.67754	0.55916	-0.06239	0.708
5283	219785_s_at	FBXO31	0.45759	0.456	0.40732	0.05027	0.35
5284	219787_s_at	ECT2	0.89083	0.86051	0.82137	0.06946	0.159
5285	219793_at	SNX16	0.23469	0.22054	0.31049	-0.0758	0.679
5286	219798_s_at	MEPCE	0.44505	0.49356	0.55339	-0.10834	0.799
5287	219800_s_at	THNSL1	0.24588	0.21186	0.1949	0.05098	0.374
5288	219801_at	ZNF34	0.0032066	0.0061286	0.040299	-0.0370924	0.607
5289	219805_at	CXorf56	0.71522	0.60128	0.60672	0.1085	0.196
5290	219806_s_at	C11orf75	0.27876	0.40198	0.38798	-0.10922	0.762
5291	219809_at	WDR55	0.39814	0.20807	0.35826	0.03988	0.395
5292	219812_at	PVRIG	0.43293	0.55606	0.49172	-0.05879	0.715
5293	219816_s_at	RBM23	0.77499	0.52211	0.62096	0.15403	0.022
5294	219818_s_at	GPATCH1	0.45164	0.38551	0.20861	0.24303	0.029
5295	219819_s_at	MRPS28	0.85113	0.72235	0.80782	0.04331	0.272
5296	219821_s_at	GFOD1	0.39165	0.43449	0.3922	-0.00055	0.513
5297	219822_at	MTRF1	0.4263	0.25761	0.27453	0.15177	0.17

5298	219833_s_at	EFHC1	0.60294	0.49646	0.5045	0.09844	0.21
5299	219834_at	ALS2CR8	0.52087	0.30488	0.49102	0.02985	0.394
5300	219841_at	AICDA	0.27316	0.51028	0.52508	-0.25192	0.944
5301	219843_at	IPP	0.40617	0.40448	0.46759	-0.06142	0.641
5302	219848_s_at	ZNF432	0.067303	0.23391	0.2587	-0.191397	0.861
5303	219849_at	ZNF671	0.19065	0.027364	0.13894	0.05171	0.382
5304	219854_at	ZNF14	0.25713	0.14678	0.077614	0.179516	0.102
5305	219858_s_at	MFSD6	0.59357	0.68651	0.69731	-0.10374	0.903
5306	219860_at	LY6G5C	0.41421	0.37812	0.10778	0.30643	0.032
5307	219861_at	DNAJC17	0.35986	0.34719	0.30226	0.0576	0.37
5308	219862_s_at	NARF	0.76347	0.68764	0.32051	0.44296	<0.001
5309	219863_at	HERC5	0.69366	0.64147	0.59426	0.0994	0.126
5310	219870_at	ATF7IP2	0.38992	0.19516	0.35327	0.03665	0.412
5311	219874_at	SLC12A8	0.55059	0.68392	0.60934	-0.05875	0.772
5312	219878_s_at	KLF13	0.42005	0.39842	0.3802	0.03985	0.38
5313	219885_at	SLFN12	0.092261	0.0023581	0.027388	0.064873	0.193
5314	219889_at	FRAT1	0.15076	0.39537	0.31471	-0.16395	0.838
5315	219891_at	PGPEP1	0.65583	0.56691	0.52869	0.12714	0.087
5316	219892_at	TM6SF1	0.17466	0.36786	0.28707	-0.11241	0.752
5317	219901_at	FGD6	0.34343	0.30208	0.41983	-0.0764	0.639
5318	219904_at	ZSCAN5A	0.12966	0.22749	0.1331	-0.00344	0.513
5319	219910_at	FICD	0.61481	0.55334	0.39575	0.21906	0.083
5320	219913_s_at	CRNKL1	0.66321	0.69137	0.59753	0.06568	0.235
5321	219915_s_at	SLC16A10	0.26744	0.35784	0.46059	-0.19315	0.944
5322	219917_at	ZCCHC4	0.3517	0.29496	0.35739	-0.00569	0.495
5323	219918_s_at	ASPM	0.7584	0.77435	0.67447	0.08393	0.263
5324	219920_s_at	GMPPB	0.24423	0.32331	0.27571	-0.03148	0.602
5325	219923_at	TRIM45	0.1665	0.28205	0.15598	0.01052	0.443
5326	219924_s_at	ZMYM6	0.75858	0.62393	0.68686	0.07172	0.214
5327	219929_s_at	ZFYVE21	0.50893	0.26504	0.33731	0.17162	0.087
5328	219931_s_at	KLHL12	0.37328	0.38478	0.44328	-0.07	0.65
5329	219933_at	GLRX2	0.74802	0.70433	0.71733	0.03069	0.383
5330	219938_s_at	PSTPIP2	0.4534	0.61407	0.48652	-0.03312	0.638
5331	219939_s_at	CSDE1	0.74094	0.73024	0.73752	0.00342	0.504
5332	219940_s_at	PCID2	0.48309	0.51104	0.50837	-0.02528	0.589
5333	219941_at	TMEM19	0.22362	0.28596	0.10343	0.12019	0.266
5334	219951_s_at	C20orf12	0.011462	0.070639	0.037572	-0.02611	0.537
5335	219956_at	GALNT6	0.23939	0.16423	0.28372	-0.04433	0.642
5336	219959_at	MOCOS	8.23E-005	0.10865	0.13338	-0.133297728	0.88
5337	219960_s_at	UCHL5	0.61099	0.60124	0.65064	-0.03965	0.67
5338	219967_at	MRM1	0.54115	0.41924	0.34985	0.1913	0.152
5339	219971_at	IL21R	0.37256	0.4437	0.39753	-0.02497	0.562
5340	219972_s_at	C14orf135	0.65482	0.63045	0.53539	0.11943	0.12
5341	219976_at	HOOK1	0.29254	0.41724	0.34111	-0.04857	0.664
5342	219979_s_at	C11orf73	0.75402	0.71693	0.7859	-0.03188	0.599
5343	219980_at	C4orf29	0.35797	0.26734	0.31114	0.04683	0.37
5344	219982_s_at	SERF1A	0.27665	0.42747	0.44069	-0.16404	0.87
5345	219988_s_at	RNF220	0.53092	0.55493	0.34222	0.1887	0.114
5346	219990_at	E2F8	0.71419	0.58521	0.77092	-0.05673	0.811
5347	219996_at	ASB7	0.27151	0.093492	3.41E-005	0.271475932	0.019
5348	219997_s_at	COPS7B	0.54489	0.36969	0.36343	0.18146	0.119
5349	219999_at	MAN2A2	0.17108	0.24489	0.18717	-0.01609	0.579
5350	220007_at	METTL8	0.71356	0.58051	0.69239	0.02117	0.393

5351	220011_at	C1orf135	0.070783	0.084691	0.032111	0.038672	0.362
5352	220012_at	ERO1LB	0.70674	0.64749	0.6112	0.09554	0.108
5353	220015_at	CASZ1	0.23378	0.29771	0.3304	-0.09662	0.766
5354	220018_at	CBLL1	0.44723	0.49338	0.49959	-0.05236	0.68
5355	220019_s_at	ZNF224	0.65504	0.61417	0.40766	0.24738	0.139
5356	220035_at	NUP210	0.36409	0.21255	0.33939	0.0247	0.417
5357	220036_s_at	LMBR1L	0.40647	0.42784	0.54786	-0.14139	0.888
5358	220046_s_at	CCNL1	0.22788	0.044538	0.22812	-0.00024	0.492
5359	220050_at	C9orf9	0.4293	0.25142	0.44674	-0.01744	0.539
5360	220052_s_at	TINF2	0.0099172	0.086298	0.068183	-0.0582658	0.645
5361	220054_at	IL23A	0.16051	0.38865	0.39284	-0.23233	0.956
5362	220058_at	C17orf39	0.45803	0.23458	0.22722	0.23081	0.084
5363	220059_at	STAP1	0.23772	0.41202	0.37539	-0.13767	0.802
5364	220060_s_at	C12orf48	0.77161	0.74261	0.79819	-0.02658	0.717
5365	220063_at	GSTCD	0.10032	0.40788	0.27091	-0.17059	0.823
5366	220066_at	NOD2	0.5034	0.19958	0.3031	0.2003	0.11
5367	220073_s_at	PLEKHG6	0.10628	0.19332	0.068307	0.037973	0.399
5368	220079_s_at	USP48	0.57171	0.57618	0.58959	-0.01788	0.565
5369	220085_at	HELLS	0.39172	0.6409	0.61269	-0.22097	0.958
5370	220089_at	L2HGDH	0.34678	0.26709	0.34354	0.00324	0.516
5371	220091_at	SLC2A6	0.4081	0.55337	0.34809	0.06001	0.385
5372	220094_s_at	CCDC90A	0.60166	0.76639	0.77916	-0.1775	0.949
5373	220099_s_at	LUC7L2	0.62432	0.65303	0.67168	-0.04736	0.673
5374	220103_s_at	MRPS18C	0.35028	0.27477	0.2078	0.14248	0.385
5375	220104_at	ZC3HAV1	0.26146	0.4843	0.45849	-0.19703	0.913
5376	220118_at	ZBTB32	0.56384	0.57168	0.36112	0.20272	0.001
5377	220121_at	LINS1	0.16544	0.27403	0.19503	-0.02959	0.579
5378	220122_at	MCTP1	0.14938	0.23087	0.24957	-0.10019	0.764
5379	220127_s_at	FBXL12	0.088942	0.17006	0.227	-0.138058	0.79
5380	220132_s_at	CLEC2D	0.4506	0.63625	0.61967	-0.16907	0.92
5381	220145_at	MAP9	0.2881	0.32106	0.19468	0.09342	0.299
5382	220146_at	TLR7	0.19264	0.49987	0.37845	-0.18581	0.866
5383	220147_s_at	FAM60A	0.39221	0.44914	0.55398	-0.16177	0.888
5384	220155_s_at	BRD9	0.59109	0.63836	0.42944	0.16165	0.041
5385	220158_at	LGALS14	0.69824	0.76779	0.6954	0.00284	0.525
5386	220159_at	ABCA11P	0.076977	0.21903	0.31116	-0.234183	0.929
5387	220161_s_at	EPB41L4B	0.47746	0.4149	0.36136	0.1161	0.234
5388	220167_s_at	LOC729355	0.38861	0.39053	0.29991	0.0887	0.332
5389	220169_at	TMEM156	0.22303	0.2352	0.43543	-0.2124	0.926
5390	220172_at	C2orf37	0.61644	0.62076	0.56167	0.05477	0.262
5391	220175_s_at	CBWD1	0.62089	0.66802	0.68085	-0.05996	0.725
5392	220176_at	NUBPL	0.5421	0.61441	0.55418	-0.01208	0.579
5393	220183_s_at	NUDT6	0.11656	0.23533	0.3261	-0.20954	0.931
5394	220189_s_at	MGAT4B	0.40605	0.55196	0.4534	-0.04735	0.664
5395	220195_at	MBD5	0.51235	0.69093	0.52916	-0.01681	0.621
5396	220199_s_at	AIDA	0.70766	0.83788	0.79599	-0.08833	0.862
5397	220200_s_at	SETD8	0.44411	0.45288	0.47701	-0.0329	0.617
5398	220201_at	RC3H2	0.29713	0.35104	0.5525	-0.25537	0.967
5399	220215_at	ZNF669	0.1012	0.19864	0.1322	-0.031	0.558
5400	220230_s_at	CYB5R2	0.074813	0.2985	0.22578	-0.150967	0.854
5401	220235_s_at	C1orf103	0.34165	0.47589	0.46979	-0.12814	0.788
5402	220236_at	PDPR	0.19538	0.23672	0.23922	-0.04384	0.584
5403	220238_s_at	KLHL7	0.40173	0.7159	0.74012	-0.33839	1

5404	220241_at	TMCO3	0.23024	0.20882	0.43797	-0.20773	0.949
5405	220244_at	LOH3CR2A	0.42269	0.31564	0.24509	0.1776	0.129
5406	220250_at	ZNF286A	0.56568	0.53896	0.54479	0.02089	0.446
5407	220251_at	C1orf107	0.28962	0.20171	0.17715	0.11247	0.281
5408	220255_at	FANCE	0.41904	0.1897	0.26433	0.15471	0.103
5409	220261_s_at	ZDHHC4	0.32027	0.54647	0.38943	-0.06916	0.663
5410	220285_at	FAM108B1	0.68984	0.71806	0.72518	-0.03534	0.73
5411	220287_at	ADAMTS9	0.18957	0.387	0.3661	-0.17653	0.894
5412	220288_at	MYO15A	0.31369	0.17913	0.28557	0.02812	0.455
5413	220305_at	MAVS	0.39834	0.29551	0.28431	0.11403	0.167
5414	220319_s_at	MYLIP	0.044463	0.1055	0.068024	-0.023561	0.584
5415	220320_at	DOK3	0.50844	0.44058	0.41258	0.09586	0.224
5416	220329_s_at	RMND1	0.38186	0.30446	0.49957	-0.11771	0.761
5417	220330_s_at	SAMSN1	0.35863	0.54879	0.6195	-0.26087	0.965
5418	220346_at	MTHFD2L	0.5907	0.41322	0.46332	0.12738	0.173
5419	220355_s_at	PBRM1	0.69784	0.52625	0.48785	0.20999	0.039
5420	220358_at	BATF3	0.16988	0.17626	0.16437	0.00551	0.504
5421	220367_s_at	SAP130	0.33222	0.26715	0.22304	0.10918	0.185
5422	220368_s_at	SMEK1	0.75929	0.81851	0.75799	0.0013	0.498
5423	220370_s_at	USP36	0.025279	0.046747	0.055743	-0.030464	0.588
5424	220371_s_at	SLC12A9	0.20729	0.29155	0.30229	-0.095	0.713
5425	220375_s_at	H2AFY	0.098569	0.12143	0.21716	-0.118591	0.636
5426	220386_s_at	EML4	0.47301	0.70594	0.75943	-0.28642	0.987
5427	220387_s_at	HHLA3	0.7309	0.64814	0.6534	0.0775	0.227
5428	220390_at	AGBL2	0.019344	0.0090227	0.075024	-0.05568	0.643
5429	220391_at	ZBTB3	0.36557	0.24223	0.28144	0.08413	0.272
5430	220399_at	NCRNA00115	0.26213	0.22217	0.21856	0.04357	0.402
5431	220417_s_at	THAP4	0.34317	0.44161	0.42879	-0.08562	0.752
5432	220419_s_at	USP25	0.44243	0.55574	0.61167	-0.16924	0.977
5433	220446_s_at	CHST4	0.022994	0.17249	0.168	-0.145006	0.824
5434	220459_at	MCM3APAS	0.019399	0.1385	0.14963	-0.130231	0.785
5435	220466_at	CCDC15	0.61231	0.16529	0.5377	0.07461	0.268
5436	220467_at	FLJ21272	0.12574	0.36766	0.28457	-0.15883	0.782
5437	220476_s_at	C1orf183	0.34493	0.23377	0.13313	0.2118	0.011
5438	220477_s_at	C20orf30	0.62142	0.63498	0.46934	0.15208	0.068
5439	220488_s_at	BCAS3	0.45173	0.56166	0.47393	-0.0222	0.583
5440	220494_s_at	C14orf43	0.15753	0.33283	0.3346	-0.17707	0.923
5441	220495_s_at	TXNDC15	0.70052	0.76603	0.61131	0.08921	0.109
5442	220500_s_at	RABL2A	0.71766	0.56402	0.48973	0.22793	0.03
5443	220525_s_at	AUP1	0.72671	0.74339	0.74541	-0.0187	0.588
5444	220547_s_at	FAM35A	0.14508	0.33506	0.33744	-0.19236	0.87
5445	220550_at	FBXO4	0.0049756	0.044056	0.039161	-0.0341854	0.572
5446	220553_s_at	PRPF39	0.70309	0.68274	0.6425	0.06059	0.256
5447	220565_at	CCR10	0.65911	0.7576	0.68918	-0.03007	0.615
5448	220566_at	PIK3R5	0.39326	0.31048	0.31561	0.07765	0.325
5449	220572_at	DKFZp547G183	0.12666	0.15259	0.12331	0.00335	0.475
5450	220577_at	GVIN1	0.38047	0.50566	0.47374	-0.09327	0.795
5451	220580_at	BICC1	0.0070077	0.073084	0.060511	-0.0535033	0.549
5452	220586_at	CHD9	0.37558	0.35046	0.42845	-0.05287	0.679
5453	220587_s_at	GBL	0.45286	0.53472	0.53297	-0.08011	0.74
5454	220588_at	BCAS4	0.2355	0.31548	0.42134	-0.18584	0.865
5455	220590_at	ITFG2	0.33345	0.30778	0.28552	0.04793	0.433
5456	220597_s_at	ARL6IP4	0.74148	0.63737	0.68126	0.06022	0.2

5457	220603_s_at	MCTP2	0.66379	0.70245	0.60643	0.05736	0.267
5458	220605_s_at	SIRT2	0.50372	0.50344	0.42634	0.07738	0.257
5459	220608_s_at	ZNF770	0.81392	0.78978	0.78238	0.03154	0.34
5460	220623_s_at	TSGA10	0.099527	0.1556	0.14287	-0.043343	0.6
5461	220643_s_at	FAIM	0.5446	0.57923	0.64941	-0.10481	0.82
5462	220647_s_at	CHCHD8	0.89294	0.88341	0.85546	0.03748	0.32
5463	220651_s_at	MCM10	0.71816	0.81783	0.7245	-0.00634	0.549
5464	220658_s_at	ARNTL2	0.68189	0.50627	0.53805	0.14384	0.064
5465	220661_s_at	ZNF692	0.60039	0.46699	0.50345	0.09694	0.216
5466	220682_s_at	KLHL5	0.67825	0.68682	0.6922	-0.01395	0.596
5467	220688_s_at	MRTO4	0.80544	0.81906	0.80657	-0.00113	0.524
5468	220690_s_at	DHRS7B	0.21867	0.16959	0.33677	-0.1181	0.822
5469	220703_at	C10orf110	0.20658	0.28907	0.33005	-0.12347	0.793
5470	220721_at	ZNF614	0.30467	0.051137	0.30008	0.00459	0.488
5471	220731_s_at	NECAP2	0.50254	0.62957	0.52053	-0.01799	0.559
5472	220735_s_at	SENP7	0.55928	0.4215	0.50445	0.05483	0.289
5473	220739_s_at	CNNM3	0.34599	0.52577	0.39047	-0.04448	0.63
5474	220740_s_at	SLC12A6	0.55751	0.4694	0.38363	0.17388	0.063
5475	220741_s_at	PPA2	0.55704	0.67941	0.6441	-0.08706	0.76
5476	220746_s_at	UIMC1	0.38714	0.26312	0.43222	-0.04508	0.609
5477	220750_s_at	LEPRE1	0.42479	0.39837	0.3198	0.10499	0.269
5478	220753_s_at	CRYL1	0.4396	0.41806	0.40155	0.03805	0.411
5479	220755_s_at	C6orf48	0.78795	0.81986	0.76725	0.0207	0.441
5480	220757_s_at	UBXN6	0.16817	0.30149	0.19292	-0.02475	0.583
5481	220761_s_at	TAOK3	0.62237	0.54576	0.61462	0.00775	0.491
5482	220768_s_at	CSNK1G3	0.80475	0.77223	0.80694	-0.00219	0.577
5483	220770_s_at	C5orf54	0.21983	0.24899	0.44083	-0.221	0.927
5484	220773_s_at	GPHN	0.76872	0.75888	0.73471	0.03401	0.348
5485	220775_s_at	UEVLD	0.59734	0.65041	0.74697	-0.14963	0.863
5486	220776_at	KCNJ14	0.26449	0.12927	0.14918	0.11531	0.235
5487	220788_s_at	IRF9	0.61419	0.62135	0.56866	0.04553	0.341
5488	220789_s_at	TBRG4	0.77898	0.7682	0.70778	0.0712	0.173
5489	220797_at	METT10D	0.18745	0.1714	0.18837	-0.00092	0.513
5490	220800_s_at	TMOD3	0.79526	0.8173	0.81134	-0.01608	0.675
5491	220826_at	TCP10L	0.39951	0.34839	0.39447	0.00504	0.463
5492	220840_s_at	C1orf112	0.55393	0.3459	0.42234	0.13159	0.124
5493	220864_s_at	NDUFA13	0.87307	0.86365	0.92413	-0.05106	0.782
5494	220865_s_at	PDSS1	0.69039	0.73922	0.57344	0.11695	0.195
5495	220885_s_at	CENPJ	0.5494	0.31244	0.51021	0.03919	0.368
5496	220890_s_at	DDX47	0.44129	0.53808	0.44852	-0.00723	0.537
5497	220892_s_at	PSAT1	0.38406	0.50629	0.44287	-0.05881	0.675
5498	220924_s_at	SLC38A2	0.76095	0.67112	0.69041	0.07054	0.131
5499	220925_at	MAK10	0.59331	0.51013	0.56386	0.02945	0.42
5500	220926_s_at	EDEM3	0.86094	0.74299	0.72373	0.13721	0.066
5501	220931_at	MGC5590	0.25843	0.085655	0.23273	0.0257	0.447
5502	220933_s_at	ZCCHC6	0.54944	0.6291	0.63967	-0.09023	0.829
5503	220934_s_at	TMEM223	0.79207	0.72757	0.75873	0.03334	0.326
5504	220935_s_at	CDK5RAP2	0.62723	0.53527	0.50985	0.11738	0.19
5505	220937_s_at	ST6GALNAC4	0.45791	0.50536	0.39571	0.0622	0.374
5506	220939_s_at	DPP8	0.74352	0.7605	0.77513	-0.03161	0.689
5507	220940_at	ANKRD36B	0.08366	0.26269	0.34827	-0.26461	0.955
5508	220941_s_at	C21orf91	0.24153	0.28183	0.24132	0.00021	0.455
5509	220943_s_at	C2orf56	0.38743	0.42135	0.44257	-0.05514	0.633

5510	220944_at	PGLYRP4	0.13048	0.39234	0.22272	-0.09224	0.686
5511	220946_s_at	SETD2	0.47325	0.38765	0.47887	-0.00562	0.568
5512	220947_s_at	TBC1D10B	0.42559	0.62813	0.48676	-0.06117	0.647
5513	220948_s_at	ATP1A1	0.4793	0.49774	0.64483	-0.16553	0.923
5514	220949_s_at	C7orf49	0.43481	0.4053	0.55662	-0.12181	0.802
5515	220953_s_at	MTMR12	0.22045	0.3156	0.35792	-0.13747	0.818
5516	220956_s_at	EGLN2	0.232	0.26586	0.22462	0.00738	0.493
5517	220957_at	CTAGE1	0.28652	0.2985	0.45724	-0.17072	0.866
5518	220964_s_at	RAB1B	0.13276	0.25064	0.23176	-0.099	0.741
5519	220980_s_at	ADPGK	0.49512	0.37208	0.20783	0.28729	0.009
5520	220985_s_at	RNF170	0.44781	0.3163	0.4283	0.01951	0.439
5521	220987_s_at	C11orf17	0.12295	0.36892	0.21509	-0.09214	0.693
5522	220988_s_at	C1QTNF3	0.10831	0.03372	0.13746	-0.02915	0.622
5523	220990_s_at	MIR21	0.4918	0.27984	0.47321	0.01859	0.44
5524	220991_s_at	RNF32	0.12351	0.21163	0.11718	0.00633	0.536
5525	220992_s_at	C1orf25	0.51404	0.48447	0.42671	0.08733	0.187
5526	220993_s_at	GPR63	0.12316	0.033711	0.076823	0.046337	0.429
5527	221002_s_at	TSPAN14	0.52687	0.54556	0.59954	-0.07267	0.739
5528	221004_s_at	ITM2C	0.27984	0.37338	0.39959	-0.11975	0.796
5529	221007_s_at	FIP1L1	0.48456	0.33793	0.44544	0.03912	0.351
5530	221011_s_at	LBH	0.56355	0.60915	0.49876	0.06479	0.347
5531	221012_s_at	TRIM8	0.42957	0.28738	0.43061	-0.00104	0.504
5532	221014_s_at	RAB33B	0.24374	0.37746	0.4601	-0.21636	0.908
5533	221020_s_at	SLC25A32	0.79387	0.62706	0.63478	0.15909	0.029
5534	221021_s_at	CTNNBL1	0.19616	0.092112	0.13485	0.06131	0.337
5535	221027_s_at	PLA2G12A	0.70663	0.66661	0.71978	-0.01315	0.597
5536	221031_s_at	APOLD1	0.017054	0.15817	0.1422	-0.125146	0.828
5537	221036_s_at	APH1B	0.22849	0.47296	0.41356	-0.18507	0.861
5538	221038_at	UTP15	0.58603	0.52934	0.46214	0.12389	0.143
5539	221039_s_at	ASAP1	0.43044	0.55749	0.46691	-0.03647	0.602
5540	221044_s_at	TRIM34	0.62974	0.64164	0.68835	-0.05861	0.744
5541	221046_s_at	GTPBP8	0.44125	0.48735	0.49267	-0.05142	0.685
5542	221059_s_at	COTL1	0.31846	0.46477	0.4241	-0.10564	0.847
5543	221069_s_at	CCDC44	0.51996	0.58376	0.50505	0.01491	0.451
5544	221079_s_at	METTL2A	0.74507	0.61775	0.62442	0.12065	0.14
5545	221080_s_at	DENND1C	0.60775	0.67686	0.58322	0.02453	0.393
5546	221081_s_at	DENND2D	0.28241	0.41895	0.38803	-0.10562	0.749
5547	221087_s_at	APOL3	0.20229	0.27534	0.35444	-0.15215	0.809
5548	221090_s_at	OGFOD1	0.7451	0.75986	0.72169	0.02341	0.383
5549	221094_s_at	ELP3	0.4952	0.44656	0.503	-0.0078	0.521
5550	221096_s_at	TMCO6	0.44629	0.53707	0.48505	-0.03876	0.583
5551	221103_s_at	WDR52	0.31722	0.18201	0.28759	0.02963	0.39
5552	221104_s_at	NIPSNAP3B	0.27537	0.42479	0.24362	0.03175	0.442
5553	221139_s_at	CSAD	0.30657	0.079915	0.27709	0.02948	0.434
5554	221142_s_at	PECR	0.37655	0.52886	0.5273	-0.15075	0.877
5555	221188_s_at	CIDEB	0.21297	0.11606	0.11598	0.09699	0.36
5556	221190_s_at	C18orf8	0.54821	0.57275	0.26273	0.28548	0.005
5557	221193_s_at	ZCCHC10	0.71254	0.60832	0.71694	-0.0044	0.523
5558	221194_s_at	RNFT1	0.4462	0.42559	0.48212	-0.03592	0.588
5559	221203_s_at	YEATS2	0.70725	0.37338	0.32576	0.38149	0.011
5560	221207_s_at	NBEA	0.28403	0.45758	0.31093	-0.0269	0.593
5561	221208_s_at	C11orf61	0.65917	0.39083	0.48943	0.16974	0.061
5562	221210_s_at	NPL	0.57464	0.53076	0.45103	0.12361	0.202

5563	221211_s_at	C21orf7	0.054396	0.27546	0.2791	-0.224704	0.935
5564	221213_s_at	ZNF280D	0.34102	0.27475	0.25805	0.08297	0.276
5565	221214_s_at	NELF	0.47652	0.54794	0.42004	0.05648	0.35
5566	221216_s_at	SCMH1	0.74381	0.62016	0.58032	0.16349	0.032
5567	221218_s_at	TPK1	0.44513	0.53291	0.54154	-0.09641	0.825
5568	221219_s_at	KLHDC4	0.00014149	0.04229	0.18454	-0.18439851	0.844
5569	221221_s_at	KLHL3	0.4474	0.43413	0.2814	0.166	0.13
5570	221222_s_at	C1orf56	0.30399	0.21093	0.24019	0.0638	0.313
5571	221229_s_at	TRMT61B	0.044401	0.16143	0.16135	-0.116949	0.832
5572	221230_s_at	ARID4B	0.84491	0.77768	0.83495	0.00996	0.45
5573	221235_s_at	LOC644617	0.67781	0.63285	0.58726	0.09055	0.303
5574	221238_at	NSBP1	0.15788	0.064978	0.092935	0.064945	0.317
5575	221244_s_at	PDPK1	0.44145	0.35836	0.42693	0.01452	0.481
5576	221245_s_at	FZD5	0.58465	0.68215	0.65533	-0.07068	0.754
5577	221247_s_at	WBSCR16	0.53308	0.56652	0.54079	-0.00771	0.521
5578	221249_s_at	FAM117A	0.6748	0.61191	0.57477	0.10003	0.162
5579	221253_s_at	TXNDC5	0.67258	0.67274	0.60821	0.06437	0.255
5580	221255_s_at	TMEM93	0.87066	0.8973	0.89015	-0.01949	0.598
5581	221256_s_at	HDHD3	0.29738	0.2697	0.14776	0.14962	0.352
5582	221258_s_at	KIF18A	0.58562	0.52016	0.50405	0.08157	0.277
5583	221260_s_at	CSRNP2	0.39017	0.47987	0.36043	0.02974	0.389
5584	221263_s_at	SF3B5	0.88496	0.88561	0.90989	-0.02493	0.725
5585	221264_s_at	LOC100128223	0.12156	0.087582	0.21105	-0.08949	0.717
5586	221265_s_at	C15orf44	0.1177	0.24503	0.43987	-0.32217	0.981
5587	221267_s_at	FAM108A1	0.67271	0.63036	0.70866	-0.03595	0.697
5588	221268_s_at	SGPP1	0.65048	0.7177	0.75654	-0.10606	0.893
5589	221269_s_at	SH3BGRL3	0.70947	0.57898	0.73137	-0.0219	0.585
5590	221277_s_at	PUS3	0.6653	0.55482	0.50757	0.15773	0.051
5591	221286_s_at	MGC29506	0.4547	0.59071	0.60399	-0.14929	0.882
5592	221381_s_at	MORF4	0.60951	0.70972	0.66923	-0.05972	0.653
5593	221423_s_at	YIPF5	0.75855	0.82737	0.66312	0.09543	0.098
5594	221425_s_at	ISCA1	0.44458	0.51559	0.59939	-0.15481	0.856
5595	221428_s_at	TBL1XR1	0.61934	0.67849	0.70027	-0.08093	0.786
5596	221430_s_at	RNF146	0.26875	0.35006	0.30877	-0.04002	0.607
5597	221434_s_at	C14orf156	0.84881	0.83594	0.87818	-0.02937	0.717
5598	221436_s_at	CDCA3	0.67928	0.79612	0.47836	0.20092	0.094
5599	221437_s_at	MRPS15	0.80485	0.86734	0.82816	-0.02331	0.624
5600	221449_s_at	ITFG1	0.7048	0.63485	0.44977	0.25503	0.042
5601	221452_s_at	TMEM14B	0.61823	0.68849	0.7337	-0.11547	0.831
5602	221464_at	OR1D2	0.30688	0.19764	0.076818	0.230062	0.113
5603	221466_at	P2RY4	0.30166	0.27617	0.36475	-0.06309	0.604
5604	221471_at	SERINC3	0.74575	0.70626	0.79002	-0.04427	0.847
5605	221474_at	MYL12B	0.5985	0.63859	0.75887	-0.16037	0.929
5606	221475_s_at	RPL15	0.92665	0.91454	0.9387	-0.01205	0.793
5607	221479_s_at	BNIP3L	0.67547	0.7192	0.53985	0.13562	0.158
5608	221483_s_at	ARPP19	0.62324	0.65593	0.62473	-0.00149	0.492
5609	221484_at	B4GALT5	0.37856	0.40851	0.41677	-0.03821	0.643
5610	221486_at	ENSA	0.28095	0.17983	0.13065	0.1503	0.126
5611	221488_s_at	CUTA	0.75484	0.79744	0.80263	-0.04779	0.666
5612	221492_s_at	ATG3	0.84849	0.84032	0.73942	0.10907	0.168
5613	221493_at	TSPYL1	0.78926	0.7858	0.7112	0.07806	0.175
5614	221495_s_at	TCF25	0.65967	0.61599	0.5605	0.09917	0.2
5615	221502_at	KPNA3	0.8579	0.85262	0.75745	0.10045	0.014

5616	221504_s_at	ATP6V1H	0.051832	0.14278	0.032729	0.019103	0.426
5617	221505_at	ANP32E	0.83101	0.74132	0.74632	0.08469	0.105
5618	221506_s_at	TNPO2	0.4052	0.4566	0.33311	0.07209	0.314
5619	221509_at	DENR	0.56283	0.64123	0.52063	0.0422	0.455
5620	221513_s_at	UTP14A	0.50408	0.51578	0.53553	-0.03145	0.632
5621	221515_s_at	LCMT1	0.71577	0.4722	0.51381	0.20196	0.213
5622	221517_s_at	MED17	0.71327	0.55286	0.7505	-0.03723	0.701
5623	221518_s_at	USP47	0.68984	0.54812	0.5309	0.15894	0.135
5624	221520_s_at	CDCA8	0.66619	0.57548	0.32966	0.33653	0.004
5625	221521_s_at	GINS2	0.66431	0.74832	0.77637	-0.11206	0.821
5626	221522_at	ANKRD27	0.67405	0.79233	0.61921	0.05484	0.34
5627	221524_s_at	RRAGD	0.36096	0.45579	0.34458	0.01638	0.494
5628	221532_s_at	WDR61	0.69352	0.78094	0.80142	-0.1079	0.818
5629	221534_at	C11orf68	0.58903	0.45707	0.58025	0.00878	0.468
5630	221535_at	LSG1	0.31509	0.40702	0.38169	-0.0666	0.673
5631	221539_at	EIF4EBP1	0.48808	0.49886	0.63771	-0.14963	0.861
5632	221542_s_at	ERLIN2	0.57372	0.34538	0.40935	0.16437	0.12
5633	221548_s_at	ILKAP	0.57799	0.50271	0.29632	0.28167	0.058
5634	221549_at	GRWD1	0.56243	0.70022	0.59923	-0.0368	0.645
5635	221550_at	COX15	0.55416	0.66344	0.58743	-0.03327	0.643
5636	221553_at	MAGT1	0.69638	0.72319	0.60355	0.09283	0.083
5637	221558_s_at	LEF1	0.074025	0.34754	0.30165	-0.227625	0.949
5638	221559_s_at	MIS12	0.63814	0.6523	0.71509	-0.07695	0.768
5639	221561_at	SOAT1	0.72712	0.63038	0.70073	0.02639	0.417
5640	221568_s_at	LIN7C	0.64371	0.71794	0.71986	-0.07615	0.754
5641	221569_at	AHI1	0.43219	0.3806	0.29482	0.13737	0.165
5642	221570_s_at	METTL5	0.85821	0.88282	0.87003	-0.01182	0.607
5643	221571_at	TRAF3	0.58958	0.60234	0.53954	0.05004	0.363
5644	221575_at	SCLY	0.64177	0.7195	0.71119	-0.06942	0.748
5645	221580_s_at	TAF1D	0.75145	0.72921	0.71246	0.03899	0.321
5646	221582_at	HIST3H2A	0.36961	0.45135	0.52479	-0.15518	0.836
5647	221586_s_at	E2F5	0.33226	0.51119	0.35813	-0.02587	0.574
5648	221587_s_at	C19orf24	0.44619	0.40269	0.42551	0.02068	0.461
5649	221591_s_at	FAM64A	0.27211	0.43523	0.31982	-0.04771	0.642
5650	221593_s_at	RPL31	0.66927	0.75657	0.84117	-0.1719	0.977
5651	221594_at	C7orf64	0.37187	0.30684	0.53897	-0.1671	0.907
5652	221595_at	DKFZP564O0523	0.25063	0.17443	0.003522	0.247108	0.053
5653	221597_s_at	TMEM208	0.83812	0.81695	0.87316	-0.03504	0.724
5654	221600_s_at	C11orf67	0.44127	0.49069	0.50528	-0.06401	0.645
5655	221602_s_at	FAIM3	0.71856	0.69543	0.69316	0.0254	0.428
5656	221610_s_at	STAP2	0.13222	0.05632	0.23165	-0.09943	0.713
5657	221614_s_at	RPH3AL	0.012729	0.13578	0.12482	-0.112091	0.769
5658	221616_s_at	TAF9B	0.44838	0.51545	0.52552	-0.07714	0.755
5659	221619_s_at	MTCH1	0.72028	0.77905	0.60568	0.1146	0.133
5660	221620_s_at	APOO	0.74687	0.77395	0.82774	-0.08087	0.855
5661	221621_at	C17orf86	0.057284	0.073862	0.11062	-0.053336	0.635
5662	221622_s_at	TMEM126B	0.78309	0.79723	0.67033	0.11276	0.142
5663	221626_at	ZNF506	0.48006	0.33658	0.29469	0.18537	0.112
5664	221632_s_at	WDR4	0.10983	0.18027	0.086497	0.023333	0.429
5665	221634_at	RPL23AP7	0.31767	0.37293	0.41318	-0.09551	0.77
5666	221637_s_at	C11orf48	0.88604	0.85006	0.82178	0.06426	0.129
5667	221638_s_at	STX16	0.78485	0.66667	0.77488	0.00997	0.431
5668	221640_s_at	LRDD	0.22076	0.33726	0.38322	-0.16246	0.903

5669	221641_s_at	ACOT9	0.41347	0.3385	0.37752	0.03595	0.379
5670	221643_s_at	RERE	0.30352	0.40208	0.38461	-0.08109	0.754
5671	221645_s_at	ZNF83	0.33619	0.34054	0.50962	-0.17343	0.882
5672	221647_s_at	RIC8A	0.14834	0.34401	0.24597	-0.09763	0.721
5673	221649_s_at	PPAN	0.36407	0.5626	0.49175	-0.12768	0.859
5674	221650_s_at	MED18	0.27124	0.24847	0.23453	0.03671	0.631
5675	221652_s_at	C12orf11	0.74968	0.65259	0.48062	0.26906	0.011
5676	221657_s_at	ASB6	0.038173	0.14002	0.099081	-0.060908	0.663
5677	221666_s_at	PYCARD	0.5128	0.68086	0.60287	-0.09007	0.787
5678	221669_s_at	ACAD8	0.65707	0.58358	0.60686	0.05021	0.318
5679	221673_s_at	CSNK1G1	0.28326	0.16587	0.31431	-0.03105	0.592
5680	221675_s_at	CHPT1	0.14737	0.30156	0.24622	-0.09885	0.825
5681	221676_s_at	CORO1C	0.59345	0.65651	0.57131	0.02214	0.468
5682	221677_s_at	DONSON	0.58978	0.61492	0.66745	-0.07767	0.734
5683	221680_s_at	ETV7	0.58446	0.26574	0.2641	0.32036	0.009
5684	221683_s_at	CEP290	0.24811	0.4956	0.37065	-0.12254	0.797
5685	221685_s_at	CCDC99	0.74699	0.6809	0.69679	0.0502	0.302
5686	221688_s_at	IMP3	0.7631	0.78328	0.81151	-0.04841	0.705
5687	221689_s_at	PIGP	0.69603	0.73505	0.80634	-0.11031	0.844
5688	221692_s_at	MRPL34	0.78266	0.6603	0.73086	0.0518	0.318
5689	221699_s_at	DDX50	0.70292	0.64498	0.67887	0.02405	0.439
5690	221700_s_at	UBA52	0.93454	0.91986	0.91895	0.01559	0.18
5691	221702_s_at	TM2D3	0.29356	0.41312	0.47246	-0.1789	0.908
5692	221703_at	BRIP1	0.67291	0.65891	0.67916	-0.00625	0.523
5693	221704_s_at	VPS37B	0.27007	0.32328	0.3954	-0.12533	0.771
5694	221708_s_at	UNC45A	0.37259	0.55251	0.38642	-0.01383	0.531
5695	221711_s_at	C19orf62	0.55411	0.58347	0.59372	-0.03961	0.643
5696	221712_s_at	WDR74	0.63919	0.67247	0.70711	-0.06792	0.761
5697	221725_at	WASF2	0.38402	0.52937	0.43235	-0.04833	0.624
5698	221727_at	SUB1	0.68001	0.39928	0.55318	0.12683	0.064
5699	221737_at	GNA12	0.34209	0.35066	0.32801	0.01408	0.451
5700	221738_at	KIAA1219	0.79799	0.51779	0.64048	0.15751	0.063
5701	221739_at	C19orf10	0.55388	0.7076	0.78002	-0.22614	0.985
5702	221741_s_at	YTHDF1	0.70204	0.73567	0.68211	0.01993	0.402
5703	221744_at	WDR68	0.62217	0.57352	0.50874	0.11343	0.136
5704	221746_at	UBL4A	0.29608	0.22173	0.32249	-0.02641	0.628
5705	221749_at	YTHDF3	0.82478	0.83416	0.89495	-0.07017	0.855
5706	221750_at	HMGCS1	0.32724	0.42561	0.51712	-0.18988	0.91
5707	221751_at	SLC2A3P1	0.70966	0.7477	0.73142	-0.02176	0.62
5708	221753_at	SSH1	0.51172	0.35349	0.42325	0.08847	0.231
5709	221758_at	ARMC6	0.16904	0.18972	0.20863	-0.03959	0.589
5710	221760_at	MAN1A1	0.25718	0.5141	0.39947	-0.14229	0.85
5711	221761_at	ADSS	0.88222	0.83994	0.83073	0.05149	0.169
5712	221763_at	JMJD1C	0.75731	0.7293	0.7399	0.01741	0.377
5713	221766_s_at	FAM46A	0.70081	0.77444	0.6876	0.01321	0.514
5714	221770_at	RPE	0.78689	0.59127	0.62148	0.16541	0.02
5715	221771_s_at	MPHOSPH8	0.62503	0.74423	0.66276	-0.03773	0.726
5716	221776_s_at	BRD7	0.22695	0.55357	0.49093	-0.26398	0.923
5717	221780_s_at	DDX27	0.51189	0.4409	0.50961	0.00228	0.468
5718	221782_at	DNAJC10	0.84201	0.82334	0.79623	0.04578	0.172
5719	221786_at	C6orf120	0.55708	0.6848	0.67572	-0.11864	0.891
5720	221791_s_at	CCDC72	0.87504	0.8385	0.88718	-0.01214	0.737
5721	221800_s_at	C17orf70	0.43305	0.38752	0.26442	0.16863	0.099

5722	221803_s_at	NRBF2	0.75551	0.76931	0.67172	0.08379	0.096
5723	221804_s_at	FAM45A	0.21807	0.18696	0.14092	0.07715	0.263
5724	221806_s_at	SETD5	0.75416	0.73897	0.76957	-0.01541	0.563
5725	221808_at	RAB9A	0.65664	0.70867	0.62154	0.0351	0.366
5726	221813_at	FBXO42	0.48601	0.28195	0.30241	0.1836	0.09
5727	221816_s_at	PHF11	0.62285	0.65754	0.55765	0.0652	0.264
5728	221817_at	DOLPP1	0.40866	0.30952	0.39905	0.00961	0.47
5729	221821_s_at	C12orf41	0.23333	0.10443	0.30076	-0.06743	0.687
5730	221823_at	C5orf30	0.54393	0.5749	0.4952	0.04873	0.336
5731	221824_s_at	MARCH8	0.53757	0.63669	0.53226	0.00531	0.514
5732	221825_at	ANGEL2	0.68878	0.75378	0.63538	0.0534	0.321
5733	221829_s_at	TNPO1	0.53691	0.58211	0.66653	-0.12962	0.84
5734	221840_at	PTPRE	0.63005	0.69888	0.7413	-0.11125	0.825
5735	221845_s_at	CLPB	0.6876	0.40897	0.46948	0.21812	0.007
5736	221847_at	LOC100129361	0.68978	0.74679	0.72228	-0.0325	0.582
5737	221853_s_at	NOMO1	0.32997	0.39274	0.3553	-0.02533	0.577
5738	221858_at	TBC1D12	0.28107	0.23122	0.23017	0.0509	0.304
5739	221864_at	ORAI3	0.77591	0.59588	0.5425	0.23341	0.009
5740	221865_at	C9orf91	0.4678	0.51411	0.48634	-0.01854	0.577
5741	221871_s_at	TFG	0.65408	0.73438	0.72023	-0.06615	0.855
5742	221873_at	ZNF143	0.70743	0.45344	0.64164	0.06579	0.271
5743	221881_s_at	CLIC4	0.24808	0.26269	0.47635	-0.22827	0.896
5744	221882_s_at	TMEM8	0.50773	0.66098	0.62129	-0.11356	0.819
5745	221896_s_at	HIGD1A	0.37649	0.35588	0.37586	0.00063	0.495
5746	221897_at	TRIM52	0.43672	0.16853	0.27585	0.16087	0.171
5747	221904_at	FAM131A	0.38229	0.19234	0.26911	0.11318	0.236
5748	221905_at	CYLD	0.4593	0.52272	0.5787	-0.1194	0.77
5749	221912_s_at	CCDC28B	0.82534	0.73836	0.75177	0.07357	0.21
5750	221915_s_at	RANBP1	0.48092	0.41722	0.43118	0.04974	0.347
5751	221918_at	PCTK2	0.65183	0.72452	0.58085	0.07098	0.187
5752	221922_at	GPSM2	0.40767	0.34062	0.44094	-0.03327	0.591
5753	221925_s_at	CSPP1	0.2463	0.33409	0.3197	-0.0734	0.663
5754	221927_s_at	ABHD11	0.40423	0.41635	0.41889	-0.01466	0.55
5755	221931_s_at	SEH1L	0.76867	0.84403	0.79782	-0.02915	0.642
5756	221932_s_at	GLRX5	0.76951	0.71864	0.72304	0.04647	0.375
5757	221935_s_at	C3orf64	0.4828	0.63222	0.4618	0.021	0.453
5758	221940_at	RPUSD2	0.30057	0.042848	0.34241	-0.04184	0.604
5759	221957_at	PDK3	0.44844	0.4716	0.32333	0.12511	0.264
5760	221960_s_at	RAB2A	0.77586	0.77959	0.74623	0.02963	0.398
5761	221962_s_at	UBE2H	0.25741	0.27178	0.27105	-0.01364	0.562
5762	221965_at	MPHOSPH9	0.48041	0.52594	0.55266	-0.07225	0.712
5763	221969_at	PAX5	0.2994	0.34438	0.42662	-0.12722	0.88
5764	221970_s_at	NOL11	0.73748	0.75634	0.79719	-0.05971	0.758
5765	221972_s_at	SDF4	0.66677	0.65795	0.68422	-0.01745	0.605
5766	221978_at	HLA-F	0.21988	0.11193	0.15926	0.06062	0.356
5767	221983_at	FAM134A	0.64289	0.62938	0.52172	0.12117	0.203
5768	221985_at	KLHL24	0.32602	0.52087	0.25046	0.07556	0.294
5769	221987_s_at	TSR1	0.78081	0.74999	0.65054	0.13027	0.023
5770	221992_at	PDXDC2	0.40073	0.38586	0.42142	-0.02069	0.549
5771	221995_s_at	MRP63	0.79375	0.69397	0.75983	0.03392	0.296
5772	221997_s_at	MRPL52	0.63874	0.70619	0.70564	-0.0669	0.729
5773	222000_at	C1orf174	0.67419	0.61746	0.64982	0.02437	0.43
5774	222006_at	LETM1	0.36128	0.44728	0.57911	-0.21783	0.921

5775	222010_at	TCP1	0.71617	0.65788	0.70814	0.00803	0.46
5776	222019_at	PFDN6	0.30217	0.11108	0.053019	0.249151	0.107
5777	222027_at	NUCKS1	0.42791	0.63638	0.57066	-0.14275	0.815
5778	222028_at	ZNF45	0.632	0.61063	0.687	-0.055	0.673
5779	222030_at	SIVA1	0.019305	0.30482	0.099327	-0.080022	0.688
5780	222031_at	LOC286434	0.13111	0.19857	0.021798	0.109312	0.237
5781	222034_at	GNB2L1	0.22887	0.23391	0.03574	0.19313	0.053
5782	222038_s_at	UTP18	0.65148	0.75153	0.68833	-0.03685	0.632
5783	222039_at	KIF18B	0.71059	0.56642	0.53659	0.174	0.084
5784	222040_at	HNRNPA1	0.27634	0.51298	0.49032	-0.21398	0.904
5785	222051_s_at	LOC100131637	0.54542	0.706	0.62601	-0.08059	0.739
5786	222052_at	C19orf54	0.5985	0.49395	0.56912	0.02938	0.402
5787	222064_s_at	AARSD1	0.65957	0.61481	0.6095	0.05007	0.337
5788	222077_s_at	RACGAP1	0.74075	0.7902	0.77024	-0.02949	0.606
5789	222088_s_at	SLC2A14	0.42276	0.19726	0.50296	-0.0802	0.742
5790	222103_at	ATF1	0.78333	0.78806	0.72798	0.05535	0.273
5791	222105_s_at	NKIRAS2	0.37514	0.39304	0.4269	-0.05176	0.658
5792	222118_at	CENPN	0.50094	0.41797	0.3747	0.12624	0.145
5793	222119_s_at	FBXO11	0.75182	0.8046	0.77719	-0.02537	0.641
5794	222125_s_at	P4HTM	0.58361	0.50353	0.61149	-0.02788	0.614
5795	222127_s_at	EXOC1	0.73916	0.6869	0.70947	0.02969	0.407
5796	222128_at	NSUN6	0.025057	0.1088	0.084334	-0.059277	0.669
5797	222130_s_at	FTSJ2	0.10815	0.079012	0.00087484	0.10727516	0.193
5798	222139_at	KIAA1466	0.54093	0.38225	0.41909	0.12184	0.216
5799	222140_s_at	GPR89A	0.69552	0.57458	0.48935	0.20617	0.042
5800	222143_s_at	MTMR14	0.57563	0.44625	0.55355	0.02208	0.435
5801	222147_s_at	ACTR5	0.18496	0.1453	0.28098	-0.09602	0.724
5802	222148_s_at	RHOT1	0.36208	0.35301	0.57845	-0.21637	0.912
5803	222150_s_at	PION	0.048717	0.39488	0.36053	-0.311813	0.989
5804	222154_s_at	LOC26010	0.55228	0.6271	0.65101	-0.09873	0.77
5805	222155_s_at	GPR172A	0.49561	0.38504	0.60299	-0.10738	0.831
5806	222175_s_at	MED15	0.57253	0.3521	0.36687	0.20566	0.124
5807	222193_at	C2orf43	0.57546	0.50525	0.42353	0.15193	0.101
5808	222199_s_at	BIN3	0.40463	0.42195	0.39423	0.0104	0.46
5809	222200_s_at	BSDC1	0.30671	0.38345	0.31326	-0.00655	0.512
5810	222201_s_at	CASP8AP2	0.76969	0.52893	0.7327	0.03699	0.297
5811	222203_s_at	RDH14	0.69619	0.55636	0.50008	0.19611	0.023
5812	222204_s_at	RRN3	0.54547	0.6267	0.63765	-0.09218	0.763
5813	222208_s_at	POLR2J4	0.12521	0.16068	0.17898	-0.05377	0.65
5814	222209_s_at	TMEM135	0.46488	0.70157	0.73767	-0.27279	0.987
5815	222212_s_at	LASS2	0.25004	0.18376	0.11831	0.13173	0.196
5816	222216_s_at	MRPL17	0.79873	0.81063	0.86834	-0.06961	0.8
5817	222228_s_at	ALKBH4	0.45244	0.046862	0.24191	0.21053	0.079
5818	222230_s_at	ACTR10	0.37801	0.47693	0.36196	0.01605	0.524
5819	222231_s_at	LRRC59	0.70404	0.69183	0.76615	-0.06211	0.821
5820	222233_s_at	DCLRE1C	0.5458	0.27295	0.51845	0.02735	0.395
5821	222235_s_at	CSGALNACT2	0.70793	0.54266	0.72569	-0.01776	0.6
5822	222239_s_at	INTS6	0.68117	0.41908	0.68953	-0.00836	0.529
5823	222243_s_at	TOB2	0.6926	0.53196	0.69907	-0.00647	0.496
5824	222244_s_at	TUG1	0.63677	0.60645	0.68724	-0.05047	0.724
5825	222250_s_at	INTS7	0.48837	0.52244	0.5044	-0.01603	0.513
5826	222262_s_at	ETNK1	0.43096	0.52106	0.46868	-0.03772	0.608
5827	222263_at	SLC35E1	0.26507	0.44036	0.44139	-0.17632	0.893

5828	222264_at	HNRNPUL2	0.26074	0.27999	0.35156	-0.09082	0.741
5829	222267_at	TMEM209	0.34429	0.47447	0.51603	-0.17174	0.869
5830	222270_at	SMEK2	0.27049	0.034733	0.063372	0.207118	0.079
5831	222273_at	PAPOLG	0.58152	0.45549	0.42842	0.1531	0.141
5832	222275_at	MRPS30	0.60121	0.69721	0.57162	0.02959	0.336
5833	222279_at	RP3-377H14.5	0.54592	0.57822	0.48308	0.06284	0.358
5834	222305_at	HK2	0.064314	0.082634	0.050687	0.013627	0.455
5835	222310_at	SFRS15	0.25154	0.33526	0.2509	0.00064	0.504
5836	222316_at	USO1	0.55987	0.45394	0.35882	0.20105	0.044
5837	222326_at	PDE4B	0.15095	0.13666	0.23741	-0.08646	0.724
5838	222344_at	C5orf13	0.36492	0.43567	0.40909	-0.04417	0.61
5839	222360_at	DPH5	0.33098	0.35721	0.37592	-0.04494	0.642
5840	222366_at	ADNP	0.14457	0.23429	0.26343	-0.11886	0.833
5841	222369_at	NAT11	0.50283	0.54006	0.57336	-0.07053	0.723
5842	222376_at	HACE1	0.56727	0.32545	0.31952	0.24775	0.073
5843	266_s_at	CD24	0.32077	0.53522	0.49889	-0.17812	0.893
5844	31845_at	ELF4	0.63499	0.61523	0.57771	0.05728	0.236
5845	32032_at	DGCR14	0.12729	0.11877	0.13102	-0.00373	0.526
5846	32091_at	SLC25A44	0.30945	0.54644	0.23394	0.07551	0.269
5847	32811_at	MYO1C	0.38136	0.54111	0.49533	-0.11397	0.782
5848	32836_at	AGPAT1	0.64536	0.58446	0.53225	0.11311	0.157
5849	33322_i_at	SFN	0.4285	0.52021	0.48825	-0.05975	0.683
5850	33494_at	ETFDH	0.62781	0.53021	0.54397	0.08384	0.275
5851	33760_at	PEX14	0.31551	0.1764	0.15515	0.16036	0.092
5852	33814_at	PAK4	0.29399	0.33654	0.29637	-0.00238	0.527
5853	33850_at	MAP4	0.24298	0.39133	0.3404	-0.09742	0.784
5854	34210_at	CD52	0.80099	0.77974	0.81796	-0.01697	0.609
5855	34221_at	HMGXB3	0.3769	0.37547	0.38479	-0.00789	0.525
5856	34260_at	TELO2	0.4892	0.52185	0.29924	0.18996	0.102
5857	34408_at	RTN2	0.11424	0.27918	0.29875	-0.18451	0.939
5858	34858_at	KCTD2	0.50802	0.44116	0.44771	0.06031	0.319
5859	34868_at	SMG5	0.58248	0.52045	0.39703	0.18545	0.097
5860	35160_at	LDB1	0.68321	0.33403	0.49425	0.18896	0.046
5861	35265_at	FXR2	0.21355	0.21049	0.20532	0.00823	0.464
5862	35626_at	SGSH	0.35474	0.32894	0.18986	0.16488	0.147
5863	35666_at	SEMA3F	0.60755	0.56232	0.49127	0.11628	0.144
5864	35671_at	GTF3C1	0.46082	0.4089	0.59351	-0.13269	0.777
5865	36019_at	STK19	0.60495	0.6355	0.55899	0.04596	0.332
5866	36030_at	IFFO1	0.66312	0.49509	0.4367	0.22642	0.009
5867	36084_at	CUL7	0.47336	0.18479	0.46442	0.00894	0.487
5868	36475_at	GCAT	0.16756	0.44462	0.30256	-0.135	0.824
5869	36545_s_at	SFI1	0.41571	0.40183	0.15913	0.25658	0.051
5870	36552_at	C2CD3	0.68514	0.7094	0.58457	0.10057	0.126
5871	36711_at	MAFF	0.54049	0.31597	0.43313	0.10736	0.181
5872	36829_at	PER1	0.4466	0.1614	0.089028	0.357572	0.018
5873	36865_at	ANGEL1	0.35657	0.17911	0.33508	0.02149	0.447
5874	36907_at	MVK	0.75862	0.74888	0.74109	0.01753	0.423
5875	36920_at	MTM1	0.51979	0.43151	0.50858	0.01121	0.487
5876	36994_at	ATP6V0C	0.84263	0.7555	0.75515	0.08748	0.133
5877	37012_at	CAPZB	0.75458	0.6872	0.56063	0.19395	0.016
5878	37028_at	PPP1R15A	0.68066	0.55355	0.55025	0.13041	0.116
5879	37079_at	YDD19	0.78815	0.77452	0.70281	0.08534	0.093
5880	37170_at	BMP2K	0.47427	0.46129	0.42883	0.04544	0.539

5881	37226_at	BNIP1	0.45586	0.44602	0.28528	0.17058	0.212
5882	37232_at	KIAA0586	0.56487	0.44775	0.46224	0.10263	0.284
5883	37254_at	ZNF133	0.2716	0.167	0.17794	0.09366	0.259
5884	37278_at	TAZ	0.53105	0.40433	0.39612	0.13493	0.187
5885	37462_i_at	SF3A2	0.59828	0.79671	0.75079	-0.15251	0.972
5886	37512_at	HSD17B6	0.35449	0.28808	0.35401	0.00048	0.527
5887	37802_r_at	FAM63B	0.10319	0.18607	0.2534	-0.15021	0.867
5888	37860_at	ZNF337	0.64965	0.55558	0.46172	0.18793	<0.001
5889	37872_at	JRK	0.4466	0.53421	0.52046	-0.07386	0.689
5890	37966_at	PARVB	0.59048	0.62071	0.65888	-0.0684	0.761
5891	38290_at	RGS14	0.34516	0.39931	0.3419	0.00326	0.503
5892	38398_at	MADD	0.61244	0.56265	0.30622	0.30622	0.02
5893	38447_at	ADRBK1	0.70858	0.58855	0.71817	-0.00959	0.531
5894	38892_at	KIAA0240	0.64348	0.59157	0.4658	0.17768	0.083
5895	39248_at	AQP3	0.49805	0.70448	0.68006	-0.18201	0.971
5896	39318_at	TCL1A	0.29503	0.24081	0.33191	-0.03688	0.667
5897	39729_at	PRDX2	0.27761	0.29445	0.30867	-0.03106	0.614
5898	39817_s_at	C6orf108	0.64324	0.65995	0.71552	-0.07228	0.745
5899	40149_at	SH2B1	0.4739	0.31906	0.3283	0.1456	0.098
5900	40255_at	DDX28	0.34821	0.20146	0.12842	0.21979	0.036
5901	40359_at	RASSF7	0.72128	0.57042	0.49953	0.22175	0.078
5902	40420_at	STK10	0.80005	0.73517	0.65408	0.14597	0.053
5903	40446_at	PHF1	0.4986	0.47399	0.39414	0.10446	0.138
5904	40465_at	DDX23	0.50048	0.72409	0.57666	-0.07618	0.687
5905	41160_at	MBD3	0.81724	0.80657	0.81919	-0.00195	0.547
5906	41220_at	SEPT9	0.83119	0.85248	0.84315	-0.01196	0.603
5907	41387_r_at	KDM6B	0.40597	0.32899	0.36236	0.04361	0.415
5908	41512_at	BRAP	0.5617	0.49631	0.53576	0.02594	0.429
5909	41577_at	PPP1R16B	0.56309	0.57348	0.49179	0.0713	0.273
5910	41858_at	FRAG1	0.14128	0.34397	0.4002	-0.25892	0.94
5911	43544_at	MED16	0.61274	0.64966	0.63355	-0.02081	0.571
5912	43977_at	TMEM161A	0.46405	0.62764	0.45681	0.00724	0.466
5913	44065_at	C12orf52	0.17227	0.2711	0.22564	-0.05337	0.646
5914	44669_at	LOC644096	0.48536	0.51479	0.6319	-0.14654	0.807
5915	44702_at	SYDE1	0.4505	0.39141	0.39219	0.05831	0.308
5916	44783_s_at	HEY1	0.62795	0.77889	0.74045	-0.1125	0.931
5917	45687_at	PRR14	0.58156	0.55031	0.56653	0.01503	0.458
5918	46167_at	TTC4	0.63059	0.65665	0.56661	0.06398	0.295
5919	46270_at	UBAP1	0.66278	0.48691	0.52329	0.13949	0.077
5920	46665_at	SEMA4C	0.49966	0.64151	0.55118	-0.05152	0.664
5921	47083_at	C7orf26	0.27093	0.47309	0.36292	-0.09199	0.731
5922	47571_at	ZNF236	0.25074	0.45219	0.29664	-0.0459	0.605
5923	47608_at	TJAP1	0.15405	0.3513	0.42062	-0.26657	0.958
5924	48531_at	TNIP2	0.39287	0.61754	0.38123	0.01164	0.441
5925	48659_at	MIIP	0.75014	0.74718	0.77828	-0.02814	0.581
5926	49306_at	RASSF4	0.61401	0.74187	0.61116	0.00285	0.51
5927	49327_at	SIRT3	0.39358	0.58905	0.40937	-0.01579	0.561
5928	49485_at	PRDM4	0.48982	0.56039	0.36189	0.12793	0.148
5929	50221_at	TFEB	0.77146	0.78966	0.77606	-0.0046	0.551
5930	50277_at	GGA1	0.65126	0.71318	0.71235	-0.06109	0.724
5931	50374_at	C17orf90	0.782	0.68373	0.80161	-0.01961	0.611
5932	51158_at	FAM174B	0.21166	0.30555	0.3833	-0.17164	0.841
5933	51774_s_at	LOC222070	0.4826	0.53875	0.55869	-0.07609	0.769

5934	52078_at	TMEM222	0.38777	0.47635	0.49375	-0.10598	0.854
5935	52164_at	C11orf24	0.38222	0.46714	0.52627	-0.14405	0.936
5936	52169_at	STRADA	0.74902	0.69455	0.76613	-0.01711	0.619
5937	52741_at	TRMT61A	0.45244	0.42017	0.32986	0.12258	0.221
5938	53076_at	B4GALT7	0.27275	0.3147	0.39189	-0.11914	0.809
5939	53912_at	SNX11	0.23223	0.36196	0.061571	0.170659	0.14
5940	53987_at	RANBP10	0.54454	0.36353	0.14642	0.39812	0.005
5941	54037_at	HPS4	0.24999	0.19382	0.14489	0.1051	0.244
5942	54051_at	PKNOX1	0.25257	0.40049	0.13965	0.11292	0.311
5943	54970_at	ZMIZ2	0.55237	0.58724	0.48838	0.06399	0.3
5944	55065_at	MARK4	0.58483	0.52425	0.58825	-0.00342	0.513
5945	55081_at	MICALL1	0.57131	0.66274	0.71213	-0.14082	0.833
5946	55662_at	C10orf76	0.47154	0.48237	0.38908	0.08246	0.349
5947	55692_at	ELMO2	0.58759	0.6283	0.75735	-0.16976	0.962
5948	55705_at	C19orf22	0.36554	0.56778	0.50422	-0.13868	0.812
5949	55872_at	ZNF512B	0.26697	0.38128	0.25206	0.01491	0.501
5950	56256_at	SIDT2	0.72433	0.75191	0.75646	-0.03213	0.702
5951	56919_at	WDR48	0.21866	0.19653	0.22507	-0.00641	0.533
5952	57082_at	LDLRAP1	0.37073	0.41241	0.56597	-0.19524	0.925
5953	57163_at	ELOVL1	0.387	0.55068	0.61475	-0.22775	0.958
5954	57539_at	ZGPAT	0.43702	0.58429	0.55843	-0.12141	0.781
5955	58308_at	TRIM62	0.1732	0.25471	0.12396	0.04924	0.436
5956	58696_at	EXOSC4	0.86216	0.8031	0.89962	-0.03746	0.735
5957	60528_at	JMJD7-PLA2G4B	0.37614	0.47437	0.43017	-0.05403	0.664
5958	63009_at	SHQ1	0.70766	0.78446	0.74846	-0.0408	0.665
5959	632_at	GSK3A	0.12614	0.42295	0.24021	-0.11407	0.687
5960	635_s_at	PPP2R5B	0.31002	0.23917	0.096787	0.213233	0.076
5961	64371_at	SFRS14	0.42373	0.31423	0.11207	0.31166	0.001
5962	64486_at	CORO1B	0.41891	0.57972	0.47356	-0.05465	0.655
5963	64488_at	IRGQ	0.11894	0.27404	0.4066	-0.28766	0.991
5964	64883_at	MOSPD2	0.15806	0.23838	0.30147	-0.14341	0.864
5965	64900_at	FLJ22167	0.14046	0.40841	0.42805	-0.28759	0.963
5966	65133_i_at	INO80B	0.63048	0.49074	0.50366	0.12682	0.121
5967	65585_at	FAM86B1	0.46225	0.48124	0.56686	-0.10461	0.805
5968	65588_at	LOC388796	0.093576	0.2447	0.27058	-0.177004	0.822
5969	65630_at	TMEM80	0.20489	0.2552	0.24202	-0.03713	0.628
5970	65770_at	RHOT2	0.72102	0.82661	0.80438	-0.08336	0.917
5971	78383_at	LOC100129250	0.4061	0.4894	0.51606	-0.10996	0.752
5972	89948_at	PCIF1	0.16496	0.39656	0.35126	-0.1863	0.87
5973	90610_at	LRCH4	0.1581	0.21958	0.29902	-0.14092	0.863
5974	91703_at	EHBP1L1	0.63949	0.66848	0.69255	-0.05306	0.73
5975	91816_f_at	MEX3D	0.097616	0.31341	0.21714	-0.119524	0.721

Supplemental Table 6. Gene pairs that are differentially coexpressed following ER stress

Affymetrix ID #1	Gene Symbol # 1	Affymetrix ID #2	Gene Symbol # 2	R - DMSO	R - Tunicamycin	Nominal P-value
200002_at	RPL35	200599_s_at	HSP90B1	0.48	0.88	4.90E-12
200010_at	RPL11	200599_s_at	HSP90B1	0.47	0.89	1.29E-13
200017_at	RPS27A	200599_s_at	HSP90B1	0.49	0.90	1.81E-13
200018_at	RPS13	200599_s_at	HSP90B1	0.47	0.89	4.96E-13
200019_s_at	FAU	200599_s_at	HSP90B1	0.46	0.86	4.22E-10
200026_at	RPL34	200599_s_at	HSP90B1	0.46	0.88	7.71E-12
200029_at	RPL19	200599_s_at	HSP90B1	0.50	0.92	3.33E-16
200032_s_at	RPL9	200599_s_at	HSP90B1	0.43	0.85	2.48E-10
200038_s_at	RPL17	200599_s_at	HSP90B1	0.46	0.88	3.28E-12
200061_s_at	RPS24	200599_s_at	HSP90B1	0.48	0.90	8.52E-14
200062_s_at	RPL30	200599_s_at	HSP90B1	0.50	0.92	1.11E-16
200063_s_at	NPM1	200599_s_at	HSP90B1	0.47	0.90	2.78E-14
200077_s_at	OAZ1	200599_s_at	HSP90B1	0.49	0.87	2.07E-10
200082_s_at	RPS7	200599_s_at	HSP90B1	0.44	0.89	7.44E-15
200092_s_at	RPL37	200599_s_at	HSP90B1	0.46	0.88	2.84E-12
200599_s_at	HSP90B1	200633_at	UBB	0.42	0.89	3.31E-14
200599_s_at	HSP90B1	200634_at	PFN1	0.37	0.86	1.47E-12
200599_s_at	HSP90B1	200674_s_at	RPL32	0.47	0.87	2.59E-11
200599_s_at	HSP90B1	200741_s_at	RPS27	0.48	0.89	9.47E-13
200599_s_at	HSP90B1	200763_s_at	RPLP1	0.50	0.91	1.17E-14
200599_s_at	HSP90B1	200819_s_at	RPS15	0.47	0.90	1.57E-14
200599_s_at	HSP90B1	200834_s_at	RPS21	0.50	0.88	6.83E-11
200599_s_at	HSP90B1	200926_at	RPS23	0.45	0.88	1.27E-12
200599_s_at	HSP90B1	201049_s_at	RPS18	0.52	0.91	6.88E-15
200599_s_at	HSP90B1	201094_at	RPS29	0.46	0.89	5.03E-14
200599_s_at	HSP90B1	201258_at	RPS16	0.50	0.88	1.41E-11
200599_s_at	HSP90B1	201429_s_at	RPL37A	0.52	0.92	1.11E-16
200599_s_at	HSP90B1	201492_s_at	RPL41	0.51	0.92	7.77E-16
201412_at	LRP10	201582_at	SEC23B	0.53	-0.17	1.35E-09
201502_s_at	NFKBIA	201626_at	INSIG1	-0.36	0.39	3.76E-10
200990_at	TRIM28	202060_at	CTR9	0.30	-0.42	8.73E-10
200599_s_at	HSP90B1	202064_s_at	SEL1L	0.72	0.11	1.65E-10
201077_s_at	NHP2L1	202115_s_at	NOC2L	0.53	-0.17	1.04E-09
200599_s_at	HSP90B1	202433_at	SLC35B1	0.57	-0.11	1.37E-09
202636_at	RNF103	202843_at	DNAJB9	0.39	-0.35	5.15E-10
201626_at	INSIG1	203276_at	LMNB1	0.35	-0.36	2.13E-09
201626_at	INSIG1	204394_at	SLC43A1	0.40	-0.34	4.94E-10
201626_at	INSIG1	204573_at	CROT	-0.33	0.42	2.32E-10
201626_at	INSIG1	204835_at	POLA1	0.21	-0.50	8.10E-10
201614_s_at	RUVBL1	205141_at	ANG	-0.33	0.42	2.75E-10
201626_at	INSIG1	205141_at	ANG	0.47	-0.33	1.16E-11
203261_at	DCTN6	205141_at	ANG	-0.44	0.39	1.04E-12
200599_s_at	HSP90B1	205158_at	RNASE4	0.54	-0.22	3.86E-11
201614_s_at	RUVBL1	205158_at	RNASE4	-0.26	0.46	8.32E-10
203082_at	BMS1	205158_at	RNASE4	-0.07	0.66	5.83E-12
203261_at	DCTN6	205158_at	RNASE4	-0.08	0.63	8.16E-11
202060_at	CTR9	205246_at	PEX13	-0.56	0.11	1.87E-09
202097_at	NUP153	205246_at	PEX13	-0.35	0.38	1.07E-09
204411_at	KIF21B	205269_at	LCP2	0.24	-0.49	3.92E-10
201626_at	INSIG1	205292_s_at	HNRNPA2B1	0.55	-0.17	3.32E-10
201144_s_at	EIF2S1	206976_s_at	HSPH1	0.80	0.28	8.71E-11
202078_at	COPS3	206976_s_at	HSPH1	0.76	0.20	4.00E-10
203743_s_at	TDG	206976_s_at	HSPH1	0.69	0.05	2.25E-10
205158_at	RNASE4	207079_s_at	MED6	-0.04	0.61	1.65E-09
202843_at	DNAJB9	208249_s_at	TGDS	0.01	0.67	1.12E-10
205141_at	ANG	208447_s_at	PRPS1	-0.33	0.40	7.12E-10
200599_s_at	HSP90B1	208499_s_at	DNAJC3	0.77	0.06	2.02E-14
202655_at	ARMET	208499_s_at	DNAJC3	0.73	-0.01	7.62E-14

200599_s_at	HSP90B1	208645_s_at	RPS14	0.50	0.92	3.33E-16
200599_s_at	HSP90B1	208692_at	RPS3	0.51	0.89	2.76E-12
202115_s_at	NOC2L	208693_s_at	GARS	0.59	-0.08	1.46E-09
200599_s_at	HSP90B1	208695_s_at	RPL39	0.43	0.86	1.83E-11
200599_s_at	HSP90B1	208697_s_at	EIF3E	0.45	0.84	1.75E-09
202413_s_at	USP1	208785_s_at	MAP1LC3B	-0.30	0.42	1.80E-09
206976_s_at	HSPH1	208787_at	MRPL3	0.80	0.29	1.34E-10
208785_s_at	MAP1LC3B	208813_at	GOT1	-0.21	0.54	5.55E-11
202246_s_at	CDK4	209336_at	PWP2	0.73	0.11	1.19E-10
205141_at	ANG	209482_at	POP7	0.11	0.73	3.69E-11
205158_at	RNASE4	209503_s_at	PSMC5	-0.07	0.60	1.19E-09
200812_at	CCT7	209577_at	PCYT2	0.42	-0.30	1.15E-09
200980_s_at	PDHA1	209577_at	PCYT2	0.33	-0.39	1.97E-09
201221_s_at	SNRNP70	209577_at	PCYT2	0.47	-0.27	2.63E-10
201484_at	SUPT4H1	209577_at	PCYT2	0.34	-0.39	8.31E-10
202268_s_at	NAE1	209577_at	PCYT2	0.44	-0.28	1.38E-09
202540_s_at	HMGCR	209577_at	PCYT2	0.37	-0.37	7.75E-10
203082_at	BMS1	209770_at	BTN3A1	-0.35	0.41	1.76E-10
203114_at	SSSCA1	209770_at	BTN3A1	-0.27	0.47	2.53E-10
207618_s_at	BCS1L	209770_at	BTN3A1	-0.28	0.45	6.36E-10
209482_at	POP7	209770_at	BTN3A1	-0.20	0.51	8.53E-10
208813_at	GOT1	210338_s_at	HSPA8	0.40	-0.34	4.51E-10
209196_at	WDR46	210338_s_at	HSPA8	0.47	-0.32	1.14E-11
209577_at	PCYT2	210386_s_at	MTX1	0.36	-0.36	1.85E-09
205158_at	RNASE4	210574_s_at	NUDC	-0.16	0.54	6.47E-10
200599_s_at	HSP90B1	211048_s_at	PDIA4	0.78	0.14	7.95E-13
202655_at	ARMET	211048_s_at	PDIA4	0.78	0.10	5.15E-14
201626_at	INSIG1	211784_s_at	SFRS1	0.49	-0.25	1.71E-10
200010_at	RPL11	211936_at	HSPA5	0.45	0.86	6.85E-11
200013_at	RPL24	211936_at	HSPA5	0.38	0.89	1.11E-16
200018_at	RPS13	211936_at	HSPA5	0.45	0.86	5.36E-11
200032_s_at	RPL9	211936_at	HSPA5	0.44	0.92	<1.00E-16
200034_s_at	RPL6	211936_at	HSPA5	0.42	0.91	<1.00E-16
200038_s_at	RPL17	211936_at	HSPA5	0.45	0.90	1.89E-15
200043_at	ERH	211936_at	HSPA5	0.37	0.82	1.25E-09
200063_s_at	NPM1	211936_at	HSPA5	0.46	0.89	6.43E-13
200089_s_at	RPL4	211936_at	HSPA5	0.42	0.92	<1.00E-16
200091_s_at	RPS25	211936_at	HSPA5	0.36	0.90	<1.00E-16
200092_s_at	RPL37	211936_at	HSPA5	0.47	0.89	2.00E-13
200099_s_at	RPS3A	211936_at	HSPA5	0.48	0.93	<1.00E-16
200718_s_at	SKP1	211936_at	HSPA5	0.27	0.79	3.38E-10
200741_s_at	RPS27	211936_at	HSPA5	0.47	0.85	1.40E-09
200805_at	LMAN2	211936_at	HSPA5	0.43	-0.40	1.94E-12
200825_s_at	HYOU1	211936_at	HSPA5	0.75	0.18	1.80E-10
200926_at	RPS23	211936_at	HSPA5	0.47	0.91	4.44E-16
200937_s_at	RPL5	211936_at	HSPA5	0.34	0.84	4.28E-12
201094_at	RPS29	211936_at	HSPA5	0.46	0.87	2.07E-11
201429_s_at	RPL37A	211936_at	HSPA5	0.49	0.88	2.64E-11
201761_at	MTHFD2	211936_at	HSPA5	0.32	0.82	2.16E-11
201859_at	SRGN	211936_at	HSPA5	0.27	0.79	1.28E-10
202064_s_at	SEL1L	211936_at	HSPA5	0.76	0.18	8.09E-11
202433_at	SLC35B1	211936_at	HSPA5	0.53	-0.46	<1.00E-16
202655_at	ARMET	211936_at	HSPA5	0.92	0.59	6.16E-14
203136_at	RABAC1	211936_at	HSPA5	0.55	-0.16	6.57E-10
203252_at	CDK2AP2	211936_at	HSPA5	0.71	0.02	6.35E-12
204102_s_at	EEF2	211936_at	HSPA5	0.40	0.86	5.38E-12
205158_at	RNASE4	211936_at	HSPA5	0.52	-0.36	1.83E-14
208499_s_at	DNAJC3	211936_at	HSPA5	0.76	0.13	2.82E-12
208649_s_at	VCP	211936_at	HSPA5	0.63	-0.09	2.95E-11
208695_s_at	RPL39	211936_at	HSPA5	0.44	0.92	<1.00E-16

211048_s_at	PDIA4	211936_at	HSPA5	0.84	0.25	2.18E-14
200599_s_at	HSP90B1	211956_s_at	EIF1	0.52	0.89	5.55E-12
211936_at	HSPA5	211956_s_at	EIF1	0.53	0.88	3.65E-10
200078_s_at	ATP6V0B	212411_at	IMP4	0.62	-0.05	5.71E-10
200812_at	CCT7	212411_at	IMP4	0.66	-0.17	9.44E-15
200877_at	CCT4	212411_at	IMP4	0.65	-0.05	3.94E-11
200881_s_at	DNAJA1	212411_at	IMP4	0.42	-0.38	1.19E-11
200910_at	CCT3	212411_at	IMP4	0.49	-0.23	6.29E-10
201066_at	CYC1	212411_at	IMP4	0.62	-0.06	3.41E-10
201241_at	DDX1	212411_at	IMP4	0.66	-0.12	4.78E-13
201252_at	PSMC4	212411_at	IMP4	0.52	-0.18	9.63E-10
201267_s_at	PSMC3	212411_at	IMP4	0.56	-0.26	1.27E-12
201400_at	PSMB3	212411_at	IMP4	0.67	0.05	8.28E-10
201405_s_at	COPS6	212411_at	IMP4	0.74	0.07	2.75E-12
201459_at	RUVBL2	212411_at	IMP4	0.56	-0.17	1.02E-10
201489_at	PPIF	212411_at	IMP4	0.65	-0.06	2.10E-11
201491_at	AHSA1	212411_at	IMP4	0.65	-0.08	8.43E-12
201532_at	PSMA3	212411_at	IMP4	0.59	-0.10	6.17E-10
201574_at	ETF1	212411_at	IMP4	0.44	-0.38	3.05E-12
201584_s_at	DDX39	212411_at	IMP4	0.69	0.01	1.47E-11
201608_s_at	PWP1	212411_at	IMP4	0.62	-0.15	2.94E-12
201629_s_at	ACP1	212411_at	IMP4	0.63	-0.11	8.82E-12
201714_at	TUBG1	212411_at	IMP4	0.66	-0.07	4.10E-12
201740_at	NDUFS3	212411_at	IMP4	0.59	-0.09	5.57E-10
201947_s_at	CCT2	212411_at	IMP4	0.65	-0.10	2.10E-12
202144_s_at	ADSL	212411_at	IMP4	0.65	0.00	4.44E-10
202209_at	LSM3	212411_at	IMP4	0.69	0.09	1.36E-09
202246_s_at	CDK4	212411_at	IMP4	0.67	-0.13	4.60E-14
202268_s_at	NAE1	212411_at	IMP4	0.50	-0.27	4.52E-11
202691_at	SNRPD1	212411_at	IMP4	0.64	-0.11	2.89E-12
202753_at	PSMD6	212411_at	IMP4	0.58	-0.15	1.22E-10
202899_s_at	SFRS3	212411_at	IMP4	0.57	-0.14	3.42E-10
203109_at	UBE2M	212411_at	IMP4	0.60	-0.17	4.42E-12
203114_at	SSSCA1	212411_at	IMP4	0.63	-0.05	1.65E-10
203150_at	RABEPK	212411_at	IMP4	0.60	-0.09	2.94E-10
203436_at	RPP30	212411_at	IMP4	0.67	-0.07	1.80E-12
203931_s_at	MRPL12	212411_at	IMP4	0.56	-0.14	6.35E-10
204331_s_at	MRPS12	212411_at	IMP4	0.69	0.09	9.09E-10
205133_s_at	HSPE1	212411_at	IMP4	0.45	-0.29	2.65E-10
205512_s_at	AIFM1	212411_at	IMP4	0.62	-0.06	3.11E-10
206445_s_at	PRMT1	212411_at	IMP4	0.74	0.02	8.35E-14
207891_s_at	HAUS7	212411_at	IMP4	0.70	0.10	8.12E-10
208696_at	CCT5	212411_at	IMP4	0.60	-0.08	5.87E-10
208758_at	ATIC	212411_at	IMP4	0.57	-0.21	4.88E-12
208821_at	SNRPB	212411_at	IMP4	0.75	0.21	2.07E-09
208972_s_at	ATP5G1	212411_at	IMP4	0.52	-0.25	2.37E-11
209103_s_at	UFD1L	212411_at	IMP4	0.63	-0.01	1.77E-09
209233_at	EMG1	212411_at	IMP4	0.65	-0.10	4.14E-12
209279_s_at	NSDHL	212411_at	IMP4	0.57	-0.21	1.02E-11
209464_at	AURKB	212411_at	IMP4	0.74	0.17	5.27E-10
209482_at	POP7	212411_at	IMP4	0.73	0.13	1.19E-10
209503_s_at	PSMC5	212411_at	IMP4	0.53	-0.22	5.83E-11
209511_at	POLR2F	212411_at	IMP4	0.63	-0.05	3.68E-10
209825_s_at	UCK2	212411_at	IMP4	0.68	-0.03	7.33E-12
210213_s_at	EIF6	212411_at	IMP4	0.66	-0.01	1.66E-10
210338_s_at	HSPA8	212411_at	IMP4	0.39	-0.37	1.12E-10
210574_s_at	NUDC	212411_at	IMP4	0.56	-0.24	1.40E-12
212115_at	HN1L	212411_at	IMP4	0.63	-0.09	2.39E-11
210338_s_at	HSPA8	212501_at	CEBPB	-0.07	-0.67	1.90E-09
212411_at	IMP4	212627_s_at	EXOSC7	0.59	-0.15	3.31E-11

212411_at	IMP4	212656_at	TSFM	0.50	-0.28	2.83E-11
209577_at	PCYT2	212766_s_at	ISG20L2	0.33	-0.44	5.87E-11
212411_at	IMP4	212766_s_at	ISG20L2	0.55	-0.18	1.66E-10
212160_at	XPOT	212825_at	PAXIP1	0.59	-0.08	1.10E-09
200881_s_at	DNAJA1	213086_s_at	CSNK1A1	-0.04	-0.69	1.29E-10
204394_at	SLC43A1	213086_s_at	CSNK1A1	-0.08	0.62	1.30E-10
210338_s_at	HSPA8	213086_s_at	CSNK1A1	0.08	-0.75	<1.00E-16
200599_s_at	HSP90B1	213101_s_at	ACTR3	0.39	0.83	7.15E-10
211936_at	HSPA5	213101_s_at	ACTR3	0.38	0.89	1.11E-16
200599_s_at	HSP90B1	213414_s_at	RPS19	0.50	0.92	<1.00E-16
200692_s_at	HSPA9	213531_s_at	RAB3GAP1	-0.39	0.33	1.24E-09
201263_at	TARS	213531_s_at	RAB3GAP1	-0.27	0.49	1.18E-10
209050_s_at	RALGDS	213531_s_at	RAB3GAP1	0.22	-0.48	2.01E-09
212501_at	CEBPB	213531_s_at	RAB3GAP1	-0.12	0.59	2.49E-10
213531_s_at	RAB3GAP1	213671_s_at	MARS	-0.19	0.52	4.53E-10
200599_s_at	HSP90B1	213687_s_at	RPL35A	0.41	0.85	6.93E-11
211936_at	HSPA5	213738_s_at	ATP5A1	0.49	0.90	6.76E-14
205158_at	RNASE4	213937_s_at	FTSJ1	0.26	0.78	4.66E-10
212411_at	IMP4	214011_s_at	NOP16	0.59	-0.19	6.70E-12
200599_s_at	HSP90B1	214167_s_at	RPLP0	0.46	0.87	3.01E-11
200063_s_at	NPM1	214328_s_at	HSP90AA1	0.98	0.89	3.18E-10
212411_at	IMP4	214670_at	ZKSCAN1	-0.60	0.09	4.21E-10
212411_at	IMP4	215416_s_at	STOML2	0.53	-0.15	1.88E-09
202961_s_at	ATP5J2	215424_s_at	SNW1	-0.44	0.31	1.95E-10
208620_at	PCBP1	215424_s_at	SNW1	-0.05	0.61	7.80E-10
209577_at	PCYT2	215905_s_at	SNRNP40	0.28	-0.45	6.36E-10
212411_at	IMP4	216194_s_at	TBCB	0.52	-0.19	6.06E-10
200599_s_at	HSP90B1	216231_s_at	B2M	0.53	0.93	<1.00E-16
200599_s_at	HSP90B1	216438_s_at	TMSB4X	0.45	0.87	9.11E-12
211936_at	HSPA5	216438_s_at	TMSB4X	0.45	0.92	<1.00E-16
200599_s_at	HSP90B1	216520_s_at	TPT1	0.51	0.90	5.09E-13
211936_at	HSPA5	216640_s_at	PDIA6	0.66	-0.20	7.77E-16
209577_at	PCYT2	216952_s_at	LMNB2	0.33	-0.40	1.18E-09
212411_at	IMP4	216952_s_at	LMNB2	0.48	-0.24	8.59E-10
209577_at	PCYT2	217188_s_at	C14orf1	0.32	-0.46	4.18E-11
211936_at	HSPA5	217719_at	EIF3L	0.33	0.83	1.23E-11
211936_at	HSPA5	217801_at	ATP5E	0.27	0.78	4.73E-10
200875_s_at	NOP56	217850_at	GNL3	0.79	0.29	1.48E-09
212411_at	IMP4	217932_at	MRPS7	0.60	-0.15	2.39E-11
212411_at	IMP4	217960_s_at	TOMM22	0.37	-0.34	1.84E-09
205141_at	ANG	217972_at	CHCHD3	-0.39	0.34	1.01E-09
205158_at	RNASE4	217972_at	CHCHD3	-0.17	0.62	1.26E-12
205246_at	PEX13	218003_s_at	FKBP3	-0.68	-0.06	7.22E-10
212411_at	IMP4	218027_at	MRPL15	0.69	0.03	8.69E-11
211936_at	HSPA5	218070_s_at	GMPPA	0.72	-0.03	8.46E-14
212411_at	IMP4	218105_s_at	MRPL4	0.74	0.05	5.10E-13
201037_at	PFKP	218145_at	TRIB3	0.43	-0.32	1.94E-10
212411_at	IMP4	218235_s_at	UTP11L	0.57	-0.16	9.51E-11
211936_at	HSPA5	218282_at	EDEM2	0.74	0.17	2.27E-10
212411_at	IMP4	218324_s_at	SPATS2	-0.55	0.21	1.98E-11
212411_at	IMP4	218336_at	PFDN2	0.67	-0.10	1.73E-13
208499_s_at	DNAJC3	218358_at	CRELD2	0.60	-0.07	8.88E-10
211048_s_at	PDIA4	218358_at	CRELD2	0.68	0.07	1.07E-09
211936_at	HSPA5	218358_at	CRELD2	0.74	-0.01	3.01E-14
205141_at	ANG	218373_at	AKTIP	-0.36	0.37	9.63E-10
213531_s_at	RAB3GAP1	218389_s_at	APH1A	-0.29	0.48	6.80E-11
209770_at	BTN3A1	218408_at	TIMM10	-0.39	0.41	1.12E-11
203109_at	UBE2M	218409_s_at	DNAJC1	-0.31	0.41	1.40E-09
204460_s_at	RAD1	218409_s_at	DNAJC1	-0.35	0.38	8.70E-10
207830_s_at	PPP1R8	218409_s_at	DNAJC1	-0.30	0.44	3.12E-10

209482_at	POP7	218409_s_at	DNAJC1	-0.22	0.54	5.31E-11
213787_s_at	EBP	218409_s_at	DNAJC1	-0.20	0.50	1.87E-09
213531_s_at	RAB3GAP1	218426_s_at	RNF216	-0.30	0.43	9.75E-10
205141_at	ANG	218488_at	EIF2B3	-0.15	0.55	5.62E-10
205158_at	RNASE4	218488_at	EIF2B3	-0.04	0.62	5.77E-10
212411_at	IMP4	218488_at	EIF2B3	0.48	-0.25	4.66E-10
212411_at	IMP4	218493_at	SNRNP25	0.71	0.13	1.08E-09
212411_at	IMP4	218512_at	WDR12	0.68	0.01	6.66E-11
218409_s_at	DNAJC1	218535_s_at	RIOK2	-0.11	0.61	6.85E-11
212411_at	IMP4	218557_at	NIT2	0.60	-0.12	7.62E-11
202843_at	DNAJB9	218647_s_at	YRDC	0.02	0.67	3.23E-10
208499_s_at	DNAJC3	218681_s_at	SDF2L1	0.64	-0.04	2.44E-10
211936_at	HSPA5	218681_s_at	SDF2L1	0.75	-0.11	<1.00E-16
201626_at	INSIG1	218726_at	HJURP	0.37	-0.35	1.23E-09
205158_at	RNASE4	218732_at	PTRH2	-0.04	0.62	8.68E-10
212411_at	IMP4	218732_at	PTRH2	0.55	-0.14	1.37E-09
212411_at	IMP4	218850_s_at	LIMD1	-0.46	0.28	4.18E-10
212411_at	IMP4	218866_s_at	POLR3K	0.65	-0.05	3.14E-11
213531_s_at	RAB3GAP1	218936_s_at	CCDC59	-0.11	0.56	1.82E-09
200599_s_at	HSP90B1	219117_s_at	FKBP11	0.74	0.03	1.92E-13
200967_at	PPIB	219117_s_at	FKBP11	0.67	0.02	4.17E-10
202655_at	ARMET	219117_s_at	FKBP11	0.81	0.25	3.75E-12
211936_at	HSPA5	219117_s_at	FKBP11	0.77	-0.09	<1.00E-16
202915_s_at	FAM20B	219270_at	CHAC1	0.23	-0.49	9.57E-10
203450_at	CBY1	219270_at	CHAC1	-0.21	0.52	3.40E-10
212411_at	IMP4	219347_at	NUDT15	0.60	-0.10	1.74E-10
211936_at	HSPA5	219600_s_at	TMEM50B	0.50	-0.37	4.19E-14
211936_at	HSPA5	219762_s_at	RPL36	0.40	0.84	2.88E-10
212411_at	IMP4	219819_s_at	MRPS28	0.59	-0.12	2.16E-10
205141_at	ANG	219960_s_at	UCHL5	-0.25	0.50	1.27E-10
205158_at	RNASE4	219960_s_at	UCHL5	0.03	0.67	7.14E-10
209577_at	PCYT2	221255_s_at	TMEM93	0.27	-0.49	5.48E-11
212411_at	IMP4	221255_s_at	TMEM93	0.54	-0.17	5.47E-10
211936_at	HSPA5	221286_s_at	MGC29506	0.77	0.23	2.90E-10
202635_s_at	POLR2K	221423_s_at	YIPF5	-0.44	0.29	8.16E-10
200599_s_at	HSP90B1	221475_s_at	RPL15	0.46	0.85	6.05E-10
212411_at	IMP4	221649_s_at	PPAN	0.56	-0.12	1.36E-09
209577_at	PCYT2	221711_s_at	C19orf62	0.41	-0.34	1.50E-10
211936_at	HSPA5	221739_at	C19orf10	0.77	0.03	3.11E-15
201275_at	FDPS	221750_at	HMGCS1	0.70	0.04	2.17E-11
208647_at	FDFT1	221750_at	HMGCS1	0.79	0.25	4.89E-11
211936_at	HSPA5	221791_s_at	CCDC72	0.40	0.90	1.11E-16
209770_at	BTN3A1	221970_s_at	NOL11	-0.40	0.31	1.93E-09
201626_at	INSIG1	222006_at	LETM1	-0.65	0.06	3.90E-11
205141_at	ANG	222231_s_at	LRRC59	0.35	0.82	1.33E-10
203579_s_at	SLC7A6	37028_at	PPP1R15A	0.47	-0.26	4.12E-10

Supplemental Table 7. Gene pairs that are differentially coexpressed following IR stress

Affymetrix ID #1	Gene Symbol # 1	Affymetrix ID #2	Gene Symbol # 2	R - 0hr	R - 2hr	R - 6hr	Nominal P-value
201849_at	BNIP3	201948_at	GNL2	-0.69	-0.52	0.06	2.62E-09
201849_at	BNIP3	202022_at	ALDOC	0.85	0.88	0.35	5.55E-13
202022_at	ALDOC	202499_s_at	SLC2A3	0.56	-0.25	-0.12	9.25E-10
201849_at	BNIP3	202856_s_at	SLC16A3	0.87	0.88	0.40	1.73E-12
202022_at	ALDOC	202856_s_at	SLC16A3	0.88	0.88	0.43	1.74E-11
202499_s_at	SLC2A3	202856_s_at	SLC16A3	0.53	-0.33	-0.05	1.06E-09
202499_s_at	SLC2A3	202887_s_at	DDIT4	0.49	-0.36	-0.30	5.34E-11
202095_s_at	BIRC5	205046_at	CENPE	0.76	0.62	0.06	6.60E-10
202535_at	FADD	206188_at	ZNF623	-0.20	0.57	-0.15	8.15E-10
201849_at	BNIP3	207543_s_at	P4HA1	0.88	0.86	0.48	2.44E-09
203487_s_at	ARMC8	208720_s_at	RBM39	0.54	-0.43	0.25	1.60E-12
205046_at	CENPE	209398_at	HIST1H1C	0.33	0.43	-0.42	2.16E-10
205046_at	CENPE	209806_at	HIST1H2BK	0.08	0.24	-0.56	2.47E-09
209311_at	BCL2L2	209894_at	LEPR	-0.12	0.63	-0.11	1.48E-10
209894_at	LEPR	211512_s_at	OGFR	-0.49	0.37	-0.20	1.52E-09
203409_at	DDB2	212453_at	KIAA1279	0.21	0.57	-0.28	2.68E-09
201849_at	BNIP3	212689_s_at	KDM3A	0.78	0.15	0.31	6.83E-10
201968_s_at	PGM1	212689_s_at	KDM3A	0.76	0.24	0.10	9.65E-10
202022_at	ALDOC	212689_s_at	KDM3A	0.75	0.22	-0.02	2.71E-11
202856_s_at	SLC16A3	212689_s_at	KDM3A	0.80	0.21	0.24	4.48E-11
202887_s_at	DDIT4	212689_s_at	KDM3A	0.61	-0.22	-0.34	2.45E-14
207543_s_at	P4HA1	212689_s_at	KDM3A	0.88	0.40	0.37	7.54E-14
208720_s_at	RBM39	212907_at	SLC30A1	0.19	-0.62	-0.10	1.70E-09
212689_s_at	KDM3A	213011_s_at	TPI1	0.49	-0.14	-0.37	6.98E-10
212689_s_at	KDM3A	214683_s_at	CLK1	-0.48	-0.06	0.39	1.81E-09
202022_at	ALDOC	218149_s_at	ZNF395	0.67	0.60	-0.18	1.49E-12
202887_s_at	DDIT4	218149_s_at	ZNF395	0.61	0.09	-0.19	2.76E-09
213011_s_at	TPI1	218149_s_at	ZNF395	0.60	0.59	-0.14	7.83E-10
201849_at	BNIP3	218507_at	C7orf68	0.84	0.34	0.04	1.11E-15
202499_s_at	SLC2A3	218507_at	C7orf68	0.56	-0.13	-0.27	2.29E-10
202856_s_at	SLC16A3	218507_at	C7orf68	0.78	0.29	0.19	2.48E-09
207543_s_at	P4HA1	218507_at	C7orf68	0.74	0.22	-0.05	2.33E-11
209566_at	INSIG2	218507_at	C7orf68	0.63	0.00	-0.28	3.65E-12
209882_at	RIT1	218507_at	C7orf68	0.59	-0.07	-0.17	2.62E-09
212689_s_at	KDM3A	218507_at	C7orf68	0.70	-0.03	-0.20	9.47E-14
218149_s_at	ZNF395	218507_at	C7orf68	0.75	0.29	0.01	2.84E-10
218527_at	APTX	218755_at	KIF20A	-0.27	-0.27	0.52	2.04E-10
202225_at	CRK	219515_at	PRDM10	-0.05	0.71	0.27	8.69E-10
219515_at	PRDM10	219627_at	ZNF767	0.18	0.71	0.00	2.07E-09
202499_s_at	SLC2A3	219862_s_at	NARF	0.52	-0.28	-0.18	1.09E-09
202225_at	CRK	220195_at	MBD5	-0.23	0.59	-0.02	1.22E-09
203487_s_at	ARMC8	220195_at	MBD5	0.00	0.67	-0.02	1.46E-09
205046_at	CENPE	220500_s_at	RABL2A	-0.41	-0.40	0.36	2.46E-09
215191_at	FBXL11	220500_s_at	RABL2A	-0.35	0.01	0.52	1.43E-09
218755_at	KIF20A	220500_s_at	RABL2A	-0.41	-0.39	0.38	7.82E-10
218703_at	SEC22A	221203_s_at	YEATS2	-0.39	0.47	0.24	1.27E-09
203674_at	HELZ	221208_s_at	C11orf61	-0.48	0.27	0.31	8.07E-10
200666_s_at	DNAJB1	222270_at	SMEK2	0.12	-0.64	0.08	1.51E-10
202499_s_at	SLC2A3	222270_at	SMEK2	-0.51	0.34	-0.23	2.80E-09
216574_s_at	hCG_2024410	222273_at	PAPOLG	0.58	-0.26	-0.19	3.27E-11

Supplemental Table 8. Genes that are differentially coexpressed following ER stress that are known to have direct protein-protein interactions

200599_s_at	HSP90B1	201049_s_at	RPS18
200599_s_at	HSP90B1	208645_s_at	RPS14
200599_s_at	HSP90B1	208692_at	RPS3
200599_s_at	HSP90B1	208697_s_at	EIF3E
200599_s_at	HSP90B1	211048_s_at	PDIA4
200599_s_at	HSP90B1	213414_s_at	RPS19
200599_s_at	HSP90B1	214167_s_at	RPLP0
211936_at	HSPA5	213738_s_at	ATP5A1
211936_at	HSPA5	216640_s_at	PDIA6
211936_at	HSPA5	217719_at	EIF3L
211936_at	HSPA5	219762_s_at	RPL36
200825_s_at	HYOU1	211936_at	HSPA5
200875_s_at	NOP56	217850_at	GNL3
200063_s_at	NPM1	211936_at	HSPA5
200063_s_at	NPM1	214328_s_at	HSP90AA1
208620_at	PCBP1	215424_s_at	SNW1
211048_s_at	PDIA4	211936_at	HSPA5
200967_at	PPIB	219117_s_at	FKBP11
200029_at	RPL19	200599_s_at	HSP90B1
200013_at	RPL24	211936_at	HSPA5
200089_s_at	RPL4	211936_at	HSPA5
200018_at	RPS13	211936_at	HSPA5
200091_s_at	RPS25	211936_at	HSPA5
201459_at	RUVBL2	212411_at	IMP4

Supplemental Table 9. Gene pairs that are differentially coexpressed after ER stress that interact via UBC3

202144_s_at	ADSL	212411_at	IMP4
202655_at	ARMET	208499_s_at	DNAJC3
202655_at	ARMET	211048_s_at	PDIA4
202655_at	ARMET	211936_at	HSPA5
208972_s_at	ATP5G1	212411_at	IMP4
207618_s_at	BCS1L	209770_at	BTN3A1
203082_at	BMS1	209770_at	BTN3A1
209770_at	BTN3A1	218408_at	TIMM10
209770_at	BTN3A1	221970_s_at	NOL11
200812_at	CCT7	209577_at	PCYT2
212501_at	CEBPB	213531_s_at	RAB3GAP1
202060_at	CTR9	205246_at	PEX13
201066_at	CYC1	212411_at	IMP4
202843_at	DNAJB9	208249_s_at	TGDS
202843_at	DNAJB9	218647_s_at	YRDC
208499_s_at	DNAJC3	218358_at	CRELD2
208499_s_at	DNAJC3	218681_s_at	SDF2L1
208647_at	FDFT1	221750_at	HMGCS1
202540_s_at	HMGCR	209577_at	PCYT2
212115_at	HN1L	212411_at	IMP4
200599_s_at	HSP90B1	211956_s_at	EIF1
211936_at	HSPA5	211956_s_at	EIF1
211936_at	HSPA5	218282_at	EDEM2
211936_at	HSPA5	218358_at	CRELD2
211936_at	HSPA5	219600_s_at	TMEM50B
200692_s_at	HSPA9	213531_s_at	RAB3GAP1
206976_s_at	HSPH1	208787_at	MRPL3
212411_at	IMP4	212656_at	TSFM
212411_at	IMP4	216194_s_at	TBCB
212411_at	IMP4	216952_s_at	LMNB2
212411_at	IMP4	218235_s_at	UTP11L
212411_at	IMP4	218336_at	PFDN2
212411_at	IMP4	218488_at	EIF2B3
212411_at	IMP4	218493_at	SNRNP25
212411_at	IMP4	218512_at	WDR12
212411_at	IMP4	218557_at	NIT2
212411_at	IMP4	218732_at	PTRH2
201626_at	INSIG1	203276_at	LMNB1
201626_at	INSIG1	204394_at	SLC43A1
201626_at	INSIG1	218726_at	HJURP
201626_at	INSIG1	222006_at	LETM1
201412_at	LRP10	201582_at	SEC23B
202209_at	LSM3	212411_at	IMP4
208785_s_at	MAP1LC3B	208813_at	GOT1
204331_s_at	MRPS12	212411_at	IMP4

209279_s_at	NSDHL	212411_at	IMP4
202097_at	NUP153	205246_at	PEX13
209577_at	PCYT2	210386_s_at	MTX1
209577_at	PCYT2	215905_s_at	SNRNP40
209577_at	PCYT2	216952_s_at	LMNB2
209577_at	PCYT2	217188_s_at	C14orf1
209577_at	PCYT2	221711_s_at	C19orf62
211048_s_at	PDIA4	218358_at	CRELD2
205246_at	PEX13	218003_s_at	FKBP3
209482_at	POP7	209770_at	BTN3A1
201489_at	PPIF	212411_at	IMP4
206445_s_at	PRMT1	212411_at	IMP4
213531_s_at	RAB3GAP1	213671_s_at	MARS
213531_s_at	RAB3GAP1	218389_s_at	APH1A
213531_s_at	RAB3GAP1	218426_s_at	RNF216
203150_at	RABEPK	212411_at	IMP4
202636_at	RNF103	202843_at	DNAJB9
200017_at	RPS27A	200599_s_at	HSP90B1
204394_at	SLC43A1	213086_s_at	CSNK1A1
201221_s_at	SNRNP70	209577_at	PCYT2
203114_at	SSSCA1	209770_at	BTN3A1
203114_at	SSSCA1	212411_at	IMP4
201484_at	SUPT4H1	209577_at	PCYT2
201263_at	TARS	213531_s_at	RAB3GAP1
209825_s_at	UCK2	212411_at	IMP4
209103_s_at	UFD1L	212411_at	IMP4
202413_s_at	USP1	208785_s_at	MAP1LC3B

Supplemental Table 10. Gene pairs that alter interactions after both ER and IR stress

216397_s_at	BOP1
214710_s_at	CCNB1
214507_s_at	EXOSC2
204781_s_at	FAS
205527_s_at	GEMIN4
214431_at	GMPS
217850_at	GNL3
200064_at	HSP90AB1
202167_s_at	MMS19
210797_s_at	OASL
200827_at	PLOD1
213483_at	PPWD1
217861_s_at	PREB
201316_at	PSMA2
213951_s_at	PSMC3IP
201699_at	PSMC6
209336_at	PWP2
202297_s_at	RER1
205748_s_at	RNF126
217833_at	SYNCRIP
209295_at	TNFRSF10B
219131_at	UBIAD1
205519_at	WDR76
